# Supplementary material for: Investigation of multiple sclerosis-related pathways through the integration of genomic and proteomic data
Source: PeerJ. 2021 Sep 6;9:e11922. doi: 10.7717/peerj.11922 (PMC11563213; doi:10.7717/peerj.11922)
Supplement: Supplemental Information 4 [file peerj-09-11922-s004.pdf]

```
Index    Name     Chr      MS2.Top Alleles  MS3.Top Alleles  MS4.Top
Alleles  MS10.Top Alleles MS11.Top Alleles MS12.Top Alleles MS13.Top
Alleles  MS14.Top Alleles MS15.Top Alleles MS16.Top Alleles MS17.Top
Alleles  MS18.Top Alleles MS19.Top Alleles MS20.Top Alleles MS21.Top
Alleles  MS22.Top Alleles MS23.Top Alleles MS24.Top Alleles MS30.Top
Alleles  MS31.Top Alleles MS32.Top Alleles MS33.Top Alleles MS34.Top
Alleles  MS35.Top Alleles MS36.Top Alleles MS37.Top Alleles MS38.Top
Alleles  MS39.Top Alleles MS40.Top Alleles MS45.Top Alleles MS47.Top
Alleles  MS48.Top Alleles MS49.Top Alleles MS66.Top Alleles MS67.Top
Alleles  MS68.Top Alleles Position
1        cnvi0111185
13       --       --       --       --       --       --       --       --
         --       --       --       --       --       --       --       --
         --       --       --       --       --       --       --       --
         --       --       --       --       --       --       --       --
         --       --       --       --       100631779
2        cnvi0111186
2        --       --       --       --       --       --       --       --
         --       --       --       --       --       --       --       --
         --       --       --       --       --       --       --       --
         --       --       --       --       --       --       --       --
         --       --       --       --       177058958
3        cnvi0111187
17       --       --       --       --       --       --       --       --
         --       --       --       --       --       --       --       --
         --       --       --       --       --       --       --       --
         --       --       --       --       --       --       --       --
         --       --       --       --       35295593
4        cnvi0111188
8        --       --       --       --       --       --       --       --
         --       --       --       --       --       --       --       --
         --       --       --       --       --       --       --       --
         --       --       --       --       --       --       --       --
         --       --       --       --       65499402
5        cnvi0111189
2        --       --       --       --       --       --       --       --
         --       --       --       --       --       --       --       --
         --       --       --       --       --       --       --       --
         --       --       --       --       --       --       --       --
         --       --       --       --       177061178
6        cnvi0111190
5        --       --       --       --       --       --       --       --
         --       --       --       --       --       --       --       --
         --       --       --       --       --       --       --       --
         --       --       --       --       --       --       --       --
         --       --       --       --       70255894
7        cnvi0111191
11       --       --       --       --       --       --       --       --
         --       --       --       --       --       --       --       --
         --       --       --       --       --       --       --       --
```

-- -- -- -- -- -- -- --
-- -- -- -- 4369249
8 cnvi0111192
8 -- -- -- -- -- -- -- --
-- -- -- -- -- -- -- --
-- -- -- -- -- -- -- --
-- -- -- -- -- -- -- --
-- -- -- -- 61788030
9 cnvi0111193
3 -- -- -- -- -- -- -- --
-- -- -- -- -- -- -- --
-- -- -- -- -- -- -- --
-- -- -- -- -- -- -- --
-- -- -- -- 33482656
10 cnvi0111194
14 -- -- -- -- -- -- -- --
-- -- -- -- -- -- -- --
-- -- -- -- -- -- -- --
-- -- -- -- -- -- -- --
-- -- -- -- 29235583
11 cnvi0111195
7 -- -- -- -- -- -- -- --
-- -- -- -- -- -- -- --
-- -- -- -- -- -- -- --
-- -- -- -- -- -- -- --
-- -- -- -- 3633076
12 cnvi0111196
17 -- -- -- -- -- -- -- --
-- -- -- -- -- -- -- --
-- -- -- -- -- -- -- --
-- -- -- -- -- -- -- --
-- -- -- -- 36358526
13 cnvi0111197
12 -- -- -- -- -- -- -- --
-- -- -- -- -- -- -- --
-- -- -- -- -- -- -- --
-- -- -- -- -- -- -- --
-- -- -- -- 14870711
14 cnvi0111198
10 -- -- -- -- -- -- -- --
-- -- -- -- -- -- -- --
-- -- -- -- -- -- -- --
-- -- -- -- -- -- -- --
-- -- -- -- 47747841
15 cnvi0111199
4 -- -- -- -- -- -- -- --
-- -- -- -- -- -- -- --
-- -- -- -- -- -- -- --
-- -- -- -- -- -- -- --
-- -- -- -- 41747880

16 cnvi0111200
16 -- -- -- -- -- -- -- --
-- -- -- -- -- -- -- --
-- -- -- -- -- -- -- --
-- -- -- -- -- -- -- --
-- -- -- -- 3216863
17 cnvi0111201
17 -- -- -- -- -- -- -- --
-- -- -- -- -- -- -- --
-- -- -- -- -- -- -- --
-- -- -- -- -- -- -- --
-- -- -- -- 35290412
18 cnvi0111202
8 -- -- -- -- -- -- -- --
-- -- -- -- -- -- -- --
-- -- -- -- -- -- -- --
-- -- -- -- -- -- -- --
-- -- -- -- 55366858
19 cnvi0111203
18 -- -- -- -- -- -- -- --
-- -- -- -- -- -- -- --
-- -- -- -- -- -- -- --
-- -- -- -- -- -- -- --
-- -- -- -- 3652738
20 cnvi0111204
16 -- -- -- -- -- -- -- --
-- -- -- -- -- -- -- --
-- -- -- -- -- -- -- --
-- -- -- -- -- -- -- --
-- -- -- -- 86599395
21 cnvi0111205
2 -- -- -- -- -- -- -- --
-- -- -- -- -- -- -- --
-- -- -- -- -- -- -- --
-- -- -- -- -- -- -- --
-- -- -- -- 5833810
22 cnvi0111206
8 -- -- -- -- -- -- -- --
-- -- -- -- -- -- -- --
-- -- -- -- -- -- -- --
-- -- -- -- -- -- -- --
-- -- -- -- 55362414
23 cnvi0111207
17 -- -- -- -- -- -- -- --
-- -- -- -- -- -- -- --
-- -- -- -- -- -- -- --
-- -- -- -- -- -- -- --
-- -- -- -- 34623346
24 cnvi0111208
8 -- -- -- -- -- -- -- --

-- -- -- -- -- -- -- --
-- -- -- -- -- -- -- --
-- -- -- -- -- -- -- --
-- -- -- -- 55382311
25 cnvi0111209
20 -- -- -- -- -- -- -- --
-- -- -- -- -- -- -- --
-- -- -- -- -- -- -- --
-- -- -- -- -- -- -- --
-- -- -- -- 21683612
26 cnvi0111211
10 -- -- -- -- -- -- -- --
-- -- -- -- -- -- -- --
-- -- -- -- -- -- -- --
-- -- -- -- -- -- -- --
-- -- -- -- 47751590
27 cnvi0111212
2 -- -- -- -- -- -- -- --
-- -- -- -- -- -- -- --
-- -- -- -- -- -- -- --
-- -- -- -- -- -- -- --
-- -- -- -- 237080164
28 cnvi0111213
13 -- -- -- -- -- -- -- --
-- -- -- -- -- -- -- --
-- -- -- -- -- -- -- --
-- -- -- -- -- -- -- --
-- -- -- -- 79171645
29 cnvi0111214
13 -- -- -- -- -- -- -- --
-- -- -- -- -- -- -- --
-- -- -- -- -- -- -- --
-- -- -- -- -- -- -- --
-- -- -- -- 100644039
30 cnvi0111215
18 -- -- -- -- -- -- -- --
-- -- -- -- -- -- -- --
-- -- -- -- -- -- -- --
-- -- -- -- -- -- -- --
-- -- -- -- 19750602
31 cnvi0111217
17 -- -- -- -- -- -- -- --
-- -- -- -- -- -- -- --
-- -- -- -- -- -- -- --
-- -- -- -- -- -- -- --
-- -- -- -- 61993016
32 cnvi0111218
8 -- -- -- -- -- -- -- --
-- -- -- -- -- -- -- --
-- -- -- -- -- -- -- --

	--	--	--	--	--	--	--	--
	--	--	--	--	55364636			
33	cnvi0111219							
1	--	--	--	--	--	--	--	--
	--	--	--	--	--	--	--	--
	--	--	--	--	--	--	--	--
	--	--	--	--	--	--	--	--
	--	--	--	--	47875450			
34	cnvi0111220							
7	--	--	--	--	--	--	--	--
	--	--	--	--	--	--	--	--
	--	--	--	--	--	--	--	--
	--	--	--	--	--	--	--	--
	--	--	--	--	155606728			
35	cnvi0111222							
17	--	--	--	--	--	--	--	--
	--	--	--	--	--	--	--	--
	--	--	--	--	--	--	--	--
	--	--	--	--	--	--	--	--
	--	--	--	--	37549924			
36	cnvi0111223							
17	--	--	--	--	--	--	--	--
	--	--	--	--	--	--	--	--
	--	--	--	--	--	--	--	--
	--	--	--	--	--	--	--	--
	--	--	--	--	34626613			
37	cnvi0111224							
1	--	--	--	--	--	--	--	--
	--	--	--	--	--	--	--	--
	--	--	--	--	--	--	--	--
	--	--	--	--	--	--	--	--
	--	--	--	--	244208615			
38	cnvi0111225							
17	--	--	--	--	--	--	--	--
	--	--	--	--	--	--	--	--
	--	--	--	--	--	--	--	--
	--	--	--	--	--	--	--	--
	--	--	--	--	36358102			
39	cnvi0111226							
8	--	--	--	--	--	--	--	--
	--	--	--	--	--	--	--	--
	--	--	--	--	--	--	--	--
	--	--	--	--	--	--	--	--
	--	--	--	--	65501722			
40	cnvi0111227							
17	--	--	--	--	--	--	--	--
	--	--	--	--	--	--	--	--
	--	--	--	--	--	--	--	--
	--	--	--	--	--	--	--	--
	--	--	--	--	35293013			

41 cnvi0111228
8 -- -- -- -- -- -- -- --
-- -- -- -- -- -- -- --
-- -- -- -- -- -- -- --
-- -- -- -- -- -- -- --
-- -- -- -- 55377867
42 cnvi0111229
13 -- -- -- -- -- -- -- --
-- -- -- -- -- -- -- --
-- -- -- -- -- -- -- --
-- -- -- -- -- -- -- --
-- -- -- -- 79164325
43 cnvi0111230
4 -- -- -- -- -- -- -- --
-- -- -- -- -- -- -- --
-- -- -- -- -- -- -- --
-- -- -- -- -- -- -- --
-- -- -- -- 41750360
44 cnvi0111231
3 -- -- -- -- -- -- -- --
-- -- -- -- -- -- -- --
-- -- -- -- -- -- -- --
-- -- -- -- -- -- -- --
-- -- -- -- 72416070
45 cnvi0111232
8 -- -- -- -- -- -- -- --
-- -- -- -- -- -- -- --
-- -- -- -- -- -- -- --
-- -- -- -- -- -- -- --
-- -- -- -- 65492442
46 cnvi0111233
2 -- -- -- -- -- -- -- --
-- -- -- -- -- -- -- --
-- -- -- -- -- -- -- --
-- -- -- -- -- -- -- --
-- -- -- -- 5828090
47 cnvi0111234
13 -- -- -- -- -- -- -- --
-- -- -- -- -- -- -- --
-- -- -- -- -- -- -- --
-- -- -- -- -- -- -- --
-- -- -- -- 100634247
48 cnvi0111235
13 -- -- -- -- -- -- -- --
-- -- -- -- -- -- -- --
-- -- -- -- -- -- -- --
-- -- -- -- -- -- -- --
-- -- -- -- 36055323
49 cnvi0111236
21 -- -- -- -- -- -- -- --

	--	--	--	--	--	--	--	--
	--	--	--	--	--	--	--	--
	--	--	--	--	--	--	--	--
	--	--	--	--	37777184			
50	cnvi0111237							
5	--	--	--	--	--	--	--	--
	--	--	--	--	--	--	--	--
	--	--	--	--	--	--	--	--
	--	--	--	--	--	--	--	--
	--	--	--	--	70326901			
51	cnvi0111239							
8	--	--	--	--	--	--	--	--
	--	--	--	--	--	--	--	--
	--	--	--	--	--	--	--	--
	--	--	--	--	--	--	--	--
	--	--	--	--	65494762			
52	cnvi0111240							
17	--	--	--	--	--	--	--	--
	--	--	--	--	--	--	--	--
	--	--	--	--	--	--	--	--
	--	--	--	--	--	--	--	--
	--	--	--	--	36356842			
53	cnvi0111241							
12	--	--	--	--	--	--	--	--
	--	--	--	--	--	--	--	--
	--	--	--	--	--	--	--	--
	--	--	--	--	--	--	--	--
	--	--	--	--	4561343			
54	cnvi0111242							
6	--	--	--	--	--	--	--	--
	--	--	--	--	--	--	--	--
	--	--	--	--	--	--	--	--
	--	--	--	--	--	--	--	--
	--	--	--	--	137818186			
55	cnvi0111243							
1	--	--	--	--	--	--	--	--
	--	--	--	--	--	--	--	--
	--	--	--	--	--	--	--	--
	--	--	--	--	--	--	--	--
	--	--	--	--	244206015			
56	cnvi0111244							
1	--	--	--	--	--	--	--	--
	--	--	--	--	--	--	--	--
	--	--	--	--	--	--	--	--
	--	--	--	--	--	--	--	--
	--	--	--	--	244219015			
57	cnvi0111245							
5	--	--	--	--	--	--	--	--
	--	--	--	--	--	--	--	--
	--	--	--	--	--	--	--	--

-- -- -- -- -- -- -- --
-- -- -- -- 70251098
58 cnvi0111246
8 -- -- -- -- -- -- -- --
-- -- -- -- -- -- -- --
-- -- -- -- -- -- -- --
-- -- -- -- -- -- -- --
-- -- -- -- 65504042
59 cnvi0111247
2 -- -- -- -- -- -- -- --
-- -- -- -- -- -- -- --
-- -- -- -- -- -- -- --
-- -- -- -- -- -- -- --
-- -- -- -- 177047858
60 cnvi0111248
7 -- -- -- -- -- -- -- --
-- -- -- -- -- -- -- --
-- -- -- -- -- -- -- --
-- -- -- -- -- -- -- --
-- -- -- -- 155612568
61 cnvi0111249
17 -- -- -- -- -- -- -- --
-- -- -- -- -- -- -- --
-- -- -- -- -- -- -- --
-- -- -- -- -- -- -- --
-- -- -- -- 34618990
62 cnvi0111250
5 -- -- -- -- -- -- -- --
-- -- -- -- -- -- -- --
-- -- -- -- -- -- -- --
-- -- -- -- -- -- -- --
-- -- -- -- 70240634
63 cnvi0111251
11 -- -- -- -- -- -- -- --
-- -- -- -- -- -- -- --
-- -- -- -- -- -- -- --
-- -- -- -- -- -- -- --
-- -- -- -- 2169863
64 cnvi0111252
13 -- -- -- -- -- -- -- --
-- -- -- -- -- -- -- --
-- -- -- -- -- -- -- --
-- -- -- -- -- -- -- --
-- -- -- -- 100636695
65 cnvi0111253
16 -- -- -- -- -- -- -- --
-- -- -- -- -- -- -- --
-- -- -- -- -- -- -- --
-- -- -- -- -- -- -- --
-- -- -- -- 86603675

66	cnvi0111254							
5	--	--	--	--	--	--	--	--
	--	--	--	--	--	--	--	--
	--	--	--	--	--	--	--	--
	--	--	--	--	--	--	--	--
	--	--	--	--	4053144			
67	cnvi0111255							
4	--	--	--	--	--	--	--	--
	--	--	--	--	--	--	--	--
	--	--	--	--	--	--	--	--
	--	--	--	--	--	--	--	--
	--	--	--	--	41745400			
68	cnvi0111256							
16	--	--	--	--	--	--	--	--
	--	--	--	--	--	--	--	--
	--	--	--	--	--	--	--	--
	--	--	--	--	--	--	--	--
	--	--	--	--	86601535			
69	cnvi0111258							
20	--	--	--	--	--	--	--	--
	--	--	--	--	--	--	--	--
	--	--	--	--	--	--	--	--
	--	--	--	--	--	--	--	--
	--	--	--	--	313807			
70	cnvi0111259							
2	--	--	--	--	--	--	--	--
	--	--	--	--	--	--	--	--
	--	--	--	--	--	--	--	--
	--	--	--	--	--	--	--	--
	--	--	--	--	5848110			
71	cnvi0111260							
13	--	--	--	--	--	--	--	--
	--	--	--	--	--	--	--	--
	--	--	--	--	--	--	--	--
	--	--	--	--	--	--	--	--
	--	--	--	--	100639143			
72	cnvi0111261							
1	--	--	--	--	--	--	--	--
	--	--	--	--	--	--	--	--
	--	--	--	--	--	--	--	--
	--	--	--	--	--	--	--	--
	--	--	--	--	244211215			
73	cnvi0111262							
7	--	--	--	--	--	--	--	--
	--	--	--	--	--	--	--	--
	--	--	--	--	--	--	--	--
	--	--	--	--	--	--	--	--
	--	--	--	--	117110389			
74	cnvi0111263							
20	--	--	--	--	--	--	--	--

-- -- -- -- -- -- -- --
-- -- -- -- -- -- -- --
-- -- -- -- -- -- -- --
-- -- -- -- 318687
75 cnvi0111264
2 -- -- -- -- -- -- -- --
-- -- -- -- -- -- -- --
-- -- -- -- -- -- -- --
-- -- -- -- -- -- -- --
-- -- -- -- 177056738
76 cnvi0111265
3 -- -- -- -- -- -- -- --
-- -- -- -- -- -- -- --
-- -- -- -- -- -- -- --
-- -- -- -- -- -- -- --
-- -- -- -- 33489118
77 cnvi0111266
2 -- -- -- -- -- -- -- --
-- -- -- -- -- -- -- --
-- -- -- -- -- -- -- --
-- -- -- -- -- -- -- --
-- -- -- -- 5845250
78 cnvi0111267
16 -- -- -- -- -- -- -- --
-- -- -- -- -- -- -- --
-- -- -- -- -- -- -- --
-- -- -- -- -- -- -- --
-- -- -- -- 3221415
79 cnvi0111268
13 -- -- -- -- -- -- -- --
-- -- -- -- -- -- -- --
-- -- -- -- -- -- -- --
-- -- -- -- -- -- -- --
-- -- -- -- 100641591
80 cnvi0111269
21 -- -- -- -- -- -- -- --
-- -- -- -- -- -- -- --
-- -- -- -- -- -- -- --
-- -- -- -- -- -- -- --
-- -- -- -- 42007642
81 cnvi0111270
7 -- -- -- -- -- -- -- --
-- -- -- -- -- -- -- --
-- -- -- -- -- -- -- --
-- -- -- -- -- -- -- --
-- -- -- -- 155603779
82 cnvi0111271
16 -- -- -- -- -- -- -- --
-- -- -- -- -- -- -- --
-- -- -- -- -- -- -- --

-- -- -- -- -- -- -- --
-- -- -- -- 4241632
83 cnvi0111272
1 -- -- -- -- -- -- -- --
-- -- -- -- -- -- -- --
-- -- -- -- -- -- -- --
-- -- -- -- -- -- -- --
-- -- -- -- 244213815
84 cnvi0111273
13 -- -- -- -- -- -- -- --
-- -- -- -- -- -- -- --
-- -- -- -- -- -- -- --
-- -- -- -- -- -- -- --
-- -- -- -- 79166765
85 cnvi0111274
14 -- -- -- -- -- -- -- --
-- -- -- -- -- -- -- --
-- -- -- -- -- -- -- --
-- -- -- -- -- -- -- --
-- -- -- -- 29246783
86 cnvi0111275
17 -- -- -- -- -- -- -- --
-- -- -- -- -- -- -- --
-- -- -- -- -- -- -- --
-- -- -- -- -- -- -- --
-- -- -- -- 35300753
87 cnvi0111276
5 -- -- -- -- -- -- -- --
-- -- -- -- -- -- -- --
-- -- -- -- -- -- -- --
-- -- -- -- -- -- -- --
-- -- -- -- 70245430
88 cnvi0111277
20 -- -- -- -- -- -- -- --
-- -- -- -- -- -- -- --
-- -- -- -- -- -- -- --
-- -- -- -- -- -- -- --
-- -- -- -- 308927
89 cnvi0111279
1 -- -- -- -- -- -- -- --
-- -- -- -- -- -- -- --
-- -- -- -- -- -- -- --
-- -- -- -- -- -- -- --
-- -- -- -- 4739340
90 cnvi0111280
2 -- -- -- -- -- -- -- --
-- -- -- -- -- -- -- --
-- -- -- -- -- -- -- --
-- -- -- -- -- -- -- --
-- -- -- -- 177050078

91 cnvi0111281
17 -- -- -- -- -- -- -- --
-- -- -- -- -- -- -- --
-- -- -- -- -- -- -- --
-- -- -- -- -- -- -- --
-- -- -- -- 3652744
92 cnvi0111282
17 -- -- -- -- -- -- -- --
-- -- -- -- -- -- -- --
-- -- -- -- -- -- -- --
-- -- -- -- -- -- -- --
-- -- -- -- 34625524
93 cnvi0111283
17 -- -- -- -- -- -- -- --
-- -- -- -- -- -- -- --
-- -- -- -- -- -- -- --
-- -- -- -- -- -- -- --
-- -- -- -- 36351076
94 cnvi0111284
6 -- -- -- -- -- -- -- --
-- -- -- -- -- -- -- --
-- -- -- -- -- -- -- --
-- -- -- -- -- -- -- --
-- -- -- -- 137820386
95 cnvi0111285
5 -- -- -- -- -- -- -- --
-- -- -- -- -- -- -- --
-- -- -- -- -- -- -- --
-- -- -- -- -- -- -- --
-- -- -- -- 4579090
96 cnvi0111286
8 -- -- -- -- -- -- -- --
-- -- -- -- -- -- -- --
-- -- -- -- -- -- -- --
-- -- -- -- -- -- -- --
-- -- -- -- 14708733
97 cnvi0111287
4 -- -- -- -- -- -- -- --
-- -- -- -- -- -- -- --
-- -- -- -- -- -- -- --
-- -- -- -- -- -- -- --
-- -- -- -- 3130032
98 cnvi0111288
8 -- -- -- -- -- -- -- --
-- -- -- -- -- -- -- --
-- -- -- -- -- -- -- --
-- -- -- -- -- -- -- --
-- -- -- -- 9408871
99 cnvi0111289
2 -- -- -- -- -- -- -- --

-- -- -- -- -- -- -- --
-- -- -- -- -- -- -- --
-- -- -- -- -- -- -- --
-- -- -- -- 5839530
100 cnvi0111290
2 -- -- -- -- -- -- -- --
-- -- -- -- -- -- -- --
-- -- -- -- -- -- -- --
-- -- -- -- -- -- -- --
-- -- -- -- 4294541
101 cnvi0111291
5 -- -- -- -- -- -- -- --
-- -- -- -- -- -- -- --
-- -- -- -- -- -- -- --
-- -- -- -- -- -- -- --
-- -- -- -- 70337373
102 cnvi0111293
7 -- -- -- -- -- -- -- --
-- -- -- -- -- -- -- --
-- -- -- -- -- -- -- --
-- -- -- -- -- -- -- --
-- -- -- -- 3639732
103 cnvi0111294
9 -- -- -- -- -- -- -- --
-- -- -- -- -- -- -- --
-- -- -- -- -- -- -- --
-- -- -- -- -- -- -- --
-- -- -- -- 4515784
104 cnvi0111296
2 -- -- -- -- -- -- -- --
-- -- -- -- -- -- -- --
-- -- -- -- -- -- -- --
-- -- -- -- -- -- -- --
-- -- -- -- 5836670
105 cnvi0111297
8 -- -- -- -- -- -- -- --
-- -- -- -- -- -- -- --
-- -- -- -- -- -- -- --
-- -- -- -- -- -- -- --
-- -- -- -- 55373524
106 cnvi0111298
19 -- -- -- -- -- -- -- --
-- -- -- -- -- -- -- --
-- -- -- -- -- -- -- --
-- -- -- -- -- -- -- --
-- -- -- -- 5004910
107 cnvi0111299
18 -- -- -- -- -- -- -- --
-- -- -- -- -- -- -- --
-- -- -- -- -- -- -- --

	--	--	--	--	--	--	--	--
	--	--	--	--	19745338			
108	cnvi0111300							
7	--	--	--	--	--	--	--	--
	--	--	--	--	--	--	--	--
	--	--	--	--	--	--	--	--
	--	--	--	--	--	--	--	--
	--	--	--	--	155609648			
109	cnvi0111301							
21	--	--	--	--	--	--	--	--
	--	--	--	--	--	--	--	--
	--	--	--	--	--	--	--	--
	--	--	--	--	--	--	--	--
	--	--	--	--	39100652			
110	cnvi0111302							
14	--	--	--	--	--	--	--	--
	--	--	--	--	--	--	--	--
	--	--	--	--	--	--	--	--
	--	--	--	--	--	--	--	--
	--	--	--	--	29231103			
111	cnvi0111303							
17	--	--	--	--	--	--	--	--
	--	--	--	--	--	--	--	--
	--	--	--	--	--	--	--	--
	--	--	--	--	--	--	--	--
	--	--	--	--	61990876			
112	cnvi0111304							
5	--	--	--	--	--	--	--	--
	--	--	--	--	--	--	--	--
	--	--	--	--	--	--	--	--
	--	--	--	--	--	--	--	--
	--	--	--	--	70332137			
113	cnvi0111305							
22	--	--	--	--	--	--	--	--
	--	--	--	--	--	--	--	--
	--	--	--	--	--	--	--	--
	--	--	--	--	--	--	--	--
	--	--	--	--	33944903			
114	cnvi0111306							
17	--	--	--	--	--	--	--	--
	--	--	--	--	--	--	--	--
	--	--	--	--	--	--	--	--
	--	--	--	--	--	--	--	--
	--	--	--	--	36347393			
115	cnvi0111308							
6	--	--	--	--	--	--	--	--
	--	--	--	--	--	--	--	--
	--	--	--	--	--	--	--	--
	--	--	--	--	--	--	--	--
	--	--	--	--	137822586			

116 cnvi0111309
1 -- -- -- -- -- -- -- --
-- -- -- -- -- -- -- --
-- -- -- -- -- -- -- --
-- -- -- -- -- -- -- --
-- -- -- -- 244216415
117 cnvi0111310
14 -- -- -- -- -- -- -- --
-- -- -- -- -- -- -- --
-- -- -- -- -- -- -- --
-- -- -- -- -- -- -- --
-- -- -- -- 29242303
118 cnvi0111311
17 -- -- -- -- -- -- -- --
-- -- -- -- -- -- -- --
-- -- -- -- -- -- -- --
-- -- -- -- -- -- -- --
-- -- -- -- 61997296
119 cnvi0111312
5 -- -- -- -- -- -- -- --
-- -- -- -- -- -- -- --
-- -- -- -- -- -- -- --
-- -- -- -- -- -- -- --
-- -- -- -- 70342847
120 cnvi0111314
5 -- -- -- -- -- -- -- --
-- -- -- -- -- -- -- --
-- -- -- -- -- -- -- --
-- -- -- -- -- -- -- --
-- -- -- -- 70353557
121 cnvi0111315
17 -- -- -- -- -- -- -- --
-- -- -- -- -- -- -- --
-- -- -- -- -- -- -- --
-- -- -- -- -- -- -- --
-- -- -- -- 61995156
122 cnvi0111317
13 -- -- -- -- -- -- -- --
-- -- -- -- -- -- -- --
-- -- -- -- -- -- -- --
-- -- -- -- -- -- -- --
-- -- -- -- 36048663
123 cnvi0111318
4 -- -- -- -- -- -- -- --
-- -- -- -- -- -- -- --
-- -- -- -- -- -- -- --
-- -- -- -- -- -- -- --
-- -- -- -- 41742920
124 cnvi0111319
8 -- -- -- -- -- -- -- --

	--	--	--	--	--	--	--	--
	--	--	--	--	--	--	--	--
	--	--	--	--	--	--	--	--
	--	--	--	--	55375645			
125	cnvi0111320							
16	--	--	--	--	--	--	--	--
	--	--	--	--	--	--	--	--
	--	--	--	--	--	--	--	--
	--	--	--	--	--	--	--	--
	--	--	--	--	86597245			
126	cnvi0111321							
1	--	--	--	--	--	--	--	--
	--	--	--	--	--	--	--	--
	--	--	--	--	--	--	--	--
	--	--	--	--	--	--	--	--
	--	--	--	--	47877630			
127	cnvi0111322							
18	--	--	--	--	--	--	--	--
	--	--	--	--	--	--	--	--
	--	--	--	--	--	--	--	--
	--	--	--	--	--	--	--	--
	--	--	--	--	4317330			
128	cnvi0111323							
2	--	--	--	--	--	--	--	--
	--	--	--	--	--	--	--	--
	--	--	--	--	--	--	--	--
	--	--	--	--	--	--	--	--
	--	--	--	--	237073488			
129	cnvi0111325							
13	--	--	--	--	--	--	--	--
	--	--	--	--	--	--	--	--
	--	--	--	--	--	--	--	--
	--	--	--	--	--	--	--	--
	--	--	--	--	100646487			
130	cnvi0111326							
16	--	--	--	--	--	--	--	--
	--	--	--	--	--	--	--	--
	--	--	--	--	--	--	--	--
	--	--	--	--	--	--	--	--
	--	--	--	--	86605815			
131	cnvi0111328							
14	--	--	--	--	--	--	--	--
	--	--	--	--	--	--	--	--
	--	--	--	--	--	--	--	--
	--	--	--	--	--	--	--	--
	--	--	--	--	29237823			
132	cnvi0111330							
13	--	--	--	--	--	--	--	--
	--	--	--	--	--	--	--	--
	--	--	--	--	--	--	--	--

-- -- -- -- -- -- -- --
-- -- -- -- 36053103
133 cnvi0111332
10 -- -- -- -- -- -- -- --
-- -- -- -- -- -- -- --
-- -- -- -- -- -- -- --
-- -- -- -- -- -- -- --
-- -- -- -- 47760066
134 cnvi0111333
10 -- -- -- -- -- -- -- --
-- -- -- -- -- -- -- --
-- -- -- -- -- -- -- --
-- -- -- -- -- -- -- --
-- -- -- -- 94439996
135 cnvi0111334
18 -- -- -- -- -- -- -- --
-- -- -- -- -- -- -- --
-- -- -- -- -- -- -- --
-- -- -- -- -- -- -- --
-- -- -- -- 19740074
136 cnvi0111335
2 -- -- -- -- -- -- -- --
-- -- -- -- -- -- -- --
-- -- -- -- -- -- -- --
-- -- -- -- -- -- -- --
-- -- -- -- 177045638
137 cnvi0111336
1 -- -- -- -- -- -- -- --
-- -- -- -- -- -- -- --
-- -- -- -- -- -- -- --
-- -- -- -- -- -- -- --
-- -- -- -- 47873270
138 cnvi0111337
10 -- -- -- -- -- -- -- --
-- -- -- -- -- -- -- --
-- -- -- -- -- -- -- --
-- -- -- -- -- -- -- --
-- -- -- -- 47741321
139 cnvi0111338
8 -- -- -- -- -- -- -- --
-- -- -- -- -- -- -- --
-- -- -- -- -- -- -- --
-- -- -- -- -- -- -- --
-- -- -- -- 55369080
140 cnvi0111339
8 -- -- -- -- -- -- -- --
-- -- -- -- -- -- -- --
-- -- -- -- -- -- -- --
-- -- -- -- -- -- -- --
-- -- -- -- 117636293

141 cnvi0111340
10 -- -- -- -- -- -- -- --
-- -- -- -- -- -- -- --
-- -- -- -- -- -- -- --
-- -- -- -- -- -- -- --
-- -- -- -- 3365477
142 cnvi0111341
14 -- -- -- -- -- -- -- --
-- -- -- -- -- -- -- --
-- -- -- -- -- -- -- --
-- -- -- -- -- -- -- --
-- -- -- -- 29233343
143 cnvi0111342
11 -- -- -- -- -- -- -- --
-- -- -- -- -- -- -- --
-- -- -- -- -- -- -- --
-- -- -- -- -- -- -- --
-- -- -- -- 4236788
144 cnvi0111343
10 -- -- -- -- -- -- -- --
-- -- -- -- -- -- -- --
-- -- -- -- -- -- -- --
-- -- -- -- -- -- -- --
-- -- -- -- 47736920
145 cnvi0111344
14 -- -- -- -- -- -- -- --
-- -- -- -- -- -- -- --
-- -- -- -- -- -- -- --
-- -- -- -- -- -- -- --
-- -- -- -- 29228863
146 cnvi0111345
14 -- -- -- -- -- -- -- --
-- -- -- -- -- -- -- --
-- -- -- -- -- -- -- --
-- -- -- -- -- -- -- --
-- -- -- -- 29240063
147 cnvi0111346
5 -- -- -- -- -- -- -- --
-- -- -- -- -- -- -- --
-- -- -- -- -- -- -- --
-- -- -- -- -- -- -- --
-- -- -- -- 70348321
148 cnvi0111347
4 -- -- -- -- -- -- -- --
-- -- -- -- -- -- -- --
-- -- -- -- -- -- -- --
-- -- -- -- -- -- -- --
-- -- -- -- 3668906
149 cnvi0111348
13 -- -- -- -- -- -- -- --

	--	--	--	--	--	--	--	--
	--	--	--	--	--	--	--	--
	--	--	--	--	--	--	--	--
	--	--	--	--	79169205			
150	cnvi0111349							
8	--	--	--	--	--	--	--	--
	--	--	--	--	--	--	--	--
	--	--	--	--	--	--	--	--
	--	--	--	--	--	--	--	--
	--	--	--	--	65497082			
151	cnvi0111350							
17	--	--	--	--	--	--	--	--
	--	--	--	--	--	--	--	--
	--	--	--	--	--	--	--	--
	--	--	--	--	--	--	--	--
	--	--	--	--	36357962			
152	cnvi0111351							
13	--	--	--	--	--	--	--	--
	--	--	--	--	--	--	--	--
	--	--	--	--	--	--	--	--
	--	--	--	--	--	--	--	--
	--	--	--	--	36046443			
153	cnvi0111352							
11	--	--	--	--	--	--	--	--
	--	--	--	--	--	--	--	--
	--	--	--	--	--	--	--	--
	--	--	--	--	--	--	--	--
	--	--	--	--	69614918			
154	cnvi0111353							
4	--	--	--	--	--	--	--	--
	--	--	--	--	--	--	--	--
	--	--	--	--	--	--	--	--
	--	--	--	--	--	--	--	--
	--	--	--	--	41740440			
155	cnvi0111354							
7	--	--	--	--	--	--	--	--
	--	--	--	--	--	--	--	--
	--	--	--	--	--	--	--	--
	--	--	--	--	--	--	--	--
	--	--	--	--	147870275			
156	cnvi0111355							
8	--	--	--	--	--	--	--	--
	--	--	--	--	--	--	--	--
	--	--	--	--	--	--	--	--
	--	--	--	--	--	--	--	--
	--	--	--	--	136681120			
157	cnvi0111356							
8	--	--	--	--	--	--	--	--
	--	--	--	--	--	--	--	--
	--	--	--	--	--	--	--	--

	--	--	--	--	--	--	--	--
	--	--	--	--	55371302			
158	cnvi0111357							
17	--	--	--	--	--	--	--	--
	--	--	--	--	--	--	--	--
	--	--	--	--	--	--	--	--
	--	--	--	--	--	--	--	--
	--	--	--	--	36353898			
159	cnvi0111358							
8	--	--	--	--	--	--	--	--
	--	--	--	--	--	--	--	--
	--	--	--	--	--	--	--	--
	--	--	--	--	--	--	--	--
	--	--	--	--	65485482			
160	cnvi0111359							
1	--	--	--	--	--	--	--	--
	--	--	--	--	--	--	--	--
	--	--	--	--	--	--	--	--
	--	--	--	--	--	--	--	--
	--	--	--	--	47879810			
161	cnvi0111360							
17	--	--	--	--	--	--	--	--
	--	--	--	--	--	--	--	--
	--	--	--	--	--	--	--	--
	--	--	--	--	--	--	--	--
	--	--	--	--	34621366			
162	cnvi0111362							
17	--	--	--	--	--	--	--	--
	--	--	--	--	--	--	--	--
	--	--	--	--	--	--	--	--
	--	--	--	--	--	--	--	--
	--	--	--	--	34628791			
163	cnvi0111363							
3	--	--	--	--	--	--	--	--
	--	--	--	--	--	--	--	--
	--	--	--	--	--	--	--	--
	--	--	--	--	--	--	--	--
	--	--	--	--	4381008			
164	cnvi0111366							
16	--	--	--	--	--	--	--	--
	--	--	--	--	--	--	--	--
	--	--	--	--	--	--	--	--
	--	--	--	--	--	--	--	--
	--	--	--	--	86607955			
165	cnvi0111368							
17	--	--	--	--	--	--	--	--
	--	--	--	--	--	--	--	--
	--	--	--	--	--	--	--	--
	--	--	--	--	--	--	--	--
	--	--	--	--	34630969			

166	cnvi0111369							
2	--	--	--	--	--	--	--	--
	--	--	--	--	--	--	--	--
	--	--	--	--	--	--	--	--
	--	--	--	--	--	--	--	--
	--	--	--	--	5825230			
167	cnvi0111370							
2	--	--	--	--	--	--	--	--
	--	--	--	--	--	--	--	--
	--	--	--	--	--	--	--	--
	--	--	--	--	--	--	--	--
	--	--	--	--	5830950			
168	cnvi0111371							
6	--	--	--	--	--	--	--	--
	--	--	--	--	--	--	--	--
	--	--	--	--	--	--	--	--
	--	--	--	--	--	--	--	--
	--	--	--	--	137815971			
169	cnvi0111372							
19	--	--	--	--	--	--	--	--
	--	--	--	--	--	--	--	--
	--	--	--	--	--	--	--	--
	--	--	--	--	--	--	--	--
	--	--	--	--	4996784			
170	cnvi0111373							
10	--	--	--	--	--	--	--	--
	--	--	--	--	--	--	--	--
	--	--	--	--	--	--	--	--
	--	--	--	--	--	--	--	--
	--	--	--	--	47756480			
171	cnvi0111374							
20	--	--	--	--	--	--	--	--
	--	--	--	--	--	--	--	--
	--	--	--	--	--	--	--	--
	--	--	--	--	--	--	--	--
	--	--	--	--	311367			
172	cnvi0111375							
5	--	--	--	--	--	--	--	--
	--	--	--	--	--	--	--	--
	--	--	--	--	--	--	--	--
	--	--	--	--	--	--	--	--
	--	--	--	--	70235620			
173	cnvi0111376							
20	--	--	--	--	--	--	--	--
	--	--	--	--	--	--	--	--
	--	--	--	--	--	--	--	--
	--	--	--	--	--	--	--	--
	--	--	--	--	21680592			
174	cnvi0111377							
2	--	--	--	--	--	--	--	--

-- -- -- -- -- -- -- --
-- -- -- -- -- -- -- --
-- -- -- -- -- -- -- --
-- -- -- -- 5842390
175 cnvi0111378
10 -- -- -- -- -- -- -- --
-- -- -- -- -- -- -- --
-- -- -- -- -- -- -- --
-- -- -- -- -- -- -- --
-- -- -- -- 47744581
176 cnvi0111379
8 -- -- -- -- -- -- -- --
-- -- -- -- -- -- -- --
-- -- -- -- -- -- -- --
-- -- -- -- -- -- -- --
-- -- -- -- 65490122
177 cnvi0111380
18 -- -- -- -- -- -- -- --
-- -- -- -- -- -- -- --
-- -- -- -- -- -- -- --
-- -- -- -- -- -- -- --
-- -- -- -- 4677763
178 cnvi0111381
2 -- -- -- -- -- -- -- --
-- -- -- -- -- -- -- --
-- -- -- -- -- -- -- --
-- -- -- -- -- -- -- --
-- -- -- -- 237082386
179 cnvi0111382
5 -- -- -- -- -- -- -- --
-- -- -- -- -- -- -- --
-- -- -- -- -- -- -- --
-- -- -- -- -- -- -- --
-- -- -- -- 70231260
180 cnvi0111383
20 -- -- -- -- -- -- -- --
-- -- -- -- -- -- -- --
-- -- -- -- -- -- -- --
-- -- -- -- -- -- -- --
-- -- -- -- 21677572
181 cnvi0111384
8 -- -- -- -- -- -- -- --
-- -- -- -- -- -- -- --
-- -- -- -- -- -- -- --
-- -- -- -- -- -- -- --
-- -- -- -- 55380089
182 cnvi0111385
17 -- -- -- -- -- -- -- --
-- -- -- -- -- -- -- --
-- -- -- -- -- -- -- --

	--	--	--	--	--	--	--	--
	--	--	--	--	34618594			
183	cnvi0111386							
17	--	--	--	--	--	--	--	--
	--	--	--	--	--	--	--	--
	--	--	--	--	--	--	--	--
	--	--	--	--	--	--	--	--
	--	--	--	--	35298173			
184	cnvi0117300							
9	--	--	--	--	--	--	--	--
	--	--	--	--	--	--	--	--
	--	--	--	--	--	--	--	--
	--	--	--	--	--	--	--	--
	--	--	--	--	40695541			
185	cnvi0117301							
9	--	--	--	--	--	--	--	--
	--	--	--	--	--	--	--	--
	--	--	--	--	--	--	--	--
	--	--	--	--	--	--	--	--
	--	--	--	--	41491154			
186	cnvi0117303							
8	--	--	--	--	--	--	--	--
	--	--	--	--	--	--	--	--
	--	--	--	--	--	--	--	--
	--	--	--	--	--	--	--	--
	--	--	--	--	95068824			
187	cnvi0117307							
11	--	--	--	--	--	--	--	--
	--	--	--	--	--	--	--	--
	--	--	--	--	--	--	--	--
	--	--	--	--	--	--	--	--
	--	--	--	--	4243900			
188	cnvi0117308							
16	--	--	--	--	--	--	--	--
	--	--	--	--	--	--	--	--
	--	--	--	--	--	--	--	--
	--	--	--	--	--	--	--	--
	--	--	--	--	30933259			
189	cnvi0117309							
7	--	--	--	--	--	--	--	--
	--	--	--	--	--	--	--	--
	--	--	--	--	--	--	--	--
	--	--	--	--	--	--	--	--
	--	--	--	--	19323727			
190	cnvi0117311							
17	--	--	--	--	--	--	--	--
	--	--	--	--	--	--	--	--
	--	--	--	--	--	--	--	--
	--	--	--	--	--	--	--	--
	--	--	--	--	31235921			

191	cnvi0117313							
17	--	--	--	--	--	--	--	--
	--	--	--	--	--	--	--	--
	--	--	--	--	--	--	--	--
	--	--	--	--	--	--	--	--
	--	--	--	--	26841469			
192	cnvi0117315							
4	--	--	--	--	--	--	--	--
	--	--	--	--	--	--	--	--
	--	--	--	--	--	--	--	--
	--	--	--	--	--	--	--	--
	--	--	--	--	147550088			
193	cnvi0117317							
4	--	--	--	--	--	--	--	--
	--	--	--	--	--	--	--	--
	--	--	--	--	--	--	--	--
	--	--	--	--	--	--	--	--
	--	--	--	--	11280440			
194	cnvi0117318							
17	--	--	--	--	--	--	--	--
	--	--	--	--	--	--	--	--
	--	--	--	--	--	--	--	--
	--	--	--	--	--	--	--	--
	--	--	--	--	70891250			
195	cnvi0117320							
18	--	--	--	--	--	--	--	--
	--	--	--	--	--	--	--	--
	--	--	--	--	--	--	--	--
	--	--	--	--	--	--	--	--
	--	--	--	--	4676938			
196	cnvi0117324							
1	--	--	--	--	--	--	--	--
	--	--	--	--	--	--	--	--
	--	--	--	--	--	--	--	--
	--	--	--	--	--	--	--	--
	--	--	--	--	176033377			
197	cnvi0117325							
9	--	--	--	--	--	--	--	--
	--	--	--	--	--	--	--	--
	--	--	--	--	--	--	--	--
	--	--	--	--	--	--	--	--
	--	--	--	--	40938056			
198	cnvi0117326							
9	--	--	--	--	--	--	--	--
	--	--	--	--	--	--	--	--
	--	--	--	--	--	--	--	--
	--	--	--	--	--	--	--	--
	--	--	--	--	42151828			
199	cnvi0117327							
4	--	--	--	--	--	--	--	--

	--	--	--	--	--	--	--	--
	--	--	--	--	--	--	--	--
	--	--	--	--	--	--	--	--
	--	--	--	--	13532666			
200	cnvi0117328							
8	--	--	--	--	--	--	--	--
	--	--	--	--	--	--	--	--
	--	--	--	--	--	--	--	--
	--	--	--	--	--	--	--	--
	--	--	--	--	112136836			
201	cnvi0117329							
9	--	--	--	--	--	--	--	--
	--	--	--	--	--	--	--	--
	--	--	--	--	--	--	--	--
	--	--	--	--	--	--	--	--
	--	--	--	--	42165317			
202	cnvi0117330							
9	--	--	--	--	--	--	--	--
	--	--	--	--	--	--	--	--
	--	--	--	--	--	--	--	--
	--	--	--	--	--	--	--	--
	--	--	--	--	42227313			
203	cnvi0117334							
9	--	--	--	--	--	--	--	--
	--	--	--	--	--	--	--	--
	--	--	--	--	--	--	--	--
	--	--	--	--	--	--	--	--
	--	--	--	--	41835841			
204	cnvi0117335							
2	--	--	--	--	--	--	--	--
	--	--	--	--	--	--	--	--
	--	--	--	--	--	--	--	--
	--	--	--	--	--	--	--	--
	--	--	--	--	5851256			
205	cnvi0117336							
7	--	--	--	--	--	--	--	--
	--	--	--	--	--	--	--	--
	--	--	--	--	--	--	--	--
	--	--	--	--	--	--	--	--
	--	--	--	--	96639702			
206	cnvi0117338							
2	--	--	--	--	--	--	--	--
	--	--	--	--	--	--	--	--
	--	--	--	--	--	--	--	--
	--	--	--	--	--	--	--	--
	--	--	--	--	172940443			
207	cnvi0117339							
9	--	--	--	--	--	--	--	--
	--	--	--	--	--	--	--	--
	--	--	--	--	--	--	--	--

-- -- -- -- -- -- -- --
-- -- -- -- 41887501
208 cnvi0117340
9 -- -- -- -- -- -- -- --
-- -- -- -- -- -- -- --
-- -- -- -- -- -- -- --
-- -- -- -- -- -- -- --
-- -- -- -- 40879795
209 cnvi0117341
15 -- -- -- -- -- -- -- --
-- -- -- -- -- -- -- --
-- -- -- -- -- -- -- --
-- -- -- -- -- -- -- --
-- -- -- -- 76408095
210 cnvi0117342
1 -- -- -- -- -- -- -- --
-- -- -- -- -- -- -- --
-- -- -- -- -- -- -- --
-- -- -- -- -- -- -- --
-- -- -- -- 91167807
211 cnvi0117343
17 -- -- -- -- -- -- -- --
-- -- -- -- -- -- -- --
-- -- -- -- -- -- -- --
-- -- -- -- -- -- -- --
-- -- -- -- 47911418
212 cnvi0117344
6 -- -- -- -- -- -- -- --
-- -- -- -- -- -- -- --
-- -- -- -- -- -- -- --
-- -- -- -- -- -- -- --
-- -- -- -- 160380131
213 cnvi0117345
14 -- -- -- -- -- -- -- --
-- -- -- -- -- -- -- --
-- -- -- -- -- -- -- --
-- -- -- -- -- -- -- --
-- -- -- -- 54406750
214 cnvi0117346
9 -- -- -- -- -- -- -- --
-- -- -- -- -- -- -- --
-- -- -- -- -- -- -- --
-- -- -- -- -- -- -- --
-- -- -- -- 42158429
215 cnvi0117349
5 -- -- -- -- -- -- -- --
-- -- -- -- -- -- -- --
-- -- -- -- -- -- -- --
-- -- -- -- -- -- -- --
-- -- -- -- 54665308

216 cnvi0117350
8 -- -- -- -- -- -- -- --
-- -- -- -- -- -- -- --
-- -- -- -- -- -- -- --
-- -- -- -- -- -- -- --
-- -- -- -- 112130824
217 cnvi0117352
16 -- -- -- -- -- -- -- --
-- -- -- -- -- -- -- --
-- -- -- -- -- -- -- --
-- -- -- -- -- -- -- --
-- -- -- -- 13543921
218 cnvi0117353
8 -- -- -- -- -- -- -- --
-- -- -- -- -- -- -- --
-- -- -- -- -- -- -- --
-- -- -- -- -- -- -- --
-- -- -- -- 14728885
219 cnvi0117357
22 -- -- -- -- -- -- -- --
-- -- -- -- -- -- -- --
-- -- -- -- -- -- -- --
-- -- -- -- -- -- -- --
-- -- -- -- 25529587
220 cnvi0117358
9 -- -- -- -- -- -- -- --
-- -- -- -- -- -- -- --
-- -- -- -- -- -- -- --
-- -- -- -- -- -- -- --
-- -- -- -- 41523872
221 cnvi0117359
9 -- -- -- -- -- -- -- --
-- -- -- -- -- -- -- --
-- -- -- -- -- -- -- --
-- -- -- -- -- -- -- --
-- -- -- -- 41988238
222 cnvi0117361
9 -- -- -- -- -- -- -- --
-- -- -- -- -- -- -- --
-- -- -- -- -- -- -- --
-- -- -- -- -- -- -- --
-- -- -- -- 40849947
223 cnvi0117362
9 -- -- -- -- -- -- -- --
-- -- -- -- -- -- -- --
-- -- -- -- -- -- -- --
-- -- -- -- -- -- -- --
-- -- -- -- 40910504
224 cnvi0117363
14 -- -- -- -- -- -- -- --

-- -- -- -- -- -- -- --
-- -- -- -- -- -- -- --
-- -- -- -- -- -- -- --
-- -- -- -- 49230250
225 cnvi0117364
14 -- -- -- -- -- -- -- --
-- -- -- -- -- -- -- --
-- -- -- -- -- -- -- --
-- -- -- -- -- -- -- --
-- -- -- -- 88392376
226 cnvi0117366
9 -- -- -- -- -- -- -- --
-- -- -- -- -- -- -- --
-- -- -- -- -- -- -- --
-- -- -- -- -- -- -- --
-- -- -- -- 40920549
227 cnvi0117367
1 -- -- -- -- -- -- -- --
-- -- -- -- -- -- -- --
-- -- -- -- -- -- -- --
-- -- -- -- -- -- -- --
-- -- -- -- 55973886
228 cnvi0117368
9 -- -- -- -- -- -- -- --
-- -- -- -- -- -- -- --
-- -- -- -- -- -- -- --
-- -- -- -- -- -- -- --
-- -- -- -- 42171057
229 cnvi0117369
9 -- -- -- -- -- -- -- --
-- -- -- -- -- -- -- --
-- -- -- -- -- -- -- --
-- -- -- -- -- -- -- --
-- -- -- -- 41975610
230 cnvi0117370
13 -- -- -- -- -- -- -- --
-- -- -- -- -- -- -- --
-- -- -- -- -- -- -- --
-- -- -- -- -- -- -- --
-- -- -- -- 36038673
231 cnvi0117371
20 -- -- -- -- -- -- -- --
-- -- -- -- -- -- -- --
-- -- -- -- -- -- -- --
-- -- -- -- -- -- -- --
-- -- -- -- 41502950
232 cnvi0117372
9 -- -- -- -- -- -- -- --
-- -- -- -- -- -- -- --
-- -- -- -- -- -- -- --

-- -- -- -- -- -- -- --
-- -- -- -- 41981637
233 cnvi0117373
9 -- -- -- -- -- -- -- --
-- -- -- -- -- -- -- --
-- -- -- -- -- -- -- --
-- -- -- -- -- -- -- --
-- -- -- -- 40854252
234 cnvi0117375
3 -- -- -- -- -- -- -- --
-- -- -- -- -- -- -- --
-- -- -- -- -- -- -- --
-- -- -- -- -- -- -- --
-- -- -- -- 121917310
235 cnvi0117376
9 -- -- -- -- -- -- -- --
-- -- -- -- -- -- -- --
-- -- -- -- -- -- -- --
-- -- -- -- -- -- -- --
-- -- -- -- 42254291
236 cnvi0117377
21 -- -- -- -- -- -- -- --
-- -- -- -- -- -- -- --
-- -- -- -- -- -- -- --
-- -- -- -- -- -- -- --
-- -- -- -- 39801044
237 cnvi0117378
9 -- -- -- -- -- -- -- --
-- -- -- -- -- -- -- --
-- -- -- -- -- -- -- --
-- -- -- -- -- -- -- --
-- -- -- -- 41905869
238 cnvi0117379
18 -- -- -- -- -- -- -- --
-- -- -- -- -- -- -- --
-- -- -- -- -- -- -- --
-- -- -- -- -- -- -- --
-- -- -- -- 63407488
239 cnvi0117380
2 -- -- -- -- -- -- -- --
-- -- -- -- -- -- -- --
-- -- -- -- -- -- -- --
-- -- -- -- -- -- -- --
-- -- -- -- 163791754
240 cnvi0117381
22 -- -- -- -- -- -- -- --
-- -- -- -- -- -- -- --
-- -- -- -- -- -- -- --
-- -- -- -- -- -- -- --
-- -- -- -- 31497875

241 cnvi0117382
7 -- -- -- -- -- -- -- --
-- -- -- -- -- -- -- --
-- -- -- -- -- -- -- --
-- -- -- -- -- -- -- --
-- -- -- -- 101368616

242 cnvi0117383
7 -- -- -- -- -- -- -- --
-- -- -- -- -- -- -- --
-- -- -- -- -- -- -- --
-- -- -- -- -- -- -- --
-- -- -- -- 20829994

243 cnvi0117386
22 -- -- -- -- -- -- -- --
-- -- -- -- -- -- -- --
-- -- -- -- -- -- -- --
-- -- -- -- -- -- -- --
-- -- -- -- 17920000

244 cnvi0117388
17 -- -- -- -- -- -- -- --
-- -- -- -- -- -- -- --
-- -- -- -- -- -- -- --
-- -- -- -- -- -- -- --
-- -- -- -- 3647965

245 cnvi0117389
9 -- -- -- -- -- -- -- --
-- -- -- -- -- -- -- --
-- -- -- -- -- -- -- --
-- -- -- -- -- -- -- --
-- -- -- -- 42236784

246 cnvi0117390
2 -- -- -- -- -- -- -- --
-- -- -- -- -- -- -- --
-- -- -- -- -- -- -- --
-- -- -- -- -- -- -- --
-- -- -- -- 12831455

247 cnvi0117391
12 -- -- -- -- -- -- -- --
-- -- -- -- -- -- -- --
-- -- -- -- -- -- -- --
-- -- -- -- -- -- -- --
-- -- -- -- 42274820

248 cnvi0117393
9 -- -- -- -- -- -- -- --
-- -- -- -- -- -- -- --
-- -- -- -- -- -- -- --
-- -- -- -- -- -- -- --
-- -- -- -- 42231331

249 cnvi0117394
16 -- -- -- -- -- -- -- --

	--	--	--	--	--	--	--	--
	--	--	--	--	--	--	--	--
	--	--	--	--	--	--	--	--
	--	--	--	--	16786098			
250	cnvi0117396							
2	--	--	--	--	--	--	--	--
	--	--	--	--	--	--	--	--
	--	--	--	--	--	--	--	--
	--	--	--	--	--	--	--	--
	--	--	--	--	237065317			
251	cnvi0117398							
18	--	--	--	--	--	--	--	--
	--	--	--	--	--	--	--	--
	--	--	--	--	--	--	--	--
	--	--	--	--	--	--	--	--
	--	--	--	--	4668972			
252	cnvi0117401							
3	--	--	--	--	--	--	--	--
	--	--	--	--	--	--	--	--
	--	--	--	--	--	--	--	--
	--	--	--	--	--	--	--	--
	--	--	--	--	33451064			
253	cnvi0117402							
5	--	--	--	--	--	--	--	--
	--	--	--	--	--	--	--	--
	--	--	--	--	--	--	--	--
	--	--	--	--	--	--	--	--
	--	--	--	--	21262652			
254	cnvi0117403							
9	--	--	--	--	--	--	--	--
	--	--	--	--	--	--	--	--
	--	--	--	--	--	--	--	--
	--	--	--	--	--	--	--	--
	--	--	--	--	40904190			
255	cnvi0117404							
9	--	--	--	--	--	--	--	--
	--	--	--	--	--	--	--	--
	--	--	--	--	--	--	--	--
	--	--	--	--	--	--	--	--
	--	--	--	--	41870281			
256	cnvi0117405							
7	--	--	--	--	--	--	--	--
	--	--	--	--	--	--	--	--
	--	--	--	--	--	--	--	--
	--	--	--	--	--	--	--	--
	--	--	--	--	40479355			
257	cnvi0117406							
1	--	--	--	--	--	--	--	--
	--	--	--	--	--	--	--	--
	--	--	--	--	--	--	--	--

	--	--	--	--	--	--	--	--
	--	--	--	--	20265488			
258	cnvi0117407							
1	--	--	--	--	--	--	--	--
	--	--	--	--	--	--	--	--
	--	--	--	--	--	--	--	--
	--	--	--	--	--	--	--	--
	--	--	--	--	207233377			
259	cnvi0117408							
15	--	--	--	--	--	--	--	--
	--	--	--	--	--	--	--	--
	--	--	--	--	--	--	--	--
	--	--	--	--	--	--	--	--
	--	--	--	--	93799559			
260	cnvi0117410							
6	--	--	--	--	--	--	--	--
	--	--	--	--	--	--	--	--
	--	--	--	--	--	--	--	--
	--	--	--	--	--	--	--	--
	--	--	--	--	15523609			
261	cnvi0117414							
1	--	--	--	--	--	--	--	--
	--	--	--	--	--	--	--	--
	--	--	--	--	--	--	--	--
	--	--	--	--	--	--	--	--
	--	--	--	--	87784151			
262	cnvi0117415							
2	--	--	--	--	--	--	--	--
	--	--	--	--	--	--	--	--
	--	--	--	--	--	--	--	--
	--	--	--	--	--	--	--	--
	--	--	--	--	144986897			
263	cnvi0117416							
17	--	--	--	--	--	--	--	--
	--	--	--	--	--	--	--	--
	--	--	--	--	--	--	--	--
	--	--	--	--	--	--	--	--
	--	--	--	--	7502316			
264	cnvi0117417							
16	--	--	--	--	--	--	--	--
	--	--	--	--	--	--	--	--
	--	--	--	--	--	--	--	--
	--	--	--	--	--	--	--	--
	--	--	--	--	64972222			
265	cnvi0117419							
9	--	--	--	--	--	--	--	--
	--	--	--	--	--	--	--	--
	--	--	--	--	--	--	--	--
	--	--	--	--	--	--	--	--
	--	--	--	--	99164667			

266 cnvi0117421
1 -- -- -- -- -- -- -- --
-- -- -- -- -- -- -- --
-- -- -- -- -- -- -- --
-- -- -- -- -- -- -- --
-- -- -- -- 155907925
267 cnvi0117422
9 -- -- -- -- -- -- -- --
-- -- -- -- -- -- -- --
-- -- -- -- -- -- -- --
-- -- -- -- -- -- -- --
-- -- -- -- 42209802
268 cnvi0117423
20 -- -- -- -- -- -- -- --
-- -- -- -- -- -- -- --
-- -- -- -- -- -- -- --
-- -- -- -- -- -- -- --
-- -- -- -- 31387917
269 cnvi0117424
12 -- -- -- -- -- -- -- --
-- -- -- -- -- -- -- --
-- -- -- -- -- -- -- --
-- -- -- -- -- -- -- --
-- -- -- -- 112319835
270 cnvi0117425
8 -- -- -- -- -- -- -- --
-- -- -- -- -- -- -- --
-- -- -- -- -- -- -- --
-- -- -- -- -- -- -- --
-- -- -- -- 131918740
271 cnvi0117427
17 -- -- -- -- -- -- -- --
-- -- -- -- -- -- -- --
-- -- -- -- -- -- -- --
-- -- -- -- -- -- -- --
-- -- -- -- 31455980
272 cnvi0117429
9 -- -- -- -- -- -- -- --
-- -- -- -- -- -- -- --
-- -- -- -- -- -- -- --
-- -- -- -- -- -- -- --
-- -- -- -- 40767865
273 cnvi0117430
4 -- -- -- -- -- -- -- --
-- -- -- -- -- -- -- --
-- -- -- -- -- -- -- --
-- -- -- -- -- -- -- --
-- -- -- -- 1723887
274 cnvi0117431
10 -- -- -- -- -- -- -- --

	--	--	--	--	--	--	--	--
	--	--	--	--	--	--	--	--
	--	--	--	--	--	--	--	--
	--	--	--	--	77148651			
275	cnvi0117432							
14	--	--	--	--	--	--	--	--
	--	--	--	--	--	--	--	--
	--	--	--	--	--	--	--	--
	--	--	--	--	--	--	--	--
	--	--	--	--	34427595			
276	cnvi0117433							
8	--	--	--	--	--	--	--	--
	--	--	--	--	--	--	--	--
	--	--	--	--	--	--	--	--
	--	--	--	--	--	--	--	--
	--	--	--	--	19001200			
277	cnvi0117439							
9	--	--	--	--	--	--	--	--
	--	--	--	--	--	--	--	--
	--	--	--	--	--	--	--	--
	--	--	--	--	--	--	--	--
	--	--	--	--	40738304			
278	cnvi0117440							
5	--	--	--	--	--	--	--	--
	--	--	--	--	--	--	--	--
	--	--	--	--	--	--	--	--
	--	--	--	--	--	--	--	--
	--	--	--	--	25063835			
279	cnvi0117441							
21	--	--	--	--	--	--	--	--
	--	--	--	--	--	--	--	--
	--	--	--	--	--	--	--	--
	--	--	--	--	--	--	--	--
	--	--	--	--	16329437			
280	cnvi0117442							
1	--	--	--	--	--	--	--	--
	--	--	--	--	--	--	--	--
	--	--	--	--	--	--	--	--
	--	--	--	--	--	--	--	--
	--	--	--	--	156074498			
281	cnvi0117443							
9	--	--	--	--	--	--	--	--
	--	--	--	--	--	--	--	--
	--	--	--	--	--	--	--	--
	--	--	--	--	--	--	--	--
	--	--	--	--	76085850			
282	cnvi0117445							
19	--	--	--	--	--	--	--	--
	--	--	--	--	--	--	--	--
	--	--	--	--	--	--	--	--

	--	--	--	--	--	--	--	--
	--	--	--	--	8959862			
283	cnvi0117446							
5	--	--	--	--	--	--	--	--
	--	--	--	--	--	--	--	--
	--	--	--	--	--	--	--	--
	--	--	--	--	--	--	--	--
	--	--	--	--	18359949			
284	cnvi0117447							
9	--	--	--	--	--	--	--	--
	--	--	--	--	--	--	--	--
	--	--	--	--	--	--	--	--
	--	--	--	--	--	--	--	--
	--	--	--	--	41858227			
285	cnvi0117449							
14	--	--	--	--	--	--	--	--
	--	--	--	--	--	--	--	--
	--	--	--	--	--	--	--	--
	--	--	--	--	--	--	--	--
	--	--	--	--	57257425			
286	cnvi0117451							
9	--	--	--	--	--	--	--	--
	--	--	--	--	--	--	--	--
	--	--	--	--	--	--	--	--
	--	--	--	--	--	--	--	--
	--	--	--	--	40720223			
287	cnvi0117452							
9	--	--	--	--	--	--	--	--
	--	--	--	--	--	--	--	--
	--	--	--	--	--	--	--	--
	--	--	--	--	--	--	--	--
	--	--	--	--	40707882			
288	cnvi0117453							
2	--	--	--	--	--	--	--	--
	--	--	--	--	--	--	--	--
	--	--	--	--	--	--	--	--
	--	--	--	--	--	--	--	--
	--	--	--	--	47690949			
289	cnvi0117454							
6	--	--	--	--	--	--	--	--
	--	--	--	--	--	--	--	--
	--	--	--	--	--	--	--	--
	--	--	--	--	--	--	--	--
	--	--	--	--	47535469			
290	cnvi0117455							
9	--	--	--	--	--	--	--	--
	--	--	--	--	--	--	--	--
	--	--	--	--	--	--	--	--
	--	--	--	--	--	--	--	--
	--	--	--	--	42230470			

291 cnvi0117456
9 -- -- -- -- -- -- -- --
-- -- -- -- -- -- -- --
-- -- -- -- -- -- -- --
-- -- -- -- -- -- -- --
-- -- -- -- 133969522
292 cnvi0117457
11 -- -- -- -- -- -- -- --
-- -- -- -- -- -- -- --
-- -- -- -- -- -- -- --
-- -- -- -- -- -- -- --
-- -- -- -- 121194790
293 cnvi0117458
1 -- -- -- -- -- -- -- --
-- -- -- -- -- -- -- --
-- -- -- -- -- -- -- --
-- -- -- -- -- -- -- --
-- -- -- -- 47522507
294 cnvi0117459
21 -- -- -- -- -- -- -- --
-- -- -- -- -- -- -- --
-- -- -- -- -- -- -- --
-- -- -- -- -- -- -- --
-- -- -- -- 45529986
295 cnvi0117460
2 -- -- -- -- -- -- -- --
-- -- -- -- -- -- -- --
-- -- -- -- -- -- -- --
-- -- -- -- -- -- -- --
-- -- -- -- 169791754
296 cnvi0117462
9 -- -- -- -- -- -- -- --
-- -- -- -- -- -- -- --
-- -- -- -- -- -- -- --
-- -- -- -- -- -- -- --
-- -- -- -- 40676025
297 cnvi0117463
12 -- -- -- -- -- -- -- --
-- -- -- -- -- -- -- --
-- -- -- -- -- -- -- --
-- -- -- -- -- -- -- --
-- -- -- -- 5069369
298 cnvi0117466
17 -- -- -- -- -- -- -- --
-- -- -- -- -- -- -- --
-- -- -- -- -- -- -- --
-- -- -- -- -- -- -- --
-- -- -- -- 59467257
299 cnvi0117467
10 -- -- -- -- -- -- -- --

	--	--	--	--	--	--	--	--
	--	--	--	--	--	--	--	--
	--	--	--	--	--	--	--	--
	--	--	--	--	81988324			
300	cnvi0117472							
9	--	--	--	--	--	--	--	--
	--	--	--	--	--	--	--	--
	--	--	--	--	--	--	--	--
	--	--	--	--	--	--	--	--
	--	--	--	--	40615468			
301	cnvi0117473							
9	--	--	--	--	--	--	--	--
	--	--	--	--	--	--	--	--
	--	--	--	--	--	--	--	--
	--	--	--	--	--	--	--	--
	--	--	--	--	41899555			
302	cnvi0117474							
5	--	--	--	--	--	--	--	--
	--	--	--	--	--	--	--	--
	--	--	--	--	--	--	--	--
	--	--	--	--	--	--	--	--
	--	--	--	--	167469340			
303	cnvi0117475							
2	--	--	--	--	--	--	--	--
	--	--	--	--	--	--	--	--
	--	--	--	--	--	--	--	--
	--	--	--	--	--	--	--	--
	--	--	--	--	55971522			
304	cnvi0117476							
16	--	--	--	--	--	--	--	--
	--	--	--	--	--	--	--	--
	--	--	--	--	--	--	--	--
	--	--	--	--	--	--	--	--
	--	--	--	--	54955111			
305	cnvi0117477							
9	--	--	--	--	--	--	--	--
	--	--	--	--	--	--	--	--
	--	--	--	--	--	--	--	--
	--	--	--	--	--	--	--	--
	--	--	--	--	41500625			
306	cnvi0117481							
11	--	--	--	--	--	--	--	--
	--	--	--	--	--	--	--	--
	--	--	--	--	--	--	--	--
	--	--	--	--	--	--	--	--
	--	--	--	--	34601568			
307	cnvi0117482							
6	--	--	--	--	--	--	--	--
	--	--	--	--	--	--	--	--
	--	--	--	--	--	--	--	--

```
        --      --      --      --      --      --      --      --
        --      --      --      --      135161187
308     cnvi0117483
9       --      --      --      --      --      --      --      --
        --      --      --      --      --      --      --      --
        --      --      --      --      --      --      --      --
        --      --      --      --      --      --      --      --
        --      --      --      --      40713622
309     cnvi0117484
2       --      --      --      --      --      --      --      --
        --      --      --      --      --      --      --      --
        --      --      --      --      --      --      --      --
        --      --      --      --      --      --      --      --
        --      --      --      --      197391755
310     cnvi0117485
9       --      --      --      --      --      --      --      --
        --      --      --      --      --      --      --      --
        --      --      --      --      --      --      --      --
        --      --      --      --      --      --      --      --
        --      --      --      --      40644455
311     cnvi0117486
5       --      --      --      --      --      --      --      --
        --      --      --      --      --      --      --      --
        --      --      --      --      --      --      --      --
        --      --      --      --      --      --      --      --
        --      --      --      --      70342609
312     cnvi0117487
8       --      --      --      --      --      --      --      --
        --      --      --      --      --      --      --      --
        --      --      --      --      --      --      --      --
        --      --      --      --      --      --      --      --
        --      --      --      --      70437446
313     cnvi0117489
6       --      --      --      --      --      --      --      --
        --      --      --      --      --      --      --      --
        --      --      --      --      --      --      --      --
        --      --      --      --      --      --      --      --
        --      --      --      --      2354916
314     cnvi0117491
9       --      --      --      --      --      --      --      --
        --      --      --      --      --      --      --      --
        --      --      --      --      --      --      --      --
        --      --      --      --      --      --      --      --
        --      --      --      --      130260179
315     cnvi0117492
15      --      --      --      --      --      --      --      --
        --      --      --      --      --      --      --      --
        --      --      --      --      --      --      --      --
        --      --      --      --      --      --      --      --
        --      --      --      --      42678815
```

316 cnvi0117493
21 -- -- -- -- -- -- -- --
-- -- -- -- -- -- -- --
-- -- -- -- -- -- -- --
-- -- -- -- -- -- -- --
-- -- -- -- 31463667
317 cnvi0117495
18 -- -- -- -- -- -- -- --
-- -- -- -- -- -- -- --
-- -- -- -- -- -- -- --
-- -- -- -- -- -- -- --
-- -- -- -- 3649321
318 cnvi0117496
16 -- -- -- -- -- -- -- --
-- -- -- -- -- -- -- --
-- -- -- -- -- -- -- --
-- -- -- -- -- -- -- --
-- -- -- -- 16768780
319 cnvi0117497
7 -- -- -- -- -- -- -- --
-- -- -- -- -- -- -- --
-- -- -- -- -- -- -- --
-- -- -- -- -- -- -- --
-- -- -- -- 156790875
320 cnvi0117498
9 -- -- -- -- -- -- -- --
-- -- -- -- -- -- -- --
-- -- -- -- -- -- -- --
-- -- -- -- -- -- -- --
-- -- -- -- 40861427
321 cnvi0117499
8 -- -- -- -- -- -- -- --
-- -- -- -- -- -- -- --
-- -- -- -- -- -- -- --
-- -- -- -- -- -- -- --
-- -- -- -- 47981996
322 cnvi0117501
6 -- -- -- -- -- -- -- --
-- -- -- -- -- -- -- --
-- -- -- -- -- -- -- --
-- -- -- -- -- -- -- --
-- -- -- -- 108477262
323 cnvi0117502
17 -- -- -- -- -- -- -- --
-- -- -- -- -- -- -- --
-- -- -- -- -- -- -- --
-- -- -- -- -- -- -- --
-- -- -- -- 21967971
324 cnvi0117503
16 -- -- -- -- -- -- -- --

	--	--	--	--	--	--	--	--
	--	--	--	--	--	--	--	--
	--	--	--	--	--	--	--	--
	--	--	--	--	4240494			
325	cnvi0117504							
18	--	--	--	--	--	--	--	--
	--	--	--	--	--	--	--	--
	--	--	--	--	--	--	--	--
	--	--	--	--	--	--	--	--
	--	--	--	--	25751233			
326	cnvi0117506							
15	--	--	--	--	--	--	--	--
	--	--	--	--	--	--	--	--
	--	--	--	--	--	--	--	--
	--	--	--	--	--	--	--	--
	--	--	--	--	32596081			
327	cnvi0117507							
10	--	--	--	--	--	--	--	--
	--	--	--	--	--	--	--	--
	--	--	--	--	--	--	--	--
	--	--	--	--	--	--	--	--
	--	--	--	--	12248966			
328	cnvi0117508							
1	--	--	--	--	--	--	--	--
	--	--	--	--	--	--	--	--
	--	--	--	--	--	--	--	--
	--	--	--	--	--	--	--	--
	--	--	--	--	200371577			
329	cnvi0117509							
9	--	--	--	--	--	--	--	--
	--	--	--	--	--	--	--	--
	--	--	--	--	--	--	--	--
	--	--	--	--	--	--	--	--
	--	--	--	--	41864254			
330	cnvi0117510							
14	--	--	--	--	--	--	--	--
	--	--	--	--	--	--	--	--
	--	--	--	--	--	--	--	--
	--	--	--	--	--	--	--	--
	--	--	--	--	63255517			
331	cnvi0117511							
18	--	--	--	--	--	--	--	--
	--	--	--	--	--	--	--	--
	--	--	--	--	--	--	--	--
	--	--	--	--	--	--	--	--
	--	--	--	--	72383875			
332	cnvi0117512							
12	--	--	--	--	--	--	--	--
	--	--	--	--	--	--	--	--
	--	--	--	--	--	--	--	--

	--	--	--	--	--	--	--	--
	--	--	--	--	108975871			
333	cnvi0117516							
6	--	--	--	--	--	--	--	--
	--	--	--	--	--	--	--	--
	--	--	--	--	--	--	--	--
	--	--	--	--	--	--	--	--
	--	--	--	--	21588676			
334	cnvi0117517							
15	--	--	--	--	--	--	--	--
	--	--	--	--	--	--	--	--
	--	--	--	--	--	--	--	--
	--	--	--	--	--	--	--	--
	--	--	--	--	93584526			
335	cnvi0117518							
9	--	--	--	--	--	--	--	--
	--	--	--	--	--	--	--	--
	--	--	--	--	--	--	--	--
	--	--	--	--	--	--	--	--
	--	--	--	--	41678852			
336	cnvi0117520							
16	--	--	--	--	--	--	--	--
	--	--	--	--	--	--	--	--
	--	--	--	--	--	--	--	--
	--	--	--	--	--	--	--	--
	--	--	--	--	30087082			
337	cnvi0117523							
6	--	--	--	--	--	--	--	--
	--	--	--	--	--	--	--	--
	--	--	--	--	--	--	--	--
	--	--	--	--	--	--	--	--
	--	--	--	--	99272580			
338	cnvi0117524							
16	--	--	--	--	--	--	--	--
	--	--	--	--	--	--	--	--
	--	--	--	--	--	--	--	--
	--	--	--	--	--	--	--	--
	--	--	--	--	61679107			
339	cnvi0117525							
15	--	--	--	--	--	--	--	--
	--	--	--	--	--	--	--	--
	--	--	--	--	--	--	--	--
	--	--	--	--	--	--	--	--
	--	--	--	--	48257150			
340	cnvi0117526							
7	--	--	--	--	--	--	--	--
	--	--	--	--	--	--	--	--
	--	--	--	--	--	--	--	--
	--	--	--	--	--	--	--	--
	--	--	--	--	35261112			

341 cnvi0117527
9 -- -- -- -- -- -- -- --
-- -- -- -- -- -- -- --
-- -- -- -- -- -- -- --
-- -- -- -- -- -- -- --
-- -- -- -- 40596813
342 cnvi0117528
9 -- -- -- -- -- -- -- --
-- -- -- -- -- -- -- --
-- -- -- -- -- -- -- --
-- -- -- -- -- -- -- --
-- -- -- -- 41512679
343 cnvi0117529
9 -- -- -- -- -- -- -- --
-- -- -- -- -- -- -- --
-- -- -- -- -- -- -- --
-- -- -- -- -- -- -- --
-- -- -- -- 41851913
344 cnvi0117530
14 -- -- -- -- -- -- -- --
-- -- -- -- -- -- -- --
-- -- -- -- -- -- -- --
-- -- -- -- -- -- -- --
-- -- -- -- 59362180
345 cnvi0117531
9 -- -- -- -- -- -- -- --
-- -- -- -- -- -- -- --
-- -- -- -- -- -- -- --
-- -- -- -- -- -- -- --
-- -- -- -- 40603127
346 cnvi0117532
9 -- -- -- -- -- -- -- --
-- -- -- -- -- -- -- --
-- -- -- -- -- -- -- --
-- -- -- -- -- -- -- --
-- -- -- -- 40761838
347 cnvi0117533
15 -- -- -- -- -- -- -- --
-- -- -- -- -- -- -- --
-- -- -- -- -- -- -- --
-- -- -- -- -- -- -- --
-- -- -- -- 44913242
348 cnvi0117535
2 -- -- -- -- -- -- -- --
-- -- -- -- -- -- -- --
-- -- -- -- -- -- -- --
-- -- -- -- -- -- -- --
-- -- -- -- 169793485
349 cnvi0117536
5 -- -- -- -- -- -- -- --

	--	--	--	--	--	--	--	--
	--	--	--	--	--	--	--	--
	--	--	--	--	--	--	--	--
	--	--	--	--	13776794			
350	cnvi0117537							
20	--	--	--	--	--	--	--	--
	--	--	--	--	--	--	--	--
	--	--	--	--	--	--	--	--
	--	--	--	--	--	--	--	--
	--	--	--	--	21493732			
351	cnvi0117539							
9	--	--	--	--	--	--	--	--
	--	--	--	--	--	--	--	--
	--	--	--	--	--	--	--	--
	--	--	--	--	--	--	--	--
	--	--	--	--	40691236			
352	cnvi0117540							
3	--	--	--	--	--	--	--	--
	--	--	--	--	--	--	--	--
	--	--	--	--	--	--	--	--
	--	--	--	--	--	--	--	--
	--	--	--	--	71717310			
353	cnvi0117541							
10	--	--	--	--	--	--	--	--
	--	--	--	--	--	--	--	--
	--	--	--	--	--	--	--	--
	--	--	--	--	--	--	--	--
	--	--	--	--	94440138			
354	cnvi0117542							
9	--	--	--	--	--	--	--	--
	--	--	--	--	--	--	--	--
	--	--	--	--	--	--	--	--
	--	--	--	--	--	--	--	--
	--	--	--	--	41516984			
355	cnvi0117546							
9	--	--	--	--	--	--	--	--
	--	--	--	--	--	--	--	--
	--	--	--	--	--	--	--	--
	--	--	--	--	--	--	--	--
	--	--	--	--	42260318			
356	cnvi0117547							
1	--	--	--	--	--	--	--	--
	--	--	--	--	--	--	--	--
	--	--	--	--	--	--	--	--
	--	--	--	--	--	--	--	--
	--	--	--	--	38500388			
357	cnvi0117548							
7	--	--	--	--	--	--	--	--
	--	--	--	--	--	--	--	--
	--	--	--	--	--	--	--	--

	--	--	--	--	--	--	--	--
	--	--	--	--	55916266			
358	cnvi0117549							
2	--	--	--	--	--	--	--	--
	--	--	--	--	--	--	--	--
	--	--	--	--	--	--	--	--
	--	--	--	--	--	--	--	--
	--	--	--	--	87498995			
359	cnvi0117550							
17	--	--	--	--	--	--	--	--
	--	--	--	--	--	--	--	--
	--	--	--	--	--	--	--	--
	--	--	--	--	--	--	--	--
	--	--	--	--	67091177			
360	cnvi0117551							
9	--	--	--	--	--	--	--	--
	--	--	--	--	--	--	--	--
	--	--	--	--	--	--	--	--
	--	--	--	--	--	--	--	--
	--	--	--	--	40891562			
361	cnvi0117552							
9	--	--	--	--	--	--	--	--
	--	--	--	--	--	--	--	--
	--	--	--	--	--	--	--	--
	--	--	--	--	--	--	--	--
	--	--	--	--	42190286			
362	cnvi0117553							
9	--	--	--	--	--	--	--	--
	--	--	--	--	--	--	--	--
	--	--	--	--	--	--	--	--
	--	--	--	--	--	--	--	--
	--	--	--	--	41875447			
363	cnvi0117556							
17	--	--	--	--	--	--	--	--
	--	--	--	--	--	--	--	--
	--	--	--	--	--	--	--	--
	--	--	--	--	--	--	--	--
	--	--	--	--	36354182			
364	cnvi0117557							
20	--	--	--	--	--	--	--	--
	--	--	--	--	--	--	--	--
	--	--	--	--	--	--	--	--
	--	--	--	--	--	--	--	--
	--	--	--	--	49768007			
365	cnvi0117559							
21	--	--	--	--	--	--	--	--
	--	--	--	--	--	--	--	--
	--	--	--	--	--	--	--	--
	--	--	--	--	--	--	--	--
	--	--	--	--	38116010			

366	cnvi0117560							
2	--	--	--	--	--	--	--	--
	--	--	--	--	--	--	--	--
	--	--	--	--	--	--	--	--
	--	--	--	--	--	--	--	--
	--	--	--	--	200595923			
367	cnvi0117562							
12	--	--	--	--	--	--	--	--
	--	--	--	--	--	--	--	--
	--	--	--	--	--	--	--	--
	--	--	--	--	--	--	--	--
	--	--	--	--	113991013			
368	cnvi0117565							
7	--	--	--	--	--	--	--	--
	--	--	--	--	--	--	--	--
	--	--	--	--	--	--	--	--
	--	--	--	--	--	--	--	--
	--	--	--	--	46636215			
369	cnvi0117567							
9	--	--	--	--	--	--	--	--
	--	--	--	--	--	--	--	--
	--	--	--	--	--	--	--	--
	--	--	--	--	--	--	--	--
	--	--	--	--	126760179			
370	cnvi0117569							
7	--	--	--	--	--	--	--	--
	--	--	--	--	--	--	--	--
	--	--	--	--	--	--	--	--
	--	--	--	--	--	--	--	--
	--	--	--	--	27122612			
371	cnvi0117570							
9	--	--	--	--	--	--	--	--
	--	--	--	--	--	--	--	--
	--	--	--	--	--	--	--	--
	--	--	--	--	--	--	--	--
	--	--	--	--	42177084			
372	cnvi0117571							
11	--	--	--	--	--	--	--	--
	--	--	--	--	--	--	--	--
	--	--	--	--	--	--	--	--
	--	--	--	--	--	--	--	--
	--	--	--	--	112823752			
373	cnvi0117575							
20	--	--	--	--	--	--	--	--
	--	--	--	--	--	--	--	--
	--	--	--	--	--	--	--	--
	--	--	--	--	--	--	--	--
	--	--	--	--	47150301			
374	cnvi0117576							
5	--	--	--	--	--	--	--	--

-- -- -- -- -- -- -- --
-- -- -- -- -- -- -- --
-- -- -- -- -- -- -- --
-- -- -- -- 167467422
375 cnvi0117577
10 -- -- -- -- -- -- -- --
-- -- -- -- -- -- -- --
-- -- -- -- -- -- -- --
-- -- -- -- -- -- -- --
-- -- -- -- 123180394
376 cnvi0117580
14 -- -- -- -- -- -- -- --
-- -- -- -- -- -- -- --
-- -- -- -- -- -- -- --
-- -- -- -- -- -- -- --
-- -- -- -- 23989280
377 cnvi0117581
9 -- -- -- -- -- -- -- --
-- -- -- -- -- -- -- --
-- -- -- -- -- -- -- --
-- -- -- -- -- -- -- --
-- -- -- -- 40755524
378 cnvi0117584
9 -- -- -- -- -- -- -- --
-- -- -- -- -- -- -- --
-- -- -- -- -- -- -- --
-- -- -- -- -- -- -- --
-- -- -- -- 40867167
379 cnvi0117585
11 -- -- -- -- -- -- -- --
-- -- -- -- -- -- -- --
-- -- -- -- -- -- -- --
-- -- -- -- -- -- -- --
-- -- -- -- 17241969
380 cnvi0117586
2 -- -- -- -- -- -- -- --
-- -- -- -- -- -- -- --
-- -- -- -- -- -- -- --
-- -- -- -- -- -- -- --
-- -- -- -- 8826225
381 cnvi0117587
5 -- -- -- -- -- -- -- --
-- -- -- -- -- -- -- --
-- -- -- -- -- -- -- --
-- -- -- -- -- -- -- --
-- -- -- -- 4578036
382 cnvi0117590
16 -- -- -- -- -- -- -- --
-- -- -- -- -- -- -- --
-- -- -- -- -- -- -- --

-- -- -- -- -- -- -- --
-- -- -- -- 46944047
383 cnvi0117591
9 -- -- -- -- -- -- -- --
-- -- -- -- -- -- -- --
-- -- -- -- -- -- -- --
-- -- -- -- -- -- -- --
-- -- -- -- 42197461
384 cnvi0117592
14 -- -- -- -- -- -- -- --
-- -- -- -- -- -- -- --
-- -- -- -- -- -- -- --
-- -- -- -- -- -- -- --
-- -- -- -- 53907370
385 cnvi0117593
13 -- -- -- -- -- -- -- --
-- -- -- -- -- -- -- --
-- -- -- -- -- -- -- --
-- -- -- -- -- -- -- --
-- -- -- -- 77564475
386 cnvi0117594
15 -- -- -- -- -- -- -- --
-- -- -- -- -- -- -- --
-- -- -- -- -- -- -- --
-- -- -- -- -- -- -- --
-- -- -- -- 34240333
387 cnvi0117596
2 -- -- -- -- -- -- -- --
-- -- -- -- -- -- -- --
-- -- -- -- -- -- -- --
-- -- -- -- -- -- -- --
-- -- -- -- 17036685
388 cnvi0117597
9 -- -- -- -- -- -- -- --
-- -- -- -- -- -- -- --
-- -- -- -- -- -- -- --
-- -- -- -- -- -- -- --
-- -- -- -- 40639289
389 cnvi0117598
7 -- -- -- -- -- -- -- --
-- -- -- -- -- -- -- --
-- -- -- -- -- -- -- --
-- -- -- -- -- -- -- --
-- -- -- -- 69054808
390 cnvi0117599
20 -- -- -- -- -- -- -- --
-- -- -- -- -- -- -- --
-- -- -- -- -- -- -- --
-- -- -- -- -- -- -- --
-- -- -- -- 58705161

391	cnvi0117600							
5	--	--	--	--	--	--	--	--
	--	--	--	--	--	--	--	--
	--	--	--	--	--	--	--	--
	--	--	--	--	--	--	--	--
	--	--	--	--	88006166			
392	cnvi0117602							
9	--	--	--	--	--	--	--	--
	--	--	--	--	--	--	--	--
	--	--	--	--	--	--	--	--
	--	--	--	--	--	--	--	--
	--	--	--	--	41472786			
393	cnvi0117603							
7	--	--	--	--	--	--	--	--
	--	--	--	--	--	--	--	--
	--	--	--	--	--	--	--	--
	--	--	--	--	--	--	--	--
	--	--	--	--	50232189			
394	cnvi0117605							
18	--	--	--	--	--	--	--	--
	--	--	--	--	--	--	--	--
	--	--	--	--	--	--	--	--
	--	--	--	--	--	--	--	--
	--	--	--	--	52889831			
395	cnvi0117606							
10	--	--	--	--	--	--	--	--
	--	--	--	--	--	--	--	--
	--	--	--	--	--	--	--	--
	--	--	--	--	--	--	--	--
	--	--	--	--	47073657			
396	cnvi0117607							
11	--	--	--	--	--	--	--	--
	--	--	--	--	--	--	--	--
	--	--	--	--	--	--	--	--
	--	--	--	--	--	--	--	--
	--	--	--	--	89161102			
397	cnvi0117608							
2	--	--	--	--	--	--	--	--
	--	--	--	--	--	--	--	--
	--	--	--	--	--	--	--	--
	--	--	--	--	--	--	--	--
	--	--	--	--	96336731			
398	cnvi0117609							
21	--	--	--	--	--	--	--	--
	--	--	--	--	--	--	--	--
	--	--	--	--	--	--	--	--
	--	--	--	--	--	--	--	--
	--	--	--	--	36931256			
399	cnvi0117610							
11	--	--	--	--	--	--	--	--

	--	--	--	--	--	--	--	--
	--	--	--	--	--	--	--	--
	--	--	--	--	--	--	--	--
	--	--	--	--	69614736			
400	cnvi0117611							
8	--	--	--	--	--	--	--	--
	--	--	--	--	--	--	--	--
	--	--	--	--	--	--	--	--
	--	--	--	--	--	--	--	--
	--	--	--	--	91097556			
401	cnvi0117612							
6	--	--	--	--	--	--	--	--
	--	--	--	--	--	--	--	--
	--	--	--	--	--	--	--	--
	--	--	--	--	--	--	--	--
	--	--	--	--	21591140			
402	cnvi0117613							
12	--	--	--	--	--	--	--	--
	--	--	--	--	--	--	--	--
	--	--	--	--	--	--	--	--
	--	--	--	--	--	--	--	--
	--	--	--	--	4560662			
403	cnvi0117614							
6	--	--	--	--	--	--	--	--
	--	--	--	--	--	--	--	--
	--	--	--	--	--	--	--	--
	--	--	--	--	--	--	--	--
	--	--	--	--	56936029			
404	cnvi0117616							
1	--	--	--	--	--	--	--	--
	--	--	--	--	--	--	--	--
	--	--	--	--	--	--	--	--
	--	--	--	--	--	--	--	--
	--	--	--	--	3364960			
405	cnvi0117617							
9	--	--	--	--	--	--	--	--
	--	--	--	--	--	--	--	--
	--	--	--	--	--	--	--	--
	--	--	--	--	--	--	--	--
	--	--	--	--	40651056			
406	cnvi0117618							
4	--	--	--	--	--	--	--	--
	--	--	--	--	--	--	--	--
	--	--	--	--	--	--	--	--
	--	--	--	--	--	--	--	--
	--	--	--	--	46280159			
407	cnvi0117619							
11	--	--	--	--	--	--	--	--
	--	--	--	--	--	--	--	--
	--	--	--	--	--	--	--	--

	--	--	--	--	--	--	--	--
	--	--	--	--	21512725			
408	cnvi0117620							
2	--	--	--	--	--	--	--	--
	--	--	--	--	--	--	--	--
	--	--	--	--	--	--	--	--
	--	--	--	--	--	--	--	--
	--	--	--	--	160166002			
409	cnvi0117622							
20	--	--	--	--	--	--	--	--
	--	--	--	--	--	--	--	--
	--	--	--	--	--	--	--	--
	--	--	--	--	--	--	--	--
	--	--	--	--	11915070			
410	cnvi0117623							
4	--	--	--	--	--	--	--	--
	--	--	--	--	--	--	--	--
	--	--	--	--	--	--	--	--
	--	--	--	--	--	--	--	--
	--	--	--	--	53727242			
411	cnvi0117624							
3	--	--	--	--	--	--	--	--
	--	--	--	--	--	--	--	--
	--	--	--	--	--	--	--	--
	--	--	--	--	--	--	--	--
	--	--	--	--	95295734			
412	cnvi0117625							
9	--	--	--	--	--	--	--	--
	--	--	--	--	--	--	--	--
	--	--	--	--	--	--	--	--
	--	--	--	--	--	--	--	--
	--	--	--	--	40669711			
413	cnvi0117626							
7	--	--	--	--	--	--	--	--
	--	--	--	--	--	--	--	--
	--	--	--	--	--	--	--	--
	--	--	--	--	--	--	--	--
	--	--	--	--	72169918			
414	cnvi0117627							
15	--	--	--	--	--	--	--	--
	--	--	--	--	--	--	--	--
	--	--	--	--	--	--	--	--
	--	--	--	--	--	--	--	--
	--	--	--	--	30092633			
415	cnvi0117628							
9	--	--	--	--	--	--	--	--
	--	--	--	--	--	--	--	--
	--	--	--	--	--	--	--	--
	--	--	--	--	--	--	--	--
	--	--	--	--	42242811			

416 cnvi0117629
13 -- -- -- -- -- -- -- --
-- -- -- -- -- -- -- --
-- -- -- -- -- -- -- --
-- -- -- -- -- -- -- --
-- -- -- -- 47537838
417 cnvi0117630
9 -- -- -- -- -- -- -- --
-- -- -- -- -- -- -- --
-- -- -- -- -- -- -- --
-- -- -- -- -- -- -- --
-- -- -- -- 40916244
418 cnvi0117632
7 -- -- -- -- -- -- -- --
-- -- -- -- -- -- -- --
-- -- -- -- -- -- -- --
-- -- -- -- -- -- -- --
-- -- -- -- 11215070
419 cnvi0117633
6 -- -- -- -- -- -- -- --
-- -- -- -- -- -- -- --
-- -- -- -- -- -- -- --
-- -- -- -- -- -- -- --
-- -- -- -- 41852269
420 cnvi0117634
3 -- -- -- -- -- -- -- --
-- -- -- -- -- -- -- --
-- -- -- -- -- -- -- --
-- -- -- -- -- -- -- --
-- -- -- -- 187917306
421 cnvi0117635
22 -- -- -- -- -- -- -- --
-- -- -- -- -- -- -- --
-- -- -- -- -- -- -- --
-- -- -- -- -- -- -- --
-- -- -- -- 25711667
422 cnvi0117637
15 -- -- -- -- -- -- -- --
-- -- -- -- -- -- -- --
-- -- -- -- -- -- -- --
-- -- -- -- -- -- -- --
-- -- -- -- 25652956
423 cnvi0117638
9 -- -- -- -- -- -- -- --
-- -- -- -- -- -- -- --
-- -- -- -- -- -- -- --
-- -- -- -- -- -- -- --
-- -- -- -- 40873481
424 cnvi0117639
9 -- -- -- -- -- -- -- --

	--	--	--	--	--	--	--	--
	--	--	--	--	--	--	--	--
	--	--	--	--	--	--	--	--
	--	--	--	--	40622069			
425	cnvi0117640							
2	--	--	--	--	--	--	--	--
	--	--	--	--	--	--	--	--
	--	--	--	--	--	--	--	--
	--	--	--	--	--	--	--	--
	--	--	--	--	109233568			
426	cnvi0117641							
14	--	--	--	--	--	--	--	--
	--	--	--	--	--	--	--	--
	--	--	--	--	--	--	--	--
	--	--	--	--	--	--	--	--
	--	--	--	--	39598771			
427	cnvi0117642							
14	--	--	--	--	--	--	--	--
	--	--	--	--	--	--	--	--
	--	--	--	--	--	--	--	--
	--	--	--	--	--	--	--	--
	--	--	--	--	94203027			
428	cnvi0117644							
19	--	--	--	--	--	--	--	--
	--	--	--	--	--	--	--	--
	--	--	--	--	--	--	--	--
	--	--	--	--	--	--	--	--
	--	--	--	--	4998696			
429	cnvi0117645							
1	--	--	--	--	--	--	--	--
	--	--	--	--	--	--	--	--
	--	--	--	--	--	--	--	--
	--	--	--	--	--	--	--	--
	--	--	--	--	20135863			
430	cnvi0117647							
6	--	--	--	--	--	--	--	--
	--	--	--	--	--	--	--	--
	--	--	--	--	--	--	--	--
	--	--	--	--	--	--	--	--
	--	--	--	--	147514872			
431	cnvi0117648							
18	--	--	--	--	--	--	--	--
	--	--	--	--	--	--	--	--
	--	--	--	--	--	--	--	--
	--	--	--	--	--	--	--	--
	--	--	--	--	45359312			
432	cnvi0117649							
9	--	--	--	--	--	--	--	--
	--	--	--	--	--	--	--	--
	--	--	--	--	--	--	--	--

-- -- -- -- -- -- -- --
-- -- -- -- 40657370
433 cnvi0117652
10 -- -- -- -- -- -- -- --
-- -- -- -- -- -- -- --
-- -- -- -- -- -- -- --
-- -- -- -- -- -- -- --
-- -- -- -- 12221184
434 cnvi0117654
4 -- -- -- -- -- -- -- --
-- -- -- -- -- -- -- --
-- -- -- -- -- -- -- --
-- -- -- -- -- -- -- --
-- -- -- -- 41736224
435 cnvi0117655
9 -- -- -- -- -- -- -- --
-- -- -- -- -- -- -- --
-- -- -- -- -- -- -- --
-- -- -- -- -- -- -- --
-- -- -- -- 41893815
436 cnvi0117656
9 -- -- -- -- -- -- -- --
-- -- -- -- -- -- -- --
-- -- -- -- -- -- -- --
-- -- -- -- -- -- -- --
-- -- -- -- 41731086
437 cnvi0117658
9 -- -- -- -- -- -- -- --
-- -- -- -- -- -- -- --
-- -- -- -- -- -- -- --
-- -- -- -- -- -- -- --
-- -- -- -- 40774753
438 cnvi0117659
3 -- -- -- -- -- -- -- --
-- -- -- -- -- -- -- --
-- -- -- -- -- -- -- --
-- -- -- -- -- -- -- --
-- -- -- -- 142917310
439 cnvi0117660
12 -- -- -- -- -- -- -- --
-- -- -- -- -- -- -- --
-- -- -- -- -- -- -- --
-- -- -- -- -- -- -- --
-- -- -- -- 54384222
440 cnvi0117662
12 -- -- -- -- -- -- -- --
-- -- -- -- -- -- -- --
-- -- -- -- -- -- -- --
-- -- -- -- -- -- -- --
-- -- -- -- 14878202

441 cnvi0117664
15 -- -- -- -- -- -- -- --
-- -- -- -- -- -- -- --
-- -- -- -- -- -- -- --
-- -- -- -- -- -- -- --
-- -- -- -- 71833196
442 cnvi0117665
9 -- -- -- -- -- -- -- --
-- -- -- -- -- -- -- --
-- -- -- -- -- -- -- --
-- -- -- -- -- -- -- --
-- -- -- -- 42222147
443 cnvi0117666
9 -- -- -- -- -- -- -- --
-- -- -- -- -- -- -- --
-- -- -- -- -- -- -- --
-- -- -- -- -- -- -- --
-- -- -- -- 41842155
444 cnvi0117667
8 -- -- -- -- -- -- -- --
-- -- -- -- -- -- -- --
-- -- -- -- -- -- -- --
-- -- -- -- -- -- -- --
-- -- -- -- 9411390
445 cnvi0117668
3 -- -- -- -- -- -- -- --
-- -- -- -- -- -- -- --
-- -- -- -- -- -- -- --
-- -- -- -- -- -- -- --
-- -- -- -- 147117181
446 cnvi0117669
10 -- -- -- -- -- -- -- --
-- -- -- -- -- -- -- --
-- -- -- -- -- -- -- --
-- -- -- -- -- -- -- --
-- -- -- -- 77734740
447 cnvi0117670
9 -- -- -- -- -- -- -- --
-- -- -- -- -- -- -- --
-- -- -- -- -- -- -- --
-- -- -- -- -- -- -- --
-- -- -- -- 42183972
448 cnvi0117671
19 -- -- -- -- -- -- -- --
-- -- -- -- -- -- -- --
-- -- -- -- -- -- -- --
-- -- -- -- -- -- -- --
-- -- -- -- 14950376
449 cnvi0117672
20 -- -- -- -- -- -- -- --

	--	--	--	--	--	--	--	--
	--	--	--	--	--	--	--	--
	--	--	--	--	--	--	--	--
	--	--	--	--	11941446			
450	cnvi0117674							
9	--	--	--	--	--	--	--	--
	--	--	--	--	--	--	--	--
	--	--	--	--	--	--	--	--
	--	--	--	--	--	--	--	--
	--	--	--	--	42272946			
451	cnvi0117675							
15	--	--	--	--	--	--	--	--
	--	--	--	--	--	--	--	--
	--	--	--	--	--	--	--	--
	--	--	--	--	--	--	--	--
	--	--	--	--	72613646			
452	cnvi0117677							
9	--	--	--	--	--	--	--	--
	--	--	--	--	--	--	--	--
	--	--	--	--	--	--	--	--
	--	--	--	--	--	--	--	--
	--	--	--	--	41725059			
453	cnvi0117678							
22	--	--	--	--	--	--	--	--
	--	--	--	--	--	--	--	--
	--	--	--	--	--	--	--	--
	--	--	--	--	--	--	--	--
	--	--	--	--	21923265			
454	cnvi0117679							
9	--	--	--	--	--	--	--	--
	--	--	--	--	--	--	--	--
	--	--	--	--	--	--	--	--
	--	--	--	--	--	--	--	--
	--	--	--	--	42248551			
455	cnvi0117680							
11	--	--	--	--	--	--	--	--
	--	--	--	--	--	--	--	--
	--	--	--	--	--	--	--	--
	--	--	--	--	--	--	--	--
	--	--	--	--	43472656			
456	cnvi0117682							
9	--	--	--	--	--	--	--	--
	--	--	--	--	--	--	--	--
	--	--	--	--	--	--	--	--
	--	--	--	--	--	--	--	--
	--	--	--	--	41485414			
457	cnvi0117683							
10	--	--	--	--	--	--	--	--
	--	--	--	--	--	--	--	--
	--	--	--	--	--	--	--	--

-- -- -- -- -- -- -- --
-- -- -- -- 87910086
458 cnvi0117684
9 -- -- -- -- -- -- -- --
-- -- -- -- -- -- -- --
-- -- -- -- -- -- -- --
-- -- -- -- -- -- -- --
-- -- -- -- 41735391
459 cnvi0117685
5 -- -- -- -- -- -- -- --
-- -- -- -- -- -- -- --
-- -- -- -- -- -- -- --
-- -- -- -- -- -- -- --
-- -- -- -- 72784400
460 cnvi0117686
9 -- -- -- -- -- -- -- --
-- -- -- -- -- -- -- --
-- -- -- -- -- -- -- --
-- -- -- -- -- -- -- --
-- -- -- -- 40663397
461 cnvi0117687
9 -- -- -- -- -- -- -- --
-- -- -- -- -- -- -- --
-- -- -- -- -- -- -- --
-- -- -- -- -- -- -- --
-- -- -- -- 41494885
462 cnvi0117690
10 -- -- -- -- -- -- -- --
-- -- -- -- -- -- -- --
-- -- -- -- -- -- -- --
-- -- -- -- -- -- -- --
-- -- -- -- 127248300
463 cnvi0117692
7 -- -- -- -- -- -- -- --
-- -- -- -- -- -- -- --
-- -- -- -- -- -- -- --
-- -- -- -- -- -- -- --
-- -- -- -- 114064626
464 cnvi0117693
6 -- -- -- -- -- -- -- --
-- -- -- -- -- -- -- --
-- -- -- -- -- -- -- --
-- -- -- -- -- -- -- --
-- -- -- -- 41510668
465 cnvi0117694
1 -- -- -- -- -- -- -- --
-- -- -- -- -- -- -- --
-- -- -- -- -- -- -- --
-- -- -- -- -- -- -- --
-- -- -- -- 61127412

466 cnvi0117695
9 -- -- -- -- -- -- -- --
-- -- -- -- -- -- -- --
-- -- -- -- -- -- -- --
-- -- -- -- -- -- -- --
-- -- -- -- 40885535
467 cnvi0117696
9 -- -- -- -- -- -- -- --
-- -- -- -- -- -- -- --
-- -- -- -- -- -- -- --
-- -- -- -- -- -- -- --
-- -- -- -- 41847034
468 cnvi0117697
18 -- -- -- -- -- -- -- --
-- -- -- -- -- -- -- --
-- -- -- -- -- -- -- --
-- -- -- -- -- -- -- --
-- -- -- -- 24432756
469 cnvi0117698
9 -- -- -- -- -- -- -- --
-- -- -- -- -- -- -- --
-- -- -- -- -- -- -- --
-- -- -- -- -- -- -- --
-- -- -- -- 40680904
470 cnvi0117699
9 -- -- -- -- -- -- -- --
-- -- -- -- -- -- -- --
-- -- -- -- -- -- -- --
-- -- -- -- -- -- -- --
-- -- -- -- 40686070
471 cnvi0117700
2 -- -- -- -- -- -- -- --
-- -- -- -- -- -- -- --
-- -- -- -- -- -- -- --
-- -- -- -- -- -- -- --
-- -- -- -- 45159603
472 cnvi0117701
4 -- -- -- -- -- -- -- --
-- -- -- -- -- -- -- --
-- -- -- -- -- -- -- --
-- -- -- -- -- -- -- --
-- -- -- -- 3130146
473 cnvi0117702
2 -- -- -- -- -- -- -- --
-- -- -- -- -- -- -- --
-- -- -- -- -- -- -- --
-- -- -- -- -- -- -- --
-- -- -- -- 41746496
474 cnvi0117703
16 -- -- -- -- -- -- -- --

	--	--	--	--	--	--	--	--
	--	--	--	--	--	--	--	--
	--	--	--	--	--	--	--	--
	--	--	--	--	28653319			
475	cnvi0117704							
2	--	--	--	--	--	--	--	--
	--	--	--	--	--	--	--	--
	--	--	--	--	--	--	--	--
	--	--	--	--	--	--	--	--
	--	--	--	--	162262751			
476	cnvi0117705							
1	--	--	--	--	--	--	--	--
	--	--	--	--	--	--	--	--
	--	--	--	--	--	--	--	--
	--	--	--	--	--	--	--	--
	--	--	--	--	23215868			
477	cnvi0117706							
9	--	--	--	--	--	--	--	--
	--	--	--	--	--	--	--	--
	--	--	--	--	--	--	--	--
	--	--	--	--	--	--	--	--
	--	--	--	--	40733425			
478	cnvi0117709							
9	--	--	--	--	--	--	--	--
	--	--	--	--	--	--	--	--
	--	--	--	--	--	--	--	--
	--	--	--	--	--	--	--	--
	--	--	--	--	40583898			
479	cnvi0117711							
3	--	--	--	--	--	--	--	--
	--	--	--	--	--	--	--	--
	--	--	--	--	--	--	--	--
	--	--	--	--	--	--	--	--
	--	--	--	--	137475534			
480	cnvi0117712							
9	--	--	--	--	--	--	--	--
	--	--	--	--	--	--	--	--
	--	--	--	--	--	--	--	--
	--	--	--	--	--	--	--	--
	--	--	--	--	41881474			
481	cnvi0117713							
9	--	--	--	--	--	--	--	--
	--	--	--	--	--	--	--	--
	--	--	--	--	--	--	--	--
	--	--	--	--	--	--	--	--
	--	--	--	--	40633262			
482	cnvi0117714							
9	--	--	--	--	--	--	--	--
	--	--	--	--	--	--	--	--
	--	--	--	--	--	--	--	--

	--	--	--	--	--	--	--	--
	--	--	--	--	41829814			
483	cnvi0117715							
22	--	--	--	--	--	--	--	--
	--	--	--	--	--	--	--	--
	--	--	--	--	--	--	--	--
	--	--	--	--	--	--	--	--
	--	--	--	--	18039490			
484	cnvi0117716							
17	--	--	--	--	--	--	--	--
	--	--	--	--	--	--	--	--
	--	--	--	--	--	--	--	--
	--	--	--	--	--	--	--	--
	--	--	--	--	11252811			
485	cnvi10167462							
7	--	--	--	--	--	--	--	--
	--	--	--	--	--	--	--	--
	--	--	--	--	--	--	--	--
	--	--	--	--	--	--	--	--
	--	--	--	--	96650614			
486	cnvi10167519							
7	--	--	--	--	--	--	--	--
	--	--	--	--	--	--	--	--
	--	--	--	--	--	--	--	--
	--	--	--	--	--	--	--	--
	--	--	--	--	96653464			
487	cnvi11363497							
7	--	--	--	--	--	--	--	--
	--	--	--	--	--	--	--	--
	--	--	--	--	--	--	--	--
	--	--	--	--	--	--	--	--
	--	--	--	--	156802689			
488	cnvi11412906							
16	--	--	--	--	--	--	--	--
	--	--	--	--	--	--	--	--
	--	--	--	--	--	--	--	--
	--	--	--	--	--	--	--	--
	--	--	--	--	204500			
489	cnvi11413137							
16	--	--	--	--	--	--	--	--
	--	--	--	--	--	--	--	--
	--	--	--	--	--	--	--	--
	--	--	--	--	--	--	--	--
	--	--	--	--	216050			
490	cnvi11413139							
16	--	--	--	--	--	--	--	--
	--	--	--	--	--	--	--	--
	--	--	--	--	--	--	--	--
	--	--	--	--	--	--	--	--
	--	--	--	--	216150			

491 cnvi11413141
16 -- -- -- -- -- -- -- --
-- -- -- -- -- -- -- --
-- -- -- -- -- -- -- --
-- -- -- -- -- -- -- --
-- -- -- -- 216250
492 cnvi11413144
16 -- -- -- -- -- -- -- --
-- -- -- -- -- -- -- --
-- -- -- -- -- -- -- --
-- -- -- -- -- -- -- --
-- -- -- -- 216400
493 cnvi11413149
16 -- -- -- -- -- -- -- --
-- -- -- -- -- -- -- --
-- -- -- -- -- -- -- --
-- -- -- -- -- -- -- --
-- -- -- -- 216650
494 cnvi11413361
16 -- -- -- -- -- -- -- --
-- -- -- -- -- -- -- --
-- -- -- -- -- -- -- --
-- -- -- -- -- -- -- --
-- -- -- -- 227250
495 cnvi11413426
16 -- -- -- -- -- -- -- --
-- -- -- -- -- -- -- --
-- -- -- -- -- -- -- --
-- -- -- -- -- -- -- --
-- -- -- -- 230500
496 cnvi11413430
16 -- -- -- -- -- -- -- --
-- -- -- -- -- -- -- --
-- -- -- -- -- -- -- --
-- -- -- -- -- -- -- --
-- -- -- -- 230700
497 cnvi11413433
16 -- -- -- -- -- -- -- --
-- -- -- -- -- -- -- --
-- -- -- -- -- -- -- --
-- -- -- -- -- -- -- --
-- -- -- -- 230850
498 cnvi11413438
16 -- -- -- -- -- -- -- --
-- -- -- -- -- -- -- --
-- -- -- -- -- -- -- --
-- -- -- -- -- -- -- --
-- -- -- -- 231100
499 cnvi12269164
16 -- -- -- -- -- -- -- --

-- -- -- -- -- -- -- --
-- -- -- -- -- -- -- --
-- -- -- -- -- -- -- --
-- -- -- -- 54317599
500 cnvi12282148
16 -- -- -- -- -- -- -- --
-- -- -- -- -- -- -- --
-- -- -- -- -- -- -- --
-- -- -- -- -- -- -- --
-- -- -- -- 54966799
501 cnvi12282160
16 -- -- -- -- -- -- -- --
-- -- -- -- -- -- -- --
-- -- -- -- -- -- -- --
-- -- -- -- -- -- -- --
-- -- -- -- 54967399
502 cnvi12282166
16 -- -- -- -- -- -- -- --
-- -- -- -- -- -- -- --
-- -- -- -- -- -- -- --
-- -- -- -- -- -- -- --
-- -- -- -- 54967699
503 cnvi12914847
16 -- -- -- -- -- -- -- --
-- -- -- -- -- -- -- --
-- -- -- -- -- -- -- --
-- -- -- -- -- -- -- --
-- -- -- -- 86601749
504 cnvi12914851
16 -- -- -- -- -- -- -- --
-- -- -- -- -- -- -- --
-- -- -- -- -- -- -- --
-- -- -- -- -- -- -- --
-- -- -- -- 86601949
505 cnvi12914858
16 -- -- -- -- -- -- -- --
-- -- -- -- -- -- -- --
-- -- -- -- -- -- -- --
-- -- -- -- -- -- -- --
-- -- -- -- 86602299
506 cnvi13942140
4 -- -- -- -- -- -- -- --
-- -- -- -- -- -- -- --
-- -- -- -- -- -- -- --
-- -- -- -- -- -- -- --
-- -- -- -- 13543202
507 cnvi13942152
4 -- -- -- -- -- -- -- --
-- -- -- -- -- -- -- --
-- -- -- -- -- -- -- --

	--	--	--	--	--	--	--	--
	--	--	--	--	13543802			
508	cnvi13942164							
4	--	--	--	--	--	--	--	--
	--	--	--	--	--	--	--	--
	--	--	--	--	--	--	--	--
	--	--	--	--	--	--	--	--
	--	--	--	--	13544402			
509	cnvi13942170							
4	--	--	--	--	--	--	--	--
	--	--	--	--	--	--	--	--
	--	--	--	--	--	--	--	--
	--	--	--	--	--	--	--	--
	--	--	--	--	13544702			
510	cnvi13942182							
4	--	--	--	--	--	--	--	--
	--	--	--	--	--	--	--	--
	--	--	--	--	--	--	--	--
	--	--	--	--	--	--	--	--
	--	--	--	--	13545302			
511	cnvi1430507							
6	--	--	--	--	--	--	--	--
	--	--	--	--	--	--	--	--
	--	--	--	--	--	--	--	--
	--	--	--	--	--	--	--	--
	--	--	--	--	21594771			
512	cnvi1430523							
6	--	--	--	--	--	--	--	--
	--	--	--	--	--	--	--	--
	--	--	--	--	--	--	--	--
	--	--	--	--	--	--	--	--
	--	--	--	--	21595571			
513	cnvi1430531							
6	--	--	--	--	--	--	--	--
	--	--	--	--	--	--	--	--
	--	--	--	--	--	--	--	--
	--	--	--	--	--	--	--	--
	--	--	--	--	21595971			
514	cnvi1430545							
6	--	--	--	--	--	--	--	--
	--	--	--	--	--	--	--	--
	--	--	--	--	--	--	--	--
	--	--	--	--	--	--	--	--
	--	--	--	--	21596671			
515	cnvi1430559							
6	--	--	--	--	--	--	--	--
	--	--	--	--	--	--	--	--
	--	--	--	--	--	--	--	--
	--	--	--	--	--	--	--	--
	--	--	--	--	21597371			

516 cnvi1430567
6 -- -- -- -- -- -- -- --
-- -- -- -- -- -- -- --
-- -- -- -- -- -- -- --
-- -- -- -- -- -- -- --
-- -- -- -- 21597771
517 cnvi1430581
6 -- -- -- -- -- -- -- --
-- -- -- -- -- -- -- --
-- -- -- -- -- -- -- --
-- -- -- -- -- -- -- --
-- -- -- -- 21598471
518 cnvi15318687
4 -- -- -- -- -- -- -- --
-- -- -- -- -- -- -- --
-- -- -- -- -- -- -- --
-- -- -- -- -- -- -- --
-- -- -- -- 85416076
519 cnvi16561592
4 -- -- -- -- -- -- -- --
-- -- -- -- -- -- -- --
-- -- -- -- -- -- -- --
-- -- -- -- -- -- -- --
-- -- -- -- 147560900
520 cnvi16561598
4 -- -- -- -- -- -- -- --
-- -- -- -- -- -- -- --
-- -- -- -- -- -- -- --
-- -- -- -- -- -- -- --
-- -- -- -- 147561200
521 cnvi16561626
4 -- -- -- -- -- -- -- --
-- -- -- -- -- -- -- --
-- -- -- -- -- -- -- --
-- -- -- -- -- -- -- --
-- -- -- -- 147562600
522 cnvi16640445
4 -- -- -- -- -- -- -- --
-- -- -- -- -- -- -- --
-- -- -- -- -- -- -- --
-- -- -- -- -- -- -- --
-- -- -- -- 151503550
523 cnvi16640453
4 -- -- -- -- -- -- -- --
-- -- -- -- -- -- -- --
-- -- -- -- -- -- -- --
-- -- -- -- -- -- -- --
-- -- -- -- 151503950
524 cnvi16640457
4 -- -- -- -- -- -- -- --

	--	--	--	--	--	--	--	--
	--	--	--	--	--	--	--	--
	--	--	--	--	--	--	--	--
	--	--	--	--	151504150			
525	cnvi16640465							
4	--	--	--	--	--	--	--	--
	--	--	--	--	--	--	--	--
	--	--	--	--	--	--	--	--
	--	--	--	--	--	--	--	--
	--	--	--	--	151504550			
526	cnvi16640469							
4	--	--	--	--	--	--	--	--
	--	--	--	--	--	--	--	--
	--	--	--	--	--	--	--	--
	--	--	--	--	--	--	--	--
	--	--	--	--	151504750			
527	cnvi16640477							
4	--	--	--	--	--	--	--	--
	--	--	--	--	--	--	--	--
	--	--	--	--	--	--	--	--
	--	--	--	--	--	--	--	--
	--	--	--	--	151505150			
528	cnvi16640482							
4	--	--	--	--	--	--	--	--
	--	--	--	--	--	--	--	--
	--	--	--	--	--	--	--	--
	--	--	--	--	--	--	--	--
	--	--	--	--	151505400			
529	cnvi18233546							
9	--	--	--	--	--	--	--	--
	--	--	--	--	--	--	--	--
	--	--	--	--	--	--	--	--
	--	--	--	--	--	--	--	--
	--	--	--	--	40305200			
530	cnvi18233842							
9	--	--	--	--	--	--	--	--
	--	--	--	--	--	--	--	--
	--	--	--	--	--	--	--	--
	--	--	--	--	--	--	--	--
	--	--	--	--	40320000			
531	cnvi18234092							
9	--	--	--	--	--	--	--	--
	--	--	--	--	--	--	--	--
	--	--	--	--	--	--	--	--
	--	--	--	--	--	--	--	--
	--	--	--	--	40332500			
532	cnvi18234310							
9	--	--	--	--	--	--	--	--
	--	--	--	--	--	--	--	--
	--	--	--	--	--	--	--	--

	--	--	--	--	--	--	--	--
	--	--	--	--	40343400			
533	cnvi18236496							
9	--	--	--	--	--	--	--	--
	--	--	--	--	--	--	--	--
	--	--	--	--	--	--	--	--
	--	--	--	--	--	--	--	--
	--	--	--	--	40502750			
534	cnvi18242850							
9	--	--	--	--	--	--	--	--
	--	--	--	--	--	--	--	--
	--	--	--	--	--	--	--	--
	--	--	--	--	--	--	--	--
	--	--	--	--	40820450			
535	cnvi18243008							
9	--	--	--	--	--	--	--	--
	--	--	--	--	--	--	--	--
	--	--	--	--	--	--	--	--
	--	--	--	--	--	--	--	--
	--	--	--	--	40828350			
536	cnvi18244421							
9	--	--	--	--	--	--	--	--
	--	--	--	--	--	--	--	--
	--	--	--	--	--	--	--	--
	--	--	--	--	--	--	--	--
	--	--	--	--	40899000			
537	cnvi18249073							
9	--	--	--	--	--	--	--	--
	--	--	--	--	--	--	--	--
	--	--	--	--	--	--	--	--
	--	--	--	--	--	--	--	--
	--	--	--	--	41231700			
538	cnvi18257367							
9	--	--	--	--	--	--	--	--
	--	--	--	--	--	--	--	--
	--	--	--	--	--	--	--	--
	--	--	--	--	--	--	--	--
	--	--	--	--	41696450			
539	cnvi18261680							
9	--	--	--	--	--	--	--	--
	--	--	--	--	--	--	--	--
	--	--	--	--	--	--	--	--
	--	--	--	--	--	--	--	--
	--	--	--	--	41912100			
540	cnvi18261775							
9	--	--	--	--	--	--	--	--
	--	--	--	--	--	--	--	--
	--	--	--	--	--	--	--	--
	--	--	--	--	--	--	--	--
	--	--	--	--	41916850			

541 cnvi18262017
9 -- -- -- -- -- -- -- --
-- -- -- -- -- -- -- --
-- -- -- -- -- -- -- --
-- -- -- -- -- -- -- --
-- -- -- -- 41928950
542 cnvi18262108
9 -- -- -- -- -- -- -- --
-- -- -- -- -- -- -- --
-- -- -- -- -- -- -- --
-- -- -- -- -- -- -- --
-- -- -- -- 41933500
543 cnvi18262401
9 -- -- -- -- -- -- -- --
-- -- -- -- -- -- -- --
-- -- -- -- -- -- -- --
-- -- -- -- -- -- -- --
-- -- -- -- 41948150
544 cnvi18270622
9 -- -- -- -- -- -- -- --
-- -- -- -- -- -- -- --
-- -- -- -- -- -- -- --
-- -- -- -- -- -- -- --
-- -- -- -- 42359204
545 cnvi19862298
17 -- -- -- -- -- -- -- --
-- -- -- -- -- -- -- --
-- -- -- -- -- -- -- --
-- -- -- -- -- -- -- --
-- -- -- -- 1541100
546 cnvi19862307
17 -- -- -- -- -- -- -- --
-- -- -- -- -- -- -- --
-- -- -- -- -- -- -- --
-- -- -- -- -- -- -- --
-- -- -- -- 1541550
547 cnvi19862452
17 -- -- -- -- -- -- -- --
-- -- -- -- -- -- -- --
-- -- -- -- -- -- -- --
-- -- -- -- -- -- -- --
-- -- -- -- 1548800
548 cnvi19862470
17 -- -- -- -- -- -- -- --
-- -- -- -- -- -- -- --
-- -- -- -- -- -- -- --
-- -- -- -- -- -- -- --
-- -- -- -- 1549700
549 cnvi19862483
17 -- -- -- -- -- -- -- --

	--	--	--	--	--	--	--	--
	--	--	--	--	--	--	--	--
	--	--	--	--	--	--	--	--
	--	--	--	--	1550350			
550	cnvi19862503							
17	--	--	--	--	--	--	--	--
	--	--	--	--	--	--	--	--
	--	--	--	--	--	--	--	--
	--	--	--	--	--	--	--	--
	--	--	--	--	1551350			
551	cnvi19862506							
17	--	--	--	--	--	--	--	--
	--	--	--	--	--	--	--	--
	--	--	--	--	--	--	--	--
	--	--	--	--	--	--	--	--
	--	--	--	--	1551500			
552	cnvi19862512							
17	--	--	--	--	--	--	--	--
	--	--	--	--	--	--	--	--
	--	--	--	--	--	--	--	--
	--	--	--	--	--	--	--	--
	--	--	--	--	1551800			
553	cnvi19862515							
17	--	--	--	--	--	--	--	--
	--	--	--	--	--	--	--	--
	--	--	--	--	--	--	--	--
	--	--	--	--	--	--	--	--
	--	--	--	--	1551950			
554	cnvi19862526							
17	--	--	--	--	--	--	--	--
	--	--	--	--	--	--	--	--
	--	--	--	--	--	--	--	--
	--	--	--	--	--	--	--	--
	--	--	--	--	1552500			
555	cnvi19862539							
17	--	--	--	--	--	--	--	--
	--	--	--	--	--	--	--	--
	--	--	--	--	--	--	--	--
	--	--	--	--	--	--	--	--
	--	--	--	--	1553150			
556	cnvi19981190							
17	--	--	--	--	--	--	--	--
	--	--	--	--	--	--	--	--
	--	--	--	--	--	--	--	--
	--	--	--	--	--	--	--	--
	--	--	--	--	7491726			
557	cnvi19981207							
17	--	--	--	--	--	--	--	--
	--	--	--	--	--	--	--	--
	--	--	--	--	--	--	--	--

	--	--	--	--	--	--	--	--
	--	--	--	--	7492576			
558	cnvi19981211							
17	--	--	--	--	--	--	--	--
	--	--	--	--	--	--	--	--
	--	--	--	--	--	--	--	--
	--	--	--	--	--	--	--	--
	--	--	--	--	7492776			
559	cnvi19981221							
17	--	--	--	--	--	--	--	--
	--	--	--	--	--	--	--	--
	--	--	--	--	--	--	--	--
	--	--	--	--	--	--	--	--
	--	--	--	--	7493276			
560	cnvi20461503							
17	--	--	--	--	--	--	--	--
	--	--	--	--	--	--	--	--
	--	--	--	--	--	--	--	--
	--	--	--	--	--	--	--	--
	--	--	--	--	34624087			
561	cnvi20461506							
17	--	--	--	--	--	--	--	--
	--	--	--	--	--	--	--	--
	--	--	--	--	--	--	--	--
	--	--	--	--	--	--	--	--
	--	--	--	--	34624237			
562	cnvi20461512							
17	--	--	--	--	--	--	--	--
	--	--	--	--	--	--	--	--
	--	--	--	--	--	--	--	--
	--	--	--	--	--	--	--	--
	--	--	--	--	34624537			
563	cnvi20461518							
17	--	--	--	--	--	--	--	--
	--	--	--	--	--	--	--	--
	--	--	--	--	--	--	--	--
	--	--	--	--	--	--	--	--
	--	--	--	--	34624837			
564	cnvi20461524							
17	--	--	--	--	--	--	--	--
	--	--	--	--	--	--	--	--
	--	--	--	--	--	--	--	--
	--	--	--	--	--	--	--	--
	--	--	--	--	34625137			
565	cnvi20461527							
17	--	--	--	--	--	--	--	--
	--	--	--	--	--	--	--	--
	--	--	--	--	--	--	--	--
	--	--	--	--	--	--	--	--
	--	--	--	--	34625287			

566	cnvi20496399							
17	--	--	--	--	--	--	--	--
	--	--	--	--	--	--	--	--
	--	--	--	--	--	--	--	--
	--	--	--	--	--	--	--	--
	--	--	--	--	36339294			
567	cnvi20496417							
17	--	--	--	--	--	--	--	--
	--	--	--	--	--	--	--	--
	--	--	--	--	--	--	--	--
	--	--	--	--	--	--	--	--
	--	--	--	--	36340194			
568	cnvi20496489							
17	--	--	--	--	--	--	--	--
	--	--	--	--	--	--	--	--
	--	--	--	--	--	--	--	--
	--	--	--	--	--	--	--	--
	--	--	--	--	36343793			
569	cnvi20496507							
17	--	--	--	--	--	--	--	--
	--	--	--	--	--	--	--	--
	--	--	--	--	--	--	--	--
	--	--	--	--	--	--	--	--
	--	--	--	--	36344693			
570	cnvi20496525							
17	--	--	--	--	--	--	--	--
	--	--	--	--	--	--	--	--
	--	--	--	--	--	--	--	--
	--	--	--	--	--	--	--	--
	--	--	--	--	36345593			
571	cnvi20701788							
17	--	--	--	--	--	--	--	--
	--	--	--	--	--	--	--	--
	--	--	--	--	--	--	--	--
	--	--	--	--	--	--	--	--
	--	--	--	--	46607601			
572	cnvi20701790							
17	--	--	--	--	--	--	--	--
	--	--	--	--	--	--	--	--
	--	--	--	--	--	--	--	--
	--	--	--	--	--	--	--	--
	--	--	--	--	46607701			
573	cnvi20727991							
17	--	--	--	--	--	--	--	--
	--	--	--	--	--	--	--	--
	--	--	--	--	--	--	--	--
	--	--	--	--	--	--	--	--
	--	--	--	--	47917751			
574	cnvi20728027							
17	--	--	--	--	--	--	--	--

	--	--	--	--	--	--	--	--
	--	--	--	--	--	--	--	--
	--	--	--	--	--	--	--	--
	--	--	--	--	47919551			
575	cnvi20728067							
17	--	--	--	--	--	--	--	--
	--	--	--	--	--	--	--	--
	--	--	--	--	--	--	--	--
	--	--	--	--	--	--	--	--
	--	--	--	--	47921551			
576	cnvi21594192							
14	--	--	--	--	--	--	--	--
	--	--	--	--	--	--	--	--
	--	--	--	--	--	--	--	--
	--	--	--	--	--	--	--	--
	--	--	--	--	29236499			
577	cnvi21594197							
14	--	--	--	--	--	--	--	--
	--	--	--	--	--	--	--	--
	--	--	--	--	--	--	--	--
	--	--	--	--	--	--	--	--
	--	--	--	--	29236749			
578	cnvi21594207							
14	--	--	--	--	--	--	--	--
	--	--	--	--	--	--	--	--
	--	--	--	--	--	--	--	--
	--	--	--	--	--	--	--	--
	--	--	--	--	29237249			
579	cnvi21594212							
14	--	--	--	--	--	--	--	--
	--	--	--	--	--	--	--	--
	--	--	--	--	--	--	--	--
	--	--	--	--	--	--	--	--
	--	--	--	--	29237499			
580	cnvi21594223							
14	--	--	--	--	--	--	--	--
	--	--	--	--	--	--	--	--
	--	--	--	--	--	--	--	--
	--	--	--	--	--	--	--	--
	--	--	--	--	29238049			
581	cnvi21594231							
14	--	--	--	--	--	--	--	--
	--	--	--	--	--	--	--	--
	--	--	--	--	--	--	--	--
	--	--	--	--	--	--	--	--
	--	--	--	--	29238449			
582	cnvi21750458							
14	--	--	--	--	--	--	--	--
	--	--	--	--	--	--	--	--
	--	--	--	--	--	--	--	--

	--	--	--	--	--	--	--	--
	--	--	--	--	37049799			
583	cnvi21750465							
14	--	--	--	--	--	--	--	--
	--	--	--	--	--	--	--	--
	--	--	--	--	--	--	--	--
	--	--	--	--	--	--	--	--
	--	--	--	--	37050149			
584	cnvi21750469							
14	--	--	--	--	--	--	--	--
	--	--	--	--	--	--	--	--
	--	--	--	--	--	--	--	--
	--	--	--	--	--	--	--	--
	--	--	--	--	37050349			
585	cnvi21750485							
14	--	--	--	--	--	--	--	--
	--	--	--	--	--	--	--	--
	--	--	--	--	--	--	--	--
	--	--	--	--	--	--	--	--
	--	--	--	--	37051149			
586	cnvi23177506							
1	--	--	--	--	--	--	--	--
	--	--	--	--	--	--	--	--
	--	--	--	--	--	--	--	--
	--	--	--	--	--	--	--	--
	--	--	--	--	1271687			
587	cnvi23177516							
1	--	--	--	--	--	--	--	--
	--	--	--	--	--	--	--	--
	--	--	--	--	--	--	--	--
	--	--	--	--	--	--	--	--
	--	--	--	--	1272187			
588	cnvi23177549							
1	--	--	--	--	--	--	--	--
	--	--	--	--	--	--	--	--
	--	--	--	--	--	--	--	--
	--	--	--	--	--	--	--	--
	--	--	--	--	1273837			
589	cnvi23177611							
1	--	--	--	--	--	--	--	--
	--	--	--	--	--	--	--	--
	--	--	--	--	--	--	--	--
	--	--	--	--	--	--	--	--
	--	--	--	--	1276937			
590	cnvi23177621							
1	--	--	--	--	--	--	--	--
	--	--	--	--	--	--	--	--
	--	--	--	--	--	--	--	--
	--	--	--	--	--	--	--	--
	--	--	--	--	1277437			

591 cnvi23177631
1 -- -- -- -- -- -- -- --
-- -- -- -- -- -- -- --
-- -- -- -- -- -- -- --
-- -- -- -- -- -- -- --
-- -- -- -- 1277937
592 cnvi23177651
1 -- -- -- -- -- -- -- --
-- -- -- -- -- -- -- --
-- -- -- -- -- -- -- --
-- -- -- -- -- -- -- --
-- -- -- -- 1278937
593 cnvi23909721
1 -- -- -- -- -- -- -- --
-- -- -- -- -- -- -- --
-- -- -- -- -- -- -- --
-- -- -- -- -- -- -- --
-- -- -- -- 38510113
594 cnvi23909742
1 -- -- -- -- -- -- -- --
-- -- -- -- -- -- -- --
-- -- -- -- -- -- -- --
-- -- -- -- -- -- -- --
-- -- -- -- 38511163
595 cnvi23909747
1 -- -- -- -- -- -- -- --
-- -- -- -- -- -- -- --
-- -- -- -- -- -- -- --
-- -- -- -- -- -- -- --
-- -- -- -- 38511413
596 cnvi23909752
1 -- -- -- -- -- -- -- --
-- -- -- -- -- -- -- --
-- -- -- -- -- -- -- --
-- -- -- -- -- -- -- --
-- -- -- -- 38511663
597 cnvi23909762
1 -- -- -- -- -- -- -- --
-- -- -- -- -- -- -- --
-- -- -- -- -- -- -- --
-- -- -- -- -- -- -- --
-- -- -- -- 38512163
598 cnvi24097155
1 -- -- -- -- -- -- -- --
-- -- -- -- -- -- -- --
-- -- -- -- -- -- -- --
-- -- -- -- -- -- -- --
-- -- -- -- 47881813
599 cnvi24097183
1 -- -- -- -- -- -- -- --

	--	--	--	--	--	--	--	--
	--	--	--	--	--	--	--	--
	--	--	--	--	--	--	--	--
	--	--	--	--	47883213			
600	cnvi24097186							
1	--	--	--	--	--	--	--	--
	--	--	--	--	--	--	--	--
	--	--	--	--	--	--	--	--
	--	--	--	--	--	--	--	--
	--	--	--	--	47883363			
601	cnvi24097189							
1	--	--	--	--	--	--	--	--
	--	--	--	--	--	--	--	--
	--	--	--	--	--	--	--	--
	--	--	--	--	--	--	--	--
	--	--	--	--	47883513			
602	cnvi24415298							
1	--	--	--	--	--	--	--	--
	--	--	--	--	--	--	--	--
	--	--	--	--	--	--	--	--
	--	--	--	--	--	--	--	--
	--	--	--	--	63788962			
603	cnvi24415301							
1	--	--	--	--	--	--	--	--
	--	--	--	--	--	--	--	--
	--	--	--	--	--	--	--	--
	--	--	--	--	--	--	--	--
	--	--	--	--	63789112			
604	cnvi24415308							
1	--	--	--	--	--	--	--	--
	--	--	--	--	--	--	--	--
	--	--	--	--	--	--	--	--
	--	--	--	--	--	--	--	--
	--	--	--	--	63789462			
605	cnvi24415312							
1	--	--	--	--	--	--	--	--
	--	--	--	--	--	--	--	--
	--	--	--	--	--	--	--	--
	--	--	--	--	--	--	--	--
	--	--	--	--	63789662			
606	cnvi24415314							
1	--	--	--	--	--	--	--	--
	--	--	--	--	--	--	--	--
	--	--	--	--	--	--	--	--
	--	--	--	--	--	--	--	--
	--	--	--	--	63789762			
607	cnvi24415318							
1	--	--	--	--	--	--	--	--
	--	--	--	--	--	--	--	--
	--	--	--	--	--	--	--	--

-- -- -- -- -- -- -- --
-- -- -- -- 63789962
608 cnvi26689105
1 -- -- -- -- -- -- -- --
-- -- -- -- -- -- -- --
-- -- -- -- -- -- -- --
-- -- -- -- -- -- -- --
-- -- -- -- 200376377
609 cnvi26689124
1 -- -- -- -- -- -- -- --
-- -- -- -- -- -- -- --
-- -- -- -- -- -- -- --
-- -- -- -- -- -- -- --
-- -- -- -- 200377327
610 cnvi26689131
1 -- -- -- -- -- -- -- --
-- -- -- -- -- -- -- --
-- -- -- -- -- -- -- --
-- -- -- -- -- -- -- --
-- -- -- -- 200377677
611 cnvi26689151
1 -- -- -- -- -- -- -- --
-- -- -- -- -- -- -- --
-- -- -- -- -- -- -- --
-- -- -- -- -- -- -- --
-- -- -- -- 200378677
612 cnvi27557913
1 -- -- -- -- -- -- -- --
-- -- -- -- -- -- -- --
-- -- -- -- -- -- -- --
-- -- -- -- -- -- -- --
-- -- -- -- 244216977
613 cnvi27557976
1 -- -- -- -- -- -- -- --
-- -- -- -- -- -- -- --
-- -- -- -- -- -- -- --
-- -- -- -- -- -- -- --
-- -- -- -- 244220127
614 cnvi27842184
13 -- -- -- -- -- -- -- --
-- -- -- -- -- -- -- --
-- -- -- -- -- -- -- --
-- -- -- -- -- -- -- --
-- -- -- -- 28367500
615 cnvi27842188
13 -- -- -- -- -- -- -- --
-- -- -- -- -- -- -- --
-- -- -- -- -- -- -- --
-- -- -- -- -- -- -- --
-- -- -- -- 28367700

616 cnvi27842190
13 -- -- -- -- -- -- -- --
-- -- -- -- -- -- -- --
-- -- -- -- -- -- -- --
-- -- -- -- -- -- -- --
-- -- -- -- 28367800
617 cnvi27995811
13 -- -- -- -- -- -- -- --
-- -- -- -- -- -- -- --
-- -- -- -- -- -- -- --
-- -- -- -- -- -- -- --
-- -- -- -- 36048850
618 cnvi27995830
13 -- -- -- -- -- -- -- --
-- -- -- -- -- -- -- --
-- -- -- -- -- -- -- --
-- -- -- -- -- -- -- --
-- -- -- -- 36049800
619 cnvi27995834
13 -- -- -- -- -- -- -- --
-- -- -- -- -- -- -- --
-- -- -- -- -- -- -- --
-- -- -- -- -- -- -- --
-- -- -- -- 36050000
620 cnvi27995842
13 -- -- -- -- -- -- -- --
-- -- -- -- -- -- -- --
-- -- -- -- -- -- -- --
-- -- -- -- -- -- -- --
-- -- -- -- 36050400
621 cnvi28858309
13 -- -- -- -- -- -- -- --
-- -- -- -- -- -- -- --
-- -- -- -- -- -- -- --
-- -- -- -- -- -- -- --
-- -- -- -- 79173749
622 cnvi28858325
13 -- -- -- -- -- -- -- --
-- -- -- -- -- -- -- --
-- -- -- -- -- -- -- --
-- -- -- -- -- -- -- --
-- -- -- -- 79174549
623 cnvi28858364
13 -- -- -- -- -- -- -- --
-- -- -- -- -- -- -- --
-- -- -- -- -- -- -- --
-- -- -- -- -- -- -- --
-- -- -- -- 79176499
624 cnvi2917243
6 -- -- -- -- -- -- -- --

	--	--	--	--	--	--	--	--
	--	--	--	--	--	--	--	--
	--	--	--	--	--	--	--	--
	--	--	--	--	99283029			
625	cnvi2917294							
6	--	--	--	--	--	--	--	--
	--	--	--	--	--	--	--	--
	--	--	--	--	--	--	--	--
	--	--	--	--	--	--	--	--
	--	--	--	--	99285579			
626	cnvi2917301							
6	--	--	--	--	--	--	--	--
	--	--	--	--	--	--	--	--
	--	--	--	--	--	--	--	--
	--	--	--	--	--	--	--	--
	--	--	--	--	99285929			
627	cnvi2917308							
6	--	--	--	--	--	--	--	--
	--	--	--	--	--	--	--	--
	--	--	--	--	--	--	--	--
	--	--	--	--	--	--	--	--
	--	--	--	--	99286279			
628	cnvi29179093							
13	--	--	--	--	--	--	--	--
	--	--	--	--	--	--	--	--
	--	--	--	--	--	--	--	--
	--	--	--	--	--	--	--	--
	--	--	--	--	95362999			
629	cnvi29179101							
13	--	--	--	--	--	--	--	--
	--	--	--	--	--	--	--	--
	--	--	--	--	--	--	--	--
	--	--	--	--	--	--	--	--
	--	--	--	--	95363399			
630	cnvi29179110							
13	--	--	--	--	--	--	--	--
	--	--	--	--	--	--	--	--
	--	--	--	--	--	--	--	--
	--	--	--	--	--	--	--	--
	--	--	--	--	95363849			
631	cnvi29179116							
13	--	--	--	--	--	--	--	--
	--	--	--	--	--	--	--	--
	--	--	--	--	--	--	--	--
	--	--	--	--	--	--	--	--
	--	--	--	--	95364149			
632	cnvi29284584							
13	--	--	--	--	--	--	--	--
	--	--	--	--	--	--	--	--
	--	--	--	--	--	--	--	--

-- -- -- -- -- -- -- --
-- -- -- -- 100637549
633 cnvi29284592
13 -- -- -- -- -- -- -- --
-- -- -- -- -- -- -- --
-- -- -- -- -- -- -- --
-- -- -- -- -- -- -- --
-- -- -- -- 100637949
634 cnvi29523305
13 -- -- -- -- -- -- -- --
-- -- -- -- -- -- -- --
-- -- -- -- -- -- -- --
-- -- -- -- -- -- -- --
-- -- -- -- 112723649
635 cnvi29523312
13 -- -- -- -- -- -- -- --
-- -- -- -- -- -- -- --
-- -- -- -- -- -- -- --
-- -- -- -- -- -- -- --
-- -- -- -- 112723999
636 cnvi29523329
13 -- -- -- -- -- -- -- --
-- -- -- -- -- -- -- --
-- -- -- -- -- -- -- --
-- -- -- -- -- -- -- --
-- -- -- -- 112724849
637 cnvi29764784
3 -- -- -- -- -- -- -- --
-- -- -- -- -- -- -- --
-- -- -- -- -- -- -- --
-- -- -- -- -- -- -- --
-- -- -- -- 9977700
638 cnvi29764799
3 -- -- -- -- -- -- -- --
-- -- -- -- -- -- -- --
-- -- -- -- -- -- -- --
-- -- -- -- -- -- -- --
-- -- -- -- 9978450
639 cnvi29764808
3 -- -- -- -- -- -- -- --
-- -- -- -- -- -- -- --
-- -- -- -- -- -- -- --
-- -- -- -- -- -- -- --
-- -- -- -- 9978900
640 cnvi29764901
3 -- -- -- -- -- -- -- --
-- -- -- -- -- -- -- --
-- -- -- -- -- -- -- --
-- -- -- -- -- -- -- --
-- -- -- -- 9983550

641 cnvi29764910
3 -- -- -- -- -- -- -- --
-- -- -- -- -- -- -- --
-- -- -- -- -- -- -- --
-- -- -- -- -- -- -- --
-- -- -- -- 9984000
642 cnvi29764918
3 -- -- -- -- -- -- -- --
-- -- -- -- -- -- -- --
-- -- -- -- -- -- -- --
-- -- -- -- -- -- -- --
-- -- -- -- 9984400
643 cnvi29764934
3 -- -- -- -- -- -- -- --
-- -- -- -- -- -- -- --
-- -- -- -- -- -- -- --
-- -- -- -- -- -- -- --
-- -- -- -- 9985200
644 cnvi29764961
3 -- -- -- -- -- -- -- --
-- -- -- -- -- -- -- --
-- -- -- -- -- -- -- --
-- -- -- -- -- -- -- --
-- -- -- -- 9986550
645 cnvi32251851
3 -- -- -- -- -- -- -- --
-- -- -- -- -- -- -- --
-- -- -- -- -- -- -- --
-- -- -- -- -- -- -- --
-- -- -- -- 137483660
646 cnvi32251853
3 -- -- -- -- -- -- -- --
-- -- -- -- -- -- -- --
-- -- -- -- -- -- -- --
-- -- -- -- -- -- -- --
-- -- -- -- 137483760
647 cnvi32251855
3 -- -- -- -- -- -- -- --
-- -- -- -- -- -- -- --
-- -- -- -- -- -- -- --
-- -- -- -- -- -- -- --
-- -- -- -- 137483860
648 cnvi32251857
3 -- -- -- -- -- -- -- --
-- -- -- -- -- -- -- --
-- -- -- -- -- -- -- --
-- -- -- -- -- -- -- --
-- -- -- -- 137483960
649 cnvi32251860
3 -- -- -- -- -- -- -- --

-- -- -- -- -- -- -- --
-- -- -- -- -- -- -- --
-- -- -- -- -- -- -- --
-- -- -- -- 137484110
650 cnvi32251862
3 -- -- -- -- -- -- -- --
-- -- -- -- -- -- -- --
-- -- -- -- -- -- -- --
-- -- -- -- -- -- -- --
-- -- -- -- 137484210
651 cnvi32251864
3 -- -- -- -- -- -- -- --
-- -- -- -- -- -- -- --
-- -- -- -- -- -- -- --
-- -- -- -- -- -- -- --
-- -- -- -- 137484310
652 cnvi32444844
3 -- -- -- -- -- -- -- --
-- -- -- -- -- -- -- --
-- -- -- -- -- -- -- --
-- -- -- -- -- -- -- --
-- -- -- -- 147133310
653 cnvi33130781
3 -- -- -- -- -- -- -- --
-- -- -- -- -- -- -- --
-- -- -- -- -- -- -- --
-- -- -- -- -- -- -- --
-- -- -- -- 181430156
654 cnvi33130789
3 -- -- -- -- -- -- -- --
-- -- -- -- -- -- -- --
-- -- -- -- -- -- -- --
-- -- -- -- -- -- -- --
-- -- -- -- 181430556
655 cnvi33130793
3 -- -- -- -- -- -- -- --
-- -- -- -- -- -- -- --
-- -- -- -- -- -- -- --
-- -- -- -- -- -- -- --
-- -- -- -- 181430756
656 cnvi33130801
3 -- -- -- -- -- -- -- --
-- -- -- -- -- -- -- --
-- -- -- -- -- -- -- --
-- -- -- -- -- -- -- --
-- -- -- -- 181431156
657 cnvi33130813
3 -- -- -- -- -- -- -- --
-- -- -- -- -- -- -- --
-- -- -- -- -- -- -- --

	--	--	--	--	--	--	--	--
	--	--	--	--	181431756			
658	cnvi33130818							
3	--	--	--	--	--	--	--	--
	--	--	--	--	--	--	--	--
	--	--	--	--	--	--	--	--
	--	--	--	--	--	--	--	--
	--	--	--	--	181432006			
659	cnvi34804470							
5	--	--	--	--	--	--	--	--
	--	--	--	--	--	--	--	--
	--	--	--	--	--	--	--	--
	--	--	--	--	--	--	--	--
	--	--	--	--	70224894			
660	cnvi34804535							
5	--	--	--	--	--	--	--	--
	--	--	--	--	--	--	--	--
	--	--	--	--	--	--	--	--
	--	--	--	--	--	--	--	--
	--	--	--	--	70228144			
661	cnvi34804747							
5	--	--	--	--	--	--	--	--
	--	--	--	--	--	--	--	--
	--	--	--	--	--	--	--	--
	--	--	--	--	--	--	--	--
	--	--	--	--	70238744			
662	cnvi3686551							
6	--	--	--	--	--	--	--	--
	--	--	--	--	--	--	--	--
	--	--	--	--	--	--	--	--
	--	--	--	--	--	--	--	--
	--	--	--	--	137813507			
663	cnvi3686557							
6	--	--	--	--	--	--	--	--
	--	--	--	--	--	--	--	--
	--	--	--	--	--	--	--	--
	--	--	--	--	--	--	--	--
	--	--	--	--	137813807			
664	cnvi3686561							
6	--	--	--	--	--	--	--	--
	--	--	--	--	--	--	--	--
	--	--	--	--	--	--	--	--
	--	--	--	--	--	--	--	--
	--	--	--	--	137814007			
665	cnvi3686564							
6	--	--	--	--	--	--	--	--
	--	--	--	--	--	--	--	--
	--	--	--	--	--	--	--	--
	--	--	--	--	--	--	--	--
	--	--	--	--	137814157			

666 cnvi3686570
6 -- -- -- -- -- -- -- --
-- -- -- -- -- -- -- --
-- -- -- -- -- -- -- --
-- -- -- -- -- -- -- --
-- -- -- -- 137814457
667 cnvi3686576
6 -- -- -- -- -- -- -- --
-- -- -- -- -- -- -- --
-- -- -- -- -- -- -- --
-- -- -- -- -- -- -- --
-- -- -- -- 137814757
668 cnvi3686583
6 -- -- -- -- -- -- -- --
-- -- -- -- -- -- -- --
-- -- -- -- -- -- -- --
-- -- -- -- -- -- -- --
-- -- -- -- 137815107
669 cnvi37061460
Y -- -- -- -- -- -- -- --
-- -- -- -- -- -- -- --
-- -- -- -- -- -- -- --
-- -- -- -- -- -- -- --
-- -- -- -- 2654900
670 cnvi37061461
Y -- -- -- -- -- -- -- --
-- -- -- -- -- -- -- --
-- -- -- -- -- -- -- --
-- -- -- -- -- -- -- --
-- -- -- -- 2654950
671 cnvi37061462
Y -- -- -- -- -- -- -- --
-- -- -- -- -- -- -- --
-- -- -- -- -- -- -- --
-- -- -- -- -- -- -- --
-- -- -- -- 2655000
672 cnvi37061463
Y -- -- -- -- -- -- -- --
-- -- -- -- -- -- -- --
-- -- -- -- -- -- -- --
-- -- -- -- -- -- -- --
-- -- -- -- 2655050
673 cnvi37061464
Y -- -- -- -- -- -- -- --
-- -- -- -- -- -- -- --
-- -- -- -- -- -- -- --
-- -- -- -- -- -- -- --
-- -- -- -- 2655100
674 cnvi37061465
Y -- -- -- -- -- -- -- --

	--	--	--	--	--	--	--	--
	--	--	--	--	--	--	--	--
	--	--	--	--	--	--	--	--
	--	--	--	--	2655150			
675	cnvi37061467							
Y	--	--	--	--	--	--	--	--
	--	--	--	--	--	--	--	--
	--	--	--	--	--	--	--	--
	--	--	--	--	--	--	--	--
	--	--	--	--	2655250			
676	cnvi37061468							
Y	--	--	--	--	--	--	--	--
	--	--	--	--	--	--	--	--
	--	--	--	--	--	--	--	--
	--	--	--	--	--	--	--	--
	--	--	--	--	2655300			
677	cnvi37061470							
Y	--	--	--	--	--	--	--	--
	--	--	--	--	--	--	--	--
	--	--	--	--	--	--	--	--
	--	--	--	--	--	--	--	--
	--	--	--	--	2655400			
678	cnvi37061471							
Y	--	--	--	--	--	--	--	--
	--	--	--	--	--	--	--	--
	--	--	--	--	--	--	--	--
	--	--	--	--	--	--	--	--
	--	--	--	--	2655450			
679	cnvi37061472							
Y	--	--	--	--	--	--	--	--
	--	--	--	--	--	--	--	--
	--	--	--	--	--	--	--	--
	--	--	--	--	--	--	--	--
	--	--	--	--	2655500			
680	cnvi37061473							
Y	--	--	--	--	--	--	--	--
	--	--	--	--	--	--	--	--
	--	--	--	--	--	--	--	--
	--	--	--	--	--	--	--	--
	--	--	--	--	2655550			
681	cnvi37061476							
Y	--	--	--	--	--	--	--	--
	--	--	--	--	--	--	--	--
	--	--	--	--	--	--	--	--
	--	--	--	--	--	--	--	--
	--	--	--	--	2655700			
682	cnvi37486671							
Y	--	--	--	--	--	--	--	--
	--	--	--	--	--	--	--	--
	--	--	--	--	--	--	--	--

-- -- -- -- -- -- -- --
-- -- -- -- 27266362
683 cnvi37490157
Y -- -- -- -- -- -- -- --
-- -- -- -- -- -- -- --
-- -- -- -- -- -- -- --
-- -- -- -- -- -- -- --
-- -- -- -- 27440662
684 cnvi37490318
Y -- -- -- -- -- -- -- --
-- -- -- -- -- -- -- --
-- -- -- -- -- -- -- --
-- -- -- -- -- -- -- --
-- -- -- -- 27448712
685 cnvi37490498
Y -- -- -- -- -- -- -- --
-- -- -- -- -- -- -- --
-- -- -- -- -- -- -- --
-- -- -- -- -- -- -- --
-- -- -- -- 27457712
686 cnvi37490679
Y -- -- -- -- -- -- -- --
-- -- -- -- -- -- -- --
-- -- -- -- -- -- -- --
-- -- -- -- -- -- -- --
-- -- -- -- 27466762
687 cnvi37490845
Y -- -- -- -- -- -- -- --
-- -- -- -- -- -- -- --
-- -- -- -- -- -- -- --
-- -- -- -- -- -- -- --
-- -- -- -- 27475062
688 cnvi37491043
Y -- -- -- -- -- -- -- --
-- -- -- -- -- -- -- --
-- -- -- -- -- -- -- --
-- -- -- -- -- -- -- --
-- -- -- -- 27484962
689 cnvi37491436
Y -- -- -- -- -- -- -- --
-- -- -- -- -- -- -- --
-- -- -- -- -- -- -- --
-- -- -- -- -- -- -- --
-- -- -- -- 27504612
690 cnvi37491605
Y -- -- -- -- -- -- -- --
-- -- -- -- -- -- -- --
-- -- -- -- -- -- -- --
-- -- -- -- -- -- -- --
-- -- -- -- 27513062

691 cnvi37491927
Y -- -- -- -- -- -- -- --
-- -- -- -- -- -- -- --
-- -- -- -- -- -- -- --
-- -- -- -- -- -- -- --
-- -- -- -- 27529162
692 cnvi37492143
Y -- -- -- -- -- -- -- --
-- -- -- -- -- -- -- --
-- -- -- -- -- -- -- --
-- -- -- -- -- -- -- --
-- -- -- -- 27539962
693 cnvi37492277
Y -- -- -- -- -- -- -- --
-- -- -- -- -- -- -- --
-- -- -- -- -- -- -- --
-- -- -- -- -- -- -- --
-- -- -- -- 27546662
694 cnvi37492578
Y -- -- -- -- -- -- -- --
-- -- -- -- -- -- -- --
-- -- -- -- -- -- -- --
-- -- -- -- -- -- -- --
-- -- -- -- 27561712
695 cnvi37492755
Y -- -- -- -- -- -- -- --
-- -- -- -- -- -- -- --
-- -- -- -- -- -- -- --
-- -- -- -- -- -- -- --
-- -- -- -- 27570562
696 cnvi37492915
Y -- -- -- -- -- -- -- --
-- -- -- -- -- -- -- --
-- -- -- -- -- -- -- --
-- -- -- -- -- -- -- --
-- -- -- -- 27578562
697 cnvi37493077
Y -- -- -- -- -- -- -- --
-- -- -- -- -- -- -- --
-- -- -- -- -- -- -- --
-- -- -- -- -- -- -- --
-- -- -- -- 27586662
698 cnvi37493317
Y -- -- -- -- -- -- -- --
-- -- -- -- -- -- -- --
-- -- -- -- -- -- -- --
-- -- -- -- -- -- -- --
-- -- -- -- 27598662
699 cnvi37493521
Y -- -- -- -- -- -- -- --

-- -- -- -- -- -- -- --
-- -- -- -- -- -- -- --
-- -- -- -- -- -- -- --
-- -- -- -- 27608862
700 cnvi37493673
Y -- -- -- -- -- -- -- --
-- -- -- -- -- -- -- --
-- -- -- -- -- -- -- --
-- -- -- -- -- -- -- --
-- -- -- -- 27616462
701 cnvi37493910
Y -- -- -- -- -- -- -- --
-- -- -- -- -- -- -- --
-- -- -- -- -- -- -- --
-- -- -- -- -- -- -- --
-- -- -- -- 27628312
702 cnvi37494115
Y -- -- -- -- -- -- -- --
-- -- -- -- -- -- -- --
-- -- -- -- -- -- -- --
-- -- -- -- -- -- -- --
-- -- -- -- 27638562
703 cnvi37494315
Y -- -- -- -- -- -- -- --
-- -- -- -- -- -- -- --
-- -- -- -- -- -- -- --
-- -- -- -- -- -- -- --
-- -- -- -- 27648562
704 cnvi37494503
Y -- -- -- -- -- -- -- --
-- -- -- -- -- -- -- --
-- -- -- -- -- -- -- --
-- -- -- -- -- -- -- --
-- -- -- -- 27657962
705 cnvi37494763
Y -- -- -- -- -- -- -- --
-- -- -- -- -- -- -- --
-- -- -- -- -- -- -- --
-- -- -- -- -- -- -- --
-- -- -- -- 27670962
706 cnvi37494914
Y -- -- -- -- -- -- -- --
-- -- -- -- -- -- -- --
-- -- -- -- -- -- -- --
-- -- -- -- -- -- -- --
-- -- -- -- 27678512
707 cnvi37495113
Y -- -- -- -- -- -- -- --
-- -- -- -- -- -- -- --
-- -- -- -- -- -- -- --

-- -- -- -- -- -- -- --
-- -- -- -- 27688462
708 cnvi37495316
Y -- -- -- -- -- -- -- --
-- -- -- -- -- -- -- --
-- -- -- -- -- -- -- --
-- -- -- -- -- -- -- --
-- -- -- -- 27698612
709 cnvi37495516
Y -- -- -- -- -- -- -- --
-- -- -- -- -- -- -- --
-- -- -- -- -- -- -- --
-- -- -- -- -- -- -- --
-- -- -- -- 27708612
710 cnvi37495707
Y -- -- -- -- -- -- -- --
-- -- -- -- -- -- -- --
-- -- -- -- -- -- -- --
-- -- -- -- -- -- -- --
-- -- -- -- 27718162
711 cnvi37495893
Y -- -- -- -- -- -- -- --
-- -- -- -- -- -- -- --
-- -- -- -- -- -- -- --
-- -- -- -- -- -- -- --
-- -- -- -- 27727462
712 cnvi37496115
Y -- -- -- -- -- -- -- --
-- -- -- -- -- -- -- --
-- -- -- -- -- -- -- --
-- -- -- -- -- -- -- --
-- -- -- -- 27738562
713 cnvi37496298
Y -- -- -- -- -- -- -- --
-- -- -- -- -- -- -- --
-- -- -- -- -- -- -- --
-- -- -- -- -- -- -- --
-- -- -- -- 27747712
714 cnvi37496596
Y -- -- -- -- -- -- -- --
-- -- -- -- -- -- -- --
-- -- -- -- -- -- -- --
-- -- -- -- -- -- -- --
-- -- -- -- 27762612
715 cnvi37496755
Y -- -- -- -- -- -- -- --
-- -- -- -- -- -- -- --
-- -- -- -- -- -- -- --
-- -- -- -- -- -- -- --
-- -- -- -- 27770562

716 cnvi37497040
Y -- -- -- -- -- -- -- --
-- -- -- -- -- -- -- --
-- -- -- -- -- -- -- --
-- -- -- -- -- -- -- --
-- -- -- -- 27784812
717 cnvi37497198
Y -- -- -- -- -- -- -- --
-- -- -- -- -- -- -- --
-- -- -- -- -- -- -- --
-- -- -- -- -- -- -- --
-- -- -- -- 27792712
718 cnvi37497359
Y -- -- -- -- -- -- -- --
-- -- -- -- -- -- -- --
-- -- -- -- -- -- -- --
-- -- -- -- -- -- -- --
-- -- -- -- 27800762
719 cnvi37497520
Y -- -- -- -- -- -- -- --
-- -- -- -- -- -- -- --
-- -- -- -- -- -- -- --
-- -- -- -- -- -- -- --
-- -- -- -- 27808812
720 cnvi37497721
Y -- -- -- -- -- -- -- --
-- -- -- -- -- -- -- --
-- -- -- -- -- -- -- --
-- -- -- -- -- -- -- --
-- -- -- -- 27818862
721 cnvi37498233
Y -- -- -- -- -- -- -- --
-- -- -- -- -- -- -- --
-- -- -- -- -- -- -- --
-- -- -- -- -- -- -- --
-- -- -- -- 27844462
722 cnvi37498639
Y -- -- -- -- -- -- -- --
-- -- -- -- -- -- -- --
-- -- -- -- -- -- -- --
-- -- -- -- -- -- -- --
-- -- -- -- 27864762
723 cnvi37500196
Y -- -- -- -- -- -- -- --
-- -- -- -- -- -- -- --
-- -- -- -- -- -- -- --
-- -- -- -- -- -- -- --
-- -- -- -- 27942612
724 cnvi37501116
Y -- -- -- -- -- -- -- --

-- -- -- -- -- -- -- --
-- -- -- -- -- -- -- --
-- -- -- -- -- -- -- --
-- -- -- -- 27988612
725 cnvi37501279
Y -- -- -- -- -- -- -- --
-- -- -- -- -- -- -- --
-- -- -- -- -- -- -- --
-- -- -- -- -- -- -- --
-- -- -- -- 27996762
726 cnvi37501436
Y -- -- -- -- -- -- -- --
-- -- -- -- -- -- -- --
-- -- -- -- -- -- -- --
-- -- -- -- -- -- -- --
-- -- -- -- 28004612
727 cnvi37501618
Y -- -- -- -- -- -- -- --
-- -- -- -- -- -- -- --
-- -- -- -- -- -- -- --
-- -- -- -- -- -- -- --
-- -- -- -- 28013712
728 cnvi37501999
Y -- -- -- -- -- -- -- --
-- -- -- -- -- -- -- --
-- -- -- -- -- -- -- --
-- -- -- -- -- -- -- --
-- -- -- -- 28032762
729 cnvi37502177
Y -- -- -- -- -- -- -- --
-- -- -- -- -- -- -- --
-- -- -- -- -- -- -- --
-- -- -- -- -- -- -- --
-- -- -- -- 28041662
730 cnvi37502356
Y -- -- -- -- -- -- -- --
-- -- -- -- -- -- -- --
-- -- -- -- -- -- -- --
-- -- -- -- -- -- -- --
-- -- -- -- 28050612
731 cnvi37502545
Y -- -- -- -- -- -- -- --
-- -- -- -- -- -- -- --
-- -- -- -- -- -- -- --
-- -- -- -- -- -- -- --
-- -- -- -- 28060062
732 cnvi37502684
Y -- -- -- -- -- -- -- --
-- -- -- -- -- -- -- --
-- -- -- -- -- -- -- --

-- -- -- -- -- -- -- --
-- -- -- -- 28067012
733 cnvi37502886
Y -- -- -- -- -- -- -- --
-- -- -- -- -- -- -- --
-- -- -- -- -- -- -- --
-- -- -- -- -- -- -- --
-- -- -- -- 28077112
734 cnvi37503072
Y -- -- -- -- -- -- -- --
-- -- -- -- -- -- -- --
-- -- -- -- -- -- -- --
-- -- -- -- -- -- -- --
-- -- -- -- 28086412
735 cnvi37503241
Y -- -- -- -- -- -- -- --
-- -- -- -- -- -- -- --
-- -- -- -- -- -- -- --
-- -- -- -- -- -- -- --
-- -- -- -- 28094862
736 cnvi37503396
Y -- -- -- -- -- -- -- --
-- -- -- -- -- -- -- --
-- -- -- -- -- -- -- --
-- -- -- -- -- -- -- --
-- -- -- -- 28102612
737 cnvi37503576
Y -- -- -- -- -- -- -- --
-- -- -- -- -- -- -- --
-- -- -- -- -- -- -- --
-- -- -- -- -- -- -- --
-- -- -- -- 28111612
738 cnvi37508318
Y -- -- -- -- -- -- -- --
-- -- -- -- -- -- -- --
-- -- -- -- -- -- -- --
-- -- -- -- -- -- -- --
-- -- -- -- 28348712
739 cnvi37510819
Y -- -- -- -- -- -- -- --
-- -- -- -- -- -- -- --
-- -- -- -- -- -- -- --
-- -- -- -- -- -- -- --
-- -- -- -- 28473762
740 cnvi37510894
Y -- -- -- -- -- -- -- --
-- -- -- -- -- -- -- --
-- -- -- -- -- -- -- --
-- -- -- -- -- -- -- --
-- -- -- -- 28477512

741 cnvi37511051
Y -- -- -- -- -- -- -- --
-- -- -- -- -- -- -- --
-- -- -- -- -- -- -- --
-- -- -- -- -- -- -- --
-- -- -- -- 28485362
742 cnvi37511438
Y -- -- -- -- -- -- -- --
-- -- -- -- -- -- -- --
-- -- -- -- -- -- -- --
-- -- -- -- -- -- -- --
-- -- -- -- 28504712
743 cnvi37511711
Y -- -- -- -- -- -- -- --
-- -- -- -- -- -- -- --
-- -- -- -- -- -- -- --
-- -- -- -- -- -- -- --
-- -- -- -- 28518362
744 cnvi37511944
Y -- -- -- -- -- -- -- --
-- -- -- -- -- -- -- --
-- -- -- -- -- -- -- --
-- -- -- -- -- -- -- --
-- -- -- -- 28530012
745 cnvi37513317
Y -- -- -- -- -- -- -- --
-- -- -- -- -- -- -- --
-- -- -- -- -- -- -- --
-- -- -- -- -- -- -- --
-- -- -- -- 28598662
746 cnvi37513676
Y -- -- -- -- -- -- -- --
-- -- -- -- -- -- -- --
-- -- -- -- -- -- -- --
-- -- -- -- -- -- -- --
-- -- -- -- 28616612
747 cnvi37513788
Y -- -- -- -- -- -- -- --
-- -- -- -- -- -- -- --
-- -- -- -- -- -- -- --
-- -- -- -- -- -- -- --
-- -- -- -- 28622212
748 cnvi37513875
Y -- -- -- -- -- -- -- --
-- -- -- -- -- -- -- --
-- -- -- -- -- -- -- --
-- -- -- -- -- -- -- --
-- -- -- -- 28626562
749 cnvi37513993
Y -- -- -- -- -- -- -- --

-- -- -- -- -- -- -- --
-- -- -- -- -- -- -- --
-- -- -- -- -- -- -- --
-- -- -- -- 28632462
750 cnvi37514090
Y -- -- -- -- -- -- -- --
-- -- -- -- -- -- -- --
-- -- -- -- -- -- -- --
-- -- -- -- -- -- -- --
-- -- -- -- 28637312
751 cnvi37514227
Y -- -- -- -- -- -- -- --
-- -- -- -- -- -- -- --
-- -- -- -- -- -- -- --
-- -- -- -- -- -- -- --
-- -- -- -- 28644162
752 cnvi37514449
Y -- -- -- -- -- -- -- --
-- -- -- -- -- -- -- --
-- -- -- -- -- -- -- --
-- -- -- -- -- -- -- --
-- -- -- -- 28655262
753 cnvi37514874
Y -- -- -- -- -- -- -- --
-- -- -- -- -- -- -- --
-- -- -- -- -- -- -- --
-- -- -- -- -- -- -- --
-- -- -- -- 28676512
754 cnvi37515721
Y -- -- -- -- -- -- -- --
-- -- -- -- -- -- -- --
-- -- -- -- -- -- -- --
-- -- -- -- -- -- -- --
-- -- -- -- 28718862
755 cnvi37516747
Y -- -- -- -- -- -- -- --
-- -- -- -- -- -- -- --
-- -- -- -- -- -- -- --
-- -- -- -- -- -- -- --
-- -- -- -- 28770162
756 cnvi37516911
Y -- -- -- -- -- -- -- --
-- -- -- -- -- -- -- --
-- -- -- -- -- -- -- --
-- -- -- -- -- -- -- --
-- -- -- -- 28778362
757 cnvi37517006
Y -- -- -- -- -- -- -- --
-- -- -- -- -- -- -- --
-- -- -- -- -- -- -- --

-- -- -- -- -- -- -- --
-- -- -- -- 28783112
758 cnvi37517080
Y -- -- -- -- -- -- -- --
-- -- -- -- -- -- -- --
-- -- -- -- -- -- -- --
-- -- -- -- -- -- -- --
-- -- -- -- 28786812
759 cnvi39944235
12 -- -- -- -- -- -- -- --
-- -- -- -- -- -- -- --
-- -- -- -- -- -- -- --
-- -- -- -- -- -- -- --
-- -- -- -- 49373083
760 cnvi39944279
12 -- -- -- -- -- -- -- --
-- -- -- -- -- -- -- --
-- -- -- -- -- -- -- --
-- -- -- -- -- -- -- --
-- -- -- -- 49375283
761 cnvi42517713
10 -- -- -- -- -- -- -- --
-- -- -- -- -- -- -- --
-- -- -- -- -- -- -- --
-- -- -- -- -- -- -- --
-- -- -- -- 48257094
762 cnvi43443850
10 -- -- -- -- -- -- -- --
-- -- -- -- -- -- -- --
-- -- -- -- -- -- -- --
-- -- -- -- -- -- -- --
-- -- -- -- 94454420
763 cnvi43625375
10 -- -- -- -- -- -- -- --
-- -- -- -- -- -- -- --
-- -- -- -- -- -- -- --
-- -- -- -- -- -- -- --
-- -- -- -- 103530660
764 cnvi43625390
10 -- -- -- -- -- -- -- --
-- -- -- -- -- -- -- --
-- -- -- -- -- -- -- --
-- -- -- -- -- -- -- --
-- -- -- -- 103531410
765 cnvi43625398
10 -- -- -- -- -- -- -- --
-- -- -- -- -- -- -- --
-- -- -- -- -- -- -- --
-- -- -- -- -- -- -- --
-- -- -- -- 103531810

766 cnvi43625437
10 -- -- -- -- -- -- -- --
-- -- -- -- -- -- -- --
-- -- -- -- -- -- -- --
-- -- -- -- -- -- -- --
-- -- -- -- 103533760
767 cnvi43625451
10 -- -- -- -- -- -- -- --
-- -- -- -- -- -- -- --
-- -- -- -- -- -- -- --
-- -- -- -- -- -- -- --
-- -- -- -- 103534460
768 cnvi44297387
11 -- -- -- -- -- -- -- --
-- -- -- -- -- -- -- --
-- -- -- -- -- -- -- --
-- -- -- -- -- -- -- --
-- -- -- -- 2016824
769 cnvi44297404
11 -- -- -- -- -- -- -- --
-- -- -- -- -- -- -- --
-- -- -- -- -- -- -- --
-- -- -- -- -- -- -- --
-- -- -- -- 2017674
770 cnvi44297412
11 -- -- -- -- -- -- -- --
-- -- -- -- -- -- -- --
-- -- -- -- -- -- -- --
-- -- -- -- -- -- -- --
-- -- -- -- 2018074
771 cnvi44297424
11 -- -- -- -- -- -- -- --
-- -- -- -- -- -- -- --
-- -- -- -- -- -- -- --
-- -- -- -- -- -- -- --
-- -- -- -- 2018674
772 cnvi44311421
11 -- -- -- -- -- -- -- --
-- -- -- -- -- -- -- --
-- -- -- -- -- -- -- --
-- -- -- -- -- -- -- --
-- -- -- -- 2718524
773 cnvi44311423
11 -- -- -- -- -- -- -- --
-- -- -- -- -- -- -- --
-- -- -- -- -- -- -- --
-- -- -- -- -- -- -- --
-- -- -- -- 2718624
774 cnvi44315160
11 -- -- -- -- -- -- -- --

	--	--	--	--	--	--	--	--
	--	--	--	--	--	--	--	--
	--	--	--	--	--	--	--	--
	--	--	--	--	2905474			
775	cnvi44315178							
11	--	--	--	--	--	--	--	--
	--	--	--	--	--	--	--	--
	--	--	--	--	--	--	--	--
	--	--	--	--	--	--	--	--
	--	--	--	--	2906374			
776	cnvi44315181							
11	--	--	--	--	--	--	--	--
	--	--	--	--	--	--	--	--
	--	--	--	--	--	--	--	--
	--	--	--	--	--	--	--	--
	--	--	--	--	2906524			
777	cnvi44315187							
11	--	--	--	--	--	--	--	--
	--	--	--	--	--	--	--	--
	--	--	--	--	--	--	--	--
	--	--	--	--	--	--	--	--
	--	--	--	--	2906824			
778	cnvi47053632							
2	--	--	--	--	--	--	--	--
	--	--	--	--	--	--	--	--
	--	--	--	--	--	--	--	--
	--	--	--	--	--	--	--	--
	--	--	--	--	8822349			
779	cnvi47053652							
2	--	--	--	--	--	--	--	--
	--	--	--	--	--	--	--	--
	--	--	--	--	--	--	--	--
	--	--	--	--	--	--	--	--
	--	--	--	--	8823349			
780	cnvi47053664							
2	--	--	--	--	--	--	--	--
	--	--	--	--	--	--	--	--
	--	--	--	--	--	--	--	--
	--	--	--	--	--	--	--	--
	--	--	--	--	8823949			
781	cnvi48890796							
2	--	--	--	--	--	--	--	--
	--	--	--	--	--	--	--	--
	--	--	--	--	--	--	--	--
	--	--	--	--	--	--	--	--
	--	--	--	--	105472518			
782	cnvi50318444							
2	--	--	--	--	--	--	--	--
	--	--	--	--	--	--	--	--
	--	--	--	--	--	--	--	--

	--	--	--	--	--	--	--	--
	--	--	--	--	177055254			
783	cnvi50318447							
2	--	--	--	--	--	--	--	--
	--	--	--	--	--	--	--	--
	--	--	--	--	--	--	--	--
	--	--	--	--	--	--	--	--
	--	--	--	--	177055404			
784	cnvi51517568							
2	--	--	--	--	--	--	--	--
	--	--	--	--	--	--	--	--
	--	--	--	--	--	--	--	--
	--	--	--	--	--	--	--	--
	--	--	--	--	237075011			
785	cnvi51517580							
2	--	--	--	--	--	--	--	--
	--	--	--	--	--	--	--	--
	--	--	--	--	--	--	--	--
	--	--	--	--	--	--	--	--
	--	--	--	--	237075611			
786	cnvi51517599							
2	--	--	--	--	--	--	--	--
	--	--	--	--	--	--	--	--
	--	--	--	--	--	--	--	--
	--	--	--	--	--	--	--	--
	--	--	--	--	237076561			
787	cnvi52086092							
20	--	--	--	--	--	--	--	--
	--	--	--	--	--	--	--	--
	--	--	--	--	--	--	--	--
	--	--	--	--	--	--	--	--
	--	--	--	--	22563000			
788	cnvi52947967							
X	--	--	--	--	--	--	--	--
	--	--	--	--	--	--	--	--
	--	--	--	--	--	--	--	--
	--	--	--	--	--	--	--	--
	--	--	--	--	6452050			
789	cnvi52947975							
X	--	--	--	--	--	--	--	--
	--	--	--	--	--	--	--	--
	--	--	--	--	--	--	--	--
	--	--	--	--	--	--	--	--
	--	--	--	--	6452450			
790	cnvi52947977							
X	--	--	--	--	--	--	--	--
	--	--	--	--	--	--	--	--
	--	--	--	--	--	--	--	--
	--	--	--	--	--	--	--	--
	--	--	--	--	6452550			

791 cnvi52947979
X -- -- -- -- -- -- -- --
-- -- -- -- -- -- -- --
-- -- -- -- -- -- -- --
-- -- -- -- -- -- -- --
-- -- -- -- 6452650
792 cnvi52947983
X -- -- -- -- -- -- -- --
-- -- -- -- -- -- -- --
-- -- -- -- -- -- -- --
-- -- -- -- -- -- -- --
-- -- -- -- 6452850
793 cnvi52947987
X -- -- -- -- -- -- -- --
-- -- -- -- -- -- -- --
-- -- -- -- -- -- -- --
-- -- -- -- -- -- -- --
-- -- -- -- 6453050
794 cnvi52974161
X -- -- -- -- -- -- -- --
-- -- -- -- -- -- -- --
-- -- -- -- -- -- -- --
-- -- -- -- -- -- -- --
-- -- -- -- 7811750
795 cnvi52974163
X -- -- -- -- -- -- -- --
-- -- -- -- -- -- -- --
-- -- -- -- -- -- -- --
-- -- -- -- -- -- -- --
-- -- -- -- 7811850
796 cnvi52974167
X -- -- -- -- -- -- -- --
-- -- -- -- -- -- -- --
-- -- -- -- -- -- -- --
-- -- -- -- -- -- -- --
-- -- -- -- 7812050
797 cnvi53039624
X -- -- -- -- -- -- -- --
-- -- -- -- -- -- -- --
-- -- -- -- -- -- -- --
-- -- -- -- -- -- -- --
-- -- -- -- 11134979
798 cnvi53788829
X -- -- -- -- -- -- -- --
-- -- -- -- -- -- -- --
-- -- -- -- -- -- -- --
-- -- -- -- -- -- -- --
-- -- -- -- 48645256
799 cnvi53788839
X -- -- -- -- -- -- -- --

	--	--	--	--	--	--	--	--
	--	--	--	--	--	--	--	--
	--	--	--	--	--	--	--	--
	--	--	--	--	48645756			
800	cnvi53788868							
X	--	--	--	--	--	--	--	--
	--	--	--	--	--	--	--	--
	--	--	--	--	--	--	--	--
	--	--	--	--	--	--	--	--
	--	--	--	--	48647206			
801	cnvi53788888							
X	--	--	--	--	--	--	--	--
	--	--	--	--	--	--	--	--
	--	--	--	--	--	--	--	--
	--	--	--	--	--	--	--	--
	--	--	--	--	48648206			
802	cnvi53788894							
X	--	--	--	--	--	--	--	--
	--	--	--	--	--	--	--	--
	--	--	--	--	--	--	--	--
	--	--	--	--	--	--	--	--
	--	--	--	--	48648506			
803	cnvi53788921							
X	--	--	--	--	--	--	--	--
	--	--	--	--	--	--	--	--
	--	--	--	--	--	--	--	--
	--	--	--	--	--	--	--	--
	--	--	--	--	48649856			
804	cnvi53788927							
X	--	--	--	--	--	--	--	--
	--	--	--	--	--	--	--	--
	--	--	--	--	--	--	--	--
	--	--	--	--	--	--	--	--
	--	--	--	--	48650156			
805	cnvi53788939							
X	--	--	--	--	--	--	--	--
	--	--	--	--	--	--	--	--
	--	--	--	--	--	--	--	--
	--	--	--	--	--	--	--	--
	--	--	--	--	48650756			
806	cnvi53788963							
X	--	--	--	--	--	--	--	--
	--	--	--	--	--	--	--	--
	--	--	--	--	--	--	--	--
	--	--	--	--	--	--	--	--
	--	--	--	--	48651956			
807	cnvi53788973							
X	--	--	--	--	--	--	--	--
	--	--	--	--	--	--	--	--
	--	--	--	--	--	--	--	--

	--	--	--	--	--	--	--	--
	--	--	--	--	48652456			
808	cnvi53843695							
X	--	--	--	--	--	--	--	--
	--	--	--	--	--	--	--	--
	--	--	--	--	--	--	--	--
	--	--	--	--	--	--	--	--
	--	--	--	--	51486860			
809	cnvi53843703							
X	--	--	--	--	--	--	--	--
	--	--	--	--	--	--	--	--
	--	--	--	--	--	--	--	--
	--	--	--	--	--	--	--	--
	--	--	--	--	51487260			
810	cnvi53843707							
X	--	--	--	--	--	--	--	--
	--	--	--	--	--	--	--	--
	--	--	--	--	--	--	--	--
	--	--	--	--	--	--	--	--
	--	--	--	--	51487460			
811	cnvi53843715							
X	--	--	--	--	--	--	--	--
	--	--	--	--	--	--	--	--
	--	--	--	--	--	--	--	--
	--	--	--	--	--	--	--	--
	--	--	--	--	51487860			
812	cnvi53843723							
X	--	--	--	--	--	--	--	--
	--	--	--	--	--	--	--	--
	--	--	--	--	--	--	--	--
	--	--	--	--	--	--	--	--
	--	--	--	--	51488260			
813	cnvi53843730							
X	--	--	--	--	--	--	--	--
	--	--	--	--	--	--	--	--
	--	--	--	--	--	--	--	--
	--	--	--	--	--	--	--	--
	--	--	--	--	51488610			
814	cnvi5386834							
8	--	--	--	--	--	--	--	--
	--	--	--	--	--	--	--	--
	--	--	--	--	--	--	--	--
	--	--	--	--	--	--	--	--
	--	--	--	--	55371797			
815	cnvi5386839							
8	--	--	--	--	--	--	--	--
	--	--	--	--	--	--	--	--
	--	--	--	--	--	--	--	--
	--	--	--	--	--	--	--	--
	--	--	--	--	55372047			

816 cnvi5386844
8 -- -- -- -- -- -- -- --
-- -- -- -- -- -- -- --
-- -- -- -- -- -- -- --
-- -- -- -- -- -- -- --
-- -- -- -- 55372297
817 cnvi5386849
8 -- -- -- -- -- -- -- --
-- -- -- -- -- -- -- --
-- -- -- -- -- -- -- --
-- -- -- -- -- -- -- --
-- -- -- -- 55372547
818 cnvi5386859
8 -- -- -- -- -- -- -- --
-- -- -- -- -- -- -- --
-- -- -- -- -- -- -- --
-- -- -- -- -- -- -- --
-- -- -- -- 55373047
819 cnvi54285248
X -- -- -- -- -- -- -- --
-- -- -- -- -- -- -- --
-- -- -- -- -- -- -- --
-- -- -- -- -- -- -- --
-- -- -- -- 76764744
820 cnvi54285371
X -- -- -- -- -- -- -- --
-- -- -- -- -- -- -- --
-- -- -- -- -- -- -- --
-- -- -- -- -- -- -- --
-- -- -- -- 76770894
821 cnvi54285538
X -- -- -- -- -- -- -- --
-- -- -- -- -- -- -- --
-- -- -- -- -- -- -- --
-- -- -- -- -- -- -- --
-- -- -- -- 76779244
822 cnvi54285839
X -- -- -- -- -- -- -- --
-- -- -- -- -- -- -- --
-- -- -- -- -- -- -- --
-- -- -- -- -- -- -- --
-- -- -- -- 76794294
823 cnvi54286040
X -- -- -- -- -- -- -- --
-- -- -- -- -- -- -- --
-- -- -- -- -- -- -- --
-- -- -- -- -- -- -- --
-- -- -- -- 76804344
824 cnvi54286381
X -- -- -- -- -- -- -- --

	--	--	--	--	--	--	--	--
	--	--	--	--	--	--	--	--
	--	--	--	--	--	--	--	--
	--	--	--	--	76821394			
825	cnvi54286547							
X	--	--	--	--	--	--	--	--
	--	--	--	--	--	--	--	--
	--	--	--	--	--	--	--	--
	--	--	--	--	--	--	--	--
	--	--	--	--	76829694			
826	cnvi54286725							
X	--	--	--	--	--	--	--	--
	--	--	--	--	--	--	--	--
	--	--	--	--	--	--	--	--
	--	--	--	--	--	--	--	--
	--	--	--	--	76838594			
827	cnvi54287050							
X	--	--	--	--	--	--	--	--
	--	--	--	--	--	--	--	--
	--	--	--	--	--	--	--	--
	--	--	--	--	--	--	--	--
	--	--	--	--	76854844			
828	cnvi54287240							
X	--	--	--	--	--	--	--	--
	--	--	--	--	--	--	--	--
	--	--	--	--	--	--	--	--
	--	--	--	--	--	--	--	--
	--	--	--	--	76864344			
829	cnvi54287425							
X	--	--	--	--	--	--	--	--
	--	--	--	--	--	--	--	--
	--	--	--	--	--	--	--	--
	--	--	--	--	--	--	--	--
	--	--	--	--	76873594			
830	cnvi54287679							
X	--	--	--	--	--	--	--	--
	--	--	--	--	--	--	--	--
	--	--	--	--	--	--	--	--
	--	--	--	--	--	--	--	--
	--	--	--	--	76886294			
831	cnvi54287846							
X	--	--	--	--	--	--	--	--
	--	--	--	--	--	--	--	--
	--	--	--	--	--	--	--	--
	--	--	--	--	--	--	--	--
	--	--	--	--	76894644			
832	cnvi54288032							
X	--	--	--	--	--	--	--	--
	--	--	--	--	--	--	--	--
	--	--	--	--	--	--	--	--

	--	--	--	--	--	--	--	--
	--	--	--	--	76903944			
833	cnvi54288125							
X	--	--	--	--	--	--	--	--
	--	--	--	--	--	--	--	--
	--	--	--	--	--	--	--	--
	--	--	--	--	--	--	--	--
	--	--	--	--	76908594			
834	cnvi54288293							
X	--	--	--	--	--	--	--	--
	--	--	--	--	--	--	--	--
	--	--	--	--	--	--	--	--
	--	--	--	--	--	--	--	--
	--	--	--	--	76916994			
835	cnvi54288467							
X	--	--	--	--	--	--	--	--
	--	--	--	--	--	--	--	--
	--	--	--	--	--	--	--	--
	--	--	--	--	--	--	--	--
	--	--	--	--	76925694			
836	cnvi54288566							
X	--	--	--	--	--	--	--	--
	--	--	--	--	--	--	--	--
	--	--	--	--	--	--	--	--
	--	--	--	--	--	--	--	--
	--	--	--	--	76930644			
837	cnvi54288740							
X	--	--	--	--	--	--	--	--
	--	--	--	--	--	--	--	--
	--	--	--	--	--	--	--	--
	--	--	--	--	--	--	--	--
	--	--	--	--	76939344			
838	cnvi54289073							
X	--	--	--	--	--	--	--	--
	--	--	--	--	--	--	--	--
	--	--	--	--	--	--	--	--
	--	--	--	--	--	--	--	--
	--	--	--	--	76955994			
839	cnvi54289253							
X	--	--	--	--	--	--	--	--
	--	--	--	--	--	--	--	--
	--	--	--	--	--	--	--	--
	--	--	--	--	--	--	--	--
	--	--	--	--	76964994			
840	cnvi54289446							
X	--	--	--	--	--	--	--	--
	--	--	--	--	--	--	--	--
	--	--	--	--	--	--	--	--
	--	--	--	--	--	--	--	--
	--	--	--	--	76974644			

841 cnvi54289620
X -- -- -- -- -- -- -- --
-- -- -- -- -- -- -- --
-- -- -- -- -- -- -- --
-- -- -- -- -- -- -- --
-- -- -- -- 76983344
842 cnvi54289720
X -- -- -- -- -- -- -- --
-- -- -- -- -- -- -- --
-- -- -- -- -- -- -- --
-- -- -- -- -- -- -- --
-- -- -- -- 76988344
843 cnvi54289900
X -- -- -- -- -- -- -- --
-- -- -- -- -- -- -- --
-- -- -- -- -- -- -- --
-- -- -- -- -- -- -- --
-- -- -- -- 76997344
844 cnvi54290069
X -- -- -- -- -- -- -- --
-- -- -- -- -- -- -- --
-- -- -- -- -- -- -- --
-- -- -- -- -- -- -- --
-- -- -- -- 77005794
845 cnvi54290192
X -- -- -- -- -- -- -- --
-- -- -- -- -- -- -- --
-- -- -- -- -- -- -- --
-- -- -- -- -- -- -- --
-- -- -- -- 77011944
846 cnvi54290368
X -- -- -- -- -- -- -- --
-- -- -- -- -- -- -- --
-- -- -- -- -- -- -- --
-- -- -- -- -- -- -- --
-- -- -- -- 77020744
847 cnvi54290538
X -- -- -- -- -- -- -- --
-- -- -- -- -- -- -- --
-- -- -- -- -- -- -- --
-- -- -- -- -- -- -- --
-- -- -- -- 77029244
848 cnvi54405221
X -- -- -- -- -- -- -- --
-- -- -- -- -- -- -- --
-- -- -- -- -- -- -- --
-- -- -- -- -- -- -- --
-- -- -- -- 82763394
849 cnvi54405231
X -- -- -- -- -- -- -- --

	--	--	--	--	--	--	--	--
	--	--	--	--	--	--	--	--
	--	--	--	--	--	--	--	--
	--	--	--	--	82763894			
850	cnvi54405238							
X	--	--	--	--	--	--	--	--
	--	--	--	--	--	--	--	--
	--	--	--	--	--	--	--	--
	--	--	--	--	--	--	--	--
	--	--	--	--	82764244			
851	cnvi54405244							
X	--	--	--	--	--	--	--	--
	--	--	--	--	--	--	--	--
	--	--	--	--	--	--	--	--
	--	--	--	--	--	--	--	--
	--	--	--	--	82764544			
852	cnvi54533503							
X	--	--	--	--	--	--	--	--
	--	--	--	--	--	--	--	--
	--	--	--	--	--	--	--	--
	--	--	--	--	--	--	--	--
	--	--	--	--	89177494			
853	cnvi54563766							
X	--	--	--	--	--	--	--	--
	--	--	--	--	--	--	--	--
	--	--	--	--	--	--	--	--
	--	--	--	--	--	--	--	--
	--	--	--	--	90690644			
854	cnvi54563772							
X	--	--	--	--	--	--	--	--
	--	--	--	--	--	--	--	--
	--	--	--	--	--	--	--	--
	--	--	--	--	--	--	--	--
	--	--	--	--	90690944			
855	cnvi54563778							
X	--	--	--	--	--	--	--	--
	--	--	--	--	--	--	--	--
	--	--	--	--	--	--	--	--
	--	--	--	--	--	--	--	--
	--	--	--	--	90691244			
856	cnvi54563788							
X	--	--	--	--	--	--	--	--
	--	--	--	--	--	--	--	--
	--	--	--	--	--	--	--	--
	--	--	--	--	--	--	--	--
	--	--	--	--	90691744			
857	cnvi54563800							
X	--	--	--	--	--	--	--	--
	--	--	--	--	--	--	--	--
	--	--	--	--	--	--	--	--

-- -- -- -- -- -- -- --
-- -- -- -- 90692344
858 cnvi54563806
X -- -- -- -- -- -- -- --
-- -- -- -- -- -- -- --
-- -- -- -- -- -- -- --
-- -- -- -- -- -- -- --
-- -- -- -- 90692644
859 cnvi54779550
X -- -- -- -- -- -- -- --
-- -- -- -- -- -- -- --
-- -- -- -- -- -- -- --
-- -- -- -- -- -- -- --
-- -- -- -- 101479844
860 cnvi54779647
X -- -- -- -- -- -- -- --
-- -- -- -- -- -- -- --
-- -- -- -- -- -- -- --
-- -- -- -- -- -- -- --
-- -- -- -- 101484694
861 cnvi54779817
X -- -- -- -- -- -- -- --
-- -- -- -- -- -- -- --
-- -- -- -- -- -- -- --
-- -- -- -- -- -- -- --
-- -- -- -- 101493194
862 cnvi54779923
X -- -- -- -- -- -- -- --
-- -- -- -- -- -- -- --
-- -- -- -- -- -- -- --
-- -- -- -- -- -- -- --
-- -- -- -- 101498494
863 cnvi54780442
X -- -- -- -- -- -- -- --
-- -- -- -- -- -- -- --
-- -- -- -- -- -- -- --
-- -- -- -- -- -- -- --
-- -- -- -- 101524444
864 cnvi54780542
X -- -- -- -- -- -- -- --
-- -- -- -- -- -- -- --
-- -- -- -- -- -- -- --
-- -- -- -- -- -- -- --
-- -- -- -- 101529444
865 cnvi54780883
X -- -- -- -- -- -- -- --
-- -- -- -- -- -- -- --
-- -- -- -- -- -- -- --
-- -- -- -- -- -- -- --
-- -- -- -- 101546494

866 cnvi54781086
X -- -- -- -- -- -- -- --
-- -- -- -- -- -- -- --
-- -- -- -- -- -- -- --
-- -- -- -- -- -- -- --
-- -- -- -- 101556644
867 cnvi54781249
X -- -- -- -- -- -- -- --
-- -- -- -- -- -- -- --
-- -- -- -- -- -- -- --
-- -- -- -- -- -- -- --
-- -- -- -- 101564794
868 cnvi54781334
X -- -- -- -- -- -- -- --
-- -- -- -- -- -- -- --
-- -- -- -- -- -- -- --
-- -- -- -- -- -- -- --
-- -- -- -- 101569044
869 cnvi54781492
X -- -- -- -- -- -- -- --
-- -- -- -- -- -- -- --
-- -- -- -- -- -- -- --
-- -- -- -- -- -- -- --
-- -- -- -- 101576944
870 cnvi55150611
X -- -- -- -- -- -- -- --
-- -- -- -- -- -- -- --
-- -- -- -- -- -- -- --
-- -- -- -- -- -- -- --
-- -- -- -- 120181969
871 cnvi55150615
X -- -- -- -- -- -- -- --
-- -- -- -- -- -- -- --
-- -- -- -- -- -- -- --
-- -- -- -- -- -- -- --
-- -- -- -- 120182169
872 cnvi55150620
X -- -- -- -- -- -- -- --
-- -- -- -- -- -- -- --
-- -- -- -- -- -- -- --
-- -- -- -- -- -- -- --
-- -- -- -- 120182419
873 cnvi55150643
X -- -- -- -- -- -- -- --
-- -- -- -- -- -- -- --
-- -- -- -- -- -- -- --
-- -- -- -- -- -- -- --
-- -- -- -- 120183569
874 cnvi55479948
X -- -- -- -- -- -- -- --

	--	--	--	--	--	--	--	--
	--	--	--	--	--	--	--	--
	--	--	--	--	--	--	--	--
	--	--	--	--	136648834			
875	cnvi55480006							
X	--	--	--	--	--	--	--	--
	--	--	--	--	--	--	--	--
	--	--	--	--	--	--	--	--
	--	--	--	--	--	--	--	--
	--	--	--	--	136651734			
876	cnvi55480030							
X	--	--	--	--	--	--	--	--
	--	--	--	--	--	--	--	--
	--	--	--	--	--	--	--	--
	--	--	--	--	--	--	--	--
	--	--	--	--	136652934			
877	cnvi55538683							
X	--	--	--	--	--	--	--	--
	--	--	--	--	--	--	--	--
	--	--	--	--	--	--	--	--
	--	--	--	--	--	--	--	--
	--	--	--	--	139585584			
878	cnvi55538689							
X	--	--	--	--	--	--	--	--
	--	--	--	--	--	--	--	--
	--	--	--	--	--	--	--	--
	--	--	--	--	--	--	--	--
	--	--	--	--	139585884			
879	cnvi55538693							
X	--	--	--	--	--	--	--	--
	--	--	--	--	--	--	--	--
	--	--	--	--	--	--	--	--
	--	--	--	--	--	--	--	--
	--	--	--	--	139586084			
880	cnvi55538700							
X	--	--	--	--	--	--	--	--
	--	--	--	--	--	--	--	--
	--	--	--	--	--	--	--	--
	--	--	--	--	--	--	--	--
	--	--	--	--	139586434			
881	cnvi55538711							
X	--	--	--	--	--	--	--	--
	--	--	--	--	--	--	--	--
	--	--	--	--	--	--	--	--
	--	--	--	--	--	--	--	--
	--	--	--	--	139586984			
882	cnvi55811072							
X	--	--	--	--	--	--	--	--
	--	--	--	--	--	--	--	--
	--	--	--	--	--	--	--	--

-- -- -- -- -- -- -- --
-- -- -- -- 153449606
883 cnvi55811096
X -- -- -- -- -- -- -- --
-- -- -- -- -- -- -- --
-- -- -- -- -- -- -- --
-- -- -- -- -- -- -- --
-- -- -- -- 153450806
884 cnvi55811136
X -- -- -- -- -- -- -- --
-- -- -- -- -- -- -- --
-- -- -- -- -- -- -- --
-- -- -- -- -- -- -- --
-- -- -- -- 153452806
885 cnvi55811171
X -- -- -- -- -- -- -- --
-- -- -- -- -- -- -- --
-- -- -- -- -- -- -- --
-- -- -- -- -- -- -- --
-- -- -- -- 153454556
886 cnvi55811205
X -- -- -- -- -- -- -- --
-- -- -- -- -- -- -- --
-- -- -- -- -- -- -- --
-- -- -- -- -- -- -- --
-- -- -- -- 153456256
887 cnvi55811246
X -- -- -- -- -- -- -- --
-- -- -- -- -- -- -- --
-- -- -- -- -- -- -- --
-- -- -- -- -- -- -- --
-- -- -- -- 153458306
888 cnvi55811288
X -- -- -- -- -- -- -- --
-- -- -- -- -- -- -- --
-- -- -- -- -- -- -- --
-- -- -- -- -- -- -- --
-- -- -- -- 153460406
889 cnvi55904322
15 -- -- -- -- -- -- -- --
-- -- -- -- -- -- -- --
-- -- -- -- -- -- -- --
-- -- -- -- -- -- -- --
-- -- -- -- 23810607
890 cnvi55904326
15 -- -- -- -- -- -- -- --
-- -- -- -- -- -- -- --
-- -- -- -- -- -- -- --
-- -- -- -- -- -- -- --
-- -- -- -- 23810807

891 cnvi55904328
15 -- -- -- -- -- -- -- --
-- -- -- -- -- -- -- --
-- -- -- -- -- -- -- --
-- -- -- -- -- -- -- --
-- -- -- -- 23810907
892 cnvi55904339
15 -- -- -- -- -- -- -- --
-- -- -- -- -- -- -- --
-- -- -- -- -- -- -- --
-- -- -- -- -- -- -- --
-- -- -- -- 23811457
893 cnvi55904344
15 -- -- -- -- -- -- -- --
-- -- -- -- -- -- -- --
-- -- -- -- -- -- -- --
-- -- -- -- -- -- -- --
-- -- -- -- 23811707
894 cnvi55904348
15 -- -- -- -- -- -- -- --
-- -- -- -- -- -- -- --
-- -- -- -- -- -- -- --
-- -- -- -- -- -- -- --
-- -- -- -- 23811907
895 cnvi55904350
15 -- -- -- -- -- -- -- --
-- -- -- -- -- -- -- --
-- -- -- -- -- -- -- --
-- -- -- -- -- -- -- --
-- -- -- -- 23812007
896 cnvi55904355
15 -- -- -- -- -- -- -- --
-- -- -- -- -- -- -- --
-- -- -- -- -- -- -- --
-- -- -- -- -- -- -- --
-- -- -- -- 23812257
897 cnvi55904359
15 -- -- -- -- -- -- -- --
-- -- -- -- -- -- -- --
-- -- -- -- -- -- -- --
-- -- -- -- -- -- -- --
-- -- -- -- 23812457
898 cnvi55904363
15 -- -- -- -- -- -- -- --
-- -- -- -- -- -- -- --
-- -- -- -- -- -- -- --
-- -- -- -- -- -- -- --
-- -- -- -- 23812657
899 cnvi55904367
15 -- -- -- -- -- -- -- --

	--	--	--	--	--	--	--	--
	--	--	--	--	--	--	--	--
	--	--	--	--	--	--	--	--
	--	--	--	--	23812857			
900	cnvi55904375							
15	--	--	--	--	--	--	--	--
	--	--	--	--	--	--	--	--
	--	--	--	--	--	--	--	--
	--	--	--	--	--	--	--	--
	--	--	--	--	23813257			
901	cnvi55904379							
15	--	--	--	--	--	--	--	--
	--	--	--	--	--	--	--	--
	--	--	--	--	--	--	--	--
	--	--	--	--	--	--	--	--
	--	--	--	--	23813457			
902	cnvi55906723							
15	--	--	--	--	--	--	--	--
	--	--	--	--	--	--	--	--
	--	--	--	--	--	--	--	--
	--	--	--	--	--	--	--	--
	--	--	--	--	23930657			
903	cnvi55906725							
15	--	--	--	--	--	--	--	--
	--	--	--	--	--	--	--	--
	--	--	--	--	--	--	--	--
	--	--	--	--	--	--	--	--
	--	--	--	--	23930757			
904	cnvi55906727							
15	--	--	--	--	--	--	--	--
	--	--	--	--	--	--	--	--
	--	--	--	--	--	--	--	--
	--	--	--	--	--	--	--	--
	--	--	--	--	23930857			
905	cnvi55906729							
15	--	--	--	--	--	--	--	--
	--	--	--	--	--	--	--	--
	--	--	--	--	--	--	--	--
	--	--	--	--	--	--	--	--
	--	--	--	--	23930957			
906	cnvi55906731							
15	--	--	--	--	--	--	--	--
	--	--	--	--	--	--	--	--
	--	--	--	--	--	--	--	--
	--	--	--	--	--	--	--	--
	--	--	--	--	23931057			
907	cnvi55906734							
15	--	--	--	--	--	--	--	--
	--	--	--	--	--	--	--	--
	--	--	--	--	--	--	--	--

-- -- -- -- -- -- -- --
-- -- -- -- 23931207
908 cnvi55906735
15 -- -- -- -- -- -- -- --
-- -- -- -- -- -- -- --
-- -- -- -- -- -- -- --
-- -- -- -- -- -- -- --
-- -- -- -- 23931257
909 cnvi55906742
15 -- -- -- -- -- -- -- --
-- -- -- -- -- -- -- --
-- -- -- -- -- -- -- --
-- -- -- -- -- -- -- --
-- -- -- -- 23931607
910 cnvi55906744
15 -- -- -- -- -- -- -- --
-- -- -- -- -- -- -- --
-- -- -- -- -- -- -- --
-- -- -- -- -- -- -- --
-- -- -- -- 23931707
911 cnvi55906746
15 -- -- -- -- -- -- -- --
-- -- -- -- -- -- -- --
-- -- -- -- -- -- -- --
-- -- -- -- -- -- -- --
-- -- -- -- 23931807
912 cnvi55906747
15 -- -- -- -- -- -- -- --
-- -- -- -- -- -- -- --
-- -- -- -- -- -- -- --
-- -- -- -- -- -- -- --
-- -- -- -- 23931857
913 cnvi55906749
15 -- -- -- -- -- -- -- --
-- -- -- -- -- -- -- --
-- -- -- -- -- -- -- --
-- -- -- -- -- -- -- --
-- -- -- -- 23931957
914 cnvi55938580
15 -- -- -- -- -- -- -- --
-- -- -- -- -- -- -- --
-- -- -- -- -- -- -- --
-- -- -- -- -- -- -- --
-- -- -- -- 25523507
915 cnvi56631861
15 -- -- -- -- -- -- -- --
-- -- -- -- -- -- -- --
-- -- -- -- -- -- -- --
-- -- -- -- -- -- -- --
-- -- -- -- 60296558

916	cnvi56631865							
15	--	--	--	--	--	--	--	--
	--	--	--	--	--	--	--	--
	--	--	--	--	--	--	--	--
	--	--	--	--	--	--	--	--
	--	--	--	--	60296758			
917	cnvi56631870							
15	--	--	--	--	--	--	--	--
	--	--	--	--	--	--	--	--
	--	--	--	--	--	--	--	--
	--	--	--	--	--	--	--	--
	--	--	--	--	60297008			
918	cnvi56631875							
15	--	--	--	--	--	--	--	--
	--	--	--	--	--	--	--	--
	--	--	--	--	--	--	--	--
	--	--	--	--	--	--	--	--
	--	--	--	--	60297258			
919	cnvi56631881							
15	--	--	--	--	--	--	--	--
	--	--	--	--	--	--	--	--
	--	--	--	--	--	--	--	--
	--	--	--	--	--	--	--	--
	--	--	--	--	60297558			
920	cnvi56631884							
15	--	--	--	--	--	--	--	--
	--	--	--	--	--	--	--	--
	--	--	--	--	--	--	--	--
	--	--	--	--	--	--	--	--
	--	--	--	--	60297708			
921	cnvi56631887							
15	--	--	--	--	--	--	--	--
	--	--	--	--	--	--	--	--
	--	--	--	--	--	--	--	--
	--	--	--	--	--	--	--	--
	--	--	--	--	60297858			
922	cnvi7371135							
19	--	--	--	--	--	--	--	--
	--	--	--	--	--	--	--	--
	--	--	--	--	--	--	--	--
	--	--	--	--	--	--	--	--
	--	--	--	--	8953400			
923	cnvi7371137							
19	--	--	--	--	--	--	--	--
	--	--	--	--	--	--	--	--
	--	--	--	--	--	--	--	--
	--	--	--	--	--	--	--	--
	--	--	--	--	8953500			
924	cnvi7371139							
19	--	--	--	--	--	--	--	--

	--	--	--	--	--	--	--	--
	--	--	--	--	--	--	--	--
	--	--	--	--	--	--	--	--
	--	--	--	--	8953600			
925	cnvi7371141							
19	--	--	--	--	--	--	--	--
	--	--	--	--	--	--	--	--
	--	--	--	--	--	--	--	--
	--	--	--	--	--	--	--	--
	--	--	--	--	8953700			
926	cnvi7371144							
19	--	--	--	--	--	--	--	--
	--	--	--	--	--	--	--	--
	--	--	--	--	--	--	--	--
	--	--	--	--	--	--	--	--
	--	--	--	--	8953850			
927	cnvi8723075							
7	--	--	--	--	--	--	--	--
	--	--	--	--	--	--	--	--
	--	--	--	--	--	--	--	--
	--	--	--	--	--	--	--	--
	--	--	--	--	20822325			
928	cnvi8723091							
7	--	--	--	--	--	--	--	--
	--	--	--	--	--	--	--	--
	--	--	--	--	--	--	--	--
	--	--	--	--	--	--	--	--
	--	--	--	--	20823125			
929	cnvi8723132							
7	--	--	--	--	--	--	--	--
	--	--	--	--	--	--	--	--
	--	--	--	--	--	--	--	--
	--	--	--	--	--	--	--	--
	--	--	--	--	20825175			
930	cnvi8723140							
7	--	--	--	--	--	--	--	--
	--	--	--	--	--	--	--	--
	--	--	--	--	--	--	--	--
	--	--	--	--	--	--	--	--
	--	--	--	--	20825575			
931	cnvi8723149							
7	--	--	--	--	--	--	--	--
	--	--	--	--	--	--	--	--
	--	--	--	--	--	--	--	--
	--	--	--	--	--	--	--	--
	--	--	--	--	20826025			
932	cnvi8934219							
7	--	--	--	--	--	--	--	--
	--	--	--	--	--	--	--	--
	--	--	--	--	--	--	--	--

-- -- -- -- -- -- -- --
-- -- -- -- 31379525
933 cnvi8934234
7 -- -- -- -- -- -- -- --
-- -- -- -- -- -- -- --
-- -- -- -- -- -- -- --
-- -- -- -- -- -- -- --
-- -- -- -- 31380275
934 cnvi9694497
7 -- -- -- -- -- -- -- --
-- -- -- -- -- -- -- --
-- -- -- -- -- -- -- --
-- -- -- -- -- -- -- --
-- -- -- -- 72952314
935 cnvi9694565
7 -- -- -- -- -- -- -- --
-- -- -- -- -- -- -- --
-- -- -- -- -- -- -- --
-- -- -- -- -- -- -- --
-- -- -- -- 72955714
936 cnvi9695162
7 -- -- -- -- -- -- -- --
-- -- -- -- -- -- -- --
-- -- -- -- -- -- -- --
-- -- -- -- -- -- -- --
-- -- -- -- 72985564
937 cnvi9695180
7 -- -- -- -- -- -- -- --
-- -- -- -- -- -- -- --
-- -- -- -- -- -- -- --
-- -- -- -- -- -- -- --
-- -- -- -- 72986464
938 cnvi9695190
7 -- -- -- -- -- -- -- --
-- -- -- -- -- -- -- --
-- -- -- -- -- -- -- --
-- -- -- -- -- -- -- --
-- -- -- -- 72986964
939 cnvi9695220
7 -- -- -- -- -- -- -- --
-- -- -- -- -- -- -- --
-- -- -- -- -- -- -- --
-- -- -- -- -- -- -- --
-- -- -- -- 72988464
940 cnvi9695228
7 -- -- -- -- -- -- -- --
-- -- -- -- -- -- -- --
-- -- -- -- -- -- -- --
-- -- -- -- -- -- -- --
-- -- -- -- 72988864

941 cnvi9695289
7 -- -- -- -- -- -- -- --
-- -- -- -- -- -- -- --
-- -- -- -- -- -- -- --
-- -- -- -- -- -- -- --
-- -- -- -- 72991914
942 rs1000002 3 GG GG AG AA AA
AG AA AA GG AG GG GG AG
AG GG AG AG AG AG GG AG
AA AA AG AA AG AG AG AG
AG AG GG GG AA AG AA
183635768
943 rs1000003 3 AA AA AA AA AA
AA AA AA AA AA AA AA AA
AA AA AA AA AA AA AA AA
AA AA AA AA AA AG AG AG
AA AA AA AA AA AA AA
98342907
944 rs10000030 4 GG AG GG GG GG
GG GG GG GG GG GG GG GG
GG GG GG GG GG GG GG GG
AG GG GG GG GG GG GG GG
GG AG GG GG AG GG AG
103374154
945 rs10000037 4 AG GG GG GG GG
GG GG AG AG AG GG GG AG
AG AG GG GG GG GG GG GG
AG AG AG GG GG AG AG AG
AG GG AA AG GG GG GG
38924330
946 rs10000041 4 AC AC AA AC AC
AC AA AC AC AC AA AA AA
AA AA AC AC AC AC AC AC
AC AA AA AA AA AA AA AA
CC AC AC AC AC AA AC
165621955
947 rs1000007 2 AA AA AA AA AA
AA AA AA AA AA AA AG AA
AA AA AA AA AA AG AG AG
AG AG AG AG AA AA AA AA
AA AG AA AA AG AG AA
237752054
948 rs1000016 2 AA AA AA AA AA
AA AA AG AA AG AA AA AA
AA AA AG AA AA AA AA AA
AG AG AG AA AA AA AA AA
GG AG AG AA AA AA AA
235690982
949 rs10000180 4 GG GG GG GG GG
GG GG GG GG AG AG AA AG

AG AG AG GG AG GG GG GG
AG AG AG AG GG AG AG GG
GG GG GG GG GG AG GG
83899764
950 rs10000272 4 AA AA AA AA AA
AA AA AG AG AG AA AA AA
AA AA GG AG AG AG AG AG
AA AA AA AA AA AA AA AA
AG AA AG AA AA AA AA
189690383
951 rs1000031 18 AG GG GG AG AG
GG AA AG AG AA AG AG GG
GG AG AG AG AA AG AG AG
AG AG AG AG AA GG AG AG
AG AG AG AG AG AG AG
46361441
952 rs10000432 4 GG GG AG GG GG
GG GG AA AA AA AG AG AG
AG GG AG AG AA GG GG GG
GG AG AG AG AA GG AG AG
AG AG AG GG GG AG GG
47511781
953 rs10000438 4 AG AA AA GG AG
AG GG AG AG AG AG AA AG
AG AG AG AG GG AG AA AG
AG AG AG AG AA AA AA AA
AG AG AG AG AA AA AG
61773762
954 rs1000047 16 AG AG AG AA AG
AG AA GG AG GG GG AG AG
AG GG GG GG GG GG GG GG
AA GG AG AA GG GG GG GG
AG GG AG AG AG AG GG
8138689
955 rs10000471 4 AG AA AG AA AA
AA AA AG AA AA AA AA AA
AA AG AG AG GG AA AA AG
AG GG GG AG AG AG AG AG
AA AG AA AG AG AA AG
80943688
956 rs1000068 17 AA AA AA AG AG
GG AG AA GG AG GG GG GG
GG AG AG AA AG AA AA AA
GG GG GG GG AG GG AG AG
AG GG AA AG GG GG GG
48298966
957 rs10000708 4 CC CC CC AC AC
AA AC AC AC AA CC CC CC
AC AA AA AC AC CC CC CC
CC CC CC CC CC AC AC AC

	CC	AC	AC	AA	AC	AC	CC	
42475352								
958	rs10000852		4	AC	AC	AC	AA	AA
	AA	AA	AA	AA	AA	AA	AA	AA
	AA	AA	AA	AA	AA	AA	AA	AA
	AA	AA	AA	AA	AA	AA	AA	AA
	AA	AA	AA	AA	AA	AA	AA	
137429284								
959	rs1000087		2	GG	AG	AA	AG	GG
	AG	GG	GG	GG	GG	AG	AA	AA
	AG	AG	AG	AA	AA	AG	AG	AA
	AG	AG	AG	AG	GG	AG	AG	GG
	AA	AG	GG	GG	AG	GG	AA	
66350062								
960	rs1000094		6	AA	AA	AA	GG	GG
	GG	GG	AG	AG	GG	GG	AG	AG
	AA	AG	AG	AG	AG	AG	AG	GG
	AG	AG	AG	AG	AA	AA	AA	AA
	AA	AA	AA	AA	AG	AA	AG	
51990683								
961	rs10000971		4	AA	AC	AC	AC	AC
	AA	CC	AA	AC	AC	AA	AA	AC
	AA	AC	AA	AA	AA	AA	AA	AA
	AA	AC	AC	AC	AA	AA	AA	AA
	AC	AA	AA	AA	AA	AA	AA	
60906671								
962	rs10000976		4	GG	GG	AG	GG	GG
	GG	AG	GG	GG	AG	GG	GG	GG
	GG	GG	GG	GG	GG	AG	AG	GG
	AG	AG	AG	GG	GG	AG	GG	AG
	AG	AG	GG	AG	AG	AG	GG	
84978944								
963	rs10000984		4	AA	AA	AA	AA	AA
	AA	AA	AA	AA	AG	AA	AA	AA
	AG	AG	AA	AA	AA	GG	GG	AG
	GG	AG	AG	AG	AG	AG	AG	GG
	AA	AA	AG	AG	AG	AG	AA	
175644564								
964	rs10000988		4	GG	AG	AG	GG	GG
	GG	GG	GG	GG	GG	GG	GG	GG
	AG	AG	GG	AG	AG	GG	GG	GG
	GG	GG	GG	GG	GG	GG	GG	GG
	GG	GG	GG	GG	GG	GG	GG	
76010255								
965	rs10001030		4	AA	AA	AA	AG	AG
	AA	AG	AA	AA	AA	AG	AA	AA
	AA	AA	AG	AG	AG	AA	AA	AA
	AA	AG	AG	AA	AG	AA	AA	AA
	AA	AG	AA	AA	AG	AA	AG	
126535503								

966 rs1000104 6 AC AA AC CC CC
CC CC CC CC CC CC CC CC
CC CC CC CC CC CC CC CC
AC AC CC CC AC AC AA CC
CC AC AC AA AA AC AA
170353312
967 rs10001131 4 GG GG GG GG GG
GG GG GG GG GG GG GG GG
GG AG GG GG GG AG GG AG
GG GG GG GG AG GG GG AG
AG AA GG AG GG GG GG
58810492
968 rs10001148 4 GG GG GG GG GG
AG GG GG GG GG GG GG GG
GG GG AG AG AG AG AG AG
AA AA AA AA AG AG AG GG
GG AG AG AG AG GG AA
164101614
969 rs10001177 4 GG GG GG GG GG
GG GG GG GG GG GG GG GG
GG AG GG GG GG GG AG GG
GG GG GG GG GG AG GG AG
GG GG AG GG GG GG AG
5249944
970 rs10001207 4 GG GG GG GG GG
GG GG GG GG GG GG GG GG
GG GG GG GG GG GG GG GG
GG GG GG GG GG GG GG GG
GG GG GG GG GG GG GG
1755491
971 rs1000121 20 AA AA AG AA AA
AA AG AG AG AA AG AA AG
AG AA AA AA AA AA AA AA
AA AA AA AA AA AA AA AA
AA AA AG AA AG AG AA
16223957
972 rs10001210 4 AG GG GG AA AA
AG AA AG AG GG GG GG GG
GG AG GG GG GG GG GG AG
GG GG GG AG GG GG GG GG
AG GG AG AG AA AA AA
165452969
973 rs10001236 4 AC AC AA CC AA
AC AC AC AC AC CC AC CC
CC CC AC AA AC AC AC AA
CC CC CC CC AC CC AC AC
AC CC CC CC AC CC AC
29763937
974 rs10001239 4 AG AG AA AG AG
AA GG AG AG AA AG AG AA

AA AG AG AG AA AG AG AG
AA AG AG AG AG AG AG GG
GG AA AG AA AA AA AA
23626018
975 rs10001348 4 AA AA AA AA AA
AA AA AA AA AA AA AG AA
AA AG AA AA AA AA AA AA
AA AA AA AA AA AA AA AA
AA AA AA AA AA AA AA
29912308
976 rs10001353 4 AC CC AC CC CC
CC CC CC CC CC -- CC CC
CC CC CC CC CC CC CC CC
AC AC AC AC CC CC CC CC
-- AC CC CC CC AC CC
7613981
977 rs1000137 7 GG GG GG AG AG
AA GG GG GG GG AG AG GG
GG AG GG GG GG AG AG GG
AA AG AA AA GG GG GG GG
GG AG AG AG AA AA AG
157644152
978 rs10001385 4 AG AA AA AA AA
AA AA AA AA AA AA AA AA
AA AG AA AA AA AG AG AA
AA AA AA AA AA AG AG AA
AA AA AA AA AA AA --
171751059
979 rs1000141 2 AG AG AG AG AA
AG AG GG AG GG AA AA AA
AA AA AA AA AG AA GG AG
AG AG AG GG AG AG GG AG
AG AG AA AG AG GG AG
234242347
980 rs10001410 4 AA AA AA AC CC
AC AC CC AC AC AC CC CC
AC AA AA AC AC AC AC AA
AA AA AA AA AA AA AA AA
AA AC AA AC CC CC AC
62474229
981 rs10001417 4 AA AA AA AA AA
AA AA AA AG AG AG AA AG
AG AG AA AG AG AA AA AA
AA AA AA AA AA AG AG AA
AA AA AA AA AA AA AA
131392651
982 rs1000144 5 AA AA AA GG GG
AG AG AA AA AA AA AA AG
AG AG AA AA AA AA AA AA
AA AA AA AA AA AG AA AA

	AA	AG	AA	AA	AA	AA	AA	
176284713								
983	rs10001447		4	AG	AG	GG	AG	GG
	GG	AG	AG	GG	AG	GG	GG	GG
	AG	GG	AA	AG	AG	AG	AG	GG
	GG	GG	AG	AG	AG	AG	AA	AG
	AG	AA	AG	AA	GG	GG	GG	
182756765								
984	rs10001464		4	AA	AG	AA	GG	GG
	GG	GG	GG	GG	GG	GG	GG	GG
	AG	AG	AG	AG	GG	GG	GG	GG
	GG	AG	AG	AG	GG	AG	AG	GG
	GG	GG	GG	GG	AG	AA	GG	
101137084								
985	rs10001470		4	AA	AA	AA	AA	AA
	AA	AA	AA	AA	AA	AA	AA	AG
	AG	AA	AG	AG	AA	AA	AA	AA
	AG	GG	GG	AA	AA	AA	AA	AA
	AA	AG	AG	AA	AA	AA	AA	
36461320								
986	rs10001483		4	AA	AA	AA	AA	AA
	AA	AA	AA	AA	AA	AA	AA	AA
	AA	AA	AA	AA	AA	AA	AA	AA
	AA	AA	AA	AA	AG	AA	AG	AA
	AA	AG	AG	GG	AG	AG	AA	
106936604								
987	rs10001487		4	AA	AG	AA	AA	AA
	AA	AA	AA	AA	AA	AG	AG	GG
	GG	GG	AA	AG	AG	AG	AG	AA
	GG	GG	GG	GG	GG	GG	GG	GG
	AA	AG	AG	AA	GG	GG	GG	
187508057								
988	rs10001495		4	AG	GG	AG	GG	AG
	GG	AG	GG	GG	GG	GG	AG	GG
	GG	AA	AG	AG	AG	AA	AG	AG
	GG	GG	GG	GG	AG	AG	AG	GG
	AG	AG	GG	AG	GG	GG	GG	
149936868								
989	rs10001565		4	AG	GG	GG	GG	GG
	GG	GG	AG	GG	AG	GG	GG	GG
	GG	GG	AG	AG	GG	GG	GG	GG
	GG	AG	GG	GG	GG	GG	GG	GG
	GG	GG	GG	GG	AG	GG	AG	
15722573								
990	rs1000160		13	AG	GG	AG	GG	AG
	AG	AG	AG	GG	GG	GG	AG	GG
	GG	GG	AG	AG	GG	GG	AG	GG
	AG	AG	AA	AA	AA	AG	AA	AG
	AG	AG	AG	GG	AA	AA	AG	
100950452								

991 rs10001651 4 AG AG AG AA AA
AA AA AA AA AA AA AA AA
AA AA AA AA AA AA AG AA
AG AA AG AG AG AA AG AG
AA AA AA AA AA AA AA
151071888
992 rs10001661 4 AA AA AG AG GG
AG AG AA AA AG GG GG AG
AA AA AA AA AA GG GG GG
AA AA AA AA AA AG AG AA
AA AG AA AG AG AA AG
89249372
993 rs1000170 4 AA AA AA AA AA
AA AA AA AA AA AA AA AA
AA AA AA AA AA AA AA AA
AA AA AA AA AA AA AA AA
AA AA AA AA AA AA AA
1867825
994 rs1000171 4 AA AA AA AA AA
AA AA AA AA AA AA AA AA
AA AA AA AA AA AA AA AA
AA AA AA AA AA AA AA AA
AA AA AA AA AA AA AA
1867771
995 rs1000176 20 AA AA AA AG AG
GG AG GG GG GG GG GG AG
AG GG AG GG AG GG GG GG
AG AG GG AG GG GG GG GG
GG GG AG GG GG GG GG
21233553
996 rs10001825 4 AA AA AG AA AA
AA AG AA AA AA AA AA AA
AA AA AG AG AA AA AA AA
AA AA AA AA AA AA AA AA
AG AA AA AA AA AA AA
4884332
997 rs10001855 4 CC CC CC CC CC
CC CC CC CC CC CC CC CC
CC CC CC CC CC CC CC CC
CC CC CC CC AC AC AC AA
CC AC CC CC CC CC CC
53744485
998 rs10001857 4 AG AA AA GG GG
AG AG AA AG AG AA AA AA
AA AA AA AG AG AG AG AA
AG AA AA AG AA AA AA AA
AG AG AA AG AG AA GG
129223349
999 rs10001894 4 AG AG AG AA AA
AA AA AA AA AG AA GG AG

AA AG AA AA AA AA AA AA
AA AA AA AA AA AA AA AA
AA AA AA AA AA AA AA
113454932
1000 rs10001933 4 AG GG AG AA AG
AG AA AA AA AA AA AA AA
AA AA AA AA AA AA AA AA
AG AA AA AA AA AA AA AA
AA AG AA AG AA AA AA
173758268
1001 rs10001960 4 AA AA AA AA AA
AA AA AA AA AA AA AA AA
AA AA AA AA AA AA AA AA
AA AA AA AA AA AA AA AA
AA AA AA AA AA AA AA
185579345
1002 rs10001989 4 GG GG GG GG GG
GG GG GG GG GG GG GG GG
GG GG GG GG GG GG GG GG
GG GG GG GG AG GG GG GG
GG GG GG GG GG GG GG
19035608
1003 rs10002090 4 AA AA AA AA AA
AA AA AA AA AA AA AA AA
AA AA AA AA AA AA AA AA
AA AA AA AA AA AA AA AA
AA AA AA AA AG AG AA
57859592
1004 rs10002142 4 AC AC CC CC AC
AC CC CC CC CC CC CC CC
CC CC CC CC CC AC AC AC
AA AC AC CC CC AC CC AC
AC AC CC CC CC CC CC
173178085
1005 rs10002151 4 GG AG AA GG GG
GG GG GG GG GG GG GG AG
GG GG GG GG GG GG GG AG
GG GG GG GG GG GG GG GG
GG AG GG GG GG GG GG
4449628
1006 rs10002187 4 GG GG AG GG GG
GG GG GG AG AG GG AG GG
GG AG AG AG AG GG GG AG
GG GG GG GG GG GG GG GG
GG GG AG GG AG GG AG
150915414
1007 rs1000221 15 GG GG GG GG GG
GG GG GG GG GG GG GG GG
GG GG GG GG GG GG GG GG
GG GG GG GG GG GG GG GG

GG GG GG GG GG GG GG
89627396
1008 rs10002254 4 AA AA AA AA AA
AA AA AG AG AA AA AA AA
AA AA AG AG GG AA AA AA
AA AA AA AA AA AA AA AA
AA AA AA AA AG AG AG
29626865
1009 rs10002261 4 GG GG GG GG GG
GG GG GG GG GG GG GG GG
GG GG GG GG GG GG GG GG
GG GG GG GG GG GG GG GG
GG GG GG GG GG GG GG
53488862
1010 rs10002311 4 AA AA AA AA AA
AA AA AA AA AA AA AC AC
AC AC AA AA AA AC AC AA
AA AC AC AC AA AA AA AA
AA AA AC AA AC AC AC
77442504
1011 rs10002340 4 AC CC AC AA AA
AA AA AA AA AA AA AA AA
AA AA AA AA AA AA AA AA
AC CC CC AA AA CC AC AC
CC AA AA AA AA AA AA
31249967
1012 rs1000236 8 AG AA AA AG GG
GG AG AG AG AG GG AG AG
AG GG AG AA GG GG GG AG
AA AG AG AA AG GG AG AG
AG AG AG AG AG GG AA
38474484
1013 rs10002384 4 AA AA AA AA AA
AA AA AA AA AA AA AA AA
AA AA AA AA AA AA AA AA
AA AA AA AA AA AA AA AA
AA AA AA AA AA AA AA
54927037
1014 rs10002388 4 AA AA AA AA AA
AA AA AG AG AG AA AA AA
AA AA GG AG AG AA AA AA
AA AA AA AA AA AA AA AA
AA AA AA AA AG AG AG
182370185
1015 rs10002393 4 AG AG GG AA AA
AA AA AG GG GG AA AA AG
AG AG AG AA AG AA AA AA
AA AG AG AG AA AA AA AA
AA AA AA AA AG AA AG
82786911

1016	rs10002437		4	GG	AG	AG	AG	AG
	AG	GG	GG	GG	GG	GG	GG	GG
	GG	GG	AG	AG	GG	GG	GG	GG
	GG	GG	GG	GG	GG	GG	GG	GG
	AG	GG	AG	AG	AG	AG	GG	
1157776								
1017	rs1000244		19	AA	AA	AA	AA	AA
	AA	AA	AA	AA	AA	AA	AA	AA
	AA	AA	AA	AA	AA	AA	AA	AA
	AA	AA	AA	AA	AA	AA	AA	AA
	AA	AA	AA	AA	AA	AA	AA	
19030467								
1018	rs10002444		4	GG	GG	GG	GG	GG
	GG	GG	GG	GG	GG	GG	GG	GG
	GG	GG	GG	GG	GG	GG	GG	GG
	GG	GG	GG	GG	GG	GG	GG	GG
	GG	GG	GG	GG	GG	GG	GG	
99926								
1019	rs10002472		4	AA	AG	AG	GG	GG
	AG	GG	AG	AG	AA	GG	GG	GG
	GG	GG	AG	AG	GG	AG	AG	AG
	GG	GG	GG	GG	GG	AG	GG	AG
	AA	GG	AA	AG	GG	AG	GG	
158867973								
1020	rs10002500		4	AG	AA	AG	GG	GG
	GG	GG	GG	GG	GG	AG	AG	AG
	AA	AG	GG	GG	GG	GG	GG	GG
	GG	GG	GG	AG	GG	GG	GG	GG
	GG	AG	GG	GG	GG	GG	GG	
47958679								
1021	rs10002518		4	AA	AA	AA	AG	AA
	AG	AA	AA	AA	AG	AA	AA	AA
	AA	AA	AA	AA	AA	AA	AA	AG
	AA	AG	AG	AA	AA	AG	AA	AG
	AA	GG	AA	AG	AG	AG	AA	
111927642								
1022	rs10002552		4	AG	AG	AA	AG	AG
	AG	AG	AA	AA	AA	AA	AG	AG
	AG	AG	AA	AA	AA	AG	AG	GG
	AA	AA	AA	AA	GG	AG	AG	GG
	AG	AG	AG	AG	AA	AA	AA	
24358882								
1023	rs10002568		4	AA	AA	AC	AA	AA
	AA	AA	AC	AA	AC	AA	AA	AA
	AA	AA	AC	AC	AC	AC	AC	AC
	AA	AA	AA	AC	AC	AC	CC	AC
	CC	AC	AC	AC	AA	AA	AA	
183514227								
1024	rs10002595		4	AA	AA	AG	AG	AG
	AA	GG	AG	AG	AG	AA	AA	AA

	AA	AA	AA	AG	AA	AA	AA	AA
	AA	AA	AA	AA	AG	AG	AA	GG
	AA	AA	AA	AA	AA	AA	AA	
110384938								
1025	rs10002598		4	AA	AG	AG	AA	AG
	AG	AG	AA	AA	GG	AA	AA	AA
	AA	AA	AG	AG	AG	AG	GG	AA
	AG	AG	AG	AG	GG	AG	AG	GG
	AA	AG	AA	AA	AA	AA	AG	
105231571								
1026	rs1000264		8	AG	AG	GG	AA	AG
	AG	AA	AG	AG	AG	AG	AG	AA
	AA	AG	AG	AG	AA	AA	AA	AG
	AA	AA	AA	AG	GG	AA	AG	AG
	AA	GG	AG	GG	AG	AG	AG	
134110278								
1027	rs10002688		4	AG	AA	AG	GG	AG
	AG	GG	GG	GG	GG	AG	AA	AA
	AG	GG	AA	AG	AG	AA	AA	AG
	AA	GG	GG	GG	GG	GG	GG	GG
	GG	AA	GG	AG	GG	AG	AG	
75261407								
1028	rs10002695		4	CC	CC	CC	CC	CC
	CC	CC	CC	CC	CC	CC	CC	CC
	CC	AC	AC	AC	AC	CC	CC	CC
	CC	CC	CC	CC	CC	AC	AC	CC
	CC	CC	CC	CC	CC	CC	CC	
116560690								
1029	rs10002703		4	GG	GG	AG	GG	GG
	GG	GG	GG	GG	AA	GG	GG	GG
	GG	GG	GG	GG	GG	GG	GG	GG
	GG	AG	AG	GG	GG	GG	GG	GG
	GG	GG	GG	GG	AG	AG	AG	
98371858								
1030	rs10002760		4	GG	GG	GG	AG	AA
	AA	AG	AG	AG	AG	AG	AG	AG
	AA	GG	GG	AG	AG	AG	AG	AG
	GG	GG	GG	GG	AG	GG	AG	AG
	GG	AG	AA	AA	GG	AG	GG	
180305067								
1031	rs10002767		4	AG	AA	AA	AG	AG
	AA	AG	AA	AA	AA	AA	AA	AA
	AA	AG	AG	AA	AG	AA	AG	AA
	AA	AA	AA	AA	AA	AA	AA	AA
	AA	AA	AA	AA	AA	AA	AG	
99298166								
1032	rs10002793		4	AG	AG	AG	AG	AG
	AA	GG	AA	GG	AG	GG	GG	GG
	GG	GG	AG	AG	AG	GG	GG	GG
	GG	GG	GG	AG	AG	GG	GG	GG

	AG	AG	GG	GG	GG	GG	AG	
186045532								
1033	rs10002802		4	AC	CC	AC	AA	AC
	AC	AA	AC	AC	AA	CC	AC	CC
	CC	AC	AA	AC	AC	AA	AA	AA
	CC	AC	AC	CC	AA	AA	AA	AA
	AC	AC	AC	AC	CC	AC	CC	
121526447								
1034	rs10002811		4	AA	AA	AA	AA	AA
	AA	AG	GG	AG	AG	AG	AG	AA
	AA	AA	AG	GG	AG	AA	AG	AG
	AA	AA	AA	AA	AA	AG	AA	AA
	AA	AG	AG	GG	AG	AA	AG	
60129838								
1035	rs1000283		1	AG	AG	AG	GG	GG
	GG	GG	GG	GG	GG	GG	GG	GG
	GG	GG	GG	GG	GG	AG	AG	AG
	GG	GG	GG	GG	GG	GG	GG	GG
	GG	AG	GG	AG	GG	GG	GG	
209894661								
1036	rs1000286		8	GG	GG	GG	GG	AG
	AG	GG	AG	AG	AG	GG	GG	GG
	GG	GG	GG	AG	AG	AG	GG	GG
	GG	GG	GG	GG	AG	GG	GG	AG
	GG	GG	GG	GG	GG	GG	GG	
96109370								
1037	rs10002861		4	AG	GG	AG	GG	GG
	GG	GG	GG	GG	GG	AG	AG	AG
	AG	AG	GG	GG	GG	AG	GG	AA
	AG	AG	GG	AG	GG	AG	AG	AG
	GG	AG	GG	AG	AA	AA	AG	
64976578								
1038	rs1000291		3	AG	AG	AA	AG	AG
	AA	GG	AA	AG	GG	GG	GG	AG
	AG	AG	AG	AG	AA	AG	AG	AG
	AA	AG	AG	AG	AG	AA	AA	AA
	GG	AG	AA	AG	GG	AG	AG	
188982808								
1039	rs10002917		4	AG	AA	AG	AA	AA
	AA	AA	AA	AG	AA	AA	AA	AA
	AA	AG	AG	AA	AA	AG	AG	AG
	AA	AA	AA	AA	AG	AG	AG	GG
	AG	AA	AG	AG	AG	AG	AG	
142169704								
1040	rs1000295		12	AG	AG	AG	AG	GG
	GG	AG	GG	GG	AG	GG	GG	GG
	GG	GG	AG	AG	AG	AG	GG	AG
	AG	AG	GG	AG	AA	GG	AG	AG
	AG	AG	GG	AG	GG	GG	AG	
104303246								

1041 rs10002958 4 AA AG GG AA GG
AG AG AG AG AG AA AA AA
AA AA AA AA AA AA AA AG
AG AA AA AG AA AG AA AA
AG AG AG AG AG AG AA
31744915
1042 rs10002960 4 GG GG GG GG GG
GG GG GG GG GG GG GG GG
GG GG GG GG GG AG AG GG
GG GG GG GG GG GG GG GG
GG GG GG GG AG GG AA
65313065
1043 rs10003001 4 AG AG GG GG GG
GG GG AG AG AG GG GG GG
GG GG GG GG GG GG GG AG
GG GG GG GG AG AG GG AG
GG GG AA AG GG GG GG
9984475
1044 rs10003040 4 GG AG AG AG GG
AG AG GG GG GG AG GG AG
AG GG GG GG GG AG AG AG
AG AG AG AA GG GG GG GG
GG GG GG GG GG GG GG
84779091
1045 rs1000313 1 AA AA AA AG AG
AG AA GG AA AG AG AG AG
AG AG AA AA AA AG AA AA
AA AA AG AA AA AA AA AA
AG AG AA AA AG AG AA
15405489
1046 rs10003132 4 AA AA AA AA AA
AA AA AA AA AA AA AA AA
AA AA AA AA AA AA AA AA
AA AA AA AA AA AA AA AA
AA AA AA AA AA AA AA
48120253
1047 rs10003194 4 AA AA AA AA AA
AA AA AA AA AA AA AA AA
AA AA AA AA AA AA AA AA
AA AA AA AA AA AA AA AA
AA AA AA AA AA AA AA
167068199
1048 rs10003267 4 AG AG GG AA AA
AG AA AG AG AG AG AG AG
AG AA GG GG GG AA AG AA
AG GG GG AG AA AG AA AG
AG AG AG AG GG GG AG
124966525
1049 rs1000329 19 AA AG AA AA AA
AA AA AG AA AA AG GG GG

AG AG AA AA AA AA AA AG
AA AA AA AA AA AA AA AA
GG AA AA AA AA AA AG
16449517
1050 rs10003330 4 AA AA AA AA AA
AA AA AA AA AA AA AA AA
AA AA AA AG AG AA AA AA
AA AA AA AA AA AA AA AA
AA AA AA AA AA AA AA
96424923
1051 rs10003357 4 GG GG AG GG GG
AG GG GG GG GG GG AG GG
GG GG GG GG GG GG GG GG
AG AG AG AG GG AG AG GG
GG GG GG GG GG GG AG
137652477
1052 rs10003413
4 -- -- -- -- -- -- -- --
-- -- -- -- -- -- -- --
-- -- -- -- -- -- -- --
-- -- -- -- -- -- -- --
-- -- -- -- 186489396
1053 rs10003420 4 AA AA AA AA AA
AA AA AA AA AA AA AA AA
AA AA AA AA AA AA AA AA
AA AC AC AC AA AA AA AA
AA AA AA AA AA AA AA
56089259
1054 rs1000346 3 AA AA AA AA AA
AA AA AA AA AA AA AA AA
AA AA AA AA AA AA AA AA
AA AA AA AA AA AA AA AA
AA AA AA AA AA AA AA
90089238
1055 rs1000352 1 AG AA AG AA AG
AA AG AA AA AA AA AA AA
AA AA AA AA AA AA AA AA
AA AA AA AA GG AA AG AG
AA AA AA AA AG AG AA
209675578
1056 rs10003547 4 -- CC CC AC CC
CC AC AA AA AC AA AA AA
AA AC AA AC AC AA AC AA
AA AC AC AC AC CC AC AC
CC AC CC AC AC CC AC
162514902
1057 rs10003585 4 AA AA AA AA AA
AA AA AG AG AA AA AA AA
AA AA AA AA AA AA AA AA
AA AA AA AA AA AA AA AA

AG AA AG AG AA AA AA
122572851
1058 rs10003626 4 AA AA AA AA AA
AA AA AC AC AC AA AA AC
AC AA CC AC AC AC AC AC
CC CC CC AC AC AC AC AC
AA AC AC AA AA AA AA
36396090
1059 rs1000373
21 -- -- -- -- -- -- -- --
-- -- -- -- -- -- -- --
-- -- -- -- -- -- -- --
-- -- -- -- -- -- -- --
-- -- -- -- 41556414
1060 rs10003746 4 GG GG AG AA AA
AG AA AG AG AA GG GG AG
AG GG AG GG AG AA AA AG
GG GG GG GG AA AG AA AG
GG AG GG AG AA AA AG
17469817
1061 rs10003762 4 AA AA AA AA AA
AA AA AA AA AA AG AA AA
AA AA AA AG AG AA AA AA
AA AA AA AA AG AA AG AG
AG AA AG AG AG AA AG
11519011
1062 rs10003771 4 AA AA AG AA AA
AA AA AA AA AA AA AG AA
AA AG AA AG AG AA AA AA
AG AG AG AA AA AA AA AA
AA AA AG AA AG AA AG
11069107
1063 rs10003774 4 AA AG AA AA AA
AA AA AA AA AA AA AG AG
AG AG AA AA AA AA AA AA
AA AA AA AA AG AG AA AG
AA AG AG AA AA AA AG
132551302
1064 rs10003787 4 -- -- -- CC CC
-- -- AC AC AC -- CC --
CC -- AC -- AC CC -- --
AC AC AC AC AC -- -- AC
AC AC AC CC -- -- --
83421440
1065 rs1000380 5 AA AA AA AG AG
AG AA AA AA AA AA AA AA
AA AA AA AA AA AA AA AA
AA AA AA AA AA AA AA AA
AA GG AG AG AA AA AA
56886189

1066	rs10003818		4	AA	AA	AG	AA	AG
	AA	AG	AA	AA	AA	AG	AG	AG
	AG	AA	AA	AA	AA	AG	AG	AA
	AA	AG	AG	AG	AG	AG	--	AG
	AG	AA	AA	AA	AA	AA	AA	
172705236								
1067	rs1000383		20	GG	GG	GG	GG	GG
	GG	GG	AG	AG	GG	GG	GG	AG
	AG	GG	AG	GG	GG	GG	AG	GG
	GG	GG	GG	GG	AG	GG	AG	AG
	GG	AG	AG	AG	GG	GG	GG	
45092921								
1068	rs10003861		4	CC	CC	CC	AC	AC
	CC	AC	CC	CC	CC	CC	CC	CC
	CC	CC	CC	CC	CC	CC	CC	CC
	CC	CC	CC	AC	CC	CC	CC	CC
	CC	CC	CC	CC	CC	CC	CC	
41212997								
1069	rs1000394		8	AG	AG	AG	GG	AG
	AG	GG	AG	AG	AG	AG	AG	AG
	AG	GG	AG	AG	AG	GG	GG	GG
	GG	GG	GG	AG	GG	AG	GG	GG
	AG	AG	GG	AG	GG	GG	GG	
135870334								
1070	rs10003943		4	GG	GG	GG	AA	AA
	AA	AG	AA	AA	AA	AG	AG	AA
	AG	AG	AG	AG	AA	AG	GG	AG
	AG	AG	AG	AG	AA	AG	AG	AA
	AG	AG	AG	AA	AG	AG	GG	
1512906								
1071	rs10004012		4	AA	AA	AA	AG	AA
	AA	AG	AG	AG	AG	AG	AG	GG
	AG	GG	AA	AA	AA	AA	AA	AA
	AA	AA	AA	AA	AA	AA	AA	AA
	AA	AA	AA	AA	AG	AG	AG	
189018613								
1072	rs10004025		4	AG	AA	AG	AG	GG
	AG	AG	GG	GG	AG	AG	GG	GG
	GG	AG	AG	GG	GG	AA	AA	AA
	AG	AG	AG	AG	AG	AG	AA	AG
	GG	AG	AG	GG	AA	AA	AA	
169491370								
1073	rs10004038		4	AG	AA	AA	AA	AG
	AG	AA	AA	AA	AA	AA	AG	AG
	AA	AG	AA	AA	AA	AA	AA	AA
	AG	AG	AG	AG	AA	AA	AA	AA
	AA	AA	AG	AA	AA	AA	AA	
47573660								
1074	rs10004074		4	GG	GG	GG	GG	GG
	GG	GG	GG	AG	GG	GG	GG	GG

	GG	GG	GG	GG	GG	GG	GG	GG
	GG	GG	GG	GG	GG	GG	GG	GG
	GG	AG	AG	AG	GG	GG	AG	
139837873								
1075	rs10004104		4	AG	AG	AG	AG	AA
	AG	AA	AA	AA	AA	AG	AA	AA
	AG	AG	AG	AA	AG	AG	AA	--
	AG	AA	AA	AA	--	AG	--	AG
	AG	--	AA	AG	AG	AG	AG	
91790439								
1076	rs1000411		20	AG	AG	AG	GG	GG
	GG	GG	GG	GG	GG	GG	GG	AG
	AG	AG	GG	GG	GG	AG	GG	AG
	AG	AG	GG	AG	AG	GG	AG	AG
	GG	GG	GG	GG	GG	GG	AG	
39634616								
1077	rs10004122		4	AG	AA	AA	AA	AG
	AG	AG	AA	AA	AA	AG	GG	AG
	GG	AG	AA	AA	AA	AA	AA	AA
	AA	AG	AG	AG	AA	AG	AA	AA
	AA	GG	AG	GG	AG	AA	AG	
46346657								
1078	rs10004154		4	AG	AG	AG	AG	AA
	AG	AA	AA	AA	AA	AA	GG	AG
	AG	AG	AA	AA	AA	AG	AG	AA
	AG	AA	AA	AA	AA	AA	AA	AA
	GG	AA	AG	AG	AG	GG	AG	
111380759								
1079	rs1000416		10	AG	GG	GG	AA	AG
	AG	AG	GG	AG	GG	GG	GG	GG
	GG	GG	GG	AG	AG	GG	GG	AG
	GG	GG	GG	GG	GG	AG	AG	GG
	AG	AG	GG	GG	GG	AG	GG	
2256731								
1080	rs1000417		1	AA	AA	AA	AG	GG
	GG	AG	AA	AA	AA	AA	AA	AA
	AA	AA	AG	AG	AG	AG	AG	AG
	AA	AA	AA	AA	AA	AA	AA	AA
	AA	AA	AA	AA	AG	AG	AA	
34007886								
1081	rs10004177		4	AC	AA	AA	CC	AC
	CC	AC	CC	CC	AC	AA	AA	AC
	AC	AC	AC	AC	AC	AA	AC	AC
	CC	CC	CC	CC	AC	CC	CC	CC
	AC	AC	AA	AA	AC	AC	CC	
161104701								
1082	rs1000421		8	AA	AA	AA	AA	AG
	AA	AG	AA	AA	AA	AA	AA	AA
	AA	AA	AA	AA	AA	AA	AA	AA
	AA	AA	AA	AA	AG	GG	GG	AG

AG AG AG GG AA AA AA
105715533
1083 rs10004227 4 AA AC CC CC CC
AC CC CC AC CC CC CC AC
CC AC CC CC CC CC CC AC
AA AA AA AC AC AA AC AC
AC AC CC CC AA AA AC
5763446
1084 rs10004285 4 AG GG GG AG GG
AG AG AA AA AA GG GG AG
AG AA AG AA AG GG GG AG
AG AA AA AG AG AA AG AA
AG AA AG AA GG AG GG
59559715
1085 rs100043 17 AG AA AA GG GG AG
AG AG AG AG AG AG AA AG
AG GG GG GG AA AG AG GG
GG AG AG GG AG GG GG AG
AG AA AG GG AG GG 67392041
1086 rs10004377 4 AA AA AC AA AA
AA AC AA AA AC AC AA AC
CC AC AC AC CC CC CC CC
AC AA AA AA AC AA AC AA
AC AC AC AA AA AA AC
110648124
1087 rs10004397 4 AC AA AC AA AC
AC AA AC AC AC AC AC AC
AA AA CC AC AC AA AA AA
AC CC CC AA AA AC AC AC
AA AC AC AA AC AC AC
45626526
1088 rs1000440 9 AG AA AG AG GG
AG GG AG AG GG GG AG GG
GG GG AG AG AG AG AG GG
AG AG AG AG AG AG AA AA
AG AG GG GG AG AG AG
101258881
1089 rs1000443 8 AG AG AG AG AG
AG GG AG AG GG AG AA AG
AG GG GG GG GG AG AG AA
GG GG GG GG GG AG AG AG
GG AG AG GG AG AA GG
21717790
1090 rs10004440 4 GG GG GG GG AG
AG GG GG GG GG GG GG GG
GG GG GG GG GG GG GG GG
AG GG GG GG GG AG GG AG
AG GG GG GG GG GG GG
80272792
1091 rs10004447 4 AG AG AG AG AG

AG AA GG GG AG GG GG AG
AG AG AG AG AG AG AG AA
AG AG AG AA AA AG AG AA
AG AA GG AG AA AG AA
169390717
1092 rs10004475 4 AG AG AG GG GG
GG GG GG GG GG AG AG AG
AG GG AG GG AG GG GG GG
GG GG GG GG GG AG GG AG
GG GG GG GG GG GG GG
157304598
1093 rs1000449 14 AA AA AA AA AA
AA AA AA AA AA AA AG AG
AA AA AA AA AG AG AA AA
AA AA AA AA AA AA AA AA
AG AG AA AA AA AA AA
71707567
1094 rs10004500 4 CC CC CC CC AC
AC CC AC CC AC AC AC AC
AC CC AA AA AA AA AA AA
AA AC AC CC AC AC CC AC
AC AC AC AC CC CC AC
177119860
1095 rs10004503 4 GG GG GG GG GG
GG GG GG GG GG GG GG GG
GG GG GG GG GG GG AG GG
GG AG AG AG GG GG GG GG
AG GG GG GG GG GG GG
793929
1096 rs10004516 4 AG AG AG AG GG
AG GG GG GG GG GG AA AG
AG AG AG AG AG GG GG GG
GG AG AG AG GG GG GG GG
AA GG AG AG AG AA AG
111398208
1097 rs10004538 4 AA AA AG AA AA
AA AA AA AG AA GG GG AG
AG AG GG GG AG AA AA AA
AG AG AG AA AA AA AA AA
AA AA AG AA AA AA AG
187378920
1098 rs10004565 4 AG AA AG AG AG
AG AA AG AG AA AA AA AG
AG AA AG AA AA AA AA AA
AA AG AA AA AG GG AG AG
AA AA AG AA AG AA AG
139605950
1099 rs1000459 2 GG GG GG AA AG
AG AG AG AG AG AA GG AG
GG GG GG GG GG AG AG AG

GG GG GG GG GG AG GG AG
GG AG GG AG GG GG GG
76501978
1100 rs1000461 8 GG GG GG AG GG
GG AG AG AG AG GG GG GG
GG GG GG GG GG GG GG AG
GG GG GG GG AA GG AG AG
AG AG GG GG GG GG AG
104186211
1101 rs1000465 17 AG AA AG GG AG
GG AG GG GG GG GG GG AG
GG GG AA AA AA GG GG GG
GG GG AG AG AA GG AG AG
AG AG AG AG GG GG GG
69803672
1102 rs1000466 17 AA AA AA AA AA
AA AA AA AA AA AA AA AA
AA AA AG AG AG AA AA AA
AA AA AA AA AA AA AA AA
AA AG AG AG AA AA AA
69803730
1103 rs10004675 4 AG AA AA AA AA
AA AA AG AG AG AG GG AG
AG GG AG AG GG AG AG AG
GG AG AG GG AG AG AG AG
AG AG AA AA AG AG AG
32260388
1104 rs10004685 4 AG AG AG -- --
-- -- -- AG -- AG AG --
-- -- AA AA AA -- -- AG
AG AG AG -- AG -- -- --
-- -- -- -- AG -- AG
13555695
1105 rs10004699 4 AA AA AA AA AA
AA AA AG AA AG AA AA AA
AA AA AA AA AA AA AA AA
AA AA AA AA AA AA AA AA
AA AA AA AA AA AA AA
180047257
1106 rs10004753 4 AA AC AC AC AC
CC AC CC CC CC AC CC CC
CC CC AC AC AC AC AA CC
CC CC CC CC AC AC AC AC
CC CC AC AC AC AC CC
99447433
1107 rs10004765 4 GG GG GG GG GG
GG GG GG GG GG GG GG GG
GG GG GG GG GG GG GG GG
GG GG GG GG GG GG GG GG
GG GG GG GG GG GG GG

1932576							
1108	rs10004779	4	CC	CC	CC	CC	CC
	CC	CC	CC	CC	CC	CC	CC
	CC	CC	CC	AC	AC	CC	CC
	CC	CC	CC	CC	AC	CC	CC
	CC	AC	AC	AC	AC	CC	AA
96347978							
1109	rs10004910	4	AA	AA	AG	AA	AA
	AA	AG	GG	GG	GG	GG	AG
	AG	AG	AA	AG	AG	AG	AG
	AG	AA	AA	AA	AG	AG	AG
	AA	AA	GG	AG	AG	AA	AG
57968444							
1110	rs1000505	X	AA	AC	CC	AA	AA
	AA	AA	CC	CC	AA	AC	AC
	AC	AC	AA	AA	AA	AC	AC
	CC	AA	CC	CC	AC	CC	AC
	AC	AA	AA	AA	AA	AA	AA
93268266							
1111	rs10005074	4	GG	AG	AG	AG	AA
	AG	AA	AG	GG	GG	AG	AA
	AG	GG	GG	AG	AG	GG	GG
	AG	AG	AG	AA	GG	AG	AG
	AG	AG	GG	GG	AG	AG	AA
59042209							
1112	rs1000521	14	AA	AA	AA	AA	AG
	AG	AA	AG	AA	AG	AG	AG
	AA	AA	AA	AA	AA	AA	AG
	AA	AA	AA	AA	AA	AA	AA
	AG	--	AA	AG	AA	AA	AA
70522484							
1113	rs10005243	4	AG	AG	AA	AG	AA
	AG	AG	AG	GG	GG	AG	AG
	AG	AG	AG	AA	AA	AG	AG
	AA	AA	AG	AG	AG	AA	AA
	AA	AA	AG	AA	AA	AG	AA
185845735							
1114	rs10005281	4	GG	GG	GG	GG	GG
	GG	GG	GG	GG	GG	AG	AG
	AG	AG	GG	GG	GG	GG	GG
	GG	GG	GG	GG	AG	AG	AG
	AG	GG	GG	GG	GG	AG	AG
161363563							
1115	rs1000530	X	GG	AG	AG	AA	AA
	AA	AA	AA	AA	AA	AA	AA
	AA	AA	AG	AA	AG	AA	AG
	AA	AA	AA	AA	AA	AA	AA
	AA	AG	GG	GG	AG	AG	AA
78124078							
1116	rs10005301	4	GG	GG	AG	AG	AG

AG AG AG AG GG AG AA AA
AA AG GG AG AG AG GG AG
AG GG GG AG GG GG GG GG
GG AG GG GG AG GG AA
123715055
1117 rs10005320 4 AG GG GG GG GG
GG GG GG GG GG GG GG GG
GG GG GG GG GG GG GG GG
GG GG GG GG GG GG GG GG
GG GG GG GG AG AG GG
57720234
1118 rs1000533 1 AA AA AA AA AG
AA AG AA AA AA AA AA AA
AA AG AA AA AA AG AG AA
AA GG AG GG AA AG AG AG
AA AG AA AG AA AA AA
168282491
1119 rs10005349 4 GG GG GG GG AA
AG AG GG GG GG AA AA AG
AG AG GG GG GG GG GG GG
AA AG AG AG AG GG GG GG
GG AG AG GG AG AG GG
180466423
1120 rs10005577 4 GG GG GG GG GG
GG GG GG GG GG GG GG GG
GG AG AA AG AG GG GG GG
AG GG GG AG GG GG GG GG
GG AG GG GG GG GG GG
23351305
1121 rs10005650 4 AG AA AA AA AA
AA AA AA AA AA AA AA AA
AA AA AA AA AA AA AA AA
AA AA AA AA AA AA AA AA
AA AA AA AA AA AA AA
7877864
1122 rs10005703 4 AG AG AG GG AG
AG GG GG AG GG GG GG GG
GG GG AG AG AG AA AA AA
GG GG GG GG GG AG GG AG
GG GG AA AG GG GG GG
183803699
1123 rs10005751 4 AA AC AA AC AA
AA AC AC AC CC AC AC AC
AC AC AA AA AA AC AC AC
AA AA AA AA CC AA AC AC
AA AA AA AA AC CC AA
25681990
1124 rs10005776 4 GG GG AG AG GG
AG GG AG AG AG AG AA AG
GG AG AA AG AA GG AG GG

GG AA AA AG AG AA AG AA
GG AG AG GG AA AA AG
111016014
1125 rs10005827 4 AA AA AA AG AA
AA AG AG AA AA AG AG AG
GG AG AG AG AG AA AA AA
AA AA AG AG AG AA AG AG
AA AA AG AG AA AA AA
186322987
1126 rs10005941 4 GG GG GG AG GG
AG GG GG GG GG AG GG GG
GG GG GG GG GG GG GG GG
GG GG GG GG GG GG GG GG
GG GG GG GG GG GG GG
85422270
1127 rs10006030 4 AA AA AA AA AA
AA AA AA AA AA AG GG GG
AG AA AA AA AA AG AA GG
AA AA AA AA AG AA AG AA
AA AA AA AA AA AA AA
108844544
1128 rs10006119 4 GG AG AG AG AA
AG AG AG AG AA AA AG AG
AG AG AA AG AG AG AG GG
AA AG AG AG AA GG AG AG
AG AG AA AA AG GG AG
165849934
1129 rs1000620 11 GG GG GG AG AG
AA GG AG GG GG AA AA AA
AG AA AA AG AA GG AG AG
AG AG AG GG AA GG AG AG
AG AA AG AA AG AG AA
72509713
1130 rs10006245 4 AA AA AA AA AA
AG AA AA AA AA AG AG AA
AA AA AA AG AG AG AG GG
AG AG AG AG AA AA AA AA
AG AA AG AA AA AA AG
189214720
1131 rs10006316 4 GG GG GG GG GG
GG GG GG GG GG GG GG AG
GG GG GG GG GG GG GG GG
GG GG GG GG GG GG GG GG
GG GG GG GG GG GG GG
46137876
1132 rs10006322 4 GG GG GG GG GG
GG GG GG GG GG GG GG GG
GG GG GG GG GG GG GG GG
GG GG GG GG GG GG GG GG
GG AG GG GG GG GG GG

183444952
1133 rs10006389 4 AA AG AA AG GG
GG AG AA AG AG AA AA AA
AA AA AG AG AG AA AA AG
AG AG AG AG AG AA AA AA
AA AG AG GG AG AA AG
186550283
1134 rs10006395 4 AG AG AG GG GG
GG GG AG GG AG GG GG AG
AG GG AG AA AG AG AG GG
GG GG GG GG GG GG GG GG
GG GG GG GG AG AG GG
6862131
1135 rs10006487 4 AA AA AA AA AA
AA AA AA AA AA AA AA AA
AA AA AA AA AA AG AG AA
AG AA AA AG AA AA AA AA
AA AA AG AA AA AA AG
86837535
1136 rs1000658 8 AA AA AA AG AG
GG AA AA AA AA AA AA AA
AA AA AA AA AA AA AA AA
AA AA AA AA AA AG AA AA
AA AG AG AG AA AA AG
27905599
1137 rs10006622 4 GG AG GG GG GG
GG AG GG GG AG GG GG GG
GG AG GG AG AG GG GG GG
GG AG AG GG AG GG AG AG
AG AG AG GG GG GG AG
131596752
1138 rs10006636 4 GG AG AG AA AG
AG AG AA AA AA AA AG AA
AA GG AG AG AA AG GG AA
AG AA AG AG AA GG AG AG
AG AG AA AG AA AA AG
157162514
1139 rs1000664 11 CC AC AC CC CC
CC CC CC CC CC AC AC CC
CC CC CC CC CC CC CC CC
AC CC AC CC CC AC AC CC
AC CC AC CC CC CC CC
45285803
1140 rs10006737 4 GG AG GG AA AA
AA AA AG AG AG AG AA AG
AG AG AA AA AA AA AA AA
AG AA AA AG AG AA AG AA
AG AA AG AG AA AA AG
112988739
1141 rs10006741 4 AG AG AA AG GG

GG AG GG GG AG AG AA AG
AG AG GG AG AG AG AG AG
AA AA AA AA GG AA AG AG
AA AA AG AA AG GG AG
25976544
1142 rs1000676 7 AG AG AG GG GG
GG GG AG AG AG AG AA AG
AG GG AG AG AG GG AG GG
GG GG GG GG GG AG AG AG
AA GG AG AG AG AG AG
129015511
1143 rs10006784 4 AC AC CC AC AA
AC AC CC AC AC CC CC AC
AC CC AC AC CC AC AC AC
AC AA AC CC CC AA AC AC
CC CC AC CC AA AA AA
16660365
1144 rs10006795 4 CC CC CC AC AC
AA AC CC CC CC AC AC AC
AC AA AC AA AA CC CC AC
AC CC AC AC CC AA AC AC
CC CC CC CC AA AA AC
143318673
1145 rs10006864 4 AG AG AA AG AG
AG AG GG GG AG AA AA AA
AA AA AA AG AG AA AA AG
GG GG GG GG AG AG AA AG
GG GG AA AG AG GG AA
171963233
1146 rs1000697 15 AG AA AG AA AA
AG AA AG AG AG AG AA AA
AA AA AA AA AA AG AA AA
AA AA AA AA AA AG AA AA
AG AA AG AA AA AA AA
57992997
1147 rs10007020 4 AG AA AG GG AG
AG AG GG AG AG GG GG GG
GG AG AG AA AG GG GG AG
AA AA AA AG GG AG GG GG
AG AG GG GG GG GG AG
60391854
1148 rs1000703 12 GG AG GG GG AG
AG AG AG AA AG AG AG AA
AA AA AG GG GG AG GG GG
AA AG AA AG AG AG AG AG
AG AG AG AA GG GG AG
95256219
1149 rs10007036 4 AA AG AA AG AG
GG AA AG AA AA AA AA AG
AA AG AA AA AG AA AA AG

AA AA AA AA AG GG GG GG
AA AG AA AG AG AA AG
190371697
1150 rs10007083 4 AG AA AG GG GG
GG GG GG GG GG AG AG AG
AG GG GG AG AG GG GG AG
AG AG AG AG AG AG AG AA
AG GG AG GG GG GG GG
162272259
1151 rs10007205 4 AA AA AA AA AA
AA AA AA AA AA AG AA AA
AA AA AA AA AA AA AA AA
AA AA AA AA AA AA AA AA
AA AA AA AA AA AA AA
21141832
1152 rs1000726 5 AA AA AA AG AG
GG AA AG AG AG AA AA AA
AA AA AA AA AA AG AA AA
AA AA AA AA AA AA AA AA
AA AG AA AG AA AA AA
19520405
1153 rs10007264 4 GG GG GG GG GG
GG GG GG GG GG GG GG GG
GG GG GG GG GG GG GG GG
GG GG GG GG GG GG GG GG
GG GG GG GG GG GG GG
108707236
1154 rs10007278 4 CC CC AC CC CC
CC CC CC CC AC CC CC CC
CC CC AC CC CC AC CC AC
CC CC CC CC CC CC CC CC
CC AC CC CC CC AC CC
106549096
1155 rs1000728 21 AC AA AC CC AA
AC AC AC AC AC CC CC CC
CC CC CC AC AC AC AA AC
CC CC CC CC AC CC CC AC
AC AA AC AA AC AA AC
15984178
1156 rs1000730 1 AA AG GG GG AA
AG AG AG GG AA AG AG AG
AG AA AG AA AG GG AG GG
AG AA AA AG AG GG GG GG
GG AG GG AG AG AG GG
231963601
1157 rs10007324 4 AG GG AG GG AG
AG GG GG GG GG GG GG GG
GG GG GG AG AG AG AG GG
GG GG GG GG AG GG GG GG
GG GG AG AG AG AG GG

179576074							
1158	rs10007349	4	GG	GG	GG	GG	GG
	GG	GG	GG	GG	GG	GG	GG	GG
	GG	GG	AG	AG	AA	GG	GG	GG
	GG	GG	GG	GG	GG	AG	GG	AG
	GG	AG	GG	GG	GG	AG	GG	
25226538							
1159	rs10007350	4	GG	GG	GG	GG	GG
	GG	GG	GG	GG	GG	GG	GG	AG
	GG	AG	GG	GG	GG	GG	GG	GG
	GG	AG	AG	AA	GG	GG	GG	GG
	AG	AG	AG	AG	AG	AG	AA	
181126498							
1160	rs10007367	4	AA	AA	AA	AA	AA
	AA	AA	AA	AA	AA	AA	AA	AA
	AA	AA	AA	AA	AA	AA	AA	AA
	AA	AA	AA	AA	AA	AA	AA	AA
	AA	AA	AA	AA	AA	AC	AC	
151214038							
1161	rs1000737	15	GG	GG	GG	GG	GG
	GG	AG	GG	AG	AG	GG	GG	GG
	GG	GG	AG	GG	GG	AG	AG	AG
	AG	AA	AA	AG	GG	GG	GG	GG
	GG	AG	GG	AG	GG	GG	GG	
94968043							
1162	rs1000738	6	AG	AG	AA	GG	GG
	AG	GG	AG	GG	GG	AG	AG	AG
	GG	GG	GG	GG	GG	GG	AG	AG
	AG	AG	AG	AG	GG	AG	AG	AG
	AG	GG	GG	GG	AG	GG	AG	
137828235							
1163	rs10007463	4	GG	GG	GG	GG	GG
	GG	AG	AG	AG	GG	GG	GG	GG
	GG	AG	GG	GG	GG	GG	GG	GG
	GG	AG	AG	GG	GG	GG	GG	GG
	GG	AG	GG	GG	GG	AG	AG	
95155719							
1164	rs10007534	4	AA	AA	AA	AG	GG
	AG	GG	GG	GG	AG	GG	GG	GG
	AG	AA	AG	AA	GG	AA	AA	AA
	AG	AA	AA	AG	GG	AG	GG	AG
	AG	AG	AA	AG	AA	AA	AA	
96798214							
1165	rs10007576	4	GG	AG	AG	AG	AA
	AG	AA	GG	GG	GG	AG	AG	AG
	AA	AG	GG	AG	AG	AG	AA	AG
	AG	AA	AA	AG	GG	GG	GG	GG
	AG	AG	GG	AG	AG	AG	GG	
59369235							
1166	rs10007590	4	AA	AA	AA	AG	AG

GG AA AA AA AA AA AA AA
AA AA AA AG AG AA AA AA
AG AG AG AA AG AG AG AG
AA AA AA AA AA AA AA
89917135
1167 rs10007601 4 GG AG AG AA AA
AA AA AA AA AA AA AA AG
AG AA AG AA AA AA AA AG
AA AA AA AA AA AG AA AG
AG AG GG GG AA AG AA
164934874
1168 rs10007616 4 AA AA AC AA AA
AA AA AC AC AC AA AC AA
AA AA AA AA AA AC AA AC
-- AA -- -- AC AC AC CC
AA -- -- -- AC AC --
59375444
1169 rs1000763 2 GG GG GG GG GG
GG GG GG GG GG AG AG AG
GG AG GG GG GG GG GG GG
GG GG GG GG GG GG GG GG
GG GG GG GG GG GG GG
118503220
1170 rs10007635 4 AG AG AA AG AG
AG AG AA AA AA AA AG AG
AG AG AA AA AA AG AG GG
AA AA AA AA GG AG AG GG
AG AG AG AG AA AA AA
24369452
1171 rs10007705 4 AG AA AA AA AA
AA AA AA AA AA AA AA AA
AA AA AA AA AA AG AG AA
AA AA AA AA AA AG AG AA
AA AA AA AA AA AA AA
171692801
1172 rs10007735 4 AG AG AG AG AG
AG GG AG AG AG AG AG AA
AA AG AA AG AG GG GG AG
AG AG AG AA AA AA AA AA
GG AA AG AA AA AG AG
6411979
1173 rs1000778 11 GG GG GG AA AA
AA AG GG GG GG AG AG GG
GG GG AG AG GG GG GG AG
AG GG AG AG AG GG AG GG
AG AG AG AA GG GG GG
61655305
1174 rs10007784 4 GG AG AA AG GG
AG GG GG GG GG AA AA AA
AA AG AA AA AA AG AG AG

AA AA AA AA AA AG AG AA
AA AG AA AA AG AG AA
81977690
1175 rs10007799 4 GG GG GG GG GG
GG GG GG GG GG AA AG AG
AG GG AG AG GG GG GG GG
GG GG GG GG GG GG GG GG
GG GG GG GG GG GG GG
32446724
1176 rs10007812 4 GG GG GG GG GG
GG GG GG GG GG GG GG GG
GG GG GG GG GG GG GG GG
GG GG GG GG GG GG GG GG
GG AG GG GG GG GG GG
31893113
1177 rs10007855
4 -- -- -- -- -- -- -- --
-- -- -- -- -- -- -- --
-- -- -- -- -- -- -- --
-- -- -- -- -- -- -- --
-- -- -- -- 131086557
1178 rs10007887 4 AG AG GG AG AG
GG AA GG GG GG GG AG AG
AG AA GG GG GG GG GG GG
AG AG AG GG AG GG GG GG
AG GG AG AG AG AG GG
10749071
1179 rs10007934 4 GG GG GG GG GG
GG GG GG GG AG GG GG GG
GG AG AG AG AG GG GG GG
GG GG GG GG GG GG GG GG
GG AG GG AG GG GG GG
137824601
1180 rs10007938 4 AG AG AG AG AA
AA AG GG GG AG AA AA AA
AA AA AG AA AG AA AA AG
AA AG AG AA AA AA AA AA
AA AA AG AA AG GG AA
32452973
1181 rs1000797 10 GG GG GG AG AG
AA GG GG GG GG GG GG GG
GG GG GG GG GG GG GG GG
GG GG GG GG GG AA AG AG
GG GG AG GG AG GG AG
129652787
1182 rs10008014 4 AA AA AA AA AA
AA AA AA AA AA AA AA AA
AA AA AA AA AA AA AA AA
AA AA AA AA AG AA AA AG
AA AA AG AG AA AA AA

47745012
1183 rs10008050 4 AA AA AA AA AA
AA AA AA AA AA AA AA AA
AA AA AA AA AA AG AA GG
AG AA AG AG AG AA AG AG
AA AA AA AA AG GG AG
153890646
1184 rs10008078 4 AG AA AA GG AG
GG AG AG AG GG GG GG GG
GG GG GG GG GG GG GG GG
GG GG GG GG GG AG AG GG
AG GG AG GG GG GG AG
155448553
1185 rs1000810 6 AG AG GG AG AA
AG AA AA AG AA AA AG AG
GG AG AG AG AA AA AA AA
AA AA AA AA AA AA AA AA
AA AG AG AG AG AG AA
52534931
1186 rs10008104 4 GG GG GG GG AG
GG AG GG GG GG AG AG AG
AG AG AG AG AA AG AG GG
GG GG GG GG GG AA AG AG
GG AG AA AG GG GG AG
25444762
1187 rs10008153 4 AG AG AG AA AG
AG AG AG AG AG GG GG GG
GG GG AG GG GG AA AA AA
AG AG AG AA GG AG AG GG
AA AG AG AG AG GG AA
185227184
1188 rs10008162 4 AA AA AG AA AA
AA AA AA AA AA AA AA AA
AA AA AG AA AA AA AA AG
AA AA AA AA AA AA AA AA
AG AA AG AA AA AG AG
164841877
1189 rs10008238 4 AA AG GG AA AA
AA AA AG AG GG AA AA AA
AA AG GG GG GG AG AG AA
AA AG AG AG AA AG AG AA
AG GG AG GG AG AA GG
87282216
1190 rs1000835 2 AG AG AG AA AA
AA AA AA AA AA AG AA AA
AA AA AG AG GG AA AA AG
AA AA AA AA AA AG AA AG
AG GG AA AG GG AG AG
26834387
1191 rs10008382 4 AG GG GG AG AG

AG AG AG AG AG AG GG GG
GG AG GG AG AG AG AG AG
GG GG GG GG GG AA AG AG
AG AG AG GG GG AG AG
7794135
1192 rs10008418 4 GG GG GG GG AG
AG GG GG GG GG GG GG GG
GG AG AG GG GG GG GG GG
GG GG GG GG GG GG GG GG
GG GG GG GG GG GG GG
108732508
1193 rs10008425 4 GG GG GG GG GG
GG GG GG GG GG GG GG GG
GG AG GG GG GG GG GG GG
GG GG GG GG AG GG GG AG
GG AA GG AG GG GG GG
58837610
1194 rs10008442 4 AG GG GG GG GG
GG GG AG AG AG GG AA AG
AG AG AA AA AA GG AG AG
AA AG AG AG GG GG GG GG
AG AG AG AG AG AG GG
101728821
1195 rs10008483 4 AC AC AA AC AC
AA CC CC CC AC CC CC AC
AC AC AA AA AA CC CC AC
CC CC CC CC CC AC AC AC
CC AC CC AC CC CC CC
40712481
1196 rs1000850 9 AA AA AA AG AA
AG AA AG AG AA AA AA AA
AA AA AA AG AG AA AA AA
AG AG AG AG AG AG AG AG
AG AG GG GG AG AG AA
96347718
1197 rs10008525 4 AA AA AA AA AA
AA AA AA AA AG AA AA AA
AA AG AG AA AG AA AA AA
AA AG AG AA AA AA AA AA
AA AG AA AG AA AA AG
30466859
1198 rs1000858 2 AG AG AG AG AG
AA AG GG GG GG GG GG GG
GG GG GG GG GG GG AA AG
AG AA GG GG GG GG GG GG
GG AA GG AG AG AG AG
127459321
1199 rs10008591 4 GG GG GG GG GG
GG GG GG GG GG GG GG GG
GG GG GG GG GG GG GG GG

GG GG GG GG GG GG GG GG
GG GG GG GG GG GG GG
132218117
1200 rs10008621 4 AG GG AG AG GG
AG AG GG AA AG GG GG GG
GG GG AG GG GG GG GG GG
GG GG GG GG GG GG GG GG
AG AG GG AG GG GG GG
187550697
1201 rs10008670 4 GG GG GG AG AG
GG AA GG AG AG AA AG AG
GG AG AG AG AA AG AG AG
AG AA AG AA AA GG AG AG
AG AG AG AG AA AG AA
68237962
1202 rs10008679 4 AG GG GG AG GG
AG GG AG AG AG AG AG AG
AG GG AG AA AG GG GG AG
GG AG AG AG AG AG AG GG
AG GG GG GG GG AG AG
24034708
1203 rs10008757 4 AG AA AG AG AA
AG AG GG AG AG AG GG GG
AG AG GG GG GG GG AG AG
GG AG AG GG AG AG AG AG
GG AG AA AA GG AG GG
76903036
1204 rs10008779 4 AC AC AC AA AC
AA AC CC CC CC AC AC AC
AC AC CC CC CC CC CC CC
AC AC AC AC CC AC AC CC
AA AC AA AC AA AA AC
168236901
1205 rs10008795 4 AG AG GG GG GG
GG GG GG GG AG GG GG AG
AG GG GG GG GG GG GG AG
GG GG GG GG GG GG GG GG
GG AG GG AG AG AA AG
147970022
1206 rs10008805 4 AA AA AG GG GG
AG AG GG GG AG AG AG GG
GG AG AA AA AA AA AA AA
GG GG GG GG AG AG AG GG
AG AA AG AA AG AG AA
138126978
1207 rs10008808 4 AG AG GG GG GG
GG GG GG GG GG GG AG GG
GG GG GG GG GG GG GG GG
GG AG AG GG GG GG GG GG
GG GG GG GG GG GG GG

23683303								
1208	rs10008894	4	AG	AG	AG	AG	AG	
	GG	AG	AA	AA	AG	AG	AA	AA
	AA	AG	AA	AA	AA	AG	AG	AA
	AA	AG	AG	AA	GG	AG	GG	AG
	AA	AG	GG	AG	AA	AA	AG	
86763097								
1209	rs10008923	4	AG	AG	AG	AA	AA	
	AA	AA	AA	AA	AA	AG	AG	AG
	AG	AA	AA	AA	AA	AA	AA	AA
	AA	AG	AA	AA	AG	AA	AA	AA
	AA	AA	AG	AG	AA	AA	AA	
13213672								
1210	rs10008933	4	GG	AG	GG	GG	GG	
	AG	GG	AG	AG	AA	AG	AG	AG
	AG	AG	GG	GG	GG	AG	GG	AG
	AG	AG	AG	AG	GG	AG	GG	AG
	AG	AG	GG	GG	AG	AG	GG	
125659601								
1211	rs10008999	4	GG	GG	GG	AA	AA	
	AA	AA	AG	AG	AA	AA	AA	AA
	AA	AA	GG	AG	AG	AA	AA	AG
	GG	AG	AG	GG	AG	AG	GG	AG
	AA	AA	AA	AA	AG	AA	GG	
24938446								
1212	rs10009007	4	GG	GG	GG	AG	AG	
	GG	AG	GG	GG	GG	GG	GG	AG
	AG	GG	GG	GG	GG	GG	GG	GG
	GG	GG	GG	GG	AG	GG	AG	AG
	GG	GG	GG	GG	AG	AG	GG	
141629826								
1213	rs10009018	4	AG	AG	AG	AA	AA	
	AA	AA	AA	GG	AG	AA	AA	AA
	AA	AA	AG	AG	AG	AA	AA	AA
	AA	AA	AG	AG	AA	AA	AA	AA
	AA	AA	AA	AA	AG	AA	AG	
186507021								
1214	rs10009030	4	AA	AC	AA	AC	AC	
	AC	AA	AC	AA	AA	CC	CC	CC
	CC	CC	AA	AC	AC	AC	AC	AA
	CC	CC	CC	CC	CC	CC	CC	CC
	AA	AC	CC	AC	CC	CC	CC	
187521037								
1215	rs10009048	4	AG	AG	AA	AG	AA	
	AG	AA	AG	AA	AA	GG	GG	GG
	AG	AG	AG	AG	GG	AG	AG	AA
	GG	AA	AA	AA	AA	AA	AA	AA
	AG	AA	AA	AA	GG	AG	GG	
74453782								
1216	rs10009064	4	AG	AG	GG	AG	GG	

GG AG GG GG GG GG GG GG
GG GG GG GG GG GG GG GG
GG GG GG GG GG AG GG AG
GG GG GG GG GG GG GG
155938012
1217 rs10009070 4 GG GG GG AG GG
AG GG GG GG GG GG GG GG
GG GG AG GG GG AG GG AG
GG AG AG GG AG GG GG AG
GG GG AG AG GG GG GG
111181565
1218 rs10009093 4 GG GG GG GG GG
AG GG GG GG GG AG GG GG
GG AG GG GG GG GG GG AG
GG GG GG GG AG GG GG GG
AG GG GG GG GG GG GG
143629934
1219 rs10009111 4 AG AA AG GG GG
AG GG GG AG GG AA AA AA
AA AG GG GG GG AG AG AA
GG GG GG AG AG GG AG AG
AG GG AG AG AG AG AG
10971423
1220 rs10009152 4 AG AG AA AA AA
AA AA AG AG AG AG AG GG
AG AG AG GG AG AA AA AG
GG GG GG AG AA AA AA AA
AG AG AA AA AA AA AA
2408397
1221 rs10009221 4 AG AG AA AA AA
AA AA AA AA AA AA AA AA
AA AA AA AA AA GG AG AG
AG AA AA AA AG AG GG AA
AG AA AA AA AA AA AA
66613120
1222 rs10009253 4 GG GG GG GG GG
GG GG GG GG GG GG GG GG
GG AG GG GG GG GG GG GG
GG GG GG GG AG GG AG AG
GG GG AG GG GG GG GG
11216238
1223 rs10009327 4 GG GG GG AG AA
AG AG GG GG GG GG GG AG
AG AG AA AG AA AG GG AG
GG GG GG GG AG AG AG AG
AA AA GG AG GG AG AG
98160778
1224 rs1000939 5 GG GG GG GG GG
AG GG AG AG AG AA AA AG
GG GG AG GG AG AG GG GG

GG GG GG GG GG GG GG GG
AG GG GG GG GG AG AG
172798489
1225 rs10009456 4 AC AC CC CC CC
CC AC AA AA AA CC CC AC
AC AC AA AA AA CC CC CC
AA AC AC AA CC AC CC AC
AC CC AA AC CC CC CC
136094675
1226 rs10009463 4 AA AA AA AA AA
AA AA AA AA AA AA AA AA
AA AA AA AA AA AA AA AA
AA AA AA AA AA AA AA AA
AA AA AA AA AA AA AA
110373682
1227 rs1000950 2 AC CC CC AC AC
AC AC AC AC AC AA AA AC
AA AA CC CC CC AC AC AC
AA AA AC AC CC AC AC CC
CC AC CC CC CC CC AC
119279230
1228 rs1000953 20 GG AG AG GG AG
GG AG AG GG AG AG AA AG
AG AG AG AA AA AA AA AG
AG AG AG AG GG AG AG AG
AG AG AG GG GG GG AG
10940119
1229 rs10009533 4 GG AG AG GG GG
GG GG AG AG AG GG GG GG
GG AG GG AG AG AG AG GG
GG GG GG GG AG GG AG GG
AG GG GG GG AG GG AG
57943382
1230 rs10009557 4 GG GG AG GG GG
GG GG GG GG GG AG GG GG
GG GG GG GG GG GG GG GG
GG GG GG GG GG AG GG AG
GG GG GG GG GG GG GG
131641319
1231 rs10009571 4 GG AG AG GG AG
AG GG AG AG AG GG GG GG
GG GG GG GG GG AG GG AG
AA AG AA AG AA GG AG AG
AG AG AG AG GG GG GG
65940796
1232 rs10009574 4 GG AG AG AG AG
AA AG AA AA AG AA AA AA
AA AA AG AA AG AA AA AA
AA AG AG AG AA AA AA AA
AG AG AA AA AA AA AA

91989547
1233 rs1000960 11 AG AG AA AG GG
GG AG AA GG GG AG AG AG
AG AG AG AG GG AG AG AA
AG AA GG AG AA AG AG AA
AA AA AA AA AG AA AG
79376133
1234 rs10009607 4 AC AC AA AA AA
AA AA AA AA AA AC AC AA
AA AC AC AA AA AA AA AA
AA AA AA AA AA AA AA AA
AA AA AA AA AC AC AC
170212887
1235 rs10009611 4 AC AC CC CC CC
CC AC AC AC AC CC CC CC
CC CC AA AA AA CC CC AC
AC CC CC AC CC CC CC CC
CC AC AC AA AA AC AA
58263866
1236 rs10009617 4 GG AG AG GG GG
GG GG GG GG GG AA AA AG
AG AG AG AG AG AG AG GG
AG AG AG GG GG AG GG AG
GG AG GG AG AG GG AG
182874271
1237 rs10009620 4 GG GG GG AG AG
AA GG AG AG AG GG AG AG
AG AG AA AG AG AG AG AG
GG AG AG GG AG AG GG AG
AA GG GG GG GG AG GG
117724087
1238 rs10009701 4 AG AG GG AG AG
GG AA AG AA AG AG AG AG
GG AG AG GG GG AG AG AG
GG AG AG AA GG AA AG AG
AG GG AG GG AG AA GG
177045953
1239 rs10009706 4 AA AA AA AG AA
AG AG AG AG AA AA AG AA
AA GG AG AG AA AA AA AA
AA AA AA GG AG AA AA AA
AA AG AA GG GG AG GG
38079475
1240 rs10009773 4 GG AG AG GG GG
AG AG AG GG AG AG AG AA
AG GG GG GG GG GG GG GG
GG GG GG AG GG GG GG GG
GG GG GG GG AG AA GG
4727223
1241 rs10009801 4 AA AA AA GG AG

AG GG AG AG AG AG AG AG
GG AG AG AG GG AG AA GG
AG AA AA AA AG AA AA AG
AG AG AG AG AA AA AA
75694208
1242 rs10009826 4 AG AG AA AA AA
AA AA AA AA AA AA AA AG
AA AA AG AG GG GG GG AG
GG AG AG AA AA GG AG AG
GG GG AA AG AG AG AG
53295856
1243 rs10009833 4 GG GG GG GG GG
GG GG GG GG GG GG GG GG
GG GG AG GG AG GG GG GG
AG AG AG GG AG GG AG AG
GG AG GG GG GG GG GG
8467375
1244 rs10009898 4 AA AA AA GG AG
AG GG AA AA AA AA AA AG
AG AA AA AA AA AA AA AG
AG AG AG AG AG AA AG AG
AG GG AG GG AG AG AA
27548905
1245 rs1000991 14 GG GG GG GG GG
GG GG GG GG GG GG GG GG
GG GG GG GG GG GG GG GG
GG GG GG GG GG GG GG GG
GG GG GG GG GG GG GG
57886132
1246 rs10009981 4 AG GG AG AA AG
AG AA AG GG GG GG AG AG
AG AG AA AA AA AA AG AA
AA GG GG AG AA GG AG AG
AA AG AA AG AA AA AA
78598313
1247 rs10009982 4 AA AA AA AA AA
AA AA AA AA AA AA AA AA
AA AA AA AA AA AA AA AA
AA AA AA AA AA AA AA AA
AA AA AA AA AA AA AA
43165682
1248 rs10009998 4 AA AA AA AA AA
AA AA AA AA AC AA AC AA
AA AC AA AA AA AC AA AC
AC AC AC AC AA AA AA AA
AA AC AC AC AA AA AA
142762276
1249 rs10010076 4 AA AA AA AA AA
AA AA AC AC AA AA AA AA
AA AA AA AA AA AA AA AA

	AA	AA	AA	AA	AC	AA	AA	AC
	AA	AC	AA	AA	AC	AA	AC	
53044281								
1250	rs10010080		4	AA	AA	AA	AA	AA
	AA	AA	AA	AA	AA	AA	AA	AA
	AA	AA	AA	AA	AA	AA	AA	AA
	AA	AA	AA	AA	AA	AA	AA	AA
	AA	AA	AA	AA	AA	AA	AA	
47449917								
1251	rs10010106		4	AA	AA	AA	AG	AG
	AA	GG	AG	AG	AG	AG	AA	AG
	AG	AA	AG	AA	AA	AA	AA	AA
	GG	AG	AG	GG	GG	AG	GG	AG
	AA	GG	AG	AG	AA	AA	AA	
108525128								
1252	rs10010115		4	AA	AA	AA	AA	AA
	AA	AA	AA	AA	AA	AA	AA	AA
	AA	AA	AA	AG	AA	AA	AA	AA
	AA	AA	AA	AA	AA	AA	AA	AA
	AA	AA	AA	AA	AA	AA	AA	
99698254								
1253	rs10010167		4	GG	GG	GG	AG	AG
	GG	AG	GG	GG	GG	AG	GG	AG
	AG	GG	GG	GG	GG	AG	GG	AG
	GG	AG	AG	AG	GG	GG	GG	GG
	GG	GG	GG	GG	AG	AG	AG	
111051486								
1254	rs10010198		4	AA	AA	AA	AA	AA
	AA	AA	AA	AA	AG	AG	AA	AG
	AG	AG	GG	AG	AG	AA	AA	AA
	AA	AA	AA	AA	AG	AA	AA	AA
	AA	AG	AG	AA	AA	AA	AA	
36573898								
1255	rs10010217		4	GG	AG	AA	AA	AA
	AA	AA	AG	AG	AG	AA	AA	AA
	AA	AA	AG	AA	AG	AA	AA	AA
	AG	AG	AG	AG	AG	AA	AA	AG
	AA	AA	AA	AA	AA	AA	AG	
80801911								
1256	rs1001026		7	AG	GG	AG	GG	GG
	GG	GG	AG	AG	AG	GG	GG	GG
	GG	GG	AA	AG	AA	AG	AA	GG
	GG	AG	AG	GG	AG	AG	AG	AA
	AA	GG	AG	AG	AG	AG	AG	
22932736								
1257	rs10010285		4	AG	GG	AG	AG	AG
	GG	AG	AG	AG	AA	AG	GG	GG
	GG	AA	AA	AG	AG	AA	AA	AA
	AG	AA	AA	GG	AG	AA	AG	AG
	AA	AG	AG	AG	AA	AA	AG	

36011362								
1258	rs10010306		4	CC	CC	CC	CC	CC
	CC	CC	CC	CC	CC	CC	CC	CC
	CC	CC	CC	CC	CC	CC	CC	CC
	CC	CC	CC	CC	CC	CC	CC	CC
	CC	CC	CC	CC	CC	CC	CC	
53463803								
1259	rs10010358		4	AA	AC	AC	AC	AC
	AC	CC	AC	AC	AC	CC	CC	CC
	CC	CC	AC	AC	CC	CC	CC	AC
	CC	CC	CC	CC	AC	AC	AC	AA
	CC	CC	CC	CC	CC	CC	CC	
5405805								
1260	rs10010423							
4	--	--	--	--	--	--	--	--
	--	--	--	--	--	--	--	--
	--	--	--	--	--	--	--	--
	--	--	--	--	--	--	--	--
	--	--	--	--	91475787			
1261	rs1001049		15	AC	AC	CC	CC	AC
	AC	AC	AA	AA	AA	AC	AA	AC
	AC	AA	AA	AC	AC	AC	AC	AA
	AC	CC	CC	AC	AA	AC	AC	AA
	AC	AC	AA	AC	AA	AC	AA	
94682435								
1262	rs10010509		4	CC	CC	CC	CC	CC
	CC	CC	CC	CC	CC	AC	AC	AC
	AC	CC	CC	AC	AC	CC	CC	CC
	CC	AC	AC	AC	CC	CC	CC	CC
	AC	AC	CC	CC	CC	CC	AC	
55157206								
1263	rs10010565		4	CC	CC	CC	CC	CC
	CC	CC	CC	CC	CC	CC	AC	CC
	CC	AA	CC	CC	CC	CC	CC	CC
	CC	CC	CC	CC	CC	CC	CC	CC
	CC	CC	CC	CC	CC	CC	CC	
153590063								
1264	rs10010598		4	GG	GG	GG	GG	GG
	GG	GG	GG	GG	GG	GG	GG	GG
	GG	GG	GG	GG	GG	GG	GG	GG
	GG	GG	GG	GG	GG	GG	GG	GG
	GG	GG	GG	GG	GG	GG	GG	
640165								
1265	rs10010638		4	CC	AC	AC	CC	CC
	CC	CC	CC	CC	CC	CC	CC	CC
	CC	CC	CC	CC	CC	CC	CC	CC
	CC	CC	CC	CC	CC	CC	CC	CC
	CC	CC	CC	CC	CC	CC	CC	
162273772								
1266	rs10010746		4	GG	GG	GG	GG	GG

GG GG GG GG GG GG GG GG
GG GG GG GG GG GG GG GG
GG GG GG GG GG GG GG GG
GG GG GG GG GG GG GG
11264261
1267 rs10010935 4 AG AG AG AA AA
AA AA AG AG AG AG AA AA
AA AA AA AA AA AA AA AA
GG AG AG GG AG AA AG AA
AA AA AA AA AA AA AA
114741032
1268 rs10011039 4 AC AA AA CC CC
CC CC AC AA AA AA AA AC
AC AC AC CC AC AA AA AC
CC CC CC AC AC AC AA AC
AC CC CC CC AC AA AC
59636452
1269 rs10011041 4 AG AG GG AA AA
AA AA AG AG GG AG AG GG
GG AA GG GG GG GG AG GG
AA AG AG AG AG AG GG AG
AA GG GG GG AG AG AG
161666367
1270 rs10011158 4 GG GG GG GG GG
GG AG AG AG GG GG GG GG
GG AG GG GG GG GG GG GG
GG AG AG GG GG GG GG GG
GG AG GG GG GG AG AG
95056300
1271 rs10011174 4 GG GG AG AG AG
AG GG AG AG AG AG AG GG
GG AG AG GG GG AG GG AG
GG GG GG GG AG GG AG AG
AG GG GG GG AA AA AG
153495515
1272 rs10011190 4 GG GG GG GG GG
GG GG GG GG GG GG GG GG
GG GG GG GG GG GG GG GG
GG GG GG GG GG GG GG GG
GG GG GG GG GG GG GG
43648726
1273 rs1001120 5 GG GG GG AG AG
AG GG GG GG GG GG GG GG
GG GG AG AG GG GG GG AG
GG GG GG GG GG GG GG GG
GG GG AG AG GG GG GG
175249549
1274 rs10011202 4 AA AA AA AA AA
AA AG AA AA AA AA AA AA
AA AA AG AA AA AA AG AA

	AA	AG	AG	AA	AG	AA	AG	AA
	AA	AA	AA	AA	AA	AA	AA	
104396376								
1275	rs10011263		4	AG	AG	AG	GG	AG
	AG	AG	AG	AG	GG	GG	GG	GG
	GG	GG	GG	GG	AG	GG	GG	GG
	GG	GG	GG	GG	AG	GG	GG	AG
	AG	AG	GG	AG	GG	GG	AG	
113728993								
1276	rs1001131		2	AA	AA	AA	AA	AA
	AA	AA	AA	AA	AA	AA	AA	AA
	AA	AA	AA	AA	AA	AA	AA	AA
	AA	AA	AA	AA	AA	AA	AA	AA
	AA	AA	AA	AA	AA	AG	AA	
115010406								
1277	rs10011310		4	AG	--	--	GG	--
	AG	AG	--	--	AA	--	AA	--
	AG	AG	--	--	--	AG	AG	AG
	--	--	--	AG	--	--	--	--
	AG	--	--	--	AG	--	AG	
2784045								
1278	rs10011402		4	CC	CC	CC	CC	CC
	CC	CC	CC	CC	CC	CC	CC	CC
	CC	CC	AA	AC	AC	CC	CC	CC
	AA	AC	AC	AC	AC	CC	CC	CC
	CC	AC	AC	CC	CC	AC	CC	
19488871								
1279	rs10011465		4	AG	AG	AG	AG	--
	--	AG	AG	AG	AG	AG	AG	--
	--	AG	AG	AG	AG	AG	AG	AG
	AG	AG	AG	--	--	--	AG	AG
	AG	AG	AG	AG	AG	AG	AG	
57699866								
1280	rs1001148		7	AA	AA	AA	GG	AA
	AG	AG	AG	GG	AG	GG	GG	GG
	AG	AG	AG	AG	AA	AG	AG	GG
	AG	AA	AA	AG	AA	GG	AG	AG
	GG	AG	AA	AA	AG	GG	AG	
51319875								
1281	rs10011584		4	CC	CC	CC	AA	AA
	AC	AC	CC	CC	CC	CC	AC	CC
	CC	CC	CC	AC	AC	CC	CC	AC
	CC	CC	CC	CC	AC	CC	AC	AC
	CC	CC	CC	CC	CC	AC	CC	
127213903								
1282	rs10011590							
4	--	--	--	--	--	--	--	--
	--	--	--	--	--	--	--	--
	--	--	--	--	--	--	--	--
	--	--	--	--	--	--	--	--

-- -- -- -- 124473935
1283 rs1001162 3 AC AA AC AA AA
AA AA AA AA AA AA AC AC
AA AA AC AA AC CC CC AC
AA AA AA AA AA AA AA AA
AA AA AC AA AA AA AC
137533519
1284 rs10011670 4 AG GG AG GG GG
AG GG AG AG AA AG AA AG
AG GG GG GG GG AA AA AA
AG AG AG GG AG GG GG GG
AG GG AG AG AG AA GG
21140045
1285 rs10011676 4 AA AG AG AA AA
AA AA AA AA GG AA AA AA
AA AA AG AA AG AA AA AA
AA AG AG AA AA AA AA AA
AA AA AA AA AG AG AG
98292053
1286 rs10011687 4 AA AA AA GG AG
AG AG AA AA AG AG AA AA
AA AA AA AA AA AA AA AG
AA AA AA AA AA AG AG AA
AA AA AA AA AA AA AA
154474250
1287 rs1001170 16 AC CC AC AA AC
AC AA CC AC CC CC AC AC
AA AC CC CC AC AC CC AC
AC AA AC AC AC AC CC AC
AA AC CC CC AC AA AC
5082231
1288 rs10011765 4 AA AA AG AA AA
AA AA AA AA AA AA AA AA
AA AA AA AA AA AA AA AA
AA AA AA AA AA AA AA AA
AA AA AG AG AA AA AA
95335021
1289 rs10011769 4 CC CC CC CC CC
CC CC CC CC CC AC CC CC
CC CC CC CC CC CC CC AC
CC CC CC AC CC AC CC AC
AC AC CC CC CC CC AC
5135979
1290 rs10011796 4 AG AG AG GG GG
GG AG AG AG AG AG AA AA
AA AA AG AG AA AA AA AG
GG AG AG AG AG GG AG GG
AA AG GG GG GG AG AG
89090877
1291 rs10011837 4 GG GG GG GG GG

GG GG GG GG GG GG GG GG
GG AG GG GG GG GG GG GG
AA AA AA AG GG AG GG AG
GG GG GG GG GG GG GG
5752753
1292 rs10011842 4 GG GG GG GG GG
AG GG AG AG AG AG GG GG
GG AG AG AG GG GG GG GG
GG GG GG GG AG AG AG AA
AG AG GG GG GG AG GG
7341243
1293 rs10011860 4 CC CC CC CC CC
CC CC CC CC CC CC CC CC
CC CC CC CC CC CC CC CC
CC CC CC CC CC CC CC CC
CC CC CC CC CC CC CC
154835465
1294 rs10011864 4 GG AG AA AA AG
AG AA GG GG GG GG GG GG
GG AG AG AA AG AG AG AA
GG GG GG GG AG GG GG GG
AG AA AG AG GG GG AG
32143073
1295 rs1001190 3 AA AA AA GG AG
GG AG AA AG AG AA AA AA
AA AA AG AG AG AG GG AA
AA AA AA AA AG AG AG AG
AG AG GG GG AA AA AA
183210972
1296 rs10011912 4 AG AG GG AG GG
AG AG GG GG GG GG GG GG
GG GG AG AG AA GG GG GG
AG GG AG GG AA AG AG AA
GG AG AG AG AG AA GG
70480327
1297 rs10011916 4 GG GG GG GG GG
GG GG GG AG GG AG AG GG
GG AG GG GG GG AG AG GG
GG GG GG GG GG AG AG GG
GG GG GG GG GG AG GG
178711450
1298 rs10011926 4 AG AA AG AG AG
GG AA AG AG AG GG GG GG
GG GG GG AG GG AA AG AG
AG GG GG GG GG GG GG GG
AA AG AG AA GG GG AG
111026927
1299 rs10011933 4 AA AG AA AA AA
AG AA AA AG AA AA AA AA
AG AA AA AA AA GG GG AG

	AG	AG	AG	AA	AG	GG	AG	AG
	AG	AG	AA	AA	GG	AG	AG	
185127748								
1300	rs1001194		12	GG	GG	GG	GG	GG
	GG	GG	GG	GG	GG	GG	GG	GG
	GG	GG	GG	GG	GG	GG	GG	GG
	GG	GG	GG	GG	GG	GG	GG	GG
	GG	GG	GG	GG	GG	GG	GG	
111722196								
1301	rs10011961		4	GG	GG	AG	GG	GG
	GG	GG	GG	GG	AG	GG	GG	GG
	GG	GG	GG	GG	GG	GG	GG	GG
	GG	GG	GG	GG	AG	AG	AG	AG
	GG	GG	GG	GG	GG	GG	GG	
102641428								
1302	rs10011990		4	GG	GG	GG	GG	GG
	GG	GG	AG	AG	GG	GG	GG	GG
	GG	GG	GG	GG	GG	GG	GG	GG
	GG	GG	GG	GG	AG	GG	GG	AG
	GG	AG	GG	GG	AG	GG	AG	
52962529								
1303	rs10011993		4	AA	AC	AC	AA	AC
	AC	AA	AC	CC	CC	AA	AA	AA
	AC	AC	AC	AA	AC	CC	AC	AC
	CC	AA	AC	AA	AC	AC	CC	AA
	AC	AC	AA	AC	AA	AA	AA	
67093452								
1304	rs10012098		4	GG	GG	GG	GG	GG
	GG	GG	GG	GG	GG	GG	GG	GG
	GG	GG	GG	GG	GG	GG	GG	GG
	GG	GG	GG	GG	GG	GG	GG	GG
	GG	GG	GG	GG	GG	GG	GG	
116420387								
1305	rs10012124		4	AA	AA	AA	AA	AA
	AA	AA	AA	AA	AA	AG	AG	AG
	AG	AA	AG	AG	AA	AA	AA	AG
	AA	AA	AA	AA	AA	AA	AA	AA
	AA	AA	AG	AA	AA	AG	AA	
158279954								
1306	rs10012134		4	GG	GG	AG	GG	GG
	GG	GG	GG	GG	GG	GG	GG	GG
	GG	GG	AG	AG	GG	GG	GG	AG
	GG	GG	GG	GG	AG	GG	AG	GG
	GG	AG	GG	GG	GG	GG	GG	
96314856								
1307	rs10012138		4	AG	AG	AA	AG	AG
	GG	AA	GG	GG	AG	GG	GG	GG
	GG	AG	GG	GG	GG	AG	AG	GG
	AA	AG	AA	AA	AG	AG	GG	AG
	AG	GG	AA	AG	GG	AG	GG	

141343133								
1308	rs1001216		4	AA	AA	AA	AA	AA
	AA	AA	AA	AG	AA	AG	AG	AG
	AG	AA	AA	AA	AA	AA	AA	AA
	AA	AA	AA	AA	AA	AA	AA	AA
	AG	AA	AA	AA	AG	AG	AA	
10168849								
1309	rs10012191		4	GG	GG	GG	AG	AG
	GG	AG	GG	GG	AG	AG	GG	GG
	AG	AG	GG	GG	GG	GG	AG	GG
	AG	AG	AG	GG	GG	GG	GG	GG
	GG	AG	AG	AA	GG	GG	AG	
91847796								
1310	rs10012193		4	AC	AC	AC	AC	AC
	AA	CC	CC	AC	CC	CC	AC	CC
	CC	AC	AA	AA	AA	CC	CC	AC
	CC	CC	CC	CC	AA	AC	AA	AC
	CC	AC	CC	AC	AC	CC	AA	
138197514								
1311	rs1001220		7	AG	AA	AG	AG	AG
	AA	AG	GG	GG	AG	AA	AA	AA
	AA	AA	GG	GG	GG	AA	AA	AA
	AA	AA	AA	AA	AG	AG	AA	AG
	AG	AA	AG	AA	AA	AA	AG	
73110603								
1312	rs10012213		4	AA	AA	AA	AA	AA
	AA	AA	AA	AA	AA	AA	AA	AA
	AA	AA	AA	AA	AA	AA	AA	AA
	AA	AA	AA	AA	AA	AA	AA	AA
	AA	AA	AA	AA	AA	AA	AA	
163006849								
1313	rs10012250		4	AG	AA	AA	AA	AA
	AA	AA	AA	AA	AA	AA	AA	AA
	AA	AA	AA	AA	AA	AA	AA	AA
	AA	AA	AA	AA	AA	AA	AA	AA
	AA	AA	AA	AA	AA	AA	AA	
34763475								
1314	rs10012274		4	GG	GG	AG	AA	AA
	AG	AA	AA	AG	AA	GG	GG	GG
	GG	AG	AG	AA	AG	GG	GG	GG
	AA	AA	AA	AG	GG	AG	AG	GG
	GG	AG	AG	AG	GG	AG	GG	
10981163								
1315	rs10012289		4	AG	AG	GG	AA	AA
	AA	AA	AG	AG	GG	AG	GG	GG
	GG	AG	AG	AA	AG	AG	AG	AG
	GG	GG	GG	GG	GG	GG	GG	GG
	AG	AA	GG	AG	AG	AA	AG	
38425619								
1316	rs10012313		4	GG	AG	GG	AA	AG

AG AG GG GG GG GG AG GG
GG GG AG GG AG GG GG GG
AG GG AG AG AG AG AG GG
GG GG GG GG AA AG AG
17405513
1317 rs10012328 4 GG GG GG AG AG
GG AG AG AG AG GG GG GG
GG GG GG GG GG GG GG GG
AG AG AG GG GG AG AG AG
GG GG GG GG GG GG GG
54277319
1318 rs10012329 4 AG AG GG AG AG
AG AG AG AA AG AG AG GG
GG AG AA AA AA AG AG AG
AG AG AG AA AG AG AG GG
AG GG GG GG AG AA AG
182740613
1319 rs10012381 4 AA AA AA AA AA
AA AA AA AA AA AA AA AA
AA AA AA AA AA AA AA AA
AA AA AA AA AA AA AA AA
AA AA AA AA AA AA AA
53362564
1320 rs10012388 4 AA AA AA AG AA
AA AG AG AG GG GG GG GG
GG AG AG AG AA AG AA AG
AG AG AG AG AA AA AA AA
AG AA GG AG AG AA AG
157662111
1321 rs10012537 4 AC AC CC CC CC
CC CC CC CC CC AC AC CC
AC CC CC AC AC CC CC CC
CC CC CC AC CC AA AC AC
CC AC CC CC AC AA CC
183381596
1322 rs10012577 4 AA AA AA AA AA
AA AA AA AG AA AA AA AA
AA AA AA AA AA AA AA AA
AA AA AA AA AG AA AG AG
AA AA AA AA AG AA AG
186522043
1323 rs1001262 5 GG AG AG AG AG
AG GG AG AA AA AG AG GG
AG AG AG AG AA AG AG AG
AA AA AA AA AA AA AA AA
AA AG AA AA GG GG AG
179807626
1324 rs10012647 4 AG GG GG GG GG
GG GG GG GG GG GG GG GG
GG GG GG GG GG GG GG GG

GG GG GG GG GG GG GG GG
GG GG GG GG GG GG GG
114856627
1325 rs10012720 4 AG AG AG GG AG
AG AG AG AG AG GG AG GG
GG AA AG AG AG AG AA AG
GG GG GG GG AG AG AG AA
AA AA AG AA AA AA AG
151772410
1326 rs10012738 4 AA AA AA AA AA
AA AA AA AA AA AA AA AA
AA AA AA AA AA AA AA AA
AA AA AA AA AA AA AA AA
AA AA AA AA AA AA AA
112580704
1327 rs10012946 4 AA AG GG AG AG
GG AA GG GG GG AG AA AA
AA AG GG AG AG GG GG GG
AA AA AA AG GG AG GG AG
GG AG AG AA AG AG GG
6293350
1328 rs10012953 4 AA AA AA AG AG
AA AG AA AA AA AA AA AA
AG AG AA AG AA AG AA GG
AA AA AA AA AG AA AG AA
AA AA AA AA AA AA AA
110964362
1329 rs1001298 12 AA AG AG AG GG
GG AG AA AG AG AA AA AA
AG AA AG AA -- AG -- AG
AA AA AA AA AG AA AG AG
AG GG AG GG AA AA AG
108846917
1330 rs10013019 4 AA AA AG AG AG
GG AA AA AA AA AA AG AG
AG AA AA AA AA AG AG AA
AA AG AA AG AA AG AG AA
AG AG GG AG AA AA AA
67439729
1331 rs10013021 4 AG GG -- AG GG
AG GG GG GG GG AG AG AG
AG AG AG AG GG AG AG AG
GG AG AG AG AA AG AG AG
GG GG AG GG GG GG GG
58060147
1332 rs10013026 4 GG GG GG GG GG
GG GG AG AG GG GG GG GG
GG GG GG AG GG GG GG GG
AG GG GG GG GG GG GG GG
GG GG GG GG GG GG AG

104532648								
1333	rs10013053		4	AA	AG	AA	AG	GG
	AG	AG	AG	AG	AG	GG	GG	GG
	AG	AA	AG	GG	AG	AG	AG	AG
	GG	AA	AA	AG	AA	GG	AG	AG
	AA	GG	AA	AG	GG	AG	AG	
105400344								
1334	rs10013055		4	AG	AG	AA	AA	AA
	AG	AA	AG	AG	GG	AG	AG	AA
	AA	AA	GG	AG	AG	GG	GG	AG
	AG	AG	AG	AG	AG	GG	GG	GG
	AG	AA	AG	AG	GG	GG	AG	
21188231								
1335	rs1001310		6	AA	AA	AA	AG	AG
	GG	AG	AG	AA	GG	GG	GG	GG
	GG	GG	AG	AA	AG	AG	AG	AG
	AA	AA	AA	AA	AG	GG	AG	GG
	AG	AG	AG	GG	GG	AG	AG	
21065787								
1336	rs10013118		4	AA	AC	CC	AA	AA
	AA	AA	AC	AA	AC	CC	--	AC
	AC	AC	AA	AA	AA	AA	AA	AA
	AC	AC	AC	AC	AA	AC	AA	AC
	AC	--	AC	AC	AA	AA	AA	
182711326								
1337	rs10013228		4	AG	AG	AG	AG	AG
	AA	AG	AG	AG	AG	AA	AA	AA
	AA	AA	AG	AG	AA	AA	AA	AA
	AA	AG	AG	AG	AA	AA	AA	AA
	AG	AA	AA	AA	AG	GG	AG	
55997340								
1338	rs10013239		4	AC	AA	AA	AC	AC
	AC	AC	AC	AC	AC	AC	CC	CC
	CC	AA	CC	AC	AC	AA	AA	AA
	AC	AC	AC	AC	AC	AC	AC	AA
	AA	AC	AC	AC	CC	AC	CC	
7680962								
1339	rs10013273		4	GG	AG	AG	GG	GG
	GG	AG	AG	GG	AG	GG	GG	AG
	GG	GG	GG	GG	GG	GG	GG	GG
	GG	GG	GG	AG	GG	GG	GG	GG
	GG	GG	GG	GG	AG	AA	GG	
4721965								
1340	rs10013416		4	AA	AA	AA	AA	AA
	AA	AA	AA	AA	AA	GG	AG	AG
	AG	AG	AA	AA	AA	AA	AA	AA
	AA	AA	AA	AA	AG	AA	AG	AA
	AA	AG	AA	AA	AA	AG	AG	
58900069								
1341	rs10013424		4	AG	AG	AG	AA	AA

AA AG AG AG AG GG AG GG
GG GG AA AA AA AG AA AG
GG AG AG GG AA AA AA AA
AG AG AA AG AG AG AG
126694369
1342 rs10013579 4 AA AA AG AA AA
AA AA AG AG AG AG AA AA
AA AG AG AG AA AG AG AG
AG AG AG AG AA AG AA AA
AA GG AA AG AA AA AA
63540378
1343 rs10013589 4 AA AA AA AA AA
AA AA AA AA AA AG AG AG
AG AA AA AG AG AA AA AA
AA AG AG AA AA GG AG AG
AA AA AA AA AA AA AG
42583029
1344 rs1001362 16 GG GG GG GG GG
GG GG GG GG GG GG GG GG
GG GG GG AG GG AG AG AG
GG AG GG GG AG AG GG AG
GG GG GG GG GG GG GG
56674858
1345 rs1001366 16 AA AA AA AA AA
AA AA AG AA AG AA AA AA
AA AA AA AA AA AA AA AA
AA AA AA AA AA AA AA AA
AG AA AA AA AA AA AG
910950
1346 rs10013728 4 AA AA AA AG AG
AG AG AG AG GG AG AA AA
AA AG AA AA AA AA AA AA
GG AG GG GG AA AG AG AA
AA AA AA AA AG AA AG
146173197
1347 rs1001373 1 AG GG AG GG AG
GG AG GG GG GG GG GG AG
AG GG AG AG AG AG AG AA
GG AG GG AG AG GG AG GG
GG GG GG GG AG AG GG
171684506
1348 rs10013756 4 AG AA AA GG AA
AG AG GG AA GG AA AA AA
AA AA AG AA AA AG AG AG
AG AG AG AA AG GG AG AG
GG AG AA AA AA AG AA
181310122
1349 rs10013783 4 AG AA AA AA AG
AG AA AG AG AG AA AA AA
AA AA AG AA AG AG AG GG

	GG	GG	GG	AG	AA	GG	AG	AG
	AG	AG	GG	AG	AA	AG	AG	
38107811								
1350	rs10013817		4	AA	AG	AG	GG	GG
	AG	AG	GG	GG	GG	AG	AA	AG
	AG	AG	GG	GG	GG	GG	GG	GG
	AG	AG	AG	AG	GG	AG	AG	GG
	GG	GG	AG	AG	GG	AG	GG	
137259828								
1351	rs10013818		4	AG	AG	AG	AG	AG
	AA	GG	GG	GG	GG	AG	AG	GG
	GG	AG	AG	AG	GG	GG	GG	GG
	AG	GG	GG	AG	GG	GG	GG	GG
	GG	AG	AA	AA	GG	GG	GG	
44293409								
1352	rs1001389		9	CC	CC	CC	AC	CC
	CC	AC	AA	CC	AC	AC	AC	AC
	AA	AC	AC	AC	CC	AC	AC	AA
	CC	CC	CC	CC	AC	AA	AC	AA
	AC	AC	AC	AA	AC	AC	AC	
3584543								
1353	rs10013963		4	GG	GG	AG	GG	GG
	GG	GG	AG	AG	AG	GG	AG	AG
	AG	AA	AG	GG	GG	GG	GG	GG
	GG	GG	GG	GG	GG	GG	GG	GG
	GG	GG	GG	GG	GG	GG	GG	
118191882								
1354	rs10013975		4	AG	AA	AA	AA	AG
	AG	AA	AA	AG	AA	AG	AG	AA
	AA	AG	AG	AG	AG	GG	GG	AG
	GG	AG	AG	AG	GG	AG	GG	AG
	AA	AA	AG	AG	AG	AA	AG	
177146627								
1355	rs10014020		4	CC	CC	AC	CC	AC
	AC	CC	CC	CC	CC	CC	CC	CC
	CC	CC	CC	CC	CC	AC	AC	AC
	CC	CC	CC	CC	CC	CC	CC	CC
	CC	CC	CC	CC	CC	CC	CC	
56596137								
1356	rs10014124		4	AA	AG	AA	AG	AG
	AA	GG	AA	AA	AA	AA	AG	AA
	AA	AA	AG	AA	AA	AA	AA	AG
	AA	AG	AG	AA	AA	AA	AA	AA
	AA	AG	AA	AG	AG	GG	AA	
118408638								
1357	rs10014145		4	AA	AA	AG	AG	AG
	GG	AG	AG	AG	AG	AA	GG	AG
	AA	AA	AA	AG	AG	AA	AA	AG
	AA	AA	AA	AA	AG	AA	AG	AA
	AG	AA	AG	AA	AG	GG	AA	

103200577
1358 rs10014285 4 GG GG GG GG GG
GG GG GG GG GG AG GG GG
GG GG GG GG GG AG GG AG
AG AG AG AG AG GG GG GG
GG GG GG GG GG GG GG
161307972
1359 rs10014286 4 AG AA AA AA AA
AA AA AG AG AA AA AA AA
AA AG AA AA AA AG AG AG
AA AA AA AA AA AG AA AG
AA AG AA AA AA AA AA
166089111
1360 rs10014328 4 AG GG GG AG AG
AA GG GG GG AG GG GG GG
GG GG AG GG AG AG AG AG
AG GG GG AG GG GG GG GG
GG AG GG GG AG AG AG
21647862
1361 rs10014387 4 AG AG GG AG GG
AG GG GG GG GG AG AG AA
AA AG GG GG GG GG GG GG
AG AA AA AG AG GG GG GG
GG GG GG GG AG AA GG
172406840
1362 rs10014396 4 AA AA AA AA AA
AA AA AA AA AA AA AA AA
AA AA AA AG AG AA AA AA
AA AA AA AA AG AA AA AG
AA AA AA AA AA AA AG
90712629
1363 rs10014397 4 AG AA AA GG GG
AG GG AA AA AA AG AA AA
AG GG AA AG AG AG AG AA
AA AA AA AA AG AG AG GG
AA AG AG GG AA AG AA
5988237
1364 rs10014431 4 AG AG AG AG AG
AA AG AA AA AA AA AG AG
AG AG AA AG AG AG AG AG
AA AA AA AA AG AG AA AG
AG AG GG GG AG AG AG
48312390
1365 rs10014445 4 AA AG GG GG GG
AG GG GG AG GG AG AG GG
GG AG GG GG GG GG GG GG
GG AG GG AG GG AG AG GG
AG GG GG GG GG AG AG
15181885
1366 rs10014500 4 GG GG GG AG AG

AG GG GG GG GG AG GG GG
GG AG AG AG AG GG GG AG
AG GG AG AG GG AG GG AG
AG AG GG AG AG GG AG
155420487
1367 rs10014516 4 AG AA AA AA AG
AG AA AA AA AA AA AA AA
AA AA AA AG AG AG AG AA
AA AA AA AA AG AA AA AA
AA AA AG AG AG AG AA
179594357
1368 rs10014575 4 CC CC CC CC CC
CC CC CC CC CC CC AC CC
CC CC CC CC CC CC CC CC
CC CC CC CC AC CC AC AC
AC AC CC CC CC CC CC
130308015
1369 rs1001469 22 AG AG GG GG AG
AG AG GG GG GG GG GG GG
GG GG GG GG GG AG AG GG
AG AG AG AG GG GG GG GG
GG GG GG GG GG AG GG
51138753
1370 rs1001471 2 GG GG GG AG GG
GG AG GG AG AG GG GG GG
AG AG AG AG AA AG AG GG
AG AG AG AG AG GG AG AG
AG GG AG GG AG AG AG
56498664
1371 rs10014745 4 AA AA AA AA AA
AA AA AA AA AA AG AA AG
AG AA AA AA AA AA AA AA
AA AA AA AA AG AA AA AA
AA AA AA AA AG AA AG
115791738
1372 rs10014791 4 AA AA AA AA AA
AA AA AA AA AA AA AA AA
AA AA AA AA AA AA AA AA
AA AA AA AA AA AA AA AA
AA AA AA AA AA AA AA
75145466
1373 rs10014828 4 GG GG GG GG GG
GG GG GG GG GG GG GG GG
GG GG AG -- GG AG GG GG
GG GG GG GG GG GG GG GG
GG GG -- AG GG GG GG
541182
1374 rs10014850 4 GG GG GG GG GG
GG GG GG GG GG GG GG GG
GG GG GG GG GG GG GG GG

GG GG GG GG GG GG GG GG
GG GG GG GG GG GG GG
53570887
1375 rs1001495 2 AA AA AA AA AA
AA AA AA AA AA AA AA AA
AA AA AA AA AA AA AA AA
AA AA AA AA AA AA AA AA
AG AA AA AA AG AA AG
241647342
1376 rs1001498 3 GG AG AG AG AG
AA AG AA AA AA AG AG GG
AG AG AG GG GG GG GG AG
AA GG AG AG GG AG GG GG
AA AG AA AA AG GG AG
69516320
1377 rs10014992 4 AA AC AC AC AC
CC AC AC AC AC AC AA AC
CC CC AA AC AC CC CC CC
CC CC CC CC CC AA AC AC
AA AA CC AC CC CC AC
91568501
1378 rs10015020 4 GG GG GG GG GG
GG GG GG GG GG GG GG GG
GG AG GG GG GG GG GG GG
GG GG GG GG AG GG GG AG
GG AA GG AG GG GG GG
58827259
1379 rs10015045 4 AA AG AG AA AA
AA AA AA AG AA AA AA AA
AA AA AA AA AA AA AA AA
AA AA AA AA AA AA AA AA
AA AG AA AG AA AA AG
178498527
1380 rs10015146 4 AA AA AG AG AA
AG AA AA AA AA AA AA AA
AA AA AA AA AA GG GG AG
AA AA AA AA AA AA AA AA
AA AG AA AA AG AG AA
82401332
1381 rs10015182 4 GG GG GG GG GG
GG GG AG AG AG GG GG GG
GG AG GG GG GG GG AG GG
GG GG GG GG GG AG AG GG
GG GG GG GG AG GG AG
91253355
1382 rs10015214 4 AG AA AG AG AG
GG AG AA AA AA GG AA AG
AG AA AG AG AG AG AG GG
GG GG GG GG AA GG AG AG
GG AG AG GG AA AG AA

112524778
1383 rs1001527 10 AA AG AG AG AG
AG AA AG AA AA AA AG AG
AG AG AA AA AA AG AA AA
AA AA AG AG AG AG AG GG
AA AA AA AA AA AA AA
82421239
1384 rs10015366 4 GG GG AG GG GG
GG GG GG GG GG GG GG AG
GG GG GG GG GG GG GG GG
GG GG GG GG GG GG GG GG
GG AG GG AG AG GG AG
47057759
1385 rs10015537 4 AG GG AG GG AG
AG AG AG AG AA AA AA AA
AG AG AA AG AG AA AA AG
AA AA AA AA AA AA AA AA
AG AG AA AG AA AA AA
45705727
1386 rs1001555 15 AA AA AA AA AA
AA AA AA AA AG AG AA AA
AA AA AA AA AA AA AG AA
AG AG AG AG AA AA AA AA
AA AA AA AA AA AA AA
32273666
1387 rs10015634 4 GG GG AG AG AG
AA GG GG GG GG GG AG GG
AG AG GG GG GG GG GG GG
GG GG GG GG AG AG AG AA
GG GG AG AG AG AG GG
57362827
1388 rs1001567 1 AA AA AA AC AC
AA CC AA AC AC CC CC AC
AC AC CC CC CC CC CC AC
AA AC AA AC AC AA AA AC
AC AA AC AC AA AC AC
9194614
1389 rs10015799
4 -- -- -- -- -- -- -- --
-- -- -- -- -- -- -- --
-- -- -- -- -- -- -- --
-- -- -- -- -- -- -- --
-- -- -- -- 57501521
1390 rs10015891 4 GG AG AA AA AG
AG AG GG GG AG GG GG GG
GG GG GG GG GG AA AA AG
GG AG AG AA AG AA AA AG
AA GG AG GG GG AG AG
46957305
1391 rs10015926 4 GG GG AG AG AG

	AG	AG	GG	GG	GG	GG	GG	GG
	GG	AG	GG	GG	GG	GG	GG	GG
	GG	AG	AG	GG	AG	AA	AG	AG
	GG	GG	AG	AG	AA	AA	AG	
27241002								
1392	rs1001594		7	AG	GG	AG	AG	AG
	AG	AG	AA	AA	AG	AG	AG	AG
	AG	AG	AA	AG	AG	AA	AA	AA
	GG	AG	AG	GG	GG	AG	AG	AG
	GG	AA	AG	AG	AG	GG	AG	
82630706								
1393	rs10015964		4	GG	GG	AG	AG	GG
	AG	GG	GG	GG	GG	GG	AG	GG
	GG	GG	GG	GG	GG	GG	GG	GG
	GG	GG	GG	GG	GG	AG	GG	GG
	GG	GG	GG	GG	GG	AG	GG	
30639658								
1394	rs10015990		4	AA	AA	AA	AC	AA
	AC	AA	AA	AA	AA	AC	AC	AC
	AC	AA	AA	AC	AC	AC	AC	AA
	AC	AA	AA	AA	AA	AA	AA	AA
	AA	AC	CC	AC	AA	AA	AA	
175035169								
1395	rs10016019		4	GG	GG	GG	GG	GG
	GG	GG	GG	GG	GG	GG	GG	GG
	GG	GG	GG	GG	GG	GG	GG	GG
	GG	GG	GG	GG	GG	GG	GG	GG
	GG	GG	GG	GG	GG	GG	GG	
110433390								
1396	rs1001605		15	GG	AG	AG	GG	GG
	GG	GG	AG	AG	AG	AG	AG	AG
	AG	AG	GG	AG	AG	GG	GG	GG
	GG	GG	GG	GG	AG	AG	AG	GG
	GG	AG	AG	GG	GG	GG	GG	
68773274								
1397	rs10016050		4	AG	AG	AA	AA	AA
	AA	AA	AA	AA	AA	AA	AG	AA
	AA	AA	AA	AA	AA	AA	AA	AA
	AA	AA	AA	AA	AA	AA	AA	AA
	AA	AA	AG	AG	AG	AG	AA	
10747573								
1398	rs10016053		4	GG	GG	AG	GG	GG
	GG	GG	GG	GG	GG	GG	GG	AG
	AG	GG	GG	GG	GG	GG	GG	GG
	GG	GG	GG	GG	AG	GG	GG	AG
	GG	GG	GG	GG	GG	GG	GG	
90551351								
1399	rs10016186		4	CC	CC	CC	CC	CC
	CC	CC	CC	CC	CC	CC	CC	CC
	CC	CC	CC	CC	CC	CC	CC	CC

CC CC CC CC CC CC CC CC
CC CC CC CC CC CC CC
184829536
1400 rs10016220 4 AA AA AA AA AA
AA AA AA AA AA AA AA AA
AA AA AA AA AA AA AA AA
AA AA AA AA AA AA AA AA
AA AA AA AA AA AA AA
79308446
1401 rs10016226 4 AA AA AA AA AG
AG AA AA AA AA AA AA AA
AA AG AA AA AA AA AA AA
AG AA AA AA AA AA AA AA
AA AA AA AA AA AA AA
47157254
1402 rs10016411 4 AA AG GG AA AA
AA AA AA AA AA AG AG GG
AA AG AA AA AA AA AA AG
AG AG AG AA AG AA AG AG
AA AG AA AA AA AA AA
4562916
1403 rs1001644 2 GG GG GG GG GG
-- GG GG GG GG AG GG AG
AA AA GG GG GG AG GG --
AG GG -- AG GG AG AG AG
AG GG AA AG AA AA AA
149587457
1404 rs10016453 4 AA AA AA AA AA
AA AA AA AA AA AA AA AA
AA AA AA AA AA AA AA AA
AA AA AA AA AA AA AA AA
AA AA AA AA AA AA AA
47187596
1405 rs10016464 4 GG GG GG GG GG
GG GG GG GG GG GG GG GG
GG GG GG GG GG GG GG GG
GG GG GG GG GG GG GG GG
GG GG GG GG GG GG GG
71420
1406 rs10016530 4 AA AA AA AA AA
AA AA AA AA AA AA AA AA
AA AA AA AA AA AA AA AA
AA AA AA AA AC AA AC AC
AA AA AA AA AA AA AA
183824984
1407 rs10016647 4 AA AA AC AC AC
AA AC AA AA AC AA AA AA
AA AC AC AC AC AC CC AA
AA AC AC AA AA AC AA AC
AA AC AA AA AA AA AA

99640223							
1408	rs10016652	4	AG	GG	GG	GG	GG
	GG	GG	AA	AA	AA	GG	AG	GG
	GG	AG	AG	GG	AG	AA	AA	AA
	AG	AG	AG	AA	AG	AG	AG	AG
	AA	AA	GG	AG	AG	AG	AG	
32020581							
1409	rs10016702	4	AG	AG	AG	AA	AA
	AA	AA	AA	AA	AA	AA	AA	AA
	AA	AA	AA	AA	AA	AA	AA	AG
	AA	AA	AA	AA	AG	AG	AA	AG
	AA	AG	AG	GG	AA	AA	AA	
10447640							
1410	rs10017183	4	AG	AA	AA	AA	AA
	AG	AA	AA	AG	AA	AA	AA	AG
	AG	AG	AA	AG	AG	AA	AA	AG
	AG	AA	AG	AG	AA	AG	AA	AG
	AA	AG	AG	AA	AG	AG	AA	
14232056							
1411	rs10017184	4	GG	GG	GG	GG	GG
	GG	GG	GG	GG	GG	GG	GG	GG
	GG	GG	GG	GG	GG	GG	GG	GG
	GG	AG	AG	AG	GG	GG	GG	GG
	GG	GG	GG	GG	GG	GG	GG	
56055617							
1412	rs10017257	4	AA	AA	AA	AA	AA
	AA	AA	AA	AA	AA	AG	AA	AG
	AG	AA	AA	AA	AA	AA	AA	AA
	AA	AA	AA	AA	AG	AG	AA	AG
	AA	AA	AA	AA	AA	AA	AA	
130985136							
1413	rs1001728	22	GG	GG	AG	GG	GG
	GG	GG	GG	GG	GG	GG	GG	GG
	GG	GG	AG	AG	AG	GG	GG	GG
	GG	GG	GG	GG	GG	GG	GG	GG
	GG	GG	GG	GG	AG	GG	AG	
45192660							
1414	rs1001729	6	GG	GG	GG	AG	AG
	GG	AA	AG	GG	AG	AG	AG	AG
	GG	AG	AA	AG	AG	GG	GG	GG
	AG	GG	GG	AG	GG	GG	GG	GG
	GG	GG	AG	GG	AG	GG	AG	
2595478							
1415	rs10017326	4	AA	AA	AA	AA	AC
	AC	AA	AA	AA	AA	AC	AC	AC
	AC	AC	AA	AA	AA	AA	AA	AA
	AC	AA	AA	AA	AA	AA	AA	AA
	AA	AA	AA	AA	AA	AA	AA	
47209075							
1416	rs10017332	4	AA	AA	AA	AA	AA

AA AA AA AA AA AA AA AA
AA AA AC AC CC AA AA AA
AC AC AC CC AC CC AC CC
AC AC AC AC AA AA AC
96342750
1417 rs1001742 9 GG GG GG GG GG
GG GG GG GG GG GG AG AG
GG GG GG GG GG GG AG GG
GG GG GG GG GG GG GG GG
GG GG GG GG GG GG GG
132979088
1418 rs10017436 4 AA AG AA GG GG
GG AG AG GG AG GG GG GG
GG AG GG AG AG AA AA AG
GG AG AG GG GG GG GG GG
AA AG GG AG GG AG AG
129737089
1419 rs10017463 4 GG AG GG AG AG
AG GG GG AG GG AG AG AG
AG GG AA AA AA AG AG GG
AA AG AA AA AG GG AG AG
GG GG AA AG GG GG GG
147351628
1420 rs10017472 4 AA AG GG GG GG
GG GG AG AG AG AG AG AA
AA AG AG GG GG GG GG GG
GG GG GG GG GG AG AG GG
AA AG GG GG AG GG AG
170037323
1421 rs10017532 4 AA AA AG AG GG
AG AG AG AG AG AG GG AG
AG AG AG AG GG GG GG AG
AG AG AG AA AG AG GG AG
GG GG GG GG AG AG AA
16204829
1422 rs10017558 4 AG AA AA AA AA
AA AA AA AA AA AA AA AA
AA AA AA AA AA AA AA AA
AA AA AA AA AA AA AA AA
AA AG AA AG AA AA AA
22536001
1423 rs10017600 4 AG AA AG GG GG
AG AG AG GG AG AG AG AG
AG AA GG AG AG AG AG AG
AG AG AG AG AA AA AA AA
AG AA AG AG AA AG AG
7186257
1424 rs10017683 4 AA AA AA AA AA
AA AA CC AC AC AC AC AC
CC AC AC AC AC AA AA AA

AA AA AA AC AA AC AA AC
AA AA AC AC AA AA AA
186641563
1425 rs10017711 4 AA AA AA AG AG
AG AG AA AA AG AA AA AA
AA AG AA AA AA AA AA AA
AA AA AA AG AA AG AA AG
AA GG AG GG AA AA AA
23955370
1426 rs10017756 4 GG GG GG AG GG
GG AG GG GG GG AG GG AG
AG GG AG AG GG AG AG AG
GG AG AG GG AA GG AG AG
GG AA GG AG GG GG GG
26197873
1427 rs10017860 4 AA AC CC AA AA
AA AA AA AC AA AC AA AA
AA AC AC AA AC AA AA AA
AA AA AA AA AC AA AA AA
AC AC AC CC AA AA AC
16594879
1428 rs10017867 4 AA AC AC AC AC
CC AA AA AA AA AA AA AA
AA AA AA AA AA AA AA AA
AC AC AC AC AA AA AA AA
AA AA AC AA CC AC AC
40071832
1429 rs10017881 4 AA AA AA AA AA
AA AA AA AA AA AG AA AG
AG AA AG AA AA AA AA AA
AA AA AA AA AA AA AA AA
AA AA AA AA AA AA AA
129696911
1430 rs10017989 4 AG AG AG AG GG
GG AG GG GG GG AG AG GG
GG GG GG GG AG GG GG GG
AG AG GG GG GG GG GG GG
AG GG AG GG AG AG GG
188730709
1431 rs10018002 4 AA AA AA AA AA
AG AA AA AA AA AA AA AA
AA AA AG AA AG AA AA AA
AG AG AG GG AG AA AA AA
AG AA AG AG AA AG AG
6797156
1432 rs10018022 4 GG AG GG AG GG
AG AG GG GG GG AG GG GG
GG GG AG AG AG AG AA AG
GG AG AG GG GG GG GG GG
AG AG GG AG AG GG AG

114678786
1433 rs10018053 4 AG AG AA AG AG
GG AA AG AG AG AG AG AG
GG AG AA AG AG AA AA AG
AA AG AG AG AA AG AG AG
GG GG AA AG AA AA AG
45015525
1434 rs10018066 4 AA AC AC CC AC
AC AC AA AA AA AC AA AA
AA AC AC AA AC AC CC AA
AC AC AC AC AA AA AA AA
AC AA AA AA AC AC AC
67961296
1435 rs10018094 4 GG GG GG GG AG
AG GG AG AG AA AG GG GG
GG GG AG AG AA AG AG AG
GG GG GG GG AA AG AA AG
AG AG GG GG GG AG AG
88770890
1436 rs1001812 8 AG GG AG AG AA
AG AA AG AG AA AA AA AG
AA AG AA AA AG AA AG AA
AG AG AG AG AG AG AG GG
AG AG AG AG AA AG AA
131498180
1437 rs10018121 4 AA AC AC AA AA
AA AC AC AC AC AC AC AA
AA AC AA AC AA AC AC AA
AA AC AC AA AC CC AC AC
AA AC CC CC AA AA AC
120846553
1438 rs10018167 4 GG GG GG GG GG
GG GG GG GG GG AG AG AG
AG GG GG GG GG GG GG AG
GG GG GG AG GG AG GG AG
AG GG GG GG GG AG GG
25176971
1439 rs1001817 3 GG GG GG AG AG
GG AA GG AG GG AG AG AG
AA AA AG AG AA AG AG AG
AA AA AG AA AA AA AA AA
AG AG AG GG GG GG AG
183995341
1440 rs10018248 4 GG AG GG AG AA
AG AG AG AG AG AA AA AA
AG GG AA AA AG AG AG AG
AA GG GG AG GG AA AG AG
AG AA GG AG AG GG AG
105398068
1441 rs10018258 4 AG GG AG AG AG

GG AG AG AA AG GG AG GG
GG AG AG AA AG AG AG AG
AA AA AA AG AG GG AG AG
AG AA AG AG GG AG AG
4731498
1442 rs10018261 4 AA AA AG GG AA
AG AG AA AA AA AA AA AA
AA AA AG AA AA AG AG AG
AA AA AA AA AG AA AA AA
GG AG AA AA AA AA AA
181888903
1443 rs10018317 4 AA AG AG AA AA
AA AA AA AA AA AG AG AA
AA AA AG AA AG AA AA AA
AG AA AA AG AA AG AG AG
AA AG AA AG AA AA AG
40447356
1444 rs10018371 4 CC CC CC CC CC
CC CC CC CC AC CC CC CC
CC CC CC CC CC CC CC CC
CC CC CC CC CC CC CC CC
CC AC CC CC CC AC CC
90334129
1445 rs10018389 4 AA AA AA AG AA
AG AG AG AG AA AA AG AA
AA AG GG AG AG AG AG GG
GG AG AG GG AG AA AA AA
AA AG AG GG GG AG GG
38046569
1446 rs10018538 4 GG GG GG AG GG
GG AG AG GG AG GG GG GG
GG GG GG GG GG GG GG GG
GG GG GG GG AG GG AG AG
AG AG AG AA AG AG GG
181205991
1447 rs10018554 4 GG GG GG AG AG
GG AG GG GG GG GG GG GG
GG AG GG GG GG GG GG GG
GG GG GG GG GG GG GG GG
AG GG GG GG GG AG GG
12305958
1448 rs10018559 4 AG AG GG AA AA
AG AA AG AG AG AG AA AG
AG AA GG GG GG AG AG AG
AG GG GG AG AA AG AA AG
GG AG AG AG GG GG AG
124946684
1449 rs10018581 4 AA AG AG AA AA
AA AA AA AA AA AA AA AA
AA AA GG AG AG AA AA AA

AA AA AA AA AA AA AA AA
AA AA AG AA AA AA AA
82422433
1450 rs10018596 4 AA AA AA AA AA
AA AA AA AG AA AA AA AA
AA AA AA AA AA AA AA AA
AA AA AA AA AA AA AA AA
AA AA AA AA AG AA AG
184694959
1451 rs10018606 4 GG AG AG GG GG
GG GG GG AG AG GG GG GG
GG GG AG AA AG GG GG GG
GG GG GG GG GG GG GG GG
GG GG AG AG AG AG GG
58138412
1452 rs10018635 4 AA AA AG GG GG
GG GG GG AG AG GG AG AG
AG AG AG AA AA GG AG GG
AG GG AG AG AG GG AG AG
GG AG AG AG AG AA GG
147783160
1453 rs10018680 4 GG GG GG AG GG
GG AG GG GG GG AG AG GG
AG GG GG GG AG GG GG GG
AG AG GG GG GG AG AG GG
AA AG AG AA AG AG GG
188311821
1454 rs1001869 5 GG GG GG GG GG
GG GG GG GG GG GG GG AG
GG AG AG AA AG GG GG GG
AG GG AG AG AG AG AG AA
GG GG AG AG GG AG GG
168760826
1455 rs10018694 4 GG GG GG GG GG
GG GG GG GG GG GG GG GG
GG GG GG GG GG GG GG GG
AG GG GG GG GG GG GG GG
GG GG GG GG GG GG GG
57488975
1456 rs1001870 15 GG GG GG GG GG
GG GG AA AA AA AG AA AA
AA AA AG GG GG AG AA GG
GG AG AG AG GG AA AG AG
AG AA AG AG AA AA AA
68151448
1457 rs10018702 4 AA AA AA AA AA
AA AA AA AA AA AA AA AA
AA AA AA AA AG AA AA AA
AA AG AG AA AA AG AA AG
AG AA AG AA AG AG AG

109832429
1458 rs10018731 4 GG GG GG AG AG
GG AG AG AG AA GG AG GG
GG AG GG GG GG AG GG AG
AA AG AA AA AA AG AG AA
GG AG AG AG GG GG GG
142728951
1459 rs1001874 X GG AG AA AG AA
GG AA AA AA AA AA AA AA
AG AG AA AA AA AA AA AA
AA GG AA AA AG AA AA GG
AA AG AA GG GG AG GG
86808168
1460 rs10018852 4 AG GG GG GG GG
GG GG GG AG AG GG GG GG
GG GG GG GG GG GG GG GG
GG GG AG AG AG GG AG AG
GG GG GG GG GG GG GG
185901323
1461 rs10018868 4 CC AC CC CC AC
AC AC CC AC AC CC CC AC
CC AC CC CC CC AA AA AA
AC AC AC AC AC CC AC AC
CC AA AA AA CC CC AC
189931630
1462 rs1001893 2 GG GG GG GG GG
GG GG GG GG GG GG GG GG
GG GG GG GG GG GG GG AG
GG GG GG GG AG GG GG AG
GG GG AG AG GG GG GG
75872246
1463 rs10018963 4 AA AA AA AA AA
AA AA AA AA AA AG AG AG
AG AG AG AA AA GG GG AG
AG GG AG AG AG AA AG AG
AG AA AG AA GG AG AG
139409081
1464 rs10019008 4 GG GG GG GG GG
GG GG AG GG AG GG GG GG
GG GG AG AG GG AG AG AG
GG AG GG GG GG AA AG AG
GG GG AA AG AG GG AA
15743573
1465 rs10019047 4 AA AA AA AG GG
AG AG GG GG GG AA AA AA
AA AA AG AG AG AA AG AA
GG GG GG GG AA GG AG AG
AG AG AA AA AA AG AA
150459436
1466 rs10019070 4 GG GG GG GG GG

GG GG AA AA AG GG GG GG
GG GG GG GG GG GG GG GG
AG AG AG GG AA GG AG AG
AA GG GG GG GG GG AG
46627357
1467 rs10019140 4 AA AA AG AA AA
AA AA AA AA AA AG AG AA
AA AG AA AA AA AA AA AA
AA AA AA AA AA AG AA AG
AA AA AA AA AA AA AA
180275389
1468 rs10019217 4 GG GG AG GG GG
GG GG GG GG AG GG GG GG
GG GG AG GG GG AG GG AG
GG GG GG GG GG GG GG GG
AG AG GG GG GG AG GG
106588509
1469 rs10019219 4 AA AA AA CC CC
CC CC AC CC CC AC AC AC
CC AC AC AC CC CC CC CC
AC CC CC CC AC AC AC AC
CC AA CC AC CC CC AC
74915427
1470 rs1001926 2 AG AG AA AA AA
AA AA AA AA AA AG AG AA
AA AA AA AA AA AA AA AA
AA AA AA AA AA AA AA AA
AA AA AA AA AA AA AA
173289239
1471 rs10019290 4 AG AA AG AG AG
AA AG AG AG GG AG AG AG
-- GG AA AA AA AG AG GG
AA AA AA AA AA GG AG AG
AA AA AA AA GG AG AG
39515476
1472 rs10019333 4 AG AG AA AG AA
AA AG AG AG AG AG AG AG
AG AA GG GG GG AA AG AG
AA AA AA AA AG AA AA AA
GG AA AA AA AA AA AA
159132582
1473 rs10019349 4 GG GG GG GG GG
GG AG AG AG AG GG GG GG
GG AG AG AG GG GG GG AG
GG GG GG GG AG GG AG GG
GG AG AG AG GG GG GG
83102836
1474 rs10019366 4 AA AA AC AA AA
AA AA AC AC AA AC AC AC
AC CC AA AA AA AC AC AC

AA AC AC AA AC AC AC CC
AA AA AA AA AA AA AA
129239583
1475 rs10019440 4 AG GG AG AG AG
GG AA GG GG GG AA AG AA
AA GG AG AG AA AA AA AG
AG GG GG AA AA GG AG AG
GG AG AG AG AG AG AG
29554873
1476 rs10019509 4 GG GG GG GG GG
GG GG GG GG GG GG GG GG
GG AG GG GG GG GG GG GG
GG AG AG AG GG GG GG GG
GG GG AG AG GG GG GG
41247773
1477 rs10019549 4 AA AA AA AC AA
AA AC AA AA AA AA AA AC
AA AC AA AA AA AA AA AA
AC AA AA AC AA AA AA AA
AA AC AC AA AA AA AA
179159462
1478 rs10019682 4 AG AG GG AG AG
GG AG GG GG GG GG GG AG
GG GG GG GG GG GG GG GG
GG AG AG AG GG GG GG GG
GG GG GG GG GG AG GG
61228770
1479 rs10019688 4 AC CC AC AC CC
AC CC CC CC CC CC CC CC
CC CC AC AC CC CC CC CC
CC CC CC CC CC CC CC CC
CC CC AC AC CC CC CC
76251731
1480 rs10019772 4 AA AA AA AA AA
AG AA AA AA AA AA AA AA
AA AA AA AA AA AA AA AA
AA AA AA AA AA AA AA AA
AA AA AA AA AA AA AA
1835546
1481 rs1001980 6 AA AA AA AA AA
AA AA AA AA AA AA AA AA
AA AA AA AA AA AA AA AA
AA AA AA AA AA AA AA AA
AA AA AA AA AA AA AA
100809495
1482 rs10019893 4 AA AA AA AA AA
AA AA AA AC AC AA AA AA
AA AA AC AC CC AA AA AA
AA AA AA AA AC AA -- AA
AA AC AA AC AC AC AA

189384671							
1483	rs10019975	4	AG	AG	GG	AG	GG
	AG	AG	AA	AG	AA	AG	AG	AA
	AA	AG	AG	AG	AA	GG	GG	AG
	GG	GG	GG	GG	AG	AG	GG	AG
	AA	AG	AA	AG	GG	GG	GG	
156874028							
1484	rs1002005	1	AA	AA	AA	AA	AA
	AA	AA	AA	AA	AA	AA	AA	AA
	AA	AG	AA	AA	AA	AA	AA	AA
	AA	AA	AA	AA	AA	AA	AA	AA
	AG	AA	AG	AA	AA	AA	AG	
61360179							
1485	rs10020075	4	GG	GG	GG	GG	GG
	GG	GG	GG	GG	GG	GG	GG	GG
	GG	GG	GG	GG	GG	GG	GG	GG
	GG	GG	GG	GG	GG	GG	GG	GG
	GG	GG	GG	GG	GG	GG	GG	
1820063							
1486	rs10020087	4	AA	AA	AA	AA	AA
	AA	AA	AA	AA	AA	AA	AA	AA
	AA	AG	AA	AA	AA	AG	AG	AA
	AA	AA	AA	AG	AA	AA	AA	AA
	AA	AA	AA	AA	AA	AA	AG	
172509887							
1487	rs10020156	4	AA	AG	GG	GG	GG
	GG	AG	GG	GG	GG	GG	GG	GG
	GG	AG	AA	AA	AA	AA	AG	AA
	AA	AG	AG	AA	AA	AG	AA	AG
	AA	AA	AA	AA	AA	AG	AG	
78349135							
1488	rs10020212	4	AA	AA	AA	AG	AG
	AG	AA	AG	AG	AG	AG	AG	AG
	AG	AA	AA	AA	AA	AA	AG	AG
	AA	AA	AA	AA	AA	AA	AA	AA
	AA	AA	AG	AA	AA	AA	AA	
160708061							
1489	rs1002026	2	AC	AA	AA	AC	AC
	AA	CC	AA	AC	AA	AC	AA	AA
	AA	AA	AA	AA	AA	AA	AA	AA
	AC	AA	AC	AA	AA	AC	AC	AA
	AC	AC	AA	AC	AA	AC	AA	
75139785							
1490	rs10020270	4	GG	GG	GG	GG	GG
	GG	GG	AG	AA	AG	AG	GG	GG
	GG	GG	GG	AG	AG	AG	AG	AG
	AG	AG	AG	GG	GG	GG	GG	GG
	AA	GG	GG	GG	AG	GG	AG	
16460247							
1491	rs10020331	4	GG	GG	GG	AG	AG

	GG	AA	GG	GG	GG	AA	AA	AG
	AG	AG	AG	AG	AG	AG	AG	AA
	AG	GG	GG	GG	GG	GG	GG	GG
	GG	GG	AG	GG	GG	AG	AG	
170670592								
1492	rs10020336		4	GG	AG	AA	AG	GG
	AG	AG	GG	GG	GG	AG	AG	AA
	AA	AA	GG	AG	AG	AG	AG	AA
	GG	AG	GG	AG	AG	AG	AG	GG
	AA	GG	AG	AG	AA	AA	AG	
16527611								
1493	rs10020349		4	GG	GG	GG	GG	GG
	GG	GG	AA	GG	AA	AG	AG	GG
	GG	AG	AA	AG	AG	GG	GG	AG
	GG	GG	GG	GG	AG	GG	GG	GG
	AG	GG	AG	GG	AG	GG	AA	
181153885								
1494	rs10020374		4	GG	GG	GG	GG	GG
	GG	AG	GG	GG	GG	GG	GG	GG
	GG	GG	GG	GG	GG	GG	GG	GG
	GG	GG	GG	GG	GG	GG	GG	GG
	GG	GG	GG	GG	AG	GG	AG	
44955964								
1495	rs1002041		9	AC	AC	AC	CC	AC
	AC	AC	AC	AC	CC	CC	AC	AC
	AC	AC	AC	AC	AC	AC	AC	AC
	CC	AC	CC	AC	CC	CC	CC	CC
	AC	AC	AC	AC	CC	CC	CC	
78203448								
1496	rs10020429		4	AG	GG	AG	GG	GG
	GG	GG	AG	GG	GG	GG	GG	GG
	GG	GG	AG	AG	AG	GG	GG	GG
	GG	GG	GG	GG	GG	GG	GG	GG
	AA	GG	GG	GG	GG	GG	GG	
189950397								
1497	rs10020534		4	AA	AA	AA	AC	AC
	AA	CC	AC	AC	AC	AA	AA	AA
	AA	AA	AA	AA	AA	AA	AA	AA
	AC	AC	AC	AA	AA	AC	AC	AC
	AC	AA	AA	AA	AA	AA	AA	
54193673								
1498	rs10020548		4	AG	AA	AG	GG	GG
	GG	GG	GG	GG	GG	GG	GG	GG
	GG	GG	GG	GG	AG	GG	GG	GG
	GG	GG	GG	GG	GG	GG	GG	GG
	GG	GG	GG	GG	GG	GG	AG	
114062948								
1499	rs10020652		4	CC	CC	CC	CC	CC
	CC	CC	CC	CC	CC	CC	CC	CC
	CC	CC	CC	CC	CC	CC	CC	CC

	CC	CC	CC	CC	CC	CC	CC	CC
	CC	CC	CC	CC	CC	CC	CC	
110415750								
1500	rs1002067		5	AA	AA	AA	AG	GG
	GG	AG	GG	GG	GG	GG	GG	GG
	GG	AG	AG	AG	GG	GG	GG	GG
	GG	GG	GG	GG	AG	AA	AA	AG
	AG	AG	AG	AA	GG	GG	GG	
160984243								
1501	rs10020676		4	AG	AG	AG	AA	AA
	AA	AA	AG	AG	GG	AG	AG	AG
	AA	AG	AA	AA	AA	AA	AA	AG
	AA	AA	AA	AG	AA	AG	AG	AA
	AA	AA	AG	AA	AA	AA	AG	
173945730								
1502	rs10020700		4	AG	AG	AG	AG	GG
	GG	AG	GG	GG	AG	AG	GG	GG
	GG	GG	GG	GG	GG	GG	GG	AG
	AG	AG	AG	GG	AG	AG	AA	AG
	GG	AA	AG	AA	GG	GG	GG	
19101375								
1503	rs10020714		4	AG	AG	GG	AG	AG
	AA	GG	AG	AG	AG	GG	AG	AG
	AG	AA	AG	AA	AA	AG	AG	AA
	AA	AA	AA	AG	AA	GG	AG	AG
	AG	AG	AA	AA	AG	AG	AA	
169456359								
1504	rs10020796		4	AA	AA	AG	AG	AG
	AG	AA	AA	AG	AA	AG	AG	AA
	AA	AG	AG	AA	AA	GG	AG	AG
	AA	AA	AA	AA	AA	AA	AA	AA
	AG	AA	AA	AA	GG	GG	GG	
153519781								
1505	rs10020813		4	AG	AA	AA	GG	AG
	AG	GG	AA	AA	AG	AG	AA	AG
	AG	AG	AG	AA	AG	AG	AG	AG
	AG	AA	AA	GG	GG	GG	GG	GG
	AA	AA	GG	AG	AA	AA	AA	
29756029								
1506	rs1002095		9	AG	AG	AA	AG	GG
	AG	GG	AG	GG	AA	AG	AG	GG
	GG	AG	AG	GG	GG	AA	AA	AA
	AG	AG	AG	AG	GG	GG	GG	GG
	AA	AG	AG	AG	AG	AG	AG	
130545950								
1507	rs10021038		4	AA	--	--	--	AA
	AA	--	--	--	--	AA	AA	AG
	AG	AA	AG	AG	AA	AA	AA	AG
	AG	--	--	AG	AG	AG	AG	AG
	AA	AG	AG	AA	AA	AG	AA	

36442633								
1508	rs10021045		4	AG	GG	GG	AG	AG
	AG	AG	AG	AG	AA	AG	AG	AA
	AA	AG	GG	GG	GG	GG	GG	AG
	AA	AG	AA	AA	AG	AG	AA	AG
	AG	GG	GG	GG	AG	GG	AA	
139203950								
1509	rs10021106		4	AA	AA	AG	AG	AG
	AG	GG	AA	AG	GG	AA	AA	AA
	AA	AG	AA	AG	AG	GG	GG	GG
	AG	GG	GG	AG	AG	AA	AG	AG
	AA	AG	AA	AA	AA	AA	AA	
131620069								
1510	rs10021207		4	GG	GG	GG	AA	AG
	AG	AG	GG	GG	AG	GG	AA	AG
	GG	GG	AG	GG	GG	AG	GG	AG
	AA	AA	AA	AA	AG	AA	AA	AG
	GG	AG	AG	AG	AG	AA	GG	
113658555								
1511	rs10021263		4	AA	AA	AA	AA	AA
	AA	AA	AA	AA	AA	AC	AA	AA
	AA	AA	AA	AA	AA	AA	AA	AA
	AA	AA	AA	AA	AA	AC	AC	AC
	AA	AC	AA	AA	AA	AA	AA	
40342618								
1512	rs10021303		4	AA	AA	AG	GG	GG
	GG	GG	AG	AG	GG	GG	GG	GG
	AG	AG	AG	AG	GG	GG	AG	GG
	GG	AG	AG	GG	AG	GG	GG	AG
	GG	GG	AA	AG	GG	GG	GG	
95856681								
1513	rs1002132		14	AA	AC	AA	CC	CC
	CC	CC	CC	AC	CC	AC	AC	AA
	AA	CC	CC	CC	CC	AC	AA	CC
	CC	CC	AC	AC	CC	CC	CC	CC
	AC	AC	AA	AA	CC	AC	AC	
52285373								
1514	rs10021331		4	AA	AA	AA	AG	AA
	AA	AG	AA	AA	AA	AA	AA	AA
	AA	AA	AA	AG	AG	AA	AA	AA
	AA	AA	AA	AA	AG	AA	AA	AA
	AA	AA	AG	AG	AA	AG	AA	
180245489								
1515	rs10021408		4	GG	GG	GG	GG	GG
	GG	GG	GG	GG	GG	GG	GG	GG
	GG	GG	GG	GG	GG	GG	GG	GG
	GG	GG	GG	GG	GG	GG	GG	GG
	GG	GG	GG	GG	GG	GG	GG	
55182106								
1516	rs1002142		8	AC	AC	AC	AC	CC

AC CC CC CC CC AC AC AC
CC CC AC AC AC CC AC CC
AC AC AC AC AC AC AC AA
AC AC AC AC CC CC CC
131489046
1517 rs10021442 4 AA AA AA AA AA
AA AA AA AA AA AA AA AG
AA AA AA AA AA AA AA AA
AA AA AA AA AA AA AA AA
AA AA AG AA AA AA AA
47589896
1518 rs10021529 4 GG GG GG AG AA
AG AG AG GG GG GG AG AG
GG GG GG GG GG AG AG GG
GG GG GG GG AG GG GG AG
GG AG GG GG GG GG GG
58202128
1519 rs10021542 4 AG AG GG AG GG
GG AG GG GG AG AA AA AA
AA AG AA AA AA AA AA AA
AG AA AA AG AG AA AG AG
AA AG AG AG AG AA AG
23555237
1520 rs1002158 2 CC AC CC CC CC
CC CC AC AC AC AA AC AC
AC CC CC CC AC AC AC AC
AA AA AC AA AC AC CC AA
AC CC CC CC AC AC CC
23538843
1521 rs10021585 4 AG GG AG AG AG
AA AG AG AA AA AA -- AG
AG GG AG AG AG AG AG AG
AG AG AG AG AA GG AG AG
AG AG AG AG AA AG AG
133118600
1522 rs10021666 4 AA AA AA AA AA
AA AA AA AA AA AA GG AG
AA AA AA AA AA AA AA AG
AA AA AA AA AA AG AG AA
AA AG AA AG AA AA AA
107763066
1523 rs10021692 4 GG GG GG GG GG
GG GG GG GG GG GG GG GG
GG GG GG GG GG GG GG AG
AG AG AG GG AG GG AG AG
AG GG GG GG GG AG GG
188080985
1524 rs10021705 4 AA AA AA AG AA
AA AG AA AA AA AA AA AA
AA AA AA AA AA AA AA AA

AA AA AA AA AA AA AA AA
AA GG AA AG AG AG AA
21793217
1525 rs1002175 15 GG AG AG AG AG
AA GG AG GG AG GG AG GG
AG GG GG AG AG GG GG GG
GG GG GG GG AG AG AG AA
GG AG GG GG AG AG GG
63686327
1526 rs1002182 5 AG AA AG GG AA
AG AG AG AG AG AA AA AA
AA AA AG AA AG AG AG AA
AA AA AG AG AA AG AG AG
AG AG AG AG AA AA AA
147236546
1527 rs10021925 4 AG AG AA AA AG
AG AG AG AG AA AA AA AA
AA AG AA AA AA AA AG AA
AG AG AG AG AG AA AA AA
GG AG AA AG AG AG AG
117659634
1528 rs10021966 4 AA AA AA AA AA
AA AA AA AA AA AG GG AG
AA AA AG AG AA AA AA AA
AG AA AA AG AG GG GG AG
AA AA AA AA AA AA AA
96450093
1529 rs10021969 4 AC AC AC CC CC
CC CC AC AC CC CC CC CC
CC AC CC CC CC CC CC CC
AC CC AC AC AC AC AC AA
AC CC AC CC CC CC CC
138535434
1530 rs10022002 4 AG AG AA AG AA
AG AG AG AG GG AA AA AA
AA AG AA AA AA AG AG GG
AG AG AG AA AA AG AG AA
AA AA AG AA GG AG AG
169484212
1531 rs10022034 4 GG GG GG GG GG
AG GG GG GG GG GG GG GG
GG GG GG GG GG GG GG GG
AG AG AG GG AG GG AG AG
GG GG AG AG GG GG GG
186947665
1532 rs1002204 7 CC AC AC AC AC
AC AC AC AC CC AA AA AA
AA AA AA AA AA CC CC CC
AC AC AC AC AC AC AA AC
CC CC AC CC AC AC AC

87141497							
1533	rs10022111	4	GG	GG	GG	AG	AA
	AG	AG	AG	AG	GG	AA	AA	AA
	AA	AG	AG	AG	AG	GG	GG	GG
	GG	AG	AG	AG	AG	AG	GG	AG
	AA	GG	AG	AG	GG	GG	GG	
169484303								
1534	rs10022128	4	AA	AA	AA	AA	AA
	AA	AA	AG	AG	AA	AA	AA	AA
	AA	AA	AG	AG	GG	GG	AG	AG
	AA	AA	AA	AA	GG	AA	AG	AG
	AA	AG	AA	AG	AG	AG	AA	
118122688								
1535	rs10022193	4	GG	GG	GG	GG	GG
	GG	GG	AA	AA	AA	GG	AG	AG
	AG	AG	AG	AG	GG	GG	AG	AG
	GG	GG	GG	GG	AG	AG	GG	AG
	GG	GG	GG	GG	GG	GG	GG	
3291694								
1536	rs10022206	4	AG	AG	GG	AG	AA
	AG	AA	AG	AG	AG	GG	GG	GG
	GG	GG	AG	AG	AG	AA	AA	AA
	AG	AG	AG	AG	GG	AG	AG	GG
	AG	GG	AG	GG	AG	AG	GG	
183023943								
1537	rs10022300	4	AA	AA	AG	AG	AG
	GG	AA	GG	GG	GG	AA	AA	AA
	AA	AA	AA	AG	AG	AA	AG	AA
	AG	AG	AG	AG	AA	AG	AA	AG
	AA	AG	AA	AG	AG	AG	AA	
157401209								
1538	rs1002233	2	AG	GG	AG	AA	AA
	AA	AA	AG	AG	AG	GG	AG	AG
	AA	AG	AA	AG	AG	AG	AG	GG
	AA	AG	AA	AA	AA	AG	AG	AA
	AG	AG	AG	GG	AG	AG	AG	
117251750								
1539	rs10022399	4	AA	AG	GG	AG	AG
	AA	GG	AG	AG	AG	AG	AA	AA
	AA	AG	AG	AA	AA	AG	AA	AG
	GG	GG	GG	GG	AG	GG	AG	AG
	AA	GG	AA	AG	GG	GG	GG	
160249722								
1540	rs1002240	3	AG	AA	AG	GG	AG
	GG	AG	GG	AG	AG	AG	AG	AG
	GG	AG	AG	GG	AG	AG	GG	AG
	GG	GG	GG	GG	AG	GG	GG	AG
	AG	AA	AG	AA	AA	AG	AG	
177552252								
1541	rs10022407	4	AA	AA	AA	AA	AG

	AG	AA	AG	AG	AG	AA	AA	AG
	AG	AG	AG	GG	AG	AA	AG	AA
	AG	GG	GG	GG	AA	AG	AA	AG
	AG	AA	AG	AG	AG	AG	AG	
91780648								
1542	rs10022442		4	CC	CC	CC	CC	CC
	CC	CC	AC	AC	CC	AC	AA	AA
	AC	CC	CC	CC	AC	AC	AA	AC
	CC	AC	AC	AC	CC	CC	CC	CC
	CC	AC	AC	CC	AA	AC	AA	
102470700								
1543	rs10022584		4	AA	AA	AA	AA	AA
	AA	AA	AA	AA	AA	AA	AA	AA
	AA	AA	AA	AA	AA	AA	AA	AG
	AA	AA	AA	AA	AA	AA	AA	AA
	AA	AA	AA	AA	AA	AA	AA	
156352066								
1544	rs10022602		4	AA	AG	GG	GG	GG
	GG	GG	GG	AG	GG	AG	AG	GG
	GG	GG	AG	AG	GG	AG	AG	AA
	GG	GG	GG	GG	AG	AG	AA	AG
	GG	AA	GG	AG	AG	AG	GG	
12741313								
1545	rs10022718		4	AA	AG	AG	--	--
	--	--	AA	AA	AA	AG	AA	AG
	AG	AA	AA	AG	AG	AG	--	AG
	--	--	--	--	AG	--	--	AG
	AG	AG	AG	AG	--	--	--	
41186226								
1546	rs10022733		4	GG	GG	GG	GG	GG
	GG	GG	GG	GG	GG	GG	GG	GG
	GG	GG	GG	GG	GG	GG	GG	GG
	GG	GG	GG	GG	GG	GG	GG	GG
	GG	GG	GG	GG	GG	GG	GG	
1642350								
1547	rs10022753		4	GG	AG	AG	GG	GG
	GG	AG	GG	GG	GG	GG	GG	GG
	GG	GG	AA	AA	AA	GG	GG	GG
	AA	GG	GG	AG	GG	GG	GG	GG
	AG	AA	AG	AA	GG	GG	AG	
94358023								
1548	rs10022773		4	AA	AA	AA	AG	AA
	AG	AG	AG	GG	AG	AG	AA	AA
	AA	AA	AA	AA	AA	AA	AA	AA
	AG	AG	AG	AA	GG	AG	GG	AG
	AG	AG	AA	AG	AG	GG	AA	
14345605								
1549	rs10022799		4	GG	AG	AG	GG	GG
	GG	GG	GG	GG	GG	AG	GG	GG
	GG	GG	GG	AG	AG	AG	AG	AG

	GG	GG	GG	GG	GG	GG	GG	GG
	GG	GG	AA	AG	GG	GG	GG	
168131448								
1550	rs1002285		4	AA	AA	AA	AA	AA
	AA	AG	AA	AA	AA	AA	AA	AA
	AA	AA	AA	AA	AA	AA	AA	AG
	AA	AA	AA	AA	AA	GG	AG	AG
	AA	AG	AG	AA	AA	AA	AA	
57772712								
1551	rs10022874		4	AA	AA	AA	AA	AA
	AA	AA	AA	AA	AA	AA	AA	AA
	AA	AA	AA	AA	AA	AA	AA	AA
	AA	AA	AA	AA	AA	AA	AA	AA
	AA	AA	AA	AA	AA	AA	AA	
56491887								
1552	rs10022885		4	AC	AA	AC	AA	AA
	AA	AA	CC	CC	AC	AA	AA	AA
	AC	CC	CC	CC	AC	AC	AA	AC
	CC	CC	CC	CC	AC	AA	AA	AC
	AC	AC	AC	CC	AC	AA	AC	
114038963								
1553	rs10022899		4	AA	AA	AA	AA	AA
	AG	AA	AA	AA	AA	GG	GG	AG
	AG	GG	AA	AA	AA	AA	AA	AG
	AG	AA	AG	GG	AA	GG	AG	AG
	AA	AG	AA	AA	GG	GG	GG	
14919063								
1554	rs10023012		4	AA	AA	AA	AA	AA
	AA	AA	AA	AA	AA	AA	AA	AA
	AA	AA	AA	AA	AA	AA	AA	AA
	AG	AG	AG	AA	AG	AA	AA	AA
	AA	AG	AA	AA	AA	AG	AA	
19410177								
1555	rs10023049		4	AA	AA	AA	AG	AG
	AA	GG	AG	AG	AG	GG	GG	AG
	GG	GG	AA	AG	AG	AA	AA	AG
	AA	AG	AG	GG	GG	AG	AG	GG
	AA	AG	GG	GG	AG	GG	AA	
177497169								
1556	rs10023055		4	AG	AG	AA	GG	AG
	AG	AG	AG	AG	AG	AG	AG	AG
	AA	AG	AG	AA	AG	AG	GG	AA
	AA	AG	AG	AG	AA	AG	AA	AG
	AA	AA	AG	AG	AG	AG	AG	
65755777								
1557	rs10023064		4	GG	AG	AG	AA	AG
	AG	AG	AG	AG	AG	AA	AA	AA
	AG	GG	AG	AG	GG	AG	AG	AG
	AG	AA	AG	AA	AA	AG	AG	AA
	AG	AG	GG	GG	AA	AA	AG	

66783283								
1558	rs10023086	4	AA	AA	AA	AA	AA
	AA	AA	AA	AA	AA	AA	AA	AA
	AA	AA	AA	AA	AA	AA	AA	AA
	AA	AA	AA	AA	AA	AA	AA	AA
	AA	AA	AA	AA	AA	AA	AA	
1910823								
1559	rs10023150	4	AG	AG	AG	AG	AA
	AG	AG	AG	AG	AG	AG	AA	AA
	AA	AG	AG	AA	AG	AA	AA	AA
	AA	AG	AG	AA	AA	AG	AA	AA
	AA	AA	AG	AA	AA	AA	AA	
34595824								
1560	rs1002317	17	AA	AA	AG	AA	AA
	AA	AA	AA	AA	AA	AA	AA	AA
	AA	AA	AG	AA	AA	GG	GG	AG
	AA	AA	AA	AA	AA	AA	AA	AA
	AG	AA	AA	AA	AG	AG	AA	
31600299								
1561	rs10023223	4	AA	AA	AA	AA	AA
	AA	AA	AG	AG	AA	AA	AA	AA
	AA	AG	AA	AA	AA	AG	AG	AG
	AG	AG	AG	AA	GG	AG	AG	GG
	AA	AA	AG	AG	AA	AA	AA	
171168353								
1562	rs1002327	17	--	AG	AA	AG	AG
	GG	AA	AG	AG	AG	AG	AG	GG
	GG	GG	AA	AA	AA	AA	AA	AG
	AA	AG	AA	AG	GG	AA	AG	AG
	AA	AG	AG	AA	AA	AA	AA	
64874615								
1563	rs10023330	4	AA	AG	GG	GG	GG
	GG	GG	GG	GG	GG	GG	GG	GG
	GG	AG	GG	AG	AG	GG	GG	GG
	GG	GG	GG	GG	AG	AG	AG	GG
	GG	AG	AG	AA	GG	GG	GG	
170558032								
1564	rs10023344	4	AG	AG	AA	AG	AA
	AG	AG	GG	GG	GG	GG	GG	GG
	AG	GG	GG	GG	GG	GG	GG	GG
	GG	GG	AG	AG	AG	GG	AG	AG
	GG	GG	GG	GG	AG	AG	AA	
185999348								
1565	rs10023425							
4	--	--	--	--	--	--	--	--
	--	--	--	--	--	--	--	--
	--	--	--	--	--	--	--	--
	--	--	--	--	--	--	--	--
	--	--	--	--	62788474			
1566	rs10023495	4	AA	AA	AA	AA	AA

	AA	AA	AA	AA	AA	AA	AA	AA
	AA	AA	AA	AG	AG	AA	AA	AA
	AG	AA	AA	AG	AA	AA	AA	AA
	AA	AA	AA	AA	AA	AA	AA	
114236227								
1567	rs10023502		4	AA	AA	AA	AA	AA
	AA	AG	AG	AG	AG	AA	AA	AA
	AA	AG	AA	AA	AA	AG	AG	AG
	AA	AA	AA	AA	AA	AG	AG	AA
	AA	AG	AA	AG	AG	AG	AA	
8313595								
1568	rs10023519		4	AG	AG	AA	AA	AA
	AA	AA	AG	AG	AG	GG	GG	GG
	AG	AA	AA	AA	AA	AA	AA	AG
	AG	AA	AA	AA	AA	AA	AA	AA
	AG	AA	AG	AG	AA	AA	AA	
75153710								
1569	rs10023523		4	GG	AG	AA	GG	GG
	GG	GG	GG	GG	GG	GG	GG	GG
	AG	GG	GG	GG	GG	AA	GG	AG
	AG	AG	AG	AG	GG	AA	AG	AG
	GG	GG	AA	AG	GG	GG	GG	
1588665								
1570	rs10023532		4	GG	GG	GG	GG	GG
	GG	GG	GG	GG	GG	AG	GG	GG
	GG	GG	GG	GG	GG	AA	GG	AG
	GG	GG	GG	GG	AG	GG	GG	GG
	GG	AG	AG	AA	GG	GG	GG	
2182450								
1571	rs10023574		4	AC	AC	AC	CC	CC
	CC	CC	AA	AA	AA	CC	AA	AC
	CC	CC	CC	AC	CC	AC	CC	AA
	AC	CC	CC	CC	AC	AC	AC	AC
	AC	AA	CC	AC	AC	AC	CC	
107781852								
1572	rs10023596		4	AA	AA	AA	GG	AG
	AG	AG	AG	AA	AG	AG	AG	GG
	GG	AA	AG	AG	AG	GG	AG	AG
	AG	AG	AG	AG	AA	AG	AA	AG
	AG	AG	AA	AA	AG	AA	AG	
154094001								
1573	rs10023609		4	GG	GG	GG	GG	GG
	GG	GG	GG	GG	GG	GG	GG	GG
	GG	GG	GG	GG	GG	GG	GG	GG
	GG	GG	GG	GG	GG	GG	GG	GG
	GG	GG	GG	GG	GG	GG	GG	
652883								
1574	rs10023670		4	AG	GG	AG	GG	GG
	GG	AG	AA	AG	AA	AA	AG	AG
	AG	AG	AG	GG	AG	AG	AG	GG

AG AG AG AG AA AA AA AA
AA AG GG GG AG GG AG
7694472
1575 rs1002371 7 AG GG GG GG GG
GG GG AG AG GG GG GG GG
GG GG GG GG GG AG AG GG
GG GG GG GG GG GG GG GG
GG AA AG AA GG GG GG
50488209
1576 rs10023877 4 GG GG GG GG GG
GG GG GG GG GG GG GG GG
GG GG AG AG GG GG GG AG
GG GG GG GG GG GG GG GG
GG GG GG GG GG GG GG
70492055
1577 rs10023965 4 GG AG AG AA AA
AA AG AG AG AG GG GG GG
GG AG AG AG AA AA AA AG
AG AG AG AA AG GG GG AG
AG GG AG AG AA AG AA
47308961
1578 rs1002399 7 GG GG GG GG GG
GG GG AG AG AG AG GG AG
AG GG GG GG GG AG GG AA
GG GG GG GG GG GG GG GG
GG GG GG GG GG GG GG
116285715
1579 rs10024051 4 AG AG AG AG AG
GG AG GG GG GG AA AA AA
AG GG GG GG GG GG AG AG
GG AG AG AG AG AA AG AA
GG AG AA AA AG GG AG
94720590
1580 rs10024057 4 AG AA AG AA AA
AA AA AA AA AA AG GG GG
AG AA AA AA AA AA AA AA
AA AA AA AA AA AA AA AA
AA AA AG AA AA AA AA
53888072
1581 rs10024173 4 AA AA AA GG AG
AG AG AG AG AG AA AA AA
AA AA AG AA AA AA AA AA
AA AA AA AA AG AA AA AG
AA AA AG AG AG AA AG
111538121
1582 rs1002420 9 AA AA AA CC CC
CC AC CC CC CC CC AC AC
AC AC AC CC CC AA AA AC
AA AA AA AA CC CC CC CC
AC CC AC AC AC AA CC

78869524							
1583	rs1002424		5	AA	AG	AG	AG	AA
	AA	AG	AG	AG	AG	AA	AA	AA
	AA	AG	AG	AG	AG	GG	GG	AG
	AG	AG	AG	AG	AG	AG	AG	AG
	AG	AG	AA	AA	AA	AA	AA	
40767397								
1584	rs1002427		9	AA	AG	AA	AA	AA
	AA	AA	AA	AA	AA	AA	AA	AA
	AA	AA	AA	AA	AA	AA	AA	AA
	AA	AG	AA	AG	AA	AA	AA	AA
	AA	AG	AG	AG	AG	AA	GG	
77651915								
1585	rs10024417		4	GG	GG	GG	GG	GG
	GG	GG	AG	AG	AG	GG	GG	GG
	GG	AG	GG	AG	AG	GG	GG	GG
	GG	GG	GG	AG	AG	AA	AA	AA
	GG	GG	GG	GG	AG	GG	AG	
20660406								
1586	rs10024431		4	AC	CC	AC	AC	AA
	AA	AC	AA	AA	AA	AC	AC	AA
	AA	AC	AC	AA	AA	AA	AA	AA
	CC	CC	CC	CC	AA	AA	AA	AA
	AC	AC	AC	AA	AA	AC	AA	
173354354								
1587	rs10024447		4	AG	AG	AG	AG	AG
	AA	AG	AA	AA	AA	AG	AG	AA
	AA	AG	GG	AG	AG	AA	AA	AA
	AG	AG	AG	AG	AG	AA	AG	AG
	AA	AA	AG	AA	AG	AA	AG	
10145890								
1588	rs10024462		4	AA	AA	AG	AA	AG
	AG	AA	AG	AG	AA	AA	AA	AG
	AG	AA	AG	AG	AG	AA	AA	AA
	AA	AA	AA	AA	AA	AA	AA	AA
	AA	AA	AA	AA	AA	AA	AA	
96666203								
1589	rs10024529		4	GG	AG	AG	GG	GG
	GG	GG	AG	AG	AG	GG	GG	GG
	AG	GG	GG	GG	GG	GG	GG	GG
	GG	GG	GG	GG	GG	GG	GG	GG
	GG	GG	GG	GG	AG	AG	GG	
1363886								
1590	rs10024768		4	AC	CC	CC	CC	CC
	CC	CC	CC	CC	CC	AC	CC	CC
	CC	CC	CC	AC	AC	AC	AC	CC
	AC	AC	AC	--	CC	AC	AC	AC
	CC	AC	--	AC	AC	AC	AC	
41605108								
1591	rs1002491		12	GG	GG	GG	GG	GG

GG GG GG GG GG GG GG GG
GG GG GG GG GG AG GG GG
GG GG GG GG GG GG GG GG
GG GG AG GG GG GG GG
79737843
1592 rs10025090 4 AG AG GG AA AG
AG AG AA AA AA GG GG GG
AG AG GG GG GG AG AG AA
GG GG GG GG AG AG AG AA
AG GG AA AG AG GG AG
57638351
1593 rs10025104 4 AG GG GG AG AG
GG AG GG GG GG AG AG GG
GG GG AG GG AG GG GG GG
AG AG AG GG AA GG AG AG
AG GG AG AG GG GG GG
47855999
1594 rs10025120 4 CC CC CC AC AA
AC AC AC AC AA AC AA AA
AA AA AC AC AA AA AA AA
AC AC AC CC AA CC AC AC
AC AC AC AA AA AA AA
84216941
1595 rs10025159 4 AG AG AA AA AA
AA AA AA AA AG AG AG GG
GG AA AA AG AG AA AA AA
AA AG AG AA AG AG GG GG
AG AA AA AA AA AA AA
37575251
1596 rs10025167 4 AA AA AA AA AA
AA AA AC AA AC AA AA AC
AA AC AA AA AA AC AC AC
AA AA AA AC AA AC AC AA
AC AA AC AC AA AA AA
185649551
1597 rs10025263 4 GG GG GG GG GG
GG GG GG GG GG GG GG GG
GG GG GG GG GG GG GG GG
GG GG GG GG GG GG GG GG
GG GG GG GG GG GG GG
110695751
1598 rs10025405 4 GG AG GG AG GG
AG AG AA AA AA AG AG GG
GG GG AG AG AG AA AA AA
AG AG GG AG AG AA AA AA
AA AA AG AA AG AA AG
187006806
1599 rs10025425 4 AG AG AG AG GG
GG AG AG AG AG GG GG AG
GG AG AG AG AG AG AG AA

	AG	GG	GG	AG	AA	GG	AG	AG
	AG	AA	GG	AG	GG	AG	GG	
177964148								
1600	rs10025530		4	AA	AA	AA	AA	AA
	AA	AA	AA	AA	AA	AA	AA	AA
	AA	AA	AA	AA	AA	AA	AA	AA
	AA	AA	AA	AA	AA	AA	AA	AA
	AA	AA	AA	AA	AA	AA	AA	
112568464								
1601	rs10025560		4	AA	AG	AG	AA	AA
	AA	AA	AA	AA	AG	AA	AA	AA
	AA	AA	AG	AA	AA	AA	AA	AA
	AA	AG	AG	AG	AA	AA	AA	AA
	AA	AA	AA	AA	AG	AA	AG	
160278606								
1602	rs10025613		4	AA	AA	AA	AA	AA
	AA	AA	AA	AA	AA	AA	AA	AA
	AA	AA	AA	AA	AA	AA	AA	AA
	AA	AA	AA	AA	AA	AA	AA	AA
	AA	AA	AA	AA	AA	AA	AA	
47316263								
1603	rs10025642		4	AG	GG	GG	AG	GG
	AG	GG	AG	AG	GG	AG	GG	GG
	GG	AG	GG	GG	GG	GG	GG	GG
	AG	GG	GG	AA	GG	GG	GG	GG
	AG	AG	GG	AG	GG	AG	GG	
24454502								
1604	rs10025665		4	AA	AA	AA	AA	AA
	AA	AA	AA	AA	AA	AA	AA	AA
	AA	AA	AA	AA	AA	AA	AA	AA
	AA	AA	AA	AA	AA	AA	AA	AA
	AA	AA	AA	AA	AA	AA	AA	
1312267								
1605	rs1002572		13	AA	AG	AA	AA	AA
	AG	AA	AG	AG	AG	AA	AA	AA
	AA	AA	AA	AA	AA	AA	AA	AA
	AA	AA	AA	AA	AA	AA	AA	AA
	AG	AG	AA	AA	AA	AA	AA	
103852619								
1606	rs10025989		4	AG	AG	AG	AA	AA
	AA	AA	AA	AA	AA	AA	AG	AA
	AA	AG	AA	AG	AG	AG	AG	AG
	AA	AA	AA	AA	AA	AA	AA	AA
	AA	AA	AA	AA	AA	AA	AA	
27459128								
1607	rs10025992		4	AA	AA	AA	AA	AA
	AA	AA	AG	AG	AG	AA	AA	AA
	AA	AA	AA	AG	AG	GG	GG	AG
	AA	AG	AA	AG	AG	AG	AG	AA
	AA	AG	AG	AA	AA	AA	AA	

63838796								
1608	rs10026016		4	AA	AG	AA	AG	GG
	GG	AG	AG	AA	AG	AG	AG	AG
	AG	AG	GG	GG	GG	GG	AG	GG
	GG	AG	GG	GG	GG	GG	GG	GG
	GG	AG	GG	GG	AG	AG	AG	
154885560								
1609	rs10026079		4	GG	GG	GG	GG	GG
	GG	GG	GG	AG	GG	GG	GG	AG
	AG	AG	GG	GG	GG	GG	GG	GG
	GG	GG	GG	GG	GG	GG	GG	GG
	GG	GG	GG	GG	GG	GG	GG	
175913798								
1610	rs10026136		4	AG	GG	GG	AG	GG
	AG	GG	AG	AG	GG	AG	AG	AG
	AG	GG	AG	AA	AG	GG	GG	AG
	GG	AG	AG	GG	AG	AG	AG	GG
	AG	GG	GG	GG	GG	AG	AG	
24000480								
1611	rs10026162		4	AG	AG	AG	AA	AA
	AA	AA	AG	AG	AA	AA	AA	AA
	AA	AA	GG	GG	GG	AA	AA	AA
	AA	AA	AA	AA	AG	GG	AG	GG
	AA	AG	AA	AA	AA	AA	AA	
53718375								
1612	rs10026174		4	GG	GG	GG	GG	GG
	GG	GG	GG	GG	GG	GG	GG	GG
	GG	GG	GG	GG	GG	GG	GG	GG
	GG	GG	GG	GG	GG	GG	GG	GG
	GG	GG	GG	GG	GG	GG	GG	
186405005								
1613	rs10026177		4	AG	AG	AG	AG	AG
	AG	AA	GG	AG	AG	AG	AG	AG
	AG	AG	AA	AA	AA	AA	AA	AA
	AG	AG	AG	AA	AG	AA	AG	AG
	AG	AA	AG	AA	AA	AG	AG	
188428740								
1614	rs10026518		4	GG	GG	GG	GG	GG
	AG	GG	AG	GG	AG	GG	GG	GG
	GG	GG	GG	GG	GG	GG	GG	GG
	GG	GG	GG	GG	GG	GG	GG	GG
	GG	GG	GG	GG	GG	GG	GG	
151588483								
1615	rs1002654		10	AG	AG	AA	AA	AA
	AG	AA	AG	AG	AG	AG	AG	AG
	AG	AG	AG	AG	AA	GG	AG	GG
	AA	AA	AG	GG	AG	AG	AG	AG
	AG	AA	AA	AA	GG	GG	AG	
8448752								
1616	rs1002656		1	AA	AA	AA	AA	AA

	AG	AA	AA	AA	AA	AG	AG	AA
	AA	AA	AG	AG	AG	AA	AA	AG
	AG	AG	AA	AG	AG	AA	AA	AG
	AG	AG	AG	AG	AA	AG	AG	
37192741								
1617	rs10026586		4	GG	GG	GG	AG	AG
	GG	AG	AG	AG	GG	GG	GG	GG
	GG	GG	GG	AG	GG	AG	AA	GG
	AG	GG	GG	AG	AG	AG	AG	AG
	AG	AG	GG	AG	GG	GG	GG	
109162596								
1618	rs10026610		4	AG	AA	AA	AA	AA
	AA	AA	AA	AA	AA	AA	AA	AA
	AA	AA	AA	AA	AA	AA	AA	AA
	AA	AA	AA	AA	AA	AA	AA	AA
	AA	AA	AA	AA	AA	AA	AA	
16462250								
1619	rs10026772		4	AA	AA	AA	AG	AG
	AA	AG	GG	GG	AG	AA	AA	AA
	AA	AA	AG	AA	AG	AG	AG	AG
	AA	AA	AA	AA	AG	AG	AG	GG
	AA	AA	AA	AA	AA	AG	AA	
42301184								
1620	rs1002688		11	AA	AA	AA	AA	AA
	AA	AA	AA	AA	AA	AA	AA	AA
	AA	AA	AA	AA	AA	AA	AA	AA
	AA	AA	AA	AA	AA	AA	AA	AA
	AA	AA	AA	AA	AA	AA	AA	
32051550								
1621	rs1002689		X	GG	GG	GG	GG	GG
	GG	GG	GG	GG	GG	GG	GG	GG
	GG	GG	GG	GG	GG	GG	GG	GG
	GG	GG	GG	GG	GG	GG	GG	GG
	GG	GG	GG	GG	GG	GG	GG	
43753297								
1622	rs10026892		4	AA	AG	AG	AG	AG
	GG	AA	AA	AG	AA	AG	AG	AG
	AG	AG	AG	GG	GG	GG	GG	GG
	AA	AA	AA	AA	AA	AG	AA	AG
	AG	GG	AG	GG	GG	AG	GG	
184511265								
1623	rs1002702		10	CC	CC	CC	CC	CC
	CC	CC	CC	CC	CC	CC	CC	CC
	CC	CC	CC	CC	CC	CC	CC	CC
	CC	CC	CC	CC	CC	CC	CC	CC
	CC	CC	CC	CC	CC	CC	CC	
35994567								
1624	rs1002703		1	AA	AA	AA	AA	AA
	AA	AA	AA	AA	AA	AA	AA	AA
	AA	AA	AA	AA	AA	AA	AA	AA

	AA	AA	AA	AA	AA	AA	AA	AA
	AA	AA	AA	AA	AA	AA	AA	
16579873								
1625	rs1002706		1	AG	GG	AG	GG	AG
	AG	AG	GG	GG	AG	GG	GG	GG
	GG	GG	AG	AG	AG	GG	GG	GG
	AG	AG	AG	AG	AA	AG	AG	AG
	GG	GG	GG	GG	GG	AG	GG	
25051153								
1626	rs1002726		1	AC	AC	CC	AC	AC
	CC	AA	AC	AC	AC	CC	CC	CC
	CC	AC	CC	CC	CC	AC	AC	CC
	CC	AC	AC	AC	CC	AC	AC	CC
	AC	CC	AA	AC	CC	CC	CC	
238726690								
1627	rs10027346		4	AG	AG	AA	AA	AG
	AG	AA	AG	AG	AG	AA	AG	AA
	AA	AA	AA	AA	AA	AG	AG	GG
	GG	GG	GG	AG	AA	AG	AG	AG
	AA	GG	AG	GG	GG	AG	GG	
31909356								
1628	rs10027363		4	AA	AA	AA	AA	AA
	AA	AA	AG	AG	AG	AG	AG	AA
	AA	AA	AA	AA	AA	AA	AA	AA
	AA	AA	AA	AA	AG	AA	AG	AA
	AA	AA	AA	AA	AA	AA	AA	
58679514								
1629	rs10027416		4	GG	GG	GG	AG	AG
	GG	AG	AG	AG	GG	AG	AG	AG
	AG	AG	GG	GG	GG	AG	AG	GG
	AG	GG	AG	AG	GG	AG	AG	GG
	GG	GG	GG	GG	GG	GG	AG	
140542427								
1630	rs1002745		10	AG	AG	AG	GG	GG
	AG	GG	GG	GG	GG	GG	AG	AG
	AG	GG	GG	AG	AG	GG	GG	GG
	GG	GG	GG	GG	AG	GG	AG	GG
	AG	AG	AG	GG	GG	AG	GG	
82656283								
1631	rs10027473		4	AA	AA	AA	AA	AA
	AA	AA	AA	AA	AA	AA	AA	AA
	AA	AA	AA	AA	AA	AA	AA	AA
	AA	AA	AA	AA	AA	AA	AA	AA
	AA	AA	AA	AA	AA	AA	AA	
111664452								
1632	rs1002748		11	CC	CC	CC	CC	CC
	CC	CC	CC	CC	CC	CC	CC	CC
	CC	CC	CC	CC	CC	CC	CC	CC
	CC	CC	CC	CC	CC	CC	CC	CC
	CC	CC	CC	CC	CC	CC	CC	

31062366
1633 rs10027496 4 AA AC AC AA AA
AA AA AC AC AC AA AA AC
AA AC AC AC AC AC AC AC
AA AA AC AC AC CC CC CC
CC AA CC AC AC AC AA
184558870
1634 rs10027536 4 AA AA AA GG GG
AG AG AG AG AG AA AA AA
GG AG AG AA GG AA AG AA
GG AG AG AA GG AA AG AG
AG AG AG AG AG AA AG
167596
1635 rs10027612 4 AA AA AG GG GG
AG GG GG AG GG GG AG GG
GG AG AG AA AA GG AG GG
GG GG GG GG GG AG GG AG
GG AA AG AG GG AG AG
147658082
1636 rs10027655 4 AC AA AC CC CC
AC CC AC AA AC AC AC AC
AC AC CC AC AC AC AC AA
AA AA AA AA AC AC AA AC
AA AC AC CC CC CC CC
156055961
1637 rs10027673 4 GG AG AG AG AA
AG AA AG GG GG AG AA AG
AG GG GG AG AG GG GG GG
AG AG AG AA GG AG AG AG
AG AG GG GG AG AG AA
59037126
1638 rs10027676 4 AA AG AG GG AG
AG AG AG AG AA AA AG AG
AA AG AA AA AA AG AG AG
AG GG GG GG AG AG AG GG
AG AA AG AG AG AA AG
55045716
1639 rs10027697 4 GG GG GG AG AG
AG AG GG GG GG GG AG AG
AG GG AG AG AA GG GG GG
GG GG GG GG AG AA AA AA
AG AG AG AA AG GG AG
7126063
1640 rs10027730 4 AA AA AA AA AA
AA AA AG AG AG AA AA AA
AA AA AA AA AA AA AA AA
AG AA AG AA AA AA AA AA
AG AA AA AA AA AA AA
62995033
1641 rs10027783 4 GG GG GG GG GG

GG GG GG GG GG GG GG GG
GG AG GG GG GG GG GG GG
GG GG GG GG GG GG GG GG
GG GG GG GG GG GG GG
121594207
1642 rs10027815 4 AA AA AA AA AG
AG AA AA AA AA AA AA AA
AA AG AG AA AA AA AA AA
AA AA AA AA AA AA AA AA
AA AA AA AA AA AA AA
108691131
1643 rs10027817 4 AA AA AA AA AG
AG AG AG AA AA AA AA AA
AA AA AG AA AG AA AA AA
AA AG AG AG AA AA AA AA
AA AG AG AA AG AG AA
69829222
1644 rs1002784 1 GG GG GG GG GG
GG GG GG GG GG GG GG GG
GG GG GG GG GG GG GG GG
GG GG GG GG GG AG GG GG
GG GG GG GG GG GG GG
76824595
1645 rs10027850 4 GG GG GG GG GG
GG GG GG GG GG AG AG AG
AG GG GG GG GG AG AG AG
AG GG GG GG AG GG AG AG
AA GG GG GG GG AG GG
165995106
1646 rs10027878 4 AA AG AA AA AG
AA AG AA AA AA AG AG AG
GG AG AA AA AG AA AA AA
AA AA AA AA AG AA AA AA
AA AA AG AA AG AA AG
189876706
1647 rs10027928 4 AC AC AA CC AC
CC AC AC AA AC AA AA AA
AA AA AC AA AA AA AA AC
AA AC AC AC AA AC AA AC
CC AA AC AA AA AA AA
176541897
1648 rs1002793
X -- -- -- -- -- -- -- --
-- -- -- -- -- -- -- --
-- -- -- -- -- -- -- --
-- -- -- -- -- -- -- --
-- -- -- -- 22152783
1649 rs1002796 2 GG GG GG GG GG
GG GG GG GG GG GG GG GG
GG GG GG GG GG GG GG GG

GG GG GG GG GG GG GG GG
GG AG GG AG GG GG GG
43314843
1650 rs10028007 4 CC CC CC CC CC
CC CC AC AC CC CC CC CC
CC CC CC CC CC CC CC CC
CC CC CC CC AC CC CC AC
CC AC CC CC AC CC AC
52997933
1651 rs1002807 5 AG GG AG GG GG
GG GG AG GG AG GG GG GG
GG GG AG GG AG AG AG GG
GG GG AG AG AG AA AA AA
GG AG AG AG GG GG GG
144528984
1652 rs10028130 4 AG AG AA AA AA
AA AA AG AG AG GG AG AG
AA AA GG AG AG AG AG AA
AA AA AA AA AG AG AG AG
AA AG GG GG AA AG AG
65517439
1653 rs10028138 4 AG AA AG GG AG
AG AG AG AG AG GG GG GG
GG AG GG GG GG GG GG GG
AG AG AG AG AG GG AG AG
GG AG GG AG AG GG AG
157530667
1654 rs10028155 4 AC AA AA AC AC
AA CC AA AA AA AC AC AC
AA AC CC AC AC AC AC CC
CC CC CC AC CC CC CC CC
CC AC CC CC AC AC AC
47906812
1655 rs10028163 4 AA AG AA AA AA
AG AA AA AA AA AG AG AG
AG AG AA AA AA AG AG AG
AA AA AA AA AA AA AA AA
AG AA AA AA AG AG AG
138470185
1656 rs10028171 4 GG AG GG AG AA
AA AG AA AA AG AG AA AA
AA AA AA AA AA AG AG AG
AA AA AA AA AG AG AG AG
AG AG AA AA GG AG AG
27735936
1657 rs10028182 4 GG GG GG GG AG
GG AG GG AG GG GG GG AG
AG GG AG AA AA GG GG GG
GG GG GG GG AG GG AG AG
GG GG GG GG AG AG GG

153701640								
1658	rs10028206		4	GG	GG	GG	GG	GG
	GG	GG	GG	GG	GG	GG	AG	GG
	GG	AG	AG	AG	GG	GG	GG	GG
	GG	GG	GG	GG	GG	GG	GG	GG
	GG	GG	GG	GG	GG	GG	GG	
37716343								
1659	rs10028207		4	AA	AA	AC	AC	AC
	CC	AC	AC	CC	CC	AC	AC	AC
	AA	AC	AC	AA	AC	AC	AC	AC
	AC	AC	AC	AC	CC	CC	CC	CC
	AA	AC	CC	AC	AC	CC	AC	
75967200								
1660	rs10028215		4	AG	AG	AG	GG	AG
	AG	AG	AA	AA	AA	AG	AA	AG
	GG	AG	GG	AG	AG	AG	AG	GG
	AG	AA	AA	AA	GG	AG	AG	GG
	AA	AG	AG	AA	GG	GG	AG	
79445470								
1661	rs1002822		14	AA	AG	AA	AA	AG
	AA	AG	AA	AG	AA	AA	AA	AA
	AA	AA	AA	AA	AA	AG	AA	AA
	AA	AA	AA	AA	AG	AG	GG	AA
	AG	AA	AA	AA	GG	AG	AG	
32777753								
1662	rs10028269		4	CC	CC	CC	CC	CC
	AC	CC	CC	CC	CC	CC	CC	CC
	AC	CC	AC	AC	AC	AC	AC	AC
	AC	AC	CC	AC	CC	CC	CC	CC
	CC	CC	CC	CC	AC	AC	CC	
189315208								
1663	rs10028296		4	GG	GG	GG	GG	GG
	GG	GG	GG	GG	GG	AA	AG	AG
	AG	AG	GG	GG	GG	GG	GG	GG
	GG	GG	GG	GG	AG	GG	AG	GG
	GG	AG	GG	GG	GG	AG	AG	
58895042								
1664	rs10028398		4	CC	CC	CC	CC	CC
	CC	CC	CC	CC	CC	CC	CC	CC
	CC	CC	CC	CC	CC	CC	CC	CC
	CC	CC	CC	CC	CC	CC	CC	CC
	CC	CC	CC	CC	CC	CC	CC	
721969								
1665	rs10028441		4	GG	GG	AG	AG	GG
	AG	GG	AG	AG	AG	GG	GG	GG
	GG	GG	GG	GG	GG	AG	AG	AG
	GG	AG	AG	GG	GG	GG	GG	GG
	AG	GG	GG	GG	AG	AA	GG	
88290265								
1666	rs10028469		4	AC	CC	CC	CC	CC

	CC	CC	AC	AA	AC	CC	CC	CC
	CC	CC	AC	CC	AC	AA	AA	AC
	CC	CC	CC	CC	CC	AC	AC	CC
	AC	CC	CC	CC	CC	CC	CC	
10909225								
1667	rs10028579		4	AG	AG	AG	AA	AA
	AA	AA	AA	AA	AA	AA	AG	AA
	AA	AG	AA	AG	AG	GG	GG	AG
	AA	AA	AA	AA	AA	GG	AG	AG
	AA	AA	AA	AA	AA	AA	AA	
28450862								
1668	rs10028728		4	GG	GG	GG	AG	AG
	AA	GG	GG	GG	GG	GG	AG	GG
	GG	GG	AA	AA	AA	AG	AG	AG
	GG	AG	GG	GG	GG	AG	GG	AG
	AG	GG	GG	GG	AG	AG	AG	
14082952								
1669	rs10028794		4	AG	GG	AG	GG	GG
	AG	GG	GG	GG	GG	AA	AG	AG
	AG	AG	AG	AG	AG	AG	GG	AG
	AG	AG	AG	AG	AA	AG	AA	AG
	AG	GG	GG	GG	AG	AG	AG	
148510474								
1670	rs10028915		4	GG	GG	GG	GG	AG
	AG	GG	AG	AG	GG	GG	GG	AG
	AG	AG	GG	GG	GG	GG	GG	GG
	AG	GG	GG	AA	AG	GG	AG	AG
	GG	AG	GG	AG	AA	AG	AG	
29953808								
1671	rs10028939		4	CC	CC	CC	CC	CC
	CC	CC	CC	CC	CC	CC	CC	CC
	CC	CC	CC	CC	CC	CC	CC	CC
	CC	CC	CC	CC	CC	CC	CC	CC
	CC	CC	CC	CC	CC	CC	CC	
56162773								
1672	rs1002897		2	GG	GG	GG	GG	GG
	GG	GG	GG	GG	GG	GG	GG	GG
	AG	AG	GG	GG	GG	GG	GG	GG
	GG	GG	GG	GG	GG	GG	GG	GG
	GG	AG	GG	AG	AG	GG	AG	
66464350								
1673	rs1002898		8	GG	GG	GG	GG	GG
	GG	GG	GG	GG	GG	GG	GG	GG
	GG	GG	GG	GG	GG	GG	GG	GG
	AG	GG	AG	GG	GG	GG	GG	GG
	AG	GG	GG	GG	AG	GG	AG	
122226775								
1674	rs10029070		4	AA	AG	GG	AG	GG
	AG	AG	AA	AA	AG	AA	AG	AG
	AA	AG	AA	AG	AG	AG	AG	AG

	GG	GG	GG	GG	AG	AA	AA	AG
	AG	AA	AG	AG	AG	AA	AG	
54851083								
1675	rs10029104		4	AA	AA	AA	AA	AA
	AA	AA	AA	AA	AA	AA	AA	AA
	AA	AA	AG	GG	AG	AG	AA	AA
	AA	AA	AA	AA	AA	AA	AA	AA
	AA	AA	GG	AG	AA	AA	AA	
514629								
1676	rs10029165		4	AA	AG	AG	AG	AG
	AG	AG	AG	AG	AG	AA	AA	AG
	AG	AG	AG	AG	AA	AA	AA	AA
	AG	AG	AG	AG	AG	AG	AG	AG
	GG	AG	AG	AG	AG	AA	GG	
27975847								
1677	rs10029207		4	GG	AG	AG	AA	AA
	AA	AA	GG	AG	AG	GG	GG	GG
	GG	GG	AG	AG	GG	AG	GG	AG
	GG	GG	GG	GG	GG	AA	AG	AG
	AG	AA	GG	AG	GG	AG	GG	
63878877								
1678	rs10029225		4	AA	AA	AA	AG	AG
	AA	AG	AG	AG	AA	AA	AA	AA
	AA	AA	AA	AG	AG	AA	AA	AA
	AA	AA	AA	AA	AA	GG	AG	AG
	AA	AA	AA	AA	AA	AA	AA	
125732734								
1679	rs10029290		4	AA	AA	AC	AA	AC
	AA	AC	AC	AA	AC	AA	AA	AC
	AC	AC	AC	CC	CC	AC	AC	AA
	AC	AC	CC	AC	CC	AA	AC	AC
	AC	AA	CC	AC	AA	AA	AA	
184270907								
1680	rs10029388		4	GG	GG	AG	GG	GG
	GG	GG	GG	GG	GG	GG	AG	AG
	AG	AG	AG	AG	AA	GG	GG	GG
	AG	AG	AG	AG	GG	AG	GG	AG
	GG	AG	AG	AG	GG	GG	GG	
120029841								
1681	rs10029407		4	AA	AA	AA	AA	AA
	AA	AA	AA	AA	AA	AA	AA	AA
	AA	AA	AA	AA	AA	AA	AA	AA
	AA	AA	AA	AA	AA	AA	AA	AA
	AA	AA	AA	AA	AA	AA	AG	
104426089								
1682	rs10029429		4	GG	GG	GG	AG	AG
	AG	AG	AG	AA	AG	AG	AG	GG
	GG	GG	GG	GG	GG	AG	AG	AG
	GG	GG	GG	AG	GG	AG	AG	GG
	GG	GG	GG	GG	AG	AG	AG	

184248133							
1683	rs1002957	14	CC	CC	CC	CC	CC
	CC	CC	CC	CC	CC	CC	CC	CC
	CC	CC	CC	CC	CC	CC	CC	CC
	CC	CC	CC	CC	CC	CC	CC	CC
	CC	CC	CC	CC	CC	CC	CC	
102644071							
1684	rs1002960	9	AA	AA	AA	AC	AA
	AC	AA	CC	AA	CC	AA	AC	AC
	AC	AC	AA	AA	AA	AA	AA	AA
	CC	AC	AC	AA	AA	AA	AA	AA
	AA	AC	CC	AC	AC	AA	AC	
88839915							
1685	rs1002966	1	AG	AA	AG	AA	AA
	AA	AA	AA	AA	AA	AG	AG	AG
	AA	AA	AA	AG	AG	AG	AG	AA
	AA	AA	AA	AA	AA	AA	AA	AA
	AA	AA	AA	AA	AA	AA	AA	
6542330							
1686	rs10029664	4	GG	GG	GG	GG	GG
	GG	GG	GG	GG	GG	GG	GG	GG
	GG	GG	GG	GG	GG	GG	GG	GG
	AG	AG	AG	GG	AA	GG	AG	AG
	AG	GG	GG	GG	GG	GG	AG	
46635167							
1687	rs10029689	4	AA	AA	AA	AA	AA
	AA	AA	AG	AG	AG	AG	AG	AG
	AA	AA	AA	AA	AA	AA	AG	AA
	AA	AA	AA	AA	AA	AA	AA	AA
	AA	AA	AA	AA	AA	AA	AA	
1533976							
1688	rs10029732	4	AA	AA	AG	AG	GG
	AG	GG	AG	AG	AA	AA	AA	AA
	AG	GG	AG	GG	AG	AA	AA	AA
	AG	AA	AA	AA	AA	AA	AA	AA
	AA	AG	AA	AA	AG	AG	AA	
48498679							
1689	rs10029781	4	AA	AC	CC	CC	AC
	CC	AC	CC	CC	CC	AC	AC	AC
	AC	AA	CC	AC	AC	AA	AA	AC
	AA	AA	AA	AA	AC	AA	AA	AA
	AA	--	AC	AC	AA	AC	AA	
158361729							
1690	rs10029809	4	GG	GG	GG	GG	GG
	GG	GG	GG	GG	GG	GG	GG	GG
	GG	GG	GG	GG	GG	AG	AG	AG
	GG	GG	GG	GG	GG	GG	GG	GG
	AG	GG	GG	GG	GG	GG	GG	
184763771							
1691	rs10029872	4	GG	GG	GG	AG	GG

AG AG AG AG GG AA AG AG
AG AG AG GG AG GG GG GG
GG GG GG GG AG AG AG AA
AG AG GG GG AG AG AG
40350763
1692 rs10029924 4 AA AA AA AA AA
AA AA AA AA AA AA AA AA
AA AA AA AA AA AA AA AA
AA AA AA AA AA AA AA AA
AA AA AA AA AA AA AA
620209
1693 rs10029948 4 GG AG AG GG GG
GG GG GG AG GG GG GG GG
GG AG AG AA AG GG GG AG
AG AG AG AG GG GG GG GG
GG GG GG GG AA AG AG
6454335
1694 rs10029966 4 AC AA AA AA AA
AC AA AA AC AA AA AA AA
AC CC AC AC AA AC AA AC
AC AC AC AC AA AC AC AA
AA AA AC AC AA AC AC
4737746
1695 rs10029975 4 AC AA AC AC AA
AA AC AA AA AA CC CC AC
AC CC AA AA AA AC AC CC
AC AA AA AC AA AC AA AA
AA CC CC CC AA AC AC
31324283
1696 rs10029979 4 AA AA AA AA AA
AA AA AA AA AA AA AA AA
AA AA AA AA AA AA AA AA
AA AA AA AA AA AA AA AA
AA AA AA AA AA AA AA
58685966
1697 rs10029981 4 AA AA AA AC AA
AA AC AA AA AA AA AA AA
AC AA AA AA AA AA AA AA
AA AA AA AA AA AA AA AA
AA AA AA AA AA AA AA
186335706
1698 rs10030005
4 -- -- -- -- -- -- -- --
-- -- -- -- -- -- -- --
-- -- -- -- -- -- -- --
-- -- -- -- -- -- -- --
-- -- -- -- 62953487
1699 rs1003001 3 AA AA AA AA AA
AA AA AG AG AG AA AA AA
AA AA AA AA AA AA AA AG

AA AG AG AA AA GG AG AG
GG GG AA AG AG AA GG
95426687
1700 rs10030212 4 AA AC CC AC AA
AC AC CC CC AC AC CC AC
AC AC AC AC AC CC CC CC
AC AC AC AC AA AC AC AA
CC CC AC CC CC CC AC
164510545
1701 rs10030322 4 AA AG GG AG AA
AG AG AG AG AG GG GG AG
AG AG AG AG AG GG GG GG
AA AA AA AA AA AG AG AA
GG GG AG GG AA AG AA
164540801
1702 rs10030326 4 AA AA AA GG GG
GG GG AG AG AA AG AG AG
AG GG AG GG AG AG AG GG
GG GG GG GG GG AG GG AG
AG AA AG AG GG GG AG
157235050
1703 rs1003047 9 CC AC AC CC CC
CC CC CC CC CC CC AC CC
AC CC CC CC CC AC CC CC
CC CC CC CC CC CC CC CC
CC CC CC CC CC CC AC
2610307
1704 rs10030493 4 GG GG AG GG GG
GG GG GG AG GG GG GG GG
GG GG GG AG AG GG GG GG
GG GG GG GG AG GG GG GG
GG GG GG GG GG GG GG
4670196
1705 rs10030663 4 AA AA AG GG AG
GG AG AA AA AA AA AA AA
AA AG AA AG AG GG AG AG
AA AA AA AA AA GG AG AG
AG GG AA AG AA AA AA
162995995
1706 rs10030781 4 AG GG GG AG AA
AG AG GG GG GG AA AA AG
AG GG AG AG GG GG GG GG
AG AG AG GG GG GG GG GG
AG AA AG AA GG GG AG
46025913
1707 rs10030903 4 AA AA AA AA AA
AA AA AC AC AA AA AA AA
AA AA AA AA AA AA AA AA
AA AA AA AA AA AA AA AA
AA AA AA AA AA AA AA

110165842							
1708	rs10030915	4	GG	GG	GG	AG	AG
	AG	GG	GG	GG	AG	GG	AG	GG
	GG	AG	AG	AA	AG	AG	AG	GG
	AG	AG	AG	AG	AG	GG	GG	GG
	AG	GG	GG	GG	AG	AG	GG	
158634769							
1709	rs10030918	4	GG	GG	GG	GG	GG
	GG	GG	GG	GG	GG	AG	AG	AG
	AG	GG	GG	GG	GG	GG	GG	GG
	GG	GG	GG	GG	GG	GG	GG	GG
	GG	GG	GG	GG	GG	GG	GG	
46520282							
1710	rs10031105	4	AA	AA	AG	AA	AA
	AA	AA	AA	AA	AA	AA	AA	AA
	AA	AA	AA	AA	AA	GG	GG	AG
	AA	AA	AA	AA	AA	AA	AA	AA
	AA	AA	AA	AA	AA	AA	AA	
56609872							
1711	rs10031140	4	CC	CC	CC	CC	CC
	CC	CC	CC	CC	CC	CC	CC	CC
	CC	CC	CC	CC	CC	CC	CC	CC
	CC	CC	CC	CC	CC	AC	AC	AC
	CC	CC	CC	CC	CC	CC	CC	
61914877							
1712	rs10031177	4	AA	AA	AA	AA	AA
	AA	AA	AA	AA	AA	AA	AA	AA
	AA	AA	AG	AA	AG	AG	AA	GG
	AA	AA	AA	AA	AA	AA	AA	AA
	AG	AA	AA	AA	AA	AA	AA	
91941942							
1713	rs10031210	4	AG	AA	AG	AG	AG
	AA	AG	AG	AG	AG	GG	GG	GG
	GG	GG	AG	GG	AG	AG	AG	AA
	GG	AG	AG	AG	AG	AG	AG	AG
	GG	AG	AA	AG	AG	GG	AA	
102714886							
1714	rs10031277	4	AA	AA	AA	AA	AG
	AG	AG	AG	GG	AG	AG	GG	AG
	AG	AA	AG	AG	AA	AA	AA	AG
	AG	AA	AG	AG	AG	AA	AG	AG
	AA	AA	GG	AG	GG	GG	AG	
14931835							
1715	rs10031344	4	GG	GG	GG	GG	GG
	GG	GG	GG	GG	GG	GG	GG	GG
	GG	GG	GG	GG	GG	GG	GG	GG
	GG	GG	GG	GG	GG	GG	GG	GG
	GG	GG	GG	GG	GG	GG	GG	
70328958							
1716	rs10031400	4	AC	AA	AA	AC	AC

	AA	AC	AC	AA	AA	AC	AC	CC
	CC	AC	CC	CC	CC	AA	AA	AA
	AA	AA	AA	AA	AA	AC	AC	AA
	AC	AA	AA	AA	AC	AC	AA	
130264384								
1717	rs10031409		4	AA	AA	AA	AA	AA
	AA	AA	AA	AA	AA	AA	AA	AA
	AA	AA	AA	AA	AA	AA	AA	AA
	AA	AA	AA	AA	AA	AA	AA	AA
	AA	AA	AA	AA	AA	AA	AA	
47456194								
1718	rs10031442		4	GG	GG	GG	AA	AA
	AA	AA	AG	AA	AG	AG	AG	AA
	AA	AA	AG	AG	AG	AA	AA	AG
	AA	AA	AG	AG	AA	GG	AG	AG
	AA	AA	GG	AG	AA	AA	AA	
186816480								
1719	rs10031516		4	AG	AG	AA	AA	AG
	AA	AG	AG	AG	GG	GG	GG	GG
	GG	AG	AG	GG	GG	AA	AA	AG
	AA	AA	AA	AA	AG	AA	AG	AG
	AG	AG	AA	AA	AG	AG	AA	
161000801								
1720	rs10031583		4	AA	AA	AA	AA	AA
	AA	AC	AC	AC	AC	AA	AA	AA
	AC	CC	AC	AC	AA	AA	AA	AC
	AC	AA	AC	AA	AC	AA	AA	AC
	AC	AC	AA	AA	AA	AA	AA	
70868585								
1721	rs1003167		6	AC	AC	AA	CC	AC
	CC	AC	AA	AA	AC	CC	AC	AC
	AA	AC	AC	AA	AC	AC	AC	AA
	AA	AA	AA	AA	CC	AC	CC	CC
	AC	CC	CC	CC	CC	AC	CC	
43855023								
1722	rs1003174		4	AG	AA	AG	AA	AA
	AA	AA	AG	AG	AG	AG	AA	AA
	AG	AG	AA	AG	AG	AA	AA	AA
	AG	AG	AG	GG	AA	AA	AA	AA
	AG	AA	AA	AA	AA	AA	AA	
99617267								
1723	rs10031771		4	AG	GG	AG	AG	AG
	AG	GG	AG	GG	GG	AG	GG	AG
	AG	GG	AG	GG	GG	AG	AG	GG
	AA	AA	AA	AA	AG	AA	AA	AA
	AA	GG	AG	AG	GG	GG	AG	
132051076								
1724	rs10031772		4	AC	AA	AC	AA	AA
	AA	AC	AA	AA	AC	AA	AA	AA
	AA	AC	AC	AA	AC	AA	AA	AA

AA AA AA AA AA AA AA AA
AA AC AA AA AA AA AA
41099333
1725 rs10031780 4 AG AA AA AA AA
AA AA AA AA AG AG AA AA
AA AG AG AG AG AA AG AA
AG AA AA AG AA AA AA AA
AG AA AA AA AA AG AA
122394649
1726 rs10031846 4 GG GG GG GG AG
AG GG AG AG GG GG GG GG
GG GG AG AG GG AG GG AA
GG GG GG GG GG GG GG GG
GG GG GG GG GG GG GG
92047321
1727 rs10031866 4 AC AC CC CC AC
AC AC CC CC CC AA AC AA
AC CC CC AC AC AC AC AC
AA AC AC AC CC AA AC AC
AA CC CC CC AC AC AC
54489838
1728 rs1003189 12 GG AG AG GG AG
AG GG AA GG GG AG GG GG
GG GG GG GG GG AG AG GG
AG AG AG AG GG GG GG GG
GG GG GG GG GG GG GG
20009179
1729 rs10031898 4 AG AA AA AG AG
AG AG AA AA AA AG AG AG
AG AA AG GG AG GG GG AG
AG AG AG AA AG AG AG AG
AA AA AG AG AA AA AA
26499696
1730 rs10031975 4 GG GG GG GG GG
GG GG AG GG GG AG AG AA
AG AA GG GG GG GG GG GG
GG GG GG GG GG GG GG GG
AG GG GG GG GG GG AG
186886395
1731 rs10032050 4 AG AG AG AG GG
AG GG GG GG AG AG AA AA
AA AA AG AG GG AG AG GG
AG AG AG GG AG GG AG GG
GG AG AG AG GG GG GG
93641094
1732 rs10032088 4 GG GG GG GG GG
GG GG GG GG GG GG GG GG
GG GG GG GG GG GG AG GG
GG GG GG GG GG GG GG GG
GG GG GG GG GG GG GG

147215026
1733 rs10032135 4 AA AA AA AA AA
AA AA AA AA AA AA AA AA
AA AA AA AA AA AC AC AA
AA AA AA AA AC AA AC AA
AA AA AC AA AC AA CC
65403406
1734 rs10032150 4 AA AA AG AA AG
AG AG AA AA AG GG AA AG
GG GG AA AG AA AG AG AA
AA AG AG AA AA AA AA AA
GG AA GG AG AA AA AG
111646618
1735 rs10032205 4 AA AA AG AG AG
AA GG AA AG AA AA AA AA
AA AA GG AG AG GG GG AG
AG AG AG AG AG GG GG GG
AA AG AG GG AG AA GG
181572734
1736 rs10032212 4 GG GG GG AG AG
AG GG GG GG GG GG GG AG
AG GG GG GG GG GG GG GG
GG GG GG GG GG GG GG GG
AA GG AG AG GG GG AG
153537671
1737 rs1003226 7 AA AA AA AA AA
AA AA AA AA AA AA AG AG
AG AA AA AA AA AA AA AA
AA AG AA AA AA AA AA AA
AA AA AA AA AA AA AA
135046552
1738 rs10032274 4 AG AG GG AG AA
AG AA AG AG AA AA AA AG
AG AA AG AG AA GG AG AG
AG AG AG AG AG AA AA AG
AG AA AG AA AG GG AG
96206860
1739 rs1003231 14 AG AA AA AA AA
AA AA AA AA AA AA AA AA
AA AA AA AA AA AG AG GG
AA AG AA AA AA AG AG AA
AA AG AG AA AA AA AA
98171325
1740 rs10032478 4 AA AA AG AA AG
AG AA GG GG AG AG AG AA
AA AG AG AG GG AA AA AA
AG AA AA AG AA AG AA AA
GG GG AG AG GG AG GG
31777553
1741 rs10032493 4 CC CC CC CC CC

CC CC AC AC AC CC CC CC
CC CC CC CC CC CC CC CC
AC AC AC CC AC CC AC AC
CC CC CC CC CC CC CC
188461119
1742 rs1003250 6 GG AG GG GG AA
AG AG AG AG AA AG AG GG
GG GG GG GG GG AA AA AG
AA AA AA AG GG AA AG AG
AG GG GG GG AA AG AG
36437412
1743 rs10032518 4 AG AA AA AG AG
AA GG AG AG AG AA AA AA
AA AA AA AA AA AG AG AG
AA AA AA AA AA AA AA AA
AA AA AG AA AA AG AA
5508665
1744 rs1003258 14 AA AA AA AA AA
AA AA AG AA AG GG GG GG
AG AA AA AA AA AA AA AA
AG AA AG AG AA AA AA AA
AA AA AA AA AA AA AA
81852849
1745 rs10032600 4 AG AG AA GG GG
GG GG AG AG AG AA AA AA
AG AG AA AA AA AG AG AG
AA AG -- AG AA GG AG AG
AA AG AA AG AG AA AG
45025291
1746 rs10032631 4 AG GG GG AG AA
AG AG GG GG GG AA AA AA
AG GG AG AG GG GG GG GG
AG AG AG GG AG GG GG AG
AG AA AG AA GG GG AG
46049120
1747 rs1003271 4 AG AG AG AG AG
AG AG GG GG GG GG AG AG
AG GG AG AG AA AG AG AG
GG GG GG GG AG GG GG GG
AG GG GG GG AG AA AG
11224411
1748 rs10032784 4 AG AG AG GG AG
GG AG AG AG AG AA AA AA
AG AA AG AG AG AG AG AG
AG AG AA AA AG GG GG GG
AA AG AG GG AA AG AG
190268759
1749 rs1003301 12 AA AA AA AA AG
AG AA AA AA AA AA AA AA
AA AA AA AA AA AA AA AA

AA AA AA AA AA AA AA AA
AA AA AA AA AA AA AA
109820777
1750 rs10033012 4 AG AG AG AG GG
GG AG GG GG GG AA AG AG
AG GG AG GG GG GG GG GG
GG GG GG GG GG GG GG GG
AG GG GG GG GG GG GG
163766726
1751 rs10033032 4 GG GG AG GG GG
GG GG GG GG GG GG GG GG
GG GG GG GG GG AG AG GG
GG GG GG GG GG GG GG GG
AG AG GG AG AA AA AG
7434017
1752 rs10033034 4 GG GG GG AG GG
AG GG AA AA AA GG GG GG
GG GG AG AG AG AG AG AA
AG AG AG GG GG AG AG GG
AG AG AG GG GG GG GG
179899458
1753 rs10033043 4 AA AA AA AA AA
AA AA AA AA AA AA AA AA
AA AA AA AA AA AA AA AA
AA AA AA AA AA AA AA AA
AA AA AA AA AA AA AA
182530821
1754 rs10033110 4 GG GG AG GG GG
GG GG AG AG AG AG AG AG
AG AG GG GG AG AG AG AA
AG AG GG AG GG AG GG AG
AG AA GG AG AA AG AA
187845057
1755 rs10033212 4 GG GG AG GG GG
GG GG AG AG AG GG GG AG
AG AG GG GG GG GG GG GG
GG GG GG GG GG GG GG GG
AG GG AG AG AG GG AA
28090064
1756 rs10033248 4 AC AA AA AA AA
AA AC AA AA AC AA AA AA
AA AC AA AA AA AA AA AA
AA AA AA AA AC AA AA AC
AA AA AA AA AC AC AA
41030604
1757 rs10033307 4 AC AC CC AC AA
AC AA AC CC CC CC CC CC
CC AC CC AC AC AC AC CC
AC AC AC AA AC CC CC AC
AA AC AC AA AC CC AA

79304226								
1758	rs10033316		4	AA	AG	AG	AA	AA
	AA	AA	AA	AA	AA	AA	AA	AA
	AA	AA	AG	AA	AA	AA	AA	AG
	AA	AG	AG	GG	AG	AG	AG	AA
	AA	AG	AA	AA	GG	AG	AG	
172112864								
1759	rs10033393		4	AC	AA	AC	AC	AC
	AA	AC	AC	AA	AA	AC	AC	AC
	AA	AC	CC	CC	CC	AA	AC	AA
	AA	AA	AA	AA	AA	AA	AA	AA
	AA	AA	AA	AA	AA	AA	AC	
66260257								
1760	rs10033422		4	AG	AA	AG	AG	GG
	AG	GG	AG	AG	AG	AG	AG	AG
	AA	AG	AG	GG	AG	AG	AG	AA
	AA	AA	AA	AG	GG	GG	GG	GG
	AG	AG	AG	AG	AA	AG	AG	
58027081								
1761	rs1003346		9	CC	AC	CC	AC	CC
	AC	AC	AA	AA	CC	AC	CC	AC
	AC	AA	AC	AC	AA	CC	CC	CC
	AC	AC	AA	AC	AC	AA	AC	AC
	AA	CC	AC	CC	AC	CC	AC	
111815340								
1762	rs10033567		4	GG	AG	AG	AG	AG
	GG	AA	GG	AG	AG	GG	GG	GG
	AG	AG	GG	GG	GG	GG	GG	AG
	GG	GG	GG	GG	GG	GG	GG	GG
	GG	GG	GG	GG	AG	AG	GG	
81253388								
1763	rs10033596		4	AC	AA	AC	AA	AA
	AA	AA	AC	AA	AA	AA	AA	AA
	AC	CC	CC	CC	CC	CC	CC	CC
	AC	CC	CC	AC	AC	AC	AC	AC
	AC	AC	AA	AC	AC	AA	AC	
80887556								
1764	rs10033629		4	AG	AA	AA	AA	AA
	AG	AA	AG	AG	AA	AG	AG	AG
	AG	AA	GG	GG	GG	AA	AA	AA
	AG	AG	AG	AG	AA	AG	AA	AG
	AA	AA	GG	AG	AG	AG	AA	
155826061								
1765	rs10033652		4	AG	AG	AG	AG	GG
	AG	AG	AG	GG	GG	GG	GG	GG
	AG	AA	GG	GG	GG	AG	AA	AG
	AA	AG	AG	AG	AG	AA	AG	AA
	GG	AG	GG	AG	AA	AG	AA	
82493067								
1766	rs10033688		4	GG	GG	GG	GG	GG

GG GG GG GG GG GG GG GG
GG GG GG GG GG GG GG GG
GG GG GG GG GG GG GG GG
GG GG GG GG GG GG GG
185376403
1767 rs10033691 4 AG AG AA AA AA
AA AG AA AA AA AA AA AA
AA AA AA AG AA AA AA AA
AG AA AA AA AA AA AA AA
AA AG AG GG AA AA AG
110995710
1768 rs10033742 4 AG AG AA AA AG
AA AG AA AA AA AA AA AG
AG AG AG AG AG AG AG AG
AA AA AA AA AA AA AA AA
AA AA AA AA AG AG AG
176825221
1769 rs1003385 12 AA AG AG AG AG
AG AG GG AG GG AG AG GG
GG GG GG GG GG GG AA GG
GG GG GG GG AG AG GG AG
GG AG AG AA AG AG AG
115751903
1770 rs10033887 4 AC AC CC AC CC
AC AC AA AA AC AA AA AA
AA AA AC AC CC AC AC CC
AA AC AC AA CC CC CC CC
AC AC AC CC AC AC CC
116927517
1771 rs10033893 4 GG GG GG GG AG
AG GG GG GG GG GG GG GG
GG AG AG GG GG GG GG GG
GG GG GG GG GG GG GG GG
GG GG GG GG GG GG GG
108726396
1772 rs10033894 4 AA AA AA AA AG
AG AA AA AA AG AA AA AA
AG GG AG AA AA AG AG AG
AG AA AA AA AA GG AG AG
AG AA AA AA AA AA AG
101118465
1773 rs10033900 4 AA AG AG AA AA
AA AG AG AG AG AG AA AG
GG GG GG GG AG GG GG GG
AG AA AA AA AG AG AG AG
AG AG AG AA AA AA AA
110659067
1774 rs10033940 4 AA AC AC AC CC
AC CC AC AC CC AC CC CC
CC CC AA AA AA AC AC AC

	CC	CC	CC	AC	AC	CC	AC	AC
	AA	AC	AA	AA	CC	AC	CC	
22161298								
1775	rs10034000		4	AG	AG	AA	GG	GG
	GG	GG	GG	AG	GG	AG	AG	AG
	AG	AA	AG	AG	AG	AA	AA	AA
	AA	AG	AA	AA	AG	AG	AG	GG
	AG	AA	AA	AA	AA	AA	AA	
153569094								
1776	rs10034047		4	AC	AC	CC	AC	AC
	CC	AC	AC	AC	AC	AC	AC	AC
	AC	CC	CC	CC	CC	AC	AC	CC
	CC	CC	CC	CC	CC	CC	CC	CC
	AC	CC	CC	CC	CC	CC	CC	
11127123								
1777	rs10034052		4	GG	GG	GG	GG	GG
	GG	GG	GG	GG	GG	GG	GG	GG
	GG	GG	AG	GG	AG	GG	GG	GG
	GG	GG	GG	GG	GG	AG	GG	GG
	GG	GG	GG	GG	GG	GG	GG	
53506457								
1778	rs10034074		4	GG	GG	GG	GG	GG
	GG	GG	AG	GG	AG	GG	GG	GG
	GG	GG	AG	AG	AG	GG	GG	AG
	GG	GG	GG	GG	GG	AA	AG	AG
	AG	GG	GG	GG	AG	AG	GG	
143863997								
1779	rs10034152		4	GG	AG	GG	GG	GG
	GG	GG	GG	GG	AG	GG	AG	GG
	GG	GG	GG	GG	GG	GG	GG	AG
	AG	GG	GG	AG	AG	GG	GG	GG
	GG	GG	GG	GG	GG	GG	GG	
124847477								
1780	rs10034168		4	CC	CC	CC	AC	CC
	AC	CC	CC	CC	CC	AA	AC	AC
	CC	CC	CC	CC	CC	CC	CC	CC
	CC	CC	CC	CC	CC	CC	CC	CC
	CC	AC	CC	AC	CC	CC	CC	
78280329								
1781	rs10034180		4	AG	AG	AG	AG	AG
	AA	GG	GG	GG	GG	AG	GG	AG
	AG	GG	AG	GG	AG	AG	AG	AA
	GG	GG	GG	AG	AG	AG	GG	AG
	GG	AG	AG	AG	AG	GG	AG	
10518525								
1782	rs10034329		4	AA	AA	AA	AA	AA
	AA	AA	AG	AA	AG	AG	AG	AG
	AG	AA	AA	AA	AA	AA	AG	AA
	AA	AA	AA	AA	AA	AA	AA	AA
	AA	AG	AA	AG	AA	AA	AG	

150902638								
1783	rs10034371	4	AA	AA	AA	AA	AA	
	AA	AA	AG	AG	AG	AA	AA	AG
	AG	AG	AG	AA	AA	AG	AG	AG
	GG	GG	GG	GG	AG	GG	AG	AG
	AA	GG	AG	AG	GG	GG	AG	
183422459								
1784	rs10034384	4	AG	AA	AG	AA	AA	
	AG	AA	AA	AA	AA	AA	AG	AG
	AG	AA	AA	AG	AG	AG	AG	AG
	AA	AA	AA	AA	AA	AA	AA	AA
	AA	AA	GG	AG	AA	AA	AG	
11235941								
1785	rs10034495	4	AA	AA	AA	AG	AG	
	GG	AA	AG	AG	AG	GG	AG	GG
	GG	AG	AG	GG	GG	GG	GG	AG
	AG	AG	AG	AG	GG	AG	AG	GG
	AG	GG	AG	AG	AG	AA	GG	
164411073								
1786	rs10034556	4	GG	GG	GG	AG	GG	
	AG	GG	AG	AG	GG	GG	GG	GG
	GG	GG	AG	AG	AG	AG	GG	AG
	AG	AA	AA	AG	AG	AG	AA	GG
	AG	AA	AG	AG	AG	AA	GG	
113449650								
1787	rs10034640	4	AG	AG	AA	AA	AA	
	AA	AA	AA	AA	AA	AA	AA	AG
	AG	AA	AA	AA	AA	AA	AA	AA
	AA	AA	AA	AA	AA	AA	AA	AA
	AA	AG	AA	AG	AG	GG	AA	
147994381								
1788	rs10034650	4	GG	GG	GG	GG	GG	
	GG	AG	AG	AG	AG	GG	GG	AG
	GG	GG	GG	GG	GG	GG	AG	GG
	AG	AA	AA	AG	AG	AA	AG	AG
	AA	AG	GG	AG	GG	GG	GG	
726322								
1789	rs10034657	4	GG	GG	GG	AA	AG	
	AG	AG	GG	GG	AG	AG	GG	AG
	AG	GG	GG	GG	GG	GG	GG	AG
	GG	GG	GG	GG	GG	AG	AG	GG
	GG	GG	GG	GG	GG	AG	AG	
154474931								
1790	rs10034686	4	GG	GG	GG	AG	AG	
	GG	AG	AG	AG	GG	GG	GG	GG
	GG	GG	GG	AG	AG	AG	GG	AG
	GG	GG	GG	GG	GG	AG	GG	AG
	AG	GG	GG	GG	GG	GG	GG	
125796096								
1791	rs1003469	19	GG	GG	GG	AG	GG	

	AG	GG	GG	GG	GG	GG	GG	GG
	GG	GG	AG	GG	GG	GG	GG	GG
	GG	GG	GG	GG	GG	GG	GG	GG
	GG	GG	GG	GG	GG	GG	GG	
5099099								
1792	rs10034725		4	GG	GG	GG	GG	GG
	GG	GG	GG	GG	GG	GG	GG	GG
	GG	GG	GG	GG	GG	GG	GG	GG
	GG	GG	GG	GG	GG	GG	GG	GG
	GG	GG	GG	GG	GG	GG	GG	
136037190								
1793	rs1003475		21	CC	CC	CC	CC	CC
	CC	CC	CC	CC	CC	CC	CC	CC
	CC	CC	CC	CC	CC	CC	CC	CC
	CC	CC	CC	CC	CC	CC	CC	CC
	CC	CC	CC	CC	CC	CC	CC	
37153247								
1794	rs10034789		4	AG	GG	GG	GG	GG
	GG	GG	AG	AG	AG	GG	GG	AG
	AG	GG	AA	AG	AG	AG	AA	GG
	AG	AG	AG	AG	GG	AG	GG	AG
	GG	AG	GG	GG	AA	AG	AA	
149265483								
1795	rs1003480		22	GG	AG	AG	AA	AG
	AG	AA	AA	AA	AG	GG	GG	AG
	AG	AG	AA	AG	AG	AG	AG	AG
	AG	AG	GG	AA	AG	AG	AG	AA
	AG	AG	AA	AG	AA	AG	AG	
31346752								
1796	rs1003490		11	--	GG	GG	--	--
	--	--	--	GG	GG	--	GG	GG
	--	GG	--	GG	--	GG	--	--
	--	--	GG	--	--	--	--	GG
	--	AG	--	AG	--	--	--	
17632306								
1797	rs1003494		22	AA	AA	AA	AA	AA
	AA	AA	AA	AA	AA	AA	AA	AA
	AA	AA	AA	AA	AA	AA	AA	AA
	AA	AA	AA	AA	AG	AA	AG	AG
	AA	AA	AA	AA	AA	AA	AA	
18062987								
1798	rs10034992		4	AG	AG	AA	AG	AA
	AG	AA	AA	AA	AA	AA	AA	AG
	AA	AA	AA	AG	AG	AG	AG	AG
	AA	AA	AA	AA	AA	AA	AA	AA
	GG	GG	AA	AG	AG	AG	AG	
53414666								
1799	rs10034996		4	GG	GG	GG	GG	GG
	GG	AG	AG	AG	GG	GG	GG	GG
	GG	GG	GG	GG	GG	GG	GG	AG

AA AG AG AA AG AG AA AG
AG GG GG GG AG AG AG
135796837
1800 rs10035001 4 AA AA AG AA AA
AA AG AA AA AA AA AA AA
AG GG AG AG AA AG AG AG
AA AA AA AA GG AG GG AG
AG AG GG AG AA AG AA
74039463
1801 rs1003509 6 AA AA AA AA AA
AA AA AA AA AA AA AA AA
AA AA AA AA AA AA AA AA
AA AA AA AA AA AA AA AA
AA AA AA AA AA AA AA
17986547
1802 rs10035095 5 AG AA AG AG AG
AA AG GG GG AG AG GG GG
AG AG GG AG GG AA AA AG
AA AA AA AA GG AG GG AG
AA AG AG AA AA AG AA
75760174
1803 rs10035105 5 AA AA AA AA AA
AA AA AA AA AA AA AA AA
AA AA AG AA AG AA AA AA
AA AA AA AA AG AG GG AG
AG AA AA AA AA AA AA
19976684
1804 rs10035187 5 AA AA AA AG AG
AA AG AA AA AA AA AA AA
AA AA GG AG AG AA AA AA
AA AA AA AA AA AG AA AG
AG AG AG AA AG AA AG
18985998
1805 rs10035392 5 AG GG AG AG GG
AG AG GG AG GG AA AG AA
AA AA AG AA AG AA AG AG
AG GG GG AG AG GG AG AG
AA AA AG AA GG AG AG
99484502
1806 rs10035433 5 AA AA AA AG AG
AA AG AA AA AA AA AA AA
AA AA AA AA AA AG AG AA
AA AA AA AA AG AA AA AA
AG AG AA AA AG AA AG
4725405
1807 rs10035484 5 CC CC CC CC CC
CC CC CC CC CC CC CC CC
CC CC CC CC CC CC CC CC
CC CC CC CC AC CC AC CC
CC CC CC CC CC CC CC

60858543								
1808	rs10035607		5	AC	CC	CC	AC	AC
	AC	AC	AA	AC	AC	CC	AC	CC
	CC	CC	CC	CC	CC	AA	CC	AA
	AA	AC	AC	AA	AC	AA	AA	AA
	AC	AC	AA	AA	AC	AA	AC	
89115932								
1809	rs10035627		5	AG	AA	AG	AG	GG
	GG	AG	AG	AG	AG	AA	AA	AG
	GG	GG	AA	AA	AA	GG	AG	AG
	AG	AG	AG	AG	AG	AG	GG	GG
	AG	AA	AG	AG	GG	GG	GG	
164578495								
1810	rs10035652		5	AA	AA	AA	AA	AA
	AG	AA	AA	AA	AA	GG	GG	AG
	AA	AA	AG	AG	AA	GG	AG	AG
	AG	AG	AG	AG	AG	AA	AA	AA
	AG	AA	AA	AA	AA	AA	AA	
143258738								
1811	rs10035653		5	AG	GG	AG	GG	GG
	GG	GG	AG	GG	AG	GG	GG	GG
	GG	GG	GG	GG	AG	GG	GG	AG
	AA	AA	GG	AG	GG	GG	GG	GG
	GG	AG	GG	AG	GG	GG	GG	
442790								
1812	rs10035789		5	AA	AA	AA	AA	AA
	AA	AA	AA	AA	AA	AA	AA	AA
	AA	AA	AA	AA	AA	AA	AA	AA
	AA	AA	AA	AA	AA	AA	AA	AA
	AA	AA	AA	AA	AA	AA	AA	
12658955								
1813	rs10035816		5	AA	AA	AA	AC	AC
	AA	AC	AA	AA	AA	AC	AC	AC
	AC	AA	AC	AA	AC	AA	AA	AA
	AC	AC	AC	AC	AA	AC	AC	AA
	AA	AA	AC	AC	AA	AC	AC	
22070632								
1814	rs10035874		5	AA	AA	AA	AG	GG
	AG	GG	GG	GG	AG	AA	AG	AG
	AA	AA	AG	AG	AA	GG	AG	AG
	AG	AG	AG	AG	AG	AG	AG	AA
	AG	AA	GG	AG	AG	GG	AG	
13609659								
1815	rs1003590		X	AA	AA	AG	AG	AA
	GG	AA	AG	AG	AA	AA	AA	AA
	AA	AA	AA	AA	AA	AA	AA	AA
	AA	AA	AA	AA	AA	AA	AA	AA
	AA	AA	AA	AA	AG	AG	AA	
48818436								
1816	rs10035917		5	GG	GG	GG	GG	GG

	GG	--	GG	GG	GG	GG	GG	GG
	GG	GG	GG	GG	GG	GG	GG	GG
	GG	GG	GG	GG	GG	GG	GG	GG
	GG	GG	GG	GG	GG	GG	GG	
81022494								
1817	rs10035956		5	CC	CC	CC	CC	CC
	AC	CC	CC	CC	CC	CC	CC	CC
	CC	CC	AC	CC	AA	CC	CC	CC
	AA	AA	AC	AC	CC	CC	CC	CC
	CC	AC	AC	AC	AC	AA	CC	
178184742								
1818	rs10035976		5	AG	GG	GG	GG	GG
	GG	GG	AG	GG	AG	GG	GG	GG
	GG	GG	AG	GG	AG	AA	GG	AG
	GG	AG	AG	GG	AG	AG	AA	AG
	AA	GG	AG	GG	AG	AG	GG	
100718992								
1819	rs10035997		5	AA	AA	AA	AA	AA
	AA	AA	AA	AA	AA	AA	AA	AA
	AA	AA	AA	AA	AA	AA	AA	AA
	AA	AA	AA	AA	AA	AA	AA	AA
	AA	AA	AA	AA	AA	AA	AA	
29876851								
1820	rs1003600		X	AA	AA	AA	AA	AA
	AA	AA	AA	AA	AA	AA	AA	AA
	AA	AA	AA	AA	AA	AA	AA	AA
	AA	AA	AA	AA	AA	AA	AA	AA
	AA	AA	AA	AA	AA	AA	AA	
18640083								
1821	rs10036026		5	AG	GG	AG	AG	AG
	AG	GG	AG	AG	AG	AG	AG	AG
	AG	AG	AG	GG	AG	GG	AG	GG
	GG	GG	GG	GG	GG	GG	GG	GG
	AA	AG	AG	GG	GG	GG	GG	
159262961								
1822	rs10036130		5	CC	CC	CC	AC	AC
	AC	AA	AA	AA	AA	AC	AC	AC
	CC	AC	AA	AA	AA	AA	AC	AC
	CC	CC	CC	CC	AC	AA	AC	AA
	AC	AA	AA	AA	CC	AC	CC	
32869602								
1823	rs1003617		2	AA	AC	CC	AC	AC
	AA	CC	AC	AC	AC	CC	AC	AC
	CC	CC	CC	CC	CC	AC	AA	CC
	CC	CC	CC	CC	CC	AC	AC	CC
	AC	AC	AC	AA	AC	AC	AC	
40922298								
1824	rs10036194		5	AA	AA	AA	AA	AA
	AA	AA	AA	AA	AA	AA	AA	AA
	AA	AA	AA	AA	AA	AA	AA	AA

	AA	AA	AA	AA	AA	AA	AA	AA
	AA	AA	AA	AA	AA	AA	AA	
8957201								
1825	rs10036252	5	AG	AG	AA	AA	AA	
	AA	AA	AA	AA	AA	AG	AA	AA
	AG	AG	AA	AA	AA	AA	AA	AA
	AA	AA	AA	AA	AA	AA	AA	AA
	AA	AA	AA	AA	AG	AA	AG	
25451819								
1826	rs10036347	5	AG	GG	AG	GG	GG	
	GG	AG	GG	GG	GG	AG	AA	AA
	GG	AG	AG	AG	AA	AA	AA	AA
	GG	AG	AG	GG	GG	GG	GG	GG
	GG	GG	GG	GG	AG	GG	AG	
56694656								
1827	rs10036362	5	AA	AA	AA	GG	AG	
	AG	AG	AG	AG	AG	GG	GG	GG
	AG	GG	AA	AG	AG	AG	GG	GG
	GG	GG	GG	GG	AA	AG	AG	AA
	AG	GG	AG	GG	AA	AG	AA	
26846426								
1828	rs10036380	5	GG	GG	GG	GG	GG	
	GG	GG	GG	GG	GG	AG	GG	GG
	GG	GG	GG	GG	GG	GG	GG	GG
	AG	AG	GG	AG	AG	GG	AG	AG
	GG	GG	GG	GG	GG	GG	GG	
11410194								
1829	rs10036399	5	AG	GG	AG	AG	AG	
	AA	AG	AA	AA	AG	AG	AA	AA
	GG	AG	AG	AG	GG	AG	AA	GG
	AG	AG	AG	AG	AG	AG	AA	GG
	AG	AG	AG	AG	AG	AG	AG	
60860146								
1830	rs10036413	5	AG	AA	AG	AG	AG	
	AA	GG	GG	GG	GG	AG	GG	GG
	GG	GG	GG	GG	GG	AG	AG	GG
	GG	GG	GG	GG	AG	AG	AG	AG
	GG	GG	AG	AG	AG	AG	GG	
124694594								
1831	rs1003644	22	AG	GG	AG	GG	AG	
	GG	AG	AG	GG	AG	GG	GG	GG
	GG	GG	GG	AG	AG	AG	AG	AG
	GG	GG	AG	GG	AG	AG	AG	AA
	GG	GG	AG	GG	GG	AG	AG	
39676744								
1832	rs10036474	5	AA	AA	AA	AA	AA	
	AG	AA	AA	AA	AG	AA	AA	AA
	AA	AA	AA	AA	AA	AA	AA	AA
	AA	AA	AA	AA	AA	AA	AA	AA
	AA	AA	AA	AA	AA	AA	AA	

54370231
1833 rs10036524 5 AA AG AG AA AA
AA AA AA AA AA AA AA AA
AA AA AA AA AA AA AA AA
AA AA AA AA AA AG AA AG
AA AA AG AG AA AA AA
19023177
1834 rs10036574 5 GG GG GG AG GG
AG AG AG AG AG AG AG AG
GG GG AA AG AG GG GG AG
AG AG GG AG AA GG AG AG
AG GG AG GG GG GG GG
25774587
1835 rs10036625 5 CC CC CC CC CC
CC CC CC CC CC CC CC CC
CC CC CC CC CC CC CC CC
CC CC CC CC CC CC CC CC
CC CC CC CC CC CC CC
24989306
1836 rs10036680 5 CC AC AA AC AA
AC AA AC AC AC AA AA AA
AC AA AC AC AA AC AC AC
AC AC AC AC AA AC AA AC
AC AC CC CC AC AC AA
18814214
1837 rs10036723 5 AA AA AA AA AA
AA AA AA AA AA AA AA AA
AA AA AA AA AA AA AA AA
AA AA AA AA AA AA AA AA
AA AA AA AA AA AA AA
14602001
1838 rs10036748 5 GG AG GG GG AG
AG GG AG AG AG AG AG GG
GG GG GG AG AG AG AG AA
GG GG GG GG GG AG GG GG
GG AG AG AA AG GG AG
150458146
1839 rs10036776 5 AG AA AA AG AG
AG GG GG GG GG AG GG GG
AG AG AG AG AG AG GG GG
AG GG AG AG GG AG GG AG
GG AA AA AA GG GG GG
79509974
1840 rs10036886
5 -- -- -- -- -- -- -- --
-- -- -- -- -- -- -- --
-- -- -- -- -- -- -- --
-- -- -- -- -- -- -- --
-- -- -- -- 101204974
1841 rs10036899 5 GG GG AG AA AA

AA AG GG GG AG AG GG AG
AG AG AG AG AG AA AG AA
AG AG AG AG AG GG GG GG
AA AG AA AG AA AG AA
76924133
1842 rs10036921 5 AC CC AC AC AC
AA CC CC CC CC AC AC AA
CC AC AA AA AA CC CC CC
CC CC CC CC AC AC AA AA
CC AC AC AA AC AC AA
165199466
1843 rs10037007 5 AG AG GG AG AG
AA GG GG GG GG GG GG AG
AG AG GG GG GG AG AA AA
GG GG GG GG GG AG AG AG
AG GG AG AG GG AG GG
170408962
1844 rs10037064 5 GG GG GG GG GG
GG GG GG GG GG GG GG GG
GG GG GG GG GG GG GG GG
GG GG GG GG GG GG GG GG
GG GG GG GG GG GG GG
165611769
1845 rs10037073 5 AG GG AG AG AG
GG AG GG GG GG AG AG AA
GG AG AG GG AG AG AG GG
AA AA AA AA AA AG AA AG
AG AA AG AG AA AA AG
20975229
1846 rs10037143 5 AG AG AG AA AG
AA AG AG AG AG GG GG GG
AG AG AG AG AG GG AG AG
AG GG GG AG AG AG AA GG
AA GG GG GG AG AG AG
42159544
1847 rs10037178 5 GG GG GG GG GG
GG GG AG GG AG GG GG GG
GG GG GG GG GG GG GG GG
GG GG GG GG GG GG GG GG
GG GG GG GG GG GG GG
24666217
1848 rs1003719 21 AG AG AG AG AG
GG AG GG GG AG AG GG AG
AG AG AG GG GG AG GG GG
GG GG GG GG AG AG AG AG
GG GG AA AG AG AA GG
38491095
1849 rs10037268 5 AA AA AA AA AA
AA AA AG AG AG AA AA AA
AA AA AA AG AG AA AA AA

AA AA AA AA AA AA AA AA
AA AA AA AA AA AA AA
121153924
1850 rs10037293 5 AC CC CC CC CC
CC AC AC AC AC AC AC AC
AC AC CC CC CC AC AC CC
CC CC CC CC AC AC AC CC
AA AC CC AC CC CC CC
90628300
1851 rs10037307 5 AC CC AC CC CC
CC CC CC CC CC AC AC AC
AC AC CC CC CC AC CC AC
CC CC CC CC AC AC AC AA
CC AC AC AC CC CC AC
125822005
1852 rs10037310 5 AG AG AG AG AG
AG GG GG GG GG -- GG AG
-- -- GG GG GG GG GG GG
-- AG AG -- GG GG GG GG
GG AG GG AG AG GG AG
83756654
1853 rs10037346 5 GG GG GG GG GG
GG GG AG AG AG GG GG GG
GG GG AG GG AG GG GG GG
GG GG GG GG GG GG GG GG
GG AG AG GG GG GG GG
115408118
1854 rs1003737 6 AC AC AA CC CC
CC AC AA AA AA AC AA AA
AA AA AC CC CC CC AC CC
AC AC AA AA AA AC AC AA
AC AA CC AC AC AC CC
160430656
1855 rs1003743 2 AG AG GG AA AA
AA AA AG AG AA AA AA AA
AA AA AA AG AA AG AG AA
AG AG AG AG AG GG AG GG
AA AA AA AA AA AA AA
155346958
1856 rs10037451 5 AG GG AG AG AG
GG AG AG AA AG GG GG GG
GG GG AG AG AG AG AG AG
AG AG AA AA AA AG AG AG
AA GG AG AG AG AG AG
177856605
1857 rs10037465 5 AG AG AG AG AG
AG AA GG GG GG AA AG AG
AA AA AG AG AA GG AG AG
GG GG GG GG AA AG AG AA
AG AG GG AG AG AG GG

136998182								
1858	rs10037553		5	AA	AA	AA	AA	AA
	AA	AA	AA	AA	AA	AA	AA	AA
	AA	AA	AA	AA	AA	AA	AA	AA
	AA	AA	AA	AA	AA	AA	AA	AA
	AA	AA	AA	AA	AA	AA	AA	
23365538								
1859	rs10037599		5	AC	AA	AA	AA	AA
	AA	AA	AA	AA	AC	AA	AC	AC
	AA	AA	AC	AA	AA	AC	AC	AC
	AC	AC	AC	AC	AC	AC	AA	AC
	AC	CC	AC	CC	AA	AC	AC	
76541325								
1860	rs10037618		5	GG	GG	GG	GG	GG
	GG	GG	GG	GG	GG	GG	GG	GG
	GG	GG	GG	GG	GG	GG	GG	GG
	GG	GG	GG	GG	GG	GG	GG	GG
	GG	GG	GG	GG	GG	GG	GG	
179301972								
1861	rs10037631		5	GG	GG	GG	GG	GG
	GG	GG	GG	GG	GG	GG	GG	GG
	GG	GG	GG	GG	GG	GG	GG	GG
	GG	GG	GG	GG	GG	GG	GG	GG
	GG	GG	GG	GG	GG	GG	GG	
176691618								
1862	rs10037632		5	AA	AA	AA	AA	AA
	AG	AA	AA	AA	AA	AA	AA	AA
	AA	AA	AG	AG	AA	AA	AA	AG
	AA	AA	AA	AA	AG	AA	AA	AA
	AA	AA	AA	AA	AA	AA	AA	
92028637								
1863	rs10037650		5	AA	AA	AA	AA	AA
	AA	AA	AA	AA	AA	AA	AA	AA
	AA	AA	AA	AA	AA	AA	AA	AA
	AA	AA	AA	AA	AA	AA	AA	AA
	AA	AA	AA	AA	AA	AA	AA	
24941722								
1864	rs10037656		5	AA	AA	AA	AA	AA
	AA	AA	AA	AA	AA	AA	AA	AA
	AA	AA	AA	AA	AA	AG	AG	GG
	AA	AA	AA	AA	AA	AA	AA	AA
	AG	AA	AA	AA	AG	AA	AG	
5183783								
1865	rs10037689		5	AA	AA	AA	AA	AA
	AA	AA	AA	AA	AA	AA	AA	AA
	AA	AA	AA	AA	AA	AA	AA	AA
	AA	AA	AA	AA	AA	AA	AA	AA
	AC	AA	AC	AA	AA	AA	AA	
13393591								
1866	rs1003769		1	AA	AA	AA	AG	AA

AA AG GG GG GG AA AA AA
AA GG AA AA AA AA AA AG
AG AG AG AG AG AA AA AG
AG AG AA AG AG AG AA
55463860
1867 rs10037698 5 GG GG GG GG GG
GG GG GG GG GG GG GG GG
GG GG GG GG GG GG GG GG
GG GG GG GG GG GG GG GG
GG GG GG GG GG GG GG
175059058
1868 rs10037793 5 AA AA AA AA AA
AA AA AA AA AA GG GG AG
AG AG AA AA AA AG AG AG
GG GG AG GG AA AA AA AA
AA AG AA AA GG GG GG
18168945
1869 rs1003792 21 CC AC CC CC CC
AC CC AC CC AC CC CC CC
CC CC AC CC CC AA CC AC
AC CC AC CC AC AC AA AC
CC AC AC AC CC AC CC
44810756
1870 rs10037981 5 AA AA AA AC AA
AC AA AA AA AA AA AA AA
AC AC AA AA AA AA AA AA
AA AA AA AA AC AA AA AA
AC AA AA AA CC AC AC
146566438
1871 rs10037996 5 AA AA AG GG AG
AG AG AA AA AA AG GG GG
GG GG AG GG GG GG AG GG
AG GG GG GG GG GG GG GG
AG AG AA AA AA AG AA
117354366
1872 rs10037999 5 GG GG GG AG GG
AG AG GG GG GG AG AA AG
GG GG AG GG GG AA AA AA
AA AG AG AG AG AG GG AA
GG AG AG AG AG AG GG
124867823
1873 rs10038020 5 AC CC CC AA AA
AA AA AA AA AA AC AC CC
AA AC AA AA AA AC AA AA
AA AA AA AA AA AA AA AA
AA AA AA AA AC CC AA
30069262
1874 rs10038032 5 AA AA AA AA AA
AA AA AA AA AA AA AA AA
AA AA AA AA AA AA AA AA

AA AA AA AA AA AA AA AA
AA AA AA AA AA AA AA
174483537
1875 rs10038063 5 AA AA AA AA AA
AA AA AA AA AA AA AA AA
AA AA AA AA AA AA AA AA
AA AA AA AA AA AA AA AA
AA AA AA AA AA AA AA
172812828
1876 rs10038065 5 GG GG GG GG GG
GG GG GG GG GG GG GG GG
GG GG GG GG GG GG GG GG
GG GG GG GG GG GG GG GG
GG GG GG GG GG GG GG
5929129
1877 rs10038101 5 AG AG GG GG GG
AG GG AG GG AG GG GG AG
GG AG AG AG GG AG AG AG
AG AA AA AG GG GG GG GG
GG AG GG AG GG AG AG
35044888
1878 rs10038113 5 AG AA AG GG AG
AG GG AG GG AG AG AG AG
AG GG AA AA AA AG AA AA
AG AG AA AG AG AG AG AA
AA AG GG GG AG AG AG
25902342
1879 rs10038115 5 AA AA AA AA AA
AA AA AA AA AA AA AA AA
AA AA AA AA AA AA AA AA
AA AA AA AA AA AA AA AA
AA AA AA AA AA AA AA
150044701
1880 rs10038136 5 AA AA AA AA AA
AA AA AA AA AA AA AA AA
AA AA AA AA AA AA AA AA
AA AA AA AA AA AA AA AA
AA AA AG AA AA AA AA
146069910
1881 rs1003816 7 GG GG AG GG GG
GG GG GG GG GG GG GG GG
GG GG GG GG GG GG GG GG
GG GG GG GG GG AG GG GG
GG AG GG AG GG GG AG
139653612
1882 rs10038170
5 -- -- -- -- -- -- -- --
-- -- -- -- -- -- -- --
-- -- -- -- -- -- -- --
-- -- -- -- -- -- -- --

-- -- -- -- 14688226
1883 rs10038211 5 CC CC AC AA AC
AA AC AC AC AC AA AA AC
AC AC AC AC AC AA CC AC
AC CC CC CC AC CC AC CC
AC AC AC CC CC CC AC
120373318
1884 rs10038337 5 CC CC CC CC CC
CC CC CC CC CC CC CC CC
CC CC CC CC CC CC CC CC
AC AC CC AC CC CC CC CC
CC CC CC CC CC CC CC
11512457
1885 rs10038356 5 GG GG GG AG AG
AA AG AA AA AA AG GG AG
GG GG AA AA AA GG AA AG
AA AA AG AG AA AA AA AA
AA AG AG AG AG GG AA
2829191
1886 rs10038374 5 GG GG GG AG AG
AA AG AA AA AA AG GG AG
GG GG AA AA AA GG AA AG
AA AA AG AG AA AA AA AA
AA AG AG AG AG GG AA
2823353
1887 rs10038387 5 AC AC AC AC CC
CC AC CC CC CC CC CC CC
CC CC CC AC AC CC CC CC
CC AC AC CC CC CC CC CC
CC AC CC AC CC CC AC
105866862
1888 rs1003839 17 GG GG GG GG GG
GG GG GG GG GG GG GG GG
GG GG AG GG GG AG AG AG
AG AG AG AG AG GG AG AG
GG GG GG GG GG GG GG
32405719
1889 rs10038404 5 GG GG GG GG GG
GG GG GG GG GG GG GG GG
GG GG GG GG GG GG GG GG
GG GG GG GG GG GG GG GG
GG GG GG GG GG GG GG
16097008
1890 rs10038452 5 GG GG GG AG AG
AG AA AA AA AA AA AA AG
AG AG AA AA AA AG AG AA
AA AA AA AA AG GG GG AG
AA AA AG AA GG AG GG
174384256
1891 rs10038454 5 GG GG GG GG GG

GG GG GG GG GG GG GG GG
GG GG GG GG GG GG GG GG
GG GG GG GG GG GG GG GG
GG GG GG GG GG GG GG
32036769
1892 rs1003855 20 AG AG GG GG AA
AG AG GG GG GG AA AA AG
AG AG GG AG AG GG AG AG
AG AG AG AG AA AG AG AG
AG AA AA AA AA AG AG
43715188
1893 rs10038582 5 AA AA AA AA AA
AA AA AG AG AG AA AA AG
AG AG AA AA AG AA AA AA
AA AA AA AA AA AA AA AA
AA AA AA AA AA AA AA
174982432
1894 rs10038615 5 AA AA AA AG AG
AG AA AA AG AA AG GG AG
AG AA AG AG AG AA AA AA
AA AA AA AA AA AG AG AA
AG AA AA AA AA AA AA
52153756
1895 rs10038623 5 GG GG GG GG GG
GG GG AG AG AG GG GG GG
AG GG GG GG GG GG GG GG
GG GG GG GG GG GG GG GG
GG GG GG GG GG GG GG
27435329
1896 rs10038650 5 AA AA AA AG AG
AA AG AA AA AA AA AA AA
AA AA AA AA AA AA AA AA
AA AA AA AA AA AG AG AG
AA AA AA AA AG AG AA
168236650
1897 rs10038739 5 AA AA AA AA AA
AA AA AA AA AA AA AA AA
AA AA AA AA AA AG AG AA
AA AA AA AA AA AG AG AA
AA AA AA AA AA AA AA
61479184
1898 rs10038745 5 GG GG GG GG GG
GG GG GG GG GG GG GG GG
GG GG AG AG GG GG GG GG
GG GG GG GG GG GG GG GG
GG GG GG GG GG GG GG
23139973
1899 rs10038754 5 AA AG GG AG AG
AG AA AA AA AG AA GG AG
AA AA AG AA AG AG AA AG

	AG	AG	AG	AG	AA	AA	AA	AA
	AA	AA	AG	AG	AG	AG	AG	
67150765								
1900	rs10038773	5	CC	CC	CC	CC	CC
	CC	CC	CC	CC	CC	AC	CC	AC
	AC	AC	CC	CC	CC	CC	AC	CC
	CC	CC	CC	CC	CC	AC	CC	AC
	CC	CC	CC	CC	AC	CC	AC	
52223516								
1901	rs1003878	6	AA	AG	AG	GG	GG
	GG	GG	GG	AG	GG	GG	GG	GG
	GG	GG	AA	AG	AG	GG	GG	GG
	GG	GG	GG	GG	GG	AG	GG	GG
	GG	AG	GG	AG	AG	AG	GG	
32299822								
1902	rs10038851	5	CC	CC	CC	CC	CC
	CC	CC	AC	CC	AC	CC	CC	AC
	CC	AC	CC	CC	CC	CC	AC	CC
	AC	AC	CC	AC	CC	CC	CC	CC
	CC	AC	AC	CC	CC	AC	AC	
25974120								
1903	rs10038863	5	GG	AG	GG	AG	AG
	AG	AA	AA	AG	AG	GG	AA	AG
	AG	AG	GG	GG	GG	AA	AA	AG
	AA	AA	AA	AA	AG	GG	AG	AG
	AG	AG	AG	AG	AG	AG	AG	
132562731								
1904	rs10038914	5	GG	AG	GG	GG	GG
	AG	GG	AG	AA	AG	AG	AG	AG
	AA	AG	AG	AG	AG	AG	GG	AG
	GG	AG	AG	GG	GG	AG	AG	GG
	AA	GG	AG	GG	AA	AG	AA	
40078936								
1905	rs10038921	5	AA	AG	AG	AA	AA
	AA	AA	AA	AA	AA	GG	GG	AG
	AG	AG	AA	AG	AG	AA	AA	AA
	AA	AA	AA	AA	GG	AA	AG	AG
	AA	AG	AA	AG	AA	AA	AG	
6428242								
1906	rs10039017	5	AA	AA	AA	AG	GG
	AG	AG	AA	AA	AA	AA	AA	AA
	AA	AA	AG	AG	AA	AA	AA	AA
	AA	AA	AA	AA	AA	AG	AA	AG
	AG	AA	AA	AA	AA	AA	AG	
28627343								
1907	rs1003902	17	AC	AC	AC	AC	AC
	CC	AC	CC	CC	AC	AC	AC	CC
	CC	CC	AC	AC	AC	AC	AC	AA
	AC	AC	AC	AC	AA	AC	AC	AA
	AA	AC	CC	AC	AC	AC	CC	

12975928
1908 rs10039049 5 AG AA AG AG AG
AG AG AA AA AA GG GG AG
AA AA AG AG GG AA AG AA
AG AG AG AG AG AA AG AA
GG AG AA AA GG AG GG
150517899
1909 rs10039073 5 AA AA AG AG AG
AG AG AA AA AA AG AG AG
AA GG AG AA AG AG AG GG
AG AA AA AG AG AG AG AG
AA AG GG GG AG AG AA
32042760
1910 rs10039077 5 GG GG GG GG AG
GG AG GG GG GG GG GG GG
GG GG GG GG GG GG GG GG
AG GG GG GG GG AG GG AG
GG GG AG GG GG GG GG
128149665
1911 rs10039101 5 CC CC CC CC CC
CC CC CC CC CC AC AC AC
CC CC CC CC CC CC AC AC
CC CC CC CC CC CC CC CC
CC CC CC CC AC AC CC
9689026
1912 rs10039139 5 AC CC AC CC CC
CC CC CC CC CC CC CC CC
CC CC CC CC CC CC CC CC
AA AA AA AA CC CC CC CC
CC AC CC CC AC CC AC
137164863
1913 rs10039206 5 AA AA AA AG AG
AG GG GG GG GG GG GG GG
GG GG GG AG AG GG GG GG
GG AG AG GG GG GG GG GG
AA GG GG GG GG AG GG
55988778
1914 rs10039219 5 GG GG GG GG GG
GG GG AA AG AA GG GG GG
GG GG AG GG AG GG GG GG
GG GG GG GG GG GG GG GG
GG GG AG GG AG GG AG
25883408
1915 rs10039221 5 AA AA AA AA AA
AA AA AG AG AG AA AA AG
GG GG AG AA AG AA AA AA
AA AA AA AA AA AA AA AA
AA AA AA AA AG AA AG
174861613
1916 rs10039241 5 GG GG GG GG GG

GG GG GG GG GG GG GG GG
GG GG GG GG GG GG GG GG
GG GG GG GG GG GG GG GG
GG GG GG GG AG AG GG
176578072
1917 rs10039287 5 CC AC CC CC CC
CC CC CC CC CC AC CC AC
AC AC CC CC CC CC CC AC
AC CC CC AC AC CC AC AC
CC CC CC CC CC CC CC
101318849
1918 rs10039290 5 AC AC CC CC CC
AC CC CC CC AC CC CC CC
CC CC AC AC AC CC CC AC
CC CC CC CC CC CC CC CC
CC CC CC CC CC CC CC
81917419
1919 rs10039326 5 AG AG AA GG GG
GG AG AG GG AG AA AA AA
AG AG GG AG AG AA AA AA
AA AA AA AA GG AG AG GG
AA AG AA AA AA AA AA
2513346
1920 rs10039348 5 GG AG AG GG GG
GG AG GG GG GG GG GG GG
GG GG GG GG GG AG GG AG
AG AG AG AG GG AA AG AG
GG GG AG GG GG GG GG
171250237
1921 rs10039354 5 GG GG AG AG AG
GG AG AG GG AG GG GG GG
GG GG AG GG AG AG AG GG
AA AG GG AG GG AG AG GG
GG GG GG GG GG GG GG
5195069
1922 rs10039391 5 GG GG GG AG GG
AG GG GG GG GG GG GG GG
GG GG GG GG GG AG AG AG
GG GG GG GG GG GG GG GG
GG GG GG GG GG GG GG
15729889
1923 rs10039414 5 AA AA AA AA AA
AA AA AA AA AA AA AA AA
AA AA AA AA AA AA AA AA
AA AA AA AA AA AA AA AA
AA AA AA AA AA AA AA
39235291
1924 rs1003944 22 GG GG GG GG GG
GG GG GG GG GG GG GG GG
GG GG GG GG GG GG GG GG

GG GG GG GG GG GG GG GG
GG GG GG GG GG GG GG
17595966
1925 rs10039447 5 AA AC CC AC CC
AC AC CC AC AC AA AC AC
CC CC AC AA AC AC CC CC
CC CC CC CC AA CC AC AC
CC AC CC AC AC AA AC
13842788
1926 rs1003945 22 GG GG GG GG GG
GG GG GG GG GG GG GG GG
GG GG GG GG GG GG GG GG
GG GG GG GG GG GG GG GG
GG GG GG GG GG GG GG
17595746
1927 rs10039490 5 GG GG GG GG GG
AG GG AG AG AG AG AG AG
AG AG AG GG GG GG AG GG
AG GG AG AG GG AG GG AG
AG AG AG AG AG AG AG
78740930
1928 rs10039512 5 AG AA AG AG AG
AG AG GG GG AG AG AA AA
AA AA AA AA AA AA AG AA
GG AG GG GG AG AG AG AG
AG AG AG GG AA AA AA
68143193
1929 rs10039539 5 AA AA AA AA AA
AA AA AA AA AA AA AA AA
AA AA AA AA AA AA AA AA
AA AA AG AA AA AA AA AA
AA AA AA AA AA AA AA
12734066
1930 rs10039593 5 GG GG GG GG AG
AG GG -- AG AA GG GG GG
GG GG GG GG GG AG GG GG
-- -- AG -- GG GG GG GG
GG GG AG AG AG GG AG
19425092
1931 rs10039608 5 GG GG GG GG GG
GG GG GG GG GG GG GG GG
GG GG GG GG GG GG GG GG
GG GG GG GG GG GG GG GG
GG GG GG GG GG GG GG
179192205
1932 rs10039671 5 GG GG GG GG GG
GG GG GG GG GG GG GG GG
GG GG GG GG GG GG GG GG
GG GG GG GG GG GG GG GG
GG GG GG GG GG GG GG

37629992
1933 rs1003968 22 AA AA AA AA AG
AG AA AA AA AA GG GG GG
GG GG AA AA AA AA AA AG
AG AG AG AG AG AG AG AA
AA AA GG AG AA AA AA
21367590
1934 rs10039717 5 CC CC CC AA AC
AC AC AC AC AC CC AC AC
AC AC CC AC AC AA AA AC
AA AC AC AA CC AA AC AC
AA CC AC CC AC AC AA
108577839
1935 rs10039735 5 AA AA AA AG AG
AG AA AA AA AA AA AA AA
AG AG GG AG AG AA AA AG
AA AA AG AG AA AA AA AA
AA AA AA AA AG GG AA
48534
1936 rs1003976 6 AG AG AG GG GG
GG GG AG AG AA GG GG GG
GG GG GG AG GG AG AG AG
GG GG GG GG GG GG GG GG
AG AG AG AG GG GG AG
33834626
1937 rs1003979 6 AG GG AG GG AG
AG GG AG AA AG AG AA AA
AA AA AG GG AA AG AG AG
AA AA AA AA AA GG AG AG
AG AA GG AG GG GG AG
33114171
1938 rs10039833 5 CC CC CC CC CC
CC CC CC CC CC CC CC CC
CC CC CC CC CC CC CC CC
CC CC CC CC CC CC CC CC
CC CC CC CC CC CC CC
17910453
1939 rs10039835 5 GG GG GG AG GG
GG AG GG GG GG GG GG GG
GG GG GG AG AG GG GG GG
GG GG GG GG AG GG AG GG
AG GG GG GG GG GG GG
155764666
1940 rs10039846 5 GG GG GG AG AG
GG AA GG GG GG GG GG GG
GG GG GG GG GG GG AG GG
AA AG AG AA GG GG GG GG
AA GG GG GG AG AA GG
103206340
1941 rs10039878 5 GG GG GG GG GG

	GG	GG	GG	GG	GG	GG	GG	GG
	GG	GG	GG	GG	GG	GG	GG	GG
	GG	GG	GG	GG	AG	GG	AG	AG
	GG	GG	AG	GG	GG	GG	GG	
20346525								
1942	rs10039886		5	GG	GG	GG	GG	GG
	GG	AG	AG	AG	AG	AG	GG	GG
	GG	GG	GG	GG	GG	GG	AG	GG
	GG	GG	GG	GG	AG	GG	GG	AG
	AG	AA	GG	AG	GG	GG	GG	
53911283								
1943	rs10039913		5	GG	GG	GG	AG	AA
	AG	AG	GG	GG	GG	GG	GG	GG
	GG	GG	AG	GG	AG	GG	AG	GG
	GG	GG	GG	GG	GG	GG	GG	GG
	AG	GG	AG	GG	GG	GG	GG	
165650569								
1944	rs1003995		3	CC	CC	CC	CC	CC
	CC	CC	AC	CC	CC	CC	CC	CC
	CC	CC	AA	AC	AC	AC	AC	CC
	CC	AC	AC	AC	CC	CC	CC	CC
	AC	AC	CC	CC	AC	CC	AC	
187089031								
1945	rs10040027		5	GG	GG	AG	AA	AA
	AA	AA	AA	AG	AA	AG	AG	GG
	AA	AG	AA	AA	AA	AG	AA	AG
	AA	AA	AA	AA	AG	AG	AA	AG
	AG	AA	AA	AA	AG	AG	AA	
31582523								
1946	rs10040067		5	AA	AA	AA	AA	AA
	AA	AA	AA	AA	AA	AA	AA	AA
	AA	AA	AA	AA	AA	AA	AA	AA
	AA	AA	AA	AA	AA	AA	AA	AA
	AA	AA	AA	AA	AG	AA	AG	
1027182								
1947	rs10040171		5	AA	AA	AA	AA	AA
	AA	AA	AA	AA	AA	AA	AA	AA
	AA	AA	AA	AA	AA	AA	AA	AA
	AA	AA	AA	AA	AA	AA	AA	AA
	AA	AA	AA	AA	AA	AA	AA	
4006435								
1948	rs10040206		5	AA	AA	AA	AA	AA
	AA	AA	AA	AA	AA	AA	AA	AA
	AA	AA	AA	AA	AA	AA	AA	AA
	AA	AA	AA	AA	AA	AA	AA	AA
	AA	AA	AA	AA	AA	AA	AA	
33992395								
1949	rs10040214		5	AC	AC	AA	AC	AC
	CC	AA	CC	CC	CC	AC	AC	CC
	AC	AC	CC	CC	CC	CC	AA	CC

CC AC AC AC AA AC AC AA
AC AC AC AC AC AC CC
116019604
1950 rs10040222 5 AA AA AA AA AA
AA AA AA AA AA AA AA AA
AA AA AA AA AA AA AA AA
AA AA AA AA AG AA AA AA
AA AA AA AA AA AA AA
26336339
1951 rs10040300 5 GG GG GG GG GG
GG GG AG AG AG GG GG GG
GG GG GG AG AG GG GG GG
GG GG GG GG GG AG GG GG
GG GG GG GG AG AG GG
154906291
1952 rs10040332 5 GG GG GG GG GG
GG GG GG GG GG GG GG GG
GG GG GG GG GG GG GG GG
GG GG GG GG AG GG AG AG
GG GG GG GG GG GG GG
19991161
1953 rs10040382 5 GG GG AG GG GG
GG GG GG GG GG AG GG AG
GG AG AG AG AG GG GG GG
GG GG GG GG GG GG GG GG
GG GG GG GG GG GG GG
52761052
1954 rs10040390 5 GG AG AG GG AG
AG GG GG AG GG AG AG AG
GG GG GG AG AG GG GG GG
AG AG AG AG GG AG AG GG
GG GG AG AG GG AG GG
30998450
1955 rs10040401 5 AG AG GG AG AG
GG AG GG GG GG AA AG AA
AA AA GG AG AG GG AA GG
AG AG AG AG AG AA AA AA
AA AG GG GG AA AG AA
91477046
1956 rs10040425 5 AA AA AA AA AA
AA AA AC AC AC AA AA AC
AA AA AA AA AA AC AA AC
AA AA AA AA AC AA AC AC
AC AA AC AC AC AA AC
3727332
1957 rs1004044 21 AG AG AG AG AG
AA AG AG AG AA AA AG AG
AG AG AA AA AA AA AA AG
AA AA AG AG AA AG AA AA
AA AA AA AA AA AA AA

31766987
1958 rs10040443 5 AA AA AA AA AA
AA AA AG GG AG AA AA AA
AG AG AA AA AA AA AG AA
AG AG AG AG AG AA AG AG
AA AG AA AA AA AA AA
141693593
1959 rs1004046 19 AA AA AA AA AA
AA AA AC AC AC AC AA AC
AA AA AC AC CC AA AA AC
AA AA AA AA AC AA AA AC
AC AA CC AC AA AC AA
30992639
1960 rs10040488 5 CC CC AC AC AC
AC AC CC CC CC AA AC AA
AC AC AC AA AA AC AC AA
AC AA AA AC AC AC AC AC
AC AC AC AC AC CC AA
44880288
1961 rs10040554 5 GG GG GG GG GG
GG GG GG GG GG GG GG GG
GG GG GG GG GG GG GG GG
GG GG GG GG GG GG GG GG
GG GG GG GG GG GG GG
37376344
1962 rs1004056 7 AG AG AG GG AA
AG AG AA AA AA AA AA AA
AA AA GG AG AG GG AG AG
AG GG GG AG AG AA AA AG
AG AA AA AA AG GG AA
29355811
1963 rs10040564 5 GG GG GG GG GG
GG GG GG GG GG GG GG GG
GG GG GG GG GG GG GG GG
GG GG GG GG GG GG GG GG
GG GG GG GG GG GG GG
134853207
1964 rs10040568 5 AG AG AG AG AG
AA GG GG AG GG GG GG GG
AG AG AA AA AG GG GG AG
GG GG GG GG AA AG AG AG
AG AG GG AG GG AG AG
1033016
1965 rs10040577 5 CC CC CC CC CC
CC CC AC CC AC CC CC CC
CC CC CC CC CC CC CC CC
CC CC CC CC CC CC CC CC
CC CC CC CC CC CC CC
25955765
1966 rs10040610 5 AG AA AA AA AA

AA AA AA AA AA AA AA AA
AA AA AG AA AG AA AG AA
AG AG AA AG AA AA AA AA
AA AG AA AA AA AA AA
6353815
1967 rs10040617 5 AA AG AA AA AA
AG AA AA AA AA AA AG AG
AA AG AG AA AG AA AA AA
AA AA AA AA AA AA AA AA
GG AG GG AG AA AA AA
35966681
1968 rs1004064 4 GG GG GG AG GG
AG GG GG GG GG GG GG GG
GG GG GG GG GG GG GG GG
GG GG GG GG GG GG GG GG
GG GG GG GG GG GG GG
38458620
1969 rs10040697 5 AG GG GG GG GG
GG GG GG GG GG AG AG AG
AG AG GG GG GG GG GG GG
AG AG AG AG AG GG AG AG
GG GG GG GG GG GG GG
95173887
1970 rs10040715 5 GG GG GG GG GG
GG GG GG GG GG GG GG GG
GG GG GG GG GG GG GG AG
GG GG GG GG GG GG GG GG
GG GG GG GG GG GG GG
57594208
1971 rs10040793 5 AG AG AG GG AG
GG AG GG GG GG GG GG GG
GG GG GG GG GG AG AG AG
GG GG GG GG AG GG AG GG
AG GG GG GG AG GG AG
123990972
1972 rs10040819 5 CC CC CC CC CC
CC CC CC CC CC CC CC CC
CC CC AC AC CC CC CC CC
CC CC CC CC CC AC CC CC
CC AC CC AC AC AC CC
147964658
1973 rs10040820 5 AG AA AA AG AG
AG AG AA AA AA AG AG AA
AA AA AA AA AA AG AG AG
AA AA AA AA AA AG AA AG
AG AG AA AA AG AG AA
50694579
1974 rs10040822 5 CC CC CC CC CC
CC CC CC CC CC CC CC CC
CC CC CC CC CC CC CC CC

	CC	CC	CC	CC	CC	CC	CC	CC
	CC	CC	CC	CC	CC	CC	CC	
177808157								
1975	rs10040896		5	GG	GG	GG	GG	GG
	GG	GG	GG	GG	GG	GG	GG	GG
	GG	GG	GG	GG	GG	GG	GG	GG
	GG	GG	GG	GG	GG	GG	GG	GG
	GG	GG	AG	GG	GG	GG	GG	
31931894								
1976	rs10040897		5	GG	GG	GG	AG	AG
	AG	GG	GG	GG	GG	GG	GG	GG
	GG	GG	GG	GG	GG	GG	GG	GG
	GG	GG	GG	GG	GG	GG	GG	GG
	GG	GG	GG	GG	GG	GG	GG	
43370188								
1977	rs10040902		5	AA	AA	AC	AA	AA
	AA	AA	AA	AA	AA	AC	AA	AC
	AA	AC	AA	AA	AA	AA	AC	AA
	AA	AA	AA	AA	AA	AA	AA	AA
	AA	AA	AA	AA	AA	AA	AA	
55896630								
1978	rs10040944		5	GG	GG	GG	AG	AG
	GG	AG	GG	GG	GG	GG	GG	GG
	GG	GG	GG	GG	GG	GG	GG	GG
	GG	GG	GG	GG	GG	GG	GG	GG
	GG	AG	GG	GG	GG	GG	GG	
178610730								
1979	rs10040966		5	GG	GG	GG	AG	AG
	AA	AG	GG	GG	GG	AG	GG	AG
	AG	AG	AG	AA	AG	AG	AG	AG
	AG	AG	AG	AG	AG	AG	AG	GG
	AG	GG	AG	GG	AG	AA	AG	
136427154								
1980	rs10040987							
5	--	--	--	--	--	--	--	--
	--	--	--	--	--	--	--	--
	--	--	--	--	--	--	--	--
	--	--	--	--	--	--	--	--
	--	--	--	--	816095			
1981	rs10041010		5	AA	AA	AA	AA	AA
	AA	AA	AA	AA	AA	AA	AA	AA
	AA	AA	AA	AA	AA	AA	AA	AA
	AA	AA	AA	AA	AA	AA	AA	AA
	AA	AA	AA	AA	AA	AA	AA	
170105482								
1982	rs10041012		5	AG	GG	AG	AG	AG
	GG	AA	AG	AG	AG	GG	GG	GG
	AG	AG	GG	AG	AG	GG	GG	GG
	GG	GG	GG	GG	GG	GG	GG	GG
	AG	AG	GG	GG	AG	GG	AG	

124177701								
1983	rs10041174		5	AA	AA	AA	AA	AG
	AG	AA	AA	AG	AA	AG	AA	AA
	AA	AA	AA	AA	AA	AA	AA	AA
	AG	AG	AA	AG	AA	AA	AA	AA
	AA	AA	AA	AA	AA	AA	AA	
11710193								
1984	rs10041204		5	GG	GG	AG	GG	GG
	GG	GG	GG	GG	GG	AG	GG	GG
	AG	GG	AG	AG	AG	GG	GG	GG
	GG	GG	GG	GG	GG	GG	GG	GG
	GG	GG	GG	GG	GG	GG	GG	
39702215								
1985	rs10041207		5	GG	GG	AG	AG	GG
	AG	GG	AG	AA	AG	AG	AA	AG
	GG	GG	GG	GG	GG	AG	GG	AG
	GG	GG	GG	GG	AG	AG	AG	AA
	GG	AG	AG	AA	AG	GG	AG	
19830072								
1986	rs1004122		X	GG	GG	GG	GG	AA
	GG	AG	AA	AG	GG	AG	GG	AG
	AG	AG	AG	AG	AA	AG	AA	GG
	GG	GG	AA	AA	AA	GG	AG	AA
	AG	AG	GG	AA	AG	AA	GG	
103825035								
1987	rs10041224		5	GG	AG	AG	GG	GG
	GG	GG	GG	GG	GG	GG	GG	AG
	AG	AG	GG	GG	GG	GG	AG	AG
	GG	GG	GG	GG	AG	GG	GG	AG
	AG	AG	GG	AG	GG	AG	GG	
168844301								
1988	rs10041231		5	AG	AG	AG	AG	GG
	GG	AG	AA	AA	AA	AA	AA	AA
	AA	AA	AA	AA	AA	AA	GG	AA
	GG	AG	AG	GG	AA	GG	AG	AG
	AA	AG	AA	AA	AA	AA	AG	
103170089								
1989	rs10041247		5	AA	AA	AA	AA	AA
	AA	AA	AA	AA	AA	AA	AA	AA
	AA	AA	AA	AA	AA	AA	AA	AA
	AA	AA	AA	AA	AA	AA	AA	AA
	AA	AA	AA	AA	AA	AA	AA	
25528829								
1990	rs1004125		1	CC	CC	CC	CC	AC
	AC	CC	AC	AC	AC	CC	CC	CC
	CC	CC	CC	CC	CC	AC	AC	AA
	CC	CC	AC	AC	CC	CC	CC	CC
	CC	CC	CC	CC	AC	CC	AC	
5540807								
1991	rs10041417		5	GG	GG	GG	GG	GG

	AG	GG	GG	GG	GG	GG	GG	GG
	AG	AG	GG	GG	GG	GG	GG	GG
	GG	GG	GG	GG	GG	GG	GG	GG
	GG	GG	GG	GG	GG	AG	GG	
33182745								
1992	rs10041463		5	GG	GG	GG	GG	GG
	GG	GG	AG	AG	AG	GG	GG	GG
	GG	GG	GG	GG	GG	GG	GG	GG
	GG	GG	GG	GG	GG	GG	GG	GG
	GG	GG	GG	GG	GG	GG	GG	
62612932								
1993	rs10041502		5	GG	GG	GG	GG	GG
	GG	GG	GG	GG	AG	GG	GG	GG
	GG	GG	GG	GG	GG	GG	GG	GG
	GG	GG	GG	GG	GG	GG	GG	GG
	GG	GG	GG	GG	GG	GG	GG	
54478686								
1994	rs1004151		17	CC	CC	CC	CC	CC
	CC	AC	CC	CC	AC	AC	AC	AC
	AA	AA	AC	AA	AA	CC	CC	CC
	CC	CC	AC	AC	AC	AC	CC	AC
	CC	CC	AA	AC	CC	AC	CC	
8275023								
1995	rs10041520		5	--	AA	--	AA	AA
	--	AA	--	--	--	AA	AA	AA
	--	--	AA	--	--	--	AG	AA
	--	--	AG	--	--	--	GG	--
	--	--	--	--	--	--	--	
142776684								
1996	rs10041589		5	CC	CC	CC	AC	CC
	AC	AC	CC	CC	CC	CC	CC	AC
	CC	AC	CC	CC	CC	CC	CC	CC
	CC	CC	CC	CC	AC	CC	CC	CC
	CC	AC	CC	AC	CC	CC	CC	
166573520								
1997	rs10041597		5	GG	GG	AG	GG	GG
	GG	GG	AG	AG	AG	AG	AA	AG
	AG	GG	GG	AG	AG	AG	AG	AG
	GG	GG	GG	GG	AG	GG	GG	AG
	GG	GG	GG	GG	GG	GG	AG	
33177406								
1998	rs10041720		5	AA	AC	AA	AC	AA
	AA	AC	AC	AC	AC	AA	AA	AA
	AA	AA	AC	AC	CC	AC	CC	AA
	AA	AA	AA	AA	AC	AC	AA	AC
	AA	AA	AC	AC	AC	CC	AA	
105993785								
1999	rs1004173		6	AG	AA	AG	AG	GG
	GG	AG	GG	AG	GG	GG	AG	AG
	AA	GG	GG	GG	AG	AG	AG	GG

	GG	GG	GG	GG	GG	AG	AG	AG
	AA	GG	GG	GG	GG	GG	GG	
47445017								
2000	rs10041745		5	AG	AG	AG	AG	AA
	AG	AG	AG	AG	AG	AG	AG	AA
	GG	GG	GG	AG	AG	AG	AA	AA
	AA	AA	AA	AA	AG	AG	AG	GG
	GG	AA	AA	AA	AA	AG	AG	
3281517								
2001	rs10041781		5	AA	AA	AA	AA	AA
	AA	AA	AG	AG	AG	AA	AA	AA
	AG	AG	AG	AG	AG	AA	AA	AA
	AA	AA	AA	AA	AA	AA	AA	AA
	AG	AG	AG	AA	AG	AG	AG	
123555271								
2002	rs10041792		5	GG	GG	AG	AG	AG
	AA	GG	GG	GG	GG	GG	GG	GG
	GG	GG	GG	GG	GG	GG	GG	GG
	AG	GG	GG	AG	AG	AG	AG	AA
	AG	GG	AG	GG	GG	GG	AG	
93032427								
2003	rs1004181		9	CC	CC	CC	CC	CC
	CC	AC	CC	CC	CC	CC	AC	CC
	CC	CC	CC	CC	CC	CC	CC	CC
	CC	CC	CC	CC	CC	CC	CC	CC
	CC	CC	CC	CC	CC	CC	CC	
138424923								
2004	rs10041822		5	AG	GG	GG	GG	GG
	GG	GG	AG	AA	AA	GG	GG	GG
	GG	GG	GG	GG	GG	GG	GG	GG
	GG	GG	GG	GG	AG	GG	AG	AG
	AG	GG	AG	AG	GG	GG	GG	
83855826								
2005	rs10041846		5	AA	AG	AG	AA	AA
	AA	AA	AA	AA	AA	AA	AA	AA
	AA	AA	AA	AA	AA	AA	AA	AA
	AA	AA	AA	AA	AA	AA	AA	AA
	AA	AA	AA	AA	AA	AA	AA	
8950621								
2006	rs1004187		16	AA	AA	AA	AA	AA
	AA	AA	AA	AA	AA	AA	AA	AA
	AA	AA	AA	AA	AA	AA	AA	AA
	AG	AA	AA	AG	AA	AA	AA	AA
	AA	AA	AA	AA	AA	AG	AG	
23916258								
2007	rs10041897		5	AG	AG	GG	GG	GG
	GG	GG	AG	AG	AG	GG	GG	GG
	GG	GG	AG	GG	AG	GG	GG	GG
	GG	GG	GG	GG	GG	AG	GG	GG
	GG	AG	AG	AG	AG	AG	GG	

169893751							
2008	rs10041935	5	AA	AC	AC	AC	AC
	AC	AA	AA	AA	AA	AA	AA
	AA	AA	AA	AA	AA	AA	AA
	AA	AA	AA	AA	AC	AA	AA
	AA	AA	AA	AA	CC	AC	AC
57214817							
2009	rs10041953	5	AG	AG	AG	AG	AG
	AG	AA	AG	GG	AG	AA	AA
	AA	AA	AG	AA	AG	GG	AA
	AG	GG	GG	AG	AG	GG	AG
	GG	GG	AA	AG	AA	AA	AA
111161200							
2010	rs10042003	5	AG	AA	AG	AG	AG
	AG	AG	AA	AG	AA	AG	AA
	AG	AG	AA	AA	AA	AA	AA
	AG	AG	AG	AG	AA	AA	AA
	AG	AA	AA	AA	AG	AG	AA
135842661							
2011	rs10042043	5	GG	GG	GG	GG	GG
	GG	GG	GG	GG	GG	AG	GG
	AG	AG	GG	GG	GG	GG	GG
	GG	GG	GG	GG	AG	GG	AG
	AG	GG	GG	GG	GG	GG	GG
15055447							
2012	rs1004206	19	AA	AA	AA	AA	AA
	AA	AA	AA	AA	AA	AG	AG
	GG	GG	AG	AA	AA	AA	AG
	AA	AA	AA	AA	AG	AA	AA
	AA	AA	AA	AA	AA	AA	AA
58106623							
2013	rs10042101	5	GG	GG	AG	GG	GG
	GG	GG	GG	GG	GG	GG	GG
	GG	GG	GG	GG	GG	GG	GG
	AG	AG	AG	AG	GG	GG	GG
	GG	GG	GG	GG	GG	GG	GG
9218895							
2014	rs1004212	14	GG	GG	GG	GG	AG
	GG	AG	GG	AG	GG	GG	AA
	AG	AG	GG	GG	GG	GG	AG
	GG	GG	GG	GG	AA	GG	AG
	GG	GG	GG	GG	GG	GG	GG
79181226							
2015	rs10042162	5	CC	CC	CC	CC	CC
	CC	CC	CC	CC	CC	CC	CC
	CC	CC	CC	CC	CC	CC	CC
	CC	CC	CC	CC	CC	CC	CC
	CC	CC	CC	CC	CC	CC	CC
19273700							
2016	rs1004222	1	GG	GG	GG	GG	GG

	GG	GG	GG	GG	GG	GG	GG	GG
	GG	GG	GG	GG	GG	GG	GG	GG
	GG	GG	GG	GG	GG	GG	GG	GG
	AG	GG	GG	GG	GG	GG	GG	
224888632								
2017	rs10042227		5	CC	CC	CC	CC	AC
	CC	AC	AC	AC	AC	AC	AC	AC
	CC	CC	CC	CC	CC	CC	CC	CC
	AC	AC	AC	AC	CC	AC	CC	AC
	CC	CC	AC	CC	CC	CC	CC	
125432585								
2018	rs10042251		5	AA	AA	AA	AA	AA
	AA	AA	AA	AA	AA	AA	AA	AG
	AA	AA	AA	AA	AA	AA	AA	AA
	AA	AA	AA	AA	AA	AA	AA	AA
	AA	AG	AA	AG	AA	AA	AA	
123869860								
2019	rs10042286		5	AA	AA	AA	AA	AA
	AA	AA	AA	AA	AA	AA	AA	AA
	AA	AA	AA	AA	AA	AA	AA	AA
	AA	AA	AA	AA	AA	AA	AA	AA
	AA	AA	AA	AA	AA	AA	AA	
125432793								
2020	rs10042288		5	AA	AA	AA	CC	CC
	CC	AC	AA	AC	AA	AC	AA	AC
	AA	CC	AC	AC	CC	AC	CC	AA
	AC	AC	AC	AC	AC	AC	CC	AA
	CC	AC	AA	AC	AC	CC	AA	
54120346								
2021	rs10042292		5	AA	AG	AG	GG	GG
	GG	GG	AA	AG	AA	AA	AG	AA
	AA	AG	AG	GG	AG	AG	AG	AG
	AG	AG	AG	AG	AA	AA	AA	AA
	AA	AG	AA	AG	AG	AG	AG	
33648317								
2022	rs10042293		5	CC	CC	CC	CC	CC
	CC	CC	CC	CC	CC	AC	AC	CC
	CC	CC	CC	CC	CC	AC	CC	CC
	CC	CC	AC	AC	AC	AC	CC	AC
	AC	CC	CC	CC	CC	CC	CC	
175010396								
2023	rs10042299		5	GG	GG	GG	AG	AG
	AA	AG	AG	AA	AG	GG	GG	GG
	GG	GG	AG	AG	GG	AG	GG	AG
	AA	AA	AA	AA	AG	AA	AG	AG
	AG	AA	AG	AA	AG	AA	GG	
139805611								
2024	rs1004230		X	AA	AA	AG	AA	AA
	AA	AA	AA	AA	AA	AA	AA	AA
	AA	AA	AA	AA	AA	AA	AA	AA

	AA	AA	AA	AA	AA	AA	AA	AA
	AA	AA	AA	AA	AA	AA	AA	
109152565								
2025	rs10042310		5	AA	AA	AA	AA	AA
	AA	AG	AG	AA	AG	AA	AG	AA
	AA	AA	AG	AA	AG	AG	AG	AA
	AA	AA	AA	AA	AA	AA	AA	AA
	AA	AA	AG	AA	AA	AA	AA	
24653755								
2026	rs1004245		7	AC	AA	AC	AC	AC
	AA	CC	AC	AC	AC	AA	AA	AA
	AC	AA	AC	AC	AC	CC	AC	AC
	CC	CC	CC	CC	CC	AC	AC	AC
	AC	AC	AA	AC	CC	AC	AC	
103088409								
2027	rs10042457		5	AG	AG	AG	GG	GG
	GG	GG	GG	GG	GG	GG	AG	AG
	GG	GG	GG	GG	GG	GG	GG	GG
	GG	GG	GG	GG	AG	GG	AG	AG
	GG	AG	GG	GG	GG	GG	AG	
125809560								
2028	rs10042476		5	GG	GG	GG	GG	GG
	GG	GG	GG	GG	GG	GG	GG	GG
	GG	AG	GG	GG	GG	GG	GG	GG
	GG	GG	GG	GG	GG	AA	AG	AG
	GG	GG	GG	GG	AG	AG	GG	
160585904								
2029	rs10042482		5	GG	GG	GG	AG	AG
	AA	AG	GG	GG	GG	AG	AG	AG
	GG	GG	GG	GG	GG	AG	AA	GG
	GG	GG	GG	GG	GG	AG	AG	AG
	AG	AG	AG	AG	AG	GG	AG	
171903310								
2030	rs10042507		5	AG	AG	GG	AG	AG
	GG	AG	AG	AA	AG	AA	GG	AG
	AG	AG	GG	GG	GG	AG	AA	AA
	AG	AG	AA	AA	AA	GG	AG	AG
	GG	GG	AG	AG	GG	GG	GG	
1927293								
2031	rs10042591		5	AG	AG	AG	GG	GG
	GG	GG	GG	GG	GG	GG	AA	AG
	AG	AG	AG	GG	AG	AG	AG	GG
	GG	GG	GG	GG	AA	GG	AG	AG
	GG	GG	GG	GG	AG	AG	GG	
100449604								
2032	rs10042606		5	AG	AA	AG	GG	GG
	AG	GG	GG	GG	GG	GG	AG	AG
	GG	GG	GG	GG	GG	AG	AA	GG
	GG	AG	AG	GG	AG	GG	GG	AG
	GG	GG	GG	GG	AG	GG	AG	

58479670
2033 rs1004262 22 AA AA AA AA AA
AA AA AA AA AA AA AA AA
AA AA AA AA AA AA AA AA
AA AA AA AA AA AA AA AA
AA AA AA AA AA AA AA
41483324
2034 rs10042703
5 -- -- -- -- -- -- -- --
-- -- -- -- -- -- -- --
-- -- -- -- -- -- -- --
-- -- -- -- -- -- -- --
-- -- -- -- 29351490
2035 rs10042752 5 AC CC AC AC AC
AA AC AC AA AC AA AC AC
AC AA AA AA AA AC CC AA
AA AA AA AA AA AA AA AA
AA AA AA AA AC AA AC
52294551
2036 rs10042857 5 AA AA AA AA AA
AA AG AA AA AA AG AG GG
AA AG AG AG AA AA AA AA
AA AA AA AA AA AA AA AA
AA AA AA AA AA AG AA
64379812
2037 rs10042881
5 -- -- -- -- -- -- -- --
-- -- -- -- -- -- -- --
-- -- -- -- -- -- -- --
-- -- -- -- -- -- -- --
-- -- -- -- 164279457
2038 rs10042924 5 AC CC AC CC CC
AC AC AC AA AC CC CC AC
AA AA AA AA AA AC AA AC
AC AC AA AA AC AC CC AC
AA AA AA AA CC AC AC
144048517
2039 rs1004299 16 AG AG GG AG AG
AA AG AG AG AG AG AA AG
AG AA AG AG AG AA AA AG
AG AG AG AG GG AG GG AG
AA AG GG AG AG AA GG
54343840
2040 rs10042997 5 AG AA AG AG AG
AG GG AG AG AG GG GG GG
GG GG GG GG GG GG GG AG
AG AG AG AG GG AG AG AG
AG GG GG GG AA AG AG
161912216
2041 rs1004302 2 AG AG AG GG GG

	GG	GG	GG	GG	GG	GG	GG	GG
	GG	GG	AA	AA	AA	GG	AG	GG
	AG	AG	GG	GG	GG	GG	GG	GG
	AG	AA	AG	AG	AG	AG	GG	
216010489								
2042	rs10043028		5	AC	CC	CC	CC	CC
	CC	CC	CC	CC	CC	AC	AC	CC
	AC	AC	CC	CC	CC	CC	CC	CC
	CC	CC	CC	CC	CC	CC	CC	CC
	CC	CC	AC	AC	AC	AA	CC	
2809055								
2043	rs10043031		5	AA	AG	GG	GG	GG
	GG	GG	GG	AG	GG	AG	AG	AG
	GG	GG	AG	GG	AG	AG	GG	GG
	GG	GG	AG	GG	GG	GG	GG	GG
	AG	AG	GG	AG	GG	GG	GG	
10260247								
2044	rs10043093		5	AC	CC	AC	AC	AA
	AA	AC	AC	AC	AC	CC	AA	AC
	AC	AC	CC	CC	CC	CC	CC	CC
	CC	CC	CC	CC	CC	AC	AC	CC
	AC	AA	CC	AC	AC	AA	AC	
40324220								
2045	rs10043127		5	AG	AG	AA	AG	GG
	AG	AG	AG	AA	AG	AG	AG	AG
	AG	AG	AA	AA	AA	AA	AA	AA
	AA	AA	AA	AA	AG	AA	AA	AA
	AG	GG	AA	AG	AA	AA	AG	
12280849								
2046	rs1004315		12	AG	GG	GG	AA	AG
	AG	AA	AA	AG	AA	AG	AG	GG
	AG	GG	GG	GG	GG	GG	AG	GG
	AG	AG	GG	AG	AG	GG	GG	AG
	GG	GG	AG	AG	GG	GG	GG	
126196117								
2047	rs10043173		5	AA	AA	AA	AA	AA
	AA	AA	AA	AA	AA	AA	AA	AA
	AA	AA	AG	AA	AG	AA	AA	AA
	AA	AA	AA	AA	AG	AA	AG	AG
	AA	AA	AA	AA	AA	AA	AA	
110303365								
2048	rs1004327		4	AG	AA	AA	AA	AA
	AA	AG	AG	AG	AG	AG	GG	AG
	AG	AG	GG	AG	AG	AG	AG	AA
	AG	GG	AG	AA	AG	AG	GG	AG
	AA	AG	AA	AA	AA	AA	AG	
10511483								
2049	rs10043301		5	AA	AA	AG	AA	AA
	AA	AA	AA	AA	AA	GG	AA	AG
	AG	AG	AG	AG	AG	AG	GG	AA

AA AG AG AA AG AA AG AA
AG AG AG AG GG GG AG
40530886
2050 rs10043349 5 GG GG GG GG GG
GG GG GG GG GG GG GG GG
GG GG GG GG GG GG GG GG
GG GG GG GG GG GG GG GG
GG GG GG GG GG GG GG
124430309
2051 rs1004340 7 GG AG AG GG GG
AG GG AG AG GG AG AG AA
AG AG AG AA AA GG GG GG
AG AG AG AG AA GG AG AG
AA GG AA AG GG GG GG
151099634
2052 rs10043438 5 AA AA AG AG AG
AA AG AA AA AA AA AG AA
AA AA AG AA AG AG AA AG
AG AG AG AG AG AA AA AA
AG AG AG AG AG AG AA
94297348
2053 rs10043442 5 AC AA AC AC AA
AA AC AC AC AC AC AC AA
AA AA AC AC AC AA AC AA
AC AC AC AC AA AC AA AC
AC AC AC AA AC AC AA
123129656
2054 rs10043453 5 AC AC AA AC AC
AA AC AC CC CC AC AC CC
CC CC CC CC CC CC CC CC
CC CC AC AC CC CC CC CC
AA AC AC AA AC CC AC
179631722
2055 rs10043454 5 AG AA AG AA AA
AA AA AG AG AG AG AG AG
AG AA AA AG AG AA AA AG
AA AA AA AA AG AA AA AG
AA AA AA AA AG AG AA
164172766
2056 rs10043517 5 AA AA AG AA AA
AA AG AA AA AA AA AA AA
AG AA AA AA AA AA AA AA
AA AA AA AA AA AG AG AA
AG GG AA AG AG AA AG
57444953
2057 rs10043527 5 GG GG GG AG AG
AG GG GG GG GG AG AG AG
AG AG GG GG GG AG GG GG
GG GG GG GG AG AG AA AG
GG GG GG GG GG GG GG

159051970								
2058	rs1004353							
X	--	--	--	--	--	--	--	--
	--	--	--	--	--	--	--	--
	--	--	--	--	--	--	--	--
	--	--	--	--	--	--	--	--
	--	--	--	--	141285862			
2059	rs10043532		5	GG	GG	GG	GG	GG
	GG	GG	AG	AG	AG	GG	GG	GG
	GG	GG	AG	AG	GG	GG	GG	GG
	GG	GG	GG	GG	GG	AG	AG	GG
	GG	GG	GG	GG	GG	GG	GG	
21428130								
2060	rs10043543		5	AG	AG	GG	AG	AG
	GG	AG	GG	GG	AG	GG	AG	AG
	GG	GG	AG	AG	AA	GG	GG	AG
	GG	GG	GG	GG	AG	AG	GG	AA
	AG	GG	AG	AG	AG	AG	GG	
55927125								
2061	rs10043546		5	AG	AG	AG	GG	GG
	AG	GG	GG	GG	GG	AG	GG	AG
	AG	AG	AG	GG	GG	GG	GG	GG
	GG	GG	GG	GG	GG	AG	GG	AG
	GG	GG	GG	GG	AG	GG	AA	
81993843								
2062	rs1004356		12	AG	AG	AG	AG	AA
	AG	AA	AA	AA	AA	AG	AA	AG
	AA	AG	AA	AA	AA	AA	AA	AA
	AG	GG	GG	AG	AA	AA	AA	AA
	AG	AA	AA	AA	AA	AA	AA	
117777372								
2063	rs10043572		5	AG	GG	AG	AG	GG
	GG	AG	AG	AG	AG	GG	GG	GG
	GG	GG	AG	GG	AG	GG	AG	GG
	GG	GG	GG	GG	GG	GG	GG	GG
	AG	AG	GG	GG	GG	GG	GG	
104490887								
2064	rs10043585		5	AA	AA	AA	AA	AA
	AA	AA	AA	AA	AA	AA	AA	AA
	AA	AA	AA	AA	AA	AA	AA	AA
	AA	AA	AA	AA	AA	AA	AA	AA
	AA	AA	AA	AA	AA	AA	AA	
41592708								
2065	rs10043596		5	CC	CC	CC	CC	CC
	CC	CC	CC	CC	CC	CC	CC	CC
	AC	CC	CC	CC	CC	CC	CC	CC
	CC	CC	CC	CC	CC	CC	CC	CC
	CC	CC	CC	CC	AC	CC	AC	
53354379								
2066	rs10043614		5	GG	GG	GG	GG	GG

	GG	GG	AG	AG	AG	AA	AA	AG
	GG	GG	GG	AG	AG	AG	AG	GG
	AG	GG	AG	AG	AG	AG	AG	AG
	GG	GG	AA	AG	AG	GG	AA	
166341947								
2067	rs1004362		9	AG	AA	AG	GG	GG
	GG	GG	GG	GG	GG	GG	AG	AG
	AG	GG	GG	GG	GG	GG	GG	GG
	GG	GG	GG	GG	GG	AG	GG	GG
	GG	AG	GG	GG	GG	GG	GG	
104390423								
2068	rs1004363		16	AG	GG	AG	AG	AG
	AA	AG	AA	AA	AA	AA	AA	AA
	AA	AA	AA	AG	AA	AG	GG	AG
	AA	AA	AA	AG	AA	AG	AG	AA
	AA	AA	GG	AG	AG	AG	AA	
57595999								
2069	rs10043634		5	AA	AA	AA	AA	AA
	AA	AA	AG	AG	AA	AA	AA	AA
	AA	AA	AA	AA	AA	AA	AA	AA
	AA	AA	AA	AA	AA	AA	AA	AA
	AA	AA	AA	AA	AA	AA	AA	
64118974								
2070	rs10043664		5	AC	AC	AC	CC	CC
	AC	CC	CC	CC	AC	CC	CC	CC
	CC	CC	CC	CC	CC	CC	CC	CC
	CC	CC	CC	CC	CC	CC	CC	CC
	CC	CC	CC	CC	CC	CC	CC	
54428310								
2071	rs10043919		5	GG	AG	AG	AA	AA
	AG	AA	AG	GG	AG	AG	AG	AG
	AG	AG	AG	AA	GG	AG	AG	AG
	GG	GG	AG	AG	GG	AG	AG	AG
	AA	AG	AG	AG	AG	GG	AA	
178165521								
2072	rs10043960		5	AA	AA	AA	AG	AG
	AG	AG	AA	AA	AG	AG	AA	AA
	AA	AA	AA	AA	AA	AA	AG	AA
	AG	AG	AG	AG	AA	AA	AA	AA
	GG	AA	AA	AA	AA	AA	AA	
74266343								
2073	rs10044000		5	AA	AG	AA	AA	AG
	AG	AG	AA	AG	AG	AG	GG	GG
	GG	GG	GG	AG	AG	GG	AG	AG
	AG	AG	AA	AA	GG	AA	AG	AG
	AA	AG	GG	AG	AA	AG	AG	
134343661								
2074	rs1004402		7	CC	CC	CC	CC	AC
	AC	CC	CC	CC	CC	CC	CC	CC
	CC	CC	CC	CC	CC	AC	CC	AC

	CC	CC	AC	AC	CC	CC	CC	CC
	CC	CC	CC	CC	CC	CC	CC	
50755208								
2075	rs10044023		5	GG	GG	GG	GG	AG
	AG	AG	GG	GG	GG	AG	AG	AA
	GG	AG	GG	GG	GG	GG	GG	GG
	GG	GG	GG	GG	GG	GG	GG	GG
	AG	AG	AA	AG	GG	GG	AG	
29286698								
2076	rs10044031							
5	--	--	--	--	--	--	--	--
	--	--	--	--	--	--	--	--
	--	--	--	--	--	--	--	--
	--	--	--	--	--	--	--	--
	--	--	--	--	180704486			
2077	rs10044122		5	GG	AG	AG	AG	AG
	GG	AA	AG	AA	AG	AG	AA	AA
	AG	AG	AG	AA	AG	AA	GG	AG
	AG	GG	GG	GG	AG	AG	AG	GG
	AG	AA	AG	AA	AG	AG	AA	
115330927								
2078	rs10044148		5	AA	AA	AA	AA	AA
	AA	AA	AA	AA	AA	AA	AA	AA
	AA	AA	AA	AA	AA	AA	AA	AA
	AA	AA	AA	AA	AA	AA	AA	AA
	AA	AA	AA	AA	AA	AA	AA	
13804691								
2079	rs10044159		5	AA	AA	AA	AA	AA
	AC	AA	AC	AC	AC	AA	AA	AA
	AA	AA	AA	AA	AA	AA	AA	AA
	AA	AA	AA	AA	AC	AA	AA	AC
	AA	AA	AA	AA	AA	AA	AA	
58414048								
2080	rs1004420		1	CC	CC	CC	CC	CC
	AC	CC	CC	CC	CC	CC	CC	CC
	CC	CC	CC	CC	CC	AC	AC	AC
	AC	CC	AC	CC	AC	CC	CC	CC
	CC	AC	AC	AC	CC	CC	AC	
32729702								
2081	rs10044238		5	AA	AG	AG	AG	AG
	AG	AA	AG	GG	AG	GG	GG	AG
	AG	AG	AA	AA	AA	AG	AA	AA
	AG	GG	GG	AG	GG	AG	AG	GG
	AG	GG	AG	AG	AA	AG	AA	
107930254								
2082	rs10044281		5	AA	AA	AA	AA	AA
	AA	AA	AA	AA	AA	AA	AA	AA
	AG	AG	AA	AA	AA	AA	AA	AA
	AA	AA	AA	AA	AA	AA	AA	AA
	AA	AA	AA	AA	AA	AA	AA	

174990352							
2083	rs10044294	5	CC	AC	AC	CC	CC
	CC	CC	CC	CC	CC	AC	CC
	CC	CC	CC	CC	AC	AC	CC
	CC	CC	CC	AC	AA	AC	AC
	AA	CC	AC	CC	AC	AC	AC
82147653							
2084	rs10044331	5	AA	AA	AA	AG	AG
	AG	AA	AA	AA	AA	AG	AG	AG
	AG	AG	AA	AA	AA	AA	AG	AA
	AA	AA	AA	AA	AA	AA	AA	AA
	AG	AG	AA	AA	AA	AA	AA
159261646							
2085	rs10044342	5	AG	AG	GG	GG	GG
	AG	GG	AG	AG	AG	AA	AA	AA
	AA	AA	AG	AG	AG	AG	AA	AA
	AA	AA	AA	AA	AG	AA	AA	AA
	AG	AG	AA	AA	AA	AG	AG
88178683							
2086	rs10044427	5	AA	AC	AA	AA	AA
	AA	AA	AA	AA	AA	AA	AA	AA
	AA	AA	AC	AC	AA	AA	AA	AA
	AA	AA	AA	AA	AC	AA	AA	AC
	AA	AA	AA	AA	AA	AA	AA
33811670							
2087	rs10044441	5	AC	AC	AC	AA	AA
	AA	AA	AA	AC	AA	AC	AC	AC
	AA	AA	AA	AA	AC	AC	AC	AC
	AC	AC	AA	AA	AA	AA	AA	AA
	AA	CC	AC	CC	AC	AA	CC
1106667							
2088	rs10044494	5	GG	GG	GG	GG	GG
	GG	GG	GG	GG	GG	GG	GG	GG
	GG	GG	GG	GG	GG	GG	GG	GG
	GG	GG	GG	GG	GG	GG	GG	GG
	GG	GG	GG	GG	GG	GG	GG
43769162							
2089	rs1004451	15	GG	GG	GG	GG	GG
	GG	GG	GG	AG	AG	AG	AG	AG
	AG	AG	AG	GG	AG	AG	GG	GG
	GG	AG	AG	AG	AG	AA	AG	AG
	GG	AG	AG	GG	GG	GG	GG
39104125							
2090	rs10044546	5	CC	CC	CC	CC	CC
	AC	AC	CC	CC	CC	AC	CC	CC
	CC	CC	CC	AC	AC	CC	AC	AC
	CC	CC	CC	CC	CC	CC	CC	CC
	CC	CC	CC	CC	CC	AC	CC
75337594							
2091	rs10044555	5	AC	AA	AC	CC	AC

	AC	AC	AA	AA	AA	AC	AC	AC
	AA	AA	AC	AC	CC	AA	AC	AA
	AC	AC	AA	AC	AC	AC	AC	CC
	AC	AC	AA	AC	AC	AA	AC	
30125447								
2092	rs10044570		5	CC	AC	CC	CC	CC
	CC	CC	AA	AC	AA	CC	AC	CC
	CC	CC	CC	CC	CC	CC	CC	CC
	CC	CC	CC	CC	CC	AC	CC	AC
	CC	CC	CC	CC	CC	AC	CC	
32760773								
2093	rs10044576		5	AA	AA	AA	AA	AA
	AA	AA	AG	AG	AG	AA	AA	AA
	AA	AA	AA	AA	AA	AG	AA	AA
	AA	AA	AA	AA	AA	AA	AA	AA
	AG	AA	AA	AA	AA	AA	AA	
113486177								
2094	rs10044577		5	GG	GG	GG	GG	GG
	GG	GG	GG	GG	GG	GG	AG	GG
	GG	GG	GG	GG	GG	GG	GG	GG
	GG	GG	GG	GG	GG	GG	GG	GG
	GG	GG	GG	GG	GG	GG	GG	
11011856								
2095	rs1004459		8	GG	GG	GG	GG	GG
	GG	GG	GG	GG	GG	GG	GG	GG
	GG	GG	AG	GG	AG	GG	AG	GG
	GG	GG	GG	GG	GG	AG	GG	GG
	GG	GG	GG	GG	AG	GG	AG	
108440057								
2096	rs10044618		5	GG	GG	AG	AA	AA
	AA	AA	AG	AG	AG	AG	AG	AG
	AG	AG	AG	AA	AA	AA	GG	AG
	AG	AG	AG	AG	AA	GG	AG	AG
	AG	AG	AG	AG	AG	GG	AA	
87781168								
2097	rs10044684		5	AG	AA	AG	AG	AG
	AG	AA	AA	AA	AA	AG	AG	AG
	AA	AA	AA	AA	AA	AG	AA	AG
	AG	AG	AG	AG	AA	AA	AA	AA
	AG	--	GG	AG	AA	--	AA	
76455453								
2098	rs10044689		5	AG	AG	AG	AA	AA
	AG	AA	AG	AG	AA	GG	AA	AG
	GG	GG	AG	AA	AA	AA	AA	AA
	AA	AA	AA	AA	AA	AG	AA	AG
	AG	AG	AA	AG	AG	AG	GG	
81990976								
2099	rs10044704		5	GG	GG	GG	GG	GG
	GG	GG	GG	GG	GG	GG	GG	GG
	GG	GG	GG	GG	GG	GG	GG	GG

	GG	GG	GG	GG	GG	GG	GG	GG
	GG	GG	GG	GG	GG	GG	GG	
124458545								
2100	rs10044726		5	AG	AG	AA	AG	AG
	AG	AA	AG	AG	AG	AG	AG	AG
	AA	AA	AA	AG	AG	AA	AA	AG
	AA	AA	AA	AA	AA	AA	AA	AA
	GG	AA	AA	AA	AG	AA	GG	
170953973								
2101	rs1004474		18	GG	AG	AG	AG	GG
	AG	GG	GG	AG	GG	AG	GG	GG
	AG	GG	AG	AG	AG	GG	GG	GG
	GG	AG	AG	GG	GG	AG	GG	AG
	AG	AG	GG	AG	AG	GG	AG	
660383								
2102	rs10044742		5	GG	GG	AG	GG	GG
	GG	GG	GG	GG	GG	AG	AG	AG
	GG	GG	GG	GG	GG	AG	GG	GG
	GG	GG	GG	GG	GG	GG	GG	GG
	GG	GG	GG	GG	GG	AG	GG	
30112930								
2103	rs10044756		5	GG	GG	GG	GG	GG
	GG	GG	GG	AG	GG	AG	GG	GG
	GG	GG	AG	AG	GG	AG	GG	AA
	AG	AA	AA	AG	GG	AG	AG	GG
	GG	GG	GG	GG	GG	GG	GG	
110939806								
2104	rs10044803		5	AG	GG	GG	AG	GG
	AG	GG	AG	GG	AG	GG	GG	GG
	GG	GG	GG	GG	GG	AG	AA	AG
	GG	GG	GG	GG	GG	AG	GG	AG
	AA	GG	GG	GG	GG	GG	GG	
24320108								
2105	rs10044817		5	AA	AA	AG	AA	AA
	AA	AA	AA	AA	AA	AG	AG	AA
	AG	AG	AA	AG	AG	AA	AA	AA
	AA	AA	AA	AA	AA	AA	AA	AA
	AA	AA	AA	AA	AA	AA	AA	
5273945								
2106	rs1004485		17	GG	GG	GG	GG	AG
	AG	AG	GG	GG	GG	GG	GG	GG
	GG	GG	AG	AG	AG	GG	GG	GG
	GG	AG	AG	AG	GG	GG	GG	GG
	GG	GG	GG	GG	GG	GG	GG	
45053997								
2107	rs10044883		5	AG	GG	AG	AA	AA
	AG	AG	AG	AG	AG	AA	AG	AG
	AG	GG	AG	AG	GG	GG	AG	GG
	AA	AG	AG	AA	GG	GG	GG	GG
	GG	AG	GG	AG	AG	GG	AA	

35885710
2108 rs10044914 5 GG GG AG AA AA
AG AA AA AA AA AG AA AA
AA AA AG AG AA AG AA AG
AA AA AA AA AA AG AG AA
GG GG AG GG AA AA AA
107320849
2109 rs10044936 5 GG GG GG GG GG
GG GG GG GG GG GG GG GG
GG GG GG GG GG GG GG GG
GG GG GG GG GG GG GG GG
GG GG GG GG GG GG GG
140653258
2110 rs1004496 15 CC AC AC AC AC
CC AC CC CC CC AC AC AA
AC AA AC AC AC AC AA AC
CC CC CC CC CC CC CC CC
CC AA AC AA CC CC CC
38941557
2111 rs10045011 5 GG GG GG GG GG
GG GG GG GG GG GG AG GG
GG GG AG AG AG AG AG GG
GG GG GG GG GG GG GG GG
AG GG GG GG GG GG GG
39175369
2112 rs10045084 5 AA AA AA AA AA
AA AG AG AG AG AA AG AG
GG AA GG GG GG AG GG AA
AG AG AG AG AG AG AG AG
AA GG AG GG AG AG AG
55333925
2113 rs10045155 5 GG GG GG GG GG
GG GG GG GG AG GG GG GG
GG GG GG GG GG GG GG GG
AG AG AG AG GG GG GG GG
GG GG AG AG GG GG GG
75757556
2114 rs10045194 5 GG AG GG GG GG
GG GG GG GG GG GG GG GG
GG GG GG GG GG GG GG GG
GG GG GG GG GG GG GG GG
GG AG GG AG AG AA GG
160665145
2115 rs10045249 5 AG -- AG -- --
AG -- AA AA AA -- -- --
AG AG -- -- -- AG -- AG
-- -- -- -- AG -- AG --
AG AG -- AG -- AG AG
123399421
2116 rs1004526 19 AA AG AG AA AA

	AA	AA	AA	AG	AG	AG	AG	AG
	AA	AG	AA	AG	AG	AG	AA	AG
	AG	AG	AG	AG	AA	AG	AG	AG
	AA	AA	AG	AA	AA	AG	AG	
58588849								
2117	rs10045264		5	GG	GG	AG	AG	AG
	AG	AG	GG	GG	GG	AA	AG	AA
	AG	AG	AG	AA	AA	AG	AG	AA
	AG	AA	AA	AG	AG	AG	AG	AG
	AG	AG	AG	AG	AG	GG	AA	
44886432								
2118	rs10045268		5	AG	AG	AG	AA	AA
	AA	AA	AG	AG	AG	AA	AA	AA
	AG	AG	AA	AA	AA	AG	AG	AG
	AG	AG	AG	AG	AG	AA	AA	AA
	AG	AA	AG	AA	AG	AG	AA	
165087285								
2119	rs10045279		5	GG	GG	AG	GG	GG
	GG	GG	GG	GG	GG	GG	GG	GG
	GG	GG	GG	GG	GG	GG	GG	GG
	GG	GG	GG	GG	GG	GG	GG	GG
	GG	GG	GG	GG	GG	GG	GG	
27913746								
2120	rs10045325		5	GG	GG	GG	GG	GG
	GG	GG	GG	GG	GG	GG	GG	GG
	GG	GG	GG	GG	GG	AG	AG	GG
	GG	GG	GG	GG	GG	GG	GG	GG
	GG	GG	GG	GG	AG	GG	AG	
125672801								
2121	rs10045332		5	AA	AA	AC	AC	AC
	CC	AA	AA	AA	AA	AA	AC	AA
	AA	AA	AC	AC	AA	AA	AA	AA
	AA	AA	AC	AA	AA	AA	AA	AA
	AC	AA	AC	AA	AA	AC	AC	
28560977								
2122	rs10045335		5	AG	AA	AA	AA	AA
	AA	AA	AA	AG	AA	AA	AA	AA
	AA	AA	AA	AA	AA	AA	AG	AA
	AA	AA	AA	AA	AA	AA	AA	AA
	AG	AA	AG	AA	AA	AA	AA	
96902807								
2123	rs10045385		5	AA	AA	AA	AA	AA
	AA	AA	AA	AA	AA	AA	AA	AA
	AA	AA	AA	AA	AA	AG	AG	AG
	AA	AA	AA	AA	AA	AA	AA	AA
	AG	AA	AA	AA	AG	AA	AG	
157822886								
2124	rs10045392		5	AG	AG	AA	AG	AG
	GG	AG	AG	GG	AG	GG	AG	GG
	AG	GG	GG	GG	GG	GG	GG	GG

GG GG GG GG GG AG AG GG
GG GG GG GG GG GG GG
51279897
2125 rs10045408 5 AG AA AA GG GG
GG GG GG GG AG AA GG AG
AG AG AG AA AG AA AG AG
AG GG AG AG AA AG AG AA
AG AA AG AA AG AG AG
76967147
2126 rs10045427 5 AA AA AA AA AA
AA AA AA AA AA AA AA AA
AA AA AA AA AA AC CC AC
CC CC CC CC AA AC AA AA
AC AC CC AC AA AA AA
148869387
2127 rs10045431 5 AC CC AC CC AC
CC AC CC CC CC AC AC AC
CC CC AC CC AC AC AC AC
CC CC CC CC AC AC AC AA
AA CC CC CC CC CC CC
158814533
2128 rs10045432 5 AC AC AC AC AC
AC CC CC CC CC CC CC CC
CC CC CC CC CC AC CC AC
CC CC CC CC CC CC CC CC
CC CC AC CC CC CC CC
43865891
2129 rs10045440 5 GG GG GG GG GG
GG GG AG AG AG GG GG GG
GG GG AG AG GG GG GG GG
GG GG GG GG GG GG GG GG
GG GG GG GG GG GG AG
21872292
2130 rs10045518 5 AA AA AA AG AA
AA AG AA AA AA AA AA AA
AG AG AA AA AA AA AA AA
AA AA AA AA AA AA AA AA
AG AA AA AA AG AG AA
149675264
2131 rs10045527 5 AA AA AA AA AA
AA AA AA AA AA AA AA AA
AA AA AA AA AA AA AA AA
AA AA AA AA AA AA AA AA
AA AA AA AA AA AA AA
30765016
2132 rs10045539 5 AA AA AA AG AA
AA AG AG AG AG AG AG AG
AA AA AG AG AA AG AG AA
GG GG GG GG AA AA AA AA
AG AG AA AA AG AA GG

166795002								
2133	rs10045606	5	GG	GG	GG	AA	AA	
	AA	AA	AA	AG	AG	AG	AG	AG
	AG	AG	AA	AA	AA	AG	GG	AG
	AG	AG	AG	AG	AA	AA	AA	AA
	AA	AA	AA	AA	GG	GG	AG	
157523151								
2134	rs1004564	4	AC	AC	AC	CC	AC	
	AC	CC	CC	CC	AC	AC	AA	AA
	AC	CC	CC	CC	CC	AC	AC	AC
	AC	CC	CC	CC	AA	AC	AC	AC
	CC	AC	AC	AC	AC	AA	CC	
55242625								
2135	rs10045723	5	GG	GG	GG	AG	AG	
	GG	AG	GG	GG	AG	GG	GG	AG
	GG	AG	AG	GG	AG	GG	AG	GG
	GG	GG	GG	GG	AG	AG	GG	AG
	GG	AG	AG	AA	AG	GG	AA	
13991502								
2136	rs10045727	5	GG	GG	AG	GG	GG	
	GG	AG	GG	GG	GG	AG	GG	GG
	GG	GG	AG	AG	AG	AG	AG	GG
	AG	GG	AG	AG	GG	GG	GG	GG
	GG	GG	GG	GG	AG	AG	AG	
75226673								
2137	rs10045730	5	GG	GG	GG	GG	GG	
	GG	GG	GG	GG	GG	GG	GG	GG
	GG	GG	GG	GG	GG	GG	GG	GG
	GG	GG	GG	GG	GG	GG	GG	GG
	GG	GG	GG	GG	GG	GG	GG	
35411297								
2138	rs10045762							
5	--	--	--	--	--	--	--	--
	--	--	--	--	--	--	--	--
	--	--	--	--	--	--	--	--
	--	--	--	--	--	--	--	--
	--	--	--	--	124722116			
2139	rs1004586	2	GG	GG	GG	AA	AG	
	AG	AA	GG	GG	GG	GG	AG	AG
	GG	AG	AG	AG	AG	AA	AA	AG
	AG	AA	AG	AG	GG	AG	AG	GG
	GG	AG	GG	AG	AG	AG	GG	
23262942								
2140	rs10045861	5	AC	CC	AC	AC	AC	
	AC	AA	CC	CC	CC	AC	AC	AC
	AC	CC	CC	CC	CC	CC	CC	CC
	CC	CC	CC	CC	AC	CC	CC	AC
	AA	CC	CC	CC	AC	AC	CC	
163927797								
2141	rs1004592	7	AA	AC	CC	AC	AA	

	AC	AA	CC	AC	CC	CC	CC	CC
	CC	AC	AC	AC	AC	AC	CC	AC
	AC	AA	AC	AC	AA	AA	AA	AA
	AC	AC	AA	AA	AC	AC	AC	
131517925								
2142	rs10045935		5	AG	AA	AG	AG	AA
	AG	AG	AA	AG	AA	GG	AG	GG
	GG	GG	AA	AG	AG	AG	AA	AG
	AG	AG	AG	AG	AG	AA	AG	AG
	AG	GG	AG	GG	AG	GG	AA	
99302219								
2143	rs10045958		5	GG	GG	GG	GG	GG
	GG	GG	GG	GG	GG	GG	GG	GG
	GG	GG	GG	GG	GG	GG	GG	GG
	GG	GG	GG	GG	GG	GG	GG	GG
	GG	GG	GG	GG	GG	GG	GG	
56996523								
2144	rs10045972		5	AG	AG	AG	AA	AA
	AA	AA	AG	AG	AG	AA	AA	AG
	AA	AG	AA	AA	AA	AG	AG	AG
	AA	AG	AA	AA	AA	AG	AG	AG
	AA	AA	AG	AG	AA	AA	AG	
168089461								
2145	rs1004599		18	AA	AG	GG	AA	AA
	AA	AA	AA	AA	AA	AA	AA	AA
	AA	AA	AA	AA	AA	AA	AA	AA
	AA	AA	AA	AA	AA	AA	AA	AA
	AG	AG	AA	AG	AA	AG	AA	
33396298								
2146	rs10046044		5	GG	GG	GG	AG	AG
	AG	AA	AG	AG	AG	AG	GG	AG
	GG	GG	GG	AG	AG	GG	AG	GG
	GG	GG	GG	GG	GG	AA	AG	AG
	GG	AG	GG	AG	GG	AG	GG	
117795654								
2147	rs10046065		5	AC	CC	AC	CC	AC
	CC	AC	AC	AC	AC	CC	CC	AC
	CC	CC	CC	CC	CC	CC	CC	CC
	AC	AC	AC	AC	CC	CC	CC	CC
	CC	AA	CC	AC	CC	AC	CC	
123867087								
2148	rs10046131		6	AC	AC	AC	AC	AA
	AC	AC	AC	CC	AC	AC	AC	AC
	AC	AC	AC	AC	CC	AC	CC	AC
	AC	AC	AA	AC	AC	AA	AC	AA
	AC	AC	AA	AA	AC	CC	AC	
3018982								
2149	rs1004617		9	AG	AG	AA	GG	GG
	GG	GG	GG	GG	GG	AG	AG	AA
	AA	AG	AA	AG	AG	AG	GG	GG

AG GG AG GG GG AA AG AG
AA AA AA AA AG AG AA
29350850
2150 rs10046303 6 GG GG GG GG GG
GG GG GG AG GG GG GG GG
GG GG AG AA GG GG GG AG
AG AG GG AG GG GG GG GG
GG GG GG GG GG AG GG
102870989
2151 rs1004632 6 GG GG GG AG AG
AA GG GG GG AG GG GG GG
GG GG AG GG GG AG AG AG
AG AG AG AG AG AG AG AG
AG AG AA AA AG AG GG
36252839
2152 rs10046330 6 AG AA AG AG AG
AG AG AA AG AA GG AG AG
AG AG GG AG AG AG AG AA
AG AG AG AG AA GG AG AG
AA AG AG AA AG AA AG
168497446
2153 rs10046510 7 AG AA AA AG AG
GG AA AA AG AA AA AA AA
AG AG AA AA AA AG AG AG
AA AG AG AA AA AG AG AG
AG AG AG GG AG AG GG
52084694
2154 rs10046543 7 AG AG GG GG GG
GG GG AG AG AG GG GG GG
GG GG AG GG GG AG GG AG
AG AA AA AG AA GG AG AG
AG AG AA AG GG GG GG
124349606
2155 rs10046720 8 AA AA AA AA AA
AA AA AA AA AA AA AA AA
AA AA AA AA AA AA AA AA
AA AA AA AA AA AA AA AA
AA AA AA AA AA AA AA
91620661
2156 rs10046992 X GG GG GG GG GG
-- GG GG GG GG GG GG GG
GG GG GG GG GG GG GG GG
GG GG GG GG GG GG GG GG
GG GG GG GG GG GG GG
112752407
2157 rs10047036 1 AG GG AG AG AG
GG AA AG AG AG AA AA AA
AA AA AA AA AA AA AA AA
AG AA AG AG AA AA AA AA
AA AA AA AA AA AA AG

54389200							
2158	rs10047085	1	AA	AA	AA	AA	AA
	AG	AA	AG	AG	AG	AG	AG	AA
	AA	AA	AA	AA	AA	AA	AA	AA
	AA	AA	AG	AA	AG	AG	AG	AG
	AA	AA	AA	AA	AG	GG	AG	
31902418							
2159	rs10047101	1	GG	GG	GG	GG	GG
	AG	GG	AG	AG	GG	GG	GG	GG
	GG	GG	GG	GG	GG	GG	GG	GG
	GG	GG	GG	GG	GG	GG	GG	GG
	GG	AG	GG	AG	GG	GG	GG	
65607538							
2160	rs10047146	1	AA	AG	AG	AA	AA
	AA	AA	AA	AA	AA	AA	AA	AA
	AA	AA	AA	AA	AA	AA	AG	AA
	AA	AA	AA	AA	AA	AA	AA	AA
	AA	AA	AA	AA	AA	AA	AG	
177103690							
2161	rs10047182	1	AG	AG	GG	GG	AG
	AG	GG	GG	GG	GG	GG	GG	GG
	AG	GG	GG	AG	AG	GG	GG	GG
	GG	GG	GG	AG	GG	GG	GG	GG
	GG	GG	GG	GG	AG	GG	AG	
4534321							
2162	rs1004720	5	AA	AA	AA	AA	AA
	AA	AA	AA	AA	AA	AA	AA	AA
	AA	AA	AA	AA	AA	AA	AA	AA
	AA	AA	AA	AA	AG	AA	AA	AG
	AA	AA	AA	AA	AA	AA	AA	
44529971							
2163	rs10047345	10	AA	AA	AA	AA	AA
	AA	AA	AA	AA	AA	AC	AA	AC
	AA	AA	AA	AA	AA	AA	AA	AA
	AA	AA	AA	AC	AA	AA	AA	AA
	AC	AA	AA	AA	AA	AA	AA	
45537344							
2164	rs1004751	18	GG	GG	AG	GG	GG
	GG	GG	GG	GG	GG	GG	GG	GG
	GG	GG	AG	GG	AG	GG	GG	GG
	GG	GG	GG	GG	GG	AG	GG	GG
	GG	AA	GG	AG	GG	GG	GG	
46103577							
2165	rs10047526	12	CC	CC	CC	CC	CC
	CC	CC	AC	CC	CC	CC	CC	CC
	CC	CC	AC	AA	AA	CC	CC	CC
	AC	AC	AC	AC	CC	CC	CC	CC
	CC	CC	AC	CC	CC	CC	CC	
11481057							
2166	rs10047529	12	GG	GG	GG	GG	GG

AG GG GG GG GG GG GG GG
GG GG GG GG GG GG GG GG
GG GG GG GG GG GG GG GG
GG GG GG GG GG GG GG
86978591
2167 rs10047581 12 GG GG GG AA AG
AA AG GG AG AG GG AG AG
AG AG GG GG GG AG GG AG
GG GG AG AG GG GG GG GG
GG AG AG AG GG GG GG
32719928
2168 rs1004766 1 GG GG GG AA AA
AA AA AG AG AG AA AA AA
AA AA AG AA AA AG GG AG
AG AG AA AG AA GG AG AG
GG AG AA AG GG AG GG
37824508
2169 rs10047718 13 AG GG AG GG GG
GG GG AG AG AG AG AG AA
AA AA AA AG AG GG AG GG
AG GG GG GG AG AG AA AG
AA AG AG GG AA AG AA
22274442
2170 rs1004772 1 GG GG GG GG GG
GG GG GG GG GG GG GG GG
GG AG AG GG AG AG GG AG
AG GG AG AG GG AG GG GG
GG AG GG AG GG GG AG
60210649
2171 rs10047826 14 GG GG GG GG GG
GG GG GG GG GG GG GG GG
GG GG GG GG GG GG GG GG
GG GG GG GG GG GG GG GG
GG GG GG GG GG GG GG
59139674
2172 rs1004784 5 AG AG AG AA AG
AG AA AG AA AG AA AA AA
AA AA AA AG AG GG AG AG
AG AA AA AA AA AG AG AA
AG AA AG AA AG AA AG
7548178
2173 rs10047842 14 GG GG GG GG GG
GG GG GG GG GG GG GG GG
GG GG GG GG GG GG GG GG
GG GG GG GG GG GG GG GG
GG GG GG GG GG GG GG
103511733
2174 rs10047844 14 GG GG GG GG GG
GG GG GG GG GG GG GG GG
AG AA GG AG AG GG GG GG

GG GG GG GG GG GG GG GG
GG AG AG AG GG GG GG
87989382
2175 rs1004787 2 AG AG AG AA AA
AA AA AA AA AG AA AA AA
GG GG AA AA AA AA AA AG
AG AA AA AG AA AA AA AA
AG AA GG AG AA AA AA
45159091
2176 rs10047931 14 AG AG AG GG GG
GG GG GG GG GG AA AG AG
AG GG GG GG GG GG GG GG
GG GG GG GG AG AG GG AA
GG GG AG AG GG GG GG
44681146
2177 rs10047935 14 GG AG GG AG AG
AA GG AG AG AG AA AA AA
GG AA AG AG AG GG GG GG
AG GG GG AG GG AA AG AG
GG AG AA AG AG AG GG
22787541
2178 rs10047936 14 AG AG GG AA AA
AA AA AA AG AA AA AA AA
AA AA AA AA AA AG AA AG
AA AA AA AA AA AA AA AA
GG AA AA AA AA AA AA
33781858
2179 rs10047960
14 -- -- -- -- -- -- -- --
-- -- -- -- -- -- -- --
-- -- -- -- -- -- -- --
-- -- -- -- -- -- -- --
-- -- -- -- 49384358
2180 rs10047991 15 AG AA AG AG AA
AG AA AA AA AA AA AA AG
AG AA AA AA AA AG AG AA
AA AA AA AA AA AA AA AA
AA AA AA AA AA AA AA
97721147
2181 rs10048003 15 AC AC CC AA AC
AC AA AA AA AA AA AA AA
AA AA AC AC AA AA AA AA
AC AA AA AA AC AC AC AA
CC AA AC AA AA AA AC
25704192
2182 rs10048047 15 GG GG GG GG GG
GG GG GG GG GG GG GG GG
GG GG GG GG GG GG AG GG
GG GG GG GG GG GG GG GG
GG GG AG GG GG GG AG

57751690
2183 rs10048050 15 AG GG AG GG GG
GG AG GG GG GG GG GG GG
GG GG AA AG AG AG AG AG
AG GG GG AG GG AG AG GG
AG AG AA AG AG AG AA
97004933
2184 rs10048092 16 GG AG GG GG GG
GG GG GG GG AG AG AG GG
GG AG GG GG GG GG GG GG
GG AG GG AG AG GG GG AG
AG AG GG AG AG AG GG
48440630
2185 rs1004811 22 GG AG AA AG GG
AG AG AG AG AG GG GG GG
AG GG AG AG AG GG GG GG
AG AG GG AG GG AG GG AG
AG AG AG GG GG GG GG
33809429
2186 rs10048146 16 AA AA AA AA AA
AG AA AG AA GG AA AA AA
AG AG AA AA AG AA AA AG
AA AA AA AA AA AA AA AA
AG AA AA AA AA AG AG
86710660
2187 rs10048187 17 GG GG GG GG GG
GG GG AG AG GG GG GG GG
GG GG AG AG AA GG GG GG
GG GG GG GG AG GG AG GG
GG AG GG AG GG GG GG
74839272
2188 rs10048192 17 GG AG AG GG GG
GG GG GG GG GG GG GG GG
AG AG GG GG GG GG GG GG
GG GG AG AG GG GG GG GG
GG GG GG GG GG GG GG
8858837
2189 rs10048330 18 AA AA AA AA AA
AA AA AA AA AA AA AA AA
AA AA AG AG AG AA AA AA
AG AG AA AG AG AG GG GG
AA AA AG AA AA AA AA
54576032
2190 rs10048366 18 GG GG GG GG GG
GG GG GG GG GG GG GG GG
GG GG GG GG GG GG GG GG
GG GG GG GG GG GG GG GG
GG GG GG GG GG GG GG
5788294
2191 rs10048368 18 CC CC CC CC CC

	CC	CC	CC	CC	CC	CC	CC	CC
	CC	CC	CC	CC	CC	CC	CC	CC
	CC	CC	CC	CC	CC	CC	CC	CC
	AC	CC	CC	CC	AC	CC	AC	
4253242								
2192	rs10048371		18	AA	AG	AG	AG	AG
	GG	AA	GG	AG	AG	AG	AG	GG
	AG	AA	GG	AG	GG	AA	AG	AG
	GG	GG	AG	GG	AG	AG	GG	AG
	GG	GG	AG	GG	AG	GG	AA	
69260566								
2193	rs1004843		20	AA	AA	AA	AA	AA
	AA	AA	AA	AA	AA	AA	AA	AA
	AA	AA	AA	AA	AA	AA	AA	AA
	AA	AA	AA	AA	AA	AA	AA	AA
	AA	AA	AA	AA	AA	AA	AA	
45312611								
2194	rs10048447		19	AA	AA	AA	AA	AA
	AA	AA	AA	AA	AA	AA	AA	AA
	AA	AA	AA	AA	AA	AA	AA	AA
	AA	AA	AA	AA	AA	AA	AA	AA
	AA	AA	AA	AA	AA	AA	AA	
12870791								
2195	rs1004845		10	GG	AG	GG	AG	AG
	AG	AG	AA	AG	AG	AA	AA	AA
	AA	AA	AG	AG	AG	AG	AG	AA
	AA	AA	AA	AG	AA	AG	AG	AA
	AG	AA	AA	AA	AA	AA	AA	
25932400								
2196	rs1004856		11	AG	AG	AA	AG	AG
	AG	AG	AA	AA	AA	AA	AA	AA
	AA	AA	AG	AA	AG	AG	AA	AA
	AG	AA	AG	AA	AG	AG	AG	AG
	AA	AG	AA	AG	GG	AG	AG	
75180967								
2197	rs1004867		16	AG	GG	AG	AG	AG
	GG	AG	GG	GG	AG	GG	GG	GG
	AG	AG	AG	AG	AG	GG	GG	GG
	AG	AG	AG	GG	GG	GG	GG	GG
	AG	GG	AG	GG	AG	GG	AG	
49635132								
2198	rs10048735		2	AG	AG	AG	AG	GG
	AG	GG	AG	GG	GG	AG	AG	AA
	AA	AA	AG	AG	AG	GG	AG	GG
	AG	AG	AG	AG	AG	AG	AA	AG
	AA	AG	GG	AG	AG	AG	GG	
198742525								
2199	rs10048799		2	AG	AG	AG	AG	GG
	AG	AG	GG	AG	AG	AA	AA	AG
	GG	GG	AG	AG	AG	GG	AG	AG

	AG	GG	AG	GG	AG	AG	AG	AA
	AG	AG	AA	AG	AG	GG	AA	
181785294								
2200	rs10048814		2	AG	AA	AA	AA	AA
	AA	AA	AA	AA	AG	AA	AG	AA
	AA	AA	AA	AA	AA	AG	AG	GG
	AA	AA	AA	AA	AG	AA	AG	AG
	AG	AA	AA	AA	AA	AA	AA	
238897755								
2201	rs1004891		5	CC	CC	CC	CC	CC
	CC	CC	AC	AC	AC	CC	CC	CC
	AC	AC	AC	AA	AA	AC	CC	AC
	CC	CC	CC	CC	AC	CC	AC	CC
	CC	AC	AC	CC	AA	AA	AA	
123462858								
2202	rs10048942		3	--	AG	AG	--	--
	AA	--	AG	--	--	AG	AG	--
	--	--	--	--	--	--	--	AG
	AG	AG	AG	--	AG	AG	--	--
	--	AG	--	--	--	AG	--	
135785491								
2203	rs10048963		3	GG	GG	GG	GG	GG
	GG	GG	GG	AG	AA	GG	GG	GG
	GG	GG	GG	GG	GG	GG	GG	GG
	GG	GG	GG	GG	GG	GG	GG	GG
	AG	GG	GG	GG	AG	GG	AG	
193925173								
2204	rs1004897		2	AG	GG	GG	GG	GG
	AG	GG	GG	GG	GG	AG	AG	AA
	AA	AG	GG	GG	GG	AG	AG	AG
	GG	GG	GG	GG	AA	AG	AG	AG
	GG	AG	GG	AG	GG	GG	GG	
240980394								
2205	rs10049082		3	AG	AG	AG	AA	AA
	AA	AA	AG	AG	AG	AG	GG	AG
	AG	AG	AG	AG	AG	GG	AG	AG
	AG	AA	AG	AG	AG	AA	AA	AG
	AA	AG	GG	AG	AG	AA	AG	
4969387								
2206	rs10049099		3	GG	GG	GG	GG	GG
	AG	GG	GG	GG	GG	GG	GG	GG
	GG	AG	GG	GG	AG	GG	GG	GG
	AG	GG	AG	GG	AG	GG	GG	GG
	GG	GG	GG	GG	AG	AA	GG	
187322482								
2207	rs10049124		3	AA	AG	AA	AG	AG
	AA	AG	AA	AA	AA	AA	AG	AG
	AA	AG	GG	AG	AG	AA	AG	AA
	AA	AA	AA	AG	AG	AG	AG	GG
	AG	AG	AA	AA	AG	GG	AA	

34149458
2208 rs10049133 3 AG AG AA AG AG
AG GG GG GG AG AG AG AG
AA AG AA AG AG GG GG AG
AG AG GG AG AA AG AA AA
AA AG AA AA AG AA GG
122202181
2209 rs10049135 3 AA AA AA AA AA
AA AA AG AG AG AA AG AG
AG AA AG GG GG AA AA AA
AA AG AA AG AA AA AA AA
AA AA AA AA AA AA AA
72648980
2210 rs1004916 X AA AG AG GG GG
GG GG GG GG GG AG AG GG
AG AG GG GG GG AG GG GG
GG GG GG GG GG GG GG GG
AG GG GG GG AA AG AA
149904762
2211 rs10049206 3 GG GG GG AA AG
AG AG AA AG AA AA AA AA
GG AG AG GG AG AA AA AG
GG GG GG GG AA AG AG AA
AG GG AA AG GG GG GG
157233698
2212 rs10049246 3 GG GG AG AG AG
AG GG AG GG GG GG GG AG
AG GG AG AG AG AG AG AG
AG AG GG AG AG AA AG AA
GG AA AG AG AA AG AG
185686741
2213 rs1004930 16 AG AG AA AA AA
AG AA AA AA AA AG GG AG
AG AG AG AG AG AA AA AA
AG AG AG AG AA AG AA AG
AG AG AA AG AA AA AA
54345296
2214 rs10049306 3 AA AA AA AA AA
AA AA AA AA AA AG AG AG
AA AA AA AA AA AG AG AG
AG AA AG AG AA AA AA AA
AA AA AA AA AA AA AA
104179183
2215 rs10049330 3 AG GG AG AA AA
AA AG AA AA AA AG AG GG
AA AG AG AG AG AG AA AG
AA AA AA AA GG AG GG AG
AA AA AA AA AA AA AA
66487542
2216 rs10049382 3 AG AA AG AG AA

AG AG AG AG GG AG AG AG
GG GG GG GG GG AG AA AG
AG AG AG AA AA AA AA AA
AA AG AG AG AG AA GG
32189391
2217 rs10049438 3 CC AC AC AA AA
AC AA AC AC AA AC AC AC
AC AC AA AA AA CC CC AC
AA AA AC AC AA AC AC AC
AC AC AA AC AC AA CC
133294203
2218 rs10049490 3 AA AC AA AA AA
AA AC CC CC AA CC AC AC
AC AC AA AC AC AC AC AC
AC AA AC CC AA AA AA AA
AC AA AC AA CC CC CC
27241776
2219 rs10049492 3 AG GG AG AG GG
AG GG GG AG AG GG GG GG
AG AG AG GG GG GG GG AG
AG AG AG AG AG GG GG AG
AG AG AA AA GG AG AG
53596532
2220 rs10049503 4 GG GG AG GG GG
GG GG AG AG AG GG GG GG
GG GG GG GG GG AG AG GG
GG GG GG GG AG AG AA AG
AG GG GG GG GG GG AG
6616948
2221 rs10049506 4 AA AA AA AG AG
AA GG AA AA AA AA AA AG
AA AA GG AG AG AA AA AA
AA AG AG AG AA AA AA AA
AA AA AA AA AA AG AA
61008776
2222 rs1004952 5 AA AA AA AA AA
AA AA AG AA AG AA AA AA
AA AA AA AG AG GG AA AG
AA AA AG AG AG AA AG AG
AA AG AA AA AG AA AG
2858054
2223 rs1004953 5 GG GG GG AG AG
AG GG AG AG AG GG GG GG
AA AA GG AG AG AG GG AG
GG GG AG AG AG GG AG AG
GG AG GG GG AG GG AG
2857885
2224 rs10049629 4 AA AA AA AG AG
AA AG AA AA AG AA AA AG
AA AA AA AA AA AA AA AA

AA AA AA AA AA AA AA AA
AA AA AA AA AA AG AA
45829664
2225 rs10049659 4 AG AG AG GG GG
GG GG AG AA AG AG AG AG
AG GG GG GG GG GG GG GG
GG GG GG GG AG AG AG AA
GG GG AG GG GG GG GG
9586764
2226 rs10049716 4 GG GG GG GG GG
GG GG GG GG GG GG GG GG
GG GG GG GG GG GG GG GG
GG GG GG GG GG GG GG GG
GG GG GG GG GG GG GG
47810497
2227 rs1004973 22 AG GG AG GG GG
GG GG AG GG GG GG GG GG
GG GG AG AG AG GG GG GG
GG GG GG GG AG AG GG AG
AG AG GG AG AG GG AG
17931566
2228 rs10049756 4 GG GG GG GG GG
GG GG GG GG GG GG GG GG
GG GG GG AG AG GG GG GG
GG GG GG GG GG GG GG GG
GG GG GG GG GG GG GG
28872833
2229 rs10049760 4 AA AA AA AA AA
AA AA AA AA AA AA AA AA
AA AA AA AA AA AA AA AA
AA AA AA AA AA AA AA AA
AA AA AA AA AA AA AA
48686817
2230 rs10049820 4 AG AA AA AA AG
AA AG AA AA AA AA AG AG
AG AA AA AA AA AA AA AA
AG AG AG AG AG AA AA AA
AA AG AA AG AA AA AA
32313387
2231 rs10049824 4 GG AG AG AA AA
AA AG AA AA AA GG GG AG
AG AG AA AA AA AG AG AG
AG AG AG AG AA AA AA AA
AA AA AG AA AA AA AA
137986727
2232 rs1004984 15 GG GG GG AA AA
AG AA GG GG GG AG AG AA
AG AA GG GG GG AA GG AG
AG AG AA AG GG AG GG GG
GG AG AG GG AG AG AG

51613529
2233 rs10049915 4 AG GG AG AG GG
AG GG GG GG GG GG GG AG
AG AG AG AG AG AG AG AG
GG GG GG AG AA GG AG AG
GG AG AG AG AG GG AG
178891411
2234 rs10049923 4 AA AA AA AA AA
AA AA AA AA AA AA AA AA
AC AC AA AC AC AA AA AA
AA AA AA AA AA AA AA AA
AA AA AA AA AA AA AA
53779226
2235 rs10049987 4 AG AG AG GG GG
GG GG AA AA AG GG GG GG
GG GG AG AG GG AG AG GG
GG GG GG GG GG GG GG GG
GG AG GG AG GG GG GG
110132858
2236 rs10050018 4 GG GG GG GG AG
GG AG GG GG GG GG GG AG
GG AG GG GG GG GG GG AG
AG AG AG GG AG GG GG GG
AG AG AG AG AA AA AG
183349618
2237 rs10050025 4 AG AG GG GG AG
AG GG AG GG AG GG GG AG
GG AG AG AG AG AG AG AG
AA AG AG AG AG AA AA AA
GG AG AA AA GG AG AG
180663940
2238 rs10050081 4 AA AA AA AA AA
AG AA AA AA AA AA AA AA
AA AA AA AA AA AA AA AA
AA AA AA AA AA AA AA AA
AA AA AA AA AA AA AA
1840843
2239 rs10050099 4 CC CC CC CC AC
CC AC AC AC AC AC AA AC
AC AC CC AC AC AC AC AC
CC CC CC CC AC CC AC AC
AC AC AC AA CC AC CC
160124407
2240 rs10050107 4 AA AA AG AG AG
AA AG AG AG AA AG AA AA
AA AA AG AG AA AA AG AG
AA AA AA AA AA AA AA AA
AA AA AA AA AG AG AA
112245090
2241 rs10050122 4 AA AA AG AG AG

	AG	AA	AA	AA	AA	AG	AG	AG
	AG	AA	AA	AA	AA	AA	AA	AA
	AG	GG	GG	GG	AA	AA	AA	AA
	AA	AG	AA	AG	AA	AA	AG	
161848243								
2242	rs1005017		5	AG	AA	AG	AG	AG
	AG	AG	AG	AA	AG	AA	AA	AA
	AA	AA	GG	GG	GG	AG	AA	AG
	GG	GG	GG	GG	AA	AG	AG	AA
	AG	AA	AG	AG	AG	GG	AA	
38481335								
2243	rs10050226		4	AA	AA	AA	AA	AG
	AG	AA	AA	AA	AA	AA	AA	AA
	AA	AA	AA	AA	AA	AG	AG	AG
	AA	AA	AA	AA	AA	AA	AA	AA
	AA	AG	AA	AA	AA	AA	AA	
55392598								
2244	rs1005032		7	AA	AA	AA	AA	AA
	AA	AA	AG	AG	AA	AA	AA	AA
	AA	AA	AA	AA	AA	AA	AA	AA
	AA	AA	AA	AA	AA	AA	AA	AA
	AG	AA	AG	AA	AA	AA	AG	
51876412								
2245	rs1005038		2	GG	GG	GG	AG	GG
	AG	GG	AA	AA	AA	GG	GG	GG
	AG	AG	AG	GG	AG	AA	AA	AA
	AG	AG	AG	AG	GG	AG	GG	AG
	AA	GG	AG	GG	GG	GG	GG	
60105682								
2246	rs10050438		5	AA	AG	AG	GG	AG
	AG	AG	AA	AA	AA	AA	AA	AA
	AG	AG	AA	AG	AG	AG	AG	AG
	AG	AG	AA	AG	GG	AG	AG	GG
	AA	AA	AA	AA	AA	AA	AA	
10139143								
2247	rs1005045		21	GG	GG	GG	GG	GG
	GG	GG	AG	GG	AG	GG	GG	GG
	GG	GG	GG	GG	GG	GG	GG	GG
	GG	AG	GG	GG	GG	GG	GG	GG
	GG	GG	GG	GG	GG	GG	GG	
45582545								
2248	rs1005051		5	CC	CC	AC	AA	AA
	AC	AC	CC	CC	CC	CC	CC	CC
	CC	CC	CC	CC	CC	AC	AA	CC
	AC	CC	AC	AC	CC	CC	CC	CC
	CC	AC	CC	CC	AA	AA	AC	
62037885								
2249	rs1005056		5	AA	AA	AA	AA	AA
	AA	AA	AA	AA	AA	AA	AA	AA
	AA	AA	AG	AG	AA	AA	AA	AA

AA AA AA AA AA AA AA AA
AG AA AA AA AA AA AA
114580742
2250 rs10050588 5 AG AG AG AA AA
AA AA AA AA AA AA AA AA
AA AA AA AA AA AA AA AA
AA AA AA AA AG AA AA AG
AA AA AA AA AA AA AA
160733095
2251 rs10050619 5 GG GG GG AG GG
AG GG AG GG AG AG GG GG
AA AG AA AG AG GG GG GG
GG GG GG GG AG GG GG GG
GG AG AG AG GG GG AG
28020311
2252 rs10050689 5 AG GG GG AG AA
AG AG AG AG AG GG GG AG
AG AG AG GG AG GG GG GG
GG GG GG GG GG AG GG AG
AA AA AA AA GG AG GG
108029900
2253 rs10050713
5 -- -- -- -- -- -- -- --
-- -- -- -- -- -- -- --
-- -- -- -- -- -- -- --
-- -- -- -- -- -- -- --
-- -- -- -- 133135029
2254 rs10050725 5 AG AA AG GG GG
GG GG AA AA AA GG GG GG
AG AG GG GG GG AA GG AA
AG AG AG AG AG AA AG AG
GG GG AA AG AG GG AG
113779421
2255 rs1005075 15 GG AG AG AG AG
AA AG AA AG GG AG GG AG
GG AG AA AA AA AG AG AG
AG AG AG AA AG AG AG AG
AA AG AG AA AG GG AG
93921835
2256 rs10050775 5 AA AA AA AA AA
AA AA GG AG GG AA AA AA
AG AA AG AG AA AA AG AG
AA AA AA AA AA AG AG AA
AG AA AA AA AA AA AA
38008042
2257 rs10050874 5 GG GG GG GG GG
GG GG AG AG AG GG GG GG
GG GG GG GG GG GG AG GG
GG GG GG GG AG GG GG AG
GG AG GG GG GG GG AG

176668089								
2258	rs10050886		5	GG	GG	GG	AG	AG
	AG	AG	GG	GG	GG	GG	GG	AG
	AG	AG	GG	GG	GG	GG	GG	GG
	GG	AG	GG	GG	GG	AG	GG	GG
	GG	GG	GG	GG	GG	GG	GG	
171575460								
2259	rs10050964		5	GG	GG	GG	GG	GG
	GG	GG	GG	GG	GG	GG	AA	AG
	AG	AG	GG	GG	GG	AG	GG	AG
	GG	GG	GG	GG	AG	AG	AG	AA
	AG	GG	AG	AG	GG	GG	GG	
101067024								
2260	rs10050984		5	AC	AC	CC	CC	CC
	CC	CC	AC	AC	AC	CC	CC	CC
	CC	AC	CC	CC	CC	AC	AC	AA
	CC	CC	CC	CC	CC	AA	AC	AC
	AC	AC	AC	AC	CC	AC	AC	
54241619								
2261	rs1005099		3	AC	AC	AC	AA	AC
	AA	AC	AA	AC	AC	AC	AC	AA
	AC	AC	AC	AA	AA	AC	AC	AA
	AC	AC	AA	AC	AA	AA	AA	AA
	AA	AA	CC	AC	CC	AC	AC	
187440506								
2262	rs10051039		5	AA	AA	AA	AA	AA
	AA	AA	AA	AA	AA	AA	AA	AA
	AA	AA	AA	AA	AA	AA	AA	AA
	AA	AA	AA	AA	AC	AA	AC	AC
	AA	AA	AA	AA	AA	AA	AA	
19013075								
2263	rs10051090		5	GG	GG	AG	GG	GG
	GG	GG	AG	GG	AG	GG	AG	GG
	GG	GG	AG	AG	GG	GG	GG	GG
	GG	GG	GG	GG	GG	GG	GG	GG
	AG	GG	AG	AG	GG	GG	GG	
114527392								
2264	rs10051091		5	AG	AG	AG	GG	GG
	GG	AG	GG	AG	GG	AA	AG	AA
	AG	AA	GG	GG	GG	GG	GG	GG
	GG	GG	GG	GG	GG	GG	GG	GG
	AG	AG	AA	AG	AG	AG	AG	
29028901								
2265	rs10051161		5	GG	GG	GG	AG	AG
	GG	AA	AG	GG	AG	AG	AG	AG
	GG	GG	AG	GG	AG	AA	AA	AA
	AG	AG	AA	AG	AG	GG	AG	AG
	GG	AG	AG	AG	AG	AG	GG	
8830876								
2266	rs10051184		5	AA	AA	AA	GG	GG

	AG	GG	AG	AG	AG	AG	AA	AA
	AA	AA	GG	GG	GG	AG	GG	AG
	AG	AG	AG	AG	GG	AG	GG	AG
	AA	AA	AA	AA	GG	AG	GG	
126372809								
2267	rs10051236		5	AG	GG	AG	AG	AG
	AG	AG	AG	AG	AG	AG	AG	AG
	AG	AG	AG	AA	AA	AG	AA	AA
	AG	AG	AG	AG	AA	AA	AA	AA
	AG	AG	AG	AG	AG	AG	AA	
85137318								
2268	rs10051270		5	CC	AC	AC	CC	CC
	CC	CC	CC	CC	CC	CC	CC	CC
	CC	CC	CC	CC	CC	CC	CC	CC
	AC	CC	CC	AC	CC	CC	CC	CC
	CC	AC	CC	AC	CC	CC	CC	
58070983								
2269	rs10051286		5	GG	GG	AG	AG	AG
	AG	AG	GG	GG	GG	AG	AG	GG
	AG	AG	GG	AG	AG	AG	GG	GG
	AA	AG	AA	AA	GG	GG	GG	GG
	GG	AG	GG	GG	AG	GG	AA	
83069019								
2270	rs10051309		5	AA	AA	AA	AA	AA
	AA	AA	AA	AA	AA	AA	AA	AA
	AA	AA	AA	AA	AA	AA	AA	AA
	AA	AA	AA	AA	AA	AA	AA	AA
	AA	AA	AA	AA	AA	AA	AA	
55543058								
2271	rs10051456		5	AA	AG	GG	GG	GG
	GG	GG	GG	GG	GG	AG	GG	AG
	AG	AG	GG	GG	GG	GG	GG	GG
	GG	AG	AG	GG	GG	GG	GG	GG
	GG	AG	GG	AG	AG	GG	AG	
58502533								
2272	rs10051599		5	AA	AG	AG	AA	AA
	AA	AG	AG	AG	AG	AA	AA	AA
	AG	AG	AA	AA	AA	AA	AG	AA
	AG	AA	AG	AG	AA	AA	AA	AA
	AG	AA	AA	AA	AA	AA	AA	
173275241								
2273	rs10051630		5	AG	AA	AG	AA	AA
	AA	AG	AA	AG	AG	AA	AA	AA
	AA	AA	AA	AA	AA	AG	AA	AG
	AA	AA	AA	AA	AA	AA	AA	AA
	AA	AG	AA	AA	AA	AG	AA	
85611175								
2274	rs10051634		5	AG	AG	AA	AG	AG
	AA	GG	AA	AA	AA	AG	AA	AG
	AG	AG	AG	GG	AG	AA	AG	AA

AG AA AA AG AA AA AA AA
AG AA AG AA GG GG AG
111515382
2275 rs1005165 19 AG AG AG GG GG
GG GG AG GG GG GG GG GG
AG AG GG GG GG GG GG AG
GG AG AG GG AG AG AA AG
AG AG AA AA GG GG GG
45909050
2276 rs10051667 5 AG AA AG AG AA
AA AG AA AA AA AG AG AG
AG AA AA AA AA AG GG AG
AG AG AG AG AA AA AA AA
AA AA GG AG AA AA AA
160898328
2277 rs10051690
5 -- -- -- -- -- -- -- --
-- -- -- -- -- -- -- --
-- -- -- -- -- -- -- --
-- -- -- -- -- -- -- --
-- -- -- -- 84361544
2278 rs10051756 5 AG AG AA GG GG
GG AG AA AA AA AA AA AA
AG AG AA AA AG AG GG AG
AA AA AA AA AG AA AA AG
AG AG AG AA AA AA AA
175878007
2279 rs10051783 5 GG AG GG GG GG
GG GG GG GG GG AG AG GG
AG GG GG GG GG GG GG GG
GG GG GG GG GG GG GG GG
GG AG AG AG GG GG GG
167822652
2280 rs10051830 5 AG AG AG AG AG
AG AA AG AG AG AG AG AA
AA AA AA AA AA GG AA AG
AG AG AG AG AA GG AG AG
AG GG GG GG AG AG AG
110452845
2281 rs10051837 5 AG AA AG AA AA
AA AA AA AA AA AA AA AA
AA AA AA AA AA AA AA AA
AA AA AA AA AA AA AA AA
AA AA AG AA AA AA AA
124327203
2282 rs10051847 5 GG AG AG GG GG
GG GG GG GG GG GG GG GG
GG GG GG AG AG GG GG AG
GG GG GG GG GG GG GG GG
GG AG GG AG GG GG GG

58271696							
2283	rs10051928	5	AG	AG	AA	AA	AG
AG	AA	AG	GG	AG	GG	GG	GG
AG	AG	GG	AG	AG	AG	AA	AG
AA	AA	AA	AA	AG	AA	AA	AA
AA	AG	AG	AA	AA	AA	AA	
6036454							
2284	rs10051930	5	AC	CC	AC	CC	CC
CC	CC	CC	CC	CC	CC	CC	CC
CC	CC	CC	CC	CC	CC	CC	CC
AC	AC	AC	AC	CC	CC	CC	CC
AC	AC	CC	AC	CC	CC	CC	
16741099							
2285	rs10051946	5	GG	GG	GG	GG	GG
AG	GG	GG	GG	GG	GG	GG	AG
GG	GG	AG	GG	GG	GG	GG	GG
GG	GG	GG	GG	GG	GG	GG	GG
GG	GG	GG	GG	GG	GG	GG	
119574410							
2286	rs10051949	5	AA	AA	AA	AA	AA
AA	AA	AA	AA	AA	AA	AA	AA
AA	AA	AA	AA	AA	AA	AA	AA
AA	AA	AA	AA	AA	AA	AA	AA
AA	AA	AA	AA	AA	AA	AA	
123065474							
2287	rs10051970	5	AG	AA	AG	AA	AA
AA	AA	AA	AA	AA	AA	AA	AA
AA	AA	AA	AA	AA	AA	AA	AA
AA	AA	AA	AA	AA	AA	AA	AA
AG	AA	AG	AA	AA	AA	AA	
16732052							
2288	rs10051975	5	AG	AG	AA	AA	AA
AA	AG	AG	GG	GG	AG	GG	AG
AG	AG	AA	AG	AG	AA	AA	AG
AG	AA	AA	AG	AG	AG	AA	AG
AG	AG	AA	AA	AA	AA	AA	
91130959							
2289	rs10051982	5	GG	GG	AG	GG	GG
GG	GG	GG	GG	GG	AA	AG	AA
AG	AG	AG	AG	AG	GG	GG	GG
GG	GG	GG	GG	AG	GG	GG	AG
GG	GG	GG	GG	AG	AG	GG	
75441876							
2290	rs10051998	5	AA	AA	AA	AG	AG
AG	AA	AG	AA	AA	AA	AA	AA
AA	AA	AA	AG	AG	AG	AA	AG
AA	AG	AG	AA	AA	AA	AA	AA
AG	AA	AA	AA	GG	AG	GG	
84018132							
2291	rs10052032	5	AG	AA	AG	AG	AG

GG AG AG GG AG AA AG AA
AA AA AA AG AG AA AG AG
GG GG GG GG AA GG AG AG
AG AA AG AG AG GG AG
25532432
2292 rs10052034 5 GG GG GG GG GG
GG GG GG GG GG GG GG GG
GG GG GG GG GG GG GG GG
GG GG GG GG GG GG GG GG
GG GG GG GG GG GG GG
74342313
2293 rs10052064 5 AG GG AG AA AG
AA AG AG GG GG AG AG AG
AA AA GG AG AG AG AG AG
AG AG AG AG AA AA AA AA
AG GG AG GG AG AA AG
133051079
2294 rs1005209 22 AA AA AA AA AA
AA AA AA AA AA AA AA AA
AA AA AA AA AA AA AA AG
AG AG AG AG AA AG AA AG
AG AA AA AA AG AG AA
17537530
2295 rs10052105 5 AA AG AA GG GG
GG GG AG AG AG AA AA AG
AG AG AA AA AA AA AA AA
GG AG AG AG AG AA AA AG
AA AG AA AG AA AA AA
123035580
2296 rs10052150 5 AC AA AA AC AA
AC AA AA AA AA AA AA AA
AA AA AC CC AC CC AC AC
AA AA AA AA AC AC AC CC
AC AA AC AC CC AC AC
10051405
2297 rs10052174 5 AA AA AG AA AA
AA AA AG AA AG AA AA AA
AA AA AA AA AA AA AA AA
AA AA AA AA AA AA AA AA
AA AG AA AG AA AA AA
31483596
2298 rs10052184 5 GG GG GG AG AG
AA GG AG AG AG AG AG AG
AG AG GG AG GG AG AG AG
AA AA AA AA AG AG AA AG
AG AG AA AG AG AG AA
175298874
2299 rs10052192 5 AA AG AG AG AG
AA GG AA AA AA AA AA AA
AA AA AA AA AA AA GG AA

AA AA AA AA AA AA AA AA
AA AG AA AG AA AG AA
119410495
2300 rs1005221 12 AA AA AA AA AA
AA AA CC AC AC AC AA AC
AA AC AA AA AA AC AA CC
AA AA AA AA AA AC AA AA
AC AC AA AA AA AC AA
5493117
2301 rs10052226 5 AG GG GG AG GG
AG AG GG GG GG GG GG GG
GG GG GG GG GG GG GG GG
GG GG GG GG GG GG GG GG
AG GG GG GG AG GG AG
41955746
2302 rs10052236 5 AA AA AA AA AA
AC AA AA AA AA AA AA AA
AA AA AA AA AA AC AA AA
AA AA AA AA AA AA AA AA
AC AA AA AA AA AA AA
121676688
2303 rs10052248 5 AG GG GG GG GG
AG GG AG AG AG AA AG AA
AA AA GG GG GG GG AG AG
GG AG AG GG AG GG AG AG
AG AG GG AG GG GG GG
86243827
2304 rs1005229 7 AG AG AG AA AA
AG AG AA AG AA AG AA AG
AG AG AA AG AG AG AG AG
AG GG AG AG AA AG AG AG
AA AG AA AG GG AG AG
156002990
2305 rs10052297 5 GG GG GG AG AG
AG GG AG AG AG GG GG GG
GG GG AG AG AG GG AA GG
GG GG GG GG GG AG GG AG
GG GG AG GG GG AG GG
76368663
2306 rs10052337 5 GG GG GG GG GG
GG GG GG GG GG GG GG GG
GG GG GG GG GG GG GG GG
GG GG GG GG GG GG GG GG
GG GG GG GG GG GG GG
21849692
2307 rs10052344 5 GG AG AG GG GG
GG GG GG GG GG GG GG GG
GG GG GG GG GG GG GG GG
AG GG AG AG GG GG GG GG
GG GG GG GG GG GG GG

173297883
2308 rs10052351 5 AA AA AA AA AA
AA AA AA AA AA AG AG AG
AG AA AA AA AA AG GG AG
AG AG AG AG AA AA AA AA
AA AA AG AA AA AA AA
160757462
2309 rs10052385 5 AG AG AG GG GG
GG AG GG GG GG GG GG GG
AG GG AG AG AA AG AA AA
AG AG AG AG AA AA AA AA
AA AG AG AG AG AG AG
26265398
2310 rs10052395 5 GG GG GG GG GG
GG GG GG GG GG AG GG GG
GG GG GG GG GG AG AG GG
AG AG AG AG AG AG GG AA
GG GG GG GG GG GG GG
34735350
2311 rs10052407 5 AG GG GG GG GG
GG GG GG GG GG GG GG GG
GG GG GG GG GG GG GG GG
GG GG GG GG GG AG GG AG
GG GG GG GG GG GG GG
50591476
2312 rs10052410 5 AA AA AA AA AG
AG AA AG AA AA AA AA AA
AA AG AG AA AG AG AG GG
AG AG AG AG AA AG AA AA
AG AG AG AG AA AA AG
155714344
2313 rs10052423 5 GG GG GG GG GG
GG GG GG GG GG GG GG GG
GG GG GG GG GG GG GG GG
-- GG GG GG GG GG GG GG
-- -- GG GG GG GG GG
28924164
2314 rs10052435 5 AA AA AA AA AA
AA AA AA AA AA AA AA AA
AG AA AA AA AA AA AA AA
AA AA AA AA AA AA AA AA
AA AA AA AA AG AG AG
20213446
2315 rs10052448 5 GG GG AG AG AG
AG AG AG AG AG AG AG AG
AG AG GG AG AG AG GG AG
AG GG GG AG AG GG AG AG
GG AG GG AG AA AG AA
84025813
2316 rs10052518 5 AG GG GG AG AG

	GG	AA	GG	GG	GG	AG	GG	GG
	AA	AG	AG	AA	AG	AG	AA	AG
	GG	AG	GG	GG	GG	GG	GG	GG
	AG	GG	GG	GG	GG	GG	GG	
67131310								
2317	rs10052526		5	GG	GG	GG	AG	AG
	GG	AG	GG	GG	GG	GG	GG	GG
	AG	GG	AG	GG	AG	GG	GG	GG
	GG	GG	GG	GG	GG	AG	AG	GG
	GG	GG	GG	GG	GG	AG	AG	
22046965								
2318	rs10052529		5	AA	AG	AG	AA	AA
	AG	AA	AA	AA	AA	AA	AA	AG
	AG	AG	AA	AA	AA	AG	AA	AG
	AA	AA	AA	AA	AA	AA	AA	AA
	AA	AG	AA	AG	AA	AA	AG	
104734927								
2319	rs10052561		5	AG	AG	AG	AG	AG
	GG	AA	AG	AA	AA	AA	AA	AA
	AG	AG	AG	AA	AG	AG	GG	GG
	GG	GG	GG	GG	AA	AA	AA	AA
	AA	AG	GG	AG	GG	AG	AG	
157318560								
2320	rs10052602		5	AA	AA	AA	AA	AA
	AA	AA	AA	AA	AC	AA	AA	AA
	AA	AA	AA	AA	AA	AA	AA	AA
	AA	AA	AA	AA	AA	AA	AA	AA
	AA	AA	AA	AA	AA	AA	AA	
54486441								
2321	rs10052657		5	CC	AC	AC	AC	AC
	AC	AC	CC	CC	CC	CC	CC	CC
	CC	CC	CC	CC	CC	CC	CC	CC
	CC	AC	AC	CC	CC	AC	CC	AC
	CC	CC	AC	CC	AC	AC	AA	
58407771								
2322	rs10052667		5	AG	AG	AG	AG	AG
	AA	GG	AG	AG	AG	AG	AA	AA
	GG	AG	GG	AG	AG	GG	GG	AG
	AG	AG	AG	AG	AG	AA	AA	AA
	GG	AA	AG	AA	AG	AA	GG	
30964809								
2323	rs10052679		5	AA	AA	AA	AG	AA
	AA	AG	AA	AA	AA	AA	AA	AA
	AA	AA	AG	AG	AG	AA	AA	AG
	AA	AA	AA	AA	AA	AA	AA	AA
	GG	AA	AA	AA	AA	AG	AA	
122157151								
2324	rs10052685		5	AA	AA	AG	AA	AA
	AA	AG	AA	AA	AA	GG	GG	AG
	AG	AG	AA	AA	AA	AA	AA	AA

AG AA AG AA GG AA AG AG
AG AG AA AG AA AA AA
6377973
2325 rs10052705 5 AA AA AA AA AA
AA AA AA AA AA AG AA AG
AG AG AA AA AA AA AA AA
AA AA AA AA AA AG AG AA
AG AG AG AG AA AG AA
107093148
2326 rs10052731 5 AA AA AA AA AA
AA AA AG AG AG AA AA AA
AA AA AG AG GG AG AG AA
AG AG AG AG AA AA AA AA
AG AA AG AA AA AG AA
135440176
2327 rs10052761 5 AG AG AG GG GG
GG GG GG GG GG AG GG GG
AG AG AG AA AG AG AG AG
GG GG GG GG GG AA AG AG
AG AG AG GG AG GG AA
18095876
2328 rs10052777 5 GG GG GG AG AG
GG AA GG AG AG AG AG AG
AG AA AA AA AA GG AG GG
AA AA AA AA GG GG GG GG
AG GG AG GG AG AG AG
158269081
2329 rs10052782 5 AG GG AG AG AG
AG GG GG GG GG GG GG GG
GG GG AG AG GG GG GG GG
GG GG GG GG GG GG GG GG
GG GG GG GG GG AG AG
179975104
2330 rs10052805 5 AA AA AA AA AA
AA AA AA AA AA AA AA AA
AA AA AA AA AA AA AA AA
AA AA AA AA AA AA AA AA
AA AA AA AA AA AA AA
168045046
2331 rs10052827 5 AG AG AG GG GG
GG GG GG GG GG GG GG GG
GG GG GG GG GG GG AG GG
GG GG GG GG AG AG GG AA
AG GG GG GG AG AG AA
39572571
2332 rs10052843 5 AG GG AG AG AA
AG AA AG AG AG AG AG AG
AA AA AA AG AG GG GG AG
AG AG AG AG AG AA AA AG
AG AA GG AG AG AA GG

148551105
2333 rs1005292 14 CC CC CC AC CC
AC CC CC CC CC CC CC CC
CC CC AC AC AC CC CC CC
CC -- CC AC CC CC CC CC
CC CC AC CC CC CC CC
81501957
2334 rs10052921 5 AA AA AA AA AA
AA AG AG AG AA AG AG GG
AG AG AG AG AG AG AA AG
AA AA AA AA AA AG AA AG
AA AA AA AA AA AG AA
76857870
2335 rs10052925 5 GG AG AG GG GG
GG GG GG GG GG AG AG GG
GG GG AG AG GG GG GG GG
GG GG GG GG GG GG GG GG
GG GG GG GG GG GG GG
174424824
2336 rs1005295 X GG AG AA GG GG
GG GG AA AA GG AA AA AA
AA AA AA AA AA AA AG AA
AA GG AA AA AG GG AG GG
AA AG AA AA AG AG AA
79342388
2337 rs10052972 5 CC AC AA AC AC
CC AC AC AC AC AA AA AC
AC AC CC CC CC CC AC AC
AC AC AC AC CC CC CC CC
CC CC CC CC CC CC AC
9980435
2338 rs10053012 5 GG GG GG AG AG
AG AG GG GG GG GG GG GG
GG GG AA AG AG GG GG GG
GG GG GG GG GG GG GG GG
GG GG GG GG GG GG GG
171522275
2339 rs1005304 20 GG GG GG GG GG
GG GG GG GG GG AG GG AG
AG AG GG GG GG AG AG AA
AG GG GG AG AG GG GG AG
GG GG GG GG GG GG GG
2735182
2340 rs10053056 5 AA AG AG AA AA
AG AG GG GG GG GG GG GG
GG GG GG AG AG GG AG GG
AG AG AG AG AA AG AA AG
AA GG GG GG AG AA GG
96043420
2341 rs10053085 5 AG AG AG AG AA

	AG	AG	GG	GG	GG	AG	AG	AG
	GG	GG	AG	AG	GG	GG	AG	AG
	AG	GG	AG	GG	AG	GG	GG	GG
	GG	AG	GG	GG	GG	GG	AG	
6842007								
2342	rs1005311		7	AA	AA	AG	AG	AA
	AG	AA	AA	AG	AG	AA	AA	AA
	AA	AA	AG	GG	AG	AG	AG	GG
	AA	AG	AA	AG	AA	AA	AA	AA
	AG	AA	AA	AA	GG	GG	AG	
39880053								
2343	rs10053137		5	AC	CC	CC	CC	CC
	CC	AC	AC	AC	AC	CC	CC	CC
	CC	CC	AC	AC	CC	AC	CC	CC
	CC	CC	CC	CC	CC	CC	CC	CC
	CC	AC	CC	CC	CC	CC	AC	
20429332								
2344	rs10053157		5	GG	AG	GG	GG	GG
	GG	GG	GG	GG	GG	GG	GG	GG
	GG	GG	AG	GG	AG	GG	GG	GG
	GG	GG	GG	GG	GG	GG	GG	GG
	GG	AG	GG	AG	GG	GG	GG	
112759591								
2345	rs1005316		17	GG	GG	AG	GG	GG
	GG	GG	AG	AG	AG	AG	AG	AG
	AG	AG	AG	AG	AG	GG	GG	GG
	AG	GG	AG	GG	GG	GG	GG	GG
	GG	GG	GG	GG	AG	AG	GG	
68990369								
2346	rs1005317		X	CC	CC	CC	AC	AA
	AA	AC	CC	CC	CC	CC	CC	CC
	CC	CC	AC	CC	AC	CC	CC	CC
	CC	CC	CC	CC	CC	CC	CC	CC
	CC	CC	CC	CC	CC	CC	CC	
142213332								
2347	rs10053229		5	GG	GG	GG	GG	AG
	GG	AG	AG	AA	AG	AA	AA	AA
	AA	AA	GG	GG	GG	AG	AG	AG
	AG	AA	AA	AG	GG	AG	GG	AG
	AG	AA	GG	AG	GG	GG	GG	
43489177								
2348	rs10053232		5	AG	GG	AG	GG	GG
	GG	GG	GG	GG	GG	GG	GG	GG
	AG	AG	AG	GG	AG	AG	AG	AG
	GG	GG	GG	GG	GG	GG	GG	GG
	GG	GG	AG	GG	GG	GG	GG	
151503347								
2349	rs10053236		5	AA	AG	AG	AG	AA
	AG	AA	AG	AG	AG	GG	AG	AG
	AA	AA	AG	AA	AG	AG	AA	AA

AA	AA	AA	AA	AA	AA	AA	AA
AA	AA	AA	AA	AA	AA	AA
16540912
2350	rs1005325	22	GG	GG	GG	AG	GG
AG	GG	GG	GG	GG	GG	GG	GG
GG	GG	AG	AA	AA	GG	GG	GG
GG	GG	GG	GG	GG	GG	GG	GG
GG	GG	GG	GG	GG	GG	GG
17634711
2351	rs1005331	17	CC	CC	CC	CC	CC
CC	CC	CC	CC	CC	CC	CC	CC
CC	CC	CC	CC	CC	CC	CC	CC
CC	CC	CC	CC	CC	CC	CC	CC
CC	CC	CC	CC	AC	AA	CC
54741504
2352	rs10053474	5	CC	CC	AC	CC	AC
AC	AC	CC	CC	CC	CC	CC	AC
AC	AC	CC	CC	CC	CC	AC	CC
CC	CC	CC	CC	CC	AC	AC	CC
CC	CC	CC	CC	AC	CC	AA
107939906
2353	rs1005348	14	AA	AA	AC	AC	AA
AC	AA	AC	AC	AC	AA	AA	AA
AA	AA	AA	AA	AA	AC	AA	AC
AA	AA	AA	AA	AA	AC	AC	AA
AC	AA	AA	AA	AC	AA	AC
95033275
2354	rs10053485	5	AA	AA	AA	AA	AA
AA	AA	AA	AA	AA	AA	AA	AA
AA	AA	AA	AA	AA	AA	AA	AA
AA	AA	AA	AA	AA	AA	AA	AA
AA	AA	AA	AA	AA	AA	AA
27497914
2355	rs10053522	5	AG	GG	GG	AG	GG
AG	AG	GG	GG	GG	GG	GG	GG
GG	GG	GG	GG	GG	GG	GG	GG
GG	GG	GG	GG	GG	GG	GG	GG
AG	GG	GG	GG	AG	GG	AG
41961622
2356	rs10053527	5	AG	AA	AG	AG	AG
AA	GG	AA	AA	AA	AG	AG	AG
AA	AA	AA	AA	AA	AA	AA	AA
AG	AG	AG	AG	AA	AA	AA	AA
AA	AA	AA	AA	AA	AA	AA
151910392
2357	rs10053616	5	AA	AA	AA	AA	AA
AA	AA	AA	AA	AA	AA	AA	AA
AA	AA	AA	AA	AA	AA	AA	AA
AA	AA	AA	AA	AA	AA	AA	AA
AA	AA	AA	AA	AA	AA	AA

26080284
2358 rs1005362 9 AG GG AG GG GG
GG AG AG AG GG GG GG GG
AG AA GG GG GG AG AA GG
AA AA AA AA AG AA AG AG
AG GG AG AG AG AG AA
130638467
2359 rs10053621 5 AG AG AG AA AA
AA AG AA AG AA GG AG GG
AG GG AA AA AA AA AA AA
AA AA AA AA AA AA AA AA
AG AG GG AG AG AG AG
29116706
2360 rs10053664 5 AG AG AG AA AA
AG AA AA AA AA GG AG GG
AG AG AA AA AA AA AA AA
AA AA AA AA AA AA AA AA
AA AA AA AA GG GG GG
40785811
2361 rs1005368 22 AA AA AA AG AG
AA GG AA AA AA AA AA AA
AA AG AA AA AA AA AA AA
AA AA AA AA AG AA AG AG
AA AG AA AG AA AA AA
45383230
2362 rs1005369 17 CC CC CC CC CC
CC CC CC CC CC AC AC AA
AC AA CC CC CC CC CC CC
AC CC AC AC CC AC CC AC
AC CC CC CC CC CC CC
16509917
2363 rs10053713 5 AG GG AG GG GG
GG GG GG GG GG AG AG AG
GG GG GG GG GG GG GG GG
GG GG GG GG GG GG GG GG
GG AA AG AG GG GG GG
172437641
2364 rs10053766 5 AA AA AA AG AG
AG AG AA AA AA AA AA AA
AA AG AA AA AA AA AA AA
AA AA AA AA AA AA AA AA
AA AA AA AA AA AA AA
35471886
2365 rs10053840 5 AG AG AA AA AA
AG AA AA AG AA AG GG AG
AG AA GG GG GG AA AA AG
AA AA AA AA AG AG AA GG
AA GG GG GG AG AA GG
35732551
2366 rs10053846 5 AG AA AA AA AA

AA AA AA AA AA AA AA AA
AA AA AA AA AA AA AA AA
AA AA AA AA AA AG AA AG
AA AA AA AA AA AA AA
50612579
2367 rs10053893 5 GG GG GG GG GG
GG GG GG GG GG GG GG GG
GG GG GG GG GG GG GG GG
GG GG GG GG GG GG GG GG
GG GG GG GG GG GG GG
50686065
2368 rs10053913 5 AG AA AG AG AA
AG AG AG AG AG GG GG GG
AG AA AA AA AA GG AG AG
AA AA AA AA AA AA AA AA
AA GG AA AG GG AG GG
164999502
2369 rs10053930 5 GG GG GG GG GG
GG GG GG GG GG GG GG GG
GG GG GG GG GG GG GG GG
GG GG GG GG GG GG GG GG
GG GG GG GG GG GG GG
169175917
2370 rs1005394 12 AG AA AG GG GG
AG AG GG AA AA AA GG AG
AG AG AG AG AG AA AA AA
AG AG AG AG AA AG AA AA
AG AG AA AA AA AG AA
1441137
2371 rs10053948 5 CC CC AC AC CC
AC AC AC CC AC AA AA AC
AC AC CC CC CC CC AC CC
CC CC AC CC CC CC CC CC
CC CC AA AC CC CC CC
24128474
2372 rs1005395 17 AG AG AG GG GG
GG GG GG GG GG GG GG GG
AG GG GG GG GG GG AG AG
GG GG GG GG AG GG GG GG
GG GG GG GG GG GG AG
14355233
2373 rs1005401 7 AG AG AG AG AG
AG AG AA AA AA AG AG AA
AG AG AA AA AA AG GG AA
AG AA AG AA GG AA AG AG
AA AG AG GG AG GG AG
26648786
2374 rs1005402 22 AA AA AG AG AA
AG AA GG AG GG AA AA AG
AG AG GG GG GG GG AG GG

AG AG AG GG GG AA AG AG
GG AA GG AG AA AG AA
41291730
2375 rs10054053 5 GG GG GG GG GG
GG GG AG AG AG AG AG AA
AG AG GG GG GG GG GG AG
AG AG AG AG GG GG GG GG
AG GG GG GG GG GG AG
123279262
2376 rs10054105
5 -- -- -- -- -- -- -- --
-- -- -- -- -- -- -- --
-- -- -- -- -- -- -- --
-- -- -- -- -- -- -- --
-- -- -- -- 110909333
2377 rs10054106 5 AG AG AG AG AG
GG AG AG GG AG GG GG GG
GG GG GG GG GG AG GG GG
GG GG GG GG AG GG GG GG
GG GG AG GG GG GG GG
24607337
2378 rs10054135 5 AG GG AG GG GG
GG GG GG GG GG AG AG AG
GG GG AG AG GG GG GG GG
GG GG GG GG GG GG GG GG
GG GG GG GG GG GG GG
169259125
2379 rs10054136 5 AA AA AA AA AA
AA AA AA AA AA AC AC AC
AA AA AA AA AA AC AA AA
AA AA AA AA AA AA AA AA
AA AA AA AA AA AA AA
153278085
2380 rs10054150 5 AC CC CC AC AC
AC AC CC CC CC CC AA AC
AC AC CC CC CC AC AC AA
CC CC CC CC AA CC AC AC
AA AC CC CC AC AC AA
50285398
2381 rs10054252 5 AG AA AG AA AA
AA AG AA AA AA AG GG GG
AA AG AG AG GG GG GG GG
AA AG AG AA AA AA AA AA
AA AA AA AA AG AA AG
56727958
2382 rs10054289 5 AG AA AA AG AG
AG GG GG GG GG AG GG GG
GG GG AA AA AA AG GG AG
AG GG GG GG GG AG AG GG
AA GG GG GG GG GG AG

120760608							
2383	rs10054295	5	AA	AA	AG	AA	AA
	AA	AA	AA	AA	AA	AA	AA	AA
	AA	AA	AA	AA	AA	AA	AA	AA
	AA	AA	AA	AA	AA	AA	AA	AA
	AA	AA	AA	AA	AA	AA	AA	
63035923							
2384	rs10054312	5	AG	AG	GG	AG	AG
	GG	AG	AA	AA	AA	AG	GG	AG
	AG	AG	AG	GG	AG	GG	AA	AG
	AG	AG	AA	AG	AG	GG	AG	AG
	GG	AG	AA	AA	AA	AA	AA	
2674526							
2385	rs10054323	5	GG	GG	GG	AG	AG
	AG	GG	GG	GG	GG	GG	AA	AG
	AG	AG	GG	AG	AG	AG	GG	AA
	AG	AG	AG	AG	AA	GG	AG	AG
	GG	GG	GG	GG	GG	GG	GG	
127078541							
2386	rs10054371	5	AG	AG	AG	GG	GG
	GG	GG	AG	AG	GG	GG	GG	GG
	GG	GG	GG	GG	GG	GG	GG	AG
	AG	AG	AG	AG	AG	AG	AG	AG
	GG	AG	GG	GG	AG	AG	GG	
57046967							
2387	rs10054440	5	GG	GG	GG	GG	GG
	GG	GG	GG	GG	GG	GG	GG	GG
	GG	GG	GG	GG	GG	GG	GG	GG
	GG	GG	GG	GG	GG	GG	GG	GG
	GG	GG	GG	GG	GG	GG	GG	
179308012							
2388	rs1005447	1	GG	AG	AG	AG	AG
	AG	AA	AA	AG	AG	AG	AG	GG
	GG	GG	GG	GG	GG	AA	GG	AG
	GG	GG	GG	GG	AG	AG	AA	AA
	AA	AG	GG	AG	AG	GG	AA	
15450551							
2389	rs10054521	5	CC	CC	AC	AC	AC
	AC	AC	CC	CC	CC	AC	AC	AA
	AC	AC	AC	AA	AA	AC	AC	AA
	CC	CC	CC	CC	AC	CC	AC	CC
	AC	CC	AA	AC	AA	AC	AA	
44576171							
2390	rs1005454	16	AA	AA	AA	AG	AA
	AG	AG	AA	AA	AA	AA	AA	AA
	GG	AG	AA	AA	AA	AA	AG	AA
	AG	AG	AA	AA	GG	AA	AG	AG
	AG	AG	AA	AG	AA	AA	AA	
1246270							
2391	rs10054678	5	GG	GG	GG	AG	AG

	AG	AG	GG	GG	GG	AG	GG	AG
	GG	AG	AG	GG	AG	AG	AA	GG
	GG	GG	GG	GG	GG	GG	GG	GG
	GG	GG	GG	GG	AA	AG	AA	
55978114								
2392	rs10054679		5	CC	AC	AC	CC	CC
	CC	CC	AC	AC	AC	AA	AC	AA
	AA	AA	AC	AC	AC	AC	AA	AC
	AA	AA	AA	AA	CC	CC	CC	CC
	AA	CC	CC	CC	AC	CC	AC	
84533754								
2393	rs10054800		5	GG	GG	GG	AG	AG
	AG	GG	GG	GG	GG	GG	GG	GG
	GG	GG	GG	GG	GG	GG	GG	GG
	GG	GG	GG	GG	GG	GG	GG	GG
	GG	GG	GG	GG	GG	GG	GG	
43733126								
2394	rs10054920		5	AA	AA	AA	AA	AA
	AC	AA	AA	AA	AA	AA	AA	AA
	AA	AA	AA	AA	AA	AC	AA	AC
	AC	AA	AA	AA	CC	AA	AC	AC
	AA	AA	AA	AA	AA	AA	AC	
120183320								
2395	rs10054927		5	GG	GG	GG	AG	AG
	AG	AG	GG	GG	GG	GG	GG	GG
	GG	GG	GG	GG	GG	AG	AG	GG
	GG	GG	GG	GG	GG	AG	AG	GG
	AG	GG	GG	GG	AG	GG	AG	
79600508								
2396	rs10054949		5	AG	AG	AA	AA	AA
	AA	AG	AA	AA	AA	AA	AA	AA
	AA	AA	AA	AA	AA	AA	AA	AA
	AA	AA	AA	AA	AG	AG	AA	AG
	AG	AA	AA	AA	AA	AA	AA	
169217218								
2397	rs1005495		14	AA	AA	AA	AA	AA
	AA	AA	AA	AA	AA	AC	AC	AC
	AA	AC	AA	AA	AA	AA	AA	AC
	AA	AA	AA	AA	AA	AA	AA	AA
	AA	AA	AA	AA	AA	AA	AA	
22641141								
2398	rs10054994		5	GG	GG	GG	GG	GG
	GG	GG	GG	GG	GG	GG	GG	GG
	GG	GG	GG	GG	GG	GG	GG	GG
	GG	GG	GG	GG	GG	GG	GG	GG
	GG	GG	GG	GG	GG	GG	GG	
179785394								
2399	rs10055011		5	GG	GG	GG	GG	GG
	AG	GG	AG	AG	AA	AA	GG	AG
	AG	AG	GG	GG	GG	GG	GG	GG

	GG	AG	GG	GG	GG	GG	GG	GG
	AA	GG	AG	AG	GG	GG	GG	
74798156								
2400	rs10055057		5	GG	GG	GG	GG	GG
	GG	GG	GG	AG	GG	GG	GG	GG
	GG	GG	GG	GG	GG	GG	AG	GG
	GG	GG	GG	GG	AG	GG	AG	AG
	AG	GG	GG	GG	GG	GG	GG	
114383501								
2401	rs1005511		11	GG	AG	AG	GG	AG
	AG	AG	GG	AG	AG	AG	AA	AG
	GG	AG	GG	GG	GG	GG	GG	GG
	AG	AA	AG	AA	AA	GG	AG	AG
	AG	GG	GG	GG	AG	GG	AG	
57366656								
2402	rs1005513		17	AA	AA	AA	AA	AA
	AA	AG	AG	AA	AG	AG	GG	AG
	AG	AG	AG	GG	GG	AG	AG	AA
	AG	AA	AG	AA	AG	AG	AG	AG
	GG	AA	AG	AA	AG	GG	AA	
49196918								
2403	rs10055144		5	GG	GG	GG	GG	AG
	GG	AG	GG	GG	GG	AG	AG	AG
	AA	AA	GG	AG	AG	AG	GG	AA
	GG	AG	AG	AG	GG	AG	GG	AG
	AG	GG	GG	GG	GG	GG	GG	
122630230								
2404	rs1005518		22	AG	AA	AA	AA	AG
	AG	AA	AA	AG	AG	AA	AA	AA
	AA	AG	AA	AA	AA	AG	AG	AA
	GG	AG	AG	GG	AA	AG	AA	AA
	AG	AA	AA	AA	AG	AA	AG	
45085284								
2405	rs1005521		6	AA	AA	AA	CC	AC
	AC	CC	AA	AC	AA	CC	AC	CC
	AC	CC	CC	AC	AC	AC	AC	AA
	AA	AC	AA	AC	AC	AC	AC	AA
	AC	AA	AA	AA	AC	AA	AC	
5704014								
2406	rs10055239		5	GG	GG	GG	AG	AG
	AG	GG	GG	GG	GG	GG	GG	GG
	GG	GG	GG	GG	GG	AG	AG	GG
	AG	AG	AG	AG	GG	GG	GG	GG
	GG	GG	GG	GG	GG	GG	GG	
38068116								
2407	rs1005526		21	AA	AA	AA	AA	AG
	AA	AG	AG	GG	GG	GG	GG	GG
	AG	GG	AG	AG	AG	AG	AG	AA
	AA	AA	AA	AA	GG	AG	GG	GG
	AA	GG	AG	AG	GG	GG	AG	

16466610
2408 rs10055267 5 GG GG GG GG GG
GG GG GG GG GG GG GG GG
AG AG GG GG GG GG GG GG
GG GG GG GG GG GG GG GG
GG GG GG GG GG GG GG
2053188
2409 rs10055279 5 AA AA AA AA AA
AA AA AA AA AA AC AA AA
AA AA AC CC CC AC AC AA
AA AA AA AA AC AA AA AC
AA AC AC CC AA AA AA
38725043
2410 rs10055302 5 GG GG GG GG GG
GG GG GG GG GG GG GG GG
GG GG GG GG GG GG GG GG
GG GG GG GG GG AG GG GG
GG GG GG GG GG GG GG
157565338
2411 rs10055305 5 AG AG AG GG GG
GG GG AG AG AG GG GG GG
GG GG GG GG GG GG GG GG
GG GG GG GG GG AG GG AG
GG GG GG GG GG GG GG
50177886
2412 rs10055309 5 AA AA AA AA AA
AG AA AA AA AA AA AA AG
AA AG AA AA AA AA AA AA
AA AA AA AA AG AA AA AG
AA AA AA AA AG AA AG
168430327
2413 rs1005531 X AA AA AA AA AA
AA AA AA AA AA AA AA AA
AA AA AA AA AA AA AA AA
AA AA AA AA AG AA AG GG
AA AA AA AA AA AA AA
42608897
2414 rs10055339 5 AG GG GG GG GG
AG GG AG AG AG AA AG AA
AG AG AG AG GG AA GG AA
AG GG GG GG AG GG AG AG
AG AG AG AA GG AG AG
114973120
2415 rs1005535 21 AG GG AG GG GG
AG GG AG AG AA AG AG GG
GG GG AG AA AG GG GG GG
AG AG GG GG GG AG GG GG
AA AG AG AG GG GG GG
32180115
2416 rs10055381 5 GG GG GG GG GG

GG GG GG GG GG GG GG GG
GG GG GG GG GG GG GG GG
GG GG GG GG GG GG GG GG
GG GG GG GG GG GG GG
57345868
2417 rs10055390 5 AA AA AA AA AG
AG AG AG AG AG AA AA AA
AA AA AG AG GG AG GG AG
AG AG AG AG AG AG GG AG
AG AG AG AA GG GG AG
24859690
2418 rs10055397 5 AC AC AA AA AA
AA AA AA AA AA AC AA AA
AA AA CC AC AC AA AA AA
AA AC AC AC AC CC CC AC
AC AA AC AC AC AA CC
120922897
2419 rs10055432 5 GG GG GG GG GG
GG AG GG GG GG GG GG GG
GG GG GG GG GG GG AG GG
GG GG GG GG GG AG AG AG
GG GG GG GG GG GG GG
170246869
2420 rs10055437 5 AG AA AA AG AG
AG AG AG GG AG AA GG AG
AG AG AG AA AA AG AG GG
AA AA AA AA GG AG AG GG
GG GG GG GG AG AG GG
50306946
2421 rs10055446 5 GG GG GG GG GG
GG AG GG GG GG GG GG GG
GG GG GG GG GG GG GG GG
GG GG GG GG GG GG GG GG
GG GG GG GG GG GG GG
58534348
2422 rs10055489 5 AG AA AA AG AG
AA GG AA AA AG AA AG AG
AG GG AG AG GG AA AG AA
AA AA AA AA AG GG AG AG
AA AG AG GG AG AA GG
13987466
2423 rs1005552 17 AG AA AG AG AG
AA AG GG AG AG AG AG AA
AA AA AG AG -- -- AA AA
-- -- -- -- -- AA -- --
AA -- -- -- -- AA AG
34949598
2424 rs10055533 5 AG AG AG AG AG
AA GG AA AG AG AG GG GG
AG AG AG AG AG AA AA AA

	AA	AA	AG	AG	AA	AG	AG	AA
	AG	AA	AA	AA	AG	AG	AG	
133223945								
2425	rs10055551		5	GG	GG	GG	GG	GG
	GG	GG	GG	GG	GG	GG	GG	GG
	GG	GG	GG	GG	GG	GG	GG	GG
	GG	AG	AG	GG	GG	GG	GG	GG
	GG	GG	GG	GG	GG	GG	GG	
101215201								
2426	rs10055586		5	AG	AA	AA	AA	AA
	AA	AA	AA	AA	AA	AG	AA	AG
	AG	AG	AA	AA	AA	AA	AA	AG
	AG	AA	AA	AG	AA	AA	AA	AA
	AG	AG	AA	AG	AG	GG	AG	
98488261								
2427	rs10055607		5	AA	AA	AA	AA	AA
	AA	AA	AA	AA	AA	AA	AA	AA
	AA	AA	AA	AA	AA	AG	AA	AA
	AG	AG	AG	AG	AA	AA	AA	AA
	AA	AA	AA	AA	AA	AA	AA	
174058371								
2428	rs10055695		5	CC	CC	CC	CC	CC
	CC	CC	CC	AC	CC	CC	CC	CC
	CC	CC	CC	CC	CC	CC	CC	CC
	CC	CC	CC	CC	CC	CC	CC	CC
	CC	CC	CC	CC	CC	CC	CC	
178246618								
2429	rs10055710		5	AG	GG	GG	AG	AG
	AA	GG	GG	AG	GG	GG	AG	AG
	GG	GG	AG	GG	AG	GG	AG	AG
	AG	AG	AG	AG	AG	AG	AA	AG
	AG	AG	GG	AG	AG	GG	AA	
11607481								
2430	rs10055720		5	GG	GG	GG	GG	GG
	GG	GG	GG	GG	GG	GG	GG	GG
	GG	GG	GG	GG	GG	GG	GG	GG
	GG	GG	GG	GG	GG	GG	GG	GG
	GG	GG	GG	GG	GG	GG	GG	
10660334								
2431	rs10055777		5	AG	GG	AG	AG	AG
	GG	AA	AA	AA	AA	AG	AG	AA
	AG	AG	AG	AA	AG	AG	AG	AG
	AG	AG	GG	GG	AA	AG	AG	AG
	GG	AA	AA	AA	GG	GG	GG	
178329316								
2432	rs1005580		X	GG	AG	AA	GG	GG
	GG	GG	AG	AG	GG	GG	GG	AG
	GG	GG	GG	GG	GG	GG	AG	GG
	GG	AA	GG	AG	AG	GG	AG	GG
	GG	GG	GG	GG	GG	GG	GG	

131942253
2433 rs1005583 8 AA AA AA AA AA
AA AA AA AA AA AA AA AA
AA AA AG AA AG AA AA AA
AA AA AA AA AA AG AA AA
AA AA AA AA AA AA AA
36787153
2434 rs10055832 5 GG GG GG GG GG
GG GG GG GG GG GG GG GG
GG GG GG GG GG GG GG GG
GG GG GG GG GG GG GG GG
GG GG GG GG GG GG GG
45131543
2435 rs1005584 21 GG GG GG GG GG
GG GG GG GG GG GG GG GG
GG GG GG GG GG GG GG GG
GG GG GG GG GG GG GG GG
GG GG GG GG AG AG GG
44477095
2436 rs10055880 5 AA AA AA AG AG
AG AG AA AA AA AA AA AA
AA AA AA AA AA AA AA AA
AA AA AA AA AA AA AA AA
AG AA AA AA AA AA AA
65864189
2437 rs10055883 5 AC AC AA AC AC
AA CC CC CC CC CC CC CC
CC CC AC CC AC CC CC CC
AC CC CC CC CC CC CC CC
AC AC CC CC AC AC CC
6574609
2438 rs10055900 5 AA AA AG AA AA
AA AA AA AA AA AG AG AG
AG AG AG AG AG AG AA AG
AA AA AA AA AG AA AG AG
AA GG AG AG AA AA AA
80486198
2439 rs10055910 5 AG GG AG GG GG
AG GG AG AG AG GG GG GG
GG GG AG GG AG AG AG AG
AA AA AA AA GG GG GG GG
GG GG GG GG AG AG AG
167553880
2440 rs10055969 5 AG GG GG GG GG
GG GG AG GG AG GG GG GG
GG GG AG GG AG AA GG AG
GG AG AG GG AG AG AA AG
AG GG AG GG AG AG GG
100620693
2441 rs10056042 5 GG GG AG GG AG

AG GG AG AA AG AG AG AG
AA AA GG AG AG GG GG AG
AG AG AG AG AG GG GG GG
AA AG GG GG GG GG GG
8295533
2442 rs10056109 5 GG GG GG AG GG
GG AG GG GG GG GG GG GG
GG GG GG GG GG GG GG AG
GG GG GG GG GG GG GG GG
AG GG GG GG GG AG GG
103164240
2443 rs1005611 20 GG AG AG AA GG
AG AG GG GG AG AA AA AA
AA AA AG AA AG GG AG AG
GG GG GG GG AG GG GG GG
GG GG GG GG GG GG AG
37232009
2444 rs10056116 5 AA AG AG GG GG
GG GG AG GG AG AA AG AA
GG GG AG AG GG AG AA AG
GG GG AA AG GG GG GG GG
AG GG GG GG GG GG GG
1676638
2445 rs1005612 X GG AG AA AA AA
AA AA AG AG AA AA AA AG
AG AG GG AG AG AA AA AA
AA AA AA AG AG AA AA GG
AA GG AA GG AA AA AA
55518239
2446 rs10056131 5 AC AC AA AC AA
AC AA AC AA AC CC CC AC
CC AC AA AA AA AA AA AA
AC AC AC AC AC AA AC AC
AC AC AA AA AC CC AC
28302858
2447 rs10056157
5 -- -- -- -- -- -- -- --
-- -- -- -- -- -- -- --
-- -- -- -- -- -- -- --
-- -- -- -- -- -- -- --
-- -- -- -- 51170435
2448 rs10056165 5 GG GG GG GG GG
GG GG GG GG GG GG AG GG
GG GG GG GG GG GG GG GG
GG GG GG GG AG GG AG AG
GG GG GG GG GG GG AG
128661339
2449 rs1005617 22 GG GG AG GG GG
GG GG GG GG GG GG GG GG
GG GG AG GG GG AG AG AG

GG AG AG GG GG GG GG GG
AG AG GG GG GG GG GG
45421242
2450 rs10056236 5 GG GG GG GG GG
GG GG GG GG GG GG GG GG
GG GG GG GG GG AG AG GG
GG GG GG GG AG GG AG AG
GG GG GG GG GG GG GG
80853708
2451 rs10056261 5 GG GG AG GG GG
GG GG GG GG GG AA AA AG
AG AG GG GG GG GG GG GG
GG GG GG GG AA GG AG AG
GG AG GG AG GG GG GG
6401960
2452 rs10056274 5 AA AA AA AA AA
AA AA AG AG AG AA AA AA
AA AA AA AA AA AA AA AA
AA AA AA AA AA AG AG AA
AA AA AA AA AG AA AG
25695377
2453 rs10056291 5 CC -- CC CC CC
CC CC CC CC CC CC CC AC
AC AC AC AC CC AC CC CC
CC CC CC CC CC AC AC CC
CC AC AC AC AC AA CC
116070510
2454 rs10056327 5 AA AA AA AA AA
AA AA AA AA AA AA AA AA
AA AA AA AA AA AA AA AA
AA AA AA AA AA AA AA AA
AA AA AA AA AA AA AA
121745574
2455 rs1005640 22 AG AG GG AA AA
AA AG AG AG AG AG GG AG
AG AG AA AA AA AA AA AG
AG AG AG AA AG AG AG AA
AG AG AG AG AA AG AG
20789074
2456 rs10056443 5 AG AA AG GG GG
AG GG GG AG AG AG GG AG
AG AG GG GG GG GG AA GG
AG AG AG AG AG GG AG AG
AG AG GG AG GG GG GG
88752584
2457 rs10056448 5 GG GG GG GG GG
GG GG GG GG GG GG GG GG
GG GG GG GG GG GG GG GG
GG GG GG GG GG GG GG GG
GG GG GG GG GG GG GG

55064760
2458 rs10056461 5 CC CC CC CC CC
CC CC CC CC CC CC CC CC
CC CC CC CC CC AC AC AC
CC CC CC CC AC CC AC AC
CC CC AC CC CC CC CC
20325196
2459 rs1005649 X GG AG AA AG GG
AA GG AG AG GG GG GG GG
GG GG GG GG GG AG GG AA
AA AA AA AA AG AA AG AA
AG AG AA GG GG GG GG
119270135
2460 rs10056503 5 GG GG GG GG GG
GG GG GG GG GG GG GG AG
GG AG GG GG GG AG AG AG
GG GG GG GG GG GG GG GG
GG GG GG GG GG GG GG
168096257
2461 rs1005651 17 AA AC AC AA AA
AA AA AA AA AA AA AA AA
AC CC AA CC CC AA AA AA
AA AC AA AC AA CC AC AC
AC AA AC AC AA AA AA
64438011
2462 rs10056525 5 GG AG GG AG GG
AG GG AG AG AG AG AG GG
GG AG AG AA AG GG AG GG
GG GG GG GG GG AG GG GG
GG GG GG GG AG GG AG
151964513
2463 rs1005655 17 AG GG GG AG AA
AG AA GG AG AG GG GG GG
GG AG GG GG GG GG GG GG
AA AG AG AG GG AA AG AG
AG GG GG GG GG AG AG
45919465
2464 rs1005656 4 CC CC AC CC CC
CC CC CC CC CC AC CC AC
CC CC AA AC AC CC CC CC
AC AC AC CC CC CC CC CC
CC CC AC CC CC CC CC
55404602
2465 rs10056588 5 AA AA AA AA AA
AG AA AA AA AA AA AA AA
AA AA AA AA AA AA AA AA
AG AG AG AG AA AG AG AG
AA AA AA AA AA AA AA
174314270
2466 rs10056632 5 CC CC AC AA AA

AC AC AC AC AC AA AC AC
AA AA AC AC AC AA AA AC
AA AA AA AA AA AC AC AA
AC AC AA AA AC AA CC
79142191
2467 rs10056747 5 AA AG GG AG AA
AG AA AG AA AG AG AA AA
AG AG AA AA AA AA AA AA
AG AG AG AG AA AA AA AA
AA AA AG AA AG AA AG
25595051
2468 rs10056772 5 AG AA AA AG GG
AG AG GG GG GG AG AG AG
AG GG AG AG AA AG AA AA
AG AG AG AG AA AA AA AA
AA AA AG AG AG AG AG
26267717
2469 rs10056811 5 GG AG AG AG AG
AA GG AG AG AA AA AG AA
AG AG GG GG GG AG GG AG
GG AG GG GG AA AG AA AG
AA AG AA AG AG GG AG
74605220
2470 rs10056822 5 GG GG GG AG AG
AG GG GG GG GG GG GG GG
GG GG GG GG GG GG GG GG
GG GG GG GG GG GG GG GG
GG GG GG GG GG GG GG
43084411
2471 rs10056849 5 CC CC CC CC CC
CC CC CC CC CC CC CC CC
CC CC CC CC CC CC CC CC
CC CC CC CC CC CC CC CC
CC CC CC CC AC AC AC
20265645
2472 rs10056890 5 AG AG GG GG GG
GG GG GG GG GG GG GG GG
GG GG GG GG GG GG GG GG
GG GG GG GG GG GG GG GG
AG AA GG AG AG AG GG
169913253
2473 rs1005693 21 AG GG AG AG AG
AG AA GG GG GG GG GG GG
AA AG GG GG -- AG GG AA
GG GG GG GG GG GG GG GG
AG AG GG AG GG GG GG
44092814
2474 rs10056943 5 CC AC AA CC CC
CC CC AC AC AC CC CC CC
AC AC AA AC AC AC AA CC

CC AC CC CC CC AC CC AC
AA CC CC CC AC CC AC
75897600
2475 rs10056950 5 AA AG AG GG AG
AG AG GG AG GG AA AG AA
AA AA AG GG AG AG AA AA
AA AA AA AA AA AA AA AA
AA AA AA AA AA AA AG
26762292
2476 rs10056970 5 AG AG GG GG GG
AG GG AG AG AG GG GG AG
AG AG GG GG GG GG AG GG
AG AG AG AG GG AG AG GG
AG AG GG GG AG AG GG
109405571
2477 rs10057007 5 AG GG AG AA AA
AA AA AA AA AG AA AA AG
AA AG GG AG AG AG AG GG
AG AG AA AG AA AA AA AA
AG AG AA AA AG GG AA
13882841
2478 rs10057009 5 AA AA AA AG AA
AG AA AA AA AA AA AG AA
AG AA AG AA AG AA AA AA
AA AA AA AA AA AA AA AA
AA AG AA AA AA AA AA
17445833
2479 rs10057011 5 GG GG GG GG GG
GG AG GG GG GG GG GG AG
AG AG GG GG GG AG GG GG
GG GG GG GG GG GG GG GG
GG AG GG GG GG GG GG
174161249
2480 rs10057028 5 GG AG AA AG AG
AG AA AA AA AA GG AA AG
AG AG GG AG AG AG AG GG
AG GG GG AG AA AG AA AG
AG AA AG AA AG AG AG
57795822
2481 rs10057041 5 GG GG GG GG GG
GG GG GG GG GG GG GG GG
GG GG GG GG GG GG GG GG
GG GG GG GG GG GG GG GG
GG GG GG GG GG GG GG
54422970
2482 rs10057043 5 AA AA AA AA AA
AA AA AA AA AA AG GG AG
AA AA AA AA AA AG GG AG
GG GG AG GG AA AA AA AA
AA AA AA AA GG GG AG

18146907
2483 rs10057069 5 GG AG AG AA AA
AA AG AG AG AG GG AG AG
AG AA AG AA AG AG AG GG
AG GG GG AG AA AA AA AA
AA GG AA AG AA AA AG
32716589
2484 rs10057084 5 GG GG GG AA AA
AG AA GG GG GG GG GG GG
GG GG AA AA AA AG AA AG
GG AG AG AG AA AG AA AG
GG GG GG GG AG AG GG
126324100
2485 rs10057104 5 GG GG GG GG GG
AG GG GG GG GG GG GG GG
GG GG GG GG GG GG GG GG
GG GG GG GG GG GG GG GG
GG GG GG GG GG GG GG
174903461
2486 rs10057158 5 AG AG AG AG AG
AG AG AA AA AA AG AG AA
GG GG AG GG AG AG AG AA
AA AA AG AG AA AG AA AG
AG AA AG AG AA AG AA
2179628
2487 rs10057196 5 AA AA AA AA AA
AA AA AA AA AA AA AG AA
AA AA AA AA AA AA AA AA
AA AA AA AA AA AA AA AA
AA AA AA AA AA AA AA
85795217
2488 rs10057220 5 GG GG GG GG GG
GG GG GG GG GG GG GG GG
GG GG GG GG GG GG GG GG
GG GG GG GG GG AG AG GG
GG GG GG GG GG GG GG
18838134
2489 rs10057241 5 AG GG GG GG GG
GG AG GG GG GG GG GG GG
AG AG GG AG AG GG GG AG
AG GG AG AG AG AG AG AG
AG AG AG AG GG AG GG
64936304
2490 rs10057258 5 AG AG AG AA AA
AG AA AA AA AA GG AG GG
GG GG AG AA AG AA AG AA
AA AA AA AA AA AG AA AG
AA AG AA AA AG AA AG
70771693
2491 rs1005726 3 GG GG GG GG GG

	AG	GG	GG	AG	AG	GG	GG	GG
	GG	GG	AG	GG	AG	GG	GG	GG
	GG	GG	GG	GG	GG	GG	GG	GG
	GG	GG	GG	GG	GG	GG	GG	
150886786								
2492	rs10057304		5	AC	AC	AC	CC	CC
	AC	CC	CC	CC	CC	CC	CC	CC
	CC	CC	CC	AC	AC	CC	CC	CC
	CC	CC	CC	CC	CC	CC	CC	CC
	CC	CC	CC	CC	CC	CC	CC	
54887522								
2493	rs10057320		5	GG	GG	GG	GG	GG
	GG	GG	AG	GG	AG	GG	GG	GG
	GG	GG	AG	AG	AA	GG	GG	GG
	GG	GG	GG	GG	GG	GG	GG	GG
	GG	AA	GG	AG	GG	GG	GG	
98250805								
2494	rs10057441		5	CC	CC	CC	CC	CC
	CC	CC	CC	CC	CC	AC	AC	AC
	CC	CC	CC	CC	CC	AC	CC	CC
	CC	CC	CC	CC	CC	CC	CC	CC
	CC	AC	CC	AC	CC	CC	CC	
172475038								
2495	rs10057451		5	AA	AA	AA	AA	AA
	AA	AA	AA	AA	AA	AA	AA	AA
	AA	AA	AA	AA	AA	AA	AA	AA
	AA	AA	AA	AA	AA	AA	AA	AA
	AA	AA	AA	AA	AA	AA	AA	
22723631								
2496	rs10057455		5	AA	AA	AA	AA	AA
	AA	AA	AA	AA	AA	AA	AA	AA
	AA	AA	AG	AA	AG	AA	AA	AA
	AA	AA	AA	AA	AA	AA	AA	AA
	AA	AA	AA	AA	AA	AA	AA	
6520414								
2497	rs10057484		5	AG	GG	AG	AA	AA
	AA	AA	AA	AG	AA	AA	AA	AG
	AA	AG	AG	AA	AG	AA	AG	AA
	AA	AA	AA	AA	AG	AA	AG	AG
	AG	AG	AG	AG	AG	GG	AA	
27592140								
2498	rs10057519		5	AA	AA	AA	AC	AC
	AA	CC	AA	AA	AA	AA	AA	AA
	AA	AA	AA	AA	AA	AC	AA	AA
	AA	AA	AA	AA	AA	AC	AA	AA
	AA	AA	AA	AA	AA	AA	AA	
175730807								
2499	rs10057525		5	GG	GG	AG	AG	AG
	GG	AG	AA	AA	AA	GG	AG	GG
	GG	GG	AG	AG	GG	AG	AG	AG

GG GG GG GG GG AG AG GG
GG GG AG GG AA AG AG
21440363
2500 rs1005753 1 AC AC AA AA AC
AA AC AC AA AA AA AA AC
AC AA AC CC AC AC AC AC
AC AC AC AC AC AA AA AA
CC AC AA AC AC AC AA
17444769
2501 rs10057538 5 AA AA AA AG AG
AG AA AG AG AG AG AG GG
GG GG AA AA AA AA AG AG
AG AG AG AG AA AA AA AA
GG AA AA AA AA AA AA
173524269
2502 rs10057544 5 AG AG AG AG GG
GG AG GG AG GG GG GG GG
GG GG GG GG GG GG GG AG
GG GG GG GG GG GG GG GG
AG AG GG AG AG AG AG
98554365
2503 rs10057549 5 AA AG AG AA AA
AA AA AG AG AG AG AA AA
AA AA AA AA AA AA AA AA
AG AA AA AG AG AA AG AA
AA AG AG AA AA AA AA
56803101
2504 rs10057630 5 AA AA AA AA AA
AA AA AA AA AA AA AA AA
AG AA AG AG AG AA AA AA
AA AA AA AA AA AA AA AA
AA AA AA AA AA AA AA
44327864
2505 rs10057632 5 AA AA AA AA AA
AA AA AA AA AA AA AA AA
AA AA AA AA AA AA AA AA
AA AA AA AA AA AA AA AA
AA AA AA AA AA AA AA
27395258
2506 rs10057680 5 AG AA AG AG AG
AG GG AG AG AG AA AA AA
AA AA AA AA AA AA AA AA
AG AG AG AG AA AA AA AA
AG AA AA AA AA AG AA
167147874
2507 rs10057748 5 CC CC CC AC AC
CC AA CC AC AC CC CC CC
CC CC AC AC AC CC CC CC
AA AC AC AA AC AC AC AA
AC CC AC AC CC CC CC

86665098
2508 rs1005776 14 GG AG AG AG AG
AA AG GG AG GG GG AA AG
AG GG GG GG GG AG AA AG
GG GG GG GG GG GG GG GG
AG AA GG AG GG GG AG
102896673
2509 rs10057806 5 GG GG GG GG GG
GG GG GG GG GG GG GG GG
GG GG GG GG GG GG GG GG
GG GG GG GG GG GG GG GG
GG GG GG GG GG GG GG
168876634
2510 rs10057809 5 CC AC AC AA AC
AC AA AA AA AC CC AC AC
AC AC AA AA AA AA AC AA
AA AA AA AA AA CC AC AC
AC AC AA AA AA AA AC
13482159
2511 rs10057863 5 AA AA AA AA AA
AA AA AA AA AA AA AA AA
AA AA AA AA AA AA AA AA
AA AA AA AA AA AA AA AA
AA AA AA AA AA AA AA
24936638
2512 rs10057867 5 CC CC CC CC CC
CC CC CC CC CC CC CC CC
CC CC CC CC CC CC CC CC
CC CC CC CC CC CC CC CC
CC CC CC CC CC CC CC
20514682
2513 rs1005790 5 GG GG GG AG AA
AG AG AG AG AG AA AA AA
AG AG AA AA AA AG GG GG
GG GG GG GG AA AG AA AA
AG AG AG AA AG AG GG
160174947
2514 rs10057902 5 GG GG GG GG GG
GG GG GG GG GG GG GG GG
GG GG AG GG AG GG GG GG
GG GG GG GG GG GG GG GG
GG GG GG GG GG GG GG
93812885
2515 rs10057965 5 AA AA AA AA AA
AC AA AC AC AC AC AC AC
AA AA AA AA AA CC AC AC
AA AA AA AA CC AA AC AC
AA AC AA AA AA AC AA
163119808
2516 rs10057969 5 AC CC CC AC AA

	AC	AC	AA	AA	AA	AA	AC	AA
	AA	AA	AA	AC	AC	AA	AC	AC
	AA	AA	AA	AA	AA	AC	AC	AA
	AC	AC	AA	AC	AC	AC	AC	
98599968								
2517	rs10057985		5	AG	AG	GG	GG	GG
	AG	GG	AG	GG	GG	AA	AG	AA
	AA	AA	AA	AG	AG	GG	AA	AG
	AA	AG	AG	AA	AG	AA	AG	AG
	AA	AA	GG	AG	AA	AG	AG	
91884247								
2518	rs10058011		5	GG	GG	GG	GG	GG
	GG	GG	GG	GG	GG	GG	GG	AG
	GG	GG	GG	GG	GG	GG	GG	GG
	GG	GG	GG	GG	GG	GG	GG	GG
	GG	AG	AG	AG	GG	GG	GG	
123846126								
2519	rs10058062		5	AA	AA	AA	AA	AA
	AA	AA	AA	AA	AA	AA	AA	AA
	AA	AA	AA	AA	AA	AA	AA	AA
	AA	AA	AA	AA	AA	AA	AA	AA
	AA	AA	AA	AA	AA	AA	AA	
37659105								
2520	rs10058091		5	AA	AA	AA	GG	GG
	GG	AG	AA	AA	AG	AG	AA	AG
	AG	AG	AG	AG	AG	GG	AA	GG
	AG	AG	AG	AG	AG	AA	AA	AA
	GG	AG	GG	AG	GG	AG	GG	
76922658								
2521	rs10058105		5	GG	GG	GG	GG	GG
	GG	GG	GG	GG	GG	GG	GG	GG
	AG	AG	GG	GG	GG	GG	GG	GG
	GG	GG	GG	GG	GG	GG	GG	GG
	GG	GG	GG	GG	GG	GG	GG	
1058718								
2522	rs1005822		16	AC	AC	AC	AC	AC
	CC	AA	AA	AA	AA	AC	AA	AC
	AC	CC	AC	AC	AC	AA	AA	AA
	AC	AA	AC	AC	AC	AC	AC	AC
	CC	AC	CC	CC	AC	AC	AC	
64876677								
2523	rs10058223		5	GG	GG	GG	GG	GG
	GG	GG	GG	GG	GG	GG	GG	GG
	GG	GG	GG	GG	GG	GG	GG	GG
	GG	GG	GG	GG	GG	GG	GG	GG
	GG	GG	GG	GG	GG	GG	GG	
31546392								
2524	rs10058259		5	AA	AA	AA	AA	AA
	AA	AA	AA	AG	AA	AA	AG	AA
	AA	AA	AG	AG	AA	AA	AG	AA

AG AG AA AG AA AA AA AA
AA AA AA AA AA AA AA
23371163
2525 rs10058270 5 AG AA AA AA AA
AA AG AA AA AA AA AA AA
AG AG AA AA AA AA AA AA
AA AA AA AA AA AA AA AA
AA AG AA AG AA AA AA
1815950
2526 rs10058291 5 AA AG AG AA AA
AA AA AA AA AA AA AA AA
AA AA AG GG AG AA AA AA
AA AA AA AA AA AA AA AA
AA AA AA AA AA AA AA
58194892
2527 rs10058292 5 AC AA AC CC AC
AC CC AA AC AA AC AC AC
AA AA AA AC AC AA AC AA
AC AC AC AC AC AA AC AC
AA AC AC CC AC AA CC
13895609
2528 rs10058293 5 GG GG GG GG GG
GG GG GG GG GG AG AG GG
GG GG GG GG GG GG GG AG
GG GG GG GG GG GG GG GG
GG GG GG GG GG GG GG
121063011
2529 rs10058297 5 AG AG AA AA AA
AA AA GG AG GG AG AG AG
GG GG AG GG AG AA AA AA
GG GG AG GG AA AG AA AG
AG AA GG AG AG AG AA
29768342
2530 rs10058333 5 AA AA AG AG AG
AA AG AA AA AA AA AA AA
AA AA AA AG AG AA AA AA
AA AA AA AA AA AG AA AG
AA AG AA AG AA AA AA
19331778
2531 rs10058372 5 GG GG GG GG --
GG GG -- GG GG GG GG GG
GG GG -- GG -- -- AG GG
GG GG -- GG GG AG AG GG
AG AG GG GG GG GG GG
106249782
2532 rs10058433 5 AA AA AA AG AA
AA AG AA AA AA AG AG AG
AA AA AA AG AG AA AA AA
AA AA AA AA AA AA AA AA
AA AA AA AA AG GG AA

163820512
2533 rs1005844 15 AG GG AG GG AG
AG GG AA AA GG AA AG AG
GG AG AG GG GG GG AG AG
GG GG GG AA AA AG AG AA
GG AG AG AA GG AG AG
100659526
2534 rs10058554 5 GG GG GG GG GG
GG GG AG AG AG GG GG GG
AG AG GG AG AG GG GG GG
GG GG GG GG GG GG GG GG
GG AG AG AA GG AG GG
34530330
2535 rs10058557 5 GG GG GG AG AG
AG GG GG GG AG GG GG GG
AG AG GG AG GG AG AG AA
GG GG GG GG AG GG GG AG
GG AG AG AG AG GG AA
73287080
2536 rs10058558 5 AG AG AG GG AG
GG AG AG AA AG AA AA AA
AA AA GG AG AG AA AG AA
AG AA AA AG GG AG GG AG
AG AA AG AA GG GG GG
43539868
2537 rs1005856 20 AA AG AG GG AG
AG AG GG GG AG AA AA AG
AG AA AG GG AG AA AA AG
GG AG AG AG AG AA AG AG
AG AA AG AG GG AG AG
23191499
2538 rs10058571 5 AA AA AA AA AA
AA AA AA AG AA AG AA AG
AA AG AA AA AA AG GG AA
AA AA AA AA AA AA AA AA
AG AA AA AA AA AA AA
54104560
2539 rs1005858 11 GG AG AG AA AG
AG AG AG GG GG AG AG AG
AG AG GG GG GG GG GG AG
AG GG AG GG AA GG AG AG
GG AG AA AG GG GG GG
68856916
2540 rs10058615 5 AA AA AC AC AC
CC AA AA AA AA AA AC AA
AA AA AC AC AA AA AA AA
AA AA AC AA AA AA AA AA
AC AA AC AA AA AC AC
28483186
2541 rs10058663 5 GG GG GG GG GG

GG GG GG GG GG GG GG GG
GG GG GG GG GG GG GG GG
GG GG GG GG GG GG GG GG
GG GG GG GG GG GG GG
25764097
2542 rs10058673 5 AA AG AG GG AG
AG AG AA AA AA AA AA AA
AG AG AG GG AG AA AG AA
AA AG AG AG GG AG GG AG
AA AG AA AG AG AG AG
7202086
2543 rs10058699 5 GG GG GG GG GG
GG GG GG GG GG GG GG GG
GG GG GG AG AG GG GG GG
GG AG AG GG GG AG AG GG
GG GG GG GG GG GG GG
110855952
2544 rs10058717 5 AA AA AA AA AA
AA AA AA AA AA AA AC AA
AA AA AA AA AA AA AA AA
AC AA AA AA AA AA AA AA
AA AA AA AA AA AA AA
124205851
2545 rs1005873 22 AA AA AA AA AA
AA AA AA AA AG AG AG AA
AA AA AA AG AG AA AA AA
AG AG AG AA AA AA AA AA
AG AG AG AG AA AA AA
19900075
2546 rs10058772 5 AG AG GG AG AG
GG AA AG AG AG AG AG AA
AA AA GG GG GG GG GG GG
GG GG GG GG AG GG AG GG
GG AG GG AG GG GG GG
180033292
2547 rs10058792 5 CC CC CC CC CC
CC CC CC CC CC CC CC CC
CC CC CC CC CC CC CC CC
CC CC CC CC CC CC CC CC
CC CC CC CC CC CC CC
92927718
2548 rs10058793 5 AA AA AA AA AA
AA AA AA AA AA AA AA AA
AA AA AA AA AA AA AA AA
AA AA AA AA AG AA AA AA
AA AA AA AA AA AA AA
4739819
2549 rs10058844 5 AA AG AA GG AG
GG AG GG GG GG AG AA AG
AA AA AG GG GG AG AA AA

	GG	AG	AG	AG	AA	AG	AA	AG
	AA	GG	AG	AG	AG	AA	AG	
117847097								
2550	rs10058864		5	GG	AG	AA	AG	AG
	AA	GG	GG	AG	GG	AA	AA	AG
	AG	AG	AG	AA	AG	AA	AG	AA
	AA	AA	AA	AA	AA	AA	AA	AA
	AA	AG	AG	GG	AG	AA	AG	
13937803								
2551	rs10058883		5	AA	AA	AA	AA	AA
	AG	AA	AG	AA	AG	AA	AA	AA
	AA	AG	AG	AA	AA	AA	AA	AA
	AA	AA	AA	AA	AA	AA	AA	AA
	AA	AA	AA	AA	AA	AA	AG	
41220990								
2552	rs1005890		12	AA	AA	AA	AA	--
	AA	--	AA	AA	AA	AA	AA	AA
	--	AA	AA	--	--	AA	AA	AA
	AA	AA	--	AA	AA	AA	AA	AA
	AA	--	AA	AA	--	--	AA	
94277882								
2553	rs10058915		5	AG	GG	GG	AG	GG
	AG	GG	GG	GG	GG	AG	AG	GG
	AG	AG	AG	GG	AG	GG	GG	AG
	GG	GG	GG	GG	GG	GG	GG	GG
	AG	GG	AA	AG	GG	AG	AG	
10115230								
2554	rs10058918		5	CC	CC	CC	CC	CC
	CC	CC	CC	CC	CC	CC	CC	CC
	CC	CC	CC	CC	CC	CC	CC	CC
	CC	CC	CC	CC	CC	CC	CC	CC
	CC	CC	CC	CC	CC	CC	CC	
43775682								
2555	rs1005893		19	AA	AA	AA	AA	AA
	AA	AA	AA	AA	AA	AA	AA	AA
	AA	AA	AA	AA	AA	AA	AA	AA
	AA	AA	AA	AA	AA	AA	AA	AA
	AA	AA	AA	AA	AA	AA	AA	
5593638								
2556	rs10058955		5	AC	AC	AC	CC	CC
	CC	AC	AC	CC	AC	AC	CC	AC
	CC	AC	CC	CC	CC	AA	AC	AC
	AC	AC	AC	AC	AC	AC	AA	CC
	AA	CC	CC	CC	AC	AC	CC	
35787873								
2557	rs10058970		5	AG	AG	AG	AA	AA
	AG	AA	AA	AA	AA	AA	AA	AA
	AA	AA	AA	AA	AA	AA	AA	AG
	AA	AA	AA	AA	AA	AA	AA	AA
	AA	AA	AA	AA	AA	AA	AA	

83030867
2558 rs10059016 5 GG AG GG GG GG
GG GG GG GG GG AA AG AG
GG AG GG GG GG GG GG GG
GG GG GG GG GG GG GG GG
GG GG GG GG GG AG GG
41103175
2559 rs1005902 12 AC CC AC AA CC
AC AC AC AC AA AC CC AC
AC AA CC CC CC AA AC AC
AA AA AA AA AC AC AC CC
AA AC AA AA AC AC CC
112667675
2560 rs10059045 5 CC AC CC AC AC
AC CC AC AC AC AC AA AC
AC AC AC CC CC CC CC CC
AA AC AC AA AA CC AC AC
AA AC AA AA AC AC AC
85687037
2561 rs10059141 5 GG GG AG AG AG
GG AG GG GG GG GG GG AG
GG AG GG GG GG GG GG GG
GG GG GG GG AG GG AG AG
GG AG GG AG AG AG GG
19195667
2562 rs10059157 5 AA AA AA AA AA
AA AA AG GG AG AG AG AA
AA AA AA AA AA AG AA AG
AA AA AA AA AG AG GG AG
AA AA GG AG AA AA AA
143216173
2563 rs10059179 5 AA AG AA AG AG
AG AG GG GG GG GG GG GG
GG AG AG AG GG AG AG AG
GG GG GG GG GG GG GG GG
GG AG GG AG GG GG AG
35516582
2564 rs10059206 5 GG AG AG GG GG
GG GG GG GG GG GG AA AG
AG GG AG AG GG GG AG GG
GG GG GG GG GG GG GG GG
GG AG GG AG GG GG GG
64106918
2565 rs10059222
5 -- -- -- -- -- -- -- --
-- -- -- -- -- -- -- --
-- -- -- -- -- -- -- --
-- -- -- -- -- -- -- --
-- -- -- -- 22003397
2566 rs10059242 5 AA AA AA AA AA

AA AA AA AA AA AA AA AA
AA AA AA AA AA AA AA AA
AA AA AA AA AA AA AA AA
AA AA AA AA AA AA AA
148182123
2567 rs10059243 5 AG AG AG AA AA
AG AA AA AA AA AA AA AA
AA AG AA AA AA AA AG AG
AA AA AA AA AG AG AA GG
AG AA AG AA AA AA AG
55355145
2568 rs10059285 5 CC CC CC CC CC
CC CC CC CC AC AC AC AC
AC AC CC CC CC CC CC CC
AC CC AC AC AA CC AC AC
AC CC CC CC CC CC AC
77572439
2569 rs10059292 5 AG AA AG AA AA
AA AA AA AA AA AA AA AA
AA AA AA AA AA AA AA AA
AA AA AA AA AA AA AA AA
AA AA AA AA AA AA AA
168637547
2570 rs1005930 18 AG AG AG AG AG
AG GG AA AG AA GG AG AG
GG AA AG AA AG AG AG AG
AA AA AA AA GG AG AG GG
AG AA AA AA GG AG GG
10143877
2571 rs10059300 5 AG AG GG GG GG
AG GG AG AG AA GG AG GG
GG GG GG AG AG AA AG AG
GG GG GG GG AG AG AG AG
AG AG GG GG GG GG GG
56368573
2572 rs10059320 5 CC CC AC CC CC
CC CC AC CC AC CC AC CC
CC CC CC CC CC CC CC CC
CC CC CC CC CC CC CC CC
CC CC AC AC CC CC CC
114531072
2573 rs10059334 5 AA AG GG AG AG
GG AA AA AA AA AA AG AG
AG AG AA AG AG AG AG AG
AG AG AG AG AG AG AG AG
GG AA GG AG AA AG AG
59517876
2574 rs10059337 5 AG AG GG GG --
AG AG AG GG AG AA AA AG
AG AG AG AA AG AG AA AA

	AG	AG	AG	AG	GG	AA	AG	AG
	AA	AA	AA	AA	AG	GG	AA	
27094544								
2575	rs1005934		11	AA	AA	AA	AA	AA
	AA	AA	AA	AA	AA	AA	AA	AA
	AA	AA	AA	AA	AA	AA	AA	AA
	AA	AA	AG	AA	AA	AG	AG	AA
	AA	AA	AA	AA	AA	AA	AA	
33302060								
2576	rs10059409		5	GG	GG	GG	GG	GG
	GG	GG	GG	GG	GG	GG	GG	GG
	GG	GG	GG	GG	GG	GG	GG	GG
	GG	GG	GG	GG	GG	GG	GG	GG
	GG	GG	GG	GG	GG	GG	GG	
124464984								
2577	rs10059437		5	GG	GG	GG	GG	GG
	GG	GG	GG	GG	GG	GG	GG	GG
	GG	GG	GG	GG	GG	GG	GG	GG
	GG	GG	GG	GG	GG	GG	GG	GG
	GG	GG	GG	GG	GG	GG	GG	
175585898								
2578	rs10059533		5	AA	AA	AG	GG	GG
	GG	AG	GG	GG	GG	GG	AG	GG
	GG	GG	GG	GG	GG	GG	AG	AG
	AG	AG	AG	AG	AG	AG	GG	AG
	GG	GG	AG	GG	GG	AG	AG	
83173714								
2579	rs10059547		5	GG	GG	GG	AG	AG
	AG	GG	GG	GG	GG	GG	GG	GG
	GG	GG	GG	AG	GG	GG	GG	GG
	AG	AG	AG	AG	GG	GG	GG	GG
	GG	GG	GG	GG	GG	GG	GG	
180109162								
2580	rs10059603		5	AG	AG	AG	AG	AG
	AG	GG	GG	GG	GG	GG	GG	GG
	GG	GG	GG	AG	AG	AG	GG	AG
	GG	GG	GG	GG	GG	GG	GG	GG
	GG	GG	AG	AG	GG	GG	GG	
43851579								
2581	rs10059632		5	AG	AG	AG	AG	AG
	AG	AA	AA	AA	AA	AA	AA	AA
	AA	AA	AA	AA	AA	AA	AG	AA
	AG	AG	AG	AG	AA	AG	AG	AA
	AG	AA	AG	AA	AA	AA	AA	
97219737								
2582	rs10059646		0	GG	GG	GG	AG	AG
	AG	GG	GG	GG	GG	AG	AG	AG
	AG	AG	GG	GG	GG	GG	GG	GG
	AG	AA	AA	AG	AG	GG	AG	AG
	AG	AA	AG	AG	GG	GG	GG	0

2583 rs10059647 5 CC CC CC AC AA
AA AC AC AC AC AC AA AA
AC AC AC CC AC AA AC AC
CC CC CC CC AA AA AA AA
CC AC AC AC CC CC AC
111607304
2584 rs10059648 5 GG AG GG GG GG
GG GG GG GG GG AG AG AG
AG AG GG GG GG GG AA GG
AA AG AG AA GG GG GG GG
AA GG GG GG GG GG GG
84465785
2585 rs10059688 5 AA AA AA AA AA
AA AA AA AA AA AA AA AA
AA AA AA AG AG AG AA AG
AA AG AG AA AA AG AA AG
AA AA AA AA AA AA AA
35893746
2586 rs10059727 5 GG GG GG GG GG
GG GG GG GG GG GG GG GG
GG GG GG GG GG GG GG GG
GG GG GG GG GG GG GG GG
GG GG GG GG GG GG GG
44566221
2587 rs10059733 5 AG AG GG AA AA
AA AA AG AG AG AA AA AG
AG AG AG GG AG AG AA AG
AG AG AG AG AA AA AA AA
AA AA AG AG AA AA AA
171908794
2588 rs10059784 5 AG AA AA AA AA
AA AA AA AA AA AA AA AA
AA AA AA AA AA AA AA AA
AA AA AA AA AA AA AA AA
AG AA AA AA AA AA AA
118942612
2589 rs10059816 5 AG AA AA AA AA
AA AG AA AA AA AG AG AG
AA AA AA AA AA AA AA AA
AG AG AG AG AG AA AA AA
AA AA AA AA AA AA AA
12579343
2590 rs10059820 5 AA AA AA AA AA
AA AA AA AA AA AA AA AA
AA AA AA AA AA AA AA AA
AA AA AA AA AA AA AA AA
AA AA AA AA AA AA AA
124366575
2591 rs10059843 5 AA AA AA AA AA
AA AA AA AA AA AA AA AA

AA AA AA AA AA AA AA AA
AA AA AA AA AA AA AA AA
AA AA AA AA AA AA AA
19654956
2592 rs10059847 5 AA AG AG AG AG
AA GG AA AA AA AG AG AA
GG GG AG AG AA AA AA AA
AA AA AA AA AA AA AA AA
GG AA AG AA AG AA GG
6757720
2593 rs10059861 5 AG GG GG AG AG
AA GG AA AG AA AA AA AA
AA AA AA AG AG AA AA AA
AA AG AG AA AA AA AA AA
AA AA GG AG AG AG AA
98425232
2594 rs1005988 3 AA AA AA AC AA
AC AC AA AA AA AA AA AA
AA AA CC AC AC AA AA AA
AA AC AA AA AA AC AC AC
AC AC AA AC AC AC AA
8896462
2595 rs10059916 5 AA AA AG AA AA
AA AA AA AA AA AG AG AA
AA AA AA AA AA AG AA GG
AA AA AA AA AA AA AA AA
AG AA AA AA AG AG AA
56908982
2596 rs10059955 5 AG AG GG GG AG
AG AG GG AG GG GG GG GG
GG GG GG GG GG AG AA AG
AG AG AG AG AG AG GG AG
GG AG AG GG GG GG GG
105425352
2597 rs10059981 5 AA AA AA AA AA
AA AA AA AA AA AA AA AA
AA AA AA AA AA AA AA AA
AA AA AA AA AA AA AA AA
AA AA AA AA AA AA AA
3619518
2598 rs10059993 5 AA AA AA AA AG
AA AG AG GG AG GG GG GG
GG GG AA AA AA AG AG AG
AG GG GG AG AA AG AA AG
AG GG AA AG AA AA AA
43598352
2599 rs10060008 5 AG AG AA AA AA
AA AA AG AG AG AA AA AA
AA AA AG AG AG AA AA AA
GG AG GG GG AG AA AG AG

AG AG AA AA AG AG AG
82072989
2600 rs10060036 5 AA AG GG AG AG
AG GG AG AG AG GG AG AG
AG GG AA AG AG GG GG AG
AG AG AG AG GG AG GG AG
AA AG GG GG GG GG GG
61366182
2601 rs10060053
5 -- -- -- -- -- -- -- --
-- -- -- -- -- -- -- --
-- -- -- -- -- -- -- --
-- -- -- -- -- -- -- --
-- -- -- -- 177120697
2602 rs10060054 5 GG GG GG GG GG
GG GG GG GG GG GG GG AG
GG GG GG GG -- GG GG GG
GG AG AG AG GG GG GG GG
GG GG AA AG GG GG GG
7749260
2603 rs10060059 5 AG GG AG GG GG
AG GG AG AG AG AG AG AG
GG AG AA AG AG AG AG AG
AA AA AA AA AG GG AG GG
GG GG GG GG GG AG AG
158860939
2604 rs1006011 6 CC AC AC AA AA
AA AC AC AA AC AA AC AC
CC AA AC AA AA AA AA AC
AA AA AA AA AC AA AA AC
AA AA AA AA AA AA AA
53858521
2605 rs1006015 22 AA AA AA AG AG
GG AG GG AG AA AG AG AA
AG AA AG AA AA GG GG GG
AG AG AG AG AG AA AA AA
AA AG AA AG AG GG AG
17722536
2606 rs1006023 7 CC AC AA AA AC
AC AA AC AC AC AA AC AC
AC AC AA AC AC CC AC AC
CC CC CC CC AA CC AC AC
AC AA AA AA AC AC AC
128389554
2607 rs10060246 5 GG GG GG GG GG
GG GG GG GG GG GG GG GG
GG GG GG GG GG GG GG GG
GG GG GG GG GG GG GG GG
GG GG GG GG GG GG GG
43060932

2608 rs10060273 5 GG GG GG AG AG
GG AG GG GG AG GG AG AG
GG GG GG GG GG GG GG GG
AG AG AG AG GG GG GG GG
GG AG AG AA AG GG AA
79618649
2609 rs10060276 5 AA AA AA AA AA
AA AA AA AA AA AA AA AA
AA AA AA AA AA AA AA AA
AA AA AA AA AA AA AA AA
AA AA AA AA AG AA AG
115385416
2610 rs10060307 5 GG GG GG GG GG
GG GG GG GG GG GG GG GG
AG GG GG GG GG GG GG GG
GG GG GG GG GG GG GG GG
GG GG GG GG GG GG GG
53737364
2611 rs10060367 5 GG GG GG AG AG
GG AG GG GG GG AG AG AG
GG GG GG GG GG GG GG GG
GG GG GG GG GG GG GG GG
AG GG -- GG AG AG AG
65859130
2612 rs10060377 5 CC CC CC CC CC
CC CC CC CC CC CC CC CC
CC CC CC CC CC CC CC CC
CC CC CC CC CC CC CC CC
CC CC CC CC CC CC CC
177532036
2613 rs10060413 5 AA AA AA AA AA
AA AA AA AA AA AA AA AA
AA AA AA AA AA AA AA AA
AA AA AA AA AG AG GG AG
AG AA AA AA AA AA AA
110143168
2614 rs10060424 5 AG GG AG AG AA
AA AG AG AG AG AG AG AG
AG AG AA AG AG AG AA AA
AA AA AA AA AA AA AA AA
GG AG AA AA AG AG AA
149114224
2615 rs10060427 5 AG GG AG AG AG
AG AG AA AG AG GG AG GG
GG GG AA AG AG AG AA AA
AA AA AA AA AA GG AG AG
AG AG AG GG AG AG AA
89951112
2616 rs10060431 5 AA AA AA AA AA
AA AA AA AA AA AA AA AA

AA AA AA AA AA AA AA AA
AA AA AA AA AA AA AA AA
AA AA AA AA AA AA AA
35417952
2617 rs1006044 3 GG GG GG GG GG
GG GG AG AG AG AG GG AG
GG AG GG GG GG GG GG GG
GG GG GG GG GG AG GG GG
GG GG GG GG GG GG AG
107757086
2618 rs10060443 5 AA AA AA AA AA
AA AA AA AA AA AG AA AA
AG AG AA AA AA AA AA AA
AA AA AG AA AA AA AA AA
AA AA AA AA AA AA AA
23761700
2619 rs10060487 5 GG GG GG GG GG
GG GG GG GG GG GG GG GG
GG GG GG GG GG GG GG GG
GG GG GG GG GG GG GG GG
GG GG GG GG GG GG GG
33693452
2620 rs10060573 5 AG AG AG AA AA
AA AA AG AG AG AG AG AA
AA AA AG AA AG AA AG AA
AA AG AA AA GG AA AG AG
AA AG AG AA AA AG AA
166087904
2621 rs10060585 5 AA AG AA AA AA
AA AA AA AA AA AA AA AA
AA AA AA AG AG AA AA AA
AG AG AG AG AA AA AA AA
AA AA AA AA AA AG AA
168179043
2622 rs10060587 5 AA AA AA AG AG
AG AA AG AG AG AA AA AA
AG AA AG AG AA AA AA AA
AG AA AA AG AA AG AG AA
AA AA AG AA AA AA AA
53382156
2623 rs10060730 5 GG GG GG AG AG
GG AG AA AG AA GG AG AG
AG AG GG GG GG AG GG AG
AG AG GG AG AG GG AG AG
AG AA AG AG AG GG AG
2207155
2624 rs1006074 6 CC AC AC AC AA
AA AC AC AC AC AA AA AA
AA AA AA AA AA AA AA AA
AA AA AA AA AA AC AC AC

	AA	AA	AA	AA	AC	AC	AA
108006922							
2625	rs10060764	5	AG	AG	AA	GG	GG
	AG	GG	GG	AG	GG	AG	AG	AG
	AG	AG	AA	AA	AG	AG	AG	AG
	AG	AG	GG	GG	AG	AA	AA	AG
	AG	GG	AG	AG	GG	AG	GG	
178369673								
2626	rs10060840	5	AG	GG	AG	GG	GG
	GG	GG	GG	GG	GG	AG	AG	AG
	AG	AG	GG	AG	AG	GG	GG	AG
	GG	GG	GG	GG	GG	AG	GG	GG
	GG	AA	AG	AA	AG	AG	GG	
173094151								
2627	rs10060856	5	AC	AC	AA	AA	AC
	AC	AC	CC	AC	CC	AC	AC	AC
	CC	CC	CC	AC	AC	AA	AA	AA
	CC	CC	AC	CC	AC	AC	AC	CC
	AC	AA	AC	AA	AC	AC	AA	
29729100								
2628	rs1006087	9	AA	AA	AA	AA	AA
	AG	AA	AA	AA	AG	AG	GG	GG
	GG	AG	GG	GG	GG	GG	GG	AG
	AA	AA	AA	AA	AG	GG	GG	AG
	AG	AG	AG	AA	GG	AG	AG	
38235659								
2629	rs10060874	5	AA	AG	AA	AG	GG
	AG	AG	AA	AG	AA	AA	AA	AA
	AA	AA	AG	AG	AA	AG	AG	AG
	AA	AA	AA	AA	AG	AA	AA	AA
	AG	AG	AA	AG	AG	AA	AG	
100582655								
2630	rs1006091	7	AA	AG	AG	AA	AA
	AA	AA	AA	AA	AA	AA	AA	AA
	AA	AA	AG	AA	AA	AA	AA	AA
	AG	AG	GG	AG	AG	AG	AA	AA
	AG	AA	AG	AG	AG	AA	AG	
122542790								
2631	rs10060923	5	CC	CC	AC	CC	CC
	CC	CC	CC	CC	CC	CC	CC	CC
	CC	CC	CC	CC	CC	AC	AC	CC
	AC	AA	AA	AC	AC	AC	AA	AC
	AC	AA	CC	AC	AC	AA	CC	
103011697								
2632	rs1006096	13	GG	AG	AG	AA	AA
	AA	AA	AA	AA	AA	AA	AA	AA
	AA	AG	AG	AG	AA	GG	GG	AG
	AA	AA	AA	AA	AG	AG	GG	AA
	AA	AG	AG	AG	AG	AA	GG	
32189143								

2633 rs1006097 3 AG AG GG AG AG
AG AA AA AA AA AG AG AG
AG AA AA AG AG AG AG AA
AG AG AG GG GG GG GG GG
AG AG AA AG GG AG GG
133411478
2634 rs10060972 5 GG AG AG GG GG
GG GG AG AG GG GG GG GG
AG GG AG AG GG GG GG GG
GG GG GG GG AA AG AG AA
GG GG AA AG AG GG AA
57286285
2635 rs1006098 3 AA AA AA AC AC
AA CC AC AC CC AC AC AC
AC CC AC AA AC AA AA AC
AA AA AC AA AA AA AA AA
AA AA CC AC AA AC AA
133411605
2636 rs10061008 5 GG GG GG AG AG
AG AG AG GG AG AG AG GG
GG GG AG AA AG GG GG AG
AG AG AG AG AG AG GG AG
AG AG GG AG GG GG AG
141744512
2637 rs10061050 5 CC AC AC AC AC
AC CC AA AA AA AC AC AC
AA AA AC AC AC AC AC AC
AC AC AC AC AC AC AC AC
AC AA AC AC CC CC AC
75220355
2638 rs1006111 19 GG GG GG GG GG
AG GG AA AA AA AA AA AA
AG AG AA AA AA AG AG AA
AG AA AG AA AA AG AG AG
AG AA AG AG AG GG AG
52717232
2639 rs10061129 5 CC CC CC CC CC
CC CC CC CC CC CC CC CC
CC CC CC CC CC CC CC CC
CC CC CC CC CC CC CC CC
CC CC CC CC CC CC CC
17939583
2640 rs10061188 5 AA AA AA AA AA
AA AA AA AA AA AG AG AG
AA AA AA AA AA AG AG AA
AA AA AA AA AA AG AA AA
AA AA AA AA AA AA AA
145892296
2641 rs10061247 5 AA AA AA AA AA
AA AA AA AA AA AA AA AA

AA AA AA AA AA AA AA AA
AA AA AA AA AA AA AA AA
AA AA AA AA AA AA AA
15850728
2642 rs10061299 5 GG GG AG AG AG
AG GG AG AG AG GG AG AG
AG GG GG GG GG GG GG GG
AG GG GG AG GG AG AG GG
AG GG GG GG GG GG GG
42419483
2643 rs10061309 5 CC AC AC AC AC
CC AA CC CC CC AA AA AA
AA AA CC AC AC AC CC AA
AC AC AC AC CC AC AC CC
AC AC AC AA AA AC AC
85392336
2644 rs10061327 5 AA AA AA AA AA
AA AA AG AG AG AA AA AA
AA AA AA AG AG AG AA AA
GG GG GG GG AA AA AA AA
AG AA AA AA AG AG AA
172818061
2645 rs10061337 5 AG GG GG GG GG
GG GG AG GG AG AG AA AG
AA GG GG GG GG GG GG GG
AG AG AG AG GG GG GG GG
GG GG GG GG GG GG AG
33769407
2646 rs10061369 5 GG GG AG GG GG
GG GG GG GG GG GG GG AG
GG AG GG GG GG AG GG GG
GG GG GG GG GG GG GG GG
GG AG GG GG GG AG GG
30000585
2647 rs10061385 5 AA AA AA AA AG
AG AA AA AA AA AA AA AG
AA AG AA AA AA AA AA AA
AG AG AG AG AA AA AA AA
AA AG AA AG AA AA AA
18030481
2648 rs1006146 17 AA AA AA AA AA
AA AA AA AA AA AA AA AA
AG AG AA AA AA AA AA AG
AG AA AG AA AG AA AG AA
AG AG AA AA AG AG AG
69360871
2649 rs10061480 5 AA AA AA GG GG
GG GG GG GG GG GG GG AG
AG AG GG AG GG GG GG AG
AA AA AG AG AA AG AG AG

AG AG GG GG AA AG AA
179470930
2650 rs1006154 2 GG GG GG AG GG
AG GG AG AG AG GG AA AG
AG AG AA AA AA AG AG GG
AG AG AG AG AA GG AG AG
GG AG GG AG GG GG AG
80551464
2651 rs10061620 5 AA AA AA AA AA
AA AA AG AA AA AA AG AA
AA AA GG AG AG AA AA AA
AG AG GG GG AG AG GG AG
GG AG AG GG AA AA AG
132633880
2652 rs10061650 5 GG GG AG GG GG
GG GG AG AG AG GG GG GG
GG GG GG GG GG AG GG AA
GG GG GG GG GG GG GG GG
AG GG GG GG GG GG GG
36584881
2653 rs10061701 5 GG GG GG GG GG
GG GG GG GG GG GG GG GG
GG GG GG GG GG GG GG GG
GG GG GG GG GG GG GG GG
GG GG GG GG GG GG GG
2922114
2654 rs1006175 6 GG GG GG GG GG
GG GG GG GG GG GG GG GG
GG GG GG GG GG GG GG GG
GG GG GG GG GG GG GG GG
GG GG GG GG GG GG GG
137754678
2655 rs10061752 5 AA AG AA AG AG
AA AG AG AG AG AA AA AA
AG AG AA AG AG AA AA AA
AG AG AG AG AG AG AG AA
AA AA AA AA AG AA AG
7418630
2656 rs10061803 5 AG AG AG AG AG
GG AA GG AG GG AG AG AG
AG GG GG AG AG AG AG AG
AA AA AG AA AG GG AG AG
GG GG AG AG AG GG AA
13872301
2657 rs10061858 5 AA AA AA AG AG
AA AG AA AA AA AG AG AA
AA AA AG AG AA AA AA AA
AA AA AA AA AG AG GG AG
AG AA AA AA AA AA AA
143464943

2658	rs10061885		5	GG	GG	GG	GG	GG
	GG	GG	AA	AA	AA	GG	GG	GG
	AG	AG	AG	GG	GG	GG	GG	GG
	GG	AG	AG	AG	GG	GG	GG	GG
	GG	GG	GG	GG	GG	GG	GG	
123888331								
2659	rs10061978		5	AC	CC	AC	AC	AC
	AC	AC	CC	CC	CC	AC	AC	AC
	AC	AC	CC	CC	CC	AC	CC	CC
	CC	CC	CC	CC	CC	CC	CC	CC
	CC	AC	AC	AA	CC	CC	CC	
153269843								
2660	rs10061989		5	--	--	--	--	--
	--	--	--	--	--	--	--	--
	--	--	AC	AC	--	AC	AC	AC
	--	--	--	--	AC	--	AC	AC
	--	--	AC	--	--	--	--	
20373617								
2661	rs10062024		5	GG	AG	GG	GG	GG
	GG	GG	GG	GG	GG	GG	GG	AG
	GG	GG	GG	GG	GG	GG	AG	AG
	AG	AG	GG	AG	AG	GG	AG	AG
	AA	AG	AG	AG	AG	GG	AG	
3710645								
2662	rs10062050		5	AG	GG	GG	GG	GG
	AG	GG	AG	AG	GG	GG	GG	GG
	GG	GG	AG	AG	AG	GG	GG	AG
	GG	GG	GG	GG	GG	AG	AG	GG
	AG	AG	GG	GG	AG	GG	AG	
79142384								
2663	rs10062092		5	AA	AA	AA	AA	AA
	AA	AA	AA	AA	AA	AG	AG	AG
	AA	AA	AA	AA	AA	AA	AA	AA
	AA	AA	AA	AA	AA	AA	AA	AA
	AA	AA	AA	AA	AA	AA	AA	
13689874								
2664	rs10062110		5	GG	AG	AG	AG	GG
	AG	GG	GG	GG	GG	AG	AG	GG
	GG	GG	AA	AG	AG	AG	AG	AG
	AG	AA	AG	AA	AG	GG	AG	AG
	AG	AG	GG	AG	AG	AA	AG	
7529177								
2665	rs10062226		5	AG	GG	GG	GG	GG
	GG	GG	GG	GG	GG	GG	GG	GG
	GG	GG	GG	GG	GG	GG	GG	AG
	GG	GG	GG	GG	GG	GG	GG	GG
	GG	GG	GG	GG	GG	GG	GG	
75614737								
2666	rs1006224		1	GG	GG	GG	GG	AG
	AG	GG	AG	AG	AG	GG	GG	AG

	AG	GG	GG	AG	AG	GG	GG	GG
	AG	AA	AG	AA	GG	GG	GG	GG
	GG	AG	AG	AG	AG	GG	AA	
184541602								
2667	rs10062290		5	AA	AA	AA	AA	AA
	AA	AA	AA	AA	AA	AA	AA	AA
	AA	AA	AA	AA	AA	AA	AA	AA
	AA	AA	AA	AA	AA	AA	AA	AA
	AA	AA	AA	AA	AA	AA	AA	
52479904								
2668	rs10062317		5	AC	AC	AC	AA	AA
	AA	AA	AA	AA	AA	AA	AA	AA
	AA	AC	AA	AA	AA	AA	AA	AA
	AA	AA	AA	AA	AA	AA	AA	AA
	AA	AA	AA	AA	AA	AA	AA	
35404922								
2669	rs1006235		1	GG	GG	GG	GG	GG
	GG	GG	GG	GG	GG	GG	GG	GG
	GG	GG	GG	AG	AG	GG	GG	GG
	GG	AG	AG	AG	GG	GG	GG	GG
	GG	GG	AG	GG	GG	GG	GG	
216037545								
2670	rs10062361		5	GG	AG	AG	GG	GG
	AG	GG	AG	AG	AA	AA	GG	AG
	AG	AG	GG	GG	GG	AG	GG	AG
	GG	AG	GG	GG	AA	AG	AA	AG
	AA	GG	AG	AG	GG	GG	GG	
74565153								
2671	rs10062371		5	AA	AA	AA	AG	AA
	AA	AG	AG	AG	AG	AG	AA	AA
	AA	AA	AA	AG	AG	AA	AA	AA
	AG	AG	AA	AA	AA	AA	AA	AA
	AA	AG	AA	AA	AA	AA	AG	
131778413								
2672	rs10062420		5	GG	GG	GG	GG	GG
	GG	GG	GG	GG	GG	GG	GG	GG
	GG	GG	GG	GG	GG	GG	GG	GG
	GG	GG	GG	GG	GG	GG	GG	GG
	GG	GG	GG	GG	GG	GG	GG	
12605306								
2673	rs10062421		5	AA	AA	AA	AA	AA
	AA	AA	AA	AA	AA	AA	AA	AA
	AA	AA	AA	AA	AA	AA	AA	AA
	AA	AA	AA	AA	AG	AG	AG	GG
	AG	AA	AG	AG	AA	AA	AA	
177652884								
2674	rs10062528		5	CC	CC	CC	AC	AA
	AA	AC	AA	AA	AA	AA	AA	AA
	AA	AA	AA	AA	AA	AA	AC	AA
	CC	CC	CC	CC	AA	AC	AA	AA

AC AC AC AA AA AA AC
160150822
2675 rs10062536 5 AG GG AG GG GG
GG GG GG GG GG GG GG GG
GG GG AG AA AG GG GG GG
GG GG GG AG GG AG GG GG
AG GG GG GG GG GG GG
148825669
2676 rs10062538 5 AG AG AG GG GG
GG GG GG GG GG GG GG GG
GG GG GG GG GG GG AG GG
GG GG GG GG AA AA AA AA
GG GG AG GG AA AA AA
39612157
2677 rs10062585 5 GG GG GG AA AA
AG AG GG GG GG AG AG AA
AG AG AG GG GG AG AG GG
GG AG AG GG GG AG GG AG
GG GG AG AG GG GG GG
44289499
2678 rs10062593 5 GG GG GG GG GG
GG GG GG GG GG GG GG GG
GG GG GG GG GG GG GG GG
GG GG GG GG GG GG GG GG
GG GG GG GG GG GG GG
177834023
2679 rs1006260 18 AA AA AG AG AG
GG AA GG AG GG GG GG AG
AG GG GG AG AG AG GG AA
GG AG GG GG AA AG AA AA
GG AG AG AG AG AG AG
19058019
2680 rs10062661 5 AA AA AA AA AA
AA AA GG AG GG AG AG AA
AA AA AA AA AA AA AG AA
AA AA AA AA AG AG AG AA
AG AA AA AA AA AA AA
100065714
2681 rs10062689 5 AA AA AA AA AA
AA AA AA AA AA AA AA AA
AA AA AA AA AA AA AA AA
AA AA AA AA AA AA AA AA
AA AG AA AA AA AG AG
26181001
2682 rs10062747 5 AA AA AA AA AA
AA AA AA AA AA AA AA AA
AA AA AA AA AA AA AA AA
AA AA AA AA AA AA AA AA
AA AA AA AA AA AA AA
23031592

2683	rs10062762		5	AA	AA	AA	CC	AC
	AC	AC	AC	AA	AC	AA	AA	AA
	AA	AA	AC	CC	AC	AC	AA	AA
	AA	AA	AA	AA	AA	AA	AA	AA
	AA	AA	AA	AA	AA	AA	AC	
26770247								
2684	rs10062785		5	AA	AA	AA	AA	AA
	AA	AA	AG	AA	AA	AA	AA	AA
	AA	AA	AA	AA	AA	AA	AA	AA
	AA	AA	AG	AG	AA	AA	AA	AA
	AG	AG	AA	AA	AA	AA	AA	
133186592								
2685	rs1006280		9	AG	AG	AG	AA	AA
	AA	AA	AA	AA	AA	AA	AA	AA
	AA	AA	AA	AA	AA	AG	AG	AG
	AA	AG	AA	AG	AG	AG	AA	AG
	AG	AA	AA	AA	AA	AA	AA	
78529571								
2686	rs10062813		5	AG	AG	AA	GG	GG
	AG	GG	AA	AA	AA	AG	AG	AG
	AG	AG	GG	AG	GG	AG	GG	AG
	AG	AG	AG	AG	AA	GG	AG	AG
	AG	AG	GG	GG	GG	GG	AG	
77579205								
2687	rs1006282		X	AA	AA	AA	GG	AA
	GG	AG	AG	AG	GG	AA	AA	AA
	AA	AA	AA	AA	AA	AA	AA	AA
	AA	AA	GG	AA	AG	AA	AG	GG
	AG	AG	AA	AA	AG	AA	GG	
142420057								
2688	rs10062839		5	AA	AA	AA	AG	AG
	AA	AG	AA	AA	AG	AA	AA	AA
	AA	AA	AA	AA	AA	AG	AG	GG
	AG	AG	AG	AG	AG	AA	AA	AG
	AA	GG	AG	AG	AA	AA	AG	
60103452								
2689	rs10062847		5	AA	AA	AA	AA	AA
	AG	AA	AA	AA	AA	AA	AA	AA
	AA	AA	AG	AA	--	AA	AA	AA
	GG	GG	AG	AG	AA	AA	AA	AA
	AA	AG	AG	AG	AG	GG	AA	
178179729								
2690	rs1006286		10	AC	AA	AA	AA	AA
	AA	AA	AA	AA	AA	AC	AC	AA
	AC	AC	AA	AC	AA	AA	AA	AA
	AC	AC	AA	AA	AA	AA	AA	AA
	AA	AA	AA	AA	AA	AA	AA	
119519010								
2691	rs10062872		5	GG	GG	GG	GG	GG
	GG	GG	GG	GG	GG	GG	GG	GG

GG GG GG GG GG GG GG GG
GG GG GG GG GG GG GG GG
GG GG GG GG GG GG GG
168683533
2692 rs10062990 5 AA AA AA AG AG
GG AG AA AA AA GG AG AG
AG AA AG AG AA AG AG AG
AA AA AA AA AG GG AG GG
AG AG AA AG AA AA AA
33685863
2693 rs10062993 5 GG AG GG GG GG
GG GG AG AG AG AG AG GG
GG GG GG GG GG GG AG GG
GG GG GG GG GG GG GG GG
AG AG GG GG GG GG AG
163221209
2694 rs10062995 5 AG AG GG GG GG
GG GG GG GG GG AA AG AG
AG AG AG AA AG GG GG GG
GG GG GG GG GG GG GG GG
GG GG GG GG AG GG AG
25412743
2695 rs10063005 5 AG GG AG AA AA
AA AA AA AA AA AA AG AA
AA AA AA AA AA AG AG AA
AG AG AG AG AA AG AG AA
AA AG AA AA AA AA AA
34576037
2696 rs10063061
5 -- -- -- -- -- -- -- --
-- -- -- -- -- -- -- --
-- -- -- -- -- -- -- --
-- -- -- -- -- -- -- --
-- -- -- -- 125943743
2697 rs10063082 5 AA AA AA AA AA
AA AA AA AA AA AA AA AA
AA AA AA AA AA AA AA AA
AA AA AA AA AA AA AA AA
AA AA AG AG AA AA AA
24229197
2698 rs10063181 5 AG AG AG AG AG
AG AA GG GG GG AA AA AG
AG AG AG GG -- AA AA AA
AG AG AG AG AG AG AG AG
AG GG GG GG GG GG GG
35275087
2699 rs10063242 5 AG AG AG AG AA
AG AA AA AA AA AG AG AG
AA AA AG AG AA AG AG AG
GG GG GG GG AG AG GG AG

AG AG AG AG AG AG AG
157037412
2700 rs10063261 5 AA AA AA AA AA
AA AA AA AA AA AA AA AA
AA AA AA AA AA AA AA AA
AA AA AA AA AA AA AA AA
AA AA AA AA AA AA AA
13253698
2701 rs10063277 5 AA AA AA AA AA
AA AA AA AA AA AA AA AA
AA AA AA AA AA AA AA AA
AA AA AA AA AA AA AA AA
AA AA AA AA AA AA AA
126834286
2702 rs10063286 5 AA AA AA AA AA
AA AA AA AA AA AA AA AA
AA AA AA AA AA AA AA AA
AA AA AA AA AG AA AA AG
AA AA AA AA AA AA AA
174878467
2703 rs10063294 5 AG AA AG GG GG
AG AG AG AG AG GG AG AG
AG AA AG GG AG AG AG AG
GG GG GG GG AA AG AA AG
AA AG AA AG AG AG GG
35877505
2704 rs1006333 8 AA AG AA GG GG
GG GG GG GG AG GG GG GG
GG GG AG AG GG GG AG AG
GG GG AG GG AG GG GG AG
GG AG GG AG AA AG AG
22797597
2705 rs10063376 5 AT AT AT TT AT
TT AT AT AT AT AA AA AA
AT AT AT AT AT AA AT AT
AT AA AA AT AT AT TT AA
TT AA AA AA AT AT AT
42184473
2706 rs10063386 5 AA AA AA AA AG
AA AG AA AG AA AG AG GG
GG GG AG AA AG AG AG AG
AG AA AA AG AG AA AA AA
AA AA AG AA AG AG AA
107288619
2707 rs10063429 5 GG GG GG GG GG
GG GG GG GG GG AG GG AG
AG AG GG AG AG AG GG AG
GG GG GG GG AG GG AG AG
GG GG GG GG GG GG GG
87870166

2708 rs10063455 5 GG GG GG GG GG
GG GG GG GG GG GG GG GG
GG GG GG GG GG GG GG GG
GG GG GG GG GG GG GG GG
GG GG GG GG GG GG GG
169639751
2709 rs10063526 5 AA AA AA GG GG
AG AG AG AG AG AG AG AG
AG AG AA AG AG AA AA AA
AG AG AG AG AG AG GG AG
AG AG AA AA AA AA AA
83145042
2710 rs10063559 5 GG GG GG AA AA
AG AA AG GG AG AG AG AA
AA AG AG GG GG GG GG GG
AA AA AA AA GG AG GG AG
GG GG GG GG GG GG GG
44244036
2711 rs1006364 8 CC CC CC CC CC
CC CC CC CC CC CC CC CC
CC CC AC AA CC AC CC CC
CC CC CC CC CC AC CC CC
AC CC CC CC CC CC CC
88934441
2712 rs10063763 5 AG GG AG GG GG
GG GG GG GG GG GG AG AG
GG GG AG AG AG AG GG AG
AG AG AG AG GG AG AG GG
GG AG GG GG GG GG GG
124704493
2713 rs10063808 5 AA AA AA AA AA
AC AA AC AC AC AA AA AA
AA AA AA AA AA AA AA AC
AC AC AA AC CC AA AC AC
AA AC AA AC AA AA AA
2087916
2714 rs10063817 5 AA AA AA AA AA
AA AA AA AA AA AG AG AG
AA AA AG AG AA AA AG AG
AA AA AA AA AA AA AA AA
AA AA AA AA AA AA AA
170798093
2715 rs10063826 5 GG AG AG GG GG
GG GG GG GG GG GG GG GG
AG AG GG GG GG GG GG GG
GG GG GG GG GG GG GG GG
GG GG AA AG GG GG GG
172004039
2716 rs10063866 5 GG GG AG GG AG
AG AG AG AG AG AG AG AG

	AG	AG	AG	GG	AG	AG	AG	AG
	AA	AG	AG	AA	AG	GG	GG	GG
	GG	AG	AG	AG	AG	AA	GG	
107504326								
2717	rs1006389		2	AA	AG	AA	AA	AA
	AA	AA	AG	AG	GG	AA	AA	AA
	AA	AG	AA	AA	AA	AG	AA	AA
	AG	AG	AG	AA	AA	GG	AG	AG
	AA	AA	AA	AA	AA	AA	AA	
207843712								
2718	rs10063962		5	AA	AC	AC	AC	CC
	AC	AC	AC	AC	AC	AC	AC	CC
	AA	AA	AA	AC	AC	AC	CC	AC
	AC	AC	AA	AA	AA	AC	AC	AA
	AA	CC	AC	CC	CC	AC	AC	
3271982								
2719	rs10063964		5	AG	AG	AA	AG	AG
	AG	AG	AG	AG	GG	AG	GG	AG
	AG	AA	AG	AA	AG	GG	AG	AG
	AG	AG	AG	AG	AA	AA	AA	AA
	AA	GG	AG	GG	AG	GG	AG	
55371201								
2720	rs10063982		5	GG	AG	AG	GG	GG
	AG	AG	AG	AG	AG	AG	AG	AG
	AG	AG	AG	AG	AG	AA	AA	AA
	AG	AG	AG	AG	AG	AG	GG	AG
	AG	GG	AA	AG	AG	AG	AG	
83661128								
2721	rs10063997		5	AG	AG	AA	AA	AA
	AA	AA	AA	AA	AA	AG	AA	AG
	AG	AG	AA	AA	AA	AA	AA	AA
	AA	AA	AA	AA	AA	AA	AA	AA
	AA	AA	AA	AA	AG	GG	AA	
65917185								
2722	rs1006405		14	AG	AA	AA	AA	AA
	AA	AA	AA	AA	AA	AG	AA	AA
	AG	GG	AG	AG	AG	GG	AG	AG
	AA	AA	AA	AA	AG	AG	AG	GG
	GG	AA	AA	AA	GG	AG	AG	
81809443								
2723	rs1006407		22	AG	AA	AA	AG	AG
	AG	AG	AA	AG	AG	AG	AG	AA
	AA	AA	AA	AA	AA	AA	AA	AA
	AA	AA	AA	AA	AG	AA	AA	AA
	AA	AA	AA	AA	AA	AA	AA	
41972032								
2724	rs1006409		12	AA	AA	AA	AA	AG
	AG	AA	AG	AG	GG	GG	AG	AG
	AA	AA	GG	AG	AG	AG	AG	AA
	GG	GG	AG	GG	GG	AG	GG	GG

AA AA AG AA AA AG AA
29435675
2725 rs1006412 13 GG AG GG AG AG
AG AG AA AA AG AG AG AG
AG AG AG AG AG GG GG GG
AG AG AG AG AG AG GG AG
AG AG AG GG AG AG GG
76218076
2726 rs1006413 2 AG AG AG AG GG
AG AG AA AA AG AG AA AG
AA AA AA AG AG GG GG GG
AA AG AA AA AG AA AA AG
AA AG GG GG GG GG GG
107400636
2727 rs10064169 5 GG GG GG GG GG
GG GG GG GG GG GG GG GG
GG GG GG GG GG GG GG GG
GG GG GG GG GG GG GG GG
GG GG GG GG GG GG GG
44949992
2728 rs10064177 5 AA AA AA AA AA
AA AG AG GG AG AA AA AG
AA AA GG AG AG AG GG AA
AA AA AA AA GG AG GG AG
AG AA AA AA AA AG AG
4439260
2729 rs1006421 2 AA AA AA AA AA
AA AA AA AA AA AA AA AA
AA AA AA AA AA AA AA AA
AA AA AA AA AA AA AA AA
AA AA AA AA AA AA AA
6914546
2730 rs10064215 5 AA AA AA GG GG
GG GG AA AA AA GG GG GG
GG GG AA AG AG GG AG AG
GG GG GG GG AG GG AG AG
GG AG GG GG AA AA AG
4547704
2731 rs10064229 5 CC CC CC CC CC
CC CC AC AC AC CC CC CC
CC CC CC CC CC CC AC CC
AC AC AC AC AC CC CC AC
CC AC AC AC CC CC CC
176779857
2732 rs10064279 5 GG AG GG AG AA
AG AG AG AG AG AG AG AG
AA AA GG GG GG AG GG AA
AG AA AG AA AG GG GG GG
AG AA GG AG AG GG AA
5608058

2733 rs10064298 5 AG GG GG AG GG
AG AG GG GG GG GG GG GG
GG GG GG GG GG GG GG GG
GG GG GG GG GG GG GG GG
AG GG GG GG AG GG AG
41901890
2734 rs10064312 5 AG AG AG AA AA
AG AG AA AA AA GG AG GG
GG GG AA AA AA AA AA AG
AG AG AG AG AA AG AG AA
AA AA AA AA AA AG AA
94519501
2735 rs10064315 5 GG AG AA AG AG
AG GG AG AG AG GG AA AG
GG GG GG GG GG AG GG AG
AG AA AG AG AA AG AA AG
AG AA AG AA AG AG AA
68245459
2736 rs10064373 5 AG AG GG AG AG
AG AG AA AA AG AG AA AA
AA AA GG AG AG AA AA AG
GG AG AG GG AG AG GG AA
AG AA AA AA AA AA AA
60857714
2737 rs10064379 5 GG GG GG GG GG
GG GG GG GG GG GG GG GG
GG GG GG GG GG GG GG GG
GG GG GG GG GG GG GG GG
GG GG GG GG GG GG GG
25152634
2738 rs10064416 5 GG GG GG GG GG
GG GG GG GG GG GG GG GG
GG GG GG GG GG GG GG GG
GG GG GG GG GG AG GG AG
GG GG GG GG GG GG GG
72809050
2739 rs10064439 5 GG GG AG GG GG
GG GG GG GG GG AG GG AG
GG AG GG GG GG AG GG AG
GG GG GG GG GG GG GG GG
AG GG GG GG GG GG GG
59233819
2740 rs10064495 5 AA AA AA AA AA
AG AG AA AG AA AG AG AG
AG AG AA AA AA AG AG AG
GG GG GG GG AA AG AG AA
AG AG AA AA AG GG AA
35223013
2741 rs10064581 5 AA AG AG AA AA
AA AA AA AA AA AA AA AA

AA AA AA AA AA AA AA AA
AA AA AG AA AG AG AG AA
AG AA AA AA AA AA AA
18726660
2742 rs10064609 5 AC CC AC CC CC
CC AC AC AC AA AC CC CC
AC AC AC AC AC AC AC AC
CC CC CC CC AC AC AC CC
AA AC AC AC AC AC AC
77278872
2743 rs10064613 5 CC CC CC CC CC
CC CC CC CC AC CC CC CC
CC CC CC AC AC CC CC CC
AC AC CC AC CC CC CC CC
CC CC CC CC AC CC AC
11926837
2744 rs10064661 5 AC AA AC AC CC
CC AC CC CC CC AC AC AC
CC CC AA AA AA CC AC AC
AC AC AC AC CC AC CC CC
AC AA AC AC CC CC CC
164668926
2745 rs10064701 5 GG GG GG GG AG
GG AG GG GG GG GG GG GG
GG GG GG GG GG GG GG GG
GG GG GG GG GG GG GG GG
GG GG GG GG GG GG GG
167168733
2746 rs1006472 2 AC AA AA AA AA
AA AA AA AA AC AA AA AC
AA AA AA AC AC AA AC AA
AA AC AA AA AA AA AA AA
AA AC AA AC CC AC AC
107329516
2747 rs10064783 5 CC CC CC CC CC
CC CC CC CC CC CC CC CC
CC CC CC CC CC CC CC CC
CC CC CC CC CC CC CC CC
CC CC CC CC CC CC CC
118698583
2748 rs10064808 5 GG GG GG GG GG
GG GG GG GG GG GG GG GG
GG GG AG AG GG GG GG GG
GG GG GG GG GG GG GG GG
GG AG GG GG AG AG GG
170017162
2749 rs10064813 5 AA AA AA AC CC
AC AC AA AA AA AC AC AC
AC AC AA AA AA AA AA AA
AA AA AA AA AC AA AA AA

AC CC AA AC AA AA AA
12268607
2750 rs10064824 5 AA AA AG AA AA
AA AA AG AG AG AA AG AG
AA AA AG AA AA AA AG AA
AA AA AA AA AG AA AA AA
AG AG AG GG AA AG AA
76638632
2751 rs10064933 5 GG AG AG GG GG
GG GG GG GG GG GG GG GG
GG GG GG GG GG GG GG GG
GG GG GG GG GG GG GG GG
GG GG GG GG GG GG GG
58140538
2752 rs1006494 6 AC AA AC CC CC
CC CC AC AC AC AA AA AC
AC AC CC CC AC CC CC CC
AC AC AC AC AC AC AC AC
AA CC AC CC CC CC AC
102985467
2753 rs10064949 5 AA AC AA AA AA
AC AC AC AA AC AA AA AA
AA AC AC AC AC AC AC AC
AC AC AC AC AC AA AA AC
AC AC CC CC AC AC AC
51484497
2754 rs10064968 5 GG GG GG GG GG
GG GG GG GG GG GG GG GG
GG GG GG AG AG GG GG GG
GG GG GG GG AG GG AG GG
AG AG GG GG GG AG AG
159693842
2755 rs1006498 9 GG GG GG GG GG
GG GG GG GG GG GG GG GG
GG GG GG GG GG GG GG GG
GG GG GG GG GG GG GG GG
GG GG GG GG AG AG GG
140514826
2756 rs10064989 5 GG GG GG GG GG
GG GG GG GG GG GG GG GG
GG GG GG GG GG GG GG GG
GG GG GG GG GG GG GG GG
GG GG GG GG GG GG GG
41866821
2757 rs10065018 5 AC CC AC AC AC
AC CC CC CC CC CC CC CC
CC CC AC AA CC CC CC CC
CC CC CC CC CC CC CC CC
CC CC CC CC AC AC AA
180027277

2758 rs10065027 5 GG GG GG GG GG
GG GG AG AG AG GG GG GG
GG GG AG AG GG GG GG GG
GG GG GG GG GG AG AG GG
GG GG GG GG GG GG GG
21401035
2759 rs10065043 5 AG AG AG AG AA
AG AG GG GG GG GG GG GG
GG GG GG AG AG GG GG GG
GG GG GG GG GG GG GG GG
GG GG GG GG AG AG GG
167169182
2760 rs1006507 7 AG AG GG AG AG
AG AG AG AG AG AA AA AA
AA AA AG AG AG AA AA AA
AG AG AA AG AG AG AA AA
AG AA AG AG AG AG AA
100814034
2761 rs10065090 5 AG AA AA AA AA
AA AA AA AA AA AA AA AA
AA AA AA AA AA AA AA AG
AA AA AA AA AG AG AA AG
AA AA AG AG AA AA AA
18433482
2762 rs1006514 7 GG AG GG AG GG
AG GG GG GG GG AG AG GG
GG GG GG GG AG GG GG GG
AG GG GG AG GG GG GG GG
GG GG GG GG GG GG GG
28961321
2763 rs10065187 5 AA AG AG GG GG
GG GG GG GG GG GG GG GG
AG AG GG GG GG AG GG GG
AG AA AG AG GG GG GG GG
GG GG GG GG GG GG GG
173951543
2764 rs10065214 5 AA AA AA AA AA
AA AA AA AA AA AA AA AA
AA AA AA AA AA AA AA AA
AA AA AA AA AA AA AA AA
AA AA AA AA AA AA AA
32996658
2765 rs10065220 5 AG AG AG AG AG
GG AA AA AG AA AA AG AA
AA AA AA AG AG AG AG AA
AG AG AG AG AG AG GG AG
GG GG AA AG AA AA AG
15384680
2766 rs10065267 5 AA AA AC AA AA
AA AA AA AA AA AA AA AA

AA AA AC AC AC AC AA AA
AA AA AA AA AC AA AA AA
AA AA AA AA AA AC AA
89525428
2767 rs10065343 5 GG GG GG GG AG
AG GG AG AG AG GG GG AG
GG AG AG GG AG GG GG GG
GG GG GG GG GG GG GG GG
GG GG AG GG GG GG GG
25120430
2768 rs10065416 5 AA AA AA AA AA
AA AA AA AA AA AA AA AA
AA AA AA AA AA AA AA AA
AA AA AA AA AA AA AA AA
AA AA AA AA AA AA AA
124372189
2769 rs1006547 16 AG GG AG AG AG
AG AA AG AA GG AG AG AA
GG GG AG AG AG AA AG AA
AA AA AA AA AG AG AA AG
GG AG AG AA AG AG AG
89844229
2770 rs10065544 5 AA AG AA AA AA
AA AA GG AG GG AA AA AA
AA AA AG AA AG AG AA AG
AA AA AA AA AA AA AA AA
AG GG AA AG AG AA AG
136564577
2771 rs10065575
5 -- -- -- -- -- -- -- --
-- -- -- -- -- -- -- --
-- -- -- -- -- -- -- --
-- -- -- -- -- -- -- --
-- -- -- -- 86714840
2772 rs10065637 5 AG AG GG GG GG
GG GG AG AG AG AG GG AG
GG AG GG GG GG GG GG GG
AG GG GG AG AG GG AG GG
GG AG GG GG GG GG GG
55438851
2773 rs1006564 12 GG GG GG GG GG
GG GG AG GG GG GG AG AG
AG AG GG AG AG GG GG GG
AG AG AG AG GG GG GG GG
GG GG GG GG AG GG AG
2668570
2774 rs1006566
17 -- -- -- -- -- -- -- --
-- -- -- -- -- -- -- --
-- -- -- -- -- -- -- --

	--	--	--	--	--	--	--	--
	--	--	--	--	14484582			
2775	rs10065689		5	AA	AA	AA	AA	AG
	AA	AG	AG	GG	AG	GG	GG	GG
	GG	GG	AA	AA	AA	AG	AG	AG
	AG	GG	GG	AG	AA	AG	AA	AG
	AG	GG	AA	AG	AA	AA	AA	
43604258								
2776	rs10065784		5	AA	AA	AA	AG	AG
	AG	AA	AA	AA	AA	AA	AA	AA
	AA	AG	AG	AA	AG	AG	AG	GG
	AG	AG	AG	AG	AA	AA	AA	AA
	AG	AA	AA	AA	AG	GG	AA	
53802693								
2777	rs10065790		5	GG	GG	GG	GG	GG
	GG	GG	GG	GG	GG	AG	GG	GG
	GG	GG	AG	AG	GG	GG	AG	GG
	GG	GG	GG	GG	GG	GG	GG	GG
	GG	GG	GG	GG	GG	GG	GG	
112911165								
2778	rs10065813		5	AG	AA	AG	GG	GG
	GG	GG	AG	GG	GG	GG	GG	GG
	GG	GG	AA	AG	AG	GG	GG	AG
	AA	AA	AA	AA	AG	GG	AG	GG
	AG	AG	AG	GG	GG	GG	GG	
152940405								
2779	rs10065828		5	AA	AA	AA	AC	AC
	AA	AC	AC	AC	AC	AC	AC	AA
	AC	AC	AA	AA	AA	AC	AC	AC
	AA	AC	AA	AA	AC	AA	AC	AA
	AC	AC	AC	CC	AC	AA	AC	
169992533								
2780	rs1006583		8	AA	AA	AA	AA	AC
	AC	AA	AC	AC	AC	CC	AC	CC
	CC	CC	AC	AA	AC	AA	AC	AC
	CC	AA	AC	AC	CC	AA	AC	AC
	AA	AC	AA	AA	AC	AA	AC	
4308969								
2781	rs10065847		5	AA	AA	AG	GG	GG
	AG	GG	AA	AA	AA	GG	AG	AG
	AG	GG	AG	AG	AG	AG	GG	AG
	AG	GG	GG	AG	AG	AG	GG	AA
	GG	AG	AG	GG	GG	GG	AG	
39094187								
2782	rs10065849		5	GG	GG	GG	GG	GG
	GG	GG	GG	GG	GG	GG	GG	GG
	GG	GG	GG	GG	GG	GG	GG	GG
	GG	GG	GG	GG	GG	GG	GG	GG
	GG	GG	GG	GG	GG	GG	GG	
30592365								

2783	rs1006586		11	AG	AG	GG	GG	GG
	GG	GG	AG	GG	GG	GG	GG	GG
	GG	GG	GG	AG	GG	GG	GG	AG
	GG	GG	GG	GG	AG	GG	AG	AG
	AG	GG	GG	GG	GG	GG	GG	
86226645								
2784	rs10065860		5	CC	CC	CC	CC	CC
	CC	CC	CC	CC	CC	CC	CC	CC
	CC	CC	CC	CC	CC	CC	CC	CC
	CC	CC	CC	CC	CC	CC	CC	CC
	CC	CC	CC	CC	CC	CC	CC	
23331411								
2785	rs10065876							
5	--	--	--	--	--	--	--	--
	--	--	--	--	--	--	--	--
	--	--	--	--	--	--	--	--
	--	--	--	--	--	--	--	--
	--	--	--	--	3657787			
2786	rs10065906		5	AC	AC	AC	AA	AC
	AC	AC	AC	AC	AC	AA	AA	AA
	AC	AC	AC	CC	AC	AA	AC	AC
	AC	AC	AC	AC	AC	AA	AA	AC
	AA	AA	AA	AA	AC	AA	AC	
159007778								
2787	rs10065943		5	GG	GG	GG	GG	GG
	GG	GG	GG	GG	GG	GG	GG	GG
	AA	AG	GG	GG	GG	GG	GG	AG
	GG	AG	GG	GG	GG	GG	GG	GG
	GG	GG	GG	GG	GG	GG	GG	
67158729								
2788	rs10065959		5	AG	GG	GG	AA	AA
	AA	AA	GG	AG	GG	GG	GG	GG
	AG	AG	AG	GG	AG	AA	AG	AG
	AG	AG	AG	AG	AG	GG	GG	GG
	GG	AG	AA	AG	AA	AA	AG	
9956601								
2789	rs10065963		5	GG	GG	GG	GG	GG
	GG	GG	GG	GG	GG	GG	GG	GG
	GG	GG	AG	AG	AG	GG	GG	GG
	AG	GG	GG	GG	GG	AG	AG	GG
	GG	GG	GG	GG	GG	GG	AG	
120776767								
2790	rs1006598		1	CC	CC	CC	CC	CC
	CC	CC	AA	AC	AA	CC	CC	CC
	AC	CC	CC	CC	CC	AC	AC	AA
	AC	CC	CC	AC	CC	CC	CC	CC
	AC	CC	CC	CC	CC	AC	CC	
87875774								
2791	rs10065995		5	AA	AG	AG	AG	AA
	AG	--	AG	--	AG	AA	AA	AA

AA AA AG AA AG AA AA AA
AG AG -- AG -- -- -- AA
-- -- AG -- AG AG AG
10215009
2792 rs10065996 5 GG GG GG AG AG
AG GG GG GG GG GG GG GG
GG GG GG AG AG GG GG GG
GG GG GG GG AG GG GG GG
GG GG GG GG GG GG GG
134404390
2793 rs10066012 5 GG GG GG GG GG
GG GG GG GG GG GG GG GG
GG GG GG GG GG GG GG GG
GG GG GG GG GG GG GG GG
GG GG GG GG GG GG GG
55285740
2794 rs10066037 5 AG GG GG GG GG
GG GG AG AG AG AA GG AG
AG AG GG GG GG AG GG AA
AG AG AG AG GG GG GG GG
AG AG GG GG GG GG AG
76554598
2795 rs10066195 5 AA AA AA AA AA
AA AA AA AA AA AA AA AA
AA AA AA AA AA AA AA AA
AA AA AA AA AA AA AA AA
AA AA AA AA AA AA AA
19134838
2796 rs10066216 5 CC CC CC CC CC
CC CC CC CC CC CC CC CC
CC CC CC CC CC CC CC CC
CC CC CC CC CC CC CC CC
CC CC CC CC CC CC CC
180190328
2797 rs10066223 5 AG GG AG AA AG
AG AA AA AA AA GG GG GG
GG GG AA AG AG GG AG GG
AG AG AG AG GG GG GG GG
AG AG AG AG AG AG GG
154499047
2798 rs10066234 5 AA AA AA AA AA
AA AA AA AA AA AG AA AA
AG AG AA AA AA AA AA AA
AA AA AG AA AA AA AA AA
AA AA AA AA AA AA AA
23823932
2799 rs10066309 5 AA AA AA AA AA
AA AA AA AA AA AA AA AA
AA AA AA AA AA AA AA AA
AA AA AA AA AA AA AA AA

AA AA AA AA AA AA AA
23049365
2800 rs10066380 5 AA AG AA AG AG
GG AA AG AG AG AA AA AA
AG AG AG GG GG AG AG AG
AG AG AG AG AA AG AA AG
AA AA GG AG GG AG GG
118653933
2801 rs10066454 5 AA AG AG AA AA
AG AG AA AA AA AA AG AA
AA AA AG AG AA AG AG AA
AG AG AG AG AG AA AG AA
AG AA AA AA AA AG AG
55920862
2802 rs10066481 5 AA AA AA AG AG
AG AA AA AA AA AA AA AA
AA AA AA AA AA AA AA AA
AA AA AA AA AA AA AA AA
AA AA AA AA AA AA AA
43252694
2803 rs10066509 5 CC CC CC CC CC
CC CC CC AC CC CC CC CC
CC CC CC CC CC CC CC CC
CC CC CC CC CC CC CC CC
CC CC CC CC CC CC CC
45377015
2804 rs10066518 5 GG AG AA AG AG
AA AG AA AA AA AG AG AG
AA AA AA AA AA AG AA AA
AG AA AG AA AA AA AA AA
AA AA GG AG AA AA AA
7518855
2805 rs10066621 5 AA AA AA AG AG
AG AA AG AG GG AA AG AA
AA AA AA AA AA AG AA AG
AG AA AG AG AA AA AA AA
AA AG GG AG AA AA AA
62416806
2806 rs10066628 5 AG AG AG AG AG
AA GG AA AA AA AG AA AA
AA AA AG AG GG AA AA AG
AG AG AG AG AG AG GG AA
AG AA AA AA AA AG AA
62416834
2807 rs10066638 5 AA AA AA AG AA
AA AG AG AG AG AG AA AG
AA AA AG AG AG AG AG GG
AA AA AA AA AA AA AA AA
GG AG AA AA AA AG AA
122209192

2808 rs10066702 5 AC AC AC CC CC
CC CC AC AC CC CC CC CC
AC AC AA AC AC AC AC AC
AC AC AC AC CC CC CC CC
CC CC CC CC CC CC AC
76296410
2809 rs10066734 5 GG GG GG GG GG
GG GG GG GG GG GG GG GG
GG GG GG GG GG GG GG GG
GG GG GG GG GG GG GG GG
GG GG GG GG GG GG GG
36875575
2810 rs10066754 5 GG GG GG GG GG
GG GG GG GG GG GG GG GG
GG GG GG GG GG GG AG GG
GG GG GG GG GG GG GG GG
GG GG AG AG GG GG GG
54774169
2811 rs10066767 5 AA AC AA AA AA
AA AA AA AA AA AA AA AA
AC AC AA AA AA AA AA AA
AA AA AA AA AA AA AA AA
AA AA AA AA AA AA AA
165909103
2812 rs1006678 10 GG GG GG AG AA
AA AG AG GG GG AG AG GG
AG AG GG GG GG AG AG GG
AG AG GG AG AA GG AG AG
AA GG AG AG AA AA AG
54882943
2813 rs10066782 5 AA AA AA AA AA
AA AA AA AA AA AA AA AA
AA AA AA AA AA AA AA AA
AA AA AA AA AA AA AA AA
AA AA AA AA AA AA AA
158432118
2814 rs10066885 5 GG GG AG GG GG
GG AG AG AG AG GG GG GG
GG GG GG GG GG AG AG AG
GG GG GG GG GG AG AG GG
GG AG AG AG AG AG GG
23296872
2815 rs10066888 5 AA AA AA AG AG
AA GG AG AG AG AG AG AG
AG AG AG GG AA AA AA AA
AA AA AG AG AA AG AG AG
GG AG AG AG AG AA GG
178172559
2816 rs10066903 5 GG GG GG GG GG
GG AG AG AG AG GG GG AG

	GG	AG	AG	AG	AA	AA	AG	AA
	AG	AG	AG	AG	GG	AG	AG	GG
	GG	AA	GG	AG	AG	GG	AG	
20401352								
2817	rs1006694		15	AA	AA	AA	AC	AA
	AC	AA	AA	AC	CC	AA	AA	AA
	AA	AA	AA	AC	AC	AA	AA	AA
	CC	AC	AC	AC	AC	AC	CC	AA
	AA	AA	AA	AA	CC	AC	AC	
93848297								
2818	rs1006697		10	AA	AA	AA	AA	AA
	AA	AA	AA	AA	AA	AA	AA	AA
	AA	AA	AA	AA	AA	AA	AA	AA
	AA	AA	AA	AA	AA	AA	AA	AA
	AA	AA	AA	AA	AA	AA	AA	
119318233								
2819	rs10066990		5	GG	GG	GG	AG	AG
	AG	GG	AG	AG	AG	GG	GG	AG
	AG	AA	GG	AG	AG	AG	AA	GG
	GG	GG	GG	GG	GG	AG	GG	AG
	GG	AG	AG	AA	GG	AG	AG	
34509825								
2820	rs10067022		5	AA	AA	AG	AA	AA
	AA	AA	AA	AA	AA	AA	AA	AA
	AG	AA	AA	AA	AA	AA	AA	AA
	AA	AA	AA	AA	AA	AA	AA	AA
	AA	AA	AA	AA	AA	AA	AA	
51277529								
2821	rs10067047		5	AA	AA	AA	AG	AG
	GG	AA	AA	AA	AA	AG	AG	AG
	AA	AA	AG	GG	AG	GG	GG	AG
	AG	AG	AG	AG	AA	AA	AA	AA
	AG	AA	GG	AG	AG	AA	GG	
148623098								
2822	rs10067065		5	AA	AA	AA	AG	AA
	AG	AG	AG	AG	AG	AG	AG	AG
	AG	AG	GG	AG	AG	AA	AG	AG
	AA	AA	AA	AA	AG	GG	AG	GG
	AA	AA	AA	AA	AA	AA	AG	
123811602								
2823	rs10067066		5	AA	AA	AA	AA	AA
	AA	AA	AG	AA	AG	AA	AA	AA
	AA	AA	AA	AA	AA	AA	AA	AA
	AA	AA	AA	AA	AA	AA	AA	AA
	AA	AG	AA	AG	AA	AA	AA	
31446716								
2824	rs10067069		5	AG	AG	AG	AA	AG
	AG	AG	AA	AG	AA	AG	AA	AA
	AA	AA	GG	AG	AG	AG	AG	AG
	AG	AG	AG	AG	AG	AG	AG	AA

AG AG GG GG AG AG AG
107420876
2825 rs10067113 5 AA AG AG AG AG
AA AG AG GG AG AG AG AA
AG AG AA AA AA GG AG AG
AA AA AG AA AA AA AA AA
AG AG AA AG AG AA AG
26016363
2826 rs10067148 5 AG GG GG GG GG
GG AG AG GG AG AG AA AG
GG GG AG GG AG AG AG AG
AA AA AG AA AG GG AG AG
GG GG GG GG AG AG AG
28112135
2827 rs10067160 5 GG AG GG GG GG
GG GG GG GG GG GG GG GG
GG GG GG GG GG GG GG GG
AG AG AG AG AG GG GG GG
AG GG GG GG AG AA GG
134623699
2828 rs1006718 11 GG GG GG GG GG
GG GG GG AG AG GG GG GG
GG GG GG GG GG AG AG AG
GG AG GG AG GG GG GG GG
GG GG GG GG AG GG AG
69959183
2829 rs10067187 5 GG GG AG AG AG
AG GG AG AG AG GG GG GG
AG GG AG AA AG GG GG GG
AG GG GG AG GG AG AG GG
AG GG AG GG GG GG GG
53364068
2830 rs10067201 5 AG GG AG AG AG
AA AG AA AA AG AG AA AG
AG AG AA AG AG AA AA AG
AG AG AG AG GG AG GG AG
AG AG AG AG AG AG AG
60867365
2831 rs10067270 5 AA AA AA AA AA
AA AA AA AA AA AA AA AA
AA AA AA AA AA AA AA AA
AA AA AA AA AA AA AA AA
AA AA AA AA AA AA AA
22053668
2832 rs10067281 5 AG AA AA AG AG
AG GG AG AA AG GG AG AG
AA AG GG AG AG AG GG AG
AG AG AG AG GG AG AG GG
AG GG AG GG AG AG AG
40121487

2833 rs10067327 5 GG AG GG AG AG
AG GG GG GG GG AG GG GG
GG GG GG GG GG AG AG AG
GG GG GG GG AG AG GG AA
AG GG AG AG GG GG GG
51867115
2834 rs10067365 5 CC CC CC CC AC
CC AC AC AC AC AC AC AC
CC CC CC CC CC CC CC CC
AC AC AC AC CC AC CC AC
CC CC AC CC CC CC CC
125401016
2835 rs10067377 5 AA AA AA AG AG
AG AA AA AA AA AA AA AA
AA AA AA AA AA AA AA AA
AA AA AA AA AA AA AA AA
AA AG AA AG AA AA AA
180433966
2836 rs10067389 5 CC CC CC AC AC
AC AA CC CC CC CC CC CC
CC CC CC CC CC CC CC CC
CC AC AC AC AC AC AA CC
CC CC AC AC AC AC AC
121808701
2837 rs10067393 5 AC AC AA AA AA
AA AA AA AA AA AA AA AA
AA AA AA AA AA AC AA AA
AA AA AA AA AA AA AA AA
AA AA AC AA AA AA AA
24620819
2838 rs10067427 5 AG GG AG AG GG
AG AG GG AG GG AA AG AA
AA AA GG AG AG AG GG AG
AG AG AG AG AG GG AG AG
AG AA AG AA GG AG AG
99342047
2839 rs10067441 5 AC AA AC CC CC
CC CC AC AC AC CC CC CC
CC CC CC AC AC CC CC CC
AC AC CC CC CC CC CC CC
AC CC CC CC CC CC AC
146201876
2840 rs10067484 5 GG GG GG GG GG
AG AG GG GG GG AG GG GG
GG GG GG GG AG GG AG AG
GG GG GG GG GG GG GG GG
GG GG GG GG GG AG GG
75393618
2841 rs1006750
X -- -- -- -- -- -- -- --

-- -- -- -- -- -- -- --
-- -- -- -- -- -- -- --
-- -- -- -- -- -- -- --
-- -- -- -- 39116720
2842 rs10067502 5 GG GG GG AG GG
GG AG GG GG GG GG GG GG
GG GG AG AG GG AG GG AG
GG GG GG GG AG GG GG GG
AA GG GG GG AG AG AG
101585209
2843 rs10067536 5 AC AC CC AC CC
AC CC AC CC AC CC CC AC
AC AC CC CC CC CC AC CC
AC AC AC AC AC CC AC AC
CC AC CC AC AA AA AC
23210213
2844 rs10067550 5 AG AA AG AG AA
AG AG AG AG AG AG GG AG
GG GG AA AA AA AA GG AA
GG GG GG GG AA AG AG AA
AG GG AG AG AG AA AG
116515829
2845 rs10067626 5 GG GG GG GG GG
GG GG GG GG GG GG GG GG
GG GG GG GG GG GG GG GG
GG GG GG GG AG GG GG AG
GG GG GG GG AG AG GG
31914966
2846 rs10067650 5 AA AA AA AG AG
AG AA AG AG AG AA AA AA
AA AA AG AG AA AA AA AA
AA AA AA AA AG AA AG AA
AG AA AA AA AA AA AA
163352472
2847 rs10067664 5 AA AA AA AA AA
AA AA AG AG AG AA AA AA
AA AA AA AG AG AA AA AA
AG AA AA AG AA AA AA AA
AA AA AA AA AG AA GG
94400017
2848 rs10067702 5 AC AC AA AC AA
AC AC AC AC AC AC AC CC
CC CC AC CC AC AA CC AA
CC CC CC CC AC AA AA AA
AA AC AA AC AC CC AA
109419718
2849 rs10067711 5 AA AA AA AA AG
AA AG AA AA AA AA AA AA
AA AA AA AA AA AG AG AA
AA AA AA AA AA AA AA AA

AA AA AA AA AA AA AA
128113882
2850 rs10067717 5 GG GG GG GG GG
GG GG GG GG GG GG GG GG
GG GG GG GG GG GG GG GG
GG GG GG GG GG GG GG GG
GG GG GG GG GG GG GG
150825197
2851 rs10067788 5 AA AA AA AC AA
AA AC AA AA AA AA AA AC
AC AA AA AA AA AC AA AA
AA AA AA AA AC AA AC AA
AA AA AA AA AA AA AA
151333011
2852 rs10067818 5 GG GG GG AA AA
AG AG GG GG GG AG AG AA
AA AG AG GG GG GG GG GG
GG AG AG GG GG AG GG AG
GG GG GG GG GG GG GG
44161816
2853 rs10067960 5 AA AA AA AG AG
AG AA AG AG AG AA AA AA
AA AA AA AA AA AG AA AG
AG AG AG AG AG GG AG AG
GG AG AA AG AA AA AA
111148786
2854 rs10067968 5 AG GG AG AG AG
GG AA AA AA AA AG AG AG
AG GG AG AG AA GG GG AG
AG AG AG AG AG AG AA AG
AG AG GG AG AG AG GG
169391558
2855 rs10067976 5 AA AA AA AA AA
AA AA AA AA AA AA AA AA
AA AA AA AA AA AA AA AA
AA AA AA AA AC AA AA AA
AC AA AA AA AA AC AC
134097290
2856 rs1006802 4 AA AG AA AA AA
AA AA AA AA AA AG AG AG
AG AG AG AG AA AG AG GG
AA AA AA AA AA AA AA AA
AA AA AG AA AG AA AG
26237157
2857 rs10068052 5 AG GG GG AA AA
AA AA AG AA AG GG AG GG
AG GG AA AA AA AG AG AG
AA AA AA AA AA AG AA AG
AG AG AA AG AA AA AA
31471676

2858	rs10068073	5	GG	GG	GG	GG	GG
GG	GG	GG	GG	GG	AG	GG	AG
GG	AG	GG	GG	GG	AG	AA	GG
GG	GG	GG	GG	GG	GG	GG	GG
AG	GG	GG	GG	GG	GG	GG	
54074654							
2859	rs10068115	5	AG	AG	GG	AG	AG
AG	AG	AA	AA	AA	AA	AA	AA
AG	AG	GG	GG	GG	AG	AA	AG
GG	GG	GG	GG	AG	AG	AG	GG
AG	AA	AG	AA	AG	GG	AG	
2252398							
2860	rs1006812	1	GG	GG	GG	AG	GG
GG	AG	GG	GG	GG	GG	GG	GG
GG	GG	GG	GG	GG	GG	GG	GG
GG	AG	GG	AG	GG	GG	GG	GG
GG	GG	AG	AG	GG	GG	GG	
38915251							
2861	rs10068159	5	AG	AG	AG	AG	AG
AG	AA	AA	AA	AA	AA	AG	AA
AA	AA	GG	GG	GG	AA	AA	AG
AA	AA	AA	AA	AG	GG	AG	AG
AG	AG	AG	AG	AA	AA	AG	
125683958							
2862	rs10068162	5	AG	AA	AA	AA	AA
AA	AA	AA	AA	AA	AA	AG	AA
AA	AA	AG	AA	AG	GG	GG	AG
GG	GG	AG	GG	AA	AG	AA	AG
AA	AA	AG	AG	AA	AA	AG	
22567214							
2863	rs10068191	5	GG	AG	GG	AG	AA
AG	AG	GG	AG	AG	GG	AG	AG
AG	AG	AA	AG	AG	GG	GG	GG
GG	GG	AG	AG	AA	AG	AG	AA
AG	GG	AG	AG	AG	AG	GG	
133310248							
2864	rs10068258	5	AG	AG	AG	GG	AG
GG	AG	AG	AA	AA	AG	AG	AG
GG	GG	AG	GG	GG	AG	GG	AG
AG	AG	GG	GG	GG	GG	GG	GG
AG	GG	GG	GG	GG	GG	GG	
133048578							
2865	rs1006826	11	AA	AA	AG	AG	AA
AG	AA	AG	GG	GG	AG	AA	AA
AG	AA	AA	AG	AG	AG	AG	AA
GG	AG	GG	AG	AA	AA	AA	AA
GG	AA	AG	AG	AA	AA	AA	
113135006							
2866	rs10068269	5	AG	AA	AG	AG	AG
AG	GG	AG	AA	AG	AG	AG	AA

AA AA AA AA AA AG AG AG
AG AA AA AG AG AA AA AG
AG GG AA AG AG AA AG
39991855
2867 rs10068310 5 AA AA AG AA AA
AA AG AA AG AA AA AA AG
AG AG AG GG AG AG AG GG
AG AG AG AG AA AA AA AA
AG AG AA AG AG AA AG
33628171
2868 rs10068316 5 AG GG GG GG GG
GG GG AG AG AG AG AG AG
GG GG GG AG AG AG AG GG
AG AG GG AG AG GG AG AG
GG AG AG AG GG GG GG
5938780
2869 rs10068357 5 AA AC AC AA AA
AA AA AA AA AA AA AC AA
AA AA AA AA AA AA AA AA
AA AA AA AA AA AA AA AA
AA AA AA AA AA AA AA
26727405
2870 rs10068385 5 AG AA AA AA AA
AG AG AG AG AA AA AA AA
AA AA AG AG AG AA AG AA
AA AA AA AA AA AG AG AA
AG AG AA AA AG AA AG
79158432
2871 rs10068395 5 GG GG GG AG AG
AG GG AG GG AG AG AG GG
GG GG GG GG GG GG GG AG
AG AG AG AG GG GG GG GG
GG GG GG GG GG GG GG
178252366
2872 rs10068414 5 GG GG GG GG GG
GG GG GG GG GG GG GG GG
GG GG GG GG GG GG AG GG
GG GG AG AG AG AA AG AG
GG GG AA AG AG AA GG
146302106
2873 rs10068434 5 AG AG AG GG AG
GG AG AG AG AG AA AA AA
AA AA AA AA AA AG AA GG
AA AG AG AA GG AA AG AG
AA AG AA AG AA AA AA
102704259
2874 rs10068445 5 AA AA AA AC AA
AA AC AA AA AA AC AC AC
AC AC AC AC CC AC AA AC
AA AA AA AA AA CC AC AC

	AC	AC	AC	AA	AA	AC	AA
106011329							
2875	rs10068476		5	GG	GG	GG	AG	AG
	AG	GG	GG	GG	GG	GG	GG	GG
	GG	GG	AG	GG	GG	AG	GG	AG
	GG	AG	AG	GG	GG	AG	GG	AG
	GG	AG	AG	AA	GG	GG	GG	
44422633								
2876	rs10068482		5	AA	AA	AA	AA	AA
	AA	AC	AC	AA	AC	AA	AA	AC
	AA	AC	AA	AC	AC	AA	AA	AC
	AA	AA	AA	AA	AC	AC	CC	AA
	CC	AA	AA	AA	AA	AA	AA	
35325555								
2877	rs10068486		5	GG	GG	GG	GG	GG
	GG	AG	AG	AG	AG	AA	AA	AG
	AG	AG	AA	AG	AG	GG	GG	AG
	AA	AG	AA	AA	GG	GG	GG	GG
	GG	AG	GG	AG	AG	GG	AG	
169865639								
2878	rs10068515		5	GG	GG	GG	GG	GG
	GG	GG	GG	GG	GG	AA	AG	AG
	AG	AG	GG	GG	GG	GG	GG	GG
	GG	GG	AG	GG	GG	GG	GG	GG
	GG	GG	AA	AG	GG	GG	GG	
24098129								
2879	rs10068517		5	AA	AA	AA	AA	AA
	AA	AA	AA	AA	AA	AA	AA	AA
	AA	AA	AA	AA	AA	AA	AA	AA
	AA	AA	AA	AA	AA	AA	AA	AA
	AG	AA	AA	AA	AA	AA	AA	
26441617								
2880	rs1006857		16	AA	AA	AA	AA	AA
	AA	AG	AA	AA	AA	AA	AA	AA
	AA	AA	AA	AA	AA	AA	AG	AA
	AA	AA	AA	AA	AA	AA	AA	AA
	AA	AA	AA	AA	AA	AA	AA	
65133617								
2881	rs10068616		5	AG	GG	GG	GG	GG
	AG	GG	AA	AA	AA	AG	GG	GG
	GG	GG	AA	AG	AG	AG	AG	AG
	AA	AG	AG	AG	AG	AG	AA	GG
	AA	AG	AG	GG	AA	AG	AG	
119298330								
2882	rs10068621		5	AA	AA	AA	AC	AC
	AC	CC	AC	AC	AC	AC	AA	AA
	AC	AC	AC	AC	AC	AA	AC	AC
	AA	AC	AC	AC	CC	AC	AC	CC
	AC	AC	AC	AC	AA	AC	AC	
119189608								

2883 rs10068624 5 AG AG AG GG GG
AG GG GG GG GG AG AG AA
AA AA GG GG GG GG GG AG
GG GG GG GG AG GG AG AG
GG AG GG GG GG AG AG
125803119
2884 rs10068633 5 GG AG GG GG GG
GG AG AG AG AG AA AG AA
AA AA AG GG GG AG AG AG
AG AG AG AG GG GG GG GG
AG GG GG GG GG AG AG
84170267
2885 rs10068658 5 AG GG GG AG AA
AG AG AA AA AA AG AG AG
AG AA AG AG GG AG GG GG
AG AG AG AG GG GG GG GG
GG GG AG AG AG AG AG
26263051
2886 rs10068685 5 AA AA AA AA AA
AA AA AA AA AA AA AG AG
AG AA AA AA AA AA AA AA
AA AA AA AA AA AA AA AA
AA AA AA AA AA AA AA
51667266
2887 rs1006877 3 AG AG GG AA AA
AA AA GG GG AG GG GG GG
AG AG GG GG GG AA AG AA
AA AA GG AA AG AA AG AG
GG GG GG GG AA AA AA
140053766
2888 rs10068809 5 GG GG GG GG GG
AG GG AG AG AG GG GG GG
GG GG GG GG GG GG GG GG
GG GG GG GG AG GG GG AG
GG GG GG GG GG GG GG
58420346
2889 rs1006888 14 AG AA AA AA GG
AG AG AA AA AA AA AA AA
AG AG AA AA AA AA AG AA
AA AA AA AA AA AA AA AA
AG AA AA AA AA AA AA
93418952
2890 rs1006893 21 AA AG AA GG GG
GG AG AG AG GG AA AG AG
AG AG AA AA AA AG AA AA
AG AA AA AA AG AG AA GG
AG AG AG AG GG AG AG
40303677
2891 rs10069 5 GG GG GG AG AG AG
AG AG GG GG GG GG GG GG

GG AG GG AG GG GG GG GG
GG GG GG GG AG GG GG AG
GG AG AG AG AG GG 180003085
2892 rs10069076 5 CC AC CC AC AC
AC AA AA AA AA AA AA AA
AA AA AA AA AA CC AA AC
CC AC AC CC AC AA AA AA
AC AC AA AA AC CC AA
93789309
2893 rs10069092 5 GG GG AG GG GG
GG GG AG AA AG GG GG AG
GG AG GG GG GG AG GG GG
AG AG AG AG AG AG GG AG
AA AG AG GG GG AG GG
30009138
2894 rs10069119
5 -- -- -- -- -- -- -- --
-- -- -- -- -- -- -- --
-- -- -- -- -- -- -- --
-- -- -- -- -- -- -- --
-- -- -- -- 4341114
2895 rs10069129 5 AG AG AG AG AA
AG AG AG AA AG AA AA AA
AG AG AG AG AA AG AG AA
AA AA GG AG AA AG AG AA
AA AA AG AG AA AA AA
3151352
2896 rs10069151 5 AA AA AA AG AA
AA AG AA AA AA AA AA AA
AG AG AA AG AG AG AA AG
AG AA AA AA AG AA AG AG
AA AA AA AA AG AA AG
125708877
2897 rs1006923 2 AG AA AG AG AG
AA GG GG GG GG GG GG AG
AG AG AG AG AG AA AG AG
AG AG AG AG AA AG AA AA
AA AG AA AG AA AA AG
145775399
2898 rs10069282 5 AA AA AA AG AG
GG AA AG AA AG AG AG AA
AG AG AA AG AA AA AA AA
AA AA AA AA AG AG AG AA
AA GG AA AG AA AA AA
177892956
2899 rs10069339 5 AA AA AG AA AA
AA AA AA AA AA AA AA AA
AA AA AA AG AG AA AG AA
AA AA AA AA AA AG AG AA
AA AA AA AA AG AA GG

107985830
2900 rs10069375 5 AC AC CC AC AC
AC AC AA AA AA AA AA AA
AA AA AC AC AA AA AA AA
CC CC AC CC AA AC AA AC
AA AC AC AC AA AA AA
2356789
2901 rs10069382 5 GG AG AG AG AG
AA GG GG GG GG AA AG AG
AA AG AA AA AA GG GG AG
GG GG GG GG AG GG AG GG
AG GG AG AG AG GG AG
31921265
2902 rs10069487 5 GG AG AA AA AG
AG AA AA AA AA AG AG AG
GG GG AG AA AG AG AA AA
AA AA AA AA AG AG AG AA
AG GG AA AG AA AA AA
9841870
2903 rs10069531
5 -- -- -- -- -- -- -- --
-- -- -- -- -- -- -- --
-- -- -- -- -- -- -- --
-- -- -- -- -- -- -- --
-- -- -- -- 162527812
2904 rs1006955 10 GG AG GG AG GG
GG AG GG GG GG AG -- AG
AG AA AG GG AG AG AG AG
AG AG AG AA AG GG AG GG
GG AA AG AG AA AA AA
62368339
2905 rs1006958 17 GG GG GG GG GG
GG GG GG GG GG GG GG GG
GG GG GG GG GG GG GG GG
GG GG GG GG GG GG GG GG
GG GG GG GG GG GG GG
16282454
2906 rs10069581 5 GG GG GG GG GG
GG GG GG GG GG GG GG GG
GG GG AG GG AG GG GG GG
GG GG GG GG AG GG GG GG
GG AG GG AG GG GG GG
4777480
2907 rs1006965 3 GG GG GG GG GG
GG GG GG GG AG GG GG GG
GG GG GG GG GG GG AG GG
AG AG AG AG GG GG GG GG
GG GG GG GG GG GG AG
31383105
2908 rs10069666 5 AA -- AA AA AA

AC AA AA AA AA AA -- AA
AA AA AC AC AA AA AA AA
AA AA AA AA AA AA AA AA
AA AA AA AA AA AC AA
161837675
2909 rs10069690 5 GG GG GG AG AG
GG AA GG GG GG GG AA AG
AG AG GG AG AG GG AG GG
AG AG GG GG AG GG GG GG
AG AG GG AG GG AG GG
1279790
2910 rs10069741 5 GG GG GG GG GG
GG GG GG GG GG GG GG GG
GG GG GG GG GG GG GG GG
GG GG GG GG GG GG GG GG
GG GG GG GG GG GG GG
26211390
2911 rs10069803 5 AG AA AG GG GG
GG GG GG GG GG AG GG AG
AG AG GG GG GG GG GG AG
AG AG AG AG AG GG AG AG
AG GG GG GG GG GG GG
101364700
2912 rs10069818 5 AG AG AG AA AA
AA AA AG AG AG AA AA AA
AA AA AG AG AG GG GG AG
AG GG AG AG GG GG GG GG
AA GG AA AG AA AG AA
77202560
2913 rs10069830 5 GG AG AA AG AG
AG AA GG GG AG AG AG GG
GG GG GG AG AG AG AG AG
AG AG AG AG AG GG GG AG
GG AG AG AA AG AG AG
61497716
2914 rs10069940 5 AA AG AA AG AG
GG AG AA AA AA AG AG GG
AG GG AG AG AG AG AA GG
GG AG AG GG AG AA AA AG
AG GG GG GG AG AG AG
51497015
2915 rs1006997 6 AG GG AG GG GG
GG GG GG GG GG AG AG GG
GG GG AG GG GG GG GG AG
GG GG GG GG GG GG GG GG
AG GG GG GG GG GG GG
69677322
2916 rs10069971 5 AC AC AC AC CC
AC AC AC AC AC AA AC AA
AA AA CC CC CC AC CC AA

AA AA AA AA AC AA AC AA
AC CC AC AC AC AA CC
124110646
2917 rs10070070 5 GG GG GG GG GG
GG GG GG GG GG GG GG GG
GG GG GG GG GG GG AG AG
GG GG GG GG GG AG AG GG
AG GG GG GG GG AG GG
103091216
2918 rs1007010 8 AA AA AA GG AG
AG AG AA AA AA AG AA AG
AA AA AG AG GG AA AA AA
AG AA AA AG AA AA AA AA
AA AG AA AA AA AG AA
3167898
2919 rs10070122 5 AA AA AA AA AA
AA AA AA AA AA AA AA AA
AA AA AA AA AA AA AA AA
AA AA AA AA AA AA AA AA
AA AA AA AA AA AA AA
167995221
2920 rs10070143 5 GG GG GG GG GG
GG GG AG AG AG GG GG GG
GG GG GG GG GG GG AG GG
GG GG GG GG AG GG GG AG
GG GG GG GG GG GG GG
176487161
2921 rs10070158 5 GG GG GG GG GG
GG GG GG GG GG GG GG GG
GG GG GG GG GG GG GG GG
GG GG GG GG GG GG GG GG
GG GG GG GG GG GG GG
121739541
2922 rs10070246 5 AG AA AA AG GG
GG AG AG AG AG GG GG GG
GG GG GG AG AG AG AG GG
GG GG GG GG AG AG GG AG
AG GG AG AG AG AA GG
106084494
2923 rs10070303 5 GG AG AG GG GG
AG AG GG GG GG AG AG AA
AA AA GG GG GG AG AG AG
GG GG GG GG GG AG AG AG
GG GG AG GG AG AG GG
177840382
2924 rs10070337 5 AG GG GG AG AG
GG AG AA AA AA AA AA AA
AG AG AG AG AA AG AA AG
AG AG AA AG AG AG AG AA
AG GG AA AG AA AG AG

2993617
2925 rs10070341 5 GG GG GG GG GG
GG AG GG GG GG GG GG GG
GG GG AG GG AG AG AG GG
GG GG GG GG GG GG GG GG
GG GG AG GG GG GG GG
24691553
2926 rs10070379 5 AG AA AA AA AA
AA AA AA AA AG AA AG AA
AA AA AG AA AG AG AA AG
AA AA AA AA AG AG AG AG
AG AA AG AA AG AG AA
67986294
2927 rs10070401 5 AA AA AG AG AG
AA AG AA AA AA AA AG AA
AA AA AG AA AG AG AA AG
AG AG AG AG GG AA AG AG
AG AG AG AG GG AG AG
94316007
2928 rs10070412 5 AG GG AG AG AG
AA AG AG AG AG AG AG GG
AG AG AA AA AA AA AA AA
AG AG AG AG AG AG GG AG
AG AG AG GG AG AA AG
30865369
2929 rs1007044 4 GG GG AG AG AG
GG AG GG GG GG AG AG AG
AA AG GG GG GG GG GG GG
AG GG GG GG GG GG GG GG
GG AA AG AA GG GG GG
75899133
2930 rs1007049 15 AA AA AA AC AC
AC AC AA AA AA AC AC AA
AC AA AA AA AA AA AA AA
AA AC AC AC AC AA AA AA
AA AC AC AC AC AA AC
57622600
2931 rs10070522 5 AA AG GG AG AG
AG GG GG GG GG AA GG AG
AG AG AA AG AG AG AG AA
AG AA AA AG GG AG GG AG
AG GG AG GG AG AG AG
57786110
2932 rs1007055 12 GG GG GG GG GG
GG GG GG GG GG GG GG GG
GG GG GG GG GG GG GG GG
GG GG GG GG GG GG GG GG
GG GG GG GG GG GG GG
13543138
2933 rs10070568 5 GG GG GG GG GG

GG GG GG GG GG GG GG GG
GG GG GG GG GG GG GG GG
GG GG GG GG GG GG GG GG
GG GG GG GG GG GG GG
168402137
2934 rs10070689 5 AG AG AG AG AG
AG GG GG GG GG GG GG GG
GG GG GG GG GG GG AG GG
GG GG GG GG GG GG GG GG
GG GG AG GG AG AG GG
99458306
2935 rs10070717 5 GG GG GG GG GG
GG GG GG GG GG GG GG GG
GG GG GG GG GG GG GG GG
GG GG GG GG GG GG GG GG
GG GG GG GG GG GG GG
9834220
2936 rs10070739 5 AA AA AA AA AA
AA AA AA AA AA AA AA AA
AA AA AG AG AA AA AA AA
AA AA AA AA AA AA AA AA
AA AA AA AA AA AA AA
66544879
2937 rs10070748 5 AA AA AA AA AA
AA AC AA AA AA AA AA AA
AC AA AA AA AA AA AA AA
CC CC AC CC AC AA AA AA
AA AC AA AC AC AA AC
26315469
2938 rs10070771 5 AA AA AA AA AA
AA AA AA AA AA AA AA AA
AA AA AA AA AA AA AA AA
AA AA AA AA AA AA AA AA
AA AA AA AA AA AA AA
90415505
2939 rs10070825 5 AA AC AA AA AA
AA AA AA AA AA AA AA AA
AA AA AC AA AC AA AA AA
AA AA AA AA AA AA AA AA
AA AC AA AC AA AA AA
112834255
2940 rs10070878 5 GG GG GG GG GG
GG GG GG GG GG AG GG GG
AA AG GG GG GG GG GG GG
GG GG GG GG GG AG GG AG
GG GG GG GG GG GG AG
28184320
2941 rs10070886 5 AC AC AA AA AA
AC AA AC AC AC AC AC AC
AC AC CC AC AC AA AC AA

CC CC CC CC AC AC AA AC
AC AA AC AC AC AC AA
174201046
2942 rs1007090 20 GG GG GG AG AG
GG AA AA AA AG GG GG AG
AG GG GG GG GG GG GG GG
GG GG GG GG AA AG AA AA
AG GG GG GG AG AA GG
32582871
2943 rs10070905 5 AG AA AG AG AG
GG AA AG AA AG GG GG GG
AG AG AG GG AG GG GG GG
GG GG GG GG GG GG GG GG
GG GG AG GG GG GG AG
143039972
2944 rs10070913 5 GG GG GG GG GG
GG GG AG AG AG GG GG GG
GG GG GG GG GG GG GG GG
GG GG GG GG GG GG GG GG
GG GG GG GG GG GG GG
163063248
2945 rs10070937 5 GG GG GG GG AG
GG AG GG GG GG GG AA AG
AG AG GG AG AG AA GG AG
GG AG AG GG AG GG AG AG
GG GG AG AG AG AG AG
101097727
2946 rs10071 12 AG AA AG GG GG GG
GG GG GG AG GG GG GG GG
GG AG AG AG GG AG GG AG
AG AG AG AG GG GG GG GG
GG GG GG GG AG AG 105567572
2947 rs10071011 5 AA AA AA AA AA
AA AA AA AA AA AA AA AA
AA AA AA AA AA AA AA AA
AA AA AA AA AA AA AA AA
AA AA AA AA AA AA AA
19412016
2948 rs10071062 5 AA AG AG AG AA
AA AG AG GG AG AG AG GG
AG AG AG AG AA AA AA AA
AG AA AA AA AA AG AG AA
AA AG AG GG AA AG AG
115293612
2949 rs10071072 5 AA AA AA AA AC
AC AA AA AA AA AA AA AA
AA AA AA AA AA AA AA AA
AC AC AA AC AA AA AA AA
AA AC AA AA AA AA AC
6486876

2950 rs1007112 12 GG GG GG GG GG
GG GG GG GG GG GG GG GG
GG GG GG GG GG GG GG GG
GG GG GG GG GG GG GG GG
GG GG GG GG GG GG GG
118087455
2951 rs10071168 5 GG GG GG GG GG
GG GG GG GG GG GG GG GG
GG GG GG GG GG GG GG GG
GG GG GG GG GG GG GG GG
GG GG GG GG GG GG GG
171182240
2952 rs10071208 5 GG AG GG GG GG
GG GG GG GG GG GG GG GG
GG GG GG GG GG GG AG GG
GG GG GG GG GG GG GG GG
GG GG AG AG GG GG GG
2690668
2953 rs10071246 5 AG AG GG AA AA
AG AA AA AA AA AA AG AG
GG GG AG AG GG AG AG AG
AG AG AA AG AG AG GG AG
GG AA AG AA AG AG AA
2768879
2954 rs10071309 5 AA AA AA AA AA
AA AA AA AA AA AA AA AA
AA AA AA AA AA AA AA AA
AA AA AA AA AA AA AA AA
AA AA AA AA AA AA AA
24828165
2955 rs10071334 5 GG GG GG GG GG
GG GG GG GG GG GG GG GG
GG GG GG GG GG GG GG GG
GG GG GG GG GG GG GG GG
AG GG AG AG GG GG GG
180599287
2956 rs1007137 20 AA AG AG AA AA
AA AA AG AG AG AA AA AA
AA AA AA AG AA AA AA AG
AA AA AA AA AA AG AG AG
GG AG AA AA AA AA AA
43816593
2957 rs10071371 5 AG AG AG AA AA
AA AG GG GG AG AG AA AG
AA AG AG AG AA AG AG AG
AA AA AA AA AG AG GG AA
AG GG AG AG AA AG AA
63309892
2958 rs1007139 5 AA AG AG AG AG
AA GG AG GG AG AA AG AG

GG AA AA AA AA GG GG AG
AA AA AA AA AG GG GG AG
AG AG GG AG AG AG AA
53874272
2959 rs10071392 5 AG AG AA GG GG
GG AG AG AG AG GG GG GG
AG AG AA AA AA AG AG GG
GG AG GG GG GG AG AG AG
GG GG GG GG AG AA AG
172338616
2960 rs10071420 5 AC AC AC AA AA
AA AC AA AA AA CC AA AC
CC CC AC AA AA AA AC AA
AC AC AC AC AC AA AA AA
AA AC AA AC AA AA AC
83932510
2961 rs10071426 5 AG AG AG AA AA
AA AA GG GG GG GG GG GG
GG GG AG AG AA AA AG AG
AG AG AA AG GG AG GG AG
AG AA AA AA AG AG AA
23610032
2962 rs10071435
5 -- -- -- -- -- -- -- --
-- -- -- -- -- -- -- --
-- -- -- -- -- -- -- --
-- -- -- -- -- -- -- --
-- -- -- -- 117390364
2963 rs10071437 5 AA AA AG AA AA
AG AG AA AA AA AG AG AG
AG AG GG GG GG AA AA AA
GG AG GG GG AG AA AA AG
GG AG AG AA AG AG AA
71230022
2964 rs10071459 5 AC CC CC CC CC
CC AC CC AC CC CC CC CC
CC CC CC CC CC AC AC AC
CC CC CC CC AC AC AC AA
CC CC CC CC AC CC AC
18878436
2965 rs10071484 5 AA AA AA AA AA
AA AA AA AA AA AA AA AA
AG AG AA AG AG AG AG GG
AG AA AA AA GG AA AG AG
AA AA AA AA AA AA AA
3689527
2966 rs10071490 5 GG GG GG GG GG
GG GG GG GG GG GG GG GG
GG GG GG GG GG GG GG GG
GG GG GG GG GG AA AG AG

GG GG GG GG GG GG GG
113940187
2967 rs10071528 5 AA AA AA AA AA
AA AA AA AA AA AA AA AA
AA AA AA AA AA AA AA AA
AA AA AA AA AA AA AA AA
AA AA AA AA AA AA AA
45685961
2968 rs1007153 X AA AG GG GG GG
GG GG AG AG GG GG GG AG
AG AG AA AG AG AG AG GG
GG GG GG AG AG AA AG AA
AG AA GG AA GG GG GG
55778139
2969 rs10071585 5 AG AA AA AG AG
AG AG AA AA AA AG AA AA
GG AG AG AA GG AA AG AG
AG AA AG AG AA AA AA AA
AG AA AA AA AA AA AA
68245920
2970 rs1007160 19 AC AC CC AA AA
AA AC AC AA AA AC CC AC
CC CC CC CC CC AC CC AC
AC CC AC CC AC CC CC AC
AC AA CC AC CC CC CC
33353061
2971 rs10071637 5 AG AG AG AG AG
AG GG GG GG GG GG GG AG
AG AG GG GG GG GG GG GG
AG AG AG AG AG GG AG AG
GG GG AG GG GG GG GG
165995991
2972 rs10071654 5 AC CC AC AA AA
AC AC AC AC AC CC CC AC
AA AA AA AC AC AA AA AA
AC AA AC AC AC AC AC CC
CC AA CC AC CC AC CC
168563018
2973 rs1007170 1 AG AA AA AG GG
AG GG AG AA AG AA AG AG
AG AG AG AA AG AG AG AG
GG AG GG AG AG AG AG AA
AA GG AA AG AA AA AA
154881461
2974 rs10071732 5 AA AA AA AA AA
AA AA AA AA AA AA AA AA
AA AA AA AA AA AA AA AA
AA AA AA AA AA AA AA AA
AA AA AA AA AA AA AA
41931157

2975 rs10071738 5 AG AG -- AG AG
AG -- AG -- AG -- -- --
-- -- AG AG AA AG AG AG
AG -- AG AG AG -- AG AG
AG AG AG AG -- -- --
178100927
2976 rs1007175 13 AA AA AA AA AA
AA AA AG AG AG AA AG AA
AA AA AG AG AA AG AA GG
AG AG AG AG GG GG GG GG
AA AG AA AG AG AG AA
97612539
2977 rs10071777 5 AA AA AA AA AG
AA AG AA AA AA AG AG AG
AG AG AG AG AG AA AG AA
AA AA AA AA AA AG AG AA
AG AA AA AA AA AA AA
117286831
2978 rs10071778 5 AG AA AG AA AA
AA AA AA AA AA AA AA AA
AA AA AG AG AG AA AA AA
AA AA AA AA AA AA AA AA
AG AA AA AA AA AA AA
123919181
2979 rs1007178 17 CC AC CC CC CC
CC CC CC AC AC CC CC CC
CC CC CC CC CC CC AC CC
CC AC AC AC CC CC CC CC
CC AC CC AC AC AC CC
21151106
2980 rs10071789 5 AA AA AA AA AA
AA AA AA AA AA AA AC AA
AA AA AA AA AA AC AA AA
AC AA AA AC AA AC AC AA
AA AA AC AA AA AA AA
84609984
2981 rs10071798 5 GG GG GG GG GG
GG GG GG GG GG GG GG GG
GG GG GG GG GG GG GG GG
GG GG GG GG GG GG GG GG
GG GG GG GG GG GG GG
12715447
2982 rs10071824 5 AG AA AG GG GG
GG GG AG AG AG AG GG GG
AG AG AG AG GG GG AG AG
GG GG GG GG AG GG GG GG
AG AG GG AG AG AG GG
24846954
2983 rs10071835 5 AA AA AA AA AA
AA AA AA AG AA AA AA AA

	AA	AA	AA	AA	AA	AA	AG	AA
	AA	AA	AA	AA	AG	AG	AG	AG
	AA	AA	AA	AA	AA	AA	AA	
969285								
2984	rs10071841		5	AG	AG	AA	AA	AG
	AG	AG	AG	AA	AG	GG	GG	AG
	AG	AG	AG	GG	AG	AG	GG	GG
	AG	AG	AG	AG	AA	GG	AG	AG
	GG	GG	GG	GG	AG	AA	GG	
27115148								
2985	rs10071870		5	AG	AG	AA	GG	GG
	AG	GG	GG	GG	GG	AA	GG	AG
	GG	AG	GG	GG	GG	--	GG	GG
	GG	GG	GG	GG	GG	GG	GG	GG
	GG	GG	GG	GG	AA	AA	AA	
40653227								
2986	rs1007191		5	AA	AA	AA	AA	AA
	AA	AA	AG	AG	AG	AA	AA	AA
	AG	AG	AG	AG	AG	AA	AA	AA
	AA	AA	AA	AA	AA	AA	AA	AA
	AA	AG	AG	AA	AG	AG	AG	
123521221								
2987	rs10071932		5	AG	GG	AG	GG	GG
	GG	AG	AA	AA	AA	AA	AA	AA
	AG	AA	AA	AA	AA	AA	AA	AA
	AG	AA	AA	AG	GG	AG	GG	AG
	AA	AG	GG	AG	AA	AG	AA	
56653871								
2988	rs10071971		5	GG	GG	GG	GG	GG
	GG	GG	GG	GG	GG	GG	GG	GG
	AG	AG	GG	GG	GG	GG	GG	GG
	GG	GG	GG	GG	GG	GG	GG	GG
	GG	GG	GG	GG	GG	GG	GG	
177518127								
2989	rs10072008		5	GG	GG	GG	AG	AG
	GG	AG	GG	GG	GG	GG	GG	GG
	GG	GG	AA	AG	AG	GG	GG	GG
	GG	GG	GG	GG	GG	AG	AG	GG
	GG	GG	GG	GG	AG	AG	AG	
79435683								
2990	rs10072077		5	AA	AA	AA	AG	AG
	GG	AG	AA	AG	AG	AA	AA	AA
	AA	AA	AG	AA	AA	AG	AA	AG
	AA	AA	AA	AA	AG	AG	AG	GG
	AA	AA	AA	AA	AA	AA	AA	
88895572								
2991	rs10072083		5	GG	GG	AG	GG	GG
	GG	GG	GG	GG	GG	AG	GG	AG
	GG	AG	GG	GG	GG	AG	GG	AG
	GG	GG	GG	GG	GG	GG	GG	GG

AG AG GG GG GG GG GG
42896083
2992 rs10072088 5 AA AG AA AA AA
AA AA AA AA AA AA AA AG
AA AA AG AG AA AG AA AA
AG AA AA AA AA AG AG AA
AA AG AG AG AA AG AG
116019417
2993 rs10072140 5 GG GG GG GG GG
GG GG GG GG GG GG GG GG
GG GG GG GG GG GG GG GG
GG GG GG GG GG GG GG GG
GG GG GG GG GG GG GG
21869461
2994 rs1007216 1 GG GG AG GG GG
GG GG AA AA AG GG GG GG
AG GG GG AG AG AG AG GG
AG AG GG AG AG AG AG GG
AG AG AA AA AG GG AA
222283216
2995 rs10072177 5 GG GG AG GG GG
GG GG GG GG GG GG AG GG
GG GG AA AG AG GG GG GG
GG GG GG GG GG GG GG GG
GG GG GG GG GG GG GG
26927503
2996 rs1007219 8 GG AG GG AG AG
GG AG AA AA AG GG GG GG
GG AG AG GG AG GG GG GG
GG GG GG GG AG GG GG GG
GG GG GG GG GG GG AG
65576786
2997 rs10072255 5 AG AG GG GG GG
GG GG GG GG GG AG GG AG
AG AG AG AG AG AA AG AG
GG GG GG GG GG GG GG GG
AG GG AG GG GG GG GG
80231174
2998 rs10072291 5 GG GG GG GG GG
GG GG GG GG GG GG GG GG
GG GG GG GG GG GG GG GG
GG GG GG GG GG GG GG GG
GG GG GG GG GG GG GG
169900524
2999 rs10072361 5 AG AA AG AG GG
AG AG AA AA AA AA AG AA
AG AG AA AA AA AA AA AA
AG AG AG AG AA AA AA AA
AA AA AA AA AA AG AA
128017544

3000 rs10072362 5 AA AC AA AA AA
AC AA AA AA AA AC CC CC
AC AC AA AA AA AC AC AA
AA AA AA AA CC AA AC AC
AA AA CC AC AA AC AC
134342720
3001 rs10072374 5 AG AG GG GG GG
GG GG GG GG GG GG GG GG
GG GG GG GG GG GG GG GG
GG GG GG GG GG GG GG GG
GG GG GG GG GG GG GG
9987909
3002 rs10072467 5 GG GG GG GG GG
GG GG AG AG AG GG GG GG
GG GG AG GG AG GG GG GG
GG GG AG GG AG GG GG GG
AG AG GG GG GG GG GG
23216237
3003 rs10072474 5 AA AA AA GG GG
GG AG AA AA AA AA AA AA
AA AG AG AG GG AA AA AA
AG AG AG AG AG AG GG AA
AG AG AA AG AG GG AA
54083159
3004 rs10072489 5 AG AA AA AA AA
AA AA AG AG AG AA AA AA
AA AA AA AA AA AG AA AG
AA AA AA AA AA AG AG AA
AG AG AA AG AG AA GG
73420143
3005 rs1007250 6 GG GG GG GG GG
GG GG AA GG AG GG AG GG
GG GG GG GG GG GG GG AG
GG GG AG AG GG AG GG AG
GG AG GG AG GG GG GG
157461985
3006 rs10072501 5 AA AA AA AA AA
AA AA AA AA AA AA AA AA
AA AA AA AA AA AA AA AA
AA AA AA AA AA AA AA AA
AA AA AA AA AA AA AA
38194499
3007 rs1007251 15 AG AG GG AG AG
GG AG GG GG GG GG GG GG
GG AG AG AA AA AG AA AG
AG AG AG AG AG AG GG AA
GG AG GG GG AG AG AA
96705400
3008 rs10072518 5 AA AG AG AG AG
AA GG AG AG AG AG AG AG

AG GG AA AA AA GG GG AG
AG AG AG AG AA AA AA AA
AG GG AG AG AG AA GG
26064803
3009 rs10072521 5 GG GG GG GG GG
GG AG AG AG AG AG AG GG
GG GG AG AA AG AG GG AG
GG GG GG GG AA AG AA AG
AG GG GG GG GG AG GG
141628405
3010 rs10072534 5 GG GG GG GG GG
GG GG GG GG GG GG GG GG
GG GG GG GG GG GG GG AG
GG GG GG GG GG GG GG GG
GG GG GG GG GG AG GG
87089700
3011 rs10072586 5 AA AA AA AA AA
AA AA AA AA AA AA AA AA
AA AA AA AA AA AA AA AA
AA AA AA AA AA AA AA AA
AA AA AA AA AA AA AA
50707590
3012 rs10072627 5 GG GG GG AG AG
AG GG GG GG GG GG AA AG
AG AG GG AG AG AG GG AA
AG AG AG AG AG GG AG AG
GG GG GG GG GG GG GG
127032021
3013 rs10072629 5 CC CC AC AC AC
AC CC AC AC AC AA AC AC
AC AC CC CC CC AC AC AC
AC AC AC AC AC AC AC AA
CC CC AA AC AA AC AC
3001216
3014 rs10072668 5 AG AG AG GG GG
AG AG AG AA AG AA GG AG
AA AA GG GG GG AG GG GG
GG GG AA AG AG AA AG AG
AA GG AA AG AA AA AA
422752
3015 rs10072778 5 AA AA AA AA AG
AA AG AG AG AG AA AA AA
AA AA AA AA AA AA AA AA
AA AA AA AA AA AA AA AA
AA AA AA AA AA AA AG
148696042
3016 rs10072824 5 AA AA AA AA AA
AA AA AA AA AA AA AA AA
AA AA AA AA AA AA AA AA
AA AA AA AA AA AA AA AA

AA AA AA AA AA AA AA
12245844
3017 rs10072840 5 GG GG GG GG GG
GG GG GG GG GG GG GG GG
GG GG GG AG AG GG GG GG
AG AG GG AG GG GG GG GG
GG GG GG GG GG GG GG
2967695
3018 rs10072841 5 AG GG AG AA AA
AA AA AG AG AG GG GG GG
GG AG AG AG GG AA AA AA
AG AG AG AG AG AA AA AG
GG AG AG GG AG GG AA
150952884
3019 rs10072848 5 AG GG AG GG GG
AG GG GG GG GG GG AG GG
GG GG GG GG GG GG GG GG
AG AG -- GG GG AG GG GG
GG GG GG GG AG GG AA
1920054
3020 rs10072866 5 AC AC CC AC AC
AA CC AA AA AA AC AA AC
AC AC AA AC AC AA AC AA
AA AA AA AA AC CC AC AC
CC AA AC AA CC AC AC
81898168
3021 rs10072872 5 AA AA AA AA AA
AA AG AA AA AA AA AA AA
AA AA AA AA AA AA AG AA
AA AA AA AA AG AA AA AA
AA AA AA AA AA AA AG
83936469
3022 rs1007289 21 AA AA AA AA AA
AA AA AG AG AA AG AA AG
AA AA AA AA AA AA AA AG
AA AA AA AA AA AA AA AA
AG AA AA AA AA AA AA
39707132
3023 rs10072910 5 AA AA AA AG AG
AA GG AA AA AA AA AA AA
AA AA GG AG AG AG GG AA
AG AG AG AG AA AA AA AA
AA AA AG AG AA AA AA
67237504
3024 rs10072921 5 GG GG GG AG GG
GG AG GG GG GG AG AA AG
AG AG AA AG AG GG GG GG
GG GG GG GG GG GG GG GG
GG GG GG GG GG GG GG
113071182

3025 rs10072922 5 AG AG AG AG GG
GG AG GG GG GG AG AG AG
AG AG GG GG GG AG AG AG
GG GG GG GG GG GG GG GG
AG AG AG AA GG AG GG
160703125
3026 rs10072929 5 GG AG GG GG GG
GG GG AA AG AA AG AG GG
GG GG AG GG AG AG GG AG
GG GG GG GG GG GG GG GG
AG AA AG AA GG GG GG
136460855
3027 rs1007294 21 AA AA AA AA AA
AA AA AC AC AA AA AA AA
AA AA AC CC CC AA AC AA
AA AA AA AA AC AA AC AA
AA AC AA AC AA AA AA
35838907
3028 rs10073052 5 GG GG AG AG AG
AG AA AA AG AA AG AA AG
AG AG AA AA AA AA AA AG
AG AA AA AG AA GG AG AG
GG AG AG AA AG AG AG
101635625
3029 rs10073138 5 AG AA AG AG AA
AA AG GG AG AG AG AG AG
AA AA AG AG AA AG AG AG
AA AA AA AA AG AG GG AG
AA AA AG AA AA AA AG
152535502
3030 rs10073231 5 AA AA AA AA AA
AA AA AA AA AA AA AA AA
AA AA AA AA AA AA AA AA
AA AA AA AA AA AA AA AA
AA AA AA AA AA AA AA
39173569
3031 rs10073251 5 AC AC AC AC CC
CC AC AC AC AC CC CC CC
CC CC AC AA AC AA AC AA
CC AC AC CC CC AC AC CC
AA AC CC AC AC CC AA
105830691
3032 rs1007328 15 AG AG GG AG AG
GG AG AG AG AG AG AA AA
AA AA AG AA AA AG AA AG
AA AA AA AA AG AA AG AA
AG AG AG GG AA AA AA
96703373
3033 rs10073299 5 GG GG GG GG GG
GG GG GG GG GG GG GG GG

GG GG GG GG GG GG GG GG
GG GG GG GG GG GG GG GG
GG GG GG GG GG GG GG
27647501
3034 rs10073306 5 GG GG GG GG GG
GG GG GG GG GG GG GG GG
GG GG GG GG GG GG GG GG
GG GG GG GG GG GG GG GG
GG AG GG AG AG AG GG
58061855
3035 rs10073413 5 AC CC AC AA AA
AA AA AA AA AA AC CC AC
AC AC AA AA AA AA AC AC
AA AC AC AA AA AA AA AA
AC AC AA AC CC AC CC
99049676
3036 rs10073441 5 AA AA AA AA AA
AA AA AA AA AA AA AA AA
AA AA AA AA AA AA AA AA
AA AA AA AA AA AA AA AA
AA AA AA AA AA AA AA
107218799
3037 rs10073464 5 GG GG AG AG AG
AA GG GG GG GG GG GG GG
GG GG GG GG GG GG GG GG
GG GG GG GG GG GG GG GG
GG GG GG GG GG AG GG
28465885
3038 rs10073556 5 AG AA AG GG GG
GG GG AG AG AG GG GG GG
AA AG GG GG GG GG GG GG
GG GG GG GG AG GG GG AG
GG GG GG GG AG AG AG
170275387
3039 rs10073580 5 AG GG AG AG AG
AG AA AA AA AA AA AA AA
AA AA AA AG AG AA AA AA
AA AA AA AA AG AG AA AG
AG AA GG AG GG GG AG
172725229
3040 rs10073581 5 AG AG AG AG AG
AG AA AG GG AG AA AA AA
AA AA AG AA AG GG AA GG
AG GG GG AG AG GG AG AG
GG GG AA AG AA AA AA
111205268
3041 rs1007361 3 GG GG GG AG AG
GG AG GG GG GG GG GG GG
GG GG GG AG AG GG GG GG
GG GG GG GG GG GG GG GG

GG AG AG GG GG GG GG
28801369
3042 rs10073636 5 AC AA AC CC CC
CC CC AA AA AA CC CC CC
AC CC AA AC AC CC AC CC
AA AC AC AA AC CC CC AC
AA AA AC AA AC AC AA
45537299
3043 rs1007368 3 AG AG AG GG GG
GG GG GG GG GG AG AG GG
GG GG GG GG GG GG GG GG
GG GG GG GG GG AG AG AG
GG GG GG GG AG AA GG
93960849
3044 rs10073723 5 CC CC AC CC AC
AC AC AA AA AA CC CC AC
CC AC AC AC AA AA AA AC
AA AA AC AA CC AC AC CC
AC AC CC AC AA AC AA
29557328
3045 rs10073796 5 GG GG GG GG GG
AG GG GG GG GG GG GG GG
GG GG GG GG GG GG GG GG
GG GG GG GG GG GG GG GG
AG GG AG AG GG GG GG
170782385
3046 rs10073831 5 GG GG GG GG GG
GG GG GG GG GG GG GG GG
GG GG -- GG GG -- GG --
GG GG GG GG GG -- -- --
GG GG GG GG GG GG GG
27049259
3047 rs10073865 5 AA AA AA AA AA
AA AA AA AA AA AA AA AA
AA AA AA AA AA AA AA AA
AA AA AA AA AA AA AA AA
AA AA AA AA AA AA AA
179506234
3048 rs1007387 5 AA AA AA AG AG
AG AA AG AG AG AA AA AA
AA AA AG AG AG AA AA AA
AA AA AA AA GG AA AG AG
AG AA AG AA GG AG GG
174661472
3049 rs1007392 11 AG AG AA AA AA
AA AA GG GG GG AG AG GG
AG AG AG AG AA AG AG AG
AA AA AA AA AG AA AA AG
GG AG GG AG AG AG AG
14774591

3050 rs10073948 5 AG AG AG AG AG
AG AG AG AG GG AA GG AG
AA AA AA AA AA AG AG AA
AG AG AG AG GG GG GG GG
GG AA AG AA GG GG GG
82134620
3051 rs10073969 5 AC AA AC AC AA
AA AC AC AC AC AA AA AA
AC AC AC CC CC AA AA AA
AA AA AA AA AA AA AA AA
AC AA AA AA AC AA AC
124318270
3052 rs10074028 5 GG -- -- -- --
-- AG GG -- GG AG AG AG
AG AG -- -- -- GG AG GG
AG AG AG AG -- AG -- AG
AG GG AG AG GG AG --
89041960
3053 rs10074057 5 AA AA AA AA AA
AA AA AG AG AG AA AG AA
AA AA AA AA AA AG AG AA
AA AG AG AG AA AA AA AA
AA AG AA AG AA AA AA
123959513
3054 rs10074148 5 AG AG AG AA AG
AA AG GG GG GG AG AG GG
AG AG GG AG AG AA AA AG
AA AA AA AA AG GG GG GG
AG AG GG GG AA AG AA
167337407
3055 rs1007416 6 AA AA AA AG AA
AG AA AA AG AA AG AG AA
AA AA AA AA AG AA AA AA
AG AG AG AG AA AA AA AA
AA AA AA AA AA AA AA
80631931
3056 rs10074172 5 AA AA AA AA AA
AG AA AA AA AA AA AA AA
AA AA AA AA AA AA AA AA
AA AA AA AA AA AA AA AA
AA AG AA AA AA AA AA
1949753
3057 rs1007419 6 AG AG GG AA AG
AG AG AG AG AG GG GG GG
AG GG GG GG GG AA AA AA
AA AA AA AA AA GG AG AG
AG GG GG GG GG GG GG
62980430
3058 rs10074207 5 GG GG GG AG AG
GG AG GG GG GG GG GG GG

	GG	GG	AG	GG	AG	GG	AG	GG
	GG	GG	GG	GG	GG	GG	GG	GG
	AG	GG	GG	GG	AG	GG	AG	
91557158								
3059	rs1007429		21	GG	GG	GG	GG	GG
	AG	GG	GG	GG	GG	AG	GG	AG
	AG	GG	GG	GG	GG	GG	GG	GG
	GG	GG	GG	GG	GG	GG	GG	GG
	GG	GG	GG	GG	GG	GG	GG	
35034653								
3060	rs10074415		5	AG	GG	AG	GG	GG
	AG	GG	GG	GG	GG	GG	GG	GG
	GG	GG	GG	GG	GG	GG	GG	GG
	GG	GG	GG	GG	AG	GG	AG	GG
	AG	GG	GG	GG	GG	GG	GG	
73548364								
3061	rs10074448		5	AA	AA	AG	AA	AA
	AA	AA	AG	AG	AG	AG	AG	AG
	AG	AG	AG	AG	AG	AG	GG	AG
	AA	AA	AA	AA	AG	AG	AA	AG
	AG	GG	AA	AG	AA	AA	AA	
82897008								
3062	rs10074451		5	GG	GG	AG	GG	GG
	GG	GG	AA	AA	AA	AG	AG	AG
	AG	GG	GG	GG	GG	AG	AA	GG
	AG	GG	GG	AG	AG	GG	GG	AG
	GG	AG	AG	AG	GG	GG	GG	
33055680								
3063	rs10074464		5	AA	AG	AA	AG	AG
	AG	AA	AA	AA	AA	AA	AA	AA
	AA	AA	AA	AA	AA	AG	AG	AA
	AA	AA	AA	AA	AA	AA	AA	AA
	AA	GG	AA	AG	AG	AA	GG	
119877678								
3064	rs10074525		5	AA	AG	AA	AG	AG
	GG	AA	AA	AA	AA	AG	AA	AG
	AG	AG	GG	GG	GG	AA	AA	AA
	AA	AA	AA	AA	AA	GG	AG	AG
	AA	AG	AG	GG	AG	AA	GG	
90209824								
3065	rs10074559		5	AA	AG	AA	AG	AA
	AG	AG	AA	AA	AA	GG	AG	GG
	GG	GG	AG	AA	AA	AG	AG	AG
	AG	AG	AG	AG	GG	AG	AG	GG
	GG	AG	GG	GG	GG	AG	GG	
128532350								
3066	rs1007460		1	AA	AG	AA	GG	AG
	AG	GG	AG	GG	AG	GG	GG	GG
	GG	AG	AG	AA	AA	AG	AG	GG
	GG	AG	AG	AG	AG	AG	AG	AG

AG AA GG AG AG GG AG
193510827
3067 rs10074608 5 AC CC AC AC CC
AC CC CC CC CC CC CC AC
AA AA CC CC CC AC AA CC
AA AA AC AC AA AC AC AA
CC CC CC CC AA AC AC
145266174
3068 rs10074613 5 AA AA AA AA AA
AA AA AA AA AA AA AA AA
AC AC AA AA AA AA AA AA
AA AA AA AA AA AA AA AA
AA AA AC AA AA AC AA
10269935
3069 rs10074634 5 AA AA AA AA AA
AA AA AA AA AA AA AA AA
AA AA AA AA AA AA AA AA
AA AA AA AA AA AA AA AA
AA AA AA AA AA AA AA
57883211
3070 rs1007468 9 GG AG GG GG GG
GG GG GG GG AG AA AG AA
AA AG GG GG GG GG GG GG
GG GG GG GG GG AG GG GG
AG AG AG AA AG AG AG
104728905
3071 rs10074734 5 AA AG AG GG AG
AG AG AG AG AG AG AG AG
GG GG GG AG AG AG AG AG
AA AA AG AA AG AG AG GG
AG AG AG AG GG GG AG
29596752
3072 rs10074748 5 AA AG AG AG AG
AA GG GG GG AG AA GG AG
AA AA AA AG AG AG AG AA
AG AA AA AG GG AG GG AG
AG AG AA AA AA AA AA
57782141
3073 rs10074792 5 AG AA AA AA AA
AA AA AA AA AA AG GG AG
GG AG GG AG AG AA AG AA
AA AG AG AA AG AG AG AG
AA AA AA AA AG AG AG
51700018
3074 rs10074795 5 AA AA AA AA AA
AA AA AA AA AA AA AA AA
AA AA AA AA AA AA AA AA
AA AA AA AA AA AA AA AA
AA AA AA AA AA AA AA
39021044

3075	rs10074809		5	AG	AG	AG	GG	GG
	AG	GG	AG	GG	AG	GG	AG	GG
	GG	GG	GG	AG	AG	GG	GG	AG
	GG	GG	GG	GG	GG	AG	GG	AG
	AG	AG	GG	AG	AG	AA	GG	
95818902								
3076	rs10074909		5	AA	AA	AA	AG	AG
	AA	GG	AG	AA	AG	AA	AG	AA
	AA	AA	AA	AG	AG	AA	AA	AA
	AG	GG	GG	AG	AA	AA	AA	AA
	AA	AA	AA	AA	AA	AA	AA	
51724318								
3077	rs10074911							
0	--	--	--	--	--	--	--	--
	--	--	--	--	--	--	--	--
	--	--	--	--	--	--	--	--
	--	--	--	--	--	--	--	--
	--	--	--	--	0			
3078	rs10074959		5	AG	AG	AG	AG	AG
	AA	GG	AG	GG	AG	GG	GG	GG
	GG	GG	GG	AG	AG	GG	AA	GG
	GG	GG	GG	GG	GG	GG	GG	GG
	GG	GG	AA	AG	GG	AG	GG	
104208013								
3079	rs10074991							
5	--	--	--	--	--	--	--	--
	--	--	--	--	--	--	--	--
	--	--	--	--	--	--	--	--
	--	--	--	--	--	--	--	--
	--	--	--	--	40790551			
3080	rs10075011		5	AG	AG	AG	AA	AA
	AA	AA	AG	AA	AG	AA	AA	AA
	AG	AA	AG	AG	AG	AG	AA	GG
	AG	AG	AG	AG	GG	AA	AG	AG
	AG	AG	AA	AG	GG	GG	GG	
38690423								
3081	rs1007506		9	GG	GG	GG	GG	GG
	GG	GG	GG	GG	GG	GG	GG	GG
	GG	GG	GG	GG	GG	GG	GG	GG
	GG	GG	GG	GG	GG	GG	GG	GG
	GG	GG	GG	GG	GG	GG	GG	
100568134								
3082	rs10075062		5	AG	AA	AG	GG	GG
	AG	GG	AA	AG	AA	GG	GG	GG
	GG	GG	GG	AG	AG	GG	GG	GG
	GG	GG	GG	GG	AG	AA	AG	AG
	AG	GG	GG	GG	AG	AG	GG	
142514131								
3083	rs10075097		5	AA	AA	AA	AA	AA
	AA	AA	AC	AC	AC	AC	AA	AA

AA AA AA AA AA AA AA AA
AC AA AA AA AA AA AA AA
AA AA AA AA AA AA AC
126401214
3084 rs10075126 5 AA AA AA AA AA
AA AA AG AG AG AA AA AA
AA AA AG AG AA AA AA AA
AA AA AA AA AA AG AG AA
AA AA AA AA AA AA AA
21527725
3085 rs10075131 5 AG AG AG AA AA
AG AG AA AG AG AA AA AA
AA AA AG AG AA AA AG AG
AG AG AG AG GG AG GG AG
AA AG AA AA AG GG AG
92047795
3086 rs10075150 5 AG AG GG AG AG
AA AG AG GG AG AG GG AG
GG AG AG AG AA AG AG AG
AG AG AG AG AA AG AG AA
AG AG AG AG AA AG AG
36242354
3087 rs1007521 10 GG GG GG GG GG
GG GG GG GG GG GG GG GG
GG GG GG GG GG GG GG GG
GG GG GG GG GG GG GG GG
GG GG GG GG GG GG GG
82290663
3088 rs10075239 5 AC CC CC CC CC
AC CC AC CC AC CC AA AC
CC CC CC CC CC CC AC CC
CC CC AC CC CC AA AC AC
CC CC AA AC CC CC CC
1370300
3089 rs10075281 5 AA AA AG GG GG
GG GG AG GG AG GG GG AG
GG GG GG GG GG GG AG GG
AA AG AG AA AG GG GG GG
AG AG AG AG AG AG AG
112060242
3090 rs10075292 5 CC AC AC CC CC
CC CC CC CC CC AA AC AC
AC AC CC CC CC CC CC CC
CC CC CC CC CC AC AC CC
AC AC CC AC AC AC AC
20533037
3091 rs10075343 5 GG GG GG GG GG
GG GG GG GG GG GG GG GG
GG GG GG GG GG GG GG GG
GG GG GG GG GG GG GG GG

GG GG GG GG GG GG GG
38118423
3092 rs1007541 2 GG AG AG AG GG
AG GG GG GG GG AG GG GG
GG GG GG GG GG GG GG GG
GG GG GG GG AG GG GG AG
GG GG GG GG GG GG GG
49209034
3093 rs10075429 5 AG GG GG AG GG
AG AG GG GG GG GG GG GG
GG GG GG GG GG GG GG GG
GG GG GG GG GG GG GG GG
AG GG GG GG AG GG AG
41952322
3094 rs10075489 5 GG AG GG AG AG
AG GG GG GG GG GG GG GG
GG GG GG GG GG GG AG AG
AG AG AG AG AG AA AG AG
AG AG AG AA GG GG GG
88872383
3095 rs10075629 5 AG AA AA AA AA
AA AG AG AA AG AG AG AA
AA AA AA AA AA AA AA AA
AG AG GG AG AA AA AA AA
AG AA AA AA AA AA AA
12216062
3096 rs10075636 5 AA AG AG GG GG
GG GG AG AA AG GG AG AG
AG AG AG AG AA GG GG AG
GG GG GG GG GG AA AG AG
GG GG GG GG GG GG GG
2479446
3097 rs10075639 5 AA AA AA AG AA
AG AG AG AG AG AA AG AA
AG AA AG AG AA AA AA AG
AG AG AG AG AG AA AA AA
GG AG AA AG AG AG AG
28387115
3098 rs10075641 5 AA AG AA AA AA
AA AA AG AG AG GG AG GG
AG AG AA AA AA GG GG GG
AG AG AG AG AA AA AA AA
GG AG GG GG AG AG GG
124544884
3099 rs1007568 19 AG AG AG GG GG
GG GG AG GG GG AG AG AA
AA AG GG AG AG AG AG AG
GG GG GG GG GG GG GG GG
AG GG AG AG GG AG AG
35980832

3100 rs10075707 5 AG AG AA AA AA
AA AA AG AA AG AG AA AA
AG AG GG AG AG AG AA AG
AG AG AA AG AG AA AG AG
AG AG AA AG GG AG AG
31179728
3101 rs1007583 3 AA AG AG AA AG
AG AG AG AG AA AA AA AA
AG AA AG GG GG AG AG AG
GG AG GG AG AA AA AA AA
AA AG AA AG AA AA AA
68207759
3102 rs10075839 5 GG GG GG AG AG
AG GG GG GG GG GG GG GG
GG GG GG GG GG AG AA GG
GG AG GG GG GG GG GG GG
GG GG GG GG AG GG AG
71166100
3103 rs1007586 5 AA AA AA AA AA
AA AA AA AA AA AA AA AA
AA AA AA AA AA AA AA AA
AA AA AA AA AA AA AA AA
AA AA AA AA AG AG AA
26942758
3104 rs10075874 5 AA AA AA AA AA
AA AA AC AA AC AA AA AA
AA AA AC AA AA AA AA AA
AA AA AA AA AA AA AA AA
AA AA AA AA AA AA AC
41079845
3105 rs10075914 5 GG GG GG AG AG
AG GG GG AG GG GG GG AG
GG AG GG GG GG AG AA GG
GG GG GG GG AG AG GG AA
AG GG GG GG GG GG AG
34525931
3106 rs10075926 5 AC AC AC AC AA
AA AC AC AC AC AC AC AA
AC AC AC CC AC AA AC AA
CC AC AC CC AA AA AA AA
AC AC CC CC CC CC AC
111590241
3107 rs10075958 5 AC AC AC AC AC
AA CC AC AC AC AC AC AC
AC CC CC AC AC CC AC AC
CC CC CC CC AC CC CC AC
CC AC CC CC AC AA CC
160494759
3108 rs10076 13 AA AA AA AG AA AG
AA AA AA AA AA AA AA AA

	AA	AA	AA	AA	AA	AA	AA	AA
	AA	AA	AA	AA	AA	AA	AA	AA
	AG	AA	AA	AA	AA	AA	49079503	
3109	rs10076027		5	AA	AA	AA	AA	AA
	AG	AA	AG	AG	AG	AG	AG	GG
	AG	AG	AG	AA	AG	AG	AA	AA
	AA	AA	AA	AA	AG	AG	AA	AG
	AA	AG	AA	AG	AG	GG	AG	
172956807								
3110	rs1007603		6	GG	GG	GG	GG	GG
	GG	GG	GG	GG	GG	GG	GG	AG
	GG	AG	GG	GG	GG	GG	GG	GG
	GG	GG	GG	GG	AG	GG	AG	AG
	GG	GG	GG	GG	GG	AG	GG	
95946807								
3111	rs10076099		5	AA	AC	AC	AA	AA
	AA	AA	AA	AA	AA	AA	AA	AA
	AA	AA	AA	AA	AA	AA	AA	AA
	AA	AA	AA	AA	AA	AA	AA	AA
	AA	AA	AA	AA	AA	AA	AA	
8928848								
3112	rs10076133		5	GG	GG	GG	AA	AA
	AG	AG	GG	GG	GG	GG	GG	GG
	GG	GG	AG	AG	GG	GG	GG	GG
	GG	GG	GG	GG	GG	GG	GG	GG
	GG	GG	GG	GG	GG	GG	GG	
178103348								
3113	rs10076201		5	GG	GG	GG	GG	GG
	GG	GG	AG	AG	AG	GG	GG	GG
	AG	AG	AG	AA	AG	GG	GG	GG
	AG	GG	GG	GG	GG	GG	GG	GG
	AG	AA	AG	AG	AG	AG	GG	
115935225								
3114	rs10076206		5	AG	AG	AA	AA	AA
	AG	AG	AA	AA	AA	AA	AG	AG
	AG	AG	AG	AA	AG	AA	AG	AA
	AG	AG	GG	AG	GG	GG	GG	GG
	AG	GG	GG	GG	AG	AG	AG	
2164702								
3115	rs1007630		1	AG	AA	AG	AA	AG
	AA	AG	AA	AG	AG	AG	AG	AG
	GG	AG	GG	GG	AG	GG	GG	GG
	AA	AA	AA	AA	GG	GG	GG	GG
	AG	AA	AG	AA	AG	GG	AA	
71085728								
3116	rs10076322		5	GG	GG	GG	AG	AG
	AG	AA	AG	AG	AG	AA	AA	AA
	AG	AG	AA	AA	AA	GG	AG	AG
	AG	AG	AG	AG	AG	GG	GG	AG
	AA	AG	AA	AG	AG	GG	AA	

123446185							
3117	rs10076325	5	AG	AA	AG	GG	GG
	GG	AG	GG	GG	GG	GG	GG	GG
	GG	GG	AG	GG	AG	AG	AG	AG
	GG	GG	GG	GG	GG	GG	GG	GG
	GG	AG	AG	AG	GG	GG	GG	
30389412							
3118	rs10076327	5	GG	GG	GG	AG	GG
	GG	AG	GG	GG	GG	GG	GG	GG
	GG	GG	AG	AG	AG	GG	GG	AG
	GG	GG	GG	GG	GG	GG	GG	GG
	AA	GG	GG	GG	GG	AG	GG	
122367347							
3119	rs10076380	5	AA	AA	AA	AG	AG
	AG	AG	AA	AA	AA	AG	AA	AG
	AG	AG	AG	AA	AA	AA	AA	AA
	AG	GG	GG	AG	AG	AA	AG	AA
	AA	AG	AA	AA	AA	AA	AA	
44088038							
3120	rs10076400	5	AG	AG	GG	AG	GG
	AG	GG	AG	AG	AG	GG	AG	GG
	GG	GG	GG	GG	GG	GG	GG	GG
	AA	AA	AG	AA	GG	GG	GG	GG
	GG	AG	GG	GG	AG	GG	AG	
25745902							
3121	rs10076408	5	AG	AG	AG	AA	AA
	AA	AA	AA	AA	AA	AA	AA	AA
	AA	AA	AA	AA	AA	AG	GG	AA
	AA	AA	AA	AA	GG	AG	AG	GG
	AG	AA	GG	AG	AG	AG	GG	
39576829							
3122	rs10076425	5	GG	GG	AG	GG	AG
	AG	AG	AG	AG	AG	AG	AG	AA
	AA	AA	GG	AG	AG	AA	GG	AA
	GG	GG	GG	GG	AA	AG	AG	AA
	GG	AG	GG	GG	AG	GG	AG	
117227611							
3123	rs10076429	5	AC	AC	CC	AC	AC
	CC	AA	AC	AC	AC	CC	AC	CC
	CC	CC	CC	CC	CC	AA	CC	AC
	AC	AA	AA	AC	AC	CC	CC	CC
	CC	CC	CC	CC	AC	AC	AC	
84003689							
3124	rs10076494	5	GG	GG	GG	AG	AG
	AG	GG	GG	GG	GG	GG	GG	GG
	AG	AG	AA	AG	AG	GG	GG	AG
	GG	GG	GG	GG	GG	GG	GG	GG
	GG	GG	GG	GG	AG	AA	GG	
38139							
3125	rs10076568						

5	--	--	--	--	--	--	--	--
	--	--	--	--	--	--	--	--
	--	--	--	--	--	--	--	--
	--	--	--	--	--	--	--	--
	--	--	--	--	60471080			
3126	rs10076581		5	GG	GG	GG	GG	GG
	GG	GG	GG	GG	GG	GG	GG	GG
	GG	GG	GG	GG	GG	GG	GG	GG
	GG	GG	GG	GG	GG	GG	GG	GG
	GG	GG	GG	GG	GG	GG	GG	
172389179								
3127	rs10076602		5	AC	AC	AC	AA	AC
	AA	AC	CC	CC	CC	CC	CC	CC
	AC	AC	AC	AA	AC	AC	AA	AA
	AC	AC	AC	AC	AC	CC	CC	AC
	CC	CC	CC	CC	CC	CC	CC	
153902050								
3128	rs10076713		5	GG	AG	GG	GG	GG
	GG	GG	AG	AA	AG	GG	AG	AG
	AA	AG	AG	AG	AG	AG	GG	AG
	GG	AG	AG	GG	GG	AG	AG	GG
	AG	GG	AA	AG	GG	GG	GG	
39924922								
3129	rs1007684		2	AG	AG	AG	AG	AG
	AG	AG	GG	AG	AG	AA	GG	AG
	GG	AG	GG	AG	GG	AA	AG	AG
	AA	AA	AA	AG	AA	GG	AG	AG
	AA	AG	GG	GG	AG	AG	AG	
220790637								
3130	rs10076865		5	AG	AA	AG	AA	AG
	AA	AG	GG	GG	GG	AG	GG	GG
	GG	GG	AA	AA	AA	AA	AA	AA
	AG	AG	AG	AG	AA	AA	AA	AA
	AA	GG	AG	AG	AA	AG	AA	
123851734								
3131	rs10076876		5	GG	GG	GG	GG	GG
	GG	GG	GG	GG	GG	GG	GG	GG
	GG	GG	GG	GG	GG	GG	GG	GG
	GG	GG	GG	GG	GG	GG	GG	GG
	GG	GG	GG	GG	GG	GG	GG	
28526981								
3132	rs1007690		11	GG	AG	GG	AG	AA
	AG	AA	AG	AG	AA	AG	AG	AA
	AA	AG	AG	AG	AA	AG	GG	AA
	AA	AA	AA	AA	AA	AG	AA	AG
	AA	AA	AG	AG	AG	AG	AA	
126274003								
3133	rs10076901		5	AA	AA	AA	AA	AA
	AA	AA	AA	AA	AA	AA	AG	AG
	AG	AA	AA	AA	AA	AA	AA	AA

	AA	AA	AA	AA	AA	AA	AA	AA
	AA	AA	AA	AA	AA	AA	AA	
51640899								
3134	rs1007691		8	AC	AC	AC	AC	AC
	AA	AC	AA	AC	AC	AA	AC	AA
	AA	AA	AC	AA	AA	AA	AC	AC
	AC	AC	AC	AC	AC	AC	AC	CC
	CC	CC	AC	CC	AC	AC	CC	
17344517								
3135	rs10076926		5	GG	GG	GG	GG	GG
	GG	GG	GG	GG	GG	GG	GG	GG
	GG	GG	GG	GG	GG	GG	GG	GG
	GG	GG	GG	GG	GG	GG	GG	GG
	GG	GG	GG	GG	GG	GG	GG	
42607899								
3136	rs10076963		5	AG	AG	GG	AG	AG
	GG	AG	GG	GG	GG	AG	AA	AG
	AG	GG	AA	AG	AG	AG	AG	AG
	GG	GG	GG	GG	AG	GG	AG	AG
	GG	AG	AG	AG	AG	GG	AG	
25658882								
3137	rs1007702		7	CC	CC	CC	CC	CC
	CC	CC	CC	CC	CC	CC	CC	CC
	CC	CC	CC	CC	CC	CC	CC	CC
	CC	CC	--	CC	CC	CC	CC	CC
	CC	CC	CC	CC	CC	CC	CC	
50565610								
3138	rs10077097		5	GG	AG	GG	AG	AA
	AG	AG	GG	GG	GG	AG	AG	AG
	AA	AA	GG	AG	AG	GG	GG	GG
	AG	AG	AG	AG	GG	AG	AG	AG
	AG	AG	GG	AG	AG	AG	GG	
166775284								
3139	rs10077147		5	AG	GG	AG	GG	GG
	GG	GG	GG	GG	GG	GG	GG	GG
	GG	AG	GG	GG	GG	GG	GG	GG
	GG	GG	GG	GG	AG	AG	AG	AG
	GG	AG	GG	AG	GG	AG	GG	
32326234								
3140	rs10077158		5	GG	GG	AG	GG	GG
	GG	GG	GG	GG	GG	AG	GG	GG
	AG	GG	AG	AG	AG	AG	AG	GG
	GG	GG	GG	GG	GG	GG	GG	GG
	GG	GG	GG	GG	GG	GG	GG	
39706933								
3141	rs10077174		5	AA	AA	AA	AA	AA
	AA	AA	AA	AA	AA	AA	AA	AA
	AA	AA	AA	AA	AA	AA	AA	AA
	AA	AA	AA	AA	AA	AA	AA	AA
	AA	AA	AA	AA	AA	AA	AA	

109487061
3142 rs10077209 5 CC CC CC CC CC
CC CC CC CC CC CC CC CC
CC CC CC CC CC CC CC CC
CC CC CC CC CC CC CC CC
CC CC CC CC CC CC CC
18469760
3143 rs1007725 20 AG GG AG AA AG
AG AA GG GG GG AA AA AG
AG AG AA AA AA AA AG AG
AA GG AA AG AG AA AA AG
AG AA GG AG AA AA AA
55401450
3144 rs10077254 5 AG AG AA GG GG
GG GG GG GG GG GG GG GG
GG GG GG GG GG AG GG AG
AG AA AG AG AG AG AG GG
AG AG GG AG GG GG AG
173006447
3145 rs10077302 5 AA AA AG AA AA
AA AA AA AA AA AA AG AG
AA AA AA AA AA AG AA GG
AA AA AA AA AA AG -- AG
AA AA AA AA GG AG AG
75923842
3146 rs10077352 5 AA AA AG AA AA
AA AG AG AG AG AA GG AG
AA AA AG AA AA AG GG AG
AG AG AG AG AG AG AG AA
AG AA AA AA AG AA GG
82171406
3147 rs10077377 5 GG GG GG GG GG
GG GG GG GG GG GG GG GG
GG GG GG GG GG GG GG GG
AG AG AG AG AG GG AG GG
GG GG GG GG GG GG GG
171839729
3148 rs100774 3 AA AA AG AA AA AA
AA GG AG AG AA AA AA AG
AG AG GG AA AA AA AA AG
AA GG AG AG AA AA AA AG
AG AG AG AG AG AA 193358734
3149 rs10077401 5 AA AA AA GG GG
AG AG AG AG AG AA AA AA
AA AA AA AA AA AA AG AA
AG AG AA AA AA AA AA AA
AG AA AG AG AG AG AG
175072244
3150 rs1007744 X GG GG GG GG GG
GG GG GG GG GG AG AG GG

	GG	GG	GG	GG	GG	GG	GG	GG
	AA	AA	AA	AG	AG	GG	GG	AA
	GG	AG	AA	GG	GG	GG	GG	
10136628								
3151	rs10077440		5	GG	GG	GG	GG	GG
	AG	AG	AA	AA	AA	GG	GG	GG
	GG	GG	GG	AG	AG	GG	GG	GG
	GG	AG	GG	GG	AA	AA	AA	AA
	AA	AG	AA	AG	AG	GG	AA	
170869582								
3152	rs10077453		5	GG	GG	GG	--	--
	--	--	AG	GG	AG	GG	AG	--
	GG	--	GG	GG	GG	GG	AG	GG
	--	--	GG	GG	GG	GG	GG	GG
	GG	GG	AG	AG	--	GG	GG	
114580652								
3153	rs10077457		5	GG	GG	GG	GG	GG
	GG	GG	GG	GG	GG	GG	GG	GG
	GG	GG	GG	GG	GG	GG	GG	GG
	GG	GG	GG	GG	GG	GG	GG	GG
	GG	GG	GG	GG	GG	GG	GG	
13714568								
3154	rs10077466		5	AA	AA	AA	AG	AG
	AA	GG	AA	AA	AA	AG	AG	GG
	AG	AA	AG	AG	AA	AG	AG	AA
	GG	GG	GG	GG	AG	AG	AA	AG
	AG	AA	AG	AG	AG	GG	AG	
162617260								
3155	rs10077481		5	AG	GG	GG	AG	GG
	AG	AG	GG	GG	GG	GG	GG	GG
	GG	GG	GG	GG	GG	GG	GG	GG
	GG	GG	GG	GG	GG	GG	GG	GG
	AG	GG	GG	GG	AG	GG	AG	
41918457								
3156	rs10077532		5	CC	CC	AC	CC	CC
	CC	CC	CC	CC	CC	CC	AC	CC
	CC	CC	CC	AC	AC	AC	CC	AC
	AC	CC	CC	AC	AC	CC	AC	AC
	CC	CC	AC	AC	CC	CC	CC	
94716149								
3157	rs1007754		17	GG	AG	AG	AA	AA
	AG	AA	AA	AA	AA	AA	AA	AA
	AA	AA	AA	AA	AG	AA	AA	AA
	AG	AA	AG	AG	AA	AA	AA	AA
	AA	AA	AA	AA	AA	AA	AA	
75995073								
3158	rs10077572		5	AA	AA	AA	AC	AC
	AC	CC	AC	AA	AA	AA	AC	AC
	AC	AC	AA	AA	AA	AA	AC	AC
	AC	AC	CC	CC	AC	AA	AA	AA

AC AC AA AA AC AC AC
132748007
3159 rs10077631 5 AG AG AG AA AA
AA AA AA AA AA AA AA AA
AG AA AG AG GG AG AA AG
AA AA AA AA AA AG AG AG
AG AG AG AG AA AA AA
167842039
3160 rs10077665 5 GG GG GG GG GG
GG GG AG AG AG AG GG GG
GG GG AA AG AG AG GG AG
AG AG AG AG GG GG GG GG
AG AG GG AG GG GG GG
66993865
3161 rs10077690 5 AA AA AA AA AA
AA AA AA AA AA AG AG AG
AA AA AG AA AG AA AA AA
AA AA AA AA AG AG GG GG
AA AA AG AA AA AA AA
147788798
3162 rs10077711 5 GG GG GG GG GG
GG GG GG GG GG GG GG GG
GG GG GG GG GG GG GG GG
GG GG GG GG GG AG GG GG
GG GG GG GG GG GG GG
179587749
3163 rs10077835 5 AA AA AA AA AA
AA AA AA AA AA AA AG AA
AA AA AG AG AG AA AG AA
AA AA AA AA AA AG AG AA
AA AA AA AA AA AG AA
116279948
3164 rs10077851 5 GG GG AG GG GG
GG GG AG GG AG GG GG GG
GG GG AG GG AG AG AG AG
GG GG GG GG GG GG GG GG
AA GG GG GG GG GG GG
94753303
3165 rs10077854 5 AA AA AA AA AA
AG AA AG AG AG AA AA AA
AA AA AG AA AG AA AA AA
AA AA AA AA AA AA AA AA
AA AA AA AA AA AA AA
171502332
3166 rs10077929 5 AA AA AA AA AA
AA AA AG AG AG AA AA AA
AA AA AA AA AA AA AG AA
AG AG AG AG AG AA AA AG
AA AG AG AG AG AG AG
176697844

3167 rs10077940 5 AC CC AC CC CC
CC CC AA AC AA AC AA AC
AC CC AA AA AA AA AA AA
AA AA AA AA AC CC AC AC
AC AC AA AC AC AC AC
28942952
3168 rs10077950 5 GG AG AG AG AG
AA GG GG GG GG AA AA AG
GG GG AG GG AG AG AG AG
AG AG AG AG GG AG AG AG
AA AG GG AG AA AA AA
178283345
3169 rs10077953 5 AA AA AA AA AA
AA AA AA AA AA AA AA AA
AA AA AA AA AA AA AA AA
AA AA AG AG AA AA AA AA
AA AA AA AA AG AG AA
147536452
3170 rs10077978 5 AA AA AA AA AA
AA AA AC AC AC AA AA AA
AA AA AA AC AC AA AA AA
AA AA AA AA AA AA AA AA
AA AA AA AA AA AA AA
128618834
3171 rs10077985 5 GG GG GG GG GG
GG GG GG GG GG GG GG GG
GG GG GG GG GG GG AG GG
GG GG GG GG GG GG GG GG
GG GG GG GG GG GG GG
148540904
3172 rs10078091 5 GG GG GG AG AG
GG AG GG AG GG GG GG AG
AG AG GG AG AG GG AG GG
GG GG GG GG GG AG AG GG
AG GG AA AG AG GG AG
25495005
3173 rs10078192 5 AA AG AG AA AA
AA AA AA AA AA AA AA AG
AA AG AG AA AG AG AA AG
AA AA AA AA AA AA AA AA
AA AA AA AA AA AA AA
14116396
3174 rs10078211 5 GG GG GG GG GG
GG AG GG GG GG GG GG GG
GG GG AG AG GG GG GG GG
GG GG GG GG GG AG AG GG
AG AA GG AG AG AG GG
57964018
3175 rs10078248 5 GG GG GG GG GG
GG GG GG GG GG AG AG AA

	GG	AG	GG	GG	GG	GG	GG	GG
	GG	GG	GG	GG	GG	GG	GG	GG
	AG	GG	AG	GG	AG	GG	AG	
23709748								
3176	rs10078270		5	GG	GG	GG	GG	GG
	GG	GG	GG	GG	GG	GG	GG	GG
	GG	GG	GG	GG	GG	GG	GG	GG
	GG	GG	GG	GG	GG	GG	GG	GG
	GG	GG	GG	GG	GG	GG	GG	
35511098								
3177	rs10078274		5	GG	GG	GG	AA	AA
	AG	AA	GG	GG	GG	AG	GG	GG
	AG	AG	AA	AA	AA	AG	AA	GG
	AA	AA	AA	AA	AA	AG	AA	AG
	GG	AG	GG	AG	AA	AA	AA	
126568929								
3178	rs10078322		5	AA	AA	AA	AC	AC
	AA	AC	AA	AA	AA	AA	AA	AA
	AA	AA	AA	AA	AA	AA	AC	AA
	AA	AA	AA	AA	AC	AC	AC	CC
	AA	AA	AA	AA	AA	AA	AA	
173163893								
3179	rs1007834		9	AG	AA	AG	AG	AG
	AA	AG	AG	GG	AG	GG	AG	AG
	AG	GG	AG	AA	AA	GG	GG	AG
	AA	AA	AG	AA	GG	AG	AG	AG
	AG	GG	AG	AG	AA	AA	AA	
120029861								
3180	rs10078386		5	AA	AA	AA	AA	AA
	AC	AA	AA	AA	AA	AC	AC	AA
	AA	AA	AA	AA	AA	AA	AA	AA
	AC	AA	AA	AA	AC	AA	AA	AC
	AA	AA	AA	AA	AA	AA	AA	
124253737								
3181	rs1007841		14	AC	AA	AA	AA	AC
	AC	AA	AC	AC	AC	AA	AA	AC
	AA	AA	AA	AA	AA	AA	AA	AA
	AC	AA	AC	AA	AC	AA	AA	AA
	AC	AA	AA	AA	AC	AA	AC	
69158128								
3182	rs10078424		5	GG	GG	GG	AG	AG
	AA	AG	AG	AG	AG	AG	AG	AA
	AG	AG	AA	AG	AG	AG	GG	AG
	AG	AG	AG	AG	AG	AG	AG	GG
	GG	AG	AG	GG	AA	AA	AA	
171936932								
3183	rs10078499		5	GG	GG	GG	GG	GG
	GG	GG	GG	GG	GG	AA	AG	AG
	AG	AG	GG	GG	GG	GG	GG	GG
	GG	GG	GG	GG	GG	GG	GG	GG

GG AG AG AG AA AG AG
138020898
3184 rs10078551 5 AG AA AG AA AA
AA AA GG GG GG AG AG AA
AA AA AA AA AA AG AG AA
AG AG GG GG AG AG AG AG
AG AG AG AA GG GG AG
148001470
3185 rs10078715 5 GG GG GG GG GG
GG GG AG AG GG GG GG GG
GG GG AG AG AG AG AG AG
GG GG GG GG GG GG GG GG
GG GG GG GG GG GG GG
77290902
3186 rs10078815 5 AA AG AG GG GG
AG GG AG AG AG AA AG AA
AA AA GG AG AG AG AG AA
AG AG AG AG AG AG AA AG
AG GG GG GG AG GG AG
78522895
3187 rs1007892 9 AC CC AC CC AA
AC AC AC CC AA AC CC CC
CC AA AC CC CC CC CC CC
CC CC CC CC CC AC CC CC
CC AA AC AC AC AC AC
101649752
3188 rs1007894 14 GG GG GG AG GG
GG AG GG GG GG GG GG GG
GG AG GG GG AG GG GG GG
GG GG GG GG AG GG AG AG
AA AG GG GG GG GG GG
53094595
3189 rs10078948 5 AA AA AA AA AA
AA AA AA AA AA AA AA AA
AA AA AA AA AA AA AA AA
AA AA AA AA AA AA AA AA
AA AA AA AA AA AA AA
112815689
3190 rs1007901 2 GG GG GG GG GG
GG GG GG GG GG GG GG GG
GG GG GG AG AG GG GG GG
GG GG GG GG GG GG GG GG
GG GG GG GG GG GG GG
84248392
3191 rs1007907 9 AA AA AA GG GG
GG AG AA AG AG AA AA AA
AA AA AG AG AG AA AG AA
AA AA AA AA AA AG AG AG
AA AA AG AG AG AA AG
19760153

3192	rs1007924	12	AA	AA	AA	AA	AG
	AG	AA	AA	AG	AG	AA	GG	AG
	GG	GG	AA	AA	AA	AG	AG	AA
	GG	GG	GG	GG	AG	AA	AG	AA
	AA	AA	AA	AA	AA	AA	AA	
7015915								
3193	rs10079256	5	GG	GG	GG	GG	GG
	GG	GG	GG	GG	GG	GG	GG	GG
	GG	GG	GG	GG	GG	GG	GG	GG
	GG	GG	GG	GG	GG	GG	GG	GG
	GG	GG	GG	GG	GG	GG	GG	
45135612								
3194	rs10079374	5	AG	AG	AG	AG	AG
	GG	AG	GG	GG	GG	GG	GG	GG
	GG	GG	GG	AG	AG	AG	AG	AA
	AG	AG	AG	AG	GG	AG	GG	AG
	AG	AG	GG	GG	GG	AG	GG	
17828138								
3195	rs1007938	12	AA	AA	AA	AA	AA
	AA	AG	AA	AG	GG	AA	AG	AG
	AG	AG	AA	AG	AG	AA	AA	AG
	AG	AG	GG	AA	GG	AA	AG	AG
	AG	AA	AG	AA	GG	GG	AG	
26802549								
3196	rs10079401							
5	--	--	--	--	--	--	--	--
	--	--	--	--	--	--	--	--
	--	--	--	--	--	--	--	--
	--	--	--	--	--	--	--	--
	--	--	--	--	30725417			
3197	rs10079469	5	AA	AC	AA	AA	AA
	AA	AA	CC	AC	CC	AA	AC	AA
	AA	AA	AA	AA	AA	AC	AA	CC
	AC	AC	AC	AC	AC	CC	AC	AC
	AC	AA	AA	AA	AA	AA	AA	
99772694								
3198	rs10079494	5	AG	AG	GG	GG	GG
	AG	GG	GG	GG	GG	AA	GG	AG
	AA	AA	AG	AG	AG	AG	AG	GG
	GG	GG	GG	GG	GG	GG	GG	GG
	AG	GG	AG	GG	GG	GG	AG	
80217130								
3199	rs10079562	5	AC	AC	CC	AC	AC
	CC	AA	AC	AA	AC	AC	AC	AA
	AA	AA	AC	AC	AA	AC	AC	AA
	CC	CC	CC	--	AA	AA	AA	AA
	AC	AA	AA	AA	AC	AC	AC	
2694637								
3200	rs10079576	5	AA	AA	AA	AA	AA
	AA	AA	AA	AA	AA	AG	AG	AG

AA AA AA AA AA AA AA AA
AA AA AA AA AA AA AA AA
AA AA AA AA AA AA AA
24801690
3201 rs10079610 5 AG GG GG AG AG
GG AA AG AA AG AG AG AA
AA AA AG AG AA GG GG AG
GG GG GG GG AA AA AA AA
AG AA AG AG AG AG AG
2694684
3202 rs1007966 9 AG AG AG AA AA
AA AA AA AG AG GG AA AG
AG AG AG AG GG GG GG GG
AG AA AG AA AG AA AG AA
AG AG GG AG GG GG GG
92213967
3203 rs10079668 5 GG GG GG GG GG
GG GG GG GG GG GG GG GG
GG GG GG GG GG GG GG GG
GG GG GG GG GG GG GG GG
GG GG GG GG GG GG GG
36590934
3204 rs10079678 5 AA AA AA AA AA
AA AA AA AA AA AA AA AA
AA AA AA AA AA AA AA AA
AA AA AA AA AA AA AA AA
AA AA AA AA AA AA AA
129506648
3205 rs10079693 5 AG AG GG AG AG
GG AG GG GG GG GG GG GG
GG GG GG GG GG GG AG GG
GG GG GG GG GG AG AG AG
GG AG GG GG GG GG GG
175131097
3206 rs10079767 5 AA AA AA AA AA
AA AA AA AA AA AA AC AA
AA AA AC AC AA AA AA AA
AA AA AA AA AA AA AA AA
AA AA AA AA AA AA AA
26859083
3207 rs10079783 5 AA AA AA AA AA
AA AA AA AA AC AA AA AA
AA AA AA AA AA AA AA AA
AA AA AA AA AA AA AA AA
AA AA AA AA AA AA AA
54790559
3208 rs10079802 5 GG GG GG GG GG
GG GG GG GG GG GG GG GG
GG GG GG GG GG GG GG GG
GG GG GG GG GG GG GG GG

GG GG GG GG GG GG GG
50678363
3209 rs10079806 5 AA AA AA AA AA
AA AA AA AA AA AA AA AA
AA AA AC AC AA AA AA AC
AC AC CC CC AC AC CC AC
AA AA AA AA AC AC AA
135335229
3210 rs10079815 5 GG GG GG GG GG
GG GG GG GG GG GG GG GG
GG GG GG GG GG GG GG GG
GG GG GG GG GG GG GG GG
GG GG GG GG GG GG GG
41771730
3211 rs1007984 5 CC CC CC AC AC
AC AA CC CC CC CC AC CC
CC CC CC AC AC AC CC AC
CC CC CC CC CC CC CC CC
AC CC AC AC CC AC CC
52636896
3212 rs10079855 5 CC CC CC CC CC
CC CC AC AC AC CC CC CC
CC CC AC AC AC CC CC CC
CC CC CC CC CC CC CC CC
CC CC CC CC CC CC CC
75809208
3213 rs10079889 5 AG AA AA AA AA
AG AA AA AG AA AA AA AG
AA AG AA AA AA AG AG AG
AA AA AA AA AA AA AA AA
AG GG AG AG AA AG AA
32975490
3214 rs1007995 9 AG AG AG AG AG
AA GG GG AG GG AG AG GG
GG AG AG AG AA AG AG GG
AG AA GG AG AG AA AA AG
GG AA AA AA AG AA GG
84371927
3215 rs10079950 5 GG GG GG GG GG
GG GG GG GG GG GG GG GG
GG GG AG AG GG GG GG GG
GG GG GG GG GG GG GG GG
GG GG GG GG GG GG GG
20322049
3216 rs10079988 5 AA AA AA AA AA
AA AA AA AA AA AA AA AA
AA AA AA AA AA AA AA AA
AA AA AA AA AA AA AA AA
AA AA AA AA AA AA AA
33512325

3217	rs10079990		5	AC	AC	CC	AA	AC
	AC	AC	AA	AC	AA	AC	AC	AC
	AC	AC	CC	AC	AC	AC	AC	AC
	AC	AA	AA	AC	AC	CC	AC	AC
	AC	AC	CC	CC	AC	AC	AC	
107437025								
3218	rs10080044		5	GG	GG	GG	AA	AA
	AG	AG	GG	AG	GG	AG	AG	AG
	AA	GG	GG	GG	GG	GG	GG	GG
	GG	GG	GG	GG	GG	AG	AG	GG
	AG	GG	GG	GG	AG	AG	GG	
50533202								
3219	rs10080049		5	AG	GG	AG	AA	AA
	AG	AG	AG	AG	AG	AA	AA	AG
	AG	AG	AA	AG	AA	AA	AA	AG
	GG	GG	AG	AG	AG	AA	AG	AA
	AA	AG	AA	AG	AG	AA	AG	
177648188								
3220	rs10080058		5	CC	CC	CC	CC	CC
	CC	AC	CC	CC	CC	CC	CC	CC
	CC	CC	CC	CC	CC	CC	CC	CC
	CC	CC	CC	CC	CC	CC	CC	CC
	CC	CC	CC	CC	CC	CC	CC	
13987061								
3221	rs1008007		14	AG	AG	AG	GG	AG
	GG	AG	AG	AG	AG	GG	GG	GG
	AG	AG	GG	GG	GG	AA	AG	AA
	GG	AG	GG	AG	GG	AG	AG	GG
	AG	GG	GG	GG	AG	AG	GG	
75026597								
3222	rs10080096		5	AC	AC	AC	CC	AC
	AC	AC	AC	AC	AC	AA	AC	AA
	AA	AA	AA	AC	AC	AC	AC	AA
	AC	AC	AC	AC	AA	AA	AA	AA
	AA	AA	AA	AA	AA	AA	AC	
112879405								
3223	rs1008012		12	AG	AA	AG	AA	AA
	AA	AA	AA	AG	AG	AA	AA	AG
	AG	AG	AG	AG	AG	AG	AG	AA
	AA	AA	AA	AG	AA	AG	AG	AG
	AA	AA	AG	AG	AA	AA	AA	
33244179								
3224	rs10080135		5	GG	GG	GG	GG	GG
	GG	AG	GG	GG	GG	AG	AG	AG
	AG	GG	GG	GG	GG	GG	GG	AG
	AG	AA	AA	AG	GG	AG	AG	GG
	AG	GG	GG	GG	GG	GG	GG	
35231987								
3225	rs10080168		5	GG	GG	GG	AG	GG
	AG	AG	GG	GG	GG	GG	GG	AG

	GG	AG	GG	GG	GG	GG	GG	GG
	GG	GG	GG	GG	AG	GG	GG	GG
	GG	AG	GG	AG	GG	GG	GG	
166649746								
3226	rs1008023		5	AA	AC	AC	CC	CC
	CC	CC	CC	CC	CC	CC	CC	CC
	CC	CC	CC	CC	CC	CC	CC	CC
	CC	CC	CC	CC	CC	CC	CC	CC
	AC	CC	CC	CC	CC	CC	CC	
32525644								
3227	rs10080234		6	AA	AA	AA	AA	AA
	AA	AA	AA	AA	AA	AA	AA	AA
	AA	AA	AA	AA	AA	AC	AC	AA
	AA	AA	AA	AA	AA	AA	AA	AA
	AA	AA	AA	AA	AA	AA	AA	
101043477								
3228	rs10080237		6	AG	AA	AG	AG	AG
	GG	AA	AG	GG	AG	AG	AG	AG
	AG	AG	AG	GG	GG	AG	AG	GG
	AG	AG	AG	AG	AG	AA	AG	AG
	AG	AA	AA	AA	GG	GG	GG	
80854470								
3229	rs10080265		6	AA	AC	AC	AA	AA
	AA	AA	AC	AA	AC	AC	AC	AA
	AA	AA	AC	AA	AA	AA	AA	AA
	AA	AA	AA	AA	AA	AA	AA	AA
	AA	AC	AA	AC	AA	AA	AA	
53917787								
3230	rs10080326		6	AA	AA	AA	AA	AA
	AA	AA	AA	AA	AA	AA	AA	AA
	AA	AA	AA	AA	AA	AA	AA	AA
	AA	AA	AA	AA	AA	AA	AA	AA
	AA	AA	AA	AA	AA	AA	AA	
135400119								
3231	rs10080352		6	GG	GG	GG	GG	AG
	AG	GG	GG	GG	GG	AG	AG	GG
	GG	GG	GG	GG	GG	GG	GG	GG
	GG	GG	GG	GG	GG	GG	GG	GG
	GG	GG	GG	GG	GG	GG	GG	
126197049								
3232	rs10080387		6	AA	AA	AA	AA	AA
	AA	AA	AG	AA	AG	AA	AA	AA
	AA	AA	AA	AG	AA	AA	AA	AG
	AG	AG	AA	AG	AA	AG	AA	AA
	GG	AA	AG	AA	AG	AG	AG	
119391206								
3233	rs10080512		6	AG	GG	AG	GG	GG
	GG	AG	AG	AG	AG	AA	AG	AG
	AG	AA	GG	GG	GG	AG	AG	AA
	AG	AG	AG	AG	AG	GG	AG	AG

GG AA AA AA GG GG AG
66814819
3234 rs10080530 6 GG GG GG GG AG
GG AG GG GG GG AG AG GG
GG GG GG GG GG GG GG GG
GG GG AG AG GG AG AG GG
GG AG GG GG GG GG GG
148242514
3235 rs10080552 6 CC CC CC CC CC
CC CC CC CC CC CC CC CC
CC CC CC CC CC CC CC CC
CC CC CC CC CC CC CC CC
CC CC CC CC CC AC CC
50529212
3236 rs10080585 6 GG AG AG AG GG
AG AG AA AA AA AG AG AA
AA AA AA AA AA AA AA AA
AG AG AG AG AG AA AA AA
AG AA AA AA AA AA AG
78876920
3237 rs10080593 6 GG GG GG GG GG
GG GG GG GG GG GG GG GG
GG GG GG GG GG GG GG GG
GG GG GG GG GG GG GG GG
GG GG AG GG GG AG GG
121710386
3238 rs1008064 7 AG AG AA AG AA
AG AA AG AG AG AG AG GG
AG GG AA AA AA AG AG AG
GG AG AA GG AG AA AA AA
AA AG AG AA GG AG GG
101116218
3239 rs10080664 6 AG AG GG AG AG
GG AA GG GG GG AG AG AG
GG AG GG GG GG GG GG GG
GG GG GG GG AA AA AA AA
AG GG AA AG AG GG AG
15864284
3240 rs10080675 6 AA AA AA AA AA
AA AA AA AA AA AA AA AA
AA AA AA AA AA AA AA AA
AA AA AA AA AA AA AA AA
AA AA AA AA AA AA AG
136863727
3241 rs10080741 6 GG GG GG GG GG
GG GG GG GG GG GG GG GG
GG GG GG GG GG AG AG GG
GG GG GG GG GG GG GG GG
GG GG GG GG GG GG GG
137643277

3242	rs1008076		19	AC	CC	AC	AA	AA
	AA	AC	AA	AC	AC	AC	AA	AA
	AA	AA	CC	AC	AC	AC	CC	CC
	AC	AC	AA	AA	AC	AC	AC	AA
	AC	AA	AC	AC	CC	AC	AC	
3968605								
3243	rs10080841		6	AG	AG	AA	AA	AA
	AA	AA	AA	AA	AA	AA	AA	AA
	AA	AA	AA	AA	AA	AG	AG	AA
	AA	AA	AA	AA	AA	AA	AA	AA
	GG	AA	AG	AA	AA	AA	AA	
137924218								
3244	rs1008091		6	AG	GG	AG	AA	AA
	AA	AA	GG	GG	GG	AG	AG	GG
	GG	GG	GG	GG	GG	AG	AG	AG
	GG	GG	GG	GG	GG	AG	GG	GG
	AG	GG	GG	GG	GG	GG	GG	
46567357								
3245	rs10080963		6	AA	AA	AA	AA	AA
	AA	AA	AA	AA	AA	AA	AA	AA
	AA	AA	AA	AA	AA	AA	AA	AA
	AA	AA	AA	AA	AA	AA	AA	AA
	AA	AA	AA	AA	AA	AA	AA	
62060128								
3246	rs10080988		6	GG	GG	GG	GG	GG
	GG	GG	GG	GG	GG	GG	GG	GG
	GG	GG	GG	GG	GG	AG	AG	GG
	GG	GG	GG	GG	GG	GG	GG	GG
	GG	GG	GG	GG	GG	GG	GG	
62408694								
3247	rs10081	11	GG	GG	GG	GG	GG	GG
	GG	GG	GG	AG	GG	AG	AG	GG
	AG	GG	GG	GG	AG	AG	AG	GG
	GG	GG	AG	GG	GG	GG	GG	GG
	GG	GG	GG	GG	GG	AG	214544	
3248	rs10081100							
6	--	--	--	--	--	--	--	--
	--	--	--	--	--	--	--	--
	--	--	--	--	--	--	--	--
	--	--	--	--	--	--	--	--
	--	--	--	--	62408452			
3249	rs10081235		7	GG	GG	GG	GG	GG
	GG	AG	AG	GG	AG	AG	GG	AG
	AG	AG	AA	AG	AG	AG	GG	AG
	AA	AG	AA	AA	GG	GG	GG	GG
	AG	AG	AG	AG	GG	GG	GG	
158322716								
3250	rs10081251		7	GG	GG	GG	GG	AG
	GG	AG	GG	GG	GG	GG	GG	GG
	GG	GG	AA	AG	AG	AA	AG	AG

GG GG GG GG GG AG AG AG
AG GG GG GG AG GG AA
51619613
3251 rs10081272 7 AA AG AA AA AA
AA AA GG AG GG AG AA AG
AG AA AA AA AA AA AA AA
AG AA AG AA AA AA AA AA
AA AA GG AG AG GG AA
20258779
3252 rs1008132 7 AG AG AG GG GG
GG GG GG GG GG AG AG GG
AG AG AG AG AG AG GG AG
GG GG GG GG AA AA AA AA
GG AA GG AG GG GG GG
20960966
3253 rs1008138 9 AC AC AC AC AC
CC AC AC AC CC CC CC CC
CC AC AA AC AA AC AC CC
CC CC CC CC CC AC AC AC
AC AC AC AC CC CC AC
116809470
3254 rs10081435 8 GG GG AG AA AA
AA AA AA AA AA AA AA AA
AA AG AA AA AA AA AA AA
AA AA AA AA AA AA AA AA
AG AG AA AG AA AA AA
136650653
3255 rs10081466 8 AA AA AA GG GG
GG AG AA AA AA AG AG AG
AG AG AA AA AA AA AA AA
AG GG AG GG AA AA AA AA
AA AA AA AA GG AG AG
2911314
3256 rs10081479 8 AA AA AA GG GG
GG GG AG AG GG AG AG AG
AA AA AG GG AG AG AG AG
AG GG GG AG AG GG GG GG
GG AG AG AA AG GG AA
2936347
3257 rs10081495 8 AA AA AG AG AA
AG AG AA AG AG AA AG AA
AA AG AA AA AG AA AA AG
AA AA AA AA AG GG AG AG
AG AG AG GG AA AG AA
49276355
3258 rs1008150 12 GG GG GG GG GG
GG GG GG GG GG GG AG GG
AG AG GG GG GG GG GG AG
AG AG GG AG AG GG AG AG
GG GG AG GG GG GG GG

47810290								
3259	rs10081561		8	AA	AG	AA	GG	GG
	AG	AG	GG	AG	AG	AG	AA	AG
	AG	AG	AG	AA	AG	AA	AG	AG
	GG	AA	AG	AG	AA	AG	AA	AA
	GG	AG	AG	AG	AG	GG	AA	
15497331								
3260	rs10081602		8	AA	AA	AA	AA	AA
	AA	AA	AA	AA	AA	AA	AA	AA
	AA	AA	AG	AG	AA	AG	AA	AG
	AA	AA	AA	AA	AG	AA	AA	AG
	AA	AA	AG	AA	AA	AA	AA	
14822104								
3261	rs1008163		4	AC	CC	CC	CC	CC
	CC	CC	CC	CC	CC	CC	CC	CC
	CC	CC	CC	AC	AC	AC	AC	AC
	AC	CC	CC	CC	AC	AC	AA	AC
	CC	AC	CC	CC	CC	CC	CC	
55054156								
3262	rs10081686		9	AG	AG	AG	AG	AA
	AG	AA	GG	GG	AA	AA	AA	AG
	AG	GG	AG	AA	AA	AG	AA	GG
	AA	AA	AA	AA	GG	AG	GG	GG
	AA	AG	AG	AG	AG	AG	AA	
73446373								
3263	rs10081702		9	AA	AA	AA	AA	AG
	AG	AA	AA	AA	AA	AA	AA	AA
	AA	AA	AG	AA	AA	AG	AA	AA
	AA	AA	AA	AA	AA	AA	AA	AA
	AA	AA	AA	AA	AA	AA	AA	
97430831								
3264	rs1008184		22	AA	AG	AA	AG	GG
	AG	GG	AA	AG	AA	AG	AG	AA
	AG	AA	AA	AA	AA	AG	AG	GG
	AG	AG	AG	AG	AA	GG	AG	AG
	AG	AG	AG	GG	AA	AG	AA	
37720268								
3265	rs10081866		X	AA	AA	AA	AA	AA
	AA	AA	AA	AA	AA	AA	AA	AA
	AA	AA	AA	AA	AA	AA	AA	AA
	AA	AA	AA	AA	AA	AA	AA	AA
	AA	AA	AA	AA	AA	AA	AA	
121009755								
3266	rs10081894		X	AA	AG	GG	AG	AA
	AA	AG	AA	AG	AA	AG	AG	AA
	AA	AA	AG	GG	AG	AA	AG	AA
	AA	AA	AA	AG	AG	AA	AG	AA
	GG	GG	GG	GG	AG	GG	AA	
126007226								
3267	rs10081923		X	AA	AA	AA	AA	AA

AA AA AA AA AA AA AA AA
AA AA AA AA AA AA AA AA
AA AA AA AA AA AA AA AA
AA AA AA AA AA AA AA
136440378
3268 rs10081942 X CC CC CC CC CC
CC CC CC CC CC CC CC CC
CC CC CC CC CC CC CC CC
CC CC CC CC CC CC CC CC
CC CC CC CC CC CC CC
100406418
3269 rs1008196 1 AA AA AA AA AA
AA AA AA AA AA AA AA AA
AG AA AA AA AA AA AA AA
AA AA AA AA AG AA AG AG
AA AG AA AG GG AG GG
30737443
3270 rs1008211 6 AA AA AA GG GG
GG AG GG AG AG GG AG GG
GG GG AG AG AA AG AG AG
AA AA AA AA AA GG AG AG
AG AA AG AG AG AA AG
21352395
3271 rs10082121 1 AA AA AA AA AA
AA AA AA AA AA AA AA AA
AA AA AA AA AA AA AA AA
AA AA AA AA AA AA AA AA
AA AA AA AA AA AA AA
65972056
3272 rs10082399 10 CC CC CC AC CC
CC AC CC CC CC AC CC AC
AC CC AC AC CC AC CC AC
CC CC AC AC CC AC AC CC
CC CC CC CC AC CC AC
70631783
3273 rs10082476 10 AA AA AA AG AA
AG AA AG AG AA AG AG AA
AA AA AG AG AG AG AA AA
AA AA AA AA AG AG AG AG
AA AA AA AA AA AA AG
124164654
3274 rs10082555 11 AA AA AA AA AA
AA AA AA AA AA AA AA AA
AA AA AA AA AA AA AA AA
AA AA AA AA AA AC AC AA
AA AA AA AA AA AA AA
130605768
3275 rs1008262 7 AG AG AG AA AA
AA AA AA AA AA AG AG AA
AG AG AA AG AA AG AG GG

	AA	GG	AG	AG	GG	AG	AG	AG
	AA	AA	AA	AA	AG	GG	AA	
28635741								
3276	rs10082676		11	AA	AA	AA	AG	AA
	AG	AA	AA	AG	AG	AA	AA	AA
	AA	AA	AA	AA	AG	AG	GG	AG
	AA	AG	AA	AG	AG	AG	AG	GG
	AG	AA	AG	AA	GG	GG	GG	
99841735								
3277	rs10082691		11	GG	GG	GG	GG	GG
	GG	GG	GG	GG	GG	GG	GG	GG
	GG	GG	GG	GG	GG	GG	GG	GG
	GG	GG	GG	GG	GG	GG	GG	GG
	GG	GG	GG	GG	AG	AA	GG	
87820858								
3278	rs1008270		21	AC	AC	AC	AA	AA
	AA	AA	AA	AA	AA	AA	AC	AC
	AC	AC	AC	AA	AC	AA	AA	AA
	AA	AA	AA	AA	AA	AA	AA	AA
	AA	AC	AA	AC	AA	AC	AA	
33002145								
3279	rs10082701		11	GG	GG	AG	GG	GG
	GG	GG	AG	GG	GG	AG	AG	GG
	GG	GG	AG	GG	GG	GG	AG	AG
	AA	AG	AA	GG	GG	AG	GG	AG
	GG	AA	GG	AG	AG	AG	GG	
1657308								
3280	rs10082730		12	AA	AA	AA	AA	AG
	AG	AA	AA	AA	AA	AG	AA	AA
	AA	AA	AA	AA	AA	AA	AA	AA
	AA	AA	AG	AA	AA	AG	AA	AA
	AA	AA	AA	AA	AA	AA	AA	
46002702								
3281	rs10082759		12	GG	AG	AG	AG	GG
	AG	GG	GG	AA	AA	GG	GG	AG
	GG	AG	GG	GG	GG	AG	AG	AG
	AG	AG	AA	AG	GG	AG	AG	AG
	AA	AG	AG	AG	AG	GG	AA	
16936820								
3282	rs10082809		12	AG	AG	AA	GG	GG
	GG	GG	GG	GG	GG	GG	GG	GG
	GG	GG	GG	AG	AG	GG	GG	GG
	GG	GG	GG	GG	GG	GG	GG	GG
	GG	GG	AG	AG	GG	AG	GG	
110515411								
3283	rs10082810		12	AG	GG	AG	GG	GG
	GG	GG	GG	GG	GG	GG	AG	AG
	AG	GG	GG	GG	GG	AG	GG	AG
	GG	GG	GG	GG	GG	GG	GG	GG
	AG	GG	GG	GG	AG	AG	GG	

75186603							
3284	rs1008283	16	AG	AG	AG	AG	AG
	AA	GG	AG	AG	AG	AG	AG	AG
	AA	AG	AG	GG	GG	AA	AA	AG
	AA	AG	AG	AA	AA	AG	AA	AG
	AA	AG	AG	AG	AG	AA	AG	
24420953							
3285	rs10082840	12	AG	AG	AG	GG	GG
	GG	GG	AG	GG	AG	AG	AG	AG
	AG	AG	AG	GG	GG	AG	AG	GG
	AG	AA	AG	AA	AG	GG	AG	GG
	AG	GG	AA	AG	GG	GG	AG	
131233805							
3286	rs10082877	12	GG	GG	GG	GG	GG
	GG	AG	AG	AG	AA	AG	GG	GG
	AG	AG	GG	AG	AG	GG	GG	AG
	AA	AA	AA	AA	AA	GG	AG	AG
	AG	GG	AG	GG	GG	GG	GG	
119498946							
3287	rs10082885	12	AG	AA	AG	AG	GG
	AG	GG	AG	GG	GG	AG	AG	AA
	GG	AG	GG	GG	GG	AA	AG	AG
	AG	AG	GG	AG	AG	AG	AG	AA
	GG	GG	AG	AG	GG	GG	GG	
16681493							
3288	rs1008290	7	AG	AG	AA	AG	AG
	GG	AA	GG	GG	GG	AG	AA	AG
	AG	AG	AA	AG	AG	GG	AG	GG
	AG	GG	GG	AG	GG	AG	GG	GG
	AG	AG	GG	AG	GG	AG	AG	
124381635							
3289	rs10082903	12	AG	AA	AA	AA	AA
	AA	AA	AG	AA	AA	AA	AA	AA
	AG	AG	AG	AG	AG	AG	AG	AG
	GG	GG	AG	GG	AG	AG	AG	AG
	AG	AG	AA	AG	AA	AG	AA	
132078060							
3290	rs10082924	12	AG	GG	AG	GG	GG
	GG	GG	AG	GG	GG	GG	GG	GG
	GG	GG	GG	GG	GG	AG	GG	AG
	GG	GG	GG	AG	AG	AG	AG	AG
	GG	AG	AG	AG	GG	AG	GG	
22847575							
3291	rs10082942	12	GG	GG	GG	GG	GG
	GG	GG	GG	GG	GG	GG	GG	GG
	GG	GG	GG	GG	GG	GG	GG	GG
	GG	GG	GG	GG	GG	GG	GG	GG
	GG	GG	GG	GG	GG	GG	GG	
129355846							
3292	rs1008297	6	AA	AA	AA	AA	AA

	AA	AG	AA	AA	AA	AG	AA	AG
	AA	AG	AG	AA	AG	AA	AA	AG
	AA	AA	AA	AA	AA	AG	AG	AG
	AA	AA	GG	AG	AA	AA	AA	
50458021								
3293	rs10083023		12	AG	AG	AA	AG	AG
	GG	AA	AA	AA	AA	AG	AG	GG
	AG	AG	GG	GG	GG	AA	AA	AG
	AG	AA	AA	AG	AA	AA	AA	AA
	AG	GG	AG	GG	AG	AA	AG	
119415451								
3294	rs10083074		12	AG	AA	AG	AA	AG
	AG	AA	AG	AG	AG	AG	AG	GG
	AA	AG	AG	AG	AG	AA	AA	AA
	AG	AG	AG	AG	AG	AG	AG	GG
	AG	AG	AA	AG	AG	AG	AA	
14436248								
3295	rs1008317		17	CC	CC	CC	CC	CC
	CC	CC	CC	CC	CC	CC	CC	CC
	CC	CC	CC	CC	CC	CC	CC	CC
	CC	CC	CC	CC	CC	CC	CC	CC
	CC	CC	CC	CC	CC	CC	CC	
16503165								
3296	rs1008318		7	AC	AC	AC	AA	AC
	AC	AA	AC	AC	AC	AC	AC	AA
	AC	AC	AC	AA	AA	CC	CC	AC
	AA	AA	AA	AA	AC	CC	AC	AC
	AC	AC	AC	AA	AC	CC	AC	
141433634								
3297	rs10083359		14	AA	AA	AA	AA	AA
	AA	AA	AA	AA	AA	AA	AA	AA
	AA	AA	AA	AG	AA	AA	AA	AA
	AA	AA	AA	AA	AA	AA	AA	AA
	AA	AA	AA	AA	AA	AA	AA	
64098555								
3298	rs10083371		14	GG	AG	AA	AG	AG
	GG	AA	GG	AG	GG	AA	AG	AA
	AA	AA	AG	GG	GG	AA	AA	AA
	AG	AG	AG	AG	GG	AG	GG	AG
	AA	AG	AA	AG	GG	AG	AG	
99229079								
3299	rs10083381		14	AA	AA	AA	AA	AA
	AA	AA	AG	AA	AG	AA	AA	AA
	AA	AA	AA	AG	AA	AA	AA	AA
	AA	AA	AA	AA	AA	AA	AA	AA
	AA	AA	AG	AG	AA	AA	AA	
32849978								
3300	rs10083493		14	GG	GG	GG	GG	GG
	GG	GG	GG	GG	GG	AG	AG	GG
	GG	GG	GG	GG	GG	GG	AG	GG

	GG	GG	GG	GG	AG	GG	AG	AG
	GG	GG	GG	GG	GG	GG	GG	
56045411								
3301	rs10083525		14	AA	AA	AA	AC	AA
	AC	AA	AC	AC	AA	AA	AA	AA
	CC	CC	AA	AA	AC	AA	AA	AA
	AC	AA	AC	AC	AA	AA	AA	AA
	AC	AA	AA	AA	AA	AA	AC	
65851556								
3302	rs10083558		15	CC	CC	CC	CC	CC
	CC	CC	CC	CC	CC	CC	CC	CC
	AC	CC	CC	CC	CC	CC	CC	AC
	AC	AA	AA	AC	AC	CC	CC	AC
	CC	CC	CC	CC	AC	CC	AA	
91202504								
3303	rs10083562		15	AC	AC	CC	AC	AC
	AC	AA	AC	CC	AC	CC	CC	CC
	AC	CC	AA	AA	AA	AA	CC	AA
	AA	AA	AA	AA	AA	AC	AC	AC
	AC	CC	AA	AC	CC	AC	AC	
55028177								
3304	rs10083582		15	GG	GG	GG	AG	AG
	AG	GG	GG	AG	AG	AG	GG	GG
	GG	GG	AG	GG	GG	AG	AG	AG
	GG	GG	GG	GG	GG	GG	GG	GG
	AG	GG	AG	AG	GG	GG	GG	
88333431								
3305	rs10083586		15	AC	AC	CC	AA	CC
	AC	AC	AC	AC	AC	AC	CC	AC
	AA	AC	CC	AC	AC	CC	CC	AC
	CC	CC	CC	CC	AC	CC	AC	AC
	CC	AC	CC	CC	CC	CC	CC	
26414375								
3306	rs10083594		15	GG	GG	GG	GG	GG
	GG	GG	GG	GG	GG	GG	GG	GG
	GG	GG	GG	GG	GG	GG	GG	GG
	GG	GG	GG	GG	GG	GG	GG	GG
	GG	GG	GG	GG	GG	GG	GG	
101165012								
3307	rs1008360		17	GG	GG	GG	GG	GG
	GG	AG	GG	GG	GG	AG	GG	GG
	GG	GG	GG	GG	GG	GG	GG	GG
	GG	GG	GG	GG	GG	GG	GG	GG
	GG	AG	GG	GG	GG	GG	AG	
11209792								
3308	rs10083634		15	CC	CC	CC	CC	CC
	CC	CC	CC	CC	CC	CC	CC	CC
	CC	CC	CC	CC	CC	CC	CC	CC
	CC	CC	CC	CC	CC	CC	CC	CC
	CC	CC	CC	CC	CC	CC	CC	

73165722
3309 rs10083670 15 GG GG GG GG GG
GG AG AG GG GG GG GG GG
GG GG GG AG AG GG GG GG
AG GG GG GG AG AG AG AG
AG GG GG GG AG GG AG
91022135
3310 rs1008368 7 GG GG GG GG GG
GG GG GG GG GG GG GG GG
AG AG AG GG AG AG GG AG
AA AG AG AG GG GG GG GG
GG AG AA AG GG GG GG
12953982
3311 rs1008375 4 AG AA AA GG AA
GG AG GG AG AG GG AG GG
GG AA GG AG AG AG AG GG
AA AG AA AA AG AA AG AG
AG GG GG GG GG GG GG
17682834
3312 rs10083789 16 AA AC AA CC CC
CC CC CC CC CC AC AC AC
CC AC AC AA AA AC AC --
AC CC CC AC CC AC CC AC
AC CC AA AC AC AC CC
23080634
3313 rs10083838 17 AC AC AC AC AC
CC AC AA AC AC AC AA AC
AC AC AA AA AA AC AA AC
AC AA AC AA AA AA AA AA
AA AC AC AC AC CC AC
57509350
3314 rs10083841 17 AA AA AA AA AA
AA AA AA AA AA AA AA AA
AA AA AA AA AA AA AA AA
AA AA AA AA AA AA AA AA
AA AA AA AA AA AA AA
71649446
3315 rs10083843 17 AG AG AG GG AG
AG AG AG GG GG AA AA AA
AG GG AA AG AG GG GG GG
AG GG GG AG AA AA AA AA
GG GG AA AG GG GG GG
42674912
3316 rs1008386 7 GG GG GG GG GG
GG GG GG GG GG GG AG AG
GG GG GG GG GG GG GG GG
AG AG AG AG AG GG GG AG
GG GG GG GG GG AG GG
51825899
3317 rs10083861 17 AG AG AG AG AG

	GG	AA	GG	AA	AG	GG	AG	AG
	AA	AG	AA	AA	AA	GG	GG	GG
	AA	AG	AG	AA	GG	AA	AG	AG
	AA	AG	GG	AG	AA	AG	AA	
55838072								
3318	rs10083888		17	GG	GG	GG	AG	AG
	GG	AG	GG	GG	GG	GG	GG	GG
	GG	GG	GG	AG	AG	GG	GG	AG
	GG	GG	GG	GG	GG	GG	GG	GG
	GG	AG	GG	GG	GG	GG	GG	
33402980								
3319	rs10083941		18	AG	GG	GG	GG	GG
	GG	GG	GG	AG	GG	GG	GG	AG
	AG	GG	GG	GG	GG	GG	GG	GG
	GG	GG	AG	AG	GG	GG	GG	GG
	AA	AG	GG	AG	AG	AG	GG	
50282122								
3320	rs10083961		18	AA	AC	AA	CC	AC
	CC	AC	AA	AA	AA	AC	AC	AC
	AC	CC	AA	AA	AC	AA	AA	AC
	AC	AC	AC	AC	AA	AC	AA	AA
	AA	AC	AA	AA	AC	AC	AC	
62166391								
3321	rs1008400		16	AG	AG	AA	GG	GG
	GG	GG	AG	AG	AA	AG	AA	AG
	AG	AG	AG	AG	GG	AG	AG	GG
	GG	GG	GG	AG	AA	GG	AG	AG
	AG	AG	AG	AG	AA	AG	AG	
54119892								
3322	rs10084013		18	AG	AA	AA	AA	AA
	AA	AG	AG	AA	AA	GG	AG	GG
	GG	AG	AG	AA	AG	AG	AA	AG
	AG	AG	AA	AA	AG	GG	GG	GG
	AG	AG	AG	GG	AA	AA	AA	
39823817								
3323	rs10084065		18	CC	CC	CC	CC	CC
	CC	CC	AA	CC	AC	AC	CC	CC
	CC	CC	AC	CC	AC	CC	CC	CC
	CC	CC	CC	CC	CC	AC	CC	CC
	CC	CC	CC	CC	CC	CC	CC	
37896392								
3324	rs1008407		1	AA	AA	AA	AG	AG
	AG	AG	GG	GG	GG	AG	AG	GG
	GG	GG	GG	GG	GG	AG	AG	AG
	AG	AG	AG	AG	AG	AA	AG	AA
	AG	GG	AG	AG	AA	AA	AG	
187062807								
3325	rs10084073		18	AA	AA	AA	AA	AA
	AA	AA	AA	AA	AA	AA	AA	AA
	AA	AA	AA	AA	AA	AA	AA	AA

	AG	AG	AA	AG	AA	AA	AA	AA
	AA	AA	AA	AA	AA	AA	AA	
74184337								
3326	rs10084110		19	AG	AG	AA	AG	AG
	GG	AG	GG	AG	AG	GG	AG	AG
	AG	AG	GG	GG	GG	GG	AG	GG
	AG	AG	AG	AG	AG	AG	AG	GG
	AG	GG	AA	AG	AG	GG	AG	
21821537								
3327	rs1008415		2	AA	AG	AA	AG	AG
	GG	AA	AG	AG	AG	AA	AG	AG
	AG	AG	AA	AG	AG	AA	AG	AG
	AG	AG	AG	AA	AG	AG	GG	AG
	AG	GG	AA	AG	AG	AG	AA	
225151655								
3328	rs10084179		2	GG	GG	GG	GG	GG
	GG	GG	GG	GG	GG	GG	GG	GG
	GG	GG	GG	GG	GG	AG	GG	AG
	GG	GG	GG	GG	AG	AG	AG	AA
	GG	AG	AG	AA	GG	GG	GG	
2382311								
3329	rs10084200		2	GG	GG	AG	AG	AA
	AG	AG	GG	GG	GG	GG	GG	GG
	GG	GG	GG	GG	GG	GG	GG	GG
	GG	GG	GG	GG	GG	GG	GG	GG
	GG	GG	GG	GG	AG	AG	GG	
77151777								
3330	rs10084238		2	AG	GG	AG	AG	AA
	AA	AG	AA	AG	AA	AG	AG	AG
	GG	GG	AG	AG	AA	AA	AA	AG
	AG	AA	AG	AG	AG	AA	AG	AA
	AA	AG	AG	GG	AG	AA	GG	
25458379								
3331	rs10084263		2	AA	AC	AA	CC	AC
	AC	CC	AC	AC	AC	AC	AC	AC
	AC	AA	AC	AA	AA	AA	AC	AA
	AC	AC	AC	CC	AA	AC	AC	AA
	AC	CC	AA	AC	AA	AA	AC	
201367356								
3332	rs10084272		2	GG	GG	GG	GG	GG
	GG	GG	GG	AG	AG	GG	GG	GG
	GG	GG	GG	GG	GG	GG	GG	GG
	AG	GG	AG	GG	GG	GG	GG	GG
	GG	GG	GG	GG	GG	GG	GG	
170947923								
3333	rs10084410		2	AA	AA	AA	AG	AG
	AG	AG	AA	AA	AA	AG	AG	AG
	AA	AA	AA	AA	AA	AA	AA	AA
	AA	AA	AG	AA	AA	AA	AA	AA
	AA	AA	AA	AA	AG	AA	AG	

32296099							
3334	rs10084473	2	AA	AA	AG	GG	AG
	AG	GG	AA	AA	AA	AA	AG	AA
	AA	AA	AA	AA	AA	GG	GG	GG
	AG	AA	AA	AG	AG	AA	AG	AA
	AG	GG	AG	GG	AG	AG	AG	
44232411							
3335	rs10084490	20	AA	AA	AA	AG	AG
	GG	AA	GG	AG	GG	AA	AA	AA
	AA	AA	AA	AG	AA	AG	AA	AG
	AG	GG	GG	AG	AA	GG	AG	AG
	AA	AA	AA	AA	GG	GG	GG	
16744794							
3336	rs1008455	1	GG	GG	GG	GG	GG
	GG	GG	GG	GG	GG	GG	GG	GG
	GG	GG	GG	GG	GG	GG	GG	GG
	GG	GG	GG	GG	GG	GG	GG	GG
	GG	GG	GG	GG	GG	GG	GG	
47673802							
3337	rs10084596	21	AG	AG	AG	GG	GG
	GG	GG	GG	GG	GG	GG	GG	GG
	GG	GG	AG	AG	AG	AA	GG	AG
	AG	GG	AG	GG	AA	AA	AA	AA
	GG	GG	AG	GG	GG	AG	GG	
44744540							
3338	rs10084615	21	GG	AG	AG	GG	GG
	GG	GG	AG	GG	GG	GG	GG	GG
	GG	GG	GG	GG	GG	GG	AG	GG
	AG	GG	GG	GG	GG	GG	GG	GG
	GG	GG	GG	GG	GG	GG	GG	
19646269							
3339	rs10084616	21	AA	AA	AA	AA	AA
	AA	AA	AA	AA	AA	AA	AA	AA
	AA	AA	AA	AA	AA	AA	AA	AA
	AA	AA	AA	AA	AA	AA	AA	AA
	AA	AA	AA	AA	AC	AC	AA	
32110767							
3340	rs10084621	21	GG	GG	GG	GG	GG
	GG	GG	GG	GG	GG	AG	AA	AG
	AG	AG	AG	AG	AG	GG	GG	AG
	AG	GG	AG	AG	GG	AG	GG	GG
	GG	AG	AG	AG	GG	GG	AG	
48049235							
3341	rs10084637	0	AA	AA	AA	AA	AA
	AA	AA	AA	AA	AA	AA	AA	AA
	AA	AA	AA	AA	AA	AA	AA	AA
	AA	AA	AA	AA	AA	AA	AA	AA
	AA	AA	AA	AA	AA	AA	AA	0
3342	rs10084713	3	GG	AG	GG	AA	AG
	AG	AG	AG	AA	AG	AA	AA	AA

AA AA AA AG AA AA AG AA
GG AG AG AG AA AA AA AA
AG AA AG AG GG GG GG
174036130
3343 rs10084768 4 GG AG AG AG AG
AG GG GG GG GG AG AG AG
AG GG GG GG GG AG AG AG
AG GG AG AG GG GG GG GG
GG AA GG AG GG GG GG
13719489
3344 rs10084772 4 AA AA AA AG AA
AG AA AG AA AG AA AA AA
AA AA AA AG AG AA AA AA
GG AG AG AG AG AG GG AG
AG AG AA AA AG AG AG
180635834
3345 rs10084836 4 AG AG GG AA AA
AA AA AG AG AG AA AA AA
AA AA AA AA AA GG GG AG
AA AA AA AG AA GG AG AG
GG GG AG GG AG AG AG
40389609
3346 rs10084852 4 AA AA AA AA AA
AA AA AA AA AA AA AA AA
AA AA AA AA AA AA AA AA
GG AG AG GG AG AA AA AA
AA AA AA AA AA AA AG
134083809
3347 rs1008499 14 AG AA AA AA AG
AA AG AA AA AA AA AA AA
AA AA AA AA AA AA AA AA
AG GG AG AG AA AA AA AA
AA AG AA AA AA AA AA
54734750
3348 rs10085025 5 AG AG AA AA AA
AA AA AA AA AA AA AA AA
AA AA AA AA AA AA AA AA
AA AA AA AA AA AA AA AA
AA AA AA AA AA AG AG
180060790
3349 rs10085098 5 GG AG GG AG AA
AG AG AG AG AG GG GG GG
GG GG GG GG GG AG GG AG
AG AG AG AG AG GG AG GG
AG AG GG AG GG GG GG
117017440
3350 rs10085144 5 AA AA AA AA AA
AA AG AG AG AG AG GG AG
AG AG AG AA AA AG AA AG
AG AG AG AG GG AA AG AG

GG AA AA AA AA AG AA
84240377
3351 rs1008515 22 AA AA AA AA AA
AA AA AG AA AG AA AA AA
AG AA AA AG AG AG AG AA
AG AG GG AA AA AA AA AA
AA AG AA AG AG AA GG
30091145
3352 rs10085155 5 AG AA AG AA AA
AA AA AG AG AG AG AG AA
AA AA AG AG AG AA AA AA
AA AA AA AA AA AA AA AA
AG AG AG AA AG AG AA
123001213
3353 rs10085169 5 GG GG GG AG GG
AG AG AG AG AG AA AG AA
AG AG AA AG AG AG GG AA
AA AG AG AG GG AG AG GG
AA GG AA AG GG AG GG
121601488
3354 rs10085205 6 AG GG AG AG GG
GG AG GG GG GG GG GG GG
GG GG AG AG GG GG GG GG
AG AG AG AG AG AA AG AA
GG GG AG GG GG GG GG
44084883
3355 rs10085207 6 AG AG GG GG GG
GG GG GG GG GG AA AA --
AG GG GG GG GG GG GG GG
GG GG GG GG GG GG GG GG
GG AG AG AG GG GG GG
67022706
3356 rs10085214 6 GG GG GG GG AG
GG AG -- AG AG GG GG GG
GG GG GG GG GG AG AG GG
GG GG GG GG GG GG GG GG
GG GG GG GG GG GG GG
87885952
3357 rs1008531 15 AC AC AA AA AA
AA AA AC AC AC AA AA AC
AC AC AC AA AC AA AA AA
AC AC AC AC AA CC AC AC
AA AC AC AC AC AA CC
25825753
3358 rs1008532 20 GG GG GG GG GG
GG GG GG GG GG GG GG GG
GG GG GG GG GG GG GG GG
GG GG GG GG GG GG GG GG
GG GG GG GG GG GG GG
54468058

3359 rs10085329 6 AG AA AG AA AA
AA AA AA AA AA AG AG AA
AA AA AA AA AA AA AG AA
AG AG AG AG AA AA AA AA
AA AA AA AA AA AA AA
139757582
3360 rs10085389 7 AG AG AG GG GG
GG GG GG AG AG AA AG AG
GG GG GG GG GG GG GG GG
GG GG GG GG GG GG GG GG
AG GG AG AG GG GG GG
38522596
3361 rs10085395 7 AA AA AA AG AG
AG AG GG GG AG AA AA AA
AA AA AA AA AA AA AG AA
AG AA AG AA AA AA AA AA
AG AA AA AA AA AA AA
17968205
3362 rs10085416 7 AA AG AA GG GG
GG AG AG AG AA AG AG AA
AA AG AG AG GG AA AA AG
GG AG AG AG AG AG AA AG
AA GG AG GG AG AG GG
14868813
3363 rs10085432 7 GG GG GG GG GG
GG GG GG GG GG GG GG GG
AG AG GG GG GG GG GG GG
GG GG GG GG GG GG GG GG
GG GG GG GG GG GG GG
156810883
3364 rs10085463 7 GG GG GG AG AG
AA GG AA AG AA GG GG GG
AG AG GG GG GG GG GG GG
GG AG GG AG AG AG AG AA
GG GG GG GG AG AG GG
29536707
3365 rs10085518 7 GG GG GG GG GG
GG GG GG GG GG AG AG AA
GG AG GG AG GG GG GG AG
GG GG GG GG AG AG AG AG
-- GG GG GG GG GG GG
6285515
3366 rs10085588 7 GG GG GG GG GG
GG GG AG AG AG AG AG GG
GG AG GG GG GG GG GG GG
GG GG AG GG GG AG GG GG
AG AG AG AG GG GG GG
96137674
3367 rs1008563
2 -- -- -- -- -- -- -- --

-- -- -- -- -- -- -- --
-- -- -- -- -- -- -- --
-- -- -- -- -- -- -- --
-- -- -- -- 219026888
3368 rs10085637 7 AA AG AG AG GG
GG AG AA AA AA AG AG GG
GG GG AG GG GG AG GG AG
AG GG GG AG AG GG AG AG
AA AG AG AG AG AG AA
95225813
3369 rs10085693 7 GG GG GG GG GG
GG AG GG GG GG GG AG AG
AG AG AG AA AA GG GG GG
GG GG GG GG GG GG GG GG
AA GG GG GG AG AG AG
114236509
3370 rs10085715
7 -- -- -- -- -- -- -- --
-- -- -- -- -- -- -- --
-- -- -- -- -- -- -- --
-- -- -- -- -- -- -- --
-- -- -- -- 41104111
3371 rs10085723 7 AA AA AA AA AG
AG AA AA AA AG AG AG GG
GG AG AG AG GG AG AA GG
AG AA AA AA GG AG AG AG
AA AG AA AA GG AG GG
14136740
3372 rs10085724 7 GG AG AG AG AG
GG AA AA AG AG GG GG AG
AG AA AG AA AA GG GG GG
AA AA AA AA GG AG AG AG
GG GG AG GG GG AG AG
87382
3373 rs10085751 7 AG -- AG AG GG
AG GG GG AG GG AG AG --
AG GG GG AG GG AG GG GG
GG GG GG GG -- GG AG AG
GG GG GG GG AG AG AG
63699812
3374 rs10085823 7 GG GG GG GG GG
GG GG GG GG GG GG GG GG
GG GG GG GG GG GG GG GG
GG GG GG GG GG GG GG GG
GG GG GG GG GG GG GG
99009237
3375 rs10085832 7 GG GG GG GG GG
GG GG GG GG GG GG GG GG
GG GG GG GG GG GG GG GG
GG GG GG GG GG GG GG GG

	GG	GG	GG	GG	GG	GG	GG
52080130							
3376	rs1008588		17	GG	GG	AG	GG	GG
	GG	AG	GG	GG	GG	AA	AA	AA
	AA	AA	AG	AG	AA	AG	AG	AG
	AG	AG	AG	AG	GG	AA	AG	AG
	AG	AG	AA	AG	GG	GG	GG	
5711950								
3377	rs10085962		8	AG	AG	AG	GG	GG
	GG	GG	GG	GG	GG	GG	GG	GG
	GG	GG	AG	AG	GG	AG	GG	GG
	AG	AG	AG	AG	AA	GG	AG	AG
	GG	GG	GG	GG	GG	AG	GG	
93431643								
3378	rs10086015		8	AG	AG	AG	GG	AG
	AG	GG	GG	GG	GG	GG	GG	GG
	GG	GG	GG	GG	GG	GG	GG	GG
	GG	GG	GG	GG	GG	GG	GG	GG
	GG	GG	GG	GG	GG	GG	GG	
3936947								
3379	rs10086080		8	GG	GG	GG	GG	GG
	GG	GG	GG	GG	GG	GG	GG	GG
	GG	GG	GG	GG	GG	AG	AG	GG
	AA	AG	AG	GG	AG	GG	AG	AG
	GG	GG	GG	GG	GG	GG	GG	
140841887								
3380	rs10086104		8	AA	AA	AA	CC	AC
	CC	AC	CC	CC	CC	CC	CC	CC
	CC	CC	AC	AC	CC	AC	AC	AA
	AC	AA	AA	AA	CC	CC	CC	CC
	AC	AC	CC	CC	CC	CC	CC	
95065264								
3381	rs1008620		17	AG	GG	GG	AA	AG
	AA	AG	AA	AA	AA	AA	AA	AA
	AA	AA	AG	AG	AG	AA	AA	AA
	AG	AG	AA	AA	AG	AA	AG	AA
	AG	GG	AA	AG	AA	AG	AG	
70362790								
3382	rs10086214		8	AA	AA	AA	GG	AG
	AG	AG	AA	AA	AA	AG	GG	AG
	AG	GG	AG	AA	AG	AA	AG	AG
	AA	AG	AG	AA	AA	AG	AA	AA
	AG	AG	AG	AG	AG	GG	AG	
69755610								
3383	rs10086223		8	AG	AG	AA	GG	GG
	GG	GG	AG	AG	AG	AG	AA	AG
	AA	AG	GG	AG	AG	GG	AA	GG
	AG	GG	GG	GG	AG	GG	GG	AG
	AG	GG	GG	GG	GG	GG	AG	
105053383								

3384 rs10086233 8 AA AA AA AA AA
AA AA AA AA AA AA AA AA
AA AA AA AA AA AA AA AA
AA AA AA AA AA AA AA AA
AA AA AA AA AA AA AA
98676837
3385 rs10086299 8 AA AA AA AG AG
AA AG AA AA AA AA AA AA
AA AA AA AA AA AA AA AA
AA AA AA AA AA AA AA AA
AA AA AG AG AA AA AA
11485259
3386 rs1008631 2 GG GG GG AG GG
GG AG AG GG GG GG GG GG
GG GG GG GG GG GG GG GG
GG AG GG AG GG GG GG GG
GG GG GG GG GG AG GG
65050550
3387 rs1008632 18 AG AA AA AG GG
AG GG GG AA AG AA AG AA
AG GG GG GG AG AG GG AG
AA AA AA AA GG AG AG AG
GG AG GG AG GG GG AG
58869249
3388 rs1008635 17 AA AA AA AA AA
AA AA AA AA AA AA AA AA
AA AA AG AA AA AA AA AA
AG AA AA AG AG AG AA AA
AA AG AA AA AA AG AA
42061695
3389 rs10086371 8 GG GG GG GG AG
GG AG GG GG GG GG GG GG
GG GG GG GG GG GG GG GG
GG GG GG GG GG GG GG GG
GG GG GG GG GG GG GG
119325270
3390 rs10086387 8 GG GG GG GG GG
AG GG GG GG GG GG GG GG
GG GG GG GG GG GG GG GG
GG GG GG GG GG GG GG GG
GG GG GG GG GG GG GG
58827592
3391 rs10086487 8 AG AA AG AG AG
AA GG GG GG GG GG GG AG
AG AA AG AG AG GG GG GG
AG AG GG GG AG GG GG GG
GG GG AA AG GG GG GG
138616654
3392 rs10086506 8 AA AC AC AA AA
AA AA AA AA AA AA AC AC

AC AA AA AA AA AA AC AA
AC AA AC AA AA AA AA AA
AA AC AC AA AC AC AC
116988947
3393 rs10086507 8 GG AG GG GG AG
AG GG AA AA AA AG AG AG
AG GG AG AG AG GG GG GG
GG GG GG GG GG AG AG AG
GG GG GG GG GG GG GG
136829636
3394 rs10086512 8 GG GG GG AG AG
AG GG GG AG AG GG AG AG
AG AG GG GG AG GG AG GG
AA AA AA AA AG GG GG GG
GG AG AA AG AG AG AG
74538698
3395 rs10086550 8 AG AG AA AA AA
AA AG GG AG AG AG AG GG
GG GG AG AA AA AA AA AA
GG GG AG GG GG AA AG AG
AG AA AA AA AA AA AA
143363037
3396 rs10086567 8 AG AG AG GG --
-- -- GG GG GG -- -- GG
GG GG GG GG -- -- GG GG
-- -- -- -- GG GG -- --
-- AG -- AG -- -- --
3167217
3397 rs10086623 8 AG GG AG GG GG
GG GG GG GG GG GG GG GG
GG GG GG GG GG GG GG GG
GG GG GG GG GG GG GG GG
GG GG GG GG GG GG GG
83488195
3398 rs10086636 8 AG GG AG AG AG
AA AG AG AG AG GG GG GG
GG AG AG GG AG GG GG AG
AG AG AG AG GG AG AG AG
AA AG AG GG AG AG GG
126604637
3399 rs10086700 8 GG GG GG GG GG
GG GG GG AG AG AA AG AG
AG GG AG AG GG GG GG GG
GG GG GG GG AG AG AG AA
GG GG GG GG AG AG GG
88541187
3400 rs10086736 8 AG AG AG AA AA
AG AA AG AG AG AG AA AG
AG AA AG AG AG GG AG GG
AA AA AA AA AG GG GG GG

	AG	AG	AA	AG	AG	AG	AG	
74661778								
3401	rs10086750		8	AA	AA	AA	AA	AA
	AA	AA	AA	AA	AA	AA	AA	AA
	AA	AA	AA	AA	AA	AA	AA	AA
	AA	AA	AA	AA	AA	AA	AA	AA
	AA	AA	AA	AA	AA	AA	AA	
137963141								
3402	rs1008676		6	GG	AG	AG	GG	AG
	AG	AG	AA	AA	AA	GG	GG	GG
	GG	GG	GG	AG	GG	AG	AA	GG
	GG	GG	AA	AA	GG	GG	GG	GG
	GG	GG	GG	GG	GG	GG	GG	
162799117								
3403	rs10086768		8	AC	AA	AC	AA	AA
	AA	AA	AA	AC	AC	AA	AA	AC
	AC	AA	AA	AA	AA	AC	AC	AC
	AA	AA	AA	AA	AC	AC	CC	AC
	AA	AC	AA	AA	--	CC	AC	
18212354								
3404	rs10086779		8	AA	AG	AA	GG	AG
	AG	AG	AG	AG	AG	AA	AA	AA
	AA	AA	GG	AG	GG	AA	AA	AA
	AG	AG	AG	AG	AG	GG	GG	AG
	AG	AA	AA	AA	AG	AA	AG	
89864220								
3405	rs1008680		1	AG	GG	AG	AA	AG
	AG	AA	GG	GG	GG	AG	AG	GG
	GG	AG	GG	AG	AG	AG	AG	AG
	AG	AA	AG	AA	GG	AG	AG	AG
	AA	AA	AG	AA	AA	AA	AG	
168285045								
3406	rs10086825		8	AA	AA	AA	AG	AG
	AA	AG	AA	AA	AA	AG	AA	AG
	AA	AA	AG	GG	AG	GG	AA	GG
	GG	AA	AG	AG	AG	AA	AG	AA
	AG	AA	AG	AG	AA	AA	AG	
13434686								
3407	rs10086856		8	GG	GG	GG	GG	GG
	GG	GG	GG	GG	GG	GG	GG	GG
	GG	AG	GG	GG	GG	AG	GG	GG
	GG	GG	GG	GG	GG	GG	GG	GG
	GG	AA	GG	AG	GG	GG	GG	
21157621								
3408	rs10086879		8	CC	CC	CC	CC	CC
	CC	CC	AC	CC	CC	AA	AC	AC
	AC	AC	AC	--	AC	CC	CC	--
	CC	AC	--	CC	AC	AC	AC	AC
	AA	--	CC	AC	--	AC	CC	
78719309								

3409 rs1008688 6 CC CC CC CC AC
AC AC CC CC CC AC AC CC
CC CC CC CC CC CC CC CC
AA AC AC AA AC CC AC CC
CC CC AC CC CC CC CC
131145224
3410 rs10086882 8 AG AA AG AG AG
AA GG AG AG AG AA AA AA
AG AG GG AG AG AG GG GG
AA AG AG AG GG AG AG AG
AG GG AA AG AG GG AG
97655880
3411 rs10086897 8 GG GG GG GG GG
GG GG GG GG GG GG GG GG
GG GG GG GG GG GG GG GG
GG GG GG GG GG GG GG GG
GG GG GG GG GG GG GG
112145769
3412 rs10086908 8 AG AA AA AA AA
AA AA AA AA AA AA AA AG
AG AG AG AA AA AA AA AA
AG AA AG AA AA AG AG AG
AA AG AG AA AA AA AA
128011937
3413 rs10086932 8 AG AG AG AA AA
AA AA AG AG AG AG AA AG
AA AA GG GG GG AG AG AG
AA AA AA AA GG AG AG AG
AG AA AG AA AG AG AG
115018622
3414 rs10086950 8 CC CC CC AC CC
AC CC CC CC CC CC CC CC
AC AC CC CC CC CC CC CC
CC CC CC CC CC CC CC CC
CC AC AC AA CC CC CC
10355378
3415 rs10086971 8 AC AA AC CC AC
AC CC CC CC CC CC CC CC
AC AA AC AC CC CC CC AC
AA AC AC AC AC CC AC CC
CC CC AC AC AC AA AC
105796187
3416 rs10087063 8 AA AA AA AA AA
AA AA AA AA AA AA AA AA
AA AA AA AA AA AA AA AA
AA AA AA AA AA AA AA AA
AA AA AA AA AA AA AA
93598778
3417 rs10087078 8 AA AA AA AG AG
AA AG AA AA AA AG AG AG

	AG	AG	AG	GG	AG	AA	AA	AG
	AG	AA	AG	AA	AA	AG	AG	AA
	AA	AG	AG	AA	AA	AG	AG	
8930740								
3418	rs10087102		8	AA	AA	AA	AA	AA
	AA	AA	AA	AA	AA	AA	AG	AA
	AA	AA	AA	AA	AA	AA	AA	AA
	AA	AG	AG	AA	AA	AA	AA	AA
	AA	AA	AA	AA	AA	AA	AA	
61849086								
3419	rs10087151		8	AA	AG	GG	AA	AA
	AA	AA	AA	AA	AA	AA	AA	AA
	AG	AG	AA	AA	AG	AG	AG	AA
	AG	AG	AG	AG	AA	AA	AA	AA
	AA	AA	AA	AA	AG	AA	AG	
121172261								
3420	rs10087168		8	AA	AG	AA	GG	GG
	GG	AG	AG	AG	AG	AG	GG	AG
	GG	GG	GG	GG	GG	GG	AG	AG
	GG	GG	GG	GG	AG	GG	GG	GG
	AG	GG	AG	AG	AG	AA	GG	
5101411								
3421	rs10087176		8	GG	GG	AG	GG	AG
	GG	AG	AA	AA	AA	AA	AG	AG
	AG	AG	AG	AG	AG	AA	AA	AA
	AA	AA	AA	AA	AG	GG	AG	GG
	AA	GG	AG	GG	GG	AG	AG	
108788603								
3422	rs10087236		8	AG	AA	AG	AG	GG
	GG	AG	GG	GG	AG	GG	GG	GG
	GG	GG	AG	AG	AG	GG	GG	GG
	GG	GG	GG	GG	AG	AG	AA	AA
	AG	GG	AA	AG	AG	GG	AG	
59387670								
3423	rs10087239		8	GG	GG	GG	GG	GG
	GG	GG	AG	AG	AG	AA	GG	AG
	GG	GG	GG	AG	GG	AG	AG	GG
	GG	GG	GG	GG	GG	AG	GG	GG
	AG	GG	AG	GG	AG	GG	AG	
53324601								
3424	rs10087240		8	GG	GG	GG	AG	GG
	AG	GG	GG	GG	GG	GG	GG	AG
	GG	AG	GG	GG	GG	AG	GG	AA
	AG	AA	AG	AA	GG	AG	GG	GG
	GG	AG	AG	AA	GG	AG	AG	
129012574								
3425	rs1008725		2	AG	GG	GG	AG	AG
	GG	AA	GG	GG	GG	AG	GG	GG
	AG	AG	AG	AA	AA	AA	AA	AG
	AG	AA	AG	AA	AA	AG	AA	AG

AG GG AG AG AG AA GG
62430208
3426 rs10087256 8 AA AA AA AA AG
AG AA AA AA AA AA AA AA
AA AA AA AA AA AA AA AA
AG AA AA AG AA AA AA AA
AA AG AG GG AA AA AG
9887159
3427 rs1008727 10 AC AA AA AC AC
AC AC CC CC CC AC AC CC
CC AC AC AC AC AC AC AA
AC AC AA AA AA AC AA AA
AC AC AC AC AA AC AC
121446251
3428 rs1008728 4 AA AA AG AA AA
AG AA AG AG GG AG AG GG
AG GG AA AG AG GG GG GG
AG AG AA AA AA AG AA AG
AA AA AA AA AA AG AA
187226673
3429 rs10087402 8 AA AA AA AA AG
AG AA AA AA AA AG AA AG
AG AA AA AA AA AA AA AA
AA AG AG AG AA AA AA AA
AA AG AA AG AA AA AA
96099666
3430 rs10087406 8 GG GG GG AG AG
GG AA GG AG AG AG AA AG
AG AG AG AG AA AG AG GG
AG AA AA AG GG AA AG AG
AA AG AG GG AG AG AG
9246598
3431 rs10087430 8 AA AA AA AA AA
AA AA AA AA AA AA AA AA
AA AA AA AA AA AA AA AA
AA AA AA AA AA AA AA AA
AA AA AA AA AA AA AA
92353968
3432 rs1008744 8 AC CC AC AA AA
AA AA AA AA AC AA AA AA
AA AA AA AC AC CC AC AC
AC AC AC AA AA AA AA AA
AC AC AA AA AA AA AA
134700316
3433 rs10087445 8 GG GG GG AG GG
GG AG AG AG AG GG GG GG
GG GG AG AG GG GG GG GG
GG GG GG GG GG GG GG GG
AG GG GG GG AG AG GG
98936178

3434 rs10087446 8 AA AG AG AA AG
AA AG GG GG GG AA AA AA
AA AA AA AG AA AA AG AA
AA AA AA AA AG AA AG AA
AA AA AA AA GG GG AG
118989097
3435 rs10087480 8 CC CC CC CC CC
CC CC CC CC CC CC CC CC
CC CC AC CC AC CC CC CC
AC CC CC CC CC CC CC CC
CC CC AC AC CC CC CC
90354767
3436 rs1008749 13 AC AC AC AA AA
AA AA AC AA AC AA AA AA
AA AA AA AA AA AA AA AA
AA AA AA AA AA AA AA AA
AA AA AA AA AA AA AA
71741816
3437 rs10087547 8 AC AC AC CC CC
CC CC CC CC CC CC CC CC
CC CC CC CC CC CC CC CC
CC CC CC CC CC CC CC CC
CC CC CC CC CC CC CC
14596241
3438 rs10087670 8 AC CC AC CC CC
CC CC CC CC CC CC CC CC
CC CC CC CC CC CC CC CC
CC CC CC CC CC CC CC CC
CC CC AC AC CC CC CC
68580393
3439 rs10087719 8 AG GG AG AG AG
AA GG GG GG AG AG AG AA
AA AA AG AA AA AG AA AG
AG GG AG GG GG AG GG GG
AA AG AA AG AG GG AG
128228863
3440 rs10087744 8 AC AC AC CC CC
AC CC AC AC CC AC AC AA
AA AC AA AC AC AA AC AA
AC AA AA AC AC AA AA AA
AC AA AC AA AC AC CC
73993200
3441 rs10087768 8 AA AC AA AA AA
AA AA AC AC AC AA AA AA
AC AC AA AA AA AC AA AC
AA AA AA AA AA AC AA AC
AA AA AA AA AC CC AA
9079297
3442 rs10087788 8 AG AA AG AG GG
AG AG AA AA AA AG AA AG

AG AA AA AA AA GG AG AG
GG AG AG AG AA AA AA AA
AA GG AA AG AG AG AA
87544174
3443 rs10087801 8 AG GG GG AG AG
AG AG GG AG AG GG AG GG
AG AG GG GG GG AG AG AA
AG AG AA AG GG GG GG GG
GG AG GG GG GG GG GG
30237882
3444 rs100879 18 AG AA AG AG AG AA
GG AG AG AG AG AG AA AA
AA GG AG AG AG AG GG AA
AG AG AG GG AG AG AG AA
AG AG AG AG GG AA 10354320
3445 rs10087900 8 AA AG AG AG AA
AG AA AG AG GG AG AG AA
AA AA AG GG GG AG AG AG
AA AA AG AG GG AG GG GG
AG GG AG GG AA AG AA
144303418
3446 rs1008799 4 GG GG AG AG GG
GG AG GG GG GG GG GG GG
GG GG GG GG GG GG GG GG
GG GG GG GG GG GG GG GG
GG AG GG GG GG GG GG
16569814
3447 rs1008800 3 AA AA AA AG AA
AA AG AA AA AA AA AA AA
AA AA AG AG AG AA AA AA
AA AA AA AA AA AG AG AG
AA AG AG AG GG AG AG
190194568
3448 rs10088001 8 GG GG GG GG GG
GG GG GG GG GG GG GG GG
GG GG GG GG GG GG GG GG
GG GG GG GG GG GG GG GG
GG GG GG GG GG GG GG
141838360
3449 rs1008803 10 AG AG GG AG AG
AA GG GG GG GG AG GG AG
GG GG AG AG AA AA AA AG
AG AG AG AG AG GG AG GG
AA GG AA AG GG GG GG
44358300
3450 rs1008812 13 GG GG GG AG GG
AG AG GG GG GG AG AG GG
AG GG GG GG GG AG AG GG
GG AA AG AA GG AG AG AG
AG AG AG AA AG AG AA

23966145
3451 rs10088123 8 GG GG GG GG GG
GG GG GG GG GG GG GG GG
GG GG GG GG GG GG GG GG
GG GG GG GG GG GG GG GG
GG GG GG GG GG GG GG
143319777
3452 rs10088178 8 CC CC CC CC CC
CC AC AC AA AC CC CC CC
CC AC CC CC CC AA CC AC
CC CC CC CC AC CC AC AC
CC CC CC CC AC CC AA
23310904
3453 rs10088201 8 AG GG AG AG AA
AA AG AA AG AG AG AA AA
AA AA AA AA AA AA AA AA
AA AA AA AA AG AA AA AA
AA AA AA AA AG AA AG
53265220
3454 rs10088218 8 GG GG AG GG GG
GG GG GG GG GG GG GG AG
GG GG GG GG AG GG GG GG
GG GG GG GG AG AG AG AA
GG AG GG GG GG GG GG
129543949
3455 rs10088250 8 CC AC AC CC CC
CC CC CC CC CC CC CC CC
CC CC CC CC AC CC CC CC
CC CC CC CC AC AC AC AC
CC AC CC CC AC AC CC
40431991
3456 rs10088262 8 GG GG GG GG GG
GG GG AG AG AA GG GG GG
GG GG AG GG AG GG GG GG
GG AG GG AG GG AA AG AG
GG GG AG AG AG AG GG
124765702
3457 rs10088326 8 GG GG GG GG GG
GG GG GG GG GG GG GG GG
GG GG GG GG GG GG GG GG
GG GG GG GG GG GG GG GG
GG GG GG GG GG GG GG
120058254
3458 rs1008833 1 AA AA AA AG AA
AA AG AG AG AG AG AA AA
AA AG AA AA AA AA AA AA
AA AG AG AG AA AG AA AG
AA AG AA AG AG AA GG
204426295
3459 rs10088339 8 AC CC CC CC CC

CC CC CC CC CC CC CC CC
CC CC CC CC AC CC AC CC
CC CC CC CC CC CC CC CC
CC CC CC CC CC CC AC
114677210
3460 rs1008835
2 -- -- -- -- -- -- -- --
-- -- -- -- -- -- -- --
-- -- -- -- -- -- -- --
-- -- -- -- -- -- -- --
-- -- -- -- 218704238
3461 rs10088359 8 AC AC AC CC CC
CC CC CC CC CC CC CC CC
CC CC CC CC CC CC CC CC
CC CC CC CC CC CC CC CC
CC CC CC CC CC CC CC
24536448
3462 rs10088365 8 GG GG GG GG GG
GG GG GG GG GG GG GG GG
GG GG GG GG GG GG GG GG
GG GG GG GG GG GG GG GG
GG GG GG GG GG GG GG
10097398
3463 rs10088377 8 AG AG AG GG GG
AG AG AA AA AA AG AA AG
AG AA AA AA AA AA AA AA
AA AA AA AA AA AA AA AA
AA AA AA AA AA AA AA
23603179
3464 rs1008838 2 AG AA AA AG AA
AG AA AA AA AA AA AA AA
AA AA AA AA AG AG AG AA
AA AA AA AA AA AA AA AA
AA AG AA AG GG AG AG
37252235
3465 rs10088394
8 -- -- -- -- -- -- -- --
-- -- -- -- -- -- -- --
-- -- -- -- -- -- -- --
-- -- -- -- -- -- -- --
-- -- -- -- 82045215
3466 rs10088399 8 GG GG GG GG GG
GG GG GG GG GG GG GG GG
GG GG GG GG GG GG GG GG
GG GG GG GG GG AG GG GG
GG GG GG GG GG GG GG
141477642
3467 rs10088400 8 AA AC CC AA AA
AC AA AC AC AC CC CC CC
AC AA AC CC AC AC AC AC

	AC	CC	CC	AC	AC	AC	CC	CC
	AC	AC	CC	AC	AA	AA	AA	
39231634								
3468	rs10088462		8	GG	GG	GG	GG	GG
	GG	GG	GG	GG	GG	GG	GG	GG
	GG	GG	GG	GG	GG	GG	GG	GG
	GG	GG	GG	GG	GG	GG	GG	GG
	GG	GG	GG	GG	GG	GG	GG	
92428617								
3469	rs10088476		8	AG	AG	GG	AG	AG
	AG	AG	GG	GG	GG	GG	AG	AG
	AG	GG	GG	GG	GG	GG	GG	GG
	GG	GG	GG	GG	GG	GG	GG	GG
	GG	GG	AG	GG	GG	GG	GG	
34331822								
3470	rs1008854		18	AA	AG	AA	AA	AA
	AA	AG	AG	GG	AG	AG	AG	AG
	AG	AA	GG	AG	AG	AG	AG	GG
	AG	AG	AG	AG	AA	AG	AA	AA
	AA	AA	AG	AG	AG	AG	GG	
25162200								
3471	rs10088564		8	AA	AG	AA	GG	GG
	GG	AG	GG	GG	AG	AA	AG	AA
	AA	AA	GG	GG	GG	AG	GG	AG
	AG	GG	AG	GG	AG	AA	AA	AA
	AG	GG	GG	GG	GG	AG	GG	
2545224								
3472	rs10088578		8	AA	AA	AA	AA	AA
	AA	AA	AA	AA	AA	AA	AA	AA
	AA	AA	AA	AA	AA	AA	AA	AA
	AA	AA	AA	AA	AA	AA	AA	AA
	AA	AA	AA	AA	AA	AA	AA	
127775057								
3473	rs1008858		X	AA	AA	AA	AA	AA
	AA	AA	AA	AA	AA	AA	AA	AA
	AA	AA	AA	AA	AA	AA	AA	AA
	AA	AA	AA	AA	AA	AA	AA	AA
	AA	AA	AA	AA	AA	AA	AA	
139429317								
3474	rs10088644		8	CC	CC	CC	CC	CC
	CC	CC	CC	CC	CC	CC	CC	CC
	CC	CC	CC	CC	CC	CC	CC	CC
	CC	CC	CC	CC	CC	CC	CC	CC
	CC	CC	CC	CC	CC	CC	CC	
6256332								
3475	rs10088657		8	GG	GG	GG	AG	AG
	GG	AA	GG	AG	AG	AG	AA	AG
	AG	AG	GG	AG	GG	GG	AG	AG
	AG	GG	GG	GG	GG	GG	GG	GG
	GG	GG	AG	GG	GG	GG	GG	

73901862
3476 rs10088698 8 GG GG GG GG AG
GG AG GG GG GG GG GG GG
GG GG AG AG GG GG GG GG
AG AG AG GG GG AG AG AG
GG GG GG GG GG AG GG
48654784
3477 rs10088713 8 GG GG GG GG GG
GG GG GG GG GG GG GG GG
GG GG GG GG GG GG GG AG
GG GG GG GG GG GG GG GG
GG GG AG AG AG AG GG
52923780
3478 rs10088735 8 AA AA AA AA AA
AA AA AA AA AA AA AA AA
AA AA AA AA AA AA AA AA
AA AA AA AA AA AA AA AA
AA AA AA AA AA AA AA
16404856
3479 rs10088781 8 CC CC AC AC --
AC AA AC AC AC AC AC CC
AC -- AC CC -- CC AC CC
CC CC CC CC AC AC -- AC
AC AC AC CC -- AC AA
117833190
3480 rs10088800 8 AA AA AA AA AA
AA AG AA AA AG AA AA AA
AA AA AG AG AA AA AA AA
AA AA AA AA AA AG AA AA
AA AA AA AA AG AG AA
65128677
3481 rs10088814 8 CC CC CC CC CC
CC CC CC CC CC CC CC CC
CC CC CC CC CC CC CC CC
CC CC CC CC CC CC CC CC
CC CC CC CC CC CC CC
4846350
3482 rs10088840 8 GG GG GG GG GG
GG GG AG AG AG GG AG AG
AG AA GG GG GG AG AG AG
AG AA AA AG AG AG AG AG
AG AG AG AG AA AG AG
64194110
3483 rs10088860 8 AA AA AA AA AA
AA AA AG GG AG AA AA AA
AA AA AA AA AA AA AG AA
AA AA AA AA AA AA AA AA
AA AA AA AA AA AA AA
81881160
3484 rs10088861

8 -- -- -- -- -- -- -- --
-- -- -- -- -- -- -- --
-- -- -- -- -- -- -- --
-- -- -- -- -- -- -- --
-- -- -- -- 109970076
3485 rs10088869 8 AA AA AA AA AA
AA AA AA AA AA AA AA AA
AA AA AA AA AA AA AA AA
AA AA AA AA AA AG AG AG
AA AA AA AA AA AA AA
136738670
3486 rs10088893 8 AA AG GG AG AG
AA GG AA AA AA GG GG GG
GG AG AG AG GG AG AG AG
AA AA AA AA AG GG GG AG
GG AG AG AG AG AG GG
113054458
3487 rs10088894 8 AA AA AA AA AA
AA AA AA AA AA AG AG AG
GG GG AG AA AG GG AG AG
AG AA AG AA AG AG AG AG
AA AA AG AG AG AG AA
410640
3488 rs10088905 8 AA AA AA AA AA
AA AA AG AG AG AA AA AA
AA AA AA AA AA AA AA AA
AA AA AA AA AA AG AA AA
AA AA AA AA AA AA AA
70648703
3489 rs1008893 3 AG AG AG AA AG
AG AG GG AA AG AG AG AG
AG AA AA AG AG GG AG GG
GG GG GG GG AA AG AA AG
AG GG AG GG AG AG GG
157421613
3490 rs10088949
8 -- -- -- -- -- -- -- --
-- -- -- -- -- -- -- --
-- -- -- -- -- -- -- --
-- -- -- -- -- -- -- --
-- -- -- -- 136772337
3491 rs10089030 8 AG AA AG AA AA
AA AA AA AA AA AA AA AA
AA AA AA AA AG AA AA AA
AA AA AA AA AA AA AA AA
AA AA AG AA AA AA AG
89021717
3492 rs10089035 8 AG GG GG GG GG
GG GG AG AG AG AG GG AG
GG GG AG AG AG AG AG AG

AG AG AG AG GG AG AG AG
GG GG AG AG GG GG GG
115537830
3493 rs10089053 8 GG GG GG GG GG
AG GG GG GG GG GG GG GG
GG GG GG GG GG GG GG GG
GG GG GG GG GG GG GG GG
GG GG GG GG GG GG GG
49498033
3494 rs10089077 8 GG AG GG AG AG
GG AA GG GG GG GG AG GG
GG GG AG AA AG AG AG AA
AG AG GG AA AG AG AG GG
AG AA AG AA AG AG AG
5348088
3495 rs10089111 8 AC AA AC AC AA
AA AC AC AC AC AC AC CC
CC CC AA AC AA AC CC AC
AA AA AA AA AC CC AC CC
AC AC AC AC AC AC CC
89119305
3496 rs10089145 8 AA AA AA AA AA
AG AG AA AA AA AA AA AA
AA AA AA AA AA AA AA AA
AA AA AA AA AA AG AG AG
AG AA AA AA AA AA AA
17237608
3497 rs10089147 8 AA AA AA AA AA
AA AA AA AA AA AA AA AA
AA AA AA AA AA AA AA AA
AA AA AA AA AA AA AA AA
AA AA AA AA AA AA AA
114878624
3498 rs10089171 8 AG AG AA AA AA
AA AG AG AG AA AA AG AG
AG AG AA AA AG AA AG AA
AA AA AA AA AA AA AA AA
AA AG AA AG AA AA AA
29402040
3499 rs10089197 8 GG GG GG AG AG
GG AA AA AA AA AG AG GG
GG GG AG GG GG AG AG AA
GG AG GG AG AG AG AG AG
GG AG AG AA AG AG AG
137364619
3500 rs10089224 8 AG GG GG AA AA
AA AG AA AG GG AA AA AA
AA AA GG GG GG GG GG AG
AG AA GG AG GG AA AG AG
AG AG AG AA AG AA GG

139702939
3501 rs1008924 14 GG AG AG GG GG
GG GG AA AG AA AG AG GG
GG AG AG AG AA GG AG AG
AA AA AG AG GG AG GG AG
AG AG AG GG AA AG AG
52083080
3502 rs10089244 8 AG GG AG AA AA
AA AA AG AG AG AG AA AG
AA AA AG AA AG AG AA AG
AA AA AA AA AG AA AA AG
AG AA AA AA AG AG AA
10176467
3503 rs10089264 8 GG GG GG GG GG
GG AG GG GG GG GG GG GG
GG GG GG GG GG GG GG GG
GG GG GG GG GG GG GG GG
GG GG GG GG GG GG GG
142097004
3504 rs1008927 21 AA AA AA AA AA
AC AA AC AC AA AA AA AA
AA AA AA AA AA AA AA AC
AA AA AA AA AA AA AA AA
AC AA AA AA AA AA AA
39824173
3505 rs10089331 8 GG AG AG AA AA
AG AA GG AG AG AG AG AG
AG AG AG GG AA AG AG AG
AG AA AA AA AG AA AA AA
AG AA AG AG AG AG AG
40569004
3506 rs10089336 8 AG AG GG GG GG
GG GG AG GG GG AG AG GG
GG GG GG AG GG AG AG GG
AG GG GG GG AG AG AG GG
GG AG AG AG AG AG GG
96370246
3507 rs10089487 8 AA AA AG GG GG
GG GG GG GG GG GG GG GG
AG GG AA AG AG AG AG AG
AG GG GG AG GG AG GG GG
AG GG AA AG AG AG GG
38858484
3508 rs1008949 8 AG GG AG AG AG
AG AG GG GG GG GG AG GG
GG AG AG AA AG GG AG GG
AA AA AA AA AA AG AA AA
AG GG AG GG GG AG AG
22553621
3509 rs10089490 8 AA AA AA AA AA

AA AA AG AG AG AA AA AA
AA AA AA AA AA AA AA AA
AA AA AA AA AA AA AA AA
AA AA AA AA AA AA AA
92248453
3510 rs1008953 20 GG GG GG AG GG
AG GG AG AG GG GG GG AG
AG GG GG GG GG GG GG AG
GG GG GG GG AG GG GG GG
GG AG GG GG GG AG GG
43980726
3511 rs1008954 11 AG AG AG AA AA
AA AA AA AA AA AA AA AA
AA AA AA AA AA GG AA AG
AA AA AA AA AA AA AA AA
AA AA AA AA GG AG AG
129974655
3512 rs10089554 8 GG GG GG GG GG
GG GG GG GG GG GG GG AG
GG GG GG GG AG GG GG GG
GG GG GG GG GG AG GG GG
GG GG AG AG GG GG GG
124723379
3513 rs1008956 1 AC AA AC AA AA
AA AC AA AC AA AA AC AA
AA AC AC AA CC AC AC CC
AA AA AA AA AC AA AC AC
CC AC AC CC AC AA AC
237335424
3514 rs10089574 8 CC CC CC AC CC
AC CC CC CC CC CC CC CC
CC CC CC CC CC CC CC AC
CC CC CC CC CC CC CC CC
CC CC CC CC CC CC CC
126063271
3515 rs10089613 8 AG AG AA AA AG
AG AA AG AG AG AG AG AA
AA AA AG AA AG AG AA AA
AG GG AG GG AA AG AA AA
AA AA AA AA AG AG AG
122526046
3516 rs10089625 8 AG AG AG GG GG
GG GG GG GG GG GG AG GG
GG GG GG GG GG GG GG AG
GG GG GG GG GG GG GG GG
GG GG GG GG GG GG GG
58179299
3517 rs10089630 8 AA AC AC AC CC
CC AC AC AA AA AA AA AA
AA AA AA AA AA AA AA AA

AA AA AA AA CC AA AC AC
AC AA AA AA AA AA AA
118862847
3518 rs10089652 8 AA AG AG AG AG
GG AG AG AG GG GG AA AG
AA AA AG AA AA AG AG GG
AG AA AG AA AA GG AG AG
GG AG GG GG AG AG AA
142671483
3519 rs10089707 8 AA AA AA AA AA
AA AA AA AA AA AA AA AA
AA AA AA AA AA AA AA AA
AA AA AA AA AA AA AA AA
AA AA AA AA AA AA AA
38313804
3520 rs10089735 8 AA AG GG GG GG
GG GG AG GG GG GG GG GG
GG AG GG GG AG GG AG GG
GG AG GG AG AA GG AG AG
AG AG AA AA GG GG GG
123769451
3521 rs10089749 8 AG AG GG AG AG
AG AG AA AG AG AA AA AA
AA AG AA AA AG AA AA AG
AG AG AG AG AA AG AA AA
AA AA AA AA AA AG AA
95032386
3522 rs10089769 8 AC AC AC AA AA
AC AA AC AC AA AA AA AA
AA AA AA AC AC AA AA AA
AC AC AA AC AA AA AA AA
AA AC AA AC AC AA CC
146271129
3523 rs10089832 8 AA AA AA AA AA
AA AA AA AA AA AA AA AA
AA AA AA AA AA AA AA AA
AA AA AA AA AA AA AA AA
AA AA AA AA AA AA AA
96077714
3524 rs1008987 1 AA AA AA AA AG
AG AA AA AA AA AG AG GG
AG AG AG AA AA AA AA AA
AA AA AA AA AG AG AA GG
AA AA AG AG AA AA AG
198333063
3525 rs1008988 14 GG GG GG GG GG
GG GG GG AG GG GG GG GG
GG GG GG GG AG AG AG GG
GG GG GG GG GG GG GG GG
AA GG GG GG GG GG GG

78260795								
3526	rs10089891		8	AA	AA	AA	AA	AA
	AA	AA	AA	AA	AA	AA	AA	AA
	AA	AA	AA	AA	AA	AA	AA	AA
	AA	AG	AG	AA	AA	AA	AA	AA
	AA	AA	AG	AA	AA	AA	AA	
4173887								
3527	rs1008998		20	AA	AA	AG	AG	GG
	AG	GG	GG	AG	GG	AA	AA	AA
	AG	AA	AG	AG	AG	AA	AG	AA
	AG	AG	AG	AA	AG	AA	AG	AA
	AG	GG	AG	AG	AG	AG	AG	
51394015								
3528	rs1009	2	AG	AG	AG	AA	AG	AA
	AG	AG	AG	AG	AG	AG	AA	AA
	AA	GG	GG	GG	AG	AG	AA	AA
	AG	AA	AA	GG	AG	--	GG	AG
	AG	AA	AA	GG	AG	AG	85808737	
3529	rs10090024		8	GG	AG	GG	AA	AA
	AG	AA	AG	GG	GG	GG	AG	AG
	AG	AG	GG	GG	GG	AG	AG	GG
	AG	GG	GG	GG	AA	AA	AA	AA
	AG	AG	AG	AG	AG	AG	AG	
92814305								
3530	rs10090064		8	AA	AA	AA	AA	AG
	AA	AG	AA	AA	AA	AA	AA	AA
	AA	AG	AA	AA	AA	AA	AA	AA
	AG	AA	AA	AA	AA	AA	AA	AA
	AG	AA	AA	AA	AG	AA	AG	
92896022								
3531	rs10090071		8	GG	GG	GG	GG	GG
	GG	GG	GG	GG	GG	GG	GG	GG
	GG	GG	GG	GG	GG	GG	GG	GG
	GG	GG	GG	GG	GG	GG	GG	GG
	GG	GG	GG	GG	GG	GG	GG	
108705946								
3532	rs10090077		8	GG	AG	GG	GG	GG
	GG	GG	GG	GG	GG	GG	GG	GG
	GG	GG	GG	GG	GG	--	GG	GG
	GG	GG	GG	GG	GG	GG	GG	GG
	GG	AG	GG	AG	GG	GG	GG	
88743769								
3533	rs10090117		8	AA	AA	AA	AA	AA
	AA	AG	AG	AA	AA	AA	AG	AA
	AA	AA	AA	AA	AA	AA	AA	AA
	AG	AG	AG	AG	AA	AA	AA	AA
	AG	AA	AA	AA	AA	AA	AA	
18381235								
3534	rs1009014		6	AA	AG	AG	AA	AG
	AG	AG	AG	GG	AG	GG	AG	AG

	AG	AG	AG	GG	GG	GG	AG	AG
	AG	AG	GG	GG	GG	AG	AG	GG
	AG	AA	GG	--	GG	AG	GG	
158487143								
3535	rs10090179		8	AG	GG	GG	AG	AG
	AA	AG	GG	GG	AG	AG	GG	AG
	AG	AG	AA	AG	AG	GG	GG	GG
	AG	AG	GG	GG	AG	AA	AA	AA
	AA	GG	AA	AG	AG	AA	AG	
144339053								
3536	rs10090180		8	AA	AA	AA	AA	AA
	AA	AA	AA	AA	AA	GG	AA	AG
	AG	AG	AG	AG	AA	AA	AA	AA
	AA	AA	AA	AA	AG	AA	AA	AA
	AA	AG	AA	AA	AA	AA	AA	
29419564								
3537	rs10090196		8	AG	AG	AG	GG	GG
	AG	GG	AG	GG	GG	AA	AG	AG
	AG	AG	AG	GG	AA	AG	AG	GG
	AG	AG	GG	GG	AG	AG	AA	AG
	AG	GG	GG	GG	AG	AG	GG	
86139996								
3538	rs1009020		18	AA	AA	AA	AA	AA
	AG	AA	AG	AA	AA	AG	AG	AG
	AG	AA	AA	AA	AA	AA	AG	AA
	AG	AG	AA	AA	GG	AG	AG	AG
	GG	AA	GG	AG	AA	AA	AG	
37681908								
3539	rs10090227		8	AA	AG	AA	AA	AA
	AA	AG	AA	AG	AG	AG	AG	AG
	AA	AA	AG	GG	AG	AG	GG	AA
	AA	GG	AG	AG	AG	AG	AA	AG
	GG	AG	AA	AG	AG	AG	AG	
8936962								
3540	rs10090231		8	AA	AA	AG	AG	AA
	AG	AA	AA	AA	AA	AA	AA	AA
	AA	AA	AA	AA	AA	AA	AG	AA
	AA	AG	AA	AG	AG	AA	AA	AG
	AA	AA	AA	AA	AA	AA	AA	
131414083								
3541	rs10090299		8	AA	AG	AG	GG	GG
	GG	AG	AG	GG	AG	AA	AA	AA
	AA	AA	GG	AG	AG	GG	GG	GG
	AG	AG	AA	AA	AG	GG	GG	GG
	AG	GG	AG	GG	GG	AG	AG	
139821059								
3542	rs10090346		8	AA	AG	AG	AA	AG
	AG	AA	AA	AA	AA	AG	GG	AG
	AA	AG	AA	AA	AG	AG	AA	AA
	GG	AG	AG	AG	AA	AG	AG	AG

AG AA GG AG AG AA AG
97630529
3543 rs10090365 8 AA AA AG AG GG
AG AG AA AA AG GG GG AG
AG AG AA AA AA AA AA AG
GG GG AG AG AG AG AG AG
AA AG AG GG AG AG GG
138638199
3544 rs10090367 8 AA AG GG AG AG
AA GG AG GG GG AG AA AA
AG AA AG AG AA AG AG AG
AA AG AG AA AG AG AG AG
GG AG AG GG AA AA AA
36825080
3545 rs10090419 8 AG AG AA AA AG
AG AA AA AA AA AA AA AA
AA AA AA AG AA AA AA AA
AA AA AA AA AG AG AA AG
AA AA AA AA AA AA AA
124028631
3546 rs10090518 8 GG GG GG AG AG
AG GG GG GG GG GG GG GG
GG GG AG GG AG GG GG GG
GG GG GG GG AG GG GG AG
GG GG AG AG GG GG GG
89761913
3547 rs10090519 8 AC AC AC AC CC
AC AC CC CC CC AC AC AA
AA AA AC AC AC AC CC AC
AC AA AC AA CC AC CC CC
AA AC AA AA AC AC AA
129597629
3548 rs10090539 8 GG GG GG AG AG
GG AA AG AG GG GG GG GG
GG GG AG GG AG GG GG GG
AG AG AG AG AG AG AG AA
GG GG GG GG AG GG AG
127344452
3549 rs10090551 8 AG AG AG GG GG
GG GG GG AG AG AG AG AG
AG AG AG AG AG AA AG AG
AA AA AA AA AG AA AG AA
GG GG AA AG AG AG GG
19555762
3550 rs10090564 8 GG GG GG GG GG
GG GG GG GG GG GG GG GG
GG GG GG AG AG GG GG GG
GG GG GG GG GG GG GG GG
GG GG GG GG GG AG GG
18586732

3551 rs10090653 8 AG AA AA AG AA
AG AA AA AA AA AG AG AG
AG AA GG AG AG AG AG GG
AA AG AA AG GG AG GG GG
GG AA AG AA AA AG AA
130383854
3552 rs1009069 5 AA AA AA AG AA
AA AG AA AA AA AA AA AA
AA AA AA AA AA AG AA AA
AA AA AA AA AG AA AA AA
AA AA AG AG AA AA AA
167873938
3553 rs10090737 8 AG AG AG AA AA
AG AG AA AA AG AG AG AA
AA AG AG GG AG AA AG AG
AG AG AA AG AG GG GG AG
AG AA GG AG AG AG AG
21547340
3554 rs10090740 8 AA AA AA AC AA
AA AC AA AA AA AA AA AA
AA AA AA AA AA AA AA AA
AA AA AA AA AA AA AA AA
AA AA AA AA AA AA AA
48468835
3555 rs10090760 8 AG AA AG AG AG
GG AA AA AG GG AG GG AG
AG GG AG GG AA AG AG AG
AA AG AG AG AG GG GG AG
GG AG AA AA AG AG AG
82158027
3556 rs1009077 4 AA AG AG AG AG
GG AG GG GG AG GG GG GG
GG GG GG GG GG AG AG GG
GG GG GG GG AG GG AG AG
GG GG AG AG GG GG GG
120627421
3557 rs10090774 8 GG AG GG AG GG
AG GG AG AG AA AG AA AG
AG AG AG AG AG AG AG AG
AG AG GG GG AG AG AG AG
AG GG AG GG AG AG GG
141826739
3558 rs1009079 2 AA AA AA AA AA
AA AA AA AA AA AA AA AA
AA AA AA AA AA AA AA AA
AA AA AA AA AA AA AA AA
AA AA AA AA AA AA AA
234562574
3559 rs10090819 8 GG GG GG GG AG
GG AG GG AG AG AG GG GG

	GG	GG	GG	GG	AG	AA	AA	AG
	GG	GG	GG	GG	GG	AG	GG	GG
	GG	AG	AG	AA	GG	GG	GG	
49628125								
3560	rs10090875		8	GG	GG	GG	GG	GG
	GG	GG	GG	GG	GG	GG	GG	GG
	GG	GG	GG	GG	GG	GG	GG	GG
	GG	GG	GG	GG	GG	GG	GG	GG
	GG	GG	GG	GG	GG	GG	GG	
49938731								
3561	rs10090896		8	AA	AA	AA	AG	AG
	AA	GG	AA	AA	AA	AG	GG	AG
	AG	GG	GG	GG	GG	GG	GG	GG
	AG	AG	AG	AA	GG	AG	GG	GG
	AG	GG	GG	GG	GG	GG	GG	
70680983								
3562	rs10090974		8	AA	AA	AA	AA	AA
	AA	AA	AA	AA	AA	AA	AA	AA
	AA	AA	AA	AA	AA	AA	AA	AA
	AA	AA	AA	AA	AA	AA	AA	AA
	AA	AA	AA	AA	AA	AA	AA	
125125078								
3563	rs10090994		8	AG	AA	AG	AG	AG
	AG	AA	--	AG	AA	--	AG	--
	--	--	AG	AG	AG	AA	AA	AG
	AG	--	--	--	AG	AG	AA	AA
	AG	--	AA	AG	AA	AA	AG	
59388531								
3564	rs10091027		8	AA	AA	AA	AG	AA
	AG	AA	AA	AA	AA	AA	AA	AA
	AA	AA	GG	AG	AG	AA	AG	AG
	AG	AG	AG	AG	AA	AG	AG	AG
	AA	AA	AA	AA	AA	AA	AA	
126264351								
3565	rs10091038		8	AA	AA	AC	AC	AC
	CC	AA	AC	AA	AC	AC	AA	AA
	AA	AC	AA	AA	AA	AC	AC	AC
	CC	CC	CC	CC	CC	AC	AC	AC
	AC	AC	AC	AA	AC	AA	CC	
29360305								
3566	rs10091057		8	AC	AC	AC	CC	AC
	AC	AC	AA	AA	AC	CC	CC	CC
	CC	AC	AC	AC	AC	CC	AC	CC
	AC	CC	CC	CC	AA	AC	AC	AC
	AA	AC	AC	AC	AA	AC	AA	
77006395								
3567	rs10091077		8	GG	GG	GG	AG	AG
	AA	GG	GG	AG	AA	AA	AA	AA
	AA	AG	GG	GG	GG	GG	AG	GG
	GG	AA	AG	AG	AA	AA	AA	AA

	GG	GG	GG	GG	GG	AG	GG
82705464							
3568	rs10091104		8	AA	AA	AG	GG	AG
	AG	AG	AA	AA	AA	AG	GG	AG
	GG	GG	AA	AA	AA	GG	GG	GG
	AG	AA	GG	AG	AG	GG	AG	AG
	AG	AA	AA	AA	AG	AG	AG	
141327474								
3569	rs1009113		1	GG	GG	GG	GG	AG
	AG	GG	GG	GG	GG	AG	AG	AG
	AG	AG	GG	AG	AG	GG	GG	GG
	GG	GG	GG	GG	GG	GG	GG	GG
	GG	AG	GG	GG	GG	AG	GG	
7143807								
3570	rs10091158		8	AG	AG	GG	GG	GG
	GG	GG	AG	GG	GG	GG	AG	GG
	GG	GG	AG	GG	AG	AA	AA	AG
	GG	AG	AG	GG	AA	GG	AG	AG
	AA	AG	GG	GG	GG	GG	GG	
40809189								
3571	rs1009128							
0	--	--	--	--	--	--	--	--
	--	--	--	--	--	--	--	--
	--	--	--	--	--	--	--	--
	--	--	--	--	--	--	--	--
	--	--	--	--	0			
3572	rs10091280		8	AG	AG	GG	AG	AA
	AG	AA	AG	GG	GG	AA	AA	AA
	AG	GG	GG	GG	AG	GG	GG	GG
	AG	AG	AG	AG	AG	GG	GG	AG
	AG	AG	AA	AA	GG	GG	AG	
123308628								
3573	rs10091303		8	AA	AA	AA	AA	AA
	AA	AA	AA	AA	AA	AA	AA	AA
	AA	AA	AA	AA	AA	AA	AA	AA
	AA	AA	AA	AA	AA	AA	AA	AA
	AA	AA	AA	AA	AA	AA	AA	
93827021								
3574	rs10091322		8	AA	AG	AA	GG	AG
	AG	AG	GG	GG	GG	AG	GG	AG
	AG	AG	GG	AG	GG	GG	GG	AG
	GG	GG	GG	GG	AG	GG	GG	AG
	AG	AG	AG	AG	GG	GG	GG	
89906765								
3575	rs1009133		17	GG	AG	AG	AG	AA
	AG	AG	AG	GG	GG	AG	AG	AG
	AG	AG	AG	GG	AG	AG	GG	AG
	AG	GG	GG	GG	GG	AG	AG	GG
	GG	GG	AG	AG	GG	GG	GG	
21183389								

3576 rs10091332 8 AA AA AA AC AC
AC AC AC AC AC AC AC AC
AC AC AC AA AA AA CC AA
AC CC CC AC AC AC CC AC
CC CC AA AC CC CC CC
18456179
3577 rs10091335 8 GG GG GG GG GG
GG GG GG GG GG GG GG GG
GG GG GG GG GG GG GG GG
GG GG GG GG GG GG GG GG
GG GG GG GG GG GG GG
118305746
3578 rs1009136 19 GG GG AG GG AA
AG AG GG GG GG GG AG AG
GG GG AG AA AG AG GG AG
AA AG AG AG GG AG AG AG
AG AA AA AA GG GG GG
19440428
3579 rs10091360 8 AA AA AA AA AA
AA AA AA AA AA AA AA AA
AA AA AA AA AA AA AA AA
AA AA AA AA AA AA AA AA
AA AA AA AA AA AA AA
71426233
3580 rs1009141 4 AG AG GG AG GG
AG GG AG AG AG GG GG GG
GG AG GG GG GG GG GG GG
GG GG GG GG AG GG GG AG
GG GG AG GG GG GG AG
78139160
3581 rs10091421 8 AC AA AC AC AC
AA AC AA AC AC AC AA AC
AA AA AC AC AA AA AA AA
CC AA AC AC AA AA AA AA
AC AA AA AA AA AA AA
13468201
3582 rs10091430 8 AG AG AG AG GG
AG GG GG GG GG GG GG AG
GG GG AG AA GG GG GG GG
GG GG GG GG GG GG GG GG
AG GG GG GG GG GG GG
125386971
3583 rs10091450 8 AG GG AG AA AA
AA AG GG GG AG AA AA AA
AG AG AG AA AG AA AA AG
AA AA AA AA AG AG AG AA
AG AA AA AA GG GG GG
131247022
3584 rs10091507 8 CC CC CC CC CC
CC CC CC CC CC CC CC CC

CC CC CC CC CC CC CC CC
CC CC CC CC CC CC CC CC
CC CC CC CC CC CC CC
12619338
3585 rs10091511 8 AA AA AG AG AA
AG AA AA AA AA AA AA AG
AA AA AA AA AA AA AA AG
AA AA AA AA AA AA AA AA
GG AA AA AA AG AA AG
129675798
3586 rs1009153
15 -- -- -- -- -- -- -- --
-- -- -- -- -- -- -- --
-- -- -- -- -- -- -- --
-- -- -- -- -- -- -- --
-- -- -- -- 22976911
3587 rs1009164 21 AA AG AG GG GG
GG GG AG AA AG GG GG GG
GG GG GG GG GG AG AG AG
AG AA AG AG AG GG GG AG
AG GG GG GG GG AG GG
22217613
3588 rs10091720 8 GG GG GG GG GG
GG GG GG GG GG GG GG GG
GG GG GG GG GG GG GG GG
GG GG GG GG GG GG GG GG
GG GG GG GG GG GG GG
134128825
3589 rs1009177 20 AC AA AA CC AA
AC AC AC AC CC CC CC CC
CC AC AA AC AC AC CC AC
AC AC CC AC AA AA AA AA
CC AC AA AC AA AA AA
38646455
3590 rs10091799 8 AA AA AA -- AA
-- -- CC -- -- -- AA AC
AC -- AC AA -- CC AA --
AA AA AA AA AA AA AA AA
-- AA -- -- -- AA --
9225872
3591 rs10091812 8 AC AA AC AC AA
AC AA AC CC CC AC CC AC
AC CC AA AC AA CC AC CC
AC AC AC AC AC CC CC AC
CC AA AC AC AC CC AC
94449518
3592 rs10091823 8 AA AA AA AA AA
AA AA AA AA AA AC AC AC
AC AA AA AA AA AA AA AA
AA AA AA AA AA AA AA AA

	AA	AA	AA	AA	AA	AA	AA
139493292							
3593	rs1009188	1	AG	GG	AG	AG	AA
	AG	AA	AA	AG	AA	AG	AG	AA
	AA	AA	AG	AG	AG	AA	AA	AA
	AG	AA	AA	AG	AA	AA	AA	AA
	AA	AA	AA	AA	AG	AA	GG	
197257090								
3594	rs10091936	8	AG	AG	AG	AA	AG
	AA	AG	AA	AA	AA	AG	AA	AG
	AG	AG	AA	AG	AA	AA	AA	AG
	AA	AA	AA	AA	AG	AA	AA	AG
	AA	AG	AG	AG	AA	AA	AA	
89894046								
3595	rs10091964	8	AA	AA	AA	AG	AA
	AG	AA	AG	AA	AA	AG	AG	GG
	AG	AA	GG	GG	GG	GG	AG	GG
	AG	AG	AG	AG	GG	AG	AG	AG
	AG	GG	GG	GG	AA	AA	AA	
123569427								
3596	rs10091996	8	AA	AA	AA	AA	AA
	AA	AA	AG	AA	AA	AG	AG	AG
	AG	AG	AA	AA	AA	AA	AA	AA
	GG	AG	AG	GG	AG	AA	AA	AG
	AG	AA	AG	AA	AA	AA	AA	
13165729								
3597	rs1009200	5	GG	GG	AG	AA	AA
	AG	AA	AG	AG	GG	AG	AG	AG
	AG	AG	GG	GG	GG	GG	AG	AG
	GG	GG	GG	GG	AG	AG	AG	AG
	AG	AG	AG	AG	GG	GG	GG	
73105275								
3598	rs10092003	8	AA	AA	AA	AA	AG
	AG	AA	AA	AA	AA	AG	AA	AG
	AG	AA	AA	AA	AG	AG	AA	AA
	AA	AG	AG	AG	AA	AA	AA	AA
	AA	AG	AA	AG	AA	AA	AA	
96036422								
3599	rs10092006	8	GG	AG	GG	AG	GG
	AG	GG	AA	AA	AA	GG	GG	GG
	GG	GG	GG	AG	AG	AA	AG	AG
	GG	GG	GG	GG	AG	AA	AA	AA
	AA	AG	AG	AA	AA	AA	AA	
5793297								
3600	rs10092021	8	AA	AA	AG	GG	GG
	AG	GG	GG	GG	GG	AG	AG	AA
	AA	AG	AA	AG	AA	AG	AG	AG
	AG	AG	AG	AA	AA	AG	AG	AG
	AA	GG	AA	AG	AG	AG	GG	
38934333								

3601 rs10092053 8 AG -- AG -- --
-- AG AG -- -- -- GG --
-- -- -- -- GG -- -- --
AG -- -- AG -- -- -- --
GG -- -- AG -- AG GG
19109719
3602 rs1009207 21 GG GG GG AG AG
AA GG GG GG GG AG AG GG
AA AG GG GG GG AG GG GG
GG AG AG AG AG GG GG AG
GG AA GG AG GG AG GG
39643666
3603 rs1009213 19 AA AA AG AG AG
AG AG AA AA AA AG AG AG
AA AG AA AA AG AG AA AA
GG AA AG AG AG AG AG GG
AG AG AA AA AG AG GG
49618778
3604 rs10092221 8 AA AA AA AA AG
AG AA AG AG AG AG AA AG
AG AG AG AA AG AG GG GG
GG GG GG GG AG AA AG AG
AG GG GG GG GG AG GG
4536318
3605 rs10092250 8 AC AC CC AC AA
AC AC AC AC AC AC AC AC
AC CC AC AC AC CC AC AC
AA AA AA AA AC AA AA AC
AC AA AC AA AC CC AA
133195856
3606 rs10092286 8 AA AA AA AA AA
AA AA AA AA AA AA AA AA
AA AA AA AA AA AA AA AA
AA AA AA AA AA AA AA AA
AA AA AA AA AA AA AA
38562599
3607 rs10092295 8 AA AA AA GG AG
AG AG AA AA AA AA AA AA
AA AA AA AA AA AA AA AA
AA AA AA AA AA AG AG AG
AA AA AA AA AA AA AA
8101641
3608 rs10092307 8 AG AG AG GG GG
AG AG GG GG AG AA AG AG
GG AA AG AG AG AG AG AG
AA AG AG AG AA AA AA AA
AA GG AA AG AA AG AA
32332633
3609 rs10092310 8 GG AG GG AG AA
AG AG AG AA AA AA AG AG

AG AG GG AG AG AG AA AA
AA AG AG AG AA AA AA AA
AA AA GG AG AA AA AA
75878055
3610 rs10092350 8 GG GG GG GG GG
GG GG GG GG GG GG GG GG
GG GG GG GG GG GG GG GG
GG GG GG GG GG GG GG GG
GG GG GG GG GG GG GG
71656711
3611 rs10092367 8 AA AA AG AA AG
AG AA AA AA AA AA AA AA
AA AA AA AA AG AA AG AA
AA AA AA AA AA AA AA AA
AA AA AA AA AG AG AG
133247202
3612 rs1009238 18 AG AA AG AA AA
AA AG AA AA AA AA AG AG
AG AG AG GG GG AG AG AG
AA AA AA AA AG AA AA AG
AG AA AG AA GG AG AG
2000079
3613 rs10092398 8 AG AG AG GG GG
GG AG AG AG GG GG AG GG
GG GG GG GG GG AG GG AG
GG GG GG GG AA GG AG AG
AA GG AG AG AA AG AA
3713043
3614 rs10092401 8 GG GG GG GG GG
GG GG GG GG GG GG GG GG
GG GG GG GG GG GG GG GG
GG GG GG GG GG GG GG GG
GG GG GG GG GG GG GG
53249098
3615 rs10092405 8 AA AA AA GG AG
GG AG AG AG AG GG GG GG
GG AG GG AG GG AG AG GG
GG AG GG GG GG GG GG GG
GG AA AG AA GG AG GG
76386803
3616 rs10092418 8 AG AG AG AA AA
AG AA GG AG AA GG GG GG
GG GG AA AA AG AA AA AG
AG AG AG AG AG GG AG GG
GG AG AG AG AA AA AG
83265571
3617 rs10092477 8 AA AG AA GG GG
GG GG AG GG GG AA AG AG
AG AG AG GG AG AG GG AG
AG GG GG GG AA GG AG AG

AA AA AA AA GG AG GG
90584054
3618 rs10092482 8 AG AG AG GG GG
GG GG AG GG GG AG AG AA
AA AG AG AG AG AG AG AA
GG GG GG GG AG GG GG GG
GG AG GG GG AG AA GG
67254723
3619 rs10092485 8 AA AA AA AA AA
AA AA AA AA AA AA AA AA
AA AA AA AA AA AA AA AA
AA AA AA AA AG AA AA AG
AA AA AA AA AA AA AA
92495292
3620 rs10092499 8 GG GG GG GG GG
GG AG GG GG GG GG GG GG
GG AG GG AG GG GG GG GG
AG GG AG GG GG GG GG GG
GG GG AG AG GG GG AG
126873484
3621 rs10092513 8 AA AA AA AG AA
AG AG AA AA AA GG AG AG
AA AA AA AA AG AA AA AA
AG AA AA AG AG AG GG GG
AG AA AA AA AA AG AA
40494700
3622 rs10092548 8 GG GG GG GG AG
AG GG GG GG GG AG AG AG
AG AG AG AG GG GG GG GG
GG AG GG AG GG GG GG GG
AG GG AG AG GG AG GG
5676058
3623 rs10092551 8 AG AG AG AA AG
AG AG AG GG AA AG AA AG
AG AG GG GG GG AG AG AG
AG AG AG AG AA AA AA AA
GG AA AG AG AG AG AG
144875945
3624 rs10092594 8 GG GG GG GG GG
GG GG AA AA AG AG AG AG
AG GG GG GG GG GG GG GG
GG GG GG AG AA AG AG AG
AG AG GG AG GG GG GG
132685663
3625 rs10092635 8 AA AA AA AG AA
AG AA AA AA AA AA AA AA
AA AA AA AG AG AG AA AA
AG AG AG AG AA AG AA AG
AG AA AG AG AA AA AA
5456893

3626 rs10092658 8 GG GG GG GG GG
GG GG GG GG GG GG GG GG
GG GG GG GG GG GG GG GG
GG GG GG GG AG GG GG AG
AG GG GG GG GG GG GG
130980472
3627 rs10092695 8 AA AA AA AA AA
AA AA AA AA AA AA AA AA
AA AA AA AA AA AA AA AA
AA AA AA AA AA AA AA AA
AA AA AA AA AA AA AA
117205470
3628 rs1009280 2 GG GG GG GG GG
GG GG AG GG GG AG GG GG
GG GG GG GG GG GG GG GG
GG GG GG GG GG GG GG GG
GG GG GG GG AG AG AG
67775047
3629 rs10092807 8 -- -- -- -- AC
AC AC -- -- -- -- -- --
-- -- -- -- -- AC -- AC
-- AC -- AC -- -- -- --
AC -- -- -- AA AC AC
4269777
3630 rs1009283 2 GG GG GG AG AG
GG AA AG AA AA GG GG GG
GG GG GG AG AG GG GG AG
AG AG AG AG GG GG GG GG
GG AG AA AG AG AG GG
241194081
3631 rs10092876
8 -- -- -- -- -- -- -- --
-- -- -- -- -- -- -- --
-- -- -- -- -- -- -- --
-- -- -- -- -- -- -- --
-- -- -- -- 81225987
3632 rs10092877 8 AG AG AG AA AA
AA AA AG AG AG GG GG GG
GG AG AA AA AA GG AG AG
GG AG AG AG AA AG AA AA
AA AG AA AG GG AG AG
89823675
3633 rs10092878 8 GG GG GG GG GG
GG GG GG GG GG GG GG GG
GG GG GG GG GG GG GG GG
GG GG GG GG GG GG GG GG
GG GG GG GG GG GG GG
62221836
3634 rs10092922 8 GG GG GG AG AG
GG AG GG GG GG GG GG GG

GG GG GG GG GG GG GG GG
GG GG GG GG GG AG GG GG
GG GG GG GG GG GG GG
13807670
3635 rs10092933 8 AA AA AA AA AA
AA AA AA AA AA AA AA AA
AA AA AA AA AA AA AA AA
AA AA AA AA AA AA AA AA
AA AA AA AA AA AA AA
14907298
3636 rs10093057 8 AC AC CC CC CC
CC CC AC AC AC AA AA AA
AA AA AC CC AC CC CC CC
AC CC CC CC AC AC AC AA
CC AC CC AC AC CC AC
104919431
3637 rs10093077 8 AC AC AC AA AA
AA AA AA AA AC AA AA AA
AA AA AA AC AA AA AA AA
AA AA AA AA CC CC CC CC
AC AC AC AA AC AC AA
1110914
3638 rs10093085 8 GG GG GG AA AG
AG AA AG AG AG GG GG AG
AG AG AG AG GG AG AG AA
AA AA AA AA AG AG GG AG
AG AA AG AG GG AG AG
131654000
3639 rs10093149 8 AG AA AA GG GG
GG GG GG GG GG GG GG GG
GG GG GG GG GG GG GG GG
GG GG GG GG AG AG AG AG
AA AG AG AG AA AG AG
141015327
3640 rs10093191 8 AA AA AA AA AA
AA AA AA AA AA AA AA AA
AA AA AA AA AA AA AA AA
AA AA AA AA AA AA AA AA
AA AC AA AC AA AA AA
89411565
3641 rs1009320 12 GG GG GG GG AG
AG GG AA GG GG AA AA AA
AG AA AG GG AG GG GG AG
AG GG AG GG GG AG GG AG
AG GG AG AG AA AG AG
133360964
3642 rs10093305 8 GG AG GG AG AG
AA GG AG AG AG AA AA AA
AA AG GG GG GG AG AA AA
GG GG GG GG AG GG GG AG

AG AA GG AG AA AG AG
88766293
3643 rs10093323 8 AA AG AA AA AA
AA AA AA AA AA AA AG AG
AG AG AA AA AA AA AA AA
AA AG AA AA AA AA AA AA
AA AA AA AA AG AG AA
85295976
3644 rs10093339 8 GG GG AG GG GG
GG GG GG GG GG GG GG GG
AG AG AG AG GG AG GG AG
GG GG GG GG GG GG GG GG
AG AG GG GG AG GG AG
112827868
3645 rs10093351 8 CC CC CC CC CC
CC CC CC CC CC CC CC CC
CC CC CC CC CC CC CC CC
CC CC CC CC CC CC CC CC
CC CC CC CC CC CC CC
8212421
3646 rs10093358 8 GG AG AG AA AA
AA AA AG AG AG AA AA AA
AA AA AG AA AG AA AA AA
AG AA AA AA AG AA AA AG
AG AG AA AA AA AA AA
96913121
3647 rs1009337 22 AG GG GG GG AG
AG GG AG AG GG GG GG GG
GG GG AG GG GG AA AA AG
GG GG GG GG AG AA AG AG
GG GG AG AG AA AG AG
30833372
3648 rs10093373 8 AG GG GG AG AG
GG AG AA AA AG AG AG GG
GG AG AA AA AA AG AG AA
GG AG GG AG AG AG GG AG
AG AG GG AG AG AG GG
132742230
3649 rs10093379 8 AG AG AG GG GG
AG GG AG AG AG AG AG GG
GG AG GG GG AG GG GG AG
GG AG AG AG AG GG AG GG
AG GG AA AG GG GG GG
87448060
3650 rs10093381 8 CC AC CC CC CC
AC CC CC CC CC AC CC AC
AC CC AC AC CC CC CC CC
AC CC CC CC AC AC CC AC
CC CC CC CC AC AC CC
87628371

3651 rs10093390 8 GG GG GG AG AG
AA AG AG AG AG AG AA AG
AG AG GG AG GG GG GG GG
GG AG AA GG GG GG GG GG
GG AG GG GG AG AG GG
26142995
3652 rs10093422 8 AG AA AA AG GG
GG AG AG AG AG AA AA AG
AA AA AG AA GG AG AG AG
AA AA AA AA AG AA AA AG
AG AG AA AA GG AG AG
102207849
3653 rs10093487 8 AA AA AA AA AA
AA AA AA AA AA AG AG AA
AA AA AG AG AA AA AA AA
AA AA AA AA AA AA AA AA
AA AA AG AG AA AA AG
109701780
3654 rs1009350 10 AG AA AA AA AA
AA AA AG AG AA AA AA AA
AA AA AA AG AA AA AA AA
AA AA AA AA AA AA AA AA
AA AA AG AG AG AG AG
125368406
3655 rs10093536 8 AA AG AA AA AG
AA AG AG AG AA AA AA AA
AA AA AG AG AG AA AA AA
AA AA AA AA AA AG AG AG
AA AG AA AA AG AG AA
72994478
3656 rs1009355 7 AG AG AG AG AG
AA GG GG AG GG GG GG AG
AA AA AA AA AG AG AG AA
GG AG AG GG AG AA AA AA
GG AA AA AA AA AA AG
33218763
3657 rs10093573 8 GG GG GG GG GG
GG GG GG GG GG GG GG GG
GG GG GG GG GG GG GG GG
GG GG GG GG GG GG GG GG
GG GG GG GG GG GG GG
49738821
3658 rs10093584 8 GG AG AA GG AG
AG GG AG AG AG GG GG GG
AG AA AA AG AG AG AA AG
AG AG AG AG AA GG AG AG
AG AG AA AG AG AG AA
105863172
3659 rs10093611 8 AA AA AA AG AG
GG AA AA AA AA AA AA AA

	AA	AA	AA	AA	AA	AA	AA	AA
	AA	AA	AA	AA	AA	AG	AA	AA
	AA	AA	AA	AA	AA	AG	AG	
52331018								
3660	rs10093645		8	AA	AA	AA	AA	AA
	AA	AA	AG	AG	AG	AA	AA	AA
	AA	AA	AA	AA	AG	AA	AA	AG
	AG	AA	AA	AG	AG	AG	AA	AA
	AG	GG	AA	AG	AG	AG	AA	
58978805								
3661	rs10093667		8	AA	AC	AA	CC	CC
	CC	CC	CC	CC	AC	CC	AC	CC
	CC	CC	AC	CC	AC	AC	AC	AC
	AC	AC	AC	AC	CC	CC	CC	CC
	CC	AA	CC	AC	CC	CC	CC	
26704875								
3662	rs10093674		8	AA	AA	AA	AA	AA
	AA	AA	AA	AA	AA	AA	AA	AA
	AA	AA	AA	AA	AA	AA	AA	AA
	AA	AA	AA	AA	AA	AA	AA	AA
	AA	AA	AA	AA	AA	AA	AA	
126295849								
3663	rs1009376		9	AG	AA	AG	AA	AA
	AA	AA	AA	AA	AA	AA	AG	AG
	AG	AG	AA	AA	AA	AA	AA	AA
	AG	AA	AG	AA	AA	AA	AA	AA
	AA	AA	AA	AA	AA	AG	AA	
77706531								
3664	rs1009385		22	AG	GG	AG	GG	GG
	GG	GG	GG	GG	GG	GG	GG	GG
	GG	GG	GG	GG	GG	AG	AG	AG
	AG	AG	AA	AG	GG	AG	AG	GG
	GG	GG	GG	GG	GG	GG	AG	
34249434								
3665	rs10093875		8	CC	CC	AC	AC	AC
	CC	AA	AC	AC	AA	CC	CC	CC
	CC	AC	CC	CC	CC	AC	AA	AC
	CC	AC	CC	AC	CC	AC	AC	AC
	CC	CC	AC	AC	CC	CC	CC	
134360780								
3666	rs1009391		16	CC	AC	CC	CC	CC
	CC	CC	CC	CC	CC	CC	CC	CC
	AC	AC	CC	CC	CC	CC	CC	CC
	CC	AC	CC	CC	CC	CC	CC	CC
	CC	CC	AC	CC	CC	CC	CC	
46889354								
3667	rs10093923		8	AA	AA	AA	AA	AA
	AA	AA	AA	AA	AA	AA	AA	AA
	AA	AA	AA	AA	AA	AA	AA	AA
	AA	AA	AA	AA	AA	AA	AA	AA

AA AA AA AA AA AA AA
96355648
3668 rs10093934 8 AC AC AC AA AA
AA AA AA AC AC AA AA AA
AA AC AC AC AC AA AA AC
AC AA AC AC AA AA AA AA
AA AA AA AA AC AC AC
77331219
3669 rs10093950 8 GG GG GG GG GG
GG GG GG GG GG GG GG GG
GG GG GG GG GG GG GG GG
GG GG GG GG GG GG GG GG
GG GG GG GG GG GG GG
48548497
3670 rs10093969 8 GG GG GG GG GG
GG GG GG GG GG GG GG GG
GG GG GG GG GG GG GG GG
GG GG GG GG GG GG GG GG
GG GG GG GG GG GG GG
50046472
3671 rs1009399 2 AA AA AG AG GG
AG GG GG GG GG AG AG GG
AA AA GG AG AG AA AA AG
AG AG GG AG GG GG GG GG
AG AG GG AG AG AA GG
46267270
3672 rs10094036 8 GG GG GG GG GG
GG GG GG GG GG GG AG GG
GG GG GG GG GG GG AG GG
GG GG GG GG AG GG AG GG
AG AA GG AG GG GG GG
15939182
3673 rs10094066 8 AA AG AA AA AA
AA AG AA AA AA AG AG AG
AG AG AG AA AG AA AA AA
AG AG AG AA AA AG AA AA
GG AA AA AA AA AA AG
131540050
3674 rs10094093 8 GG GG GG GG AG
AG GG AG AG AG AG GG AG
GG GG GG GG GG AG GG AA
AG AG AA GG GG GG GG GG
GG AG GG GG AG GG AG
4505914
3675 rs10094094 8 CC CC CC AC CC
CC AC CC CC CC CC CC CC
CC CC CC CC CC CC CC AC
AC CC AC CC CC CC CC CC
CC CC CC CC CC CC CC
112417948

3676 rs10094122 8 AA AC AA AA AA
AA AA CC AC AC AC AC AA
AA AA AA AA AC AA AA AA
AC AC AC AC CC AC AC AC
AC AC AA AC AA AA AC
88356270
3677 rs1009414 20 GG AG AG AG GG
AG GG AA GG AG AA AA AA
AA AA AG AG AG AA AA AG
AA AG AG AA AA AG AA AA
AA AG AG GG AA AA AG
12368613
3678 rs10094238 8 AG AG AG AA AA
AA AA AA AA AA GG AG AG
AG AG AG AA GG AG AA AA
GG GG GG GG AA AA AA AA
AG AA AA AA AG AG AA
85833439
3679 rs10094303 8 AA AA AA AA AA
AA AA AA AA AA AA AA AG
AA AA AA AA AA AA AA AA
AA AA AA AA AG AA AA AG
AA AA AA AA AA AA AA
128218724
3680 rs1009433 22 CC CC CC AC CC
CC AC AC CC CC AC AC CC
CC AC AC AC AC AA AC AC
CC CC CC CC CC AA AC AC
AA CC CC CC AC AC AC
43053813
3681 rs10094425 8 GG GG GG GG GG
GG GG GG GG GG GG GG GG
GG GG GG GG GG GG GG GG
GG GG GG GG GG GG GG GG
GG GG GG GG GG GG GG
72151918
3682 rs10094464 8 GG GG GG GG GG
GG GG GG GG GG GG GG AG
GG GG GG GG GG GG GG GG
GG GG GG GG GG GG GG GG
GG GG GG GG GG GG GG
132165751
3683 rs10094493 8 AG AG GG AA AG
AG AA GG GG GG GG GG GG
GG GG GG AG GG AG GG AG
AG AG AG AG GG AA AG AG
AG GG GG GG AG AG GG
105878832
3684 rs10094541 8 GG GG GG AG AG
AG AG AG GG GG GG GG GG

	GG	GG	AG	GG	GG	GG	AA	GG
	GG	AG	AG	GG	AG	GG	AG	GG
	AG	GG	GG	GG	GG	GG	GG	
19235381								
3685	rs10094564		8	GG	AG	GG	AG	AG
	AA	GG	GG	GG	AG	AA	AA	AA
	AA	AA	AA	AG	AA	AG	AG	AG
	GG	AG	GG	AG	GG	AA	AG	AG
	GG	AG	AG	AG	AG	GG	AA	
673561								
3686	rs10094577		8	AA	AA	AA	AA	AA
	AA	AA	AA	AA	AA	AA	AA	AA
	AA	AA	AA	AA	AA	AA	AA	AA
	AA	AA	AA	AA	AA	AA	AA	AA
	AG	AA	AA	AA	AA	AA	AA	
42087576								
3687	rs10094734		8	AA	AG	AA	AA	AA
	AA	AA	AG	AG	AA	AG	AG	GG
	AG	AG	AA	AA	AG	AA	AG	AA
	AG	AG	AG	AG	AG	AG	AG	GG
	AG	AG	AG	AG	AA	AG	AA	
131503217								
3688	rs10094736		8	AA	AG	AA	AA	AA
	AG	AG	AG	AA	AA	AG	AG	GG
	GG	AG	AG	AG	AG	GG	AG	AG
	GG	AA	AG	AG	AG	AG	AG	GG
	GG	AG	AA	AA	AG	AA	GG	
17130582								
3689	rs10094744		8	GG	GG	GG	GG	GG
	GG	GG	GG	GG	GG	GG	GG	GG
	GG	GG	GG	GG	GG	GG	GG	GG
	GG	GG	GG	GG	GG	GG	GG	GG
	GG	GG	GG	GG	GG	GG	GG	
87162515								
3690	rs10094785		8	AG	AG	AG	AG	AG
	GG	AA	AG	AG	AG	AG	GG	AG
	AG	AG	GG	AG	AG	AG	AA	AG
	GG	AA	AG	AG	AG	AA	AG	AG
	AA	AG	GG	AG	AA	AA	AG	
3976837								
3691	rs10094787		8	AA	AA	AA	AA	AA
	AA	AA	AA	AA	AA	AA	AA	AA
	AA	AA	AA	AA	AA	AG	AA	AA
	AA	AA	AA	AA	AA	AA	AA	AA
	AA	AA	AA	AA	AA	AA	AA	
77000132								
3692	rs10094824		8	AA	AA	AG	GG	AG
	GG	AG	AG	AG	AG	AG	AG	GG
	AG	AA	AG	GG	AG	AG	AG	AG
	AG	GG	AG	GG	GG	AG	AG	AG

AG GG AA AG AA AG AA
117636863
3693 rs10094837 8 AA AA AA AA AA
AA AA AG AG GG AA AA AA
AA AG AG AG AG AA AA AA
AA AA AA AG AG AA AG AG
AA AA AA AA AA AA AG
135205136
3694 rs10094856 8 GG GG GG AG GG
AG GG AG AG GG AG AG GG
GG GG GG GG GG GG GG GG
GG GG GG GG GG AG GG GG
AG AG GG AG GG AG GG
133323802
3695 rs10094913 8 AA AG AG AA AA
AA AA AA AA AA GG AG AG
AA AA AA AG AA AG AG AG
AA AA AA AA AA AA AA AA
AA AA AG AA AA AA AG
108891863
3696 rs10094981
8 -- -- -- -- -- -- -- --
-- -- -- -- -- -- -- --
-- -- -- -- -- -- -- --
-- -- -- -- -- -- -- --
-- -- -- -- 40831294
3697 rs10095131 8 AG AG AG AA AA
AA AA AA AA AA AG AG AG
AA AA AA AG AA AG AG AG
GG AG AG AG AA AA AA AA
AG AG AA AA AA AA AA
57576173
3698 rs10095184 8 AA AA AA AA AA
AA AA AA AA AA AA AA AA
AA AA AA AA AA AA AA AA
AA AA AA AA AA AA AA AA
AA AA AA AA AG AG AA
72226102
3699 rs1009521 3 AG AG AG GG AG
GG AG AA AA AG AG AG AG
AG AG AG AG AG GG GG GG
AG AG GG AA GG GG GG GG
AA AG GG GG AG AG GG
97386798
3700 rs10095228 8 AG AG AG GG GG
AG GG GG AG AA GG GG GG
GG AG AA AA AA AG AA GG
AA AA AA AA AA AG AG AG
AG GG AA AG AA AA AA
21668241

3701	rs10095238		8	GG	GG	GG	GG	GG
	AG	GG	AG	GG	GG	GG	GG	GG
	GG	GG	GG	GG	GG	GG	AG	GG
	GG	GG	GG	GG	GG	GG	GG	GG
	GG	AG	GG	GG	GG	AG	AG	
70857871								
3702	rs10095284		8	AG	AG	AG	GG	GG
	AG	GG	AG	AG	GG	GG	GG	GG
	GG	GG	GG	GG	GG	AG	AG	AG
	AG	GG	GG	AG	GG	AG	GG	GG
	GG	GG	GG	GG	AG	GG	AG	
70469428								
3703	rs10095298		8	AA	AA	AA	GG	GG
	GG	GG	AG	AA	AA	AG	AA	AG
	AG	AG	GG	GG	GG	AG	GG	GG
	AG	AG	AG	AG	GG	AG	AG	AG
	GG	GG	AG	AG	AG	AA	GG	
95484581								
3704	rs10095316		8	AG	AG	AG	GG	GG
	GG	GG	GG	AG	AG	GG	GG	GG
	GG	GG	GG	GG	GG	GG	AG	GG
	GG	AG	AG	AG	GG	AG	AG	AG
	GG	GG	GG	GG	GG	GG	GG	
72062212								
3705	rs10095326		8	AG	AA	AG	AG	AG
	AG	AG	GG	AG	AA	AG	AG	GG
	GG	GG	GG	GG	AG	GG	GG	AG
	GG	GG	GG	GG	AG	AG	AG	AG
	AG	AG	AA	AG	AA	AA	AA	
66073614								
3706	rs10095339		8	AA	AA	AA	--	--
	AA	--	AA	AA	AA	AA	AA	AA
	AA	AA	AA	AA	AA	--	AA	--
	--	--	--	AG	AG	--	--	--
	AA	--	AA	--	--	--	AG	
10287438								
3707	rs10095371		8	AC	CC	AC	CC	CC
	CC	AC	AC	AC	AC	CC	AC	CC
	CC	CC	CC	CC	CC	AC	AA	AC
	AC	CC	AC	CC	AC	AC	CC	AC
	CC	CC	CC	CC	CC	CC	CC	
5667902								
3708	rs10095393		8	GG	GG	GG	GG	AG
	AG	GG	GG	GG	GG	GG	GG	GG
	AG	AG	AG	AG	AG	AG	AG	GG
	GG	AG	GG	AG	GG	GG	GG	GG
	GG	GG	GG	GG	GG	GG	GG	
131686649								
3709	rs1009540		12	CC	CC	CC	CC	CC
	CC	CC	AC	CC	AC	CC	CC	CC

	AC	AC	CC	CC	CC	CC	CC	CC
	CC	CC	CC	CC	CC	CC	CC	CC
	CC	CC	CC	CC	CC	CC	CC	
63414872								
3710	rs10095429		8	AC	AC	AC	AC	AC
	CC	AA	AC	AC	AC	CC	CC	CC
	CC	AC	AC	CC	CC	AC	AC	CC
	AC	AA	AC	AC	AC	AA	AA	AA
	AA	CC	CC	CC	AC	AC	AC	
136563532								
3711	rs10095440		8	--	GG	--	GG	GG
	GG	GG	GG	--	--	GG	GG	GG
	GG	GG	GG	GG	GG	GG	GG	GG
	GG	GG	GG	GG	GG	GG	GG	GG
	GG	GG	GG	GG	GG	GG	GG	
70770391								
3712	rs10095443		8	GG	GG	GG	AA	AA
	AA	AA	AG	AG	AG	AA	AG	AG
	AG	GG	AG	AA	GG	GG	AG	AG
	AG	AA	AA	AA	GG	AG	AG	AG
	AG	GG	AG	GG	AA	AG	AA	
97269738								
3713	rs10095466		8	AC	AC	CC	CC	CC
	CC	CC	CC	CC	CC	AA	CC	AC
	AC	AC	CC	CC	CC	AC	AC	AA
	CC	AA	AA	AC	CC	AA	AC	AC
	AA	CC	CC	CC	CC	CC	CC	
48150346								
3714	rs1009547		7	AG	AG	AG	GG	GG
	GG	GG	GG	GG	GG	GG	GG	GG
	GG	GG	GG	GG	GG	AG	GG	AG
	GG	GG	GG	GG	GG	GG	GG	GG
	AG	GG	GG	GG	GG	GG	GG	
27342727								
3715	rs10095562		8	AA	AA	AA	AA	AG
	AG	AA	AA	AA	AA	AA	AA	AA
	AA	AA	AA	AG	AA	AA	AA	AA
	AA	AA	AA	AA	AG	AA	AG	AA
	AA	AG	AA	AG	AA	AA	AA	
97114632								
3716	rs1009560		X	AA	AG	GG	AG	GG
	GG	AG	GG	GG	GG	GG	GG	GG
	GG	GG	GG	GG	GG	GG	AG	GG
	GG	GG	GG	GG	GG	GG	GG	GG
	GG	GG	GG	GG	GG	GG	GG	
111234241								
3717	rs1009564		22	AG	GG	GG	GG	GG
	GG	GG	GG	GG	GG	GG	GG	GG
	GG	GG	GG	AG	AG	GG	GG	GG
	GG	GG	GG	GG	GG	GG	GG	GG

GG GG GG GG GG GG GG
31386212
3718 rs10095649 8 AG AG AG AA AA
AA AA AG AG AG AA AA AA
AA AG AG AG AG AG AA AA
AA AA AA AG AA AA AA AA
AA AA AG AA AA AG AA
135164948
3719 rs10095650 8 AG GG AG AA AA
AA AA AA AA AA AG AG GG
GG GG GG AG GG AG AG GG
AG AA AG AG AA AA AA AA
GG AG AG AG AG AG AG
82918392
3720 rs10095724 8 AA AG AG GG AG
GG AG AG AG AG GG AG AG
AG GG AG AG AG AA AA AG
AG GG GG AG AA GG AG AG
GG GG GG GG GG GG AG
53739232
3721 rs10095753 8 GG GG GG AG AG
AG GG GG GG GG GG GG GG
GG GG AG GG AG AG AG AG
GG AA AA AG GG GG GG GG
GG GG AG AG GG AG GG
38236890
3722 rs10095762 8 AG AG AG GG GG
GG GG GG GG AG GG GG AG
GG AG AG AG AG AA AA AA
GG AG GG AA AA AG AA AA
GG GG AG GG GG AG AG
129498584
3723 rs10095814 8 AA AG AA AA AA
AA AG AA AA AA AA AA AA
AA AA AA AG AG AA AG AA
AA AA AA AA AG AA AA AA
AA AA AG AG AG AG AA
138877271
3724 rs1009584 9 AA AA AA AA AA
AA AA AA AA AA AA AA AA
AA AA AA AA AA AA AA AA
AA AA AA AA AA AA AA AA
AA AA AA AA AA AA AA
4882386
3725 rs10095849 8 AA AA AA AC AC
CC AA AC AC AC AA AA AA
AA AA AA AA AA AC AC AC
AA AA AA AA AA AA AA AA
AA AC AA AC AC AA CC
39408586

3726 rs10095856 8 GG GG AG GG AG
GG AG GG GG GG GG GG GG
GG GG GG GG GG AG GG AA
GG GG GG GG AG AG AA AG
GG AG GG AG GG GG GG
119470337
3727 rs10095924
8 -- -- -- -- -- -- -- --
-- -- -- -- -- -- -- --
-- -- -- -- -- -- -- --
-- -- -- -- -- -- -- --
-- -- -- -- 3625780
3728 rs10095927 8 CC CC CC AC CC
AC CC AC AC AC AA AC AA
AC AC CC CC CC CC CC AC
AC CC AC CC CC AC CC AC
AA AC AC CC CC AC AC
10729177
3729 rs10095930 8 AA AG AG AA AA
AA AA AA AA AA AA AG AG
AG AA AA AA AA AA AG AA
AG AA AG AA AA AA AA AA
AA AG AG AA AG AG AG
116974302
3730 rs10095940 8 AA AA AA AA AA
AA AA AG AG AG AA AA AA
AA AA AG AA AA AG AG AG
AA AA AA AA AG AG AG AG
AA AG AA AG AG AG AG
137435391
3731 rs10095951 8 CC CC CC CC CC
CC CC AC CC CC CC CC CC
CC CC CC CC CC CC CC CC
CC AC AC AC AC AC AC CC
CC CC CC CC CC CC CC
82463838
3732 rs10096012 8 AG AG AG AG AG
AA AG AA AA AA AA AA AA
AA AA AA AA AA AA AA AA
AA AA AA AA AA AA AA AA
AA AA AA AA AA AG AA
22303439
3733 rs10096019 8 AG AA AG GG GG
GG AG GG GG GG AG AG GG
GG GG GG GG AG AG AG GG
AG AA AA AA AG GG GG GG
AG AG AG AG GG GG GG
66304887
3734 rs10096091 8 AA AA AA AA AA
AA AA AA AA AA AG AA AG

	AG	AA	AA	AA	AA	AA	AA	AA
	AA	AA	AA	AA	AA	AA	AA	AA
	AA	AA	AA	AA	AA	AA	AA	
21243216								
3735	rs10096114		8	AA	AC	AA	AC	AC
	CC	AC	AC	AC	AC	AC	AC	CC
	CC	AC	AC	CC	CC	CC	AC	CC
	AC	AC	AC	AA	AA	AC	AC	AC
	AA	AC	AA	AC	AC	AA	CC	
132401613								
3736	rs10096148		8	AA	AA	AA	AA	AA
	AA	AA	AA	AA	AA	AG	AG	AA
	AA	AA	AG	AG	AA	AA	AA	AA
	AA	AA	AA	AA	AA	AA	AA	AA
	AA	AA	AG	AG	AA	AA	AG	
109710863								
3737	rs1009619		12	AA	AA	AA	AA	AA
	AA	AA	AA	AA	AA	AA	AG	AG
	AA	AG	AA	AA	AA	AG	AA	AA
	AA	AA	AA	AA	AA	AA	AA	AA
	AA	AG	AA	AG	AA	AA	AA	
89554226								
3738	rs1009621		3	AG	AG	AG	GG	GG
	GG	GG	GG	GG	GG	AG	AG	AG
	GG	AG	AG	GG	AA	AG	GG	AG
	GG	GG	AG	GG	AA	GG	AG	AG
	AG	AG	GG	AG	GG	GG	GG	
148190748								
3739	rs1009622		1	GG	GG	GG	AG	GG
	AG	GG	AG	AA	AG	AG	AA	AG
	AG	AG	GG	GG	GG	GG	GG	AG
	GG	GG	GG	GG	GG	GG	GG	GG
	GG	GG	GG	GG	GG	GG	GG	
212412332								
3740	rs10096258		8	AG	GG	AG	GG	AG
	AG	GG	AG	GG	GG	AG	AG	AG
	AG	GG	GG	GG	GG	GG	GG	GG
	AG	AG	GG	GG	GG	AG	GG	GG
	AG	GG	GG	GG	AG	AG	GG	
139604673								
3741	rs10096263		8	AC	AA	AA	AC	AA
	AC	AA	AC	AC	AC	AA	AA	AA
	AA	AA	AC	AA	CC	AA	AC	AA
	AA	AA	AA	AA	AA	AA	AA	AA
	AC	AA	AA	AA	AC	AA	CC	
114763795								
3742	rs10096332		8	GG	GG	GG	AA	AG
	AG	AG	AA	AA	AA	AG	AG	AG
	AA	AA	AG	AA	AG	GG	AG	GG
	AA	AG	AG	AA	GG	GG	GG	GG

GG AG GG GG AG AG AG
10914233
3743 rs10096388 8 AA AG AG AG AG
GG AG AA AG GG GG GG AG
AG AG GG GG GG GG GG GG
GG GG AG AG GG GG GG GG
AA GG AA AG AA AA AG
139745582
3744 rs10096415 8 AG AA AG AG AG
GG AA AG AG AA GG AG AG
AG AA AG AG GG GG AG GG
AA AA AA AA AG GG AG AG
AG GG AG GG AA AG AG
69406281
3745 rs10096434 8 AA AA AA AG AA
AG AA AG AG AA AA AA AG
AG AG AA AG AG AA AA AA
AG AG AG GG GG AG GG GG
GG AA AA AA AA AA AG
138041153
3746 rs10096443 8 AG AG AG AG GG
AG AG AA AA AA AG AG GG
GG AG GG GG AG GG GG AG
GG AG AG AG AG GG AG GG
AG GG AA AG GG GG GG
87453603
3747 rs10096469 8 GG GG GG GG GG
GG GG GG GG GG GG GG GG
GG GG GG GG GG GG GG GG
GG GG GG GG GG GG GG GG
GG GG GG GG GG GG GG
92636368
3748 rs1009647 14 AG AG GG GG AG
GG AG AG AG AG AG GG AG
AG AA AG GG AG GG AG GG
AG AG GG GG GG AG AG GG
GG GG AG AG AG AG AG
55880047
3749 rs10096472 8 AA AA AA AA AA
AA AA AA AA AA AA AA AA
AA AA AA AA AA AA AA AA
AA AA AA AA AA AA AA AA
AA AA AA AA AA AA AA
106353086
3750 rs10096487 8 GG GG GG GG GG
GG GG GG GG GG GG GG GG
GG GG AG AG AG GG GG GG
GG GG GG GG GG GG GG GG
GG GG GG GG GG GG GG
72493864

3751 rs10096496 8 GG AG AA AG GG
AG GG AG AG AG AG AG GG
GG AG GG GG GG GG GG GG
GG GG GG GG AG AG AA AG
GG GG GG GG GG GG GG
121917686
3752 rs10096561 8 GG GG GG AG AG
AG GG GG GG GG GG GG GG
GG GG AG GG GG AG GG GG
GG GG GG GG GG GG GG GG
GG AG AG AA AG GG AG
19720833
3753 rs10096565 8 AA AC AA AA AA
AA AA AA AC AC AA AA AA
AA AA AC CC AA AA AA AA
AA AA AA AA AA AC AA AA
AA AA AA AA AC AA AC
58670352
3754 rs10096596 8 AA AC AA AC AC
AA CC AC AC AC CC AC AC
AC AC CC CC CC CC CC AC
CC AC AC AC AC AA AC AC
AA CC AC AC AC AC AA
73332213
3755 rs10096610 8 GG GG GG AG AG
GG AG GG GG GG GG GG GG
GG GG GG GG GG GG GG GG
GG AG AG GG AG AA AG AA
GG GG GG GG GG GG AG
10289663
3756 rs1009671 2 AC AC CC CC AC
AC CC CC CC AC AC AC AA
CC CC CC AC CC CC CC CC
CC CC CC CC CC AC AC CC
CC CC AC AC CC AC CC
38092846
3757 rs10096732 8 CC CC CC CC CC
CC CC CC CC CC CC CC CC
CC CC CC CC CC CC CC CC
CC CC CC CC CC CC CC CC
CC CC CC CC CC CC CC
48566521
3758 rs1009674 17 GG GG GG GG AG
AG GG AG AG GG AA AA AA
AA AA AG GG GG AG AG AA
AA AA AA AA GG AA AG AG
AA AG GG GG AG AG GG
57468542
3759 rs10096760 8 GG GG AG GG GG
GG GG GG AG AG GG AA AG

AG AG GG GG AG GG GG AG
GG GG GG GG AG AG AG AG
AG AG AG AA GG AG GG
49361164
3760 rs10096780 8 AA AG AA AA AG
AA AG AG GG AG AA AA AA
AA AA AA AA AG AG AG AG
AA AA AA AA AA AA AA AA
AA AG AA AA AG AG AG
77402241
3761 rs10096790 8 AA AA AA GG GG
GG AG AA AA AA AA AG AA
AG AG GG AG AG AA GG AG
GG AG GG AG AA GG AG AG
AA GG AG AG AG AG AA
3600743
3762 rs10096791 8 GG AG AG AG AG
AA GG GG GG GG GG GG AG
AG AG AG GG AA GG AG GG
GG GG GG GG AG AG AG AA
AA AG AG AG GG GG AG
125232152
3763 rs10096833 8 AA AA AG AG AG
GG AA GG GG GG GG GG GG
GG AG GG GG GG AG AG AG
GG AG GG AG AG GG GG GG
AG GG GG GG AG AG GG
136792211
3764 rs1009685 18 CC CC CC AC AC
AA AC AA CC AA CC AC CC
CC CC AC AC CC AC AC CC
AA AA AC AC AC AC AC AA
AC AC CC AC AC AC AC
23041828
3765 rs10096857 8 GG GG GG GG GG
GG GG GG GG GG GG GG GG
GG GG GG GG GG GG GG GG
GG GG GG GG GG GG GG GG
GG GG GG GG GG GG GG
128820864
3766 rs1009686 5 AA AA AA AC AC
CC AA AC AC AC AC AA AA
AC AC AA AC AC AA AC AC
AA AA AA AA AA AC AC AA
AA AA AC AC AC AC AA
129863694
3767 rs10096906 8 AA AA AG AA AA
AA AA GG GG GG AG AG AA
AA AA AG GG AG AA AA AA
AG AG AG AA AA AA AA AA

AA AA AA AA AA AA AA
132132160
3768 rs10096970 8 AA AG AA GG GG
GG GG AG GG GG AA AG AG
AG AG GG GG GG GG GG AG
AG GG GG GG AA GG AG AG
AG AA AG AG GG AG GG
90574472
3769 rs10096979 8 AG AG AA GG GG
GG GG GG GG GG AG GG AG
AG GG GG GG AG GG AG GG
GG GG GG GG GG AA AG AG
AG AG AA AG AG AA AG
97102537
3770 rs10097002 8 GG GG GG GG GG
GG GG GG GG GG GG GG GG
GG GG GG GG GG AG GG GG
GG GG GG GG GG GG GG GG
GG GG GG GG GG GG GG
13713602
3771 rs10097028 8 AA AG AG AA AA
AA AA AG AG AG AG AG AA
AA AA AA AA AA AA AA AG
AA AA AA AA AA AA AA AA
AA AA AG AA AA AG AA
115892114
3772 rs10097031 8 AG GG GG GG AG
AG AG GG GG AG AG AG AG
AG AG AA AG AA AA AA AA
AG AA AG AG AG AA AG AA
AA AA AG AG GG GG AG
131721915
3773 rs1009708 11 GG GG GG GG AG
AG GG AG GG GG GG GG GG
AG GG GG GG GG GG GG AG
GG AG GG AG GG GG GG GG
GG GG GG GG GG GG GG
72345345
3774 rs1009715 17 AA AA AA AA AA
AA AA AA AA AA AA AA AA
AA AA AA AA AA AA AA AA
AA AA AA AA AA AA AA AA
AA AA AA AA AA AA AA
16250624
3775 rs10097220 8 AA AG AA AA AA
AG AA AA AA AA AG AG GG
GG GG AA AG AA AG AA AA
AA AG AG AG AG AG GG AG
GG AA AG AG AA AA AA
92300107

3776 rs10097238 8 GG GG AG GG GG
GG GG GG GG GG AA AA AG
AG AG GG AG AG AG GG GG
AG AG AG AG GG AG AG AG
GG GG GG GG AG AG AG
135910193
3777 rs10097248 8 GG GG GG GG GG
GG GG GG GG GG GG GG GG
GG GG GG GG GG GG GG AG
GG GG GG GG GG GG GG GG
GG AG GG GG GG GG GG
62398829
3778 rs10097250 8 AG AG GG GG GG
GG GG AG AG AG AA AA AA
AA AA AG GG AA GG GG GG
AG GG GG GG AG AG AG AA
GG AG GG AG AG GG AG
104672044
3779 rs1009730 22 GG AG AG GG GG
GG GG GG GG GG AG AG GG
AG GG GG GG GG GG GG GG
AG AG GG AG GG GG GG GG
GG AA AG AA GG AG GG
34260738
3780 rs10097305 8 GG GG GG GG GG
GG GG AA AG AG GG GG GG
GG GG GG GG GG GG GG GG
GG AG GG AG GG GG GG GG
GG GG GG GG AA AG AG
8764824
3781 rs10097321 8 AA AA AA AA AA
AA AA AA AA AA AA AA AA
AA AA AA AA AA AA AA AA
AA AA AA AA AA AA AA AA
AA AA AA AA AA AA AA
130944766
3782 rs10097323 8 GG GG GG AA AA
AA AA AA AA AG AG AG AA
AA AA GG GG AG GG AG AG
AA AG AA GG AA GG AG AG
GG AG GG GG GG GG GG
131641522
3783 rs10097386 8 AG AG AA AG AG
AG AG AG AG AG AG AG GG
GG AG AA AG AG GG GG GG
AA AG AG AG AG AG AG GG
AG AG GG GG AA AA AG
93645236
3784 rs10097481 8 AA AA AA AA AA
AA AA AA AA AG AA AG AG

	AG	AG	AA	AA	AA	AA	AA	AA
	AA	AG	AG	AA	AA	AA	AA	AA
	AA	AA	AA	AA	AA	AG	AA	
72670417								
3785	rs10097488		8	AA	AA	AA	GG	GG
	GG	AG	AA	AA	AG	AG	GG	AG
	AG	AG	GG	AG	GG	GG	GG	GG
	AG	AG	GG	AG	AA	AA	AA	AA
	GG	AA	GG	AG	GG	GG	GG	
27885140								
3786	rs10097508		8	GG	GG	GG	GG	AG
	GG	AG	GG	GG	GG	GG	GG	GG
	GG	GG	AG	AG	GG	GG	GG	GG
	AG	GG	GG	GG	GG	GG	GG	GG
	GG	GG	GG	GG	GG	AG	GG	
48745344								
3787	rs10097529		8	AC	AC	AA	CC	CC
	CC	CC	CC	CC	CC	CC	CC	CC
	CC	CC	CC	CC	CC	AC	AA	AC
	CC	CC	CC	AC	CC	AC	CC	CC
	CC	CC	CC	CC	CC	AC	CC	
133704712								
3788	rs10097597		8	AA	AA	AA	AA	AA
	AA	AA	AA	AA	AA	AG	AG	AG
	AG	AA	AA	AA	AA	AA	AA	AA
	AA	AA	AA	AA	AA	AA	AA	AA
	AA	AA	AA	AA	AA	AA	AA	
139652806								
3789	rs10097622		8	AG	AA	AA	AG	GG
	GG	AG	AG	AG	AG	AA	AG	AG
	GG	GG	GG	AG	GG	AA	AA	AG
	AG	AG	AG	GG	GG	GG	GG	GG
	AG	AG	AG	GG	AG	GG	AG	
57520504								
3790	rs10097671		8	GG	GG	GG	GG	GG
	GG	GG	GG	GG	GG	GG	GG	GG
	GG	GG	AG	AG	AG	GG	GG	GG
	GG	GG	GG	GG	GG	GG	GG	GG
	GG	GG	GG	GG	GG	GG	GG	
72454049								
3791	rs1009773		X	GG	GG	GG	GG	GG
	GG	GG	GG	GG	GG	AA	AG	AG
	GG	GG	GG	GG	GG	GG	GG	GG
	GG	GG	GG	AG	GG	GG	GG	GG
	GG	GG	GG	GG	GG	GG	GG	
144883233								
3792	rs10097735		8	GG	GG	GG	AG	GG
	AG	GG	GG	GG	GG	AG	AG	AA
	AG	GG	GG	GG	AG	GG	AG	AG
	GG	GG	GG	GG	GG	GG	GG	GG

GG AG GG GG GG AG GG
116902508
3793 rs10097743 8 CC AC CC CC CC
AC AC CC AC AC AC CC AC
CC CC AC CC AC CC CC CC
CC AC CC AC CC AC CC CC
CC CC CC CC CC AC AC
14153650
3794 rs10097788 8 GG GG GG GG GG
GG GG GG GG GG GG GG GG
GG GG GG GG GG GG GG GG
GG GG GG GG GG GG GG GG
GG GG GG GG GG GG GG
112572752
3795 rs10097803 8 AG AG AG GG GG
GG GG GG GG AG AG GG AG
AG GG AG AA GG AG GG GG
GG GG GG GG GG GG GG GG
GG GG GG GG GG AG GG
79809610
3796 rs10097833 8 AC CC CC CC AC
AC CC CC CC AC AC CC AC
CC CC CC AC AC CC CC CC
AC AC AC AC AC AC AC AC
CC AC CC AC AC CC AA
142731374
3797 rs10097870 8 AA AA AA AG AG
AA GG AG AG AG GG GG GG
AG AG AG GG AG AG GG AG
GG GG GG GG AA GG AG AG
GG AG AG AA AG AG GG
11444516
3798 rs10097910 8 GG GG GG GG GG
GG GG GG AG AG GG GG GG
GG GG GG GG AG GG GG GG
GG GG GG GG GG GG GG GG
GG AG AG AA GG GG GG
49606500
3799 rs10097923 8 GG GG GG GG GG
GG GG GG GG GG GG GG GG
GG GG GG GG GG GG GG GG
GG GG GG GG AG GG AG AG
GG GG GG GG GG GG GG
40033476
3800 rs10097948 8 AA AA AA -- --
-- -- AA AA AA AG AG GG
AG AA AA AG AG AA AG AA
AG AG AG AG GG AG GG --
AG AA AA AA AA AA AA
101676200

3801 rs10097956 8 AA AG AA AG AG
AG AA GG AG AA AA AA AA
AA AA AA AG AG AG AA AG
AA AG AG AA AA AG AA AA
AG AA AA AA AG AA GG
24094711
3802 rs10098 21 AA AA AA AA AA AG
AA AG AG AG AG AA AG AA
AA AA AG AG GG AA GG AG
AG AG GG AG GG GG AG AG
AG GG GG GG GG GG 46715545
3803 rs10098010 8 AA AA AA AA AA
AA AA AA AA AA AA AA AA
AA AA AA AA AA AA AA AA
AA AA AA AA AA AA AA AA
AA AA AA AA AA AA AA
121641466
3804 rs10098013 8 AG AG AA AG AA
AG AA GG GG GG AG AG GG
GG GG AG AG AG AA AA AG
GG AG AG AG AG GG GG AG
AG GG AG AG AG AA AG
105144079
3805 rs10098057 8 AG AG AG AG AA
AG AG AG AA AA AG AG GG
GG GG AG AG GG AG AG AG
AA AG AG AA AA AG AG AG
AG GG AG AG GG GG AG
51067154
3806 rs10098137 8 GG GG GG GG GG
GG GG GG GG GG GG GG GG
GG GG GG GG GG GG GG GG
GG GG GG GG GG GG GG GG
GG GG GG GG GG GG GG
146060481
3807 rs10098154 8 AA AA AA AA AA
AA AA AA AA AA AA AA AA
AA AA AA AA AA AA AA AA
AA AA AA AA AA AA AA AA
AA AA AA AA AA AA AA
112314853
3808 rs1009820 11 CC CC AC AC AC
AC AC AC AC AC AA AC AC
AA AC AA AC AA CC AC AC
AA CC AA CC AC AC AC AA
AC AA AC AA CC CC AC
105426802
3809 rs10098238 8 AG AG AG AA AA
AA AG AA AA AA AG AG AA
AA AG AG AA AG AG AG AG

	AA	AA	AA	AA	AA	AA	AA	AA
	AA	AG	AA	AG	AA	AG	AA	
66130782								
3810	rs10098245		8	AA	AA	AA	AA	AA
	AA	AA	AA	AA	AA	AA	AA	AA
	AA	AA	AA	AA	AA	AA	AA	AA
	AA	AA	AA	AA	AA	AA	AA	AA
	AA	AA	AA	AA	AA	AA	AA	
50560660								
3811	rs1009825		3	AA	AA	AA	AG	AG
	AA	AG	AA	AA	AG	AA	AG	AA
	AA	AA	AA	AA	AA	AG	AA	AA
	GG	AG	GG	GG	AA	AA	AA	AA
	AA	AA	AG	AG	AA	AA	AA	
10508130								
3812	rs10098379		8	CC	CC	CC	CC	CC
	CC	CC	AA	AA	AC	AC	AC	AC
	AC	CC	CC	CC	CC	CC	CC	CC
	CC	CC	CC	CC	AC	CC	AC	CC
	CC	AC	CC	AC	CC	CC	CC	
132680657								
3813	rs10098474		8	AG	AG	AG	AA	AG
	AG	AA	AA	AA	AA	AA	AA	AA
	AA	AA	AA	AA	AA	AA	AA	AA
	AA	AA	AA	AA	GG	AA	AG	AG
	AA	AA	AG	AG	AA	AA	AA	
9911617								
3814	rs10098494		8	AG	AG	AG	GG	AG
	GG	AG	GG	GG	GG	GG	GG	GG
	GG	GG	GG	GG	GG	GG	GG	GG
	GG	GG	GG	GG	GG	GG	GG	GG
	GG	AG	GG	GG	GG	GG	GG	
75407809								
3815	rs10098545		8	AA	AC	AA	AC	AC
	AA	AC	AA	AA	AC	AC	AC	AC
	AC	AA	AA	AA	AA	AA	AC	AA
	AC	AA	CC	AC	AA	AA	AA	AA
	AA	AA	AC	AC	AA	AA	AA	
138413211								
3816	rs10098632		8	GG	GG	GG	GG	GG
	GG	GG	GG	GG	GG	GG	GG	GG
	GG	GG	GG	GG	GG	GG	GG	GG
	GG	GG	GG	GG	GG	GG	GG	GG
	GG	GG	GG	GG	GG	GG	AG	
95196794								
3817	rs10098655		8	AA	AA	AA	AA	AA
	AG	AA	AG	GG	GG	AA	AG	AG
	AG	AG	AA	AG	AA	AG	GG	AG
	AA	AA	AA	AA	AG	AG	GG	AG
	AA	AA	AA	AA	AA	AA	AA	

90886015								
3818	rs10098667		8	GG	GG	GG	AG	GG
	AG	GG	GG	GG	GG	GG	GG	GG
	GG	GG	GG	GG	GG	AG	GG	AG
	AG	GG	GG	AG	GG	GG	GG	GG
	GG	GG	GG	GG	AG	AG	AG	
8729900								
3819	rs10098680		8	AA	AA	AA	AG	AG
	AA	AG	AA	AA	AA	AG	AG	AA
	AA	AG	AA	AA	AA	AA	AA	AA
	AG	GG	AG	AG	AA	AA	AA	AA
	AG	AA	AG	AG	AA	AG	AA	
131620314								
3820	rs10098707		8	AA	AA	AA	AA	AA
	AA	AA	AA	AC	AC	AA	CC	AC
	AC	AA	AA	AA	AA	AA	AA	AC
	AA	AA	AA	AA	AA	AA	AA	AA
	AC	AA	AC	AC	AA	AA	AA	
49424260								
3821	rs10098726		8	GG	GG	GG	GG	GG
	GG	GG	GG	GG	GG	GG	GG	GG
	GG	GG	GG	GG	GG	AG	AG	AG
	GG	GG	GG	GG	GG	GG	GG	GG
	GG	GG	GG	GG	GG	GG	GG	
91603745								
3822	rs10098729		8	AA	AA	AA	AA	AA
	AA	AA	AA	AA	AA	AG	AG	AA
	AA	AA	AA	AA	AA	AA	AA	AA
	AA	AA	AA	AA	AA	AA	AA	AA
	AA	AA	AA	AA	AA	AA	AA	
128564798								
3823	rs10098811		8	GG	GG	GG	GG	GG
	GG	GG	GG	AG	AG	GG	GG	GG
	GG	GG	GG	GG	AG	GG	GG	GG
	GG	GG	GG	GG	GG	GG	GG	GG
	GG	AA	AG	AA	GG	GG	GG	
49552005								
3824	rs10098874		8	GG	GG	GG	AG	AG
	GG	AG	GG	GG	GG	GG	GG	GG
	GG	GG	GG	GG	GG	GG	GG	GG
	GG	GG	GG	GG	GG	AG	GG	AG
	AG	GG	GG	GG	GG	GG	GG	
11662885								
3825	rs10098898							
8	--	--	--	--	--	--	--	--
	--	--	--	--	--	--	--	--
	--	--	--	--	--	--	--	--
	--	--	--	--	--	--	--	--
	--	--	--	--	41147578			
3826	rs10098922		8	AA	AG	AA	GG	GG

GG AG AG AG AG AG GG AG
AG AA AA AG AA AA AA AG
AA AG AG AA GG AG GG GG
AG GG AA AG AG AG AG
26034528
3827 rs10098937 8 AA AA AA AG AG
GG AA AG AG AG AA AG AG
AG AA AA AA AG AA AA AA
AA AA AA AA AA AA AA AA
AA AG AA AA AG AG AA
123449477
3828 rs10098985 8 CC AC CC AC AC
CC AC CC CC CC AA AA AA
AC AC CC CC CC AC AA CC
CC CC CC CC AC AC AC CC
CC CC AA AC AC AC AC
128355019
3829 rs10098992 8 CC CC CC AC CC
AC CC CC CC CC CC CC CC
CC CC CC CC CC CC CC CC
CC CC CC CC CC CC CC CC
CC CC CC CC CC CC CC
118831856
3830 rs10098995 8 GG GG GG AG GG
GG AG GG GG GG GG GG GG
GG GG GG GG GG GG GG GG
GG GG GG GG AG GG GG AG
GG GG GG GG AG AG GG
102419151
3831 rs10099003 8 AA AA AA AA AA
AA AA AG AG AG AA AA AA
AA AA AA AG AG AA AA AA
AG AG GG GG AA AA AA AA
AA AG AA AA AG AA AG
144645460
3832 rs10099016 8 GG GG GG GG GG
GG GG GG GG GG GG GG GG
GG GG GG GG GG GG GG GG
GG AG AG GG GG GG GG GG
GG AG GG GG GG GG GG
16259612
3833 rs10099034
8 -- -- -- -- -- -- -- --
-- -- -- -- -- -- -- --
-- -- -- -- -- -- -- --
-- -- -- -- -- -- -- --
-- -- -- -- 128267338
3834 rs10099072 8 AA AA AA AA AA
AA AA AG AA AA AA AG AA
AA AA AA AG AG AG AG AA

	AA	AG	AA	AG	AG	GG	AG	AG
	AA	AA	AA	AA	AA	AA	AA	
8437123								
3835	rs10099098		8	AG	AG	AG	AG	AG
	GG	AA	GG	GG	GG	AG	AG	GG
	GG	AG	AG	GG	AG	AG	AG	AG
	AG	GG	GG	GG	GG	GG	GG	GG
	GG	AG	GG	AG	GG	AG	GG	
66134803								
3836	rs10099099		8	AA	AA	AA	AA	AA
	AA	AA	AA	AA	AA	AA	AA	AA
	AA	AA	AA	AA	AA	AA	AA	AA
	AA	AA	AA	AA	AA	AA	AA	AA
	AA	AA	AA	AA	AA	AG	AA	
115908822								
3837	rs10099182		8	CC	CC	CC	CC	CC
	CC	CC	CC	CC	CC	CC	CC	CC
	AC	AC	CC	CC	CC	CC	CC	CC
	CC	CC	CC	CC	CC	CC	CC	CC
	CC	CC	CC	CC	CC	CC	CC	
10112034								
3838	rs1009919		14	AA	AA	AA	AG	AG
	AG	AG	AA	AA	AA	AA	AA	AA
	AA	AA	AA	AA	AG	AA	AA	AA
	AA	AA	AA	AA	AA	AA	AA	AA
	AA	AA	AA	AA	AA	AA	AA	
70385611								
3839	rs10099194		8	AA	AA	AG	AA	AA
	AA	AA	AA	AA	AA	AG	AG	AA
	AA	AA	AG	AG	AG	AA	AA	AA
	AA	AA	AA	AA	AA	AA	AA	AA
	AA	AA	AA	AA	AA	AA	AA	
132145040								
3840	rs10099209		8	GG	GG	GG	GG	GG
	GG	GG	GG	GG	GG	GG	GG	GG
	GG	GG	GG	GG	GG	GG	GG	GG
	GG	GG	GG	GG	GG	GG	GG	GG
	GG	GG	GG	GG	GG	GG	GG	
139221334								
3841	rs1009924		12	AA	AA	AA	AG	GG
	AG	AG	AA	AA	AA	AG	AG	AA
	AG	AG	AG	GG	GG	AG	GG	GG
	AA	AA	AG	AA	AG	GG	GG	GG
	GG	AG	AG	GG	AA	AA	AG	
85167936								
3842	rs10099242		8	AA	AA	AA	AG	AA
	AG	AG	AG	AG	AG	AA	AA	AA
	AA	AA	AG	AA	AA	AA	AA	AA
	GG	AG	GG	AA	AA	AA	AA	AA
	AG	AA	AA	AA	AG	AG	AG	

132960628							
3843	rs10099281	8	GG	AG	AG	AA	AA
	AA	AA	GG	GG	GG	GG	AG	AG
	AG	AG	AA	AG	AA	AG	AA	GG
	AA	AA	AA	AA	AG	AG	AA	AG
	GG	AG	AG	AG	AG	GG	AA	
118476814							
3844	rs10099354	8	CC	CC	CC	CC	CC
	CC	CC	CC	CC	CC	CC	CC	CC
	CC	CC	AC	CC	AC	CC	CC	CC
	CC	CC	CC	CC	CC	CC	CC	CC
	CC	CC	AA	AC	CC	CC	CC	
51577030							
3845	rs10099364	8	AA	AA	AA	AG	AA
	AG	AA	AA	AA	AG	AA	AA	AA
	AA	AA	AA	AA	AA	AA	AG	AA
	AA	AG	AA	AG	AA	AA	AA	AA
	AA	AA	AA	AA	AG	AA	AG	
3069893							
3846	rs10099390	8	AA	AA	AA	AC	AC
	AC	AA	AC	AA	AA	AC	AC	AA
	AA	AC	AC	AC	AA	AA	AA	AA
	CC	AC	AC	AC	AA	AC	AA	AA
	AC	CC	AC	CC	AA	AA	AC	
72909680							
3847	rs1009941	1	AA	AA	AA	AG	AG
	GG	AA	AA	AA	AA	AA	AA	AA
	AA	AA	AA	AG	AG	AA	AA	AA
	AA	AG	AA	AG	AA	AA	AA	AA
	AA	GG	AG	GG	AG	AG	AG	
9275194							
3848	rs10099462	8	AG	AG	GG	GG	AG
	AG	AG	GG	GG	GG	GG	GG	GG
	AG	AG	GG	GG	GG	GG	GG	AG
	AG	AG	AG	AA	GG	GG	GG	GG
	GG	GG	GG	GG	GG	GG	GG	
49156890							
3849	rs1009948	X	GG	GG	GG	GG	GG
	GG	GG	GG	GG	GG	AG	AG	AG
	AG	AG	GG	GG	GG	GG	GG	GG
	GG	GG	GG	GG	GG	GG	GG	GG
	AG	GG	GG	GG	GG	GG	GG	
73053673							
3850	rs10099482	8	AG	AA	AA	GG	GG
	GG	GG	GG	GG	GG	GG	GG	GG
	GG	GG	AA	AG	AA	GG	GG	GG
	AG	GG	GG	GG	AG	GG	AG	GG
	GG	AG	GG	GG	GG	GG	GG	
102690192							
3851	rs10099507	8	GG	GG	GG	AA	AA

AA AA GG AG AG GG AA AG
AA AA AA AA AA AA AA AA
GG GG GG GG GG AA AG AG
GG AG AG AG AA AG AG
142566820
3852 rs10099533 8 CC CC CC AA AC
AC AC CC CC CC CC CC AC
AC AC CC CC CC CC AC CC
CC AC CC AA AA AC AC AC
AC CC CC CC CC CC CC
136294856
3853 rs10099571 8 AA AA AA AA AA
AA AA AA AA AA AA AA AA
AA AA AA AA AA AG AG AA
AA AG AG AA AA AA AA AA
AA AA AA AA AA AA AA
8285302
3854 rs10099602 8 AA AA AA AC AA
AC AA AC CC CC AC CC AC
AC CC AA AC AA CC AC AC
AC AC AC AC AA AC AA AA
CC AA AA AA AA AC AC
94627082
3855 rs10099610 8 AG AG AG AG AG
GG AA GG GG AG GG GG GG
GG AG GG GG AG GG GG AG
GG AA AA AG GG GG GG GG
AG AA GG AG GG GG AG
62337601
3856 rs10099616 8 AG AG AA AA AA
AA AA AA AA AG AA AG AG
AG AA AA AA AA AG AG AA
AA AA AA AA AA AA AA AA
AA AG AA AA AA AA AA
29321914
3857 rs1009962 17 GG GG GG AG AG
GG AG GG GG GG GG GG GG
GG GG GG GG GG GG GG GG
AG GG AG GG GG AG AG AG
AG AG GG AG GG GG GG
59544716
3858 rs1009964 1 CC AC CC AA AA
AA AA AC AA AC AC AC AC
AC AA AC AC AC AA AA AA
AA AC AA AC CC AA AC AC
AA AC AC AC CC AC CC
90265971
3859 rs10099656 8 CC CC CC CC CC
CC CC CC CC CC CC CC CC
CC CC CC CC CC CC CC CC

CC CC CC CC CC CC CC CC
CC CC CC CC CC CC CC
48507687
3860 rs10099670 8 AA AA AA AA AA
AA AA AA AG AG AA AA AA
AA AA AG AG AA AA AA AA
AA AA AA AA AG AA AA AA
AA AG AA AA AA AA AA
73748305
3861 rs10099679 8 AC AC CC CC AC
AC CC AC AC AC AC AC AA
AA AA AA AA AC CC CC AC
AC AC AC AC AC CC AC CC
CC CC AC AC CC CC CC
125752167
3862 rs1009977 14 AC AC AA AA AC
AA AC AC AC AC AC AA AC
AC CC CC AC CC AA AC AA
AA AA AA AA CC AC CC AC
AA AA AC AC AC AC AA
55603002
3863 rs10099784 8 AA AA AA GG AG
AG GG AG AG AG AA AA AA
AG GG AA AG AA AA AG AA
AG AA AG AA AG AA AA AG
AG AG AA AG AA AA AA
121629752
3864 rs10099804 8 GG GG GG GG GG
GG GG GG GG GG AG AG GG
GG AG GG AG GG AG AG AG
GG GG GG GG GG GG GG GG
GG GG AG GG AG AG AG
129449877
3865 rs10099888 8 AA AA AA AA AA
AA AA AA AA AA AG AG AA
AA AA AA AA AA GG AG GG
AG AG AG AG AG AA AG AA
AA AG AG AA AA AG AA
89147717
3866 rs10099900 8 AG GG AG AG AG
GG AG AG AG AG GG AG GG
GG GG GG AG AG AG AG AG
AG GG AG GG AG GG AG GG
AG AA AG AA AG GG AG
15935790
3867 rs10099909 8 AA AA AA AA AA
AA AA AA AA AA AA AA AA
AA AA AA AA AA AA AA AA
AG AA AG AA AA AG AA AG
AA AA AA AA AA AA AA

11287371							
3868	rs10099981	8	AG	GG	AG	GG	GG
	GG	GG	GG	GG	GG	GG	GG
	GG	GG	GG	GG	GG	GG	GG
	GG	GG	GG	GG	AG	GG	GG
	AG	GG	GG	GG	GG	AG	GG
73198650							
3869	rs10100001	8	CC	CC	CC	AA	AC
	AC	AA	CC	CC	CC	CC	CC
	CC	CC	AC	AA	AC	CC	CC
	AC	AC	AC	AC	AC	CC	AC
	AC	AC	AC	AA	CC	AC	CC
5617213							
3870	rs10100025	8	GG	GG	GG	GG	GG
	GG	GG	GG	GG	GG	GG	GG
	GG	GG	GG	GG	GG	GG	GG
	GG	GG	GG	GG	GG	GG	GG
	GG	GG	GG	GG	GG	GG	GG
141716284							
3871	rs1010003	18	AA	AA	AA	AA	AA
	AA	AA	AA	AG	AA	AA	AG
	AG	AG	AG	AA	AG	AA	AA
	GG	GG	GG	GG	AA	AA	AA
	AA	AA	AA	AA	AG	AG	AG
965990							
3872	rs10100072	8	GG	AG	AA	AA	AA
	AA	AA	AA	AA	AA	AA	AA
	AG	AG	AA	AA	AG	AA	AA
	AA	AG	AG	AG	AG	AA	AA
	AG	AA	AA	AA	GG	AG	AG
39152863							
3873	rs10100122	8	AA	AA	AA	AA	AA
	AA	AA	AA	AA	AA	AA	AA
	AA	AA	AA	AA	AA	AA	AA
	AA	AA	AA	AA	AA	AA	AA
	AA	AA	AA	AA	AA	AA	AA
118163334							
3874	rs10100132	8	GG	AG	GG	AG	AG
	AA	GG	GG	GG	GG	AG	AG
	AG	AG	GG	GG	GG	GG	GG
	GG	GG	GG	GG	GG	AG	AG
	AG	GG	GG	GG	GG	AG	GG
706008							
3875	rs10100186	8	CC	AC	AA	AC	AC
	AC	AA	AC	AA	AA	AA	AA
	AA	AA	AC	AA	CC	AA	AA
	AA	AA	AA	AA	AA	AC	AA
	AA	AC	AA	AA	AC	AA	CC
36727529							
3876	rs10100191	8	AA	AA	AA	AG	AA

AA AG AG AG AG AA AA AA
AA AA AA AA AA AG AA AA
AA AA AA AA AA AA AA AA
AA AA AG AG AG AA AG
97559162
3877 rs10100209 8 AG GG AG AA AG
AG AA AG GG GG AA AA AA
AG AG GG AG AG AG AA AG
AG AA AA AG AG AA AG AA
AA AG AG GG AG AG AA
10584212
3878 rs10100237 8 GG AG AG AA AA
AA AA AG AG AG AG AG AG
AG AG AG AA AA AG AA AA
AA AA AA AA AA AG AA AA
AA AG GG AG AA AA AG
135328582
3879 rs10100238 8 AG AA AA AG AA
AG AG AG AG AG AA AA AA
AA GG AG AG AA GG GG AG
AG AG AG AG GG AG AG AG
AG AA AG AA AA AG AA
58962794
3880 rs1010028 2 AG AG AG AG AA
AG AG GG GG GG AG AG AA
AA AA GG AG AG AG AG AA
AG GG AG AG AG AG AA AG
AA AG AA AG AA AG AA
175418960
3881 rs10100391 8 GG AG GG GG GG
GG GG GG GG GG GG AG GG
GG GG GG AG AG GG GG GG
GG GG GG GG GG GG GG GG
GG AG GG AG GG GG GG
19585176
3882 rs10100395 8 AG AG AG AA AA
AA AA AA AA AG AG GG AG
AG AA AG AA AG AG AG AG
AA AG AA AG AA AA AA AA
AG AA AA AA AA AA AA
124495368
3883 rs10100402 8 AA AA AA AA AA
AA AA AA AA AA AA AA AA
AA AA AA AA AA AA AA AA
AA AA AA AA AA AA AA AA
AA AA AA AA AA AA AA
49713458
3884 rs1010041 X AA AC AC AC AA
AA AC AC AC CC AA AA AA
AA AA AC AC CC AC AC AA

	AA	AA	AA	AA	AC	AA	AA	AA
	AC	AA	CC	AA	AA	AA	AA	
3019465								
3885	rs10100465		8	AG	AG	AG	AG	GG
	AG	GG	GG	AG	AG	GG	GG	GG
	GG	GG	GG	GG	AG	AG	GG	AG
	GG	GG	GG	GG	GG	GG	GG	GG
	GG	GG	GG	GG	GG	AG	GG	
118626279								
3886	rs10100487		8	GG	AG	GG	AG	AG
	GG	AA	AG	AG	AG	AA	AG	AG
	AG	AG	AA	AA	AA	AA	AA	AG
	AA	AG	AG	AG	AG	GG	AG	AG
	GG	AA	AG	AG	AG	AG	GG	
73294051								
3887	rs10100514		8	AA	AA	AA	AA	AG
	AA	AG	AA	AA	AA	GG	AG	AG
	AG	AA	AA	AA	AA	AA	AA	AG
	AA	AG	AG	AG	AG	AA	AG	AA
	AA	AA	AA	AA	AA	AA	AA	
96985292								
3888	rs10100523		8	GG	GG	GG	GG	GG
	GG	GG	GG	GG	GG	GG	GG	GG
	GG	GG	GG	GG	GG	GG	GG	GG
	GG	GG	GG	GG	GG	GG	GG	GG
	GG	GG	GG	GG	GG	GG	GG	
126372097								
3889	rs1010053		14	AG	AG	AA	AA	AA
	AA	AA	AG	AG	AG	AG	AG	AA
	AG	AG	AG	GG	GG	AA	GG	AG
	GG	GG	GG	GG	GG	AG	GG	AG
	GG	AG	AA	AA	AA	AA	AA	
61005625								
3890	rs10100569		8	AA	AA	AA	AA	AA
	AA	AA	AA	AA	AA	AA	AA	AA
	AA	AA	AA	AA	AA	AA	AA	AC
	AA	AA	AA	AA	AA	AA	AA	AA
	AA	AA	AA	AA	AA	AA	AA	
114349314								
3891	rs10100582		8	AG	AG	GG	AG	AG
	AG	AG	AG	GG	GG	AG	AG	GG
	GG	GG	GG	GG	GG	GG	GG	GG
	GG	GG	GG	GG	GG	GG	GG	GG
	GG	GG	GG	GG	GG	GG	GG	
94092846								
3892	rs10100593							
8	--	--	--	--	--	--	--	--
	--	--	--	--	--	--	--	--
	--	--	--	--	--	--	--	--
	--	--	--	--	--	--	--	--

	--	--	--	--	13619237		
3893	rs10100603		8	GG	GG	GG	GG	GG
	GG	GG	GG	GG	GG	AG	AG	GG
	GG	GG	GG	GG	AG	GG	GG	GG
	GG	GG	GG	GG	GG	GG	GG	GG
	GG	GG	GG	GG	AG	AG	GG	
94868279								
3894	rs10100606		8	GG	AG	AG	AG	AG
	AA	GG	AG	GG	GG	AA	GG	AG
	GG	GG	AG	GG	GG	GG	GG	AG
	AG	GG	AG	GG	GG	AG	AG	AG
	GG	GG	AG	AG	AG	AG	GG	
142677833								
3895	rs10100608		8	AA	AA	AA	GG	AG
	AG	GG	GG	GG	GG	AA	AG	AG
	AG	AG	GG	GG	AG	GG	GG	AG
	AG	AG	AG	AG	GG	AG	GG	GG
	GG	GG	GG	GG	GG	GG	GG	
95056476								
3896	rs1010069		1	AG	AG	AA	AG	AG
	GG	AA	AG	AG	AA	AG	AG	AG
	--	AG	AA	AA	AA	AG	AG	GG
	AA	AA	AG	AA	GG	AG	AG	AG
	AG	AA	GG	AG	AG	AG	AG	
16352937								
3897	rs10100706		8	AA	AA	AA	GG	AG
	AG	GG	AG	AA	AA	AG	AG	AG
	AA	AA	AA	AG	AG	AA	GG	AA
	AG	GG	GG	AG	AA	GG	AG	AG
	GG	AA	AA	AA	AG	AG	AG	
9321780								
3898	rs10100741		8	AG	AG	AG	GG	GG
	GG	GG	GG	GG	AG	GG	GG	GG
	GG	GG	AG	GG	AG	GG	GG	GG
	GG	GG	GG	GG	AA	AG	AA	AA
	AG	AG	AA	AG	AG	GG	AG	
59321015								
3899	rs10100885		8	GG	GG	GG	GG	GG
	GG	GG	AA	AA	AG	GG	AG	GG
	GG	GG	AG	AG	GG	GG	GG	GG
	GG	AG	AG	GG	GG	GG	GG	GG
	GG	GG	GG	GG	GG	AG	GG	
2117238								
3900	rs10100935		8	AG	AG	AA	AG	AG
	AA	GG	GG	AG	GG	GG	AG	GG
	GG	GG	AG	AA	AA	AA	AA	AA
	GG	GG	GG	GG	GG	AA	AG	AG
	GG	AG	AA	AA	AG	GG	AA	
143425720								
3901	rs10100940		8	GG	GG	GG	AG	AG

	AG	GG	AG	GG	GG	AA	AA	AA
	AA	AA	AA	AA	AA	AG	GG	AA
	AG	GG	AG	GG	AA	AA	AA	AA
	AG	AG	AG	GG	GG	AG	AG	
13918143								
3902	rs1010096		12	AG	AA	AG	AG	AG
	AG	AG	AA	AA	AA	AG	AA	AA
	AA	AA	AA	AA	AA	AA	AA	AA
	AA	AA	AA	AA	AA	AA	AA	AA
	AG	AA	AG	AA	AG	GG	AG	
27844230								
3903	rs10100960		8	AA	AG	AG	AA	AA
	AA	AA	AG	AA	AA	GG	GG	GG
	GG	GG	AA	AG	AA	AG	AA	AA
	AA	AA	AA	AA	AA	AA	AA	AA
	AG	AG	AG	AG	AG	AG	AA	
119696794								
3904	rs10101006		8	AA	AC	AA	AA	AA
	AA	AA	AA	AA	AC	AA	AA	AA
	AA	AA	AA	AA	AA	AC	AC	AC
	AC	AC	AA	AA	AA	AC	AA	AA
	AA	AC	AC	CC	AC	AA	AC	
141121745								
3905	rs10101023		8	GG	GG	GG	GG	GG
	GG	GG	GG	GG	GG	GG	GG	GG
	GG	GG	GG	GG	GG	GG	GG	GG
	GG	GG	GG	GG	GG	GG	GG	GG
	GG	GG	GG	GG	GG	GG	GG	
61692247								
3906	rs10101071		8	GG	AG	GG	GG	GG
	GG	GG	GG	GG	GG	GG	GG	GG
	GG	GG	GG	GG	GG	GG	GG	GG
	GG	GG	GG	GG	GG	GG	GG	GG
	AG	GG	AG	AG	GG	GG	GG	
141054583								
3907	rs10101073		8	AG	AG	AG	AA	AA
	AA	AA	AG	AG	AG	GG	GG	GG
	AG	AA	AG	GG	AA	GG	AG	AG
	AA	AA	AA	AA	AA	AA	AA	AA
	AA	AG	AA	AA	AG	AA	GG	
112279583								
3908	rs10101082		8	GG	GG	GG	GG	GG
	GG	GG	GG	GG	GG	GG	GG	GG
	AG	AG	GG	GG	GG	GG	GG	AG
	GG	GG	GG	GG	GG	GG	GG	GG
	AG	AG	GG	GG	GG	GG	GG	
117145998								
3909	rs10101109		8	AG	GG	AG	AG	AG
	AG	AG	AA	AA	AA	AG	AA	AA
	AA	AG	AA	AA	AA	AG	AA	AG

AA AA AA AA AA AG AA AA
AA AA AA AA AA AA AA
16087631
3910 rs10101117 8 CC CC CC CC CC
CC CC CC CC CC AC CC CC
CC AC AC AC CC AC CC AC
AC CC AC CC CC AC CC CC
AC AC AC AA AC AA CC
29802727
3911 rs10101155 8 CC AC CC AC AC
CC AA AC AC CC AC AC CC
CC CC AC CC AC CC AC CC
AC CC CC CC CC CC CC CC
AC CC AA AC CC AC AC
72972017
3912 rs10101168 8 GG GG GG AG AG
AG GG GG GG GG GG GG GG
GG GG AG GG AG AG AG AG
GG AA AA AG GG GG GG GG
GG GG AG AG GG AG GG
38193518
3913 rs10101195 8 CC CC CC AC AC
AC AC AC AC AC AA AA AA
AC AC CC AC AC AC AC AC
CC AA AC AC AC AC AC AC
AC AC CC CC CC AC AC
11623212
3914 rs10101200 8 AA AA AA GG GG
AG AG AA AA AA AG AA AA
AA AG AG AA AG AG AG AA
AA AG AG AA AG AG AA AG
AA AG AA AG AA AA AA
16339528
3915 rs10101252 8 AA AA AA AG AG
AA AG AA AA AA AA AG AA
AG AG AA AA AA AA AG AA
AA AA AA AA AG AA AA AA
AA AA AA AA AA AA AA
3389173
3916 rs1010127 5 AA AG AG AG AG
GG AA GG AG AG AA AG AA
AA AA AG AA AG AG GG GG
AG AG AG AG AA AA AA AA
GG AG AG AG AA AA AG
92226214
3917 rs10101286 8 AG AA AA AG AG
GG AG AA AA AG GG AA AG
AG AG GG AG AG AA AA AA
AG AG AA AA AG GG GG GG
GG AA GG AG AG GG AG

144376938
3918 rs1010133 X CC CC AC AC CC
AA CC AC AC CC CC CC AC
AC CC CC CC CC CC CC CC
CC CC CC CC AC CC AC AA
CC CC CC CC CC CC CC
4045697
3919 rs10101334 8 AA AA AA AA AA
AA AA AA AA AA AA AA AA
AA AA AA AA AA AA AA AA
AA AA AA AA AA AA AA AA
AA AA AA AA AA AA AA
141884599
3920 rs10101336 8 AC CC AC AA AC
AC AC CC CC CC CC CC CC
CC CC AC AC AC AC AA AC
CC CC CC CC CC AC AC AC
AC CC AA AC CC CC CC
72329553
3921 rs10101375 8 AA AA AA AA AG
AA AG GG AG AG AA AA AA
AA AA AA AA AA AA AG AA
AA AA AA AA AA AA AA AA
AA AA AA AA AA AA AA
122832434
3922 rs10101376
8 -- -- -- -- -- -- -- --
-- -- -- -- -- -- -- --
-- -- -- -- -- -- -- --
-- -- -- -- -- -- -- --
-- -- -- -- 1244374
3923 rs10101451 8 CC CC CC CC CC
CC CC CC CC CC CC CC CC
CC CC CC CC CC CC CC CC
CC CC CC CC CC CC CC CC
CC CC CC CC CC CC CC
91664583
3924 rs10101508 8 CC CC CC AC CC
CC AC -- CC CC CC -- --
-- -- CC -- AC -- CC CC
-- -- CC -- -- CC CC --
-- -- CC CC CC CC CC
121398874
3925 rs10101523 8 AG AA AG GG AG
AG GG AA AG AG GG GG GG
GG GG AG GG AA AA AA AG
GG GG GG GG AA AG AA AA
GG GG AG GG GG AG GG
95770914
3926 rs10101539 8 AA AA AA GG GG

	AG	GG	AA	AG	AG	AG	AA	AG
	AG	AA	AG	AG	AA	GG	GG	AG
	AA	AA	AA	AA	GG	AG	AG	AG
	AG	AG	AG	AG	AG	GG	AA	
72701380								
3927	rs1010156		8	AG	AG	AG	AG	AG
	AA	AG	AG	AG	AG	GG	AG	AG
	AG	AG	AG	AA	AG	AG	GG	AA
	AG	AG	AG	AG	AA	GG	AG	AG
	AG	AG	AG	GG	AA	AA	AG	
23190941								
3928	rs10101680		8	GG	GG	GG	AG	GG
	GG	AG	GG	GG	GG	GG	GG	GG
	GG	AG	GG	GG	GG	GG	GG	GG
	AG	AG	AG	AG	GG	GG	GG	GG
	GG	GG	AG	AG	GG	GG	GG	
89027706								
3929	rs10101779		8	AA	AA	AA	AG	AG
	GG	AG	AG	AG	AG	AA	AA	AA
	AA	AG	AA	AA	AA	AA	AA	AA
	AG	AA	AG	AG	AG	AG	AG	AG
	AA	AA	AA	AA	GG	GG	AG	
27450535								
3930	rs10101790		8	GG	GG	GG	AA	AA
	AA	AG	AG	AG	GG	GG	GG	GG
	AG	AG	AG	AA	AG	AG	AG	GG
	GG	AG	GG	AA	GG	GG	GG	GG
	GG	GG	GG	GG	GG	AG	GG	
131693352								
3931	rs10101821		8	AG	AG	AG	AG	AA
	AA	AG	AG	AG	AG	AA	AA	AA
	AA	AG	AA	AA	AG	AA	AA	AA
	AG	AG	AG	AG	AG	AA	AG	AA
	AG	AG	GG	GG	AA	AA	AG	
88590643								
3932	rs10101843		8	GG	GG	GG	GG	GG
	GG	GG	GG	GG	GG	GG	GG	GG
	GG	GG	GG	GG	GG	GG	GG	GG
	GG	GG	GG	GG	GG	GG	GG	GG
	GG	GG	GG	GG	GG	GG	GG	
96053089								
3933	rs10101872		8	AA	AC	CC	AA	AA
	AA	AA	AC	AC	AC	AC	CC	AC
	AC	AA	AC	AC	AA	AA	AA	AC
	AC	AC	AC	AC	AC	AA	AA	AC
	AA	AA	AC	AA	CC	AC	CC	
114814714								
3934	rs10101931		8	AA	AA	AA	AC	CC
	CC	AC	AA	AA	AA	AC	AA	AC
	AC	AA	CC	AC	AC	AA	AC	AC

	AA	AC	AC	AC	CC	CC	CC	CC
	AC	AC	AC	AC	AC	AC	AC	
98237755								
3935	rs10101943		8	AA	AA	AA	CC	AA
	AC	AC	AA	AC	AC	AA	AA	AA
	AA	AA	AA	AC	AA	AC	AA	AC
	AC	AA	AC	AA	AA	AA	AA	AA
	AC	AA	AC	AC	AC	AC	AC	
121089222								
3936	rs10101991		8	AA	AA	AA	AA	AA
	AA	AA	AA	AA	AA	AA	AA	AA
	AA	AA	AA	AA	AA	AA	AA	AA
	AA	AA	AA	AA	AA	AA	AA	AA
	AA	AA	AA	AA	AA	AA	AA	
121082057								
3937	rs10102037		8	GG	GG	GG	GG	GG
	GG	GG	GG	GG	GG	GG	GG	GG
	GG	GG	GG	GG	GG	GG	GG	GG
	GG	GG	GG	GG	GG	GG	GG	GG
	GG	GG	GG	GG	GG	GG	GG	
130815151								
3938	rs10102049		8	GG	GG	GG	AA	AG
	AA	AG	AA	AG	AG	AG	AG	GG
	GG	GG	AG	AG	AG	GG	GG	GG
	AG	AA	AA	AA	GG	AG	AG	AG
	AA	GG	AA	AG	AA	AA	AG	
90289326								
3939	rs10102107		8	AG	AG	AG	GG	AG
	GG	AG	AG	AG	AG	AA	AA	AA
	AA	AG	AA	AA	AG	AA	AA	AG
	GG	GG	GG	GG	AG	AG	AG	GG
	AG	AG	GG	GG	AG	GG	AA	
112325064								
3940	rs10102170		8	--	AG	AG	AG	--
	AG	GG	AG	AG	AG	GG	GG	GG
	GG	GG	AG	GG	AG	AG	GG	AG
	AG	GG	GG	AG	GG	GG	GG	GG
	GG	GG	GG	GG	AG	AG	GG	
9611470								
3941	rs10102187		8	AC	AC	AC	AC	AC
	AC	CC	AC	CC	CC	CC	CC	CC
	CC	CC	AC	AC	AC	AC	CC	AA
	AA	CC	AC	AC	AA	AC	AC	AC
	AC	CC	AA	AC	CC	CC	CC	
18962087								
3942	rs10102211		8	CC	CC	CC	CC	CC
	CC	CC	CC	CC	CC	CC	CC	CC
	CC	CC	CC	CC	CC	CC	CC	CC
	CC	CC	CC	CC	CC	CC	CC	CC
	CC	CC	CC	CC	CC	CC	CC	

125258381
3943 rs1010222 19 AA AA AG GG AG
GG AG AG GG GG GG AA AG
AA AA GG GG GG GG AG GG
AG AG AG AA GG AG GG GG
AG AA GG AG AG AG AA
13048608
3944 rs10102248 8 GG GG GG AG AG
GG AG AA AA AG GG AG GG
GG GG AA AG AG AA AG AG
GG AG AG GG GG GG GG GG
AA AG AG AA AG AA GG
3426669
3945 rs10102256 8 AC AA AC AC AA
AA AC AA AA AA AA AC AC
AC CC CC AC CC AC AC CC
AA AA AA AA AA AC AA AA
AC AA AC AA AA AC AA
78216194
3946 rs10102300 8 GG GG GG GG GG
GG GG GG AG AG GG AG AG
AG GG AG GG AG GG GG GG
GG AG AG AG GG AA AG AG
AG GG AG GG GG GG GG
40335724
3947 rs10102306 8 GG GG GG GG GG
GG GG GG GG GG GG GG GG
GG GG GG GG GG GG GG GG
GG GG GG GG GG GG GG GG
GG GG GG GG GG GG GG
13762061
3948 rs10102334 8 AG AG GG AG AG
AA GG AG AG AG GG GG AG
AG AA AG AG AG AA AA AA
AG GG AG GG AG AG AA AA
AG AG AA AG AG AA GG
139184955
3949 rs10102337 8 AG GG AG GG GG
GG AG AA AG GG GG GG GG
GG AA AG GG AG GG GG GG
AG AA AG AG GG AG AG AG
AG AG GG GG AG AA GG
22648756
3950 rs1010236 22 AA AA AA AA AA
AA AA AA AA AA AA AA AA
AA AA AA AA AA AA AA AA
AA AA AA AA AA AA AA AA
AA AA AA AA AA AA AA
46671988
3951 rs10102377 8 AG AA AG AG AG

	AG	GG	GG	AG	AG	AG	AG	GG
	GG	AG	AG	AG	AG	GG	GG	AG
	AG	GG	AG	AG	AG	GG	AG	GG
	AA	AG	GG	AG	GG	AG	GG	
83762822								
3952	rs1010238		18	GG	GG	GG	GG	GG
	AG	GG	GG	GG	GG	AA	AA	AA
	AG	AG	AA	AG	AA	AG	GG	GG
	AG	AG	GG	AG	AG	GG	AG	AG
	AG	AG	GG	GG	GG	AG	GG	
65722568								
3953	rs1010239		2	GG	GG	AG	AG	AA
	AA	AG	GG	GG	GG	GG	GG	GG
	GG	GG	AA	AG	AG	AG	AG	AA
	AG	AA	AG	AG	AG	AG	AG	AG
	AG	AG	GG	AG	GG	GG	GG	
86451502								
3954	rs10102518		8	GG	GG	GG	GG	GG
	GG	GG	GG	GG	GG	GG	GG	GG
	GG	GG	AA	AG	AG	AG	GG	AG
	GG	GG	GG	GG	AG	GG	AG	AG
	AG	GG	GG	GG	GG	GG	GG	
26866413								
3955	rs10102558		8	AG	GG	AG	GG	AG
	AG	AG	AA	AA	AG	GG	GG	GG
	GG	AG	AG	AG	AA	AA	AA	AG
	AG	AG	GG	GG	AA	AG	AG	AG
	AA	GG	AG	AG	AA	AG	AA	
77059673								
3956	rs10102598		8	AG	AG	GG	GG	AG
	GG	AG	GG	GG	GG	AA	GG	AG
	AG	AG	AG	AG	GG	AG	AG	AA
	AG	AA	AA	AG	GG	AA	AG	AG
	AA	GG	AG	AG	GG	AG	AG	
48089751								
3957	rs10102602		8	AG	AA	AA	GG	AG
	AG	AG	AA	AA	AA	AG	AG	GG
	GG	AG	AA	AA	AA	GG	AG	AG
	AA	AG	AA	AG	AG	AG	AG	AA
	AG	AA	AA	AA	AA	AA	AA	
134012038								
3958	rs10102621		8	GG	GG	GG	GG	AG
	AG	GG	AG	AG	AG	GG	GG	GG
	GG	GG	GG	AG	AG	GG	AG	AG
	AG	GG	AG	GG	AG	AG	AG	AG
	GG	AG	GG	AG	GG	AG	GG	
10140875								
3959	rs10102673		8	AA	AC	AA	AC	AC
	AC	AA	AA	AA	AA	AC	AC	CC
	CC	AC	AA	AA	AA	AC	AA	AA

AA AA AA AA AA AC AC AC
AA AA AA AA AA AA AA
92398290
3960 rs10102694 8 AA AA AA AG AG
AG AA AA AA AA AA AA AA
AA AA AA AA AA AA AA AA
AA AA AA AA AA AA AA AA
AA AA AA AA AA AA AA
60338539
3961 rs10102703 8 GG AG GG GG GG
GG AG GG GG GG GG GG GG
GG GG AG AG GG AG GG AG
GG GG GG GG GG GG GG GG
GG AG GG AG AG GG AG
9206425
3962 rs10102749 8 AG AG AG AG AA
AG AG GG GG GG AG AG AA
AA AG AG AG GG AA AA AG
AA AG AG AG AG AA AG AA
GG AA GG AG AG AG AG
87407333
3963 rs10102753 8 AA AG AA AG AG
AG AG AG AG GG AG AG AG
AG GG GG GG GG AG GG AG
AG AG AG AG AA AG AG AG
AG AA AG AG AG AG GG
27213804
3964 rs10102757 8 GG GG GG AG AA
AG AG GG GG AG AA AA AG
AG AG GG GG GG GG GG GG
AA AA AG AG AG AG AG AG
GG AG AG AA AG AG AA
138650367
3965 rs10102812 8 AA AA AA AA AA
AA AA AA AA AA AA AA AA
AA AA AA AA AA AA AA AA
AA AA AA AA AA AA AA AA
AA AA AA AA AA AA AA
113552776
3966 rs1010282 22 AG AA AG GG GG
GG GG GG GG GG GG GG GG
GG GG GG GG GG GG GG GG
AG GG GG AG AG GG GG GG
AG AG AG AG GG GG GG
50474943
3967 rs10102851 8 AG GG AG GG AG
AG AG AA AA AA AG AG AG
AG AG AA AA AA AA AA AA
AA AA AA AA AA AG AA AG
AA AA AA AA AG AA AG

6374065
3968 rs10102855
8 -- -- -- -- -- -- -- --
-- -- -- -- -- -- -- --
-- -- -- -- -- -- -- --
-- -- -- -- -- -- -- --
-- -- -- -- 114676890
3969 rs10102884 8 CC CC CC CC CC
CC CC CC CC CC CC CC CC
AC AC CC CC CC CC CC CC
CC CC CC CC CC CC CC CC
AC AC CC AC CC CC CC
114207044
3970 rs1010294 1 AA AG AG AA AG
AG AG AG AA AG GG GG GG
GG GG AG AG AA AA AG AA
AA AG AG AG AA AA AA AA
AA AG AG AG AA AA AA
15708540
3971 rs10102944 8 AG AG AA AA AA
AA AG GG AG GG AG AG GG
GG GG GG AG AG AG AG AG
GG GG AG GG GG AG AG AG
AG AG AG AG AA AA AA
143384314
3972 rs10102998 8 AC CC AC AA AC
AC AC AA AA AA CC CC CC
CC CC CC AC CC AC AC CC
AC AC AC AC AC AC AC CC
CC CC CC CC CC CC CC
83007997
3973 rs10103003 8 GG GG GG AG GG
GG AG AG AA AA AG AG GG
GG GG AG AA AG AG AA AG
GG GG GG GG GG AG GG GG
GG AG GG AG AA AA AA
118853829
3974 rs1010303 7 AA AA AA AG AG
AG AG AA AG GG GG GG GG
AA AG AG AG AA AA AA AG
AG GG AG GG AG AG GG GG
AG GG AG AG AG AG AG
2412032
3975 rs1010304 20 AA AA AA AA AA
AA AA AA AA AA AA AA AA
AA AA AA AA AA AA AA AA
AA AA AA AA AG AA AA AA
AA AG AG AA AA AA AA
40101647
3976 rs10103058 8 AA AA AG AA AG

AA AG AG AA AA AA AA AA
AG AG AG AA AG AA AG AG
GG AG GG AG AA AG AA AA
AG AG AG AG AA AA AA
120903070
3977 rs1010308 11 AA AA AG GG GG
GG GG GG AG AG AA AA AG
AG AG AG GG GG AG AG AG
AG GG AA GG AG AG GG GG
AG AA AG AG AG AG AG
87307553
3978 rs1010310 20 CC CC AC CC AC
CC AC AA AA AA CC CC CC
CC CC AC AC AC CC AC AC
AC AC AC AC AA CC AC AC
CC AC AC AC AC AA CC
44835044
3979 rs10103102 8 AA AA AA AA AA
AA AA AA AA AA AA AA AA
AA AA GG AG AG AA AA AA
AA AA AA AA AG AA AG AA
AG AA AA AA AG AA AG
13565045
3980 rs10103105 8 AA AA AA AA AA
AA AA AA AA AA AA AA AA
AA AA AA AA AA AA AA AA
AA AA AA AA AA AA AA AA
AA AA AA AA AA AA AA
91549288
3981 rs10103170 8 AA AG AA AG AA
AG AA AG AA AA GG AG AG
AG AA AG GG AG AG GG GG
AG AG AG AG AG AA AA AG
AG AG GG AG AG GG AG
92909705
3982 rs10103186 8 CC CC CC CC AC
AC CC AC CC CC CC CC CC
CC CC CC CC CC CC CC CC
CC CC CC CC CC AC CC CC
CC CC CC CC CC CC CC
93892987
3983 rs10103200 8 AA AA AA AA AA
AA AA AA AA AA AA AA AA
AA AA AA AA AA AA AA AA
AA AA AA AA AA AA AA AA
AA AA AA AA AA AA AA
141868388
3984 rs10103250 8 AA AA AA AA AA
AA AA AA AA AA AG AA AA
AA AA AA AA AA AA AA AA

AG AG AG GG AA AA AA AA
AA AA AG AA AA AA AA
41951881
3985 rs10103251 8 GG GG GG GG GG
GG GG GG GG GG GG GG GG
GG GG AG GG AG GG GG GG
GG AG AG GG GG GG GG GG
GG GG AG GG GG GG AG
70580284
3986 rs10103267 8 AA AA AA AA AG
AG AA AA AA AA AA AA AA
AA AA AG AG AA AG AA AG
AA AA AA AA AG AA AA AG
AA AG AA AG AG AG AA
94296841
3987 rs10103273 8 GG GG GG GG GG
GG GG GG GG GG GG GG GG
GG GG GG GG GG GG GG GG
GG GG GG GG GG GG GG GG
GG GG GG GG GG GG GG
146049225
3988 rs10103353 8 GG GG GG GG GG
AG GG GG GG GG AA AA AA
AA AG AG AG AG AG AA AG
GG AA AG AG AA AA AA AA
GG AG GG GG GG AG GG
82849452
3989 rs10103355 8 GG GG GG GG GG
GG GG GG GG GG GG GG GG
GG GG GG GG GG GG GG AG
GG GG GG GG GG GG GG GG
GG GG GG GG GG GG GG
17918934
3990 rs10103387 8 AG AA AG AG GG
AG AG AG AG GG GG GG AG
GG GG GG AG GG AG AG AG
GG GG GG GG GG GG GG GG
AA AG GG GG AG AG AG
126828417
3991 rs10103391 8 AA AA AA AA AA
AA AA AA AA AA AA AA AA
AA AA AA AA AA AA AA AA
AA AA AA AA AA AA AA AA
AA AA AA AA AA AA AA
132521299
3992 rs10103410 8 AA AA AG AG AG
AA GG AA AA AA GG AG AG
AG AG AG AG GG AG AG AG
AA AA AA AA AG GG GG AG
GG AG AG AG AG AA GG

113040590								
3993	rs1010344		7	AG	AG	GG	GG	AG
	GG	AG	GG	GG	AG	GG	GG	GG
	GG	AG	AG	GG	GG	AG	AG	GG
	GG	GG	GG	GG	GG	AG	AG	AG
	GG	AG	AG	AG	GG	GG	GG	
81908393								
3994	rs10103478		8	GG	GG	GG	GG	GG
	GG	GG	GG	GG	GG	GG	GG	GG
	GG	GG	GG	GG	GG	GG	GG	GG
	GG	GG	GG	GG	GG	GG	GG	GG
	GG	GG	GG	GG	GG	GG	GG	
144048976								
3995	rs10103493		8	AA	AA	AA	CC	AC
	AC	CC	AC	AC	AC	AA	AA	AA
	AA	AA	AA	AC	AA	AA	AA	AA
	AC	AC	AC	AC	AA	AA	AA	AA
	AA	AA	AA	AA	AC	AA	AC	
97352536								
3996	rs10103498		8	GG	AG	GG	GG	GG
	GG	GG	GG	GG	GG	AG	GG	AG
	GG	GG	GG	GG	GG	AG	GG	GG
	GG	AG	GG	AG	GG	AG	GG	AG
	AG	AG	AA	AA	AA	AA	AG	
4189879								
3997	rs10103580		8	AA	AA	AA	AG	AG
	AG	AA	AA	AA	AA	AA	AA	AA
	AA	AA	AG	AA	AG	AA	AA	AA
	AA	AG	AG	AA	AA	AA	AA	AA
	AA	AA	AG	AA	AA	AA	AG	
70583728								
3998	rs1010360		18	AC	AA	AA	AA	AC
	AA	AC	AA	AA	AA	AC	AA	AA
	AA	AA	AA	AA	AA	AA	AC	AC
	AC	AC	AC	AC	AA	AA	AA	AA
	AA	AA	AA	AA	AC	AC	AC	
50928441								
3999	rs10103620		8	GG	GG	GG	GG	GG
	GG	GG	GG	GG	GG	GG	GG	GG
	GG	GG	GG	GG	GG	GG	GG	GG
	GG	GG	GG	GG	GG	GG	GG	GG
	GG	GG	GG	GG	GG	GG	GG	
49611423								
4000	rs10103634		8	GG	GG	GG	AG	AG
	AG	GG	AG	GG	GG	GG	AG	GG
	GG	GG	AG	GG	GG	GG	AG	GG
	GG	AG	AG	GG	AG	AG	GG	AG
	GG	GG	AG	AG	GG	GG	GG	
19890612								
4001	rs10103656		8	GG	AG	GG	AG	AG

	AG	GG	AA	AA	AG	GG	AA	AG
	AG	AG	AG	AA	AG	AA	AA	AA
	AA	AG	AG	AA	AG	GG	GG	AG
	AA	AG	AG	AA	AG	AG	AG	
21281008								
4002	rs10103661		8	AA	AA	AA	AA	AA
	AA	AA	GG	AG	AA	AA	AA	AA
	AA	AA	AA	AA	AA	AG	AA	AA
	AA	AA	AA	AA	GG	AA	AG	AG
	AA	AA	GG	AG	AG	AA	AG	
83936116								
4003	rs1010367		7	CC	CC	CC	CC	CC
	CC	CC	CC	CC	CC	CC	CC	CC
	CC	CC	CC	CC	CC	CC	CC	CC
	CC	CC	CC	CC	CC	CC	CC	CC
	CC	CC	CC	CC	CC	CC	CC	
7230043								
4004	rs10103715		8	AG	AA	AA	GG	GG
	GG	GG	AA	AA	AA	AG	GG	AG
	GG	GG	AG	AG	AG	AA	AG	AA
	GG	AG	GG	AG	AA	GG	AG	AG
	GG	GG	GG	GG	AG	AA	GG	
108544206								
4005	rs10103740		8	AA	AA	AA	AA	AA
	AA	AA	AA	AA	AA	AA	AA	AA
	AA	AA	AA	AA	AA	AA	AA	AA
	AA	AA	AA	AA	AA	AA	AA	AA
	AA	AA	AA	AA	AA	AA	AA	
94398475								
4006	rs10103750		8	CC	AC	AA	AC	AC
	AC	AA	AA	AA	AA	AA	AA	AA
	AA	AA	AA	AA	AC	AA	AA	AA
	AC	AA	AA	AC	AA	AA	AA	AA
	AA	AA	AA	AA	AC	AC	AA	
32596998								
4007	rs10103797		8	AC	CC	CC	AC	AC
	CC	AC	CC	CC	CC	CC	CC	CC
	CC	AC	CC	CC	CC	CC	AC	AC
	CC	CC	CC	CC	CC	AC	CC	CC
	CC	AC	AC	AA	CC	CC	CC	
138006031								
4008	rs10103803		8	AA	AA	AA	AA	AA
	AA	AA	AA	AA	AA	AA	AA	AA
	AC	AC	AA	AA	AA	AA	AA	AA
	AA	AC	AA	AC	AA	AA	AA	AA
	AA	AA	AA	AA	AA	AC	AA	
142904797								
4009	rs10103826		8	GG	GG	GG	AG	GG
	AG	GG	GG	GG	GG	GG	GG	GG
	GG	GG	AA	AA	AA	AG	GG	AG

GG GG GG GG GG AG AG AG
GG GG GG GG GG GG GG
135496060
4010 rs10103835 8 AA AA AA AA AA
AA AA AA AA AC AA AA AA
AA AA AC AC AC AA AA AA
AA AA AA AA AC AC AA AC
AA AA AA AA AA AA AA
132338453
4011 rs10103838 8 AA AA AA AA AA
AA AA AA AA AA AA AA AA
AA AA AA AA AA AA AA AA
AA AA AA AA AA AC AC AC
AA AC AC CC AA AA AA
87756048
4012 rs1010386 1 AG AG AG AG AG
AG AG AG AG AG AA AA AA
AA AA AG GG AG AG AG AG
AA AA AA AA AG AG AG AG
GG AG AG AA AG AG AA
110679596
4013 rs10103877 8 AA AA AA AA AA
AA AA AA AG AG AA AA AA
AA AA AA AA AA AA AA AG
AA AA AA AA AA AA AA AA
AA AG AA AG AA AA AA
87826561
4014 rs10103886 8 AG AA AG GG AG
AG GG AG AG AG GG GG GG
GG AG AG AG GG AG AG AA
GG AG GG AG AG GG GG AG
AG AA GG AG AG AA GG
120727398
4015 rs1010389 15 AG AG GG GG GG
GG GG GG GG GG AG GG GG
GG GG GG GG GG GG GG GG
GG GG GG GG GG GG GG GG
GG GG GG GG AG GG AG
78534110
4016 rs10103893 8 AA AA AA AA AA
AA AA AA AA AA AA AA AA
AA AA AA AA AA AA AA AA
AA AA AA AA AA AA AA AA
AA AA AA AA AA AA AA
49727342
4017 rs10103953 8 CC CC CC AA AC
AC AA AC CC CC AA AA AA
AC AC AC AA AC CC AC AC
AC AC AC AC CC CC CC CC
AC AC AA AC CC AC CC

8834240							
4018	rs10103970	8	AA	AA	AA	AA	AA
	AA	AA	AA	AA	AA	AA	AA
	AA	AA	AA	AA	AA	AA	AA
	AA	AA	AA	AA	AA	AA	AA
	AA	AA	AA	AA	AA	AA	AA
9428673							
4019	rs10104018	8	GG	GG	GG	GG	GG
	GG	GG	GG	GG	GG	GG	GG
	GG	GG	GG	GG	GG	GG	GG
	GG	GG	GG	GG	GG	GG	GG
	GG	GG	GG	GG	GG	GG	GG
92725917							
4020	rs10104069	8	AA	AA	AG	AA	AA
	AA	AA	AG	AG	AG	AG	AG
	AA	AA	AG	AG	AA	AG	AA
	AA	AA	AA	AA	AA	AG	AG
	AA	AA	AA	AA	AA	AA	AA
121861465							
4021	rs10104103	8	GG	GG	GG	GG	GG
	GG	GG	GG	GG	GG	GG	GG
	GG	GG	GG	GG	GG	GG	GG
	GG	GG	GG	GG	GG	GG	GG
	GG	GG	GG	GG	GG	GG	GG
49811626							
4022	rs1010412	3	AG	AG	AA	AG	AG
	AA	AG	AA	AA	AA	AA	AG
	AG	AG	AG	AA	AG	AG	AG
	AG	AG	AG	AG	AG	AG	GG
	AA	AA	AA	AA	AA	AG	AA
13006661							
4023	rs10104122	8	AG	AG	AG	AG	AG
	GG	AG	AG	AA	AG	GG	GG
	GG	AG	AG	GG	AG	GG	GG
	AG	AG	AG	AG	AG	AG	GG
	AG	GG	AA	AG	AG	GG	AG
65940453							
4024	rs10104139	8	AA	AA	AC	CC	CC
	CC	CC	CC	CC	CC	CC	CC
	CC	AC	CC	CC	CC	CC	CC
	CC	CC	CC	CC	CC	CC	CC
	AC	AC	CC	AC	CC	CC	CC
136663746							
4025	rs10104140	8	AA	AA	AA	AA	AA
	AA	AA	AA	AA	AA	AA	AA
	AA	AA	AA	AA	AA	AA	AA
	AG	AA	AA	AG	AA	AA	AA
	AA	AA	AA	AA	AA	AA	AG
52138162							
4026	rs10104215	8	AA	AG	AA	GG	GG

	GG	GG	AG	AG	AG	AG	AG	AG
	GG	GG	AA	AG	AG	GG	AG	GG
	GG	GG	GG	GG	GG	GG	GG	GG
	GG	GG	AA	AG	GG	GG	AG	
4863084								
4027	rs10104270		8	AG	AG	AG	AA	AG
	AA	AG	AG	AG	AG	AG	GG	AG
	AG	AG	GG	GG	GG	AG	GG	AA
	GG	AG	GG	AG	AA	AG	AG	AG
	AA	AG	GG	AG	AA	AA	AG	
110866734								
4028	rs10104319		8	GG	AG	GG	AG	AG
	AG	AG	GG	GG	GG	GG	GG	GG
	GG	AG	AG	AG	AG	GG	GG	GG
	AG	AG	GG	AG	AA	AG	AA	AA
	AG	AG	AG	GG	GG	AG	GG	
26326808								
4029	rs10104327		8	GG	GG	GG	AG	AG
	AA	AG	GG	GG	AG	AA	AA	AA
	AA	AA	AG	AG	AG	GG	GG	GG
	AA	AG	AG	GG	AG	GG	GG	GG
	GG	AA	AG	AA	AG	AG	AA	
138711327								
4030	rs10104356		8	CC	CC	CC	CC	CC
	CC	CC	CC	CC	CC	CC	CC	CC
	CC	CC	CC	CC	CC	CC	CC	CC
	CC	CC	CC	CC	CC	CC	CC	CC
	CC	CC	CC	CC	CC	CC	CC	
114380426								
4031	rs10104421		8	GG	GG	GG	AG	AA
	AG	AA	AG	AG	AG	GG	GG	GG
	GG	GG	GG	GG	GG	GG	GG	AG
	GG	GG	GG	GG	GG	AG	AG	AG
	GG	AG	AG	GG	AG	GG	AG	
124702195								
4032	rs10104514		8	AG	GG	GG	GG	AG
	GG	AG	AG	AA	AA	AA	AG	AA
	AA	AA	AG	AG	AG	AA	AA	AG
	AG	GG	GG	AG	AG	AG	AG	AG
	GG	GG	GG	GG	GG	GG	AG	
52070410								
4033	rs10104525		8	AG	AG	AG	AG	AA
	AA	AG	AG	AG	AG	AA	AG	AA
	AA	AG	AG	GG	AG	AG	AG	AG
	AG	GG	GG	GG	AG	AG	GG	GG
	AG	GG	GG	GG	AA	AG	AG	
61626087								
4034	rs10104541		8	GG	GG	GG	AG	AG
	GG	AA	GG	AG	AG	AG	GG	AG
	GG	GG	GG	GG	GG	AG	AG	GG

GG GG GG GG AG AA AG AG
AG GG GG GG GG GG GG
8779418
4035 rs10104571 8 GG GG GG GG GG
GG GG AG GG GG GG GG GG
GG GG GG AG AG GG AG GG
AA AG AG AA GG GG GG GG
GG GG GG GG GG GG GG
10896697
4036 rs10104574 8 AC AC AA CC CC
CC CC CC CC CC AA AC AC
AC AC AC AC AC AC AC CC
AC CC CC CC AC CC CC AC
CC CC CC CC CC CC AC
106696670
4037 rs10104637 8 AA AG AG AA AA
AA AA AA AA AA AA AA AA
AA AA AA AA AA AA AA AA
AA AA AA AA AA AA AA AA
AA AA AA AA AG AG AA
95393925
4038 rs10104706 8 AA AA AA AA AA
AA AA AA AA AA AA AA AA
AA AA AA AA AA AA AA AA
AA AA AA AA AA AA AA AA
AA AG AA AG AA AA AA
89406894
4039 rs10104711 8 GG GG GG GG GG
GG GG AG GG GG GG GG GG
GG GG GG GG GG GG GG GG
GG GG GG GG GG GG GG GG
AG AG AG AG AG GG AG
39858026
4040 rs10104739 8 AG AA AG AA AA
AG AA AA AA AA AA AA AA
AA AA AG AA GG AA AA AA
AG AG AA AG AG GG GG GG
AA AA AG AG AG AG AG
28001680
4041 rs10104790 8 GG GG GG GG GG
GG GG GG GG GG GG GG GG
GG GG GG GG GG GG GG GG
GG GG GG GG GG GG GG GG
GG GG GG GG GG GG GG
38224286
4042 rs1010482 5 CC CC CC CC CC
CC CC CC CC CC CC CC CC
CC CC CC CC CC CC AC CC
CC CC CC CC AC AC CC AC
CC CC CC CC CC AC CC

172223045
4043 rs10104846 8 TT TT TT TT TT
TT TT TT TT TT TT TT TT
TT TT TT TT TT TT TT TT
TT TT TT TT TT TT TT TT
TT TT TT TT TT TT TT
55370971
4044 rs10104895 8 GG GG GG GG GG
GG GG GG GG GG GG GG GG
GG GG GG GG GG GG GG GG
GG GG GG GG GG AG GG GG
GG AG GG GG GG GG GG
61876044
4045 rs10104906 8 AG AA AG AA AA
AA AG AG AG GG AA AG AA
AA AA AG AG AA AA AG AG
AA AA AA AA GG AA AG AG
AA AA AG AA AA AG AA
2391471
4046 rs10104910 8 GG GG GG GG GG
GG GG GG GG GG GG GG GG
GG GG GG GG GG GG GG GG
GG GG GG GG GG GG GG GG
GG GG GG GG AG AG GG
4217893
4047 rs10104950 8 AG AG AG GG GG
GG GG AA AG AG AG GG AG
GG GG GG AG AG GG GG GG
GG GG GG GG AG GG AG AG
GG AG GG AG AG GG AG
8557220
4048 rs10104973 8 AA AG GG AA AA
AA AA AA AA AA AA AA AA
AG AG AA AA AG AG AG AA
AG AG AG AG AA AA AA AA
AA AA AA AA AG AA AG
121104385
4049 rs10104977 8 AG GG GG AG AG
AG AG AG AG AG GG GG GG
GG GG AA AG AA AG AG AA
AG AG AG AG AG AA AG AA
GG AG GG AG AG AG AA
119723897
4050 rs10104993 8 AG GG AG GG GG
AG GG GG GG AG GG AG AG
AG AA AA AA AG AG GG GG
GG AA AA AG AA GG AG AG
AG AA GG AG AG GG AG
69346829
4051 rs10105038 8 AA AA AA AA AA

	AA	AA	AA	AA	AA	AA	AA	AA
	AA	AA	AG	AG	AG	AA	AA	AA
	AA	AA	AA	AA	AA	AA	AA	AA
	AA	AA	AA	AA	AA	AA	AA	
72479954								
4052	rs10105193		8	GG	GG	AG	AG	GG
	AG	GG	AG	AG	AG	GG	GG	GG
	AG	AG	AG	GG	AG	GG	GG	GG
	GG	GG	GG	GG	GG	GG	GG	GG
	AA	GG	GG	GG	AG	GG	AG	
113565333								
4053	rs10105219		8	AG	AG	AG	AG	AA
	AA	AG	GG	AG	AG	AG	AG	GG
	GG	AG	AA	AG	AA	GG	GG	AG
	AA	AG	AG	AG	AG	GG	AG	GG
	AG	AA	AG	AA	GG	GG	AG	
83857175								
4054	rs10105238		8	GG	GG	GG	GG	GG
	GG	GG	GG	GG	GG	AA	AG	AG
	AG	GG	AG	AG	GG	GG	GG	GG
	GG	GG	GG	GG	GG	GG	GG	GG
	GG	GG	AG	GG	GG	GG	GG	
97252713								
4055	rs10105278		8	AC	AC	AC	AC	AC
	AC	CC	CC	CC	AC	AA	AC	AA
	AA	CC	AA	AC	AC	CC	AC	CC
	CC	AC	AA	CC	AC	AC	CC	CC
	AA	AC	AC	CC	CC	CC	CC	
26600068								
4056	rs10105297		8	GG	GG	GG	GG	GG
	GG	GG	GG	GG	GG	AG	GG	AG
	GG	GG	GG	GG	GG	GG	GG	GG
	GG	GG	GG	GG	GG	GG	GG	GG
	GG	GG	GG	GG	AG	GG	AG	
121937245								
4057	rs1010532		7	AA	AG	AG	GG	AA
	AG	AG	AG	GG	AG	AA	AG	AG
	AG	AG	GG	GG	GG	GG	AA	GG
	GG	GG	AG	AG	GG	AG	GG	GG
	AG	AG	AG	AA	GG	GG	GG	
51851265								
4058	rs10105377		8	AA	AA	AA	GG	AG
	AG	GG	GG	AG	AG	GG	AG	GG
	AG	AG	AG	GG	AG	AG	GG	AG
	GG	AG	AG	GG	AA	GG	AG	AG
	GG	AA	AG	AA	AG	AG	GG	
10975733								
4059	rs10105423		8	AA	AC	AA	AC	AC
	AA	CC	AC	CC	CC	AC	AC	CC
	CC	CC	AA	AC	AC	AC	AC	AC

AC AC AC AC CC AC CC CC
AC AC CC CC AA AC AA
139353715
4060 rs10105450 8 AG GG AG AG GG
GG AG AA AA AA AA AG AG
GG AG AA AA AG GG AG AG
AA AG AG AG AA GG AG AG
AG AA AG AA AG GG AA
104373506
4061 rs10105465 8 GG AG GG GG GG
GG GG GG GG GG AG AG AG
AG AG GG GG GG AG GG GG
GG AG GG AG GG GG GG GG
GG AG GG AG AG GG AG
4208391
4062 rs1010547 9 AG AA AG AG AG
AA GG GG AG AG AA AG AG
AG AG AG AG GG GG AG GG
GG GG GG AG GG GG GG GG
AG AG AG GG AA AG AG
17599511
4063 rs10105491 8 AA AA AA AA AG
AA AG GG GG GG AG AA AG
AA AA AG AG AG AA AA AG
AA AA AA AA GG AG AG AG
AG AG AA AG GG AG AG
118217271
4064 rs10105493 8 AA AG AG AA AA
AA AA GG GG GG GG AG AG
AA AA AG AA AG GG GG AG
AA AG AA AG AG AA AG AA
AA AA AA AA AG AA GG
113653436
4065 rs10105518 8 AA AA AA AG AG
AA GG AA AA AA AA AA AA
AA AA AA AA AG AG AG AA
AG GG AG GG AG AG GG GG
AG AA AG AG AG AG AA
31403217
4066 rs10105529 8 AA AA AA AA AA
AA AA AA AA AA AA AA AA
AA AA AA AA AA AA AA AA
AA AA AA AA AA AA AA AA
AA AA AA AA AA AA AA
89555659
4067 rs1010553 3 AG AA AG GG GG
GG AG GG AG AG GG AG AG
AG AG AA AG AG AG AA GG
GG AG AG GG AG AG GG AA
AG AA AA AA AG AG AG

52540773								
4068	rs10105543	8	AA	AA	AA	GG	AG	
	AG	GG	GG	AG	AG	AG	AA	AG
	AA	AA	AG	GG	AG	AG	GG	AG
	AG	GG	GG	AG	AG	GG	GG	AG
	GG	AA	GG	AG	AG	AG	GG	
9323515								
4069	rs10105604	8	CC	CC	CC	AC	CC	
	AC	AC	AC	AC	AC	CC	CC	CC
	CC	CC	CC	AC	AC	CC	AC	AC
	AA	AC	AA	CC	AC	CC	AC	AC
	CC	AC	AA	AA	AC	AC	CC	
134986491								
4070	rs1010564	16	CC	CC	CC	CC	CC	
	CC	AC	AC	AA	AA	AC	AA	AC
	AC	CC	AC	AC	CC	AC	AA	CC
	AC	AC	AC	AC	AC	AC	AC	AC
	CC	CC	CC	CC	AC	AC	AA	
64874336								
4071	rs10105642	8	GG	GG	GG	GG	GG	
	GG	GG	GG	GG	GG	GG	GG	GG
	GG	GG	GG	GG	GG	GG	GG	GG
	GG	GG	GG	GG	GG	GG	GG	GG
	GG	GG	GG	GG	GG	GG	GG	
91836322								
4072	rs10105657	8	GG	GG	GG	AG	AG	
	AA	GG	GG	GG	GG	AG	AG	AG
	AG	AG	AA	AG	AG	AG	GG	AG
	GG	GG	GG	GG	AG	GG	AG	AG
	GG	AG	GG	AG	GG	AG	GG	
607251								
4073	rs10105730	8	AG	AG	AA	AG	AA	
	AG	AA	AA	AA	AA	AA	AA	AA
	AA	AA	AA	AA	AG	AG	GG	AA
	AA	AA	AA	AA	AA	AG	AG	AG
	AA	AG	AA	AA	AA	AA	AA	
128251421								
4074	rs1010577	2	GG	AG	AG	AG	AG	
	GG	AG	GG	GG	GG	AG	AG	GG
	AG	AG	GG	GG	GG	GG	GG	GG
	GG	GG	GG	GG	AA	GG	AG	AG
	AG	AA	AG	AA	AG	AG	GG	
167242123								
4075	rs10105795	8	AA	AC	AA	CC	CC	
	CC	CC	AA	AC	AC	AC	AC	CC
	CC	AC	CC	CC	CC	CC	CC	AC
	CC	AA	AA	AC	AC	AC	AC	AC
	AC	AA	CC	AC	CC	CC	AC	
68201031								
4076	rs10105826	8	AG	AG	GG	AG	AA	

	AG	AA	AA	AA	AA	AA	AA	AG
	AA	AA	AA	AA	AA	AA	AG	AA
	AG	AG	AG	AG	AG	AG	AG	GG
	AA	AG	AG	AG	AA	AA	AA	
131477907								
4077	rs10105842		8	GG	GG	GG	GG	GG
	GG	GG	GG	GG	AG	GG	GG	GG
	GG	GG	AA	AG	AG	GG	GG	GG
	GG	GG	GG	GG	GG	AG	AG	AG
	GG	GG	AG	GG	GG	AG	GG	
143671615								
4078	rs10105845							
8	--	--	--	--	--	--	--	--
	--	--	--	--	--	--	--	--
	--	--	--	--	--	--	--	--
	--	--	--	--	--	--	--	--
	--	--	--	--	144192458			
4079	rs10105853		8	AA	AA	AA	AA	AA
	AA	AA	AA	AA	AA	AA	AA	AA
	AA	AA	AA	AA	AA	AA	AA	AA
	AA	AA	AA	AA	AA	AA	AA	AA
	AA	AA	AA	AA	AA	AA	AA	
48318654								
4080	rs1010587		9	CC	CC	CC	AA	AA
	AC	AC	CC	CC	CC	AC	AA	AC
	AC	CC	AC	CC	CC	CC	CC	CC
	CC	AC	CC	AC	AA	CC	AC	AC
	CC	AC	AC	CC	CC	AC	CC	
29218907								
4081	rs10105874		8	AG	AA	AG	AG	GG
	AG	AG	AA	AG	AG	GG	AG	AG
	AG	GG	AG	AG	GG	GG	GG	GG
	AG	AG	AG	AG	GG	GG	GG	GG
	AG	GG	GG	GG	AG	AA	GG	
56396012								
4082	rs1010588		8	AA	AA	AG	AA	AA
	AA	AA	AA	AA	AA	AG	AG	AA
	AA	AA	AA	AA	AA	AA	AA	AA
	AA	AA	AA	AA	AA	AG	AA	AA
	GG	AG	AA	AG	AA	AA	AA	
38546707								
4083	rs1010592		9	AA	AA	AA	AA	AA
	AA	AA	AA	AA	AA	AA	AA	AA
	AA	AA	AA	AA	AA	AA	AA	AA
	AA	AA	AA	AA	AA	AA	AA	AA
	AA	AA	AA	AA	AA	AA	AA	
8766370								
4084	rs10105927		8	AA	AA	AA	AA	AA
	AA	AA	AA	AA	AA	AA	AA	AA
	AA	AA	AA	AA	AA	AA	AA	AA

AA AA AA AA AA AA AA AA
AA AA AA AA AA AA AA
144740379
4085 rs10105978 8 CC CC CC CC CC
CC CC CC CC CC CC CC CC
CC CC CC CC CC CC CC CC
CC CC CC CC CC CC CC CC
CC CC CC CC CC CC CC
5433456
4086 rs10106001 8 AA AG AG AA AG
AG AA AG AA AA GG AG AG
AA AA AG AA AA AG AG AG
AG AG AG AG AA AG AG AG
AA AA GG AG GG GG AG
142703492
4087 rs10106032 8 AA AG AA AG AG
GG AA GG AG AG AA AA AG
AG AA GG GG GG AA AA AA
AG AA AA AG AG AG AG GG
AG AG AG AA AG GG AG
18514100
4088 rs10106082 8 AG AG AG GG GG
AG GG AG GG GG GG GG GG
AG AA GG GG GG GG GG AG
AG GG GG AG GG AG GG GG
AA AG AG AA GG AG GG
49103543
4089 rs10106087 8 AG AA AG GG AG
GG AG AA AA AG AG GG AG
AG AG AG GG AA AG AA AA
AA AG AG AG GG AA AG AG
AA AG GG GG GG AG AG
77896845
4090 rs10106093 8 AG AG AG GG GG
GG GG GG GG GG GG AG GG
GG GG AG GG AG AG GG AG
GG GG GG GG GG AG AG AG
AG GG AG AG GG GG GG
29355430
4091 rs10106155 8 AC AC AC CC CC
AC CC CC AC AC AC AC AC
AC CC AA AA AA AA AC AC
CC AC AC CC AC AA AC AA
AC AC AA AC CC CC CC
20489396
4092 rs10106201 8 CC CC CC CC CC
CC CC CC CC AC CC CC CC
CC AC AC CC CC AC CC AC
CC CC CC CC CC AC AC AC
CC AC CC AC AC CC AC

135809634								
4093	rs10106213	8	AG	AA	AG	AA	AG	
	AG	AG	AG	AG	AG	AG	GG	AG
	GG	GG	GG	AG	AG	GG	GG	GG
	AG	AG	AG	AG	AG	AG	AA	AG
	AG	AA	AG	AG	AA	AG	AA	
5676046								
4094	rs1010626	5	AG	GG	AG	AG	AG	
	GG	AA	AG	GG	AG	AG	AG	AA
	AG	AG	AG	AG	AA	AG	AA	AA
	AG	AG	AA	AG	AG	AG	AA	AA
	AG	AG	AG	AG	AA	AA	AA	
1490594								
4095	rs10106273	8	GG	GG	GG	GG	GG	
	GG	GG	GG	GG	GG	GG	GG	GG
	GG	GG	GG	GG	GG	GG	AG	GG
	GG	GG	GG	GG	AG	GG	AG	GG
	GG	AG	AG	AG	GG	GG	GG	
15929038								
4096	rs10106310	8	AA	AG	AA	AG	AG	
	GG	AA	AG	GG	GG	AG	AA	AG
	AG	AG	AA	AG	AG	AG	AG	AA
	AA	AA	AA	AA	AG	AG	GG	AG
	AG	AA	AA	AA	AA	AA	AG	
91175874								
4097	rs10106329	8	AG	AG	AA	AG	AG	
	AG	AA	GG	AG	AA	AG	AA	AG
	AA	AG	AG	GG	AA	AA	AA	AA
	AA	AA	AA	AA	AA	AG	AA	AA
	AG	AA	AA	AA	AG	AG	AA	
32214317								
4098	rs10106346	8	AA	AA	AA	AG	AA	
	AA	AG	AA	AA	AA	AA	AA	AA
	AG	AG	AA	AA	AA	AA	AA	AA
	AA	AA	AA	AA	AG	AA	AA	AG
	AA	AA	AG	AG	AG	AG	AA	
102387329								
4099	rs10106358	8	AC	AC	AC	AC	AC	
	AC	CC	AC	AC	AC	AC	CC	AC
	AC	CC	AC	CC	AA	CC	AC	CC
	CC	AC	AC	CC	CC	AA	AC	AC
	AC	CC	AC	AC	AC	AC	AC	
70349040								
4100	rs10106375	8	GG	GG	GG	GG	GG	
	GG	GG	GG	GG	GG	AG	AG	AA
	AG	GG	GG	GG	GG	AG	AG	AA
	GG	GG	GG	GG	GG	AG	GG	GG
	GG	GG	GG	GG	GG	GG	AG	
128545370								
4101	rs10106386	8	GG	AG	AG	GG	AG	

GG AG AG AG AG AA AG AG
AG AA AG AG AA AG AG AA
AA AA AA AA AA AG AA AA
AG AG AA AA AG AG GG
106753057
4102 rs10106514 8 AA AA AA AA AA
AA AA AA AA AA AA AA AA
AA AA AA AA AA AA AA AA
AA AA AA AA AA AA AA AA
AA AA AA AA AA AA AA
141821803
4103 rs10106557 8 AC CC CC AA AC
AC AC AC AC CC CC AA AC
AC AC AC AC AC AC AC CC
AA AA AA AA AC AC CC CC
AC AC CC CC AC AC AC
142696638
4104 rs10106570 8 AG AA AG GG AG
AG AG GG GG GG AG AG AA
AA AA GG GG GG AG GG GG
GG AG AG AG AG AG AA AG
AG AG AA AG AG GG AG
92010477
4105 rs1010658 2 AG AG AG AG AG
AG AG AG GG AG GG GG GG
AA AG AG AA GG GG GG GG
AG GG AG AG GG AG AG GG
AG AA GG AG GG GG AG
25595194
4106 rs10106674 8 AG GG AG GG AG
AG AG AA AA AG AA AG AA
AG AG AA AG AG AA AA AA
AA GG AG AG AA AA AA AA
AA AA AG AA AA AA AA
3361471
4107 rs10106732 8 AA AA AA AA AG
AG AG AA AA AA AA AG AG
AG GG AA AA AA AA AA AA
AA AG AG AG AG AA AG AA
AG AG AA AA AG AA AG
92049496
4108 rs10106748 8 AG AA AG AA AG
AG AA AA AA AG AG AA AG
AA AA AG AG AA AG AA GG
AA AG AA AG AA AA AA AA
AG AA AA AA AA AG AG
2576054
4109 rs10106787 8 GG GG GG AG AG
GG AG AG AG GG GG GG GG
AG AG GG GG GG AA AA AG

	AG	GG	GG	AG	AG	GG	AG	AG
	GG	GG	AG	AG	AG	AG	GG	
1430681								
4110	rs1010680		6	AG	GG	AG	AG	GG
	AG	GG	GG	GG	GG	GG	GG	GG
	GG	GG	GG	GG	GG	GG	GG	GG
	GG	GG	GG	GG	GG	AG	GG	GG
	GG	GG	GG	GG	AG	GG	AG	
77164823								
4111	rs10106813		8	AG	AG	GG	GG	GG
	GG	GG	GG	GG	GG	GG	GG	GG
	GG	GG	GG	GG	GG	GG	GG	GG
	GG	GG	GG	GG	GG	GG	GG	GG
	GG	GG	GG	GG	GG	GG	GG	
35934498								
4112	rs10106829		8	GG	GG	GG	GG	GG
	GG	GG	GG	GG	GG	GG	GG	GG
	GG	GG	GG	GG	GG	AA	GG	AG
	GG	GG	GG	GG	GG	GG	GG	GG
	GG	GG	AG	AG	AG	GG	AG	
9184628								
4113	rs10106855		8	GG	AG	GG	GG	GG
	GG	GG	GG	GG	GG	GG	AG	AG
	AG	AG	AA	AA	AG	GG	AG	GG
	GG	GG	GG	GG	AG	AA	AG	AA
	AG	GG	GG	GG	AG	AG	AA	
87340319								
4114	rs10106907		8	AG	AA	AG	GG	AA
	AG	AG	GG	GG	GG	GG	GG	GG
	GG	GG	AA	AA	AA	GG	GG	GG
	GG	GG	GG	GG	AG	AG	GG	AG
	GG	AA	GG	AG	GG	GG	GG	
96786148								
4115	rs1010692		1	AC	AC	AC	CC	CC
	CC	CC	AA	AC	AA	AA	AC	AA
	AA	AC	AA	AA	AA	AA	AA	AA
	CC	AC	AC	AC	AC	AA	AC	AA
	AC	AA	AA	AA	AC	AA	AC	
209220739								
4116	rs10106920		8	GG	GG	GG	GG	GG
	GG	GG	GG	GG	GG	GG	GG	GG
	GG	GG	GG	GG	GG	GG	GG	GG
	GG	GG	GG	GG	GG	GG	GG	GG
	GG	GG	AG	GG	GG	GG	GG	
4379269								
4117	rs10106924							
8	--	--	--	--	--	--	--	--
	--	--	--	--	--	--	--	--
	--	--	--	--	--	--	--	--
	--	--	--	--	--	--	--	--

	--	--	--	--	135545718		
4118	rs10106960	8	GG	AG	GG	AA	AA
	AA	AA	AA	AA	AA	GG	GG	GG
	GG	GG	GG	GG	GG	AG	AG	AG
	AG	AG	AG	AG	AA	AA	AA	AA
	AG	AG	AA	AG	AG	AA	AG	
3333127								
4119	rs1010698	11	AC	AC	AC	AC	AC
	CC	AA	AC	CC	CC	AA	CC	AC
	AC	AC	AA	AC	AA	CC	AC	AC
	AC	AC	AC	AC	CC	AC	AC	CC
	AC	AC	CC	AC	AC	AC	AC	
102701021								
4120	rs10107002	8	GG	GG	GG	GG	GG
	AG	GG	GG	GG	AG	AA	AG	AG
	AG	GG	GG	AG	GG	GG	GG	GG
	GG	GG	GG	GG	GG	GG	GG	GG
	GG	GG	GG	GG	GG	AG	AG	
80970355								
4121	rs10107014	8	GG	AG	GG	GG	GG
	GG	GG	AA	AG	AG	AG	AG	GG
	GG	GG	GG	GG	AG	GG	GG	GG
	AG	AG	AG	AG	AA	AG	AG	AG
	AG	AG	GG	AG	GG	GG	AG	
88421332								
4122	rs10107030	8	GG	AG	GG	AG	GG
	AG	GG	GG	GG	GG	GG	GG	GG
	GG	GG	AG	AG	AA	AG	GG	AA
	GG	GG	GG	AG	AG	GG	GG	AG
	GG	AG	GG	GG	AG	AA	GG	
130300407								
4123	rs10107034	8	AA	AA	AA	AA	AA
	AA	AA	AA	AA	AA	AA	AC	AA
	AC	AA	AA	AA	AA	AA	CC	AA
	AA	AA	AA	AA	AC	AC	CC	AC
	AC	CC	AA	AC	AA	AC	AA	
15939341								
4124	rs10107128	8	GG	AG	AA	AA	AG
	AA	AG	AA	AG	AG	GG	GG	GG
	AG	AA	AA	AA	AA	AA	AA	AA
	AA	AA	AA	AA	AG	AG	GG	GG
	AA	AG	AA	AA	AA	AA	AA	
52497762								
4125	rs10107214	8	AG	AG	AA	GG	GG
	AG	GG	AG	AA	AA	AG	AG	AA
	AA	AA	GG	GG	GG	AG	AG	AG
	AA	AG	AG	AG	AG	GG	GG	GG
	GG	GG	AG	GG	GG	GG	GG	
31297579								
4126	rs10107257	8	GG	GG	AG	AG	GG

	AG	GG	AG	AG	AG	AG	AG	GG
	AG	AG	AG	AA	AG	GG	AG	GG
	GG	GG	GG	GG	AG	AG	AA	AG
	AG	AG	AG	AG	AG	AG	GG	
121291145								
4127	rs10107260		8	AG	AG	AA	GG	GG
	AG	GG	AA	AG	AG	GG	GG	GG
	GG	GG	GG	AG	AG	GG	GG	GG
	GG	GG	AG	GG	GG	AG	GG	GG
	GG	AG	GG	AG	GG	GG	AG	
146168128								
4128	rs10107280		8	CC	CC	CC	CC	CC
	CC	CC	CC	CC	CC	CC	CC	CC
	CC	CC	CC	CC	CC	AC	AC	AC
	CC	CC	CC	CC	CC	CC	CC	CC
	CC	CC	CC	CC	CC	CC	CC	
51469405								
4129	rs10107281		8	AG	GG	AG	GG	GG
	GG	GG	GG	GG	GG	GG	AG	GG
	GG	GG	GG	GG	GG	GG	GG	GG
	GG	GG	GG	GG	GG	GG	GG	GG
	GG	GG	GG	GG	AG	AG	GG	
13691166								
4130	rs10107285		8	AC	AC	AC	AA	AA
	AA	AA	AA	AA	AA	AA	AA	AA
	AA	AA	AA	AA	AA	AC	AA	AA
	AC	AC	AC	AA	AA	AA	AA	AA
	AC	AA	AA	AA	AA	AA	AA	
136319523								
4131	rs10107332		8	CC	CC	CC	CC	CC
	CC	CC	CC	CC	CC	CC	CC	CC
	CC	CC	CC	CC	CC	CC	CC	CC
	CC	CC	CC	CC	CC	AC	AC	AC
	CC	CC	CC	CC	CC	CC	CC	
145111615								
4132	rs10107411		8	AG	AA	AA	AG	AA
	AA	AG	AG	AG	AG	AG	AG	AA
	AA	AA	AG	AA	GG	AG	AG	GG
	AG	AA	AG	AA	AA	AA	AA	AA
	AA	AA	AA	AA	AG	AG	AG	
114509172								
4133	rs1010743		4	CC	AC	CC	CC	AC
	CC	AC	AC	AC	AC	AC	AC	AC
	AC	CC	AC	CC	AC	AA	AA	AC
	AA	AC	AC	AA	AC	CC	CC	CC
	CC	AC	AC	CC	AC	AA	CC	
19333182								
4134	rs10107434		8	AA	AG	AA	AG	AA
	AG	AA	AG	AG	GG	AG	GG	AG
	AG	AG	AG	AA	AA	AG	AG	AG

	AG	AG	AA	AA	AG	AG	AG	AG
	AG	AA	AG	AA	GG	GG	AG	
141672565								
4135	rs10107472		8	GG	AG	GG	GG	GG
	GG	GG	GG	GG	GG	AG	GG	AG
	GG	GG	GG	GG	GG	AG	GG	GG
	GG	AG	GG	AG	GG	AG	GG	AG
	GG	AG	AG	AA	AA	AG	AG	
4190874								
4136	rs10107483		8	AG	AA	AA	GG	AG
	GG	AG	GG	GG	GG	GG	GG	GG
	GG	GG	AG	GG	AA	GG	GG	GG
	AG	GG	GG	GG	AG	GG	AG	GG
	AG	AA	GG	AG	GG	GG	GG	
102745291								
4137	rs1010749		3	AA	AA	AA	AA	AA
	AA	AA	AA	AA	AA	AA	AA	AA
	AA	AA	AA	AA	AA	AA	AA	AA
	AA	AA	AA	AA	AA	AA	AA	AA
	AA	AG	AA	AA	AA	AA	AG	
68342411								
4138	rs10107525		8	GG	GG	GG	GG	GG
	GG	GG	AG	AG	AG	AG	GG	AG
	AG	AG	GG	AG	GG	GG	GG	GG
	GG	GG	GG	GG	GG	GG	GG	GG
	AG	GG	AG	GG	AA	AG	AG	
93438176								
4139	rs10107557		8	AA	AA	AA	AA	AA
	AA	AA	AA	AA	AA	AA	AA	AA
	AA	AA	AA	AA	AG	AA	AA	AG
	AA	AA	AA	AA	AA	AA	AA	AA
	AG	AG	AA	AA	AA	AA	AG	
62380439								
4140	rs1010757		5	CC	CC	CC	AC	AC
	CC	AA	CC	CC	CC	CC	CC	CC
	AC	AC	AC	CC	AC	CC	AC	AC
	CC	CC	CC	CC	AC	CC	AC	CC
	CC	AC	AC	CC	CC	CC	CC	
58076467								
4141	rs10107586		8	AG	GG	GG	AG	AG
	GG	AG	AA	AG	AG	GG	AG	GG
	AG	AG	AG	AA	AA	GG	GG	AG
	AG	AA	AG	AA	AA	AG	AA	AA
	GG	AG	AG	GG	GG	AG	AG	
142647889								
4142	rs10107611		8	CC	CC	CC	AC	CC
	AC	AC	AA	AA	AA	AA	AA	AA
	AA	AA	AA	AC	AC	AA	AA	AA
	AA	CC	AC	AC	AC	AC	AC	AA
	AA	AA	AC	AC	AA	AA	AA	

3950542
4143 rs10107633 8 AG AG AG AA AG
AG AA AG AG GG AA AA AA
AG AG AG AA AG AA AA AA
AG AA AG AA AA AG AA AA
AG AA AG AG AG AG AG
126569784
4144 rs10107657 8 GG AG GG AA AA
AA AA GG AG AG AA AG AG
AG AA AG AG AA AG AG AA
AG AA AA AA AG AA AA AA
AG AG AG GG AA AA AA
56540235
4145 rs10107664 8 AA AA AA AA AA
AA AA AA AA AA AA AA AA
AA AA AG AG AG AA AA AA
AA AA AA AA AA AA AA AA
AA AA AA AA AA AG AA
27030123
4146 rs10107666
8 -- -- -- -- -- -- -- --
-- -- -- -- -- -- -- --
-- -- -- -- -- -- -- --
-- -- -- -- -- -- -- --
-- -- -- -- 14697130
4147 rs1010776 4 AG GG AG AG AG
GG AA AA AG AA GG GG GG
GG GG AG AG GG GG GG AG
GG GG GG GG GG GG GG GG
AG GG GG GG GG AG GG
4942150
4148 rs1010777 9 AG AG AG AA GG
AG AG AA AA AG AA GG AG
AG AA AG -- -- AG -- --
AG AG AG AG AG AA AA AA
AG AG AG AA -- -- AG
100767860
4149 rs10107791 8 GG AG AG AG GG
GG AG GG GG GG GG GG GG
GG GG GG GG GG GG GG GG
GG GG GG GG AA GG AG AG
GG GG GG GG GG GG GG
100970952
4150 rs10107799 8 GG GG GG AG GG
AG GG GG GG GG GG AG GG
AG AG GG AG AG AG AA AG
AG AG AG AG AG AA AG AG
AG AG AG GG AA AG AA
5959744
4151 rs10107814 8 GG GG -- AG AG

GG AG AA AA AG AA AA AG
AG AG AA AG AA AG AG AG
GG AG GG AG AG AG GG AG
AG AG GG AG AG AG GG
132810827
4152 rs10107822 8 AA AG AA AA AA
AG AA AA AA AA AG AG GG
GG GG AA AG AA AG AA AA
AA AG AG AG AG AG GG AG
GG AA AG AG AA AA AA
92307978
4153 rs10107831 8 GG AG GG GG GG
GG GG GG GG GG GG GG GG
AG AG AG GG AG GG AG AG
AA GG AG AG GG AG GG GG
AA AG AG AG AA AA AA
15478125
4154 rs10107838 8 GG GG GG GG GG
GG GG GG GG GG GG GG GG
GG GG GG GG GG GG GG GG
GG GG GG GG GG GG GG GG
GG GG GG GG GG GG GG
9025795
4155 rs10107854 8 GG GG GG GG GG
GG GG GG GG GG GG GG GG
AG AG GG GG GG GG GG GG
GG GG GG GG AG GG AG AG
GG AG GG GG GG GG GG
143589208
4156 rs10107927 8 GG AG GG AG AG
GG AA GG GG GG GG AG GG
GG GG GG GG GG GG GG AG
AG AA AA AG GG GG GG GG
AG AG GG AG GG GG GG
26877573
4157 rs1010796 X GG GG AG AG AA
GG AA GG GG AA AG AG AA
AA AA AA AA AA AA AA AA
AA GG AA AA AA AA AA AA
AG AA GG AA AA AG AA
84152731
4158 rs10107973 8 GG GG GG GG GG
GG GG GG GG GG GG GG GG
GG GG GG GG GG GG GG GG
GG GG GG GG GG GG GG GG
GG GG GG GG GG GG GG
17916752
4159 rs1010798 17 AG AG GG AG AG
GG AG GG GG AG GG GG GG
GG GG AG GG GG AG GG AG

	GG	GG	GG	GG	GG	AA	AG	AG
	GG	GG	AG	AG	GG	GG	GG	
11528429								
4160	rs10108002		8	GG	GG	GG	AG	GG
	AG	AG	AA	AG	AG	GG	GG	GG
	GG	GG	AG	AG	GG	AG	AG	GG
	GG	GG	GG	GG	AG	GG	AG	AG
	AG	GG	GG	GG	AG	AG	AG	
70490015								
4161	rs10108016		8	AA	AG	GG	AG	AG
	AA	GG	AG	AA	AA	AG	GG	AG
	GG	GG	GG	AG	AG	AA	AA	AA
	AA	AA	AA	AA	AG	AA	AG	AA
	AA	AA	AG	AG	AG	AA	GG	
121772783								
4162	rs10108019		8	AG	AG	GG	AG	AG
	AG	GG	AG	AG	AG	GG	GG	GG
	GG	GG	AG	GG	AG	AG	AG	AG
	AG	GG	GG	AG	GG	GG	GG	GG
	AG	AG	GG	AG	AG	AG	AA	
39439518								
4163	rs10108100		8	AA	AC	AA	AA	AC
	AC	AC	AC	AC	CC	AC	AC	AC
	AC	AC	AC	AC	AA	CC	CC	AC
	AC	CC	CC	AC	AC	AC	AC	CC
	AC	AA	CC	AC	AC	CC	AC	
3153107								
4164	rs1010811		18	AA	AG	AG	AA	AA
	AA	AG	AG	AG	AG	GG	AG	AG
	AG	AG	AG	AG	AA	AG	AA	GG
	GG	AG	AG	AG	AA	AG	AG	AG
	AA	AG	AA	AA	AG	AG	AA	
19677786								
4165	rs10108150		8	AG	AG	AG	GG	GG
	GG	AG	AG	AG	AG	AA	AA	AA
	AA	AA	AG	GG	GG	AG	AG	AA
	GG	GG	AG	GG	AG	AA	AA	AA
	GG	AG	GG	AG	AG	GG	AG	
145687475								
4166	rs10108236		8	CC	CC	CC	AC	CC
	CC	AC	CC	CC	CC	CC	CC	CC
	CC	CC	CC	CC	CC	CC	CC	AC
	AC	CC	AC	CC	CC	CC	CC	CC
	CC	CC	CC	CC	CC	CC	CC	
112439615								
4167	rs10108241		8	GG	GG	GG	AG	GG
	AG	GG	GG	GG	GG	GG	GG	GG
	GG	GG	AG	AG	AG	GG	GG	GG
	AG	AG	AG	GG	GG	GG	GG	GG
	AG	GG	GG	GG	GG	GG	GG	

134339152
4168 rs10108264 8 AG GG AG AG AG
AG GG AG AG AG AG GG AG
AG GG AG GG AA AA AG AA
GG GG GG GG GG AA AG AG
GG AA AG AG GG AG GG
69468914
4169 rs10108278 8 AA AG AA AG AA
AG AA AA AG GG AG AG AG
AG AG AG AA AA AA AA AG
AG AG AA AA AG AG AG AG
AA AA AA AA AG AA AG
141660770
4170 rs10108298 8 AA AA AA AG AA
AA AG AG AA AA AG GG AG
AG GG AA AA AA AA AA AA
AA AA AA AA GG AG GG GG
GG AG AG AG AG AG AA
71380505
4171 rs1010830 8 AC CC CC AC CC
AC CC CC CC CC AC CC AC
AC AA AC AC AA AC AA CC
AC CC AC CC AA AC AA AA
CC AC AC AC AA AA AC
121257231
4172 rs10108312 8 AG AG AG AA GG
AG AG AG AG AG AA AG AG
AG AA AG AA GG GG AG GG
AG AA AG AA AA AG AG AG
AA AA GG AG GG GG GG
114034042
4173 rs10108355 8 AG AG AG AG AA
AA AG AA AG AG AA AA AA
AA AG AG AG AG AA AA AA
AG AG AG AG AG AA AG AG
AG AA AA AA GG GG GG
81175152
4174 rs10108383 8 GG GG GG GG GG
GG GG GG GG GG GG GG GG
GG GG GG GG GG GG GG GG
GG GG GG GG GG GG GG GG
GG GG GG GG GG GG AG
115335219
4175 rs1010840 2 AA AA AA AA AA
AA AA AA AA AA AA AA AA
AA AA AA AA AA AA AA AA
AA AA AA AA AA AA AA AA
AA AA AG AG AA AA AA
110432886
4176 rs10108415 8 AA AC AA AC CC

AC CC CC CC CC CC CC CC
CC CC AA AC AC CC CC CC
AC CC CC AC AC CC AC CC
CC AC CC AC CC CC AC
9086451
4177 rs10108451 8 AG AA AG AA AA
AA AA AA AA AA AA AG AA
AG AG AG AA AG AA AA AA
AA GG AG AG AA AA AA AA
AA AG AA AA AA AG AA
6562092
4178 rs1010848 8 CC AC CC CC CC
CC CC AC CC CC CC CC CC
CC AC AC CC AC CC AC AC
CC AC AC CC AC CC CC CC
AC AC AC AC AC AA CC
23351759
4179 rs10108503 8 AA AA AC AA AA
AA AA AC AC AC AC AA AC
AA AA AA AC AC AA AA AA
AC AA AA AA AA AC AA AA
AC AA AA AA AC AC AC
108151432
4180 rs10108534 8 AC CC AC CC AC
CC AC AC AC AC AC CC AC
AC CC AC AA CC AC AC AA
AC AC AC AC AA AA AA AA
AC CC AC AC AC AA CC
97883333
4181 rs10108561 8 GG GG GG GG GG
GG GG GG GG GG GG GG GG
GG GG GG GG GG GG GG GG
GG GG GG GG GG GG GG GG
GG GG GG GG GG GG GG
38291212
4182 rs10108572 8 AA AC AA AA AA
AA AA AA AA AA AA AA AA
AA AA AA AA AA AA AA AC
AA AA AA AA AA AA AA AA
AA AA AA AA AA AA AA
92258939
4183 rs10108615 8 AA AA AA AA AA
AA AA AA AA AA AA AA AA
AA AA AA AA AA AA AA AA
AA AA AA AA AA AA AA AA
AA AA AA AA AA AA AA
141668236
4184 rs10108622 8 AC AA AA AC AC
AA CC AC AC AC CC AC AC
AA AA AC AC AC AC AC AC

	AC	AC	AC	AC	AA	AC	AC	AC
	AA	AA	CC	AC	AA	AA	AC	
115562322								
4185	rs10108630		8	AA	AG	AA	AA	AA
	AA	AA	AG	AG	AG	AG	GG	AG
	AG	AG	AA	AA	AA	AG	AG	AG
	AG	AG	AG	AG	GG	AG	GG	AG
	AG	AA	AG	AG	AA	AG	AA	
3484731								
4186	rs10108662		8	CC	CC	CC	AA	AA
	AA	AA	AC	AC	AC	AC	CC	AC
	CC	CC	AC	AA	CC	AC	AC	AA
	CC	CC	CC	CC	AA	AC	AC	AC
	CC	AC	CC	AC	CC	AC	AC	
39779989								
4187	rs10108668		8	AA	AA	AA	AA	AA
	AA	AA	AA	AA	AA	AA	AA	AA
	AA	AA	AA	AG	AA	AG	AA	AG
	AG	AA	AG	AA	AA	AA	AA	AA
	AG	AA	AG	AG	AG	GG	AA	
29836592								
4188	rs1010874		10	GG	AG	AG	GG	GG
	GG	GG	GG	GG	GG	GG	GG	GG
	GG	GG	GG	GG	GG	GG	GG	GG
	GG	GG	GG	GG	GG	GG	GG	GG
	GG	GG	GG	GG	GG	GG	GG	
84501091								
4189	rs10108786		8	AC	AC	AC	CC	CC
	CC	CC	CC	CC	AC	CC	CC	CC
	CC	CC	CC	CC	CC	AC	AC	AC
	AA	CC	AC	AC	AC	CC	AC	AC
	CC	CC	AC	CC	AC	CC	AA	
74466473								
4190	rs10108855		8	GG	AG	AG	GG	GG
	GG	GG	GG	GG	GG	GG	GG	GG
	GG	GG	AG	GG	AG	GG	GG	GG
	AG	GG	GG	GG	GG	GG	GG	GG
	GG	GG	GG	GG	GG	GG	GG	
56467152								
4191	rs10108874		8	AG	GG	AG	GG	GG
	GG	GG	GG	GG	GG	GG	GG	GG
	GG	AG	AG	GG	AG	GG	GG	GG
	GG	GG	GG	GG	GG	GG	GG	GG
	AG	GG	GG	GG	AG	AG	GG	
80095928								
4192	rs1010889		10	AA	AA	AG	AA	AG
	AA	AG	GG	GG	GG	GG	AG	AG
	AG	GG	AG	AA	AG	AG	GG	AA
	AG	AG	AG	AA	AA	GG	AG	AG
	AA	GG	GG	GG	AA	AG	AA	

64839442
4193 rs10108910 8 AA AG AA AG GG
AG AG AA AG AG GG AG GG
GG GG GG AG AG AG AG AG
AA GG AG AG GG AA AG AG
GG AA AG AA GG GG GG
8419612
4194 rs10108984 8 AA AA AA GG GG
GG GG AG AA AA AA AG AG
GG AG AA AA AG GG AA GG
GG GG GG GG AG AA AA AG
GG GG AG GG AA AG AA
123973561
4195 rs10109057 8 AG GG GG GG GG
GG GG GG GG GG GG GG GG
GG GG GG GG GG GG GG GG
GG GG GG GG GG GG GG GG
GG GG GG GG GG GG GG
114557532
4196 rs10109061 8 AA AG AG AG AA
AG AA AG GG AA GG AG GG
AG AG GG GG GG AA AA AA
AG AG AG AG AG AA AA AA
GG AG AA AA AG AG AA
144239859
4197 rs10109063 8 AG GG AG GG AG
GG AG GG AG AG GG GG GG
GG GG AG GG AG AG AA AG
AG GG GG GG GG AG GG GG
AG GG AG GG GG GG GG
80745551
4198 rs10109178 8 AC AA AC AC AC
CC AC AA AC AC AA AA AA
AA AA AA AC AC AC AA CC
AA AA AA AA AC AA AC AA
AC AC AC CC AA AA AC
12876558
4199 rs10109186 8 AA AG AA AA AA
AA AA AG AG AG AA AG AA
AG AG -- GG -- GG AG AG
-- AG AG GG AG AG AG --
AG GG AG AG AG AA AG
5104646
4200 rs10109209 8 CC CC CC AC AA
AC AC CC CC CC CC AC CC
CC CC CC CC CC CC CC CC
CC CC CC CC CC CC CC CC
CC AC CC CC CC AC CC
5528944
4201 rs10109238 8 GG GG GG GG GG

GG GG GG GG GG GG GG GG
GG GG GG GG GG GG GG GG
GG GG GG GG GG GG GG GG
GG GG GG GG GG GG GG
49664864
4202 rs10109245 8 GG GG GG GG GG
GG GG AG AG GG GG GG GG
GG GG GG AG GG GG GG GG
GG GG GG GG GG GG GG GG
GG AG GG AG GG GG GG
73152314
4203 rs10109251 8 AG AG AG AG AA
AG AG AA AA AA AG AG AG
AG AG AG AG AG GG AG AG
GG GG GG GG AG GG GG GG
AG AG AG AG GG GG AG
416697
4204 rs10109252 8 AA AG AA AG AG
AG AA AA AA AA AG AA AG
AA AA AA AA AA AG AA AG
AG AG AA GG AA AA AA AA
AA AG AG AA AA AA AA
13973465
4205 rs10109279 8 AA AA AA AA AA
AA AA AA AA AA AA AA AA
AA AA AA AA AA AA AA AA
AA AA AA AA AA AA AA AA
AA AA AA AA AA AA AA
5035590
4206 rs10109286 8 AA AA AA AA AA
AA AA AA AA AA AA AA AG
AG AA AA AA AA AG AA GG
AA AG AG AA AA AA AA AA
AA AG AA AA AG AA AG
18243603
4207 rs10109346 8 AG AA AA AA AA
AA AA AG AG AG AG AA AG
AA AA AG AG AG AG AG AG
AG AG AG AG AA AG AG AG
AA AA AG AG AA AA AA
115536093
4208 rs10109373 8 AA AC AC CC AC
AC CC AC AC CC CC CC CC
AC AC AC AC AC AC AC CC
AC AC AC AC CC AC AC AC
AC AC AC AA CC AC CC
140521806
4209 rs10109406 8 AA AA AA CC AC
AC AC AC CC AC CC AC AC
AC AA AA AA AA AC AC AC

	AA	AA	AA	AA	CC	AA	AC	AC
	AC	AC	AA	AC	AA	AA	AA	
72632682								
4210	rs10109496		8	GG	GG	GG	GG	GG
	GG	GG	AG	AG	AG	AG	AG	AA
	AG	GG	GG	GG	GG	GG	GG	GG
	GG	GG	GG	GG	AG	GG	GG	AG
	AG	GG	GG	GG	GG	AG	GG	
117838623								
4211	rs10109510		8	AA	AA	AA	AG	AG
	AG	AA	AG	AA	AA	GG	GG	GG
	GG	GG	AA	AG	AA	GG	GG	AG
	AA	AA	AA	AA	AG	AG	GG	GG
	AG	AA	AG	AG	AA	AA	AG	
64882069								
4212	rs1010957		2	CC	CC	CC	CC	CC
	CC	CC	CC	CC	CC	CC	CC	CC
	CC	CC	CC	CC	CC	AC	AC	CC
	CC	CC	CC	CC	AC	CC	CC	CC
	CC	AC	CC	CC	CC	CC	CC	
15069050								
4213	rs10109589		8	AG	AA	AG	GG	GG
	GG	GG	GG	GG	GG	GG	GG	GG
	GG	GG	GG	GG	GG	GG	GG	GG
	GG	GG	AG	AG	AG	AG	AG	AG
	GG	GG	GG	GG	AG	GG	AG	
141530057								
4214	rs10109609		8	GG	AG	AG	GG	GG
	GG	GG	GG	GG	GG	AG	GG	AG
	GG	GG	AG	AG	GG	AG	AG	AG
	GG	GG	GG	GG	GG	GG	GG	GG
	AG	GG	GG	GG	GG	GG	GG	
107637500								
4215	rs10109697		8	AG	AG	AG	GG	AG
	GG	AG	GG	GG	GG	AA	AA	AA
	AG	AG	AG	AG	AG	AA	AA	AA
	AG	AA	AA	AA	AG	AA	AA	AA
	AG	GG	AG	GG	AG	AG	AG	
56514919								
4216	rs10109771		8	GG	GG	GG	AG	AG
	GG	AG	GG	GG	GG	GG	GG	GG
	AG	AG	GG	GG	GG	GG	GG	GG
	AG	AG	AG	AG	GG	GG	GG	GG
	AA	AG	AG	GG	AG	AA	GG	
143488721								
4217	rs1010978		X	AA	AA	AG	AG	AA
	AA	AG	AG	AG	AA	AG	AG	GG
	AG	AG	AA	AG	AG	AG	AG	AA
	AA	GG	AA	AG	AA	AA	AA	AA
	AG	GG	GG	GG	AA	AA	AA	

129479460
4218 rs10109833 8 GG AG GG GG GG
GG GG AG GG GG GG GG GG
GG GG GG GG GG AG GG AG
AG GG GG AG GG GG GG GG
GG GG GG GG GG AG GG
19156367
4219 rs10109846 8 GG AG AG GG GG
GG GG GG GG GG GG AG AG
AG GG AG AA GG GG AG AG
AG GG AG GG GG GG GG GG
GG AG AG GG AG AG AG
117077882
4220 rs10109851 8 AA AG AG GG GG
GG GG GG GG GG GG GG AG
AG AA GG AG AG AG AG AA
AG AG GG GG GG AG GG GG
GG AG GG AG GG GG GG
138420975
4221 rs10109866 8 AG AG AA GG GG
GG GG AG GG GG GG GG AG
AG AG GG GG GG GG GG GG
GG GG GG GG GG GG GG GG
GG GG AA AG AG AG AA
139751900
4222 rs10109984 8 AG AG AA AG AG
AA GG AA AG AG AA AA AA
AG AG AG AG AA AG AG GG
GG GG GG GG AG GG AG AG
GG AG AG AG AA AG AA
48803675
4223 rs10109988 8 CC CC CC AC AC
AA CC CC CC CC CC CC CC
CC CC AC AC CC CC CC CC
CC AC CC AC CC AA AC AC
AA AC AC AC AC AC AC
108850819
4224 rs1010999 8 AA AA AA AA AA
AA AA AG GG AG AG AG GG
GG AG AA AA AA AG AA GG
AA AA AA AA AG AA AA AG
AG AG AG AG AG AG AG
21395310
4225 rs10109996 8 AG AG AG AG AG
GG AA AG AA AA GG GG GG
AG AA AG AG AG AG AG AG
GG GG GG GG AG AA AA AA
AG GG GG GG GG GG GG
51988578
4226 rs10110 9 AA AA AA AC CC AC

CC AA AC AA AC AC AC AC
AC AC AA AC AA AC AA AA
AC AC AC AA AA AA AA AC
AA AA AA AA AA AA 140449582
4227 rs10110023 8 AA AA AA AA AA
AG AA AG AA AA AA AA AG
AG AA AG AA AA AA AA AA
AG AA AG AA AG AG AG GG
AG AA AA AA AG AA GG
17140378
4228 rs10110041 8 CC CC CC CC CC
CC CC CC CC CC CC CC CC
CC CC CC CC CC CC AC CC
CC CC CC CC CC CC CC CC
CC CC CC CC CC CC CC
127650064
4229 rs10110073 8 GG AG GG GG GG
GG AG AG AG AG GG GG GG
AG AG AG AG AA AG AG GG
GG GG GG GG GG AG GG GG
GG GG GG GG AG GG AG
1294073
4230 rs1011009 13 AA AA AG AG AG
AG AG AA AA AA AA AA AG
AA AA AA AA AA AA AA AA
AA AG AA AA AA AA AA AA
AA AA AA AA AA AA AA
43409346
4231 rs10110115 8 AA AA AA AA AA
AA AG AG AG AG GG AG GG
AG AG AG AA AA AG AG AA
GG GG AA AG AG AG AA AA
GG AA AA AA GG AG GG
143975633
4232 rs10110117 8 AA AC AC CC CC
CC AC CC CC AC AC AA AA
AA AC AC AC CC CC CC AC
AA AC CC AC AC AC AC AC
AC AC AA AC AC AC CC
31135931
4233 rs10110123 8 AA AA AA AC AA
AC AC CC CC CC AA AA AA
AA AA AC CC AC AA AC AC
CC AA AC AC AA AA AA AA
AC AC AA AA AA AC AA
10889623
4234 rs10110132 8 AC AC AC AC CC
AC CC AC AC AC CC AC AC
AC AC CC CC CC AC AC CC
AC CC CC CC AC CC CC AC

CC AC AC CC AA AC AC
106256501
4235 rs10110140 8 GG AG GG GG GG
AG AG AG GG GG AA AG AA
AA AA GG AG GG AG AG GG
AG AA AA AG GG GG GG GG
GG AG AA AA AG AG AG
27036631
4236 rs10110146 8 AA AA AA AC AA
AC AA AC AC AC AA AA AA
AA AA AC AA AC AC AA AC
AC AA AA AC AA AA AA AA
AA AC AA AC AC AC AC
9685362
4237 rs10110184 8 AA AA AA AA AA
AA AA AA AA AA AG AA AG
AA AA AA AA AA AG AA AG
AG AA AA AG AA AG AA AG
AG AG AA AA AA AA AG
3135739
4238 rs1011024 7 AA AA AA AA AG
AG AA AA AA AA GG AG AG
GG AA AG AA AA AA AA AA
AA AA AA AA AA AA AA AA
AA AG AG AG AA AA AG
99197401
4239 rs10110243 8 GG AG GG AG AG
GG AA AA AA AA AG AG AG
AG AG GG AG GG AG AG AG
AG AG AG GG AG AA AG AA
AA AG AG AG AG GG AA
128805020
4240 rs10110372 8 AA AA AA AA AA
AA AA AA AA AA AG AG AG
AG AA AA AA AA AA AA AA
AA AA AA AA AA AA AA AA
AA AA AA AA AA AA AA
127372598
4241 rs1011038 13 AA AG AA AA AA
AG AA AA AA AA AG GG AG
AG AG AA AG AG AG AA AG
GG AG GG AG AG AG AA AG
GG GG GG GG AG AG AG
20695554
4242 rs10110380 8 AG AA AG GG AG
AG GG AG AG AG AG AG AG
GG GG GG GG GG AG AA AA
AG GG GG AG AG GG AG GG
GG AG AG AA AA AG AG
10077806

4243	rs10110395		8	AA	AA	AA	AA	AA
	AA	AG	GG	GG	AG	AA	AG	AA
	AG	AG	AG	AG	AA	AA	AA	AA
	AG	GG	AG	GG	AA	AG	AG	AA
	AA	AA	AA	AA	AA	AG	AA	
2121846								
4244	rs10110414		8	GG	GG	GG	GG	GG
	GG	GG	GG	GG	GG	GG	GG	GG
	GG	GG	GG	GG	GG	GG	GG	GG
	GG	GG	GG	GG	GG	GG	GG	GG
	GG	GG	GG	GG	GG	GG	GG	
125642247								
4245	rs10110416		8	GG	GG	GG	GG	GG
	GG	GG	AA	AG	GG	GG	GG	GG
	GG	GG	GG	GG	GG	GG	GG	GG
	GG	GG	GG	GG	GG	GG	GG	GG
	AG	GG	AG	AG	GG	GG	AG	
69180842								
4246	rs10110528		8	AG	GG	GG	GG	AG
	GG	AG	AG	GG	GG	AG	AG	GG
	AG	AA	AG	AG	GG	AG	GG	AG
	AG	AA	AG	AA	AG	AG	GG	AG
	AA	AG	AG	AG	AG	AG	GG	
122686768								
4247	rs10110535		8	AA	AA	AA	AA	AA
	AA	AA	AA	AA	AA	AA	AA	AA
	AA	AA	AG	AG	AG	AA	AA	AA
	AA	AA	AA	AA	AA	AA	AA	AA
	AA	AA	AA	AA	AA	AA	AA	
72476365								
4248	rs1011056		10	AA	AA	AG	AG	GG
	GG	AG	AG	AG	AG	AG	AG	GG
	GG	GG	GG	AG	GG	AG	GG	AA
	GG	AG	AG	AG	GG	AG	AG	AG
	AA	GG	AG	AG	AG	AG	AA	
125370973								
4249	rs10110576		8	AG	AA	AG	AA	AA
	AA	AG	GG	GG	GG	GG	GG	AG
	GG	AG	GG	AG	GG	AG	AG	AG
	AG	AG	AG	AG	AA	GG	AG	AG
	GG	AA	AA	AA	AG	GG	AG	
128699454								
4250	rs10110578		8	AG	AG	AG	GG	AG
	GG	AG	GG	GG	GG	GG	GG	GG
	GG	GG	AG	AG	GG	AG	GG	AG
	AG	AG	AG	AG	AG	GG	AG	GG
	AG	GG	AG	AG	GG	GG	GG	
119294282								
4251	rs10110591		8	CC	AC	CC	CC	AC
	AC	AC	AA	AA	AA	AA	AC	AA

	AC	AC	AC	AA	AC	AC	AA	AC
	AA	AA	AA	AA	AA	AA	AA	AA
	AA	AA	AC	AC	AC	AC	AC	
8576608								
4252	rs10110604		8	AA	AA	AA	AG	AG
	AG	AG	AG	AA	AA	AG	AG	AG
	AG	AG	GG	AG	AG	GG	AG	AG
	AA	AA	AA	AA	AA	AA	AA	AA
	AA	AG	GG	GG	AA	AG	AA	
16503082								
4253	rs10110615		8	GG	GG	GG	GG	AG
	GG	AG	AG	GG	GG	GG	GG	GG
	GG	GG	AG	GG	AG	GG	GG	GG
	AG	GG	GG	GG	GG	GG	GG	GG
	AG	GG	GG	GG	GG	GG	GG	
80733696								
4254	rs10110636		8	AG	GG	GG	AG	AG
	AA	AG	GG	AG	GG	AG	AG	AA
	AG	AG	AA	AA	AA	GG	GG	GG
	GG	GG	GG	GG	AG	GG	GG	GG
	AG	GG	AA	AG	AA	AA	AA	
143233312								
4255	rs10110655		8	AA	AG	AG	AG	AG
	GG	AA	AG	AG	AA	AG	AG	AA
	AA	AA	AA	AG	AA	AA	AA	AA
	AG	AG	AG	AG	AG	AA	AA	AG
	AA	AA	GG	AG	AA	AG	AG	
125575619								
4256	rs10110697		8	AG	AG	AG	GG	GG
	AG	GG	AG	GG	GG	GG	GG	GG
	AG	AG	AG	AG	GG	GG	GG	AG
	AG	GG	AG	GG	GG	GG	GG	GG
	AG	GG	AG	GG	GG	AG	GG	
15669454								
4257	rs10110701		8	AC	CC	AC	AC	AC
	AC	AA	AC	AA	AC	AA	AA	AA
	AA	AA	CC	CC	AC	AC	AC	CC
	AC	AC	CC	CC	AA	AC	AC	AC
	AC	AC	AC	AA	AC	CC	AA	
77234939								
4258	rs10110727		8	GG	GG	GG	AG	AG
	GG	AG	GG	GG	GG	GG	AG	GG
	GG	GG	AA	AG	AG	AG	GG	AG
	GG	GG	GG	GG	GG	AG	GG	GG
	--	AG	GG	AG	AG	AA	GG	
14324437								
4259	rs10110780		8	GG	GG	GG	GG	GG
	GG	GG	GG	GG	GG	GG	GG	GG
	GG	GG	GG	GG	GG	GG	GG	GG
	GG	GG	GG	GG	GG	GG	GG	GG

GG GG GG GG GG GG GG
142120286
4260 rs10110812 8 GG GG GG GG GG
GG GG GG GG GG AG AG AG
AG AG GG GG GG GG GG GG
AG GG GG AG AG AG AG GG
GG GG AG GG GG GG GG
6179115
4261 rs1011088 6 AA AA AA AG AG
GG AA GG GG GG AA AA AG
AA AG AG AG AG AA AA AA
AA AG AA AG AA AA AA AA
AG GG AA AG AA AG AA
4481421
4262 rs10110897 8 AA AA AG GG AG
AG GG AA AA AA AG AG AG
AG AG AA AG AG AG AA AA
GG GG GG GG AA GG AG AG
AG GG AG AG GG GG GG
139114168
4263 rs10110940 8 AG AG AA AA AA
AA AA AA AA AG AA AG AG
AG AA AA AA AA AG AG AA
AA AA AA AA AA AA AA AA
AA AA AA AA AA AA AA
29287001
4264 rs10110946 8 AA AA AA AG AG
GG AA GG GG GG AA AA AA
AA AA AA AA AA AG AG AA
AG AA AG AA AA AG AA AA
AA AA AG AA AG AA AG
140685621
4265 rs1011097 16 AG AG AA GG GG
GG GG AA AA AA AG AG AG
AG GG AA AA AA AG AA GG
AA AA AA AA AG AA AG AA
AA AG AG GG AA AA AA
80753476
4266 rs10110988 8 GG GG GG GG GG
GG GG GG GG GG AG AG AG
GG GG GG GG GG GG GG AG
GG AG AG GG GG GG GG GG
AG AG GG GG GG AG GG
448295
4267 rs10110999 8 AG AG AG AA AG
AG AG AG AG GG GG GG GG
AG AG AG GG GG GG GG GG
GG GG AG AG GG AG AG AG
AG GG AG AG GG GG GG
141372344

4268 rs10111 11 GG GG GG GG AG AG
GG AG GG GG GG GG GG GG
GG GG GG GG GG GG GG GG
GG GG AG GG AG GG AG GG
GG GG GG AG AG GG 118886117
4269 rs1011100 6 GG AG AG AG GG
GG AG AG GG AA GG GG GG
GG GG GG AG AG GG GG AG
AA AA AA AG AG AG GG GG
AG GG AG AG GG GG GG
41643245
4270 rs10111012 8 AA AA AA AA AA
AA AA AA AA AA AA AA AA
AA AA AA AA AA AA AC AA
AA AA AA AA AA AA AA AA
AA AA AA AA AA AA AA
110153393
4271 rs10111027 8 GG GG GG GG GG
GG GG GG GG GG GG GG GG
GG GG GG GG GG GG GG GG
GG GG GG GG GG AG GG GG
GG GG GG GG GG GG GG
40270318
4272 rs10111060 8 AA AG AA AA AA
AA AA AG AG AG AG AA AG
AG AA AG AG AG GG GG GG
AG AG AG AG AG GG GG AG
GG AG GG GG AG AG AG
91491152
4273 rs10111087 0 GG GG GG AG AG
GG AG GG GG GG GG AG AG
AA AA AG GG GG AA AA AG
AG GG AG GG GG GG GG GG
AG AG GG AG AG AG GG 0
4274 rs10111139 8 AA AA AG GG AG
AG AG AA AA AA AG AG AG
AG AG AA AG AG AA AG AA
GG GG GG GG AG GG GG GG
AA GG AG AG GG GG GG
139084283
4275 rs10111291 8 AG AA AA AA AA
AA AA AA AA AA AA AA AA
AA AA AG AG AG AG AA AA
AG AA AG AA AG AA AG AA
AA AA AA AA GG AG AG
121266654
4276 rs10111298 8 GG AG GG GG AG
AG GG AA AA AA GG GG GG
GG GG GG GG GG GG GG AG
GG GG GG AG AG GG GG GG

GG GG GG GG GG GG AG
135923654
4277 rs1011130 16 GG GG GG GG GG
GG GG GG GG GG GG GG GG
GG GG GG GG GG GG GG GG
AG GG AG AA GG GG GG GG
GG AG GG GG GG GG GG
57108821
4278 rs10111305 8 GG GG GG GG GG
GG GG GG GG GG GG GG GG
GG GG GG GG GG GG GG GG
GG GG GG GG GG GG GG GG
GG GG GG GG GG GG GG
141365387
4279 rs10111331 8 AG AA AG AA AG
AG AA AA AA AA AG AG AG
AA AA GG AG GG AA AA AA
AG AG AG AG GG GG GG GG
AG GG AG AG AA AG AG
125850756
4280 rs10111362 8 AA AG GG AG AA
AA AG AA AA AG AA AA AA
AA AA AA AA AA AG AG AA
AA AA AA AA AG AG GG AG
AA AA AA AA AG AG AG
125804500
4281 rs10111398 8 AA AC AA CC CC
AC CC CC CC AC AC CC AC
AC CC AC AC AC AC AC AA
AA AC AC AC AA AC AC AC
AC AC AC CC CC CC AC
83542128
4282 rs10111402 8 CC AC CC CC CC
AC CC AC AC AC AC CC AC
AC AC AC AA CC AC AC AC
CC CC CC CC AA AC AA AA
AA AA AA AA CC AC CC
91235642
4283 rs10111409 8 AG AA AG GG AG
AG GG AG AG AA GG GG GG
GG AG AG AG AG AA AG AG
GG GG GG AG AG GG GG AG
AG AG AG AG AG AG AG
133118482
4284 rs10111417 8 AA AA AA AA AA
AA AA AA AA AA AA AA AA
AA AA AA AA AA AA AA AA
AA AA AA AA AA AA AA AA
AA AA AA AA AA AA AA
123852196

4285 rs10111457 8 AA AA AG AG AG
GG AA AA AA AA AG GG AG
AG AG GG GG AG AG GG AG
AA AA AA AA AG AG AA AG
GG AA AG AG AG AG AA
109433315
4286 rs10111470 8 AG AA AG AA AA
AA AG AG AA AA AA AG AA
AG AG AG AG AA AG AG AG
AG AG AA GG GG AG AG AG
AA AG AG AA AA AG AG
14919557
4287 rs10111473 8 GG GG GG GG GG
GG GG GG GG GG GG GG GG
GG GG GG GG GG GG GG GG
GG GG GG GG GG GG GG GG
GG GG GG GG GG GG GG
142090080
4288 rs10111517 8 GG GG GG GG GG
AG GG AG AG GG GG AG AG
AG AA GG AG GG AG AG AA
GG AG AG GG GG GG GG GG
AG AG AG AG AG AG GG
69901440
4289 rs10111524 8 AC AC AC AA CC
AC AC AC AC CC AA AA AC
AC CC CC AC AC AC CC CC
CC AC CC AC AC AA AA AC
AA CC AC AC AC AC CC
124908497
4290 rs10111587 8 AA AA AA AA AA
AA AA AA AA AA AA AA AA
AA AA AA AA AA AA AA AA
AA AA AA AA AA AA AA AA
AA AA AA AA AA AA AA
112813437
4291 rs10111597 8 AG AG AG AG AG
GG AG AG AG GG AG AG AG
GG GG GG AG GG GG AA GG
AG AG AA GG AG GG GG GG
AG GG AG AG GG GG GG
1161954
4292 rs1011160 15 AA AA AA AG AA
AG AA AG AG AG AA AA AA
AA AA AA AA AA AG AG GG
AA AA AA AG AG AG GG AA
AA AG AA AG AG AG AA
102080355
4293 rs10111612 8 GG GG GG GG GG
GG GG GG GG GG GG GG GG

GG GG GG GG GG GG GG GG
GG GG GG GG GG GG GG GG
GG GG GG GG GG GG GG
38677907
4294 rs10111713 8 CC AC AC CC CC
CC CC AC AA AA CC CC CC
CC CC AC AA CC AC AC AA
CC AC AC CC CC CC CC CC
CC CC CC CC AC AC AA
54237672
4295 rs10111815 8 AA AA AA AA AA
AA AA AA AA AA AG AG AG
AG AG AA AA AA AA AA AA
AA AA AA AA AA AG AG AA
AA AA AA AA AA AA AA
2706777
4296 rs10111831 8 GG GG GG AG AG
AA GG AG AG AA AA AA AA
AA AA AA AA AA AG AA AA
AG AA AG AG AA AA AA AA
AG AA AG AG AG AG AA
82791846
4297 rs10111833 8 GG GG GG GG GG
GG GG GG GG GG GG GG GG
GG GG GG GG GG GG GG GG
GG GG GG GG GG GG GG GG
GG GG GG GG GG GG GG
48335305
4298 rs10111852 8 AA AG AA AG AA
AG AA AG AG GG AG GG AG
AG AG AG AG AG AG AG AG
AG AG AA AA AG AG AG AG
AG AA AG AA AG GG AG
141937541
4299 rs10111872 8 GG GG GG GG GG
GG GG GG GG GG GG GG GG
GG GG GG GG GG GG GG AG
GG GG GG GG GG GG GG GG
GG AG GG GG GG AG GG
10881408
4300 rs10111995 8 CC CC CC CC CC
CC CC CC CC CC CC CC CC
CC CC CC CC CC CC CC CC
CC CC CC CC CC CC CC CC
CC CC CC CC CC CC CC
91466739
4301 rs10112035 8 AG AA AG AA AG
AA AG AA AA AA AA AA AA
AA AA AG AA AG AA AA AA
AA AA AA AA AG AA AA AA

	AA	AG	AA	AG	AG	AG	AG
60010000							
4302	rs1011205	3	AA	AG	AA	AA	AA
	AA	AA	AA	AA	AA	AA	AA	AA
	AA	AA	AA	AG	AG	AA	AA	AG
	AA	AA	AA	AA	AA	AG	AG	AG
	AA	AG	AA	AA	AA	AG	AA	
97994268								
4303	rs10112126	8	GG	GG	GG	AG	GG
	AG	AG	GG	GG	GG	AG	GG	AG
	GG	GG	GG	GG	GG	GG	GG	GG
	GG	GG	GG	GG	GG	AG	AG	GG
	GG	AG	AG	AA	GG	GG	GG	
5535139								
4304	rs10112163	8	AA	AG	AA	AG	AA
	AA	AG	AA	AA	AA	AA	AG	AG
	AG	AG	AA	AG	AA	GG	AG	AG
	AA	AG	AA	AA	AA	AG	AA	AA
	GG	AA	AA	AA	GG	AG	GG	
85657553								
4305	rs10112191	8	GG	GG	GG	GG	GG
	GG	GG	GG	GG	GG	GG	GG	GG
	GG	GG	GG	AG	GG	AG	GG	GG
	GG	GG	GG	GG	GG	GG	GG	GG
	GG	GG	GG	GG	GG	GG	GG	
77052260								
4306	rs10112200	8	AA	AA	AA	AG	AA
	AG	AG	AG	AG	AG	AA	AA	AA
	AA	AA	AG	AA	AA	AA	AA	AA
	GG	GG	GG	AG	AA	AA	AA	AA
	AG	AG	AA	AG	AG	AG	AG	
132953350								
4307	rs10112201	8	AA	AA	AA	AA	AA
	AA	AA	AA	AA	AA	AA	AA	AA
	AA	AA	AA	AA	AA	AA	AA	AA
	AA	AA	AA	AA	AA	AA	AA	AA
	AA	AA	AA	AA	AA	AA	AA	
142382757								
4308	rs10112217	8	AA	AA	AA	AG	AG
	GG	AA	AG	AG	GG	AA	AA	AA
	AG	AG	AG	GG	AG	AA	AG	AA
	AA	AG	AA	AG	AG	AG	AG	GG
	AA	AG	AG	AA	AG	AA	GG	
4005375								
4309	rs1011223	16	GG	GG	GG	GG	GG
	GG	AG	GG	GG	GG	GG	GG	GG
	GG	GG	GG	GG	GG	AG	AG	AG
	GG	GG	GG	GG	GG	GG	GG	GG
	GG	AG	AG	GG	GG	GG	GG	
56607856								

4310 rs10112245 8 AA AA AA AC AC
AA AC AA AA AA AA AA AA
AA AA AC AC AA AA AC AA
AC AA AC AA AC AA AC AC
AA AA AC AA AC AA AC
5137057
4311 rs1011233 18 AA AG GG AG AG
AG GG GG GG GG GG GG GG
GG GG AA AG AG AG AG GG
GG GG AG GG GG GG GG GG
AG GG GG GG GG GG GG
76315261
4312 rs10112364 8 AG AG AA AA AA
AA AA AA AA AA AA AA AA
AA AA AG AA AG AG AA AA
AA AA AA AA AA AG AG AG
AA AA AA AA AA AA AA
95682296
4313 rs10112377 8 -- -- -- GG --
-- GG -- -- -- GG -- GG
GG -- -- GG -- GG -- GG
-- -- -- GG GG GG GG --
GG GG -- -- GG -- --
95353114
4314 rs10112382 8 GG AG GG AG AG
GG AA AA AA AA AG AG AG
GG GG GG AG GG AG AG AG
AG AG AG GG AG AA AG AA
GG AG AG AG GG GG GG
128784397
4315 rs10112386 8 AA AA AA AA AA
AA AA AA AA AA AG AG AA
AA AA AA AA AA AG AG GG
AG AA AA AA AA AG AG AG
AA AA AG AA AA AA AA
74281152
4316 rs10112398 8 GG GG GG AG AG
AG AG AG GG GG GG GG GG
GG AG AG AG AG AG AG AG
GG GG GG GG GG AA AG AG
AG AG GG GG AA AA AG
93795052
4317 rs10112439 8 GG AG GG GG AG
AG AG GG GG GG AG AG AA
AA AA GG GG GG AG GG GG
GG AG AG AG AG GG AG GG
AG GG AA AG AG GG AG
92016879
4318 rs10112481 8 CC AC CC AA AA
AA AA AC AC AC AC AC AC

```
        AA      AA      CC      AC      AC      AA      CC      AA
        AA      AA      AA      AA      AA      AA      AA      AA
        AA      AA      CC      AC      AA      AA      AC
4875172
4319    rs1011254       8       GG      AG      GG      GG      AG
        AG      AG      GG      GG      GG      AG      AG      AA
        AA      AA      GG      GG      GG      AG      GG      GG
        GG      AG      AG      AG      AG      GG      AG      GG
        AG      GG      AA      AG      AG      GG      AG
91907491
4320    rs10112564      8       GG      GG      GG      GG      GG
        GG      GG      GG      GG      GG      GG      GG      GG
        GG      GG      GG      GG      GG      GG      GG      GG
        GG      GG      GG      GG      GG      AG      AG      AG
        GG      GG      GG      GG      GG      GG      GG
103838004
4321    rs10112584      8       GG      GG      GG      GG      GG
        GG      GG      GG      GG      GG      AG      GG      AG
        AG      AG      GG      GG      GG      GG      AG      GG
        GG      GG      GG      GG      AG      GG      AG      GG
        GG      GG      GG      GG      AG      AA      GG
10279503
4322    rs10112596      8       GG      GG      GG      AG      AG
        AG      GG      AG      AG      AG      GG      GG      GG
        GG      GG      AG      AG      GG      GG      GG      GG
        AG      GG      GG      AG      AG      GG      AG      GG
        GG      GG      AG      GG      AA      AG      AG
11579802
4323    rs10112642      8       AA      AA      AA      AA      AA
        AA      AA      AA      AA      AA      AA      AA      AA
        AA      AA      AA      AA      AA      AA      AA      AA
        AA      AA      AA      AA      AA      AA      AA      AA
        AA      AA      AA      AA      AA      AA      AA
144203983
4324    rs10112655      8       AA      AA      AA      AA      AA
        AA      AA      AA      AA      AA      AA      AA      AA
        AG      AG      AA      AA      AA      AA      AA      AG
        AG      AA      AA      AG      AA      AA      AA      AA
        AG      AG      AA      AG      AA      AA      AA
49064147
4325    rs10112708      8       AA      AA      AA      AC      AC
        AA      CC      AC      AC      AC      AC      AA      AC
        AC      AA      CC      AC      AC      AC      AC      AC
        AC      AC      AC      AC      CC      AA      AC      AC
        AA      AA      AA      AA      AA      AA      AA
80539224
4326    rs10112712      8       AG      GG      AG      AG      AG
        AA      GG      GG      GG      GG      AA      AG      AG
        AG      AA      GG      GG      GG      AA      AG      AG
        AA      AA      AA      AA      AG      AA      AA      AG
```

AG AA AG AA GG AG GG
114876935
4327 rs10112737 8 AA AA AA AA AG
AA AG AA AA AG AA AA AA
AA AA AG AA AG GG GG GG
AG AG AG AG AG AG AG AA
AG AG AG AG AG AA AG
126285326
4328 rs10112772
8 -- -- -- -- -- -- -- --
-- -- -- -- -- -- -- --
-- -- -- -- -- -- -- --
-- -- -- -- -- -- -- --
-- -- -- -- 82381567
4329 rs10112844 8 AA AA AA GG GG
GG GG GG GG GG AG AG GG
GG GG AG AG AG AG AG GG
AG AG AG GG AA AG AG AG
AA GG AG AG AG GG AG
73122967
4330 rs10112865 8 GG AG GG GG GG
GG GG AA AG AG AG AG GG
GG GG GG GG AG GG GG GG
AG AG AG AG AA AG AG AG
AG AG GG AG GG GG AG
88440271
4331 rs10112884 8 AA AA AA AA AA
AA AG AG AG AG AG AG AG
AG GG AG AG AG AG GG GG
AA AG AG GG GG AG AG AG
AG AA AG AG AG AG AA
138777847
4332 rs10112900 8 AG AG AG GG GG
GG GG GG AG AG AG GG GG
GG AG GG GG GG GG AG AG
GG GG GG GG GG GG GG GG
AG GG GG GG GG GG AG
19646515
4333 rs10112939
8 -- -- -- -- -- -- -- --
-- -- -- -- -- -- -- --
-- -- -- -- -- -- -- --
-- -- -- -- -- -- -- --
-- -- -- -- 123884361
4334 rs10112963 8 AG GG GG AG AA
AA AG GG GG GG GG GG GG
GG GG GG GG AG AG AG AG
GG GG GG GG AG GG GG AG
GG AG AA AG AA AG AG
102162717

4335 rs10112969 8 AA AA AA AA AA
AA AA AA AA AA AA AA AA
AA AA AA AA AA AA AA AA
AA AA AA AA AA AA AA AA
AA AA AA AA AA AA AA
72852443
4336 rs10112981 8 GG AG AG GG AA
AG AG AA AA AA GG AG AG
AG GG GG GG AG GG GG AG
AG AA AG AA AG AG GG AG
GG GG AG AG AA AG AA
114965243
4337 rs10113026 8 AG AG AG GG GG
GG GG GG GG GG GG GG GG
GG GG GG GG GG GG GG GG
GG GG GG GG GG GG GG GG
GG GG GG GG GG GG GG
22262630
4338 rs1011308 9 AG AA AG AA AA
AA AA AG AG GG AA AG AA
AA AA AG AA AA AG AA AG
AA AA AA AA GG AA AG AG
AA AA AA AA AA AG AG
73288256
4339 rs1011311 11 AG AG GG AG GG
GG AG GG AG AG GG GG GG
GG GG GG GG AG GG GG GG
GG AG GG AG AG GG GG AG
GG GG AG GG GG GG GG
78945216
4340 rs10113126 8 AA AA AG AA AA
AA AA AA AA AG AG AG AA
AA AA AG AA AA AG AG AG
AA AG AA GG AG AG AG AG
AA AG AA AG AA AG AA
136032287
4341 rs10113205 8 GG GG GG GG GG
GG GG GG GG GG GG GG GG
GG GG GG GG GG GG GG GG
GG GG GG GG GG GG GG GG
GG GG GG GG GG GG GG
92084087
4342 rs10113210 8 AA AG AA GG GG
GG GG AG AG GG AA GG AG
AG AG AA AG AA AA AA AA
AG AG AG AG GG GG GG GG
GG AG AA AA AA AG AA
25965198
4343 rs10113242 8 AA AA AA AA AA
AC AA AC AC AC AA AC AA

AC AC AA AA AA CC CC AC
AA AC AC AA AC AA AA AA
AA AC AA AA CC AC AC
39976970
4344 rs10113295 8 AG AA AA AA AG
AA AG AG AA AA AG AG AA
AG GG AG AG AA AG AA AG
AG GG AG GG AG AG AA AG
GG AG AG AG AG AG AA
122692580
4345 rs10113311 8 AA AA AA AA AA
AA AA AA AA AA GG AG AG
AG AA AG GG AG AG AG AA
AG AG AG AG AA AG AA AA
GG AG AG AA GG AG GG
61888469
4346 rs10113359 8 AG AA AG AA AA
AA AA AG AG AG AA AA AA
AA AA AA AA AA AA AA AA
AA AA AA AA AA AG AG AA
AA AA AA AA AG AA AG
2407398
4347 rs1011340 11 CC AC AC CC CC
CC CC CC CC CC AC CC AC
AC AC CC CC CC CC AC CC
AC CC AC CC AC CC AC AC
AC CC AA AC AC CC AC
42217564
4348 rs10113404 8 GG GG GG AG GG
AG GG GG GG GG GG GG GG
GG GG AA AA AA AG GG AG
GG GG GG GG GG AG AG AG
GG GG GG GG GG GG AG
135499696
4349 rs1011347 12 CC AC CC AC CC
AC CC CC AC AC CC CC AC
CC AC AC AC AC CC CC CC
CC CC CC CC AA AC AC AA
AC AC AC CC AC CC AC
131164897
4350 rs10113507 8 AC AC AC AA AA
AC AA CC CC CC AC AC CC
CC AC AA AC AC AC AC AC
AA AA AA AA AC AC CC AC
AC AA AC AC AA AA AC
91153308
4351 rs10113591 8 GG GG AG AA AA
AA AA AG AG AG AG AG GG
AG AA GG GG GG AG GG AG
AG AG AG AG AG AG GG AG

AA AA GG AG AG AG GG
115345232
4352 rs10113673 8 -- CC AC AA AA
AA AA AA AA AA AA AA AA
AA AA AC -- AA AA AC --
AA AA AA AA -- AA AC AC
-- -- -- AA AA AA --
12703642
4353 rs10113732 8 GG GG GG AG GG
AG GG GG GG GG GG GG GG
GG GG GG GG GG GG GG GG
GG GG GG GG GG GG GG GG
GG GG GG GG GG GG GG
113311911
4354 rs10113741
8 -- -- -- -- -- -- -- --
-- -- -- -- -- -- -- --
-- -- -- -- -- -- -- --
-- -- -- -- -- -- -- --
-- -- -- -- 4032622
4355 rs10113804 8 AG AA AA AG AG
AG AA AG AG AG AG AA AA
AA AA GG AG AG AA AA AA
AG AG AG AG AA AG AG AG
AG AG AG GG AA AA AG
40687433
4356 rs10113822 8 AC AC AC AA AA
AA AA AA AA AA AA AA AA
AA AA AA AA AA CC CC AC
AA AA AA AA AC AA AC AC
AC AA AA AA AA AA AA
64335958
4357 rs10113823 8 GG GG GG GG GG
GG GG GG GG GG GG GG GG
GG AG GG GG GG GG GG GG
GG GG GG GG AG GG AG AG
GG GG GG GG GG GG AG
62044143
4358 rs10113837 8 AA AA AA GG AG
AG AG GG GG AG AG AG AG
GG GG AG AG AG AG AG GG
GG AA AG AG AA AA AA AA
AG AG AG GG AG AA GG
1116809
4359 rs1011387 8 AA AA AA AA AA
AA AA AA AA AA AA AA AA
AA AA AA AG AA AA AG AG
AA AA AA AA AA AA AA AA
AA AA AA AA AG AA AG
128281911

4360 rs1011388
10 -- -- -- -- -- -- -- --
-- -- -- -- -- -- -- --
-- -- -- -- -- -- -- --
-- -- -- -- -- -- -- --
-- -- -- -- 45757282
4361 rs10113912 9 GG GG AG AA AG
AG AA GG GG GG AG AG GG
AG AG GG AG AG AG GG AG
GG AG GG AG AA GG AG AG
AG AA AA AA GG GG AG
131700209
4362 rs10113922 9 AA AA AA AA AA
AA AA AA AA AA AA AA AA
AA AA AA AA AA AA AA AA
AA AA AA AA AA AA AA AA
AA AA AA AA AA AA AA
80880267
4363 rs10113949 9 AA AA AA AA AA
AA AA AA AA AA AA AA AA
AA AG AA AA AA AA AA AA
AA AA AA AA AA AA AA AA
AA AA AA AA AA AA AA
72027706
4364 rs1011397 2 AG GG AG AA AA
AA AA AG AA AA AG GG AG
AA AA AA AA AA AA AG AA
AG AA AA AG AG AA AA AG
AA AA AA AA AA AA AA
144690117
4365 rs10113989 9 AA AG AA AA AA
AA AG AA AA AA AA AG AG
AG AA AA AG AA AA AA AA
AA AA AA AA AA AA AA AA
AA AA AG AA AA AA AA
109185446
4366 rs10113990 9 AA AA AA AG AG
AG AA AA AA AA AA AA AA
AA AA AA AA AA AA AA AA
AA AA AA AA AA AA AA AA
AA AA AA AA AA AA AA
23231298
4367 rs10113994 9 AG GG AG GG GG
GG GG GG AG GG GG GG GG
GG GG GG GG GG GG GG GG
GG GG GG GG GG GG GG GG
GG GG GG GG GG GG AG
16185621
4368 rs1011402 11 GG AG GG AG GG
GG AG AG AG AG AG GG AG

GG	AG	AG	AG	GG	GG	AG	AG
AA	AG	AA	AG	AG	GG	AG	GG
GG	AA	AG	AA	GG	GG	AG	

131291414
4369 rs10114099 9 AA AA AA AA AA

AA	AA	AA	AA	AA	AA	AA	AA
AA	AA	AA	AA	AG	AA	AA	AA
AA	AA	AA	AA	AA	AA	AA	AA
AA	AA	AA	AA	AA	AA	AA	

132766685
4370 rs1011415 20 GG GG GG GG GG

GG	GG	GG	AG	GG	AG	AG	AG
GG	AG	AG	AG	AG	GG	GG	GG
GG	AG	GG	AG	GG	GG	GG	GG
GG	AG	GG	GG	GG	GG	AG	

60245860
4371 rs1011416 3 AA AG AA AA AG

AG	AA	AA	AA	AA	AG	AG	AA
AG	AG	AA	AG	AG	AG	AA	AA
GG	AG	GG	AG	AA	AG	AA	AG
AA	AG	AA	AA	AA	AA	AA	

28796677
4372 rs10114161 9 AG GG AG AG AG

AG	AG	AA	GG	AA	AG	AG	AG
AG	GG	AA	AA	AA	AG	AG	AG
AG	AG	AG	AG	AA	AA	AA	AA
AA	AA	AA	AA	AA	AA	AA	

83822268
4373 rs10114299 9 CC CC CC AA AC

AC	AA	CC	CC	CC	AA	CC	AC
CC	CC	CC	CC	CC	CC	AC	CC
CC	CC	CC	CC	CC	CC	CC	CC
AC	CC	AC	CC	CC	CC	AC	

12182768
4374 rs10114302 9 AC CC AC AA AA

AA	AA	AA	AC	AA	AC	AA	AA
AA	AC	AA	AA	AA	AA	AA	AA
AC	AA	AA	AA	AC	AC	AC	AA
AA	AA	AA	AA	AA	AA	AA	

138718981
4375 rs10114341 9 GG GG GG AG AA

AG	AA	GG	GG	GG	AA	AA	AA
AA	AG	AG	AA	AA	AG	AG	AG
AA	AA	AA	AA	AG	GG	AG	AG
AG	AA	GG	AG	AG	AG	AG	

96919182
4376 rs10114417 9 GG GG GG GG AG

GG	AG	AA	GG	AG	AA	AA	AA
AG	AA	AA	AA	AA	GG	AA	GG
AG	AG	AG	AA	AG	AA	AA	AG

AG AA AG AA AA AG AG
1459663
4377 rs10114425 9 GG GG GG GG GG
GG GG GG GG GG GG GG GG
GG AG GG GG GG GG AG GG
GG AG GG AG GG GG GG GG
GG AA GG AG GG GG GG
111152200
4378 rs10114470 9 GG GG AG AG AG
AA GG AG AG AA GG GG GG
GG GG GG AG GG GG GG AG
GG GG AG GG AG AG AG AG
AG AG GG AG AG GG AA
117547772
4379 rs10114488 9 AA AA AA GG AG
GG AG AG AA AA AG AA AG
AG AA AG AA AG AA AA AA
AG AA AG AA AG AG AA AG
AA AA AA AA AA AG AG
92526756
4380 rs1011449 7 AA AA AA AA AA
AA AC AA AA AA AC AC CC
CC AC AC AC AC AA AA AC
AA AA AA AA AA AA AA AA
AA AC AA AC AA AA AA
114454337
4381 rs10114491 9 AA AA AA AG GG
AG AG AA AA AA AG AA AG
AG AG AA AA AG GG GG AG
AA AG AA AG AA AA AA AA
AA AG AA AA AG AG AG
105093304
4382 rs1011450 16 AA AA AA AA AA
AA AA AG AG AG AA AA AA
AA AA AA AA AA AA AA AA
AG AA AG AG AA AA AA AA
AG AA AA AA AA AA AA
47742330
4383 rs10114507 9 AG GG AG AA AA
AA AA AA AA AA AA AA AA
AA AG AG AG AG AA AA AG
AA AA AA AA AA AG AA AA
AA AA AG AA AG AG AG
34536873
4384 rs1011459 14 AA AA AA AA AA
AA AA AG AA AG AA AA AA
AA AG AA AA AA AA AA AA
AA AA AA AA AA AA AA AA
AA AA AA AA AA AA AA
95400455

4385	rs10114627		9	GG	GG	GG	GG	GG
	GG	GG	GG	GG	GG	GG	GG	GG
	GG	GG	GG	GG	GG	GG	GG	GG
	GG	GG	GG	GG	GG	GG	GG	GG
	GG	GG	GG	GG	GG	GG	GG	
136909280								
4386	rs10114675		9	GG	GG	GG	GG	GG
	GG	GG	AG	AG	AG	GG	GG	GG
	GG	GG	GG	GG	GG	GG	GG	GG
	GG	GG	GG	GG	GG	AG	AG	AG
	GG	GG	GG	GG	GG	GG	GG	
82759463								
4387	rs10114729		9	AG	GG	AG	AG	AA
	AG	AG	AA	AA	GG	AA	GG	AG
	GG	GG	AA	AA	AG	AA	AA	AG
	AG	AG	AG	AG	AG	GG	GG	AG
	AA	AG	AG	AG	GG	GG	GG	
11519008								
4388	rs1011474		15	GG	GG	GG	AG	AG
	GG	AG	AG	AG	AA	GG	GG	GG
	GG	GG	AG	AG	AG	AG	GG	AG
	GG	GG	GG	GG	AG	GG	AG	GG
	GG	GG	GG	GG	GG	GG	AG	
97373752								
4389	rs1011476		11	AC	CC	CC	AC	AC
	CC	AC	CC	CC	CC	CC	AC	AC
	CC	AC	AC	CC	CC	CC	CC	CC
	AC	CC	AC	CC	AC	AC	AA	AC
	AC	CC	CC	CC	AC	CC	AC	
2299035								
4390	rs10114761		9	AG	AA	AG	AA	GG
	AG	AG	AA	AG	AA	AA	AA	AA
	AA	AG	AA	AG	AA	AA	AA	AA
	AA	AA	AA	AA	AG	GG	AG	AG
	AA	GG	AG	AG	AG	AA	GG	
101688707								
4391	rs10114830		9	CC	CC	CC	CC	CC
	CC	CC	CC	CC	CC	AC	CC	AC
	AC	CC	CC	CC	CC	CC	CC	CC
	CC	CC	CC	CC	CC	CC	CC	CC
	CC	CC	CC	CC	AC	AC	CC	
85835108								
4392	rs10114841		9	AA	AA	AA	AA	AA
	AA	AA	AA	AA	AA	AA	AA	AA
	AA	AA	AA	AA	AA	AA	AA	AA
	AA	AA	AA	AA	AA	AA	AA	AA
	AA	AA	AA	AA	AA	AA	AA	
72299527								
4393	rs10114863		9	GG	GG	GG	GG	AG
	AG	GG	GG	GG	GG	GG	GG	GG

GG GG GG GG GG GG GG GG
GG GG GG GG GG GG GG GG
GG GG GG GG GG GG GG
99509624
4394 rs10114872 9 GG AG GG GG GG
GG GG GG GG GG GG GG GG
GG GG AG GG GG GG GG GG
AG GG AG GG GG GG GG GG
AA AG AG AA AG GG AG
71574757
4395 rs10114882 9 AA AA AA GG GG
GG GG AA AA AA AG GG AG
AA AA AG AG AG AG AG AG
AG AA AG AA AG AG AG AG
AA AG AG AA GG AG AG
128303425
4396 rs1011489 16 AG GG AG AA AA
AA AA GG AG GG GG GG GG
AA AG AA AG AA AA AA AA
AG AA AA AG GG AA AG AG
GG AG AA AG AA AA AA
5667393
4397 rs10114890 9 AA AA AA AA AA
AA AA AC AC AC AC AC CC
CC AA AA AC AA AC AC AA
AC AC AC AC AA AC AA AA
AA CC AA AC AC AC AC
102314775
4398 rs10114899 9 AA AA AA AA AA
AA AA AA AA AA AA AA AA
AA AA AA AA AA AA AA AA
AA AA AA AA AA AA AA AA
AA AA AA AA AA AA AA
24959557
4399 rs10114901 9 AA AA AA AA AA
AA AA AA AA AA AA AA AA
AA AA AA AA AA AA AA AA
AA AA AA AA AA AA AA AA
AA AA AA AA AA AA AA
81645417
4400 rs10114937 9 AA AA AA AA AA
AA AA AA AA AG AA AA AG
AG AG AA AG AG AG AG GG
GG AG GG AG AA AA AA AA
AG AG AA AA AG AA AG
35271817
4401 rs10114966 9 AA AA AA AA AA
AA AA AA AA AA GG GG GG
GG GG AA AA AA AA AG AA
AG AA AG AA AA AA AA AA

AG GG AG GG AG GG AG
29679114
4402 rs10115026 9 AA AG AA GG GG
GG AG AA AA -- AA AG AG
AA AA AG AG AG AG AG AG
AG AG AG AG AA GG AG AG
AG -- AA AA AG AG AA
129345624
4403 rs10115085 9 AA AA AA AA AG
AG AA AG AG AG AG AG GG
GG GG AA AA AG AG AG GG
AG AA AG AA AG AA AA AA
AA AG AA AG AA AA AG
108663489
4404 rs10115098 9 AC CC AC CC AA
AC AC CC CC CC CC CC CC
CC CC AC AC AC CC AC CC
AC AC AC AC CC CC CC CC
AA CC CC CC AC CC AC
98358683
4405 rs10115130 9 AA AA AA AA AA
AA AA AA AA AA AA AA AA
AA AA AA AA AA AA AA AA
AA AA AA AA AA AA AA AA
AA AA AA AA AA AA AA
88950903
4406 rs10115153 9 AA AA AA AC AA
AC AA AC AA AA AA AA AA
AC AA AC AA AA AA AA AA
AA AA AA AA AA AA AA AA
AC AA AA AA AA AA AA
2466509
4407 rs10115200 9 AA AA AA AA AA
AA AA AA AA AA AA AA AA
AA AA AA AA AA AG AG AG
AA AA AA AA AA AA AA AA
AA AA AA AA AA AA AA
84066010
4408 rs10115264 9 AG GG AG GG GG
GG GG GG AA GG AG GG AG
AG GG AA AA AA AG AG AA
GG GG GG GG GG AA AG AG
GG AA AG AA GG GG GG
84140380
4409 rs1011535 20 AC AC AA AA AA
AA AA AA AA AA AA AA AA
AA AA AA AC AC AA AC AA
AA AA AA AA AA AA AA AA
AA CC AA AC AA AA AA
43793512

4410 rs1011539 7 GG GG GG GG GG
GG GG GG GG GG AG GG AG
AG GG GG GG GG GG GG GG
GG AG GG AG GG AG AG AG
GG GG GG GG AG AG GG
14294598
4411 rs10115421 9 AC CC AC AC CC
AC CC CC AC AC AA AC AA
AA AA AA AA AA AA AA AC
AC AC AC AC CC CC CC CC
AC AA CC AC AC AC AC
89738445
4412 rs10115467 9 AG AA AG AA AG
AG AA GG GG GG AG AG GG
GG GG AG GG AG AG GG AG
AG GG AG GG GG GG GG GG
AA AG AG GG GG AG GG
5311171
4413 rs10115526 9 CC CC CC CC AC
AC CC CC AC CC AC CC CC
CC AC CC CC CC AC CC CC
CC AC AC AA CC CC CC CC
CC CC AC CC AC AA CC
88914554
4414 rs10115576 9 GG GG GG GG GG
GG GG AA AA GG GG GG GG
GG AG GG GG GG GG GG GG
GG AG AG AG GG AG AG AG
GG GG GG GG GG AG GG
115617822
4415 rs1011559 7 AA AA AA AA AC
AA AC AA AA AA AA AA AA
AA AA AA AA AA AA AA AA
AA AC AC AA AC AA AA AC
AC AC AA AA AA AA AA
20703948
4416 rs1011565 18 AG AG AG AG GG
GG AG GG AG GG GG GG GG
GG GG GG GG GG GG GG GG
GG GG GG GG GG GG GG GG
AG GG GG GG AG AG GG
53186254
4417 rs10115653 9 AG GG AG GG GG
GG GG AG AG AA AA AG AA
AA AG AG AG AG GG GG GG
AA AG AA AG AG AA AA AG
GG AG AA AA AA AA AG
37768742
4418 rs10115703 9 GG GG GG GG GG
GG GG GG GG GG GG GG GG

	GG	GG	GG	GG	AG	GG	GG	GG
	GG	AG	GG	AA	GG	GG	GG	GG
	AG	AA	GG	AG	GG	GG	GG	
14722616								
4419	rs10115707		9	GG	GG	GG	GG	GG
	GG	GG	GG	GG	GG	AG	GG	GG
	GG	GG	GG	GG	GG	GG	GG	GG
	GG	GG	GG	GG	GG	GG	GG	GG
	GG	GG	GG	GG	GG	GG	GG	
7169311								
4420	rs10115735		9	AA	AC	AA	CC	CC
	CC	CC	CC	CC	CC	CC	CC	CC
	CC	CC	CC	AC	AC	CC	CC	CC
	CC	AC	CC	AC	CC	CC	CC	CC
	CC	CC	AC	AC	CC	AC	CC	
78900161								
4421	rs1011575		2	GG	GG	GG	GG	GG
	GG	GG	AG	GG	AG	GG	AG	AG
	GG	GG	GG	GG	GG	AG	GG	GG
	AG	AG	GG	AG	GG	GG	GG	GG
	GG	GG	AG	AG	AG	GG	AG	
12617507								
4422	rs10115769		9	AG	AG	AG	GG	GG
	GG	GG	AG	AG	GG	GG	GG	GG
	GG	AG	GG	GG	GG	GG	GG	GG
	GG	GG	GG	GG	GG	GG	GG	GG
	GG	GG	GG	GG	GG	AG	GG	
111396742								
4423	rs10115776		9	GG	GG	GG	GG	GG
	GG	GG	AG	GG	AG	AG	GG	AG
	AG	GG	GG	AG	GG	AG	GG	AG
	GG	GG	GG	GG	AG	AG	AA	AA
	GG	GG	GG	GG	AG	GG	AA	
126850913								
4424	rs10115790		9	GG	GG	GG	AG	GG
	AG	GG	AG	AG	GG	GG	AG	GG
	GG	GG	GG	GG	AG	AG	GG	AA
	GG	GG	GG	AG	GG	GG	GG	GG
	GG	GG	GG	GG	AG	AG	AG	
7345304								
4425	rs10115815		9	AA	AG	AA	AG	AG
	AA	AG	AA	AA	AA	AG	GG	AG
	AA	AA	AA	AG	AA	AA	AA	AA
	AG	AG	AG	AG	AG	AG	GG	AG
	AA	AA	AG	AG	AA	AG	AG	
132819947								
4426	rs10115948		9	AC	CC	AC	CC	AC
	AC	CC	CC	CC	AC	CC	CC	CC
	AC	AC	CC	CC	AC	CC	CC	AC
	AC	CC	AC	CC	CC	AA	AC	AC

CC CC CC CC AC AA CC
12163583
4427 rs1011595 20 AC AC CC AC AC
CC AA AC AC CC AA AA AA
AA AC AA AA AC CC AC AC
AC AC AC AC AC CC AC AC
AA AC AA AA AC CC AC
45055061
4428 rs10115985 9 AA AA AA AA AA
AA AA AA AA AA AA AG AA
AA AA AA AA AA AA AA AG
AA AA AA AA AA AG AG AG
AA AA AA AA AA AA AG
24823390
4429 rs10115989 9 AG AA AG AG AA
AA AG AA AA AA AA AA AA
AA AA AG AG AA AA AG AA
AA AA AA AA AG AA AG AA
AA AG AG AG AG AG GG
90641936
4430 rs10116017 9 AG AG AG AG AG
GG AG GG GG GG GG GG GG
GG AG AG GG GG GG GG GG
AG AG AG AG AG AG AA AA
GG AG AG GG GG GG GG
114837337
4431 rs1011609 3 AG AG AG GG AG
GG AG GG GG GG GG GG GG
GG GG GG GG GG AG AG AA
GG AG GG AG GG AG GG GG
AG AG AA AA AG AG AG
87401121
4432 rs10116105
9 -- -- -- -- -- -- -- --
-- -- -- -- -- -- -- --
-- -- -- -- -- -- -- --
-- -- -- -- -- -- -- --
-- -- -- -- 81438539
4433 rs10116204 9 GG GG GG GG GG
GG GG GG GG GG GG GG GG
GG GG GG GG GG GG GG GG
GG GG GG GG GG GG GG GG
GG GG GG GG GG GG GG
74052978
4434 rs10116267 9 AG AA AG AG AA
AA AG AG AA GG GG AG AG
AG AG AG AG AG AA AA AG
AG AG AG AG AA GG AG AG
AA GG AG GG AA AA AA
80921732

4435 rs10116275 9 AG GG AG AA AA
AA AA AA AG AA AG AA AA
AA AG AA AA AA AA AA AA
AG AA AA AA AG AG AG AA
AA AA AA AA AA AA AA
138755777
4436 rs10116287 9 AC AC AC CC AA
AC AC AA AA AA AC AA AA
AA AC AC AA AC AA AA AC
AA AA AA AA AC AA AC AA
AC AC AC CC AA AA AA
87477274
4437 rs1011631 4 AA AG GG AG AG
GG AA AG AA AA AA AA AG
AG AG GG GG GG GG GG GG
AA AG AG AG AG AG GG AA
GG GG AG AG AG GG AG
82440730
4438 rs10116340 9 GG GG GG GG GG
GG GG AG AG GG GG GG GG
GG GG GG GG GG AG AG AG
AG GG AG GG GG GG GG GG
AG GG GG GG GG GG GG
79477693
4439 rs10116353 9 CC CC CC CC CC
CC CC AC AC CC AC CC AC
AC CC AC AA AA CC CC CC
AC CC AC CC AC AC CC CC
AA CC AC CC AA AC AC
111950088
4440 rs10116388 9 AA AC AA AA AC
AC AA AA AA AA AA AA AA
AA AA AC AC AC CC AC AC
AA AA AA AA AA AC AA AA
AA AA CC AC CC AC AC
129061773
4441 rs1011642 22 GG AG AG GG AG
AG GG GG AG GG GG GG GG
GG GG GG AG GG GG GG GG
AG GG AG GG GG GG GG GG
GG AG GG AG GG GG GG
49399524
4442 rs1011646 2 AA AA AA AA AA
AA AA AA AA AA AA AA AA
AA AA AA AA AA AA AA AA
AA AA AA AA AA AA AA AA
AA AA AA AA AA AA AA
1668867
4443 rs10116548 9 AG AG AA GG AG
AG GG AG AG AG AA GG AG

	AG	AG	GG	GG	GG	AG	GG	AG
	GG	GG	GG	AG	GG	GG	GG	GG
	GG	GG	GG	GG	AG	AG	GG	
12234642								
4444	rs10116554		9	AG	AG	AG	AA	GG
	AG	AG	AA	AA	AA	AG	GG	AG
	AG	GG	GG	GG	GG	AG	AA	AA
	AG	AA	AG	AA	AA	GG	AG	AG
	AG	AA	AG	AA	AG	AG	AG	
92489679								
4445	rs1011656		12	GG	GG	GG	GG	GG
	GG	GG	GG	AG	AG	GG	GG	GG
	AG	AG	GG	GG	GG	GG	GG	AG
	GG	GG	GG	GG	GG	GG	GG	GG
	GG	GG	GG	GG	GG	GG	GG	
25157453								
4446	rs10116577		9	AA	AA	AA	AG	AG
	AG	AA	AA	AA	AA	AA	AA	AA
	AA	AA	AA	AA	AA	AG	AG	AG
	AA	AG	AA	AG	AA	AA	AA	AA
	AA	AA	AA	AA	AA	AA	AA	
29281634								
4447	rs1011659		6	GG	AG	AG	AG	AA
	AA	AG	AG	GG	AG	AG	AG	AA
	AG	AA	AG	AG	AA	AG	AG	GG
	AG	AG	GG	AG	GG	AA	AG	AG
	AA	GG	AG	AG	AG	AG	AG	
106091250								
4448	rs10116611		9	GG	GG	GG	GG	GG
	GG	GG	AG	AG	GG	GG	GG	GG
	GG	GG	GG	GG	GG	AG	AG	GG
	AG	GG	AA	GG	AG	GG	GG	GG
	AG	AG	AG	AA	GG	AG	AG	
121452775								
4449	rs10116618		9	GG	GG	GG	GG	GG
	AG	AG	AG	AG	GG	GG	GG	GG
	GG	GG	GG	GG	GG	AG	GG	AA
	AG	GG	AG	GG	GG	AG	GG	GG
	AG	GG	AG	GG	GG	GG	AG	
38769003								
4450	rs10116650		9	AG	AG	GG	AG	GG
	GG	AG	GG	GG	GG	GG	GG	GG
	GG	GG	AG	GG	GG	AG	AG	AG
	GG	GG	GG	AG	AG	GG	GG	AG
	AA	GG	GG	GG	GG	AG	GG	
8397775								
4451	rs10116662		9	GG	GG	GG	GG	GG
	GG	AG	GG	GG	GG	AA	AG	AA
	AA	AA	AG	GG	GG	GG	GG	GG
	GG	GG	GG	GG	GG	AG	GG	GG

GG GG GG GG GG GG GG
78163305
4452 rs1011669
18 -- -- -- -- -- -- -- --
-- -- -- -- -- -- -- --
-- -- -- -- -- -- -- --
-- -- -- -- -- -- -- --
-- -- -- -- 23198525
4453 rs10116708 9 AA AA AA AA AA
AA AA AA AA AA AA AA AA
AA AA AA AA AA AA AA AA
AA AA AA AA AA AA AA AA
AA AA AA AA AA AA AA
140376668
4454 rs10116739 9 AC AC AA AC AC
AA CC AA AC AC AA AC AC
AC AC AA AA AA AA AC AC
CC CC CC AC AA AC AC AC
AC AA AC AA AC AC AC
24771927
4455 rs10116746 9 GG GG GG AG GG
GG AG GG AG AG GG GG GG
GG AG AG AG AG GG AG AG
GG GG GG GG GG GG GG GG
GG AG AG AG GG GG AG
96795797
4456 rs10116800 9 AG AG AG AG AA
AG AA AA AA AG AG AG GG
GG AG AG AG AG GG GG AG
AG AA AG AA AG AG GG AG
AA AG AG GG AG AG AG
79034262
4457 rs10116869 9 CC CC CC CC CC
CC CC CC CC AA CC AC AC
AC CC CC CC CC AC AC AC
CC CC CC CC CC AC AC AC
CC AC AC AA CC CC AC
111963366
4458 rs10116883 9 GG GG GG GG GG
GG GG GG GG GG AG GG AG
AG AG GG GG GG GG GG GG
GG GG GG GG GG GG GG GG
GG GG GG GG GG GG GG
1836746
4459 rs10116897 9 AA AA AA AA AA
AA AA AG AG AA AA AA AA
AA AG AA AA AA AA AA AA
AA AG AG AG AA AG AG AG
AA AA AA AA AA AG AA
115633479

4460 rs10116976 9 GG GG GG GG AG
GG AG GG GG AG GG AA AG
AG GG AG AA AG AG AG AG
AG AG AG AG AG GG GG GG
AG AG AG GG AA AA AG
100739888
4461 rs10117035 9 GG AG GG AG GG
AG AG AG GG AG GG GG GG
AG AG AG GG GG AG AG GG
GG GG GG GG AG GG GG GG
GG AG AG AG AA AG AG
121796354
4462 rs10117170 9 AC AC AA AC AA
AC AC AA AA AC CC AA AC
AA AA AC AA AA CC AA AC
AA AA AA AA AA AA AA AA
AA AA AC AC AA AC AA
9898870
4463 rs10117193 9 GG GG GG GG GG
GG AG GG GG AG GG GG GG
GG AG GG GG GG GG GG GG
AG AG AG AG GG GG GG GG
AG GG AG GG GG GG GG
116433501
4464 rs10117251 9 AA AG AA GG AG
GG AG AG AG AG GG AA AG
AG AG GG AG AG AG AG AG
AG AG GG GG AG AG AG GG
AG GG AG AG AA AG AA
85909882
4465 rs1011731 1 GG GG GG AA AG
AA AG AG AA AG AG AG AA
AA AG AG AG AG AA AG AA
GG AG GG AG AG GG AG GG
AG AA AA AA GG AG GG
172346548
4466 rs10117367 9 CC CC CC CC CC
CC CC CC CC CC CC CC CC
CC CC CC CC CC CC CC CC
CC CC CC CC CC CC CC CC
CC CC CC CC CC CC CC
98567362
4467 rs10117516 9 GG GG GG AG AG
GG AG GG GG AG AA AG AA
AG AG AG AG AA AA AG AG
AG AG AG AA GG GG GG GG
AA AG GG AG GG AG GG
14749434
4468 rs10117522 9 GG AG AG AG AG
AG GG AG AG AA GG AG GG

GG GG AG AG AG GG GG GG
GG GG GG GG AG GG AG GG
GG AA GG AG AG AG GG
26719332
4469 rs10117534 9 AA AA AA GG GG
GG GG AA AA AA AG GG AG
AG AA AG GG GG AA AA AG
AG GG AG GG AG AG AA AG
AG AG AA AG AA AG AG
36709347
4470 rs10117560 9 AA AG AG AG AG
AG AG AG AG AA AA AG AG
AG AG AG AA AG AA AG AA
AG GG AG GG AG AA AA AG
AG AG AA AG AA AA AA
13930857
4471 rs1011757 3 AG AG GG AA AA
AA AA AA AG AG AA AA AA
AG AA GG GG AG AA AG AA
AA AA AG AA AA AA AA AA
AA AA AG AG AA AA AG
140022990
4472 rs10117574 9 AA AA AA AA AA
AA AA AA AA AA AA AA AA
AA AA AA AA AA AA AA AA
AA AA AA AA AA AA AA AA
AA AA AA AA AA AA AA
33380702
4473 rs10117618 9 GG AG AG GG AA
AG AG AA AA AG AG AA AG
GG AG AG AG GG AG GG AG
AG AA AG AG AG AG AA GG
AG GG AG AG GG GG AG
6750556
4474 rs10117679 9 GG GG GG GG GG
GG GG GG GG GG GG GG GG
GG GG GG GG GG GG GG GG
AG AG AG AG GG GG GG GG
GG GG GG GG GG GG GG
104378479
4475 rs10117752 9 AG GG AG AG AG
AG GG AG AG GG AG GG GG
GG GG GG AG AG AA AG AG
AG GG AG GG GG AG AG AG
AG AG AG AG GG GG AG
25658015
4476 rs10117759 9 GG AG AA AG AG
AA GG AA AA AA GG GG GG
GG AG AA AG AA AG AA AG
AG AG AG AG AG AG GG AG

AG AG GG GG AG AG AG
134428539
4477 rs10117770 9 AA AA AA AA AA
AA AA AG AA AG AA AG AG
AG AG GG GG GG AA AA AA
AA AA AA AA AA AA AA AA
AA AA AA AA AA AA AA
82086682
4478 rs1011780 7 AC AC AC AA AC
AC AC AA AA AA AC AA AC
AC AA AA AA AA AA AA AA
AA AA AA AA AC AA AA AC
AA AA AA AA AA AA AA
70124648
4479 rs10117805 9 GG GG GG GG AA
AG AG GG AG GG AG AG GG
GG AG GG GG GG GG GG GG
GG GG GG GG AG AG GG AG
GG GG AA AG GG GG GG
88242953
4480 rs10117817 9 GG GG GG GG GG
GG GG GG GG GG AG GG AG
AG GG GG GG GG GG GG GG
GG GG GG GG GG GG GG GG
GG GG AG GG GG GG GG
85855600
4481 rs10117819 9 AG AG AG AG GG
AG GG GG AG AG AG AG AA
AA AG AG AG GG AA AG AG
AG AA AA AA AA AA AA AA
AA AG AG AG GG GG GG
89193482
4482 rs10117822 9 GG AG AG GG AG
AG GG AG AG AG AA AA AG
AG AA AA AA AA AG GG AG
AA AG AG AG GG AG GG GG
AG AG GG AG AG AG AA
139602652
4483 rs10117870 9 GG GG GG GG GG
GG GG GG AG AG AG GG GG
GG AG AG GG AG AG GG GG
GG GG GG GG AA GG AG AG
GG AA GG AG AG GG AG
89646140
4484 rs10117909 9 GG AG AG AA AA
AA AA AG GG AG AA AA AA
AA AA AA AA AA AG AG AA
GG AG GG AA AG AG AA AG
AA AA AA AA AA AA AA
19492973

4485 rs10117920 9 GG AG AG AG AG
AG AG AG AG AG GG GG GG
GG GG GG GG GG GG GG GG
AG AG AG GG GG GG GG GG
GG AG GG AG GG GG GG
13864265
4486 rs10117941 9 GG GG GG GG GG
GG GG GG GG GG GG GG GG
GG GG GG GG GG GG GG GG
GG GG GG GG GG GG GG GG
GG GG GG GG GG GG GG
99340974
4487 rs1011797 2 GG GG GG GG AA
AG AG AG AG GG AG AG AG
AG AG AG GG AG GG GG GG
AG AG AG AG GG GG GG GG
AG GG AG AG GG GG AG
44757217
4488 rs10117983 9 AA AA AA AG AA
AG AA AA AA AA AG AA AG
AG AG AA AA AA AA AA AA
AA AA AA AG AG AA AA AG
AA AG AA AA AA AA AA
1815727
4489 rs10117990 9 CC AC AC CC CC
AC CC AA AA AC CC CC CC
CC CC AC CC CC CC CC CC
AC AC AC AC CC AC CC CC
CC CC CC CC AC CC AC
30555391
4490 rs10118058 9 GG AG GG AG AG
AG AG AG AG GG AA GG AG
AG AG AG AG AG AA AA AA
AG GG AG GG AA AA AA AA
AA AG AA AA GG AG AG
81589458
4491 rs10118130 9 AA AA AA AA AA
AA AG AA AA AG AG AA AG
AG AG AG GG GG AG AG AG
AG AA AG AA AA AG AA AA
GG AG AA AA AA AA AA
35250666
4492 rs10118161 9 GG GG GG AG AG
AG AG GG GG GG AG GG AG
AG GG GG GG GG GG GG GG
GG GG GG GG GG GG GG GG
AA GG GG GG GG GG GG
79763338
4493 rs10118162 9 AA AA AA GG GG
AG AG GG AG AA AG AA AA

AA AA AA AA AA AG AA AA
AA AA AA AA AA AG AA AA
AG AG AG AG AA AA AA
26572537
4494 rs10118215 9 GG GG GG GG GG
GG GG AG GG AG GG GG GG
GG GG GG GG GG AG AG AG
GG GG GG GG AG GG GG AG
GG GG AA AG GG GG GG
83589051
4495 rs10118217 9 CC CC CC CC CC
CC CC CC CC CC CC CC CC
CC CC CC CC CC AC AC AC
CC CC CC CC CC CC CC CC
CC CC CC CC CC AC CC
83707208
4496 rs10118224 9 GG GG GG GG AG
AG GG AG AG AG GG AG GG
GG GG GG AG AG GG GG AG
GG GG GG GG GG GG GG GG
GG AG GG GG GG GG GG
77545238
4497 rs10118264 9 AA AA AC AA AA
AA AA AA AA AA AA AA AA
AA AA AC AC CC AA AA AA
AA AA AA AA AA AA AA AA
AA AA AA AA AA AA AA
118484799
4498 rs10118278 9 AG AG AG GG GG
GG AG AG GG AA AA AG AG
AG GG AG GG GG AG AG GG
AA AG AA AG AG AA AA AA
AG AA AG AA AA AG AG
104801485
4499 rs10118280 9 AA AA AA AA AA
AA AA AA AA AA AA AA AA
AA AG AG AA AA AG AA AG
AA AA AA AA AA AA AA AA
AA AG AA AA AA AA AA
74021451
4500 rs10118286 9 AG AG AG GG AG
AG AG AG AG GG GG AG AG
AG AG AG GG GG AG AG AG
GG AG GG AG GG AG AG AG
AG AG AG AG GG GG GG
78212566
4501 rs10118309 9 AA AA AA AA AA
AA AA AA AG AG AA AA AG
AG AG AG AA AA AG AG AA
AA AA AA AA AA AA AA AA

AA AA AA AA AA AA AA
18767830
4502 rs10118347 9 GG AG AG AG AG
AA AG GG GG AG AG AG GG
GG GG GG GG GG AG AG GG
AG AG AG AG AG GG GG AG
GG GG GG GG AG AG GG
32870406
4503 rs10118363 9 AC AC AC CC CC
CC CC CC AC CC CC CC CC
CC CC AA AA AA AA AA AC
CC AC AC AC AC AC CC AC
CC AC AC AC AC AC AC
92339924
4504 rs10118365 9 AA AA AA AC AC
-- AC AC AC AA AC AC AA
AA AC AC -- -- AA AA AC
AA AC AA AC AA AA AA AA
AC AC AC AA AA AA AA
129781126
4505 rs10118366 9 AA AA AA AA AA
AA AA AA AA AA AA AA AA
AA AA AA AA AA AA AA AA
AA AA AA AA AA AG AA AA
AA AA AA AA AA AA AA
28563341
4506 rs10118394 9 AG GG AG AG AG
AA AG AG AG GG AG AA AA
AA AG AG GG GG AG AG AG
AG GG AG GG AG AG GG AG
GG AA GG AG GG AG AG
36463744
4507 rs10118516 9 AC AC CC CC CC
CC CC CC AC AC CC CC CC
CC CC CC CC CC CC CC CC
CC CC CC AC CC CC CC CC
CC AC CC CC CC AC CC
22613796
4508 rs1011857 8 GG AG GG GG GG
GG AG GG AG AA GG AG GG
GG GG AG AG GG GG GG GG
AG GG GG GG GG AA AG AG
GG GG GG GG AG AG GG
65137604
4509 rs10118699 9 AA AA AA AA AA
AA AA AA AA AA AA AA AA
AA AG AA AA AA AA AA AA
AA AA AA AA AA AA AA AA
AA AA AA AA AA AA AA
121409332

4510 rs10118705 9 GG GG GG GG AG
AG GG GG GG AG GG GG GG
GG AG AG AA AG GG GG GG
GG GG GG GG GG GG GG GG
GG AG AG AG AG GG AG
111528780
4511 rs10118736 9 GG AG AG GG GG
GG AG GG GG GG GG GG GG
GG GG GG GG GG GG GG AG
AG AG AA AA GG GG GG GG
GG GG GG GG GG GG AG
139633733
4512 rs1011875 20 CC AC CC AC CC
CC AC AC AA AA AC AC CC
AC AC AA AA AA AC AA AA
AA AA AA AA AC AA AC AA
AA CC AC AC AA AA AA
8778089
4513 rs10118760 9 AA AA AA AA CC
AC AC AA AA AA AC AC AA
AA AC AA AC AC CC CC CC
AC AC CC CC AC CC CC AC
CC AA AC AA AC CC AC
88854546
4514 rs10118806 9 AC AC CC AC AA
AC AA AA AC AC AA AC AC
AC AC AC AA AA AC AC CC
AA AA AC AA AC AC CC CC
AC CC AC CC AC AC AC
118615701
4515 rs10118875 9 AA AA AA AA AA
AA AA AA AA AA AA AA AA
AA AA AA AA AA AA AA AA
AA AA AA AA AA AA AA AA
AA AA AA AA AA AA AA
99295966
4516 rs10118886 9 AC AC AC CC CC
CC CC AC AC AC AC AC AA
AA CC CC CC CC CC AC CC
CC AC CC AC AC CC AC AC
CC CC CC CC CC CC CC
99798293
4517 rs10119016 9 GG GG GG GG AG
AG GG GG GG GG GG GG GG
GG GG GG GG GG GG GG GG
GG GG GG GG GG GG GG GG
GG GG GG GG GG GG GG
99849281
4518 rs1011903 1 GG GG GG GG GG
GG GG AG GG AG GG GG GG

GG GG GG AG GG GG GG GG
GG GG GG GG GG GG GG GG
GG GG GG GG GG GG AG
94143719
4519 rs1011910 6 AC CC AC AA AA
AA AA CC AC CC CC CC CC
AC AA CC CC AC AC AC AC
AC AC AC AC AC AA AC AC
AC AC AA AA AA AC AC
114417219
4520 rs10119113 9 CC CC CC CC CC
CC CC AC AC AC CC AC AC
AC AC CC AC CC AC AC AC
CC CC CC CC CC CC CC CC
AC AC AC CC CC CC CC
12765117
4521 rs10119218 9 AA AA AA AG AA
AG AA AG GG AG GG AA AG
AG AG AG AG GG AG AG AG
AA AG AA GG AG AG AG GG
AG GG AG AG GG AG GG
83519579
4522 rs10119277 9 GG AG AG GG GG
GG GG GG GG GG GG GG GG
GG GG GG GG GG GG GG GG
GG GG GG GG GG GG GG GG
GG GG GG GG GG GG GG
10130546
4523 rs10119355 9 AG AG AA AA AA
AA AA AA AA AA AG AA AA
AA AA AA AG AA AA AG AA
AA AG AA AG AG AA AG AA
AG AA GG AG AA AA AA
4183453
4524 rs10119371 9 GG AG GG AG AG
AA AG AG AG AG AA AA AA
AG AG AG AG GG GG AG AG
AA AA AA AA AG AA AG AG
AA AG AA AA AG AG AA
129528628
4525 rs10119380 9 AG AG GG GG GG
GG AG GG GG GG GG GG GG
GG GG GG GG GG GG GG GG
GG AG AG AG GG GG GG GG
GG GG GG GG AG GG AA
137837317
4526 rs10119442 9 AA AG AA AA AA
AA AA AA AA AA AA AA AA
AA AA AA AA AA AG AG AA
AA AA AA AA AA AA AA AA

AA AA AA AA AG AG AA
85314307
4527 rs10119492 9 GG GG GG GG GG
GG GG GG GG GG AA GG AG
AG AG GG AG AG GG AG GG
GG GG GG AG GG GG GG GG
AG GG AG AG GG GG GG
1413700
4528 rs1011961 4 GG GG GG AG GG
AG AG GG GG GG AA AA AA
AG GG GG GG GG GG GG GG
GG AG AG AG GG GG GG GG
GG AG AG AG GG GG GG
95810540
4529 rs10119610 9 AA AA AA AA AA
AA AA AA AA AA AA AA AA
AA AA AA AA AA AA AA AA
AA AA AA AA AA AA AA AA
AA AA AA AA AA AA AA
129359384
4530 rs1011967 7 GG GG GG GG GG
GG GG GG GG GG GG GG GG
GG GG GG GG AG GG GG GG
GG GG GG GG GG GG GG GG
GG GG AG AG GG GG GG
25290055
4531 rs10119678 9 CC CC CC CC CC
CC CC AC AC CC AA AC AC
AC AC AC AC AC AC CC AC
CC AC CC AC CC CC CC CC
CC CC CC CC CC CC CC
21340786
4532 rs1011970 9 CC CC CC AC AC
AC AC CC CC CC CC AC CC
CC AC CC CC CC CC CC CC
CC CC CC CC AC AC AC CC
CC CC AA AC CC AC CC
22062134
4533 rs10119719 9 CC CC CC CC CC
CC CC CC CC CC CC CC CC
CC CC CC CC CC CC CC CC
CC CC CC CC CC CC CC CC
CC CC CC CC CC CC CC
76277900
4534 rs10119795 9 GG AG GG GG AG
AG GG GG AG GG AA GG AG
AG AG GG GG AG AG AG GG
GG GG GG GG GG GG GG GG
AG GG AG GG GG GG GG
100637339

4535	rs10119818	9	AA	AA	AG	AA	AA
AA	AA	AA	AA	AA	AA	AA	AA
AA	AA	AG	AA	AG	AA	AA	AA
AA	AA	AA	AA	AA	AA	AA	AA
AA	AA	AA	AA	AA	AA	AA	
133021887							
4536	rs10119859	9	GG	GG	GG	GG	GG
GG	GG	GG	GG	GG	GG	GG	GG
GG	GG	GG	GG	GG	GG	GG	GG
GG	GG	GG	GG	GG	GG	GG	GG
GG	GG	GG	GG	GG	GG	GG	
74594399							
4537	rs10119867	9	AC	AA	AC	CC	CC
CC	CC	AA	CC	AA	AC	CC	AC
AC	CC	CC	CC	CC	CC	CC	CC
AC	CC	AC	CC	AC	AC	AC	CC
AA	CC	AC	AC	CC	CC	AC	
83941281							
4538	rs10119909	9	AG	AA	AG	AG	AA
AG	AA	AA	AA	AA	AG	AA	AG
AG	AA	AA	AA	AA	GG	AG	AG
AA	AG	AG	AG	GG	AG	AG	AG
AG	AG	AA	AA	AA	AA	AA	
110649553							
4539	rs10119958	9	CC	CC	CC	CC	CC
CC	CC	AC	CC	AC	CC	CC	CC
CC	CC	CC	CC	CC	CC	CC	CC
AC	CC	CC	CC	CC	CC	CC	CC
CC	AC	CC	AC	CC	CC	CC	
89342374							
4540	rs10119990	9	AG	AG	AG	AA	GG
AG	AG	AG	AG	GG	GG	GG	GG
GG	GG	GG	AG	AG	AG	AA	AG
AA	AA	AA	AA	AG	GG	GG	GG
AG	AA	GG	AG	GG	GG	AG	
96534118							
4541	rs10120023	9	GG	AG	AA	GG	GG
GG	AG	AG	AG	GG	GG	GG	GG
GG	GG	GG	GG	GG	AA	AG	AA
GG	AG	AG	AG	AG	AG	AA	AG
AG	AG	AG	AG	GG	AG	GG	
137810259							
4542	rs10120039	9	AG	AG	AG	GG	GG
GG	AG	AG	AG	AG	AG	AG	GG
GG	AG	AG	AG	AG	GG	GG	GG
GG	GG	GG	GG	AA	AG	AG	AG
GG	AA	AA	AA	GG	GG	GG	
127406961							
4543	rs10120169	9	AA	AA	AA	AA	AA
AA	AA	AA	AA	AA	AA	AA	AA

AA AA AA AA AA AA AA AA
AA AA AA AA AA AA AA AA
AA AA AA AA AA AA AA
74561883
4544 rs10120190 9 GG GG GG GG GG
GG GG GG GG GG GG GG GG
GG GG GG GG GG GG GG AG
AG GG AG GG GG GG GG GG
GG GG GG GG GG GG GG
8517545
4545 rs10120219 9 GG AG GG GG AG
AG GG GG GG AG GG GG GG
GG GG AA AG AG GG GG GG
AG AG AG AG AG AG GG GG
GG GG GG GG GG GG GG
98364547
4546 rs10120239 9 AA AA AA AA AA
AA AA GG AA AG AA AG AA
AG AA AG AA AA AG AG AA
AA AA AA AA AA AG AA AA
AA GG AA AG GG AG AG
1417953
4547 rs10120248 9 AG AG AG GG GG
GG GG AA AA GG AG GG AG
AG GG AG AG AG AG AG GG
AA AG AA AG AG AG AG AG
GG GG GG GG AG AA AG
110857507
4548 rs10120252
9 -- -- -- -- -- -- -- --
-- -- -- -- -- -- -- --
-- -- -- -- -- -- -- --
-- -- -- -- -- -- -- --
-- -- -- -- 82645562
4549 rs10120358 9 AG AG AG AA AG
AG AA AA AA AA AA AA AA
AA AA AA AG AA AA AG AA
AA AA AA AA AA AA AA AA
AA AG AA AA AA AA AA
111650007
4550 rs1012036 7 GG GG GG GG AG
AG GG AG GG AA AA AA AA
AG AG GG GG AG GG AG GG
AG AG AG AG AG AG AA AG
AG GG GG GG AG AA GG
52472450
4551 rs1012040 19 AA AA AA AG AA
AG AG GG AG AG AA AA AA
AG AG AG GG AG GG AG AG
AA AA AA AA AG AA AA AG

AA AA AA AA AA AA AA
21672414
4552 rs10120416 9 AA AA AA AA AA
AA AA AA AA AA AA AA AA
AA AA AA AA AA AA AA AA
AA AA AA AA AA AA AA AA
AA AA AA AA AA AA AA
89398963
4553 rs1012042 21 AA AA AA AA AA
AA AA AA AA AA AG AG AA
AA AA AA AA AA GG AG AG
AA AA AA AA AA AA AA AA
AA AA AA AA AG AG AG
31319562
4554 rs10120424 9 GG GG GG GG GG
GG GG GG GG GG GG GG GG
GG GG GG GG GG GG GG GG
GG GG GG GG GG GG GG GG
GG GG GG GG GG GG GG
140559850
4555 rs1012049 6 AG AG GG GG AG
AG GG AA GG AA AG AG GG
GG GG GG GG GG GG AG AG
GG GG AG GG AG AG AA AG
GG GG GG GG AG AA AG
128530307
4556 rs10120508 9 AA AA AA AA AA
AA AA GG AG AA AA AG AG
AA AG AG AG AG AA AA AA
AG AA AG AA GG AA AG AG
GG AA AA AA AG AG GG
122202000
4557 rs10120562 9 AA AA AA AG AG
AG AG AA AA AA AA AA AA
AA AA AA AA AA AA AA AA
AA AA AA AA AA AA AA AA
AA AA AA AA AA AG AA
16793104
4558 rs1012066 1 GG AG GG GG GG
GG GG AG GG AG AG AG AA
AG AG AG AA AA AA AG AG
AG GG GG GG GG GG GG GG
AG AG GG GG GG GG GG
178964604
4559 rs1012068 22 AC AC AC AA AA
AA AA AA AA AA AA AA AA
AA AA CC AC AC AC AC AC
AC AC AC AA AA AA AA AA
AC AC AA AA AA AA AA
32265903

4560 rs10120700 9 AA AA AA AA AA
AA AC AA AA AC AC CC AC
AC AC AC CC AC AA AA AA
AA AC AA AC AC AC AC CC
AC AA AC AA CC AC AA
133548589
4561 rs10120776 9 AG AG AG AG GG
GG AG GG AG GG GG AG AG
AG AG AA AG AG AG AG GG
GG GG GG GG AG AG AG AA
GG GG GG GG GG GG GG
80210750
4562 rs1012078 2 AG AA AG AA AG
AG AA AA AA AA AA AA AA
AA AA AA AA AA AA AA AA
AA AA AA AA AA AA AA AA
AG AA AA AA AA AA AA
171422060
4563 rs10120788 9 GG GG GG GG AG
GG AG GG GG GG GG GG GG
AG GG AG AG GG AA GG AG
GG GG GG GG AG AA AG AA
AG AG AG AA GG GG GG
7231323
4564 rs10120911 9 GG GG GG GG GG
GG GG GG GG GG GG GG GG
GG GG GG GG GG GG GG GG
GG GG GG GG AG GG AG AG
GG GG GG GG GG GG GG
118001034
4565 rs1012092 5 AA AG AG AA AA
AA AA AA AA AA AA AA AG
AG AG AG AG AA GG GG GG
AG AG AG AG AA AG AG AA
AA AG AA AA AA AG AG
11176387
4566 rs10120928 9 GG AG AG GG GG
GG AG AG GG GG AA AG AG
AG GG AG AG AG GG GG GG
GG GG GG GG AG GG AG GG
GG AG AG AG GG GG AG
27253049
4567 rs10120959 9 AG AG GG AG AG
AG AG AG AA GG AG AG GG
AG GG AG GG GG GG GG GG
AA AA AA AA AG AG AG AG
AG AG AG AG GG GG GG
4240743
4568 rs10121020 9 GG GG GG GG GG
GG GG GG GG GG GG GG GG

GG GG GG GG GG GG GG GG
GG GG GG GG GG GG GG GG
GG GG GG GG GG GG GG
101946041
4569 rs10121026 9 GG GG GG GG GG
GG GG AG GG AG GG GG GG
GG GG AG AG GG GG GG GG
GG GG GG GG GG GG GG GG
GG AG GG AG AG GG AG
134497703
4570 rs1012105 11 GG AG AG AA AA
AG AA AA AG AG AA AA AG
GG GG AG AG AA AA AA AA
GG GG AG AG AA AA AA AA
AA AG AG AG AA AA AG
17769755
4571 rs1012107 7 AA AA AA AG AA
AA AG AG AG AA AG AG AA
AA AG GG AG AG AG AG GG
AG AG AG AG GG AG GG GG
AA AG AG AG GG GG GG
96943610
4572 rs10121097 9 GG GG GG GG GG
GG GG GG GG GG GG GG GG
GG GG GG GG GG GG GG GG
GG GG GG GG GG GG GG GG
GG GG GG GG GG GG GG
82405749
4573 rs1012120 1 CC CC CC CC CC
CC CC AC AC CC CC CC CC
CC CC CC CC CC CC CC CC
CC CC CC CC CC CC CC CC
CC AC CC AC CC CC AC
43606731
4574 rs10121215 9 AG AG GG AG AG
GG AA AG AG AG GG AG AG
AG AG AA AA AA AG AG GG
AG AA AG AA AA AA AA AA
AG GG AG GG AG AG AG
26169347
4575 rs10121375 9 AA AA AA AA AA
AA AA AA AA AA AA AA AA
AA AA AA AA AA AA AA AA
AA AA AA AA AA AA AA AA
AA AA AA AA AA AA AA
138070682
4576 rs10121376 9 CC CC CC CC AC
AC CC AA AC AA AC AA AC
AC AA AC AA AC AC AC AC
AA AA AA AA AC AA AA AC

AC AC AC CC AA AA AC
85634575
4577 rs10121424 9 GG GG GG AG AG
AA GG AG AG AG GG AG AG
AG AG GG GG GG GG GG AG
AG AA AG AA AG AG AG AG
AG AG AG AG AG AA GG
116800606
4578 rs1012145 14 CC CC CC AC CC
AC CC AC AC CC AC AC AC
CC CC AA AC AC AC CC AA
AC CC AC AC CC CC CC CC
CC CC AC AC CC CC CC
63940082
4579 rs1012152 7 AA AA AA AA AA
AA AA AA AA AA AG AG AA
AA AA AA AA AA AA AA AA
AA AA AA AA AG AA AG AA
AA AA AA AA AA AA AA
25450086
4580 rs1012154 12 AA AA AA AA AA
AA AA AG AA AG AG AA AA
AA AA AA AA AA AA AA AG
AG AG AG AG AA AG AG AG
AG AA AA AA AG AG AA
31627584
4581 rs10121557 9 AA AA AG AA AA
AA AG AA AG AA AA AG AG
AG GG AA AG AG AA AG AA
AG AG AG AG AG AG AG AG
AG GG AG AG AG GG AA
127654617
4582 rs10121600 9 GG GG GG GG GG
GG GG AG GG AG GG AG GG
GG AG GG GG GG GG GG GG
AG AG AG AG AG AG AA AA
AA AG AA AA GG GG AG
104378003
4583 rs10121603 9 GG GG GG GG GG
GG GG GG GG GG GG GG GG
GG GG GG GG GG GG GG GG
GG AG GG AG GG GG GG GG
GG GG GG GG GG GG GG
128650665
4584 rs10121611 9 AA AA AA AA AA
AA AA AA AA AA AA AA AA
AA AA AA AA AA AA AA AA
AA AA AA AA AA AA AA AA
AA AA AA AA AA AA AA
83973239

4585 rs10121676 9 GG GG GG GG AG
GG AG GG GG GG AG GG AG
AG GG AA AG AG GG GG GG
GG GG GG GG GG AG GG GG
AG GG GG GG GG GG GG
99260247
4586 rs1012169 12 CC AC AC AC AC
CC AA CC AC AC AA AC AC
AC AC AC AC AC AC AC AA
AC AC AA AC AC AA AA AC
AC AC CC AC CC AC AC
13382921
4587 rs1012173 5 AG AA AG AG AG
AG AG AG AG AG AG AG AA
AG AG AG GG AG AA AG AA
AA AA AA AA AA AG AG AG
AA AA AG AG AG AG AA
149713921
4588 rs1012176 5 AC CC CC CC CC
CC CC AC CC AC CC CC CC
AC AC AC CC AC CC CC CC
CC CC CC CC AC AA AC AC
AA CC CC CC AC AC AC
11320538
4589 rs10121803 9 GG GG GG GG GG
-- GG GG GG GG GG GG GG
GG GG GG GG GG GG GG GG
GG GG GG GG GG GG GG GG
-- GG GG GG GG GG GG
127329424
4590 rs10121882 9 AG AG AG GG GG
GG GG AG AG AG AA AG AA
AA AA AA AG AG AA AA AG
GG AG AG AA GG GG GG GG
AG GG GG GG GG AG AG
86330583
4591 rs10121888 9 GG GG GG GG GG
GG GG GG GG GG GG GG GG
GG GG GG GG GG GG GG GG
GG GG GG GG AG GG GG AG
GG GG GG GG GG GG GG
6314179
4592 rs10121915 9 GG AG AG AA AG
AG AG AG AA AG GG AG AG
GG GG GG GG GG AG AG GG
GG AG GG AG AG AA AA AA
GG GG AG AG GG GG GG
129447032
4593 rs1012196 21 GG GG GG AG AA
AG AA AA AA AG AG GG AG

AA AA AG AG AG AA AG AA
AA AG AG AG AA AA AA AA
AG AA AG AG AA AG AA
20675783
4594 rs10121993 9 AA AA AA AG AA
AG AA AA AA AA AA AG AA
AA AA AA AA AA AA AA AG
GG AG GG AG AA AG AA AA
AG AG AG AG AA AA AG
71328378
4595 rs10122013 9 GG GG GG GG GG
GG GG GG GG GG GG GG GG
GG GG GG GG GG GG GG GG
GG GG GG GG GG GG GG GG
GG GG GG GG GG GG GG
1062925
4596 rs1012202 X AA AA AA AG GG
AA AG AA AA AA AA AA AA
AG AG AA AG AG AG AG AA
AA AA AA AA AA AA AA AA
AG AA AA AA AA AG AA
43051345
4597 rs10122029 9 AA AA AA AA AA
AA AA AA AA AA AA AA AA
AA AA AA AA AA AA AA AA
AA AA AA AA AA AA AA AA
AA AA AA AA AA AA AA
82127171
4598 rs10122040 9 AG AG AA GG GG
GG GG GG GG GG GG GG GG
GG GG AG AG AA GG GG GG
GG GG GG GG AA GG AG AG
GG GG AG GG GG GG GG
15279214
4599 rs10122048 9 AG AG AG AG AA
AA AG AA AG AA AA AG AA
AA AA AA AA AA AA AA AA
GG AG GG AG GG AG GG GG
AG GG AG AG AA AA AA
81693987
4600 rs10122058 9 CC CC CC AC CC
AC CC AC AC CC CC CC CC
CC CC CC CC CC CC CC CC
CC CC CC CC AC CC AC AC
CC CC CC CC CC CC CC
111000640
4601 rs10122079 9 AA AA AA AA AA
AA AA AA AA AA AA AA AA
AA AA AA AA AA AA AA AA
AA AA AA AA AA AA AA AA

	AA	AA	AA	AA	AA	AA	AA
135455126							
4602	rs10122096	9	AG	AG	GG	AA	AA
	AA	AA	AA	AA	AA	AA	AA	AA
	AA	AA	AA	AG	AA	AA	AG	AA
	AA	AA	AA	AA	AA	AG	AA	AG
	AA	AG	AA	AG	AG	AA	AG
9405431							
4603	rs10122194	9	AG	GG	GG	GG	GG
	GG	GG	GG	GG	GG	GG	GG	GG
	GG	GG	GG	GG	GG	GG	GG	GG
	GG	GG	GG	GG	GG	GG	GG	GG
	GG	GG	GG	GG	GG	GG	GG
128913654							
4604	rs10122200	9	GG	GG	GG	GG	GG
	GG	GG	AG	AG	GG	GG	GG	GG
	GG	GG	GG	GG	GG	GG	GG	GG
	GG	GG	GG	GG	GG	GG	GG	GG
	GG	GG	GG	GG	GG	GG	GG
79489779							
4605	rs10122209	9	GG	AG	GG	AA	AG
	AA	AG	AG	AG	AG	AG	AG	GG
	GG	AG	GG	GG	GG	AG	AG	AA
	GG	GG	GG	GG	AG	AG	AG	GG
	GG	AG	AG	AG	AG	AG	AG
81812343							
4606	rs10122211	9	AA	AG	AA	AG	GG
	AG	GG	AG	AG	GG	GG	AG	AG
	AG	GG	AG	AG	GG	GG	AG	GG
	AA	AG	AG	AG	AG	AG	AG	AA
	GG	GG	AA	AG	GG	AG	GG
92809471							
4607	rs10122237	9	AG	GG	AG	GG	GG
	GG	GG	GG	AG	AG	AG	AG	AA
	AG	AA	GG	GG	GG	AG	GG	AG
	AG	AG	AG	GG	AG	AG	AG	AG
	GG	GG	GG	GG	GG	AG	GG
4412949							
4608	rs1012224	X	GG	GG	GG	AG	AA
	AA	AG	GG	GG	GG	GG	GG	GG
	GG	GG	AG	GG	AG	AG	AG	GG
	GG	GG	GG	GG	GG	AA	AG	GG
	AG	AG	GG	GG	AG	AA	GG
7851066							
4609	rs10122246	9	AG	AG	AA	AA	AA
	AA	AA	AA	AA	AA	AA	AA	AA
	AA	AA	AA	AA	AA	AA	AA	AA
	AA	AA	AA	AA	AA	AA	AA	AA
	AA	AA	AG	AG	AA	AA	AA
4327048							

4610 rs10122321 9 AG AA AG GG AG
GG AG AG GG GG AG GG GG
GG AG GG GG GG GG GG GG
GG AA AG AA GG AG GG GG
GG GG GG GG GG AG GG
90125146
4611 rs1012235 14 AC AA AA AC AC
AA CC AA AC AA AA AC AC
CC AC AA AC AC AC AA AC
AA AC AC CC AA AA AA AA
AC AA AA AA AA AA AA
26786854
4612 rs10122479 9 AG GG AG GG GG
GG GG GG GG GG AG GG GG
GG GG GG GG GG GG GG GG
GG GG GG GG GG GG GG GG
GG GG GG GG GG GG GG
74251194
4613 rs1012248 16 CC AC CC AC AC
AC CC CC CC CC CC CC CC
CC CC CC CC CC AC AA CC
AC AC CC AC AC CC CC AC
CC CC CC CC CC CC CC
63418779
4614 rs10122600 9 GG AG GG GG AG
AG GG GG AA GG GG AG AG
AG AG GG GG GG AG AG AG
AG AG AG GG GG AG GG GG
GG GG GG GG GG GG GG
82949474
4615 rs10122609 9 AA AA AA AA AA
AA AA AG AG AA AA AA AA
AG AG AA AA AA AA AG AA
AA AA AA AG AA AA AA AA
AA AA AA AA AA AA AA
9283557
4616 rs10122614 9 CC CC CC CC CC
CC CC CC CC CC CC CC CC
CC CC CC CC CC CC CC CC
CC CC CC CC CC CC CC CC
CC CC CC CC CC CC CC
75945036
4617 rs1012268 17 AG AG GG AG AG
GG AA AA AA AA AG AG AA
AA AA AA AG AG AA AA AA
AA AA AA AA AG AG GG AG
AA AG AA AA AG AA GG
60812041
4618 rs10122709 9 AA AA AA AG AG
GG AA AG AA AA AA AA AG

	AG	AG	AG	AG	AG	AA	AA	AG
	AA	AA	AA	AA	AA	AA	AA	AA
	AG	AA	AG	AG	GG	AG	AG	
19290143								
4619	rs10122718		9	CC	CC	CC	AA	CC
	AC	AC	AC	AC	CC	AA	AC	AA
	AA	AA	AC	AC	CC	AC	CC	AC
	CC	AC	CC	AC	AC	AC	AA	CC
	AC	AC	AC	AC	CC	CC	AC	
6999846								
4620	rs10122770		9	GG	GG	GG	GG	GG
	GG	GG	GG	GG	GG	GG	GG	GG
	GG	AG	GG	GG	GG	GG	GG	GG
	GG	GG	GG	GG	GG	GG	GG	GG
	GG	GG	GG	GG	GG	GG	GG	
117807508								
4621	rs10122775		9	GG	GG	GG	GG	GG
	GG	GG	AG	AG	GG	GG	GG	GG
	GG	AG	GG	GG	GG	GG	GG	AG
	AG	AG	AG	AG	GG	AG	GG	GG
	AG	AG	AG	AG	AG	GG	AA	
32884983								
4622	rs10122835		9	GG	GG	GG	GG	GG
	GG	GG	GG	GG	GG	GG	GG	GG
	GG	GG	GG	GG	GG	GG	GG	GG
	GG	AG	GG	AG	GG	GG	GG	GG
	GG	GG	GG	GG	GG	GG	GG	
128662963								
4623	rs10122842		9	AA	AG	AA	AG	GG
	AG	GG	AG	AG	AA	AG	AA	AG
	AG	GG	GG	AG	GG	AG	GG	AG
	GG	AG	GG	AG	AG	AA	AG	AG
	AG	GG	AG	GG	GG	GG	AG	
114335885								
4624	rs10122848		9	CC	CC	CC	CC	CC
	AC	CC	CC	CC	CC	CC	CC	CC
	CC	CC	CC	AC	AC	CC	CC	CC
	AA	AC	AA	AC	CC	CC	CC	CC
	CC	CC	CC	CC	CC	CC	CC	
25368388								
4625	rs10122874		9	AC	CC	AC	AA	AC
	AC	AA	AA	AA	AA	AA	AC	AC
	AC	AC	AC	AC	AC	AC	AA	AC
	AC	AC	AC	AC	AC	AA	AA	AC
	AA	CC	AC	AC	AC	AC	AA	
72359990								
4626	rs10122900		9	GG	GG	GG	GG	AG
	GG	AG	GG	GG	GG	AG	GG	AG
	AG	GG	AA	AG	AG	GG	GG	GG
	GG	GG	GG	GG	GG	GG	GG	GG

AG GG GG GG GG GG GG
99207540
4627 rs10122927
9 -- -- -- -- -- -- -- --
-- -- -- -- -- -- -- --
-- -- -- -- -- -- -- --
-- -- -- -- -- -- -- --
-- -- -- -- 1294000
4628 rs10122984 9 CC CC CC CC CC
CC CC CC CC CC CC CC CC
CC CC CC CC CC CC CC CC
CC CC CC CC CC CC CC CC
CC CC CC CC CC CC CC
89410675
4629 rs10123002 9 AG AG AG GG GG
GG GG AG AG AG AA AG AA
AA AA AA AG AG AA AA AG
GG AG AG AA GG GG GG GG
AG GG GG GG GG AG AG
86272344
4630 rs10123003 9 CC CC CC CC CC
CC CC AC CC CC CC CC CC
CC AC CC CC CC AC CC AC
CC CC CC CC AC CC AC CC
AC AC CC AC CC CC CC
21620510
4631 rs10123044 9 AA AA AA AG AG
AA AG AG GG AA GG AA AG
AG AA AG AG AG AA AG AA
AG GG AG AG GG AA AG AG
AA AA AA AA AG AA AG
16373545
4632 rs10123088 9 AG AG AG GG GG
GG GG GG GG GG GG GG GG
GG GG GG AG AG GG GG GG
GG GG GG GG AG AG AG GG
GG GG GG GG GG GG GG
85930972
4633 rs10123118 9 GG GG GG AG AG
AG AG GG GG GG GG GG GG
GG GG GG GG GG GG GG GG
GG AG GG AG GG GG GG GG
GG GG GG GG GG GG GG
16778780
4634 rs10123148 9 GG GG GG GG GG
GG GG GG GG GG GG GG GG
GG GG GG GG GG GG GG GG
GG GG GG GG GG GG GG GG
GG GG GG GG GG GG GG
101712532

4635 rs1012315 12 AG AG AG GG AG
AG AG GG GG GG GG GG GG
AG GG AG GG GG GG AG GG
GG AG AG AG AA GG AG AG
GG AG GG AG GG AG GG
97639144
4636 rs10123153 9 AC AC CC CC AC
AC CC AA AC CC AC CC AC
AC AC CC AC AC AC AC AA
AA AC AC AC AA CC AC AC
CC CC AA AC AC AA CC
117728895
4637 rs10123254 9 AA AA AA AA AA
AA AA AA AA AA AA AA AA
AA AA AA AA AA AA AA AA
AA AA AA AA AA AA AA AA
AA AA AA AA AA AA AA
88557211
4638 rs10123280 9 AG GG AG AG AG
AG GG AA AA AG AG AG GG
GG GG GG GG GG GG GG AG
AG GG AG GG AG GG GG AG
AA AG AG AG AG AG AG
37627872
4639 rs10123329 9 AA AA AA AG AG
GG AA AA AA AA AA AA AA
AA AA AA AG AG AG AG AG
AA AA AA AA AA AA AA AA
AG AA AA AA AA AA AA
84102451
4640 rs10123338 9 CC CC CC CC CC
CC CC CC CC CC CC CC CC
CC CC CC CC CC CC CC CC
CC CC CC CC CC CC CC CC
CC CC CC CC CC CC CC
1766255
4641 rs10123371 9 GG GG GG AG GG
GG AG GG GG GG GG GG GG
AG GG GG GG GG GG GG GG
GG GG GG GG GG GG GG GG
AG GG GG GG AG GG AG
2361656
4642 rs10123375 9 AA AA AA AA AA
AA AA AG AG AA AA AA AA
AA AA AA AA AA AA AG AA
AG AG AA AA AA AA AA AA
AG AG AA AA AA AA AG
139746992
4643 rs10123378 9 GG AG GG AA GG
AG AG AG AA GG AA AA AA

	AA	AG	AG	AA	AA	GG	AG	GG
	GG	AG	GG	AG	AA	AA	AA	AA
	AA	AA	AG	AA	AG	AA	AG	
96375217								
4644	rs10123382		9	AG	AG	AA	GG	GG
	GG	GG	GG	GG	GG	AG	AG	AG
	AG	AG	GG	AG	GG	AG	GG	AG
	GG	GG	GG	GG	GG	GG	GG	GG
	AG	GG	AG	GG	GG	AG	AG	
4757649								
4645	rs10123416		9	AA	AA	AA	AA	AA
	AA	AA	AA	AC	AC	AC	AA	AA
	AA	AC	AC	AA	AC	AA	AA	AA
	AA	AA	AA	AA	CC	AA	AC	AC
	AA	CC	AA	AC	AC	AA	AC	
89640141								
4646	rs10123421		9	AG	AG	AG	AA	AA
	AA	AA	AA	AA	AG	AG	AA	AA
	AA	AG	AA	AA	AA	AA	AA	AA
	AG	AG	AG	AG	AA	AA	AA	AA
	AA	AA	AA	AA	AG	AA	AG	
77713279								
4647	rs10123481		9	AG	AG	AA	AG	AG
	AG	AA	AA	AA	AG	AA	AA	AA
	AA	AA	GG	GG	GG	AG	AG	AG
	GG	AG	GG	AG	AG	AG	AG	AA
	AA	GG	GG	GG	AA	AA	AG	
18465657								
4648	rs10123567		9	AG	GG	AG	GG	GG
	GG	GG	AG	AG	GG	GG	GG	GG
	GG	AG	GG	GG	GG	AA	AG	AG
	AG	AG	AA	AG	AG	GG	GG	GG
	AG	AG	GG	GG	GG	GG	GG	
112382522								
4649	rs10123595		9	GG	AG	AG	GG	GG
	GG	AG	AA	AA	GG	AA	AG	AA
	AG	AG	GG	GG	GG	GG	GG	GG
	AA	AA	AA	AG	AG	AG	AG	AG
	GG	AG	GG	GG	GG	AG	GG	
8799819								
4650	rs10123632		9	CC	CC	CC	CC	CC
	CC	CC	CC	CC	CC	CC	CC	CC
	CC	CC	CC	CC	CC	CC	CC	CC
	CC	CC	CC	CC	CC	CC	CC	CC
	CC	CC	CC	CC	CC	CC	CC	
129715104								
4651	rs10123637		9	AC	CC	AC	CC	CC
	CC	CC	AC	CC	CC	AA	AC	AC
	AC	CC	CC	CC	CC	CC	CC	CC
	AC	AC	AC	AC	AC	CC	CC	AC

CC CC AA AC CC AC AC
21721939
4652 rs1012365 4 GG GG GG GG GG
AG AG GG GG GG GG GG GG
GG GG AG GG AG GG GG GG
AG AG AG AG GG GG GG GG
GG GG AG AG GG GG GG
2917608
4653 rs10123658 9 AG GG AG AG AA
AG AA GG GG AA AA AG AG
AG AG GG GG GG AG AG AA
AG GG AA GG AG AG GG GG
GG AG AG AG GG AG AG
116486584
4654 rs10123659 9 AA AA AA AA AA
AA AA AA AC AA AA AA AA
AA AA AA AA AA AA AA AA
AA AA AA AA AC AA AA AC
AA AA AA AA AA AA AA
83235735
4655 rs10123671 9 AA AA AA AA AA
AA AA AA AA AA AG AA AA
AA AA AA AG AG AG GG AG
AA AA AA AA AG AA AG AA
AA AA AA AA AA AA AA
29161218
4656 rs1012370 X GG GG GG GG GG
GG GG GG GG GG GG GG GG
GG GG GG GG GG GG GG GG
GG GG GG GG GG GG GG GG
GG GG GG GG GG GG GG
17925927
4657 rs10123732 9 AG AG AG AA AG
AG AA AG AA AA AG AA AA
AA AG AG AA AG AG AG AG
AA AG AG AG AG AG AG AA
AG AA GG AG AA AA AA
90395684
4658 rs10123752 9 AG AG AG AA AG
AG AA AG AG GG AG AA AG
AG AA AA AA AA AG AG AG
AG AG AG AG AA AA AA AA
AA AG AA AG AG AG AA
79298723
4659 rs10123800 9 AA AA AA AA AG
AA AG AG AA AA AA AA AA
AA AA AA AA AA AG AA AG
AA AA AA AA AA AA AA AA
AA AA AA AA AG AA AG
3012372

4660 rs10123838 9 AG AA AG GG GG
GG AG AG AG GG AA AG AA
AA AA GG AG AG AG AG GG
GG AG GG AG AG AA AG AA
AG AG AG AG AG GG AA
78071910
4661 rs10123887 9 GG GG GG AG AG
AG GG AG AG GG GG AG AG
AG AG GG GG GG AA AG AG
AG AA AG AG GG GG GG GG
AG GG GG GG AA AA AA
13593125
4662 rs10123913 9 CC CC CC AC CC
CC AC AC AC AC AC CC CC
CC AA AC AA AC AA AA AA
AA AA AA AA AC AC CC CC
AC AC AC AA AC AA AC
103063826
4663 rs10123925 9 AA AA AA AG AG
AG AG AA AA AA AG AA AA
AA AG AA AA AA AA AA AG
AG AG AG AG AG AA AG AA
GG AG AA AG AG AG AG
36906843
4664 rs10123927 9 GG GG AG AG GG
AG AG AA AA AG GG AG AG
AG GG GG AG AG AG AG AG
AG AG AG AG AG AG GG GG
GG GG AG GG GG AG AG
120994586
4665 rs10123937 9 AA AG AG AG AG
AA AG GG AG AG AA AG AG
AG AG AA AA AG AA AG AA
AA AG AA AG AG AA AA AG
AG AG AA AA AG AG AA
13929064
4666 rs10123973 9 AG AG GG GG GG
GG GG AA AG AA GG AG GG
AG AG GG AG GG GG GG AG
AG GG AG GG GG AG AG GG
GG GG AA AG AG AG GG
14672443
4667 rs10123982 9 AA AA AA AG AG
AA GG AG GG AA GG AA AG
AG AA AG AG GG AG AG AA
GG GG GG AG AG AA AA AG
AA AA AA AA AA AA AG
16369067
4668 rs1012402 7 GG GG GG GG GG
GG GG GG GG GG GG GG GG

GG GG AG GG AG GG GG GG
GG AA AG AA GG GG GG GG
GG AG GG AG AG AG AG
16211816
4669 rs10124033 9 GG GG GG GG AG
AG GG GG GG GG GG GG GG
GG GG GG AG GG GG GG GG
GG GG GG GG GG GG GG GG
GG GG GG GG GG GG GG
99614104
4670 rs10124038 9 GG GG GG AG AA
AG AG AA AA GG AA AG AG
AG GG GG GG GG AA AA AA
AA AG AA AG AA AG AG AG
AG GG AG GG GG AG AG
110872527
4671 rs10124142 9 GG GG GG GG GG
GG GG GG AG AA AG AA AG
AG GG GG AG GG GG GG GG
GG AG GG AG GG GG GG GG
AG AA AG AA AG GG AG
118186082
4672 rs1012417 X AA AA AG AG GG
GG AG GG GG GG AG AG AA
AA AG GG AG AG AA AA AA
GG GG GG GG AG AA AA AA
GG AG AA AA GG AG GG
8473743
4673 rs10124204 9 AA AA AA AA AA
AA AA AA AA AA AA AA AA
AA AA AA AA AG AA AA AG
AA AA AA AA AA AA AA AA
AA AA AA AA AG AG AG
131129207
4674 rs1012421 12 AA AA AA AA AG
AA AG AG AG AA AA AA AA
AA AA AA AA AA AG AA AA
AA AA AA AA AA GG AG AG
AA AA AA AA AA AA AA
89425822
4675 rs10124221 9 AA AA AA AA AA
AA AA AA AA AA AA AC AC
AC AC AA AC AC AC AC AC
AC AC AC AC AA AA AA AA
AC AA AA AA CC AC AC
85023902
4676 rs10124246 9 CC CC CC CC CC
CC CC CC CC CC CC CC CC
CC CC AC AA AA CC CC CC
AA AC AA AC AA CC AC AC

CC AC AC CC CC CC CC
34713129
4677 rs10124250 9 GG AG AG GG AG
GG AG AG AG AG AG GG AG
AG AG GG AG AG GG AG GG
AA AA AA AA AG AG AG AG
GG AG AA AG AG AA GG
6161686
4678 rs10124291 9 AG GG AG GG GG
GG GG AG GG AG GG AA AG
AG AG AG GG AG GG GG AG
GG GG GG GG AG AG AG GG
AG AG AG AG AG AG GG
90068509
4679 rs10124300 9 CC AC CC AC AA
AC AC AA AA AC AA AC AC
AC AC CC AC AC CC AC CC
AC AC CC AC AA CC AC AC
AA AA CC AC CC CC CC
112277740
4680 rs10124314 9 GG AG -- AG GG
AG AG AG AG GG GG GG --
AG AG AG AG AA AG GG AG
-- GG -- GG -- AG AG AG
AA -- -- -- AG AG AG
125384197
4681 rs10124376 9 AA AA AA AA AG
AG AA AG AA AA AG AA AG
AG AG AG AG GG AG AG AG
AA AA AA AA AA AG AA AA
AG AA AA AA AA AA AA
118173811
4682 rs10124379 9 AA AA AG GG AG
AG GG AA AA AG GG AG AG
GG GG GG GG GG AA AG AG
GG AG GG AG AG AG GG GG
AG GG AA AG AG AG AA
129141450
4683 rs10124457 9 AA AG AG AA AG
AA AG AG AA AG AG AA AG
AG AG AG AA AA AA AG AA
-- AG GG AG GG AG AG AG
AA AA GG AG AG AG AA
2348820
4684 rs10124561 9 AA AA AC AA AA
AA AA AA AA AA AA AA AA
AA AA AA AA AC AA AA AA
AA AA AA AA AA AA AA AA
CC AA AA AA AC AA AC
119655679

4685 rs10124645 9 AG AG AA GG GG
GG AG AA AA AG GG AG GG
AG AG AG AA AA AG AG AA
AG AA AG AA AA AG AG AA
AA AA AG AG AG AA AG
11321119
4686 rs10124685 9 AA AG AG AG AG
AA AG AA AA AA AA AA AA
AA AA AA AA AA AG AG AA
AA AA AA AA AA AG AG AG
AA AA AG AA AG AG AA
118152551
4687 rs10124717 9 CC CC CC CC CC
CC CC CC CC CC CC CC CC
CC CC CC CC CC CC CC CC
AC AC AC CC CC CC CC CC
AC CC CC CC CC CC CC
18296065
4688 rs10124779 9 AA AG AA GG AG
GG AG AA AG AA AG AG AA
AA AG AA AG AG AG AG AA
AG AA AG AA AG AG AG GG
AG AG AA AA AG AA AG
85519130
4689 rs10124780 9 AA AA AA AA AA
AA AA AA AA AA AA AA AA
AA AA AA AA AA AA AA AA
AA AA AA AA AA AA AA AA
AA AA AA AA AA AA AA
88574936
4690 rs10124785 9 AA AG AA GG AG
AG GG AA AA AG AG GG AG
AG GG AG AG AG AG AG AG
GG AG GG AG GG AG AG AG
AA AG GG AG GG GG GG
77814984
4691 rs10124839 9 AA AA AA AA AA
AA AA AC AA AC AA AA AA
AC AC AC AA AA AA AA AA
AA AA AA AA AA AC AA AC
AA AA AA AA AA AA AA
14761260
4692 rs10124840 9 AG AA AA AG AG
AA AG AG AA AG AA AG AA
AA AA AG AA AG AA AA AG
AA AA AA AA AA AG AA AA
AA AG AA AA AA AA AA
138616058
4693 rs10124859 9 AA AA AA AA AA
AA AA AA AA AA AA AA AA

AA AA AA AA AA AA AA AA
AA AA AA AA AA AA AA AA
AA AA AA AA AA AA AA
75129891
4694 rs10124903 9 AG GG AG AG AG
AA GG GG AG AG GG -- GG
GG GG AA AG AG AG -- AG
AA AA AA AA AG AG AG AA
-- AG GG AG AG AG AG
25659613
4695 rs10124911 9 CC CC CC CC CC
CC CC CC CC CC CC CC CC
CC CC CC AC CC CC CC CC
CC CC CC CC CC CC CC CC
CC CC CC CC CC CC CC
99567384
4696 rs10124926 9 AA AA AG AA AA
AA AG AG AG AA AA AA AA
AA AA AA AA AA AG AG AG
AA AG AG AG -- AG AG GG
AA GG AA AG AG GG AG
137883639
4697 rs10125011 9 AA AA AG AG AA
AG AG AA AG AA AA AA AA
AA AA AG AA AA GG GG AG
AA AA AA AA AA AA AA AA
AA AG AG AG AA AA AA
129506906
4698 rs10125015 9 AG AA AG AG AG
AG AG AG AA AA AA AG AG
AA AG AA AG AA GG AA AG
AA AG AA AG AG AA AA AG
AA AA GG AG AG AA GG
6609578
4699 rs10125056 9 GG GG GG GG GG
GG GG GG GG GG GG GG GG
GG GG GG GG GG GG GG GG
GG GG GG GG GG GG GG GG
GG GG GG GG GG GG GG
138655979
4700 rs10125071 9 AG AG GG AG AG
AG AG AG AA AA AG AA AA
AG AA AG AA AG AA AA AA
AA AA AA AA AA AG AA AA
AG GG AA AG AG AG AA
2679579
4701 rs10125101 9 GG AG AG GG GG
GG GG GG GG GG GG GG GG
GG GG GG GG GG GG GG GG
GG GG GG GG GG GG GG GG

GG GG GG GG GG GG GG
1184213
4702 rs10125156 9 AA AG AG AA AA
AA AA AA AA AA AA AA AA
AA AA AG AA AA AA AG AG
AA AA AA AA AA AA AA AA
AA AG AA AG AG AA AG
10355941
4703 rs10125159 9 GG GG GG GG GG
GG GG GG GG GG GG AG GG
GG GG GG GG GG AG GG AG
AG GG AG GG GG GG GG GG
GG AG GG GG GG GG AG
4150202
4704 rs1012518 4 GG GG GG GG AG
GG AG GG AG AG GG GG GG
AG GG AG GG GG AG AG AG
GG GG GG GG GG AG AG GG
GG GG GG GG GG AG GG
185965017
4705 rs10125228 9 GG GG GG GG GG
GG GG AA AA AG GG GG AG
AG AG AG AG AG GG GG AG
GG GG GG GG AG GG GG AG
AG GG AG AG AA AG AG
19428721
4706 rs10125253 9 AA AG AG AG AG
AA GG GG AG AA AA AA AA
AA AG GG GG AG AG AG AG
AG GG AA GG AA AA AA AA
AG AG GG GG GG AG GG
118012841
4707 rs10125301 9 GG AG GG GG GG
GG GG GG GG GG GG GG GG
GG GG AG AG AG GG AG GG
GG GG GG GG GG AG AG AG
GG GG GG GG AG GG AG
71331990
4708 rs1012531 19 AA AA AA AA AA
AC AA AA AA AA AC AC AC
AA AA AA AA AA CC AC AC
AA AA AA AA AA AC AA AA
AA AA AC AC AC AA CC
53118278
4709 rs10125337 9 AA AA AA AA AA
AA AA AA AA AA AA AA AA
AA AA AA AA AA AA AA AA
AA AA AA AA AA AA AA AA
AA AA AA AA AA AA AA
97444038

4710 rs10125370 9 GG GG GG GG GG
GG GG GG GG GG GG GG GG
GG GG GG GG GG GG GG GG
GG GG GG GG GG GG GG GG
GG GG GG GG GG AG GG
90650880
4711 rs1012541 15 GG GG GG AG GG
GG AG GG GG GG GG GG GG
GG GG GG GG GG GG GG GG
GG GG GG GG AG GG GG AG
AG GG GG GG GG AG GG
81246028
4712 rs1012553 10 AG AA AA GG GG
GG GG GG GG GG GG GG GG
GG GG AG GG AG GG AG AG
AG AG AG AA AG GG GG AG
GG GG AG GG GG AG GG
50740143
4713 rs10125538
9 -- -- -- -- -- -- -- --
-- -- -- -- -- -- -- --
-- -- -- -- -- -- -- --
-- -- -- -- -- -- -- --
-- -- -- -- 96821924
4714 rs10125592 9 AA AA AA GG AG
AG GG AA AA AA AA AA AA
AA AG AA AA AA AA AA AA
AA AG AA AG AG AA AG AG
AA AG AA AG AA AG AA
123161735
4715 rs10125595 9 GG GG GG GG GG
GG GG GG GG GG GG GG GG
GG GG GG GG AG GG GG GG
GG GG GG GG AG AG AG AG
GG GG GG GG AG GG AG
7585382
4716 rs10125602 9 GG GG GG GG GG
GG GG GG GG GG GG AG GG
GG GG AG GG GG GG GG GG
GG GG GG GG GG GG GG GG
GG GG AG GG GG GG GG
31340536
4717 rs10125617 9 GG AG GG AG AG
GG AG AG AG AG GG GG GG
GG AG AA AG AG GG AG GG
GG AG GG AG GG AG GG GG
GG AG GG GG AG AG AA
72918167
4718 rs10125623 9 AA AA AA AG AA
AA AG AG AG AA AA AA AA

AA AA AA AA AA AG GG AG
AG AA AG AA AA AA AA AA
AG AA AA AA AG AA AG
99463916
4719 rs10125646 9 -- -- GG AA AA
AG AA AG AG AA GG GG GG
GG AG AA AG AG GG AG AG
GG AG GG AA GG GG GG GG
AG GG AG GG AG AG AG
19045502
4720 rs10125655 9 GG GG AG GG AG
AG GG AG AA AG GG AG AG
AG AA AG AG GG AG GG GG
AG AG AG AG AG AG GG AG
AG AG AA AA AA AG AG
134674287
4721 rs10125657 9 AA AA AA AA AA
AA AA AC AA AC AC AA AC
AC AA AA AA AA AA AA AA
AA AA AA AA AA AA AA AA
AA AC AA AA AA AA AC
82241503
4722 rs10125662 9 GG AG GG GG GG
GG GG GG GG GG GG GG GG
GG GG GG AG AG AG AG AG
GG GG GG GG GG GG GG GG
GG GG GG GG GG GG GG
83867125
4723 rs10125737 9 AA AA AA AA AG
AA AG AA AG AA GG GG GG
GG GG AG GG GG GG GG GG
GG GG GG GG AA AA AA AA
AG AA AG AG AG AG AA
81083897
4724 rs10125775 9 GG AG AG AG AG
AA AG GG GG GG GG GG GG
GG AG AA AA AA AG AG AA
GG AG GG AG GG AG GG GG
AG AG AA AG AG AG AG
36993071
4725 rs10125793 9 CC CC CC CC CC
CC CC CC CC CC CC CC CC
CC CC CC CC CC CC CC CC
CC CC CC CC CC CC CC CC
CC CC CC CC CC CC CC
134784950
4726 rs10125846 9 GG GG GG AG AG
AG AG GG AG GG GG GG GG
AG GG GG GG GG AG AG GG
GG GG GG GG AG AG AG GG

	GG	AA	GG	AG	AA	AA	AA
2831891							
4727	rs10125854	9	AA	AA	AA	AA	AG
	AG	AA	AA	AG	AG	AA	AA	AA
	AA	AA	AA	AG	AA	AA	AA	AA
	AA	AA	AA	AA	AA	AA	AA	AA
	AA	AA	AA	AA	AA	AA	AG
5464065							
4728	rs10125891	9	AG	AA	AA	GG	GG
	GG	GG	GG	AG	AG	GG	GG	GG
	GG	GG	GG	GG	AG	GG	AG	GG
	GG	GG	GG	GG	GG	AG	GG	GG
	AA	AG	AG	AG	AA	AA	AG
128921921							
4729	rs10125894	9	AA	AC	AC	AA	AA
	AA	AA	AA	AA	AA	CC	AA	AC
	AA	AA	AA	AA	AA	AC	AA	AA
	AA	AA	AA	AA	AA	AC	AC	AA
	AA	AC	AC	AA	AA	AA	AA
8366757							
4730	rs1012593	9	AG	AA	AG	AA	AA
	AA	AA	AA	AG	AG	GG	AG	GG
	GG	GG	GG	GG	GG	GG	GG	AG
	AG	GG	GG	GG	AA	AA	AA	AA
	AG	GG	AA	AG	AG	AG	GG
89709884							
4731	rs10125937	9	AG	AA	AG	GG	GG
	GG	GG	AG	GG	AG	AG	AG	AA
	AG	GG	AG	AA	AG	GG	AG	GG
	GG	AG	GG	AG	GG	AG	AG	AG
	AG	GG	GG	GG	GG	GG	GG
124790623							
4732	rs10125965	9	AG	GG	AG	AA	AG
	AA	AG	AG	AG	AG	AA	AG	AA
	AG	AA	AA	AA	AG	AG	AG	AG
	AG	GG	AG	AG	AG	AG	AA	GG
	AG	GG	AA	AG	AG	GG	AA
5333154							
4733	rs10125969	9	AG	AG	AG	AG	AG
	AA	AG	AA	AA	AG	AG	AA	AG
	AG	GG	GG	AG	AG	AG	AG	AG
	AA	AA	AA	AA	AG	AG	AG	AG
	AG	AA	AG	AA	AG	AG	AG
117126307							
4734	rs1012597	2	AA	AA	AA	AA	AA
	AA	AA	AA	AA	AA	AA	AA	AA
	AA	AA	AA	AA	AA	AA	AA	AA
	AA	AA	AA	AA	AA	AA	AA	AA
	AA	AA	AA	AA	AA	AA	AA
200702887							

4735 rs10126007 9 GG GG GG AG GG
AG GG GG GG GG AG GG GG
AG GG AG AG AG GG AA GG
GG AG GG GG GG GG GG GG
GG GG GG GG AG GG AG
1644465
4736 rs10126039 9 AC AC AC AA AC
AC AA AA AA AA AA AA AA
AA AC AA AC AA AC AC AA
AA AA AA AA AA AA AA AA
AA AA AA AA CC CC CC
99085363
4737 rs10126080 X GG GG GG AG GG
GG AG GG GG GG GG GG GG
GG GG GG GG GG GG GG GG
GG GG GG GG GG GG GG GG
GG AA GG AA GG GG GG
123961263
4738 rs1012619 2 AG AG AA AA AA
AA AA AG AG AG AA AA AA
AA AA AA AA AA AA AA AA
AA AA AA AA AA AA AA AA
AA AA AG AG AA AA AA
40988880
4739 rs10126199 X GG AG AA AG AA
GG AA AA AA AA AG AG AG
AA AA AA AG AG AA AA AA
AA GG GG AA GG AA AG GG
AG AA AA AA GG AG GG
46417031
4740 rs1012620 2 AA AA AA AA AG
AG AA AA AA AA AA AA AA
AA AA AA AA AA AA AA AA
AA AA AA AA AA AA AA AA
AA AA AA AA AA AA AA
40989061
4741 rs1012624 6 AA AA AA AA AA
AA AC AC AC AC AC AA AC
AA AC AA AA AA AC AC AC
AC AC AC AC CC AC CC CC
AA AA AC AC AC AA AC
53818262
4742 rs10126244 X AA AA AA AA AA
AA AA AA AA AA AA AA AA
AA AA AA AA AA AG AA GG
AA AA AA AA AA AA AA AA
AG AA AA AA AA AA AA
115521351
4743 rs10126302 X AA AA AA AA AA
AA AA AA AA AA AA AA AA

AA AA AA AA AA AA AA AA
AA AA AA AA AA AA AA AA
AA AA AA AA AA AA AA
106853641
4744 rs10126345 X AA AA AA AG AA
AA AG AA AA AA AA AA AA
AA AA AA AA AA AA AA AA
AA AA AA AA AA AA AA AA
AA AA AA AA AG AG AA
121646883
4745 rs10126348 X GG GG AG AG GG
AA GG AG AG GG GG GG GG
GG GG AG GG AG AG AG GG
GG GG AA GG AA GG AG AA
AG AG AA GG GG GG GG
5710628
4746 rs10126412 X GG GG GG GG GG
GG GG GG GG GG GG AG AG
GG GG AG AG GG GG AG GG
GG GG GG GG GG AA AG GG
AG GG GG GG GG AG GG
105229625
4747 rs10126447 X AA AA AA AA AA
AA AA AA AA AA AA AA AA
AA AA AA AA AA AA AA AA
AA AA AA AA AG GG GG AA
AG AA AA AA AA AA AA
104988706
4748 rs10126585 X GG GG GG GG GG
GG GG GG GG GG GG GG GG
GG GG GG GG -- GG GG GG
GG GG -- GG GG GG GG GG
GG GG GG GG GG GG GG
136649762
4749 rs1012668 2 AA AA AA AA AA
AA AA AG AG AA AA AA AA
AA AA AG GG AA AA AA AA
AA AA AG AA AA AA AA AA
AA AA AA AA AA AA AG
36874250
4750 rs10126696 X GG GG GG GG GG
GG GG CG CG CC CG CG CG
GG GG CG CG CC CG GG CC
CC GG GG CG CC GG CG CC
CC GG GG GG CG CC GG
48098473
4751 rs10126698 X AA AG AG GG GG
GG GG AA AG GG AG GG AG
AG AG GG GG GG AG GG AA
GG GG AA AA AG GG GG AA

AG AA GG AA AG AA GG
103560394
4752 rs10126716 X GG GG GG GG GG
-- GG GG GG GG GG GG AG
AG GG GG GG GG AG AG GG
GG GG AA GG GG GG GG GG
GG GG GG GG GG GG GG
6059593
4753 rs1012672 12 AG AG GG AG AG
GG AG GG GG GG GG GG GG
GG GG GG GG GG GG GG GG
GG GG GG GG GG GG GG GG
AG GG GG GG GG GG GG
12284915
4754 rs10126793 X GG GG GG GG GG
GG GG AG AG AA GG GG GG
GG GG AG GG GG GG GG GG
GG GG GG GG GG GG GG GG
GG GG GG GG AG AG GG
24466015
4755 rs10126799 X AA AG AG AA AA
AA AA AA AA AA AG AG AA
AG AG AG AG AA AA AA AA
AA AA AA AA AG AA AG GG
AA AA AA AA GG AG GG
40076904
4756 rs10126810 X GG GG GG GG GG
GG GG GG GG GG GG GG GG
GG GG GG GG GG GG GG GG
GG GG GG GG GG GG GG GG
GG GG GG GG GG GG GG
129824677
4757 rs10126819 X AA AA AA AA AA
AA AA AG AG GG AG AG GG
AG AG AA AA AA AA AA AA
AA GG AA AG AG AA AA GG
AG AA AA AA AG AA GG
25174003
4758 rs10126826 X GG GG AG GG GG
GG GG AG AG GG GG GG GG
GG GG GG AG AG AG AG GG
AA AA AA AG GG GG GG GG
AG GG GG GG AG AA GG
20784137
4759 rs10126870 X AA AG GG AA AA
AA AA AA AA AA AA AA AA
AA AA AG AA AG AA AA AA
GG GG AA AG AA AA AA AA
AG AA AA AA AG GG AA
113479991

4760 rs10126881 X AA AA AA AA AA
AA AA AA AA AA AA AA AA
AA AA AA AA AA AA AA AA
AA AA AA AA AA AA AA AA
AA AA AA AA AA AA AA
153365761
4761 rs10126906 X AA AA AA AA AA
AA AA AG AG AA AA AG AG
GG GG AG AG AA AA AA AA
AA AA AA AA AG GG GG GG
AA AA AA AA AA AA AA
146281665
4762 rs10126923 X GG GG GG GG GG
GG GG GG GG GG GG GG GG
GG GG GG GG GG GG GG GG
GG GG GG GG GG GG GG GG
GG GG GG GG GG GG GG
68067527
4763 rs10126924 X AA AA AA AA AA
AA AA AA AA AA AA AA AA
AA AA AG AG AG AG AG AA
AA AA AA AA AA AA AA AA
AA AA AA AA AA AA AA
30933971
4764 rs10126934 X GG GG GG AG GG
AA GG GG GG GG GG GG GG
AG AG AG GG AG GG GG GG
GG AA GG AG AA GG AG AA
GG AG GG GG GG GG GG
114579251
4765 rs1012695 5 AA AA AA AC AC
AC AA CC CC CC AC AA AC
AA AA AA AC AC AA AA AA
CC CC CC CC AA AA AA AA
AA AC AC AC AA AA AA
117891968
4766 rs10126973 X AA AA AA AA AA
AA AA AA AA AA AA AA AA
AA AA AA AA AA AA AA AA
AA AA AA AA AA AA AA AA
AA AA AA AA AA AA AA
154913353
4767 rs10127016 X AA AA AA AA AA
AA AA AA AA AA CC CC AC
AC AC AA AA AA AA AC AA
AA AA AA AA AC AA AC AA
AC AC AA AA AA AA AA
53152096
4768 rs10127037 X GG GG AG AA AA
AA AG AG AG AA AG AG GG

GG GG AG GG AG AG GG AA
AA GG GG AG AG GG GG GG
GG AG GG GG AG GG AA
45109404
4769 rs10127045 X AA AA AA AA AA
AA AA AA -- AA AA AA AA
AG AG AA AG AG AA AG AA
AA AA AA AA AG GG GG GG
AA AG AA AA AA AA AA
40745226
4770 rs10127070 X AA AA AA AA AA
AA AA AA AA AA AA AA AA
AA AA AA AA AA AA AA AA
AA AA AA AA AA AA AA AA
AA AA AA AA AA AA AA
154056384
4771 rs10127106 X GG GG AG AG GG
AA GG GG GG GG AG AG GG
GG GG GG AG AG AA AA AA
GG GG AA GG GG GG GG GG
AG AG GG AA AG AG GG
144016184
4772 rs10127108 X CC CC CC CC CC
CC CC CC CC CC CC CC CC
CC CC CC CC CC CC CC CC
CC CC CC CC CC CC CC CC
CC CC CC CC CC CC CC
111720962
4773 rs10127121 X AA AG AG AG AA
GG AA GG GG GG GG GG GG
GG GG GG AG AG GG GG GG
GG GG GG GG AG AA AG AA
AA GG GG GG AA AG AA
108053565
4774 rs10127219 X AA AA AA AA AA
AA AA AA AA AA AA AA AA
AA AA AA AA AA AA AA AA
AA AA AA AA AA AA AA AA
AA AA AA AA AA AA AA
41837771
4775 rs1012727 16 AA AA AA AA AA
AA AA AA AA AA AA AA AA
AC AA AA AA AA AC AC AA
AA AC AA AA AA AA AA AA
AC AA AA AA AA AA AA
21243437
4776 rs10127303 X GG GG GG GG GG
GG GG GG GG GG GG GG GG
GG GG GG GG GG GG GG GG
GG GG GG GG GG GG GG GG

GG	GG	GG	GG	GG	GG	GG
20743778
4777	rs1012734	2	AG	AG	AG	AG	AG
AA	GG	AA	AA	AA	AA	AG	AG
AA	AA	GG	AG	AG	AG	GG	AG
AG	GG	AG	AG	AG	AG	AA	GG
AG	AG	AG	GG	AG	AG	AA
130376011
4778	rs10127373	X	CC	CC	CC	CC	CC
CC	CC	CC	CC	CC	CC	CC	CC
CC	CC	CC	CC	CC	CC	CC	CC
CC	CC	CC	CC	CC	CC	CC	CC
CC	CC	CC	CC	CC	CC	CC
39328307
4779	rs10127431	X	GG	GG	GG	GG	GG
GG	GG	GG	GG	GG	GG	GG	GG
GG	GG	GG	GG	GG	GG	GG	GG
GG	GG	GG	GG	GG	GG	GG	GG
GG	GG	GG	GG	GG	GG	GG
30443613
4780	rs10127438	1	GG	GG	GG	GG	GG
AG	GG	GG	AG	AA	AG	AG	AG
AG	AG	GG	GG	AG	AG	GG	AG
AG	GG	AG	GG	GG	GG	GG	GG
GG	AG	AG	GG	GG	GG	GG
62560928
4781	rs10127471	1	AC	AC	AA	AA	AC
AA	AC	AA	AA	AA	AA	AA	AA
AA	AA	AC	AA	AC	AA	AA	AA
AA	AC	AA	AC	AC	AC	AA	AA
AC	AC	AA	AA	AC	AC	AA
145671775
4782	rs10127480	1	AG	GG	AG	GG	AG
AG	GG	AA	AA	AA	AG	AG	AA
AA	AA	AA	AG	AG	GG	AG	AG
AA	GG	AG	GG	AG	GG	AG	AG
AG	GG	AG	GG	AG	AG	AG
216844423
4783	rs10127622	1	AC	AA	AC	AC	AA
AA	AC	AA	AA	AA	AA	AA	AA
AA	AA	AC	AC	AA	AA	AA	AA
AC	AC	AC	AC	AA	AA	AA	AA
CC	AC	AC	AA	AA	AA	AC
151468060
4784	rs1012768	7	CC	AC	CC	AA	AC
AC	AA	AC	AC	AC	AC	AC	CC
CC	CC	AC	AC	CC	AC	AC	AC
CC	CC	CC	CC	AC	AC	AC	AA
AC	AC	AC	AC	AC	AA	CC
24356504

4785 rs10127769 1 AA AA AA AA AG
AG AA AG AG AG AA AA AA
AA AA AG AA AG AG AG AA
AG AG AG AA AA AA AA AA
AA AA AA AA AA AA AA
239746732
4786 rs10127776 1 AA AG AG AG GG
GG AG GG GG GG AG AG AA
AA AA AG GG AG AA AG AG
AA AA AA AA AG GG AG AG
AG GG AA AG GG GG GG
226474479
4787 rs10127824 1 GG GG GG GG GG
GG GG GG GG GG GG GG GG
GG GG GG GG GG GG GG GG
GG GG GG GG GG GG GG GG
GG GG GG GG GG GG GG
64047615
4788 rs10127838 1 GG GG GG GG GG
GG GG AG GG GG GG GG GG
GG GG AG GG GG GG GG GG
AG AG GG GG GG AG GG GG
AG GG GG GG AG AG AA
7881780
4789 rs10127971 1 GG GG GG GG GG
GG GG GG GG GG GG GG GG
GG GG GG GG GG GG GG GG
GG GG GG GG GG GG GG GG
GG GG GG GG GG GG GG
5628914
4790 rs10127983 1 GG GG GG AG GG
GG AG AG AA AG AA AG AG
AG AG AG AG GG GG GG GG
AG AG AG AG GG AA AG AG
GG GG GG GG AG GG AG
153923276
4791 rs10128 7 AA AC AC CC AC AC
CC AC AC AC AC CC AC AC
AA AA AC AC AA AA AA AA
AA AA AA AC AA AC AC AA
AA AC AC AC AC AA 130151694
4792 rs10128001 1 GG GG GG GG GG
AG GG GG GG GG GG GG GG
GG GG GG GG GG GG GG GG
GG GG GG GG GG GG GG GG
GG AG GG GG GG GG GG
71544839
4793 rs10128102 10 AC CC CC CC AC
AC CC AC AA AA CC CC CC
CC CC AC AC AC AC CC AC

AA AA AA AC AC AC AC AC
CC AA CC AC CC CC CC
58430237
4794 rs10128131 10 AA AA AA AC AC
AA CC AC AA CC AA AA AC
AA AC AC CC AC AA AA AA
AC AC AA AC AA AC AC AA
AA AA AA AA AA AA AC
1935402
4795 rs10128186 10 AA AA AA AA AG
AA AG AA AA AA AA AA AA
AA AA AG AG AA AA AG AA
GG GG AA AG AG AA AG AA
AA AA AA AA AA AA AG
91520045
4796 rs10128261 10 AA AA AA AG AG
AG AA AG AG AA AG AA AG
AG AG AA AA AA AA AA AA
AA AA AA AA AA AA AA AA
AA AA AA AA AA AA AA
85312966
4797 rs10128290 10 GG GG GG GG GG
GG GG GG GG GG GG GG GG
GG GG GG AG GG GG GG GG
GG GG GG GG GG GG GG GG
GG GG GG GG GG GG GG
130565555
4798 rs10128297 10 GG AG AG AG GG
GG AG AG GG AG GG GG GG
GG AA GG GG GG GG AG GG
AG AG AG AG GG GG GG GG
GG AA GG AG GG AG GG
86845915
4799 rs10128306 10 GG GG GG GG GG
GG GG GG GG GG GG GG GG
GG GG GG GG GG GG GG GG
GG GG GG GG GG GG GG GG
GG GG GG GG GG GG GG
45897857
4800 rs10128322 10 AC CC CC CC CC
CC CC CC CC CC CC CC CC
AC AC CC CC CC CC CC CC
CC CC CC CC CC CC CC CC
CC CC AC AC CC CC CC
43206700
4801 rs1012837 5 GG GG AG GG GG
GG GG GG GG GG GG GG GG
GG GG GG GG GG GG GG GG
GG GG GG GG GG GG GG GG
GG GG GG GG GG GG GG

40010351								
4802	rs10128422		10	AG	AG	AG	AG	AG
	AG	AG	AA	AA	AG	GG	GG	GG
	GG	GG	AA	AG	AG	AG	AG	GG
	GG	AG	AA	AG	AG	AG	AA	AG
	AG	AA	AG	AG	AA	AG	AG	
130947838								
4803	rs1012844		4	GG	GG	GG	AG	AG
	GG	AA	GG	GG	GG	AG	GG	AG
	GG	AG	AA	AG	AG	AG	AG	AA
	AA	AA	AA	AG	AA	AA	AA	AA
	AA	AG	AG	AG	AG	AG	AG	
47952676								
4804	rs10128454		10	AA	AA	AA	AG	AA
	AA	AG	AA	AA	AG	AA	AA	AG
	AG	AG	AA	AA	AA	AA	AA	AA
	AA	AA	AG	AA	AA	GG	AG	AG
	GG	AA	AG	AA	AG	GG	AA	
2794048								
4805	rs1012849		11	AG	AA	AG	AA	AG
	AA	AG	AA	AA	AA	AA	AA	AA
	AG	AA	AA	AA	AA	AA	AA	AA
	AA	AA	AA	AA	AA	AA	AA	AA
	AA	AG	AA	AG	AA	AG	AG	
123349468								
4806	rs10128493		10	AC	CC	AC	AC	AC
	AC	AC	AC	AC	AC	AC	AC	CC
	AC	AC	AC	AC	CC	AC	AA	AA
	CC	CC	CC	CC	CC	CC	CC	CC
	CC	AC	CC	CC	CC	CC	CC	
53276747								
4807	rs10128501		10	AG	AA	AA	GG	AA
	AG	AG	AA	AG	AG	GG	AG	AG
	AG	AG	AA	AG	AG	AG	GG	AA
	AA	AA	AA	AA	AG	AG	GG	AA
	GG	AA	AG	AG	GG	GG	GG	
61508784								
4808	rs10128543		11	GG	GG	GG	GG	AG
	GG	AG	GG	AG	AG	AG	AG	AG
	AG	AG	GG	GG	AG	AG	AG	AG
	GG	GG	GG	AG	AG	AA	AA	AG
	AG	AG	AA	AA	AG	AA	AG	
76420184								
4809	rs10128544		11	AG	AG	AG	AG	AA
	AA	AG	AA	AG	AG	AA	AG	AA
	AG	AA	AG	GG	AG	AA	AA	AA
	AG	AA	AG	AA	AG	AG	AG	GG
	AA	GG	AA	AG	GG	GG	AG	
98461972								
4810	rs10128573		11	AG	AG	AG	GG	GG

	GG	GG	GG	GG	GG	GG	GG	GG
	GG	GG	AA	AA	AG	GG	GG	AG
	GG	GG	GG	GG	GG	AG	AG	GG
	AA	GG	GG	GG	GG	AG	AG	
124470426								
4811	rs10128580		11	AA	AA	AA	AG	AA
	AG	AA	AA	AA	AA	AA	AA	AA
	AA	AA	AA	AA	AA	AA	AG	AA
	AG	AG	AG	AA	AG	AA	AG	AA
	AA	AG	AA	AG	AA	AG	AA	
114115298								
4812	rs10128586		11	GG	AG	AA	AA	AA
	AA	AA	AA	AA	AA	AA	AG	AG
	AA	AG	AA	AA	AA	AA	AA	AA
	AA	AG	AA	AG	AA	AA	AA	AA
	AA	AA	AA	AA	GG	AG	AG	
35245907								
4813	rs10128596		11	AC	AC	CC	CC	CC
	CC	CC	AC	CC	CC	AC	AC	CC
	CC	CC	CC	CC	CC	CC	CC	CC
	AC	AC	AA	AC	AC	AA	AA	AA
	CC	AC	AC	AC	CC	AC	AC	
26770983								
4814	rs10128621		11	GG	GG	GG	GG	GG
	GG	GG	AG	GG	GG	AG	GG	AG
	AG	AG	AG	AG	GG	AG	AG	AG
	GG	AG	GG	AG	GG	AG	AG	AG
	GG	AG	AG	GG	AG	AG	GG	
86979132								
4815	rs10128640		11	AA	AA	AA	AA	AG
	AG	AA	AA	AG	AG	AA	AA	AA
	AG	AA	GG	GG	AG	AA	AA	AA
	AA	AA	AA	AA	AG	GG	AG	AG
	AA	AA	AA	AA	AG	AA	AG	
90086953								
4816	rs10128650		11	AA	AC	AC	AA	AA
	AA	AA	AA	AA	AA	AA	AC	AC
	AA	AC	AA	AA	AA	AA	AA	AA
	AA	AA	AA	AA	AA	AC	AC	AA
	AA	AA	AA	AA	AA	AA	AA	
33932051								
4817	rs10128711							
11	--	--	--	--	--	--	--	--
	--	--	--	--	--	--	--	--
	--	--	--	--	--	--	--	--
	--	--	--	--	--	--	--	--
	--	--	--	--	18632984			
4818	rs10128751		11	AA	AG	AA	AA	GG
	AG	AG	AG	AG	AA	GG	AG	AG
	AG	GG	AG	AG	GG	AG	GG	AG

	AA	AG	AA	AG	AG	AG	GG	AA
	AG	AG	AA	AA	GG	AG	GG	
124112076								
4819	rs1012876		1	CC	AC	AC	AC	AA
	AC	AC	AA	AA	AA	CC	CC	AC
	AA	AC	AA	AA	AA	AC	AA	AC
	AC	AA	AC	AA	AC	AC	AC	AA
	--	AC	AC	AC	AA	AA	AA	
175173206								
4820	rs10128762		12	AA	AA	AA	AG	AG
	AG	AA	AG	AG	--	AG	AA	AG
	AA	AA	GG	AG	AG	AA	AA	AG
	AA	AG	AA	AG	AA	AA	AA	AA
	AA	AA	AG	AG	AA	AG	AA	
74681898								
4821	rs10128809		12	AG	GG	AG	AA	AG
	AG	AA	AG	GG	AG	AG	AA	AA
	AG	AG	AG	AG	AG	GG	GG	GG
	AG	AG	GG	AG	AG	AG	AG	AG
	GG	AA	AA	AA	GG	AG	AG	
43141068								
4822	rs10128825		12	GG	GG	GG	AG	AG
	AA	GG	AG	AA	AA	AG	AG	AG
	GG	GG	AG	AG	AG	AG	GG	GG
	GG	GG	GG	GG	GG	AG	AG	AG
	AG	GG	GG	GG	GG	GG	GG	
59882428								
4823	rs10128877		12	AA	AG	AG	AG	AA
	AG	AG	AA	AA	AG	AA	AA	AA
	AA	AA	AG	AG	AG	AG	AG	AG
	AG	GG	AA	GG	AA	AA	AA	AA
	GG	AA	AA	AA	AG	AG	AA	
66454490								
4824	rs1012891		20	GG	AG	GG	AG	GG
	AG	GG	AG	AG	AG	AA	AG	AG
	AG	GG	AG	GG	GG	AG	GG	GG
	GG	AG	GG	AG	GG	GG	GG	GG
	GG	GG	GG	GG	GG	AG	GG	
7980390								
4825	rs10128931		12	GG	GG	GG	AG	AG
	AG	AG	GG	AG	GG	AG	AG	AG
	GG	GG	AG	GG	GG	GG	AG	GG
	AG	GG	AG	GG	GG	GG	GG	GG
	GG	AG	GG	AG	GG	AG	AG	
46520182								
4826	rs10128967		12	AA	AA	AA	AA	AA
	AA	AA	AA	AA	AA	AA	AA	AA
	AA	AA	AA	AA	AA	AA	AA	AA
	AA	AA	AA	AA	AA	AG	AA	AA
	AA	AA	AA	AA	AA	AA	AA	

48452709
4827 rs10128984 12 GG GG GG AG AG
AG GG GG GG GG GG GG GG
AG GG GG GG GG GG GG GG
GG GG GG GG AA AG AG AG
GG GG GG GG GG GG GG
4696290
4828 rs10129047 12 GG GG GG GG GG
GG GG GG GG GG GG GG GG
GG GG GG GG GG GG GG GG
GG GG GG GG GG GG GG GG
GG GG GG GG GG AG GG
112864028
4829 rs10129050 12 AA AA AA AA AG
AG AA AA AA AA AG AG GG
AA AA AA AG AG AA AG AG
AA AA AA AA AA AG AG AG
AA AG AA AA AA AA AA
26222768
4830 rs1012909 X -- AA AA AA --
AA AG AG AG AA AA AA --
-- -- GG GG GG GG GG --
-- -- -- AG AG GG GG GG
GG -- GG -- GG GG GG
37867772
4831 rs1012911 13 AA AA AA AA AG
AG AA AG AG AG AG GG AG
GG GG AG AG AG AA AA AA
AA AG AA AA AA AA AA AA
AA AG AA AG AG AG AG
57701979
4832 rs10129158 14 GG AG AG GG GG
GG GG GG GG GG GG AG AG
AG GG AG AG GG AG GG AG
GG GG GG GG GG GG GG GG
GG GG GG GG AG AG GG
82382950
4833 rs10129164 14 GG GG AG AG AA
AG AA AG GG AG AG AG AA
AA AA AG GG GG AG AA AG
GG AG AG AG AA AG AG AA
AG AG AG AG AG GG AG
58536728
4834 rs10129176
14 -- -- -- -- -- -- -- --
-- -- -- -- -- -- -- --
-- -- -- -- -- -- -- --
-- -- -- -- -- -- -- --
-- -- -- -- 21298802
4835 rs1012919 14 CC CC AC CC AC

AC CC CC CC CC AA AC AC
AC CC AC AC AC AC AC CC
CC CC AC AC AC CC AC CC
AC AC CC AC CC AC CC
35884639
4836 rs10129213 14 CC CC CC CC CC
CC CC CC CC CC AC CC CC
AC CC CC CC AC CC CC CC
CC CC CC CC CC CC CC CC
AC AC AC AC AC CC AC
24856202
4837 rs10129280 14 AA AA AA AC AC
AC AC CC AC CC AC AC AA
AA AA AC AA AC AA AA AA
CC CC CC CC AC AA AC AC
AA AC AC AA AA AC AC
78068493
4838 rs1012932 10 AG GG GG AG GG
GG AG AG GG AG GG GG GG
GG AG AG GG AG AG AG AG
GG GG AA AA GG AG AG AG
AG AG AG AA GG GG GG
85812975
4839 rs1012934 14 GG GG GG GG GG
GG GG GG GG GG GG GG GG
GG GG GG GG GG GG GG GG
GG GG GG GG GG AG AG GG
GG AG GG AG GG AG AG
54568041
4840 rs10129344 14 AA AG AG AG AA
AG AA AG AG AG GG GG GG
GG AA GG GG AG AA AG AA
AA AA AG AG AG AG GG AA
AG GG GG GG AG AG AG
33427617
4841 rs1012941 21 AA AA AA AA AA
AA AA AA AA AA AA AA AA
AA AA AA AA AA AA AA AA
AA AA AA AA AA AA AA AA
AA AA AA AA AA AA AA
36263999
4842 rs10129433 14 CC CC CC CC CC
CC CC CC CC CC CC CC CC
CC CC CC CC CC CC CC CC
CC CC CC CC CC CC CC CC
CC CC CC CC CC CC CC
99345445
4843 rs1012944 21 AG AG AG GG GG
AG GG AG AG AG GG GG GG
GG GG AG GG GG GG AG AG

AG AG AG AG GG GG GG GG
AA AG GG GG AG GG AG
35825266
4844 rs10129446 14 AC AC AA AC AC
AC AC AA AC AA AC CC AC
CC AC CC AC AC CC AC AC
AA AA AA AA CC CC CC CC
AC AA AC AA AC CC AC
80212154
4845 rs10129472 14 GG AG GG GG GG
GG GG GG GG GG GG GG GG
GG GG GG GG GG GG GG GG
GG GG GG GG GG GG GG GG
GG GG GG GG GG GG AG
66188209
4846 rs10129511 14 GG GG AG GG GG
GG GG AG AG GG GG GG GG
AG AG GG AG AG GG GG GG
GG AG GG AG GG GG GG GG
GG GG GG GG GG GG GG
63829461
4847 rs10129516 14 GG GG GG GG GG
GG GG GG GG GG GG GG GG
GG GG GG GG GG GG GG GG
GG GG GG GG GG GG GG GG
AG GG GG GG GG GG GG
63600090
4848 rs1012954 21 CC CC CC CC CC
CC CC CC AC AC CC CC CC
CC CC CC CC CC CC CC CC
CC CC CC CC CC CC CC CC
CC CC CC CC AC CC AC
20823060
4849 rs10129595 14 GG GG AG GG GG
GG GG GG GG GG GG GG GG
GG GG GG AG GG GG GG GG
GG GG GG GG GG GG GG GG
AG GG GG GG AG AG GG
65257272
4850 rs10129606 14 AG GG GG AG AA
AG AG AA AA AA AG AG AG
AG AG AG AA AG AG GG GG
AG AA AG GG AA AG AG AA
GG AA AA AA GG GG AG
22756270
4851 rs10129659 14 AA AA AA AA AA
AA AA AA AA AA AA AA AA
AA AA AA AA AA AG AA AA
AA AA AA AA AA AA AA AA
AA AA AA AA AA AA AA

42746119
4852 rs10129687 14 AA AA AA AA AA
AA AA AA AA AA AA AA AA
AA AA AA AA AA AA AA AA
AA AA AA AA AA AA AA AA
AA AA AA AA AA AA AA
50171626
4853 rs1012969 21 AG AA AG AG AG
AA AG AG AG GG AA AA AA
AA AA GG AG AG AG AG GG
AA AA AG AG AA AG AA AA
AG AA AA AA AG AG AA
31996108
4854 rs10129694 14 AA AA AA AA AA
AA AA AA AA AA AA AA AA
AA AC AA AA AA AA AA AA
AA AA AA AA AA AC AC AA
AA AC AA AC AA AC AC
54607345
4855 rs1012971 12 AG AG AG AG GG
GG AG AG AG AG AG GG AG
AA AG AG AA AA AA GG AA
AA AA AG AA GG GG GG GG
AG AG GG GG GG GG GG
97827796
4856 rs10129724 14 GG GG GG GG GG
GG GG GG GG GG GG GG GG
GG GG GG GG GG GG GG GG
GG GG GG GG GG GG GG GG
GG GG GG GG GG GG GG
55290372
4857 rs10129747 14 AA AA AA AG AG
AA GG AG AG AG AA AA AA
AA AA GG AG AG AG AG AA
GG AA GG AG GG AG GG AG
AG AG AA AA GG GG GG
77433198
4858 rs10129761 14 AG GG GG AA AG
AG AA AG AA AG AG AG AA
AA AG AA AA AA AA AG AG
AA AA AA AA AG AA AA AG
AG AA AG AG AA AG AA
103731797
4859 rs10129913 14 AA AG AG AA AA
AA AA AA AA AA AA AA AA
AA AA AG AG AG AA AA AA
AA AA AA AA AA AA AA AA
AA AA AA AA AA AA AA
93796301
4860 rs10129941 14 AA AA AA AA AA

	AA	AA	AA	AA	AA	AA	AA	AA
	AA	AA	AA	AA	AA	AA	AA	AA
	AA	AA	AA	AA	AA	AA	AA	AA
	AA	AA	AA	AA	AA	AA	AA	
23329266								
4861	rs10129955		14	AC	CC	AC	AA	AC
	AC	AA	AC	AA	AC	CC	AC	AC
	AC	AC	AA	AA	AA	AA	AC	AA
	AC	AC	CC	CC	CC	CC	CC	CC
	AC	AA	AC	AC	CC	AC	AC	
33895784								
4862	rs10129968		14	AA	AA	AA	AA	AA
	AA	AA	AA	AA	AA	AA	AA	AA
	AA	AA	AA	AA	AA	AA	AA	AA
	AA	AA	AA	AA	AA	AA	AA	AA
	AA	AA	AA	AA	AA	AA	AA	
59117756								
4863	rs1012998		7	AG	AG	AG	AG	AA
	AG	AA	GG	GG	GG	AG	AG	GG
	AG	AG	AG	AG	AG	AG	AA	GG
	AG	AG	GG	AG	AG	AG	AA	AA
	AG	AA	AA	AA	AA	AA	AA	
10621699								
4864	rs10130000		14	CC	CC	CC	CC	CC
	CC	CC	CC	CC	CC	CC	CC	CC
	CC	CC	CC	CC	CC	CC	CC	CC
	CC	CC	CC	CC	CC	CC	CC	CC
	CC	CC	CC	CC	CC	CC	CC	
35068168								
4865	rs10130003		14	AA	AG	AA	AG	AG
	AA	AG	AA	AA	AA	AA	AA	AA
	AA	AA	AA	AA	AA	AA	AA	AA
	AA	AG	AG	AG	AA	AA	AA	AA
	AA	AA	AG	AG	AA	AA	AA	
95382880								
4866	rs10130009		14	GG	GG	GG	GG	GG
	GG	GG	GG	GG	GG	GG	GG	GG
	GG	GG	GG	GG	GG	GG	GG	AG
	GG	GG	GG	GG	GG	GG	GG	GG
	AG	GG	GG	GG	GG	GG	GG	
88111177								
4867	rs1013001		21	AG	AA	AG	GG	GG
	GG	GG	GG	GG	GG	GG	GG	GG
	AG	GG	AG	AG	GG	AG	AG	AG
	GG	GG	GG	GG	GG	GG	GG	GG
	GG	GG	GG	GG	AG	AG	GG	
15659254								
4868	rs10130063		14	AA	AA	AA	AA	AA
	AA	AA	AA	AA	AA	AA	AA	AA
	AA	AA	AA	AA	AA	AA	AA	AA

	AA	AA	AA	AA	AA	AA	AA	AA
	AA	AA	AA	AA	AA	AA	AA	
52148758								
4869	rs10130070		14	AA	AA	AG	GG	GG
	GG	GG	AG	AG	AG	GG	AG	AG
	AA	AG	AG	AG	AG	AA	AG	AG
	AG	GG	AG	AG	AG	GG	GG	AG
	AG	AA	AG	AG	AG	AA	AG	
99397996								
4870	rs1013009		5	AA	AA	AA	AA	AA
	AA	AA	AA	AA	AA	AC	AA	AC
	AC	AC	AA	AA	AA	AA	AA	AA
	AA	AC	AA	AA	AA	AA	AA	AA
	AA	AA	AA	AA	AA	AC	AA	
80704269								
4871	rs10130091		14	GG	AG	AG	AG	AA
	AA	AG	GG	GG	GG	GG	GG	GG
	GG	GG	AG	AG	GG	GG	GG	GG
	AG	AG	AG	GG	GG	AG	AG	GG
	AA	AG	GG	GG	AG	AG	GG	
74734625								
4872	rs10130093		14	GG	GG	GG	AG	AG
	AA	GG	AG	AG	AG	GG	GG	GG
	GG	GG	GG	GG	GG	AG	GG	AA
	AG	GG	AG	AG	AG	GG	AG	GG
	GG	AG	GG	AG	GG	GG	AG	
89799122								
4873	rs10130129		14	AA	AA	AA	AA	AA
	--	AA	AC	AC	CC	AC	AC	AC
	AA	AC	AC	AA	AA	AC	AC	AC
	AC	AA	AA	AC	AC	AA	AC	AA
	AA	AA	AA	AA	AA	AC	AA	
21228818								
4874	rs1013013		17	AG	AG	GG	AG	GG
	GG	AG	GG	GG	GG	GG	GG	GG
	AG	AG	GG	AG	AG	AG	AG	GG
	GG	GG	AG	AG	AG	GG	AG	GG
	AG	AG	--	AG	AG	AG	GG	
72912862								
4875	rs10130202		14	AA	AA	AA	AA	AA
	AA	AA	AA	AA	AA	AA	AA	AA
	AA	AA	AA	AA	AA	AA	AA	AA
	AA	AA	AA	AA	AG	AA	AG	AA
	AA	AA	AA	AA	AA	AA	AA	
36165418								
4876	rs10130206		14	AA	AG	AA	AG	AG
	AG	AG	AA	AA	AA	AA	AA	AA
	AA	AA	AA	AA	AA	AA	AG	AA
	AA	AG	AA	AA	AG	AA	AG	AG
	AA	AA	AA	AA	AG	GG	AA	

67169471
4877 rs10130245 14 AG GG GG GG GG
GG GG GG GG GG GG GG GG
GG AG GG GG GG GG GG GG
GG GG GG GG AG GG GG AG
GG GG GG GG GG GG GG
94249470
4878 rs10130248 14 AA AG AG AG GG
AG GG AG AG AG AA AG AA
AA AG GG AG GG AG AG AA
AA AA AA AA GG AG AG GG
AA AG AG AA AG AG AG
28119601
4879 rs10130324 14 AC AC AC CC CC
CC CC AC CC AC CC AC AC
AC CC CC AC CC CC CC CC
CC CC CC CC CC CC CC CC
CC CC AA AC CC CC CC
40118909
4880 rs1013033 X AA AG GG AA GG
AA AG AG AG AA GG GG GG
AG AG GG GG GG AG GG AA
AA AA AA AG AA GG AG AA
AG GG GG GG AG AA GG
85106265
4881 rs10130335 14 AA AA AA AA AA
AA AA AA AA AA AA AA AA
AA AA AA AA AA AA AC AC
AC AA AA AA AA AA AA AA
AA AA AA AA AA AA AA
96856579
4882 rs10130379 14 GG GG GG GG GG
GG GG GG GG GG GG GG GG
GG GG GG GG GG GG GG GG
GG GG GG GG GG GG GG GG
GG GG GG GG GG GG GG
103492332
4883 rs1013038 22 GG GG AG AG GG
GG AG GG AG GG GG GG GG
GG AG GG AG AG GG GG GG
GG GG GG GG GG GG GG GG
AG GG AG AG AG AG AG
44123502
4884 rs10130441 14 AA AA AA AA AA
AA AA AA AA AA AA -- AA
AA AA -- AA AG AA -- --
-- -- -- AA AA AA AA AA
AA AA AA AA AA AA AA
83974320
4885 rs10130473 14 GG GG GG GG GG

GG GG GG GG GG AG GG AG
AG GG GG GG GG GG GG GG
AG AG GG GG GG GG GG GG
GG GG GG GG GG GG GG
53970655
4886 rs10130504 14 AA AA AA AA AA
AA AA AG AA AG AG AA AG
AG AG AG AG AA AG AG GG
AG AA AA AA AA GG AG AG
AG AG AA AA AG GG AA
35061728
4887 rs10130539 14 GG AG GG AA AA
AA AA AG AA AG AA AG AG
AG AG AA AA AA AA AA AG
AG AA AA AA AA AA AA AA
AA AA AG AG AA AA AA
41119990
4888 rs10130575 14 GG GG GG GG GG
GG GG GG GG GG GG GG GG
GG GG GG GG GG GG GG GG
GG AG GG GG AG AG AA AG
GG GG GG GG AG AG GG
59485903
4889 rs10130644 14 AA AA AG AG AG
AA GG AG AG AG GG AA AG
AG AG AG AG AG AG AG GG
AA AA AA AA AG GG GG AG
AG AG AG AA GG GG GG
91128309
4890 rs10130689 14 GG GG GG GG GG
GG GG GG GG GG AA AG AG
AG GG GG GG GG GG AG GG
AG AG AG AG GG GG GG GG
GG AG GG GG AA AG AA
51391471
4891 rs10130699 14 AA AG AA AG AG
AG AG AA AG AA GG GG GG
GG AG GG GG GG AG AA AG
AA AA AA AA AA AA AA AA
AG AA AG AG AG AG AG
37420572
4892 rs10130729 14 AG AA AG AG GG
GG AG GG AG GG GG GG GG
GG GG GG AG GG AG AG AG
GG GG GG GG GG GG GG GG
AA AG GG GG GG GG GG
70307792
4893 rs10130737 14 AA AA AA AA AA
AA AA AA AA AA AA AA AA
AA AA GG AG AG AG AA AA

AG AA AA AA AA AA AA AA
AA AA AA AA AA AA AA
28334694
4894 rs10130789 14 AA AA AG GG AA
AG AG AG GG AG AA AA AA
AG AG AA AA AA AA AG AA
AG AA AA AA AG AA AG AA
GG AA AG AG AA AA AG
93610191
4895 rs10130801 14 AA AA AG AA AG
AG AA AG AG AG AG AG AG
AG AA AG AG AG AG AG AG
AA AA AA AA AA AG AA AG
AA AG AA AA AG GG AA
88528910
4896 rs10130812
14 -- -- -- -- -- -- -- --
-- -- -- -- -- -- -- --
-- -- -- -- -- -- -- --
-- -- -- -- -- -- -- --
-- -- -- -- 81909200
4897 rs10130832 14 GG GG GG GG GG
GG AG GG GG GG AG AG AG
GG GG GG AG AG GG AG GG
GG GG GG GG GG GG GG GG
GG GG GG GG GG GG AG
97564255
4898 rs10130838 14 GG GG AG GG GG
GG GG GG GG GG AG AG AA
AA AG AG AG AG AG GG GG
GG GG GG GG GG GG GG GG
GG GG GG GG AG GG AG
101487504
4899 rs10130854 14 CC CC CC CC CC
CC CC CC CC CC CC CC CC
CC AC AC CC CC AC CC AA
AC AC CC CC CC AA AC AC
CC CC AC AC AC CC AC
47061822
4900 rs10130856 14 AC AC AA AC AA
AC AA AA AA AA AA AA AA
AA AA AA AA AA AC AC AA
AA AC AA AC AC AC AC AC
AC AA AA AA AA AC AA
83020296
4901 rs10130881 14 AA AA AA GG GG
GG GG AA AA AA AA AA AA
AA AA AA AA AA AG AG AA
AG AG AG AG AG GG GG AG
AA AG AG AG AG AA AG

34017669
4902 rs10130949 14 GG GG GG GG GG
GG GG GG GG GG GG GG GG
GG GG GG GG GG GG GG GG
GG GG GG GG GG GG GG GG
GG GG GG GG GG GG GG
103517195
4903 rs10131135 14 GG GG GG GG GG
GG GG AG GG AG AG GG AG
GG GG AG AG AG GG GG GG
GG GG GG GG GG GG GG GG
AG GG GG GG AG AG GG
97717525
4904 rs10131165 14 AA AA AA AA AA
AA AA AA AA AA AA AA AA
AA AA AA AA AA AA AA AG
AA AA AA AA AA AA AA AA
AA AA AA AA AA AA AA
83048812
4905 rs10131167
14 -- -- -- -- -- -- -- --
-- -- -- -- -- -- -- --
-- -- -- -- -- -- -- --
-- -- -- -- -- -- -- --
-- -- -- -- 21399577
4906 rs10131172 14 GG GG GG AG AG
GG AA GG AG GG AG GG AG
GG AG GG GG GG GG GG AG
AG AG AG GG AG GG AG GG
GG AG GG AG GG AG GG
24854692
4907 rs10131212 14 GG GG GG GG AG
GG AG GG GG GG GG GG GG
GG GG GG GG GG GG AG GG
GG GG GG GG GG GG GG GG
GG GG GG GG GG GG GG
67792367
4908 rs10131217 14 -- -- AG GG GG
GG -- GG -- GG GG GG GG
-- -- -- -- GG -- -- --
-- -- -- GG AG -- AG AG
GG -- AG AG -- AG GG
62673825
4909 rs10131230 14 AG GG GG GG AA
AG AG GG GG GG AG GG AG
AG AG AG AG AG AG AG AG
GG GG GG GG AG AG AA AG
GG GG GG GG GG GG GG
52166742
4910 rs1013124 17 AC AA AA AA AA

AA AA AC AC AC AA AA AA
AA AA AA AA AC AA AA AA
AA AC AC AC AA AA AA AA
AA AA AA AA AC AC AA
13525001
4911 rs10131241 14 AA AA AA AA AG
AG AA AA AG AA AA AG AA
AA AA AA AA AA AA AG AA
AA AA AA AA AA AA AA AA
AA AA AA AA AG AG AA
28769405
4912 rs10131260 14 CC CC CC CC AC
AC CC CC CC CC CC CC CC
AC AA CC CC AC CC CC CC
CC CC CC CC AC CC CC CC
CC CC CC CC CC CC AC
62974709
4913 rs10131278 14 GG AG AG GG AG
AG AG GG GG GG GG AA AG
AG GG GG GG GG AG AG AG
GG AG AG AG GG AA AG AG
GG GG AA AG AG AG AG
96543481
4914 rs1013129 21 GG AG GG AG AG
AG GG GG GG GG AG GG AG
AG AG GG GG GG GG GG AG
GG GG GG GG GG GG GG GG
GG GG AG GG GG AG GG
40498109
4915 rs10131300 14 AA AA AA AA AA
AA AA AC AA AC AA AA AA
AA AA CC AC AC AC AA AC
AC AC AC AC AA AA AA AA
AA AC AA AC AA AC AA
72939495
4916 rs10131337 14 AG GG AG GG AG
AG GG AA AG AA AG AG GG
GG GG GG GG GG GG GG GG
GG GG GG GG GG GG GG GG
GG GG GG GG AG GG AG
37144516
4917 rs10131371 14 CC CC CC CC AC
AC CC CC CC CC CC CC CC
CC CC CC CC CC CC CC CC
CC CC CC CC CC CC CC CC
CC CC CC CC AC CC AC
83587297
4918 rs10131380 14 GG GG GG GG GG
GG GG GG GG GG GG GG GG
GG GG GG GG GG GG GG GG

	GG	GG	GG	GG	GG	GG	GG	GG
	GG	GG	GG	GG	GG	GG	GG	
105920871								
4919	rs1013144		15	AA	AA	AA	AA	AA
	AA	AA	AA	AA	AA	AA	AA	AA
	AA	AA	AA	AA	AA	AA	AA	AA
	AA	AA	AA	AA	AA	AA	AA	AA
	AA	AC	AA	AA	AA	AA	AA	
29595240								
4920	rs10131444		14	GG	GG	GG	GG	GG
	GG	GG	GG	AG	GG	GG	GG	GG
	GG	AG	GG	GG	GG	AG	GG	GG
	GG	GG	GG	GG	GG	AG	GG	AG
	GG	GG	GG	GG	GG	AG	GG	
55970787								
4921	rs10131470		14	AC	AA	AA	AC	AA
	AC	AA	AA	AA	AA	AA	AA	AA
	AA	AA	AC	AA	AC	AA	AA	AC
	AA	AC	AA	AA	AA	AA	AA	AA
	AC	AA	AC	AC	AC	AC	AC	
82260546								
4922	rs10131536		14	AA	AA	AA	AG	AG
	AG	AA	AG	AG	AA	AA	AG	AA
	AA	AA	AG	GG	AG	AG	AA	AA
	AG	AG	AA	AA	AG	AG	GG	AA
	AA	AA	AG	AG	AA	AG	AA	
22313016								
4923	rs10131598		14	AG	AG	AG	AA	AA
	AA	AA	AG	AG	AG	AG	AG	AA
	AA	AA	AA	AA	AA	AA	AG	AA
	AG	AA	AA	AA	AA	AA	AA	AA
	AA	AG	AA	AA	GG	AG	GG	
51358794								
4924	rs10131610		14	CC	CC	CC	CC	CC
	CC	CC	AC	CC	AC	CC	CC	CC
	CC	CC	CC	CC	CC	CC	CC	CC
	AA	CC	AC	AC	CC	CC	CC	CC
	CC	CC	CC	CC	CC	CC	CC	
94501249								
4925	rs1013164		2	AA	AG	AG	AA	AG
	AG	AA	AG	AG	AG	GG	GG	GG
	AA	AA	AG	AG	AA	AG	AG	AG
	AG	GG	AG	AG	AG	AG	AA	GG
	GG	AA	AG	AG	AG	AG	AG	
51355911								
4926	rs10131659		14	AG	GG	GG	AG	AG
	AA	AG	AA	AG	AA	GG	GG	GG
	AG	AA	AA	AG	AA	AG	AG	GG
	GG	GG	GG	GG	AA	AG	AG	AA
	AG	AG	GG	GG	GG	GG	GG	

62939312								
4927	rs10131663		14	AA	AA	AA	AA	AA
	AA	AA	AA	AA	AA	AA	AA	AA
	AA	AA	AG	AA	AA	AG	AG	AG
	AG	AA	AG	AA	AG	AA	AG	AA
	AA	AA	AA	AA	AA	AA	AA	
86692923								
4928	rs10131715		14	GG	AG	GG	GG	GG
	GG	GG	GG	AG	AG	AG	AG	GG
	GG	GG	GG	AG	GG	GG	AG	GG
	AG	AA	AG	AG	AA	GG	AG	AG
	GG	AG	AG	AG	AG	AG	AG	
66157373								
4929	rs10131762		14	GG	GG	GG	GG	GG
	GG	GG	GG	GG	GG	GG	GG	GG
	GG	GG	GG	GG	GG	AG	GG	AG
	GG	GG	GG	GG	GG	GG	GG	GG
	GG	GG	GG	GG	GG	GG	GG	
97258521								
4930	rs10131789		14	AG	AA	AA	GG	AA
	AG	AG	AA	AA	AA	AG	AG	AA
	AA	AA	AA	AA	AA	AA	AA	AA
	AA	AA	AA	AA	AA	AG	AA	AG
	AA	AA	AA	AA	AA	AA	AA	
68977500								
4931	rs10131809		14	AA	AA	AA	AA	AG
	AG	AA	AA	AG	AA	AA	AG	AA
	AA	AA	AA	AA	AA	AA	AG	AA
	AA	AA	AA	AA	AA	AA	AA	AA
	AA	AA	AG	AG	AG	AG	AA	
28790430								
4932	rs10131858		14	AG	AA	AA	AG	AA
	AG	AA	AA	AA	AA	GG	GG	AG
	AA	AA	GG	AG	AG	GG	GG	AG
	AA	AA	AA	AA	AA	GG	AG	AG
	AG	AA	GG	AG	AA	AA	AA	
71151044								
4933	rs1013187		2	AC	AA	AA	AA	AC
	AC	AA	AA	AA	AA	AA	AC	AA
	AA	AA	AA	AA	AA	AA	AC	AA
	AA	AA	AA	AA	AA	AA	AA	AA
	AC	AA	AA	AA	AC	AA	AC	
41059165								
4934	rs10131882		14	AG	AG	AA	AA	AA
	AA	AA	AG	GG	AG	AA	AG	AG
	GG	GG	AA	AA	AA	AA	AG	AA
	AA	GG	AG	AG	AG	AG	GG	AA
	AA	AA	AA	AA	AG	AG	AA	
94325860								
4935	rs10131911		14	AG	AA	AA	AA	AG

	AA	AG	AG	AG	AG	AG	AG	AG
	GG	AA	GG	AG	AG	GG	AA	AG
	AA	AG	AG	AG	AA	AG	AA	AG
	AG	GG	AA	AG	AA	AG	AA	
29033633								
4936	rs10131919		14	GG	AG	AA	AA	AG
	AA	AG	AA	AA	AA	AG	AG	AG
	AG	AA	AG	AA	AA	AG	AG	AA
	AG	AA	AG	AA	AA	AA	AA	AA
	AA	AA	GG	AG	AG	AA	AG	
90186879								
4937	rs1013193		5	AA	AA	AA	AA	AA
	AA	AA	AA	AA	AA	AA	AA	AA
	AA	AA	AA	AA	AA	AA	AA	AA
	AA	AA	AA	AA	AA	AA	AA	AA
	AG	AG	AA	AA	AA	AA	AA	
169199323								
4938	rs10131977		14	AG	GG	AG	AA	AA
	AA	AA	AG	AA	AG	GG	AG	GG
	AG	GG	GG	GG	AG	AA	AA	AA
	AG	AG	AG	AG	AA	AG	AA	AG
	GG	AG	AA	AA	AA	AG	AA	
30840016								
4939	rs10132025		14	AG	AG	GG	GG	AA
	AG	AG	GG	GG	GG	AG	AG	GG
	GG	GG	GG	AG	GG	GG	GG	GG
	GG	GG	AG	AG	GG	GG	GG	GG
	GG	AA	AG	AA	GG	GG	AG	
33792629								
4940	rs1013210		8	GG	AG	GG	AA	AA
	AG	AG	AG	GG	GG	GG	GG	GG
	GG	GG	GG	AG	AG	AG	GG	AG
	GG	GG	GG	GG	AG	GG	GG	GG
	AA	GG	GG	GG	GG	GG	AG	
24116722								
4941	rs10132103		14	GG	AG	AG	AG	AG
	AG	AG	AA	AG	AA	GG	AG	AG
	--	--	AA	AA	AG	--	AG	--
	AG	AG	AG	GG	GG	AG	--	AG
	AA	AG	AG	AG	AG	AG	--	
74726710								
4942	rs10132160		14	AA	AG	GG	GG	GG
	GG	AG	GG	GG	GG	GG	GG	GG
	GG	GG	GG	GG	GG	AG	GG	GG
	GG	GG	GG	GG	AG	GG	AG	GG
	GG	GG	GG	GG	GG	GG	GG	
103058023								
4943	rs10132229		14	AA	AA	AA	AG	AA
	AG	AA	AG	AG	AA	AA	AA	AA
	AG	AG	AA	AA	AA	AA	AA	AA

AG AG AG AG AA AA AA AA
AG AG AA AG AA AA AG
65777560
4944 rs10132233 14 AG AG AG GG GG
GG GG GG GG GG AG GG AG
AG GG GG GG GG GG GG GG
GG GG AG AG AG GG AG GG
AG GG GG GG GG GG GG
34895977
4945 rs10132264 14 AG AG GG AG AA
AG AA AG AG AG AA GG AG
AG AA AG AA AA AG AG GG
AG AA AG AA GG GG GG GG
AG AA AA AA AA AA AG
86923368
4946 rs10132267 14 AA AC AA AC AC
CC AA AA AA AA AA AC AC
AC CC AA AA AA AA AC AA
AC AC AC AC AA AC AA AC
AC AA AC AA AA AC AC
55185767
4947 rs10132280 14 AC AC AC AC CC
CC AC AC AC AC AC CC CC
CC CC CC AC CC CC AC AC
AC AC AC AA AC CC CC AC
AC CC AC CC CC CC CC
25928179
4948 rs10132295 14 AA AA AA AA AA
AA AA AA AA AA AA AA AA
AA AA AG AG AG AA AA AA
AA AA AA AA AA AA AA AA
AA AA AG AA AA AA AA
66604368
4949 rs10132309 14 CC CC CC CC CC
CC CC CC CC CC CC CC CC
CC CC CC CC CC CC CC CC
CC AC AC AC CC CC CC CC
CC CC CC CC CC CC CC
101464984
4950 rs10132321 14 AA AA AA AA AA
AA AA AA AA AA AA AA AA
AA AA AA AA AA AA AA AA
AA AA AA AA AA AA AA AA
AA AA AA AA AA AA AA
59783246
4951 rs10132368 14 GG GG GG GG GG
GG GG AG AG GG GG GG GG
GG GG GG AG GG GG GG GG
AG AG GG GG GG AG GG AG
GG GG AG AG GG GG GG

23645718								
4952	rs10132370		14	AC	CC	CC	AC	AC
	AC	AC	AC	AC	AC	AA	AA	AA
	AC	CC	AA	AA	AA	AA	AC	AC
	AC	AC	CC	CC	AC	AC	CC	AC
	AC	AC	CC	AC	AC	CC	AA	
57127513								
4953	rs1013240		16	AA	AA	AA	AA	AG
	AG	AA	AG	AA	AG	AA	AA	AA
	AG	AA	GG	GG	AG	AA	AG	AA
	AA	AG	AG	AA	AA	AA	AA	AA
	GG	AA	AA	AA	AA	AG	AA	
8229813								
4954	rs10132439		14	GG	GG	GG	GG	GG
	GG	GG	GG	GG	GG	GG	GG	GG
	GG	GG	GG	GG	GG	GG	GG	GG
	GG	GG	GG	GG	GG	GG	GG	GG
	GG	GG	GG	GG	GG	GG	GG	
37056763								
4955	rs10132473		14	AA	AA	AA	AA	AA
	AA	AA	AA	AA	AA	AA	AA	AA
	AA	AA	AA	AA	AA	AA	AA	AA
	AA	AA	AA	AA	AA	AA	AA	AA
	AA	AA	AA	AA	AA	AA	AA	
66643917								
4956	rs1013251		14	GG	AG	GG	AG	AA
	AG	AA	GG	GG	GG	AG	AA	AG
	AG	AG	GG	GG	GG	GG	AG	AG
	GG	GG	GG	GG	GG	AG	GG	AG
	AG	GG	AG	GG	GG	GG	GG	
32472089								
4957	rs10132530		14	AA	AA	AA	AA	AA
	AA	AA	AA	AA	AA	AA	AA	AA
	AA	AA	AA	AA	AA	AA	AA	AA
	AA	AA	AA	AA	AA	AA	AA	AA
	--	AA	AA	AA	AA	AA	AA	
54279433								
4958	rs10132534		14	AG	AG	AG	AA	GG
	AG	AG	GG	GG	GG	AG	GG	AG
	GG	GG	GG	GG	GG	GG	GG	GG
	GG	GG	GG	GG	GG	GG	GG	GG
	GG	GG	AG	AG	GG	GG	GG	
94364287								
4959	rs10132541		14	AG	AG	AA	AA	GG
	AG	AG	AG	AG	AG	GG	GG	GG
	GG	GG	GG	GG	GG	AG	AG	GG
	AG	GG	GG	GG	GG	AA	AG	AG
	GG	AG	GG	GG	GG	GG	GG	
53770644								
4960	rs10132588		14	CC	CC	CC	CC	CC

CC CC CC CC CC CC CC CC
CC CC CC CC CC CC CC CC
CC CC CC CC CC CC CC CC
CC CC CC CC CC CC CC
99163563
4961 rs10132640 14 AA AA AA AG AA
AG AA AA AA AA AA AA AA
AA AG AA AA AA AG AG AA
AG AA AG AG AA AA AA AA
AA AA AA AA AA AA AA
93684814
4962 rs10132660 14 AA AA AA AG AG
GG AA AA AA AA AA AA AA
AA AA AA AA AA GG GG AG
GG AG GG GG AA AA AA AA
AA AG AG AA AA AA AA
75948416
4963 rs10132666 14 AA AC AC CC CC
CC CC CC CC CC CC CC CC
CC CC AC CC CC CC CC CC
CC CC CC CC CC AC CC AC
CC CC CC CC CC CC CC
59458059
4964 rs10132674 14 AG AG AG AG GG
AG GG AG GG AG GG GG GG
GG GG GG GG GG GG GG GG
GG GG GG GG GG AG GG AG
GG GG AG GG AA AG AG
32372496
4965 rs10132679 14 AG GG GG AA AA
AA AG AG AG AA AG AG AA
AA AA AG AG AA GG AG AG
AG GG AG GG GG AG AG GG
AA GG GG GG AA AA AA
63677622
4966 rs10132712 14 GG GG GG GG GG
GG GG GG GG GG GG GG GG
GG GG GG GG GG GG GG GG
GG GG GG GG GG GG GG GG
GG GG GG GG GG GG GG
54124068
4967 rs10132721 14 AA AA AA AA AA
AA AA AA AA AA AA AA AA
AA AA AA AA AA AA AA AA
AA AA AA AA AA AA AA AA
AA AA AA AA AA AA AA
56039000
4968 rs1013273 11 AA AG AG AA AG
AG AA AG AA AA AA AA AG
AA AG GG AG GG AG AG AA

GG AG AG AA AA AA AA AA
AA AA AA AA GG AG GG
81534503
4969 rs10132763 14 AG AA AG AG GG
AG GG GG GG GG GG GG GG
GG GG AG AG AG AG AG AG
AA AG AA AA AA AA AA AA
GG GG AG AG AG AG AG
87853446
4970 rs10132767 14 AG GG AG AG AG
AA GG AG GG AG GG GG AG
AA AG AA AA AG AA AG AA
AG AG AG AG AA GG AG AG
AG AA AG AA AG AA AG
62639984
4971 rs1013282 9 AG AG AG GG AG
AG GG AG GG AG AG AA AG
AG AG AA AG AG AG AG AA
AG AG AA AG GG GG GG GG
GG GG GG GG AG AG GG
122426409
4972 rs10132870 14 AA AA AA AA AA
AA AA AA AA AA AA AA AA
AA AA AA AA AA AA AA AA
AA AA AA AA AA AA AA AA
AA AA AA AA AA AA AA
103368103
4973 rs10132900 14 GG GG AG AG GG
GG AG GG GG GG GG GG GG
AG AG GG GG GG GG GG GG
GG GG GG GG GG AG GG AG
AG GG GG GG AA AG AG
78307336
4974 rs10132916 14 GG AG AG AG AA
AG AA AA AA AA AG AA AA
AA AA AA AG AG AA AA AA
AA AG AA AA AG AA AG AA
AG GG GG GG GG AG GG
101514371
4975 rs10132990 14 AG AG AA AA AG
AG AG AG AG AG AG AA AG
AG AG GG AG GG AA AA --
AA AA AA AA AA AG AA AG
AG AG AG AG -- -- AA
21982677
4976 rs1013303 6 GG AG GG AA AA
AA AA AA AA AA AG AA AA
GG AA AG AA AG GG AG AG
AG AG AG AG AA GG AG AG
AG GG AA AG AG AA GG

1749928								
4977	rs10133034		14	AA	AA	AG	AG	AG
	GG	AA	GG	AG	GG	AG	GG	AG
	AG	AG	AG	AG	GG	AG	AG	AG
	AG	GG	AG	GG	GG	GG	GG	GG
	AG	AG	GG	AG	AG	AA	GG	
81264861								
4978	rs10133043							
14	--	--	--	--	--	--	--	--
	--	--	--	--	--	--	--	--
	--	--	--	--	--	--	--	--
	--	--	--	--	--	--	--	--
	--	--	--	--	74230592			
4979	rs1013307		7	AG	AG	AA	AA	AG
	AG	AG	AA	AA	AA	AG	AG	AA
	AA	AA	AA	AA	AA	AA	AA	AA
	AA	AA	AA	AA	AA	AA	AA	AA
	AA	AA	AA	AA	AA	AA	AG	
108260762								
4980	rs10133092		14	AG	GG	GG	AG	AA
	AG	AG	AG	GG	AA	GG	GG	GG
	AG	AG	AG	AG	GG	GG	AG	GG
	GG	AG	GG	AG	GG	AG	AG	GG
	AA	GG	AG	AG	GG	AG	GG	
63434918								
4981	rs10133101		14	AG	AG	AA	AA	AA
	AA	AA	AG	AG	AG	AA	AA	AA
	AA	AA	AG	AG	AA	AA	AA	AG
	AA	AG	AA	AA	AA	AG	AA	AG
	AA	AA	AA	AA	AA	AA	AA	
79680160								
4982	rs10133111		14	AG	GG	GG	GG	GG
	GG	GG	GG	GG	GG	GG	GG	GG
	GG	GG	GG	GG	GG	GG	GG	GG
	GG	GG	GG	GG	AG	GG	GG	AG
	GG	GG	AG	GG	GG	AG	GG	
103377321								
4983	rs10133120		14	CC	CC	CC	CC	AC
	CC	AC	CC	CC	CC	CC	CC	CC
	CC	CC	CC	CC	CC	CC	AC	CC
	CC	CC	CC	CC	CC	CC	CC	CC
	CC	CC	CC	CC	CC	CC	CC	
67115671								
4984	rs10133171		14	AG	AA	AG	GG	GG
	GG	GG	GG	GG	GG	AG	GG	GG
	AG	AA	GG	GG	GG	GG	AG	GG
	AG	GG	AG	AG	GG	AA	AG	AG
	AG	GG	AA	AG	AA	AG	AG	
89592955								
4985	rs10133203		14	AA	AG	AG	AA	AA

	AA	AA	AA	AG	AA	AG	AG	GG
	GG	AA	AG	AG	AA	AG	GG	AG
	AG	AG	AG	AG	AA	AA	AA	AA
	AA	GG	AG	GG	AA	AG	AA	
52355387								
4986	rs10133256		14	GG	GG	GG	GG	GG
	GG	GG	GG	GG	GG	GG	GG	GG
	GG	GG	GG	GG	GG	GG	GG	GG
	GG	GG	GG	GG	GG	GG	GG	GG
	GG	GG	GG	GG	GG	GG	GG	
21575631								
4987	rs1013326		8	GG	GG	GG	GG	GG
	GG	GG	GG	GG	GG	AG	AG	GG
	GG	GG	AG	GG	AG	GG	AG	GG
	GG	GG	GG	GG	GG	GG	GG	GG
	GG	GG	GG	GG	GG	GG	AG	
122320560								
4988	rs10133270		14	AA	AA	AG	AA	AA
	AA	AA	AA	AA	AA	AA	AA	AA
	AA	AA	AA	AA	AA	AA	AA	AA
	AA	AA	AA	AA	AA	AG	AA	AG
	AA	AA	AA	AA	AA	AA	AA	
91199626								
4989	rs10133275		14	AG	AA	AA	GG	AG
	AG	GG	AG	AG	AG	AG	AG	AA
	AA	GG	AG	AG	GG	AG	AA	GG
	AA	AG	AG	AG	AG	AG	GG	AA
	AG	AG	AG	AG	AA	AG	AG	
34451267								
4990	rs10133276		14	AG	AG	AG	GG	GG
	GG	GG	AG	AG	AG	AG	AG	AG
	GG	AG	GG	AG	GG	GG	GG	GG
	GG	GG	AG	AG	GG	GG	GG	GG
	GG	GG	GG	GG	GG	GG	GG	
23274831								
4991	rs10133301		14	GG	GG	GG	GG	GG
	GG	AG	GG	GG	GG	AG	GG	GG
	GG	AG	GG	GG	GG	GG	GG	GG
	AA	AG	AG	AG	GG	GG	GG	GG
	GG	GG	AG	GG	GG	GG	AG	
105179194								
4992	rs10133310		14	GG	GG	GG	AG	AG
	AG	AG	GG	GG	GG	AG	GG	AG
	GG	AG	GG	GG	GG	AG	AG	GG
	GG	GG	GG	GG	GG	GG	GG	GG
	AG	AG	AA	AG	AG	AG	AG	
83264437								
4993	rs10133354		14	AG	AA	AG	AA	AG
	AG	AA	AA	AA	AA	GG	GG	AG
	AG	AG	AG	AA	AG	AA	AA	AA

	AA	AG	AA	AA	AG	AA	AG	AG
	GG	AA	AG	AA	AA	AA	AA	
69062850								
4994	rs10133359		14	AC	AC	AC	AC	AC
	AC	AC	AC	AC	AC	AC	AC	AC
	AA	AC	AC	AC	AC	AA	CC	AC
	AC	AC	AC	AA	AA	AC	AA	AC
	AA	AA	CC	AC	AC	CC	AC	
26719152								
4995	rs10133403		14	AA	AA	AA	AA	AA
	AA	AA	AA	AA	AA	AA	AA	AA
	AA	AG	AA	AA	AA	AA	AA	AA
	AA	AA	AA	AA	AA	AA	AA	AA
	AA	AA	AA	AA	AA	AA	AA	
60945298								
4996	rs10133451		14	GG	GG	AG	AG	AA
	AA	AG	AA	AG	AA	AA	AA	AA
	AA	AG	AG	AA	AA	AA	AG	AA
	AG	AG	AA	AA	AA	AA	AA	AA
	AA	AA	AA	AA	AA	AA	AA	
57002857								
4997	rs10133496		14	AA	AA	AA	AA	AA
	AA	AA	AA	AA	AA	AA	AA	AA
	AA	AA	AA	AA	AA	AA	AA	AA
	AA	AA	AA	AA	AA	AA	AA	AA
	AA	AA	AA	AA	AA	AA	AA	
104681161								
4998	rs10133550		14	AA	AG	AA	AA	AG
	AG	AA	AG	AA	AG	GG	AG	AG
	AG	AG	AA	AA	AA	AA	AG	AA
	AA	AA	AA	AA	GG	GG	GG	GG
	AG	AA	AA	AA	AG	AA	AG	
33914037								
4999	rs1013359		20	AA	AA	AA	AA	AA
	AA	AA	AA	AA	AA	AG	AA	AG
	AG	AG	AA	AA	AA	AG	AG	AA
	AA	AA	AA	AA	AA	AG	AA	AA
	AA	AG	AA	AA	AA	AA	AA	
3975011								
5000	rs10133593		14	AG	AA	AG	GG	GG
	GG	GG	GG	GG	GG	AA	AG	AA
	AA	AA	GG	AG	AG	GG	AG	GG
	AG	GG	AG	AG	GG	AA	AG	AG
	AG	GG	AA	AG	AA	AG	AG	
89587535								
5001	rs10133631		14	AA	AA	AA	AA	AG
	AG	AG	AG	AA	AG	AG	GG	AG
	GG	AG	AG	AG	AG	AG	GG	GG
	AG	GG	GG	GG	AA	AG	AG	AA
	AG	GG	AA	AG	AG	AA	AG	

96019331							
5002	rs10133677	14	AA	AA	AA	AA	AA
	AA	AA	AG	AG	AG	AA	AA	AA
	AA	AA	AA	AA	AA	AA	AA	AG
	AA	AA	AA	AA	AA	AA	AA	AA
	AG	AG	AG	AG	AA	AA	AG	
47746980							
5003	rs10133689	14	GG	GG	GG	AG	GG
	GG	AG	GG	GG	GG	GG	AG	AG
	GG	GG	GG	GG	GG	AG	AG	GG
	GG	GG	GG	GG	GG	AG	AG	GG
	GG	AG	GG	AG	GG	GG	GG	
75605055							
5004	rs10133718	14	AA	AA	AA	AG	AA
	AA	AG	AA	AA	AA	AA	AA	AA
	AA	AA	AA	AA	AA	AG	AG	AG
	AA	AG	AA	AG	AA	GG	AG	AG
	AA	AA	AA	AA	AA	AA	AA	
79198368							
5005	rs10133743	14	AA	AA	AG	AA	AA
	AA	AA	AA	AA	AA	AA	GG	AG
	GG	GG	AG	AA	AA	AG	AG	AA
	AA	AG	AG	AG	AG	AG	AG	AG
	AA	AG	AA	AA	AA	AG	AA	
96635599							
5006	rs10133849	14	AG	AG	AG	AG	GG
	GG	AG	AG	AA	AG	AA	AG	AG
	AG	AG	AG	AA	AG	AA	AA	AA
	AG	AG	GG	GG	GG	GG	GG	GG
	AA	GG	GG	GG	GG	AG	GG	
51121700							
5007	rs10133946	14	AA	AA	AA	AG	AA
	AG	AG	AA	AA	AA	AA	AA	AA
	AA	AA	AA	AA	AA	AG	AG	AG
	AA	AA	AA	AA	AA	AA	AA	AA
	AG	AA	AA	AA	AA	AA	AA	
22060768							
5008	rs10133953	14	GG	GG	GG	GG	GG
	GG	GG	GG	GG	GG	GG	GG	GG
	GG	GG	GG	GG	GG	GG	GG	GG
	GG	GG	GG	GG	AG	AG	AG	AG
	AA	AG	GG	AG	AG	AG	GG	
41517011							
5009	rs1013397	4	GG	AG	GG	GG	GG
	GG	GG	AG	GG	AG	AG	AA	AA
	AA	AG	GG	GG	GG	AG	GG	AG
	GG	GG	GG	GG	GG	GG	GG	GG
	GG	GG	GG	GG	GG	AG	AG	
143375877							
5010	rs10133988	14	AA	AC	AC	CC	AC

	AC	AC	CC	CC	CC	CC	CC	CC
	CC	CC	CC	CC	AC	AC	AA	AA
	CC	CC	CC	CC	CC	CC	CC	CC
	AA	AC	AC	AC	AC	AC	CC	
22214659								
5011	rs10134002		14	AG	AG	GG	AA	AG
	AG	AA	AG	GG	AG	AG	AA	AG
	AG	AG	AG	AG	AG	AG	GG	AG
	AA	AA	AG	AG	AA	AG	AA	AG
	AG	GG	AG	GG	AA	AA	AA	
50459609								
5012	rs10134026		14	AG	AG	GG	AG	AG
	GG	AA	AA	GG	AG	AG	AG	AG
	AG	AG	GG	AG	AG	AG	GG	GG
	AG	AG	AG	AG	GG	GG	GG	GG
	GG	AG	AA	AA	AG	GG	AA	
104785827								
5013	rs10134041		14	GG	AG	GG	GG	GG
	GG	GG	AG	AG	AG	AA	AG	AG
	AG	AG	AG	AG	AG	GG	AG	AG
	AA	AG	GG	GG	GG	AG	AG	GG
	AG	GG	GG	GG	AG	GG	AG	
47437326								
5014	rs10134051		14	CC	AC	CC	AC	AC
	AC	AC	AC	AA	AC	CC	AC	AC
	AC	AC	AA	AC	AA	AC	AA	AC
	AC	CC	AC	AC	AC	AC	AC	AA
	AA	AC	AA	AA	AC	AC	CC	
54683987								
5015	rs10134114		14	GG	GG	AG	GG	GG
	GG	GG	AG	AG	AG	GG	GG	GG
	GG	AG	GG	GG	GG	GG	GG	GG
	GG	GG	GG	GG	GG	GG	GG	GG
	AG	GG	GG	GG	GG	GG	AG	
49546880								
5016	rs1013416		11	AC	CC	AC	AA	AC
	AC	AA	AC	AA	AA	AA	AA	AA
	AA	AA	AA	AA	AA	AA	AA	AC
	AC	AC	AC	AC	AC	CC	CC	AC
	AA	AC	AA	AC	AA	AA	AA	
115220254								
5017	rs10134248		14	AA	AG	AG	AG	AA
	AG	AA	GG	AG	GG	AA	AA	AA
	AA	AA	AA	AA	AG	GG	AG	GG
	AG	GG	AG	GG	AG	AG	AG	AA
	AG	AA	AA	AA	AG	AG	AG	
76948862								
5018	rs10134257		14	CC	AC	CC	AC	CC
	AC	AC	AC	AC	AC	AC	CC	AC
	CC	AC	AC	CC	CC	AA	AC	AC

	AC	AC	CC	CC	CC	CC	CC	CC
	AC	CC	CC	CC	AC	AC	CC	
23445536								
5019	rs10134271		14	AA	AA	AA	AA	AA
	AA	AA	AA	AA	AA	AG	AG	AA
	AA	AA	AA	AA	AA	AA	AG	AG
	GG	AG	AA	AA	AG	AA	AG	AG
	AG	AA	AG	AA	AA	AG	AA	
56869007								
5020	rs10134299		14	AA	AA	AA	AA	AA
	AA	AA	AC	AA	AC	AA	AA	AA
	AA	AA	AC	AC	AC	AA	AA	AA
	AA	AA	AA	AA	AA	AA	AA	AA
	AA	AC	AC	AA	AA	AA	AA	
93037133								
5021	rs10134310		14	GG	GG	GG	GG	GG
	GG	GG	GG	AG	AG	GG	GG	GG
	GG	GG	GG	GG	GG	AG	GG	AG
	GG	GG	GG	GG	AG	GG	GG	AG
	GG	AG	GG	GG	GG	GG	GG	
104340346								
5022	rs10134338		14	GG	GG	GG	GG	GG
	GG	GG	GG	GG	AG	GG	GG	GG
	GG	GG	GG	GG	GG	GG	GG	GG
	GG	GG	GG	GG	AG	GG	GG	AG
	AG	GG	AA	AG	GG	GG	GG	
23269234								
5023	rs10134344		14	AA	AG	AA	AG	AA
	AG	AA	AA	AA	AA	AA	AA	AA
	AA	AA	AG	AG	AG	AA	AA	AA
	AA	AA	AA	AA	AA	AA	AA	AA
	AA	AA	AA	AA	AA	AG	AA	
66552393								
5024	rs1013437		2	GG	AG	GG	GG	GG
	GG	GG	AG	AG	AA	GG	AG	AG
	AG	AG	GG	GG	GG	GG	AG	GG
	AG	AG	AG	GG	GG	GG	GG	GG
	GG	AG	AA	AG	GG	GG	GG	
230344697								
5025	rs1013439		17	GG	GG	GG	GG	GG
	GG	GG	GG	GG	GG	GG	GG	GG
	GG	GG	GG	GG	GG	GG	GG	GG
	GG	GG	AG	AG	GG	GG	GG	GG
	GG	GG	GG	GG	GG	GG	GG	
10128048								
5026	rs10134399		14	AA	AG	AG	AG	AG
	GG	AA	AG	GG	GG	AG	AG	AG
	AG	AG	GG	GG	GG	AG	AG	AA
	AG	AG	AG	AG	GG	GG	GG	GG
	AA	AG	GG	AG	GG	GG	GG	

104083457							
5027	rs1013441	19	GG	GG	GG	GG	GG
GG	GG	GG	GG	GG	GG	GG	GG
GG	GG	GG	AG	AG	GG	AG	GG
AG	AG	GG	AA	GG	GG	GG	GG
AA	GG	GG	GG	AG	GG	AA	
32686646							
5028	rs10134414	14	GG	GG	AG	AG	GG
GG	AG	AG	AG	AG	GG	GG	GG
GG	GG	AG	GG	GG	GG	AA	GG
AG	GG	GG	GG	AG	AG	AG	GG
GG	AG	GG	AG	GG	GG	GG	
52644007							
5029	rs10134442	14	AA	AG	AA	AA	AA
AA	AA	AG	AA	AG	AA	AG	AG
AG	AG	AA	AA	AA	AA	AA	AG
AA	AG	AA	AG	AA	AA	AA	AA
GG	AA	AA	AA	AA	AA	AA	
75841937							
5030	rs10134448	14	GG	GG	GG	GG	GG
GG	GG	AG	AG	AG	AG	AG	GG
GG	GG	AG	AG	AG	GG	GG	GG
AG	AG	GG	GG	GG	GG	GG	GG
GG	GG	GG	GG	GG	GG	GG	
50644736							
5031	rs10134488	14	AA	AA	AC	AA	AA
AA	AA	AA	AA	AA	AC	CC	CC
AC	AA	AC	AA	CC	AA	AA	AA
AA	AA	AA	AA	AA	AA	AA	AA
AC	AC	AA	AA	AA	AA	AC	
80252676							
5032	rs10134524	14	CC	CC	CC	AC	CC
AC	CC	CC	CC	CC	CC	CC	CC
AC	AC	CC	CC	CC	CC	CC	CC
CC	AC	CC	AC	CC	CC	CC	CC
CC	CC	CC	CC	CC	CC	AC	
65121634							
5033	rs10134551	14	GG	AG	AG	AA	AG
AA	AG	AA	AA	AA	AG	AA	AG
AG	GG	GG	GG	GG	AA	AA	AA
AG	AG	AA	AA	AA	GG	AG	AG
AG	AG	AA	AA	AG	AA	GG	
94474350							
5034	rs10134586	14	AA	AA	AG	AG	AG
GG	AG	AA	AA	AA	AG	AG	AG
AG	AG	AA	AA	AG	AA	AA	AG
AG	AA	AA	AA	GG	AG	GG	AG
GG	AG	AG	AG	GG	GG	GG	
59534837							
5035	rs1013459	18	AA	AA	AA	AA	AA

AG AA AA AA AA AG AA AA
AA AA AG AA AG GG AG AG
AG AA AG AG AA AA AA AA
AA GG AG GG AA AA AA
11700534
5036 rs10134609 14 AA AA AA AA AA
AA AA AA AA AA AA AA AA
AA AA AA AA AA AA AA AA
AA AA AA AA AA AA AA AA
AA AA AA AA AA AA AA
65517753
5037 rs10134624 14 AG AA AA GG AG
AG GG AG AA AG AA AA AA
AA AA AG AA AA GG AA AG
AG AG AG AG GG AA AG AG
AG AA AG AG AA AG AA
38437611
5038 rs10134629 14 AA AG AG AA AA
AA AA AG AG AG AA GG AG
GG AG GG AG AG GG AG GG
AG AG AG AG AA AG AA AG
AG GG AG GG AG GG AG
25008758
5039 rs1013466
20 -- -- -- -- -- -- -- --
-- -- -- -- -- -- -- --
-- -- -- -- -- -- -- --
-- -- -- -- -- -- -- --
-- -- -- -- 50555324
5040 rs10134703 14 GG GG GG GG GG
GG GG GG GG GG GG AG AG
AG GG AG GG GG AG GG AG
GG GG GG GG AG GG AG GG
AG GG AG GG AG GG AG
34601345
5041 rs10134724 14 GG GG GG GG GG
GG GG GG GG GG GG GG GG
GG GG GG GG GG AG GG GG
AG GG AG AG GG GG GG GG
GG GG GG GG AG GG AG
90891917
5042 rs10134755 14 GG GG GG GG GG
GG GG GG GG GG GG GG GG
GG AG GG GG GG GG GG GG
GG GG GG GG GG AG AG GG
GG GG GG GG GG GG GG
94326457
5043 rs10134764 14 AA AA AA AA AA
AA AA AG AA AG AG AA AG
AA AA AG AG AG AA AA AA

AA AA AA AA AA AA AA AA
AG AA AA AA AG AG AA
97732592
5044 rs1013477 2 GG GG GG AA AG
AG AG AG AG AA AA AG AG
AG AG AG AG GG GG GG GG
AG GG AG AG AG AG GG AA
AG AG AG AA AG AA GG
113777408
5045 rs10134780 14 AA AG AA AG AG
AG AG AA AA AA AA AA AA
AA AA AA AA AA AA AG AA
AA AG AA AA AG AA AG AG
AA AA AA AA AG GG AA
67151389
5046 rs10134782 14 AA AG GG GG AG
GG AG GG GG GG AG GG AG
AG AG AG AG AG GG GG GG
AG GG AG GG AA GG AG AG
AG AG AG GG AA AA AG
81129305
5047 rs10134797 14 AC CC CC CC AC
AC CC CC CC CC CC CC CC
CC CC CC CC CC CC CC CC
CC CC CC CC CC CC CC CC
AC CC AC CC CC CC CC
51894002
5048 rs10134821 14 AA AA AA AA --
AG AA AA -- AA AA AA AA
AA -- -- -- -- -- AG AA
AA -- AG -- -- AA -- AA
AA -- AA -- -- -- AA
31568292
5049 rs10134946 14 GG GG GG GG GG
AG GG GG GG GG GG AG GG
GG GG AG GG GG GG GG AG
GG GG GG GG AG AA AA AG
AG AG GG GG GG AG GG
105654606
5050 rs10134952 14 GG GG GG GG GG
GG GG GG GG GG GG GG GG
GG GG GG GG GG GG GG GG
GG GG GG GG GG GG GG GG
GG GG GG GG GG GG GG
92217064
5051 rs1013496 5 AA AA AA AG AG
AG AA GG GG GG AA AA AG
AG AG AG GG GG AG AA AG
AA AA AA AA AG AA AG AA
AA AG AG AA AG AG AG

123541241
5052 rs10134972 14 AA AA AA AA AA
AA AA AA AA AA AA AA AA
AA AA AA AA AA AA AA AC
AA AA AA AA AA AC AC AA
AC AA AA AA AA AA AA
43869269
5053 rs1013500 6 GG AG AG GG GG
GG GG AG AG GG GG GG GG
GG AG GG AG GG AG AG AG
AG AG AG GG AG GG AG GG
GG GG GG GG AG GG AG
47664649
5054 rs1013506 17 CC AC AA AC AC
AC AA AA AA AA AC AC AA
AA AA AA AA AA CC CC AC
AA AA AC AC AA AA AA AA
AA AC AA AA AC CC AC
68483505
5055 rs10135064 14 AA AA AA AA CC
AC AC AA AA AA AA AA AA
AC AC AC AC AA AA AA AA
AA AA AA AA CC AA AC AC
AA CC AC CC AC AC AA
57668859
5056 rs10135073 14 AA AA AA AA AA
AA AA AA AA AA AA AA AA
AA AA AA AA AA AA AA AA
AA AA AA AA AA AA AA AA
AA AA AA AA AA AA AA
93518545
5057 rs10135093 14 -- -- -- -- --
-- -- -- GG GG -- GG GG
GG -- -- -- -- -- -- --
-- -- -- -- -- -- -- --
-- -- -- -- -- -- --
58874863
5058 rs10135117 14 CC AC CC AC AC
AC AC CC CC CC CC CC CC
CC CC CC CC CC CC AC CC
CC CC CC CC AC CC AC AC
CC CC CC CC AC AC CC
67399391
5059 rs10135123 14 GG GG GG GG GG
GG GG AG AG AG AG GG AG
GG AG GG GG GG AG GG AG
GG GG GG GG GG GG GG GG
GG GG GG GG GG GG AG
97192013
5060 rs10135156 14 GG GG GG GG GG

	GG	GG	GG	GG	GG	GG	GG	GG
	GG	GG	GG	GG	GG	GG	GG	GG
	GG	GG	GG	GG	GG	GG	GG	GG
	GG	GG	GG	GG	GG	GG	AG	
55570529								
5061	rs10135170		14	GG	GG	GG	GG	AG
	AG	GG	GG	AG	GG	GG	AG	GG
	GG	GG	GG	GG	GG	GG	AG	GG
	GG	GG	GG	GG	GG	GG	GG	GG
	GG	GG	GG	GG	AG	AG	GG	
28767363								
5062	rs10135189		14	AG	AG	AA	GG	AG
	AG	GG	GG	GG	GG	GG	GG	GG
	GG	AG	GG	GG	GG	GG	AG	AG
	AG	AG	AG	AG	AA	AG	AG	AA
	AA	AG	AG	GG	AG	AA	AG	
49351716								
5063	rs10135196		14	AA	AG	AG	GG	AG
	GG	AG	GG	AG	GG	AG	AA	AG
	AG	AG	AG	AG	GG	GG	AG	GG
	AG	AG	AG	AG	AA	GG	AG	AG
	GG	AG	AG	GG	GG	GG	GG	
37203697								
5064	rs10135262		14	AC	AC	AC	AC	CC
	AC	CC	AC	AC	AC	AA	AA	AA
	AA	AC	AA	AA	AA	AA	AC	AA
	AA	AC	AC	AC	AA	CC	AC	AC
	AC	AA	AA	AA	AC	AC	AC	
92666291								
5065	rs10135283		14	AA	AG	GG	AG	AG
	GG	AA	AA	AA	AA	AG	AG	AG
	GG	AG	GG	AG	AG	AG	AA	GG
	AG	GG	GG	GG	GG	AA	AG	AG
	AG	AA	AG	AG	AG	AA	GG	
97252629								
5066	rs10135285		14	AA	AG	AA	AA	AG
	AA	AG	AG	AG	AG	AG	AA	AG
	AG	AG	GG	GG	AG	AG	AA	AG
	AA	AA	AA	AA	AG	AA	AG	AA
	AG	AA	AG	AG	AA	AG	AA	
37687840								
5067	rs10135310		14	AC	CC	CC	AC	AC
	CC	AC	CC	CC	CC	CC	CC	CC
	CC	CC	CC	AC	CC	CC	AC	CC
	CC	CC	CC	CC	CC	CC	CC	CC
	CC	CC	AC	AC	CC	CC	CC	
64574140								
5068	rs1013532		4	GG	GG	GG	GG	GG
	GG	GG	GG	GG	GG	AG	AG	AG
	AG	AG	GG	GG	GG	AG	AG	AG

	GG	GG	GG	GG	AG	GG	GG	GG
	GG	GG	GG	GG	GG	GG	GG	
27157313								
5069	rs10135326		14	GG	GG	GG	GG	GG
	GG	GG	GG	GG	GG	GG	GG	GG
	GG	GG	GG	GG	GG	GG	GG	GG
	GG	GG	GG	GG	GG	GG	GG	GG
	GG	GG	GG	GG	GG	GG	GG	
105955668								
5070	rs1013534		16	AA	AA	AA	AG	AG
	AG	AA	AA	AG	AG	AA	AA	AA
	AG	AA	AA	AA	AA	AA	AA	AA
	AA	AG	AA	AA	AA	AA	AA	AA
	AA	AA	AA	AA	AA	AA	AA	
25426202								
5071	rs10135367		14	AG	GG	GG	GG	GG
	AG	GG	GG	GG	AG	AG	AG	AG
	AG	AG	AG	AA	AA	GG	GG	GG
	GG	GG	AG	AG	GG	GG	GG	GG
	GG	GG	AA	AG	AG	AA	GG	
21257750								
5072	rs10135425		14	AA	AA	AA	AA	AA
	AA	AA	AA	AA	AA	AA	AA	AA
	AA	AA	AA	AA	AA	AA	AA	AA
	AA	AA	AA	AA	AA	AA	AA	AA
	AA	AA	AA	AA	AA	AA	AA	
91432118								
5073	rs1013545		13	AA	AG	AA	GG	AA
	AG	AG	AG	AG	AG	GG	AG	AG
	AG	AG	GG	AG	AG	AG	AG	AA
	AA	AA	AA	AA	AA	AA	AA	AA
	GG	AA	AA	AA	AA	AA	AA	
73971803								
5074	rs1013547		X	AA	AA	AA	AA	AA
	AA	AA	AA	AA	AA	AG	AG	AG
	AG	AG	AA	AA	AA	AA	AG	AA
	AA	AA	AA	AA	AA	AA	AA	AA
	AG	AA	AA	AA	AA	AA	AA	
47849441								
5075	rs10135486		14	GG	GG	GG	GG	AG
	AG	GG	AG	GG	AG	GG	GG	GG
	GG	GG	GG	GG	GG	GG	GG	AG
	AG	AG	AG	AG	AG	AA	AG	AA
	AG	AG	AA	AG	AG	AG	GG	
96523110								
5076	rs10135529		14	AG	AG	AG	AG	AA
	AG	AA	AA	AA	AA	AA	AG	AG
	AG	AA	AG	AG	AG	AA	AG	AG
	AA	AA	AA	AA	AG	AA	AA	AG
	AG	AG	AA	AG	AG	AA	AG	

85662421
5077 rs10135631 14 AG GG GG GG GG
GG GG GG GG GG AG GG GG
GG GG GG GG GG GG GG GG
GG GG GG GG GG GG GG GG
GG GG GG GG GG GG GG
92131816
5078 rs1013568 4 GG GG GG GG GG
GG GG GG GG AG AG GG AG
AG GG GG GG GG GG GG GG
GG GG GG GG GG GG GG GG
AA GG GG GG GG GG GG
21494694
5079 rs10135704 14 AA AA AA AG AA
AG AA AA AA AA AA AG AG
AG AA GG AG AG AA AA AG
AA AA AA AA AA AA AA AA
AG AA AA AA AA AA AA
97331618
5080 rs10135736 14 AG AG AG AG AA
AA AG AA AG AA AG AA AG
AA AA AG AA AA AA AA AA
AG AA AG AA AG GG GG GG
AG AA AA AA AG AA AG
76875446
5081 rs10135750 14 AG AG GG AA GG
AG AG AA AA AA AG AG GG
AA GG AA AA AA AG AA AA
AG AA AA GG AG AG AA GG
AA AA AA AA AG AG GG
25672680
5082 rs10135768 14 AA AA AC -- --
CC -- -- -- -- -- CC --
-- AC -- -- -- -- AC --
AC -- AC AC -- AC -- AC
AC AA -- AC -- -- --
51671017
5083 rs10135786 14 AA AA AA AA AA
AA AA AA AA AA AA AG AG
AG AG AA AA AA AA AA AA
AA AA AA AA AG AG GG AG
AA AG AA AA AA AA AA
80879836
5084 rs10135821 14 GG GG GG GG GG
GG GG AG GG AG AG GG GG
GG AG GG GG GG GG GG GG
GG AG GG GG GG GG GG GG
GG GG GG GG GG GG GG
97199889
5085 rs1013586 4 AC AA AA CC CC

	CC	CC	CC	CC	CC	AA	AC	AA
	AA	AC	AC	AC	AA	CC	CC	CC
	CC	CC	CC	CC	CC	CC	CC	CC
	AC	AC	CC	CC	CC	CC	CC	
41760431								
5086	rs10135894		14	GG	GG	GG	GG	GG
	GG	GG	GG	GG	GG	GG	GG	GG
	GG	GG	GG	GG	GG	GG	GG	GG
	GG	GG	GG	GG	GG	GG	GG	GG
	GG	GG	GG	GG	GG	GG	GG	
37050739								
5087	rs10136083		14	GG	GG	GG	GG	GG
	GG	GG	GG	GG	GG	GG	GG	GG
	GG	GG	GG	GG	GG	GG	GG	GG
	GG	GG	GG	GG	GG	GG	GG	GG
	GG	GG	GG	GG	GG	GG	GG	
44820190								
5088	rs10136099		14	GG	GG	GG	AG	AG
	GG	AG	GG	AG	AG	AG	AG	AG
	AG	AG	GG	GG	GG	GG	AG	GG
	GG	GG	GG	GG	GG	GG	GG	GG
	GG	GG	GG	GG	GG	GG	GG	
64609604								
5089	rs10136113		14	AA	AA	AG	AG	AG
	GG	AA	GG	AA	GG	AG	GG	AG
	AG	GG	AA	AA	AG	AG	AA	AA
	GG	GG	GG	--	AA	AG	AA	AG
	AA	AA	AG	AA	AG	AG	GG	
39041292								
5090	rs10136120		14	CC	CC	CC	AC	CC
	AC	CC	CC	CC	CC	CC	AC	AC
	AC	CC	CC	CC	CC	CC	CC	CC
	CC	CC	CC	CC	CC	CC	CC	CC
	CC	CC	CC	CC	CC	CC	CC	
56072086								
5091	rs10136137		14	AG	AG	AA	GG	GG
	GG	GG	AG	GG	AG	AG	AG	AG
	AG	GG	GG	AG	GG	AA	AG	AG
	GG	GG	GG	GG	GG	AA	AG	AG
	AG	AA	AG	AA	AG	AG	AG	
30396038								
5092	rs10136159		14	AA	AG	GG	AG	AG
	AA	AG	AG	AG	AG	AA	AG	AA
	AA	AA	AG	AA	AA	AG	AG	AA
	AG	AA	AG	AG	AA	AA	AA	AA
	AA	AG	AG	GG	AA	AA	AA	
99544443								
5093	rs1013620		10	GG	GG	GG	AG	GG
	GG	AG	GG	GG	GG	GG	GG	GG
	GG	GG	GG	GG	GG	GG	GG	AG

	AG	GG	GG	GG	GG	GG	GG	GG
	AG	GG	AG	GG	AG	AG	GG	
120187722								
5094	rs10136204		14	AA	AA	AC	CC	CC
	CC	CC	AC	CC	AC	AC	AC	AA
	AA	AC	CC	CC	CC	CC	CC	CC
	CC	CC	CC	CC	CC	CC	CC	CC
	CC	CC	AC	AC	AC	AC	AC	
46610740								
5095	rs10136208		14	GG	AG	AG	AA	AA
	AA	AA	AA	AA	AA	AG	AG	AG
	AA	AA	AA	AA	AA	AA	AA	AA
	AG	AA	AA	AA	AA	AA	AA	AA
	AA	AG	AA	AA	AA	AA	AA	
61958044								
5096	rs10136259		14	AG	GG	GG	AA	AA
	AA	AA	AA	AG	AA	AA	AA	AA
	AA	AG	AA	AA	AA	AG	AG	AG
	AG	AG	AG	AG	AG	AA	AG	AA
	AA	AA	AG	AG	AG	AA	GG	
33596724								
5097	rs10136276		14	GG	GG	GG	GG	GG
	GG	GG	GG	GG	GG	GG	GG	GG
	GG	GG	GG	GG	GG	GG	GG	GG
	GG	GG	GG	GG	GG	GG	GG	GG
	GG	GG	GG	GG	GG	GG	GG	
95518762								
5098	rs10136316		14	GG	AG	GG	GG	GG
	GG	GG	GG	AG	GG	GG	GG	AG
	AG	AG	GG	GG	GG	GG	GG	AG
	GG	GG	GG	GG	GG	AG	AG	GG
	GG	AG	GG	GG	AA	AG	AA	
69063521								
5099	rs10136352		14	AA	AG	AG	AA	AG
	AG	AA	AG	AA	AG	AA	AG	AG
	AG	AA	AG	AA	AA	AG	AA	GG
	AG	AA	AA	AA	AG	AG	AG	AG
	AG	AG	AA	AA	AG	AG	AG	
35334555								
5100	rs10136379		14	GG	GG	GG	GG	GG
	GG	GG	GG	GG	GG	GG	GG	GG
	GG	GG	GG	GG	GG	GG	GG	GG
	GG	GG	GG	GG	GG	GG	GG	GG
	GG	GG	GG	GG	GG	GG	GG	
50199680								
5101	rs10136408		14	GG	AG	AG	AG	AA
	AA	AG	GG	AG	GG	GG	AG	AG
	AG	AA	GG	GG	GG	AA	GG	AG
	AG	AG	GG	GG	AG	AA	AG	AG
	GG	GG	AG	GG	AG	AA	GG	

56079726								
5102	rs10136448		14	AA	AA	AA	AA	AA
	AA	AA	AA	AA	AA	AA	AA	AA
	AA	AA	AA	AA	AA	AA	AA	AA
	AA	AA	AA	AA	AA	AA	AA	AA
	AA	AA	AA	AA	AA	AA	AA	
93235885								
5103	rs10136485		14	AC	CC	AC	AC	AC
	CC	AC	AA	AC	AA	AA	CC	AC
	AC	AA	AC	AC	AC	AC	CC	CC
	AC	AA	AC	AC	CC	AA	AC	AC
	AC	CC	AC	AC	CC	AC	CC	
102846856								
5104	rs10136536		14	AG	AG	AA	AG	AA
	AG	AA	AG	AG	AG	AA	AA	AA
	AA	AG	AA	AA	AA	AG	AG	AA
	AA	AG	AG	AG	GG	AA	AG	AG
	AA	AA	AA	AA	AG	AA	AG	
39587348								
5105	rs10136541		14	GG	GG	GG	GG	GG
	GG	GG	AG	AG	AG	GG	GG	GG
	GG	AG	GG	GG	GG	GG	GG	GG
	GG	GG	GG	GG	AG	GG	AG	GG
	GG	GG	GG	GG	AG	AA	GG	
36443674								
5106	rs1013655		1	GG	GG	GG	GG	GG
	GG	GG	GG	GG	GG	GG	GG	GG
	GG	GG	GG	GG	GG	GG	GG	GG
	GG	GG	GG	GG	GG	GG	GG	GG
	GG	GG	GG	GG	GG	GG	GG	
38955374								
5107	rs10136653		14	AA	AA	AA	GG	GG
	GG	GG	AA	AA	AA	AA	AG	AG
	GG	AG	AA	AG	AG	GG	AG	AG
	AG	AG	AG	AG	AA	AA	AA	AA
	GG	AA	GG	AG	AG	AA	AG	
89134686								
5108	rs10136662		14	AA	AA	AA	AG	AG
	AA	AG	AA	AG	AG	AG	AG	AG
	AG	AG	AA	AA	AA	AA	AG	AG
	AA	AA	AA	AA	AA	AA	AA	AA
	AA	AA	AA	AA	AA	AA	AA	
64597186								
5109	rs10136671		14	CC	CC	CC	AC	AA
	AC	AA	AC	CC	AC	CC	AC	CC
	CC	AC	AC	CC	CC	AC	AA	AA
	AA	CC	AC	AC	AA	AA	AA	AA
	AA	AC	CC	AC	CC	CC	CC	
101474982								
5110	rs10136683		14	CC	CC	AC	AC	AC

	CC	AA	AA	AC	AA	AC	CC	AC
	AC	AC	AA	AC	AC	CC	CC	AC
	AC	AC	AC	AC	AA	AA	AA	AA
	AC	AC	AC	AA	CC	CC	AC	
52000575								
5111	rs10136708		14	AA	AA	AA	AA	AC
	AC	AC	AC	AC	AC	AC	AC	AC
	AC	AC	AA	AA	AA	CC	AC	CC
	AA	AA	AA	AA	AA	AC	AA	AC
	AA	AA	AA	AA	AA	AC	AA	
59927376								
5112	rs10136774		14	GG	AG	AG	AG	AG
	AG	GG	AA	AA	AG	AG	AA	AG
	AA	AG	AG	AA	AG	GG	GG	GG
	AA	AG	AG	AA	GG	GG	GG	GG
	AG	AA	AG	AA	AG	AG	AA	
22048083								
5113	rs10136789		14	AA	AA	AA	AA	AG
	AG	AA	AA	AA	AA	AA	AA	AA
	AA	AA	AA	AG	AA	AA	AA	AA
	AA	AG	AA	AG	AA	AA	AA	AA
	AA	AA	AA	AA	AA	AA	AA	
63356253								
5114	rs10136804		14	CC	CC	CC	CC	CC
	CC	CC	CC	CC	CC	CC	CC	CC
	CC	CC	CC	CC	CC	CC	CC	CC
	CC	CC	CC	CC	CC	CC	CC	CC
	CC	CC	CC	CC	CC	CC	CC	
96976335								
5115	rs10136885		14	CC	CC	CC	AC	AC
	AC	AC	AC	AC	AC	CC	AC	AC
	AC	AC	CC	AC	AC	AC	AC	AA
	AC	AA	AC	AC	CC	CC	CC	CC
	CC	CC	CC	CC	CC	CC	AC	
54208049								
5116	rs10136905		14	AA	AC	CC	AC	AC
	AC	AC	AA	AA	AA	CC	AC	CC
	AC	AA	AC	CC	CC	AC	AC	AC
	AC	CC	CC	CC	AC	CC	AC	CC
	AC	CC	AC	AC	AC	CC	AC	
94064755								
5117	rs1013693		2	GG	GG	GG	GG	GG
	GG	AG	GG	GG	AG	AA	AG	AG
	GG	GG	AG	AG	AG	AG	AG	AG
	GG	AG	AG	GG	GG	AG	GG	GG
	GG	GG	AG	GG	GG	GG	GG	
154861176								
5118	rs10136937		14	AG	AA	AA	AA	AA
	AA	AA	AA	AA	AA	AA	AA	AA
	AA	AG	AG	AG	AG	AG	AG	AG

AA AG AG AG AA AA AA AA
AG AG AA AA GG AG AG
105078153
5119 rs10136966 14 GG GG GG GG GG
GG GG GG GG GG GG GG GG
GG GG GG GG GG GG GG GG
GG GG GG GG GG GG GG GG
GG GG GG GG GG GG GG
55310058
5120 rs1013702 8 GG GG GG GG GG
GG GG GG GG GG GG GG GG
GG GG GG GG GG AG GG AG
GG GG GG GG GG GG GG GG
GG GG AA AG GG GG GG
4361441
5121 rs10137035 14 GG GG AG GG GG
GG GG GG AA AG AG AA AA
AA AG AG AG AG GG GG AG
AA GG AG AG GG AA AG AG
GG AG GG AG AG AG GG
103244840
5122 rs10137053 14 GG GG GG GG GG
GG GG GG GG GG GG GG GG
GG GG GG GG GG GG GG GG
GG AG AG AG GG GG GG GG
GG GG GG GG GG AG GG
104758887
5123 rs1013708 1 CC CC CC CC CC
CC CC CC CC CC CC CC CC
CC CC CC CC CC CC CC CC
CC CC CC CC CC CC CC CC
CC CC CC CC CC CC CC
61565605
5124 rs1013719 7 AA AA AA AA GG
AG AG AA AA AA AA AA AA
AG AG AG AG AG AA AA AA
AA AG AA AG AA AA AA AA
AA AA AA AA GG AG AG
10554750
5125 rs10137191 14 GG AG AG AG AA
AA AG GG AG GG AA AG AA
AG AG AG AA AA AG AG AG
AA AA AA AA GG AA AG AG
AG AA AG AG AG AG AG
94184499
5126 rs10137204 14 AA AA AA AA AA
AA AA AA AA AA AA AA AA
AA AA AA AA AA AA AA AA
AA AA AA AA AA AA AA AA
AA AA AA AA AA AA AA

61624070							
5127	rs10137242	14	AG	AG	GG	GG	GG
	GG	GG	GG	GG	GG	GG	GG	GG
	GG	GG	GG	AG	GG	GG	GG	GG
	AG	AG	GG	GG	GG	GG	GG	GG
	GG	GG	AG	GG	GG	GG	AG	
52590539								
5128	rs10137249	14	GG	GG	GG	GG	GG	
	GG	GG	GG	GG	GG	GG	GG	GG
	GG	GG	GG	GG	GG	GG	GG	GG
	GG	GG	GG	GG	GG	GG	GG	GG
	GG	GG	GG	GG	GG	GG	GG	
54037705								
5129	rs10137287	14	GG	AG	GG	AA	AA	
	AA	AA	AG	AG	AG	AG	AG	AG
	AG	AG	GG	GG	AG	AG	GG	AA
	GG	AG	AG	AG	AA	AA	AA	AA
	GG	AG	AA	AG	AG	AA	AG	
28294108								
5130	rs10137294	14	AA	AA	AG	AA	GG	
	AG	AG	AA	AA	AA	AA	AA	AA
	AA	AG	AG	AG	AG	AA	AG	AA
	AA	AA	AA	AA	AA	AG	AA	AG
	AA	AA	AG	AG	AG	AG	AA	
51261672								
5131	rs10137318	14	GG	GG	GG	AA	AG	
	AA	AG	AG	AG	AG	AG	AG	GG
	GG	AG	AA	AA	AA	AA	AA	AA
	AA	AA	AA	AA	AG	AG	AA	GG
	AA	AG	AG	GG	AG	GG	AA	
44903433								
5132	rs10137321	14	AA	AA	AA	AA	AA	
	AA	AA	AA	AA	AA	AG	AA	AG
	AA	AG	AA	AA	AA	AA	AA	AA
	AA	AA	AA	AA	AA	AA	AA	AA
	AA	AA	GG	AG	AA	AG	AA	
83348149								
5133	rs10137327	14	AA	AG	AG	GG	GG	
	GG	GG	AG	AG	AG	GG	GG	GG
	GG	GG	GG	GG	AG	GG	GG	GG
	GG	GG	GG	GG	GG	AG	AG	GG
	GG	GG	GG	GG	AG	AG	AA	
58753901								
5134	rs10137391	14	GG	GG	GG	GG	GG	
	GG	GG	AG	GG	AG	GG	GG	GG
	GG	GG	GG	GG	GG	GG	GG	GG
	GG	GG	GG	GG	GG	GG	GG	GG
	GG	GG	GG	GG	GG	GG	GG	
98675398								
5135	rs10137435	14	AA	AA	AA	AA	AA	

AA AA AA AA AA AG AG AA
AA AA AG AG AG AA AA AA
AA AG AA AA AG AG GG AG
AG AA AG AA AA AA AA
62548587
5136 rs1013745 7 GG GG GG GG GG
GG GG AA AA AA GG GG GG
GG GG GG GG GG GG AG GG
AG AG AG AG GG GG GG GG
AG AG AG AG GG GG GG
109590629
5137 rs10137489 14 CC CC CC AC AC
AA CC CC CC CC CC CC CC
AC AA AC CC CC AC AA AC
CC CC CC CC CC AA AC AC
CC CC AC CC CC AC CC
99099947
5138 rs10137523 14 CC CC CC AC CC
AC CC AC CC AC AC AC CC
CC AA AC AC -- CC CC CC
AA AC CC CC AC CC AC CC
AA AC AC AA AC AC AA
43308739
5139 rs10137551 14 AA AA AA AA AA
AA AA AA AA AA AA AA AA
AA AA AA AA AA AA AA AA
AA AA AA AA AA AA AA AA
AA AA AA AA AA AA AA
103572314
5140 rs10137566 14 AA AA AA AA AA
AA AA AA AA AA AA AA AA
AA AA AA AA AA AA AA AA
AA AA AA AA AA AA AA AA
AA AA AA AA AA AA AA
104311251
5141 rs10137571 14 AA AG AA AA AG
AG AA AG AG AG AG AG AG
AG AG AG AG AG GG GG AG
AG AA AG AG AA AA AA AA
AA AA AG AG AA AA AA
59505553
5142 rs10137630 14 AA AA AA AA AA
AA AA AA AA AA AA AA AA
AA AA AA AA AA AA AA AA
AA AA AA AA AA AA AA AA
AA AA AA AA AA AA AA
104320544
5143 rs10137683 14 AG AG GG AG GG
GG AG AG GG AG AG GG AG
AG AG AG AG AA GG AG GG

GG AG AG AG GG AG GG AG
AA GG GG GG AA AA AG
34308644
5144 rs10137726 14 GG GG GG GG GG
GG GG GG GG GG GG GG GG
GG GG AG AG AG AA GG AG
GG GG GG GG AG GG AG GG
GG GG GG GG GG GG GG
90375260
5145 rs10137770 14 GG GG AG AA GG
AG AG AA AG AA AG AG AG
AG AG AG GG GG AG AG AG
AA AA AA AA AA AG AG AA
AG AA AG AG AA AG AG
92922442
5146 rs10137809 14 AG GG GG GG GG
GG GG GG GG GG AG GG GG
GG GG GG GG GG GG GG GG
GG GG GG GG GG GG GG GG
GG GG GG GG GG GG GG
92139032
5147 rs10137814 14 AA AA AG GG GG
AG AG AA AA AA AA AA AA
AA AG AA AA AA AA AA AG
AA AG AG AG AA AA AA AA
AA GG AA AG AG AG AA
105088609
5148 rs10137845 14 AA AA AA AG AG
AA AG AG AA AG AA AA AA
AA AA AA AA AA AA AA AA
AG AA AA AA AA AA AA AA
AG AG AA AG AA AA AA
96485365
5149 rs10137860 14 AA AA AA AA AA
AA AA AA AA AA AA AA AA
AA AA AA AA AA AA AA AA
AA AA AA AA AA AA AA AA
AA AA AA AA AA AA AA
60293407
5150 rs10137889 14 GG GG GG GG GG
GG GG GG AG AG GG GG GG
GG GG GG GG GG GG GG GG
GG GG GG GG GG GG GG GG
GG GG GG GG GG GG GG
104415782
5151 rs1013789 7 GG GG GG GG GG
GG GG GG GG GG GG GG GG
GG GG GG GG GG GG GG GG
GG GG GG GG GG GG GG GG
GG GG GG GG GG GG GG

41864241
5152 rs10137908 14 AA AA AA AA AA
AA AA AA AA AA AA AA AA
AA AA AA AG AA AA AA AA
AA AA AA AA AA AA AA AA
AA AA AA AA AA AA AA
64092355
5153 rs1013792 9 GG GG GG AG AG
GG AA AG AG AG GG GG GG
GG GG AG AG AG AA AA AG
AA AA AA AA AG AG AG GG
GG AG AG AG AA AG AA
78306320
5154 rs10137952 14 AA AC AA AA AC
AC AA AA AA AA AA AA AA
AA AC CC AC AC AA AA AC
AA AA AA AA AC AA AC AC
AC AC AA AA AA AA AC
50478230
5155 rs10137961 14 GG GG GG GG GG
GG GG GG GG GG GG GG GG
GG GG GG GG GG GG GG GG
GG GG GG GG GG GG GG GG
GG GG GG GG GG GG GG
65900195
5156 rs10137995 14 AA AA AA AA AG
AG AA AA AA AA AG AG AA
AA AA AA AA AA AA AG AA
AA AA AA AA AG AA AG AG
AA AA AG AA AA AA AA
56173687
5157 rs10137999 14 AG AG AG AA AA
AA AA AG AG AG GG AG GG
AA GG AG AA AG AA AA AA
AA AA AA AA AA AA AA AA
AG AA AA AA AG AG AA
23047799
5158 rs1013803 7 AG AG GG GG AG
AG GG AG AG AG AA AA AG
GG AG AG AG AG AG GG AA
AG GG GG AG AG AA AA AG
GG AA GG AG GG GG AG
92623141
5159 rs10138053 14 GG GG GG AA GG
AG AG GG GG GG AG AG AA
AA GG AA AA AA AG GG AG
AG AA AG AG AG AG AA AG
GG GG AA AG AA AG AA
51615152
5160 rs10138056 14 AA AA AA AG AA

AG AA AA AA AA AA AA AA
AA AA AA AA AA AA AA AA
AA AA AA AA AA AA AA AA
AA AA AA AA AA AA AA
103424854
5161 rs10138062 14 GG AG AG AA AA
AA AA GG AG AG GG GG GG
GG AG AG GG GG AG AA AG
GG AG GG GG GG GG GG GG
AA AG AA AG GG AG GG
103218818
5162 rs1013812 7 GG GG GG GG AG
GG AG AG AA AA GG GG GG
GG GG GG GG GG GG GG GG
AG AG AA AG GG GG GG GG
AG GG AG GG AG AG GG
8722688
5163 rs10138125 14 AA AA AA AA AA
AA AA AG AA AG AG AA AG
AA AA AG AG AG AA AA AA
AA AA AA AA AA AA AA AA
AG AA AA AA AA AA AA
97763841
5164 rs10138134 14 CC CC CC CC CC
CC CC CC CC CC AC AC CC
CC CC CC CC CC AC CC AA
AC CC CC CC AC AC AC AC
CC AC AC AC CC CC CC
37176928
5165 rs10138135 14 AG GG GG AG GG
GG AG AG AA AG GG GG GG
GG GG GG GG GG AG AG AG
AG GG AG AG AA GG AG AG
AA AG AG AG AA AA AG
78557203
5166 rs1013815 22 CC CC CC CC CC
CC CC CC CC CC CC CC CC
CC CC CC CC CC CC CC CC
CC CC CC CC CC CC CC CC
CC CC CC CC AC CC AA
26330370
5167 rs10138170 14 GG GG GG AG AG
AG GG AG AG GG AG AA AG
GG AG AG AA AG AG GG GG
AG AG GG GG AG AG AA GG
GG GG AG AG GG AG GG
22279576
5168 rs10138228 14 AG GG GG AG GG
AG GG GG AG GG GG AG AG
AG AG GG GG GG AG GG AG

	AG	AG	AG	AG	GG	GG	GG	GG
	AG	AG	AG	AA	AG	GG	AG	
49778543								
5169	rs10138279		14	AA	AA	AA	AA	AA
	AA	AA	AA	AA	AA	AA	AA	AA
	AA	AA	AA	AA	AA	AA	AA	AA
	AA	AA	AA	AA	AA	AA	AA	AA
	AA	AA	AA	AA	AA	AA	AA	
43064028								
5170	rs10138386		14	AG	GG	GG	AG	GG
	AG	GG	GG	AG	GG	AG	AG	GG
	AA	AA	AA	AG	AA	AG	GG	AA
	AG	AA	AG	AA	AG	GG	AG	AG
	AA	GG	AA	AG	AG	GG	AA	
74900886								
5171	rs10138430		14	AG	AG	AG	AG	AG
	AG	AG	GG	AA	GG	AA	AG	AG
	AG	AG	AA	AA	AA	AG	GG	GG
	GG	GG	GG	GG	GG	GG	GG	GG
	AG	GG	AG	AG	AG	AA	AG	
44000927								
5172	rs10138457		14	GG	GG	AG	AG	AG
	GG	AG	GG	GG	GG	AG	GG	AG
	AG	AG	GG	GG	GG	GG	GG	GG
	GG	GG	GG	GG	GG	GG	GG	GG
	GG	GG	GG	GG	GG	GG	GG	
102458052								
5173	rs10138460		14	AA	AA	AG	AA	AG
	AG	AA	GG	AG	GG	AA	AA	AA
	AA	AA	AG	AG	AG	GG	AA	AG
	AA	AA	AA	AA	AA	AA	AA	AA
	AA	AA	AA	AA	AG	AG	AA	
58465141								
5174	rs1013848		12	GG	AG	GG	AG	AA
	AA	AG	GG	AG	AG	GG	AG	AG
	AA	AA	AA	AA	AA	AG	AG	AA
	AA	AG	AG	AG	AA	AA	AA	AA
	GG	AG	AG	AG	AA	AG	AG	
92516807								
5175	rs10138505		14	--	AG	--	AG	AG
	AG	AG	--	--	GG	GG	GG	--
	GG	--	--	--	--	--	AG	--
	--	AG	GG	GG	AG	--	AG	AG
	--	--	--	--	AG	AA	GG	
66946020								
5176	rs10138506		14	AA	AA	AG	AA	AA
	AA	AA	AA	AA	AA	GG	GG	AG
	AA	AA	AG	AG	AG	AA	AA	AA
	AA	AA	AA	AA	AA	AA	AA	AA
	AA	AA	AA	AA	AG	AG	AA	

65388243
5177 rs10138598 14 GG GG GG GG GG
GG GG GG GG GG GG GG GG
GG GG GG GG GG GG GG GG
GG GG GG GG GG GG GG GG
GG GG GG GG GG GG GG
97311321
5178 rs10138602 14 GG GG GG AG AG
GG AA AG AA AG GG GG GG
AG AA AG GG GG GG GG GG
GG AA AG AG AG GG AG GG
AG AA GG AG GG GG GG
94232953
5179 rs10138605 14 AA AA AA AA AA
AA AA AA AA AA AA AA AA
AA AA AA AA AA AA AA AA
AA AA AA AA AA AA AA AA
AA AA AA AA AA AA AA
101135965
5180 rs10138608 14 GG AG AG AG AA
AA AG AG AA AG AG AG AA
AA AA AG GG AG AA AG AA
AA AG AG AG AA AA AA AA
GG AA AA AA AG AA GG
55930132
5181 rs1013861 18 AG AG AG AA AA
AA AA AA AA AA AG GG GG
AG GG AA AA AA AG AG GG
AA AA AA AA AA AA AA AA
AA AA GG AG AA AA AG
2205343
5182 rs10138629 14 AA AA AA AA AA
AA AA AA AA AA AA AA AA
AA AA AA AA AA AA AA AA
AA AA AA AA AA AA AA AA
AA AA AA AA AA AA AA
21171545
5183 rs10138647 14 AA AC AC AA CC
AC AC CC AC CC AC AA AA
AA AA CC CC CC AC AC AA
AA AA AA AA AA AC AA AC
AC AC AA AC AC AC AA
94612496
5184 rs10138671 14 GG GG GG GG GG
GG GG GG AG AG AA AA AG
GG GG GG AG GG GG AA GG
AG AG AG AG AA GG AG AG
GG AA AG AG AG AG GG
65860092
5185 rs1013870 7 GG GG GG GG GG

GG GG GG GG GG GG GG GG
GG GG GG GG GG GG GG GG
GG GG GG GG GG GG GG GG
GG GG AG GG GG GG GG
70037996
5186 rs10138713 14 GG GG GG GG GG
GG GG GG GG GG GG GG GG
GG GG GG GG GG GG GG GG
GG GG GG GG GG GG GG GG
GG GG GG GG GG GG GG
60232394
5187 rs10138749 14 GG GG GG GG GG
AG GG GG GG GG GG AG AG
AG GG GG GG GG GG GG GG
AG GG GG GG GG GG GG GG
GG GG GG GG GG GG GG
105538190
5188 rs10138768 14 AA AA AA AG GG
AG AG AA AG AG AG AG AG
AG AG AA AG AG AG AA AA
AA AG AG AG GG AA AG AG
AA AG AA AA AA AA AA
104190527
5189 rs10138807 14 AG AA AA AG AG
AG GG AG AG AG GG AG GG
AG GG AA AA AG AA AG AG
AG AG AG AG GG GG GG GG
AG GG GG GG AG GG AA
21492310
5190 rs10138842 14 AA AA AA AA AA
AA AA AA AA AA AA AA AA
AA AA AA AA AA AA AA AA
AA AA AA AA AA AA AA AA
AA AA AA AA AA AA AA
103917029
5191 rs10138851 14 AA AA AA AA AA
AA AA AA AA AA AA AA AA
AA AA AA AA AA AA AA AA
AA AA AA AA AA AA AA AA
AA AA AA AA AA AA AA
67481686
5192 rs1013888
15 -- -- -- -- -- -- -- --
-- -- -- -- -- -- -- --
-- -- -- -- -- -- -- --
-- -- -- -- -- -- -- --
-- -- -- -- 69689440
5193 rs1013890 14 AA AA AA AA AA
AA AA AA AA AA AA AA AA
AA AA AG AG AG AA AG AA

	AA	AA	AA	AA	AA	AA	AA	AA
	AA	AA	AA	AA	AA	AA	AA	
81386625								
5194	rs10138910		14	GG	GG	GG	GG	GG
	GG	GG	GG	GG	GG	AG	AG	GG
	GG	GG	AG	AG	AG	AG	GG	GG
	GG	GG	GG	GG	AG	GG	AG	GG
	GG	GG	GG	GG	GG	GG	GG	
40309496								
5195	rs1013894		6	GG	AG	AG	GG	AG
	AG	GG	GG	GG	GG	GG	GG	GG
	GG	GG	AG	GG	GG	AG	AG	AG
	AG	AG	AG	AG	AG	AG	AG	AG
	GG	AG	GG	GG	AG	GG	AG	
57183734								
5196	rs10138940		14	CC	CC	AC	AA	AC
	AC	AA	AA	AA	AA	AC	AA	AC
	AA	AA	AA	AA	AA	AA	AA	AA
	AA	AA	AA	AA	AC	AA	AC	AA
	AA	AA	AA	AA	AA	AA	AA	
98816835								
5197	rs1013895		2	AG	AG	AG	AA	AG
	AG	AA	AA	AG	AG	AA	AG	AA
	AA	AA	AG	GG	GG	AG	GG	GG
	AA	AA	AG	AG	AG	AG	GG	AG
	AA	AA	AA	AA	AA	AA	AG	
191668839								
5198	rs10138951		14	AA	AA	AA	AA	AA
	AA	AA	AG	AA	AG	AA	AA	AA
	AA	AA	AA	AA	AA	AA	AA	AA
	AG	AA	AG	AG	AA	AA	AA	AA
	AA	AA	AA	AA	AA	AA	AG	
81026314								
5199	rs10138952		14	AA	AG	AA	AG	AG
	AG	AG	AA	AA	AA	AA	AA	AA
	AA	AA	AA	AA	AA	AA	AG	AA
	AA	AA	AA	AA	AG	AA	AG	AG
	AA	AA	AA	AA	AG	AG	AA	
67536019								
5200	rs10138962		14	AC	AC	AC	AA	AA
	AA	AA	AC	AA	AC	AC	AC	AC
	AC	AA	AA	AA	AA	AC	AC	AC
	AA	AC	AC	AC	CC	AA	AC	AC
	CC	AA	AA	AA	CC	AC	CC	
104995893								
5201	rs10138982		14	--	--	--	--	--
	--	--	--	--	--	--	--	--
	--	--	AG	--	--	--	AA	--
	--	AG	AG	--	AA	AA	AA	AA
	AA	AA	AA	AA	AG	AG	AG	

41916228
5202 rs10139082 14 CC AC CC CC CC
CC CC CC CC CC CC CC CC
CC CC CC CC CC CC CC CC
CC CC CC CC AC AC CC AC
CC AC AC CC AC CC AC
75223338
5203 rs1013910 1 AA AA AA AG AA
AA AG AA AA AA AA AG AG
AA AG AA AA AG AA AA AA
AG AA AG AA AG AA AA AA
AA AA AA AA AA AA AA
117411383
5204 rs10139118 14 AC AC AC AA CC
AC AC AC CC AC CC CC CC
CC CC AC AA AC CC AC CC
AC AA AA CC AC CC AC CC
CC CC CC CC AC CC AA
26563796
5205 rs10139155 14 AA AA AA AA AA
AA AA AA AA AA AA AA AA
AA AA AA AA AA AA AA AA
AA AA AA AA AA AA AA AA
AA AA AA AA AA AA AA
56122314
5206 rs10139177 14 GG GG GG GG GG
GG GG GG GG GG GG GG GG
GG GG GG GG GG GG GG GG
GG GG GG GG GG GG GG GG
GG GG GG GG GG GG GG
92403677
5207 rs10139183 14 AA AG GG AA AG
AG AG GG GG GG GG AG AG
AG GG GG GG GG AG AG AG
GG AG AG AG GG GG GG GG
GG AG GG GG GG GG GG
98633873
5208 rs10139204 14 AG GG AG GG GG
GG GG GG GG GG GG GG GG
GG GG GG GG GG GG GG GG
GG GG GG AG AG GG AG GG
GG GG GG GG GG GG GG
24382905
5209 rs10139212 14 AG AA AA GG AG
AG AG GG AG GG GG GG GG
GG GG AG AG AG AG AA AG
AG GG GG GG GG GG GG GG
AG AG GG GG AG AA AG
98992552
5210 rs10139231 14 AG AG AG AG GG

GG AG AA AA AA AG AG AA
AA GG AG AG AG GG AA AG
AA AA AA AA AA AA AA AA
AG AA GG AG AG GG AG
52575630
5211 rs10139251 14 CC CC CC CC CC
CC CC CC CC CC CC CC AC
AC AC CC CC CC AC CC CC
CC CC CC CC CC CC CC CC
CC CC CC CC CC CC CC
60959958
5212 rs10139268 14 GG AG GG GG GG
GG GG GG GG GG GG GG AG
GG GG GG GG AG GG GG GG
GG GG GG GG GG AG AG GG
GG AG AG GG GG AG GG
63311176
5213 rs10139287 14 CC CC CC AC AC
AC AC CC AC CC AA AC AA
AC AC CC CC CC AC CC CC
CC CC CC CC CC CC CC CC
CC AC AC AA CC AC CC
91896534
5214 rs10139298 14 AG AA AA AG AG
AA GG GG GG GG AA AA AA
AA AG AA AA AA AG AG AG
AA AG AA AA AG AA AG AA
AG AG AG AA AA AG AA
88008255
5215 rs10139324 14 AG AG AG AA AA
AA AA GG AG GG AG AG AA
AA AG AA AA AA AG AG AG
AG AA AA AA GG GG GG GG
AG AG AA AA AG GG AG
56240420
5216 rs10139335 14 AG GG GG GG GG
GG GG GG GG GG GG GG GG
GG GG GG GG GG AG GG GG
GG AG AG AG GG GG GG GG
GG GG GG GG GG GG GG
91567882
5217 rs10139337 14 AG AG GG GG AG
GG AG AG AG AG AG GG AG
AG AA AA AG AA GG AG GG
GG GG GG GG AA AG AA AG
GG GG GG GG AG AG GG
55742113
5218 rs10139351 14 GG GG GG GG GG
GG GG GG GG GG GG GG GG
GG GG GG GG GG GG GG GG

	GG	GG	GG	GG	GG	GG	GG	GG
	GG	GG	GG	GG	GG	GG	GG	
35517992								
5219	rs1013937		1	CC	CC	CC	CC	CC
	CC	CC	CC	CC	CC	CC	CC	CC
	CC	CC	AC	AC	AC	AA	AA	AA
	CC	AC	AC	AC	AA	AC	AA	AC
	CC	AC	CC	AC	CC	CC	CC	
199282009								
5220	rs10139376		14	AG	GG	GG	GG	GG
	GG	GG	AG	GG	AG	AG	AG	AG
	AA	GG	AA	AG	AG	GG	GG	GG
	GG	GG	GG	GG	GG	GG	GG	GG
	GG	AA	GG	AG	GG	AG	GG	
29032833								
5221	rs10139382		14	GG	GG	GG	GG	AG
	GG	AG	GG	GG	GG	GG	GG	GG
	AG	GG	GG	GG	GG	GG	GG	GG
	GG	GG	GG	GG	GG	GG	GG	GG
	GG	GG	GG	GG	GG	GG	GG	
24300497								
5222	rs10139403		14	AG	GG	GG	AG	GG
	AG	GG	AG	AG	AG	AG	GG	AG
	AG	AG	AG	GG	GG	GG	AG	AG
	AG	GG	AG	AG	AG	AG	AG	AG
	GG	GG	AA	AG	GG	AG	GG	
101194561								
5223	rs10139427		14	AA	AA	AA	AA	AA
	AA	AA	AA	AA	AA	AA	AA	AA
	AA	AA	AA	AA	AA	AA	AA	AA
	AA	AA	AA	AA	AA	AA	AA	AA
	AA	AA	AA	AA	AA	AA	AA	
52682284								
5224	rs10139429		14	AC	AA	AC	CC	AC
	AC	CC	AC	AC	AC	CC	AC	AC
	AC	CC	AA	AC	AC	AC	AC	CC
	CC	AC	CC	CC	CC	CC	CC	CC
	CC	AC	CC	AC	CC	AC	AC	
94572431								
5225	rs10139453		14	AA	AA	AA	AA	AA
	AA	AA	AA	AA	AA	AA	AA	AA
	AA	AA	AA	AA	AA	AA	AA	AA
	AA	AA	AA	AA	AA	AA	AA	AA
	AA	AA	AA	AA	AA	AA	AA	
106752607								
5226	rs1013948		17	AA	AA	AA	AA	AA
	AA	AA	AA	AA	AA	AG	AA	AA
	AA	AA	AA	AA	AA	AA	AG	AA
	AA	AA	AA	AA	AG	AG	AG	AG
	AA	AA	AA	AA	AA	AG	AA	

29530709								
5227	rs1013950		16	AA	AA	AA	AG	AG
	AG	AG	AA	AG	AA	AA	AA	AA
	AA	AG	AG	AA	AA	AG	AG	GG
	AG	AG	AG	AG	AA	AA	AA	AA
	AG	AA	GG	AG	AG	AG	AA	
20737729								
5228	rs10139504		14	AA	AG	GG	AG	AG
	GG	AA	AA	AA	AA	AG	GG	AG
	GG	GG	AG	AG	AG	GG	GG	GG
	AG	AG	AA	AA	GG	GG	GG	GG
	AG	GG	AG	AG	AG	GG	AA	
99135675								
5229	rs10139525		14	AG	AG	AG	AA	AA
	--	AA	GG	AG	GG	AG	GG	GG
	GG	GG	GG	GG	GG	GG	GG	GG
	GG	GG	GG	GG	GG	AG	AG	GG
	AG	GG	GG	GG	AG	GG	AA	
105285159								
5230	rs10139559		14	GG	GG	GG	AG	AG
	AG	AG	GG	GG	GG	GG	AG	GG
	AG	AA	AA	AG	AG	AG	AG	AA
	AG	AG	AG	GG	GG	AA	AG	AG
	AG	AG	AA	AA	AG	AA	AG	
84463196								
5231	rs1013962		12	GG	GG	--	AG	AG
	--	AA	GG	AG	AG	GG	AA	AG
	AG	AG	GG	AG	AG	GG	--	AG
	GG	GG	GG	GG	GG	GG	GG	GG
	AG	GG	AG	AG	GG	AG	GG	
4494954								
5232	rs10139645		14	GG	GG	GG	GG	GG
	GG	GG	GG	GG	GG	GG	GG	GG
	GG	GG	GG	GG	GG	GG	GG	GG
	GG	GG	GG	GG	GG	GG	GG	GG
	GG	GG	GG	GG	GG	GG	GG	
98959525								
5233	rs10139651		14	AA	AA	AA	AA	AA
	AA	AA	AA	AA	AA	AA	AA	AA
	AA	AG	AA	AA	AA	AA	AA	AA
	AA	AA	AA	AA	AA	AA	AA	AA
	AA	AA	AA	AA	AA	AA	AA	
61519304								
5234	rs10139652		14	AA	AA	AA	AC	AA
	AC	AA	AA	AC	AA	AA	AA	AA
	AA	AC	AA	AA	AA	AC	AA	AA
	AA	AA	AA	AA	AC	AC	AC	CC
	AA	AA	AA	AA	AA	AC	AA	
55976428								
5235	rs10139668		14	AC	CC	CC	AA	AC

AC AC CC CC CC AC AC AA
AC AC AC AC AC AA AC AC
AC AA AA AA CC CC CC CC
CC AC CC CC CC AC CC
100022233
5236 rs10139750 14 GG AG GG GG AA
AG AG AG AA AG AA AA AA
AA AG AG AA AA AG AG AG
AG GG GG GG AA GG AG AG
AA AG AA AA AG AA GG
53494122
5237 rs10139797 14 GG GG GG GG GG
GG GG GG AG GG GG GG GG
GG GG AG AG AG GG GG GG
AG GG GG GG GG GG GG GG
GG GG AG AG GG GG GG
101708609
5238 rs10139817
14 -- -- -- -- -- -- -- --
-- -- -- -- -- -- -- --
-- -- -- -- -- -- -- --
-- -- -- -- -- -- -- --
-- -- -- -- 89348612
5239 rs10139819 14 AG AG AG GG AG
AG GG AG GG AG GG AG GG
AG AG AG AA AA AA AA AA
AA GG AG AG AA GG AG AG
AA AG GG AG GG GG GG
98767593
5240 rs10139839 14 GG GG GG GG GG
GG GG GG GG GG GG GG GG
GG GG GG GG GG GG GG GG
GG GG GG GG GG GG GG GG
GG GG GG GG GG GG GG
105643976
5241 rs10139863 14 AA AA AA AG AA
AA AG AG AG AG AA AA AA
AA AA AA AA AA AA AA AA
AA AA AA AA AA AA AA AA
AA AA AA AA AG AA AG
28026321
5242 rs10139869 14 CC CC CC CC AC
CC AC CC CC CC CC CC CC
CC CC CC CC CC CC CC CC
CC CC CC AC CC CC CC CC
CC CC CC CC CC CC AC
24346429
5243 rs10139874 14 GG GG GG GG GG
GG GG GG GG GG GG GG GG
GG GG GG GG GG GG GG GG

	GG	AG	GG	GG	GG	GG	GG	GG
	GG	GG	GG	GG	GG	GG	GG	
85607274								
5244	rs1013994		17	AA	AA	AG	AA	AA
	AA	AA	AG	AA	AA	AG	AG	AA
	AA	AA	AA	AA	AA	AG	AA	AG
	AA	AA	AA	AA	AA	AA	AA	AA
	AA	AA	AA	AA	AA	AA	AA	
68063716								
5245	rs10139978		14	CC	CC	CC	AC	CC
	AC	CC	CC	CC	CC	CC	CC	CC
	CC	CC	CC	CC	CC	AC	CC	CC
	CC	CC	CC	CC	CC	CC	CC	CC
	CC	CC	CC	CC	CC	CC	CC	
70880373								
5246	rs10140007		14	AA	AA	AA	AA	AG
	AG	AG	GG	GG	GG	GG	GG	AG
	AG	AG	AA	AA	AA	AG	AG	GG
	AA	AA	AA	AA	AA	AG	AA	AG
	AA	AG	AA	AA	AA	AG	AA	
59951278								
5247	rs10140052		14	AA	AA	AA	AA	AA
	AA	AA	AG	AG	AG	AG	AA	AA
	AA	AA	AA	AA	AA	GG	AG	GG
	AA	AG	AA	AA	AA	AG	AG	AA
	AA	AA	AA	AA	AA	AA	AA	
98169326								
5248	rs10140061		14	GG	GG	GG	GG	AG
	AG	GG	GG	GG	GG	GG	GG	GG
	GG	GG	GG	GG	GG	GG	GG	GG
	GG	GG	GG	GG	GG	GG	GG	GG
	AG	GG	GG	GG	GG	GG	GG	
63424135								
5249	rs10140081		14	GG	AG	AG	GG	AA
	AG	AG	AG	AG	AG	AG	GG	AG
	AG	AG	AG	GG	AG	AG	AG	AA
	AA	AG	GG	GG	AG	AG	AG	GG
	GG	AA	AA	AA	AG	AG	GG	
55925629								
5250	rs10140168		14	GG	GG	GG	GG	GG
	GG	GG	GG	GG	GG	GG	GG	GG
	GG	GG	GG	GG	GG	GG	GG	GG
	GG	GG	GG	GG	GG	GG	GG	GG
	GG	GG	GG	GG	GG	GG	GG	
36374152								
5251	rs10140174		14	GG	GG	GG	GG	GG
	GG	GG	GG	GG	GG	GG	GG	GG
	GG	GG	GG	GG	GG	GG	GG	GG
	GG	GG	GG	GG	GG	GG	GG	GG
	GG	GG	GG	GG	GG	GG	GG	

103312440
5252 rs10140203 14 GG GG GG GG GG
GG GG GG GG GG GG GG GG
GG GG GG GG GG GG GG GG
GG GG GG GG GG GG GG GG
GG GG GG GG GG GG GG
67837898
5253 rs10140228 14 AA AA AA AA AA
AA AA AA AG AA AA AG AG
GG AG AA AA AA AA AA AA
AA AA AA AA AG AA AG AA
AA AG AA AG GG GG AG
95141693
5254 rs10140256 14 AA AA AG AA AA
AA AA AA AA AA AA AA AA
AG AG AA AG AG AA AA AA
AA AG AA AG AA AA AA AA
AA AA AA AA AA AA AA
63833977
5255 rs1014027 17 AA AA AG AA AA
AA AG GG GG GG AA AG AA
AA AA AG AG AG AG AA AA
AA AG AG AG AA GG AG AG
AA GG AG AG AA AA AA
15223722
5256 rs10140275 14 AA AA AA AA AA
AA AA AA AA AA AA AA AA
AA AA AA AA AA AA AA AA
AA AA AA AA AA AA AA AA
AA AA AA AA AG AG AG
95472704
5257 rs10140287 14 AA AG AG AG AG
GG AA AG AG AA AG AG AG
GG AG AG GG AG AA AG AA
GG AG AG GG AA AA AA AA
AG AG AG AG AG AG GG
22069779
5258 rs10140310 14 GG GG GG GG AG
AG GG AA AA AA AA AA AA
AA AG AG AG AG GG AG AG
GG AG GG GG AG AG AA GG
AA AG AA AA AG AA AG
85226972
5259 rs10140316 14 CC AC AC AC CC
AC CC CC CC CC CC CC CC
CC AC CC CC CC CC CC CC
AC CC AC AC CC CC CC CC
CC CC CC CC CC CC CC
88533552
5260 rs1014032 8 GG GG GG GG GG

GG GG AG AG AG GG AG AG
AG AG GG AG GG AG GG GG
GG GG GG GG GG AA AG AG
GG GG AG GG AA AG AG
83761875
5261 rs10140357 14 GG GG GG GG GG
GG GG GG GG GG GG GG GG
GG GG GG GG GG GG GG GG
GG GG GG GG GG GG GG GG
GG GG GG GG GG GG GG
106381916
5262 rs10140359 14 AA AA AG AG AG
AG AG GG AG GG AG GG AG
AG AG AA AG AA AG GG AG
GG GG GG AG AA AG AA AG
AG AG GG AG AG GG AG
25540265
5263 rs1014036 4 GG GG GG GG AG
AG AG AG AG AG GG GG AG
GG GG AG GG AG GG GG GG
GG AG AG AG GG AG GG GG
GG AG AG AG AG GG AG
42943177
5264 rs10140406 14 CC AC AC AC AC
AA CC AC CC AC CC CC CC
CC CC AC AC AC CC CC CC
AC CC CC CC CC AC AC CC
CC AC CC AC CC CC CC
100639981
5265 rs10140469 14 AA AG AG AG AA
AA AG AA AA AA AG AA AG
AA AA AA AA AA AA AA AA
AG AG AG AG AA GG AG AG
AA AG AA AA AA AG AA
92534114
5266 rs10140470 14 GG GG AG GG AG
AG AG GG AG GG AG GG GG
AG AG AG GG GG GG GG GG
GG GG GG GG GG GG GG GG
GG AG AG AG GG GG GG
98551202
5267 rs1014056 4 GG AG AG GG GG
GG AG GG GG GG GG GG GG
GG GG GG GG GG GG GG GG
AG GG GG GG AG AG AG GG
AG AG GG GG GG AG GG
54325990
5268 rs10140578 14 AA AG AG AG GG
AG GG AG AG AG AG AG GG
GG AG AA AA AA AA AA AA

	AG	GG	GG	GG	GG	GG	GG	GG
	AG	GG	AG	AG	GG	GG	GG	
62257603								
5269	rs10140750		14	AA	AG	AA	AG	AA
	AA	AG	AA	AA	AA	GG	GG	GG
	AA	AA	AG	AG	AG	AA	AA	AG
	AG	AA	AG	AA	AA	AA	AA	AA
	AG	AA	AA	AA	AG	AA	GG	
66269872								
5270	rs1014076		5	AG	AG	GG	AA	AA
	AG	AA	GG	AG	GG	AG	AA	AG
	AA	GG	AG	GG	GG	GG	GG	AG
	AG	AG	AG	AG	AG	AA	AA	AG
	AG	GG	AG	GG	AG	AG	GG	
50495353								
5271	rs10140787		14	AA	AA	AA	AA	AA
	AA	AA	AA	AA	AA	AA	AA	AA
	AA	AA	AA	AA	AA	AA	AA	AA
	AA	AA	AA	AA	AA	AA	AA	AA
	AA	AA	AA	AA	AA	AA	AA	
54746463								
5272	rs10140804		14	AA	AG	AA	AG	AG
	AG	AG	AA	GG	AA	AA	AG	AG
	AG	AG	AA	AG	AG	GG	AG	AG
	AA	AA	AA	AA	AA	AA	AA	AA
	AA	AA	AG	AA	GG	AG	GG	
38557201								
5273	rs10140830		14	AC	AC	CC	CC	AC
	AC	CC	CC	AC	CC	AC	CC	CC
	CC	AC	CC	CC	CC	CC	CC	CC
	CC	AC	CC	CC	AC	CC	AC	CC
	CC	AC	CC	CC	AC	CC	AC	
98220751								
5274	rs10140859		14	AA	AA	AG	GG	GG
	GG	GG	GG	GG	GG	AG	AA	AG
	AG	GG	GG	GG	GG	AG	GG	AG
	GG	AA	AG	AG	AA	AG	AA	AG
	AG	AG	AG	AG	AG	GG	AG	
101710360								
5275	rs1014086		8	AA	AA	AA	AA	AA
	AA	AA	AA	AA	AA	AA	AA	AA
	AA	AA	AA	AA	AA	AA	AA	AA
	AA	AA	AA	AA	AA	AA	AA	AA
	AG	AA	AA	AA	AA	AA	AA	
57644072								
5276	rs10140869		14	AG	AG	GG	GG	AG
	GG	AG	AG	AG	AG	AG	GG	AG
	AG	AA	AA	AG	AA	GG	AG	GG
	GG	GG	GG	GG	AA	AG	AA	AG
	GG	GG	AG	AG	AG	AG	GG	

55795283
5277 rs10140873 14 AA AA AA AA AA
AA AA AA AA AA AA AA AA
AA AA AA AA AA AA AA AA
AA AA AA AA AA AA AA AA
AA AA AA AA AA AA AA
62852656
5278 rs10140885 14 AA AA AA AA AA
AA AA AA AA AA AA AA AA
AA AA AA AA AA AA AA AA
AA AA AA AA AA AA AA AA
AA AA AA AA AA AA AA
67038200
5279 rs10140897 14 GG GG GG GG GG
GG GG GG GG GG GG GG GG
GG GG GG GG GG GG GG GG
GG GG GG GG GG GG GG GG
GG GG GG GG GG GG GG
72333196
5280 rs10140947 14 AA AA AA AA AA
AA AA AA AA AA AA AA AA
AA AA AA AA AA AA AA AA
AA AA AA AA AG AA AG AA
AA AA AA AA AA AG AA
100040382
5281 rs1014095 7 GG GG AG GG GG
AG GG AA GG AG GG GG GG
GG GG AG AG AG GG GG GG
AG AG AG AG GG GG GG GG
AG GG GG GG GG AG AG
148463860
5282 rs10140999 14 GG GG GG GG GG
GG GG GG AG GG GG GG GG
GG AG GG GG GG AG GG GG
GG GG GG GG GG AG GG AG
GG GG GG GG GG AG GG
55973715
5283 rs10141083 14 GG GG GG GG GG
GG GG GG GG GG GG GG GG
GG GG GG GG GG GG GG GG
GG GG GG GG GG GG GG GG
GG GG GG GG GG GG GG
35689477
5284 rs10141091 14 AG AG AG AA AG
AG AA AA AA AA AA AA AA
AA AA AA AA AG AA AA AA
AA AA AA AA AA AA AA AA
AG AA AG AG AG AA AG
24422366
5285 rs10141099 14 CC CC AC CC AC

AC CC CC CC CC CC CC CC
CC AC AC CC CC CC CC CC
AC AA AC AC CC CC CC CC
AA CC CC CC AC AC CC
49960957
5286 rs10141127 14 AA AC AC AC AC
AC AC AA AA AA AA AA AA
AC CC AC CC CC AC CC AC
CC AA CC CC AC CC AC CC
AA CC CC CC AC AC AC
87773053
5287 rs10141159 14 AG AA AA AA AG
AG AA AA AA AA AA AA AA
AA AA AG AG AA AG AG AA
AG AG AG AG AA AA AA AA
AA AA AG AG AA AA AA
83877181
5288 rs10141237 14 AA AA AA GG AG
AG GG AG AG AG AA AA AA
AA AA AA AA AG AA AG AA
AA AA AA AA AA AA AA AA
AG AG AA AG AA AA AA
35772940
5289 rs1014124 16 CC CC CC CC CC
CC CC CC CC CC CC CC CC
CC CC CC CC CC CC CC CC
CC CC CC CC CC CC CC CC
CC CC CC CC CC CC CC
7781828
5290 rs10141289 14 GG GG GG GG GG
GG GG GG GG GG GG GG GG
GG GG GG GG GG GG GG AG
GG GG AG AG AG AG AA GG
GG AG GG AG AG AG AG
36301996
5291 rs1014136 7 AG AG GG AG AA
AG AA AA AG AA GG AG AG
AG AG AG AG GG AA AA AA
AG AG GG AA AA AA AA AA
AA AA AG AA AA AA AA
38229107
5292 rs10141377 14 CC CC CC CC CC
CC CC CC CC CC CC AC AC
AC CC CC CC CC CC CC AC
CC CC CC CC CC CC CC CC
AC CC CC CC CC CC CC
52600092
5293 rs10141526 14 AC AC AC AC CC
AC CC AC AC AC CC CC CC
CC CC AA AA AA CC CC AC

	CC	CC	CC	CC	CC	CC	CC	CC
	AC	CC	AC	AC	CC	CC	CC	
62303252								
5294	rs1014161		17	GG	GG	GG	GG	GG
	GG	AG	GG	GG	GG	GG	GG	GG
	GG	GG	GG	GG	AG	GG	GG	GG
	GG	GG	GG	GG	GG	GG	GG	GG
	GG	GG	GG	GG	GG	GG	GG	
34065695								
5295	rs10141612		14	GG	GG	GG	GG	GG
	GG	GG	GG	GG	GG	GG	GG	GG
	GG	AG	GG	GG	GG	GG	GG	GG
	GG	GG	GG	GG	GG	AG	GG	AG
	AG	GG	AG	GG	GG	GG	GG	
51500337								
5296	rs10141640		14	AA	AG	AG	GG	AA
	AG	AG	GG	AG	GG	AG	GG	GG
	AG	GG	GG	AG	AG	AA	AG	AA
	AG	AA	AA	AA	AG	GG	GG	AG
	GG	AA	AG	AG	GG	AG	AG	
27186292								
5297	rs10141646		14	CC	AC	AC	CC	AC
	AC	CC	CC	CC	CC	CC	CC	CC
	CC	CC	CC	CC	CC	AC	AA	CC
	CC	AC	CC	CC	CC	AC	AC	CC
	AC	CC	CC	CC	CC	CC	CC	
88799607								
5298	rs10141671		14	AA	AA	AA	AA	AA
	AA	AA	AA	AA	AA	AA	AA	AA
	AA	AA	AA	AA	AA	AA	AA	AA
	AA	AA	AA	AA	AG	AA	AG	AA
	AA	AA	AA	AA	AA	AA	AA	
36205383								
5299	rs10141674		14	AG	AG	AG	AG	AG
	GG	AA	GG	AA	GG	GG	GG	GG
	GG	GG	GG	GG	GG	GG	AG	GG
	GG	GG	GG	GG	AG	AG	AG	AG
	GG	AG	AG	AA	AG	AG	GG	
39138238								
5300	rs10141685		14	GG	GG	GG	AG	GG
	GG	AG	GG	GG	GG	GG	GG	GG
	GG	AG	GG	GG	GG	AG	GG	AA
	GG	GG	GG	GG	GG	AG	GG	AG
	GG	GG	GG	GG	GG	GG	GG	
53873841								
5301	rs10141716		14	CC	CC	CC	CC	CC
	CC	CC	CC	CC	CC	CC	CC	CC
	CC	CC	CC	CC	CC	CC	CC	CC
	CC	CC	CC	CC	CC	CC	CC	CC
	CC	CC	CC	CC	CC	CC	CC	

101089858
5302 rs1014174 2 AG AG AG AG AA
AA AG GG AG GG AG AG AA
AA AA AG AG GG GG AA AG
AA AA AA AG AG AG AA AG
AG AG AA AG AA AA AG
229951964
5303 rs10141836 14 AC AC AA AC AC
AA CC AC AC AC AC AA AC
AA AC CC CC CC CC CC CC
CC CC CC CC CC AC CC AC
AC CC CC CC AA AC AA
20663078
5304 rs1014184 7 AA AA AA AA AA
AA AA AA AG AG AG AA AG
AG AA AA AA AA AA AA AA
AA AG AA AG AA AA AA AA
AA AG AA AA AA AG AA
11501528
5305 rs10141863 14 AG AG AG AA AG
AG AA AA AG AA GG GG GG
GG AG GG AG AG AG AA AG
GG AG AG AG GG AG AG GG
AA AA AG AG AG AG AG
100276707
5306 rs10141879 14 GG GG GG GG AG
GG AG GG GG GG GG GG GG
GG GG GG GG GG GG AG GG
GG GG GG GG GG GG GG GG
GG GG GG GG GG GG GG
67695190
5307 rs10141935 14 AA AG AA AG AA
AA AG GG AA GG AG AG AG
GG AA AG AA AA AG AG AA
AG AG AG AG AG AG AG AG
AG AA AG AG AG AG AA
29006754
5308 rs10141953 14 AG AG AA GG GG
GG GG AA GG AA AG AG AA
AA GG GG GG -- AG AG AG
GG AG AG AG GG AG GG AG
AG AA GG AG GG GG AG
43489721
5309 rs10142079 14 AG AA AA AA AA
AA AA AA AA AA AA AA AA
AA AA AG AG AG AA AA AA
AA AA AA AA AA AA AA AA
AA AA AA AA AA AA AA
48867664
5310 rs10142090 14 GG GG GG GG GG

GG GG GG GG GG GG GG GG
GG GG GG GG GG GG GG GG
GG AG GG GG GG GG GG GG
GG GG GG GG GG AG GG
87926629
5311 rs10142119 14 AA AA AA AA AG
AG AA AG AA AG AA AA AA
AA AA AA AA AA AA AA AG
AG AA AG AG AA GG AG AG
GG AG AA AG AA AG AG
98486545
5312 rs10142143 14 CC AC CC AC AC
AC AC CC CC CC CC CC CC
CC CC CC CC CC CC AC CC
CC AC CC CC AC CC AC AC
CC CC CC CC AC AA CC
66859548
5313 rs10142149 14 AA AA AA AA AA
AA AA AA AA AA AA AA AA
AA AA AA AA AA AA AA AA
AA AA AA AA AA AA AA AA
AA AA AA AA AA AA AA
98905488
5314 rs10142150 14 GG GG GG GG GG
GG GG GG GG GG GG GG GG
GG GG GG GG GG GG GG GG
GG GG GG GG GG GG GG GG
GG GG GG GG GG GG GG
55637951
5315 rs10142203 14 GG GG AG AA AA
AA AA AA AA AA AA AA AA
AA AA AA AA AA AA AG AA
AA AA AA AA AA AG AA AG
AG GG AA AG AG AA AG
51704790
5316 rs10142208 14 GG GG GG GG GG
GG GG GG AG GG GG GG GG
GG GG GG GG GG GG GG GG
AG AG AG AG GG GG GG GG
GG GG AG AG AG GG AG
101890398
5317 rs10142247 14 AA AA AA AG AG
AA GG AA AG AA AA AG AG
AG AG AA AA AG AG AA AA
AA AG AG GG AA AA AA AA
AG AA AA AA AA AA AA
26784913
5318 rs10142253 14 GG GG AG GG GG
GG GG GG AG GG GG AG GG
GG GG GG AG AG AG AG GG

	GG	GG	GG	GG	GG	AG	GG	AG
	GG	GG	GG	GG	AG	AG	AG	
85071223								
5319	rs1014226		2	CC	CC	CC	CC	CC
	CC	CC	AC	CC	CC	AC	AC	AA
	AA	AA	AC	AC	AC	CC	CC	CC
	CC	--	--	CC	CC	AC	AC	CC
	AC	CC	CC	CC	CC	CC	CC	
199412464								
5320	rs10142288		14	GG	GG	GG	GG	GG
	GG	GG	GG	GG	GG	GG	GG	GG
	GG	GG	GG	GG	GG	GG	GG	GG
	GG	GG	GG	GG	GG	GG	GG	GG
	GG	GG	GG	GG	GG	GG	GG	
67005434								
5321	rs10142315		14	AC	AC	CC	CC	AC
	AC	CC	CC	CC	CC	CC	CC	CC
	CC	CC	AC	CC	CC	CC	CC	CC
	AC	AC	AC	CC	CC	AC	AC	CC
	AC	AC	AC	CC	CC	AC	AC	
76074898								
5322	rs10142370		14	GG	AG	AG	GG	GG
	GG	GG	GG	GG	GG	GG	GG	GG
	GG	GG	GG	GG	AG	GG	GG	GG
	GG	AG	GG	AG	GG	GG	GG	GG
	GG	GG	GG	GG	GG	GG	GG	
79211948								
5323	rs10142401		14	GG	GG	GG	GG	GG
	GG	GG	GG	GG	GG	GG	GG	AG
	AG	AG	GG	GG	GG	AG	GG	GG
	GG	GG	GG	GG	GG	GG	GG	GG
	GG	GG	GG	GG	GG	GG	GG	
61039403								
5324	rs10142406		14	AA	AA	AA	AA	AA
	AA	AA	AA	AA	AA	AA	AA	AA
	AA	AA	AA	AA	AA	AA	AA	AA
	AA	AA	AA	AA	AA	AA	AA	AA
	AA	AA	AA	AA	AA	AA	AA	
58114717								
5325	rs10142427		14	AA	AC	CC	AC	AA
	AC	AA	AA	AA	AA	AC	CC	CC
	AC	AA	AC	AC	AC	AA	AA	AA
	AA	AC	AA	AA	CC	CC	CC	CC
	AA	CC	AA	AC	AA	AA	AA	
99673324								
5326	rs10142446		14	AG	AG	AG	AG	AG
	AG	AG	AG	AG	AG	AG	GG	GG
	AG	AA	AG	AG	AG	AA	AG	AG
	AG	AG	AG	AG	AG	GG	AG	GG
	AA	AA	AG	AG	AG	AG	AG	

95219827								
5327	rs10142463		14	AA	AA	AA	AC	AA
	AA	AC	AA	AA	AA	AA	AA	AA
	AA	AA	AC	AA	AA	AA	AA	AA
	AC	AC	AA	AA	AA	AA	AA	AA
	AA	AA	AA	AA	AA	AA	AA	
56980878								
5328	rs10142526							
14	--	--	--	--	--	--	--	--
	--	--	--	--	--	--	--	--
	--	--	--	--	--	--	--	--
	--	--	--	--	--	--	--	--
	--	--	--	--	47968008			
5329	rs10142528		14	AA	AA	AA	GG	AA
	AG	AG	AA	AA	AA	AG	AG	AG
	AA	AA	AA	AG	AG	AG	AA	AG
	AG	AG	AG	AG	AA	AA	AA	AA
	AA	AG	AA	AG	AG	AA	GG	
92330790								
5330	rs10142596		14	GG	GG	GG	GG	GG
	GG	GG	AG	AG	AG	GG	AG	AG
	GG	GG	AA	AG	AG	GG	GG	GG
	GG	GG	AG	AG	GG	GG	GG	GG
	GG	GG	GG	GG	AG	GG	AA	
56859468								
5331	rs10142601		14	AG	AG	AA	AA	AA
	AA	AA	AA	AA	AA	AA	AA	AA
	AA	AA	AG	AG	AG	AA	AG	AA
	AA	AG	GG	AG	AG	AG	AA	GG
	AA	AG	AA	AG	AA	AA	AA	
21143334								
5332	rs10142618		14	AA	AA	AA	AG	AG
	AG	AG	AG	GG	AG	AA	AG	AG
	AA	AA	AA	AA	AA	AG	AG	GG
	AA	GG	AA	AG	AG	AG	AG	AA
	AA	AG	AA	AA	AA	AA	AA	
72622760								
5333	rs1014262		4	AA	AA	AA	AA	AG
	AA	AG	AA	AA	AA	GG	AG	AG
	AG	AG	AA	AA	AA	AA	AA	AA
	GG	GG	GG	AG	AG	AA	AG	AG
	AA	AA	AA	AA	AA	AA	AG	
24762301								
5334	rs10142654		14	GG	GG	GG	AG	GG
	GG	AG	GG	GG	GG	GG	GG	GG
	AG	AA	GG	GG	GG	GG	GG	AG
	GG	GG	GG	GG	GG	AA	AG	AG
	AG	AG	GG	AG	GG	GG	GG	
58290779								
5335	rs10142682		14	AA	AA	AA	AA	AA

	AA	AA	AA	AA	AA	AA	AA	AA
	AA	AA	AA	AA	AA	AA	AA	AA
	AA	AA	AA	AA	AA	AA	AA	AA
	AA	AA	AA	AA	AA	AA	AA	
105783425								
5336	rs10142690		14	AG	GG	AG	AG	GG
	AG	GG	AG	GG	AG	GG	GG	AG
	AA	AG	AG	AG	AG	AG	AG	AA
	AG	GG	AG	AG	AG	GG	AG	AG
	AG	AG	AG	AA	GG	AG	AG	
62673192								
5337	rs1014276		15	GG	AG	AG	AG	AG
	AA	GG	GG	AG	AG	GG	GG	GG
	AA	GG	AG	AG	AG	AG	AG	AA
	AG	GG	GG	AG	GG	GG	GG	GG
	AA	AG	AG	GG	GG	GG	GG	
87946927								
5338	rs10142790		14	GG	GG	AG	AA	AA
	AA	AA	AG	GG	AG	AA	AA	AA
	AA	AA	GG	AG	AG	AG	AG	AA
	AG	AG	AA	AA	AG	AA	AG	AA
	AA	AG	AA	AG	AG	AG	AG	
95066522								
5339	rs10142801		14	AA	AA	AA	AA	AA
	AA	AA	AA	AA	AA	AA	AA	AA
	AA	AA	AA	AA	AA	AA	AA	AA
	AA	AA	AA	AA	AA	AA	AA	AA
	AA	AA	AA	AA	AA	AA	AA	
54014818								
5340	rs10142808		14	GG	GG	GG	GG	AG
	GG	AG	GG	GG	GG	GG	GG	GG
	GG	GG	GG	GG	GG	GG	GG	GG
	GG	GG	GG	GG	GG	AG	GG	AG
	GG	GG	GG	GG	GG	GG	GG	
94316720								
5341	rs10142830		14	GG	AG	AA	AG	AG
	GG	AG	GG	AG	GG	GG	AA	AG
	AG	AG	AG	GG	GG	GG	AG	GG
	GG	AG	AG	AG	AG	AA	AA	AG
	GG	GG	AG	AG	GG	GG	AG	
96717797								
5342	rs10142838		14	AA	AA	AA	AA	AA
	AA	AA	AA	AA	AA	AA	AA	AA
	AA	AC	AA	AA	AA	AA	AA	AA
	AA	AA	AA	AA	AA	AA	AA	AA
	AA	AA	AA	AA	AA	AA	AA	
88567917								
5343	rs10142843		14	GG	GG	GG	GG	AG
	GG	AG	AA	AG	AA	AG	GG	GG
	GG	GG	AG	AG	GG	AG	AA	AG

	AG	GG	GG	GG	AG	AG	GG	AA
	AG	GG	AG	AG	GG	AG	AG	
29714864								
5344	rs1014290		4	AA	AA	AG	AG	AG
	AA	AG	AG	AG	AG	AG	AG	AG
	AG	AG	AA	AA	AA	GG	GG	AG
	GG	AG	GG	AG	AA	AA	AA	AA
	GG	AG	AA	AG	AA	AA	AA	
10001861								
5345	rs10142958		14	GG	AG	GG	AG	GG
	AG	GG	AG	AG	GG	GG	GG	GG
	AG	AG	GG	GG	GG	GG	GG	GG
	GG	AG	GG	GG	GG	GG	GG	GG
	AG	AG	GG	AG	GG	GG	AG	
65760996								
5346	rs10143011		14	AA	AA	AA	AA	AA
	AA	AA	AA	AA	AA	AA	AA	AA
	AA	AA	AA	AA	AA	AA	AA	AA
	AA	AA	AA	AA	AA	AA	AA	AA
	AA	AA	AA	AA	AA	AA	AA	
91492896								
5347	rs10143036		14	AC	AA	AA	AA	AA
	AA	AA	AA	AA	AA	AA	AC	AC
	AC	AA	AA	AA	AA	AA	AC	AA
	AA	AA	AA	AA	AC	AC	AC	AC
	AA	AA	AA	AA	AC	AC	AA	
90774914								
5348	rs1014306		9	GG	AG	GG	AG	GG
	AG	GG	GG	AG	GG	AG	GG	AG
	AG	AG	AA	AG	AG	GG	GG	GG
	GG	GG	GG	GG	GG	GG	GG	GG
	GG	GG	AG	AG	AG	AG	GG	
90157451								
5349	rs10143067		14	AA	AA	AA	AG	AA
	AG	AA	AA	AA	AA	AG	AA	AA
	AA	AG	AA	AA	AA	AG	GG	AG
	AA	AG	AA	AA	AA	AG	AA	AG
	AA	AA	AA	AA	AG	AA	AG	
88752821								
5350	rs1014308		9	GG	GG	GG	GG	GG
	GG	AG	AG	AA	GG	GG	GG	GG
	GG	AG	AG	AA	AG	GG	AG	GG
	GG	AG	GG	AG	GG	AG	GG	AG
	AA	AA	AG	AG	AA	AG	AA	
16053792								
5351	rs10143084		14	CC	CC	CC	CC	CC
	CC	CC	CC	CC	CC	CC	CC	CC
	CC	CC	CC	CC	CC	CC	CC	CC
	CC	CC	CC	CC	CC	CC	CC	CC
	CC	CC	CC	CC	CC	CC	CC	

32799694
5352 rs10143113 14 GG AG GG GG GG
GG GG GG AG GG GG GG GG
GG GG AG AG AG GG AG GG
GG GG AG AG AG AG AA GG
AG AG GG AG GG GG GG
33111257
5353 rs10143148 14 GG AG AG AA AA
AA AA AG AG AG AA AG AG
AG AG AA AG AA AG AA AA
AA AA AA AA AG AA AG AA
AA AA AG AG AG AA AG
39964127
5354 rs10143156 14 GG AG GG AG AG
AG AG AG AA AG GG AG AG
AG GG AA AG AA AA AA AA
AG GG AG AG GG GG GG GG
AA GG AG GG AG AG GG
54752308
5355 rs10143181 14 AG AG AG GG GG
GG GG GG GG GG GG GG GG
GG GG GG GG GG GG GG GG
GG AG GG AG AG GG GG GG
GG GG GG GG GG GG AG
74203668
5356 rs10143207 14 AA AA AA AC AC
AA CC AA AC AC AA AA AA
AA AA AA AA AA AA AA AA
AA AA AA AA AC AA AC AA
AA AA AA AA AA AA AA
104267761
5357 rs10143215 14 GG GG GG GG GG
GG GG GG GG GG GG GG GG
GG GG GG GG GG GG GG GG
GG GG GG GG GG GG GG GG
GG GG GG GG GG GG GG
31111860
5358 rs10143306 14 AA AA AA AA AA
AA AA AA AA AA AA AA AA
AA AA AA AA AG AA AA AA
AA AA AG AG AA AA AA AA
AA AA AG AG AA AA AA
33861252
5359 rs10143355 14 AG AG AG GG GG
GG GG GG GG GG GG GG GG
GG GG GG AG GG AG GG AG
GG GG GG GG AG GG GG GG
GG AG AG AA GG GG GG
52115376
5360 rs10143389 14 AG GG AG AG GG

AG GG AG GG GG GG GG GG
GG GG AG AG AG AG GG AG
GG GG GG GG AG GG GG AG
AG AG AG AG GG GG GG
104452640
5361 rs10143394 14 AA AA AA AA AA
AA AA AA AA AA AA AA AA
AG GG AA AA AA AA AA AA
AA AG AA AA AA AA AA AA
AA AA AA AA AA AA AA
98407879
5362 rs10143413 14 GG AG GG AG GG
GG AG AG AG AG AA AA AA
GG AA AG AG AA AG GG GG
GG GG AG AG AG AG GG AA
GG AA AG AA AG AG AG
22526881
5363 rs10143478 14 AA AA AG AA AG
AG AA AG AA AG AA AA AA
AA AA AA AA AA AA AA AA
AA AA AA AA AG AA AG AA
AA AA AA AA AA AA AA
33442622
5364 rs10143506 14 GG GG GG GG GG
GG GG GG GG GG GG GG GG
GG GG GG GG GG GG GG GG
GG GG GG GG GG GG GG GG
GG GG GG GG GG GG GG
56546218
5365 rs10143523 14 AA AA AG AA AA
AA AA AG AG AG AG AG AA
AA AA AA AA AA AA AA AA
AA AA AA AA AG GG GG GG
AA AG AA AG AA AA AA
60367870
5366 rs10143550 14 GG GG GG GG GG
GG GG GG GG GG GG GG GG
GG AG AG GG GG GG AG GG
AG AG AG AG AG GG AG GG
GG AG GG GG GG GG GG
37218627
5367 rs10143554 14 AA AA AA GG AG
AG GG GG GG GG AA AG AG
AG AG GG GG GG AG GG AG
AG AA AA AA GG GG GG GG
AA AG AA AA GG AG AG
51874109
5368 rs10143616 14 AG AG AG AA AA
AA AG AG AG AG GG GG AG
AG AG AG GG GG GG AG AG

GG AG GG AG AG AG AG AA
AG AG AA AA AG AG GG
64740175
5369 rs10143694 14 AA AA AA AG AG
AG AG AA AA AA AA AA AA
AA AA AA AA AA AA AG AG
AG AG AA AA AA AA AA AA
AA AA AA AA AA AA AA
34005226
5370 rs10143719 14 AA AA AA AA AA
AA AA AA AA AA AA AA AA
AA AA AA AA AA AA AA AA
AA AA AA AA AA AA AA AA
AA AA AA AA AA AA AA
99298359
5371 rs10143737 14 CC CC AC AC AC
AA CC CC CC CC CC CC AC
AC CC CC CC CC CC CC AC
AC CC CC CC AC CC CC CC
AA AC AC AC AC AC AA
59528006
5372 rs10143739 14 AA AA AG AA AA
AA AA AA AA AA AA AA AA
AA AG AA AA AA AA AA AA
AA AA AA AA AG AA AG AA
AA AA AA AA AA AA AA
28086730
5373 rs10143775 14 GG GG GG GG GG
GG GG GG AG GG GG GG GG
GG AG GG GG GG AG GG GG
GG GG GG GG GG AG GG AG
GG GG GG GG GG AG GG
55944078
5374 rs1014381 4 AG AG AG GG AG
GG AG AG AA AG AA AA AA
AA AA GG AG AG AG AG GG
AA AG AG AG AG AG AA AG
GG AG GG AG GG GG AG
176548782
5375 rs10143813 14 AG AA AG AG AG
GG AA AG AA AG AA AA AG
GG AG GG GG AG GG AG GG
AG AG AG AG AG AA AA AA
AG GG AG GG AG GG AG
62647441
5376 rs10143836 14 AA AA AG AA AA
AA AA AA AA AA AA GG AG
GG GG AG AA AA AG AG AA
AA AG AG AG AG AG AG AG
AA AG AA AA AA AG AA

96640876								
5377	rs1014384		7	AA	AA	AA	GG	AA
	AG	AG	AA	AA	AG	AA	AA	AA
	AA	AA	AA	AG	AA	AG	AA	AG
	AA	AA	AG	AG	AA	AA	AA	AA
	AG	AA	AA	AA	AA	AA	AA	
50847646								
5378	rs10143848		14	AG	AG	GG	AG	AG
	AG	AG	GG	AG	GG	AG	AG	AA
	AA	AG	AG	GG	AG	AA	AA	AG
	AG	AG	AA	AA	AA	AG	AA	AG
	AA	AA	AA	AA	AA	AA	AA	
50844119								
5379	rs10143855		14	GG	GG	GG	GG	GG
	GG	GG	GG	GG	GG	GG	GG	GG
	GG	GG	GG	GG	GG	GG	GG	GG
	GG	GG	GG	GG	GG	AG	AG	GG
	GG	GG	GG	GG	GG	GG	GG	
92906763								
5380	rs10143866		14	AG	AA	AA	AG	AA
	AG	AA	AA	AA	AA	AA	AA	AA
	AA	AA	AG	AA	AA	AA	AA	AA
	AA	AA	AA	AA	AA	AG	AG	AA
	AA	AA	AA	AA	AA	AA	AA	
52884355								
5381	rs10143875		14	GG	AG	GG	AA	AA
	AG	AA	GG	GG	AG	GG	GG	GG
	AG	GG	AA	AA	AG	GG	GG	AG
	AG	AA	AA	AG	GG	GG	GG	GG
	AG	AG	AA	AG	AG	GG	AG	
23584265								
5382	rs1014394		1	AA	AA	AA	AG	AA
	AG	AA	AA	AG	AA	AG	AG	AG
	GG	AG	AG	AG	AG	AA	AG	AA
	AA	AG	AA	AG	AA	AG	AG	AG
	AG	AG	AA	AA	AA	AG	AA	
179800985								
5383	rs10143967		14	AA	AA	AA	AC	AC
	AA	AC	--	--	--	AA	AA	--
	AA	--	--	AC	AC	AC	AC	AC
	--	AA	AA	AA	AC	AA	AC	AA
	AC	--	AA	AA	AC	AA	AC	
96429201								
5384	rs10143973		14	AA	AA	AA	AA	AA
	AA	AA	AA	AA	AA	AA	AA	AA
	AA	AA	AA	AA	AA	AA	AA	AA
	AA	AA	AA	AA	AA	AA	AA	AA
	AA	AA	AA	AA	AA	AA	AA	
49512733								
5385	rs10144029		14	GG	AG	GG	AG	AG

	AA	GG	AG	AG	AG	GG	AG	AG
	AG	GG	AG	AG	GG	AA	GG	AG
	GG	GG	AG	AG	GG	GG	GG	GG
	GG	GG	AG	GG	GG	GG	GG	
38646010								
5386	rs10144090		14	GG	GG	GG	GG	AG
	GG	AG	GG	GG	GG	GG	GG	GG
	GG	GG	GG	GG	GG	GG	AG	GG
	GG	GG	GG	GG	GG	GG	GG	GG
	GG	GG	GG	GG	GG	GG	GG	
67708388								
5387	rs10144091		14	AA	AG	GG	AA	AG
	AG	AG	GG	GG	GG	GG	AG	AG
	AG	GG	GG	GG	GG	AG	AG	AG
	GG	AG	AG	AG	GG	GG	GG	GG
	GG	AG	GG	GG	GG	GG	GG	
98640452								
5388	rs10144115		14	GG	GG	GG	GG	AG
	GG	AG	GG	GG	GG	GG	GG	GG
	GG	GG	GG	GG	GG	AG	GG	AA
	GG	GG	AG	AG	GG	GG	GG	GG
	GG	GG	AG	AG	AG	AG	GG	
50638660								
5389	rs1014415		1	GG	AG	GG	AG	GG
	GG	AG	AA	AG	AA	AA	AA	AA
	AG	AA	AA	AA	AA	AG	AG	AG
	AA	AA	AA	AA	AA	AG	AA	AA
	AG	AG	AA	AG	AA	AA	AG	
92502060								
5390	rs10144216		14	GG	GG	GG	GG	GG
	GG	GG	GG	GG	GG	GG	GG	GG
	GG	GG	GG	GG	GG	GG	GG	GG
	GG	GG	GG	GG	GG	GG	GG	GG
	GG	GG	GG	GG	GG	GG	GG	
50158183								
5391	rs10144225		14	AA	AA	AA	AG	AG
	AA	AG	AA	AA	AA	AA	AA	AA
	AG	AG	AA	AA	AA	AA	AA	AA
	AA	AA	AA	AA	AA	AA	AA	AA
	AA	AA	AA	AA	AG	AA	AG	
64704994								
5392	rs10144271		14	GG	GG	GG	GG	GG
	GG	GG	AG	AG	AA	GG	GG	GG
	GG	GG	GG	GG	GG	AG	AG	AG
	GG	GG	GG	GG	AG	GG	AG	GG
	AG	GG	GG	GG	AG	AG	AG	
104447711								
5393	rs10144278		14	GG	AG	GG	AG	AG
	AG	GG	AA	GG	AG	AG	AG	AG
	AG	AG	AG	AA	AG	AG	AA	AG

	AG	GG	AG	AA	GG	AG	GG	AG
	GG	AG	AG	AG	GG	AG	GG	
23749595								
5394	rs10144289		14	GG	GG	GG	AG	GG
	AG	GG	GG	AG	GG	GG	GG	GG
	AG	AG	GG	GG	AG	GG	AG	GG
	GG	AG	GG	AG	AG	AG	AG	AA
	GG	AG	GG	GG	AG	AG	GG	
70994611								
5395	rs10144318		14	AC	CC	CC	AC	AC
	CC	AA	CC	CC	CC	AC	CC	AC
	AC	CC	AA	AC	AA	AC	CC	AC
	CC	CC	CC	CC	AA	CC	AC	AC
	AC	AA	AA	AA	AA	AA	AA	
53189731								
5396	rs10144339		14	GG	GG	AG	AG	GG
	AG	GG	AG	GG	AG	AG	GG	AG
	AG	AA	GG	GG	GG	AG	GG	GG
	AG	AG	GG	GG	AG	GG	GG	AG
	GG	GG	GG	GG	AA	AA	AG	
33106344								
5397	rs10144371		14	AA	AA	AA	AA	AC
	AA	AC	AA	AA	AA	AA	AA	AA
	AA	AA	AA	AA	AA	AA	AC	AA
	AA	AA	AA	AA	AA	AA	AA	AA
	AA	AA	AA	AA	AA	AA	AA	
67604637								
5398	rs1014440		19	AA	AA	AA	AA	AG
	AG	AA	AG	AG	AG	AA	AA	AA
	AG	AG	AA	AA	AA	AG	AG	AG
	AA	GG	AG	AA	GG	GG	GG	GG
	AA	AG	AA	AG	AA	AA	AA	
29621891								
5399	rs10144421		14	AA	AA	AG	AA	AG
	AG	AA	AA	AG	AA	AA	AG	AG
	GG	AG	AA	AA	AA	AG	AG	AG
	AG	AG	GG	GG	AA	AA	AA	AA
	AG	GG	AA	AG	AG	AA	AG	
95911835								
5400	rs10144478		14	AA	AA	AA	AA	AA
	AA	AA	AA	AA	AA	AA	AA	AA
	AA	AA	AA	AA	AA	AA	AA	AA
	AA	AA	AA	AA	AA	AA	AA	AA
	AA	AA	AA	AA	AA	AA	AA	
66132965								
5401	rs10144492		14	AG	AG	AA	AG	AA
	AG	AG	GG	GG	GG	AG	GG	GG
	GG	GG	GG	AG	AG	AG	GG	GG
	GG	AA	AG	AG	AG	GG	GG	AG
	GG	AG	AA	AG	AA	AG	AG	

101991506
5402 rs10144550 14 AA AA AA AG AG
AG GG AA AA AA GG AG GG
AA GG AA AG AG AG AG AG
AG AG GG GG AA AG AG AA
AA GG AG AG AA AG AA
20635795
5403 rs10144553 14 AG GG GG AG AA
AG AA GG AG GG GG AG AG
AG AG AG AG AG AG AG AG
GG AG GG GG AA GG AG AG
GG GG AG AG AG AG AG
73200847
5404 rs10144554 14 CC CC CC CC CC
CC CC AC CC AC CC CC CC
CC CC CC CC CC CC CC CC
CC AA CC AC AC CC AC AC
CC CC CC CC CC CC CC
65321514
5405 rs10144583 14 GG GG GG GG GG
GG GG GG GG GG GG GG GG
GG GG GG GG GG GG GG GG
GG GG GG GG GG GG GG GG
GG GG GG GG GG GG GG
105893411
5406 rs10144593 14 GG GG GG GG GG
GG GG GG GG GG GG GG GG
GG GG GG GG GG GG GG GG
GG GG GG GG GG GG GG GG
GG GG GG GG GG GG GG
97862721
5407 rs10144609 14 AG AG AA AG AA
AG AA GG GG GG AA AG AG
AG AG AG AG AG AG AA GG
AA AA AA AA AA AA AA AA
AG AA GG AG GG AG AG
87325168
5408 rs10144615 14 AA AG AG AG AA
AA AG AG AG AG GG AG GG
GG GG AG AG AG AG AA AG
AA AA AA AA AG AG GG AG
AG GG AA AG AG AA AG
80885386
5409 rs10144627 14 GG AG AA GG AG
AG GG AG AA AG GG AA AG
AG AG AA AG AG GG AG GG
AA AG AA AA AG AG GG AA
AG AG GG GG GG AG AG
89696533
5410 rs10144660 14 AG AA AA AG AG

	AA	GG	AA	AA	AA	AA	AA	AA
	AA	AA	AA	AA	AA	AA	AA	AA
	AA	AA	AG	AG	AA	AA	AA	AA
	AA	AA	AG	AA	AA	AA	AG	
53476894								
5411	rs1014472		13	AG	GG	AG	GG	GG
	GG	GG	GG	GG	AG	AG	GG	AG
	AG	AA	AG	GG	AG	GG	GG	GG
	GG	GG	GG	GG	GG	GG	GG	GG
	GG	AG	AG	AG	GG	GG	GG	
27478404								
5412	rs10144720		14	AA	AA	AA	AC	AA
	AC	AA	AA	AA	AA	AA	AA	AA
	AA	AA	AA	AC	AA	AA	AA	AA
	AA	AA	AA	AA	AA	AA	AA	AA
	AA	AA	AA	AA	AA	AA	AA	
65687536								
5413	rs10144748		14	AA	AG	GG	AA	AA
	AA	AA	AA	AA	AA	AA	AA	AA
	AA	AA	AG	AA	AA	AG	AG	AA
	AG	AA	AG	AG	AG	AA	AA	AG
	AG	AA	AA	AA	AA	AA	AG	
99552213								
5414	rs10144771		14	AG	AG	AG	AG	AG
	GG	AA	GG	AG	GG	GG	AG	GG
	GG	AG	AG	GG	GG	GG	AG	GG
	AG	GG	AG	AG	AG	GG	AG	GG
	AG	GG	GG	GG	GG	GG	AG	
94778653								
5415	rs10144775		14	CC	CC	CC	CC	CC
	CC	CC	CC	CC	CC	AA	AC	AC
	AC	CC	CC	CC	AC	CC	AC	CC
	CC	CC	AC	AC	CC	CC	CC	CC
	AC	CC	AC	AC	AC	CC	AC	
33856533								
5416	rs10144817		14	AG	GG	GG	GG	GG
	GG	GG	GG	GG	GG	AG	GG	AG
	AG	AG	GG	AG	GG	AG	GG	GG
	GG	GG	GG	GG	GG	AG	AG	GG
	AA	GG	GG	GG	GG	GG	AG	
40823504								
5417	rs10144843		14	AA	AA	AA	AA	AA
	AA	AA	AA	AA	AA	AA	AA	AA
	AA	AA	AA	AA	AA	AA	AA	AA
	AA	AA	AA	AA	AA	AA	AA	AA
	AA	AA	AA	AA	AA	AA	AA	
93662275								
5418	rs10144870		14	AA	AA	AA	AA	AG
	AA	AG	AA	AA	AA	AA	AA	AA
	AA	AA	AA	AA	AA	AA	AG	AA

AA AA AA AA AA AA AA AA
AA AA AA AA AA AA AA
67543424
5419 rs10144879 14 AA AA AA AC AA
AC AA AA AC AA AC AC CC
AC AA AA AA AA AC AA AA
AA AA AA AA AA AA AA AA
AA AC AA AC AA AC AA
91991503
5420 rs10144899 14 AA AA AG AA AA
AA AA AA AA AA AA AA AA
AA AA AA AA AA AA AA AG
AG AA AG AG AA GG AG AG
AA AA AA AA AA AG AA
82058340
5421 rs10144903 14 AC AC CC AA AC
AC AA AA AA AA AC AA AC
AA AC AA AA AA AA AC AA
AC AC AC AC AC AA AA AC
AA AC AC CC AA AA AA
30272309
5422 rs10144909 14 AA AA AA AA AA
AA AA AA AA AA AA AA AA
AA AA AA AA AA AA AA AA
AA AA AA AA AA AA AA AA
AA AA AA AA AA AA AA
49743567
5423 rs10144921 14 GG GG GG GG GG
GG GG GG GG GG GG GG GG
GG GG GG GG GG GG GG GG
GG GG GG GG GG GG GG GG
GG GG GG GG GG GG GG
31784104
5424 rs10144954
14 -- -- -- -- -- -- -- --
-- -- -- -- -- -- -- --
-- -- -- -- -- -- -- --
-- -- -- -- -- -- -- --
-- -- -- -- 51832412
5425 rs1014497 2 AA AG AA AG AA
AG AA AA AA AA AA AA AA
AA AA AA AA AA AA GG AG
AA AA AA AA AA AG AA AG
AA AG AA AG AA AA AA
200241229
5426 rs10144975 14 GG GG GG AG GG
AG GG AG AG GG AG AG AG
AG AG GG GG GG GG AG GG
AG AG AG AG GG GG GG GG
AG AA AG AA GG GG AG

65773982								
5427	rs10144981		14	AA	AA	AA	AG	AA
	AG	AA	AG	AA	AG	AA	AA	AA
	AA	AA	AA	AA	AA	AA	AA	AA
	AA	AA	AA	AA	AA	AG	AG	AA
	AA	AA	AA	AA	AA	AA	AA	
53119201								
5428	rs1014499		11	AG	AG	AA	GG	AA
	AG	AG	GG	AG	AG	AA	AG	AG
	AG	AG	AG	AG	AG	AG	AG	GG
	AA	AG	AG	AG	GG	GG	GG	GG
	AG	AG	AG	AG	GG	AG	AG	
93934545								
5429	rs10144999		14	GG	GG	GG	GG	AG
	GG	AG	GG	GG	GG	GG	GG	GG
	GG	AG	AG	GG	GG	GG	GG	GG
	AG	AG	GG	GG	GG	GG	GG	GG
	GG	GG	GG	GG	GG	GG	AG	
42552103								
5430	rs10145013		14	GG	GG	GG	AG	AG
	AA	AG	GG	GG	AG	GG	GG	GG
	AG	AG	GG	GG	GG	AG	GG	GG
	AA	AG	AA	AA	AG	AG	AG	AA
	AG	AG	AG	AG	AG	AG	AG	
64941591								
5431	rs10145014							
14	--	--	--	--	--	--	--	--
	--	--	--	--	--	--	--	--
	--	--	--	--	--	--	--	--
	--	--	--	--	--	--	--	--
	--	--	--	--	23436283			
5432	rs10145117		14	GG	GG	GG	GG	GG
	GG	GG	GG	GG	GG	GG	GG	GG
	GG	GG	GG	GG	GG	GG	GG	GG
	GG	GG	GG	GG	GG	GG	GG	GG
	GG	GG	GG	GG	GG	GG	GG	
50693291								
5433	rs10145239		14	AA	AA	AA	AA	AA
	AA	AA	AA	AA	AA	AA	AA	AA
	AA	AA	AA	AA	AA	AA	AA	AA
	AA	AA	AA	AA	AA	AA	AA	AA
	AA	AA	AA	AA	AA	AA	AA	
63075442								
5434	rs10145379		14	AC	AA	AA	AC	AC
	AC	AC	AC	AC	AC	AC	CC	AC
	AC	AA	AA	AA	AC	AC	CC	AC
	CC	AC	AC	AC	AC	CC	CC	AC
	AC	AC	AC	AC	AC	AC	AC	
38065813								
5435	rs10145411		14	GG	GG	GG	GG	GG

	GG	GG	GG	GG	GG	GG	GG	GG
	GG	GG	GG	GG	GG	GG	GG	GG
	GG	GG	GG	GG	GG	GG	GG	GG
	GG	GG	GG	GG	GG	GG	GG	
62966803								
5436	rs10145423		14	GG	GG	GG	GG	GG
	GG	GG	GG	GG	GG	GG	GG	GG
	GG	GG	GG	GG	GG	GG	GG	GG
	GG	GG	GG	GG	GG	GG	GG	GG
	GG	GG	GG	GG	GG	GG	GG	
21679775								
5437	rs10145477		14	AC	AC	AC	AA	AA
	AA	AA	AA	AA	AA	AC	AA	AC
	AC	AA	AA	AC	AC	AA	AC	AA
	AA	AA	AA	AA	AA	AA	AA	AA
	AA	AC	AA	AA	AA	AA	AA	
43203919								
5438	rs10145485		14	AA	AA	AA	AA	AA
	AA	AA	AA	AA	AA	AA	AA	AA
	AA	AA	AA	AA	AA	AA	AA	AA
	AA	AA	AA	AA	AA	AA	AA	AA
	AA	AA	AA	AA	AA	AA	AA	
95632174								
5439	rs10145507		14	AA	AA	AA	AA	AA
	AA	AA	AA	AA	AA	AA	AA	AA
	AA	AA	AA	AA	AA	AA	AA	AA
	AA	AA	AA	AA	AA	AA	AA	AA
	AA	AA	AA	AA	AA	AA	AA	
32971060								
5440	rs10145516		14	AG	AG	AA	AG	GG
	AG	GG	AG	AG	AG	AG	AG	GG
	GG	AG	AG	GG	GG	GG	GG	AG
	AG	AG	GG	GG	GG	GG	GG	GG
	GG	AG	GG	GG	GG	GG	GG	
38721995								
5441	rs10145536		14	AA	AG	AG	AA	AG
	AA	AG	AG	AG	AG	AG	AA	AG
	AG	AG	GG	GG	AG	AG	AA	AG
	AA	AA	AA	AA	AG	AA	AG	AA
	AG	AA	GG	AG	AA	AG	AA	
37738197								
5442	rs10145558		14	AA	AA	AA	GG	AA
	AG	AG	AG	AA	AG	AA	AA	AA
	AA	AA	AA	AA	AA	AG	AA	AA
	AA	AG	AG	AG	AA	AA	AA	AA
	AG	AA	AA	AA	AG	AA	AG	
94971978								
5443	rs10145623		14	GG	GG	GG	GG	GG
	GG	GG	GG	GG	GG	GG	GG	GG
	GG	GG	GG	GG	GG	GG	GG	GG

GG GG GG GG GG GG GG GG
GG GG GG GG GG GG GG
56677450
5444 rs10145636 14 AG AG GG GG GG
AG GG AG AA GG AG GG AG
GG AG AA AA AA AG GG AG
AG AG AA AA GG AG AG GG
GG AG AG AG AG AG AG
21271573
5445 rs10145648 14 AG AG AG AA AA
AA AA AG AA AG GG AA AG
AG AG AA AG AG AA AA AA
AA AG AA AA AG AA AG AA
AA AA AA AA AA AA AA
101305120
5446 rs1014570 7 GG GG GG AG AA
AA AG GG GG GG GG GG GG
GG GG AG GG GG AG AG AG
GG AG GG AG AG GG AG AG
AG GG GG GG AG GG AG
38510787
5447 rs10145758 14 AG AG AA AG GG
AG GG GG GG GG GG GG GG
GG GG GG GG GG GG GG GG
AG GG GG GG AG AG AA GG
GG GG GG GG GG GG GG
95727480
5448 rs10145768 14 AG AG AG AA AA
AA AA AA AG AA AA AG AG
AG AA AA AG AA AG AA AA
AA AA AA AA AA AG AA AG
AG AG AA AG AG AA AG
42941645
5449 rs1014577 13 AA AA AC AC AA
AC AA AA AA AA AC AC CC
AC AC AA AA AA AC AC AC
CC AA CC CC AA AA AA AA
AA AC AA AA AA AA AA
60207346
5450 rs10145791 14 CC CC CC CC CC
CC CC CC CC CC CC CC CC
CC CC CC CC CC CC CC CC
CC CC CC CC CC CC CC CC
CC CC CC CC CC CC AC
54881570
5451 rs10145843 14 AA AA AA AG AA
AA AG AA AA AA AA AA AA
AA AA AA AA AA AA AA AG
AA AG AG AG AA AG AG AA
AG AA AA AA AG AG AA

46154010							
5452	rs10145865	14	GG	GG	GG	GG	GG
	GG	GG	GG	GG	GG	GG	GG	GG
	GG	GG	GG	GG	GG	GG	GG	GG
	GG	GG	GG	GG	AG	GG	GG	AG
	GG	GG	GG	GG	GG	GG	GG	
92919834							
5453	rs1014588	5	AG	AG	AG	AG	GG
	GG	AG	GG	GG	GG	GG	GG	GG
	GG	GG	AG	AG	AG	GG	GG	AG
	AG	GG	GG	AG	AG	GG	AG	GG
	GG	GG	AA	AG	GG	GG	GG	
41149284							
5454	rs10145934	14	GG	GG	GG	GG	GG
	GG	GG	GG	GG	GG	GG	GG	GG
	GG	GG	GG	GG	GG	GG	GG	GG
	GG	GG	GG	GG	GG	GG	GG	GG
	GG	GG	GG	GG	GG	GG	GG	
103928819							
5455	rs1014595	3	AA	AA	AA	AA	AA
	AA	AG	AA	AA	AG	AA	AA	AA
	AA	AA	AA	AG	AG	AA	AA	AA
	AA	AA	AA	AA	AA	AA	AA	AA
	AA	AG	AG	AG	AA	AA	AG	
60516133							
5456	rs10146025	14	GG	GG	GG	GG	GG
	GG	GG	GG	GG	GG	GG	GG	GG
	GG	GG	GG	GG	GG	GG	GG	GG
	GG	GG	GG	GG	GG	GG	GG	GG
	GG	GG	GG	GG	GG	GG	GG	
54075835							
5457	rs1014604	21	AA	AG	AG	AG	GG
	GG	AG	AG	GG	GG	GG	GG	GG
	AG	GG	GG	AG	AG	GG	AG	GG
	AG	AG	GG	AG	AG	AA	AG	AA
	AG	AG	AG	AG	GG	GG	GG	
18341062							
5458	rs10146061	14	AA	AA	AA	GG	GG
	AG	GG	AG	AG	AG	AG	AA	AG
	AG	AG	AG	AG	AG	AA	AA	AA
	AA	AA	AA	AA	AA	AA	AA	AA
	GG	GG	AG	AG	AA	AA	AA	
21114562							
5459	rs10146063	14	GG	GG	GG	AG	GG
	AG	GG	GG	GG	GG	AG	GG	AG
	GG	GG	GG	GG	GG	GG	GG	AG
	GG	AA	GG	AG	AG	GG	GG	AG
	GG	GG	GG	GG	GG	GG	GG	
91226196							
5460	rs10146152	14	GG	GG	GG	GG	GG

GG GG GG AA GG GG GG GG
GG AG GG GG AG GG GG GG
GG GG GG GG AG GG GG AG
AG AG AG AG GG GG GG
34020584
5461 rs10146172 14 AA AA AA AA AG
AG AA AA AA AA AG AA AG
AG AG AA AA AG AA AA AA
AA AA AA AA AA AA AA AA
AA AA AA AA AA AA AA
75042338
5462 rs10146183 14 CC CC CC CC CC
CC CC CC CC CC CC CC CC
CC CC CC CC CC CC CC CC
CC CC CC CC CC CC CC CC
CC CC CC CC CC CC CC
106863833
5463 rs10146237 14 AA AG AA AG AG
GG AA GG AA GG AG AA AG
AG GG GG AG AG GG AG GG
GG GG GG GG GG AG GG AG
GG AG AG AG GG AG GG
45089146
5464 rs10146263 14 AG GG GG AG GG
GG AG GG GG GG AA GG AG
GG GG GG GG GG GG GG GG
GG AG GG GG GG AG AG GG
AG GG GG GG AG GG AG
94942391
5465 rs10146334 14 GG GG GG GG AG
GG AG GG GG GG GG GG GG
GG GG AA AG AG GG GG GG
GG GG GG GG GG GG GG GG
GG GG GG GG GG GG GG
21549654
5466 rs10146335 14 GG AG AG AG GG
GG AG GG AG GG AG GG AG
GG GG GG GG GG AG GG AG
AG AG AG AG GG AA AG AG
AG AG AG AG GG AG GG
92423309
5467 rs10146416 14 AA AA AA AG AA
AA AG AA AA AA AG AG AG
AG AG AA AG AG AA AA AG
AG AG AG AG AG AG AA GG
AA AA AA AA AA AA AA
21519599
5468 rs10146422 14 AG GG AG AA AA
AA AA AG AA AG AG AG AG
AG AG AG AG AA AA AA AA

	AG	AG	AG	AG	AA	AA	AA	AA
	GG	AG	AA	AA	AA	AG	AA	
30839557								
5469	rs10146559		14	CC	CC	CC	AC	AC
	AC	AC	CC	CC	CC	CC	CC	CC
	AC	CC	CC	CC	AC	AC	CC	AC
	CC	CC	CC	AC	AC	AC	AC	AC
	CC	CC	AC	AC	AC	CC	AC	
25579277								
5470	rs10146581		14	AG	GG	AG	AA	AG
	AG	AA	AA	AA	AA	AA	AA	AA
	AG	AG	AA	AA	AA	AA	AA	AA
	AA	AA	AA	AA	AA	AA	AA	AA
	AA	AA	AA	AA	AA	AA	AA	
96919159								
5471	rs10146584		14	AG	GG	GG	GG	GG
	GG	GG	AG	AG	AG	GG	GG	GG
	GG	GG	GG	GG	GG	GG	GG	GG
	GG	GG	GG	GG	GG	GG	GG	GG
	GG	AG	GG	AG	GG	GG	GG	
77019135								
5472	rs10146661							
14	--	--	--	--	--	--	--	--
	--	--	--	--	--	--	--	--
	--	--	--	--	--	--	--	--
	--	--	--	--	--	--	--	--
	--	--	--	--	35696826			
5473	rs10146730		14	AA	AA	AA	AA	AA
	AA	AA	AA	AA	AA	AA	AA	AA
	AA	AA	AA	AA	AA	AA	AA	AA
	AA	AA	AA	AA	AA	AA	AA	AA
	AA	AA	AA	AA	AA	AA	AA	
103277914								
5474	rs10146733		14	GG	GG	GG	GG	AG
	GG	AG	GG	GG	GG	AG	AG	AG
	GG	AG	GG	GG	GG	AG	GG	GG
	GG	GG	GG	GG	GG	GG	GG	GG
	GG	GG	AG	GG	GG	GG	GG	
22132807								
5475	rs10146827		14	AA	AA	AA	AA	AA
	AA	AA	AA	AA	AA	AA	AA	AA
	AA	AA	AA	AA	AA	AA	AA	AA
	GG	AA	GG	AG	AG	GG	GG	GG
	AG	AA	AA	AA	AG	AG	AG	
73894496								
5476	rs10146912		14	AA	AA	AG	AA	AG
	AG	AA	AA	AG	AA	AA	AA	AA
	AA	AG	AG	AA	AA	AA	AG	AA
	AG	GG	AG	AG	AA	AA	AA	AA
	AA	AG	AA	AA	GG	AG	AG	

50164452							
5477	rs10146962	14	AG	AA	AA	AA	AA
	AA	AG	AG	GG	AG	AG	AA	AG
	AG	AG	AG	AA	AA	AA	AG	AG
	AA	AA	AA	AA	AG	AG	AG	AG
	AA	AA	GG	AG	AA	AG	AG	
101170540							
5478	rs10146966	14	CC	AC	AC	CC	AC
	AC	CC	AC	CC	AC	AC	AC	CC
	CC	AC	AC	AC	AC	CC	AC	CC
	AA	AA	AA	AA	AC	CC	CC	AC
	AC	CC	CC	CC	AC	AA	CC	
39974741							
5479	rs10146983	14	AA	AA	AG	GG	GG
	GG	GG	GG	GG	GG	GG	AG	AG
	AG	AG	GG	AG	GG	GG	AG	GG
	AG	AG	AG	AA	GG	GG	GG	GG
	GG	AG	AG	AG	AG	AA	AG	
72268119							
5480	rs10147011	14	CC	CC	CC	CC	AC
	CC	AC	AA	AA	AA	CC	AC	CC
	CC	CC	CC	CC	CC	CC	CC	CC
	AC	CC	AC	AC	CC	AC	CC	CC
	CC	CC	CC	CC	CC	CC	CC	
81557279							
5481	rs1014706	X	GG	AG	AA	AA	AA
	AA	AA	AA	AA	AA	AA	AA	AA
	AA	AA	AA	AA	AA	AA	AA	AA
	AA	AA	GG	AA	AA	AA	AA	AA
	AA	AA	AA	AA	AA	AA	AA	
8780971							
5482	rs10147093	14	GG	GG	GG	GG	GG
	GG	GG	GG	GG	GG	AG	GG	GG
	GG	GG	AG	AG	AG	GG	GG	AG
	GG	GG	GG	GG	GG	GG	GG	GG
	GG	GG	GG	GG	GG	AG	GG	
26415776							
5483	rs10147171	14	AA	AA	AA	AA	AA
	AA	AA	AA	AA	AA	AG	AA	AG
	AA	AA	AA	AA	AA	AA	AA	AA
	AA	AA	AA	AA	AA	AA	AA	AA
	AA	AA	AG	AA	AA	AA	AA	
96727874							
5484	rs10147224	14	AA	AA	AG	AA	AA
	AA	AA	AA	AA	AA	AA	AA	AA
	AA	AA	AA	AA	AA	AA	AA	AG
	AG	AG	AG	AG	AA	AA	AA	AA
	AG	AG	AA	AA	AA	AA	AA	
58881694							
5485	rs1014724	X	GG	AG	AA	AA	AA

AA AG AG AG AA AA AA AG
AG AG GG GG GG AG AG GG
GG GG GG GG AG GG GG AA
AA AA GG AA AG AG GG
29419910
5486 rs10147261 14 GG GG GG GG GG
GG GG GG GG GG GG GG GG
GG GG GG GG GG GG GG AG
GG GG AG AG GG AG AG GG
GG AG GG AG AG AG AG
36198782
5487 rs10147286 14 AG AA AA AA AA
AA AA AA AG AA AA AA AA
AA AG AA AA AA AG AA AA
AA AG AG AG AG AA AG AA
AA AA AA AA AG AA AG
39696065
5488 rs10147304 14 GG GG GG GG AG
GG AG GG GG GG GG GG GG
GG GG GG GG GG GG GG GG
GG GG GG GG GG GG GG GG
GG GG GG GG AG GG AA
79079176
5489 rs10147312 14 AA AG AG GG GG
GG GG AA AG AA GG GG GG
AG AA AG AG AG GG AG AG
AA AA AA AA AA AA AA AA
AA AG AG AA AG AG AG
93314692
5490 rs10147320 14 AA AA AG AA AG
AG AA AG AA AG AA AA AA
AA AG AG AA AA AG AA AA
AA AG AG AG AA AA AA AA
GG AA AG AA GG AG GG
48978532
5491 rs10147384 14 AA AA AA AA AA
AA AA AA AA AA AC AC AA
AA AA AA AA AA AA AA AA
AA AA AA AA AC AA AA AA
AA AA AA AA AA AA AA
47981900
5492 rs10147449 14 AA AA AA AA AA
AA AA AG AA AG AA AA AA
AA AG AA AA AA AA AA AG
AA AA AA AA AA AA AA AA
AA AA AA AA AA AA AA
44024330
5493 rs10147461 14 GG AG AA GG AG
AG AG AA AA AA AA AG AG
AG AA AA AA AA AG AG AG

AA AG AG AG AA AA AA AA
AA AG AA AA AA AA AA
98618803
5494 rs10147469 14 GG GG GG GG GG
GG GG GG GG GG GG GG GG
GG GG GG AG AG GG AG AG
GG GG GG GG GG AG AG GG
AG AG GG AG GG GG GG
91043669
5495 rs1014752 X GG AG AA AA GG
AA AG AA AA AA AA AA AG
AA AA AA AG AG AG GG AA
GG AA GG AA AA GG AG AA
GG AG AA AA AA AG AA
136663679
5496 rs10147554 14 AA AA AA AA AA
AA AA AA AA AA AA AA AA
AA AA AA AA AA AA AA AA
AA AA AA AA AA AA AA AA
AA AA AA AA AA AA AA
89902967
5497 rs1014756 20 AG GG GG AG GG
GG AG GG GG AG AA AA AA
AA AA AA AG AG AG GG GG
GG AG GG GG GG GG GG GG
AG AG GG AG AG AA GG
49808801
5498 rs10147579 14 AG AG AA AG AG
GG AG AG GG AG AA GG AG
AG AG GG AG AG AG AG AG
GG AG GG GG AG AG AG AG
AG AG AG AG AG GG AG
101999598
5499 rs10147611 14 GG GG GG AG GG
GG AG GG GG GG GG GG GG
GG AG GG GG GG GG AG GG
GG GG AG AG GG GG GG GG
GG GG GG GG GG GG AG
54234753
5500 rs1014767 19 AG AG AG AG AG
AA AG AG AG AG AA AG AA
AA AG AA AG AG AG GG AG
GG GG GG GG AG GG GG AG
AG AG GG AG AG AG AG
58528303
5501 rs10147689 14 GG AG AG AA AG
AA AG GG AG GG AA AA AA
AG GG AG AG AG AA AG AG
GG GG GG GG GG AG GG AG
GG AG AA AG AA AG AA

93274211							
5502	rs10147734	14	AA	AA	AA	AG	AA
	AG	AA	AG	AA	AG	AA	AA	AA
	AA	AA	AA	AA	AA	AA	AA	AA
	AA	AA	AA	AA	AA	AA	AA	AA
	AA	AA	AA	AA	AA	AA	AA	
35802564							
5503	rs10147766	14	AG	GG	AG	AG	AA
	AG	AA	AA	AG	AA	AA	AA	AA
	AG	GG	AA	AG	AG	AG	AA	GG
	AA	AG	AA	AA	AG	AG	AG	AG
	AG	AA	AG	AA	AA	AA	AA	
84789495							
5504	rs10147815	14	GG	AG	GG	GG	GG
	GG	GG	GG	AG	GG	GG	GG	GG
	GG	AG	GG	GG	GG	GG	GG	AG
	GG	AG	AA	AA	AG	AG	AG	AG
	GG	AA	GG	AG	GG	AG	GG	
35985525							
5505	rs1014784	X	AA	AA	AA	AA	AA
	AA	AA	AA	AA	AA	AA	AA	AA
	AA	AA	AA	AA	AA	AA	AA	AA
	AA	AA	AA	AA	AA	AA	AA	AA
	AA	AG	AA	GG	AA	AA	AA	
41858939							
5506	rs10147843	14	GG	AG	AG	GG	GG
	GG	GG	AG	GG	AG	GG	GG	GG
	GG	GG	GG	GG	AG	AG	AG	GG
	AG	GG	AG	AG	GG	GG	GG	GG
	GG	AA	AG	AG	AG	AG	AG	
78739801							
5507	rs10147875	14	AA	AA	AA	AA	AA
	AA	AA	AA	AA	AA	AA	AA	AA
	AA	AA	AA	AA	AA	AA	AA	AA
	AA	AA	AA	AA	AA	AA	AA	AA
	AA	AA	AA	AA	AA	AA	AA	
52963178							
5508	rs10147923	14	AG	GG	AG	AG	GG
	AG	GG	AG	GG	GG	GG	GG	GG
	GG	GG	AG	AG	AG	AG	GG	AG
	GG	GG	GG	GG	AG	GG	GG	AG
	AG	AG	AG	AG	GG	GG	GG	
104441925							
5509	rs10147953	14	AA	AA	AA	AA	AA
	AA	AG	AG	AA	AG	AG	AG	AA
	AA	AA	AA	AG	AG	AA	AA	AA
	AA	AA	AA	AA	AA	AG	AG	AA
	AA	AA	AG	AG	AG	AG	AA	
104624689							
5510	rs10147980	14	GG	GG	GG	GG	GG

	GG	GG	AG	AG	AG	GG	GG	GG
	GG	GG	GG	GG	GG	GG	GG	GG
	GG	GG	GG	GG	GG	GG	GG	GG
	GG	GG	GG	GG	GG	GG	GG	
57915163								
5511	rs10147988		14	AG	AG	GG	AA	AA
	AA	AG	AG	AA	AG	AG	AA	AA
	AA	AG	AA	AG	AG	AA	AA	AA
	AG	AG	AA	AA	GG	AA	AG	AG
	AG	AA	AA	AA	AG	AA	AG	
101297515								
5512	rs10147990		14	AA	AA	AA	AG	AA
	AG	AA	AA	AA	AA	GG	AG	GG
	AG	AG	AA	AG	AG	GG	AG	GG
	AG	AG	AG	AG	AA	GG	AG	AG
	AG	GG	AA	AG	GG	GG	AG	
93703854								
5513	rs10148008		14	AG	AG	AG	AG	AA
	AG	AA	GG	AG	GG	AA	AA	AA
	AA	AA	AA	AA	AA	AA	AG	AA
	AA	AA	AA	AA	AA	GG	AG	AG
	AA	AG	AA	AA	AG	AA	AG	
53609869								
5514	rs1014803		21	AA	AG	AA	AG	AG
	GG	AA	GG	GG	AG	AA	AG	AG
	AG	GG	AG	AG	AG	AG	GG	GG
	AA	AG	AG	AG	AA	AG	AA	AG
	AA	AG	AA	AA	AG	GG	AA	
37094040								
5515	rs10148054		14	AC	AA	AC	AA	AC
	AC	AA	CC	CC	AC	CC	AA	AC
	AA	AC	AC	AC	CC	AA	AA	AC
	CC	AC	CC	CC	AC	CC	AC	CC
	AC	CC	CC	CC	AA	AA	AC	
83439714								
5516	rs10148111		14	--	--	--	AG	--
	--	--	AG	AG	AG	--	--	AG
	--	--	AG	AG	--	--	AG	AG
	AG	AG	AG	--	--	--	--	--
	AG	AG	--	--	AG	AG	AA	
33012142								
5517	rs1014812		14	AG	AG	AA	GG	AG
	GG	AG	GG	GG	GG	AG	AG	AG
	AG	GG	GG	GG	GG	GG	GG	AG
	AG	AG	AG	AA	GG	AG	AG	GG
	GG	AG	AG	GG	GG	GG	AG	
90933577								
5518	rs10148163		14	GG	GG	GG	AG	GG
	AG	GG	GG	GG	GG	GG	GG	GG
	GG	GG	GG	GG	GG	GG	GG	GG

GG	GG	GG	GG	GG	AG	GG	AG
AG	GG	GG	GG	GG	GG	GG	

52287675

5519	rs10148171	14	AA	AA	AA	AA	AA
AA	AA	AG	AG	AG	AA	AA	AA
AA	AA	AA	AG	AA	AA	GG	AG
AG	AG	AG	AG	AA	AG	AA	AG
AG	AA	AG	AA	AG	AG	AG	

26064623

5520	rs10148188	14	AA	AA	AA	AA	AA
AA	AA	AA	AA	AA	AA	AA	AA
AA	AA	AA	AA	AA	AA	AA	AA
AA	AA	AA	AA	AA	AA	AA	AA
AA	AA	AA	AA	AA	AA	AA	

56051138

5521	rs10148212	14	AA	AA	AC	AA	AC
AA	AC	AA	AA	AA	AA	AA	AA
AA	AA	AA	AA	AA	AA	AC	AA
AA	AA	AA	AA	AA	AA	AA	AA
AA	AA	AA	AA	AA	AA	AA	

67095613

5522	rs10148219	14	AG	AA	AG	AA	AA
AA	AA	AA	AA	AA	AA	AA	AA
AA	AA	AA	AA	AA	AG	AA	AG
AA	AA	AA	AA	AA	AA	AA	AA
AG	AA	AG	AG	AA	AA	AA	

63736429

5523	rs10148225	14	AA	AA	AA	AA	AA
AA	AA	AA	AA	AA	AG	AA	AG
AG	AG	AG	AA	AG	GG	AA	GG
AG	GG	GG	GG	AA	GG	AG	AG
GG	AA	AG	AG	AG	AA	AG	

47128419

5524	rs10148272	14	AG	AG	GG	AA	AA
AA	AA	AA	AA	AA	AA	AA	AA
AA	AA	AG	AA	AG	AG	AA	AA
AG	AA	AG	AG	AA	AG	AA	AG
--	GG	GG	GG	AA	AG	AG	

72650820

5525	rs10148286	14	AA	AA	AA	AA	AA
AA	AA	AA	AA	AA	AA	AA	AA
AA	AA	AA	AA	AA	AA	AG	AA
AA	AA	AG	AG	AA	AA	AA	AA
AA	AA	AA	AA	AA	AA	AA	

54252529

5526	rs10148313	14	AA	AA	AA	AA	AA
AA	AA	AA	AA	AA	AA	AA	AA
AA	AA	AA	AA	AA	AA	AA	AA
AA	AA	AG	AG	AA	AA	AA	AA
AA	AA	AG	AG	AA	AA	AA	

33885682
5527 rs10148359 14 CC CC CC CC CC
CC CC CC CC CC AC AC AC
CC AC AA AC AC AC AA AC
AC AC CC CC CC AC AC CC
AC CC CC CC CC CC CC
20829966
5528 rs10148371 14 GG GG GG GG GG
GG GG GG GG GG GG GG GG
GG GG GG GG GG GG GG GG
GG GG GG GG GG GG GG GG
GG GG GG GG GG GG GG
55609418
5529 rs10148408 14 AA AA AA AA AA
AA AA AC AC AC AA AA AA
AA AA AA AA AA AA AA AA
AA AA AA AA AA AA AA AA
AA AA AA AA AA AA AA
22795126
5530 rs10148455 14 AA AA AA AA AA
AA AA AA AA AA AA AA AA
AA AA AA AA AA AA AA AA
AA AA AA AA AA AA AA AA
AA AA AA AA AA AA AA
105777094
5531 rs10148482 14 AA AA AG AA AA
AA AA AA AA AA AG AG AA
AA AA AA AA AA AG AG AG
AA AA AA AA AA AA AA AA
AA GG AA AG AG AG AG
35878431
5532 rs1014851 7 AA AA AA AG AG
AG GG AA AG AA AA AA AA
AG AG GG GG GG AA AA AG
AG GG AG AG AA AG AG AG
AA GG AA AG GG AG AG
155980396
5533 rs10148581 14 AG AG AG AG AA
AA AG AG AA AG AG AG AA
AA AG AA AA AG AG AA AA
AG AG AA AA AA AA AA AA
AG AG AG AG AA AA AA
44338004
5534 rs10148628 14 AA AG AG AG AA
AG AA AA AG AA AG AA AG
AG AA AG AA AA AG AG AA
AA AA AA AA AA AG AA AG
AA AA AG AG AA AA AA
47702350
5535 rs10148669 14 AA AA AA AA AA

AA AA AA AA AA AA AA AA
AA AA AA AA AA AA AA AA
AA AA AA AA AA AA AA AA
AA AA AA AA AA AA AA
96584980
5536 rs10148684 14 AA AA AA AC AC
AA AC AA AA AA AC AC CC
CC AC AC CC CC AC AC CC
AA CC AC AC AA AA AA AA
AC AA AA AA AA AC AC
60131361
5537 rs10148748 14 AA AG AA AG AA
AG AA AA AA AA AA AA AA
AA AA AG AG AG AA AA AA
AA AA AA AA AA AA AA AA
AA AA AA AA AA AG AA
66553785
5538 rs10148762 14 GG GG GG GG AG
GG AG GG GG GG GG GG GG
GG GG GG GG AG GG GG GG
GG GG GG GG GG GG GG GG
GG GG GG GG GG GG GG
39765378
5539 rs10148795 14 AA AA AA AG AA
AA AG AA AG AA AA AA AA
AA AG AG AA AG AA AA AG
GG GG GG GG AA AG AG AA
AA AA AG AG GG GG AG
33845322
5540 rs10148862 14 AG AG GG AG AG
AG AG AG AG AG AA AG AG
AG AA GG GG AG AA AG AG
GG AG AG AG AA GG AG AG
AA AA GG AG AG GG AA
34711456
5541 rs10148890 14 GG GG GG GG GG
GG GG GG GG GG GG GG GG
GG GG GG GG GG GG GG GG
GG GG GG GG GG GG GG GG
GG GG GG GG GG GG GG
105805055
5542 rs10148925 14 AA AA AA AA AA
AA AA AA AG AA AA AA AA
AA AA AA AA AA AA AA AA
AA AA AA AA AA AA AA AA
AA AA AA AA AA AA AA
60087910
5543 rs10148938 14 AA AA AA AA AA
AA AA AA AA AA AC AA AA
AA AA AA AA AA AA AA AA

AA AA AA AA AA AA AA AA
AA AC AA AA AC AA AC
105493605
5544 rs10148947 14 GG AG AA AA GG
AG AG AG AA AG AA AG AA
AA AG AG AG AG GG AG AG
GG AG GG GG GG AG AG GG
AG AG AG AG AG GG AG
85615564
5545 rs10148969 14 AG AA AG AG AG
AG AG AG AG AG GG AA AG
AA AG AG AA AA AG GG AG
AG AA AG AA AG AG GG AA
AG AG GG AG AG AG AA
90968374
5546 rs10148970 14 AA AG AG AA AG
AG AA AA GG AG AG AA AG
AG AG AA AG AG AA AG AA
AA AA AA AA AG AG AG AG
AA AA AG AA AG AG GG
103892000
5547 rs10148975 14 AC AC AA AC AC
AC AC AA AA AA AA AA AA
AC AC AA AA AA AA AC AA
AA AC AA AA AC AC AC CC
AA AA AA AA AC CC AC
67292937
5548 rs10149100 14 AA AA AA AA AA
AA AA AA AA AA AA AA AA
AA AA AA AA AA AA AA AA
AA AA AA AA AA AA AA AA
AA AA AA AA AA AA AA
92248315
5549 rs10149105 14 GG GG AG GG GG
GG GG GG GG GG AA GG AG
GG GG AG AA AA GG GG GG
GG AG GG GG GG AG AG GG
GG GG AG GG GG GG GG
85615940
5550 rs1014911 X AA AG AG GG GG
GG GG GG GG GG GG GG GG
GG GG GG GG GG GG GG GG
GG GG GG GG GG GG GG GG
GG GG GG GG GG GG GG
146524544
5551 rs10149162 14 AA AA AA AA AG
AG AA AA AA AA GG AG AG
AG AG AA AG AA AA AA AA
AG AA AA AA AG AA AA AA
AA AA AA AA AG AG AA

51821239							
5552	rs10149197	14	AG	AA	AA	AG	AA
	AA	AG	AG	AA	AG	AG	AG	AG
	AG	AG	AA	AA	AA	AG	AA	AA
	AA	AA	AA	AA	AA	AA	AA	AA
	AA	AG	AG	AG	AG	GG	AA	
32641377							
5553	rs10149279	14	AC	AC	AA	AA	AA
	AA	AA	AC	CC	AC	AC	AA	AA
	AA	AC	CC	AC	AC	AA	AA	AA
	AA	AA	AA	AA	AA	AA	AA	AA
	AA	AC	AA	AC	AA	AA	AA	
80579214							
5554	rs10149339	14	AA	AA	AA	AA	AA
	AA	AA	AA	AA	AA	AA	AA	AA
	AA	AA	AA	AA	AA	AA	AA	AA
	AA	AA	AA	AA	AA	AG	AG	AA
	AA	AA	AA	AA	AA	AA	AA	
23780222							
5555	rs1014936	13	CC	CC	CC	CC	AA
	AC	AC	AC	CC	AC	AA	AA	AC
	AA	AA	AC	AA	AA	AC	AC	AC
	AC	CC	AC	AC	AC	AC	CC	AC
	AA	AA	AC	AA	AC	AC	AC	
61281243							
5556	rs10149382	14	AC	AC	CC	AC	AA
	AC	AA	AA	AA	AA	AA	AC	AC
	AC	AA	AA	AA	AA	AA	AA	AA
	AC	AA	AA	AA	AC	AC	CC	AA
	AA	AA	AA	AA	AA	AA	AA	
95712417							
5557	rs10149384	14	CC	CC	CC	CC	CC
	CC	CC	CC	CC	CC	CC	CC	CC
	CC	CC	CC	CC	CC	CC	CC	AC
	CC	CC	CC	CC	CC	CC	CC	CC
	CC	CC	CC	CC	AC	CC	AC	
61846130							
5558	rs10149385	14	AC	CC	CC	AC	CC
	CC	AC	AC	AC	AC	AC	AA	AC
	AC	CC	AC	AC	AC	AC	AC	AC
	AC	CC	AC	AC	CC	AC	AC	AC
	CC	AC	CC	AC	CC	CC	CC	
79985997							
5559	rs10149388	14	GG	GG	GG	GG	GG
	GG	GG	AG	GG	AG	GG	GG	GG
	AG	AG	GG	GG	GG	GG	GG	GG
	GG	GG	GG	GG	AG	GG	GG	AG
	AG	GG	GG	GG	GG	GG	GG	
94466834							
5560	rs10149419	14	AA	AA	AA	AA	AA

	AA	AA	AA	AA	AA	AA	AA	AA
	AA	AA	AA	AA	AA	AA	AA	AA
	AA	AA	AA	AA	AA	AA	AA	AA
	AA	AA	AA	AA	AA	AA	AA	
66720945								
5561	rs1014942		12	AC	CC	CC	CC	AC
	AC	CC	AA	AC	AC	CC	AC	AC
	AA	AC	AC	AC	CC	AC	AC	AC
	AC	AC	AA	AC	AC	CC	CC	CC
	CC	AC	AC	AA	AC	AA	AC	
114326583								
5562	rs10149439		14	GG	GG	AG	AA	AA
	AA	AA	AA	AA	AA	AA	AA	AA
	AA	GG	AA	AA	AA	AA	AG	AA
	GG	AG	AG	AG	AA	AG	AA	AG
	AG	GG	AA	AG	AA	AG	AA	
51585637								
5563	rs10149496		14	AA	AG	AA	AG	AA
	AA	AG	AA	AG	AA	AA	AA	AA
	AA	AA	AA	AG	AA	AG	AG	AG
	AA	AA	AA	AA	AA	AA	AA	AA
	AG	AG	AA	AA	AA	AA	AA	
70309775								
5564	rs10149498		14	AA	AA	AA	AA	AA
	AA	AA	AA	AA	AA	AA	AA	AA
	AA	AA	AA	AA	AA	AA	AA	AA
	AA	AA	AA	AA	AA	AA	AA	AA
	AA	AA	AA	AA	AA	AG	AA	
83624717								
5565	rs10149555		14	AG	AG	AG	AG	GG
	AG	GG	AG	AA	AG	GG	GG	GG
	AA	AA	AG	GG	AG	AG	AG	AG
	AG	GG	AG	AG	GG	GG	GG	GG
	AG	AG	AG	AG	AG	AG	GG	
66277657								
5566	rs10149564		14	GG	GG	GG	GG	GG
	GG	GG	GG	GG	GG	GG	GG	GG
	GG	GG	GG	GG	GG	GG	GG	GG
	GG	GG	GG	GG	GG	GG	GG	GG
	GG	GG	GG	GG	GG	GG	GG	
101013990								
5567	rs10149590		14	AA	AA	AA	AG	AA
	AG	AG	AG	AG	AG	GG	AG	GG
	AG	GG	AG	AG	AG	GG	AG	AG
	AG	GG	GG	AA	AG	GG	AG	GG
	AG	AG	GG	AG	AG	GG	AG	
24194104								
5568	rs10149608		14	GG	GG	GG	AG	AG
	AA	GG	GG	GG	GG	GG	GG	GG
	GG	GG	GG	GG	GG	AG	AG	AG

AA AG AG AG AG GG AG GG
AG GG AG GG GG GG GG
104843010
5569 rs10149652 14 CC CC CC CC CC
CC CC CC CC CC CC CC CC
CC CC CC CC CC CC CC CC
CC CC CC CC CC CC CC CC
CC CC CC CC CC CC CC
61096475
5570 rs10149659 14 GG GG AG AG AG
GG AA AG AG AG GG GG GG
GG GG GG GG AG AG -- GG
GG GG -- GG AA GG AG AG
GG AG GG GG AG AG GG
28190767
5571 rs1014971 22 AA AG AA AG AA
AG AA GG AA GG AG AG AA
AA AA GG GG GG AG AG AA
AA AA AA AG AG AG GG AG
AA AG GG GG AA AA AA
39332623
5572 rs10149717 14 GG AG AG AA AG
AA AG AA AA AA AG AG AA
AG AG GG AG AG AA AA AA
AA AA AA AA AG AG AG AG
AG AG AG AA AG AA GG
94913559
5573 rs10149734 14 AC AA AC AC AA
AC AA AC AA AA AA AA AA
AA AA AC AC AC AC AA AC
AA AA AA AA AC AA AA AC
AC AC AC AC AA AA AA
104424583
5574 rs10149787 14 AC AA AA AC AA
AC AA AA AC AA AC AC AA
AC AC AC AA AC AC AA CC
CC CC CC CC AC AA AC AC
CC AA CC AC AC AA CC
74925452
5575 rs10149796 14 CC CC AC CC AA
AC AC CC CC CC CC CC CC
CC CC CC CC AC CC CC CC
CC CC CC CC AC AC AC AC
CC AA AC AC CC CC CC
34273570
5576 rs10149808 14 AG AG AG GG GG
GG GG GG GG GG GG GG GG
GG GG GG GG GG GG GG GG
AG GG AG AG GG GG GG GG
GG GG AG GG GG GG GG

97420338
5577 rs10149831 14 GG AG AG AG AA
AG AA AG GG AG GG GG GG
GG GG GG GG AG AG GG AG
AA AG GG GG AG AG AG AG
GG GG GG GG AG AG AA
42187916
5578 rs1014984 6 AA AG AG GG GG
GG AG GG AG GG GG AG GG
GG GG AG AG AA AG AG AG
GG GG GG GG AG GG AG GG
GG GG AG AG AG GG AA
16670279
5579 rs10149867 14 AA AA AA AG AA
AG AA AA AA AA GG AG AG
AG AG AA AA AA AG GG AG
AG AG AG AG AA AA AA AA
AA AG AA AA GG AG GG
51393929
5580 rs1014988
1 -- -- -- -- -- -- -- --
-- -- -- -- -- -- -- --
-- -- -- -- -- -- -- --
-- -- -- -- -- -- -- --
-- -- -- -- 1680219
5581 rs10149896 14 GG AG AG GG AG
AG GG AG GG AG AG AG GG
GG AG AG AG AG GG AG GG
AA AA AA AA AG GG GG AG
AG GG GG GG AG AA GG
39850261
5582 rs10149902 14 GG GG GG GG GG
GG GG GG GG GG GG GG GG
GG GG GG GG GG GG GG GG
GG GG GG GG GG GG GG GG
GG GG AG GG GG AG GG
56882742
5583 rs10149930 14 AG GG GG AG GG
GG AG -- GG -- GG GG GG
GG GG AG AG -- GG GG GG
GG GG GG GG AG GG AG GG
GG AG AG AG GG GG GG
32882641
5584 rs10149939 14 AA AG GG GG GG
GG GG GG GG GG GG GG GG
GG GG GG GG GG GG GG GG
GG GG GG GG AG GG GG AG
GG GG AG GG GG AG GG
103345467
5585 rs1014995

21 -- -- -- -- -- -- -- --
-- -- -- -- -- -- -- --
-- -- -- -- -- -- -- --
-- -- -- -- -- -- -- --
-- -- -- -- 39747504
5586 rs10150005 14 AA AA AG AG AA
AG AA AG AG AA AA AA AA
AG AG AA AG AA AA GG AA
AA AG AA AG AG AG GG AG
AG GG AA AG GG AG AG
65694256
5587 rs1015004 21 GG GG GG AA AG
AG AG AG AG GG GG GG GG
GG GG GG GG GG GG GG AG
GG GG GG GG AG GG GG AG
AG GG AG AG GG GG GG
27129093
5588 rs10150088 14 AA AG AG AG AA
AA AG AA AA AA AG AG AG
AA AG AG AA AA AA AA AA
AG GG GG GG GG GG GG GG
AA GG AA AG AG AG GG
92503576
5589 rs10150101 14 AG AG AG AA AA
AA AA GG AA GG AA AG AG
AG AA AG AA AA AA AA AA
AA AA AA AA AG GG GG AG
GG AG AA AA AA AA AG
43131317
5590 rs10150134 14 AA AG GG AG AA
AG AA AA AA AA AA AG AG
AG AA AA AA AA AA AG AG
AA AA AA AA AA AG AG AA
GG AG AG AG AA AA AA
85624714
5591 rs10150174 14 AG AA AA GG GG
GG AG AG AA AG AA AA AG
GG AG GG GG AG AG AG AG
AG GG AG GG AG AA AA AA
GG GG AA AG AG AG AG
62585953
5592 rs10150182 14 AA AA AA AA AA
AA AA AA AA AA AA AA AA
AA AA AA AA AA AA AA AA
AA AA AA AA AA AA AA AA
AA AA AA AA AA AA AA
62662990
5593 rs1015020 21 AA AA AA AA AC
AA AC AC AC AA AC AA AC
AC AC AA AA AA AA AA AA

	AC	AC	AA	AA	AA	AA	AA	AA
	AA	AA	AA	AA	AC	AA	AC	
24334028								
5594	rs10150279		14	GG	AG	AA	AG	AA
	AG	AG	GG	AG	GG	AG	GG	AG
	AG	AG	AG	AA	AA	GG	GG	AG
	AG	GG	GG	GG	AG	AG	AA	GG
	AG	AG	AG	AA	AA	AG	AG	
100333150								
5595	rs10150307		14	AA	AA	AC	AA	AA
	AA	AA	AA	AA	AA	AA	AA	AA
	AA	AA	AA	AA	AA	AA	AA	AC
	AC	AC	AC	AC	AA	AA	AA	AA
	AC	AC	AA	AA	AA	AA	AA	
58889815								
5596	rs10150309		14	GG	GG	GG	GG	GG
	GG	GG	GG	GG	GG	GG	GG	GG
	GG	GG	GG	GG	GG	GG	GG	GG
	GG	GG	GG	GG	GG	GG	GG	GG
	GG	GG	GG	GG	GG	GG	GG	
23119368								
5597	rs10150317		14	GG	GG	GG	GG	GG
	GG	GG	GG	GG	GG	GG	GG	GG
	GG	GG	GG	AG	GG	AA	GG	AG
	GG	GG	GG	GG	GG	GG	GG	GG
	AG	GG	GG	GG	GG	GG	GG	
40353935								
5598	rs10150369		14	AG	AG	AG	AG	AG
	GG	AA	AG	AA	AG	AA	AG	AG
	AG	AA	AA	AA	AG	GG	AA	AG
	AG	AG	GG	GG	AA	GG	AG	AG
	AG	AA	AA	AA	AG	GG	AG	
54803765								
5599	rs10150398		14	GG	GG	GG	GG	GG
	GG	GG	GG	GG	GG	GG	AG	AG
	AG	GG	GG	GG	GG	GG	GG	GG
	GG	GG	GG	GG	GG	GG	GG	GG
	GG	GG	GG	GG	AG	AG	GG	
95232683								
5600	rs10150455		14	--	--	--	--	AG
	AA	AG	--	--	--	AA	AA	--
	--	--	--	--	--	--	--	--
	--	--	--	--	--	--	--	--
	AG	--	--	--	AG	--	AG	
41791748								
5601	rs10150567		14	AA	AA	AA	AA	AA
	AA	AA	AA	AA	AA	AA	AA	AA
	AA	AA	AA	AA	AA	AA	AA	AA
	AA	AA	AA	AA	AA	AA	AA	AA
	AA	AA	AA	AA	AA	AA	AA	

92315241							
5602	rs10150574	14	AG	GG	GG	GG	AA
	AG	AG	GG	GG	GG	GG	GG	GG
	GG	GG	AG	GG	AG	GG	AG	GG
	GG	GG	GG	GG	AG	GG	AG	GG
	GG	GG	GG	GG	AG	AG	GG	
32812532							
5603	rs10150580	14	AA	AA	AG	AA	AA
	AA	AA	AA	AA	AA	AA	AA	AA
	AG	AG	AA	AG	AG	AA	AA	AA
	AA	AG	AA	AG	AA	AA	AA	AA
	AA	AA	AA	AA	AA	AA	AA	
63830354							
5604	rs10150603	14	AA	AG	AG	AG	AG
	AA	GG	GG	AG	GG	AG	AA	AG
	AA	AA	GG	GG	GG	AG	AG	AA
	AA	GG	AG	AG	AA	GG	AG	AG
	AA	AG	AG	AG	GG	GG	AG	
92956184							
5605	rs10150681	14	CC	CC	CC	CC	CC
	CC	CC	CC	CC	CC	CC	CC	CC
	CC	CC	CC	CC	CC	CC	CC	CC
	CC	CC	CC	CC	CC	CC	CC	CC
	CC	CC	CC	CC	CC	CC	CC	
105297232							
5606	rs1015070	21	CC	AC	CC	CC	CC
	AC	CC	CC	CC	AC	CC	CC	CC
	CC	CC	CC	CC	CC	CC	CC	CC
	AA	CC	CC	CC	CC	CC	CC	CC
	AA	CC	AC	CC	CC	CC	AC	
40029361							
5607	rs10150708	14	CC	CC	CC	CC	CC
	CC	CC	CC	CC	CC	CC	CC	CC
	CC	CC	CC	CC	CC	CC	CC	CC
	CC	CC	CC	CC	CC	CC	CC	CC
	CC	CC	CC	CC	CC	CC	CC	
64895774							
5608	rs10150709	14	AG	AG	AG	AG	AA
	AG	AA	AG	AA	AG	AA	AA	AA
	AA	AG	AA	AA	AA	AG	AG	AA
	AA	AA	AA	AA	GG	AA	AG	AG
	AA	AA	AA	AA	AA	AA	AG	
39440106							
5609	rs10150720	14	AG	GG	AG	AG	GG
	GG	AG	GG	GG	GG	GG	AG	AG
	AG	GG	AG	AG	GG	AG	AG	GG
	GG	GG	GG	GG	GG	AG	AG	GG
	GG	GG	GG	GG	GG	GG	AG	
83311943							
5610	rs10150723	14	GG	GG	GG	GG	AG

	AG	GG	AG	AG	AG	GG	GG	GG
	GG	GG	AG	AG	GG	GG	GG	AG
	GG	AG	AA	AA	GG	GG	GG	GG
	AG	AG	AG	AG	GG	AG	AG	
47928236								
5611	rs10150761		14	AA	AA	AA	AA	AC
	AC	AA	AA	AA	AA	AA	AA	AA
	AA	AA	AA	AA	AA	AA	AA	AA
	AA	AA	AA	AA	AA	AA	AA	AA
	AA	AC	AA	AA	AA	AA	AA	
33942839								
5612	rs1015077		6	AA	AA	AA	AG	AG
	AG	AA	AA	AA	AA	AA	AG	AG
	AG	AG	AA	AA	AA	AG	AG	AG
	AA	AA	AA	AA	AA	AA	AA	AA
	AA	AA	AA	AA	AA	AA	AA	
167526506								
5613	rs10150771		14	GG	GG	GG	GG	AA
	AG	AG	GG	GG	GG	GG	GG	GG
	AG	AG	AG	AG	GG	GG	GG	GG
	GG	GG	GG	GG	AA	GG	AG	AG
	GG	AA	AG	AA	AG	AG	GG	
57675185								
5614	rs10150823		14	GG	AG	AG	AA	AA
	AA	AA	AA	AG	AA	AG	GG	AG
	AG	AG	AA	AG	AA	AG	AG	AA
	AA	AG	AG	AG	AA	AG	AG	AA
	AA	GG	AA	AG	AG	AA	GG	
50051751								
5615	rs10150830		14	GG	GG	GG	GG	GG
	GG	GG	GG	GG	GG	GG	GG	GG
	GG	GG	GG	GG	GG	GG	AG	GG
	GG	GG	GG	GG	AG	AG	GG	AG
	AG	GG	GG	GG	GG	GG	GG	
54624542								
5616	rs10150849		14	GG	GG	GG	GG	GG
	GG	GG	AG	GG	AG	GG	GG	GG
	GG	GG	AG	AG	AG	GG	AG	GG
	GG	AA	AG	AG	GG	AG	GG	AG
	AG	AG	GG	GG	AG	AA	AG	
60211444								
5617	rs10150889		14	AA	AG	AA	AG	GG
	AG	GG	AG	AA	AG	AG	AG	GG
	GG	GG	AG	AG	AG	AG	AG	AG
	AG	GG	GG	GG	GG	AA	AG	AG
	GG	AG	GG	AG	AG	AG	GG	
48277276								
5618	rs10150936		14	AC	AC	AC	AC	AA
	AC	AA	CC	AC	CC	CC	CC	CC
	AA	AA	CC	CC	AC	CC	CC	CC

	CC	AC	CC	AC	CC	CC	CC	CC
	CC	AC	CC	CC	AA	AC	AC	
71162467								
5619	rs10150977		14	AA	AA	AA	AA	AA
	AA	AA	AA	AA	AA	AA	AA	AA
	AA	AA	AA	AA	AA	AA	AA	AA
	AA	AA	AA	AA	AA	AA	AA	AA
	AA	AA	AA	AA	AA	AA	AA	
93572813								
5620	rs10151114		14	AA	AA	AA	AG	GG
	AG	AG	AG	AG	AA	AG	GG	AG
	AG	AG	AG	GG	AG	AG	AG	AG
	AG	AG	AA	AA	AG	AG	GG	AA
	AA	AA	AG	AA	AA	AA	AA	
22179595								
5621	rs10151120		14	AA	AA	AA	AA	AA
	AA	AA	AA	AA	AA	AA	AA	AA
	AA	AA	AA	AA	AA	AA	AA	AC
	AA	AC	AC	AA	AA	AA	AA	AA
	AC	AA	AA	AA	AA	AA	AA	
21060287								
5622	rs10151131		14	AA	AA	AA	AA	AA
	AA	AA	AA	AA	AA	AA	AA	AA
	AA	AA	AA	AA	AA	AA	AA	AA
	AA	AA	AA	AA	AA	AA	AA	AA
	AA	AA	AA	AA	AA	AA	AA	
67724778								
5623	rs1015123		7	GG	GG	GG	GG	AG
	AG	GG	GG	AG	AG	GG	GG	GG
	GG	GG	GG	GG	GG	GG	GG	AG
	GG	AG	GG	AG	GG	GG	GG	GG
	AG	AG	GG	AG	GG	AG	AG	
4651184								
5624	rs1015126		3	AA	AA	AA	AA	AA
	AA	AA	AA	AA	AA	AA	AA	AA
	AA	AA	AA	AA	AA	AA	AA	AA
	AA	AA	AA	AA	AA	AA	AA	AA
	AA	AA	AA	AA	AA	AA	AA	
89364451								
5625	rs10151267							
14	--	--	--	--	--	--	--	--
	--	--	--	--	--	--	--	--
	--	--	--	--	--	--	--	--
	--	--	--	--	--	--	--	--
	--	--	--	--	63061920			
5626	rs10151272		14	CC	CC	CC	CC	CC
	CC	CC	CC	CC	CC	CC	CC	CC
	AC	CC	CC	CC	CC	CC	CC	CC
	CC	CC	CC	CC	CC	CC	CC	CC
	CC	CC	CC	CC	AC	AC	CC	

24464590								
5627	rs10151339		14	AC	AC	CC	CC	CC
	CC	CC	AC	AC	AC	AC	AC	AC
	AA	AC	AC	AA	AA	AC	AA	AC
	AA	AA	AA	AA	AA	AC	AA	AC
	AA	AC	CC	CC	CC	CC	CC	
60813416								
5628	rs10151365		14	AA	AA	AA	AA	AA
	AA	AA	AA	AA	AA	AG	AA	AG
	AG	AG	AA	AA	AA	AA	AA	AG
	AA	AA	AA	AA	AA	AA	AA	AA
	AA	AA	GG	AG	AA	AG	AG	
51767408								
5629	rs10151380		14	AG	GG	AG	AG	AA
	AG	AA	AA	AA	AA	AA	AA	AA
	AG	GG	AG	AA	AA	AA	AA	AA
	AA	AA	AA	AA	AG	AA	AA	AG
	AA	AA	AG	AG	AA	AA	AA	
88494613								
5630	rs10151399		14	AA	AC	AA	AC	CC
	CC	AC	AA	AA	AA	AA	AA	AA
	AA	AA	AA	AA	AA	AC	AA	AC
	AA	AC	CC	CC	AA	AA	AA	AA
	AA	AA	AA	AA	AA	AA	AA	
44471675								
5631	rs1015140		1	AA	AA	AA	AA	AA
	AA	AA	AA	AA	AA	AA	AA	AA
	AA	AA	AA	AG	AG	AA	AA	AA
	AA	AA	AA	AA	AG	AG	AG	AG
	AA	AA	AA	AA	AA	AA	AA	
200278759								
5632	rs10151407		14	AA	AA	AA	AA	AA
	AA	AA	AA	AA	AA	AG	AA	AG
	AG	AA	AA	AA	AA	AA	AA	AG
	AA	AA	AA	AA	AG	AG	AG	AG
	GG	AA	AA	AA	AG	AA	AG	
43867179								
5633	rs10151427		14	GG	AG	GG	AG	GG
	AG	GG	GG	GG	GG	GG	GG	GG
	GG	AG	GG	GG	GG	AG	GG	GG
	GG	GG	GG	GG	AG	GG	GG	AG
	--	GG	GG	GG	AG	AA	GG	
33043130								
5634	rs10151454		14	AA	AA	AA	AA	AC
	AA	AC	AA	AA	AA	AA	AA	AA
	AA	AA	AA	AA	AA	AA	AA	AA
	AA	AA	AA	AA	AC	AA	AC	AA
	AC	AA	AA	AA	AC	AA	AC	
41776000								
5635	rs10151487		14	GG	GG	GG	GG	AG

GG AG GG GG GG GG GG GG
GG GG GG GG GG GG AG GG
GG GG GG GG GG GG GG GG
GG GG GG GG GG GG GG
67369566
5636 rs1015149 6 AA AG AG AG AG
AA GG AA AA AG AG AG AG
GG AG AG GG GG AA AA AG
AG AG AG AA GG AG AG AG
GG AG AA AG AG AG AG
41658889
5637 rs10151491 14 GG GG GG GG GG
GG GG GG GG GG GG GG GG
GG GG GG GG GG GG GG GG
GG GG GG GG GG GG GG GG
GG GG GG GG GG GG GG
100263406
5638 rs10151509 14 AA AG AG AG AG
AG AG AA AA AA AG AG AA
AA AA AA AA AA AA AA AA
AA AA AA AA AG AG AA AG
AG AA AA AA GG AG GG
48481000
5639 rs10151525 14 AA AA AA AA AA
AA AA AA AA AA AA AA AA
AA AA AA AA AA AA AA AA
AG AG AA AA AG AG AG GG
AA AA AA AA AA AA AA
58772018
5640 rs10151532 14 AG AG AA GG GG
GG GG GG AG AG AG AG AG
AA AG AA AG AG AG GG AG
AA AA AA AA AG AA AG AA
GG AG GG AG AG GG AG
83271898
5641 rs10151617 14 AA AG GG AA AA
AA AA AA AA AA AA AA AA
AA AA AA AA AA AA AA AA
AA AA AA AA AA AA AA AA
AG AG AA AA AG GG AA
98996159
5642 rs10151656 14 GG GG GG AG AG
AA AG GG GG GG GG GG GG
AG AG GG GG GG AG GG AG
GG AG GG AG GG AG GG AG
AG AG GG GG AG AG AG
65061688
5643 rs10151712 14 AG AA AA AG AG
GG AA AG AA AG AA AA AA
AA AA GG AG AG AA AG AA

	AA	GG	AA	AG	AA	AA	AA	AA
	AA	AA	AG	AA	AA	AG	AG	
78052809								
5644	rs10151764		14	CC	CC	CC	CC	CC
	CC	CC	CC	CC	CC	CC	CC	CC
	CC	CC	CC	CC	CC	CC	CC	CC
	CC	CC	CC	CC	CC	AC	CC	AC
	CC	CC	CC	CC	CC	CC	CC	
21865259								
5645	rs10151904		14	AG	AA	AA	AA	AA
	AA	AA	AA	AA	AA	AA	AA	AA
	AA	AA	AA	AA	AA	AG	AA	AA
	AA	AA	AA	AA	AA	AA	AA	AA
	AA	AA	AA	AA	AA	AA	AA	
40241401								
5646	rs10151927		14	GG	GG	GG	GG	GG
	GG	GG	GG	GG	GG	GG	GG	GG
	GG	GG	GG	GG	GG	GG	GG	GG
	GG	GG	GG	GG	GG	GG	GG	GG
	GG	GG	GG	GG	GG	GG	GG	
62908503								
5647	rs1015195		11	AA	AA	AG	AA	AG
	AG	AA	AA	AG	AG	AG	AG	AA
	AA	AA	AA	AA	AA	AA	AG	AA
	AG	AG	AG	AG	AA	AA	AA	AA
	AG	AA	AG	AA	AG	AA	GG	
56544017								
5648	rs10151974		14	GG	GG	GG	GG	GG
	GG	GG	GG	GG	GG	GG	GG	GG
	GG	GG	GG	GG	GG	GG	GG	GG
	GG	GG	GG	GG	GG	GG	GG	GG
	GG	GG	GG	GG	GG	GG	GG	
104048078								
5649	rs10151986		14	AA	AA	AA	AA	AA
	AA	AA	AA	AA	AA	AA	AA	AA
	AA	AA	AA	AA	AA	AA	AA	AA
	AA	AA	AA	AA	AA	AA	AA	AA
	AA	AA	AA	AA	AA	AA	AA	
55297009								
5650	rs1015199		20	AA	AA	AA	AA	AA
	AA	AA	AA	AA	AA	AA	AA	AA
	AA	AA	AA	AA	AA	AA	AA	AA
	AG	AA	AG	AA	AA	AG	AA	AA
	AA	AA	AA	AA	AA	AA	AA	
59535190								
5651	rs10152049		14	AG	AG	AG	AG	AA
	AA	AG	AG	AG	AG	AG	AG	AA
	AG	AG	AG	GG	AA	GG	AG	AG
	AG	AG	AG	AG	GG	GG	GG	GG
	AA	AG	AG	AG	AG	GG	AG	

78795293
5652 rs10152064 14 AG AA AA AG AA
AA AG AA AG AA AG AG AG
GG AG AG AG AA AA AA AG
AA AG AA AA AA AG AA AA
AA AG AA AA AA AA AG
80856675
5653 rs10152067 14 AA AA AA AA AA
AA AA AG AG AA AG AG AG
AA AA GG AG AG AG AA GG
AG AA AG AG AA AA AA AA
AA AA AG AG AA AA AA
63916963
5654 rs10152081 14 AA AA AA AG AG
GG AA AG AG AG AG AG AA
AA AA GG AG AG AA AG AA
AG AG AG AG GG AA AG AG
AG GG AG AG AA AG AA
95421335
5655 rs10152118 15 AG GG AG GG GG
GG GG AG GG GG AG GG GG
GG GG GG GG GG GG AG GG
GG GG GG GG GG GG GG GG
AG GG GG GG AG GG AG
70128996
5656 rs1015213 8 AG GG GG GG GG
GG GG GG GG GG GG GG GG
AG AG AG GG AG GG GG AG
GG GG GG GG GG GG GG GG
GG GG GG GG GG AG GG
52887541
5657 rs1015215 20 AG AA AG AA AA
AA AA AA AA AA AA AA AA
AG AA AA AG AG AA AA AG
AA AA AA AA AA AG AA AA
AA AA AA AA AA AA AA
59369565
5658 rs10152193 15 GG GG GG GG GG
GG GG AG AA GG GG GG GG
GG GG AG GG AA AG AG AG
AG GG GG GG GG AA AG AG
AG GG GG GG AA AG AG
38129559
5659 rs10152238 15 GG GG GG AG AG
AG GG GG GG GG GG GG GG
GG GG AG AG AG GG GG GG
GG GG GG GG AG GG GG AG
AG AG AG AA AG GG AG
32270318
5660 rs10152251 15 AA AA AA AG AA

	AA	AG	AG	AG	AG	AG	AG	AG
	AG	AG	AA	AA	AA	GG	AG	AG
	GG	AG	AG	AG	AG	AA	AG	AA
	GG	AG	GG	AG	AA	AA	AG	
78679837								
5661	rs10152266		15	GG	GG	GG	GG	GG
	GG	GG	GG	GG	GG	GG	GG	GG
	GG	GG	GG	GG	GG	GG	GG	GG
	GG	GG	GG	GG	GG	GG	GG	GG
	GG	GG	GG	GG	GG	GG	GG	
82450403								
5662	rs10152278		15	AG	GG	AG	AG	AG
	AG	AG	GG	GG	GG	GG	GG	GG
	GG	AG	GG	GG	GG	AG	AG	GG
	AG	AG	AG	AG	AG	GG	AG	AG
	AG	GG	GG	GG	GG	GG	GG	
23086929								
5663	rs10152333		15	AG	AG	AA	AG	GG
	AG	AG	AG	GG	GG	AG	AG	AG
	GG	AG	GG	AG	AG	AG	AG	AG
	AA	GG	GG	AG	AA	GG	AG	AG
	AG	AA	GG	AG	AG	AG	GG	
86854359								
5664	rs10152344		15	CC	CC	CC	CC	CC
	CC	CC	CC	CC	CC	AC	AC	AC
	CC	AC	CC	CC	CC	CC	CC	CC
	CC	CC	CC	CC	CC	AC	CC	CC
	CC	CC	CC	CC	AC	AC	AC	
59696523								
5665	rs1015235		1	AG	GG	AG	GG	GG
	GG	GG	AG	AG	AG	GG	GG	GG
	GG	AG	AG	AG	GG	AG	AG	GG
	GG	GG	GG	GG	GG	GG	GG	GG
	AG	AG	GG	AG	GG	GG	GG	
147043542								
5666	rs10152417		15	GG	GG	GG	AG	AG
	AG	GG	GG	GG	GG	AG	AG	AG
	AG	AG	AG	AA	AA	AG	AG	GG
	AG	AG	AG	AG	GG	AA	AG	AG
	AG	GG	AG	GG	AG	AG	AA	
79333228								
5667	rs10152421		15	GG	GG	GG	GG	AG
	AG	GG	GG	GG	GG	GG	AG	GG
	GG	GG	AG	AG	GG	GG	GG	AG
	AG	AG	AG	AG	AG	GG	AG	AG
	GG	GG	GG	GG	GG	AG	GG	
26985509								
5668	rs10152433		15	AG	AG	AA	GG	GG
	GG	GG	GG	GG	GG	AG	AG	AG
	GG	AG	AA	AG	AG	AG	GG	AG

	AG	AG	AG	AG	AG	GG	AG	AG
	AA	GG	AG	AG	AG	AG	AG	
53222705								
5669	rs10152440		15	CC	CC	CC	CC	CC
	CC	AC	CC	CC	AC	CC	CC	CC
	CC	CC	CC	AC	AC	AC	CC	AA
	AC	AC	AC	CC	AC	CC	CC	AC
	CC	AC	AC	AC	AC	AC	AA	
97644074								
5670	rs10152445		15	GG	GG	GG	GG	GG
	GG	GG	GG	GG	GG	GG	GG	GG
	GG	GG	GG	GG	GG	GG	GG	GG
	GG	GG	GG	GG	GG	GG	GG	GG
	GG	GG	GG	GG	GG	GG	GG	
80816685								
5671	rs10152458		15	AC	AC	CC	AC	AC
	CC	AA	CC	CC	AC	AC	AC	AC
	AA	AC	AC	AC	AC	CC	CC	CC
	AA	AA	AA	CC	CC	AC	CC	AC
	AC	AC	AA	AC	AC	AC	AC	
101890811								
5672	rs10152478		15	GG	GG	GG	GG	GG
	GG	GG	GG	GG	GG	GG	GG	GG
	GG	GG	GG	GG	GG	GG	GG	GG
	GG	GG	GG	GG	GG	GG	GG	GG
	GG	GG	GG	GG	GG	GG	GG	
38887698								
5673	rs10152507		15	GG	GG	GG	GG	GG
	GG	GG	GG	GG	GG	GG	GG	GG
	GG	GG	GG	GG	GG	GG	GG	GG
	GG	GG	GG	GG	GG	GG	GG	GG
	GG	GG	GG	GG	GG	GG	AG	
27672091								
5674	rs10152510		15	AA	AA	AA	AC	AA
	AC	AA	AA	AA	AA	AA	AC	AA
	AC	AC	AA	AC	AA	AC	CC	AA
	AA	AA	AA	AA	AA	AA	AA	AA
	AA	AA	AA	AA	AA	AA	AA	
100389079								
5675	rs10152574		15	AA	AA	AA	AA	AA
	AA	AA	AA	AA	AA	AA	AA	AA
	AA	AA	AA	AA	AA	AA	AA	AA
	AA	AA	AA	AA	AA	AA	AA	AA
	AA	AA	AA	AA	AG	AG	AA	
50660128								
5676	rs10152580							
15	--	--	--	--	--	--	--	--
	--	--	--	--	--	--	--	--
	--	--	--	--	--	--	--	--
	--	--	--	--	--	--	--	--

	--	--	--	--	23221689			
5677	rs1015260		17	AG	AA	AG	AG	GG
	AG	GG	GG	GG	GG	AG	GG	GG
	GG	GG	GG	GG	GG	AG	AG	AG
	GG	AG	AG	GG	AG	GG	GG	GG
	AG	AG	GG	AG	GG	GG	GG	
48116785								
5678	rs10152674		15	GG	GG	GG	AA	AA
	AA	AG	GG	AG	GG	GG	AG	AG
	AG	AG	AG	AG	AG	GG	GG	GG
	AG	GG	GG	GG	AA	AG	AA	AG
	AA	AA	GG	AG	AG	AG	AG	
34198118								
5679	rs10152696		15	AA	AA	AA	AA	AA
	AA	AA	AA	AA	AA	AA	AA	AA
	AA	AA	AA	AA	AA	AA	AA	AA
	AA	AA	AA	AA	AA	AA	AA	AA
	AA	AA	AA	AA	AA	AA	AA	
38357162								
5680	rs10152718		15	AG	--	AG	AG	AG
	AG	AG	AA	AA	AA	AG	AA	AG
	AG	AG	--	AG	AG	AA	AG	AA
	AA	AA	AA	AA	AA	AG	AA	AA
	AG	AG	AA	AG	AA	AG	AA	
38483564								
5681	rs10152719		15	GG	AG	AG	GG	GG
	GG	GG	AG	GG	AG	GG	GG	GG
	GG	GG	AG	AG	AG	AG	AG	AG
	AG	AG	AG	GG	AG	AG	AG	GG
	GG	AG	AG	AG	GG	GG	GG	
61114657								
5682	rs10152807		15	GG	GG	GG	GG	GG
	GG	GG	GG	GG	GG	GG	GG	GG
	GG	GG	GG	GG	GG	GG	GG	GG
	GG	GG	GG	GG	GG	GG	GG	GG
	GG	GG	GG	GG	GG	GG	GG	
68913743								
5683	rs10152812		15	AG	AG	AA	GG	GG
	GG	GG	AG	GG	GG	GG	GG	GG
	GG	GG	AG	AG	AG	AG	AG	AG
	AG	AG	AG	AG	AG	GG	AG	GG
	GG	AA	GG	AG	GG	GG	GG	
95362466								
5684	rs1015287		12	AG	AG	GG	AG	AG
	AG	GG	GG	GG	GG	GG	AG	GG
	AG	GG	GG	GG	GG	GG	AG	AG
	AA	AA	AA	AA	GG	AG	GG	GG
	AG	AG	GG	GG	GG	AG	GG	
2605386								
5685	rs1015291		12	GG	GG	GG	AA	AG

AG AG GG AA AA AG AG AA
GG AG AA AG AG GG AG GG
GG GG GG GG AA AA AA AA
AG AG AA AG AG AG AA
19984779
5686 rs10152968 15 AG AA AG AA AA
AA AA AA AA AA AG AA AG
AG AG AA AA AA AA AA AA
AG AA AA AA AG AG AG GG
AA AG AA AA AA AA AA
60604436
5687 rs10152981 15 AG GG AG GG GG
GG GG AG GG AA AA AG AG
GG AG AG AA AG GG GG GG
AG AA AA AA AG AG AA GG
AG AA GG AG AG AG GG
27508143
5688 rs10153006 15 AA AA AA AA AA
AA AG AA AA AA AA AA AA
AA AA AG AG AA AA AA AA
AA AA AG AA AG AA AG AG
AA AA AA AA AA AA AA
46019664
5689 rs10153042 15 AG AG AA AA AA
AG AA AG AG GG AG AG AG
GG AG GG AG GG AG AA AA
AG AG AG AG AG AG AG AG
AG AG AA AG AA AA AA
33879608
5690 rs10153083 16 AA AA AA AA AA
AA AA AA AA AA AA AA AA
AA AA AA AA AA AA AA AA
AA AA AA AA AA AA AA AA
AA AA AA AA AA AA AA
563922
5691 rs10153114 16 AA AG AA AG GG
GG AG GG AG GG AA AG AG
AG AG AG AG GG AG GG GG
AG GG GG AG AG AG AG GG
AA AG AG AA GG GG AG
10412872
5692 rs10153128 16 AA AA AA AA AA
AA AA AA AA AA AC AC AA
AA AA AA AA AA AA AA AA
AA AA AA AA AA AA AA AA
AA AA AC AC AC AA CC
81656818
5693 rs10153131 16 AG AA AG AA AA
AA AA AG GG AG AA AA AA
AA AA AA AA AA AA AA AA

AG AA AA AG AG AA AA AA
AG AG AG AG AA AA AA
6552545
5694 rs10153160 16 AA AA AA AA AA
AA AA AA AA AA AA AA AA
AA AA AA AA AA AA AA AA
AA AA AA AA AA AA AA AA
AA AA AA AA AA AA AA
15173005
5695 rs10153199 16 AA AG AA AA AA
AA AA AA AA AA AA AG AG
AG AG AG AA AA AG AA AA
AA AA AA AA AG AA AA AA
AG AA AA AA AA AA AA
860673
5696 rs10153210 16 AA AA AA AA AA
AA AA AA AA AA AA AA AA
AA AA AA AA AA AA AA AA
AG AG AG AA AA AA AA AA
AA AA AA AA AA AA AG
89967308
5697 rs10153233 16 AA AA AA AA AA
AA AA AG AA AA AA AA AA
AA AA AA AA AA AA AA AA
AA AG AA AA AG AA AG AG
AG AA AA AA AG AA AG
56039886
5698 rs1015330 6 AG AG AG GG GG
GG GG GG GG GG GG GG AG
GG AG GG GG GG AG AG AG
GG GG GG GG AG AG AG AG
GG GG GG GG GG GG GG
36786072
5699 rs10153314 17 AG GG AG GG AG
AG AG AG AG AG AA AA AA
AA AA AG AG GG AG GG AA
AG AG AG AG AG AG AA AG
AG AG AG GG AA AG AG
71132676
5700 rs10153317 17 GG AG AG GG GG
GG GG GG GG GG GG AG GG
AG AG GG GG GG GG GG GG
GG GG GG GG GG GG GG GG
AG AG GG GG GG GG GG
13325226
5701 rs10153321 18 GG GG GG GG GG
GG AG GG GG GG AG GG AG
GG AG GG GG GG GG GG AG
GG GG GG GG GG AG GG AG
GG AG GG GG GG GG GG

3176377
5702 rs10153360 18 AG GG AG AG AA
AA AG GG GG GG GG AA AG
AG AG AG AA AG AG AG AG
AG GG AG AA AG AG AG AG
GG GG AG AG AA AA AA
42766728
5703 rs10153390 18 AC AC AC AC AA
AC AC AC CC CC AA AA AA
AA AA AC AA AC CC AA CC
CC CC CC CC CC AC CC CC
AC AC AC AA CC CC AC
49960237
5704 rs1015341 6 AG AG GG AG GG
GG AG AG AA AG GG GG AG
GG GG AA AA AA AG AG AA
AG AG AG AG GG AG AG GG
GG AG GG GG AG GG AA
144035464
5705 rs10153410 18 AG AG AG GG GG
AG AG AA AG AG AA AA AA
AA AA AG GG GG GG GG AG
AG AA AA AA GG AA AG AG
AG AG AG AG AA AG AG
3173964
5706 rs10153422 18 AG AG AG GG GG
AG AG GG GG GG AA AG AA
AA AG AG AG AA AA AG AA
AA AA AG AG AG AG AG AG
AG GG AG GG GG AG GG
10163628
5707 rs10153433 18 AA AG AG GG AG
GG AG AG GG GG AG GG AG
AG GG AG AA AG GG AG GG
AG AG AG AA GG GG GG GG
GG AG AG AA AG AA AG
50073517
5708 rs10153456 19 GG GG GG GG GG
GG GG GG GG GG GG GG GG
AG AA AG GG GG GG GG GG
GG GG GG GG AG AG AG AA
GG GG AA AG GG GG GG
52349499
5709 rs10153461 19 AA AC AA AA AA
AA AA CC AC AC AC AC AC
AC AC AC AC AA AA AC AA
AA AC AC AA AC AA AC AA
AC CC AC CC AA AC AC
18095251
5710 rs10153463 19 GG GG GG GG GG

	GG	AG	AG	GG	GG	GG	GG	GG
	GG	GG	AG	AG	AG	AG	GG	GG
	GG	AG	AG	GG	GG	GG	GG	GG
	AG	GG	GG	GG	AG	GG	AG	
58021704								
5711	rs10153500		19	AA	AA	AA	AA	AA
	AA	AA	AA	AA	AA	AA	AA	AA
	AG	AG	AA	AA	AA	AA	AA	AA
	AA	AA	AA	AA	AA	AA	AA	AA
	AA	AA	AG	AG	AA	AA	AA	
38073267								
5712	rs10153513		19	GG	GG	GG	GG	GG
	GG	GG	GG	GG	GG	GG	GG	GG
	GG	GG	GG	GG	GG	GG	GG	GG
	GG	GG	GG	GG	GG	GG	GG	GG
	GG	GG	GG	GG	GG	GG	GG	
58819706								
5713	rs10153521		19	AG	AG	AA	GG	GG
	GG	AG	AG	GG	GG	AA	AG	AA
	AA	AA	AG	AG	AG	AG	AA	GG
	GG	AG	AG	GG	AG	GG	GG	AG
	AA	GG	AG	GG	AA	AG	AG	
57983457								
5714	rs10153686							
2	--	--	--	--	--	--	--	--
	--	--	--	--	--	--	--	--
	--	--	--	--	--	--	--	--
	--	--	--	--	--	--	--	--
	--	--	--	--	42306258			
5715	rs10153797		2	GG	GG	GG	GG	GG
	GG	GG	AA	AG	AA	GG	AG	GG
	GG	GG	GG	GG	GG	AG	AG	AA
	GG	GG	GG	GG	GG	GG	GG	GG
	AA	GG	AG	GG	GG	GG	GG	
619653								
5716	rs10153813		2	AG	AG	AA	AG	AA
	AG	AG	GG	GG	GG	GG	AA	AG
	AG	AG	GG	GG	GG	AG	AG	AA
	AA	AA	AA	AA	GG	GG	GG	GG
	AG	AA	AG	AG	GG	GG	AG	
76826142								
5717	rs10153880		2	GG	AG	GG	AG	AG
	AA	GG	AA	AA	AA	AA	AA	AA
	AG	AG	AA	AA	AG	AG	AA	AG
	AG	AA	AA	AG	AG	AA	AA	AG
	AA	AA	GG	AG	AA	AA	AG	
141365171								
5718	rs10153883		2	AA	AA	AA	AG	AA
	AG	AG	AG	AG	AG	AA	AA	AG
	GG	GG	GG	AG	AG	AA	AA	AA

	AG	GG	AG	AG	AG	AA	AG	AA
	AG	AA	AA	AA	AA	AA	AG	
174206181								
5719	rs1015390		12	AG	GG	AG	GG	GG
	GG	GG	AG	GG	AG	AG	GG	AG
	GG	GG	GG	GG	GG	GG	GG	GG
	GG	GG	GG	GG	GG	GG	GG	GG
	GG	GG	GG	GG	GG	GG	GG	
48344038								
5720	rs10153955		2	AG	AG	AG	AA	AA
	AA	AA	AA	AA	AA	AA	--	AG
	AG	AG	AA	AA	AA	AA	AA	AA
	AA	AA	AA	AA	AG	AA	AG	AA
	--	--	AA	AA	--	AA	--	
101264880								
5721	rs10154012		20	AG	AG	AA	AG	GG
	AG	GG	GG	GG	AG	GG	GG	GG
	GG	GG	AG	AG	AA	AG	AG	AA
	AG	AG	AG	AG	AG	AG	AG	AG
	GG	AG	GG	AG	AG	AG	GG	
36290136								
5722	rs1015419		1	GG	GG	GG	GG	GG
	GG	GG	GG	AA	GG	GG	GG	GG
	AG	GG	GG	GG	GG	GG	AA	GG
	AG	AG	AA	AG	AG	GG	AG	GG
	GG	GG	AG	GG	GG	AG	GG	
176767452								
5723	rs10154196		21	GG	GG	GG	AG	AG
	GG	AG	GG	GG	GG	GG	GG	GG
	GG	GG	GG	GG	GG	GG	GG	GG
	GG	GG	GG	GG	GG	GG	GG	GG
	GG	GG	AG	AG	GG	AG	GG	
43024004								
5724	rs10154234		21	GG	AG	GG	AG	AG
	GG	AA	GG	AG	GG	GG	AG	AG
	GG	AG	AA	AG	AG	GG	AG	GG
	GG	GG	GG	GG	AG	AA	AA	AG
	GG	GG	AG	AG	GG	GG	GG	
42525007								
5725	rs1015438		16	GG	GG	GG	AG	AG
	AA	AG	AA	AA	AG	GG	GG	GG
	GG	GG	AG	GG	AG	AG	AG	GG
	AA	AG	AA	AG	AG	AG	AG	AA
	GG	GG	GG	GG	GG	GG	AG	
51177517								
5726	rs1015445		11	AG	AG	AG	AA	AG
	AA	AG	AA	AA	AA	AA	AA	AA
	AG	AG	AG	AG	AA	AG	AG	AG
	GG	GG	AG	AG	AA	AA	AA	AA
	GG	AG	GG	GG	GG	AG	AG	

17868673
5727 rs1015455 6 CC CC CC CC CC
CC CC CC CC CC AC AC CC
AC CC CC CC CC CC CC CC
CC CC CC CC AC CC CC CC
AC AC AC AC CC CC AC
93005995
5728 rs10154700 22 CC CC CC CC CC
CC CC AC CC AC CC CC AC
AC AA AA AC AC AC AC AC
CC CC CC CC CC CC CC CC
AC CC CC CC AC AC CC
42365073
5729 rs1015471 22 GG GG GG GG AG
AG GG AA AA AG GG GG GG
GG GG AA AA AA GG GG GG
AA AA AA GG GG GG GG GG
AG AA GG AG AG AG AG
19878514
5730 rs1015477 9 CC CC CC AA CC
AC AC AA CC AC AC CC CC
CC AA AC AA AC AC AA AA
AC CC AC CC CC AA AC AC
CC AC AC AC CC CC AC
90225414
5731 rs10154867 3 AA AA AA AA AA
AA AA AA AA AA AA AA AA
AA AA AA AA AA AA AA AA
AA AA AA AA AC AA AA AA
AA AC AA AC AA AA AA
137307649
5732 rs10154880 3 AG AG AG AA AG
AG AG AG AA AA AA AA AA
AG AA AA AA AA AA AA AG
GG GG AG GG AA AA AA AA
AA AG AA AA AG AG GG
157451200
5733 rs10155052 3 AC AA AA AC AC
AC AA AA AA AA CC CC AC
AC CC AC AC AC AC AA AC
AC AA AC AA AA AA AA AA
CC AC AC AC AA AA AC
183335081
5734 rs10155062 4 GG GG GG AG AG
AG AG GG GG GG GG AG GG
GG AG AG AG AA GG GG GG
GG GG GG GG GG AA AG AG
GG GG GG GG AG GG AG
7133905
5735 rs10155152 4 AC AA AA AA AA

	AA	AA	AC	AA	AC	AA	AA	AA
	AA	AC	AC	AC	CC	AC	AC	AC
	AA	AA	AA	AA	AA	AA	AA	AA
	AC	AA	AA	AA	AC	AA	AC	
14359888								
5736	rs10155292		4	GG	GG	AG	GG	GG
	GG	GG	GG	GG	GG	GG	GG	AG
	GG	GG	GG	GG	GG	AG	AG	GG
	GG	GG	GG	GG	AG	GG	GG	AG
	GG	GG	GG	GG	GG	GG	GG	
58277976								
5737	rs10155296		4	AA	AG	GG	GG	GG
	GG	GG	GG	AG	GG	AG	AG	GG
	GG	AG	GG	GG	GG	GG	GG	GG
	GG	AG	GG	AG	GG	AG	AG	GG
	GG	GG	GG	GG	GG	GG	AG	
15175539								
5738	rs1015534		X	GG	GG	GG	GG	GG
	GG	GG	AG	AG	GG	GG	GG	GG
	GG	GG	GG	GG	GG	GG	GG	GG
	GG	GG	GG	GG	GG	GG	GG	GG
	GG	GG	GG	GG	GG	GG	GG	
11207896								
5739	rs10155359		4	CC	CC	CC	CC	CC
	CC	CC	CC	CC	CC	CC	CC	CC
	CC	CC	CC	CC	CC	CC	CC	CC
	CC	CC	CC	CC	CC	CC	CC	CC
	CC	CC	CC	CC	CC	CC	CC	
108676210								
5740	rs10155441		4	GG	GG	GG	GG	GG
	GG	GG	GG	GG	GG	GG	GG	GG
	GG	GG	GG	GG	GG	GG	GG	GG
	GG	GG	GG	GG	GG	GG	GG	GG
	GG	GG	GG	GG	GG	GG	GG	
56262802								
5741	rs1015547		14	GG	AG	AG	AA	AA
	AA	AA	GG	GG	GG	AA	AA	AA
	AA	AA	GG	AG	AG	AA	AA	AA
	AA	AA	AA	AA	AA	AA	AA	AA
	GG	AA	AG	AG	AA	AA	AA	
57450468								
5742	rs10155470		4	AA	AA	AA	AG	AA
	AG	AG	AA	AG	AG	AA	AA	AA
	AA	AA	AA	AA	AG	AG	AG	AG
	AA	AA	AA	AA	AA	GG	AG	AG
	AG	AA	AA	AA	AG	AA	GG	
186882290								
5743	rs10155508		5	AA	AA	AA	AA	AA
	AA	AA	AA	AA	AA	AA	AA	AA
	AA	AA	AA	AA	AA	AA	AA	AA

	AA	AA	AA	AA	AA	AA	AA	AA
	AA	AA	AA	AA	AA	AA	AA	
136194623								
5744	rs10155516		5	AG	GG	AG	AG	AG
	AG	AG	AG	AG	AG	AA	AA	AA
	AA	AA	AG	GG	AG	AA	AA	AG
	GG	GG	AG	AG	AA	AG	AA	AG
	AG	AA	AA	AA	GG	GG	AG	
141865255								
5745	rs1015562		17	AG	AG	AG	GG	AG
	AG	AG	GG	AG	GG	AA	GG	AG
	AG	AG	GG	GG	GG	AG	AG	GG
	GG	AG	AG	GG	AG	AA	AG	AG
	AG	AG	AG	AA	AG	AA	GG	
48483283								
5746	rs1015568		6	AA	AA	AA	AG	GG
	AG	GG	AG	AG	AG	AA	AG	AA
	AA	AA	AA	AA	AA	AA	AA	AA
	AG	AG	AG	AG	AG	GG	AG	GG
	AG	AA	AG	AA	AG	AG	AG	
16432284								
5747	rs10155686		6	AA	AA	AA	GG	GG
	GG	GG	GG	GG	GG	GG	AG	GG
	AG	AG	AA	AA	AG	AG	GG	AG
	GG	GG	GG	GG	AG	GG	GG	GG
	GG	AG	AG	AG	AG	AG	AG	
162254146								
5748	rs10155709		6	AA	AA	AA	AA	AA
	AA	AA	AA	AA	AA	AA	AA	AA
	AA	AA	AA	AA	AA	AA	AA	AA
	AG	AG	AA	AG	AA	AA	AA	AA
	AA	AA	AA	AA	AA	AG	AA	
151619282								
5749	rs10155760		6	GG	GG	GG	GG	GG
	GG	GG	GG	GG	GG	GG	GG	GG
	GG	GG	GG	GG	GG	GG	GG	GG
	GG	GG	GG	GG	GG	GG	GG	GG
	GG	GG	GG	GG	GG	GG	GG	
99901608								
5750	rs10155761		6	AA	AA	AA	AA	AG
	AG	AA	AA	AA	AA	AA	AA	AA
	AA	AA	AA	AA	AA	AG	AG	AA
	AG	AG	AA	AG	AA	AA	AA	AA
	AA	AA	AA	AA	AA	AA	AA	
114733016								
5751	rs10155813		7	GG	AG	AG	GG	GG
	GG	GG	AG	AA	AA	AA	AG	AG
	AG	AG	AA	AG	AG	AG	AG	AA
	AG	AG	AG	AG	AA	GG	AG	AG
	GG	AG	AG	GG	AG	GG	AG	

153623251
5752 rs10155834 7 AG AG GG AG AG
GG AA AG AA AG AA AA AA
GG AG AG GG AG AG AG GG
AG AA AG AA GG AA AG AG
AA AG GG AG AA AG AG
27372021
5753 rs10155847 7 AA AA AA AA AA
AA AA AA AA AA AA AA AA
AA AA AA AA AA AA AA AA
AA AA AA AA AA AA AA AA
AA AA AA AA AA AA AA
95405690
5754 rs1015588 5 AA AA AA GG AG
AG GG AA AA AA AA AA AA
AA AA AA AA AA AA AA AA
AG AG AG AG AG AA AG AG
AA AA AA AA AG AG AG
9831983
5755 rs10155889 7 AA AA AA AA AA
AA AA AA AA AA AA AA AA
AA AA AA AA AA AA AA AA
AA AA AA AA AA AA AA AA
AA AA AA AA AA AA AA
3808359
5756 rs10155894 7 GG GG GG GG GG
GG GG GG GG AG AG AA AG
GG GG AG GG GG GG GG GG
AA AA AG AG GG GG GG GG
GG GG GG GG AG AG GG
76027576
5757 rs10155935 7 AC AC AC CC CC
CC CC CC CC CC CC CC CC
CC CC AC AC AC AC CC AA
AA AC CC AC CC CC CC CC
CC AC CC CC CC CC CC
67661167
5758 rs10155953 7 -- -- AG AA AG
AG AA AG AG AA AA AA AA
AA -- -- AG GG AA AG AA
AA AA AA AA AG AG AG --
AA -- AG -- AA AG AA
64267756
5759 rs10155966 7 AA AA AA AA GG
AG AG AA AA AA AG AA AG
GG AA AG AA AA AA AA AA
AA AA AA AA AA AA AA AA
AA AG AG AG AA AA AG
98981233
5760 rs10155975 7 GG AG GG AG GG

AG GG GG GG GG GG GG GG
GG GG AG GG GG AG AG AG
GG AA AG AA GG GG GG GG
GG GG GG GG GG GG AG
13493700
5761 rs10156067 7 AA AA AG AA AG
AA AG AA AA AG AA AA AA
AA AA AA AG AA AG AA AG
AG GG AG AA AA AA AA AA
AA AA AA AA GG AG AG
51158612
5762 rs10156082 7 -- AG -- AG AG
-- GG AG AG AG GG GG AG
AA AA AG GG GG AG AG AA
AG AG AA GG AG -- AG AG
-- AG GG -- AG GG --
35654476
5763 rs10156134 7 AG AA AA AA AA
AA AA AA GG GG AA AA AA
AA AG AA AA AA AA AA AA
AA AA AA AA AG AA AG AG
AG AA AA AA AA AA AA
152255494
5764 rs10156165 7 AA AA AA AA AA
AA AA AA AA AA AA AA AA
AA AA AA AA AA AA AA AA
AA AA AA AA AA AA AA AA
AA AA AA AA AA AA AA
54207048
5765 rs10156225 8 GG GG AG AA AG
AG AA GG GG GG GG GG GG
AG AG GG AG AG GG AG GG
AG AA AG AA AA AG AG AG
AA AG GG AG GG AG GG
117732100
5766 rs10156227 8 GG GG GG GG GG
GG GG GG GG GG GG GG GG
GG GG GG GG GG GG GG GG
GG GG GG GG GG GG GG GG
GG GG GG GG GG GG GG
76573860
5767 rs10156270 8 CC CC CC AC AC
CC AA CC CC CC AC AC CC
CC CC CC CC CC AC AC CC
CC CC CC CC CC CC CC CC
CC CC CC CC CC CC CC
115308011
5768 rs10156288 8 CC CC CC CC CC
CC CC CC CC CC CC CC CC
CC CC AC CC CC CC CC CC

CC	CC	CC	CC	CC	CC	CC	CC
CC	CC	CC	CC	CC	CC	CC
136193650
5769	rs1015629	7	AG	GG	AG	AA	AG
AG	AA	AG	AG	GG	AA	AA	AA
AA	GG	AG	AG	AG	AA	AA	AA
GG	AG	AG	GG	GG	GG	GG	GG
AA	AG	AG	GG	GG	GG	GG
83846895
5770	rs10156297	8	AA	AA	AA	AC	AC
AA	AC	AA	AA	AA	AA	AA	AA
AA	AA	AA	AA	AA	AA	AA	AA
AA	AC	AA	AC	AC	AA	AC	AA
AC	AC	AA	AA	AA	AC	AC
127551826
5771	rs10156332	8	GG	AG	GG	AA	AA
AG	AA	AG	GG	GG	GG	AG	AG
AG	AG	GG	GG	GG	AG	AG	GG
AG	GG	GG	GG	AA	AA	AA	AA
AG	AG	AG	AG	AG	AG	AG
92799912
5772	rs10156375
8	--	--	--	--	--	--	--	--
--	--	--	--	--	--	--	--
--	--	--	--	--	--	--	--
--	--	--	--	--	--	--	--
--	--	--	--	64302442
5773	rs10156395	9	AA	AA	AA	AA	AA
AA	AA	AA	AA	AA	AA	AA	AA
AA	AA	AA	AA	AA	AA	AA	AA
AA	AA	AA	AA	AA	AA	AA	AA
AA	AA	AA	AA	AA	AA	AA
102813042
5774	rs10156409	9	GG	GG	GG	AG	AG
GG	AA	AG	AG	GG	AA	AA	AA
AA	AG	AA	AA	AA	AG	AG	AG
AA	AA	AA	AA	AA	AG	AA	AA
AA	AG	AA	AA	GG	AG	AG
30995741
5775	rs10156438	9	AA	AA	AA	AA	AG
AA	AG	AA	AA	AA	AA	AA	AA
AA	AA	AG	AA	AG	AA	AA	AA
AA	AA	AA	AA	AA	AA	AA	AA
AA	AG	AA	AG	AG	AA	GG
82796396
5776	rs10156573	9	GG	GG	GG	GG	GG
GG	GG	GG	GG	GG	GG	GG	GG
GG	GG	GG	GG	GG	GG	GG	GG
GG	GG	GG	GG	GG	GG	GG	GG
GG	GG	GG	GG	GG	GG	GG

139590097
5777 rs1015663 17 GG GG GG GG GG
GG GG GG GG GG GG GG GG
GG GG GG GG GG AG AG GG
AG GG GG GG GG GG GG GG
AG GG GG GG GG GG GG
14238087
5778 rs10156653 9 GG GG GG AG GG
AG GG GG GG GG AG GG GG
GG GG GG GG GG GG AG GG
GG GG GG GG GG GG GG GG
GG GG GG GG GG GG GG
75586627
5779 rs10156664 9 AG AG AG AA AA
AA AA AG AG AG AA AA AA
AA AA AG GG GG AA AA AA
AG AG AG AG AA AA AA AA
AG AG AG AG AG AG AG
79700008
5780 rs1015667 13 AA AG AA AA AA
AA AA GG GG GG AA AA AA
AA AA GG GG GG AG AG AA
AA AG GG AG GG GG GG GG
AG GG AG AG AG AG AA
76739424
5781 rs1015669 5 AG AG AA GG GG
GG AG GG AG GG AG GG GG
GG GG GG GG GG GG GG GG
GG GG GG GG GG GG GG GG
AG GG GG GG GG GG GG
15750881
5782 rs1015673 17 AG AG AA AG AG
AA AG GG GG GG AG AA AG
GG AG AG AG AG GG GG AG
GG AG AG AG AG AA AG AG
AG AA AG AG AG AG AG
34477386
5783 rs1015675 19 GG GG AG AA AA
AA AG AG GG GG GG GG GG
GG GG GG GG GG GG AA GG
AG AA GG GG GG AG GG GG
GG GG AG AG AG GG AG
5336389
5784 rs10156915 X GG GG GG GG GG
GG GG GG GG GG GG GG GG
GG GG GG GG GG GG GG GG
GG GG GG GG GG GG GG GG
GG GG GG GG GG GG GG
48432463
5785 rs10156930 X GG GG GG GG GG

	GG	GG	GG	GG	GG	GG	GG	GG
	GG	GG	GG	GG	GG	GG	GG	GG
	GG	GG	GG	GG	GG	GG	GG	GG
	GG	GG	GG	GG	GG	GG	GG	
106168019								
5786	rs1015695		2	GG	GG	GG	AA	GG
	AG	AG	GG	GG	GG	GG	GG	GG
	AA	AA	AG	AG	AG	AG	AG	AA
	AA	AA	AG	AA	GG	AA	AG	AG
	GG	AG	AA	AG	AG	GG	AA	
36959885								
5787	rs10156956		X	CC	CC	CC	CC	CC
	CC	CC	CC	CC	CC	CC	CC	CC
	CC	CC	CC	CC	CC	CC	CC	CC
	CC	CC	CC	CC	CC	CC	CC	CC
	CC	CC	CC	CC	CC	CC	CC	
3627768								
5788	rs1015696		2	AA	AA	AA	AA	AA
	AA	AA	AA	AA	AA	AA	AA	AA
	AA	AA	AA	AA	AA	AG	AA	AG
	AA	AA	AA	AA	AA	AG	AG	AA
	AA	AG	AG	AG	AG	AA	AG	
36959860								
5789	rs10156978		X	AA	AA	AA	AA	AA
	AA	AA	AG	AG	AA	AA	AA	AA
	AA	AG	AG	AG	GG	AA	AA	AA
	GG	GG	AA	GG	AA	AA	AA	AA
	AG	AG	GG	AA	AA	AA	AA	
11295971								
5790	rs10156988		X	AA	AA	AA	AA	AA
	AA	AA	AA	AA	AA	AA	AA	AA
	AA	AA	AA	AA	AA	AA	AA	AA
	AA	AA	AA	AA	AA	AA	AA	AA
	AA	AA	AA	AA	AA	AA	AA	
146924877								
5791	rs10157024		1	GG	GG	GG	GG	GG
	GG	GG	GG	GG	GG	GG	GG	GG
	GG	GG	GG	GG	GG	GG	GG	GG
	GG	GG	GG	GG	GG	GG	GG	GG
	GG	GG	GG	GG	GG	GG	AG	
38231069								
5792	rs10157078		1	CC	CC	CC	CC	CC
	CC	CC	CC	CC	CC	CC	CC	CC
	CC	CC	AC	AA	AA	AC	AC	AC
	CC	CC	AC	CC	AC	CC	AC	CC
	CC	CC	CC	CC	AA	AC	AC	
42891550								
5793	rs10157084		1	AG	GG	AG	GG	GG
	GG	GG	GG	AG	AG	GG	GG	GG
	GG	GG	GG	GG	GG	AG	GG	AG

GG GG GG GG AA AG AA AA
AG GG GG GG GG GG GG
46820975
5794 rs10157087 1 AG GG AG GG GG
GG GG GG GG GG GG GG GG
GG GG AG AG GG GG GG GG
GG GG GG GG GG GG GG GG
GG GG GG GG GG GG GG
104697678
5795 rs10157138 1 GG GG GG GG GG
GG GG GG GG GG GG GG GG
GG GG GG GG GG GG GG GG
GG GG GG GG GG GG GG GG
GG GG GG GG GG GG GG
151849195
5796 rs10157140 1 AG AG AA GG GG
GG GG AG AG AA GG AG AG
AG AG AA AA AA GG AG AG
AG AG AG GG AG AG GG AA
GG AA AG AA AA AA AA
1527083
5797 rs10157258 1 CC CC CC CC CC
CC CC CC CC CC CC CC CC
CC CC CC CC CC CC CC CC
CC CC CC CC CC CC CC CC
CC CC CC CC CC CC CC
221663814
5798 rs10157373 1 GG GG GG GG GG
GG GG GG GG GG GG GG GG
GG GG GG GG GG GG GG GG
GG GG GG GG GG GG GG GG
GG GG GG GG GG GG GG
153582852
5799 rs1015739 2 AG AG AG GG GG
AG GG AG AG AG GG AG GG
GG GG AG AG AG GG AG AG
GG GG GG GG AG AG AA AG
GG AA GG AG AA AG AA
140791757
5800 rs10157434 1 AA AA AA AA AA
AA AA AA AA AA AA AA AA
AA AA AA AA AA AA AA AA
AA AA AA AA AA AA AA AA
AA AA AA AA AA AA AA
150211153
5801 rs10157450 1 GG GG GG GG GG
GG GG GG GG GG GG GG GG
GG GG GG GG GG GG GG GG
GG GG GG GG GG GG GG GG
GG GG GG GG GG GG GG

68887272
5802 rs10157480 1 AA AA AA GG AG
AG GG AG AG AG AG AG AG
GG AG GG AG AG AG AG AG
GG GG GG GG AA GG AG AG
GG AA AG AG GG GG GG
177873333
5803 rs10157519 1 GG GG GG GG AA
AG AG AA AA AA AG AG AG
GG AG GG AG AG AG AA GG
AG GG AG GG AG AG AA AG
AA AA AG AG GG AG GG
172485007
5804 rs10157535 1 AA AA AA AA AA
AA AA AA AA AA AA AA AA
AA AA AA AA AA AA AA AA
AA AA AA AA AA AA AA AA
AA AA AA AA AA AA AA
145755813
5805 rs10157587 1 CC CC CC CC CC
CC CC CC CC CC CC CC CC
CC CC AC CC CC CC CC CC
CC AC CC AC AC CC AC CC
CC AC AC AA CC CC CC
37473994
5806 rs1015759 17 AA AG AG AG AG
AA AG AA GG AG AG AA AG
AG GG AG AG AG AA AA AA
AG AG AG AG AG AA AA AA
AG AG GG AG AA AA AG
49532237
5807 rs10157622 1 AA AG AA GG GG
GG GG GG AG AG GG GG GG
AG GG AG AG AG AG AG AG
AA AG AA AG AA GG AG AG
AG GG GG GG GG GG GG
68337493
5808 rs10157674 1 AA AA AA AA AA
AA AA AA AA AA AA AA AA
AA AA AA AA AA AA AA AA
AA AA AA AA AA AA AA AA
AA AA AA AA AA AA AA
64463775
5809 rs10157686 1 AC CC AC AC AC
AA CC AA AA AA AA AA AC
AA AA AA AC AC AA AA AA
CC AC AC AC AC AA AA AC
AA AC CC CC AA AA AC
36890010
5810 rs10157709 1 AA AA AA AA AA

AA AA AA AA AA AA AA AA
AA AA AG AA AG AA AA AA
AA AA AA AA AA AG AA AA
AA AA AA AA AA AA AA
249154567
5811 rs10157710 1 AG AG AG AA AA
AA AA AA AA AA AA AA AA
AA AA AA AG AG AG AG AG
AA AG AA AA AA AA AA AA
GG AA AG AA AA AA AG
47961691
5812 rs1015775 22 GG GG AG AG GG
GG AG GG GG GG GG GG GG
GG GG AG AG AG GG GG GG
AG AG GG AA GG GG GG GG
AG GG GG GG AG GG AG
37251477
5813 rs10157778 1 GG GG GG GG GG
GG GG GG AG AG GG GG GG
GG GG GG GG GG GG AG GG
GG GG GG GG GG GG GG GG
AG AG GG GG GG GG GG
9542612
5814 rs10157812 1 AG AG AG AA AG
AG AA AG AG AG AA AA AA
AA AA AG AG AA AG AG AG
AA AA AA AA AA AA AA AA
GG AA GG AG AA AA AA
98883541
5815 rs10157829 1 AA AG AA AA AA
AA AA AG AG AG AA AA AA
AA AA AG AA AA AA AA AG
AG AG AG AG AA AA AA AA
AA AA AG AG AA AA AG
179586314
5816 rs10157835 1 AG AA AG AA AG
AA AG AG GG GG GG GG GG
GG GG AG AG AG AA AA AA
AA AA AA AA AA AA AA AA
AA AA AA AA AG AG AA
4975565
5817 rs10157845 1 GG GG GG GG GG
GG GG GG GG GG GG GG GG
GG GG GG GG GG GG GG GG
GG GG GG GG GG GG GG GG
GG GG GG GG GG GG GG
48813496
5818 rs1015787 19 GG GG GG GG GG
GG GG GG GG GG GG GG GG
GG GG GG GG GG GG GG GG

GG GG GG GG GG GG GG GG
GG GG GG GG GG GG GG
28233191
5819 rs10157919 1 CC CC CC AC CC
AC CC CC CC CC AC AC CC
CC CC CC CC CC CC CC CC
CC CC CC CC AC CC AC CC
CC CC CC CC CC CC CC
181171768
5820 rs10157927 1 CC CC CC AC AC
CC AA AC CC AC AC AC CC
CC CC CC CC CC CC AC CC
CC CC CC CC CC CC CC CC
CC AA CC AC AC CC AC
10974329
5821 rs10158007 1 AA AG AG AG AA
AA AG AA AG AA GG GG AG
AG AG AG GG GG AG AA AG
GG AG GG AG AA GG AG AG
AG AA GG AG AG GG AA
14575256
5822 rs1015801 4 GG GG GG AG AG
GG AG GG GG GG GG GG GG
GG GG GG GG GG GG GG GG
GG GG GG GG GG GG GG GG
GG GG AG GG AG AG GG
54461548
5823 rs10158029 1 -- AG -- -- AG
AG -- AG -- AG AG AG AA
AG AG -- AG AG -- AG AG
AG AG -- AG AG -- AG AG
AG AG AG -- -- -- --
219482935
5824 rs10158153
1 -- -- -- -- -- -- -- --
-- -- -- -- -- -- -- --
-- -- -- -- -- -- -- --
-- -- -- -- -- -- -- --
-- -- -- -- 106979036
5825 rs10158210 1 AG AA AG AA AG
AG AA AG GG AG GG GG GG
AG AG AA AG AA GG GG AG
AA AA AA AA AA AA AA AA
AG AA AG AA AG AA AG
158311985
5826 rs10158231 1 AG GG AG AG AG
AG GG GG GG GG GG GG GG
GG GG GG AG AG AA AG AA
AG AG AG AG AG GG AG GG
GG AA AG AG GG GG AG

45035621							
5827	rs10158245	1	AC	AA	AC	AA	AC
	AC	AA	CC	CC	CC	AC	CC	AC
	AC	CC	CC	CC	CC	AA	AC	AC
	AC	CC	CC	CC	AC	AC	AC	CC
	CC	CC	AC	CC	CC	CC	AC
243833397							
5828	rs10158279	1	AC	AA	AC	AC	AC
	AA	CC	AC	AC	AC	AC	CC	AC
	AC	AA	AC	AC	AA	AC	AC	AC
	AC	AA	AC	AA	AC	AC	AA	AC
	AC	AA	AC	AC	AC	AC	AC
66033696							
5829	rs10158333	1	AA	AA	AA	AA	AG
	AA	AG	AA	AA	AA	AG	AG	GG
	AG	GG	AA	AA	AA	AG	AG	GG
	AA	AA	AA	AA	AG	GG	GG	AG
	AA	AA	AA	AA	AG	AG	AG
85939476							
5830	rs10158384	1	GG	GG	GG	GG	GG
	GG	GG	GG	GG	GG	GG	GG	GG
	GG	GG	GG	GG	GG	GG	GG	GG
	GG	GG	GG	GG	GG	GG	GG	GG
	GG	GG	AG	GG	GG	GG	GG
41225520							
5831	rs10158410	1	CC	CC	CC	CC	AC
	AC	CC	CC	CC	CC	CC	CC	CC
	CC	CC	AA	AC	AC	CC	CC	CC
	CC	CC	CC	CC	CC	AC	CC	AC
	CC	CC	AC	AC	CC	CC	CC
198150496							
5832	rs10158464	1	AA	AA	AA	AA	AA
	AA	AA	AA	AA	AA	AA	AA	AA
	AA	AA	AA	AA	AA	AA	AA	AA
	AA	AA	AA	AA	AA	AA	AA	AA
	AA	AA	AA	AA	AA	AA	AA
33347605							
5833	rs10158471	1	AG	AG	AA	AG	AG
	AG	AG	AG	GG	AG	GG	GG	GG
	AG	AG	AG	AG	AG	AG	AA	GG
	AG	AG	AA	AG	AG	GG	GG	GG
	AA	AG	AA	AA	GG	GG	GG
217455903							
5834	rs10158522	1	AA	AA	AA	AA	AA
	AA	AA	AA	AA	AA	AA	AA	AA
	AG	AG	AA	AA	AA	AG	AA	AA
	AA	AA	AA	AA	AA	AA	AA	AA
	AA	AA	AA	AA	AA	AA	AA
177938712							
5835	rs10158541	1	AA	AA	AA	AA	AA

```
        AA      AA      AA      AA      AA      AA      AA      AA
        AA      AA      AA      AA      AA      AA      AA      AA
        AA      AA      AA      AA      AA      AA      AA      AA
        AA      AA      AA      AA      AA      AA      AA
55582671
5836    rs1015855       5       AA      AA      AG      --      AG
        AG      AG      AA      --      AA      AG      AG      AA
        AG      AG      AA      AG      AG      AG      AA      AA
        AG      AG      AG      AG      AG      AA      AA      AA
        AA      AG      AG      AG      AA      AA      AG
9716549
5837    rs10158555      1       AA      AA      AA      AA      AA
        AG      AA      AA      AA      AA      AA      AA      AA
        AA      AA      AA      AA      AA      AA      AA      AA
        AA      AA      AA      AA      AA      AA      AA      AA
        AA      AA      AG      AA      AA      AA      AA
79729175
5838    rs10158605      1       AA      AA      AA      AG      AG
        AG      AA      AA      AG      AG      AA      AA      AA
        AA      AA      AG      AG      AG      AG      AG      AA
        AA      AA      AA      AA      AA      AG      AG      AG
        AA      AA      AA      AA      AG      AA      AG
48859681
5839    rs10158671      1       GG      GG      GG      AG      AG
        AG      GG      AG      AG      AG      AA      AA      AG
        AG      GG      AG      GG      AG      GG      GG      GG
        GG      GG      GG      GG      AG      AG      GG      AG
        AG      GG      GG      GG      GG      GG      GG
84120354
5840    rs10158700      1       GG      GG      GG      GG      GG
        GG      GG      GG      GG      GG      GG      GG      GG
        GG      GG      GG      GG      GG      GG      GG      GG
        GG      GG      GG      GG      GG      GG      GG      GG
        GG      GG      GG      GG      GG      GG      GG
220677200
5841    rs10158763      1       AA      AA      AA      AA      AA
        AA      AA      AA      AA      AA      AA      AA      AA
        AA      AA      AA      AA      AA      AA      AA      AA
        AA      AA      AA      AA      GG      AA      AG      AG
        AA      AA      AA      AA      GG      AG      GG
30731138
5842    rs10158777      1       AA      AA      AA      AA      AA
        AA      AA      AA      AA      AA      AA      AA      AA
        AA      AA      AA      AA      AA      AA      AA      AA
        AA      AA      AA      AA      AA      AA      AA      AA
        AA      AA      AA      AA      AA      AA      AA
100742399
5843    rs1015878       13      AA      AG      AA      GG      AG
        AG      AG      GG      AG      GG      AG      AA      AG
        AG      AG      GG      GG      GG      GG      AG      AG
```

GG AG GG GG AG AG AA AG
GG GG GG GG AA AA AG
50736491
5844 rs10158783 1 GG GG GG GG GG
GG GG GG GG GG GG GG GG
GG GG GG GG GG GG GG GG
GG GG GG GG GG GG GG GG
GG GG GG GG GG GG AG
4263519
5845 rs10158850 1 GG GG GG GG GG
GG GG GG GG GG GG GG GG
GG GG GG GG GG GG GG GG
GG GG GG GG GG GG GG GG
GG GG GG GG GG GG GG
160808668
5846 rs1015896
11 -- -- -- -- -- -- -- --
-- -- -- -- -- -- -- --
-- -- -- -- -- -- -- --
-- -- -- -- -- -- -- --
-- -- -- -- 17883386
5847 rs10159003 1 GG GG GG GG GG
GG GG GG GG GG GG AG AG
AG GG GG GG GG GG GG GG
GG GG GG GG GG GG GG GG
GG GG GG GG GG GG GG
215373684
5848 rs1015905 8 AG GG AG GG AG
GG AG GG GG GG GG GG GG
GG GG AG AG GG GG GG GG
GG GG GG GG GG AG GG GG
AG GG GG GG GG GG AG
62690015
5849 rs10159070 1 AG GG AG AG AG
AG GG AG AG AG AA AA AA
AG AA AA AG AA AG GG GG
GG GG GG GG AG AG AG AA
GG GG AG GG GG AG GG
89685357
5850 rs10159088 1 GG GG GG GG --
GG GG GG GG GG GG GG GG
GG GG GG GG GG GG GG GG
GG GG GG GG GG GG GG GG
GG GG GG GG GG GG GG
63385069
5851 rs10159099 1 AA AA AA AA AA
AA AA AA AA AA AA AA AA
AA AA AA AA AA AA AA AA
AA AA AA AA AA AA AA AA
AG AA AA AA AG AA AG

56800736
5852 rs10159165 1 CC CC CC CC CC
CC CC CC CC CC AC AC --
CC CC AC AA AA AC AC AC
CC CC AC -- AC CC AC CC
CC CC CC CC AA AC AC
42989379
5853 rs10159187 1 AA AA AA AA AA
AA AA AA AA AA AA AA AA
AA AA AA AA AA AA AA AA
AA AA AA AA AA AA AA AA
AA AA AA AA AA AA AA
1731756
5854 rs10159232 1 CC CC CC CC CC
CC CC AC AC AC CC CC CC
CC CC CC CC CC CC CC CC
CC CC CC CC CC CC CC CC
CC CC CC CC CC CC CC
88052301
5855 rs1015924 6 AA AG AG AG AG
AG AG GG AG GG GG GG AG
AG AG AG AG AA AG AG GG
AG AG AG AG AG AA AA AA
AG AG AA AG AG GG AA
96357307
5856 rs10159271 1 GG GG GG AG AG
AG AA GG GG GG GG GG GG
GG AG AA AG AG AG AA AG
AA AA AG AA AA GG AG AG
GG AG AG AG AG GG AG
15913121
5857 rs1015928 X CC AC AC AA AA
AA AC AC AC AA AA AA AC
AA AA AC AC AC CC CC CC
CC CC CC AC AA AA AA AA
AA AA AA AA AC AC CC
29361952
5858 rs10159282 1 AG GG AG AG AG
AA GG GG AG GG AG AG AG
AG AG GG AG AG GG AG GG
GG GG GG GG AG GG AG GG
GG AG GG GG GG GG GG
177918821
5859 rs10159416 1 GG GG GG GG GG
GG GG GG GG GG AG GG AG
AG GG GG GG GG GG AG GG
GG AG AG AG AA AG AA AG
GG GG GG GG AG AG AA
235814507
5860 rs10159513 10 GG GG AG GG GG

	GG	GG	AG	AG	GG	GG	AG	AG
	AG	AA	GG	GG	GG	GG	GG	AG
	AA	AA	GG	GG	GG	GG	GG	GG
	GG	GG	AG	AG	AG	AG	GG	
89865548								
5861	rs10159576		10	GG	GG	AG	GG	GG
	GG	GG	GG	GG	GG	GG	GG	GG
	GG	GG	GG	GG	GG	GG	GG	GG
	GG	GG	GG	GG	AG	GG	GG	AG
	GG	GG	GG	GG	GG	GG	AG	
89897044								
5862	rs10159746		10	AG	AA	AG	AG	GG
	GG	AG	GG	GG	GG	AG	AG	AG
	AG	AG	GG	AG	GG	GG	--	AG
	AA	AG	GG	AG	GG	AG	AG	GG
	AA	AG	AA	AG	GG	GG	GG	
133742677								
5863	rs10159814		10	GG	GG	GG	GG	GG
	GG	GG	AG	AG	GG	AG	AG	GG
	GG	GG	GG	GG	GG	GG	GG	GG
	GG	GG	GG	GG	GG	GG	GG	GG
	GG	GG	AG	GG	GG	GG	GG	
12103485								
5864	rs10159886		10	AA	AA	AA	AA	AA
	AA	AA	AG	AG	AA	AA	AA	AA
	AA	AA	AG	GG	AG	AA	AA	AG
	AA	AA	AA	AA	AG	AA	AG	AG
	AA	AG	AA	AA	AG	AG	AA	
86712004								
5865	rs1015990		16	AC	CC	AC	AA	AA
	AA	AA	CC	CC	CC	CC	CC	CC
	CC	CC	AA	AC	AC	CC	AC	CC
	AC	AC	AC	AC	AC	CC	CC	CC
	CC	AC	CC	CC	AA	AC	AA	
8342368								
5866	rs1015997		7	AG	AA	AA	AA	AA
	AA	AA	AA	AA	AA	GG	AG	AG
	AG	GG	AA	AG	AG	AG	GG	GG
	AG	AG	AG	AG	GG	AG	GG	GG
	AA	AG	AG	AG	AA	AA	AG	
152758651								
5867	rs10160031		10	CC	CC	CC	AC	CC
	AC	CC	CC	CC	CC	CC	CC	CC
	CC	CC	CC	CC	CC	CC	CC	CC
	CC	CC	AC	AA	CC	CC	CC	CC
	AC	CC	AA	AC	CC	CC	CC	
11263204								
5868	rs10160050		10	AG	AG	AA	GG	AG
	GG	AG	AG	GG	AG	AG	AG	AG
	AG	AG	AA	AG	AG	GG	GG	GG

	AG	AG	GG	GG	GG	GG	GG	GG
	AG	AG	GG	GG	AA	AG	AG	
2034806								
5869	rs10160111		10	GG	GG	GG	GG	GG
	GG	GG	GG	GG	GG	GG	GG	GG
	GG	GG	GG	GG	GG	GG	GG	GG
	GG	GG	GG	GG	GG	GG	GG	GG
	GG	GG	GG	GG	GG	GG	GG	
44936177								
5870	rs10160121		10	AA	AG	GG	AA	AA
	AA	AA	AG	AA	AA	AG	GG	AG
	AG	AG	AG	AG	AG	AA	AG	AA
	AA	AA	AA	AG	AG	AG	AG	AG
	AA	AG	AG	GG	AG	AG	GG	
57166573								
5871	rs10160238		11	AA	AA	AC	AC	AA
	AC	AA	AA	AA	AA	AA	AA	AA
	AC	AC	CC	AC	AC	AA	AA	AA
	AA	AA	AA	AA	AC	AA	AA	AA
	AA	CC	AA	AC	AA	AA	AA	
29757305								
5872	rs10160262		11	GG	AG	GG	GG	GG
	GG	GG	GG	GG	GG	GG	GG	GG
	GG	GG	GG	AG	AG	GG	AG	GG
	GG	GG	GG	GG	AG	GG	AG	GG
	AG	AG	GG	GG	GG	GG	GG	
132708916								
5873	rs10160397		11	GG	GG	AG	GG	GG
	GG	AG	AA	AA	AA	AA	AA	AA
	AG	AA	AA	AA	AA	GG	GG	GG
	AG	AA	AG	AA	GG	GG	GG	GG
	AA	AG	GG	GG	GG	AG	GG	
61749766								
5874	rs10160437		11	GG	AG	AA	GG	AG
	AG	AG	AA	AA	AA	AA	AA	AA
	AG	AA	AA	AA	AG	AA	AG	AG
	--	AA	--	AA	GG	--	GG	AG
	AG	AG	AG	AA	AA	AG	AG	
46181739								
5875	rs10160548		11	AC	AC	AA	AC	AC
	CC	AA	AA	AC	AC	AA	AC	AC
	AA	AC	AA	AA	AA	AA	AA	AC
	AA	AC	AA	AC	AA	AA	AA	AA
	AA	AC	AC	AC	AC	AC	AC	
113856681								
5876	rs10160595		11	GG	GG	GG	GG	GG
	GG	GG	GG	GG	GG	GG	GG	GG
	GG	GG	GG	GG	GG	GG	GG	GG
	GG	GG	GG	GG	GG	GG	GG	GG
	GG	GG	GG	GG	GG	GG	GG	

33357625
5877 rs1016062 2 GG GG GG GG GG
AG GG GG GG GG GG GG GG
GG GG GG GG AG GG GG GG
GG GG GG AG GG GG GG GG
AG AG GG AG GG GG GG
234894893
5878 rs10160649 11 AA AA AG AA AA
AA AA AA AA AA AG AA AG
AA AA AA AA AA AG AA AA
AA AA AA AA AA AA AA AA
AA AA AG AA AA AA AA
3938385
5879 rs10160702 11 AA AA AA AG AG
AG AG AA AA AA AG AG AG
AA AG AG GG AG AG AG GG
AG AG AG AA AG AG AG AG
AG GG AA AG AA AA AA
126362042
5880 rs10160742 11 GG GG GG GG GG
GG GG GG AG AG GG GG GG
AG GG AG AG AG GG GG GG
GG GG GG GG GG GG GG GG
AG GG GG GG AG AA GG
115239397
5881 rs10160797 11 GG GG GG GG GG
GG GG GG GG GG GG GG GG
GG GG GG GG GG GG GG GG
GG GG GG GG GG GG GG GG
GG GG GG GG GG GG GG
57284715
5882 rs1016083 6 AA AA AA AA AA
AA AA AA AA AA AG AG AA
AG AG AA AA AA AA AA AA
AA AA AA AA AA AA AA AA
AA AG AA AG AA AA AA
118249495
5883 rs1016085 6 AA AA AA AA AA
AA AA AG GG AG AG AA AA
AA AA AA AA AA AG AA GG
AG AG AA AA AA AA AA AA
AA AG AA AA AA AG AA
162242404
5884 rs10160857 12 AA AA AA AA AA
AA AA AA AA AA AA AA AA
AA AA AA AA AA AA AA AA
AA AA AA AA AA AA AA AA
AA AA AA AA AA AA AA
114654259
5885 rs1016088 20 AG GG GG GG GG

GG GG AG AG AG GG GG AG
AG AG GG GG GG GG GG GG
GG GG GG GG AG AG GG AG
GG GG GG GG GG GG AG
47004466
5886 rs10160981 12 AG AG AA GG GG
GG GG GG GG GG GG GG GG
GG GG GG AG AG GG GG GG
GG GG GG GG GG GG GG GG
GG GG GG GG GG AG GG
110904075
5887 rs10160990 12 AA AA AA AC AC
AC AC AA AA AC AA AA AA
AA AA AA AA AA AA AA AA
AA AA AA AA AA AA AA AA
AA AA AA AA AC AA CC
39217830
5888 rs10160994 12 AA AA AA AA AG
AA AG AA AA AA AA AA AA
AA AG AA AA AA AA AA AA
AG AG AG AG AA AA AA AA
AG AG AA AA AA AG AA
77526094
5889 rs1016110 22 AA AA AA AA CC
AC AC AC AA AA AC AC AC
AC CC AA AC AC AC AA AC
AC AC AC AC AC AC CC CC
AA AA AC AA AC AA AC
42836337
5890 rs10161112 12 CC CC CC CC AA
AC AC CC AC CC CC CC CC
CC CC CC CC CC AC AC AC
CC CC CC CC CC CC CC CC
AC CC CC CC CC CC CC
130952454
5891 rs10161145 12 CC CC CC CC CC
CC CC CC CC CC CC CC CC
CC CC CC CC CC CC CC CC
CC CC CC CC CC CC CC CC
CC CC CC CC CC CC CC
89345037
5892 rs10161147 12 GG GG GG GG AG
AG AG GG AG AG AG GG GG
GG GG GG GG GG GG GG GG
GG GG GG GG AG AG GG GG
GG AG GG AG AG GG AA
20423727
5893 rs10161149 12 GG GG GG GG GG
GG GG AG AG GG GG GG GG
GG GG GG GG GG GG GG AG

GG GG GG GG GG GG GG GG
GG GG GG GG GG GG GG
78075142
5894 rs10161163 12 AA AA AA AA AA
AA AA AG AG AA AA AA AA
AA AA AA AA AA AA AA AA
AA AA AA AA AA AA AA AA
AA AA AA AA AA AA AA
44433103
5895 rs10161172 12 AA AA AA AA AA
AA AA AA AA AA AA AA AA
AA AA AA AA AA AA AA AA
AA AA AA AA AA AA AA AA
AA AA AA AA AA AA AA
34053818
5896 rs1016122 2 AA AA AA AA AA
AA AA AA AA AA GG AG AG
AG AG AA AA AA AA AA AA
AA AA AA AA AA AG AA AA
AG AA AA AA AA AA AA
14372491
5897 rs10161237 12 AA AA AA AA AA
AA AA AA AA AA AA AA AA
AA AA AA AA AA AA AA AA
AA AA AA AA AA AA AA AA
AA AA AA AA AA AA AA
42091024
5898 rs10161263 12 GG GG GG AG GG
AG GG AG AG AG AG AG AG
GG GG AG AG AG AG GG GG
AG GG AG GG AG GG AG AG
GG GG AG AG GG GG AG
54573343
5899 rs10161275 12 AG GG AG AG AG
AA AG AG AG AG GG AG AG
AG AG GG AG AG AG AA GG
AA AA AA AA AG AG GG AG
AG AA AG AG AG AG AG
2771018
5900 rs10161282 12 GG AG GG AG AG
AG GG AG AG GG GG AG AG
GG AG GG AG AG GG GG GG
GG GG GG GG GG AG AG GG
GG AG GG AG GG GG AG
98344244
5901 rs10161283 12 AA AA AG AA AG
AG AA GG GG GG AG AA AG
AA AG AG AA AG GG AA GG
GG AG AG GG AG AA AG AA
AA AA AA AA AG AG AA

117616150								
5902	rs1016129	9	GG	GG	GG	GG	GG	
	GG	GG	AG	GG	GG	GG	GG	GG
	GG	GG	GG	AG	AG	GG	GG	GG
	AG	AG	AG	AG	GG	AG	GG	GG
	AG	AG	GG	AG	GG	GG	GG	
21122681								
5903	rs10161338	12	AA	AA	AA	AA	AG	
	AG	AA	GG	AG	AG	AG	AG	AA
	AG	AG	AA	AA	AA	AA	AA	AA
	AA	AA	AA	AA	AA	AA	AA	AA
	AA	AG	AA	AA	AA	AA	AG	
115865303								
5904	rs10161383	12	AG	AG	AG	GG	GG	
	GG	GG	GG	GG	GG	GG	GG	GG
	GG	GG	GG	GG	GG	GG	GG	GG
	GG	GG	GG	GG	GG	GG	GG	GG
	GG	GG	GG	GG	GG	GG	GG	
111916281								
5905	rs10161436	12	GG	AG	AG	AA	AG	
	AG	AA	AG	AG	AG	AG	AA	AA
	AA	AG	AA	AA	AA	AA	AG	AA
	AG	AG	AG	AG	AA	AA	AA	AA
	AG	AA	AG	AA	AG	GG	AA	
117000032								
5906	rs1016145	12	AG	AG	AA	AA	AA	
	AA	AA	GG	AA	AA	GG	GG	AG
	GG	AG	AG	AA	AG	AA	AA	AG
	AG	AG	GG	AG	AA	AG	AA	AA
	GG	AA	AG	AA	AA	AG	AA	
20526181								
5907	rs10161471	12	GG	GG	GG	GG	GG	
	GG	GG	GG	GG	GG	GG	GG	GG
	GG	GG	GG	GG	GG	GG	GG	GG
	GG	GG	GG	GG	GG	GG	GG	GG
	GG	GG	GG	GG	GG	GG	GG	
110575951								
5908	rs1016173	21	AG	AG	GG	GG	GG	
	GG	GG	GG	GG	GG	AA	AG	AG
	AG	AG	GG	GG	GG	GG	GG	GG
	AG	GG	GG	GG	GG	GG	GG	GG
	GG	GG	GG	GG	GG	AG	GG	
22743803								
5909	rs1016184	4	AG	AG	AA	AA	AA	
	AA	AA	AG	AA	AG	AA	AA	AA
	AG	AA	AG	AA	AA	AA	AA	AG
	AA	AA	AA	AA	AG	AA	AA	AA
	AA	AA	AA	AA	AA	AG	AA	
180072951								
5910	rs10161901	13	AA	AG	AA	AG	AG	

	AG	AG	GG	GG	GG	GG	GG	GG
	GG	GG	GG	GG	GG	AG	AG	AG
	GG	AG	AG	AG	GG	GG	GG	GG
	GG	GG	GG	GG	AG	AG	GG	
40795656								
5911	rs10162004		13	AG	AG	AA	GG	AG
	GG	AG	GG	GG	GG	GG	GG	AG
	GG	GG	GG	GG	GG	AG	AA	GG
	AG	AG	AG	AG	GG	AG	AG	GG
	AG	AG	GG	GG	GG	GG	GG	
79186915								
5912	rs1016201		2	AG	AG	AG	GG	GG
	AG	AG	GG	GG	GG	GG	AG	AG
	AA	AG	GG	AG	AG	AG	GG	AG
	AA	AA	AA	AA	GG	AA	AG	AG
	AA	AG	GG	AG	GG	AG	GG	
220687059								
5913	rs10162035		13	GG	GG	AG	AG	AG
	AG	AA	AA	AA	AA	AG	AA	AA
	AA	AA	AG	AG	AA	AG	GG	AA
	AG	AG	AG	AG	AA	GG	AG	AG
	AG	AG	AG	AA	AA	AG	AG	
103995014								
5914	rs1016211		20	AA	AC	CC	AC	AC
	AA	AC	AC	AC	AA	AA	AA	AA
	AA	AC	AA	AC	AA	AC	AA	AA
	AA	AA	AA	AA	CC	AA	AC	AC
	AC	AC	CC	CC	AA	AC	AC	
17489413								
5915	rs1016214		20	GG	GG	GG	AG	GG
	AG	GG	GG	AG	GG	AA	AA	AA
	AA	AG	GG	GG	AG	AG	GG	GG
	GG	GG	GG	GG	AG	GG	GG	GG
	AG	AG	AG	GG	AG	AA	AG	
16992615								
5916	rs10162163		13	--	GG	GG	GG	GG
	GG	GG	AG	AG	AG	GG	GG	GG
	GG	GG	--	--	GG	GG	GG	GG
	GG	GG	GG	GG	AG	AA	AA	AG
	AG	AG	GG	AG	GG	AG	GG	
21855294								
5917	rs10162199		13	AG	GG	GG	GG	GG
	GG	GG	GG	GG	GG	GG	GG	GG
	GG	GG	GG	GG	GG	GG	AG	AG
	GG	GG	GG	GG	GG	GG	GG	GG
	GG	GG	GG	GG	GG	GG	GG	
95838077								
5918	rs1016222		6	GG	AG	AG	AG	AG
	AA	GG	GG	GG	AG	AA	AG	AA
	AA	AA	GG	AG	AG	AG	AG	GG

	GG	GG	GG	GG	AG	GG	AG	GG
	GG	AA	GG	AG	GG	GG	GG	
16421663								
5919	rs10162243		13	AG	AA	AG	AA	AA
	AA	AA	AA	AA	AA	AG	AA	AG
	AA	AA	AA	AA	AA	AA	AA	AA
	AA	AA	AA	AA	AA	AA	AA	AA
	AA	AA	AA	AA	AA	AA	AA	
34104405								
5920	rs10162279		13	AG	AG	AG	AG	AG
	AA	GG	AA	AG	AA	GG	GG	GG
	GG	GG	AA	AG	AG	AG	AG	AA
	AG	AA	AA	AG	GG	AG	AG	GG
	AG	GG	AG	GG	AA	AA	AG	
71871781								
5921	rs10162293		14	AA	AA	AA	AG	AG
	AG	AG	AA	AA	AA	AA	AA	AA
	AA	AG	AA	AA	AG	AA	AA	AA
	AA	AA	AA	AA	AA	AG	AG	AA
	AG	GG	AG	GG	AG	AA	GG	
54913860								
5922	rs10162396		14	AG	GG	AG	GG	AG
	GG	AG	GG	AG	GG	AG	AA	AA
	AA	AA	AG	GG	AA	AA	AG	AG
	AG	AG	AG	AA	AG	GG	GG	AG
	AA	AA	AA	AA	AG	AG	AA	
79681450								
5923	rs10162419		14	AA	AA	AG	GG	GG
	GG	GG	AG	GG	AG	AG	AA	AG
	AG	AG	AA	AA	AG	AG	GG	AA
	AG	AA	AA	AA	AA	AA	AA	AA
	GG	AG	AG	GG	GG	AG	AG	
35252097								
5924	rs10162434		14	GG	GG	GG	AG	GG
	GG	AG	GG	GG	GG	GG	GG	GG
	GG	GG	GG	--	AG	GG	GG	--
	AG	--	AG	AG	GG	GG	GG	GG
	AG	AG	AG	AA	GG	GG	AG	
68891494								
5925	rs10162436		14	AA	AA	AC	CC	AC
	AC	CC	CC	CC	CC	AC	AC	AA
	AC	CC	--	AC	CC	AA	AC	--
	AC	--	AC	AC	AC	AC	AA	AC
	AC	CC	AC	CC	AC	CC	AC	
82197228								
5926	rs10162458		14	AG	AG	AG	AA	AG
	AG	AA	AA	AG	AA	AA	AG	AG
	AG	AG	AA	AG	AG	GG	AG	GG
	AA	AG	AA	AA	AG	AA	AA	AA
	GG	AG	AG	GG	AA	AA	AA	

78105188
5927 rs10162482 14 AG AG AA AG AG
AG AG AA AA AA AG AG GG
GG AG AG AA AG GG GG AG
AG AG GG GG GG AG GG AG
GG GG GG GG AG GG AA
50847784
5928 rs10162542 14 GG GG GG AG GG
GG AG GG GG GG AG GG GG
GG GG AG AG AA GG GG AG
GG GG GG GG GG AG GG AG
GG GG GG GG GG AG GG
26462905
5929 rs1016256 5 AG GG AG AA AA
AA AA AG AG AG AA AA AG
GG GG GG GG GG AA AA AA
AA AA AG AG GG GG GG GG
AA AG AG AG GG GG AG
147528249
5930 rs10162574 14 CC CC CC CC CC
CC CC CC CC CC CC CC CC
CC CC CC CC CC CC CC CC
CC CC CC CC CC CC CC CC
CC CC CC CC CC CC CC
106141427
5931 rs10162599 14 AA AA AG AG GG
AG AG GG AG GG AG AG AG
AG AA AG GG GG AG AG AA
AG GG AG AG GG GG GG GG
AG GG AA AG AA AA AA
60403314
5932 rs10162627 15 AG AA AG AG AA
AG AG AA AG AG GG GG GG
AA GG GG GG GG AG AA GG
AA AA AA AG AG AG GG AA
AA AA AG AA AG GG AG
74299870
5933 rs10162642 15 AG AG AA GG GG
GG GG AG GG AG GG GG GG
GG GG GG GG GG GG AA GG
GG GG GG GG AG GG GG GG
GG GG AG GG AG GG AG
58577163
5934 rs10162653 15 AG GG AG GG GG
GG GG GG GG GG GG AG AG
GG AG GG GG GG GG GG GG
GG GG GG GG GG GG GG GG
GG GG AA AG GG GG GG
69905995
5935 rs10162686 15 AG AA AG AG GG

	AG	AG	AG	AG	GG	AG	AA	AA
	AA	AA	GG	AG	AG	AG	AG	AG
	AG	AA	AA	AG	AG	AG	AG	AG
	AA	AA	AG	AG	GG	AG	AG	
96015083								
5936	rs10162699		15	GG	GG	GG	GG	GG
	GG	GG	GG	GG	GG	GG	GG	GG
	GG	GG	GG	GG	GG	AG	AG	AG
	AG	AG	AG	AG	GG	GG	GG	GG
	GG	GG	GG	GG	GG	GG	GG	
82170051								
5937	rs10162720		15	AA	AA	AA	AA	AA
	AA	AA	AA	AA	AA	AA	AA	AA
	AA	AA	AA	AA	AA	AA	AA	AA
	AA	AA	AA	AA	AA	AA	AA	AA
	AA	AA	AA	AA	AA	AA	AA	
22796572								
5938	rs10162740		15	AG	AA	AG	AG	AA
	AG	AG	AA	AA	AG	AG	AG	GG
	AG	AA	GG	GG	GG	AA	AG	AG
	GG	GG	GG	AG	AG	GG	AG	GG
	AA	AG	AA	AG	AG	AG	AA	
101177226								
5939	rs10162803		15	GG	GG	GG	GG	GG
	GG	GG	GG	GG	GG	GG	GG	GG
	GG	GG	GG	GG	GG	GG	GG	GG
	GG	GG	GG	GG	GG	GG	GG	GG
	GG	GG	GG	GG	GG	GG	GG	
34417557								
5940	rs10162817		15	AA	AA	AA	AG	AG
	AA	AG	AG	GG	AA	AA	AG	AA
	AA	AA	AG	AG	AG	AG	AG	AG
	AA	AG	AG	AG	AA	AG	AG	AG
	AA	AA	AA	AA	AA	AA	AA	
37848963								
5941	rs10162860		15	AA	AA	AA	AA	AA
	AA	AA	AA	AA	AA	AG	AA	AG
	AA	AG	AG	AG	AA	AA	AG	AA
	AA	AA	AA	AA	AA	AG	AA	AG
	AA	AA	AA	AA	AA	AA	AA	
29255744								
5942	rs10162904		15	GG	GG	GG	GG	GG
	GG	GG	AG	AA	AA	GG	GG	GG
	AG	GG	AG	GG	GG	GG	GG	AG
	AG	AG	AG	GG	AA	AG	AG	AA
	GG	GG	GG	GG	GG	GG	GG	
71768511								
5943	rs10162924		15	AG	AA	AG	AG	AG
	AG	AA	AA	AG	AG	AA	AA	AA
	AA	AA	AA	AA	AA	AG	AG	AA

	AA	AA	AA	AA	AG	AA	AG	AA
	AA	AG	AG	AG	AA	AA	AA	
70490464								
5944	rs10162953		15	AG	AA	AG	AA	AG
	AG	AA	AG	AG	AG	AG	AG	AA
	AG	GG	GG	GG	GG	AG	GG	AA
	AA	AA	AA	AA	GG	AG	GG	AG
	AG	AA	AG	AA	AG	AG	AA	
100000885								
5945	rs10162968		15	AA	AC	AC	CC	AC
	AC	CC	AA	AA	AC	AC	CC	AC
	AC	CC	CC	CC	CC	CC	CC	CC
	AC	AA	AA	AC	AC	AA	AA	AC
	AC	AC	CC	AC	AA	AA	AC	
94728892								
5946	rs10162979		15	AA	AG	AG	GG	GG
	GG	GG	GG	GG	GG	AG	GG	AG
	GG	AG	GG	GG	GG	GG	GG	GG
	AG	GG	GG	GG	GG	GG	GG	GG
	GG	GG	GG	GG	GG	GG	GG	
35846891								
5947	rs1016298		X	GG	GG	GG	GG	GG
	GG	GG	GG	GG	GG	GG	GG	GG
	GG	GG	GG	GG	GG	AG	GG	AA
	GG	GG	GG	AG	AG	GG	GG	AA
	GG	GG	GG	GG	GG	GG	GG	
119334499								
5948	rs1016301		14	AC	AC	AA	AA	AA
	AA	AA	AA	AA	AA	AA	AA	AA
	AA	AA	AC	AA	AA	AA	AA	AA
	AA	AA	AA	AA	AA	AA	AA	AA
	AC	AA	AC	AA	AC	AC	AA	
89823153								
5949	rs10163046		15	AG	GG	AG	GG	AG
	AG	AG	AA	AA	AA	AA	AG	AA
	AA	AA	AG	AA	AA	AA	AA	AG
	AA	AA	AA	AA	AG	AA	AG	AA
	AG	AA	AA	AA	AA	AA	AA	
74133049								
5950	rs1016306		3	AC	AA	AC	AC	AA
	AC	AA	AA	AA	AC	AC	AC	AC
	AC	AA	AC	AC	AC	AC	AC	AC
	AC	CC	CC	AC	AA	AA	AA	AA
	AC	AC	AC	CC	AA	AC	AA	
85082588								
5951	rs10163073		15	CC	CC	CC	CC	CC
	CC	CC	CC	CC	CC	CC	CC	CC
	CC	CC	CC	CC	CC	CC	CC	CC
	CC	CC	CC	CC	CC	CC	CC	CC
	CC	CC	CC	CC	CC	CC	CC	

24031107
5952 rs10163100 15 AA AG AG GG AG
AG GG AA AA AG AG AG AG
AG AG AA AA AA AA AA AG
AG AG AA AA AA AA AA AA
AG GG AA AG GG GG GG
24217764
5953 rs10163113 15 AA AA AA AA AA
AA AA AA AA AA AA AA AA
AA AA AA AA AA AA AA AA
AA AA AA AA AA AA AA AA
AA AA AA AA AA AA AA
24217920
5954 rs10163131 15 AA AG AG AA AA
AA AA AA AA AA AG AG AG
AG AG AG GG GG AA AA AA
AA AG AG AA AG AG GG AA
AG GG GG GG AA AG AG
88439774
5955 rs10163147 15 AA AA AA AA AA
AA AA AA AA AA AA AA AA
AA AA AA AA AA AA AA AA
AA AA AA AA AA AA AA AA
AA AA AA AA AA AA AA
22860886
5956 rs10163171 15 AG AG AA GG GG
GG GG GG GG GG AG AG AG
GG AG AA AG AG AG GG AG
GG GG AG GG GG GG GG GG
AA GG AG AG AG AG AG
53209322
5957 rs10163226 16 GG GG GG GG GG
GG GG AG GG AG GG GG GG
GG GG GG GG GG GG GG GG
GG GG GG GG AG GG AG AG
GG GG GG GG GG GG GG
7616188
5958 rs10163302 16 AG AA AG AG AG
AG AG AG GG AG AA AA AA
AG AG AG AA AG AG AG AG
AA AA AA AA AG GG GG GG
GG AA AA AA AG AG GG
3392984
5959 rs10163339 16 AG GG AG AA AA
AA AA AG GG AA AG AG AA
AA AA AA AA AA AA AA AA
AG AA AA AG AG AA AG AA
AA AA AA AA AA AA AA
77838896
5960 rs10163354 16 AA AG AA AA AA

AA AA AA AA AG AG AG AA
AA AA AA AA AA AA AA AA
AA AG AA AA AG AA AA AG
AG AG AA AG AG AG AA
48210719
5961 rs1016339 2 GG GG GG AG GG
AG GG AG AG AG GG GG GG
GG GG GG GG GG AG AG AA
GG GG GG GG AG AG AA AG
GG AG GG GG GG GG GG
56421972
5962 rs1016345 3 GG GG GG GG GG
GG GG GG GG GG GG GG GG
GG GG GG GG GG GG GG GG
GG GG GG GG GG GG GG GG
GG GG GG GG GG GG GG
136785925
5963 rs1016347 2 AA AA AA AA AA
AA AA AG AA AG AA AG AG
AG AG AG AG AA AA AA AA
AA AA AA AA AA AA AA AA
AG AA AA AA AA AA AA
77584897
5964 rs10163509 17 AA AG AG AG AG
GG AG AG AG AA AG GG GG
GG GG AA AA AG AG AG GG
GG GG GG GG AG AA AA AA
AG AA AA AA AA AA AG
6031324
5965 rs10163540 18 GG GG GG GG GG
GG GG GG GG GG GG GG GG
GG GG GG GG GG GG GG GG
GG GG GG GG AG AG AG AG
GG AG GG GG GG GG GG
8142163
5966 rs10163677 18 GG GG GG GG GG
GG GG GG GG GG GG AG AG
AG AG GG GG GG GG GG GG
GG GG GG GG GG GG GG GG
GG GG GG GG GG GG GG
2392819
5967 rs10163682 18 AG AA AG AA AG
AA AG AG AG GG GG AA AG
AG AG AG AG AA AA AG AA
AG AG GG GG AG AG AA AA
AA AA AG AA AG AG AA
40701725
5968 rs10163723 18 GG GG GG AG AG
GG AG GG GG GG GG GG GG
GG GG GG GG GG GG GG GG

GG GG GG GG AG GG GG AG
GG GG GG GG AG AG GG
19423695
5969 rs10163732 18 GG GG GG GG GG
GG GG GG GG GG AG AG AG
AG GG GG GG GG GG GG GG
GG AG GG GG AG AG AG GG
GG GG GG GG GG GG GG
19540467
5970 rs10163754 18 GG GG GG GG GG
GG GG GG GG GG GG GG GG
GG GG AG AG AG GG GG GG
GG AG AG GG GG GG GG GG
AG GG GG GG AG GG AG
2817052
5971 rs10163809 18 AA AA AA AA AA
AA AA AA AA AA AA AG AA
AA AA AG AA AG AG AA AG
AG AG AA AG AA AA AA AA
AA AA AA AA AA AA AA
61129765
5972 rs10163818 18 GG GG GG GG GG
GG GG AG GG GG GG GG GG
GG GG GG GG GG GG GG GG
GG GG GG GG GG GG GG GG
GG AG GG AG GG GG GG
3847600
5973 rs10163904 18 AG AG GG GG AG
GG AG AG AA AG AG AA AG
AG AA AG AG GG GG AG GG
AG AG AA AG AA AA AA AA
AA AA AA AA AG GG AG
61139211
5974 rs10163918 18 AA AA AA AG AG
AG AA AA AA AA AG AA AG
AG AA AA AA AA AA AA AA
AA AA AA AA AA AA AA AA
AA AA AA AA AA AA AA
61875586
5975 rs1016396 2 AG GG AG AA AG
AG AG AG AG AG AG AG AG
AA AG AG AG AG GG AA AG
AA AA AG GG AG GG AG AG
AA AA AA AA AG AG GG
211570448
5976 rs10163981 18 GG GG AG AG AG
GG AA AA AG AA AG AG AG
AG AG GG AG AG AG AG GG
AG AG GG AG AG AG AG AG
AA AG AG AG AA AG AG

51246517
5977 rs10164030 18 AG GG AG AG AG
AG AA AA AG AA AA AA AA
AA AA GG AG AG AG AG AG
AA AA GG AG AG AG AG AG
AG AA AG AA AA AG AG
51373602
5978 rs10164065 18 GG GG GG GG GG
GG GG GG GG AG GG AG GG
GG GG AG GG GG GG GG GG
AG GG GG AG GG GG GG GG
GG AG GG GG GG GG AG
1746421
5979 rs10164146 18 AA AA AA AA AA
AA AA AA AA AA AA AA AA
AA AA AA AA AA AA AA AA
AA AA AA AA AA AA AA AA
AA AA AA AA AA AA AA
10611414
5980 rs10164234 18 GG GG AG GG GG
GG GG GG GG GG GG GG GG
GG GG GG GG GG GG GG GG
GG GG GG GG GG GG GG GG
GG GG GG GG GG GG GG
7004307
5981 rs10164276 19 GG GG GG GG AG
GG AG AG GG GG GG GG GG
AG AG GG GG GG AG GG GG
GG GG GG GG AG GG GG AG
AG AG GG AG GG GG GG
12342177
5982 rs10164308 19 GG GG GG GG GG
GG GG GG GG GG GG GG GG
GG GG GG GG GG GG GG GG
GG GG GG GG GG GG GG GG
GG GG GG GG GG GG GG
24429443
5983 rs10164335
19 -- -- -- -- -- -- -- --
-- -- -- -- -- -- -- --
-- -- -- -- -- -- -- --
-- -- -- -- -- -- -- --
-- -- -- -- 2867717
5984 rs10164338 19 AA AA AA AA AA
AA AA AA AA AA AA AA AA
AA AA AA AA AA AA AA AA
AA AA AA AA AA AA AA AA
AA AA AA AA AA AA AA
18570154
5985 rs10164353 19 GG GG GG GG GG

GG GG GG GG GG GG GG GG
GG GG GG GG GG GG GG GG
GG GG GG GG GG GG GG GG
GG GG GG GG GG GG GG
32080830
5986 rs1016441 21 AA AA AA CC CC
CC AC CC CC AA AC AC AA
AA AA CC CC CC AA AC AC
AA AA AA AA AC AA AC AA
AA AA AC AA AC CC AA
36537097
5987 rs10164440 2 AA AA AA AA AA
AA AA AA AA AA AA AA AA
AA AA AA AA AA AA AA AA
AA AA AA AA AG AA AG AG
AA AA AA AA AA AA AA
240288363
5988 rs10164499 2 GG GG GG GG GG
GG GG GG GG GG GG GG GG
GG GG GG GG GG GG GG GG
GG GG GG GG GG GG GG GG
GG GG GG GG GG GG GG
58264394
5989 rs1016462 6 GG AG AG GG GG
GG GG GG GG GG GG GG GG
GG GG GG GG GG GG GG GG
GG GG GG GG GG GG GG GG
GG GG AG AG GG GG GG
92918349
5990 rs10164633 2 AA AA AA GG AA
AG AG AG AG AG AA AA AA
AA AA AG AG AG AG AG AA
AG GG AG AG AA AA AA AA
AA AA AA AA AG AG AG
46028107
5991 rs10164639 2 AA AA AG AG GG
GG AG AG AG AG AA GG AG
AG AA GG AG AG AG GG GG
AG AG AG AG GG AG GG GG
AG GG AA AG AG GG AG
242255378
5992 rs10164649 2 AG AA AA AG AG
AA GG AA AA AA AA AA AA
AA AA AA AA AA AA AG AA
AA AA AA AA AG AA AG AA
AA AA AG AG AA AA AA
109515871
5993 rs10164651 2 GG GG GG GG GG
GG GG GG GG GG AG AG AG
GG AG AG GG AG GG GG GG

GG GG GG GG GG GG GG GG
GG GG GG GG GG GG GG
118250637
5994 rs10164739 2 AA AA AA AG GG
AG AG AG AG GG AG AG AG
AA AA AA AA AA AA AA AA
AG -- AG AG AA AA AA AA
AG GG AG GG GG AG AG
181470028
5995 rs10164844 2 AA AA AG AG AG
AG AG AG AG AG AG AA AA
AG AG GG GG AG AG AG GG
GG AG GG AG AG AG AG GG
AG GG AA AG AG AG GG
63652403
5996 rs10164931 2 AG AG AG AG AG
AA GG AG AA AA AA AA AA
AA AA AA AA AA AA AA AA
AG AG AG AG AG AA AG AG
AG AG AA AA AG AG AA
237840220
5997 rs10165024 2 CC CC CC CC CC
CC CC CC CC CC CC CC CC
CC CC CC CC CC CC CC CC
CC CC CC CC CC CC CC CC
CC CC CC CC CC CC AC
16372453
5998 rs1016503 6 GG GG GG AG AG
AA GG AG AG AG GG GG GG
GG GG AG GG AG AG AG GG
AG AG AG AG GG GG GG GG
GG AG AG AG GG GG GG
132992748
5999 rs10165034 2 GG GG GG GG GG
GG GG GG GG GG AG GG GG
GG GG GG GG GG GG GG GG
GG GG GG GG GG GG GG GG
GG AG GG AG GG GG GG
4451884
6000 rs10165099 2 AA AG AA AA AG
AA AG GG AG GG AG AG AG
GG GG AG GG GG AG AG AG
GG GG GG GG AG AG AG AA
GG GG GG GG AA AG AG
3402591
6001 rs1016510 4 AC AC AC CC AC
AC CC AC AC CC CC CC CC
CC AC AC CC AC AC AC CC
AC CC CC AC AA AC AC AA
CC CC AA AC CC CC AC

86819377
6002 rs1016512 8 GG GG GG AG AG
GG AG AG GG GG AG AG AG
AG AG AG AG GG GG GG AG
AA GG AG AG GG GG GG GG
GG AG AG GG GG AG GG
8881820
6003 rs10165176 2 AA AA AA AA AA
AA AA AA AA AA AA AA AA
AA AA AA AA AA AA AA AA
AA AA AA AA AA AA AA AA
AA AA AA AA AA AA AA
168483509
6004 rs10165187 2 GG GG GG GG GG
GG GG AG AG AG GG GG GG
GG GG AG AG AG AG AG AG
GG GG GG GG GG GG GG GG
AG AG GG AG AG GG AA
71167577
6005 rs10165220 2 AG AG AG GG GG
GG GG AG AG AG AG AA AA
AG AG AG AG GG AA AA AA
AG GG AG AG GG GG GG GG
AG GG GG GG AG GG AG
95537000
6006 rs10165268 2 AA AA AA CC AC
AC CC AC CC CC AC CC CC
AA AA AC AC CC AC AC AA
AC AA AA AA AC AA AC AA
AC AA AC AC AA AA AA
49700419
6007 rs10165310 2 AA AA AA AA AA
AA AA AA AA AA AA AA AA
AA AA AA AA AA AA AA AA
AA AA AA AA AA AA AA AA
AA AA AA AA AA AA AA
16650585
6008 rs10165323 2 GG GG GG GG GG
GG GG GG GG GG GG GG GG
AG AG AG GG AG AG GG AG
GG GG AG GG GG GG GG GG
GG GG GG GG GG GG GG
38010296
6009 rs10165386 2 AG AA AA GG GG
AG GG AA AA AA AA GG AG
AG AG AG AG AA AA AA AA
AG AA AG AG AG AG GG AA
AA AG AG AA AG AG AG
99301833
6010 rs10165398 2 AG AG GG AA AA

AA AA AA AA AA AG GG AG
GG GG AG AG AA AA AG AA
AG GG AA AA AA AG AG AA
AA AG AG AG AG AA AG
120217053
6011 rs10165470 2 AG AG GG AA AA
AA AA AA AA AA AA AA AA
AA AA AG AA AG AG AA AA
AA AG AA AA AA AG AA AG
AA AA AG AA AA AG AA
126466408
6012 rs1016553 3 GG GG GG GG GG
GG GG GG GG GG GG GG GG
GG GG GG GG GG GG GG GG
GG GG GG GG GG GG GG GG
GG GG GG GG GG GG GG
65379294
6013 rs1016555 6 GG GG GG GG AG
AG GG GG AG GG AG AG AG
AG GG AG GG GG GG GG AG
GG GG GG GG AG GG GG GG
AA AG AG AG GG AG GG
77422630
6014 rs10165566 2 CC CC AC AC AC
AA CC CC CC CC AA AA AA
CC CC AC CC CC CC CC CC
AA AC AA AC AA CC AC AC
CC CC CC CC AC CC AC
61941368
6015 rs1016560 10 AG GG GG AG AG
GG AA GG GG AG AG AG GG
GG AG AG AG GG GG AG GG
GG GG GG GG AA AG AG AG
AA AG AG AG AG AG AA
124790152
6016 rs10165614 2 GG GG GG GG GG
GG GG GG GG GG GG GG GG
GG GG GG GG GG AG GG AG
GG GG GG GG GG GG GG GG
GG GG GG GG GG GG GG
112698460
6017 rs10165688 2 AG AA AG AG AG
AG GG AG GG GG AA AG AA
AA AA AA AA AA AG AG AA
GG AG AG GG AA AG AG AG
AG AA AG AA AA AA AA
149892352
6018 rs1016569 2 AA AA AA AA AA
AA AA AA AA AA AA AA AA
AA AA AA AA AA AA AA AA

	AA	AA	AA	AA	AA	AA	AA	AA
	AA	AA	AA	AA	AA	AA	AA	
200086752								
6019	rs10165754		2	AA	AG	AA	AA	AA
	AA	AA	AA	AG	AG	GG	AG	GG
	AG	GG	AA	AA	AG	AG	AA	AG
	AA	AA	AA	AA	AA	AG	AA	AG
	AA	AG	AG	AA	AA	AA	AA	
219287370								
6020	rs10165759		2	AG	GG	AG	AG	AG
	AG	AA	GG	GG	GG	AG	GG	GG
	GG	GG	AG	AG	AG	GG	GG	GG
	AG	AG	AG	AG	GG	AG	GG	GG
	AG	GG	GG	GG	AG	AG	GG	
241458609								
6021	rs10165836		2	AG	AG	AG	AA	AG
	AA	AG	AG	AA	AG	AA	AG	AG
	GG	GG	AG	AG	AG	AA	AG	AA
	GG	GG	GG	GG	AG	AA	AA	AA
	GG	GG	AG	AG	AA	AG	AG	
3433368								
6022	rs10165842		2	GG	AG	GG	GG	AG
	AG	AG	AG	AG	AG	GG	GG	GG
	GG	GG	AG	AG	AG	AG	AA	AA
	AG	AG	AG	AG	GG	AG	AG	AG
	AG	GG	AG	AG	AA	AG	AG	
241313129								
6023	rs10165843		2	AA	AA	AA	AA	AA
	AA	AA	AA	AA	AA	AA	AA	AA
	AA	AA	AA	AA	AA	AA	AA	AA
	AA	AA	AA	AA	AA	AA	AA	AA
	AA	AA	AA	AA	AA	AA	AA	
238400285								
6024	rs10165859		2	GG	GG	GG	GG	GG
	GG	GG	GG	GG	GG	GG	GG	GG
	GG	GG	GG	GG	GG	GG	GG	GG
	GG	GG	GG	GG	GG	GG	GG	GG
	GG	GG	GG	GG	GG	GG	GG	
109790885								
6025	rs1016586							
16	--	--	--	--	--	--	--	--
	--	--	--	--	--	--	--	--
	--	--	--	--	--	--	--	--
	--	--	--	--	--	--	--	--
	--	--	--	--	23588966			
6026	rs10165876		2	GG	GG	GG	AG	AG
	AA	GG	AG	AG	AG	AG	AA	AA
	AA	AA	AG	AG	GG	GG	GG	AG
	GG	GG	GG	GG	AG	AG	AG	AG
	GG	AA	GG	AG	AG	AG	GG	

240574731
6027 rs10165917 2 AA AG AA AA AG
AA AG AG AA AA AG AG GG
GG GG AA AA AA AG AA AA
AG AG AG AA AA AG AG AA
AG AA AG AG AG AA AG
235292064
6028 rs10165920 2 AG GG AG GG GG
GG GG AG GG AG GG GG GG
GG GG GG GG GG GG GG GG
AG AG GG GG GG GG GG GG
GG GG GG GG GG GG GG
215576391
6029 rs10165956 2 GG GG GG GG GG
GG GG GG GG AG GG GG GG
GG GG GG GG GG AG AG AG
GG GG GG GG GG GG GG GG
GG GG GG GG GG GG GG
238086404
6030 rs10165964 2 AA AG AA AA AA
AA AG AG AG AG AA AG AG
AG AG AA AG AA AG AG AG
AG AG AG AG AG AG AG AA
GG AA AG AA AA AA AA
4677204
6031 rs10165970 2 GG AG AG AG GG
GG AG GG GG GG AG AG AA
AG AG GG GG GG AG AG GG
GG GG GG GG AG AG AG AG
AG AG GG AG GG GG GG
101456989
6032 rs10166048 2 AA AA AA AA AA
AA AA GG AG GG AG AA AA
AA AA AG AG AA AA AA AA
AG AG AG AG AA AA AA AA
AA AA AG AA AG GG AA
101395850
6033 rs10166107 2 CC AC AC CC CC
CC CC CC CC CC AC AC CC
AC AC CC CC CC CC CC CC
AC CC AC AC AC AC AA CC
CC CC CC CC CC CC CC
173557660
6034 rs1016612 1 AC AA AC AA AA
AA AC AA AA AA AA AA AC
CC AA AC AA AA AA CC AA
AA AC AC AC AA CC AC AC
AC CC CC CC AC CC AA
176878679
6035 rs10166150 2 AC CC AC AA CC

AC AC CC CC CC CC CC CC
CC CC CC CC CC CC CC CC
CC CC CC CC CC CC CC CC
CC CC CC CC CC CC CC
18332162
6036 rs1016618 3 AA AA AA AA AA
AA AA AA AA AA AA AA AA
AA AA AA AA AA AA AA AA
AA AA AA AA AA AA AA AA
AA AA AA AA AG AG AA
191228990
6037 rs10166217 2 CC CC CC CC CC
CC AC AC AC AC AC AC AA
AC AC CC CC CC AC AA CC
AC AC AC AC CC AC AC AC
AC CC CC CC CC AC CC
141398489
6038 rs10166260 2 GG GG GG GG GG
GG GG GG GG GG GG GG GG
GG GG GG GG GG GG GG GG
GG GG GG GG GG GG GG GG
GG GG GG GG GG GG GG
200337137
6039 rs10166334 2 AA AA AA AA AA
AA AA AA AA AA AA AG AA
AA AA AA AA AA AG AA AA
AG AG AG AG AA AA AA AA
AA AA AA AA AA AA AA
237740508
6040 rs10166377 2 AG GG GG GG GG
GG GG AA AA AG AG AG AG
AG AG GG GG GG GG GG AG
GG GG GG GG GG GG GG GG
GG GG GG GG AA AA AG
44312697
6041 rs10166392 2 AA AG AA AG GG
AG AG AG AG AG AG AG GG
AG AG AA AA AG AG AA AG
AA AA AG AA AG AA AG AG
AG AG AG AA GG GG AG
4086161
6042 rs10166397 2 AC AA AA AC AA
AA AC CC CC AC AA AA AC
AA AA AA AA AC CC CC AC
AA AC AC AA AA AA AA AA
AC AA AC AC AC AC AC
44372909
6043 rs10166445 2 AC CC AC AC AA
AC AA CC AA AC AC CC AC
AC AC AC AA AA CC AA AC

AA AA AA AA AA AA AA AA
AA AA AA AA AC AC AC
210773693
6044 rs10166446 2 AC AC AC AC AA
AA AC CC CC CC AC CC CC
AC AC CC CC CC AC AC AA
CC CC CC CC AA AC AA AC
AC AA CC AC AA AA AA
57723036
6045 rs10166471 2 GG GG GG GG GG
GG GG GG GG GG GG GG GG
AG AG AA AG AG GG GG GG
GG AG AG GG GG GG GG GG
AG AG GG AG AG GG AA
157216692
6046 rs10166475 2 AG AA AG GG AG
AG GG GG AG GG AA AG AG
AA AA GG GG GG GG GG GG
AG GG GG AG GG GG GG GG
AG AG AG AA AG AG GG
13606441
6047 rs1016656 17 AA AA AA AA AA
AA AA AA AA AA AA AA AA
AA AA AA AA AA AA AA AA
AA AA AA AA AA AA AA AA
AA AA AA AA AA AA AA
26203825
6048 rs10166563 2 GG GG GG GG AG
AG GG GG GG GG GG GG GG
GG GG GG GG GG GG GG GG
GG AG GG GG GG GG GG GG
GG GG GG GG GG GG GG
86163082
6049 rs10166589 0 GG GG GG GG GG
GG GG GG GG GG GG GG GG
GG GG GG GG GG GG GG GG
GG GG GG GG GG GG GG GG
GG GG GG GG GG GG GG 0
6050 rs10166601 2 AA AA AA AA AA
AA AA AA AA AA AA AA AA
AA AA AA AA AA AA AA AA
AA AA AA AA AA AA AA AA
AA AA AA AA AA AA AA
42242885
6051 rs10166612 2 AA AA AA AA AA
AA AA AG AG AG AA AG AA
AA AA AA AA AA AA AA AA
AA AA AA AA AA AG AA AG
AA AG AA AA AG AA AG
85804096

6052 rs10166619 2 AA AA AA AA AA
AA AA AA AA AA AA AA AA
AA AA AA AA AA AA AA AA
AA AA AA AA AA AA AA AA
AA AA AA AA AA AA AA
42418583
6053 rs10166654 2 GG GG GG AG GG
GG AG AG AG GG AG GG GG
GG GG GG GG GG GG AG GG
AG AG AG AG GG GG GG GG
GG GG GG GG GG GG GG
236611263
6054 rs10166677 0 CC CC CC CC CC
CC CC CC CC CC CC CC CC
CC CC CC CC CC CC CC CC
CC CC CC CC CC CC CC CC
CC CC CC CC CC CC CC 0
6055 rs10166692 2 AG AA AA AA AA
AA AA AA AA AA AA AA AA
AA AA AG AA AG AA AA AA
AG AG AA AG AA AA AA AA
AA AA AA AA AA AA AA
46077038
6056 rs10166703 2 AG GG AG GG GG
GG AG GG GG GG AG AA AG
GG GG GG GG AG GG GG AG
GG GG GG GG GG AG GG GG
GG GG GG GG GG GG GG
153323026
6057 rs10166726 2 GG GG GG GG GG
GG GG GG GG GG GG GG GG
GG GG GG GG GG GG GG GG
GG GG GG GG GG GG GG GG
GG GG GG GG GG GG GG
109979216
6058 rs10166731 2 GG AG GG GG GG
GG GG AG GG GG GG GG GG
GG GG GG GG AG AG AG AG
AG AG AG AG GG GG GG GG
AG GG GG GG GG GG AG
240737039
6059 rs10166750 2 GG GG GG GG GG
GG GG GG GG GG GG GG GG
GG GG GG GG GG GG GG GG
GG GG GG GG GG GG GG GG
GG GG GG GG GG GG GG
63759028
6060 rs10166756 2 GG GG GG GG GG
GG GG GG GG GG GG AG AG
GG GG AG AG GG GG GG AG

GG GG GG GG AA GG AG AG
GG GG AG GG AG AG AG
13299918
6061 rs10166792 2 AC AC AC CC CC
CC CC AC AC AC CC CC CC
CC CC CC CC CC AC AC AC
CC CC CC CC CC CC CC CC
CC CC CC CC CC CC CC
20777149
6062 rs10166800 2 AA AA AA AA AA
AA AC AA AA AA AA AA AA
AA AA AA AA AA AA AA AA
AC AA AC AC AA AA AA AA
AA AA AA AA AA AC AA
12128492
6063 rs10166805 2 AA AA AA AA AA
AA AA AA AA AA AA AA AA
AA AA AA AA AA AA AA AA
AA AA AA AA AA AG AA AG
AA AA AG AA AA AA AA
88959086
6064 rs1016686 14 AG AG AG AG AG
GG AA GG AG GG GG AA AG
AG AG AG AG AG GG GG GG
AG GG AG AG AG GG AG GG
AG GG GG GG GG GG AG
94839240
6065 rs10166863 2 AG AG GG AG AG
AG AA GG GG GG AG AA AG
AG AG AA AA AA AA AA AA
GG AA GG GG AG AA AA AG
AG AG AA AG AG GG AA
101979822
6066 rs10166909 2 AA AG AA AA AA
AA AA AA AA AA AA AG AA
AA AA AA AA AA AA AA AA
AG AG AG AG AA AG AG AA
AA AG AA AG AA AA AA
227372171
6067 rs1016694 21 AG AA AG AG AG
AA GG AG AG AG GG GG GG
AG AG GG GG GG AA AA AG
AG AG AG AG GG GG GG GG
AG AG AG AA AA AA AA
39234818
6068 rs10167061 2 GG GG GG GG GG
GG GG GG GG GG GG GG GG
GG GG GG GG GG GG GG GG
GG GG GG GG GG GG GG GG
GG GG GG GG GG GG GG

177977075
6069 rs10167072 2 AG AA AG AG AG
AA GG GG AG GG GG AA AG
AG AG AG GG AG AG AG AG
AG GG AG AG AG AG AG AG
AA AA AA AA AA AA AA
7072935
6070 rs10167103 2 AA AG AA AG AG
AG GG AA AA AA AA AG AG
AG AG AG AG AG AG AA AG
AA AG AA AA GG AG GG AG
AA AA AA AA AG AA AG
127578744
6071 rs10167258 2 AA AA AA GG GG
AG GG GG AG AA GG GG GG
GG GG AG GG GG AG GG AG
AG AA AA AG AG AG AG AA
AA GG GG GG AG AG AA
157596697
6072 rs1016726 7 GG GG GG GG GG
GG GG GG GG GG AG AG GG
GG GG GG GG GG GG GG AG
GG GG GG GG GG AG GG GG
GG GG GG GG GG GG GG
29356403
6073 rs10167316 2 AA AG AG AA AA
AG AG AG AG AG AG AG GG
GG GG AG AA AA GG AG AG
GG AG AG GG AA GG AG AG
AA AG GG AG GG AG AG
159237284
6074 rs1016739 X GG GG GG GG GG
GG GG GG GG GG GG GG GG
GG GG GG GG GG GG GG GG
GG GG GG GG GG GG GG GG
GG GG GG GG GG GG GG
102220144
6075 rs1016740 22 GG GG GG AG AG
AA GG AG GG AG GG GG AG
AG AG AG AG AG AG AG AA
AG AG AG AA AG AA AA AA
GG AA AG AG AG AG AG
32963983
6076 rs10167468 2 GG GG GG GG GG
GG GG GG GG GG AG GG AG
GG GG GG GG GG GG GG GG
AG GG AG GG GG GG GG GG
GG GG GG GG GG GG GG
69314667
6077 rs10167538 2 GG GG GG GG GG

	AG	GG	GG	GG	GG	GG	GG	GG
	GG	GG	GG	GG	AG	GG	GG	AG
	GG	GG	GG	GG	GG	GG	GG	GG
	GG	GG	GG	GG	GG	GG	GG	
134922529								
6078	rs10167564		2	GG	GG	GG	AA	AA
	AA	AA	GG	GG	GG	AG	AG	GG
	GG	GG	AG	AG	AA	AG	AA	AG
	GG	GG	GG	GG	AG	GG	AG	GG
	AG	AG	AG	AG	GG	GG	GG	
108988625								
6079	rs10167584		2	AG	AG	AG	AG	AG
	GG	AG	AA	AG	AA	AG	AG	GG
	GG	GG	GG	AG	AG	AG	AG	GG
	AG	AG	AG	AG	GG	GG	GG	GG
	AA	AA	AA	AA	GG	AG	AG	
82331873								
6080	rs1016762		3	GG	GG	GG	GG	AG
	AG	AG	AG	AG	GG	AA	AA	AA
	GG	AG	GG	GG	GG	AA	AA	AG
	AG	AG	AG	GG	AG	AA	AG	AA
	AA	GG	AG	AG	GG	GG	AG	
41206998								
6081	rs10167628		2	--	--	--	AG	--
	AG	--	--	AG	--	--	GG	--
	AG	AG	--	--	--	AG	AG	--
	AG	AG	AG	AG	AG	--	AG	--
	AG	AG	--	AG	AG	AA	AG	
84035363								
6082	rs10167702		2	AA	AA	AA	AA	AA
	AA	AA	AA	AA	AA	AG	AG	AA
	AA	AA	AA	AA	AA	AA	AA	AA
	AA	AA	AA	AA	AA	AA	AA	AA
	AA	AA	AA	AA	AA	AA	AA	
232940143								
6083	rs10167726		2	GG	--	AG	AG	GG
	GG	--	GG	GG	GG	GG	AG	GG
	AG	AG	GG	GG	GG	GG	--	GG
	AG	AG	AG	AG	AG	AG	AG	AG
	GG	AG	AG	GG	AG	AG	GG	
36879521								
6084	rs10167803		2	AA	AA	AA	AA	AA
	AA	AA	AA	AA	AA	AA	AA	AA
	AA	AA	AA	AA	AA	AA	AA	AA
	AA	AA	AA	AA	AA	AA	AA	AA
	AA	AA	AA	AA	AA	AA	AA	
169938853								
6085	rs10167828		2	AG	AG	AG	AA	AA
	AG	AA	AA	AA	AA	AG	GG	GG
	GG	GG	AA	AA	AA	GG	AG	AG

GG GG AG AG AA AA AA AA
AG AA AA AA AA AA AA
117463256
6086 rs10167856 2 AA AA AA AG AG
AA AG AA AG AG AA AA AA
AA AA AG AA AA AA AG AA
AA AA AA AA AA AA AA AA
AA AG AA AG AA AA AA
167540475
6087 rs10167878 2 AG AA AG AA AG
AG AA AA AA AA AG AG AA
AA AA AG AG GG AG AG AA
AG AA AG AG AG AG GG AA
AG AA AA AA GG AG GG
172048487
6088 rs10167879 2 GG GG GG GG GG
GG GG GG GG GG GG GG GG
GG GG GG GG GG GG GG GG
GG GG GG GG GG AG GG AG
GG GG AG GG GG GG GG
88867996
6089 rs10167883 2 AG AG AA AA AG
AA AG AA AG AG AG AA AA
AA AA AA AA AG AA AA AA
AA AA AA AA AG AA AA AG
AG AA AA AA GG AG GG
46256399
6090 rs10167905 2 AA AA AA AA AC
AC AC AA AA AA AA AA AA
AA AA AC AC AC AA AC AA
AC AC AA AA AA AA AA AA
AC AA AA AA AA AA AA
184680313
6091 rs10167913 2 AG AG AA GG GG
GG AG AG GG GG GG GG AG
AG AG AA AG AG AG GG AG
GG AG AG GG AG AG AG GG
AG GG AA AG AA AA AG
165183257
6092 rs10167990 2 CC CC CC CC CC
CC CC AC AC AA CC CC CC
CC CC CC CC AC CC CC AC
CC AC CC CC AC CC CC AC
CC AA CC AC CC CC CC
31101646
6093 rs1016815 1 GG AG AG GG AG
AG GG AA GG AA AA AA AA
AA AA AA AG AG AG AG GG
AG AG GG AG AG AG AG GG
AG GG AA AG GG AG AG

175428957							
6094	rs10168151	2	AG	AG	GG	AA	GG
AG	AG	AG	AG	AG	GG	AA	AG
AA	AA	AG	AG	GG	AG	AG	AG
GG	AG	GG	AG	AG	AA	AA	AA
AG	AG	AG	AG	GG	GG	AG	
67492984							
6095	rs10168156	2	AG	AA	AG	AG	AG
GG	AG	GG	GG	GG	AG	AG	AG
GG	GG	GG	GG	GG	AG	GG	AA
AG	GG	AG	GG	GG	GG	GG	GG
GG	GG	GG	GG	AA	AG	AA	
175506631							
6096	rs1016824	X	AA	AA	AA	AG	AA
AA	AG	AG	AA	GG	GG	GG	GG
GG	GG	AG	AA	AG	GG	AG	GG
GG	GG	GG	GG	AG	AA	AG	AA
AG	GG	GG	GG	AG	AA	GG	
140230896							
6097	rs10168253	2	AG	GG	AG	GG	AG
AG	GG	AG	AG	AA	AA	AA	AA
AA	AA	GG	GG	GG	AA	AG	AA
GG	AG	GG	GG	AG	GG	GG	GG
GG	AA	AG	AA	AG	AG	AA	
180659575							
6098	rs10168275	2	GG	GG	GG	AG	AG
AG	GG	AG	GG	AG	GG	GG	GG
GG	GG	AG	AG	AA	AG	GG	GG
AG	AG	AG	GG	AG	GG	AG	AG
GG	GG	GG	GG	GG	GG	GG	
231623555							
6099	rs10168339	2	GG	GG	GG	GG	GG
GG	GG	GG	GG	GG	GG	GG	GG
GG	GG	GG	GG	GG	GG	GG	GG
GG	GG	GG	GG	GG	GG	GG	GG
GG	GG	GG	GG	GG	GG	GG	
64105723							
6100	rs10168340	2	AG	GG	AG	AG	AG
AA	AG	AG	AA	AG	AG	AA	AG
AG	AG	AG	AG	AG	AG	AG	AG
GG	AG	GG	GG	AG	GG	GG	GG
AG	GG	AG	AG	AG	GG	AA	
5723564							
6101	rs10168342	2	AG	AA	AA	AA	GG
AG	AG	AG	AA	AA	GG	GG	AG
AA	AA	AG	AG	AA	AA	AA	AG
AG	AG	GG	GG	AG	AA	AG	AA
AA	AG	AG	AG	GG	AG	GG	
46932523							
6102	rs1016839	2	AG	GG	GG	AG	AG

AG AA GG AG GG AG AG AG
GG GG GG AG AG AG AG GG
GG AG GG GG AG GG AG GG
AG AG AG AA GG AG AG
88770768
6103 rs1016842 21 AG GG AG GG GG
GG GG GG GG AA GG GG GG
GG GG GG AG AG GG AG GG
AG AA AA AA AG AG AG AG
AG GG AA AG AA AA AG
39535579
6104 rs10168424 2 GG GG GG AG GG
GG AG AA AG AA GG GG GG
AG AG GG GG GG AG AG AA
AG AG AG AG GG AG AG GG
GG GG AG AG GG GG GG
2720083
6105 rs1016855 6 GG AG AG GG GG
GG GG GG AG GG AG AG AA
AA AA GG GG GG AG AG AG
AG AG AG AA AG AG AG AG
GG AG AG AG GG AG GG
25277419
6106 rs10168551 2 AG AG GG AA AA
AA AA AG AG AG AA AA AA
AA AA AA AA AA AG AA AA
AA AA AA AA AG AA AA AG
AA AG AA AA AA AA AA
168597986
6107 rs10168570 2 AG AG AG AG AA
AG AG AG AG AG GG GG GG
GG GG AA AA AG GG GG AG
AG AG AG GG AG GG GG GG
GG AG AG AG GG AG GG
222219604
6108 rs1016858 2 AG AA AA AG AA
AA AG AA AA AA AG AG AG
AA AA AA AG AG AG AG AA
AA AA AA AA AA AA AA AA
AA AG AG GG AA AA AA
88750032
6109 rs10168607 2 AA AG AG AA AG
AG AA AG AG AA AA AG AG
AG AG GG AG AG GG AG AG
AG AA AA AG AG AG GG AA
AA AA AA AA AA AG AA
38692619
6110 rs10168628 2 GG AG GG AG AG
AG GG AG AA AA GG GG GG
GG GG AG AG AG AG GG AG

AG AG AG AG GG AA AG AG
AG AG GG AG GG GG GG
237841705
6111 rs10168635 2 AA AA AA AA --
AA AA AA AA AA AA AA AA
AA AA AG AG -- AG AG AA
AA AA -- AA AA AA AA AA
AA AA AA AA AA AA AA
86389421
6112 rs10168688 2 CC AC CC CC AC
CC AC CC CC CC CC CC CC
CC CC CC CC CC CC CC CC
CC CC CC CC CC CC CC CC
CC CC AC AC CC CC CC
235703159
6113 rs10168752 2 AA AA AA AA AA
AA AA AA AA AA AA AA AA
AA AA AA AG AG AA AA AA
AA AA AA AA AA AA AA AA
AA AA AA AA AA AA AA
176283913
6114 rs10168758 2 AG AG AG GG GG
GG GG GG GG GG GG AG GG
GG GG GG GG GG GG GG GG
GG GG AG GG GG GG GG GG
GG GG GG GG AG AG GG
11188257
6115 rs10168801 2 GG AG GG GG GG
GG AG AG GG GG AA AA AA
AG AA GG AG AG AG GG AG
AA AA AA AG AG AA AG AG
AG GG AA AG AG AA GG
218580550
6116 rs10168803 2 AG GG GG GG GG
GG AG AG AG AG AA AG AA
AG AG GG AG GG AG AG AG
GG GG GG GG GG AG GG AG
GG GG AG AG AG AG AG
72319253
6117 rs1016883 2 GG GG GG GG GG
GG GG AG AG AG AG GG GG
GG GG AG AG AG AA GG AG
AG AG AG GG GG AG GG AG
GG AG GG GG GG GG GG
198881668
6118 rs10168890 2 AA AG AA AG GG
AG GG AG GG AG AA AA AA
AA AA GG AG GG GG AG GG
AG AG AG AG GG AA AG AG
AG GG AA AG AA AG AG

8003686							
6119	rs1016895	22	AC	AC	CC	AA	AC
	AC	AA	AA	AC	AA	AC	AC	AA
	AA	AA	AC	AA	AA	AA	AA	AA
	AA	AA	AC	AA	AA	AA	AA	AA
	AC	AA	AA	AA	AA	AA	AA	
27746118								
6120	rs10168954	2	CC	CC	AC	AC	AC	
	AA	CC	CC	CC	CC	CC	CC	AC
	AC	AC	CC	AC	CC	AC	AC	CC
	AC	AC	AC	AC	CC	AC	AC	CC
	AC	CC	AC	AC	AC	AC	AA	
43151394								
6121	rs10168963	2	GG	GG	GG	GG	GG	
	GG	GG	GG	GG	GG	GG	GG	GG
	GG	GG	GG	GG	GG	GG	GG	AG
	GG	GG	GG	GG	GG	AG	GG	GG
	AG	GG	GG	GG	GG	GG	GG	
16729410								
6122	rs10169073	2	AG	AG	AG	GG	AG	
	GG	AG	GG	GG	GG	GG	GG	GG
	GG	GG	GG	AG	GG	GG	GG	AG
	AG	AG	AG	AG	AG	AG	AG	AG
	GG	AG	AG	GG	GG	GG	GG	
237439358								
6123	rs10169099	2	AC	AA	AA	AA	AA	
	AA	AA	AA	AA	AA	AA	AA	AA
	AA	AA	AA	AA	AA	AA	AA	AA
	AC	AA	AC	AC	AC	AC	CC	AA
	AA	AA	AA	AA	AA	AA	AA	
88315954								
6124	rs10169105	2	AA	AA	AA	AG	AG	
	AA	GG	AG	AA	AA	AA	AA	AA
	AA	AA	AA	AA	AA	AA	AA	AA
	AG	AG	AG	AG	AA	AA	AA	AA
	AG	AG	AA	AA	AG	AG	AA	
237805870								
6125	rs10169121	2	CC	CC	AC	CC	AA	
	AC	AC	CC	CC	CC	CC	CC	AC
	CC	CC	CC	AC	AC	AC	AC	CC
	AC	AC	CC	AC	AC	CC	AC	CC
	CC	AC	CC	CC	AC	AC	AC	
35068284								
6126	rs10169175	2	GG	GG	GG	GG	GG	
	GG	GG	GG	GG	GG	GG	GG	GG
	GG	GG	GG	GG	GG	GG	GG	GG
	GG	GG	GG	GG	GG	GG	GG	GG
	GG	AG	GG	GG	GG	GG	GG	
88521610								
6127	rs10169238	2	GG	AG	AG	AG	AG	

	GG	AA	AA	AA	AA	AA	AG	AG
	AG	AG	AA	AA	AA	AG	AG	AG
	AG	AG	AG	AG	AA	GG	AG	AG
	AG	AG	GG	AG	AG	AG	AA	
36020401								
6128	rs10169258		2	AG	AG	AG	AG	AG
	AA	GG	AA	AG	AA	AG	AG	AG
	AG	GG	GG	AG	AG	AG	AG	AG
	AG	AG	AG	AG	AG	AG	AG	AA
	GG	AG	AA	AA	GG	AG	AG	
16764536								
6129	rs10169262		2	GG	AG	GG	GG	GG
	GG	GG	AG	AA	AG	GG	AG	GG
	GG	GG	AG	GG	GG	GG	GG	GG
	GG	GG	GG	GG	AG	AG	AG	AG
	GG	GG	GG	GG	GG	GG	GG	
9549753								
6130	rs1016927		1	GG	GG	GG	GG	GG
	GG	GG	GG	GG	GG	GG	GG	GG
	GG	GG	GG	GG	GG	GG	GG	GG
	GG	GG	GG	GG	GG	GG	GG	GG
	GG	GG	GG	GG	GG	GG	GG	
153128548								
6131	rs10169401		2	AC	AA	AA	AA	AA
	AA	AA	AA	AA	AA	AA	AC	AA
	AA	AA	AC	AC	AA	AA	AA	AA
	AA	AC	AA	AC	AA	AA	AA	AA
	AA	AA	AA	AA	AA	AA	AA	
76625113								
6132	rs10169419		2	GG	GG	GG	GG	GG
	GG	GG	GG	GG	GG	GG	GG	GG
	GG	GG	GG	GG	GG	GG	GG	GG
	GG	GG	GG	GG	GG	GG	GG	GG
	GG	GG	GG	GG	GG	GG	GG	
98647924								
6133	rs10169431		2	GG	AG	AG	AG	AG
	AA	GG	AG	AG	AG	GG	GG	AG
	AG	AG	AG	AA	AG	GG	GG	GG
	AA	AA	AA	AA	AG	GG	AG	GG
	AA	GG	GG	GG	AA	AG	AA	
47823949								
6134	rs10169437		2	--	--	--	--	--
	--	--	GG	--	GG	--	GG	--
	--	--	--	--	--	--	--	--
	--	--	--	GG	--	GG	--	--
	--	--	--	--	--	--	GG	
193363722								
6135	rs10169441		2	AG	AG	AG	AA	AA
	AA	AA	AA	AA	AA	AG	AA	AA
	AA	AA	AG	AG	AA	AA	AA	AA

	AA	AA	AA	AA	AA	AG	AG	AA
	AG	AG	AG	AA	AA	AA	AA	
98740085								
6136	rs10169464	2	AG	AG	AG	GG	AA
	AG	AG	GG	GG	GG	AG	GG	GG
	GG	GG	AG	AA	AG	AA	AA	AG
	AG	AG	AG	AG	AA	GG	AG	AG
	AG	AA	AG	AG	AG	AG	AG	
69147960								
6137	rs10169497	2	AA	AA	AA	AG	AA
	AA	AG	AG	AG	AG	AG	AA	AA
	AG	AG	AA	AA	AA	AG	AG	AG
	AA	AG	AG	AG	AA	AG	AG	AA
	AA	AG	AA	AA	AG	AA	AG	
105776156								
6138	rs10169506	2	GG	GG	GG	GG	GG
	GG	GG	AG	GG	GG	GG	GG	GG
	GG	GG	GG	GG	AG	AG	AG	AA
	AG	GG	GG	AG	GG	AG	GG	GG
	GG	AG	AG	AA	AG	GG	AG	
168896548								
6139	rs10169662	2	GG	AG	AA	AA	AA
	AA	AA	AG	AG	AG	AA	AA	AA
	GG	GG	AG	AG	AG	AG	AG	GG
	AA	AG	AA	AG	GG	AA	AG	AG
	GG	GG	GG	GG	AA	AA	AG	
61507936								
6140	rs10169682	2	GG	AG	GG	GG	AG
	GG	AG	AG	AA	AG	AG	GG	GG
	AG	AG	AG	AG	AG	GG	GG	AG
	GG	GG	AG	GG	GG	AA	AG	AG
	GG	GG	GG	GG	AG	AG	AA	
3086833								
6141	rs1016973	7	AG	AA	AG	AA	AA
	AA	AA	AA	AA	AA	AG	AG	AA
	AA	AA	AG	AG	AG	GG	AG	GG
	AG	GG	AG	AG	GG	AA	AG	AG
	AG	AG	--	AG	AA	AA	AA	
126063696								
6142	rs10169752	2	GG	AG	GG	AG	GG
	AG	AG	GG	AG	AG	GG	GG	GG
	GG	GG	GG	GG	AG	AG	AG	AG
	GG	GG	GG	AG	AG	AG	AG	AA
	GG	AG	GG	AG	AG	GG	AG	
233872049								
6143	rs10169757	2	AG	AA	AG	AG	GG
	AG	AG	AA	AA	AA	AA	AG	AG
	AG	AG	AA	AG	AA	AA	AG	AA
	AA	AA	AA	AG	AG	AG	GG	AG
	AA	AA	AA	AA	AA	AA	AA	

228127905
6144 rs10169797 2 AA AA AA AA AA
AA AA AA AA AA AA AA AA
AA AA AA AA AA AA AA AA
AA AA AA AA AA AA AA AA
AA AA AA AA AA AA AA
113038409
6145 rs10169879 2 AG GG AG GG GG
GG GG AA AG AG GG GG GG
AG AG AA AG AG GG GG AG
GG GG GG GG GG GG GG GG
GG GG AA AG GG AG GG
170039594
6146 rs1016988 5 AG AA AG AA AA
AG AA AA AG AG AA AG AA
AG AG GG AG AG AA AG AG
AG AG AG AG AA AG AG AA
GG AG AG GG AA AG AA
131744574
6147 rs10169925 2 GG GG GG AG GG
GG AG AG AG AG GG GG GG
AG AG GG GG GG AG AG AA
AG AG GG GG AG AG AG AG
AA AG GG GG AG GG AG
113014605
6148 rs10169963 2 AG AG AG AG AG
GG AG AG AG AG GG GG GG
AG AG GG GG AG AG AG AG
AG AG GG AG AA AG AG AG
AG AG AG AA AG AG AG
12652931
6149 rs10170127 2 AC AC AA CC AA
AC AC AC AA AA CC CC CC
AA AA AA AC AA AA AA AA
AA AC AC AC AC AA AC AA
CC AC AA AC AA AC AA
51729681
6150 rs10170138 2 GG GG GG GG GG
GG GG GG GG GG GG GG GG
GG GG AG AG GG AG AG AG
GG GG GG GG AG AG AG AG
GG AG GG GG GG GG GG
77021928
6151 rs10170192 2 GG AG GG GG GG
GG GG GG GG GG GG GG GG
GG GG GG GG GG GG GG GG
GG GG GG GG GG GG GG GG
GG GG GG GG GG GG GG
14480276
6152 rs10170218 2 AA AA AA AA AA

	AA	AA	AA	AA	AA	AA	AC	AA
	AA	AA	AC	AA	AA	AC	AC	AC
	AA	AA	AA	AA	AA	CC	AC	AC
	AC	AA	AC	AC	AA	AA	AA	
188814444								
6153	rs10170284		2	AG	AA	AG	AA	AA
	AA	AA	AG	AG	AG	AA	AG	AA
	AA	AA	AA	AG	AG	AG	AG	AG
	AG	AG	AG	AA	AA	AA	AA	AA
	AA	AA	AA	AA	AA	AG	AG	
217446590								
6154	rs10170288		2	AG	AG	GG	AA	AA
	AA	AA	AG	AA	AA	AA	AA	AA
	GG	GG	AA	AG	AA	AG	AG	AG
	AG	AG	GG	GG	AG	AG	AG	AG
	AG	AG	AG	AG	AG	AG	AG	
49640979								
6155	rs10170310		2	AA	AA	AA	AG	AG
	AA	AG	AG	GG	AG	AA	AA	AA
	AG	AG	GG	AG	AG	AG	AA	AG
	AA	AA	AA	AA	AA	AG	AG	AG
	AA	AG	AA	AA	AA	AA	AA	
139278922								
6156	rs1017036		7	CC	CC	CC	CC	CC
	CC	CC	CC	CC	AC	CC	CC	CC
	AC	AC	AC	AC	AC	CC	AC	AC
	CC	CC	AC	AC	CC	CC	CC	CC
	AC	CC	CC	CC	CC	CC	AC	
77080061								
6157	rs10170466		2	CC	AC	CC	AA	AA
	AC	AA	CC	CC	CC	CC	AC	CC
	AC	CC	AC	CC	AC	AA	AC	AA
	CC	CC	AC	AC	AC	AA	AC	AA
	AC	AC	CC	CC	AC	AC	AA	
119056234								
6158	rs10170500		2	AA	AA	AA	AA	AA
	AA	AG	AA	AA	AA	AG	AG	AG
	AA	AA	AG	AA	AG	AA	AG	AA
	AA	AA	AA	AA	AA	AA	AA	AA
	AG	AA	AG	AA	AA	AA	AA	
169009565								
6159	rs1017051		2	AG	AG	GG	GG	GG
	GG	GG	AG	AG	AG	GG	AG	GG
	GG	GG	GG	AG	AG	GG	GG	AG
	AG	AG	AG	AG	AG	GG	GG	AG
	AG	GG	AG	AG	AG	AG	AG	
75865277								
6160	rs10170645		2	GG	GG	GG	GG	AA
	AG	AG	AG	GG	AG	AG	AG	AG
	GG	GG	GG	GG	GG	AG	AG	GG

GG GG GG GG GG GG GG GG
GG GG GG GG AG AG GG
88392018
6161 rs10170745 2 AG AG AA AG GG
AG GG GG AG AG AG AG AA
AG AG AA AA AA AG AA GG
AG GG AG AG AG AA AA AG
AA AA AA AA AG AG AA
173040217
6162 rs1017077 17 AC AC CC CC CC
CC CC AC AC AC CC AC CC
CC CC CC CC AC AC CC AC
AC AC CC CC AC CC AC AC
AA CC CC CC CC CC CC
14005072
6163 rs10170771 2 AA AG AA GG AA
AG AG AG AA AG AG AA AA
AA AA AG AA GG AA AA AG
AA AA AA AA GG AG GG GG
AA AG AA AA AG AA AG
20883216
6164 rs10170836 2 GG GG GG AG AG
AA GG GG AG AG GG AG AG
AG AG GG AG GG AA AG AA
AG AG AG AG AG AG AA AG
AG AA AA AA AG AG GG
225259356
6165 rs10170867 2 GG GG GG GG GG
GG GG GG GG GG GG GG GG
GG GG GG GG GG GG GG GG
GG GG GG GG GG GG GG GG
GG GG AG GG GG GG GG
141544431
6166 rs10170902 2 AG AG AA AA AG
AG AA AA AA AA AA AA AA
AA AA AA AA AA AA AG AA
AG AA AG AA AA AG AG AA
AA AA AG AA AA AA AA
170066702
6167 rs10170918 2 AA AA AG AA AA
AA AA AA AA AA AA AA AA
AA AA AA AA AA AG AG AG
AA AG AA AA AG AA AA AG
AA AA AA AA AG AA AG
80266897
6168 rs10170933 2 AA AA AA AA AA
AA AA AA AA AA AA AG AA
AA AA AA AA AA AA AA AA
AA AA AA AA AG AA AA AG
AA AA AG AA AG AA AG

35947071							
6169	rs1017095	17	AA	AA	AA	AA	AA
	AA	AA	AA	AA	AA	AA	AA
	AA	AA	AG	AG	AG	AA	AA
	AA	AA	AA	AA	AG	AA	AA
	AG	AG	AA	AA	AA	AA	AA
16419756							
6170	rs1017099	17	CC	CC	CC	CC	AC
	AC	AC	CC	CC	CC	AC	CC
	CC	CC	AC	AC	AC	CC	CC
	CC	AA	AC	AC	AC	AC	CC
	AA	CC	CC	CC	CC	CC	CC
52879063							
6171	rs10171011	2	AG	GG	AG	AG	AG
	AA	AG	AG	AG	AG	AG	AG
	AG	AG	GG	AG	AG	AA	AA
	GG	AG	GG	GG	AG	AG	AA
	AG	AG	AA	AG	AA	AA	AA
123910170							
6172	rs1017102	19	AA	AC	AC	AA	AA
	AA	AA	AC	AC	AC	AA	AC
	AC	AC	AA	AA	AA	AA	AA
	AC	AC	AC	AC	AA	AA	AA
	AA	AA	AA	AA	AA	AA	AA
56892632							
6173	rs1017103	19	AG	AA	AG	GG	GG
	AG	GG	GG	GG	GG	AG	AG
	AA	AA	AG	AG	AG	GG	AG
	AA	AG	AA	AA	GG	GG	GG
	AA	GG	GG	GG	AG	AG	AA
2822045							
6174	rs1017104	7	AA	AA	AA	AG	AA
	AA	AG	AA	AA	AA	AA	AA
	AA	AA	GG	AG	AG	AA	AA
	AA	AA	AA	AA	AG	AG	AA
	AG	AA	AA	AA	AG	AA	GG
90043182							
6175	rs10171051	2	AC	CC	AC	CC	CC
	AC	CC	CC	CC	CC	AC	AA
	AC	AC	CC	CC	CC	CC	AC
	AC	AC	AC	AC	CC	AA	AC
	CC	CC	CC	CC	AC	CC	AC
230031082							
6176	rs10171076	2	AG	AG	GG	AG	GG
	AG	GG	AG	AA	AA	AA	AA
	AA	AA	AG	GG	AG	AA	AA
	AA	AA	AA	AA	AG	AA	AG
	AA	GG	AG	AG	AG	AA	AG
69081746							
6177	rs10171102	2	AA	AA	AA	AA	AA

AG AA AA AA AA AA AG AA
AA AA AA AA AG AA AA AG
AA AA AA AA AA AA AA AA
AG AA AG AA AA AA AG
221201418
6178 rs10171121 2 AA AG AA AG AG
AG AG AA AA AA AG AG AG
GG GG AG GG GG AA AG AA
AA AA AA AA AG AG AG AA
AA AA AG AA GG GG GG
150843084
6179 rs10171160 2 GG GG GG GG GG
GG GG GG GG GG GG GG GG
GG GG GG GG GG GG GG GG
GG GG GG GG GG GG GG GG
GG GG GG GG GG GG GG
113709004
6180 rs10171167 2 AC AC AC AA AA
AC AA AC AC AA AC AC CC
AC AC AA AA AA AC AC AC
AA AA AC AC AA AC AC AA
AA AA AC AA AA AA AA
110256233
6181 rs10171235 2 AA AA AA AA AA
AA AA AG AG AG AA AA AA
AA AA AG AA AA AA AA AA
AG AG AG AA AA AA AA AA
AA AA AA AA AA AA AA
15232825
6182 rs10171250 2 AG AG AG AA AA
AA AA AG AA AA AA AA AA
AA AA GG AG AG AG AA AG
AG AG AG AG AG AA AG AG
AA AA AA AA AA AA AA
226281773
6183 rs10171282 2 AG AG AG AA AA
AG AA AG AG AA AG AG GG
AG AG GG GG GG AG AG AG
AA AA AG AG AG AG AG AG
AA AA AG AA AA AA AG
110256391
6184 rs10171287 2 AG AG AG GG GG
GG GG GG AG GG GG GG GG
GG GG AG AG GG GG GG AG
AG AG AG AG GG GG GG GG
GG AG GG AG GG GG GG
150729453
6185 rs1017129 11 GG GG GG GG GG
GG GG GG GG GG GG GG GG
GG GG GG GG GG GG GG GG

GG GG GG GG GG GG GG GG
GG GG GG GG GG GG GG
17162545
6186 rs10171294 2 CC AC CC CC CC
CC CC CC CC CC CC CC CC
CC CC AC CC AC CC CC CC
AC AC CC CC CC CC CC CC
AC AC CC AC CC CC CC
123878592
6187 rs10171391 2 AA AA AA AA AA
AA AA AA AA AA AA AA AA
AA AA AA AA AA AA AA AA
AA AA AA AA AA AA AA AA
AA AA AA AA AA AA AA
102488684
6188 rs10171590 2 AG AA AG AA AG
AA AG AG AG AG GG GG GG
AG AG AG AA AA AG AG GG
AA GG AG AA AA AG AA AA
GG AG AA AG AG AA GG
14505837
6189 rs10171634 2 GG GG GG GG GG
GG GG GG GG GG GG GG GG
GG GG GG GG GG GG GG GG
GG GG GG GG GG AG GG AG
GG GG AG GG GG GG GG
88953736
6190 rs10171637 2 AA AG AA GG AG
AG GG AG AG AG AG AA AA
AG AA AA AG AA AG AG AG
GG GG GG GG AG GG AG GG
GG AG AG AG AA AG AA
19916997
6191 rs10171646 2 AA AA AA AA AA
AA AA AA AA AA AA AA AA
AA AA AG AG AA AA AA AA
AA AA AA AA AG AA AA AG
AA AA AA AA AA AA AA
110977357
6192 rs10171661 2 GG AG AG AG GG
AG AG AA AG AA AA AG AA
AA AA AA AA AA AA AA AG
AG GG AG GG AG AG GG AA
AA AG GG GG GG GG AG
77659859
6193 rs10171675 2 AA AA AA AA AA
AA AA AA AA AA AA AA AA
AA AA AA AA AA AA AA AA
AA AA AA AA AA AA AA AA
AA AA AA AA AA AA AA

58257842
6194 rs10171678 2 AA AA AA AG AG
GG AG GG AG AG GG AG GG
GG AG GG AG AG AG GG AG
GG GG AG AG AG AG AG AA
GG GG AG AG AA AA AG
205154872
6195 rs10171740 2 CC CC CC CC CC
CC CC CC CC CC CC CC CC
CC CC CC CC CC CC CC CC
CC CC CC CC CC CC CC CC
CC CC CC CC CC CC CC
97877591
6196 rs10171921 2 CC CC CC CC CC
CC CC AC AC CC CC CC CC
CC CC CC CC CC AC CC CC
CC CC CC CC CC CC CC CC
CC CC CC CC CC CC CC
238870937
6197 rs10171943 2 GG GG GG GG GG
GG GG GG GG GG GG GG GG
GG GG GG GG GG GG GG GG
GG GG GG GG GG GG GG GG
GG GG GG GG GG GG GG
238576689
6198 rs10171980 2 AG GG AG GG GG
GG GG GG AG AG GG GG GG
GG GG GG GG GG GG GG GG
GG GG GG GG GG GG GG GG
AG AG GG GG GG GG GG
174495916
6199 rs10172020 2 AG GG GG AG GG
AG GG GG GG GG GG GG GG
GG GG GG GG GG GG GG GG
GG GG GG GG GG GG GG GG
GG GG GG GG GG GG GG
56555581
6200 rs10172149 2 AA AG AA AG AG
AG AG AA AA AA AG AG AG
GG GG AG GG GG AA AG AA
AA AA AA AA AG AG AG AA
AA AA AG AA GG GG GG
150814234
6201 rs1017219 19 AA AA AA AG AG
AA AG AG AG AG AG AA AA
AG GG AG AG AG AG AG AG
AA AA AA AA AG AG AG AA
AA AG AA AG AG AG AA
49573438
6202 rs10172196 2 GG GG GG AG GG

	AG	GG	GG	GG	GG	GG	GG	GG
	GG	GG	GG	GG	GG	AG	GG	AA
	GG	GG	GG	GG	GG	GG	GG	GG
	AA	GG	GG	GG	AG	GG	AA	
36780549								
6203	rs10172290		2	AA	AG	AA	AA	AA
	AA	AA	AA	AA	AA	AA	AA	AA
	AA	AA	AA	AA	AA	AA	AA	AA
	AA	AA	AA	AA	AA	AA	AA	AA
	AA	AA	AA	AA	AG	AA	AG	
184203775								
6204	rs1017234		16	AG	AG	GG	AG	AG
	AA	GG	AG	AG	AA	AA	AA	AA
	AG	AA	GG	AG	AG	AA	AG	AA
	GG	AG	GG	GG	AG	AG	AA	GG
	AG	AA	AG	AA	GG	AG	AG	
83705819								
6205	rs10172452		2	GG	GG	GG	GG	GG
	GG	GG	AA	AG	AA	AG	AG	AG
	AG	AG	GG	GG	GG	GG	GG	GG
	GG	GG	GG	GG	GG	GG	GG	GG
	GG	GG	AG	GG	AA	AG	AG	
142942925								
6206	rs10172508		2	CC	CC	CC	CC	CC
	CC	CC	CC	CC	CC	CC	CC	CC
	CC	CC	CC	CC	CC	CC	CC	CC
	CC	CC	CC	CC	CC	CC	CC	CC
	CC	CC	AC	AC	AC	CC	AC	
145220327								
6207	rs10172567		2	GG	GG	GG	GG	GG
	GG	GG	GG	GG	GG	GG	GG	GG
	GG	GG	GG	GG	GG	GG	GG	GG
	GG	GG	GG	GG	GG	GG	GG	GG
	GG	GG	GG	GG	GG	GG	GG	
88397583								
6208	rs10172591		2	AA	AA	AA	AA	AA
	AA	AA	AA	AA	AA	AA	AA	AA
	AA	AA	AA	AA	AA	AA	AA	AA
	AA	AA	AA	AA	AA	AA	AA	AA
	AA	AA	AA	AA	AA	AA	AA	
1354088								
6209	rs10172595		2	GG	AG	GG	AG	AA
	AG	AG	AG	AA	AG	AA	AA	AA
	AA	AA	AG	GG	AG	GG	AG	GG
	AG	AG	AG	AG	AG	AA	AG	AG
	GG	GG	AG	AG	AA	AA	AG	
232387063								
6210	rs10172659		2	GG	GG	GG	GG	GG
	GG	GG	GG	GG	GG	GG	GG	GG
	GG	GG	GG	GG	GG	GG	GG	GG

GG GG GG GG GG GG GG GG
GG GG GG GG GG GG GG
61994215
6211 rs10172674 2 GG GG GG GG GG
GG GG GG GG GG AA AA AA
AG AG AG AG GG GG GG GG
GG GG GG GG AG GG AG AG
GG GG AG GG GG GG GG
14298244
6212 rs1017272 2 GG GG GG GG AG
GG AG GG GG GG AA AA AA
AG AG GG GG AG AG AG AA
GG AG GG AG AG AG GG AG
AG AA AA AA GG GG GG
56696778
6213 rs10172735 2 AA AA AA AA AA
AA AA AA AA AA AA AA AA
AA AA AA AG AA AA AA AA
AA AG AG AG AA AA AA AA
AA AA AA AA AA AA AA
221426974
6214 rs1017280 4 GG AG AG AA AG
AG AG GG GG GG GG AG GG
GG AG GG GG GG AG AG GG
GG AG AG AA AG AG AG AA
AG GG AG AG AG GG AG
55011123
6215 rs10172809 2 GG GG GG AG AG
AG GG GG GG GG GG GG GG
GG GG GG GG GG GG GG GG
GG GG GG GG GG GG GG GG
GG GG GG GG GG GG GG
145991467
6216 rs10172811 2 AA AA AA AA AA
AA AA AA AA AA AA AA AA
AA AA AA AA AA AA AA AA
AA AA AA AA AA AA AA AA
AA AA AA AA AA AA AA
167236815
6217 rs10172841 2 AA AA AA AA AA
AA AA AA AA AA AG AA AA
AA AA AA AA AA AA AA AA
AG AA AG AG AA AG AA AG
AA AA AA AA AA AA AA
101025811
6218 rs10172930 2 AA AA AA AA AA
AA AA AA AA AA AA AA AA
AA AA AA AA -- AA -- AA
-- -- AA AA AA AA AA AA
AA AA -- AA AA AA AA

39887548							
6219	rs10172996	2	AA	AG	AA	AA	AG
AG	AG	AG	GG	AG	GG	GG	GG
GG	GG	GG	GG	GG	AG	AG	GG
GG	GG	GG	GG	GG	GG	GG	GG
GG	GG	GG	GG	AG	GG	AG	
180137861							
6220	rs1017301	12	CC	AC	AC	AC	AC
CC	AC	CC	AC	AC	AA	AA	AC
AA	AC	AC	AC	AC	AC	AC	AC
AC	AC	AA	AC	AA	AA	AA	AA
CC	AA	AC	AC	AC	CC	AC	
9362931							
6221	rs10173022	2	GG	GG	GG	GG	GG
GG	GG	GG	GG	GG	GG	GG	GG
GG	GG	GG	GG	GG	GG	GG	GG
GG	GG	GG	GG	GG	GG	GG	GG
GG	GG	GG	GG	GG	GG	GG	
197589824							
6222	rs1017307	5	GG	GG	GG	GG	GG
GG	GG	GG	GG	GG	GG	GG	GG
GG	GG	GG	AG	AG	GG	AG	AG
AG	AG	GG	GG	AG	AG	GG	AG
AA	AG	AG	AG	AA	AG	AG	
132578203							
6223	rs10173073	2	AG	AG	GG	GG	GG
GG	AG	AG	AG	AG	GG	GG	GG
AG	AG	AG	AG	AA	AA	AA	AG
AG	AA	AG	AG	GG	AG	GG	AG
GG	GG	AG	AG	AG	AG	AG	
80189383							
6224	rs10173092	2	AC	AC	AC	CC	AC
AC	CC	CC	CC	CC	CC	CC	AC
AA	AA	CC	CC	CC	CC	CC	AC
AA	CC	AA	AC	CC	AA	AC	AC
CC	AC	AC	CC	AC	AC	CC	
174321840							
6225	rs10173102	2	GG	GG	GG	AG	AG
AA	AG	GG	GG	GG	AA	AG	AG
AG	AG	GG	GG	GG	AG	GG	AG
GG	GG	GG	GG	GG	GG	GG	GG
GG	GG	GG	GG	GG	GG	GG	
146736345							
6226	rs10173144	2	GG	AG	AG	GG	GG
--	GG	GG	GG	GG	GG	AG	GG
GG	GG	GG	GG	GG	GG	GG	GG
GG	GG	GG	GG	GG	AG	AG	GG
--	GG	GG	GG	AG	AG	GG	
45842844							
6227	rs10173180	2	AG	AG	AG	AA	AA

AG AA AA AA AA AA AA AA
AA AA AA AA AA AA AA AA
AA AA AA AA AA AA AA AA
AA AA AA AA AA AA AA
113664817
6228 rs10173228 2 GG GG GG AG AG
AG GG AG GG AG AG AA AA
GG GG GG AG AG AG GG GG
AG GG GG GG GG AG AG GG
AG AG AG AG AG AG AG
133167263
6229 rs10173297 2 AC AC AC -- --
-- -- -- -- -- -- -- --
-- -- -- AC -- AC AC AC
AC AC AC -- AC -- AC --
-- AC -- -- AC AC AC
3714129
6230 rs10173398 2 AG GG AG GG GG
GG AG GG AG AG AG AA AG
GG GG GG GG AG AG GG AA
GG GG GG GG GG AG GG GG
GG GG AG GG GG GG GG
153354994
6231 rs10173406 2 AA AA AA AG AA
AG AG AG AA AA GG AA AG
AG AG AG AA AG AA AA AA
AG GG GG GG AG AG AG GG
AA GG AG AG GG GG AG
224360395
6232 rs1017342 8 AG AG AG AG GG
GG AG AG AG GG AG AG AA
AG GG AG GG AG AG GG AG
AG AG AG AG GG GG GG GG
GG AG GG AG GG GG GG
125713791
6233 rs10173479 2 GG AG AG AA AG
AA AG AG AG AG GG GG GG
AA AA AA AA AA AA AA AA
AA AA AA AA AA AA AA AA
GG AA AA AA AA AA AA
52948828
6234 rs10173487 2 GG AG AG AA AG
AG AA GG AG AG AG AG AG
AG AG GG AG AG GG GG GG
AG GG AG GG AG AG AG AG
AG AG GG GG AG AG GG
173229472
6235 rs10173491 2 AA AA AA AC AA
AC AA AC AC AC AA AA AA
AA AA AA AA AA AA AA AA

AC CC AC AA AC AC CC AC
AA AA AC AC AC CC AC
15451774
6236 rs10173564 2 CC CC CC CC AC
AC CC CC CC CC CC CC CC
CC CC CC CC CC AA CC AA
AC AC AC CC CC CC CC CC
CC CC CC CC CC CC CC
193335578
6237 rs10173571 2 AA AA AA AA AC
AC AA AA AA AA AA AA AA
AA AA AA AA AA AC AC AC
AA AC AA AA AA AA AA AA
AA AC AA AA AA AA AA
83857943
6238 rs10173591 2 GG AG AG GG GG
GG GG AG AA AG AG GG AG
GG GG GG GG GG AG AG AG
AG GG AG AG AG GG AG GG
GG GG AA AG GG GG AG
78332806
6239 rs10173620 2 AA AA AA AA AA
AA AA AA AA AA AA AA AA
AA AA AA AA AA AA AA AA
AA AA AA AA AA AA AA AA
AA AA AA AA AA AA AA
101743983
6240 rs10173657 2 CC CC CC CC CC
CC CC CC CC CC CC CC CC
CC CC CC CC CC CC CC CC
CC CC CC CC CC CC CC CC
CC CC CC CC CC CC CC
39699141
6241 rs10173687 2 GG GG GG GG AG
AG GG AG AG GG GG GG GG
AG AG GG GG AG AG AG AG
GG GG GG GG AG AG GG AA
AG GG GG GG AA AG AG
44036438
6242 rs10173738 2 AA AA AA AA AA
AA AA AG AA AA AA AA AA
AA AA AA AA AA GG AA AG
AA AA AG AG GG AG GG AG
AA AA AA AA AA AA AA
198988387
6243 rs10173769 2 GG GG GG AG AG
AA GG AA GG AG GG GG AG
AA AA AG AG AG GG GG GG
AG AA AG AG GG GG GG GG
GG AG AG AA GG AG GG

181807231
6244 rs10173792 2 AA AC AC AC AC
AC AC AA AA AA AC CC CC
AA AA AC AC AA AC AC AA
CC CC CC CC AC AA AC AC
AA AA AC AA CC CC CC
52897938
6245 rs10173794 2 AG GG AG AA AG
AA AG AA AA AA AG AA AA
AA AA AA AA AG AA AA AG
AA AA AA AA AA AA AA AA
AA AA AG AA AA AG AA
37629222
6246 rs10173806 2 GG GG GG AA AA
AA AA AA AA AA GG AG AG
GG GG AG AG AG AG AG AG
AG GG GG AG GG GG GG GG
AA AA AA AA AG GG AA
142238800
6247 rs10173816 2 AG AG GG AG GG
GG AG GG GG GG GG GG GG
AG AG GG GG GG GG GG GG
GG GG GG GG AA GG AG AG
AG AG GG GG GG GG AG
37808868
6248 rs10173922 2 AA AA AA AG GG
AG GG AG AG AG AG AG AG
AA AG GG GG GG GG GG AG
AA AA AG AA AG AA AA AG
AG AG AG GG AA AA AG
18924471
6249 rs10173947 2 GG GG GG GG GG
GG GG GG GG GG GG GG GG
GG GG AG AG AG GG GG GG
GG GG GG GG AG GG AG GG
GG GG GG GG GG GG GG
151150611
6250 rs10174031 2 GG GG GG GG GG
GG GG GG GG GG GG GG GG
GG GG GG GG GG GG GG GG
GG GG GG GG GG GG GG GG
GG GG GG GG GG GG GG
172137732
6251 rs10174055 2 GG GG GG GG GG
GG GG GG GG GG GG GG GG
GG GG GG GG GG AA AG AA
GG GG GG GG GG GG GG GG
GG GG AG AG AG GG AG
172858214
6252 rs10174088 2 AG GG AG AG AG

	GG	AG	GG	GG	GG	AA	AG	AG
	AG	AG	GG	GG	AG	AG	AG	AG
	GG	GG	GG	GG	GG	AG	AG	AG
	GG	GG	AG	GG	GG	AG	AG	
140923936								
6253	rs1017414		18	AC	CC	AC	AC	AC
	AC	CC	CC	CC	CC	AC	AA	AA
	AA	AC	AC	AC	AA	CC	AC	CC
	CC	CC	CC	CC	AC	CC	CC	AC
	AC	CC	CC	CC	AA	AA	AA	
24405600								
6254	rs10174149		2	AA	AA	AA	AG	AA
	AG	AG	AA	AG	AA	AA	AA	AA
	AA	AA	AG	AG	AG	AA	AG	AA
	AA	AA	AA	AA	AG	AA	AA	AA
	AA	AG	AA	AG	AG	AG	AA	
181059434								
6255	rs10174157		2	AA	AA	AA	GG	GG
	GG	GG	GG	GG	GG	GG	AG	AG
	AA	AA	AA	AA	AG	GG	AG	GG
	GG	AG	GG	AG	GG	AA	AG	AG
	GG	AG	AG	AG	AA	AG	AA	
158968794								
6256	rs1017419		2	AG	AA	AA	AA	AG
	AA	AG	AG	AG	AG	AA	AA	AA
	AA	AA	GG	AG	AG	AA	AA	AA
	AA	AG	AG	AG	AA	AG	AA	AG
	AA	AA	AA	AA	AA	AG	AA	
52048275								
6257	rs10174302		2	AA	AA	AA	AA	AA
	AA	AA	AA	AA	AA	AA	AA	AA
	AA	AA	AA	AA	AA	AA	AA	AA
	AA	AA	AA	AA	AA	AA	AA	AA
	AA	AA	AA	AA	AA	AA	AA	
146597420								
6258	rs1017432		4	AA	AA	AA	AA	AA
	AA	AA	AA	AA	AA	AA	AA	AA
	AA	AA	AG	AA	AA	GG	AG	AG
	AA	AG	AG	AG	AA	AG	AA	AG
	AA	AA	AG	AG	AA	AA	AG	
109474255								
6259	rs10174463		2	AG	AG	AG	AG	AA
	AA	AG	AG	AG	AG	AA	GG	AG
	AG	AG	AG	GG	GG	AG	GG	AG
	AA	AA	AA	AG	GG	AA	AG	AG
	AA	AG	AA	AA	AG	GG	AG	
222074201								
6260	rs10174698		2	GG	AG	AG	AG	AG
	GG	AA	AG	AG	GG	AG	GG	AG
	AA	AA	AA	AA	AA	AG	AG	AA

	AG	AG	GG	AG	AG	GG	GG	AG
	GG	AG	AG	AG	AG	AG	GG	
34404072								
6261	rs10174761		2	GG	GG	GG	AG	GG
	AG	GG	GG	GG	GG	AG	AA	AA
	GG	GG	GG	GG	GG	GG	GG	GG
	GG	GG	GG	GG	GG	GG	GG	GG
	GG	GG	GG	GG	AG	GG	AG	
61949300								
6262	rs10174774		2	GG	AG	GG	GG	GG
	GG	AG	AG	AG	GG	GG	GG	AG
	AA	AA	AG	AA	AA	GG	AG	GG
	AA	AG	AA	AG	AG	AG	GG	AG
	AA	AG	AA	AG	GG	AG	AG	
180096561								
6263	rs10174914		2	AG	AA	AG	AA	AA
	AA	AA	AA	AA	AA	AA	AA	AA
	AA	AA	AA	AG	AG	AG	AG	AG
	AA	AA	AA	AA	AA	AA	AA	AA
	AA	AA	AA	AA	AA	AA	AA	
181678814								
6264	rs10174932		2	AA	AA	AA	AA	AA
	AA	AA	AA	AA	AA	AA	AA	AA
	AA	AA	AA	AA	AA	AA	AA	AA
	AA	AA	AA	AA	AA	AA	AA	AA
	AA	AA	AA	AA	AA	AA	AA	
88380836								
6265	rs10174972		2	AG	GG	AG	AA	AA
	AA	AA	AA	AA	AA	AA	AA	AA
	AG	AA	AG	AG	AG	AG	AA	GG
	AA	AA	AA	AA	AA	AG	AA	AG
	AG	AG	AG	AG	AA	AA	AA	
216356666								
6266	rs1017502		3	GG	GG	GG	GG	GG
	GG	GG	GG	GG	GG	GG	GG	GG
	GG	AG	GG	GG	GG	GG	GG	GG
	GG	GG	GG	GG	GG	AG	AG	GG
	AG	GG	GG	GG	GG	AG	GG	
185151885								
6267	rs10175058		2	GG	GG	GG	GG	GG
	GG	GG	GG	AG	AG	AG	AG	AA
	AG	AG	GG	GG	GG	GG	AG	GG
	GG	AG	GG	AG	AG	GG	AG	GG
	AG	AG	AG	AG	AG	GG	AG	
162276981								
6268	rs10175075		2	AG	GG	AG	GG	GG
	GG	GG	GG	AA	AA	AG	AA	AA
	AA	AG	GG	AG	AG	AG	AG	AG
	AA	AA	AA	AA	AA	GG	AG	AG
	AA	AG	AG	GG	AG	AG	GG	

242486521								
6269	rs10175176		2	AG	AG	AA	AG	AG
	AA	AG	AA	AG	AG	AG	AG	AA
	AA	AA	AA	AA	AA	AA	AA	AA
	AA	AG	AG	AA	AA	AA	AA	AA
	AG	GG	AG	GG	AA	AA	AG	
160058776								
6270	rs10175190		2	AC	AC	CC	AC	AC
	AC	AA	AC	CC	AC	AA	AA	AC
	CC	CC	AA	AC	AA	AC	CC	AA
	CC	AC	AC	CC	AC	AC	CC	AC
	AA	CC	AC	CC	AA	AC	AC	
154944691								
6271	rs10175198		2	AG	AG	AG	AA	AG
	AA	AG	AG	AA	AA	AA	AG	AG
	AG	AG	AA	AG	AA	AA	AA	AA
	GG	AG	AG	GG	AA	AA	AA	AA
	GG	AG	AA	AG	GG	GG	AG	
46054114								
6272	rs1017520		15	AA	AC	AC	AA	AA
	AA	AC	AA	AA	AA	AC	AA	AA
	AA	AA	AC	AA	AA	AC	AC	AA
	AC	AC	AC	AC	AA	AA	AA	AA
	AA	AA	AA	AA	AA	AA	AA	
98901499								
6273	rs10175222		2	CC	AC	AC	AC	CC
	CC	AC	AC	AC	AC	AC	CC	CC
	AC	AC	AC	AA	AC	AA	AA	AC
	CC	CC	CC	CC	AC	CC	CC	AC
	CC	AC	CC	CC	AC	CC	AC	
50763645								
6274	rs10175330		2	AA	AC	CC	AC	CC
	CC	AC	AA	AA	AA	AC	AA	AC
	AA	AA	CC	AC	AC	AC	AC	AC
	AC	CC	AC	AC	AC	AC	AC	AC
	AC	CC	CC	CC	AA	AA	AA	
86414114								
6275	rs10175357		2	AA	AA	AA	AA	AA
	AA	AA	AA	AA	AA	AA	AA	AA
	AA	AA	AG	AG	AA	AG	AG	AA
	AA	AA	AA	AA	AG	AG	AG	AG
	AA	AG	AA	AG	AA	AA	AA	
35161672								
6276	rs10175361		2	AG	AG	AG	AG	AA
	AG	AG	AA	AA	AA	AG	AA	AG
	AG	AG	AA	AA	AA	AA	AA	AA
	AA	AA	AA	AA	AA	AA	AA	AA
	AG	AG	AG	AA	GG	AG	AG	
71993330								
6277	rs10175370		2	AG	AA	AG	AA	AG

AG AG AG GG GG GG GG GG
GG GG AG AG AA GG GG GG
AG AG AG AG AA GG AG AG
GG AG GG AG GG GG AG
228465748
6278 rs10175599 2 AG AA AG GG AG
AG AG AG AG AG AG AG AG
AA AA AG AG GG AG AG AA
AA AG AA AA AG AG AA GG
AG AG AA AA AG GG AG
100787635
6279 rs1017560 4 AA AA AC AC AA
AC AA AC AC AA AC CC AC
AA AA AA AA AA AC AC AA
AA AC AC AA CC AC AC CC
CC AC CC AC AA AA AA
85434987
6280 rs10175607 2 GG GG GG GG GG
GG GG GG GG GG GG GG GG
GG GG AG GG AG GG GG GG
GG GG GG GG GG AG GG AG
GG AG AG GG AG AG GG
106292197
6281 rs1017568 9 AA AA AA AA AG
AG AA AA AA AG AG AG GG
AG GG AA AA AA AA AG AG
AA AA AA AA AA AA AA AA
AA AG AA AA AG AG AG
3948365
6282 rs10175694 2 GG GG GG GG AG
AG GG AG AG AG AA AA AA
AG AG AG AG AA GG AG GG
AG GG AG AG AG AA AG AA
AA AG AG AA GG AG GG
173711641
6283 rs10175703 2 CC CC CC CC CC
CC CC CC CC CC CC CC CC
CC CC CC CC CC CC CC CC
CC CC CC CC CC CC CC CC
CC CC CC CC CC CC CC
56982949
6284 rs10175707 2 AG AG AG AG AG
-- AA AA AA AA AA AA AA
AA AA AA AA AA AA AA AA
AG AA AG AA AG AA AG AG
AG AA AG AG AA AA AA
60376723
6285 rs10175708 2 AG AG AG GG GG
GG AG AG AG AG GG AG GG
AG GG AA AG AA AG GG AA

	GG	AG	AG	AG	AA	AA	AA	AA
	AG	AG	AG	AG	AG	AA	AG	
218899066								
6286	rs1017571		5	AA	AA	AG	GG	GG
	GG	AG	AG	AG	AG	AA	AG	AA
	GG	AG	GG	AG	AG	AG	AA	AG
	AA	AA	AG	AA	AG	AG	GG	AG
	AG	AG	AG	AG	AG	AA	AG	
13694723								
6287	rs1017575		16	AA	AA	AA	AA	AA
	AA	AA	AA	AA	AA	AA	AA	AA
	AA	AA	AG	AG	AG	AG	AG	AG
	AA	AA	AA	AA	AA	AA	AA	AA
	AA	AA	AA	AA	AA	AA	AA	
27690168								
6288	rs10175781		2	AG	GG	GG	GG	GG
	GG	GG	GG	GG	GG	GG	GG	GG
	GG	GG	AG	AA	AG	AG	AG	AG
	GG	GG	GG	GG	AG	AG	AG	AG
	GG	AA	GG	AG	GG	AG	GG	
77287190								
6289	rs10175865		2	AA	AA	AA	AA	AA
	AA	AA	AA	AA	AA	AA	AA	AA
	AA	AA	AA	AA	AA	AA	AA	AA
	AA	AA	AA	AA	AA	AA	AA	AA
	AA	AA	AA	AA	AA	AA	AA	
109483776								
6290	rs1017592		2	GG	GG	GG	GG	GG
	GG	GG	GG	GG	GG	GG	GG	GG
	GG	GG	GG	GG	GG	AG	GG	AG
	GG	GG	GG	GG	GG	GG	GG	GG
	GG	GG	GG	GG	GG	GG	GG	
177359947								
6291	rs10175942		2	GG	GG	GG	AA	AG
	AA	AG	AA	AA	AA	GG	GG	GG
	AA	AA	AG	AA	GG	GG	GG	GG
	AG	AG	AG	AG	AG	AA	AG	AA
	AA	AG	AA	AG	AG	AG	AA	
43259164								
6292	rs10175998		2	AG	AG	AG	AA	AA
	AA	AA	AA	AA	AG	AG	AG	AG
	AA	AA	AG	AA	AG	AA	AG	AA
	AA	AA	AA	AA	AG	AA	AG	AA
	AA	AA	AA	AA	AG	AG	AA	
38559325								
6293	rs10176032		2	GG	GG	GG	AG	AG
	AG	GG	GG	GG	GG	GG	GG	GG
	GG	GG	GG	GG	GG	GG	GG	GG
	GG	GG	GG	GG	GG	GG	GG	GG
	GG	AG	GG	AG	GG	AG	AG	

225896014
6294 rs10176108 2 AC AC AC AC AC
AA CC AA AA AA AA CC AC
AC AC AA AA AA AC AC AC
AA AC AA AC AC CC CC CC
AA AA AC AC AA AA AA
68970181
6295 rs10176201 2 GG GG GG GG GG
AG GG GG GG GG AG GG AG
GG GG GG GG GG GG GG AG
AG GG AG AG AA GG AG AG
GG GG AG GG GG GG GG
98471378
6296 rs10176270 2 GG AG AA GG AA
AG AG GG GG GG AA AG AA
GG GG AG AA GG AG AG AG
AA AA AG AA AA AG AG AA
GG GG AG AG GG GG GG
37237450
6297 rs10176306 2 GG GG GG GG GG
GG GG GG GG GG GG GG GG
GG GG GG GG GG GG GG GG
GG GG GG GG GG GG GG GG
GG GG GG GG GG GG GG
14269460
6298 rs10176321 2 AA AC AA AA AC
AA AC AA AA AC AA AC AA
AA AA AA AA AA AA AA AA
AA AA AA AC AA AA AA AA
AC AC AA AA AC AC AC
235897062
6299 rs10176396 2 AG AG GG GG GG
GG GG AG AG AG AA AA AG
AG AG AG GG AG AG AG AG
AG AG AG AG GG GG GG GG
GG AA AA AA GG GG GG
46584573
6300 rs10176418 2 GG GG GG AA AG
AG AG GG GG GG GG AG AG
AG AA GG GG GG GG GG GG
GG GG GG GG GG AG GG AG
GG GG AG GG GG GG GG
196289817
6301 rs1017643 6 AG AG AA AG AA
AA AG AG GG AG AA AG AA
AA AA AA AG AG AA AA AA
AA AA AG AA AA AA AA AA
AA AA AA AA AG AA GG
156794133
6302 rs10176485 2 GG GG GG GG GG

GG GG GG GG GG GG GG GG
GG GG GG GG GG GG GG GG
GG GG GG GG GG GG GG GG
GG GG GG GG GG GG GG
88368922
6303 rs10176532 2 AA AA AA AA AA
AA AA GG AG GG AG AG AG
AA AA AA AA AG AA AA AA
AA AA AG AA AG AG AG GG
AA AA AG AA AA AA AG
16770723
6304 rs1017654 8 AG AG AG GG GG
AG GG AG AG AG GG AG GG
AG AG AG AG GG GG AA AG
AG AG GG AA GG AA AG AG
GG AG GG AG AG AG AG
15703097
6305 rs10176558 2 AC AC AC AC AC
CC AC AA AA AA AA AA AA
AC AC AC AA AA CC AC AC
AA AC AC AA AC AA AC AA
AA AC AC AA CC CC CC
140608231
6306 rs10176563 2 AG AA AA AA AA
AA AA AA AA AA AA AA AA
AA AA AA AG AA AA AA AA
AA AA AA AA AA AA AA AA
AA AA AA AA AA AA AA
53643093
6307 rs10176602 2 AG AG GG GG AG
AG AG AG AG AG GG GG GG
AG AG GG GG GG GG GG GG
AA AG AA AG GG AG GG AG
GG GG AG GG GG GG GG
74120785
6308 rs10176649 2 CC CC CC AC CC
AC CC CC AC AC AC AC AC
AC CC AC AC AC CC AC CC
CC CC AC AC AC CC CC CC
CC AC AC AC CC CC AC
192044040
6309 rs10176656 2 AA AG AA AA AA
AA AA AG GG AG AA AG AA
AA AA AG AA AA AA AA AA
AA AA AA AA AG AG AG AG
AA AA AA AA AA AA AA
9588091
6310 rs10176682 2 GG GG GG GG GG
GG GG GG GG GG GG GG GG
GG GG GG GG GG GG GG GG

	GG	GG	GG	GG	GG	GG	GG	GG
	GG	GG	GG	GG	GG	GG	GG	
5552777								
6311	rs10176733		2	AG	AG	AG	AA	AA
	AA	AA	AA	AA	AA	AA	AA	AA
	AA	AA	AA	AG	AA	AG	AG	AG
	GG	AG	GG	AG	AA	AG	AG	AA
	AA	AA	AG	AG	GG	AG	AG	
67107863								
6312	rs10176810		2	AA	AA	AA	AA	AA
	AA	AG	AG	AG	AG	AG	AG	GG
	GG	GG	AG	AA	AA	AA	AA	AA
	AA	AA	AA	AG	AG	AA	AA	AA
	AA	AA	AA	AA	AG	GG	AG	
193366488								
6313	rs10176846		2	GG	GG	GG	GG	AG
	GG	AG	GG	GG	GG	AA	GG	AG
	GG	AG	GG	GG	GG	GG	AA	AG
	GG	GG	GG	GG	GG	GG	GG	GG
	GG	AG	GG	GG	GG	GG	GG	
223349309								
6314	rs1017690		19	AA	AA	AA	AC	AA
	AA	AC	AA	AA	AA	AC	AA	AC
	AA	AA	AA	AA	AA	AA	AA	AA
	AA	AA	AA	AA	AA	AA	AA	AA
	AC	AA	AA	AA	AA	AA	AA	
16533057								
6315	rs10176932		2	GG	GG	GG	AG	AG
	AG	AG	AG	AG	AG	AG	AG	AG
	AA	AA	GG	AG	GG	GG	AG	AG
	AG	AG	AG	AG	AG	AA	AG	AA
	AG	GG	GG	GG	AA	AA	AA	
39045885								
6316	rs10177059		2	GG	AG	GG	GG	GG
	GG	GG	GG	AA	AG	AA	AG	AG
	GG	GG	AA	AG	AG	AG	AG	GG
	GG	GG	GG	GG	AA	GG	AG	AG
	GG	AG	GG	AG	AG	GG	AA	
151201049								
6317	rs10177101		2	AA	AC	AA	AA	AA
	AA	AA	AA	AA	AA	CC	AC	CC
	AC	AC	AA	AC	AA	AA	CC	AA
	AA	AA	AA	AA	AC	AA	AA	AC
	AA	AA	AA	AA	AC	AC	AC	
151090995								
6318	rs10177104		2	GG	AG	AG	AA	GG
	AG	AG	AG	AG	AG	AG	GG	GG
	AG	AG	AG	AA	AG	AA	AA	AG
	AA	AA	AA	AA	AG	GG	GG	AG
	GG	AA	GG	AG	AG	AG	AG	

50809998							
6319	rs10177144	2	AC	CC	CC	AC	CC
	CC	AC	AA	AA	AA	CC	CC	CC
	CC	CC	AC	AC	AA	CC	CC	CC
	CC	CC	CC	CC	CC	CC	CC	CC
	AC	CC	AC	AC	CC	CC	CC	
104415982								
6320	rs10177148	2	AA	AA	AA	AA	AA
	AA	AA	AA	AA	AA	AA	AA	AA
	AA	AA	AA	AA	AA	AA	AA	AA
	AA	AA	AA	AA	AA	AA	AA	AA
	AA	AA	AA	AA	AA	AA	AA	
87032574								
6321	rs10177156	2	CC	AC	CC	AA	AC
	AC	AA	AC	AC	CC	AC	CC	AC
	AA	AA	AC	AC	AA	AC	CC	AC
	AC	AC	CC	AC	AC	CC	CC	AC
	AC	AC	AC	AC	AC	AC	CC	
34491664								
6322	rs10177168	2	GG	GG	GG	AA	AG
	AG	AG	GG	GG	GG	GG	AG	GG
	GG	GG	AG	GG	AG	AG	AG	AG
	AG	GG	AG	AG	GG	GG	GG	GG
	AG	GG	AG	AG	AG	AG	GG	
97280240								
6323	rs1017720	15	AA	AG	AG	GG	AG
	AG	AG	GG	GG	AG	AA	GG	AG
	AG	AG	AG	GG	AG	GG	AG	GG
	GG	GG	GG	GG	GG	AG	GG	AG
	AG	GG	GG	GG	GG	GG	AG	
31768529								
6324	rs10177211	2	AA	AA	AA	AA	AA
	AA	AC	AA	AA	AA	AA	AA	AA
	AA	AA	AA	AA	AA	AA	AA	AA
	AA	AA	AA	AA	AA	AA	AA	AA
	AC	AC	AA	AC	AA	AA	AA	
212475771								
6325	rs10177224	2	CC	CC	CC	CC	AC
	CC	AC	CC	CC	CC	CC	CC	CC
	CC	CC	CC	CC	CC	CC	AC	AC
	CC	CC	CC	CC	AC	CC	AC	CC
	CC	AC	CC	AC	CC	CC	CC	
69979290								
6326	rs10177230	2	GG	AG	AA	AA	AG
	AG	AA	GG	GG	GG	AG	AA	AA
	AG	AG	AG	GG	AA	AG	AG	AA
	AG	AG	AG	AG	GG	GG	GG	GG
	AA	GG	AG	GG	AG	AG	AG	
53715181								
6327	rs10177265	2	AG	AG	AG	AG	AA

AG AG AG AG AG AG AG AG
AG GG AA AA AA AG AA AA
GG GG AG AA AG GG AG AG
AA GG AG AG GG AG AG
199618879
6328 rs1017730 11 GG GG AG GG GG
GG GG AG AG AG AG GG AG
AG AG GG GG GG AG GG AG
AA AG AA AG GG GG GG GG
GG AG GG AG AG AG GG
47733494
6329 rs10177310 2 GG AG GG GG GG
GG GG GG AG AG GG GG GG
GG GG GG GG GG AG GG AG
GG GG GG GG GG GG GG GG
GG AG GG AG GG GG GG
192369816
6330 rs10177440 2 AA AA AG GG GG
GG GG AG GG AG AG AG AG
AG AG AG AG AA AG AG GG
AA GG AA AG AG GG AG GG
AG AG AG AG AG AA AG
78025242
6331 rs10177476 2 AG AG AG AA AA
AA AG AG AG AG AA AA AA
AG AA AG AG AG GG GG GG
AA AG AG AG AG AA AG AG
AG AG GG GG GG AG AG
220228371
6332 rs10177485 2 GG AG AG AG AG
GG AA AG AG AA AA AG AG
AG AG AA AA AA AG AG AG
GG GG AG GG AA GG AG AG
AG AG GG AG AG AG AA
36019937
6333 rs1017756 6 GG GG GG GG GG
GG GG AG AG AG GG GG GG
GG GG GG GG AG AG GG GG
GG GG GG GG GG AG AG AG
GG GG GG GG AG AG GG
10425031
6334 rs10177601 2 GG GG GG AG AG
AG AG GG AG GG AG AG AG
GG GG GG GG AG AG AG AG
AA AG AG AA AG AG AG GG
AG GG AG GG GG GG GG
17271863
6335 rs10177684 2 GG GG GG GG GG
GG GG GG GG GG GG GG GG
GG GG GG GG GG GG GG AG

GG AG GG AG GG GG GG GG
GG GG GG GG GG GG GG
184591219
6336 rs10177696 2 AG AG AG AA AA
AA AA AA AA AA AG AA AA
AA AA AA AA AA AA AA AA
AA AA AA AA AA AG AA AG
AA AA AA AA AG AG AG
99351716
6337 rs1017771 2 AA AA AA AA AA
AA AA AA AA AA AA AA AA
AA AA AA AA AA AA AA AA
AA AA AA AA AA AA AA AA
AG AA AA AA AA AA AA
170817760
6338 rs10177733 2 AA AA AA AA AA
AA AA GG GG GG AA AA AA
AA AA AG AG AG AG AG GG
AG AG AG AG GG AA AG AG
AG GG GG GG AA AA AG
186283241
6339 rs10177762 2 AA AA AC AA AA
AA AA AC AC AC AA AA AA
AA AA AA AA AA AC AC AC
AA AA AA AA AC CC CC CC
AC AA AA AA CC AC AC
238635137
6340 rs1017781 18 AG AA AG GG AG
AG AG AA AA AA AA AA AA
AA AA AG AG AA AA AG AA
AA AA AA AA AA AA AA AA
AA AA AA AA AA AA AA
44242505
6341 rs10177816 2 CC CC CC CC CC
CC CC CC CC CC CC CC CC
CC CC CC CC CC CC CC CC
CC CC CC CC CC CC CC CC
CC -- CC CC CC CC CC
84133759
6342 rs10177866 2 AA AA AA AG AG
AA GG AA AA AA AA AA AG
AG AG AA AA AA AG AA AG
AA AA AA AA AG AG AG AG
AA AA AA AA AG AA AG
127582332
6343 rs10178017 2 GG AG GG GG GG
GG AG AG AA AG AA AA AA
AA AA AA AA AA AG AG AG
AG AG AA AG AG AA AA AA
AA AA AA AA AG AG AA

9893138
6344 rs10178021 2 AC AA AC AC AC
AA AC CC AC CC AA AC AC
CC CC AC AC AA CC AC AC
AC AA AA AC CC AA AC AC
CC AA AC AA AC AC CC
139878422
6345 rs10178040 2 AA AG AG GG GG
GG GG GG GG GG GG GG GG
AG AG GG AG GG GG GG GG
AA AG AA AA GG AG AG GG
GG GG AG AG AG GG AA
45484777
6346 rs10178048 2 CC CC CC CC CC
CC AC CC AC CC CC CC CC
CC CC AC AC AC CC AC CC
CC CC CC CC AC AC AC CC
CC CC CC CC CC CC CC
6325751
6347 rs10178069 2 AA AA AA AA AA
AA AA AG AG AG AA AA AA
AA AA AG AG AG AA AA AA
AA AA AA AA AA AG AA AG
AA AA AA AA AA AA AG
217720468
6348 rs1017809 15 AA AA AA AA AA
AA AA AA AA AA AA AA AA
AA AA AA AG AG AA AA AA
AA AA AA AA AA AG AG AA
AA AA AA AA AA AA AA
73461466
6349 rs10178108 2 GG GG AG AG AG
GG AA AA AA AA AA AA AG
AG AG AG AA GG GG GG AG
GG AG AG GG GG AG AG GG
AG AG AA AG AG AG AG
43391756
6350 rs10178148 2 AA AG AA AA AA
AA AA AA AA AA AG AG AA
AA AA AA AA AA AA AG AG
AG AA AA AG AA AA AA AA
AA AA AA AA AA AA AA
144000004
6351 rs10178155 2 GG GG GG GG GG
GG GG GG GG GG GG GG GG
GG GG GG GG GG GG GG GG
GG GG GG GG GG GG GG GG
GG GG GG GG GG GG GG
62670813
6352 rs1017818 19 GG GG GG AG GG

	AG	GG	GG	GG	GG	GG	GG	GG
	GG	GG	AG	GG	GG	GG	GG	GG
	GG	GG	GG	GG	GG	GG	GG	GG
	GG	GG	GG	GG	GG	GG	GG	
5155308								
6353	rs10178199		2	GG	GG	GG	AG	AG
	GG	AG	AG	AG	AG	GG	GG	GG
	GG	GG	GG	GG	GG	AG	GG	AG
	GG	GG	GG	GG	GG	GG	GG	GG
	GG	AG	GG	AG	GG	GG	GG	
127756954								
6354	rs10178271		2	GG	AG	AG	GG	GG
	GG	GG	GG	AG	GG	GG	GG	GG
	GG	GG	GG	AG	AG	GG	GG	AG
	GG	GG	GG	GG	AG	GG	AG	GG
	GG	GG	AG	AG	GG	GG	GG	
84443628								
6355	rs10178349		2	AG	AA	AG	AA	AG
	AG	AA	AG	GG	GG	AA	AA	AA
	AG	AG	AG	AA	AA	AG	AG	AG
	AA	AG	AA	AG	AA	AG	AG	AG
	AA	AG	AG	AA	AG	AG	AG	
167639589								
6356	rs10178418		2	AA	AA	AA	AG	AA
	AG	AA	AA	AG	AA	AA	AA	AA
	AG	AG	AA	AA	AA	AG	AG	AA
	AG	AG	AG	AG	AG	AA	AG	AA
	AG	AG	AA	AG	AG	GG	AG	
84056231								
6357	rs1017843		15	GG	GG	GG	GG	GG
	GG	GG	AG	GG	AG	AG	AA	AG
	AA	AG	AG	AG	AG	GG	AA	GG
	GG	AG	AG	AG	AA	AG	AA	AA
	AA	AG	GG	AG	AG	GG	AG	
40528155								
6358	rs10178492		2	AA	AG	GG	AA	AA
	AA	AA	AA	AA	AA	AA	AA	AA
	AA	AA	AA	AG	AA	AA	AA	AA
	AA	AG	AA	AG	AG	AA	AA	AA
	AG	AG	AA	AG	AA	AA	AA	
55958891								
6359	rs10178547		2	AC	AA	AC	AC	AC
	AC	AC	--	CC	CC	CC	AC	--
	AC	AC	AA	AC	AC	AC	--	AA
	--	AC	CC	--	CC	CC	--	CC
	AC	AA	AC	AA	AC	--	AA	
124144870								
6360	rs10178597		2	CC	CC	CC	CC	CC
	CC	CC	CC	CC	CC	CC	CC	CC
	CC	CC	CC	CC	CC	CC	CC	CC

-- CC CC CC CC CC CC CC
CC CC CC CC CC CC CC
17136171
6361 rs1017863 4 AG AG AG AA AA
AA AA AA AA AA AA AA AA
AA AA AA AA AA AA AG AA
AA AA AA AA AG AA AA AA
AA AA AA AA AG AA AG
127093759
6362 rs10178648 2 AC CC AC AC CC
AC AC CC CC CC CC CC CC
CC CC AA AA AA AC AC AA
AA AC AA AA AC CC CC AC
AA CC AA AC AC AC CC
112444459
6363 rs10178652 2 AA AG GG AG GG
GG AG AG AG AG GG GG GG
GG GG GG AG AG AG AG GG
AG AG AG AG AG AG AG GG
AG GG AG AG AG AG AG
60296223
6364 rs10178717 2 AA AA AA AA AA
AA AA AA AA AA AA AA AA
AA AA AA AA AA AA AA AA
AA AA AA AA AA AA AA AA
AA AA AA AA AG AA AG
110452645
6365 rs10178738 2 GG GG GG AA AG
AG AG GG AG AG AA AG AA
AA AG GG GG GG GG GG GG
GG GG GG GG AG AA AG AG
GG GG GG GG AG AA AG
240864911
6366 rs1017874 X GG GG AG GG GG
GG GG GG GG GG GG GG GG
GG GG GG GG GG GG GG GG
GG GG GG GG AG GG AG GG
GG GG GG GG AG GG AA
19869646
6367 rs1017875 11 AG GG GG AG AG
GG AG GG GG GG GG AG AG
GG AG GG GG GG AG AG GG
GG GG GG GG GG GG GG GG
GG AG AG GG GG GG GG
47999218
6368 rs10178814 2 GG GG GG GG GG
GG GG GG GG GG GG GG GG
GG GG AG AG AG GG GG GG
GG GG GG GG GG GG GG GG
GG AG GG GG GG GG GG

199123438							
6369	rs10178826	2	AA	AA	AA	AA	AA
	AA	AA	AA	AA	AA	AA	AA	AA
	AA	AA	AC	AC	AC	AA	AA	AA
	AA	AA	AA	AA	AA	AA	AA	AA
	AA	AA	AA	AA	AA	AA	AA	
11011192							
6370	rs1017885	15	AG	AG	GG	GG	GG
	GG	GG	GG	GG	GG	AG	AG	AG
	GG	AG	GG	AG	AG	AG	GG	GG
	GG	AG	AG	AG	GG	GG	GG	GG
	AG	GG	AG	GG	GG	GG	GG	
59602200							
6371	rs10178875	2	AG	AA	AG	AA	AA
	AA	AA	AG	AG	AG	AA	AA	AA
	AA	AA	AA	AA	AG	AA	AA	AA
	AA	AG	AG	AA	AG	AA	AA	AG
	AG	AG	AA	AG	AA	AA	AA	
142634758							
6372	rs10178881	2	AA	AA	AA	AA	AG
	AG	AG	AA	AG	AG	AA	AG	AA
	AA	AA	AA	AA	AA	AA	AA	AG
	AA	AA	AG	GG	AG	AG	AA	AG
	AA	AG	AA	AG	AA	AA	AA	
202652583							
6373	rs10178913	2	AA	AA	AA	AG	AA
	AG	AA	AG	AG	AG	AA	AA	AA
	AA	AA	AA	AA	AA	GG	AA	AG
	AG	AG	AG	AA	AA	AA	AA	AA
	AA	AA	AA	AA	AA	AG	AA	
181002168							
6374	rs10178921	2	AA	AA	AA	AA	AG
	AG	AG	AA	AA	AA	AA	AA	AA
	AG	AG	AG	AG	AG	AA	AA	AA
	AA	AA	AA	AA	AG	AG	AG	AA
	AA	AG	AA	AA	AA	AG	AA	
165603512							
6375	rs10179007	2	AA	AA	AA	AA	AA
	AA	AA	AA	AA	AA	AA	AA	AA
	AA	AA	AA	AA	AA	AA	AA	AA
	AA	AA	AA	AA	AA	AA	AA	AA
	AA	AA	AA	AA	AA	AA	AA	
170752459							
6376	rs10179015	2	AA	AA	AA	AG	AA
	AG	AA	AG	GG	AG	AA	AG	AA
	AA	AA	AG	AA	AG	AA	AA	AA
	AG	AG	AA	AG	AA	AG	AG	AG
	GG	AG	GG	AG	GG	AG	GG	
9814374							
6377	rs10179086	2	AA	AA	AA	AG	AA

	AA	AG	AG	AG	GG	AA	AA	AA
	AA	AA	AG	AA	AA	AA	AG	AG
	AG	AG	AG	GG	AA	AA	AA	AA
	AA	AA	AA	AA	AA	AA	AA	
232744209								
6378	rs1017912		4	AA	AA	AA	AC	AA
	AA	AC	AC	AA	AC	CC	CC	AC
	AC	AC	AA	AC	AC	AA	AA	AC
	AA	AC	AC	CC	AC	AC	AC	CC
	AA	AA	CC	AC	AC	CC	AA	
177529489								
6379	rs1017913		3	AC	AA	AC	AC	AC
	AA	CC	AC	AC	AC	CC	AC	CC
	AC	AC	AC	AA	AC	AC	AC	AC
	AA	CC	AC	AA	AA	AA	AA	AA
	AC	AC	AC	AC	AC	AA	AC	
105238615								
6380	rs10179149		2	AA	AG	AG	AG	AA
	AG	AA	AA	AA	AA	AG	AG	AG
	GG	GG	AA	AA	AA	AG	AA	AA
	AG	AA	AG	AG	AA	AG	AG	AA
	AA	AG	AA	AA	AA	AG	AA	
171595545								
6381	rs10179156		2	GG	GG	GG	GG	GG
	GG	GG	GG	GG	GG	GG	--	GG
	GG	GG	GG	GG	GG	GG	GG	GG
	GG	GG	GG	GG	GG	GG	GG	GG
	GG	GG	GG	GG	GG	GG	GG	
10483613								
6382	rs10179194		2	AA	AA	AA	AA	AG
	AG	AA	AA	AA	AA	AG	AG	AG
	AA	AA	AA	AG	AG	AA	AA	AG
	AA	GG	AA	AG	AA	AG	AA	AG
	GG	AA	AA	AA	AA	AA	AA	
180298412								
6383	rs10179280		2	GG	GG	GG	GG	GG
	GG	GG	GG	GG	GG	GG	GG	GG
	GG	GG	GG	GG	GG	GG	GG	GG
	GG	GG	GG	GG	GG	GG	GG	GG
	GG	GG	GG	GG	GG	GG	GG	
85880472								
6384	rs10179288		2	GG	GG	GG	GG	GG
	GG	GG	AG	AG	AG	AG	AG	AG
	AG	AG	GG	AG	GG	GG	GG	GG
	AG	AA	AG	GG	AG	AG	AA	AG
	GG	GG	AG	AG	AG	AA	GG	
15310820								
6385	rs10179320		2	GG	GG	GG	GG	GG
	GG	GG	GG	GG	GG	AG	AG	AA
	AA	AA	AG	GG	GG	GG	GG	GG

GG GG GG GG GG GG GG GG
GG GG AG AG GG GG GG
177955756
6386 rs10179323 2 GG GG GG GG GG
GG GG GG GG GG GG GG GG
GG GG GG GG GG GG GG GG
GG GG GG GG AG GG GG AG
GG GG GG GG GG GG GG
98618247
6387 rs10179343 2 GG GG AG GG GG
GG GG GG GG GG GG GG GG
GG GG GG GG GG AG AG GG
AG GG AG AG GG GG GG GG
GG GG GG GG GG GG GG
99993176
6388 rs10179361 2 GG GG GG GG GG
GG GG GG GG GG GG GG GG
GG GG GG GG GG GG GG GG
GG GG GG GG GG AG GG AG
GG GG AG GG GG GG GG
88855673
6389 rs10179370 2 AG GG AG AA AA
AA AA AG AG AG AA AA AA
AA AA AA AA AA AA AG AA
AA AA AA AA AA AG AA AG
AA AA AA AA AG AA AG
851775
6390 rs1017939 11 AA AA AA AG AG
AA AG AA AA AA AA AA AA
AA AA AA AA AA AA AA AA
AA AA AA AA AA AA AA AA
AA AA AA AA AA AA AA
7343762
6391 rs10179510 2 GG GG GG AG AG
AG GG AG AA AG AG AG AG
GG GG AG GG AG AG AG AG
GG AG AG GG GG AG AG GG
GG AG AG AG GG AG AG
4317473
6392 rs10179524 2 AA AA AA AA AA
AA AA AA AA AA AA AA AA
AA AA AA AA AA AA AA AA
AA AA AA AA AA AA AA AA
AA AA AA AA AA AA AA
134988860
6393 rs10179529 2 AG GG AG AA AG
AG AG GG AG GG AG AG AG
GG GG AG AA AG AG AG AG
AG AG AG AG GG AG GG AG
AG AA AA AA AG AG GG

89001656
6394 rs10179616 2 GG AG AG GG AG
AG GG GG GG AG AG GG AG
GG GG GG GG GG AG AG GG
AA AA AG AA AG AG AG AG
AA AG AA AG AG AG AG
35666876
6395 rs10179618 2 AG AG GG GG GG
AG GG GG GG AG AG AG GG
GG GG AA AA AG AG AA AG
GG AA AG AG AG AG GG AG
AA AG AA AG AG AG AA
155815731
6396 rs1017964 4 AG AG AG AG GG
AG AG AA AA AA AA AG AA
AA AA AG AG AG AA AA AA
AG AG AG AG AA AG AG AA
AG AG AG AG AG GG AG
118463264
6397 rs10179648 2 AG AA AG AG AG
GG AA AG AG GG AA AA AG
GG GG GG AG AG AG AG GG
AA AA AA AA GG AA AG AG
AA AG AG AA AA AA AA
43808065
6398 rs10179714 2 GG GG GG GG GG
GG GG GG GG GG GG GG GG
GG GG GG GG GG GG GG GG
GG GG GG GG GG GG GG GG
GG GG GG GG GG GG GG
113805118
6399 rs10179787 2 GG GG GG GG GG
GG GG GG GG GG GG AG AG
AG AG GG GG GG GG AG GG
GG GG GG GG GG GG GG GG
GG GG GG GG AG GG AG
217986622
6400 rs10179797 2 AG GG AG AA AA
AA AA AA AA AA AA AA AA
AA AA AG AA AG AG AG AG
AG AG AA AA AA AA AA AA
AA AA AA AA AA AA AG
109836825
6401 rs10179812 2 AA AA AA AA AA
AA AA AA AA AA AA AA AA
AA AA AA AA AA AA AA AA
AC AA AC AC AA AC AC AA
AA AA AC AA AA AA AA
179775893
6402 rs10179820 2 AG AG AG AA AG

AA AG GG AG AG GG GG GG
GG GG GG GG AG AG AG AA
GG GG GG AG AG AG AG GG
GG AG AG AA GG GG AG
227970914
6403 rs10179904 2 GG GG GG GG GG
GG GG AG AG AG GG AG GG
GG GG GG GG GG GG GG GG
GG GG GG GG GG AG GG AG
GG AG GG GG AG GG AG
85780107
6404 rs1017994 2 AA AG AA AA AA
AG AA AA AA AA AG AG AG
AA AA AG AG AG AA AA AG
AA AG AG AA AA AA AA AA
AG AG AG AA GG AG GG
143607317
6405 rs10179941 2 AG AA AG AA AA
AA AG AG GG AG GG GG GG
AG AG AA AG AG GG GG GG
AG AG AG AG AG AA AA AG
GG GG AG GG AA AA AA
76385773
6406 rs10179948 2 AA AA AA AA AA
AA AA AA AA AA AA AA AA
AA AA AA AA AA AA AA AG
AA AA AA AA AA AA AA AA
AA AA AA AA AA AA AA
112770821
6407 rs10179986 2 AG AG AG AG AG
AG AG AA AG AA AA AA AA
AA AA AG AA AG AG AG AA
AG AG AG AG AA AA AA AA
AG GG AG AG AG AG GG
134864344
6408 rs1018015 8 GG GG GG AG AG
AG AA AG AG AA AA AA AA
AA AA AA AG AA AG AG GG
GG AA AA AG AG AA AA AA
AA GG AG AG AG AA GG
71919570
6409 rs10180162 2 GG GG GG GG GG
GG GG GG GG GG GG GG GG
GG GG GG GG GG GG GG GG
GG GG GG GG GG GG GG GG
GG GG GG GG GG GG GG
13156189
6410 rs10180187 2 GG AG AG GG GG
GG GG GG GG AG GG GG GG
GG GG AG AA GG AG AG GG

AG AG AG AG GG AG AG GG
AG AA GG AG GG GG GG
44452127
6411 rs10180227 2 AC AA AC CC CC
CC CC CC CC CC CC CC CC
CC CC AC CC AC AC AC AC
AC AC CC CC CC CC CC CC
CC CC CC CC CC CC AC
109805536
6412 rs10180230 2 AA AA AA AA AA
AA AA AG AG AG AA AA AA
AA AA AA AA AG AA AA AA
AA AA AA AA AA AA AA AA
AA AA AA AA AA AG AA
5431246
6413 rs10180248 2 CC CC CC CC CC
CC CC CC AC AC AA AC AA
AA AC CC CC CC CC CC CC
CC CC CC AC CC CC CC CC
AC AC AC AA AC CC AC
205608382
6414 rs10180263 2 AA AA AA AG AG
AA AG AA AG AG AA AA AA
AA AA AA AA AG AA AA AA
AA AA AA AA AA AA AA AA
AA AG GG AG AA AA AA
168587946
6415 rs10180282 2 AA AA AA AG AG
AG AG AA AA AA AG GG GG
AG AG AG AG AA AG AG AG
GG AG GG AG AG AG GG AG
GG AG GG GG AG AG AA
60578843
6416 rs10180287 2 CC CC CC AC CC
CC AC CC AC AC AA AA AA
AC AC AC AC CC AC AC CC
CC AC AC AC CC CC CC CC
AA AA AC AA AA AA AC
52382382
6417 rs10180293 2 AA AA AA AG GG
AG AG AA AA AA AG AG AG
AG AG AA AA AA AG AG AA
AA AA AA AA AA AA AA AA
AA AG AA AG AA AG AA
206622576
6418 rs10180362 2 GG GG GG AG GG
GG AG GG GG GG GG AG GG
GG GG GG GG GG GG AG AG
GG GG GG GG GG AG GG GG
GG GG AG GG GG GG AG

238137652
6419 rs10180416 2 AG AG AG AG AA
AA AG AG AA AG GG GG GG
GG GG AG AG AG GG GG AG
AG AG AA AG AG AA AG AA
GG AG AG AA AA AG AA
24677866
6420 rs10180451 2 CC AC AC CC CC
CC CC CC AC CC CC AA AC
AA AA AC AC CC CC CC CC
CC AC CC CC AC CC CC AC
AC CC CC CC CC CC CC
85926238
6421 rs1018047 5 CC AC CC AA AC
AA AC AA AA AA AC AC CC
AC AC AA AA AA AA AA AA
AC AC AC AC AA AC AC AA
AA CC AA AC AA AC AA
123896355
6422 rs10180494 2 AA AA AA AG AG
GG AA AA AG AG GG AA AG
AA AG AA AA AA AG AG AG
AA AA AG GG AA AG AA AG
AG AG GG AG GG GG AG
212796981
6423 rs10180512 2 AA AA AA AA AC
AC AA AA AC AA AC AC AC
AC AC AA AA AC AC AC AC
AC AA AC CC AA AA AA AA
CC AC AA AA AA AC AA
15268841
6424 rs10180549 2 AG AG AG AG GG
GG AG GG GG GG AA AA AA
AG AG AA AG AG GG GG AG
AA AG AA AG AG AA AA AA
AG AA GG AG AA AG AG
67406814
6425 rs10180596 2 AA AC AC AA AC
AA AC AA AA AA AC AA AA
AC AC AC CC AC CC CC CC
AA CC AC AC AC AA AC AA
AC AC CC AC CC AC AC
49418070
6426 rs10180601 2 AG AG AG GG GG
AG GG GG GG GG AG AG AG
GG GG GG GG GG GG GG GG
AG GG GG AG GG GG GG GG
GG AG AG AA GG GG GG
138135771
6427 rs10180670 2 GG GG GG GG AG

	AG	GG	GG	GG	GG	AG	GG	GG
	GG	GG	GG	AG	GG	GG	AG	AG
	GG	GG	GG	GG	GG	GG	GG	GG
	AG	AG	AG	AG	GG	GG	AG	
5607349								
6428	rs10180684		2	GG	GG	GG	GG	GG
	GG	GG	GG	GG	GG	GG	GG	GG
	GG	GG	GG	GG	GG	GG	GG	GG
	GG	GG	GG	GG	GG	GG	GG	GG
	GG	GG	GG	GG	GG	GG	GG	
160351339								
6429	rs10180746		2	AG	AG	GG	AG	AG
	AG	AA	GG	GG	GG	AG	AG	AA
	AA	AA	AG	AG	AG	AG	AG	AG
	GG	AG	GG	GG	GG	AG	AG	AG
	AG	AG	AA	AG	AG	GG	AG	
87852863								
6430	rs10180761		2	AG	AG	AA	AG	AG
	AA	GG	AG	AG	AG	AG	AG	AG
	GG	GG	AG	AG	AG	AA	AA	AG
	AG	AG	AG	AG	AG	AG	GG	AA
	GG	AG	GG	AG	AA	AA	AA	
39722668								
6431	rs10180853		2	GG	AG	GG	GG	GG
	GG	GG	GG	GG	GG	GG	GG	GG
	GG	GG	GG	GG	AG	GG	GG	GG
	AG	AG	GG	AG	AG	GG	AG	GG
	GG	GG	GG	GG	GG	AG	GG	
18043777								
6432	rs10180871		2	AA	AA	AA	AA	AA
	AA	AA	AA	AA	AA	AA	AA	AA
	AA	AA	AA	AA	AA	AA	AA	AA
	AA	AA	AA	AA	AA	AA	AA	AA
	AA	AA	AA	AA	AA	AA	AA	
88989846								
6433	rs10180924		2	AG	AG	GG	GG	GG
	GG	GG	AA	AG	AA	AA	GG	AG
	AG	AG	GG	GG	GG	GG	GG	GG
	GG	AA	GG	GG	AG	GG	AG	GG
	GG	GG	AA	AG	AG	AG	AG	
86021919								
6434	rs10180951		2	GG	GG	GG	GG	GG
	GG	GG	AG	AG	AG	GG	GG	GG
	GG	GG	GG	GG	GG	GG	GG	GG
	AG	GG	AG	GG	GG	GG	GG	GG
	GG	AG	GG	AG	GG	GG	GG	
80056097								
6435	rs1018100		15	GG	AG	AG	AG	AG
	AA	GG	GG	AG	AG	AA	AG	AG
	AG	AG	AA	AG	AG	AA	AG	AA

	AA	AG	AG	AG	AA	AG	AG	AA
	AG	GG	GG	GG	AG	AA	AG	
76447753								
6436	rs10181045		2	AA	AA	AA	AA	AA
	AA	AA	AA	AA	AA	AA	AA	AA
	AA	AA	AA	AA	AA	AA	AA	AA
	AA	AA	AA	AA	AA	AA	AA	AA
	AA	AA	AA	AA	AA	AA	AA	
237885618								
6437	rs1018105		15	AG	AA	AG	AG	AA
	AG	AA	AA	AA	AA	AG	AA	AA
	AA	AA	AA	AA	AA	AA	AA	AA
	AA	AA	AA	AA	AA	AG	AA	AG
	AA	AG	AA	AA	AA	AA	AA	
28083423								
6438	rs10181071		2	AA	AA	AA	AA	AA
	AA	AA	GG	AA	AG	AG	AG	AG
	AA	AA	AA	AA	AA	AA	AA	AA
	AA	AA	AA	AA	AA	AG	AG	AG
	AA	AA	AA	AA	AG	AG	AA	
142862830								
6439	rs1018109		3	AG	GG	AG	AG	AG
	GG	AG	GG	AG	GG	AG	AA	AG
	AG	GG	AG	AA	GG	GG	AG	GG
	GG	GG	GG	GG	AA	GG	AG	AG
	AG	GG	GG	GG	GG	AG	AG	
4827533								
6440	rs10181102		2	AA	AA	AA	AA	AG
	AA	AG	AA	AA	AG	AA	AA	AA
	AA	AA	AA	AA	AA	AA	AA	AA
	AA	AA	AA	AA	AA	AA	AA	AA
	GG	AA	AA	AA	AA	AA	AA	
111835489								
6441	rs10181110		2	AG	GG	AG	AG	GG
	AG	GG	AG	GG	GG	AG	AG	AG
	AA	AA	AA	AG	AG	AA	GG	AA
	GG	AG	AG	AA	AG	AA	AA	AA
	GG	GG	AG	GG	AG	AG	AA	
183747442								
6442	rs1018113		3	AA	AG	AG	AA	AG
	AG	AG	GG	AG	AG	AG	GG	AG
	AG	AA	AA	AG	AG	AG	GG	AA
	AA	AG	AA	AA	AG	AG	AA	AG
	GG	GG	AA	AG	AA	AA	AA	
8533268								
6443	rs10181138		2	AG	GG	GG	GG	GG
	GG	GG	GG	GG	GG	GG	GG	GG
	GG	GG	GG	GG	GG	GG	GG	GG
	GG	GG	GG	GG	GG	GG	GG	GG
	GG	GG	GG	GG	GG	GG	GG	

97115019
6444 rs10181163 2 AA AG AG AG AG
AG AG AG AG AG AA AA AA
AG AG AG AG GG AA AA AA
AA AG AA AA AG AA AG AA
AA AG AA AG AA AA AG
116841792
6445 rs10181201 2 AA AA AA AA AA
AA AA AA AA AA AG GG GG
AA AA AA AA AA AA AA AA
AA AA AA AA AA AA AA AA
AA AA AA AA AA AA AA
54799174
6446 rs10181206 2 AA AA AA GG GG
GG AG AA AG AA AA AA AG
AA AA AA AA AG AA AA AA
AG AA AA AG AA AA AA AA
GG AA AA AA AA AG AG
138563356
6447 rs10181221 2 GG GG GG GG GG
GG GG GG GG GG GG GG GG
GG GG GG GG GG GG GG GG
GG GG GG GG GG GG GG GG
GG GG GG GG GG GG GG
231326148
6448 rs10181238 2 GG GG GG AA AG
AG AG GG AG AG AG GG GG
GG GG GG GG GG AA AG AG
AG AG AG AG GG AG GG GG
AG AG AG GG GG AG GG
240709496
6449 rs10181293
2 -- -- -- -- -- -- -- --
-- -- -- -- -- -- -- --
-- -- -- -- -- -- -- --
-- -- -- -- -- -- -- --
-- -- -- -- 240857843
6450 rs10181295 2 GG GG GG AA AG
AA AG AG AG AG AG AG AG
AG AG AG GG GG GG GG AG
AG AA AG GG AG AA AA AG
AG GG AG AG AA AA AA
15441492
6451 rs10181344 2 AG GG GG GG AG
GG AG AG AA AG AG AG GG
GG GG AG AG AA GG GG AG
AG GG AG AG AG AG GG AA
AG GG GG GG AG AG GG
99692829
6452 rs10181354 2 AC CC CC CC CC

	CC	CC	CC	CC	CC	CC	CC	CC
	CC	CC	CC	CC	CC	CC	CC	CC
	CC	CC	CC	CC	CC	CC	CC	CC
	CC	CC	CC	CC	CC	CC	CC	
79732746								
6453	rs10181383		2	CC	CC	CC	CC	CC
	CC	CC	CC	CC	CC	CC	CC	CC
	CC	CC	CC	CC	CC	CC	CC	CC
	CC	CC	CC	CC	CC	CC	CC	CC
	CC	CC	CC	CC	CC	CC	CC	
175969196								
6454	rs1018139		4	GG	GG	GG	AG	AG
	AG	GG	GG	GG	GG	AG	GG	GG
	GG	AG	GG	GG	GG	AG	AG	AG
	AG	AG	AG	GG	AG	AG	GG	AG
	GG	GG	GG	GG	GG	GG	GG	
166848001								
6455	rs10181393		2	AG	GG	AG	AG	AG
	AA	GG	AA	AA	AA	AA	AG	AG
	GG	GG	AG	AG	AA	GG	AG	GG
	AG	GG	AG	AG	AA	AA	AA	AA
	AG	AG	AG	AG	AG	AG	AA	
5361397								
6456	rs10181427		2	AG	AG	AA	GG	GG
	GG	GG	GG	GG	GG	GG	GG	GG
	GG	GG	GG	GG	GG	GG	GG	GG
	GG	GG	GG	GG	GG	GG	GG	GG
	AG	GG	AG	GG	AG	AG	GG	
164270138								
6457	rs1018143		11	AA	AA	AA	AA	AA
	AA	AA	AG	AA	AA	AG	AG	GG
	AG	AG	GG	AG	AG	AG	AG	AG
	AA	AA	AA	AA	AG	AG	AG	AG
	AA	AA	AG	AA	AG	AG	AG	
16415904								
6458	rs10181455		2	AA	AA	AA	AA	AA
	AA	AA	AA	AA	AA	AA	AA	AA
	AA	AA	AA	AA	AA	AA	AA	AA
	AA	AA	AA	AA	AA	AA	AA	AA
	AA	AA	AA	AA	AA	AA	AA	
60941805								
6459	rs10181492		2	AA	AG	AA	AG	GG
	AG	AG	AG	AG	AG	AA	AG	AA
	AA	AA	AG	GG	AG	AG	GG	GG
	AA	AA	AA	AA	AA	AG	AA	AG
	AG	GG	AG	AG	AA	AA	AG	
6675782								
6460	rs10181512		2	AA	AA	AA	AA	AA
	AA	AA	AA	AG	AA	AG	GG	GG
	GG	GG	AG	AG	AG	GG	AG	GG

AA AG AG GG AG AG AA AG
AG AA AG AG AA AA AA
188796294
6461 rs10181515 2 GG GG GG GG GG
GG GG GG AG AG GG GG GG
GG GG GG GG AG GG AG GG
AA AA AA AG GG AG GG AG
AG GG AG AG GG GG GG
227019461
6462 rs10181540 2 GG GG GG GG GG
GG GG GG GG GG GG GG GG
GG GG GG GG GG GG GG GG
GG GG GG GG GG GG GG GG
GG GG GG GG GG GG GG
86576825
6463 rs1018156 6 AC CC AC AA AC
AC AA AC AA CC AC AC CC
CC CC AC AC AA CC CC CC
CC CC CC CC CC CC CC CC
AA AC AC AA AC AA CC
39731701
6464 rs10181603 2 GG AG AG GG GG
GG GG GG GG GG GG GG GG
GG GG GG GG GG GG GG GG
AG GG GG AG GG GG GG GG
GG AG GG AG GG GG GG
40159265
6465 rs10181605 2 GG GG GG AG AG
GG AG GG GG GG GG AG AG
AG AG AG AG AG GG GG GG
GG GG GG AG GG GG GG GG
GG GG GG GG AG AA GG
216452392
6466 rs10181613 2 AA AA AA AA AA
AA AA AA AA AA AG AG AA
AA AA AA AG AA GG AG AG
AG AG AG AA AA AA AA AA
AA AA AA AA AA AA AG
232833186
6467 rs10181644 2 AA AA AA AA AA
AA AA AA AA AA AA AA AA
AA AA AA AA AA AA AA AA
AA AA AA AA AA AA AA AA
AA AA AA AA AA AA AA
110015309
6468 rs10181687 2 AA AA AA CC CC
CC CC AC CC AC AC CC CC
CC CC AC CC AC CC CC AC
AC AA CC AC AC CC CC AC
AC CC AC AC CC CC AC

28748916
6469 rs10181704 2 AC AA AC AC AC
CC AC AC AC AC CC AC CC
CC CC CC CC CC CC AC CC
AC AC CC AC AC AC CC CC
AC AC CC AC CC AC CC
9465687
6470 rs10181744 2 AA AA AA AA AA
AA AA AA AA AA AA AA AA
AA AA AA AA AA AA AA AA
AA AA AA AA AA AA AA AA
AA AA AA AA AA AA AA
14090052
6471 rs1018175 3 AA AG AA AG AA
AG AA AA AA AG AG AA AG
AG AA AA AA AA AG AG AA
AA AG AA AG AA AA AA AA
AA AA GG AG GG AG AG
43362597
6472 rs10181765 2 AA AA AA AA AA
AA AA AA AA AA AA AA AA
AA AA AA AA AA AA AA AA
AA AA AA AA AA AA AA AA
AA AA AA AA AA AA AA
100142456
6473 rs10181784 2 GG GG GG GG GG
GG GG GG GG GG GG GG GG
GG GG GG GG GG GG AG GG
AG AG GG AG GG AG AG AG
GG GG GG GG GG GG GG
7581220
6474 rs1018183 3 AA AA AA AG AA
AG AA AA AA AG AA AA AA
AA AA AA AA AA AA AA AA
AA AG AA AA AA AA AA AA
AA GG AG GG AA AG AA
11979988
6475 rs10181857
2 -- -- -- -- -- -- -- --
-- -- -- -- -- -- -- --
-- -- -- -- -- -- -- --
-- -- -- -- -- -- -- --
-- -- -- -- 224015791
6476 rs10181895 2 AG AG AA AA AG
AG AA AG AA AG GG GG GG
AG AG AG AG AA GG GG GG
AG GG AG GG GG AG AG GG
GG GG AG GG AG GG AA
75620383
6477 rs10181900 2 GG GG GG GG GG

	GG	GG	GG	GG	GG	GG	GG	GG
	GG	GG	GG	GG	GG	GG	GG	GG
	GG	GG	GG	GG	GG	GG	GG	GG
	GG	GG	GG	GG	GG	GG	GG	
56900064								
6478	rs10181932		2	--	AG	AG	AG	GG
	AG	GG	GG	GG	GG	AA	AA	AA
	AA	AA	AA	AA	AA	AG	AG	GG
	AA	AA	AA	AG	AA	AG	AA	AG
	AG	AG	GG	AG	GG	AG	AG	
199094168								
6479	rs10181968		2	AG	AA	AG	AG	AG
	AG	AA	GG	GG	GG	AG	AA	AA
	AA	AA	AA	AG	AG	AG	AG	AG
	AA	AA	AA	AA	AA	AA	AA	AA
	AA	AG	AG	AG	AG	AA	AG	
129215649								
6480	rs1018197		18	AA	AG	GG	GG	AG
	AG	GG	AG	GG	AG	AG	AA	AG
	AA	AG	GG	AG	GG	AA	AA	AA
	AG	AG	AG	AG	GG	AG	AG	GG
	AG	AA	AA	AA	GG	GG	AG	
1441750								
6481	rs10182021		2	GG	GG	GG	GG	GG
	GG	GG	GG	GG	GG	GG	GG	GG
	GG	GG	GG	GG	GG	GG	GG	GG
	GG	GG	GG	GG	GG	AG	AG	AG
	AG	GG	AG	GG	GG	GG	GG	
11854012								
6482	rs10182025		2	GG	GG	GG	GG	GG
	GG	GG	GG	GG	AG	GG	GG	AG
	GG	GG	GG	GG	GG	GG	AG	GG
	GG	GG	GG	GG	GG	GG	GG	GG
	GG	GG	GG	GG	AG	AG	GG	
107426060								
6483	rs10182108		2	AG	AG	AG	AG	AG
	GG	AG	AA	AA	AA	AG	AG	AG
	AA	AA	AG	GG	AA	AG	AG	AG
	AG	AG	AG	AG	AG	AA	AG	AA
	AA	AG	AG	AA	AG	AA	AG	
137506665								
6484	rs10182136		2	AG	GG	AG	AA	AG
	AG	AA	GG	AG	AG	GG	GG	GG
	AG	AG	AG	AG	AG	AA	AA	AA
	AG	AA	AG	AG	AA	AG	AG	AA
	AA	AG	AG	GG	AG	AA	GG	
173179471								
6485	rs10182147		2	CC	CC	CC	CC	CC
	CC	CC	CC	CC	CC	CC	CC	CC
	CC	CC	CC	CC	CC	CC	CC	CC

CC	CC	CC	CC	CC	CC	CC	CC
CC	CC	CC	CC	CC	CC	CC
88470966
6486	rs10182250	2	AG	AA	AG	GG	AG
GG	AG	GG	GG	GG	GG	GG	GG
GG	GG	AG	GG	AG	AG	AG	AG
AG	AG	GG	GG	GG	GG	GG	GG
GG	GG	GG	GG	AG	AG	AG
109875980
6487	rs10182302	2	AC	AA	AC	CC	AA
AC	AC	CC	CC	CC	AC	AC	AC
AC	AC	CC	CC	CC	CC	CC	AC
AC	CC	CC	AC	AC	CC	AC	CC
CC	CC	AC	CC	CC	CC	AC
42458640
6488	rs10182313	2	GG	GG	GG	GG	GG
GG	GG	GG	GG	GG	GG	GG	GG
GG	GG	GG	GG	GG	GG	GG	AG
GG	GG	GG	GG	GG	AG	GG	GG
AG	GG	GG	GG	GG	GG	GG
16733412
6489	rs10182324	2	GG	AG	AG	GG	GG
GG	GG	GG	GG	GG	GG	GG	GG
GG	GG	GG	AG	GG	GG	GG	GG
GG	GG	GG	GG	GG	GG	GG	GG
GG	GG	GG	GG	GG	GG	GG
59047973
6490	rs10182442	2	AG	AA	AG	AA	AG
AA	AG	AA	AA	AA	AA	AA	AA
AA	AA	AG	AA	AA	AA	AG	AG
AG	AA	AG	AG	AA	AG	AA	AG
AG	AA	AA	AA	AA	AA	AA
2550499
6491	rs10182443	2	AG	GG	AG	AA	AA
AA	AA	AG	AG	AA	AG	AA	AA
AA	AA	AG	AG	AA	AG	AG	GG
AA	AA	AG	AG	AA	AA	AA	AA
AA	AA	AA	AA	AG	AA	AG
107411525
6492	rs10182473	2	AG	AA	AG	AA	AA
AA	AA	AA	AA	AA	AA	AA	AA
AA	AA	AG	AG	AG	AA	AG	AA
AA	AA	AA	AA	AG	AA	AG	AA
AG	AG	AA	AA	AA	AA	AG
166873723
6493	rs10182489	2	AG	GG	AG	AA	AG
AA	AG	AG	AG	AG	AG	AG	AA
AA	AA	AA	AG	AA	AG	AG	AA
AG	GG	GG	AG	AA	GG	AG	AG
GG	AA	AG	AG	GG	GG	AG

43829540
6494 rs10182513 2 AA AG AA AA AA
AA AA AA AA AA AA AA AA
AA AG AA AG AG AG AG AG
AG AG AG AA AA AA AA AA
AA AA AA AA AA AA AA
200957953
6495 rs10182533 2 GG GG GG GG GG
GG GG GG GG GG GG GG GG
GG GG GG GG GG GG AG GG
AG AA AA AG AG GG GG AG
GG GG GG GG AG GG AG
152672843
6496 rs1018254 9 GG AG AG AG AG
AA GG AA AA AG GG GG GG
GG GG AG AG AG GG GG AG
AA AG AA AG AA AG AA AA
AG GG GG GG GG AG GG
30143230
6497 rs10182590 2 AA AA AA AG AA
AG AA AA AA AA AA AA AA
AA AA AA AA AG AA AA AA
AA AA AA AA AA AA AA AA
AG AA AG AA AG AA AG
25584582
6498 rs10182649 2 AA AA AA AA AA
AA AA AA AA AA AA AA AA
AA AA AA AA AA AA AA AA
AA AA AA AA AA AA AA AA
GG AA AA AA AG AG AG
68109558
6499 rs10182687 2 GG GG GG GG GG
GG GG GG GG GG GG GG GG
GG GG GG GG GG GG GG AG
GG GG GG GG GG GG GG GG
GG GG AG AG GG GG GG
84712523
6500 rs10182729 2 GG GG AG GG GG
GG GG AA AA AA AA AA AA
AG AG AA AG AA AG AG GG
AA AG AA AA GG AG AG GG
AG AG GG GG GG AG AG
50871418
6501 rs10182735 2 GG GG GG AG AG
GG AG GG GG GG GG GG GG
GG GG GG GG GG GG GG GG
GG GG GG GG AG GG GG GG
GG GG GG GG GG GG GG
206690019
6502 rs10182738 2 AG GG GG GG GG

	GG	GG	AA	AG	AA	GG	AG	AG
	AA	AA	AG	GG	AG	AA	AA	AG
	AA	AA	AA	AA	AG	AG	GG	AA
	AG	AG	GG	AG	GG	GG	AG	
104243342								
6503	rs10182783		2	GG	GG	GG	GG	GG
	GG	GG	AG	AG	AG	GG	GG	GG
	GG	GG	GG	GG	GG	AG	GG	GG
	GG	GG	GG	GG	GG	GG	GG	GG
	GG	GG	GG	GG	GG	GG	AG	
13021683								
6504	rs10182802		2	GG	GG	GG	GG	AG
	AG	GG	GG	GG	GG	AG	AG	AG
	AG	AG	GG	GG	GG	AG	AG	GG
	GG	GG	GG	AG	GG	GG	GG	GG
	AG	AG	AG	AG	GG	GG	GG	
206088211								
6505	rs1018284		15	AA	AG	AG	AG	AG
	GG	AA	AA	AA	AA	GG	AA	AG
	AG	AG	AA	AA	AA	GG	AG	GG
	GG	GG	GG	GG	GG	AG	AG	AG
	AA	GG	GG	GG	AG	AG	AG	
66564114								
6506	rs10182844		2	GG	AG	GG	GG	GG
	GG	GG	AG	GG	AG	GG	GG	GG
	GG	GG	GG	GG	GG	GG	GG	GG
	AG	AG	AG	AG	AG	GG	AG	AG
	AG	GG	GG	GG	GG	GG	AG	
231757630								
6507	rs10182846		2	GG	AG	AG	AG	AA
	AG	AG	AA	AA	AA	AA	AG	AG
	AG	AG	AG	GG	AG	GG	AG	AG
	AA	AA	AG	AG	AG	AG	AA	GG
	GG	AA	GG	AG	AG	AG	AA	
106637427								
6508	rs10182928		2	GG	GG	GG	AG	GG
	AG	GG	GG	AG	AG	GG	GG	GG
	GG	AG	AG	GG	GG	GG	GG	GG
	GG	GG	AG	AG	AA	AA	AA	AA
	GG	GG	AG	AG	AG	GG	AG	
200452591								
6509	rs10182941		2	AG	GG	GG	GG	GG
	AG	GG	GG	GG	GG	AG	GG	AG
	GG	GG	GG	GG	GG	GG	GG	AG
	AG	GG	AG	AG	AA	GG	AG	AG
	GG	GG	AG	GG	GG	GG	GG	
98533752								
6510	rs1018298		12	AA	AA	AA	AA	AA
	AA	AA	AG	AG	AG	AA	AG	AA
	AG	AA	AG	AG	AG	AA	AA	AA

AA AA AA AA AA AA AA AA
AA AA AA AA AA AG AA
19182772
6511 rs10183017 2 AC AC AC AC AC
CC AA AC AC AC AC CC CC
AA AA AA AA AC AA AA AA
AA AC AA AC AC AA AC AC
AA AC CC CC AA AA AA
56546483
6512 rs10183022 2 AG GG GG GG GG
GG GG AG AG AG AG AG GG
GG GG AA AA AG AG GG AG
AA AA AA AA GG AG AG AG
AG GG AG GG AA AG AG
237481969
6513 rs10183029 2 GG GG GG GG GG
GG GG GG GG GG GG GG GG
GG GG GG GG GG GG GG GG
GG GG GG GG GG GG GG GG
GG GG GG GG GG GG GG
13310457
6514 rs10183044 2 AA AG GG AG AA
AA AG AG AG AG AG AA AA
AG AG AG GG AG AG AG AG
GG AG GG AG AG AA AG AG
AG AG AG AA GG AG GG
68222743
6515 rs1018306 1 AG GG GG AG AG
GG AA AG AA AA AA AA AG
AG GG AA AA AA AA AA AA
AA AG AA GG AG AG AA AG
AG GG AG GG AA AA AG
4567165
6516 rs10183071 2 AA AA AA AA AA
AA AA AA AA AA AG AG GG
AG AG AA AA AG AG AG AA
AA AA AA AA AA AA AA AA
AG AA AA AA AA AG AG
154171376
6517 rs10183073 2 CC CC CC AC AC
AA CC CC CC CC CC CC CC
CC CC CC CC CC CC AC CC
AC CC CC AC CC CC CC CC
CC CC AC CC CC CC CC
146408408
6518 rs1018308 6 AG AG GG GG AA
AG AG GG GG GG AG AG AG
AG AG AG GG GG AG AG GG
AA AA AA AA AG GG GG GG
GG GG AG AG AA AA AA

93562872							
6519	rs10183087	2	AA	AA	AA	AA	AC
	AC	AA	AC	AA	AA	AA	AC	AA
	AA	AA	AA	AC	AC	AC	AA	AC
	AA	AA	AA	AA	AC	AC	AC	CC
	AA	CC	AA	AC	CC	CC	CC	
204824324							
6520	rs10183112	2	CC	AC	AC	CC	AC
	AC	CC	CC	CC	AC	AC	AC	AC
	CC	CC	CC	AC	CC	CC	CC	CC
	CC	CC	CC	CC	CC	CC	CC	CC
	CC	AC	CC	CC	CC	CC	CC	
38445779							
6521	rs10183136	2	GG	GG	GG	GG	GG
	AG	GG	AG	GG	GG	AG	AG	AG
	GG	GG	AG	AA	AG	GG	GG	GG
	AG	AA	AA	AG	AG	AA	AA	AG
	AA	GG	GG	GG	AA	AG	AG	
154619808							
6522	rs1018317	2	GG	AG	AG	AG	AA
	AG	AG	AG	AG	GG	AG	AG	AG
	GG	GG	AG	AG	AA	AA	AG	AA
	AG	AA	AG	AG	GG	AG	AG	GG
	AG	AA	AG	AA	AA	AA	AG	
113463525							
6523	rs10183172	2	AC	AA	AC	AC	AA
	AC	AC	AC	AA	AA	AC	AA	AA
	AA	AA	AC	AA	AA	AC	AC	AC
	AA	AA	AA	AA	AA	AC	AA	AA
	AA	CC	AA	AC	AC	AC	AA	
241995014							
6524	rs10183203	2	AG	AG	AA	AG	GG
	GG	AG	GG	AG	GG	AG	GG	AG
	GG	GG	GG	GG	GG	GG	GG	AG
	GG	AG	GG	GG	AG	AG	AA	GG
	GG	AA	AG	AA	AG	AG	GG	
102095080							
6525	rs10183217	2	AA	AA	AA	AA	AA
	AA	AA	AA	AA	AA	AA	AA	AA
	AA	AA	AA	AA	AA	AA	AA	AA
	AA	AA	AA	AA	AA	AA	AA	AA
	AA	AA	AA	AA	AA	AA	AA	
75997264							
6526	rs10183220	2	AG	GG	AG	GG	GG
	GG	GG	GG	AG	GG	GG	GG	GG
	AG	GG	GG	GG	GG	AG	AG	GG
	AG	GG	AG	AG	GG	GG	GG	GG
	GG	AG	GG	GG	AG	AG	AG	
20746932							
6527	rs1018324	15	AC	AC	CC	CC	CC

CC CC AC CC AA AA AA AA
AC AA AC CC AC AC CC AA
CC CC CC CC AA CC AC AC
CC AC CC CC AC CC AA
36481099
6528 rs10183242 2 GG GG GG GG GG
AG GG GG GG GG GG AG GG
AG AG AG AG GG AG AG AA
AG AG AG AG GG AG AG GG
AA GG AG AG AG AG AA
106266007
6529 rs10183260 2 AA AA AA AA AA
AA AA AA AA AA AA AA AA
AA AA AA AA AA AA AA AA
AA AA AA AA AA AA AA AA
AA AA AA AA AA AA AA
175325449
6530 rs1018330 9 AC AC AA AA AC
AC AA AA AC AC AA AA AC
AC AA AA AA AA AA AC AA
AA AA AA AA AC AC AA AC
AA AC AA AA AA AA AC
139980385
6531 rs10183301
2 -- -- -- -- -- -- -- --
-- -- -- -- -- -- -- --
-- -- -- -- -- -- -- --
-- -- -- -- -- -- -- --
-- -- -- -- 238480686
6532 rs10183305 2 AA AA AA AA AA
AA AA AA AA AA AA AA AA
AA AA AA AA AA AA AA AA
AA AA AA AA AA AA AA AA
AA AA AA AA AA AA AA
197460984
6533 rs10183344 2 AG AA AG GG AA
AG AG AG AG GG AG AG AG
AG AG GG AG GG GG AA AG
AG AG AG AG AG GG AG GG
GG GG AG GG AG GG AA
42423645
6534 rs10183359 2 GG GG GG AG AA
AG AA GG GG GG GG GG GG
GG GG AG AG AA GG GG GG
AG AG GG AG AG AG AA AA
AA AG AA AG AG AG GG
10733044
6535 rs1018340 3 AA AA AA AG AA
AG AA AA AA AA AA AA AA
AG AG AA AA AA GG GG AG

AG AA AG AA AA AG AG AG
AG AG AA AA AG GG AA
70918127
6536 rs10183410 2 AA AA AA AG AA
AG AA AA AG AA AA AA AA
AG AG AA AA AA AG AG AA
AG AG AG AG AG AA AG AA
AG AG AG AG AG GG AG
84006399
6537 rs1018342 2 AA AC CC AC AC
CC AA AA AA AA AA AA AA
AA AA AA AA AA AC AC AA
AA AA AA AA AC CC AC CC
AA AC AA AC AC CC AA
73471653
6538 rs1018343 3 GG GG GG AG AA
AA AG GG GG GG AG AG AA
AG GG GG GG GG AG AG GG
GG GG AG AG AG GG AG AG
GG GG AA AG AG GG AG
189732606
6539 rs10183434 2 AG GG AG AG GG
AG GG GG AG AG AA AA AA
AA AA AG AG AG AG GG AG
GG GG GG AG GG AG AG GG
AA GG AA AG GG AG GG
210195717
6540 rs10183501 2 AA AG AA GG AG
AG AG AG AG AG AG AA AG
AG AG -- -- AA AG AG GG
GG AG GG GG GG GG GG GG
AG AG GG GG GG AG GG
100534633
6541 rs10183541 2 AA AA AA AA AA
AA AA AG AA AA AA AA AA
AA AA AA AA AA AA AA AA
AA AG AA AA AA AA AA AA
AG AA AA AA AA AA AA
50716768
6542 rs10183551 2 AG AG AA AG GG
AG AG AG AA AA AA AA AA
AA AA AA AA AA AG AG AA
AA AA AA AA AG AA AA AG
AA AA AG AA AA AA AA
166838735
6543 rs10183565 2 GG GG GG AG AG
AG GG AA GG AG GG GG AG
AG AG GG GG GG GG GG GG
GG GG GG GG GG GG GG GG
GG AG AG AG GG AG AG

150033334
6544 rs1018361 2 AG AA AG AG GG
AG AG AG AA AA AA AA AA
AA AG AA AA AA AG AA AA
GG GG AG AA AG AA AA AA
AG AG AA AA AA AA AA
204684835
6545 rs10183675 2 GG GG GG GG GG
GG GG GG GG GG AG AG GG
GG GG GG GG GG GG GG AG
GG GG GG GG GG GG GG GG
GG GG AG GG AG AA GG
176070435
6546 rs10183727 2 AC AC AC CC AC
AC AC AC CC AC CC AC AC
AA AA CC CC CC CC AC AC
CC CC CC CC CC AC CC AC
AA CC AA AC AA AA AA
1619121
6547 rs10183848 2 GG GG GG GG GG
GG GG GG GG GG AG AG AG
GG GG AG AG GG GG AG AG
GG GG GG GG GG AG AG GG
GG GG GG GG GG GG GG
127359823
6548 rs1018391 1 AA AG AG AG GG
GG AG AG GG AG GG GG GG
GG AG AA AA AA AG AA GG
AA AG AA AG GG AA AG AG
AG AA AA AA AA AA AA
171340430
6549 rs10183942 2 AA AA AG AG AA
AA AG AA AA AA AA AA AA
AG AG AA AG AG AG AG AA
AG AG AG AG AA AA AA AA
GG AG AA AA AA AA AA
34554042
6550 rs10183963 2 CC CC CC CC CC
CC CC CC CC CC CC CC CC
CC CC CC CC CC CC CC CC
CC CC CC CC CC CC CC CC
CC CC CC CC CC CC CC
164121221
6551 rs10183968 2 GG GG GG GG GG
GG GG GG GG GG AG GG GG
AG AG AG GG AA GG GG GG
GG AG GG GG GG GG GG GG
GG GG AG GG GG GG --
45319604
6552 rs1018401

6	--	--	--	--	--	--	--	--
	--	--	--	--	--	--	--	--
	--	--	--	--	--	--	--	--
	--	--	--	--	--	--	--	--
	--	--	--	--	22846052			
6553	rs1018405		20	AG	GG	GG	GG	GG
	GG	GG	GG	GG	AG	GG	GG	GG
	GG	GG	AG	GG	GG	AG	AG	GG
	GG	GG	GG	GG	AA	GG	AG	AG
	GG	AA	AG	AA	AG	AA	AG	
29889768								
6554	rs10184085		2	GG	AG	GG	GG	GG
	GG	GG	GG	GG	GG	GG	GG	GG
	GG	GG	GG	GG	GG	GG	GG	AG
	GG	GG	GG	GG	GG	GG	GG	GG
	GG	GG	GG	GG	GG	GG	GG	
176692909								
6555	rs10184153		2	AA	AA	AA	GG	AG
	AG	GG	AA	AG	AG	AA	AA	AA
	AA	AA	AA	AA	AA	AA	AG	AA
	AG	AA	AG	AA	AA	AG	AG	AA
	AA	AA	AA	AA	AA	AG	AA	
182220184								
6556	rs1018418		1	AA	AA	AA	AA	AA
	AA	AA	AA	AA	AA	AA	AA	AA
	AC	AA	AA	AA	AA	AA	AA	AC
	AC	AC	AC	AA	AC	AC	AC	AA
	AA	AA	AA	AA	AC	AC	AC	
69174297								
6557	rs10184234		2	GG	GG	GG	GG	GG
	GG	GG	GG	GG	GG	GG	GG	GG
	GG	GG	GG	GG	GG	GG	GG	GG
	GG	GG	GG	GG	GG	GG	GG	GG
	GG	GG	GG	GG	GG	GG	GG	
232523843								
6558	rs10184246		2	AA	AG	GG	AG	GG
	GG	AG	AA	AA	AA	AG	AA	AG
	AA	AA	GG	AG	AG	AG	AG	AG
	AG	GG	AG	AG	AG	AG	AG	AG
	AG	GG	GG	GG	AA	AA	AA	
86341328								
6559	rs10184259		2	GG	GG	GG	AG	GG
	GG	AG	GG	GG	AG	GG	GG	AG
	GG	GG	AG	GG	AG	AG	GG	GG
	GG	GG	GG	GG	GG	AA	AG	AG
	GG	GG	AG	AG	GG	AG	GG	
113838628								
6560	rs10184263							
2	--	--	--	--	--	--	--	--
	--	--	--	--	--	--	--	--

-- -- -- -- -- -- -- --
-- -- -- -- -- -- -- --
-- -- -- -- 51663763
6561 rs10184287 2 AG GG AG GG GG
GG GG GG GG GG GG GG GG
AG AG GG GG GG GG GG GG
GG GG GG GG GG GG GG GG
GG GG GG GG GG GG GG
55726222
6562 rs10184288 2 GG GG GG GG GG
GG GG GG GG GG GG GG GG
GG GG GG GG GG GG GG GG
GG GG GG GG GG GG GG GG
GG GG GG GG GG GG GG
99327591
6563 rs10184380 2 AC CC AC CC CC
CC AC AC AC AC CC AC AC
AA AA CC CC CC CC CC CC
CC AC CC AC CC CC CC CC
CC AC AC AC CC CC CC
79914884
6564 rs10184386 2 AA AA AA AA AA
AA AA AA AA AA AA AA AA
AA AA AA AA AA AA AA AA
AA AA AA AA AA AA AA AA
AA AA AA AA AA AA AA
203137995
6565 rs1018443 20 GG GG GG AG GG
AG GG AG GG AG GG AG AG
GG AG GG GG GG AA AG AG
GG AG GG AG GG GG GG GG
AG AG AG AA GG GG GG
8626903
6566 rs10184453 2 CC AC CC AC AA
AC AA CC CC CC AC CC CC
CC CC AC CC AC AA AA AA
CC AC CC CC CC CC CC CC
AC AC CC AC AC AC CC
23130359
6567 rs10184457 2 AA AG AA AG AA
AA AG AG AA AG AG AG AA
AG AG AA AG AG GG GG AG
AG GG AG AG AA AG AA AG
GG AA AA AA AG GG AG
98579729
6568 rs10184640 2 GG AG GG AG AA
AA AG AA AA AA AA AA AA
AA AA AG AG AG AG AG AA
AA AA AA AA AA AG AG AA
AA AA AA AA AG AG AA

229590473
6569 rs1018466 14 AC AA AC AC AC
CC AA AA AC AA CC AC AC
AC CC AA AA AA AC AC AC
AC AC AC AC CC AC AC CC
AA AC AC AC AC AC AC
37123250
6570 rs10184663 2 AG GG AG AG AG
AA GG AG AA AA AA AA AA
AA AA AA AA AA AG AG AG
GG GG GG AG GG AA AG AG
AA AA GG AG AA AG AA
228277430
6571 rs10184750 2 AA AA AA AA AA
AA AA AA AA AA AA AA AA
AA AA AA AA AA AA AA AA
AA AA AA AA AA AA AA AA
AA AA AA AA AA AA AA
145435967
6572 rs10184764 2 AG AA AA AA AA
AA AA AG AG AA AA AA AA
AA AA AG AA AA AG AA AG
AG AG AG AG AA AA AA AA
AG AA AG AA GG AG AG
237484856
6573 rs10184780 2 GG GG GG GG GG
GG GG GG GG GG GG GG GG
GG GG GG GG GG GG GG GG
GG GG GG GG GG GG GG GG
GG GG GG GG AG GG AG
20054392
6574 rs10184788 2 AG GG GG AG AA
AG AA AA AA AA AG AA AG
AA AA AG AG GG AA AA AA
AA AG AA AA GG AG AG GG
AG AG AG AG AA AA AA
88413825
6575 rs10184838 2 AA AC AA CC CC
AC CC AC AC AC AC AA AC
AC AC AA AC AC CC AA AC
AC AC AC AA AC AA AA AA
AC AC AC AC AC AC AC
230059595
6576 rs10184847 2 AA AA AA AA AA
AA AA AA AA AA AA AA AA
AA AA AA AA AA AA AA AA
AA AA AA AA AA AA AA AA
AA AA AA AA AA AA AA
203344218
6577 rs10184858 2 AG GG AG AG AG

	AG	AG	AG	GG	GG	AG	AG	AA
	AG	AG	AA	AG	AA	AG	AG	AG
	AG	AA	AA	AG	AG	AA	AG	AA
	AA	AG	AG	AG	AA	AA	AG	
147465195								
6578	rs10184879		2	GG	AG	AA	AG	AA
	AG	AA	GG	AG	GG	GG	AG	AG
	AG	AG	AA	AG	AG	AG	AG	AA
	AA	AG	AA	AA	GG	AG	AG	GG
	AG	AG	AG	AG	AG	GG	AA	
86818630								
6579	rs1018488		22	AA	AA	AG	AA	AA
	AA	AA	AA	AA	AA	AA	AA	AA
	AA	AA	AA	AA	AA	AA	AA	AA
	AA	AA	AA	AA	AA	AA	AA	AA
	AA	AA	AA	AA	AA	AA	AA	
31992376								
6580	rs1018490		22	AC	AA	AA	AA	AC
	AC	AC	CC	CC	AC	AA	AA	AC
	AC	AC	AA	AA	AA	AC	AC	AC
	AA	AA	AC	AA	AC	AA	AC	AC
	AC	AC	CC	CC	AA	AC	AA	
31996625								
6581	rs10184902		2	AA	AG	AA	AA	AA
	AG	AA	GG	AA	GG	AA	AA	AG
	AG	AG	GG	GG	GG	GG	AG	GG
	AA	AA	AG	AA	AA	GG	AG	AG
	GG	AG	AA	AA	AG	AG	AG	
3922214								
6582	rs1018491		20	AG	AG	AG	AG	GG
	AG	GG	GG	GG	GG	AA	AA	AA
	AA	AA	AG	GG	GG	AA	AG	AA
	AG	AA	AA	AG	AA	AG	AA	AA
	AG	GG	AA	AG	AG	AA	AG	
3701132								
6583	rs1018493		20	AG	AG	AG	AG	GG
	AG	GG	GG	GG	GG	AA	GG	AG
	AG	AG	AG	GG	GG	AA	AA	AA
	AG	AA	AA	AG	AA	AG	AA	AA
	AA	GG	AA	AG	AG	AA	AG	
3682011								
6584	rs10184938		2	GG	GG	GG	GG	GG
	GG	GG	GG	GG	GG	AA	AG	AA
	AA	AA	GG	AG	GG	GG	GG	GG
	GG	AG	AG	GG	GG	GG	GG	GG
	AG	GG	GG	GG	GG	GG	GG	
9913006								
6585	rs10185002		2	AC	CC	CC	CC	CC
	CC	CC	CC	CC	CC	CC	CC	CC
	CC	CC	CC	CC	CC	CC	CC	CC

	CC	CC	CC	CC	CC	CC	CC	CC
	CC	CC	CC	CC	CC	CC	CC	
97249593								
6586	rs1018505		2	AA	AA	AA	AA	AA
	AA	AA	AA	AA	AG	AA	AA	AA
	AA	AA	AG	AG	AG	AA	AA	AG
	AA	AA	AG	AA	AG	AA	AG	AA
	AG	AA	AG	AA	AA	AA	AA	
31587235								
6587	rs10185068		2	AG	AG	AG	AA	AG
	AG	AG	GG	GG	GG	AG	GG	GG
	GG	GG	GG	GG	GG	GG	AA	GG
	AG	AG	AG	GG	AG	GG	GG	GG
	AG	AG	GG	GG	GG	GG	AG	
192924987								
6588	rs10185085		2	AA	AG	GG	AG	GG
	AG	GG	AG	AG	AG	AA	GG	AG
	AG	AG	GG	AG	AG	AG	AG	GG
	GG	GG	GG	GG	AG	GG	AG	GG
	AG	GG	AG	AG	GG	GG	GG	
86660080								
6589	rs1018509		14	GG	GG	GG	AG	AG
	GG	AA	GG	AA	GG	GG	AA	AG
	AG	GG	GG	GG	GG	AA	AG	AG
	AG	AA	AA	AA	AA	AG	AG	AA
	AA	AG	AG	AG	GG	GG	AG	
29064386								
6590	rs10185201		2	AG	GG	GG	GG	GG
	--	GG	AG	GG	GG	AG	AG	GG
	AG	AG	AG	GG	AG	AG	AG	AG
	GG	GG	GG	GG	GG	AA	AG	AG
	--	GG	GG	GG	GG	GG	GG	
46122164								
6591	rs10185213		2	AA	AC	CC	AA	AC
	AC	AA	AA	AA	AA	AA	AA	AC
	AA	AA	AA	AA	AA	AA	AA	AA
	AA	AA	AA	AA	CC	AA	AC	AC
	AA	AA	AA	AA	AA	AA	AA	
49000874								
6592	rs1018527		3	GG	AG	GG	AA	AG
	AG	AG	AA	AG	AG	AG	AG	AG
	GG	GG	GG	GG	GG	AG	AG	GG
	AG	AA	AG	AA	AG	GG	AG	AG
	GG	GG	AG	GG	AA	AA	AA	
197080008								
6593	rs10185275		2	AC	AA	AC	CC	CC
	AC	CC	CC	CC	CC	CC	CC	CC
	AC	AC	AC	AC	AC	CC	CC	CC
	AC	CC	AC	AC	AC	CC	AC	CC
	AC	CC	AC	AC	CC	CC	AC	

122252266
6594 rs10185326 2 AG AA AA AG GG
AG AG AA AA AA AG GG GG
AG AG AG AG AA AA AA AA
AG GG AG AG GG AG AG GG
AG AA AA AA AG AG AA
111632235
6595 rs10185339 2 GG GG GG GG GG
GG GG GG GG GG GG GG GG
GG GG AG GG GG GG GG GG
GG GG GG GG GG GG GG GG
GG GG GG GG GG GG GG
15944021
6596 rs10185344 2 AA AA AA AA AA
AA AA AA AA AA AA AA AA
AA AA AA AA AA AA AC AA
AC AC AA AC AC AC AC AC
AA AA AA AA AC AA AC
33571790
6597 rs10185359 2 GG GG GG AG AG
GG AA GG AG GG AG GG GG
GG GG GG GG GG AA GG AG
GG GG GG GG GG AG AG AG
GG GG GG GG GG AG AG
145155731
6598 rs10185384 2 AG AG AG AA AA
AA AA AA AA AA GG AG AG
GG GG AA AA AA AA AG AA
AG GG GG AG AG GG AG GG
AA -- -- -- -- -- AA
142196011
6599 rs10185403 2 AA AA AA AA AA
AA AA AA AA AA AA AA AA
AA AA AA AA AA AA AA AA
AA AA AA AA AA AG AG AG
AA AA AA AA AG AG AG
11206343
6600 rs10185448 2 AA AA AA AA AA
AA AA AA AA AA AA AA AA
AA AA AA AA AA AA AA AA
AG AG AG AG AA AA AA AA
AA AA AA AA AA AA AA
232667302
6601 rs10185452 2 AA AG GG AG GG
GG AG AA AA AA AG AA AG
AA AA GG AG AG AG AG AG
AG GG AG AG AG AG AG AG
AG GG AG GG AA AA AA
86241607
6602 rs10185472 2 AA AA AA AA AA

	AA	AA	AA	AA	AA	AA	AA	AA
	AG	AG	AA	AA	AG	AG	AG	AG
	AG	AA	GG	AG	AA	AA	AA	AA
	AG	AA	AA	AA	AA	AA	AA	
28151354								
6603	rs10185490		2	AA	AG	GG	AA	GG
	AG	AG	AG	AG	AG	AG	AG	AG
	GG	GG	AG	AG	AG	GG	GG	GG
	AG	GG	AG	GG	GG	GG	GG	GG
	AG	AG	AA	AG	GG	GG	GG	
71166840								
6604	rs10185554		2	AG	GG	AG	AG	AG
	AA	GG	GG	GG	GG	GG	GG	GG
	GG	GG	GG	GG	GG	GG	GG	GG
	AA	GG	AA	AG	GG	GG	GG	GG
	GG	GG	GG	GG	GG	GG	AG	
174560184								
6605	rs10185589		2	AA	AA	AA	AG	AG
	AG	AA	AG	GG	AG	GG	AG	GG
	AA	AA	AA	AA	AA	AG	GG	AA
	AA	AA	AA	AA	AG	AG	GG	AG
	AA	AA	AA	AA	AA	AA	AA	
144587571								
6606	rs10185605		2	AG	GG	AG	GG	GG
	GG	GG	GG	GG	GG	AA	GG	AG
	AG	AG	AG	AA	AG	GG	GG	GG
	GG	GG	GG	GG	GG	GG	GG	GG
	GG	GG	GG	GG	GG	GG	GG	
10656284								
6607	rs10185631		2	AA	AA	AA	AA	AA
	AA	AA	AA	AA	AA	AA	AA	AA
	AA	AA	AA	AA	AA	AA	AA	AA
	AA	AA	AA	AA	AA	AA	AA	AA
	AA	AA	AA	AA	AA	AA	AA	
234740490								
6608	rs10185640		2	GG	GG	GG	GG	GG
	GG	GG	GG	GG	GG	GG	GG	GG
	GG	GG	GG	GG	GG	GG	GG	GG
	GG	GG	GG	GG	GG	GG	GG	GG
	GG	GG	GG	GG	GG	GG	GG	
146405634								
6609	rs10185660		2	AA	AA	AA	AA	AA
	AA	AA	AA	AA	AA	AG	AA	AA
	AA	AA	AG	AA	AG	AA	AA	AG
	AA	AA	AA	AA	AA	AA	AA	AA
	AA	AG	AA	AG	AG	GG	AA	
88463237								
6610	rs10185662		2	GG	GG	GG	GG	GG
	GG	GG	GG	GG	GG	GG	GG	GG
	GG	GG	GG	GG	GG	GG	GG	GG

GG GG GG GG GG AG AG GG
GG GG GG GG AG AG AG
1964906
6611 rs1018567 9 AG AG AA AG AA
AG AA AG AA AG AG AG AG
AA AG AA AG AG AG AG AG
AA AG AA AA GG AA AG AG
GG AA AG AG AG AA GG
860584
6612 rs10185678 2 GG GG GG GG GG
GG GG AG AG GG GG GG AG
AA AA AG AA AA AG AA GG
AG AG AG AA AG AG AG GG
AA GG AG GG GG GG AG
179732050
6613 rs10185692 2 GG GG GG GG GG
GG GG GG GG GG GG GG GG
GG GG GG GG GG GG GG GG
GG GG GG GG GG GG GG GG
GG GG GG GG GG GG GG
146921487
6614 rs10185725 2 AG GG AG GG GG
GG AG AA AG AG AG GG GG
AG AG AA AA AA GG GG GG
AG GG AG GG GG GG GG GG
AG AG AG AG AG GG AA
155463709
6615 rs10185841 2 GG GG GG GG GG
GG GG GG AG GG AG AG AA
AG AG GG GG AG GG GG GG
GG GG GG GG GG GG GG GG
GG GG GG GG GG GG GG
146261295
6616 rs10185855 2 AA AA AA AA AA
AA AA AG GG AG AG AG GG
AG AG AG AG AA AG AG AA
AG AG AG AG AG AA AA AG
AG AA AA AA AG GG AA
101642260
6617 rs1018593 14 GG GG GG AG AG
AG AG AG AG AG GG AA AG
AG AG AG GG GG AG AG GG
AG GG AG AG GG AG AG GG
AG AA GG AG GG AG AG
89610043
6618 rs10185931 2 AG AA AA AA AA
AA AA AA AA AA AA AA AA
AA AA AA AA AA AA AA AA
AA AA AA AA AA AA AA AA
AG AA AA AA AA AA AA

74961853
6619 rs10185935 2 GG GG GG GG GG
GG GG GG GG GG GG GG GG
GG GG GG GG GG GG GG GG
GG GG GG GG GG AG AG GG
AG GG GG GG GG GG GG
170299584
6620 rs10186002 2 AC CC AC CC CC
CC CC CC CC CC CC AC AC
CC CC AC CC CC AC AC AC
CC CC CC CC CC CC CC CC
CC CC CC CC CC CC CC
14100897
6621 rs1018603 10 AC AC AA AA AA
AA AA AA AA AA AA AA AA
AA AA AC AA AC AC CC AC
AC AC AA AC AC AA AA AC
AC AC AC AC AA AA AA
50728371
6622 rs10186133 2 AA AA AA AC AA
AA AC AA AA AC AC AA AC
AA AA AC AA AC AC AA AA
AA AA AA AA AA CC AC AC
AA AA AC AC AA AC AA
113836944
6623 rs10186235 2 GG GG GG GG GG
GG GG GG GG GG AA AA AA
AG AG AG AG AA GG GG GG
AG GG AG AG GG GG GG GG
AG GG GG GG GG GG GG
15884555
6624 rs1018627 1 GG AG GG AG AG
AG AA AA AG AG AA AA AG
AG AG AA AG AG GG GG AG
AA AG AG AA GG AG GG GG
GG AG GG GG AA AG AG
49484622
6625 rs10186301 2 AA AA AA AA AA
AA AA AA AA AA AA AA AA
AA AA AA AA AA AA AA AA
AA AA AA AA AA AA AA AA
AA AA AA AA AA AA AA
203147560
6626 rs10186317 2 AA AA AA AA AA
AA AA AA AA AA AA AA AA
AA AA AA AA AG AG AG AA
AA AG AA AG AA AG AA AG
AA AG AA AA AG AG AG
172955011
6627 rs10186325 2 AC AA AA AA AA

	AA	AA	CC	CC	CC	AA	AA	AA
	CC	CC	AC	AC	AC	AC	AC	CC
	AA	AC	AA	AC	AA	AA	AA	AA
	AC	AC	AC	AA	AA	AA	AC	
61757721								
6628	rs1018634		10	GG	GG	GG	GG	AG
	AG	AG	AG	AG	AG	AA	AG	AG
	AG	AG	GG	GG	GG	AG	AA	AG
	AG	AG	GG	GG	AG	GG	GG	AG
	AG	AG	AG	AG	GG	GG	AG	
95401292								
6629	rs10186364		2	AG	GG	GG	AG	AG
	AG	AG	GG	GG	GG	GG	GG	GG
	GG	GG	GG	GG	GG	GG	GG	GG
	GG	GG	AG	AG	AG	GG	GG	AG
	AA	AG	GG	GG	AA	AA	AA	
119006422								
6630	rs10186377		2	GG	GG	GG	GG	GG
	GG	GG	GG	GG	GG	GG	GG	GG
	GG	GG	GG	GG	GG	GG	GG	GG
	GG	GG	GG	GG	GG	GG	GG	GG
	GG	GG	GG	GG	GG	GG	GG	
201691502								
6631	rs10186392		2	AC	AC	CC	AC	CC
	CC	AC	AC	AC	AC	CC	AA	AC
	AA	AA	CC	AC	AC	AA	AA	AC
	AC	AC	AC	AC	AC	CC	AC	CC
	AC	AC	CC	AC	AC	CC	AA	
98686948								
6632	rs1018644		13	AG	GG	GG	GG	GG
	GG	GG	GG	GG	GG	GG	GG	GG
	GG	AG	AG	AG	GG	GG	GG	AG
	AG	AG	GG	GG	GG	GG	GG	GG
	AG	GG	AG	AG	GG	GG	GG	
111096711								
6633	rs10186514		2	GG	GG	GG	GG	AG
	GG	AG	GG	GG	GG	GG	GG	GG
	GG	GG	GG	GG	GG	AG	AG	GG
	AG	AG	AG	AG	AG	GG	GG	GG
	GG	GG	AG	GG	GG	GG	GG	
239023528								
6634	rs1018654		9	GG	GG	GG	GG	GG
	GG	GG	AG	AG	GG	GG	GG	GG
	GG	GG	AA	AA	AG	AG	AG	GG
	GG	GG	GG	GG	GG	GG	GG	GG
	AG	GG	AG	AG	GG	GG	AG	
7236712								
6635	rs10186544		2	GG	GG	GG	AG	AG
	GG	AA	AG	AA	AG	GG	GG	GG
	GG	GG	AG	GG	AA	GG	GG	GG

	AG	GG	AA	AG	GG	GG	GG	GG
	GG	AA	AG	AA	AG	AG	AA	
28683174								
6636	rs10186560		2	AA	AA	AA	AA	AA
	AA	AA	AA	AA	AA	AC	AC	AC
	AA	AA	AA	AA	AA	AA	AA	AA
	AA	AA	AA	AA	AA	AA	AA	AA
	AA	AA	AA	AA	AA	AA	AC	
182101001								
6637	rs1018669		1	AC	AC	AC	AA	AA
	AA	AC	AA	AA	AA	AC	AC	AA
	AC	AC	AC	AC	AC	CC	CC	CC
	AC	AC	AA	AC	AC	AC	CC	AC
	AA	CC	AC	AC	CC	AC	AC	
178687459								
6638	rs10186749		2	AG	AG	AG	GG	GG
	GG	GG	AG	GG	AG	GG	GG	GG
	GG	GG	GG	GG	GG	GG	GG	GG
	GG	GG	GG	GG	GG	GG	GG	GG
	GG	GG	GG	GG	GG	GG	GG	
20768247								
6639	rs10186803		2	AA	AC	AA	AC	AC
	AC	AA	AA	AA	AA	AA	AA	AA
	AA	AA	AC	AC	AA	AA	AA	AC
	CC	AC	CC	CC	AA	AA	AA	AA
	AA	AA	AA	AA	AA	AA	AC	
102227623								
6640	rs10186816		2	AA	AA	AA	AA	AA
	AA	AA	AA	AA	AA	AA	AA	AA
	AA	AA	AA	AA	AA	AA	AA	AA
	AA	AA	AA	AA	AA	AA	AA	AA
	AA	AA	AA	AA	AA	AA	AA	
89061977								
6641	rs10186852		2	GG	GG	GG	GG	GG
	GG	GG	GG	GG	GG	GG	GG	GG
	GG	GG	GG	GG	GG	GG	GG	GG
	GG	GG	GG	GG	GG	GG	GG	GG
	GG	GG	GG	GG	GG	GG	GG	
6456705								
6642	rs10186881		2	AA	AA	AA	AA	AA
	AA	AA	AA	AA	AA	AA	AA	AA
	AA	AA	AA	AA	AA	AA	AA	AA
	AA	AA	AA	AA	AA	AA	AA	AA
	AA	AA	AA	AA	AA	AA	AA	
12699621								
6643	rs10186882		2	AG	AG	AG	GG	GG
	GG	GG	GG	GG	GG	GG	AG	GG
	AA	AA	GG	GG	GG	AA	GG	AG
	GG	GG	GG	GG	GG	GG	GG	GG
	GG	GG	AG	AG	GG	GG	GG	

146344233							
6644	rs10186957	2	AG	AA	AG	AG	AA
	AG	AG	AG	GG	GG	AG	AA	AG
	AG	AG	AA	AA	AA	AG	AA	AG
	AA	AA	AA	AA	AG	AG	AA	AG
	AA	AA	AA	AA	AA	AA	AA	
207486889							
6645	rs10186968	2	AA	AA	AA	AA	AA
	AA	AA	GG	GG	GG	AA	AA	AA
	AA	AA	AA	AA	AA	AA	AA	AA
	AA	AA	AA	AA	AA	AA	AA	AA
	AA	AG	AG	AG	AA	AA	AA	
129212869							
6646	rs10187018	2	AG	AG	AG	AG	AG
	GG	AA	AG	AA	AA	AA	AA	AA
	AA	AA	AA	AA	AA	AA	AG	AA
	AG	AG	AA	AA	AG	AA	AA	AA
	AG	AG	AG	AA	AA	AA	AA	
203888858							
6647	rs1018702	10	GG	AG	AG	GG	GG
	GG	GG	AG	GG	GG	GG	GG	GG
	GG	GG	GG	AG	AG	GG	GG	GG
	GG	GG	GG	GG	GG	GG	GG	GG
	GG	AG	AA	AG	AG	GG	AA	
45371418							
6648	rs10187022	2	CC	CC	CC	AC	CC
	CC	AC	AC	AC	CC	CC	CC	CC
	CC	CC	CC	AC	AC	AC	AC	AC
	AC	AC	AC	AC	CC	CC	CC	CC
	AC	CC	AA	AC	CC	CC	AC	
34341570							
6649	rs10187088	2	GG	GG	AG	GG	GG
	GG	GG	AA	AA	AA	AA	AA	AA
	AA	AA	AG	AA	GG	AG	GG	GG
	AA	AG	AA	AA	AG	AA	AA	AG
	AA	AG	AG	AG	AA	AA	AA	
127463952							
6650	rs1018710	1	AA	AG	AG	AA	AA
	AA	AA	AA	AG	AA	GG	GG	GG
	GG	GG	AA	AA	AA	AA	AA	AG
	AA	GG	AG	GG	AG	AG	GG	AG
	AG	AG	GG	GG	GG	GG	AG	
170220451							
6651	rs10187119	2	AG	AG	GG	AG	AG
	GG	AG	GG	GG	GG	GG	GG	AG
	AG	AG	GG	GG	GG	GG	GG	AG
	AG	GG	GG	AG	GG	GG	GG	GG
	GG	GG	GG	GG	GG	GG	GG	
154664805							
6652	rs10187145	2	AA	AG	AA	AA	AA

	AA	AG	AG	AG	AG	AA	AA	AA
	AA	AA	AA	AA	AG	AA	AA	AA
	AA	AA	AA	AA	AA	AA	AA	AA
	AG	AG	AA	AG	AG	AA	AG	
8072360								
6653	rs1018716		13	AA	AA	AA	AA	AA
	AA	AA	AA	AA	AA	AC	AA	AA
	AC	AC	AA	AA	AA	AA	AA	AA
	AC	AC	AA	AA	AA	AA	AA	AA
	AA	AA	AA	AA	AC	AA	AC	
104247930								
6654	rs10187173		2	AA	AC	AC	AC	AC
	CC	AA	AA	AA	AA	CC	CC	AC
	AA	AA	AA	AC	AA	AA	AA	AA
	AC	AC	AC	AC	AC	AC	AC	AC
	AC	CC	AC	AC	AA	AA	AA	
49092438								
6655	rs10187222		2	AG	AG	AG	AA	AA
	AA	AA	AG	AA	AG	AA	AA	AA
	AA	AA	AA	AA	AG	AG	AA	AG
	AA	AA	AA	AA	AA	AA	AA	AA
	AA	AA	AG	AA	AA	AA	AA	
151249628								
6656	rs10187272		2	AC	CC	AC	AC	AC
	AC	CC	CC	AA	AC	AC	AC	AC
	CC	CC	CC	CC	CC	CC	AC	AC
	CC	AC	AC	CC	CC	CC	CC	CC
	AC	AC	CC	CC	AC	CC	AA	
146050955								
6657	rs10187298		2	AA	AA	AA	AA	AA
	AA	AA	AA	AA	AA	AA	AA	AA
	AA	AA	AA	AA	AA	AG	AG	AA
	AA	AA	AA	AA	AG	AA	AG	AA
	AA	AA	AA	AA	AA	AA	AA	
38668336								
6658	rs10187354		2	AA	AA	AA	AC	AA
	AA	AC	AA	AA	AA	AC	CC	CC
	AC	AC	AA	AA	AA	AA	AA	AA
	AA	AC	AA	AC	AC	AA	AC	AA
	AC	CC	AC	AC	AC	AC	CC	
71710068								
6659	rs10187424		2	AG	AG	AG	AA	AG
	AA	AG	AG	AG	AG	AG	AG	AA
	AA	AA	GG	GG	GG	AG	AG	AA
	AA	AG	AA	AA	GG	AG	AG	GG
	AG	AG	AA	AA	GG	AG	AG	
85794297								
6660	rs10187434		2	GG	GG	GG	GG	GG
	GG	GG	GG	GG	GG	GG	GG	GG
	GG	GG	GG	GG	GG	GG	GG	GG

GG GG GG GG GG GG GG GG
GG GG GG GG GG GG GG
109872806
6661 rs10187483 2 GG GG GG GG GG
GG GG GG GG GG GG GG GG
GG GG GG GG GG GG GG GG
GG GG GG GG GG AG GG AG
GG GG AG GG GG GG GG
88903769
6662 rs10187501 2 AG AA AG AA AA
AA AA AG AG AG AA AA AG
AG AG AG AG AG AA AG AA
AA AA AA AA GG AG AG AG
AA AG AA AA AG GG AA
165532454
6663 rs10187530 2 GG GG GG AG AG
AG AA AA AG AG GG AG GG
GG GG AG AG AG GG GG AG
AG GG GG GG GG AG GG AG
AA AA AA AA GG AG AG
217899352
6664 rs10187587 2 AA AA AA AA AA
AA AA AA AA AA AA AA AA
AA AA AA AA AA AA AA AA
AA AA AA AA AA AA AA AA
AA AA AA AA AA AA AA
88991829
6665 rs10187600 2 GG GG GG AG GG
GG AG AG AG AG AG GG GG
AG AG GG GG GG GG GG GG
AG AG AG AG GG GG GG GG
GG GG AG GG GG GG GG
105729122
6666 rs10187631 2 GG GG GG GG GG
GG GG AG AG AG GG GG GG
GG GG AG GG AG GG GG GG
GG GG AG AG AG GG GG AG
GG GG GG GG GG GG GG
120343832
6667 rs1018764 22 GG GG GG GG GG
GG GG AA AA AG GG AG AG
AG AG GG GG GG GG GG GG
GG GG GG GG GG GG GG GG
GG GG GG GG GG GG GG
19172006
6668 rs10187649 2 CC CC CC CC CC
CC CC CC CC CC CC CC CC
CC CC CC CC CC CC CC CC
CC CC CC CC CC CC CC CC
CC CC CC CC CC CC CC

113648769
6669 rs10187665 2 AG AG AG GG GG
GG GG GG GG GG GG AG AG
AG AA GG GG AG AA AA AG
AG GG AA AG AG GG AG GG
AG GG GG GG AG GG AG
28324130
6670 rs10187675 2 GG GG GG GG GG
GG GG GG GG GG GG GG GG
GG GG GG GG GG GG GG GG
GG GG GG GG GG GG GG GG
GG GG GG GG GG GG GG
15713919
6671 rs10187702 2 AG AA AA GG AG
GG AG AA AA AA AA AA AA
AG AG AA AA AA AG AG AG
AG AG AG AG AG AA AG AG
AA AA AA AA GG AG AG
58869775
6672 rs1018786 22 AA AA AA AA AA
AA AA AA AC AA AA AA AA
AA AA AC AC AC AA AA AA
AA AA AA AA AA AA AA AA
AC AA AA AA AC AC AA
34206039
6673 rs10187880 2 AA AG GG AG GG
AG GG AA AG AA AA AG AG
AG AG GG AG AG AG AG GG
GG AG GG GG AA AG AG AA
AG AG AG AG AG AA GG
86775189
6674 rs1018793 22 GG AG AG AG GG
AG GG AG AG AA AG AG GG
GG GG AG AG AG GG AG GG
AG GG GG AG AG GG GG GG
AA GG AG GG GG AG AG
48536673
6675 rs1018795 22 AA AA AG GG AG
GG AG AA AA AA GG GG GG
GG AG AG GG GG AA AA AA
AA AA AA AA AA AA AA AA
AA AG AG AA GG AG AG
44429762
6676 rs10188025 2 CC CC CC AC AC
CC AA CC CC CC AC AA AC
AC AC AC AC CC AC AC CC
CC AC CC CC CC CC CC CC
AC AA CC AC AC AC AC
49001294
6677 rs10188066 2 GG AG GG GG GG

	GG	AG	GG	AG	GG	GG	AG	GG
	GG	GG	AA	AA	AA	AA	AG	AG
	AG	GG	GG	AG	GG	AA	AG	AG
	AG	GG	AA	AG	AA	AA	AG	
136539513								
6678	rs10188086		2	AG	GG	AG	AA	AA
	AA	AG	AG	GG	AG	AG	GG	GG
	GG	GG	AG	AG	AG	GG	GG	GG
	GG	GG	GG	GG	AG	AG	GG	AG
	AG	AG	AG	AG	GG	GG	GG	
139112953								
6679	rs10188093		2	--	--	--	--	--
	--	GG	--	--	GG	--	--	--
	--	--	--	--	--	--	--	--
	--	--	--	GG	--	--	--	--
	--	GG	--	--	--	--	--	
66773438								
6680	rs10188117		2	AA	AA	AA	AG	AG
	AA	AG	AA	AA	AA	AA	AA	AA
	AA	AA	AG	AG	AG	AA	AA	AA
	AA	AA	AA	AA	AA	AA	AA	AA
	AA	AG	AG	AA	AG	AG	AA	
160290519								
6681	rs10188169		2	AG	GG	GG	AG	AA
	AG	AG	GG	GG	GG	AG	AA	AA
	AG	AG	AA	AG	AG	AG	GG	GG
	AG	AA	AG	AG	AA	AG	AG	AA
	AG	GG	AG	AG	AG	AG	GG	
111644595								
6682	rs10188215		2	AG	AA	AG	AG	AG
	AG	AA	AA	GG	AG	AA	AA	AA
	AA	AA	AA	AA	AA	AA	AG	AG
	AA	AG	AG	AA	AA	AA	AA	AA
	AG	AG	AA	AA	AG	AA	GG	
146082440								
6683	rs10188217		2	AA	AG	GG	GG	AG
	AG	GG	AG	AG	AG	AG	GG	GG
	AA	AA	GG	GG	AG	GG	GG	AG
	GG	AG	GG	AG	AA	AG	AA	AG
	AA	AG	AA	AG	AG	AG	AA	
61217542								
6684	rs10188246		2	AA	AA	AA	AA	AA
	AA	AA	AA	AG	AG	AA	AA	AA
	AA	AA	AA	AA	AA	AA	GG	AA
	AA	AA	AA	AA	AA	AA	AA	AA
	AA	AA	AA	AA	AA	AA	AA	
190171008								
6685	rs10188277		2	GG	GG	GG	GG	GG
	GG	GG	GG	GG	GG	GG	GG	GG
	GG	GG	GG	GG	GG	GG	GG	GG

GG GG GG GG GG GG GG GG
GG GG GG GG GG GG GG
147323502
6686 rs1018829 1 GG GG GG AG AG
GG AG GG GG GG GG GG GG
GG GG AG GG GG GG AG AG
GG GG GG GG GG GG GG GG
GG GG GG GG GG GG AG
175109722
6687 rs10188315 2 AA AA AA AA AA
AA AA AA AA AA AA AA AA
AA AA AA AA AA AA AA AA
AA AA AA AA AA AA AA AA
AA AA AA AA AA AA AA
84527176
6688 rs10188319 2 AG AG AG AA AA
AA AA AA AA AA AA AA AA
AG AG AA AG AG AG AG AG
AG AG AG AG AA AA AA AA
AG AA AG AA AG AG AG
34329717
6689 rs10188328 2 GG GG GG GG GG
GG GG GG GG GG GG GG GG
GG GG GG GG GG GG GG GG
GG GG GG GG GG GG GG GG
GG GG GG GG GG GG GG
197958795
6690 rs1018836 8 AG AG AG AA AA
AA AA AG AG AG GG AG AG
AG AA AA AG AA GG GG GG
AG AG AG AG AG GG GG AG
GG GG GG GG AG AG AG
91570362
6691 rs10188406 2 GG GG GG GG GG
GG GG GG GG GG GG GG GG
GG GG GG GG GG GG GG GG
GG GG GG GG GG GG GG GG
GG GG GG GG GG GG GG
237137222
6692 rs10188431 2 GG GG GG GG GG
GG GG GG GG GG GG GG GG
GG GG GG GG GG GG GG GG
GG GG GG GG GG GG GG GG
GG GG GG GG GG GG GG
197698349
6693 rs1018845 9 AG GG GG AG AA
AG AA AG GG AG AG AG AA
AA GG AG AG GG AG AG AA
AA AA AA AA AA GG AG AG
AA AA AG AA AG GG AG

118311164
6694 rs10188470 2 AG AG AG AG AG
AA AG AG AG AG GG AG AG
GG GG AA AA AG AG AG AA
AG AG AA AG GG AG AG AG
AA AG AG GG AA AA AG
12660466
6695 rs10188503 2 AA AC AA CC AC
AC CC CC CC CC AC AC AC
AC CC AC AC CC AC AC CC
CC AC AC CC CC CC CC CC
CC AC AC AC CC AC AC
21134040
6696 rs10188512 2 AG AG AG AA AG
AA AG GG AG AG GG AA AG
GG -- GG AG AG GG GG AG
AG GG -- GG AA AG AG AA
AG AA AG AG AG AG GG
67940962
6697 rs1018854 7 AG AA AG AG AG
GG AA AA AA AA GG GG GG
GG AG AG AG AG GG GG AG
AA GG AG AA AA AG AA AA
AG AA AG AG AG GG AG
126657545
6698 rs10188572 2 GG GG GG GG GG
GG GG GG GG GG GG GG GG
GG GG GG GG GG GG GG GG
GG GG GG GG GG GG GG GG
GG GG GG GG GG GG GG
175992205
6699 rs1018859 7 GG GG GG GG GG
GG GG GG GG GG AG GG AG
AG GG GG GG GG GG GG GG
GG GG GG GG GG GG GG GG
AG GG GG GG GG GG GG
116067347
6700 rs10188630 2 GG AG GG AG GG
GG AG GG GG GG GG GG AG
GG GG AG GG AG AG AG AG
AG AG GG GG GG GG GG GG
GG GG GG GG GG GG AG
109765916
6701 rs10188669 2 AG AG AG AG AG
AA GG GG GG AG AA AA AA
AG AG AG GG GG AG AG AG
GG AG AG GG AA AA AA AA
AA GG GG GG AA AA AG
151753903
6702 rs10188678 2 AA AA AA GG AG

	AG	AG	AA	AA	AA	AA	AA	AA
	AA	AG	AG	GG	GG	AG	AG	AG
	AA	AA	AA	AA	AG	AA	AA	AA
	AA	AA	AA	AA	AA	AA	AA	
242025882								
6703	rs10188695		2	GG	GG	GG	GG	GG
	GG	GG	GG	GG	GG	GG	GG	GG
	GG	GG	AG	GG	GG	GG	AG	AG
	AG	AG	GG	GG	GG	AG	AG	GG
	GG	AG	GG	GG	AG	GG	AG	
211387772								
6704	rs10188716		2	AA	AA	AA	AA	AA
	AA	AA	AG	AA	AG	AG	AG	GG
	AA	AG	AA	AA	AA	AA	AA	AA
	AA	AA	AA	AA	AA	AA	AA	AA
	AG	AA	AA	AA	AA	AA	AA	
148418820								
6705	rs1018873		X	GG	GG	GG	GG	GG
	GG	GG	GG	GG	GG	GG	GG	GG
	GG	GG	GG	GG	GG	GG	GG	GG
	GG	GG	GG	GG	GG	GG	GG	GG
	GG	GG	GG	GG	GG	GG	GG	
127652828								
6706	rs10188765		2	AG	AG	AG	GG	GG
	AG	GG	AG	AG	AG	AA	AG	AA
	AG	AG	AG	AG	AA	AA	AA	AG
	AG	AG	GG	GG	AA	AG	AG	AA
	AG	GG	GG	GG	GG	AG	GG	
113715676								
6707	rs10188829		2	AA	AA	AA	AA	AA
	AA	AA	AA	AA	AA	AA	AA	AA
	AA	AA	AA	AA	AA	AA	AA	AA
	AA	AA	AA	AA	AA	AA	AA	AA
	AA	AA	AA	AA	AA	AA	AA	
197435645								
6708	rs10188861		2	AA	AG	AG	AG	AA
	AA	AG	AG	AG	AG	AA	AG	AA
	AA	AA	AA	AA	AG	AA	AG	AA
	AG	AA	AA	AG	AA	AG	AA	AG
	GG	AG	AG	GG	GG	AG	AG	
43766110								
6709	rs10188915		2	AA	AA	AA	AA	AA
	AA	AA	AA	AA	AA	AA	AA	AA
	AA	AA	AA	AA	AA	AA	AA	AA
	AA	AA	AA	AA	AA	AA	AA	AA
	AA	AA	AA	AA	AA	AA	AA	
147274116								
6710	rs10188925		2	GG	GG	AG	GG	AG
	GG	AG	AG	AG	AA	AG	AA	AA
	GG	GG	GG	GG	GG	AA	AG	AG

	AG	AA	AG	AG	AA	AA	AA	AA
	GG	AG	AG	AG	GG	GG	GG	
31421582								
6711	rs10188973							
2	--	--	--	--	--	--	--	--
	--	--	--	--	--	--	--	--
	--	--	--	--	--	--	--	--
	--	--	--	--	--	--	--	--
	--	--	--	--	5810657			
6712	rs10188985		2	AG	GG	AG	GG	AA
	AG	AG	AG	GG	AG	AG	GG	GG
	GG	GG	GG	AG	AG	AG	AG	AG
	AG	GG	AG	AG	AG	AA	AA	AG
	GG	AG	AA	AG	AA	AG	AA	
85941074								
6713	rs10189022		2	GG	GG	GG	GG	AG
	AG	AG	GG	GG	GG	AG	AA	AA
	AG	AG	AG	AG	GG	GG	GG	GG
	AG	GG	AG	GG	AG	GG	GG	AG
	GG	AA	GG	AG	AG	AG	GG	
73427222								
6714	rs1018903		17	AG	GG	AG	AA	AA
	AA	AA	GG	AG	AG	AG	GG	GG
	GG	AG	AG	GG	GG	GG	GG	GG
	AA	AG	AG	AG	AG	AA	AG	AG
	AG	GG	AG	AG	GG	AG	AG	
45889840								
6715	rs10189080		2	AA	AA	AA	AA	AA
	AA	AA	AA	AA	AA	AA	AA	AA
	AA	AA	AA	AA	AA	AA	AA	AA
	AA	AA	AA	AA	AA	AG	AA	AG
	AA	AA	AG	AA	AA	AA	AA	
88855325								
6716	rs10189138		2	CC	CC	CC	AC	CC
	AC	CC	CC	CC	CC	CC	CC	CC
	AC	AC	CC	CC	CC	CC	CC	CC
	CC	CC	CC	CC	CC	AA	AC	AC
	CC	CC	CC	CC	CC	CC	CC	
58153676								
6717	rs1018915		7	CC	CC	CC	AC	AA
	AC	AC	AC	AC	AC	CC	CC	CC
	CC	CC	AC	AC	AC	CC	AC	CC
	AC	CC	CC	AC	AC	AA	AC	AC
	AC	AC	CC	AC	AC	AC	AA	
111651812								
6718	rs10189153		2	GG	GG	GG	GG	GG
	GG	GG	GG	GG	GG	GG	GG	GG
	AG	AG	GG	GG	AG	AG	AG	AG
	AG	AG	GG	AA	AG	AG	AG	GG
	GG	GG	GG	GG	GG	GG	GG	

166526941
6719 rs10189159 2 AG GG GG AA GG
AG AG AG AG AG AA AA AA
AG AG AG GG AG AG AG AG
AG AA AG AG AA AG AG AA
AG AG GG AG AG AG AA
50714571
6720 rs10189180 2 GG GG GG AG AG
AG AG GG GG GG AG AG AG
AG AG AG GG AG AG AG AA
AG AG AG GG GG GG GG GG
AA AG GG AG AG AG AA
15685949
6721 rs10189214 2 AG AG GG GG GG
GG GG GG GG AG GG GG GG
GG GG GG GG GG GG GG GG
GG AA AG AG AG GG AG GG
GG GG AG AG GG GG GG
156786929
6722 rs10189216 2 AG AG AG AG AA
AG AA AG AG AG GG GG GG
GG GG AA AG AG AG GG GG
GG GG GG GG AG AG AA AA
AG AG GG AG GG AG GG
8046782
6723 rs10189240 2 GG AG GG AA AA
AA AA GG AG AG AG -- GG
AA AA AA AG AA AA AG AA
AA AG AG AA AG AG AA AG
AG AA AA AA AA AA AG
144484493
6724 rs10189267
2 -- -- -- -- -- -- -- --
-- -- -- -- -- -- -- --
-- -- -- -- -- -- -- --
-- -- -- -- -- -- -- --
-- -- -- -- 242945309
6725 rs10189296 2 CC CC CC CC CC
CC CC CC CC CC CC CC CC
CC CC CC CC AC AC CC AC
CC CC CC CC CC CC CC CC
CC CC AC AC AC CC AC
36961691
6726 rs10189341 2 AA AA AA AA AA
AA AA GG GG GG AA AA AA
AA AA AG AG AG AA AA AA
AG AA AG AA AA GG AG AG
AG AG GG GG GG GG AG
176435942
6727 rs10189364 2 AA AA AA AG AA

AA AG AA AA AA AG AG AA
AG AG AG AA GG AG GG AA
AA AA AA AA AA AA AA AA
AG AA AA AA AG AG GG
37945865
6728 rs10189484 2 AA AA AA AA AA
AA AA AA AA AA AG AA AA
AA AA AA AA AA AA AG AA
AA AA AA AA AG AG AA AG
AA AG AA AA GG AG AG
155084230
6729 rs10189542 2 AA AC AA AA --
AC AA AA AC AA AA AA AA
AA AA AC AA AC AA AA AC
AA AA AA AA AA AA AA AA
AA AA AA AA AC CC AC
24655725
6730 rs10189572 2 GG AG AA AG AG
GG AA AA AG AA AG AG AG
AG AG AA AG AG AA AA AA
GG AA GG AG AA AG AG AA
GG AA GG AG AG GG AA
79055423
6731 rs1018965 5 CC CC CC CC CC
CC CC CC CC CC CC CC CC
CC CC CC CC CC AC AC AC
CC CC CC CC CC CC CC CC
CC CC CC CC CC CC CC
11175485
6732 rs10189659 2 GG GG GG GG GG
GG GG GG GG GG GG GG GG
GG GG GG GG GG GG AG GG
GG GG GG GG GG GG GG GG
GG GG GG GG GG GG GG
194236059
6733 rs10189682 2 GG GG GG GG GG
GG GG AG GG GG GG GG GG
GG GG AG AG AG AG GG AG
GG GG GG GG GG AG AG GG
AG AG GG GG AG AG GG
217646555
6734 rs10189685 2 GG AG GG AG AG
GG AG AA AG AG AA AG AA
AA AG AG AG AG AG GG AG
AA AA AG GG GG AG AG GG
GG GG GG GG AA AG AG
203488449
6735 rs10189731 2 AA AA AA AA AA
AA AA AA AA AA AA AA AA
AA AA AA AA AA AA AA AA

AA AA AA AA AA AA AA AA
AA AA AA AA AA AA AA
40605729
6736 rs10189774 2 GG AG AG GG GG
GG AG GG GG GG AG AG AG
AG AG AG AA AG AG AG AG
GG GG GG GG GG GG GG GG
GG AG GG GG GG AG GG
171375497
6737 rs10189776 2 AG AG AG GG GG
GG GG AG AA AG AG AG GG
GG GG GG AG AG AA AG AG
AG AG AG AG AA GG AG AG
GG GG AG GG AA AG AG
182030229
6738 rs10189788 2 AA AA AA AA AA
AA AA AG AA AA AA AA AA
AA AA AG AA AA AA AA AA
AA AA AA AA AA AG AA AG
AA AG AA AG AG AG AA
197129767
6739 rs10189806 2 CC CC CC CC CC
CC CC CC CC CC CC CC CC
CC CC CC CC CC CC CC CC
CC CC CC CC CC CC CC CC
CC CC CC CC CC CC CC
88402913
6740 rs10189899 2 AA AG AA GG GG
GG GG AG AG AG AG AA AA
GG AG GG GG GG GG GG GG
AG GG AA AG AG GG GG AG
AG AA GG AG AG AG AG
28520083
6741 rs10189932 2 GG GG GG GG GG
GG GG GG GG GG GG GG GG
AG AG AG AG AG GG GG GG
GG GG GG GG GG GG GG GG
AG GG GG GG GG GG GG
64895272
6742 rs10189953 2 AG GG AG AG AG
AG GG GG GG GG AG AA AG
AG GG GG AG AG AG AA AG
AG AG GG AG AG GG AG AG
GG GG AG AG AG AG GG
1253705
6743 rs10189976 2 CC CC CC AA AA
AA AA CC AC CC AA AC AA
AC AC AC AC CC AC AC AC
AA AC AA AA AA AC AA AC
AA AA AA AA AC AA AC

100295638
6744 rs10190089 2 AA AA AA AG AG
GG AG AG AG GG AA AA AG
GG GG AG AA AA GG GG GG
GG AG AG GG AA GG AG AG
AA AG GG AG AG AG AA
159249741
6745 rs10190128 2 GG GG GG GG GG
GG GG AG AG AG GG GG GG
GG GG GG AG GG GG GG GG
AG AG AG AG AG GG AG AG
AG AG GG GG AG AG GG
236287266
6746 rs10190161 2 AC AC CC CC AC
AC CC CC CC CC AC CC AC
AC AC AA AC AC CC CC CC
CC CC CC CC AC AC AC AC
AC CC AC CC AC CC AC
44187829
6747 rs1019018 7 AG AG AG GG AG
GG AG GG GG GG GG GG GG
GG GG GG GG GG GG GG AG
GG GG GG GG GG GG GG GG
GG GG GG GG GG GG GG
40631826
6748 rs10190267 2 AA AA AA GG AG
AG GG AG AG AG AG AG AG
AG GG GG AG GG AG AG GG
AG AG GG AG AG AA AG AA
AG GG AA AG GG AG AG
28655153
6749 rs1019031 2 AA AC AA AC CC
AC AC AC AA AC AC AC AC
AC AC CC AC AC AC AC CC
AC AC AC AC AA AC AA AC
AC AC AC AC CC AC AC
102574838
6750 rs10190346 2 AA AA AA AA AA
AA AA AA AA AA AA AA AA
AA AA AA AA AA AA AA AA
AA AA AA AA AA AA AA AA
AA AA AA AA AA AA AA
238182067
6751 rs1019035 2 AG AG AG AG AG
GG AG AA AA AA AG GG AG
AG AG AG AA AG AG GG AG
AG AG AG AG GG AG GG GG
GG AG AA AA GG GG AG
151802844
6752 rs10190378 2 AG AG AG AA AA

AA AA GG AA AG AA AA AG
AG AG AA AA AG AG AA AG
AG AG AG AG AA AA AA AA
AA AA AG AA AA AA AA
151131188
6753 rs10190397 2 AA AG AA AG AA
AA AG AG AG AG AA AA AG
GG GG AG AG AG AA AA AG
AA AA AA AA AA AA AA AA
AG AG AA AA AA AA AA
3516085
6754 rs10190482
2 -- -- -- -- -- -- -- --
-- -- -- -- -- -- -- --
-- -- -- -- -- -- -- --
-- -- -- -- -- -- -- --
-- -- -- -- 203297928
6755 rs10190487 2 AA AA AA AA AA
AA AA AA AA AA AA AA AA
AA AA AA AA AA AA AA AA
AA AA AA AA AA AA AA AA
AA AA AA AA AA AA AA
200074619
6756 rs10190488 2 AA AA AA AA AA
AA AA AA AA AA AA AA AA
AA AA AC AC AC AA AC AA
AA CC AA AC AA AA AA AA
AA AA AA AA AA AA AC
179684398
6757 rs1019053 X AA AG AG AG AA
AA AG AA AA AA AA AA AA
AA AA AA AA AA AG GG AA
GG GG AA AA AG AA AA GG
AA AG AA GG AA AG AA
111186193
6758 rs10190545 2 AG GG AG AA AA
AA AA AA AA AA AA AA AA
AA AA AG AA AG AG AG AG
AG AG AA AA AA AA AA AA
AA AA AA AA AA AA AG
109829714
6759 rs10190730 2 AA AA AA AA AA
AA -- -- AA -- -- AA AA
AA AA AA -- AA AA -- AA
AA AA AA AA AA AA AA AA
AA -- AA AA AA AA --
142384798
6760 rs10190743
2 -- -- -- -- -- -- -- --
-- -- -- -- -- -- -- --

-- -- -- -- -- -- -- --
-- -- -- -- -- -- -- --
-- -- -- -- 113047747
6761 rs10190801 2 AA AA AA AA AA
AA AA AA AA AA AA AA AA
AA AA AA AA AA AA AA AA
AA AA AA AA AA AA AA AA
AA AA AA AA AA AA AA
63327431
6762 rs1019083 7 AG AA AA AA AA
AA AA AA AA AA AA AA AA
AA AA AG AG AG AG AA AA
AA AG AG AA AG AA AA AA
AA AA AG AG AA AA AA
39414081
6763 rs10190832 2 GG GG GG AA AA
AA AA AG AG AG AA AG AA
AA AA AA AG AG AG AG AA
AG AG AG GG GG AA AG AG
AG AA AG AA AG AA GG
235509738
6764 rs10190870 2 AG AG GG GG AG
AG AG GG AG AG AG AG AG
AA AA AA AA AA AG AG AG
AG GG GG AG AG GG GG AG
AA AA GG AG AA AG AG
161646558
6765 rs10190923 2 AA AA AA AA AA
AA AA AA AA AA AA AA AA
AA AA AA AA AA AA AA AA
AA AA AA AA AA AA AA AA
AA AA AA AA AA AA AA
146398556
6766 rs10190937 2 GG GG GG GG GG
GG GG GG GG GG GG GG GG
GG GG GG GG GG GG GG GG
GG GG GG GG GG GG GG GG
GG GG GG GG GG GG GG
10881368
6767 rs10190938 2 GG GG GG GG GG
GG GG AG GG GG GG GG GG
GG GG GG GG GG GG GG GG
GG GG GG GG GG GG GG GG
GG GG GG GG AA AG AG
154331653
6768 rs10190998 2 GG GG GG GG GG
GG GG GG GG GG GG GG GG
GG GG GG GG GG GG GG GG
GG GG GG GG GG GG GG GG
GG GG GG GG GG GG GG

15619986
6769	rs10191044	2	AG	AG	AG	--	GG
GG	AG	GG	GG	GG	GG	GG	GG
GG	GG	GG	GG	GG	GG	GG	GG
GG	GG	GG	GG	AG	GG	GG	AG
GG	AG	GG	AG	AA	AG	AA
18716201
6770	rs10191091	2	AG	AG	GG	GG	AG
GG	AG	AG	AA	AA	AA	AA	AG
AA	AA	AG	GG	AA	AA	AA	AG
AG	AA	AG	AG	GG	AA	AG	AG
AA	AA	AA	AA	AA	AG	AA
46573210
6771	rs10191117	2	CC	CC	CC	CC	CC
CC	CC	CC	CC	CC	CC	CC	CC
CC	CC	CC	CC	CC	CC	CC	CC
CC	CC	CC	CC	CC	CC	CC	CC
CC	CC	CC	CC	CC	CC	CC
14622012
6772	rs10191141	2	AG	GG	AG	AA	AA
AA	AG	GG	GG	GG	AG	GG	AG
GG	GG	GG	AG	AG	AG	AG	AG
GG	GG	GG	GG	AG	AG	AG	AG
GG	GG	AG	AG	AG	AA	AG
77103474
6773	rs10191167
2	--	--	--	--	--	--	--	--
--	--	--	--	--	--	--	--
--	--	--	--	--	--	--	--
--	--	--	--	--	--	--	--
--	--	--	--	214756500
6774	rs10191260	2	AA	AA	AA	AA	AA
AA	AA	AG	AA	AA	AG	AA	AA
AA	AA	AA	AA	AA	AG	AG	GG
AG	AG	AG	AG	AG	AG	GG	GG
AA	AG	AA	AG	AA	AG	AA
237948930
6775	rs10191261	2	GG	AG	AG	GG	GG
GG	GG	GG	GG	GG	GG	GG	GG
AG	AG	GG	GG	AG	GG	GG	AG
AA	AG	AA	AA	GG	AG	GG	AG
GG	AG	GG	GG	AA	AG	AG
50618708
6776	rs10191266
2	--	--	--	--	--	--	--	--
--	--	--	--	--	--	--	--
--	--	--	--	--	--	--	--
--	--	--	--	--	--	--	--
--	--	--	--	20824708
6777	rs10191330	2	GG	GG	GG	GG	GG

	GG	GG	GG	GG	GG	GG	GG	GG
	GG	GG	GG	GG	GG	GG	GG	GG
	GG	GG	GG	GG	GG	GG	GG	GG
	GG	GG	GG	GG	GG	GG	GG	
102408327								
6778	rs10191360		2	AA	AA	AA	AG	AG
	AG	GG	GG	AG	GG	AG	AA	AG
	AG	AG	AA	AA	AA	AA	GG	AG
	AA	AA	AA	AA	AA	AG	AA	AG
	AG	AG	AA	AA	GG	AG	AG	
136884679								
6779	rs10191412		2	AA	AA	AA	AC	CC
	AC	CC	AA	AA	AA	AC	AC	AA
	CC	CC	AC	AA	AC	AC	AC	AC
	AA	AC	AC	AA	AA	AA	AA	AA
	CC	AA	AA	AA	AC	AA	CC	
46011945								
6780	rs10191479		2	GG	AG	AG	GG	GG
	GG	GG	GG	GG	GG	GG	GG	AG
	GG	GG	GG	GG	GG	AG	AG	AG
	AG	AG	AG	AG	GG	AA	AG	AG
	GG	GG	GG	GG	GG	GG	AG	
47469711								
6781	rs10191492		2	GG	AG	GG	AA	AG
	AG	AG	GG	GG	GG	AA	GG	AG
	AG	AG	AG	GG	GG	AG	AG	GG
	AG	AA	AG	AG	AG	AG	AA	AG
	AG	AG	AA	AG	GG	GG	GG	
6380352								
6782	rs10191600		2	GG	GG	GG	GG	GG
	GG	AG	GG	GG	GG	AG	AG	GG
	AG	AG	GG	AG	GG	AG	AA	AA
	AG	GG	AG	AG	GG	GG	GG	GG
	AG	AG	AA	AG	GG	GG	AG	
171476133								
6783	rs10191609		2	AG	AG	AG	AG	AG
	GG	AG	AG	AG	AG	AG	AG	AA
	AA	AA	AG	AG	AG	GG	GG	AG
	AG	AG	AG	AA	AG	AG	AG	AA
	AA	GG	AA	AG	AG	AG	AA	
181074667								
6784	rs10191613		2	GG	AG	AA	GG	GG
	GG	GG	AG	AG	AG	AG	AG	AA
	GG	GG	AG	AG	GG	GG	AG	GG
	AA	AG	AG	AA	GG	AG	AG	GG
	AG	GG	AG	GG	AG	GG	AG	
40736331								
6785	rs10191694		2	AC	AC	AC	CC	AC
	AC	AC	CC	CC	CC	CC	CC	CC
	CC	CC	CC	CC	AC	AC	AC	AC

CC CC CC CC AC AC AA AC
AA CC AC AC AC AC AC
224857266
6786 rs10191729 2 GG GG GG AG AG
GG AG GG GG GG GG AG AG
GG GG GG AG AG GG AG GG
GG GG GG GG AG AA AG AG
AA AG AG AG GG GG GG
12705146
6787 rs1019173 7 AA AG AA AA AA
AA AA AA AG AG AG AG GG
GG GG AA AA AA AA AG AA
GG AA GG GG AG GG AG AG
AG AA AG AG AA AG AA
151710547
6788 rs10191788 2 AA AA AA AA AA
AA AA AA AA AA AA AA AA
AA AA AA AA AA AA AA AA
AA AA AA AA AA AA AA AA
AG AA GG AG AA AA AA
235480791
6789 rs1019179 7 AA AG AA AG AG
AA GG AG AA AG AA AA AA
AA AA AG AA AA AG AA AG
AG AA AA AG AA GG AG AG
AG AA AA AA AG AG AG
38218976
6790 rs10191793 2 AG GG AG AA AA
AA AA AG AG AG AA AA AG
AA AA AA AA AA AG GG GG
AA AA AA AA AA AA AA AA
AA AA AA AA AG AG AA
107425876
6791 rs10191808 2 AA AC AC AA AA
AA AA AA AA AA CC CC CC
AA AA AA AA AA AC AC AC
AC AC AC AC AA AC AA AC
AA AC AC AA AC AC CC
50268555
6792 rs10191864 2 AG AG AG AG AA
AG AG GG AG GG AA GG AG
AG AG AG AA AA AA AG AA
AG GG AG AG GG AA AG AG
AG GG GG GG AG GG AA
7644366
6793 rs10191917 2 GG GG GG GG GG
GG GG AG GG AG GG AG AG
AG AG GG GG GG GG GG GG
AG GG AG AG AG AA AA AG
GG GG AG AG GG GG GG

103950575
6794 rs10191931 2 GG GG GG GG GG
GG GG GG GG GG GG AA AG
AG AG GG GG GG AG AG GG
GG GG GG GG GG AG AG GG
GG GG GG GG AA AG AG
217220670
6795 rs10191938 2 AA AA AA AA AA
AA AA AA AA AA AA AA AA
AA AA AA AA AA AA AA AA
AA AA AA AA AA AA AA AA
AA AA AA AA AA AA AA
48627956
6796 rs10192 6 CC CC CC CC AC AC
CC AC CC AC CC CC CC CC
CC AC CC AC AC AC AA AC
AC CC AC AA AC AC AA CC
CC CC CC CC CC CC 145174098
6797 rs10192011 2 CC AC AC AC CC
AC CC CC CC CC CC CC CC
CC CC CC CC AC CC CC CC
CC CC CC CC CC CC CC CC
AC CC CC CC CC CC CC
53804261
6798 rs10192012 2 AA AA AA AG AG
AG AG AG AG AG AA AA AA
GG GG AG AA AG AA AA AG
AG AG GG GG GG AA AG AG
AG AG AA AG AG AG AA
50247620
6799 rs10192049 2 AG AA AG AG GG
AG AG AA AA AA AG AG AA
AA AA AA AA AA AA AG AG
AA AA AA AA AA AA AA AA
AG AG AA AG AA AG AA
171534221
6800 rs10192053 2 AC AC AC AC CC
AC CC AC CC AC AA AC AC
AC AC AC CC AC CC AC CC
AC AC AC AC AC CC AC AC
AA AC AC AC CC CC CC
7090819
6801 rs10192079 2 AA AA AA AG AG
AG AA AG AG AG AA AA AA
AG AG AG AG GG AG AA AA
AG AG AA AA AA AA AA AA
AG AA AA AA AA AA AA
115943835
6802 rs10192088 2 CC AC CC CC CC
CC CC CC CC CC CC CC CC

CC CC CC AC CC CC CC CC
CC CC CC CC CC CC CC CC
AC CC CC CC CC CC CC
4656639
6803 rs1019216 11 AG AG AG AA AA
AA AA AA AA AA AA AA AA
AA AA AA AA AA AA AA AA
AA AA AA AA AA AG AG AA
AA AA AA AA AA AA AA
22843220
6804 rs10192176 2 AG GG AG AG AA
AG AA AG GG GG GG GG GG
GG GG GG AG GG GG GG GG
GG GG GG GG AG GG AG AG
GG AA AG AG AG GG AG
69126125
6805 rs10192183 2 CC AC AC AA AA
AC AA CC CC CC -- AA AC
AA AA AA AC AA AA AA AA
AA AC AC AC AC AC CC AA
AC AC AC CC AA AC AA
123388562
6806 rs10192223 2 GG GG GG GG GG
GG GG GG GG GG GG GG GG
GG GG GG GG GG GG GG GG
GG GG GG GG GG GG GG GG
GG GG GG GG GG GG GG
197776652
6807 rs10192234 2 AC AC AC AC AA
AC AA CC AC CC AC AC AC
AC CC AA AC AC AA AA AC
AA AC AA AC AC AA AC AC
AA AC AA AC AC CC AC
184613551
6808 rs10192285 2 AA AA AA AA AA
AA AA AA AA AA AA AA AA
AA AA AA AA AA AA AA AA
AA AA AA AA AA AA AA AA
AA AA AA AA AA AA AA
231492816
6809 rs10192294 2 AA AA AA AA AA
AA AA AA AA AA AA AA AA
AA AA AA AA AA AA AA AA
AA AA AA AA AA AA AA AA
AA AA AA AA AA AA AA
237422845
6810 rs10192326 2 AA AG AG GG AA
AG AG AG AG AG AA AA AA
AA AA AA AA AA AA AA AA
AA AG AG AG AA AG AA AG

AA AA AG AG AA AA AA
47522368
6811 rs10192369 2 GG GG GG AG AG
AA GG AG GG GG AG AG AG
AA AA AG AA AA GG AA AG
AA AG AG AA AA AG AA AA
AG AA AG AG AA AG AG
161380888
6812 rs10192375 2 AA AA AA AG GG
AG GG AA AA AA AG AA AA
AG AA AA AA AA GG -- --
AA AA AA AA AA AG AG AA
GG AG AG AG AG AG AA
28892116
6813 rs10192429 2 AA AA AA AA AA
AA AA AC AA AC AA AC AC
AC AC AA AA AA AC AC AC
AA AA AA AA AA AC AA AC
AA AA AA AA AA AA AA
88562285
6814 rs10192440 2 AG AA AG AG AG
AA AG AG AG AG AA AA AG
AG AG AA AA AA AG AG AA
AA AA -- AA AA AA AA AA
AG AA AA AA AG AG AA
156647746
6815 rs10192459 2 AA AA AA AA AA
AA AA AA AA AG AA AA AA
AA AA AA AA AA AA AA AA
AG AG AG AG AA AA AA AA
AA AA AA AA AA AA AA
152517979
6816 rs1019248 7 CC CC CC CC CC
CC CC CC CC CC CC CC CC
CC CC CC CC CC CC CC CC
CC AC AC CC CC CC CC CC
CC CC AC CC CC CC CC
20085995
6817 rs10192523 2 GG GG GG GG AG
AG AG AG AG AG GG GG GG
GG GG GG GG GG AG AA AG
AG GG AG AG AG GG AG AG
AG GG GG GG GG GG GG
2574682
6818 rs10192541 2 AA AA AA AA AA
AA AA AA AA AA AA AA AA
AA AA AA AA AG AA AA AG
AA AA AA AA AA AA AA AA
AA AG AA AG AA AA AA
59457661

6819 rs10192549 2 AG AA AG AA AA
AA AA AA AG AG AA AA AA
AA AA AA AA AA AA AA AA
AG AA AG AA AG AA AA AA
AA AA AA AA AG AA AG
180316070
6820 rs10192562 2 AA AA AA AG AG
AA AG AA AA AA AA AA AA
AA AA AA AA AA AA AA AA
AA AA AA AA AA AA AA AA
AA AA AA AA AA AA AG
145184316
6821 rs1019257 16 GG GG GG GG GG
GG GG GG GG GG GG GG GG
AG GG AG AG AA GG AG AG
GG GG GG GG GG GG GG GG
GG GG GG GG GG AG GG
6749612
6822 rs10192578 2 AG AA AG AG AA
AG AA AG AG AG AA AA AA
AG AG AG AG GG AA AA AA
GG AG GG AG AG AG AA AG
AA AA AG AG AA AA AG
58615998
6823 rs10192594 2 AG AG AG AA AA
AA AA AA AA AA AA AG AA
AA AA AG AA AA AA AA AA
AA AA AG AA AG AA AA AA
AG AA GG AG AA AA AA
9082822
6824 rs10192643 2 AA AA AG AG AG
AG GG AA AA AA AG AA AG
AG AG AA AA AA AG AA AG
AA AA AA AA AA AG AG AA
AA AA AA AA AG AG AA
110022219
6825 rs10192647 2 AA AA AA AA AA
AA AA AA AA AA AA AA AA
AA AA AA AA AA AA AA AA
AA AA AA AA AA AA AA AA
AA AA AA AA AA AA AA
8631081
6826 rs101927 4 AG AG AG AA AA AA
AA AA AA AG GG AA AG AG
AA AG AA AA AA AG AA AA
AG AG AA AG AG AA AG AA
GG AA AG AA AA AG 125435781
6827 rs10192718 2 GG GG GG AA AA
AA AA GG GG GG AG AG AG
AG GG AG GG GG GG GG AG

AG AG AA AG GG AA AG AG
AG AG AG AG GG GG GG
206862015
6828 rs10192751 2 AC AC AC CC CC
CC CC AC AC AC CC CC CC
CC CC CC CC CC CC CC CC
CC CC CC CC CC CC CC CC
AC AC CC AC CC CC CC
9419106
6829 rs10192788 2 GG GG GG AA AG
AG AA GG GG GG AG GG AG
AG AG AA AG AA GG GG AG
AA AA AA AA AG AG GG AA
AG AA AG AG AG AG AG
38272672
6830 rs10192798 2 CC AC CC AC AC
CC AC CC CC CC AC CC CC
CC CC AC AA AC CC CC AC
CC AC CC CC AC CC CC AC
CC CC AC CC CC CC CC
117912256
6831 rs10192799 2 GG GG GG GG GG
GG GG AG GG AG GG GG GG
GG GG GG GG GG GG GG GG
GG GG GG GG GG GG GG GG
GG GG GG GG GG GG GG
159089280
6832 rs10192840 2 AG AA AG AA AG
AG AA AG AG GG AA AA AA
AG AA AG AA AA AA AA AA
AG AG GG AG AA AA AA AA
AA AA AG AA AA AA AA
209794478
6833 rs10192863 2 AG AG AA AG GG
AG GG AG GG GG AG AG AG
GG GG GG AG GG AG AA GG
AG GG GG AG GG GG GG GG
AA GG AG GG GG GG GG
165275535
6834 rs1019287 7 AG GG AG AA AA
AA AA AA AA AG GG AG AG
AA AA AA AA AG AA AA AA
AA AA AA AA AA AG AA AA
AG AA AG AG AA AA AA
38460546
6835 rs1019288 7 CC AC CC AC AA
AA AC CC CC CC CC CC CC
CC CC AC AC AC AC AC AA
CC AC CC AC AC AC AC AC
AC CC CC CC AA AC AC

38503023
6836 rs10192881 2 AG AA AG AA AA
AA AG AA AG AA AA AG AG
GG GG AA AG AG AA AA AG
AA AG AA AA AA AG AG AA
AA AA AA AA AA AA AG
79562010
6837 rs10192894 2 AA AA AA GG AA
AG AG AG AG AG AA AA AA
AG AG AA AA AA AA AA AG
AA AA AA AA AG GG AG AG
AG GG AG GG AA AA AG
62838936
6838 rs10192895 2 AA AA AA AA AA
AA AA AA AA AA AA AA AA
AA AA AA AA AA AA AA AA
AA AA AA AA AA AA AA AA
AA AG AA AA AA AA AA
88521935
6839 rs10192922 2 GG GG GG GG GG
GG GG GG GG GG AG GG AG
GG GG GG GG GG GG GG GG
GG GG GG GG GG GG GG GG
GG AG GG GG GG AG GG
88704644
6840 rs10192975 2 AA AA AA AG AA
AG AA AA AA AA AA AA AA
AA AA AA AA AA AA AA AA
AA AA AA AA AA AA AA AA
GG AA AA AA AG AA AG
10887075
6841 rs10193 16 AA AA AA AA AA AA
AA AA AA AA AC AC AC AC
AC AA AA AA AA AA AA AA
AA AA AA AA AA AA AA AA
AA AA AA AA AA AA 50705747
6842 rs10193051 2 AG AA AG AG AA
AG AA AA AA AA AA AA AG
AG AG AG AG AG AA AA AA
AA AA AA AA AA AA AA AA
AG AA AA AA AA AG AA
164370435
6843 rs10193058 2 GG AG GG AG AG
GG AG AA AG AA AG AG AA
AA AA AG GG GG GG GG GG
GG GG GG GG GG AG GG GG
GG AA AG AG AA AA AA
142878411
6844 rs10193067 2 AA AA AA AC AA
AA AC AA AA AA AA AA AA

AA AA AA AA AA AA AA AA
AA AA AA AA AA AC AC AA
AA AA AA AA AA AA AA
52690413
6845 rs10193072 2 AG GG GG AG AG
AG AG AG AA AA AA AA AA
AA AA AA AA AA GG GG GG
AG AG AG AG AA AA AA AA
AG AA AA AA AG AG AA
48528035
6846 rs10193126 2 AG AA AG AA GG
AG AG AA AG AA GG AG AG
GG GG AG AG AG AG AG AA
GG AG GG GG GG GG GG GG
AG AA GG AG GG GG AG
14317807
6847 rs10193128 2 AA AA AA AG AG
AG AA AG AA AA AA AA AA
AA AA AG AA AA AG AG AG
GG GG GG AG AG AG AA AG
AG AA AA AA AA AG AG
233987722
6848 rs10193163 2 GG GG AG AG AG
AA AG AG AG AG GG AA AG
AA AA AG AG GG AG AG AA
AG AG AG AG AG GG GG AG
GG GG AA AG AG AA GG
79352537
6849 rs10193182 2 AA AA AA AG GG
AG GG AG AG AG AG GG GG
AG AG GG AG AG GG GG GG
GG AG GG AG AA AG AG AA
AG AA GG AG AG AG AG
60554216
6850 rs10193188 2 AG GG AG AG AG
GG AG GG GG AG GG GG GG
GG GG GG GG GG GG AA GG
AG AA AA AG AG GG GG AG
AG AG GG GG AG GG AG
152551210
6851 rs10193208 2 AG AG AG AA AA
AA AA AA AA AA AA AA AA
AA AA AG AG AA AA AG AA
AG AA AG AG AG AA AA AA
AG AA AA AA AG AG GG
6398203
6852 rs10193228 2 GG AG AG AG GG
GG AG GG AG GG GG GG GG
GG GG GG AG AG AG AG AA
GG GG GG GG AG GG AG GG

GG GG AG AG GG GG GG
84532773
6853 rs1019325 2 AG AG AG AG AA
AG AA AA AG AA GG GG GG
GG GG AA AG AG AA AG AG
GG GG GG GG AG AG AA AA
AG AA AG AA GG AG GG
8055446
6854 rs10193281 2 AG AG AG AG AG
AA GG GG GG GG GG GG GG
GG GG AG AG AA GG GG GG
GG GG GG AG GG AG GG AG
AG AG AG AA GG GG GG
230373798
6855 rs10193289 2 GG GG AG AG AG
AA GG GG GG GG GG AG GG
AG AG AG AG GG GG GG GG
GG AG AG GG AG AG GG AA
GG AA AG AA GG GG GG
44306480
6856 rs1019329 12 GG GG GG GG GG
GG GG GG GG GG GG GG GG
GG GG GG GG GG GG GG GG
GG GG GG GG GG GG GG GG
GG GG GG GG GG GG GG
16453225
6857 rs10193315 2 AA AA AA AA AA
AA AA AA AA AA AA AA AA
AA AA AA AA AA AA AA AA
AA AA AA AA AA AA AA AA
AA AA AA AA AA AA AA
236662381
6858 rs10193337 2 AA AA AA AA AA
AA AA AA AA AA AA AA AA
AA AA AA AA AA AA AA AA
AA AA AA AA AA AA AA AA
AA AC AA AA AA AA AA
65586769
6859 rs10193417 2 AA AA AA AA AA
AA AA AA AA AA AA AA AA
AC AC AA AA AA AA AA AA
AA AA AA AA AA AA AA AA
AA AC AA AC AA AA AA
146800596
6860 rs10193484 2 GG GG GG GG GG
GG GG GG GG GG GG GG GG
GG GG GG GG GG GG GG GG
GG GG GG GG GG GG GG GG
GG GG GG GG GG GG GG
176248855

6861 rs10193521 2 GG AG GG GG GG
GG GG AG AG AG GG GG GG
GG GG GG GG GG AA AA AG
GG GG GG GG GG GG GG GG
GG GG AG GG GG GG GG
29785679
6862 rs10193630 2 AG AA AG AG AG
AG AG GG GG GG AA AG AG
GG GG GG GG GG GG AG GG
AG AG AG AG AG AG GG AG
AA AG AG GG AG AG AG
140494663
6863 rs10193636 2 CC CC CC AC CC
AC CC CC CC CC CC CC CC
CC CC CC AC CC AC AC AC
CC CC CC CC CC CC CC CC
CC AC CC AC AC AC AC
39522448
6864 rs10193683 2 AG AA AG AG GG
AG AG AA AA AA AA AA AG
AG AG AG AG AG AG AA AG
AA AG AG AA AG AG AG AA
AA AA AG AG AA AA AA
168796136
6865 rs10193706 2 AA AC AA CC CC
CC CC AC AA AC AC AA AC
AA AA CC CC AC AA CC AC
CC AC AC CC AC AC AC AA
CC CC AC AC CC CC CC
146316319
6866 rs10193725 2 AG AA AG AA AA
AA AA AG AA AA AA AA AA
AG AA AG AG AG AA AG AA
AG AG AG AG AA AA AA AA
AA AG AG AA AA AA AG
219726498
6867 rs10193760 2 AA AG GG GG AA
AG AG AG AG AG AA AA AA
AA AA AA AG AG AG AG AA
AG AA AG AA AG AG AG AA
AA AG AG AG GG AG GG
57930015
6868 rs10193766 2 GG AG AG GG AG
AG GG AG GG GG GG GG AG
GG GG AA AG AG AA AA AA
GG AG AG GG AG GG GG AG
AG AG AA AA AG AG AA
46454212
6869 rs10193767 2 AG GG AG AG GG
AG AG AA AG AG GG GG GG

	GG	GG	AG	AG	AG	AA	GG	AA
	GG	AA	AG	AG	GG	GG	GG	GG
	GG	GG	GG	GG	AG	GG	AG	
167174792								
6870	rs10193771		2	AA	AA	AA	AA	AA
	AA	AA	AA	AA	AA	AA	AA	AA
	AA	AA	AA	AA	AA	AA	AA	AA
	AA	AA	AA	AA	AA	AA	AA	AA
	AA	AA	AA	AA	AA	AA	AA	
59707186								
6871	rs10193866		2	GG	GG	GG	GG	GG
	GG	GG	AA	GG	AG	AA	AA	AG
	GG	GG	AG	AG	AG	GG	GG	GG
	AG	AG	AG	AG	GG	GG	GG	GG
	AG	AG	AG	GG	GG	GG	AG	
183489249								
6872	rs1019389		2	GG	GG	GG	AG	AG
	AG	AG	AG	AG	AG	AG	AA	AG
	GG	GG	AG	AG	AA	AG	GG	AG
	AA	AA	AA	AA	AG	AG	GG	AG
	AA	GG	AG	AG	AG	AG	AA	
144664940								
6873	rs10193902		2	AA	AA	AG	GG	AG
	AG	GG	AA	AA	AG	AA	AG	AA
	AA	AA	AG	GG	AA	AA	AA	AA
	AG	AG	AG	AG	AA	AA	AA	AA
	AA	AA	GG	AG	AA	AA	AG	
43411001								
6874	rs10193920		2	GG	GG	GG	GG	GG
	GG	GG	GG	GG	GG	GG	GG	GG
	GG	GG	GG	GG	GG	GG	GG	GG
	AG	GG	AG	GG	AG	GG	GG	AG
	GG	GG	GG	GG	AG	GG	AG	
69292262								
6875	rs10193942		2	AA	AC	AC	AC	AA
	AC	AC	AA	AA	AA	AA	AA	AA
	AC	AC	AA	AA	AA	CC	AA	AC
	AC	AA	AC	AC	AA	AA	AA	AA
	AA	AA	AA	AA	AA	AA	AA	
173608201								
6876	rs10193943		2	AA	AA	AC	AC	CC
	AC	AC	CC	CC	CC	AC	AA	AC
	AC	AC	AC	AC	AA	AA	AA	AC
	AC	AC	AC	AC	CC	AC	AC	CC
	AC	AA	AA	AA	AC	AC	AA	
105992771								
6877	rs10193983		2	GG	GG	GG	GG	GG
	GG	GG	GG	GG	GG	AG	AG	AG
	GG	GG	GG	AG	AG	AG	AG	AA
	GG	GG	GG	GG	GG	GG	GG	GG

AG GG GG GG GG GG GG
1423283
6878 rs1019412 17 GG GG GG GG GG
GG GG AG GG GG AA GG AG
AG GG AG AA AG AG AA AG
GG GG GG GG GG AG GG GG
AG AG GG AG GG GG GG
31488753
6879 rs10194143 2 AA AA AA AA GG
AG AG AA AG AA AG AG AG
AA AA AA AA AA AA AA AA
AA AA AA AA AA AA AA AA
AA AG AA AA AA AA AA
16176894
6880 rs10194165 2 AA AA AA AA AA
AA AA AA AA AA AA AA AA
AA AA AA AA AA AA AA AA
AA AA AA AA AA AA AA AA
AA AA AA AA AA AA AA
199424790
6881 rs10194179 2 AA AA AA AA AA
AA AA AA AA AA AA AA AA
AA AA AA AA AA AA GG AA
AA AG AA AG AA AA AA AA
AA AA AA AA AA AA AA
173097848
6882 rs10194191 2 AG AA AG AA AG
AG AA AG AG AG AA AG AG
AG AG AG AG AA AA AA AA
AA AG AA AA AA AA AA AA
AG AA AA AA AG GG AG
13953494
6883 rs10194249 2 CC CC CC AC CC
CC AC CC AC AC AC AC AA
AA AA CC CC CC CC CC CC
AC AC AC AC CC AC AC CC
AC CC CC CC CC CC CC
235921753
6884 rs10194289 2 CC CC CC AC AC
AA CC CC CC CC AC AC AA
AA AA AC AC AC AA CC AC
CC CC CC CC CC AC AC CC
CC CC CC CC CC CC CC
235402656
6885 rs10194318 2 GG GG GG GG GG
GG GG GG GG GG GG GG GG
GG GG GG GG GG GG GG GG
GG GG GG GG GG GG GG GG
GG GG GG GG GG GG GG
238406446

6886	rs1019433		18	GG	GG	AG	AG	AG
	GG	AA	AA	AG	AG	GG	GG	GG
	GG	AG	AG	AA	GG	AG	GG	AG
	AG	AG	AG	AG	GG	AG	AG	AG
	AA	AA	AG	AA	GG	AG	AG	
62023881								
6887	rs10194368		2	CC	CC	CC	CC	CC
	CC	CC	CC	CC	CC	CC	CC	CC
	CC	CC	CC	CC	CC	CC	CC	CC
	CC	CC	CC	CC	CC	CC	CC	CC
	CC	CC	CC	CC	CC	CC	CC	
97340657								
6888	rs10194375		2	AC	AC	CC	AC	AC
	AC	AA	AA	AA	AA	CC	CC	CC
	AC	AC	CC	AC	CC	AC	CC	AC
	AC	AC	AC	AC	CC	CC	CC	CC
	AC	AA	AC	AC	AC	AC	CC	
127839781								
6889	rs10194381		2	GG	AG	GG	GG	GG
	GG	AG	AG	AG	AG	AG	GG	GG
	AG	AG	AG	AG	AG	AG	AA	AG
	GG	AG	GG	GG	GG	GG	GG	GG
	AG	GG	AG	GG	AG	GG	AG	
5672063								
6890	rs10194386		2	AA	AC	AA	AA	CC
	AC	AC	CC	CC	CC	CC	CC	CC
	CC	CC	CC	AC	CC	CC	CC	CC
	CC	CC	CC	CC	CC	AC	CC	CC
	CC	CC	CC	CC	AC	AA	CC	
18260937								
6891	rs10194413		2	AA	AA	AA	AA	AA
	AA	AA	AG	AA	AG	AG	AA	AA
	AA	AA	AA	AA	AA	AG	AG	AA
	AA	AA	AA	AA	AA	AG	AG	AA
	AA	AA	AA	AA	AA	AA	AA	
101518910								
6892	rs10194449		2	AA	AA	AA	AA	AA
	AA	AA	AA	AA	AA	AA	AA	AA
	AA	AA	AA	AA	AA	AA	AA	AA
	AA	AA	AA	AA	AA	AA	AA	AA
	AA	AA	AA	AA	AA	AA	AA	
236160573								
6893	rs1019445		19	GG	GG	GG	AG	GG
	AG	GG	GG	GG	GG	AG	GG	GG
	GG	GG	GG	GG	GG	GG	GG	GG
	AG	GG	GG	AG	GG	GG	GG	GG
	GG	AG	GG	AG	AG	GG	AG	
18453124								
6894	rs10194509		2	AA	AA	AA	AC	AC
	AA	AC	AC	AC	AC	AA	AA	AA

	AA	AA	AC	AC	AA	--	AC	AC
	AC	AA	AC	AC	AA	AC	AA	AC
	AA	AC	AA	AC	AC	--	AA	
127762988								
6895	rs10194520		2	AA	AG	AA	GG	GG
	GG	AG	AG	AG	AG	AG	GG	AG
	AG	AG	GG	GG	AG	AG	GG	GG
	AG	AG	AA	AG	AG	AG	GG	AG
	GG	GG	AG	GG	AA	AA	AG	
5611779								
6896	rs10194544		2	AG	GG	GG	AG	AG
	AG	AG	AA	AA	AA	GG	AG	AG
	AG	AG	AA	AG	AA	GG	AA	GG
	AA	AA	AG	AG	AA	AG	AG	AA
	GG	AG	AG	AA	AG	AG	AG	
125499930								
6897	rs10194641		2	AG	AG	AA	AG	AA
	AG	AA	AG	AG	GG	AG	AG	AA
	AA	AA	GG	AG	AG	AA	AG	AA
	AG	AG	AA	GG	GG	AA	AG	AG
	AG	AA	AA	AA	AG	AA	GG	
163848125								
6898	rs10194682		2	AA	AA	AA	AA	AA
	AA	AA	AA	GG	GG	AG	AA	AA
	AA	AA	AA	AG	AG	AG	GG	AG
	AG	AA	AG	AA	AG	AA	AG	AG
	GG	AG	GG	AG	AG	AG	AG	
189906743								
6899	rs10194743		2	AG	AG	AG	GG	GG
	GG	GG	GG	AG	AG	GG	GG	GG
	GG	GG	GG	GG	GG	AG	AG	AA
	AG	GG	GG	GG	GG	AG	AG	GG
	AG	AG	AG	AA	AG	AG	GG	
223518151								
6900	rs10194749		2	AA	AA	AA	AA	AA
	AA	AA	AG	GG	AG	AA	AA	AA
	AA	AA	AA	AA	AA	AA	AA	AA
	AA	AA	AA	AA	AG	AG	GG	GG
	AA	AA	AA	AA	AG	AA	AG	
9008904								
6901	rs10194786		2	CC	CC	CC	CC	CC
	CC	AC	AC	AA	AC	AA	AC	AC
	AC	AC	AA	AC	AC	CC	CC	CC
	AA	AC	AA	AA	CC	AA	AC	AC
	AC	AC	AC	AC	AC	CC	AC	
9267131								
6902	rs10194838		2	GG	GG	GG	AG	GG
	GG	AG	GG	GG	GG	GG	GG	GG
	GG	GG	GG	GG	GG	AG	AG	GG
	GG	GG	GG	GG	GG	GG	GG	GG

GG GG GG GG GG AG GG
49003566
6903 rs10194845 2 GG GG GG GG GG
GG GG GG GG GG GG GG GG
GG GG GG GG GG GG GG GG
GG GG GG GG GG GG GG GG
GG GG GG GG GG GG GG
238017470
6904 rs10194900 2 AC AA AA AA AA
AA AC AC AC AC CC AC CC
AC AC AA AC AA AC AC AC
AA AA AA AA AC AC AC AC
AC AA AC AC AA AA AC
72325404
6905 rs1019496 5 GG GG GG AG AG
AG GG GG AG GG GG GG GG
GG GG GG GG GG GG AG GG
AG AG AG AG AG GG GG AG
GG GG AG GG GG GG GG
37773877
6906 rs10195039 2 GG GG GG GG GG
GG GG GG GG GG GG GG GG
GG GG GG GG GG GG GG GG
GG GG GG GG GG GG GG GG
GG GG GG GG GG GG GG
101200213
6907 rs10195063 2 AA AA AA AA AA
AA AA AA AA AA AA AA AA
AA AA AA AC AC AA AA AA
AA AA AA AA AA AA AA AA
AA AA AA AA AA AA AA
84085641
6908 rs1019512 5 AA AG AA AG AG
GG AG GG GG GG AG AG AG
AA AG AA AG AG GG GG AG
GG AG AG GG GG AG GG AG
GG GG AA AG AA AA AG
32317439
6909 rs10195126 2 GG GG GG AG AG
GG AG GG AG GG GG AA AG
AG AG AA AG AA AA AA AG
AG AG AG AG AA AA AA AA
GG AG GG GG AG GG AA
12331619
6910 rs10195227 2 AC AA AC AA AA
AA AA AC AC AA AA AA AA
AA AA AA AA AA AA AA AA
AA AA AA AA AA AA AA AA
AA AC AA AC AC AA AC
154135071

6911	rs10195252	2	AG	AA	AG	AA	AA
	AA	AA	AG	AG	AG	AA	AA	AG
	AG	AG	AG	AG	AG	AA	AG	AA
	AA	AA	AA	AA	GG	AG	AG	AG
	AA	AG	AG	AG	AG	GG	AA	
165513091								
6912	rs1019531	16	AA	AA	AA	AA	AA
	AA	AA	AA	AA	AA	AA	AA	AA
	AA	AA	AA	AA	AA	AA	AA	AA
	AA	AA	AA	AA	AA	AA	AA	AA
	AA	AA	AA	AA	AA	AA	AA	
76191537								
6913	rs10195328	2	AA	AA	AA	AA	GG
	AG	AG	AG	AA	AA	AA	AA	AA
	AA	AA	AA	AA	AA	AG	AG	AG
	AA	AA	AA	AA	AA	AA	AA	AA
	AA	AA	AA	AA	GG	AG	AG	
65328694								
6914	rs10195398	2	AA	AA	AA	AA	AA
	AA	AA	AA	AA	AA	AA	AA	AA
	AA	AA	AA	AA	AA	AA	AA	AA
	AA	AA	AA	AA	AA	AA	AA	AA
	AA	AC	AA	AC	AC	AC	AA	
172272690								
6915	rs10195414	2	AG	AA	AG	AA	AA
	AA	AA	AG	AA	AG	AA	AA	AG
	AG	AG	AA	AA	AA	AA	GG	AA
	AA	AG	AG	AA	AA	AA	AA	AA
	AG	AA	AA	AA	AA	AA	AG	
157478890								
6916	rs10195488	2	AG	AG	AG	GG	AG
	GG	AG	GG	AG	GG	AA	AA	AA
	AA	AA	AA	AA	AG	AG	AA	AA
	AG	AG	AA	AG	AG	AG	AA	AG
	AA	AG	AG	GG	GG	AG	AG	
3824135								
6917	rs10195517	2	GG	GG	AG	GG	GG
	GG	GG	AA	AG	AA	GG	AG	AG
	AA	AA	AG	GG	AG	AA	AA	AG
	GG	AA	GG	GG	AG	AG	AG	AG
	GG	AG	AA	AA	GG	GG	AG	
85364093								
6918	rs1019553	16	AC	AC	CC	CC	CC
	CC	CC	CC	CC	CC	CC	AC	AC
	AC	AC	CC	CC	CC	CC	CC	CC
	CC	CC	CC	CC	AC	CC	CC	AC
	CC	CC	CC	CC	CC	CC	CC	
11396350								
6919	rs10195538	2	AG	GG	AG	GG	GG
	GG	GG	AG	AG	AG	AG	AA	AG

GG GG AG AG GG AG AG AA
GG GG GG GG GG GG GG GG
AG AG AG AA GG AG GG
37917363
6920 rs10195540 2 GG GG GG AG AG
AG AG GG GG GG GG GG GG
GG GG AG GG AG GG GG GG
GG GG GG GG GG GG GG GG
AG GG GG GG GG GG GG
23003253
6921 rs10195542 2 AG AG AG GG GG
AG GG GG GG GG GG AG AG
AG AG GG GG GG GG GG AG
GG AG GG GG AG GG GG AG
GG GG AG GG GG GG GG
103562368
6922 rs10195619 2 GG GG AG AG GG
GG AG AG AG AG AG AG AG
GG GG AG GG AG AG AG AG
AG GG AA AA GG AA AG AG
GG AG GG AG GG GG GG
112765562
6923 rs10195682 2 AG AA AG AG AG
GG AA AA AG AA AA AA AA
AA AA AA AA AA AG AA AG
AA AA AA AA AA AA AA AA
AG AG AA AG AA AA AA
143988118
6924 rs10195777 2 GG GG GG GG GG
GG GG AG AG AG AG AG AG
AG AG GG AG GG GG GG GG
AG AA AG GG AG AG AA AG
GG GG AG AG AG AA GG
15345668
6925 rs10195794 2 AA AA AA AA AG
AA AG AA AA AA AA AA AA
AA AA AG AA AG AA AA AA
AA AA AA AA AA AA AA AA
AG AA AA AA AA AA AA
23016634
6926 rs10195812 2 GG AG AG AA AA
AA AA GG GG GG AG AG AG
AA AA AA AG AA AA AA AA
AA AG AG AG AG AG GG AA
AG AG AG GG AA AG AA
123329724
6927 rs10195834 2 AG AG AA AA AA
AA AA AA AA AA AA AA AA
AA AA AA AA AA AA AA AA
AA AA AA AA AA AA AA AA

	AA	AA	AA	AA	AA	AA	AA
66827052							
6928	rs10195907		2	AA	AA	AA	AA	AA
	AA	AA	AA	AA	AA	AG	AA	AA
	AA	AA	AG	AA	AG	AA	AA	AA
	AG	AA	AG	AA	AA	AG	AA	AG
	AA	AG	AA	AG	AG	GG	AA	
78240557								
6929	rs10195935		2	AA	AG	GG	GG	AG
	AG	GG	AA	AA	AA	AA	AA	AA
	AG	AG	AA	AA	AA	AG	AG	AA
	AG	GG	GG	GG	AG	AG	GG	AA
	AG	GG	AA	AG	GG	AG	AG	
52696911								
6930	rs10195948		2	GG	AG	GG	GG	AG
	GG	AG	GG	GG	GG	GG	AG	AG
	AG	AG	GG	GG	GG	AG	AG	GG
	GG	AG	GG	GG	AG	GG	GG	AG
	AG	GG	GG	GG	GG	GG	AG	
103125736								
6931	rs10195950		2	AA	AA	AA	AA	AG
	AA	AG	AG	AA	AG	AA	AA	AA
	AA	AA	AA	AA	AA	AA	AA	AA
	AA	AA	AA	AA	AA	AG	AG	AG
	AA	AG	AA	AA	AA	AA	AA	
238344285								
6932	rs10195957		2	AA	AA	AA	AA	AA
	AA	AA	AA	AA	AA	AA	AA	AA
	AA	AA	AA	AA	AA	AA	AA	AA
	AA	AA	AA	AA	AA	AA	AA	AA
	AA	AA	AA	AA	AA	AA	AA	
61420408								
6933	rs10195959		2	GG	GG	GG	GG	GG
	GG	GG	GG	GG	GG	GG	GG	GG
	GG	GG	GG	GG	GG	GG	GG	GG
	GG	GG	GG	GG	AG	GG	AG	AG
	GG	GG	GG	GG	GG	GG	GG	
239162318								
6934	rs10196	14	AA	AG	AA	AA	AA	AA
	AA	AA	AA	AA	AG	AG	AG	AG
	AG	AG	GG	GG	AA	AA	AA	AG
	AG	AG	AG	GG	AG	AG	GG	AG
	AG	AG	AG	AA	AA	AG	21485506	
6935	rs10196011		2	GG	GG	GG	AG	AG
	AA	GG	GG	GG	GG	AG	GG	GG
	AG	AG	AA	AA	AA	AA	AG	AG
	AA	AG	AG	AA	GG	GG	GG	GG
	GG	AG	AG	GG	GG	GG	AG	
138283751								
6936	rs10196068		2	AG	AG	AG	GG	AG

	AG	AG	AA	AG	AG	AA	AG	AG
	AG	AG	AA	AA	AA	AG	AA	AG
	AA	AA	AA	AA	AA	AG	AA	AG
	AG	AG	AA	AG	AA	AA	AG	
212338628								
6937	rs1019610		2	AA	AA	AA	AA	AA
	AA	AA	AA	AA	AA	AA	AA	AA
	AA	AA	AA	AA	AA	AA	AA	AA
	AA	AA	AA	AA	AA	AA	AA	AA
	AA	AA	AA	AA	AA	AA	AA	
146879550								
6938	rs10196118		2	GG	GG	GG	GG	GG
	GG	GG	GG	GG	GG	GG	GG	GG
	GG	GG	GG	GG	GG	GG	GG	GG
	GG	GG	GG	GG	GG	GG	GG	GG
	GG	GG	GG	GG	GG	GG	GG	
61304523								
6939	rs1019612		2	AA	AG	AA	AG	AA
	AG	AA	AG	AG	AA	AG	AG	AA
	AA	AA	AG	AG	AG	AG	AG	GG
	AA	AA	AA	AA	AA	AA	AA	AA
	AG	AG	AA	AA	AA	AG	AA	
181602526								
6940	rs1019614		2	AG	AG	AA	GG	AA
	AG	AG	GG	GG	GG	AG	AA	AA
	AG	AG	AG	AA	AG	AA	AA	AA
	AA	AA	AA	AA	AA	GG	AG	AG
	AA	AG	AG	AA	AG	AG	AG	
60131157								
6941	rs10196158		2	GG	AG	GG	AA	AA
	AA	AG	AA	AA	AA	AA	AA	AA
	AG	AG	AG	AG	GG	AA	AG	AA
	AA	GG	AG	AG	AG	AA	AA	AG
	AG	AG	GG	GG	AG	AG	GG	
128932760								
6942	rs10196178		2	GG	GG	GG	GG	GG
	GG	GG	GG	GG	GG	GG	GG	GG
	GG	GG	GG	GG	GG	GG	GG	GG
	GG	GG	GG	GG	GG	GG	GG	GG
	GG	GG	GG	GG	GG	GG	GG	
241148909								
6943	rs10196223		2	AA	AA	AA	AA	AA
	AA	AA	AG	AG	AG	AA	AA	AA
	AA	AA	AA	AA	AA	AA	AA	AA
	AA	AG	AA	AA	AA	AA	AA	AA
	AA	AA	AA	AA	AA	AA	AA	
112392625								
6944	rs10196272		2	AG	AG	AA	AA	AG
	AG	AA	AG	AG	AG	AG	GG	AG
	AG	AG	AG	AA	GG	AG	AA	GG

	GG	GG	GG	GG	AG	AG	AG	AG
	AA	AG	AA	AA	AA	AG	AA	
70826094								
6945	rs10196456		2	AG	AG	AG	AA	AA
	AA	AA	AA	AG	AA	GG	AA	AG
	AA	AA	AG	AG	AA	AA	AA	AA
	AA	AA	AA	AA	AA	GG	AG	AG
	AA	AA	AG	AA	AA	AA	AA	
73158476								
6946	rs10196485		2	GG	GG	GG	GG	GG
	GG	GG	GG	GG	GG	GG	GG	GG
	GG	GG	GG	GG	GG	GG	GG	GG
	GG	GG	GG	GG	GG	GG	GG	GG
	GG	GG	GG	GG	GG	GG	GG	
198067004								
6947	rs10196514		2	GG	GG	GG	GG	GG
	GG	GG	GG	GG	GG	AG	GG	GG
	GG	GG	GG	GG	GG	GG	GG	GG
	GG	GG	GG	GG	AG	GG	GG	GG
	GG	GG	AG	AG	GG	GG	GG	
1494373								
6948	rs10196530		2	AA	AA	AG	AA	AA
	AG	AA	AA	AA	AA	AG	GG	AG
	AA	AA	AA	AA	AA	AA	AA	AA
	AG	AA	AA	AA	AA	AG	AG	AA
	AA	AG	AA	AA	AA	AG	AG	
131762450								
6949	rs10196553		2	GG	GG	GG	GG	GG
	GG	GG	GG	GG	GG	GG	GG	GG
	GG	GG	GG	GG	GG	GG	GG	GG
	GG	GG	GG	GG	GG	GG	GG	GG
	GG	GG	GG	GG	GG	GG	GG	
199690089								
6950	rs10196564		2	AA	AA	AA	AA	AA
	AA	AA	AA	AA	AA	AG	GG	GG
	AA	AA	AG	AA	AG	AA	AA	AG
	AA	AA	AA	AA	AA	AA	AA	AA
	AA	AG	AA	AG	AG	AG	AA	
59423256								
6951	rs10196575		2	AA	AA	AG	AA	AA
	AA	AA	AA	AA	AA	AG	AG	AG
	AA	AG	AA	AG	AG	AA	AA	AG
	AA	AA	AA	AA	AG	AA	AA	AG
	AA	AG	AA	AG	AA	AA	AA	
119533754								
6952	rs10196602		2	CC	AC	AA	CC	AC
	AC	CC	AC	AC	AC	AC	AC	AC
	CC	CC	AC	AC	AC	CC	CC	CC
	AC	AC	AC	AC	AC	AC	CC	AA
	CC	AC	CC	AC	CC	CC	CC	

39772004
6953	rs10196612	2	AG	AG	AG	AG	AA
	AG	AA	AG	AA	AA	AG	AG	GG
	GG	GG	AG	AG	AG	AA	AG	AA
	AG	AG	AG	AG	AG	AG	GG	AG
	GG	AG	AA	AG	AG	AG	AA
198894139
6954	rs10196645	2	AG	AG	AA	AG	GG
	AG	GG	AG	AG	AG	GG	AG	AG
	GG	GG	GG	GG	GG	GG	AG	GG
	AG	AG	AG	AG	AG	AG	AG	AG
	GG	AG	GG	AG	GG	GG	AG
33951513
6955	rs10196649	2	GG	GG	GG	GG	GG
	GG	GG	GG	GG	GG	GG	GG	GG
	GG	GG	GG	GG	GG	GG	GG	GG
	GG	GG	GG	GG	GG	GG	GG	GG
	GG	GG	GG	GG	GG	GG	GG
112056290
6956	rs10196671
2	--	--	--	--	--	--	--	--
	--	--	--	--	--	--	--	--
	--	--	--	--	--	--	--	--
	--	--	--	--	--	--	--	--
	--	--	--	--	186809595
6957	rs1019668	15	AA	AC	AC	CC	AC
	AC	AC	AA	AA	AA	CC	CC	CC
	AA	CC	AA	AA	AA	CC	CC	CC
	AC	AA	AA	AA	AC	AC	CC	AA
	AA	CC	AC	AC	AC	CC	AA
84381209
6958	rs1019673	3	CC	CC	CC	AA	AA
	AA	AA	AC	AC	AA	CC	CC	CC
	CC	AC	AC	AC	AC	AC	AC	AC
	AA	AA	AA	AA	AC	AC	AC	AC
	AC	CC	AC	AC	CC	CC	CC
188258905
6959	rs10196737	2	AG	GG	AG	AG	GG
	AG	GG	GG	GG	GG	AG	AA	AA
	AG	AG	AG	GG	GG	GG	GG	AG
	AG	AG	AG	AG	GG	AG	AG	GG
	GG	AA	GG	AG	GG	GG	GG
57805439
6960	rs10196764	2	AG	GG	AG	GG	GG
	GG	GG	AG	GG	GG	GG	GG	GG
	GG	GG	AG	AG	AG	GG	GG	GG
	AG	AG	AA	AG	GG	GG	GG	GG
	GG	GG	AG	GG	AG	GG	AG
207229528
6961	rs10196774

2 -- -- -- -- -- -- -- --
-- -- -- -- -- -- -- --
-- -- -- -- -- -- -- --
-- -- -- -- -- -- -- --
-- -- -- -- 237421250
6962 rs10196800 2 GG GG GG GG GG
GG GG GG GG GG GG GG GG
GG GG GG GG GG GG GG GG
GG GG GG GG GG GG GG GG
GG GG GG GG GG GG GG
62556261
6963 rs10196857 2 AA AA AA AA AA
AA AA AA AG AG AG AG GG
GG GG AA AA AA AA AG AA
AA AA AA AA GG AA AG AG
AA AA AA AA AA AA AA
241674337
6964 rs10196869 2 CC CC CC CC CC
CC CC CC CC CC CC CC CC
CC CC CC CC CC CC CC CC
CC CC CC CC CC CC CC CC
CC CC CC CC CC CC CC
147250316
6965 rs10196882 2 GG GG GG GG GG
GG GG GG GG GG GG GG GG
GG GG GG GG GG GG GG GG
AG GG GG AG AG AG AA AG
AG GG GG GG AG GG AG
160891055
6966 rs10196888 2 AA AA AG AA AG
AA AG AA AA AA AA AA AA
AA AA AA AA AA AA AA AA
AA AG AA AG AA AA AA AA
AA AA AA AA AA AA AA
56036226
6967 rs10196901 2 GG GG GG GG GG
GG GG GG GG GG GG GG GG
GG GG GG GG GG GG GG GG
GG AG GG GG GG GG GG GG
GG GG AG GG AG AG GG
176732191
6968 rs10196948 2 GG GG GG AG AG
GG AG GG GG GG GG GG GG
GG GG GG GG GG GG AG GG
GG GG GG GG GG GG GG GG
GG AG GG GG AG GG AG
130111979
6969 rs10196949 2 AG AA AG AG AG
AA AG AA AG AG AG AG GG
AG AG AG AG GG AG AG GG

GG AG AG GG AG AG AG GG
AG AA AG AA GG AG AG
159680230
6970 rs10196996
2 -- -- -- -- -- -- -- --
-- -- -- -- -- -- -- --
-- -- -- -- -- -- -- --
-- -- -- -- -- -- -- --
-- -- -- -- 149167935
6971 rs10197003 2 AG AG AG AG AG
AG AG AG AG AG AG AG AG
AG AG AG AG AG GG GG AG
AG AA AA AG AG AG AG AG
AA AG AG AG AA AG AG
137102171
6972 rs10197071 2 AA AA AA AA AA
AA AA AA AA AA AA AA AA
AA AA AA AA AA AA AA AA
AA AA AA AA AA AA AA AA
AA AA AA AA AA AA AA
38833093
6973 rs1019709 12 AG AG AG AA AA
AA AA AG GG GG AG AG AA
AG AG AG AG AG AA AA AA
AG GG AG GG GG AG AG AG
AG AA AA AA AG AA AG
40802150
6974 rs10197121 2 AA AA AA GG AG
AG AG AG AG AG AA AG AG
AG AG AG AA AG AG AA AA
AG GG AG AG AA AA AA AA
AG GG AA AG AG AA GG
10293689
6975 rs10197140 2 AG AA AA AG GG
AG AG AA AA AA AG GG GG
AG AG AG AG AA AA AA AA
AG GG AG AG GG AG AG GG
AG AA AA AA AG AG AA
111609943
6976 rs10197180 2 AA AC CC AA AC
AC AA AC AA AA CC CC CC
AA AA AC AC AC AA AA AC
AA AA AA AA AA AA AA AA
AA AA AA AA AA AC AA
67198954
6977 rs10197202 2 AG AA AG AA AG
AG AA AA AA AA AG AA AG
AG AA AA AG AG AA AA AA
AA AA AG AG AA AG AG AA
AG AA AG AG AA AA AA

197522966
6978 rs10197222 2 AG GG AG AA AG
AA AG GG GG GG GG GG GG
GG GG AA AG AG AG AG AA
AA AG AA AA AG GG GG AG
AG GG AG GG AG AG GG
112627223
6979 rs10197237 2 AG AG GG AG AG
AG GG GG AG AG AA GG AG
AG AG GG AG AG GG GG GG
AG AG AG AG GG GG GG GG
AG GG AG GG GG GG GG
242322640
6980 rs10197243 2 GG GG GG GG AG
GG AG AG AG AG GG GG GG
GG GG GG GG AG GG GG GG
GG GG GG GG AG GG GG GG
GG GG GG GG GG GG GG
55052493
6981 rs10197258 2 AA AA AA AA AG
AG AA AA AA AA AA AA AA
AA AA AA AA AA AA AG AG
AA AG AA AG AG AA AG AG
AG AA AA AA AG -- AG
178012446
6982 rs10197319 2 GG AG GG AG GG
AG GG AA GG GG AA AG AA
AA AA AA AG AG GG GG AG
AG AG AG AG AG AA AG AG
AA GG AG AG AG AG AG
204763044
6983 rs10197322 2 AG AG AG GG GG
AG AG AG AA AA GG GG GG
AG AG AG AG AA AG AG GG
AA AG AG AA AA GG AG AG
GG AG AA AA AG AG AG
146483034
6984 rs10197378 2 GG GG GG GG GG
GG GG AA AG AA AA AG AG
GG GG AG GG AG GG GG GG
AG GG AA AG GG AG AG GG
AG GG GG GG AA AG AG
29181107
6985 rs10197382 2 AA AA AA GG GG
GG GG AA AA AA AG AG GG
GG GG AG GG AA AG GG GG
AG AG AG AG AG GG GG GG
GG AG AG AA AA AA AG
225574650
6986 rs10197426 2 GG GG GG GG GG

	GG	GG	GG	GG	GG	GG	GG	GG
	GG	GG	GG	GG	GG	GG	GG	GG
	GG	GG	GG	GG	GG	GG	GG	GG
	GG	AG	GG	AG	GG	AG	GG	
203845646								
6987	rs1019743		5	AA	AA	AA	AG	GG
	GG	AG	AA	AA	AA	AA	AA	AG
	AA	AA	AA	AA	AA	AA	AG	AA
	AA	AA	AA	AA	AG	AA	AA	AA
	AA	AG	AG	AG	AA	AA	AA	
111682654								
6988	rs10197459		2	AG	AG	AG	AA	AA
	AA	AA	AA	AA	AA	AA	AA	AA
	AG	AA	AA	AA	AA	AA	AA	AA
	AG	AG	AG	AG	AA	AG	AG	AG
	AA	AG	AG	AA	AA	AA	AA	
148282050								
6989	rs10197460		2	AC	AC	AC	AC	CC
	AC	AC	AC	AC	AC	AC	AC	CC
	CC	CC	AC	AC	AA	AC	AC	AC
	CC	CC	CC	CC	AC	AC	CC	AC
	AC	AC	AC	AA	CC	AC	AC	
234589190								
6990	rs10197470		2	AC	AC	CC	AA	AA
	AA	AA	AC	AC	AC	AC	AC	AA
	AC	AC	AC	AA	AC	AA	AC	AA
	AA	AC	AA	AC	AC	AC	CC	AC
	AC	AC	AA	AC	AA	AA	AC	
164609151								
6991	rs10197525		2	AA	AA	AA	AC	AA
	AA	AC	AA	AA	AA	AA	AA	AA
	AA	AA	AA	AA	AA	AC	AA	AC
	AA	AA	AA	AA	AC	AC	AC	CC
	AA	AC	CC	CC	AA	AA	AA	
2368632								
6992	rs10197566		2	AG	AG	AG	AG	AG
	AA	AG	AA	AA	AA	AA	AA	AA
	AA	AA	AA	AA	AA	AA	AA	AA
	AA	AG	AG	AG	AG	AA	AG	AA
	AA	AG	AA	AG	AA	AA	AA	
133188533								
6993	rs10197595		2	AA	AA	AA	AA	AA
	AA	AA	AA	AA	AA	AA	AA	AA
	AA	AA	AA	AA	AA	AA	AA	AA
	AA	AA	AA	AA	AA	AA	AA	AA
	AA	AA	AA	AA	AA	AA	AG	
10956867								
6994	rs10197614		2	AG	AG	AG	GG	GG
	GG	GG	GG	GG	GG	GG	AG	GG
	AG	AG	GG	GG	GG	AG	GG	AG

GG GG GG GG GG GG GG GG
GG GG AG AG GG GG GG
146164786
6995 rs1019768 5 GG GG AG GG GG
GG GG AA AG AA GG AG GG
AG GG AG AG AA AA AA AA
AG AG AA AG AG GG AG AG
AG GG AA AG AG AG AG
28950461
6996 rs10197778 2 AG AG AG AG AA
AG AG AG GG GG AA GG AG
AG AG GG GG GG AG GG AG
GG GG GG AG AG AG AG AA
GG AA AA AA GG AG GG
223698442
6997 rs10197781 2 AA AA AA AA AA
AA AG AA AA AA AA AA AA
AA AA AA AG AG AG AA AG
AG AA AG AA AA AA AA AA
AA AA AA AA AA AA AA
186173063
6998 rs10197797 2 AA AA AA AA AA
AA AA AA AA AA AA AA AA
AA AA AA AA AA AA AA AA
AA AA AA AA AA AA AA AA
AA AA AA AA AA AA AA
198261089
6999 rs10197805 2 AA AA AA AG AG
AG AA AA AA AA AA AA AA
AA AA AA AG AA AG AG AA
AA AA AA AA AA AA AA AA
AG AG AA AA AA AA AA
225750753
7000 rs10197833
2 -- -- -- -- -- -- -- --
-- -- -- -- -- -- -- --
-- -- -- -- -- -- -- --
-- -- -- -- -- -- -- --
-- -- -- -- 201904466
7001 rs1019786 19 GG GG GG GG GG
GG GG GG GG GG GG GG GG
GG GG GG AG GG GG GG GG
AG GG GG AG GG GG GG GG
GG AG GG GG AG GG AG
48581142
7002 rs10197888 2 AG AG AG AG AG
AG AG AG AG AG GG AG AG
GG AG GG GG AG AG AG AG
GG GG AG GG GG GG GG GG
GG GG GG GG GG GG AG

23370317
7003 rs10197906 2 GG GG AG GG GG
GG GG AG AG AG GG GG GG
GG GG GG GG GG GG GG GG
GG GG GG GG GG GG GG GG
GG GG GG GG AG GG AG
36432419
7004 rs10197919 2 AG AA AA AG GG
AG AG GG GG AG AA AA AA
AG AG AA AA AA AG AG GG
AG GG AA AA AG AG GG AA
GG AG GG AG GG AG AG
112924973
7005 rs10197940 2 GG AG AG AA AA
AA AA AA AA AA AG AG GG
AG AG AG AG AG AA AA AA
AG AG AG AG AA AG AA AA
AG AG GG AG AA AG AG
152253918
7006 rs10197964 2 AA AA AA AA AA
AA AA AG AG AG AA AA AA
AA AA AA AA AG AA AG AA
AA AA AA AA AA GG AG AG
AA AG AG GG AA AG AG
140948967
7007 rs1019798 16 AG AA AG GG AG
GG AG AA AA AA AA AA AA
AG AA AA AA AA AA AG AG
AA AA AA AA AG AG GG AG
AG AA AG AG AA AA AA
9534199
7008 rs10198004 2 GG GG GG GG GG
GG GG GG AG AG AG AA AA
GG GG GG GG GG GG GG GG
GG GG GG GG GG GG GG GG
GG GG GG GG AG AG GG
52885422
7009 rs1019808 5 AG AG GG AA AA
AA AA AA AA AA AA AA AA
AG AG AA AG AG AG AG GG
AG AG AA AA AA AG AG AG
AA AG AA AG AA AG AA
175037358
7010 rs10198084 2 GG GG GG GG AG
AG AG AA AG AG AA AG AA
AA AG AA AG AG GG AA GG
AG AG GG GG AG AG AG GG
AA GG AG GG GG GG AG
205147331
7011 rs1019810 5 AG AG AA GG GG

GG AG GG AG GG AG AG AG
AG GG AG AG GG GG GG GG
GG GG GG GG GG GG GG GG
GG GG AG GG AA AA AA
15598629
7012 rs10198113 2 AA AA AA AG AG
AG AG AG AG AA AA AG AG
GG GG AA AG AA AA AG AA
AG AA AG AA AA AG AG AG
AA AA AA AA GG AG AG
156808365
7013 rs10198127 2 AA AA AA AA AA
AA AA AA AA AA AA AA AG
GG GG AG AA AA AA AA AA
AA AA AA AA AG AA AA AA
AA AA AG AA AA AA AA
177912203
7014 rs1019813 16 AA AG AG AA AG
AA AG AG AG AG GG GG GG
AG AG AG GG AG GG GG AG
AA AG AA AA AG AG AG AG
AG AG AG GG AA AA AG
11940829
7015 rs10198160 2 AG AG AG AG AG
AA GG AA AA AG AG AG AG
GG GG AG AA GG AA AG AG
AG GG GG AG AG AA AG AA
AG GG AG GG GG AG GG
38611930
7016 rs10198215 2 AA AA AG AG AA
AA AG AG GG AG AA AA AA
AA AA AA AG AG GG AG AG
GG GG GG GG AG AA AG AG
AG GG AG AG AA AA AA
239165909
7017 rs10198258 2 AA AA AA AG AG
GG AA AA AA AG AA AA AA
AA AA AG GG AG AG AG AG
AA AA AG AG GG AG AG GG
AA GG AG AG AA AG AA
121202941
7018 rs10198291 2 AA AA AA AA AG
AG AA AG AG AA AG AG AG
AG AG AA AA AA AG -- --
AA AA AA AA AA AA AA AA
AA AA GG AG AA AA AG
44539009
7019 rs10198323 2 AG AG AG AA AA
AA AA AA AA AG AG AG AG
AA AA AG AA AG AA AG AA

AA AA AA AA AG AA AG AA
AA AA AA AA AG AG AA
38571255
7020 rs10198324 2 AA AA AA AA AA
AA AA AA AA AA AA AA AA
AA AA AA AA AA AC AA AA
AA AA AA AA AA AA AA AA
AA AA AA AA AA AA AA
195417908
7021 rs10198382 2 AA AA AA AA AA
AA AA AA AA AA AA AA AA
AA AA AA AA AA AA AA AA
AA AA AA AA AA AA AA AA
AA AA AA AA AA AA AA
58332757
7022 rs1019841 2 AA AA AA AA AA
AG AG GG GG GG GG GG AG
AG AG AG AG AA GG AG GG
GG GG GG GG AG GG GG AG
GG GG AG AG AG AG GG
133514933
7023 rs10198430 2 GG GG GG GG GG
GG GG GG GG GG GG GG GG
GG GG GG GG GG GG GG GG
GG GG GG GG GG GG GG GG
GG GG AG AG GG GG GG
235434525
7024 rs10198445 2 GG AG GG AG AG
GG AA GG AG GG AA AA AG
AG GG AA AA AA AG GG AG
GG GG GG GG AG GG AG AG
AG AG AA AG AA AG AA
184392834
7025 rs10198541 2 GG GG AG AA AG
AG AA GG GG GG AG AA AG
AG AG GG AG GG GG AG AG
AG AG AG AG GG AG AG GG
GG GG AG AG AA AG AG
41892171
7026 rs1019856 3 GG GG GG AG AG
GG AG AG AG AG AG GG GG
AG AG AG AG GG GG AG GG
AG GG AG AG GG GG GG GG
GG GG GG GG GG GG GG
30696816
7027 rs10198564 2 AA AA AA AA AA
AA AA AA AG AG AG AA AA
AA AA AA AA AA AA AA AA
AA AA AA AA AA AA AA AA
AA AG AA AG AA AG AA

51975932
7028 rs10198586 2 GG AG AG GG GG
GG GG GG GG GG GG GG GG
GG GG GG GG GG GG GG GG
GG GG GG GG GG GG GG GG
GG GG GG GG GG GG GG
59082438
7029 rs10198592 2 AA AA AA AA AA
AA AA AA AA AA AA AA AA
AA AA AG AG AA AA AA AA
AA AA AA AA AA AA AA AA
AA AA AA AA AA AA AA
98935976
7030 rs10198663 2 GG GG GG AG GG
AG GG AG AG AG GG GG GG
GG GG GG GG GG GG AA GG
GG GG GG GG GG AG AG GG
GG GG GG GG GG GG GG
190357442
7031 rs10198735 2 GG GG GG GG GG
GG AG AG AG AG AG AG AA
AG AG AG AG AG GG AA GG
AG AA AA AA AG GG GG AG
AG GG AG GG AA AG AG
133307991
7032 rs10198749 2 AA AC AA AA AA
AA AA AA AC AA AA AA AA
AA AA AA AA AA AA AA AA
AA AA AA AA AA AC AC AA
AA AC AA AC AC AC AA
210044484
7033 rs10198766 2 AG AA AG AA AA
AA AA AA AA AA AA AA AA
AA AA AA AA AA AA AA AG
AA AA AA AA AA AA AA AA
AA AA AA AA AA AA AA
168280495
7034 rs1019880 8 CC CC CC AC CC
AC AC CC CC AC CC CC CC
CC CC AA AC AC CC CC AC
AA AC AC AA AC CC AC AC
AC AC CC CC AC CC AC
73408980
7035 rs10198807 2 AG AA AA AG AA
AA AG AA AA AA AA AA AA
AG AG AG GG AA AG AG AG
AG AG AG AG AA GG AG AG
AG AA AG AA GG AG AG
33216805
7036 rs10198826 2 GG GG AG AA GG

	AG	AG	AG	AG	AG	AG	AA	AA
	GG	GG	AG	AG	AA	AG	AG	AG
	AG	AG	AG	AG	AG	AA	AG	AG
	AG	AG	AG	AA	GG	AG	GG	
60910033								
7037	rs10198903		2	GG	GG	GG	AG	GG
	AG	GG	AG	AG	AG	AG	GG	GG
	GG	GG	GG	AG	GG	GG	GG	GG
	AG	AA	AG	AA	GG	AG	GG	AG
	GG	AG	AG	GG	GG	GG	GG	
71907425								
7038	rs10198972		2	GG	GG	GG	GG	GG
	GG	GG	GG	GG	GG	GG	GG	GG
	GG	GG	GG	AG	GG	GG	GG	GG
	GG	GG	GG	GG	GG	GG	GG	GG
	AG	GG	GG	GG	GG	GG	GG	
21453211								
7039	rs10198978		2	GG	GG	GG	GG	GG
	GG	GG	GG	GG	GG	GG	GG	GG
	GG	GG	GG	GG	GG	GG	GG	GG
	GG	GG	GG	GG	GG	GG	GG	GG
	GG	GG	GG	GG	GG	GG	GG	
3466385								
7040	rs1019898		1	CC	CC	CC	CC	CC
	CC	CC	CC	CC	CC	CC	CC	CC
	CC	CC	CC	CC	CC	CC	CC	CC
	CC	CC	CC	CC	CC	CC	CC	CC
	CC	CC	CC	CC	CC	CC	CC	
214204458								
7041	rs10198982		2	AA	AA	AA	AG	AA
	AG	AA	AG	AA	AG	AA	AA	AA
	AA	AA	AA	AA	AA	AG	AA	AG
	AG	AG	AA	AA	AA	AG	AG	AA
	AG	GG	AA	AG	AA	AA	AA	
188888319								
7042	rs10198989		2	GG	GG	GG	GG	GG
	GG	GG	AG	AG	AG	GG	AG	AG
	AG	AG	GG	GG	GG	GG	GG	AG
	GG	AG	GG	AG	AG	AG	AG	AG
	GG	GG	GG	GG	GG	GG	GG	
71335872								
7043	rs10198990		2	AA	AG	AG	AA	AG
	AG	AA	AG	AG	AG	AA	AA	AA
	AA	AA	AG	AA	AA	AA	AA	AA
	AA	AG	AA	AG	AA	AA	AA	AA
	AA	AA	AG	AG	AG	AG	AG	
64299693								
7044	rs10199004		2	GG	GG	GG	AG	AG
	GG	AG	GG	GG	GG	GG	GG	GG
	GG	GG	GG	GG	GG	GG	AG	GG

	GG	GG	GG	GG	GG	GG	GG	GG
	AG	GG	GG	GG	GG	GG	GG	
143244498								
7045	rs10199014		2	GG	AG	AG	AG	AG
	AG	AG	GG	GG	GG	GG	GG	GG
	GG	GG	GG	GG	GG	GG	GG	GG
	GG	AG	AG	AG	GG	GG	GG	GG
	GG	AG	GG	AG	GG	GG	GG	
52514493								
7046	rs10199049		2	AA	AA	AA	AA	AA
	AA	AA	AA	AA	AA	AA	AA	AA
	AA	AA	AA	AA	AA	AA	AA	AA
	AA	AA	AA	AA	AA	AA	AA	AA
	AA	AA	AA	AA	AA	AA	AA	
88402255								
7047	rs10199068		2	AA	AA	AA	AA	AA
	AA	AA	AA	AA	AA	AA	AA	AA
	AA	AA	AA	AA	AA	AA	AA	AA
	AA	AA	AA	AA	AA	AA	AA	AA
	AA	AA	AA	AA	AA	AA	AA	
46119646								
7048	rs10199119		2	GG	GG	GG	GG	GG
	GG	GG	GG	GG	GG	GG	GG	GG
	GG	GG	GG	GG	GG	GG	GG	GG
	GG	GG	GG	GG	GG	GG	GG	GG
	GG	GG	GG	GG	GG	GG	GG	
203210680								
7049	rs10199128		2	AA	AA	AA	AA	AG
	AA	AG	AA	AA	AA	AA	AA	AA
	AA	AA	AA	AA	AA	AA	AA	AA
	AA	AA	AA	AA	AA	AA	AA	AA
	AA	AA	AA	AA	AA	AA	AA	
224102115								
7050	rs10199249		2	AG	GG	AG	GG	GG
	GG	GG	AG	AG	GG	GG	GG	GG
	GG	GG	GG	GG	GG	AG	AG	AG
	GG	GG	GG	GG	GG	GG	GG	GG
	AG	AG	AG	GG	GG	GG	GG	
163571544								
7051	rs10199285		2	AA	AA	AA	AA	AA
	AA	AA	AA	AA	AA	AA	AA	AA
	AA	AA	AA	AA	AA	AA	GG	AA
	AG	AG	AG	AG	AA	AA	AA	AA
	AG	AA	AA	AA	AA	AA	AA	
134195981								
7052	rs10199328		2	AG	AA	AG	AG	AG
	GG	AA	AG	GG	AG	AA	AG	AG
	AG	AG	GG	GG	GG	GG	GG	AG
	GG	AG	AG	GG	AG	GG	AG	GG
	AA	AG	AG	AA	AA	AG	AG	

17487397
7053 rs10199331 2 AA AA AA AA AA
AA AA AA AA AA AA AA AA
AA AA AA AA AA AA AA AA
AA AA AA AA AA AA AA AA
AA AA AA AA AA AA AA
89034338
7054 rs10199334 2 AG GG AG AG AA
AG AA AA AA AA AA AA AA
AA AA AA AA AA AA AA AA
AG AA AG AG AA AA AA AA
AA AA AG AG AA AA AA
24392618
7055 rs1019934 5 AA AA AA AA AA
AA AA AA AA AA AA AA AA
AA AA AA AA AA AA AA AA
AA AA AA AA AA AA AA AA
AA AA AA AA AA AA AA
171870436
7056 rs1019937 19 GG GG AG AG AG
GG AA AA AA AA GG AG AG
GG GG AG AG AA AG AG AG
GG GG GG GG GG AG AG AG
AA AG AG AG AG AA AG
30836248
7057 rs10199402 2 GG GG GG GG GG
GG GG GG GG GG GG GG GG
GG GG GG GG GG GG GG GG
GG GG GG GG GG GG GG GG
GG GG GG GG GG GG GG
62958658
7058 rs10199423 2 GG GG GG GG AG
GG AG AG GG AG GG AG AG
GG GG AG AG AG AG AG AG
AG AG AA AG AG GG GG GG
AG AG GG AG AA AG AG
14420731
7059 rs10199432 2 AG AA AG AA AG
AG AA AG AA AA AA AA AA
AG AG AA AA AA AA AA AA
GG AG GG AG AA AA AA AA
AA AA AA AA AA AG AA
68049568
7060 rs10199463 2 GG GG GG GG GG
GG GG GG GG GG GG GG GG
GG GG GG GG GG GG GG GG
GG GG GG GG GG GG GG GG
GG GG GG GG GG GG GG
98830162
7061 rs10199474 2 AG GG AG GG GG

GG GG GG GG GG GG GG GG
GG GG GG GG GG AA GG AG
GG GG GG GG GG GG GG GG
AG GG GG GG AG AG GG
156476895
7062 rs10199512 2 GG AG GG AG AG
AG GG AG GG GG AG AG AA
AA AA AG AG GG GG GG GG
AG AG AG AG AG GG AG AG
GG AG GG GG AG GG AG
234691401
7063 rs10199515 2 CC CC AC AC AC
CC AC CC CC CC CC CC CC
CC CC CC CC CC AC CC CC
CC CC CC CC AC CC AC CC
AC CC AC CC CC CC CC
114493315
7064 rs10199559 2 GG GG GG GG GG
GG GG GG GG GG GG GG GG
GG GG GG GG GG GG GG GG
GG GG GG GG GG GG GG GG
GG GG GG GG GG GG GG
111547987
7065 rs10199614 2 AG GG AG GG GG
GG GG GG GG GG GG GG GG
GG GG GG GG GG GG GG GG
GG GG GG GG GG AG AG AG
AG GG GG GG GG GG AG
20292618
7066 rs10199676 2 CC CC CC AC CC
AC CC CC CC CC CC CC CC
CC CC CC AC CC CC CC AC
CC CC CC CC AA CC AC AC
AC CC CC CC AC AC CC
170208975
7067 rs10199699 2 CC AC CC AA AA
AA AA AA AC AC AA AC AA
AC AC AA AA AA CC AA AC
AA AC AC AA AC AA AA AC
AA AA AC AC AA AA AA
146300691
7068 rs10199714 2 GG AG AG GG GG
GG GG AG AG AG AA AA AA
GG GG GG GG GG AG AG AG
GG AG AG AG GG AG AG GG
AG AG AA AA GG GG AG
52326591
7069 rs10199719 2 CC AC CC CC CC
CC CC AC AC AC AA AA AA
AC AC AC AC AC AC AA AC

CC CC CC CC CC AC CC CC
AC AC AA AC AC CC AC
149159429
7070 rs10199753 2 AA AA AA AG AG
AG AA GG AG GG AA AA AA
AA AA AG AG AA AA AA AA
AG AG AG AG AA AG AA AG
AA AA AG AA AG AG AG
101084600
7071 rs10199771 2 AA AA AA AA AG
AA AG AA AG AA AG AA AA
AA AA AG AG GG AG AG GG
AG GG AA AG GG AA AG AG
AG AA AA AA AG AA AG
25921254
7072 rs10199787 2 AG AG AG AA AG
AG AG AG AG AG GG GG GG
GG GG GG AG GG AG AG AA
GG GG GG GG AA AG AA AA
AG GG GG GG GG AG GG
11092613
7073 rs10199796 2 AC CC CC CC CC
AC AC AC AC AA AC AC AC
AA AC AA AA AA AC AC AC
AA AC AA AA AA AC AA AC
AA AC AA AA AC AC AA
115891114
7074 rs10199838 2 AA AA AA AA AA
AA AA AA AA AA AA AA AA
AA AA AA AA AA AA AA AA
AA AA AA AA AA AA AA AA
AA AA AA AA AA AA AA
57380815
7075 rs10199914 2 AG GG GG GG GG
AG GG AG AA AA AA AG AA
AA AA AG AG AG AA AG AG
AG AG AG AG AG AG AG AG
AG AG AA AG AA AG AA
239896861
7076 rs1019995 8 AA AA AA AA AG
AA AG AA AA AA AA AA AA
AA AA GG AG AG AA AG AA
AA AA AA AA AA AA AA AA
AA AA AG AA AG AG AA
69119399
7077 rs1019999 2 AG GG AG AG AG
GG AA AA AA AA AA AA AA
AA AA AG AG GG AG AG GG
AG AA AG AG GG AA AG AG
AA AG AA AA AG AG AG

14805647								
7078	rs10200021		2	AA	AA	AA	AA	AA
	AA	AA	AG	AA	AA	AA	AA	AA
	AA	AA	AA	AG	AG	AG	AA	GG
	AA	AA	AA	AA	AA	GG	AG	AG
	AA	AA	GG	AG	AA	AG	AG	
196414587								
7079	rs1020010		2	AG	AA	AG	GG	GG
	GG	AG	GG	AG	GG	AG	AG	AG
	GG	GG	AG	AG	AG	AA	AA	AG
	GG	AG	GG	GG	AG	AA	AA	AA
	AG	AG	AA	AG	AA	AG	AA	
178870030								
7080	rs10200136		2	AA	AA	AA	AA	AA
	AA	AA	AA	AA	AA	AA	AA	AA
	AA	AA	AA	AA	AA	AA	AA	AA
	AA	AA	AA	AA	AA	AA	AA	AA
	AA	AA	AA	AA	AA	AA	AA	
167092252								
7081	rs10200179		2	AG	GG	AG	AG	AG
	AG	AA	AG	AG	AG	GG	AG	GG
	AG	AG	GG	GG	AG	AA	AG	AG
	AA	AA	AA	AA	AG	AA	AG	AA
	GG	AG	AG	AA	AA	AA	AA	
140962137								
7082	rs10200201		2	GG	GG	GG	GG	AG
	GG	AG	AG	AG	AG	GG	GG	GG
	AG	AG	GG	GG	GG	AG	AG	AA
	AG	AA	GG	GG	AG	AG	AA	GG
	AA	AG	AA	AG	AA	AG	AG	
112928815								
7083	rs10200219		0	AG	AG	AG	AG	AG
	--	AG	AG	AG	AG	AG	GG	GG
	AG	AG	AG	AG	GG	AG	AG	GG
	AG	GG	AG	AG	GG	AG	AG	GG
	AA	AG	GG	AG	GG	GG	GG	0
7084	rs10200243		2	AG	AG	AG	GG	GG
	GG	GG	GG	GG	GG	GG	GG	GG
	GG	GG	AA	AG	AG	GG	GG	GG
	GG	AA	GG	AG	GG	GG	GG	GG
	GG	GG	GG	GG	GG	GG	GG	
182651182								
7085	rs10200259		2	GG	GG	GG	GG	GG
	GG	GG	GG	GG	GG	GG	GG	GG
	AG	AG	GG	GG	GG	AG	AG	GG
	GG	AG	GG	AG	AG	GG	AG	AG
	AG	AG	AA	AA	AA	AA	AG	
64509799								
7086	rs10200262		2	AA	AA	AA	AA	AA
	AA	AA	AA	AA	AA	AA	AA	AA

AA AA AA AA AA AA AA AA
AA AA AC AA AA AA AA AA
AA AA AA AA AA AA AA
1631215
7087 rs10200294 2 AG GG AG AG AA
AG AA AG AG GG AG AG AA
AA AA GG AG AG AG GG AA
AG AG AA GG GG AA AG AG
AG AA AG AA AG AA GG
163885183
7088 rs1020031 12 AA AA AA AA AA
AA AA AA AA AA AA AA AA
AA AA AA AA AA AG AA AA
AA AA AA AA AG AA AA AA
AA AA AA AA AA AA AA
59295453
7089 rs10200332 2 GG GG GG GG GG
GG GG GG GG GG GG GG GG
GG GG GG GG GG GG GG GG
GG GG GG GG GG GG GG GG
GG GG GG GG GG GG GG
88818091
7090 rs10200368 2 AA AA AA AG AA
AA AG AA AA AA GG GG GG
GG GG AG AG AG AA AA AA
AG AG AG AA AA AA AA AA
AG AA AA AA AA AA AG
234945784
7091 rs10200371 2 AG AG AA AG GG
AG GG AG AG AG AG AG AA
AG AG AG AA AG AG AG AG
GG AA GG AG GG AA AG AG
GG AA GG AG AA AG AA
79530813
7092 rs10200387 2 AA AG AA AG AG
AA GG GG GG GG GG GG GG
AA AA GG AG GG AG AG GG
AG GG AA AA AG GG GG AG
AG AG AG AA AG GG AG
121981210
7093 rs10200396 2 AG AG AA AA AA
AA AG AG GG GG AA AA AA
AA AA AA AA AA AA AG AG
AA AA AA AA AA AA AA AA
AA AA AG AA AA AA AA
166432227
7094 rs10200397 2 CC CC CC CC CC
CC CC CC CC CC CC CC CC
CC CC CC CC CC CC CC CC
CC CC CC CC CC CC CC CC

CC CC CC CC CC CC CC
238024408
7095 rs10200433 2 AA AG AA AG AG
GG AA AG AG AG AA AA AA
AA AA GG GG AG GG AA AG
AA AA AA AA AG AG AA AA
AA GG AG GG AG AG AA
239466567
7096 rs10200446 2 AG GG GG GG GG
GG GG GG GG GG GG GG GG
AG AG GG AG GG GG GG AG
AG AG AG AG AG GG AG GG
GG GG GG GG GG GG GG
130258186
7097 rs10200464 2 GG GG GG GG GG
GG GG GG GG GG GG GG GG
GG GG GG GG GG GG GG GG
GG GG GG GG GG GG GG GG
GG GG GG GG GG GG GG
163950227
7098 rs10200467 2 AG AG AG GG AG
AG GG GG AG GG GG AG AG
AG AG GG GG AG AA AG AG
AG GG AG AG GG AG AG AG
AG AG GG GG GG GG GG
6937593
7099 rs1020049 5 CC CC CC CC CC
CC CC CC AC CC CC CC CC
CC CC CC CC CC CC CC AC
AC AA AA AC AC CC AC AC
AA CC CC CC CC CC CC
104127642
7100 rs10200535 2 GG GG GG GG GG
AG GG AG AG AG GG GG GG
GG GG GG GG GG GG AG GG
GG GG GG GG GG GG GG GG
GG GG GG GG GG GG GG
122982362
7101 rs10200599 2 GG GG GG AG GG
AG AG AG AA AG AG AG AA
AG AG GG AG GG AG AA AA
AG GG AA AG AG AG AG GG
AG GG GG GG GG GG AG
2838270
7102 rs10200604 2 AG GG GG AG GG
AG GG AG AG AG AG GG GG
GG GG GG GG GG GG GG GG
GG GG GG GG GG GG GG GG
GG GG GG GG GG GG GG
56575020

7103 rs10200640 2 AA AG AG AG AG
AG AG GG GG GG GG GG GG
GG GG AG AG AA AG AG GG
AG GG AA AA GG GG GG GG
AG AG AG AG GG AG GG
128576820
7104 rs10200658 2 GG GG GG GG GG
GG GG GG GG GG GG GG GG
GG GG GG GG GG GG GG GG
GG GG GG GG GG GG GG GG
GG GG GG GG GG GG GG
201974276
7105 rs10200711 2 GG GG GG GG GG
GG GG GG GG GG GG GG GG
GG GG GG GG GG GG GG GG
GG GG GG GG GG GG GG GG
GG GG GG GG GG GG GG
111781425
7106 rs10200728 2 AA AA AA AA AA
AA AA AA AA AA AA AA AA
AA AA AA AA AA AA AA AA
AA AA AA AA AA AA AA AA
AA AA AA AA AA AA AA
63536223
7107 rs10200731 2 AG GG AG AA AA
AA AA AG AG AG AA AA AA
AG AG AA AG AA AA GG AA
AG AA AA AG AG AG AG AG
AG AG AG AG AG AG AA
137259124
7108 rs10200750 2 GG GG GG GG GG
GG GG GG AG GG GG AG GG
AG AG AG AG AG GG AA GG
AA AA AA AA GG GG GG GG
GG GG AG GG GG GG AG
138940776
7109 rs10200751 2 GG GG GG GG GG
GG GG GG GG GG GG GG GG
GG GG GG GG GG GG GG GG
GG GG GG GG AG GG GG AG
GG GG GG GG GG AG GG
79155348
7110 rs10200778 2 AA AA AA AC AC
AC AA AA AC AC AA AA AA
AA AA AA AA AA AA AA AA
AA AA AA AA AC AC AC AC
AA AA AA AA AA AA AA
241274172
7111 rs10200850 2 GG GG GG GG GG
GG GG GG GG GG GG AG GG

GG GG GG GG GG GG GG GG
GG GG GG GG GG AG AG AG
GG GG GG GG GG GG AG
240234901
7112 rs10200908 2 AA AA AA AA AA
AA AA AA AA AA AA AA AA
AA AA AA AA AA AA AA AA
AA AA AA AA AA AA AA AA
AA AA AA AA AA AA AA
203132385
7113 rs10200922 2 AG AA AG AA AA
AA AA AG AG AG AA AG AG
GG GG AA AA AA AG AG AA
AG AG AG AG AG AG AG GG
AA AG AA AA AG AA GG
139530239
7114 rs10200926 2 CC CC CC CC CC
CC CC CC CC CC CC AC AC
AC AC CC CC CC CC CC CC
CC CC CC CC CC CC CC CC
CC CC CC CC CC CC CC
8834580
7115 rs10200961 2 AA AA AA AG GG
AG AG AA AA AA AA AA AA
AA AA AG AG AG AA AA AA
AA AA AA AA AA AA AA AA
AA AG GG GG AA AA AA
168710172
7116 rs10200965 2 GG GG GG GG GG
GG GG GG GG GG GG GG GG
AG AG GG GG GG GG GG GG
GG GG GG GG GG GG GG GG
GG GG GG GG GG GG GG
62281648
7117 rs1020102 3 AG GG AG AG AG
GG AG GG GG GG GG GG GG
GG GG GG GG GG GG GG GG
AG AG AG AG AG GG GG AG
GG GG GG GG GG GG GG
7805209
7118 rs10201079 2 GG AG AG GG GG
GG GG AG AG AG GG AG GG
GG GG GG GG GG AG AG AG
AG AA AG AG GG GG GG GG
AA GG GG GG GG GG GG
99192649
7119 rs10201099 2 GG GG GG AG AG
GG AA GG GG GG GG GG GG
GG GG GG GG GG AG GG GG
GG GG GG GG GG AG AG GG

	GG	GG	AG	AG	AG	GG	AG
2257857							
7120	rs10201136	2	GG	AG	GG	AA	AG
	AG	AG	GG	GG	GG	AG	GG	AG
	AG	AG	GG	GG	GG	GG	GG	GG
	AG	GG	AG	AG	GG	GG	GG	GG
	GG	AG	AG	AG	GG	GG	AG	
101286023								
7121	rs10201181	2	AG	GG	AG	AA	AA	
	AA	AA	AG	AG	AG	AA	AA	AA
	AG	AG	AA	AA	AA	AG	AA	AG
	AG	AG	AG	AG	AG	AA	AG	AA
	AG	GG	AG	AG	AG	AA	AG	
158717966								
7122	rs10201223	2	AA	AA	AA	AC	AA	
	AC	AA	CC	CC	CC	CC	AC	AC
	AC	AC	AA	AA	AA	AA	AA	AA
	AC	CC	AA	AC	AC	AA	AA	AA
	AC	AA	AC	AC	AA	AA	AC	
12272871								
7123	rs10201243	2	AG	GG	AG	AG	AG	
	GG	AG	AG	GG	GG	GG	GG	AG
	AA	AA	AG	GG	GG	GG	GG	AG
	GG	GG	GG	GG	GG	GG	GG	GG
	GG	AG	AG	AG	GG	GG	GG	
168402224								
7124	rs1020126	2	CC	AC	CC	CC	CC	
	CC	AC	AC	CC	CC	CC	CC	CC
	CC	CC	AC	AA	AA	CC	CC	CC
	AC	AC	CC	AC	CC	CC	CC	CC
	CC	CC	AC	AC	AC	AC	CC	
212703601								
7125	rs10201406	2	AA	AA	AA	AA	AA	
	AA	AA	AA	AA	AA	AA	AA	AA
	AA	AA	AA	AA	AA	AA	AA	AA
	AA	AA	AA	AA	AA	AA	AA	AA
	AA	AA	AG	AG	AA	AA	AA	
235668847								
7126	rs10201422	2	AA	AA	AG	AG	AG	
	AG	AG	AA	AG	AA	AA	AG	AA
	AG	AG	GG	GG	GG	AG	AG	AG
	AG	AG	AG	AG	AA	GG	AG	AG
	AG	AG	AA	AG	AG	GG	AA	
86999171								
7127	rs10201439	2	GG	GG	GG	AA	GG	
	AG	AG	GG	GG	GG	GG	GG	GG
	AG	GG	AG	GG	AG	AG	AG	GG
	GG	GG	GG	GG	GG	GG	GG	GG
	AG	AG	AG	AG	GG	GG	GG	
16381942								

7128 rs1020145 15 CC CC CC CC CC
CC CC AC AC AC AC AC AC
AC AC AC CC CC AA AA AC
AC CC CC CC CC CC CC CC
AA AC CC AC AC AC CC
90000871
7129 rs10201452 2 AA AA AA AA AA
AA AA AA AA AA AA AA AA
AA AA AA AA AA AA AA AA
AA AA AA AA AA AA AA AA
AA AA AA AA AA AA AA
12965088
7130 rs10201457 2 AA AG GG AG GG
GG AG AA AA AA AG AA AG
AA AA GG AG AG AG AG GG
AG GG AG AG AG AG AG AG
AG GG AG AG AA AA AA
86457739
7131 rs1020147 7 GG GG GG GG AG
AG GG GG GG GG AA AG AG
AG AG GG GG GG GG GG AG
AA GG AG AG AG GG AG GG
GG GG GG GG GG GG GG
41374447
7132 rs10201517 2 AG AA AA AG AA
AG AA AA AA AA AG AA AA
AG AG AA AA AA AG AG AG
AA AA AA AA AA AA AA AA
AA GG AA AG AG AG AA
66953286
7133 rs10201523 2 AA AA AC AC AC
AC AA AA AA AA AA AA AA
AC AC AA AA AA AC AA AA
AC AC AC AC AA AA AA AA
AC AA AC AC AA AA AA
126602160
7134 rs10201535 2 AG AG GG AG AG
AG GG AG AA AA AG GG AG
AG AA AG AG AG AG AG AG
AG AG AG AG GG AG GG GG
AG AG GG AG GG GG GG
242517966
7135 rs10201541 2 AG AA AG AG AA
AG AA AG AG AA AA AA AA
AA AA AG AG AA AA AA AA
AA AA AA AA AA AA AA AA
AA AA AA AA AA AA AA
30157544
7136 rs10201568 2 AA AA AG AA GG
AG AG AG AG AG AG AG GG

AG AG AA AA AG AA AA AA
AA AG AG AA AA AA AA AA
AA AA AA AA GG AG AG
46125865
7137 rs10201616 2 AG GG AG AA AA
AA AA AA AA AA AG AA AG
AA AA AA AA AA AA AA AA
AA AA AA AA AG AG AA GG
AG AG AA AA AG GG AA
75675860
7138 rs10201627 2 CC CC CC CC CC
CC CC CC CC CC CC CC CC
CC CC CC CC CC CC CC CC
CC CC CC CC CC CC CC CC
CC CC CC CC AC CC AC
231774377
7139 rs10201680 2 GG GG AG GG GG
GG GG GG GG GG AG AG AG
AG AG GG GG GG AG AG GG
GG GG GG GG GG GG GG GG
GG AG GG GG AG GG AG
121395161
7140 rs10201685
2 -- -- -- -- -- -- -- --
-- -- -- -- -- -- -- --
-- -- -- -- -- -- -- --
-- -- -- -- -- -- -- --
-- -- -- -- 60067498
7141 rs10201692 2 GG GG GG GG GG
GG GG GG GG GG GG GG GG
GG GG GG GG GG GG GG GG
GG GG GG GG GG GG GG GG
AG GG GG GG GG GG GG
104652419
7142 rs10201710 2 AA AA AG AA AA
AA AA AA AA AA AG GG GG
GG GG AG AG AG AA AA AA
AG AG AG AG AA AA AA AA
AG AA AG AG AG AG AA
54372931
7143 rs10201732 2 CC CC CC CC CC
CC AC CC CC CC CC CC CC
CC CC CC CC CC CC CC CC
CC CC CC CC CC CC CC CC
CC AC CC CC AC AC AC
12262421
7144 rs10201738 2 AA AG AG AG AG
AG AA GG GG GG AG AG AA
AA AA GG GG GG AG AG AG
AA AA AG AG GG AG AG GG

AG GG GG GG AG GG AG
106386294
7145 rs10201778 2 CC CC CC CC CC
CC CC AC AC CC CC CC CC
CC CC CC CC CC CC CC CC
CC CC CC CC CC CC CC CC
CC CC CC CC CC CC CC
112868161
7146 rs10201844 2 AA AA AG AA AG
AA AG AA AA AA AG AA AA
AA AA AG AA AA AG AG AG
AG AG AG AG AA AG AG AA
AA AG AA AG AA AA AG
64041944
7147 rs10201854 2 AC CC CC AC AC
AC AC AC AC AA AC CC CC
AC AC AC CC AC AC CC CC
CC CC AC AC CC CC CC CC
AC AC AA AC AC CC AA
115866745
7148 rs10201889 2 AA AA AA AA AG
AG AA AA AA AA AA AA AA
AA AA AG AA AG AA AA AA
AA AA AA AA AG AA AG AA
AA AA AA AA AG AA AG
19988877
7149 rs10201919 2 AA AA AA AA AA
AA AA AA AA AA AA AA AA
AA AA AA AA AA AA AA AA
AA AA AA AA AA AA AA AA
AA AA AA AA AA AA AA
88402840
7150 rs10202013 2 GG GG GG AG GG
GG AG AG GG AG AG AG AG
AG AG GG GG GG AG AG AG
GG GG AG GG GG AG AG AG
AG GG AG GG GG GG AG
16536980
7151 rs10202061 2 AG GG AG GG GG
GG AG AG AG AG GG GG AG
AG AG GG GG GG AG AG GG
GG GG GG GG GG GG GG GG
AG GG GG GG AG AG GG
156753946
7152 rs1020207 10 AG AA AA AA AA
AA AA AG AG AG AA AA AA
AA AA AA AG AG AA AA AA
AG AG AA AG AG AG GG AA
AA AA AG AA AA AA AA
56018508

7153 rs10202071 2 AA AG AA AG GG
AG AG AA AG AA AA AG AG
AG AG AA AA AA AG GG AG
AG AA AG AG GG AA AG AG
AG AG GG AG AG AA GG
6692014
7154 rs10202096 2 AG AG AG GG GG
GG GG GG AG GG AG AA AA
AG AA GG GG GG AG AG AG
AA AG AA AA AG AG AG AG
AG GG GG GG AG AG GG
28596317
7155 rs10202112 2 AG AG AG AG GG
AG AG AG AG AG AG AG AG
AG AA GG GG GG AG GG GG
AA AA AG GG AG AA AG AG
GG AG AG AG AA AG AG
199627907
7156 rs10202141 2 AA AA AC AC AC
AC CC AA AA AA AC AA AC
AC AC AA AA AA AC AA AC
AA AA AA AA AA AC AC AA
AA AA AA AA AC AC AA
110030964
7157 rs10202203 2 AA AA AA AA AA
AA AA AA AA AA AA AA AA
AA AA AA AG AA AA AA AA
AG AG AG AG AA AG AA AA
AG AA AA AA AG AG AG
240484995
7158 rs10202227 2 AA AA AA AA AA
AA AA AA AA AA AA AA AA
AA AA AA AA AA AA AA AA
AA AA AA AA AA AA AA AA
AA AA AA AA AA AA AA
57002106
7159 rs1020227 8 AA AA AA AA AA
AA AA AA AA AA AA AA AA
AA AA AA AA AA AA AA AA
AA AA AA AA AA AA AA AA
AA AA AA AA AA AA AA
14510380
7160 rs10202299 2 AA AG AG AG AA
AA AG AG AG AG GG AG GG
AA AA AA AA AA GG AG AG
AG AG AG AG AG AG GG AA
AG AA GG AG AG AG AG
41506889
7161 rs10202342 2 AG AA AG AG AG
AG AG AA AG AA GG GG GG

GG GG GG AG GG AG AA GG
AG AA AA AG GG GG GG GG
AA AG AG AG GG GG AG
141796256
7162 rs10202351 2 GG GG GG GG GG
GG GG GG GG GG AG AA AG
GG GG GG GG GG GG GG GG
GG AG AG AG AG GG GG AG
GG AG GG GG GG GG GG
49116885
7163 rs10202379 2 AA AG AG AA AA
AA AA AG AG AG AG AA AA
AG AG AG AG AA AG AG AA
AG AG AG AG AG AG GG AA
GG GG GG GG AG AG AG
100596865
7164 rs10202391 2 AA AA AA AA AA
AA AA AA AA AA AA AA AA
AA AA AA AA AA AA AA AA
AA AA AA AA AG AA AA AG
AA AA AG AG AA AA AA
155399506
7165 rs10202432 2 AG AA AA AG AA
AA AG AG GG AG AG AG AG
AG AG AG AG AA AA AA AA
GG AG GG GG AA AG AG AA
AG AG AG GG AG AA GG
88403318
7166 rs1020248 8 AG AG AG GG GG
AG GG AG AG AG GG GG GG
AG AG GG GG GG GG AG GG
AG AG AG AG GG AG AG AG
AG GG GG GG AG AA GG
13007470
7167 rs10202501 2 AA AA AA AA AA
AA AA AA AA AA AC AC AA
AA AA AC AA AA AA AC AA
AA AA AA AA AA AA AA AA
AA AA AA AA AC AC AA
231285570
7168 rs10202544 2 AA AA AG AA AG
AA AG AA AA AA AA AA AA
AA AA AG AG AA AA AA AG
AG AG AA AA AG AG AA GG
AA AA AA AA AA AA AA
105887549
7169 rs10202641 2 GG GG GG GG GG
GG GG GG GG GG GG GG GG
GG GG GG GG GG AG AG AG
GG GG GG GG GG GG GG GG

GG GG AG AG GG GG GG
236238043
7170 rs10202644 2 AG AG AA GG AG
AG AG GG GG GG AG GG AG
GG GG GG GG GG GG GG GG
GG GG GG GG AG AG AG AG
GG GG AG AG GG GG GG
97611396
7171 rs1020265 4 AG AG AA AA AA
AA AA GG AG AG GG GG AG
AA AG AG AG GG AG GG AA
AA AA AA AA GG AG GG AG
AA AA GG AG AG AA AG
4327912
7172 rs10202656 2 AA AA AA AA AA
AA AA AA AA AA AA AA AA
AA AA AA AA AA AA AA AA
AA AA AA AA AA AA AA AA
AA AA AA AA AA AA AA
147123366
7173 rs1020267 4 GG GG GG AG AG
AG AG AG AG AG GG AG AG
AG AG GG GG GG AG GG AA
GG GG GG GG AG GG GG GG
GG AG AG AG GG GG GG
142821772
7174 rs10202670 2 AA AA AA AG AG
AA AG AA AA AA AA AA AA
AA AA AG AG AG AA AA AA
AA AA AA AA AA AA AA AA
AA AG AA AA AG AG AA
160310246
7175 rs10202684 2 AG AA AA AA AA
AA AA AA AA AA AA AA AA
AA AA AA AA AA AA AA AA
AA AA AA AA AG AA AG AA
AA AA AA AA AA AA AA
125543111
7176 rs10202702 2 AA AC AC CC CC
AC CC CC AC AA AC AC AC
CC CC AC CC AC AC CC AC
AC AC AA CC AC CC CC AC
AC AC CC CC AC AC AA
157583475
7177 rs10202768 2 AA AA AA AA AA
AA AA AA AA AA AA AA AA
AA AA AA AA AA AA AA AA
AA AA AA AA AA AA AA AA
AA AA AA AA AA AA AA
237089290

7178 rs10202871 2 AA AA AA AA AA
AA AA AG AG AA AA AA AA
AA AA AA AA AA AA AA AA
AA AA AA AA AA AA AA AA
AA AA AA AA AA AA AA
112898848
7179 rs10202912 2 AG AG GG AG AG
AA GG AG AA AA AG AG AG
GG GG AA AA AA AA AA AG
AG GG AG GG AG AG GG AG
AG AG GG AG AA AA AG
66786405
7180 rs10202915 2 GG GG GG GG GG
GG GG GG GG GG GG AG AG
AG AG GG GG GG GG GG GG
GG GG GG GG GG GG GG GG
GG GG GG GG GG GG GG
4747638
7181 rs10202944 2 AA AA AA AA AA
AA AA AA AA AA AA AA AA
AA AA AA AA AA AA AA AA
AA AA AA AA AA AA AA AA
AA AA AA AA AA AA AA
72257858
7182 rs10202955 2 GG GG GG GG GG
GG GG AA GG AG GG GG GG
GG GG GG AG AG AG GG AG
AG AG AG AG AA GG AG AG
GG AA AG AA GG GG AG
186309738
7183 rs10202956 2 AA AA AA AA AA
AA AA AA AA AA AA AA AA
AA AA AA AA AA AA AA AA
AA AA AA AA AA AA AA AA
AA AA AA AA AA AA AA
168680848
7184 rs10202963 2 CC CC CC AC CC
AC CC AC CC CC AC AA AC
AC AC CC AC AC AC AC AC
CC CC AC AC CC CC CC CC
AA AC AA AC AC AC CC
202241907
7185 rs10203017 2 AG GG AG GG GG
GG GG AG AG AG AG AG AA
AG AG GG GG GG AA AA AA
AG AG AG AG AG AG AA GG
AA GG AG AG AA AG AG
41123765
7186 rs10203023 2 AA AA AA AA AA
AA AA AA AA AA AA AA AA

```
        AA      AA      AA      AA      AA      AA      AA      AA
        AA      AA      AA      AA      AA      AA      AA      AA
        AA      AA      AA      AA      AA      AA      AA
203661193
7187    rs10203034      2       AA      AA      AA      AG      AA
        AA      AG      AG      AG      AG      AG      AG      AA
        AA      AA      GG      AG      AG      AG      AG      AA
        AG      AG      AG      AG      AG      AA      AA      AG
        AA      AG      AG      AG      AA      AA      AA
36062875
7188    rs10203047      2       GG      GG      GG      GG      GG
        GG      GG      GG      GG      GG      GG      GG      GG
        GG      GG      GG      GG      GG      GG      GG      GG
        GG      GG      GG      GG      GG      GG      GG      GG
        GG      GG      GG      GG      GG      GG      GG
99095950
7189    rs10203049      2       AA      AA      AA      AA      AA
        AA      AA      --      AA      AC      AC      AC      AC
        AA      AA      AA      AA      AA      AA      AA      AC
        AA      AA      AA      AA      AA      AA      AA      AA
        AA      AA      AA      AA      AA      AA      AA
142969568
7190    rs10203090      2       AG      AA      AG      AG      AG
        AG      AG      GG      GG      AG      AA      AA      AA
        AG      AA      AG      GG      AA      AG      AG      GG
        AG      GG      AA      AG      AG      GG      GG      AG
        AG      GG      AA      AG      AG      AG      AA
30275291
7191    rs10203100      2       AA      AC      AA      AA      AA
        AA      AA      AA      AA      AA      AA      AA      AA
        AA      AA      AC      AC      AC      AA      AC      AA
        AA      AA      AA      AA      AA      AA      AA      AA
        AC      AC      AA      AA      AA      AA      AA
211993656
7192    rs1020312       8       GG      AG      GG      AG      AG
        AA      GG      GG      GG      GG      AG      AG      GG
        GG      GG      GG      GG      GG      GG      GG      GG
        GG      GG      GG      GG      AG      AG      AA      AA
        GG      GG      AG      GG      AG      AG      AG
72830625
7193    rs10203199      2       GG      AG      AG      GG      AG
        AG      GG      AG      AG      AG      AA      AA      AA
        AG      AG      AG      AG      AA      GG      AG      GG
        AG      AG      AG      AA      AA      AA      AA      AA
        AA      AG      AG      AA      GG      GG      GG
173655524
7194    rs10203212      2       AA      AG      AG      GG      AG
        AG      GG      GG      GG      GG      AG      AG      AG
        AA      AA      AG      AG      GG      AA      AG      AA
        AG      AA      AG      AA      AA      AA      AA      AA
```

AA AA AA AA AA AA AA
170917648
7195 rs10203213 2 AG AA AG GG AG
AG AG AA AA AA AG AG AG
AA AA AG GG AG GG GG GG
AG AA AG AG AA AA AA AA
AA GG AG GG AA AA AA
88758598
7196 rs10203253 2 GG GG GG GG GG
GG GG GG GG GG GG GG GG
GG GG GG GG GG GG AG GG
AG GG AG GG AG GG AG AG
GG GG GG GG GG GG GG
180948331
7197 rs10203263 2 CC CC CC AC CC
AC CC CC CC CC CC CC CC
CC CC AC AC AC CC CC AC
CC CC CC CC CC AC CC AC
AC AC CC CC CC CC CC
63454401
7198 rs10203272 2 CC CC CC AA AC
AA AC AC AC AC CC CC CC
CC CC AC AC CC CC CC CC
CC AC CC CC CC CC CC CC
CC CC CC CC CC CC CC
86033879
7199 rs10203280 2 AG AA AA AG AA
AG AA AA AA AA AA AA AA
AA AA AA AA AA AA AA AA
AA AA AA AA AA AA AA AA
AA AA AA AA AA AA AA
56552703
7200 rs10203313 2 GG AG GG AG AA
AG AA AG AA AG GG AG AG
GG AG AG AG AA GG GG AG
AG AG AA AG GG AG AG GG
GG AA AG AA AA AA AA
28714750
7201 rs10203375 2 AC AA AC AC AC
AA CC AA AA AA AA AA AC
AC AC AC AC AA CC AC AC
AC AA AC AC AA AA AA AA
AA AA AC AA AC AA CC
2763249
7202 rs10203385 2 AA AA AA AA AA
AA AA AA AA AA AA AA AA
AA AA AA AA AA AA AA AA
AA AA AA AA AA AA AA AA
AA AA AA AA AA AA AA
100621968

7203 rs1020339 10 AA AA AA AG AA
AG AA AG AG AG AG AG AA
AA AA GG AG AG AA AA AG
AG AG AG AG GG AG GG GG
AG AG AG AG AG GG AG
17399616
7204 rs10203422 2 AA AA AA AA AA
AA AA AA AA AA AA AA AA
AA AA AA AA AA AA AA AA
AA AA AA AA AA AA AA AA
AA AA AA AA AA AA AA
57953650
7205 rs10203437 2 AA AG AG AG AG
AG AA AG AG AG AA AA AA
AG AG AG GG AA GG GG AG
AA AA AA AA AA AA AA AA
AA AA AG AA AG AG AG
98892107
7206 rs10203445 2 AC AC AC AA AA
AA AA AA AA AA AA AA AA
AA AA AA AA AA AA AA AA
AA AA AA AA AC AA AC AC
AA AA AA AA AC AC AA
229833153
7207 rs1020351 X GG AG AA AA AA
AA AA AG AG GG AA AA AG
AA AA AG AG AG AA AA AA
AA GG AA AG GG AA AG GG
AG AG GG GG AG AA GG
25765001
7208 rs10203511 2 AG AA AA AA AA
AA AA GG AG AG AG AA AA
AA AA AA AA AA AA AA AA
GG AG GG GG AG AA AG AA
AG AG AA AG AG GG AA
50540027
7209 rs10203538 2 AA AA AA AA AA
AA AA AA AA AG AA AA AA
AA AA AA AA AA AA AA AA
AA AA AA AA AA AA AA AA
AA AA AA AA AA AA AA
235850039
7210 rs1020360 5 GG GG AG GG GG
GG GG GG GG GG GG GG GG
GG GG AG GG GG GG GG GG
GG GG GG GG GG GG GG GG
GG GG GG GG GG GG GG
40258578
7211 rs1020361 5 AA AA AA AA AA
AA AA AA AA AA AA AA AA

	AA	AA	AA	AA	AA	AA	AA	AA
	AA	AA	AA	AA	AA	AA	AA	AA
	AA	AA	AA	AA	AA	AA	AA	
40369682								
7212	rs10203664		2	AA	AA	AA	AG	GG
	AG	GG	AA	AA	AA	AA	AA	AA
	AA	AA	AG	AG	GG	AA	AA	AA
	AG	AG	AA	AG	AG	GG	GG	GG
	GG	AG	GG	AG	GG	AG	GG	
10752377								
7213	rs10203676		2	AA	AA	AA	AA	AA
	AG	AA	AA	AA	AG	AA	AA	AA
	AA	AA	AG	GG	AG	AA	GG	AG
	AA	AG	AA	AG	AG	AG	AA	AG
	GG	AG	AA	AA	AA	AG	AG	
155919140								
7214	rs10203697		2	GG	GG	GG	GG	GG
	GG	GG	GG	GG	GG	GG	GG	GG
	GG	GG	GG	AG	GG	GG	GG	GG
	GG	GG	GG	GG	GG	GG	GG	GG
	GG	GG	AG	GG	AG	AG	AG	
235960254								
7215	rs10203763		2	GG	AG	GG	AG	AG
	AG	GG	GG	AG	GG	AG	GG	GG
	GG	GG	AG	AG	AG	AG	GG	AG
	AG	AG	AG	AG	AG	AA	AA	AG
	GG	GG	AG	GG	AG	GG	AG	
146011003								
7216	rs10203798		2	GG	GG	GG	GG	GG
	GG	GG	GG	GG	GG	GG	GG	GG
	GG	GG	GG	GG	GG	GG	GG	GG
	GG	GG	GG	GG	GG	GG	GG	GG
	GG	GG	GG	GG	GG	GG	GG	
147187683								
7217	rs10203812		2	AG	AG	GG	AG	GG
	AG	AG	AA	AA	AA	AG	GG	GG
	AG	AG	AG	GG	AG	AG	AG	GG
	GG	GG	GG	GG	AA	AA	AA	AA
	AG	GG	AG	AG	AG	AA	GG	
76811778								
7218	rs10203819		2	AC	CC	AC	CC	AC
	AC	AC	AC	CC	CC	AC	CC	AC
	AC	CC	CC	AC	AC	CC	CC	CC
	AC	AC	AC	AA	CC	CC	CC	CC
	CC	AC	AC	AC	CC	CC	CC	
197576167								
7219	rs1020382		19	AA	AG	AG	AG	AG
	GG	AG	AG	GG	AG	AA	AA	AA
	AA	AA	AA	AA	AA	AA	AA	AG
	AA	AG	AG	AG	AA	AG	AG	AG

AG AG AG AG AA AG AG
267039
7220 rs10203825 2 GG GG GG GG GG
GG GG GG GG GG GG GG GG
GG GG GG GG GG GG GG GG
GG GG GG GG GG GG GG GG
GG GG GG GG GG GG GG
40116788
7221 rs10203831 2 AG AG AG AG AG
GG AA AG GG GG GG GG GG
GG GG GG GG GG GG AG GG
AG AG AG AG AG GG AG AG
AG AG GG GG AG AG GG
237841816
7222 rs10203851 2 CC CC CC CC CC
CC AC CC CC CC AA AC AC
CC CC CC CC CC CC CC CC
CC CC CC CC CC CC CC CC
CC CC CC CC CC CC CC
123407739
7223 rs10203870 2 AC CC CC CC CC
CC AC AC AC AC AA CC AC
CC CC CC AC CC AC AC AC
CC CC CC CC AC AC AC AC
AC CC CC CC CC CC AC
72340493
7224 rs10203969 2 CC AC CC AC CC
AC AC CC AC AC AA AC AA
AC AA CC AC AC CC CC CC
CC CC CC CC AA AC AC AA
AC CC CC CC AC CC AC
217485550
7225 rs10204 16 GG GG GG GG GG GG
GG GG GG GG GG GG GG GG
GG AG AG AG GG GG AG GG
GG GG GG AG GG GG AG GG
GG GG GG GG GG GG 29826538
7226 rs10204058 2 GG GG GG GG GG
GG GG GG GG GG GG GG GG
GG GG GG GG GG GG GG GG
GG GG GG GG GG GG GG GG
GG GG GG GG GG GG GG
164032029
7227 rs10204095 2 AG AA AA AA AA
AA AA AA AA AA AA AA AA
AG AG AG AA AA AA AA AG
AA AA AA AA AA AA AA AA
AA AA AA AA AA AA AA
57799040
7228 rs10204096 2 GG GG AG GG GG

	GG	GG	GG	AG	AG	AA	AA	AA
	GG	GG	GG	GG	AG	GG	GG	AG
	AG	GG	GG	GG	GG	AG	GG	AG
	AG	AA	AG	AA	GG	GG	AG	
46883739								
7229	rs10204114		2	AA	AA	AA	AA	AA
	AA	AA	AA	AA	AA	AA	AA	AA
	AA	AA	AA	AA	AA	AG	AG	AA
	AA	AA	AA	AA	AG	AG	AG	AG
	AA	AA	AA	AA	AG	AG	AA	
35205952								
7230	rs10204120		2	AC	CC	AC	CC	CC
	CC	CC	CC	CC	CC	AC	CC	AC
	AC	AC	CC	CC	CC	AC	AC	AC
	CC	CC	CC	CC	CC	CC	CC	CC
	CC	AC	CC	AC	AC	CC	AC	
76121914								
7231	rs10204148		2	AG	AA	AA	GG	GG
	AG	GG	AG	AG	AA	AG	AG	GG
	AG	GG	AA	AG	AG	AA	AA	AA
	AG	AG	AG	AG	AA	AA	AA	AA
	AA	GG	AA	AG	AG	AG	AG	
119435628								
7232	rs10204202		2	GG	GG	GG	GG	GG
	GG	GG	GG	GG	GG	GG	GG	AG
	GG	GG	AG	GG	AG	AG	AG	AG
	AG	GG	GG	GG	GG	AG	AG	GG
	GG	GG	GG	GG	GG	GG	GG	
47514011								
7233	rs10204219		2	AA	AA	AA	AA	AA
	AA	AA	AA	AA	AA	AA	AA	AA
	AA	AA	AA	AA	AA	AA	AA	AA
	AA	AA	AA	AA	AA	AA	AA	AA
	AA	AA	AA	AA	AA	AA	AA	
6859220								
7234	rs1020426		2	GG	GG	GG	GG	GG
	GG	AG	AA	AG	AG	GG	GG	GG
	GG	GG	GG	GG	GG	GG	GG	GG
	AG	GG	GG	AG	GG	GG	GG	GG
	AG	GG	AG	AG	GG	GG	GG	
143272903								
7235	rs10204265		2	AG	AG	AG	GG	GG
	GG	AG	AG	GG	AG	GG	GG	GG
	GG	GG	GG	AG	AG	GG	GG	GG
	GG	AG	GG	GG	GG	GG	GG	GG
	AA	GG	GG	GG	GG	GG	GG	
176603842								
7236	rs10204281		2	GG	GG	GG	GG	GG
	GG	GG	GG	GG	GG	GG	GG	GG
	AG	AG	GG	GG	AG	GG	GG	GG

GG GG GG GG GG AG AG GG
GG AG GG GG AG AA GG
45172258
7237 rs10204328 2 AA AA AA GG AG
AG GG AA AA AA GG GG GG
GG GG AA AG AG AA AA AG
AG AG AG AG GG AA AG AG
AA AA AA AA AG AG AA
185969194
7238 rs10204385 2 GG GG GG GG GG
GG GG AG GG GG GG GG GG
GG GG GG GG GG GG GG GG
GG GG GG GG GG GG GG GG
GG GG GG GG AA AG AG
154190197
7239 rs10204418 2 AA AA AA AG GG
AG AG AG AG AG AA AA AA
AA AA AA AG AA AA AA AG
AA AA AA AA AG AA AG AG
AA AG AA AG AG AG AG
6352799
7240 rs10204437 2 AG AG GG AG GG
AG GG AG AG AG GG AA AG
AG AG GG GG GG AA AA AG
GG GG GG GG GG GG GG GG
AG AG GG GG GG GG GG
71059763
7241 rs10204481 2 AA AG AA GG AG
GG AG GG AA AG GG GG GG
GG GG GG AG AG GG AG GG
AG AG AG AG AG AG AG GG
AG AG GG AG AA AA AA
232500132
7242 rs10204566 2 AG AG AA AA AG
AA AG GG GG GG AG AA AA
AG AG AA AA AA AA AA AG
AA AG AA AG AG GG AG AG
AG AG AA AG AA AA AA
58999505
7243 rs10204574 2 AC AC AA CC AC
CC AC AC AC AA AA AA AA
AC AC AC CC AC AA AC AC
AA AA AA AA AA AA AA AA
CC AA AC AC CC AC CC
31445272
7244 rs10204611 2 AG AG GG GG AG
GG AG GG GG GG GG GG GG
GG GG GG AG AG GG GG GG
GG GG GG GG AG AG AA GG
GG AG AG GG GG GG AG

51354717
7245 rs10204694 2 AA AA AA AA AA
AA AA AA AA AA AA AA AA
AA AA AA AA AA AA AA AA
AA AA AA AA AA AA AA AA
AA AA AA AA AA AA AA
111447446
7246 rs10204710
2 -- -- -- -- -- -- -- --
-- -- -- -- -- -- -- --
-- -- -- -- -- -- -- --
-- -- -- -- -- -- -- --
-- -- -- -- 205646988
7247 rs10204714 2 AG AA AG GG AG
AG GG AA AA AA AA AG AA
AG AG AG AG AA GG GG GG
AG AG AG AG AG AG AG AG
AG GG AG GG AA AA AG
44151353
7248 rs10204763 2 AA AA AA AA CC
AC AC AC AC AA AA AC AA
AC AC AA AA AA CC AC AC
AC AC AA AC AA AA AA AA
AC AA AC AA AA AA AA
34059750
7249 rs10204782 2 AA AA AA AA AG
AG AA AG AG AG AA AA AA
AA AA AA AA AA AA AA AA
AG AG AA AG AA AG AA AA
AA AA AA AA AG AG AA
17114343
7250 rs10204844 2 CC CC CC CC CC
CC CC CC CC CC CC CC CC
CC CC CC CC CC CC CC CC
CC CC CC CC CC CC CC CC
CC CC CC CC CC CC CC
111502682
7251 rs10204847 2 AG GG AG AG AG
AA AG AG AG AG AA AG AA
AA AA AA AA AA AA AA AA
AG AG AA AG AG AG AA AA
AG AG AA AG AA AG AA
9431636
7252 rs10204907 2 GG GG AG AG AA
AG AG GG GG GG GG GG GG
GG GG GG GG GG GG GG GG
GG GG GG GG GG GG GG GG
GG AG GG GG GG GG GG
80151340
7253 rs10204926 2 AC AC AC AA AA

AA AA AA AA AA AC AC AC
AC AC AA AA AA AA AA AC
AA AA AA AA AA AC AA AC
AA AA AA AA AA AA AA
129395115
7254 rs10204935 2 GG GG GG GG GG
GG GG GG GG GG GG GG GG
GG GG GG GG AG GG AG AG
GG AG GG AG GG AG AG AG
GG GG GG GG GG GG AG
166390873
7255 rs10204940 2 AA AA AA AA AA
AA AA AA AA AA AA AA AA
AA AA AG AA AA AG AG AG
AA AA AA AA AA AA AA AA
AA AA AA AA AA AA AA
14221803
7256 rs10204944 2 AA AG AA AG AA
AG AA AA AA AA AA AA AA
AA AA AA AG AG AA AA AA
AA AA AG AG AA AA AA AA
AA AA AG AA AG AG AA
184941520
7257 rs10204994 2 GG GG GG GG GG
GG GG AG AG AG GG GG AG
AG AG AA AG AA AG AG GG
AG AG AG AG AG GG AG GG
AG GG AG GG AG GG AA
35443726
7258 rs10204998 2 GG AG GG GG GG
GG GG AG GG GG GG AG GG
GG GG AG AG AG AG AG AA
AG AG AG AA AG GG AG AG
GG AG AG AA AA AG AA
203042078
7259 rs10205 17 GG AG GG AG AG GG
AG AG GG GG GG GG GG GG
GG AG GG AG GG GG AG GG
GG AG AG GG AA AG AG GG
AA AG AG GG GG GG 27207120
7260 rs10205008 2 AA AA AA AG GG
GG AG AA AG AA AG AG AG
AG AA AA AG AA AG AG AG
AA AA AG AA AA AG AG AA
AG AG AG AA AG AG AG
28765309
7261 rs10205024 2 AG AG AG GG AA
AG AG AA AG AG GG GG AG
GG GG GG GG GG AG AG AA
AA AA AA AA GG GG GG GG

	AA	AA	GG	AG	AG	AG	AA
46385339							
7262	rs10205049	2	GG	AG	AG	AG	AG
	AG	AA	GG	GG	GG	GG	GG	AG
	AG	AG	AG	AA	AG	AG	GG	GG
	GG	AG	GG	GG	AA	GG	AG	AG
	AG	AA	GG	AG	AG	AA	GG
119899933							
7263	rs10205183	2	AA	AA	AA	AG	AA
	AA	AG	AG	AG	AA	AA	AA	AA
	AA	AA	AA	AA	AA	AG	AA	AG
	AG	AG	AG	AG	AA	AA	AA	AA
	AG	AG	AG	AG	AA	AA	AA
235978207							
7264	rs1020522	2	AA	AA	AA	GG	GG
	AG	AG	AA	AA	AA	AA	AA	AA
	AA	AA	GG	GG	AG	AA	AA	AA
	AG	AG	AG	AG	AA	AG	AA	AA
	AA	AA	AA	AA	AA	AA	AG
147819724							
7265	rs10205230	2	AA	AA	AA	AA	AA
	AA	AA	AA	AA	AA	AA	AA	AA
	AA	AA	AA	AA	AA	AA	AA	AA
	AA	AA	AA	AA	AA	AA	AA	AA
	AA	AA	AA	AA	AA	AA	AA
102484747							
7266	rs10205233	2	GG	GG	GG	AG	GG
	AG	AG	AG	AG	AG	GG	GG	GG
	GG	GG	GG	GG	AG	GG	GG	GG
	GG	GG	GG	GG	GG	GG	GG	GG
	GG	GG	GG	GG	GG	GG	GG
227614005							
7267	rs10205234	2	AC	CC	AC	CC	AC
	AC	CC	CC	CC	CC	AC	CC	AC
	AC	CC	CC	AC	AC	CC	CC	CC
	CC	CC	AC	AC	CC	CC	CC	CC
	CC	CC	AC	AC	CC	CC	CC
197567620							
7268	rs10205240	2	AA	AA	AA	AA	AA
	AA	AA	AA	AA	AA	AA	AA	AA
	AA	AA	AA	AA	AA	AA	AA	AA
	AA	AA	AA	AA	AA	AA	AA	AA
	AA	AA	AA	AA	AA	AA	AA
109429262							
7269	rs10205350	2	CC	CC	CC	CC	CC
	CC	CC	AC	AC	AA	AC	AC	AC
	CC	CC	CC	CC	CC	CC	CC	CC
	AA	AC	AC	AA	CC	CC	CC	CC
	AC	AA	CC	AC	CC	CC	CC
31146497							

7270 rs10205387 2 AA AC CC AC AC
AC AC AC AC AC AC CC CC
CC CC AC CC AC CC CC AC
AA AC AA AC CC AC AC CC
CC CC CC CC CC AC CC
59313101
7271 rs10205399 2 AC AA AC AA AC
AA AC AC AC AC CC AC CC
AC AC AA AA AC CC CC CC
AC AC AC AC CC AA AC AC
CC AC CC CC CC AC AC
41197708
7272 rs10205402 2 GG AG AA AG GG
AG GG GG GG GG AA AG AA
GG GG AG AG AG GG AG GG
AG AG AG AG AG AG AG AG
AG GG AG GG AG GG AG
40710953
7273 rs10205406 2 CC CC CC CC CC
CC CC CC CC CC CC CC CC
CC CC AC CC CC CC CC AC
CC CC CC CC AC CC CC CC
AC CC CC CC CC CC CC
13468004
7274 rs10205428 2 AA AA AA AA AA
AA AA AA AA AA AA AA AA
AA AA AA AA AA AA AA AA
AA AA AA AA AA AA AA AA
AA AA AA AA AA AA AA
57452693
7275 rs10205484 2 GG GG GG GG GG
GG GG GG GG GG GG GG GG
GG GG AG GG AG GG GG GG
GG GG GG GG GG GG GG GG
GG GG GG GG GG GG GG
85468002
7276 rs10205524 2 AA AA AA AC AC
CC AC CC AC AC CC AC CC
CC AC CC AC AC AA CC AA
CC CC AC AA AC AC AC AA
CC CC AC AC AA AC AC
205167348
7277 rs10205561 2 AG AG AG GG GG
GG GG GG GG GG AG AG AG
AG GG GG AG AG GG GG GG
AG AG AG AG AA GG AG AG
GG GG AG AG GG GG AG
201809704
7278 rs10205580 2 AA AA AA AG AA
AA AG AA AA AA AA AA AA

AA AA AA AA AA AA AA AG
AA AA AA AA AA AG AA AG
AG AA AA AA AA AA AA
84323155
7279 rs10205590 2 AC AA AA AC AA
AA AC CC CC CC CC CC CC
AC AC CC CC AC AA AA AC
AA AA AA AA AC AA AC AC
CC AA AC AA AC CC AA
53991707
7280 rs10205624 2 AA AA AA AG AG
AA GG AG AG AG AA AA AA
AG AG AA AA AA AG AG AA
AG AA AG AG AA AA AA AA
AA AG AA AA AG AA AG
16889001
7281 rs10205628 2 AA AA AG AA AG
AA AG AA AA AA AA AA AA
AA AA AA AG AA AA AA AG
AA AA AA AA AA AA AA AA
AA AA AG AA AA AA AA
53593857
7282 rs10205651 2 AA AA AA AA AA
AG AA AA AA AA AG AA AA
AA AA AA AA AA AA AA AA
AA AA AA AA AA AA AA AA
AA AA AA AA AA AA AA
123249909
7283 rs10205689 2 AG AG GG AA AG
AG AG GG AG AG AG AG AG
GG GG AG AA AG AG AA AG
AG GG AG GG AG AG AG AA
GG AG AG GG AG AG GG
160363669
7284 rs10205696 2 AA AG AG GG GG
GG GG AG GG GG AG AG AA
AG AG GG AG GG GG AA GG
GG AG GG AG AG GG GG AG
GG GG AG AG AG GG AG
165805055
7285 rs10205697 2 AC AC AC AC AC
CC AA AC AA AA AA AA AA
AA AA AA AA AA AA AC AA
AC AC AA AA AC AA AA AA
AC AC AC AA AA AA AA
203845301
7286 rs1020572 20 AA AA AA AC AC
AA CC AC AC CC CC CC CC
CC CC AA AC AA AC CC AC
AC CC CC AC CC AC AC AC

AA AA AC AA CC AC AC
12659751
7287 rs10205766 2 AA AC AC AA AA
AC AA AA AC AA CC CC CC
AC AC CC CC CC AC AC AC
AC AC AA AA AC AA AC AA
CC AC AC AC AA AC AC
105637431
7288 rs10205779 2 AA AA AA AA AA
AA AA AA AG AA AA AA AA
AA AA AA AA AA AA AA AA
AA AA AA AA AA AG AA AG
AA AA AA AA AG AG AA
988346
7289 rs1020579 11 AA AA AG AA AG
AG AA AA AA AA AA AA AA
AG AA AG AG GG AA AG AG
AG AA AA AG GG AG GG AG
AA AG GG GG GG AG GG
40280436
7290 rs10205791 2 AG GG GG GG AG
AG GG AG AG GG GG GG GG
AG AG AG AG GG GG GG GG
AG GG GG AG GG GG GG GG
GG AG AG AG GG GG GG
41839007
7291 rs10205801 2 AG AA AG AG AA
AA AG AA AG AA AA AA AA
AA AA AG AA AA AA AG AA
GG GG GG AG AG AA AG AG
AA AG AA AG AG AG GG
233597196
7292 rs10205809 2 AC AC AC CC AA
AC AC AC AC AC CC CC CC
AC AC CC AC CC AC AC CC
CC AC CC AC AC AA AC AA
CC AC CC AC CC AC AC
16088880
7293 rs10205814 2 AA AA AA AA AG
AG AG AA AA AA AA AA AA
AA AA AG AG AG AA AG AA
AG AG AA AA AA AA AA AA
AG AA AA AA AA AA AA
184818243
7294 rs1020590 15 CC CC CC CC CC
CC CC CC CC CC CC CC CC
CC CC CC CC CC CC CC CC
CC CC CC CC CC CC CC CC
CC CC CC CC CC CC CC
99707518

7295	rs10205909	2	AA	AG	AA	GG	AG
	GG	AG	AG	GG	AG	AA	AG	AG
	GG	GG	AG	AG	AG	AG	AG	GG
	AA	AA	AA	AA	AG	GG	GG	GG
	GG	AG	AA	AA	GG	AG	AG	
3526603								
7296	rs10205913	2	AG	AG	AG	GG	AG
	GG	AG	AA	AG	AG	AA	GG	AG
	AA	AA	AA	AG	AG	AA	AA	AG
	AG	AA	AG	AA	GG	AG	AG	GG
	AG	GG	AG	AG	AG	GG	AA	
67903473								
7297	rs10205938	2	GG	GG	GG	AG	AG
	AA	GG	GG	GG	GG	GG	GG	GG
	GG	GG	GG	GG	GG	GG	AG	GG
	GG	GG	GG	GG	AG	GG	GG	GG
	GG	GG	GG	GG	AG	AG	GG	
236031163								
7298	rs10205941	2	AG	GG	GG	AA	AA
	AA	AG	AG	AG	AG	GG	GG	GG
	AG	AG	GG	AG	GG	GG	GG	AG
	AG	AG	AG	AG	AG	AG	AA	GG
	GG	AA	AG	AG	AA	AG	AA	
130191184								
7299	rs10205956	2	AA	AA	AA	AA	AG
	AG	AA	AG	AA	AG	AA	AA	AA
	AG	AG	AA	AA	AA	AA	AA	AA
	AA	AA	AA	AA	AA	AA	AA	AA
	AG	AA	AA	AA	AA	AA	AA	
13937811								
7300	rs1020605	2	GG	GG	GG	GG	GG
	GG	GG	GG	GG	GG	GG	GG	GG
	GG	GG	GG	GG	GG	GG	GG	GG
	GG	GG	GG	GG	GG	AG	GG	GG
	GG	GG	AG	AG	GG	GG	GG	
12397435								
7301	rs10206058	2	AG	GG	AG	AG	GG
	GG	AG	GG	GG	AG	AG	AG	AG
	AG	AG	AA	AA	AA	AA	GG	AG
	GG	AG	AG	GG	AG	AG	AG	AG
	AA	AA	AG	AA	AA	AA	AG	
42583667								
7302	rs1020608	2	AG	AA	AG	AG	AG
	GG	AG	GG	GG	GG	AA	GG	AG
	AG	AG	GG	AG	GG	GG	GG	AG
	GG	AG	GG	GG	AA	GG	AG	AG
	AG	GG	GG	GG	GG	GG	GG	
12427969								
7303	rs10206100							
2	--	--	--	--	--	--	--	--

	--	--	--	--	--	--	--	--
	--	--	--	--	--	--	--	--
	--	--	--	--	--	--	--	--
	--	--	--	--	13461622			
7304	rs10206101		2	AC	AC	AC	CC	AC
	AC	AC	AC	AC	AC	CC	AC	CC
	CC	CC	CC	CC	CC	AC	CC	AC
	AC	AC	AC	AC	CC	CC	CC	CC
	AC	CC	AA	AC	AA	AA	AA	
241835543								
7305	rs10206131		2	AA	AA	AA	AA	AA
	AA	AA	AA	AA	AA	AA	AA	AG
	AG	AG	AA	AG	AG	AA	AA	AA
	AA	AA	AA	AA	AA	AA	AA	AA
	AA	AA	AA	AA	AA	AA	AA	
2721958								
7306	rs10206255		2	AA	AA	AA	AA	AA
	AA	AA	AA	AA	AA	AA	AA	AA
	AA	AA	AA	AA	AA	AA	AA	AA
	AA	AA	AA	AA	AA	AA	AA	AA
	AA	AA	AA	AA	AA	AA	AA	
112834876								
7307	rs10206257		2	AA	AA	AA	AA	AA
	AA	AA	AA	AA	AA	AA	AA	AA
	AA	AA	AA	AA	AA	AA	AA	AA
	AA	AA	AA	AA	AA	AA	AA	AA
	AA	AA	AA	AA	AA	AA	AA	
198818229								
7308	rs10206280		2	GG	GG	GG	GG	GG
	GG	GG	GG	GG	GG	GG	GG	GG
	GG	GG	GG	GG	GG	GG	GG	GG
	GG	GG	GG	GG	GG	GG	GG	GG
	GG	GG	GG	GG	GG	GG	GG	
170381925								
7309	rs10206319		2	AG	AG	AG	AG	AG
	GG	AA	GG	GG	GG	AA	GG	AG
	GG	GG	GG	AG	AG	GG	GG	GG
	AG	GG	AG	GG	GG	AG	AG	GG
	AG	GG	GG	GG	AG	GG	AG	
71056098								
7310	rs10206343		2	AA	AG	AG	AA	AA
	AA	AA	AA	AA	AA	AG	AG	AA
	AA	AA	GG	AG	AG	AA	AA	AG
	AA	AA	AA	AA	AA	AA	AA	AA
	AG	AA	AA	AA	AA	AG	AA	
46165972								
7311	rs10206405		2	AG	AG	AG	GG	AG
	AG	GG	AG	GG	GG	AA	AA	AA
	AA	AA	AG	AA	AA	AA	GG	AG
	GG	AA	GG	AG	AA	GG	AG	AG

AG AA AG AG AA AG AA
182167100
7312 rs10206439 2 AG AG AG AG AG
AG AG AA AA AA AA AA AA
AG AG AG AG AG GG AG AG
AA AG AG AA AA AA AA AA
AA AA AG AA AA AG AG
140588462
7313 rs10206500 2 GG GG GG GG GG
GG GG GG GG GG GG GG GG
GG GG AG GG GG GG GG GG
AG AG AG AG GG GG GG GG
GG GG GG GG AG GG AG
231213706
7314 rs10206521 2 GG GG GG GG AG
GG AG AA AA AA GG AG AG
AG AG GG GG AG AA AA AA
AG AG AG AG AG AA AA AG
GG GG GG GG AA AG AA
21420828
7315 rs10206546 2 GG GG GG GG GG
GG AG GG AA AG AG AG AG
GG GG AG AG AG AG GG AG
AG AG AG AG AA AA AA AA
GG AG AG AG AG GG AG
145121339
7316 rs10206656 2 GG GG GG GG GG
GG GG GG GG GG GG GG GG
GG GG GG GG GG GG GG GG
GG GG GG GG GG GG GG GG
GG GG GG GG GG GG GG
86499071
7317 rs10206684 2 AA AG AA AG AA
AA AG AG AA AG AG AG AA
AG AG AA AG AG GG GG AG
AG GG AG AG AA AG AA AG
GG AA AA AA AG GG AG
98467028
7318 rs10206763 2 AA AA AA AA AA
AA AG AA AA AA AA AA AA
AA AA AA AA AA AA AA AA
GG AA GG AG GG AG GG AG
AG AA AA AA AG AG AA
77729748
7319 rs10206788 2 AG AG AG GG AG
AG GG GG GG AG GG GG AG
AA AA GG GG GG AG AG AA
GG GG GG GG GG GG GG GG
GG GG AG AG GG GG GG
38056403

7320	rs1020679		11	GG	GG	GG	GG	GG
	GG	GG	GG	GG	GG	GG	GG	GG
	GG	GG	GG	GG	GG	GG	GG	GG
	GG	GG	GG	GG	GG	AG	AG	GG
	GG	AG	AG	AA	GG	AG	GG	
11832343								
7321	rs10206814		2	GG	AG	GG	GG	GG
	GG	GG	AG	GG	AG	GG	AG	GG
	GG	GG	AG	AG	AG	GG	AA	AG
	AG	AG	AG	AG	GG	GG	GG	GG
	AG	GG	AG	AG	GG	GG	GG	
235550191								
7322	rs10206840		2	GG	AG	GG	AG	AG
	AA	GG	AA	GG	AA	GG	AG	AG
	AG	AG	AG	AG	AG	AG	AG	AG
	GG	GG	AG	GG	GG	AG	AG	GG
	AA	AG	GG	GG	AG	AG	GG	
3957337								
7323	rs10206851		2	AG	GG	AG	GG	AG
	AG	GG	GG	GG	GG	AA	GG	AG
	GG	GG	GG	GG	AG	AG	AG	AG
	GG	AG	AG	GG	AG	AG	AG	GG
	GG	GG	GG	GG	AA	AA	AG	
5464026								
7324	rs10206869		2	CC	CC	CC	CC	CC
	CC	CC	CC	CC	CC	CC	CC	CC
	AC	AC	CC	AC	CC	CC	CC	CC
	CC	CC	CC	CC	AC	CC	CC	CC
	AC	CC	AC	AC	CC	CC	CC	
57237043								
7325	rs10206876		2	CC	CC	CC	CC	CC
	CC	CC	CC	AC	CC	CC	AC	AC
	AC	AC	CC	CC	CC	CC	CC	AC
	AC	AA	AC	AC	CC	AC	AC	CC
	AC	AC	CC	CC	AC	CC	AC	
85508065								
7326	rs10206877		2	AA	AA	AA	AG	AA
	AG	AA	AG	AG	AG	AA	AA	AA
	AA	AA	AA	AA	AA	AA	AA	AG
	AA	AG	AA	AA	AG	AA	AG	AG
	AG	AG	AA	AG	GG	AG	GG	
8439905								
7327	rs1020689							
6	--	--	--	--	--	--	--	--
	--	--	--	--	--	--	--	--
	--	--	--	--	--	--	--	--
	--	--	--	--	--	--	--	--
	--	--	--	--	68728797			
7328	rs10206909		2	AA	AA	AA	AA	AA
	AA	AA	AA	AA	AA	AA	AA	AA

AA AA AA AA AA AA AA AA
AA AA AA AA AA AA AA AA
AA AA AA AA AA AA AA
40832068
7329 rs10206916 2 AA AA AA GG AA
AG AG AG AA AA AA AA AA
AA AA AG AA AG AA AA AA
AA AA AA AA AG AA AA AA
AG AA AA AA AG AA AG
67437597
7330 rs10206987 2 -- -- AC AC --
AC -- AC AC AC -- -- --
-- -- AC AC -- -- -- AC
-- AC AC AA AC AC -- AC
-- -- AC -- AC AC --
17030374
7331 rs10207002 2 AG AA AA AA AA
AA AA AA AG AA AG AA AG
AG AG AA AA AA AG AG AG
AA AA AA AA GG AA AG AG
AG AA AA AA AA AA AA
78254270
7332 rs10207015 2 GG GG GG GG GG
GG GG GG GG GG GG GG GG
GG GG GG GG GG GG GG GG
GG GG GG GG GG GG GG GG
GG GG GG GG GG GG GG
58252514
7333 rs10207020 2 AG GG AG AA AG
AG AA AG GG GG GG AG GG
AG GG AG AG AG GG AG AG
AG AG AG GG GG AG GG AG
GG AG GG AG AG AG GG
213107619
7334 rs10207030 2 GG GG GG GG GG
GG GG GG GG GG GG GG GG
GG GG GG GG GG GG GG GG
GG GG GG GG GG GG GG GG
GG GG GG GG GG GG GG
88885077
7335 rs1020705 2 AA AA AA AA AA
AA AA AA AC AC AA AA AA
AA AA AA AA AA AA AA AA
AA AA AA AA AA AA AA AA
AA AA AA AA AA AA AA
199770190
7336 rs10207061 2 AA AA AA AA AA
AA AA AG AA AG AA AA AA
AG AG AA AG AA AG AG AG
AA AA AA AA AA AA AA AA

AA AG AG AG AA AA AA
134717465
7337 rs10207082 2 GG GG GG GG GG
GG GG GG GG GG GG GG GG
GG GG GG GG GG GG GG GG
GG GG GG GG GG GG GG GG
GG AG GG AG GG GG GG
56942624
7338 rs10207133 2 GG AG GG GG GG
AG AG GG GG GG AG GG GG
AG AG GG AG AG GG GG GG
GG AG GG GG AA GG AG AG
GG GG GG GG AG AG GG
119905653
7339 rs10207138 2 GG GG GG AG AG
GG AA GG GG GG GG GG AG
AA AA AG AG -- GG AG AG
AA AA AG AA GG AG GG AG
GG GG AG GG GG GG AG
36977577
7340 rs1020723 19 AA AA AA AG AA
AG AA AA AA AA AG AA AA
AA AA AA AA AA AA AA AA
AA AA AA AA AG AA AA AA
AA AA AA AA AA AA AA
16727129
7341 rs10207288 2 AA AG AA AG AG
AA AG AG AA AG AA AA AA
AA AA AG AA AA AA AA AA
AG AG AA AA AG AA AA AA
AA AA AG AG AA AA AA
212541377
7342 rs10207325 2 AA AG AA AA AA
AA AA AA AA AA AA AG AA
AA AA AG AA AG AG AG GG
AG AA GG AG AA AA AA AA
AA AA GG AG AA AA AA
10357818
7343 rs10207336 2 CC CC CC CC CC
CC CC CC AC AC AC CC CC
CC CC CC CC CC CC CC CC
CC CC AC AA CC CC CC CC
CC CC CC CC CC CC CC
196924449
7344 rs10207341 2 -- -- -- AG AG
AG AG -- -- GG AG AG AG
AA -- -- AG -- -- -- AG
AA AG AG AG -- -- -- --
AG -- -- AG AG AA --
134538314

7345 rs10207351 2 AG AG GG AA AG
AG AA AG AG AG AA AA AA
AG AG GG AG GG AG GG AA
GG AG AG GG AG AG GG AG
AG AG AA AG AA AG AG
164625727
7346 rs10207371 2 AA AA AG GG GG
GG GG AG AG AG AA AG AA
AA AA AG AA AG AG AG GG
AA AG AA AA AG GG GG AG
AA AA AA AA AG AG AG
101763227
7347 rs10207380 2 GG AG GG AG AG
AA GG AG GG GG GG AG GG
GG GG GG GG GG AG AG AA
AG AG AG AG GG GG GG GG
AA GG GG GG GG AG GG
241860911
7348 rs10207388 2 GG GG GG GG GG
GG GG AG AG AG GG GG GG
GG GG GG GG GG GG GG GG
GG GG GG GG GG GG GG GG
GG GG GG GG GG GG GG
123769845
7349 rs10207392 2 AA AA AA AA AG
AA AG AG AG GG AA AA AA
AA AA AA AA AA AA AA AA
AG GG AG AG AA AA AA AA
GG AA AA AA AA AA AA
111849659
7350 rs10207397 2 GG GG GG GG GG
GG GG GG GG GG GG GG GG
GG GG GG GG GG GG GG GG
GG GG GG GG GG GG GG GG
GG GG GG GG GG GG GG
239990096
7351 rs1020740 8 AG AA AA GG GG
GG GG GG GG AG AG AG AA
AA AG AG AG AG AG AG AA
GG AG GG AG AG GG AG GG
GG GG GG GG GG GG GG
133350326
7352 rs10207406 2 AA AG AA AG AG
AA GG AG AA AG AA AA AG
AG AG AA AA AA AA AA AA
GG GG AG GG AA AA AA AA
AG AA AG AA AA AA AA
3456506
7353 rs10207452 2 AG AG AA AG AG
GG AA AA AA AA AA AA AA

AA AA AA AA AA AA AA AA
AA AA AA AA AG AA AA AG
AG AG AG AG AG AG AG
171233990
7354 rs10207525 2 CC CC CC CC AC
CC AC CC CC CC CC CC CC
CC CC AC CC CC CC CC AC
AC CC AC CC CC CC CC CC
AC AC CC CC CC CC CC
59711640
7355 rs10207540 2 GG GG GG GG GG
GG GG GG GG GG GG GG GG
GG GG GG GG GG GG GG GG
GG GG GG GG GG GG GG GG
GG GG GG GG GG GG GG
98426994
7356 rs1020759 4 GG GG AG GG GG
GG GG GG GG AG AG GG AG
AG AG AG GG AG AG AG GG
GG AG AG GG GG AG AG GG
AG GG GG GG AA AG AA
103510511
7357 rs10207608 2 GG AG AG AG AG
AG AA GG GG GG AG AG AG
AG AG GG GG GG AG GG AG
AG AA GG GG GG AG AG GG
GG GG GG GG AG AG GG
110042294
7358 rs10207672 2 AA AG AA AG AA
AA AG GG GG GG AA AA AA
AA AA GG GG AG AG GG AG
GG GG GG AG GG AG GG AG
AG AA AA AA GG AG GG
234888161
7359 rs10207678 2 GG GG AG AA GG
AG AG GG GG GG GG GG GG
GG GG AG GG AG AG AG AG
GG AG AG AG GG GG GG GG
GG GG GG GG GG GG GG
51626179
7360 rs10207716 2 AG AG GG AG AG
AA AG AA AA AA AG AG GG
AG AG AG AG GG GG AG GG
AA GG AG AG AG GG GG AG
AG AG AA AG AG AG GG
166266805
7361 rs10207739 2 AA AA AA AA AA
AA AA AA AG AG AG AG GG
GG GG AA AA AA AA AA AA
AA AA AA AA AA AG AA AG

AA AA AG AG AA AA AA
233684097
7362 rs1020775 9 AG GG AG AG GG
AG GG AA AA AG GG AG AG
AG AG AA AA AA AG GG AG
AG AG AG AG AA GG AG AG
AG AG GG AG AG AA GG
76495550
7363 rs10207753 2 AA AA AA AC AC
AA AC AC AA AA AA AA AC
CC CC AC AA AC AC AC AC
AC AC AA CC AC AC AC AA
AA AC AA AC AA AA AA
166492582
7364 rs10207820 2 AA AA AG AG AA
AA AG AG AG AG AG AG AG
AA AA AG AA AG GG AG GG
GG AG GG GG AA GG AG AG
GG GG AA AG GG AG AG
112699350
7365 rs10207855 2 AA AA AA AA AA
AA AA AA AA AA AA AA AA
AA AA AA AA AA AA AA AA
AA AA AA AA AA AA AA AA
AA AA AA AA AA AA AA
97063520
7366 rs10207860 2 GG GG GG AG GG
AG GG GG GG GG GG GG GG
GG GG GG GG GG GG GG GG
GG GG GG GG GG AG AG GG
GG GG GG GG GG GG GG
190146586
7367 rs10207872 2 AC AC AC AC CC
AC CC CC CC CC AA AA AA
AA AA AC AA AA AC AA CC
AA AA AC AC AC AC AC CC
AC AC AC AC AC CC AC
185300641
7368 rs10207958 2 AA AC CC CC --
AC CC AC AC AC AC CC CC
AA AA CC CC AC CC CC AC
CC AC CC AC AA AC AA AC
AA AC AA AC AC AC AA
61225330
7369 rs10208 17 AG AA AA AG AG GG
AG GG GG GG AG GG AG GG
GG AG GG GG AG AG GG GG
AG AG AG GG AA AG AG AG
AA GG AG AG AG AA 42300278
7370 rs10208010 2 AA AA AA AA AG

	AG	AA	AG	GG	AG	AA	AA	AA
	AA	AA	AG	AG	AA	AA	AA	AG
	GG	AG	AG	GG	AA	AG	AA	AA
	AA	AA	GG	AG	AG	AG	AA	
17050261								
7371	rs10208050		2	AA	AA	AA	AA	AA
	AA	AG	AA	AA	AA	AA	AA	AA
	AA	AA	AA	AA	AA	AA	AA	AA
	AA	AA	AA	AA	AA	AA	AA	AA
	AA	AA	AA	AA	AA	AA	AA	
187063564								
7372	rs10208058		2	AG	AG	AG	AA	AA
	AA	AA	AA	AG	AG	AG	AG	AG
	AA	AA	AG	AG	AG	GG	AG	AG
	AA	AG	AG	AA	AA	AA	AA	AA
	GG	GG	AG	AG	AG	AA	AG	
150184523								
7373	rs10208070		2	AA	AG	AA	AG	AA
	AA	AG	AG	AA	AG	AG	AG	AA
	AG	AG	AA	AG	AG	GG	GG	AG
	AG	GG	AG	AG	AA	AG	AA	AG
	GG	AA	AA	AA	AG	GG	AG	
98461802								
7374	rs1020808		4	GG	GG	GG	GG	GG
	GG	GG	GG	GG	GG	GG	GG	GG
	GG	GG	GG	GG	GG	GG	GG	GG
	GG	GG	GG	GG	GG	GG	GG	GG
	GG	GG	AG	GG	GG	AG	AG	
36949073								
7375	rs10208080		2	AG	GG	GG	AG	AG
	AA	GG	GG	GG	AG	GG	AG	GG
	GG	GG	GG	AG	AG	GG	AG	GG
	AG	GG	GG	AG	GG	AA	AG	AG
	AG	GG	AG	AG	GG	GG	AG	
43106956								
7376	rs10208137		2	GG	AG	AA	AA	AG
	AG	AA	AG	AG	AG	GG	GG	GG
	AG	AG	AG	AA	AG	AG	AG	GG
	AA	AG	AA	AG	GG	GG	GG	GG
	GG	GG	GG	GG	AG	AG	GG	
61178171								
7377	rs10208189		2	GG	GG	GG	GG	AG
	--	AG	AG	GG	AG	AG	AG	AG
	AA	AA	AG	AG	AG	GG	GG	AG
	AG	GG	AA	AG	GG	AA	AG	AG
	AA	AG	GG	GG	AG	AG	AA	
20020598								
7378	rs1020819		3	CC	CC	CC	AA	AA
	AA	AA	AA	AA	AA	AA	AC	AC
	AA	AC	AC	AC	AC	AA	AA	AC

	AA	AC	AC	AA	AA	AA	AA	AA
	AA	AA	AA	AA	AA	AC	AC	
53762713								
7379	rs10208207		2	AG	GG	AG	AG	AG
	GG	AA	GG	AG	AG	AG	AG	GG
	GG	GG	AA	AG	AA	GG	GG	GG
	AG	GG	AG	GG	AG	GG	GG	AG
	GG	GG	AG	GG	GG	GG	GG	
169074972								
7380	rs10208221		2	GG	AG	GG	GG	GG
	GG	GG	AG	GG	GG	GG	GG	GG
	GG	GG	GG	GG	GG	AG	GG	AG
	GG	GG	GG	GG	AG	GG	GG	GG
	GG	GG	AA	AG	GG	GG	GG	
213432425								
7381	rs10208250		2	AG	AG	GG	AA	AA
	AA	AA	GG	AG	AG	AG	AG	AG
	AA	AA	AA	AA	AA	AG	GG	AA
	AG	AG	AA	GG	GG	GG	GG	GG
	AA	AG	AA	AA	AA	AA	AA	
168529511								
7382	rs10208260		2	AG	AA	AA	AA	AG
	AA	AG	AG	GG	AG	AG	AG	AA
	AA	AA	GG	GG	GG	AA	AA	AG
	AG	AA	AG	AG	AG	AG	AG	AG
	AG	AA	AA	AA	AG	AG	AA	
99546423								
7383	rs10208262		2	GG	GG	GG	GG	GG
	GG	GG	GG	GG	GG	GG	GG	GG
	GG	GG	GG	GG	GG	GG	GG	GG
	GG	GG	GG	GG	GG	GG	GG	GG
	GG	GG	GG	GG	GG	GG	GG	
77154515								
7384	rs10208300		2	AA	AA	AA	AA	AG
	AA	AG	AA	AA	AA	AA	AA	AA
	AA	AA	AA	AA	AA	AA	AA	AA
	AA	AA	AA	AA	AG	AA	AG	AA
	AG	AG	AA	AA	AA	AA	AG	
51316822								
7385	rs10208330		2	AA	AA	AA	AA	AA
	AA	AA	AA	AA	AA	AA	AA	AA
	AA	AA	AC	AA	AA	AA	AA	AC
	AA	AA	AA	AA	AC	AA	AA	AA
	AC	AA	AA	AA	AA	AA	AA	
13431193								
7386	rs10208333		2	AC	AC	AC	CC	CC
	AC	CC	CC	CC	CC	CC	AA	AC
	AC	AC	CC	CC	AC	AC	CC	AA
	AC	AA	AA	AC	CC	CC	CC	CC
	AC	AC	AC	CC	CC	CC	AC	

221201659
7387 rs10208358 2 AA AA AA GG GG
GG AG AG AA AG AA AA AA
AA AA AA AA AG GG AG GG
AG AG GG AG AA AG AA AA
AG AG AG AG AG AG GG
9554255
7388 rs10208367 2 AG GG GG GG GG
GG GG AG AG AG GG GG AG
AG AG AG AG GG AG GG GG
GG GG GG GG GG GG GG GG
GG GG GG GG GG AG GG
42881492
7389 rs10208385 2 GG GG GG GG AG
GG AG GG GG GG AA AA AA
AG AG GG GG AG AG AG AA
GG AG GG AG AG AG GG AG
AG AG AA AG GG GG GG
56778009
7390 rs10208426 2 AG GG AG AA AA
AA AA AA AG AA GG AG GG
GG GG AG AG AG AG AG AG
AA AA AA AA AG GG AG AG
AG AA GG AG GG GG GG
6573786
7391 rs10208431 2 GG GG GG GG GG
GG GG GG GG GG GG GG GG
GG GG GG GG AG GG GG GG
GG AG GG GG GG GG GG GG
GG GG GG GG GG AG GG
49507819
7392 rs10208486 2 AA AA AA AA AA
AA AA AA AA AA AA AA AA
AA AA AA AA AA AA AA AA
AA AA AA AA AA AA AA AA
AA AA AA AA AA AA AA
147285785
7393 rs10208502 2 AA AA AA AA AA
AA AA AA AA AA AA AA AA
AA AA AA AA AA AA AG AG
AA AA AA AA AA AA AA AA
AA AG AA AA AG AG AA
237734309
7394 rs10208525 2 AA AA AA AA AA
AA AA AA AG AG AG AA AA
AA AA AA AG AG AG AA AG
AG AA AG AA AG AA AG AG
GG AG GG AG AG AG AG
189904203
7395 rs1020853 2 AC CC AC CC CC

```
        CC      CC      CC      CC      CC      CC      CC      CC
        CC      CC      AC      AC      AC      CC      AC      CC
        CC      CC      CC      CC      AC      CC      AC      CC
        AC      AC      CC      CC      CC      CC      AC
166879782
7396    rs10208604      2       AA      AA      AA      AG      AA
        AA      AG      AA      AA      AA      AA      AA      AA
        AG      AG      AA      AA      AA      AG      AA      AG
        AG      AG      AA      AG      AG      AG      AA      AG
        AG      AG      GG      GG      GG      AG      AG
2296426
7397    rs10208677      2       AG      AG      AG      GG      AG
        AG      AG      AG      AG      AG      AG      AG      AG
        GG      GG      GG      GG      AG      AG      AG      AG
        AG      AG      AG      AG      AA      AA      AA      AA
        AG      AA      AG      AA      AG      AG      AA
72089937
7398    rs10208695      2       AA      AG      AG      AG      AA
        AG      AA      AG      AA      AA      AA      AA      AA
        AA      AA      AA      AA      AA      AA      AA      AG
        AG      AG      AG      AG      AA      AA      AA      AA
        AA      AA      AG      AA      AA      AA      AG
167430697
7399    rs10208787      2       AA      AA      AA      AA      AA
        AA      AA      AA      AA      AA      AA      AA      AA
        AA      AA      AA      AA      AA      AA      AA      AA
        AA      AA      AA      AA      AA      AA      AA      AA
        AA      AA      AA      AA      AA      AA      AA
101217817
7400    rs10208804      2       CC      CC      CC      AC      CC
        AC      CC      CC      CC      CC      AA      AC      AA
        AA      AA      CC      CC      CC      AC      CC      AC
        CC      CC      AC      CC      AA      AC      AA      AA
        AC      CC      CC      CC      AC      AC      AA
12013686
7401    rs1020884
9       --      --      --      --      --      --      --      --
        --      --      --      --      --      --      --      --
        --      --      --      --      --      --      --      --
        --      --      --      --      --      --      --      --
        --      --      --      --      100158052
7402    rs10208875      2       AG      AG      AG      AA      AA
        AG      AA      AA      AA      AA      AA      AA      AG
        AG      AG      AG      AG      AA      AA      GG      AG
        AG      AA      AA      AA      GG      AG      GG      AG
        GG      AG      AA      AG      GG      GG      AG
134753056
7403    rs1020890       15      GG      GG      GG      GG      GG
        GG      GG      GG      GG      GG      GG      GG      GG
        AG      GG      GG      GG      GG      GG      GG      GG
```

GG GG GG GG GG GG GG GG
GG GG GG GG GG GG GG
57400274
7404 rs10208925 2 AG AA AA AA AA
AA AA AA AA AA AG AG AG
AG AG AA AA AA GG GG GG
AG AG AG AA AG AA AG AA
AG AG AG AG AG AG AA
79152829
7405 rs10208979 2 AG AG AG AG AG
GG AG GG GG GG AG AG AG
AG AG AA AA AA GG AA GG
AG AG AA AG AG GG AG AG
GG AG GG GG AG AG AG
210335716
7406 rs10209074 2 AA AA AA AA AA
AA AA AA AA AA AA AA AA
AA AA AA AA AA AA AA AA
AA AA AA AA AA AA AA AA
AA AA AA AA AA AA AA
238287987
7407 rs10209084 2 AC AA AA AA AC
AA AC AA AA AC AA AA AA
AA AA AA AC AA AA AA AA
AA AA AA AA AA AC AC AC
AC AA CC AC AC AC AC
240010764
7408 rs10209087 2 AA AA AA AA AA
AA AA AA AA AA AA AA AA
AA AA AA AA AA AA AA AA
AA AA AA AA AA AA AA AA
AA AA AA AA AA AA AA
175095555
7409 rs10209140 2 GG GG GG AG AG
AG AG GG GG GG GG GG GG
GG GG GG GG GG GG GG GG
AG AG AG AG GG GG GG GG
GG GG AG AG GG AG GG
63922369
7410 rs10209160 2 GG GG GG GG GG
GG GG GG GG GG AG GG GG
GG GG GG GG GG GG GG GG
GG GG GG GG AG GG AG GG
GG GG GG GG GG GG GG
113495785
7411 rs10209196 2 CC CC CC CC CC
CC CC CC CC CC CC CC CC
CC CC CC CC CC CC CC CC
CC CC CC CC CC CC CC CC
CC CC CC CC CC CC CC

237075973
7412 rs10209260 2 CC CC CC CC CC
CC CC CC CC CC CC CC CC
CC CC CC CC CC CC CC AC
CC CC CC CC CC CC CC CC
CC CC CC CC CC CC CC
112662407
7413 rs10209275 2 GG AG AG GG GG
GG GG AG AG AG GG GG GG
GG GG GG AG GG GG GG GG
GG GG GG GG AG AG AG GG
GG AG GG AG AA AG AA
59791233
7414 rs10209318 2 AG GG AG AG GG
AG GG AA GG AG GG GG GG
GG GG GG AG AG GG AG GG
GG GG GG GG AG AG AG AA
GG GG GG GG GG GG GG
176590041
7415 rs10209382 2 AG GG AG AG AG
GG AG GG AG GG GG GG GG
GG GG AG GG GG GG GG GG
AA GG AG GG AG GG AG AG
GG GG AG AG GG GG GG
183304603
7416 rs10209401 2 CC CC CC CC CC
CC CC CC CC CC CC AC AC
AC AC CC CC CC CC CC CC
CC CC CC CC CC CC CC CC
CC CC CC CC CC CC AC
218204681
7417 rs10209450 2 GG GG GG GG GG
GG GG AG AG AG GG GG GG
GG GG AG GG AG AG AG AG
AG GG AG AG GG GG GG GG
GG GG GG GG GG GG GG
129539213
7418 rs10209471 2 AC CC AC AC AC
AA AC AC AC AC AA AA AA
AA AA AA AC AC AA AA AA
AA AA AA AA AA AA AA AA
AA CC AC AC CC AC CC
212159787
7419 rs10209493 2 -- -- -- -- --
-- -- AG AG -- -- -- --
-- -- -- -- -- -- -- --
-- -- -- -- -- -- -- --
-- -- -- -- AG AG --
238032491
7420 rs10209564 2 AG AG AG GG GG

	GG	GG	GG	GG	GG	GG	GG	GG
	GG	GG	GG	GG	GG	AG	GG	AG
	GG	GG	GG	GG	GG	GG	GG	GG
	GG	GG	GG	GG	GG	GG	GG	
239459603								
7421	rs10209612		2	AG	AA	AA	AA	AA
	AA	AA	AA	AA	AA	AA	AA	AA
	AA	AA	AA	AG	AG	AA	AA	AA
	AA	AA	AA	AA	AG	AA	AA	AG
	AA	AA	AG	AG	AA	AA	AA	
79869978								
7422	rs10209625		2	CC	AC	AA	AC	AA
	AC	AA	CC	AC	CC	CC	AC	AC
	AC	AC	AA	AC	AC	AC	AC	AA
	AA	AC	AA	AA	CC	AC	AC	CC
	AC	AC	AC	AC	AC	CC	AA	
86806649								
7423	rs1020963		9	GG	GG	GG	AG	AG
	GG	AA	GG	GG	GG	AG	GG	AG
	AG	GG	GG	GG	GG	GG	GG	GG
	AG	GG	AG	GG	GG	GG	GG	GG
	AG	GG	GG	GG	GG	GG	GG	
97789745								
7424	rs10209732		2	AA	AA	AA	AA	AA
	AA	AA	AG	AA	AG	AA	AA	AA
	AA	AA	AA	AA	AA	AA	AA	AA
	AA	AA	AA	AA	AA	AA	AA	AA
	AA	AA	AA	AA	AA	AA	AA	
159106259								
7425	rs10209739		2	AA	AA	AA	AA	AA
	AA	AA	AG	AA	AG	AA	AG	AG
	AA	AA	AG	AG	AA	AG	AG	AG
	AA	AA	AA	AA	GG	AA	AG	AG
	AG	AG	AG	AG	AG	AA	GG	
13372691								
7426	rs10209754		2	AA	AA	AA	AA	AA
	AA	AA	AG	AG	AG	AA	AA	AA
	GG	GG	AG	AA	AG	AA	AA	AA
	AA	AA	AA	AA	AG	AA	AA	AG
	AG	AA	AG	AA	AA	AA	AA	
45404169								
7427	rs1020977		16	AA	AA	AA	AG	AG
	AA	AG	AA	AG	AG	AA	AA	AA
	AA	AA	GG	GG	GG	AG	AA	AG
	GG	AG	GG	AG	AG	AA	AA	AG
	AG	GG	AG	AG	AA	AG	AG	
58942066								
7428	rs10209795		2	AC	AC	AC	AC	AC
	AC	AC	AC	AC	AC	AA	AA	AA
	AA	AA	AC	AC	CC	AA	AC	AA

AC AC AC AC AA AA AA AA
AA AC AC CC CC CC AC
114050116
7429 rs10209807 2 AA AA AA AG AG
AA GG AG AG AG AA AG AG
AG AG AA AA AG AG AG AG
AG GG GG GG AG AA AA AA
AG AA AA AA AG GG AA
222542378
7430 rs1020986 4 AG AG AG AA AA
AA AA AG AG GG AG GG AG
AG AA AG GG AG AG AG AG
AG AG AG AG GG GG GG GG
AG AA AG AG AA AG AA
57011624
7431 rs1020987 15 AA AG AG AA AA
AA AA GG AG AA AG AA AG
AG AA GG GG GG AG AG GG
GG AG AG AA AG AG AG AG
AA GG AG GG AA AA AA
95308129
7432 rs10209938 2 GG GG GG GG GG
GG GG GG GG GG GG GG GG
GG GG GG GG GG GG GG GG
GG GG GG GG GG GG GG GG
GG GG GG GG GG GG GG
240264825
7433 rs10209954 2 AA AA AA AG AG
GG AA AG AG AG AA AA AA
AA AA GG AG AG AG AG AG
GG GG AG AG GG GG GG GG
GG AG AG GG AG AA AG
216795091
7434 rs10209961 2 AA AG AG AG AA
AA AG AA AG AA AA AA AA
AA AA AA AG AG AG AG GG
AA AA AA AA AG AA AG AA
AA AA AG AG AA AA AA
84544632
7435 rs10209970 2 AA AC AA AA AA
AA AA AA AA AA AC AC AA
AC AC AA AC AC CC CC AC
AC AC AC AC AA AC AA AC
AC AA AA AA AA AC AC
98622697
7436 rs10210 18 AC AC AC CC CC AC
CC AC CC AC CC AC AC AC
AC AA AA AA AC CC AC AC
AC AC AC AC AC AC CC AA
AA AC AC AA AC AA 20838286

7437 rs10210025 2 GG GG GG GG GG
GG GG GG GG GG GG GG GG
GG GG GG GG GG GG GG GG
GG GG GG GG GG GG GG GG
GG GG GG GG GG GG GG
239048353
7438 rs10210100 2 CC CC CC CC CC
CC CC CC CC CC CC CC CC
CC CC CC CC CC CC CC CC
CC CC CC CC CC CC CC CC
CC CC CC CC CC CC CC
199947488
7439 rs10210118 2 AG AG AG AG AG
GG AG GG GG GG GG GG GG
GG GG GG AG AG GG AG GG
AA GG AA AG AG GG GG GG
GG AG GG AG GG GG AG
180378253
7440 rs10210192 2 AA AA AA AG AG
GG AA AG AG AG AA AA AA
AA AA GG AG AG GG AG GG
AA AG AA AG AA AA AA AA
AA AA AG AA AA AA AA
174924658
7441 rs10210251 2 AA AA AA AA AA
AA AA AA AA AA AA AA AA
AA AA AA AA AA AA AA AA
AA AA AA AA AA AA AA AA
AA AA AA AA AA AA AA
63058145
7442 rs10210254 2 AA AG AA GG GG
GG GG AG GG GG GG AG AG
AG AG AG GG GG GG AG AG
AG AG AG GG AG AG AG GG
GG GG AA AG AA AA AG
231032372
7443 rs10210295 2 GG GG GG GG GG
GG GG GG GG GG GG GG GG
GG GG GG GG GG GG GG GG
GG GG GG GG GG GG GG GG
GG GG GG GG GG GG GG
88460235
7444 rs10210302 2 AG GG AG GG GG
GG GG AG AG AG AA AA AA
AA AA AA AA AG AA GG AG
AG AG AG GG AG AG GG AG
AG AG AA AG AG GG AG
234158839
7445 rs10210332 2 AA AA AA AA AA
AA AA AA AA AA AA AA AA

	AA	AA	AA	AA	AA	AA	AA	AA
	AA	AA	AA	AA	AA	AA	AA	AA
	AA	AG	AA	AG	AG	AG	AA	
172286041								
7446	rs10210369		2	GG	GG	GG	AA	AG
	AG	AA	AG	AG	AG	GG	GG	GG
	GG	GG	GG	GG	GG	AG	AG	AG
	GG	GG	GG	GG	AG	AG	AA	AG
	AG	AG	AA	AG	AG	GG	AG	
167028525								
7447	rs1021037		14	GG	GG	GG	GG	GG
	GG	GG	GG	AG	GG	GG	AA	AG
	AG	GG	GG	GG	GG	GG	GG	GG
	GG	GG	GG	GG	GG	GG	GG	GG
	GG	AG	AG	GG	GG	GG	GG	
85548222								
7448	rs10210389		2	GG	GG	GG	GG	GG
	GG	GG	GG	GG	GG	AG	GG	GG
	GG	GG	GG	GG	AG	GG	GG	GG
	GG	GG	GG	GG	GG	GG	GG	GG
	GG	GG	GG	GG	GG	GG	GG	
67195459								
7449	rs10210465		2	GG	GG	GG	AA	AG
	AG	AG	GG	GG	GG	GG	GG	GG
	GG	GG	GG	AG	GG	GG	GG	GG
	GG	GG	GG	GG	AG	GG	AG	AG
	GG	AG	GG	AG	GG	GG	AG	
231039578								
7450	rs10210490		2	AA	AA	AA	AA	AA
	AA	AA	AA	AA	AA	AA	AA	AA
	AA	AA	AA	AA	AA	AA	AA	AA
	AA	AA	AG	AG	AA	AA	AA	AA
	AA	AA	AA	AA	AA	AA	AA	
111849913								
7451	rs10210521		2	GG	GG	GG	GG	GG
	GG	GG	GG	GG	GG	GG	GG	GG
	GG	GG	GG	GG	GG	GG	GG	GG
	GG	GG	GG	GG	GG	AG	GG	AG
	AG	AG	GG	AG	GG	GG	GG	
49073574								
7452	rs10210526		2	GG	GG	GG	AG	AG
	AA	GG	GG	GG	GG	GG	GG	GG
	GG	GG	GG	GG	GG	GG	GG	GG
	GG	GG	GG	GG	GG	GG	GG	GG
	GG	GG	GG	GG	GG	GG	AG	
130088717								
7453	rs1021059		8	AG	GG	GG	GG	GG
	GG	AG	AA	AA	AA	GG	GG	AG
	AG	AG	AA	AG	AG	GG	AG	GG
	AA	AG	AA	GG	AG	AG	AG	AG

AG GG AG GG AG AG GG
136153043
7454 rs10210665 2 AA AA AG AA AG
AG AA AA AA AA AA AA AA
AG AG AA AA AA AA AA AG
AA AA AA AA AA AA AA AA
AA AA AA AA AA AA AA
40532749
7455 rs10210670 2 AC AC AC AA AC
AA AC AC AC AC CC AC AC
AC AC AA AA AA AC AC AA
AA AA AA AA AA AC AA AC
AC AA AA AA CC AC AC
220877302
7456 rs10210671 2 GG GG GG GG GG
GG GG GG GG GG GG GG GG
GG GG GG GG GG GG GG GG
GG GG GG GG GG GG GG GG
GG GG GG GG GG GG GG
86861739
7457 rs10210687 2 AG AG AG AG AG
AG AG AG AG AG AG AG AA
AG AG AG AA GG AA AA AG
AA AA AA AA AA AG AG AA
GG AG AA AG AA AA AG
137389497
7458 rs10210780 2 CC AC CC AC AC
CC AC CC CC CC CC CC CC
CC CC CC CC CC AC AC CC
CC CC CC CC AC CC AC AC
CC CC CC CC CC CC CC
12591069
7459 rs10210794 2 CC CC CC CC CC
CC CC CC CC CC CC CC CC
CC CC CC CC CC CC CC CC
CC CC CC CC CC CC CC CC
CC CC CC CC CC CC CC
176129254
7460 rs10210850 2 AA AA AA AA AA
AA AA AA AA AA GG GG AG
AA AA AA AA AA AG AG AA
AG GG AG AG AA GG AG AG
AG AG AA AG AG AG AG
127527100
7461 rs10210854 2 GG GG GG GG GG
GG GG AG AG GG GG AG AG
AG AG GG AG AG AG GG AG
GG GG GG GG GG AA AG AG
GG AG AG AG AG AA GG
146365598

7462 rs1021087 8 AA AA AA AC AC
AC AA AA AA AA AA AA AA
AA AA AA AA AA AA AC AA
AA AA AA AA AA AC AA AA
AA AA AA AA AC AC AC
13456131
7463 rs10210926 2 GG GG GG GG GG
GG GG GG GG GG GG GG GG
GG GG GG GG GG GG GG GG
GG GG GG GG GG GG GG GG
GG GG GG GG GG GG GG
201917346
7464 rs10210937 2 AC AC AC AA AA
AA AA AC AA AA AA AC AA
AA -- AA AC AC AA AC AC
AC AC AC CC AA AC AC AA
AA CC AA AC AC AA CC
202828666
7465 rs10210959 2 AG AG AA GG GG
GG GG AG AG AG AG GG GG
AA AA AA AA AA GG GG AG
AG AG AG AG -- AG AG GG
GG AG AG GG GG GG AG
57614310
7466 rs10210965 2 GG GG AG AG AG
GG AG AA AA AA GG AG AG
AG AG GG AG GG AG AG AG
AG AG AG AG AA GG AG AG
AG GG AG AG AG GG AG
127497136
7467 rs10210989 2 AG AG AG AG AA
AG AG AG AG AA GG AG AG
AA AG GG GG GG AG AG AG
AG AG AA AG AG AG AA AG
AG AG AA AA GG GG GG
214867770
7468 rs10211034 2 GG GG GG GG GG
GG GG AG AG AA GG GG GG
GG GG GG GG GG GG AG GG
GG GG GG GG AG AG GG AG
AG GG GG GG GG GG AG
150492237
7469 rs10211067 2 AC AC AA AC AA
AC AC AC AC AC AC CC CC
CC CC AC AC CC CC CC CC
CC AC CC CC CC AC AC CC
AC AC AC CC AC AA CC
98703659
7470 rs10211073 2 GG AG AG AG AA
AG AG AG AG GG AG AG AG

	GG	GG	AG	AG	AA	AG	GG	AG
	AG	AA	AG	AG	GG	AG	AG	GG
	GG	AA	AG	AA	AA	AA	AG	
113457049								
7471	rs10211119		2	GG	GG	GG	AG	GG
	AG	GG	GG	GG	GG	AG	AA	AA
	GG	GG	GG	GG	AG	GG	GG	GG
	GG	GG	GG	GG	GG	GG	GG	GG
	AG	AG	GG	AG	GG	GG	GG	
64824348								
7472	rs10211133		2	GG	GG	GG	GG	GG
	GG	GG	GG	GG	GG	AG	AG	AG
	GG	AG	GG	GG	GG	GG	GG	GG
	GG	GG	GG	GG	GG	GG	GG	GG
	GG	GG	GG	GG	GG	GG	GG	
118496598								
7473	rs10211190		2	AA	AA	AA	AG	GG
	AG	GG	AA	AA	AA	AA	AA	AA
	AA	AA	AG	AG	GG	AA	AA	AA
	AG	AG	AA	AG	AG	AG	GG	GG
	GG	AG	GG	AG	AG	AG	AA	
10725428								
7474	rs10211229		2	GG	AG	GG	GG	AG
	AG	GG	GG	AG	GG	GG	GG	AG
	AA	AA	AG	AG	AG	AG	GG	AG
	GG	AG	GG	AG	GG	GG	GG	GG
	GG	AA	GG	AG	AG	GG	AG	
182253217								
7475	rs10211242		2	GG	GG	GG	GG	GG
	GG	GG	GG	GG	GG	GG	GG	GG
	GG	GG	GG	AG	GG	GG	AG	GG
	GG	GG	GG	GG	GG	GG	GG	GG
	GG	GG	GG	GG	AG	AG	GG	
172104703								
7476	rs10211268		2	GG	GG	GG	GG	AG
	AG	GG	AG	AA	AG	GG	GG	GG
	GG	GG	AG	AG	AG	GG	GG	AG
	AA	AG	AG	AA	GG	AG	GG	GG
	GG	GG	AA	AG	AG	AG	GG	
17056464								
7477	rs10211296		2	AG	AG	AA	AA	AG
	AG	AA	AA	AA	AA	AG	AG	AG
	AA	AA	AG	AG	AG	AA	AG	AG
	AA	AG	AA	AG	AG	AA	AA	AG
	AA	AG	GG	AG	AA	AA	AG	
166490343								
7478	rs1021135		3	AA	AA	AA	AA	AA
	AA	AA	AA	AA	AA	AA	AA	AA
	AA	AA	AA	AA	AA	AA	AA	AA
	AA	AA	AA	AA	AA	AA	AA	AA

AA AA AA AA AA AA AA
138620638
7479 rs10211370 2 AG GG GG AG AA
AA AG GG GG GG GG GG GG
AG AG AG AG GG AA AG AG
AG GG AA AG AG AG AG AG
GG GG AG AG GG GG AG
31280917
7480 rs10211381 2 AA AG GG AG AG
AG AG GG GG GG AG AA AA
GG GG GG GG AG AG AG AG
GG GG GG GG AG AG AA AG
AG GG AA AG AA AA AA
62722292
7481 rs10211385 2 AA AA AA AA AA
AA AA AA AA AA AA AA AA
AA AA AA AA AA AA AA AA
AA AA AA AA AA AA AA AA
AA AA AA AA AA AA AA
238961997
7482 rs1021140 11 GG GG AG GG GG
GG GG AG GG GG GG GG GG
GG GG GG GG GG AG AG AG
GG GG GG AG GG GG GG GG
AG GG AA AG AG GG AG
19040177
7483 rs10211422 2 CC CC CC CC CC
CC CC CC CC CC CC CC CC
CC CC CC CC CC CC CC CC
CC CC CC CC CC CC CC CC
CC CC CC CC CC CC CC
88991895
7484 rs10211427 2 AG AA AA AA AA
AA AA AA AA AA AA AA AA
AA AA AA AG AG AA AA AA
AA AA AA AA AA AA AA AA
AA AG AA AG AA AA AA
77259215
7485 rs10211429 2 AG AA AG AG AG
GG AG AG AG AG GG GG GG
GG GG AG AG GG AG AG GG
AG AG GG AG AG GG AG AG
GG GG AG AG GG GG GG
12579179
7486 rs1021147 2 AA AA AA CC AC
AC AC AA AA AA AC AC CC
CC CC AA AA AA AA AA AA
AA AC AA AC CC AA AC AC
AA AA AA AA AC AC AA
185952716

7487 rs10211472 2 GG AG GG AG AG
GG AA GG GG GG AA AG AG
AG AG GG GG AG GG GG GG
AG AG AG AG AA AG AA AG
AG GG AG GG AG GG AG
229883422
7488 rs10211505 2 AG GG AG AG AA
AA AG GG AG GG AG GG GG
AG AG AG AG GG AG AG AA
GG AG GG GG AG AA AA AG
GG GG AG GG GG GG AG
88721248
7489 rs10211532 2 GG GG GG GG GG
GG GG GG AG GG GG AG GG
GG GG GG GG GG GG GG GG
GG GG GG GG GG GG GG GG
GG GG GG GG GG GG GG
2894549
7490 rs10211539 2 GG AG AA GG AA
AG AG AG AA AA AG GG AG
GG GG AA AA AA AG AG AG
AG AG AG AG AG GG GG GG
AG GG AG GG AG AA AG
69019549
7491 rs10211565 2 CC AC CC AC AC
CC AA AA AC AA AC AA AA
AC AC CC CC CC CC AC CC
CC CC CC CC AC AA AC AA
AC AA AA AA AA AC AA
142889154
7492 rs10211596 2 AG GG AG GG GG
GG GG GG AA AG AG AG AA
AA AA AG GG GG GG AG GG
AA AA AA AG AG AG AG AA
GG AG AG AA AG AG AA
233699415
7493 rs10211612 2 AA AA AA AA AA
AA AA AA AA AA AA AA AA
AA AA AA AG AG AA AA AA
AA AA AA AA AA AA AA AA
AG AA AG AG AA AA AA
74548807
7494 rs10211630 2 AG GG GG AA AG
AA AG GG GG GG GG GG GG
AG AG GG GG GG AG AG AG
AG AG AG AG AG GG AG AG
GG GG GG GG AA AG AG
58906981
7495 rs10211644 2 GG GG GG GG GG
GG GG GG GG GG GG GG GG

	GG	GG	GG	GG	GG	GG	GG	GG
	GG	GG	GG	GG	GG	GG	GG	GG
	GG	GG	GG	GG	GG	GG	GG	
113767207								
7496	rs10211673		2	AA	AG	GG	AG	AG
	AG	AG	AG	AG	AG	AG	GG	GG
	GG	GG	AG	GG	AG	GG	GG	AG
	AA	AG	AA	AG	GG	AG	AG	GG
	GG	GG	GG	GG	GG	AG	GG	
59320547								
7497	rs10211770		20	AG	AA	AA	GG	AG
	AG	GG	GG	GG	AG	AA	AA	AG
	AG	AA	GG	GG	GG	GG	AA	AG
	GG	AG	GG	AG	AG	GG	AG	GG
	AG	GG	AG	AG	AG	GG	AG	
54396791								
7498	rs10211789		20	GG	AG	GG	GG	GG
	GG	GG	GG	GG	GG	GG	GG	GG
	GG	GG	GG	GG	GG	GG	GG	GG
	GG	GG	GG	GG	AG	AG	AG	GG
	GG	GG	GG	GG	GG	GG	GG	
8537044								
7499	rs10211805		20	AG	AG	GG	AG	GG
	GG	AG	AA	AG	AA	GG	AG	AG
	AG	AG	GG	GG	GG	GG	GG	GG
	GG	AG	GG	AG	GG	GG	GG	GG
	AG	GG	GG	GG	AG	GG	AG	
59960651								
7500	rs10211816		20	GG	GG	GG	GG	GG
	GG	GG	GG	GG	GG	GG	GG	GG
	GG	GG	GG	GG	GG	GG	GG	GG
	GG	GG	GG	GG	GG	GG	GG	GG
	GG	GG	GG	GG	GG	GG	GG	
42114419								
7501	rs1021192		15	AA	AA	AA	AG	AG
	AA	AG	GG	AG	GG	AA	AG	AG
	AG	AG	AA	AA	AA	AG	GG	AA
	AA	AA	AG	AA	AA	AG	AG	AG
	AG	AA	AG	AA	AG	AA	GG	
46714227								
7502	rs10212	11	AA	AG	AG	AA	AG	AG
	AG	AG	AG	AG	AA	AA	AA	AA
	AA	AA	AA	AG	AG	AG	AA	AA
	AA	AA	AG	AA	AG	AA	AG	AA
	AG	AG	AG	AG	AA	AG	60108278	
7503	rs1021201		8	AG	AG	AG	AA	AA
	AA	AG	AG	AG	AG	AG	AG	AA
	AA	AA	AG	AG	AG	AA	GG	AG
	GG	GG	AG	GG	AG	AA	AG	AA
	GG	GG	GG	GG	AG	GG	AA	

20196093
7504 rs10212087 22 GG GG GG GG GG
GG AG GG GG GG GG GG GG
GG GG GG GG GG GG GG GG
GG GG GG GG GG GG GG GG
GG AG AG GG GG GG GG
20171251
7505 rs10212196 3 AA AG AA AG AA
AG AA AA AG AA AG AG GG
GG AG AA AA AA AA AA AG
AG AA AG AG AG GG GG AG
AG AG AA AA AG AA AG
3545416
7506 rs10212208 3 GG GG GG AG GG
GG AG GG AG GG AG AG AG
AA AG AA AA AA AG AG AA
AG GG AG GG GG AG AG GG
AG GG AA AG AG AA AG
166613118
7507 rs1021224 5 AA AC AC AA AC
AA AC AA AC AA AA AA AA
AA AA AA AA AA AA AC AC
AA AA AA AA AC AA AA AA
AC CC CC CC AC AC AC
96837053
7508 rs10212419 3 AG AG AA GG AG
GG AG GG GG AG GG GG GG
AG AG GG AG GG AG GG AG
AG AG AG AG AA GG AG AG
AG AG GG AG GG GG GG
139004920
7509 rs10212452 3 GG GG GG AA AA
AA AA AG AG AA AG AA AG
AG AG AG AG AA AG AG GG
AA AA AG AA AG AG GG GG
AG GG AG AG AA AA AA
120817063
7510 rs10212454 3 GG GG GG GG GG
GG GG GG GG GG GG GG GG
GG GG GG GG GG GG GG GG
GG GG GG GG GG GG GG GG
GG GG GG GG GG GG GG
71942584
7511 rs10212466 3 CC AC CC CC CC
CC AC AA AA AA CC AC AC
AA AA AC AC AC AC AC CC
CC CC CC CC AC AC CC AA
CC AA AC AA AA AA AC
43994607
7512 rs10212574 3 AG AG AG AA AG

	AA	AG	AA	AA	AA	AA	AA	AA
	AA	AA	AA	AA	AA	AA	AA	AA
	AA	AA	AA	AA	AA	AA	AA	AA
	AA	AA	AA	AA	AA	AA	AA	
177639827								
7513	rs1021258		12	AG	AA	AG	GG	GG
	GG	GG	GG	GG	GG	GG	GG	GG
	GG	GG	GG	GG	GG	GG	GG	GG
	GG	GG	GG	GG	GG	GG	GG	GG
	GG	GG	GG	GG	GG	GG	GG	
39132495								
7514	rs10212614		3	GG	GG	GG	GG	GG
	GG	GG	GG	GG	GG	GG	GG	GG
	GG	GG	GG	GG	GG	GG	GG	GG
	GG	GG	GG	GG	GG	GG	GG	GG
	AG	GG	GG	GG	GG	GG	GG	
103831303								
7515	rs10212653		4	AA	AA	AA	AG	AA
	AA	AG	AA	AA	AA	AA	AG	AA
	AA	AG	AG	AA	AG	AA	AA	AA
	AA	AA	AA	AA	AG	AG	AG	AA
	AA	AG	AA	AA	AA	AA	AG	
158898321								
7516	rs1021267		12	AG	GG	AG	GG	GG
	GG	AG	GG	GG	GG	GG	GG	GG
	GG	GG	GG	GG	GG	AG	GG	AG
	AG	AG	AG	AG	GG	GG	GG	GG
	AA	AG	GG	GG	AG	GG	AG	
18876001								
7517	rs1021270		11	AC	AA	AA	AC	CC
	AC	CC	AA	AC	AC	AA	AA	AA
	AA	AA	AC	AA	CC	AC	AC	AC
	CC	AA	AC	AC	CC	AC	CC	AC
	CC	AA	AA	AA	AC	CC	AC	
41232860								
7518	rs10212780		4	AG	AA	AG	AG	AA
	AG	AA	AG	AG	AG	GG	AG	AG
	AA	AA	AG	AA	AG	AA	AA	AA
	AA	AA	AA	AA	AG	AG	AG	AG
	AA	AA	AA	AA	AA	AA	AA	
65475782								
7519	rs10212827		4	GG	GG	--	AA	AA
	AA	AA	GG	AG	AG	GG	GG	GG
	AG	AG	AG	GG	AG	AA	AG	AA
	AG	AG	AG	AG	GG	AG	AG	GG
	AA	GG	AG	AG	AG	AA	GG	
72481753								
7520	rs10212888		4	AA	AA	AG	AG	AG
	AG	GG	AA	AG	AA	AA	AA	AA
	AA	AA	AA	AG	AG	AG	AG	GG

AG AG GG AG AG AA AA AA
AG AG AG AG AA AG AA
184924281
7521 rs1021289 8 AA AA AA AA AA
AA AA AA AA AA AA AA AA
AA AA AA AA AA AA AA AA
AA AA AA AA AA AA AA AA
AA AA AA AA AA AA AA
89982596
7522 rs10212960 4 GG GG AG GG GG
GG GG GG AG GG GG AG GG
GG AG GG AG AG GG AG GG
AG AG AG AG AG GG AG AG
AG GG GG GG GG GG GG
4822309
7523 rs10212997 4 AG AG AG GG GG
AG GG AG AG AG GG GG GG
GG GG GG GG GG AG GG AG
GG GG GG GG AG GG AG AG
GG GG GG GG AG GG AA
5013564
7524 rs10213019 4 AA AA AA AA AA
AA AA AA AA AA AA AA AA
AA AA AA AG AG AA AA AA
AA AA AA AA AG AA AA AG
AG AA AG AA AA AA AA
43147514
7525 rs1021303 3 GG AG GG AA AA
AG AA AG AA AA AA AA AG
AG AA AG AA AA AA AA AA
AA AA AA AA AA AA AA AA
AG AA AA AA AA AA AA
173589827
7526 rs10213076 4 GG GG AG GG GG
GG GG GG GG AG GG GG GG
GG GG AG AG GG AA AA AA
GG AG AG AG AG AG AA AG
AG GG GG GG AG GG AG
47642773
7527 rs10213094 4 GG GG GG GG GG
GG GG GG GG GG GG GG GG
GG GG GG GG GG GG GG GG
GG GG GG GG GG GG GG GG
GG GG GG GG GG GG GG
2441550
7528 rs10213170 4 GG AG GG AA AA
AA AA GG GG AG GG AA AG
AG AG AG AG AG GG GG AG
AG GG GG AG AG GG AG AG
GG GG GG GG GG AG AG

127224641								
7529	rs10213204	4	AG	AA	AA	AA	AA	
	AA	AA	AA	AA	AA	AA	AA	AG
	AG	AG	AA	AA	AA	AA	AA	AA
	AA	AA	AA	AA	GG	AA	AG	AG
	AG	AA	AG	AA	AG	AA	AG	
176601113								
7530	rs10213251	4	GG	GG	GG	AG	AG	
	GG	AG	AG	AG	AG	GG	AA	AG
	GG	GG	GG	AG	GG	GG	AG	AG
	GG	GG	GG	GG	GG	GG	GG	GG
	AG	GG	AG	GG	GG	GG	GG	
103549149								
7531	rs10213271	4	GG	GG	AG	GG	GG	
	GG	GG	AA	AG	AG	AG	AG	AG
	GG	GG	AG	AG	AA	GG	GG	AG
	GG	AG	AG	AG	GG	GG	GG	GG
	GG	GG	GG	GG	GG	GG	AG	
63883185								
7532	rs10213286	4	GG	GG	GG	GG	GG	
	GG	GG	GG	GG	GG	GG	GG	GG
	GG	GG	GG	GG	GG	GG	GG	GG
	GG	GG	GG	GG	GG	GG	GG	GG
	GG	GG	GG	GG	GG	GG	GG	
4861780								
7533	rs10213304	4	GG	GG	AG	GG	GG	
	GG	GG	GG	GG	AG	GG	GG	GG
	GG	AG	GG	GG	GG	AG	AG	AG
	GG	GG	GG	GG	GG	AG	GG	GG
	GG	GG	GG	GG	GG	GG	GG	
39489554								
7534	rs10213323	4	GG	GG	GG	AG	AG	
	GG	AG	GG	GG	GG	GG	GG	GG
	GG	AG	GG	GG	GG	AG	GG	AG
	AG	AG	AG	AG	AG	GG	GG	AG
	AG	GG	AA	AG	GG	AG	GG	
111780482								
7535	rs1021342	11	AA	AA	AA	AA	AA	
	AA	AG	AA	AA	AA	AA	AA	AA
	AA	AA	AG	AG	AG	AA	AA	AA
	AA	AA	AA	AA	AG	AA	AA	AA
	AA	AG	AA	AA	AA	AA	AA	
1650264								
7536	rs10213444	4	AA	AG	GG	AA	AG	
	AG	AG	GG	GG	AG	AA	AA	AA
	AA	AG	GG	GG	GG	GG	GG	GG
	AG	GG	GG	AG	GG	AG	GG	AG
	AG	GG	AG	GG	AG	AG	GG	
87232968								
7537	rs10213461	4	AG	GG	GG	GG	GG	

	GG	AG	AG	AG	GG	AG	GG	AG
	AG	GG	AA	AG	AG	AA	AA	AA
	GG	AG	AG	GG	GG	GG	GG	GG
	AG	AA	AG	AA	AG	AG	GG	
134201194								
7538	rs10213489		4	AA	AA	AA	AG	GG
	AG	AG	AA	AA	AG	AG	AA	AG
	AA	AA	AA	AA	AA	AA	AA	AG
	AG	AG	AG	AG	AG	AA	AA	AG
	AA	AA	AA	AA	AG	AA	AG	
57434245								
7539	rs10213491		4	AA	AG	GG	GG	AG
	AG	GG	GG	GG	GG	AG	AG	AG
	AG	GG	AG	AA	AG	AG	AG	AG
	AG	GG	GG	GG	GG	GG	GG	GG
	AG	GG	GG	GG	AG	AG	AG	
55624059								
7540	rs10213504		4	GG	GG	GG	GG	GG
	GG	AG	AG	AG	GG	GG	GG	GG
	GG	AG	GG	GG	GG	GG	GG	GG
	GG	AG	AG	GG	GG	GG	GG	GG
	GG	GG	GG	GG	GG	AG	AG	
95468433								
7541	rs10213507		4	AC	AA	AA	CC	AC
	AC	AC	CC	AC	CC	AA	AA	AA
	AA	AA	AC	AC	CC	AC	AC	AA
	AC	CC	AC	AA	AC	AA	AA	AA
	AC	AA	AC	AC	AA	AA	AA	
14911772								
7542	rs10213525		4	AA	AA	AA	GG	GG
	GG	GG	GG	GG	AG	AG	AG	AG
	AG	AA	AG	AG	AG	GG	GG	AG
	GG	AG	AG	AA	GG	AG	AG	GG
	AG	AG	AG	AG	AG	GG	AG	
174129740								
7543	rs10213562		4	AA	AA	AC	AA	AA
	AA	AC	AC	AC	AC	AA	AA	AA
	AA	AA	AA	AC	AA	AA	AA	AA
	AA	AC	AC	AC	AC	CC	AC	CC
	AA	AA	AA	AA	AA	AA	AA	
110494732								
7544	rs1021358		1	AG	AG	AG	GG	AG
	AG	GG	GG	GG	GG	AA	GG	AG
	AA	AG	AG	AG	AA	GG	GG	GG
	AG	GG	GG	AG	GG	AG	GG	AG
	GG	AA	AG	AA	AG	AA	AG	
238544006								
7545	rs10213659		4	AA	AA	AA	GG	AG
	AG	GG	AA	AA	AA	AG	AA	AG
	AA	AA	AG	AA	AG	AA	AA	AG

AG AA AA AA AA AA AA AA
AA AG AA AA AG AA GG
57039609
7546 rs10213765 5 AG AG AG GG GG
GG GG GG AG GG GG GG GG
GG GG GG GG GG GG GG GG
GG GG AG GG GG AG AG GG
GG GG GG GG GG GG AG
16848200
7547 rs1021378 1 AA AG AA GG AG
AG GG AG GG AG AG AA AG
AG AG AA AG AG AG AG AG
GG AG AG AG AG AG AG AG
AA AG AG AA GG GG GG
212700618
7548 rs10213801 5 AA AA AG AA AA
AA AG AG AG AG AG GG AG
AG AG AA AA AA GG GG GG
AG AG AG AG AG AA AG AG
GG AG AA AA AG AG AA
90249900
7549 rs1021381 4 GG GG AG AA AA
AA AA AG AA AG AA AG AA
AA GG GG GG GG AG AA GG
AA AA AA AA AG GG AG AG
AA AG AA AG AA AA AG
155257709
7550 rs10213903 5 GG GG GG GG GG
GG GG GG GG GG GG GG GG
GG GG GG GG GG GG GG GG
GG GG GG GG GG GG GG GG
GG GG GG GG GG GG GG
56783042
7551 rs10213918 5 AG AG AG GG GG
AG GG GG GG GG AG AA AG
GG GG AG GG GG AA AA AA
GG GG GG GG AG GG GG AG
GG AG GG GG GG GG GG
124963890
7552 rs10213971 5 CC CC AC AC AC
CC AA AC AC AC AA AA AC
AA AC CC CC CC AC CC CC
CC CC CC CC AC AC AC CC
AC CC CC CC AC CC AA
27532751
7553 rs10214010 5 GG GG GG GG GG
GG GG GG GG GG GG AG AG
AG GG GG GG GG GG GG GG
GG GG GG GG GG GG GG GG
GG GG GG GG GG GG GG

51598623
7554 rs10214013 5 AA AA AA AA AA
AA AA AA AA AA AA AA AA
AA AA AA AA AA AA AA AA
AA AA AA AA AA AA AA AA
AA AA AA AA AA AA AA
41783432
7555 rs10214074 5 GG GG GG GG GG
GG GG GG GG GG AG GG GG
AG AG GG GG GG GG GG GG
GG GG AG GG GG GG GG GG
GG GG AG AG GG GG GG
24192188
7556 rs10214089 5 GG GG GG GG GG
GG GG GG GG GG GG GG GG
GG GG GG GG GG GG GG GG
GG GG GG GG GG GG GG GG
GG GG GG GG GG GG GG
37732735
7557 rs10214122 5 AA AA AA AA AA
AA AA AA AA AA AA AA AA
AA AA AA AA AA AA AA AA
AA AA AA AA AA AA AA AA
AA AA AA AA AA AA AA
14683609
7558 rs10214135 5 AA AA AA AA AA
AA AA AG AG AG AA AA AA
AA AA AA AA AA AG AG AG
AA AA AA AA AA AG AG AA
AA AG AA AA AA AA AA
38366662
7559 rs10214163 5 AG AG AA AG AG
AG AA AG AG GG AG AA AA
AA AA AA AG AA AG AA AG
AA AA AA AA AG AG GG AA
AG AA AA AA AA AA AA
75294410
7560 rs1021420
4 -- -- -- -- -- -- -- --
-- -- -- -- -- -- -- --
-- -- -- -- -- -- -- --
-- -- -- -- -- -- -- --
-- -- -- -- 108158105
7561 rs10214211 5 AA AA AA AG AG
AG AA AG AG AG AA AA AA
AA AA AA AA AA AA AA AA
AG AG AG AG AA AA AA AA
AA AA AA AA AA AA AG
159400511
7562 rs10214212 5 AG AG AG AA AA

	AA	AA	GG	GG	GG	AA	AA	AA
	GG	AG	AA	AG	AG	AG	AA	AA
	AA	AG	AA	AA	GG	AG	AG	AG
	AG	AG	AA	AA	AA	AG	AG	
167607224								
7563	rs10214226		5	AA	AA	AA	AA	AA
	AA	AA	AA	AA	AA	AC	AA	AA
	AA	AA	AA	AA	AA	AA	AA	AA
	AA	AA	AA	AA	AA	AA	AA	AA
	AA	AA	AA	AA	AA	AA	AA	
36971490								
7564	rs10214230		5	AA	AA	AA	AA	AA
	AA	AA	AA	AA	AA	AA	AA	AA
	AA	AA	AA	AA	AA	AA	AA	AA
	AA	AA	AA	AA	AA	AA	AA	AA
	AA	AA	AA	AA	AA	AA	AC	
18640685								
7565	rs10214231		5	GG	GG	GG	GG	GG
	GG	GG	GG	GG	GG	GG	GG	GG
	GG	GG	GG	GG	GG	GG	GG	GG
	GG	GG	GG	GG	GG	GG	GG	GG
	GG	GG	GG	GG	GG	GG	GG	
57590049								
7566	rs10214244		5	AA	AA	AA	AA	AA
	AA	AA	AA	AA	AA	AA	AA	AA
	AA	AA	AA	AA	AA	AA	AA	AA
	AA	AA	AA	AA	AA	AA	AA	AA
	AA	AA	AA	AA	AA	AA	AA	
38300133								
7567	rs10214261		5	GG	GG	GG	GG	GG
	GG	GG	GG	GG	GG	GG	GG	GG
	GG	GG	GG	GG	GG	GG	GG	GG
	GG	GG	GG	GG	GG	GG	GG	GG
	GG	GG	GG	GG	GG	GG	GG	
31592666								
7568	rs10214269		5	GG	GG	GG	GG	GG
	GG	GG	GG	GG	GG	GG	GG	GG
	GG	GG	GG	GG	GG	GG	GG	GG
	GG	GG	GG	GG	GG	GG	GG	GG
	GG	GG	GG	GG	GG	GG	GG	
124561232								
7569	rs10214273		5	AA	AC	AA	AA	AA
	AC	AA	AA	AA	AA	AA	AC	AC
	AA	AC	AC	AA	AC	AA	AA	AA
	AA	AA	AA	AA	AA	AA	AA	AA
	CC	AA	CC	AC	AA	AA	AA	
35883986								
7570	rs10214275		5	AA	AA	AA	AA	AA
	AA	AA	AA	AA	AA	AA	AA	AA
	AA	AA	AA	AA	AA	AA	AA	AA

AA AA AA AA AA AA AA AA
AA AA AA AA AA AA AA
112343005
7571 rs10214302 5 GG GG GG GG GG
GG GG GG GG GG GG GG GG
GG GG AG GG AG GG GG GG
GG GG GG GG GG GG GG GG
AG GG GG GG GG GG GG
148238456
7572 rs10214306 5 AC CC CC AC CC
AC CC AC CC AC CC CC CC
CC CC CC CC CC AC AC AC
CC CC CC CC CC AC CC AC
AA CC CC CC CC CC CC
24279987
7573 rs10214316 5 AG AA AG AG AG
AA AG AA AA AG AA AA AA
AG AG AA AA AA AA AA AA
AA AA AA AA AA AG AA AG
AA AA AG AG AG AA AG
55444040
7574 rs10214334 5 AA AA AA AA AA
AG AA AA AA AA AG GG AG
AA AA AG AA AA GG GG GG
AA AA AA AA AG AA AA AG
AA AG AA AA AA AA AA
124964532
7575 rs1021441 9 AC AC AC AA CC
AC AC AC CC AC AC AC AC
AC AA AA AC AC AC AA AC
AC CC AC CC AC AA AA AA
AC AC AC AC AC AC AC
94379459
7576 rs10214423 6 AA AA AA AA AA
AA AA AA AA AA AA AA AA
AA AA AA AA AA AA AA AA
AG AG AA AA AA AA AA AA
AA AA AA AA AG AG AA
164673314
7577 rs10214441 6 AG AG GG AG GG
AG GG AG AG AG AG AA AG
AA AG GG GG GG AA AA AA
GG GG GG GG AG AA AA AA
AG AG AA AG AA AG AA
67658860
7578 rs10214453 6 AA AA AA AA AA
AA AA AA AA AA AA AA AA
AA AA AA AA AA AA AA AA
AA AA AA AA AA AA AA AA
AA AA AA AA AA AA AA

52759791
7579 rs10214456 6 AA AA AA AA AA
AA AG AA AG AA AA AA AG
AG AG AG AG AG AG AA AA
AG AA AG AA AA AG AA AA
AA GG AG AG AG AG AA
6592095
7580 rs10214475 6 AG AG AG AG AG
AA GG AG AA AG AA AA AA
AG AA AA AA AG AG AG AG
AG AA AG AA AA AA AA AA
AA AA AG AA AG AA AG
6903148
7581 rs10214692 6 AG AA AG AG GG
AG GG GG GG GG AA AA AG
AA AG AG AA AA AG AG GG
AA AA AA AG GG AG GG GG
AA GG AG AG AA AA AA
25080685
7582 rs10214711 6 AA AA AA AA AA
AA AA AA AA AA AA AA AA
AA AA AA AA AA AA AA AA
AA AA AA AA AA AA AA AA
AA AA AA AA AA AA AA
51300402
7583 rs1021475 17 AA AA AA AA AA
AA AA AA AA AA AA AA AA
AA AA AA AA AA AA AA AA
AA AA AA AA AA AA AA AA
AA AA AA AA AA AA AA
30995042
7584 rs1021481 16 AG AG GG AG AG
AG GG GG GG GG GG GG GG
GG GG GG GG GG GG AG GG
GG GG GG GG GG AA AG AG
GG GG GG GG AG AG GG
85959020
7585 rs10214819 6 AC AC AA AA AA
AA AA AA AA AA AA AA AA
AA AA AC AC AC AA AA AA
AC AC AA AC AA AA AA AA
AA AA AA AA AA AA AA
107592142
7586 rs1021493 9 GG GG GG GG GG
GG GG GG GG GG GG GG GG
GG GG AG AA AA GG AG GG
GG GG GG GG GG AG AG GG
GG AG AG AG GG GG GG
14848892
7587 rs10214949 7 AG AG AA AA AG

AG AA AA AA AG AG AA AG
AG AA GG AG AG AA AA AA
AA AA AA AA AG AA AA AG
AG AG AA AG AG AG AA
79048593
7588 rs10214977 7 AC AA AC CC AC
CC AC AC AC AA AC AC CC
CC CC CC CC CC AA CC AC
CC CC CC CC CC AC AC AC
AC AC CC CC CC AC CC
94727327
7589 rs10215028 7 AA AA AA AA AA
AA AA AA AA AA AA AA AA
AA AA AA AA AA AA AA AA
AA AA AA AA AA AA AA AA
AA AC AA AA AA AA AA
14436385
7590 rs10215031 7 GG AG AG AG GG
AG GG AA AG AG AA AG AG
GG GG AG AG GG AG AG AG
AA GG AG GG GG AG AG AG
AA AA AG AA AG AG AG
4024024
7591 rs10215076 7 AA AA AA AA AA
AA AA AA AA AA AA AA AA
AA AA AA AA AA AA AA AA
AA AA AA AA AA AA AA AA
AA AA AA AA AA AA AA
51012008
7592 rs10215085 7 AA AA AA AA AA
AA AA AA AA AA AA AA AA
AA AA AA AA AA AA AA AA
AA AG AA AA AG AA AA AA
AA AA AA AA AA AA AA
140812939
7593 rs10215108 7 AA AA AA GG AG
AG GG AG AG AG AG AG AG
AG GG GG GG GG GG GG GG
GG GG GG GG AG AG GG GG
GG AG AG AG AG AG AG
10930439
7594 rs10215130 7 CC AC AC AA AA
AA AA AA AA AA AC AA AC
AC AC AA AA AA AA AA AA
AA AA AA AA AA AA AA AA
AA AC AA AA AA AA AA
64941453
7595 rs10215134 7 AG AG GG AA AG
AG AA AG AG AG AA AA AA
AG AG AA AG AG AG AG AG

GG AG AG GG AG AG AG GG
AG AG AG AG AA AG AA
48194935
7596 rs1021516 5 CC CC CC AC AC
AC AA CC CC CC AC AA AC
AA AA CC CC CC CC AA CC
AC AA AA AA CC CC CC CC
AA AA AA AA AC AC AC
116544172
7597 rs1021517 5 AA AA AA AG AA
AA AG AA AA AA AG AG AG
AG AG AA AA AA AA AA AA
AA AG AG AG AA AA AA AA
AA AA AG AG AA AA AA
116574744
7598 rs10215172 7 AG AG AA GG GG
GG GG AA AG AA AG GG AG
GG GG AG AG AG AG AG AG
GG AA GG GG AG AA AA AA
AA AG AG GG AA AA AA
151588043
7599 rs10215222 7 AG AG AA AG AG
GG AG AG AG GG GG GG GG
GG GG GG GG GG AG AG AG
GG GG GG GG AG GG GG GG
GG GG GG GG GG GG GG
155741450
7600 rs10215277 7 GG GG AG GG AG
AG GG AA AG AA GG GG GG
GG GG GG AG AG GG GG GG
AG GG AG AG GG GG GG GG
GG GG AA AG GG GG GG
141845717
7601 rs10215358 7 AA AA AG AG AG
GG AA GG AG GG AG AA AG
AG GG AG AA AA AG AG AA
AA AG AA AA GG AA AG AG
AG AA AG AA AA AG AG
131363167
7602 rs10215367 7 AG AG AA GG GG
GG AG AG AA AG AA AA AA
AA AA AA AG AG AA AA AA
AG AA AG AG AG GG AG AG
AG AA AG AA AA AA AA
132945754
7603 rs10215395 7 GG GG AG AG AG
AG AG AG AG AG GG AG AG
GG GG GG GG GG GG AG GG
GG GG GG GG GG AG GG GG
AA GG AG AG GG GG GG

47358377
7604 rs10215514 7 AA AA AA AG AA
AA AG AG AG AA AA AA AA
AA AA AG AA AG AA AA AA
AA AA AA AA AA AG AA AA
AA AG AA AA AA AA AA
15486094
7605 rs10215532 7 AG GG AG AG GG
AG AG AA AA AG GG AA AG
AG AA AG GG AG AG GG AA
AG AG AG AG AA AG AA AA
GG AG AA AG AG AG GG
12813460
7606 rs10215624 7 AA AA AA AA AA
AA AA AA AA AA AA AA AA
AA AA AA AA AA AA AA AA
AA AA AA AA AA AA AA AA
AA AA AA AA AA AA AA
95835678
7607 rs10215689 7 AA AA AA AA AA
AA AA AG AG AA AA AA AA
AA AA AA AA AA AA AA AA
AA AA AA AA AA AA AA AA
AG AA AG AA AA AA AG
51907832
7608 rs10215692 7 AA AA AA AA AA
AA AA AA AA AA AA AA AA
AA AA AA AA AG AA AA AA
AA AA AA AA AG AA AG AA
AA AA AG AG AA AA AG
24856032
7609 rs10215771 7 AG AA AG AG GG
AG GG AA AA AA AG AA AG
GG AG AA AA AA AG AA AG
AA AA AA AA AA AA AA AA
AG AG AA AA AG GG AA
70638282
7610 rs10215793 7 GG GG GG AG AG
GG AG AG AG AG GG GG GG
GG GG AG GG GG GG AG AG
GG GG GG GG AG GG AG AG
GG AG GG AG AG AG AG
124008463
7611 rs10215830 7 GG GG GG GG GG
GG GG GG GG GG GG GG GG
GG GG GG GG GG GG GG GG
GG GG GG GG GG GG GG GG
GG GG GG GG GG GG GG
101796783
7612 rs10215856 7 AA AA AA AA AA

	AA	AA	AA	AA	AA	AA	AA	AA
	AA	AA	AA	AA	AA	AA	AA	AA
	AA	AA	AA	AA	AA	AA	AA	--
	AA	AA	AA	AA	AA	AA	AA	
6339313								
7613	rs10215877		7	AG	AG	AG	AA	AA
	AA	AG	AA	AA	AA	AA	AG	AG
	AG	GG	AA	AA	AA	AA	AA	AG
	AA	AA	AG	AG	AG	AA	AG	AA
	AA	AG	AA	AG	AA	AA	AG	
76429959								
7614	rs10215904		7	GG	GG	GG	GG	AG
	GG	AG	GG	GG	GG	GG	GG	AG
	GG	AG	GG	GG	GG	GG	GG	GG
	GG	AG	GG	AG	GG	GG	GG	GG
	GG	AG	GG	GG	GG	GG	GG	
2449733								
7615	rs10215927		7	AG	AA	AG	AG	AA
	AG	AA	GG	GG	GG	AG	AA	AG
	AG	AG	AA	AA	AA	AA	AG	AA
	GG	AG	AA	AG	AG	AG	GG	AG
	GG	AG	GG	AG	AG	AG	AA	
80945405								
7616	rs1021593		16	AA	AA	AA	AG	AA
	AA	AG	AG	GG	AA	AA	AA	AA
	AG	AA	AG	AA	AG	AG	AG	AA
	AG	GG	GG	AG	AA	AA	AA	AA
	AG	AG	AG	AG	AG	AA	GG	
76899703								
7617	rs10215963							
7	--	--	--	--	--	--	--	--
	--	--	--	--	--	--	--	--
	--	--	--	--	--	--	--	--
	--	--	--	--	--	--	--	--
	--	--	--	--	26583781			
7618	rs1021598		18	AA	AA	AA	AA	AA
	AA	AA	AA	AA	AA	AA	AA	AA
	AA	AA	AG	AG	AA	AG	AG	GG
	AA	AA	AA	AA	AA	AA	AA	AA
	AA	AG	AG	AG	GG	AG	AG	
30215152								
7619	rs10215999		7	AG	AG	AG	GG	AG
	AG	GG	GG	GG	GG	GG	AG	AG
	GG	GG	GG	GG	GG	AG	AG	GG
	AG	GG	GG	GG	GG	GG	GG	GG
	AG	GG	GG	GG	AA	AA	AG	
13673538								
7620	rs10216009		7	AG	AG	AA	AG	AA
	AG	AA	AG	AA	AA	AA	AA	AA
	AG	AG	AA	AA	AG	AG	AG	AA

AG AA AA AA AG AA AG AG
AA AA GG AG AA AA AA
3598581
7621 rs10216024 7 AA AA AG AA AA
AA AA AA AA AA AA AA AA
AA AA AA AA AA GG AG GG
AA AG AA AA AA AA AA AA
GG AA AA AA AA AA AA
142198547
7622 rs10216044 7 GG AG GG GG GG
GG GG AG AG AG GG GG GG
AA AA GG AG GG GG GG AG
GG AA AG AG AG AG AG AA
AA GG GG GG GG GG AG
41812483
7623 rs10216133 7 GG AG AG AG AA
AA AG GG GG AG AG GG AG
GG GG AG AG GG AA AG AG
GG GG GG GG AG AA AA AG
AA AA AG AA AG AG AG
57238635
7624 rs10216141 7 GG GG GG GG AG
GG AG GG GG GG GG GG GG
GG GG GG GG GG GG GG GG
GG GG GG GG GG GG GG GG
GG GG GG GG GG GG GG
19532215
7625 rs10216184 7 AA AA AA AG AG
AA GG GG GG AG AA AG AG
AG AG AA AA AA AG AA AG
GG GG GG GG AG AA AG AA
AG AA GG AG AG AG AA
80262830
7626 rs10216186 7 CC CC CC CC AC
AC CC CC CC AC AC AC CC
CC AC CC CC CC CC CC CC
AC AC CC CC CC CC CC CC
CC CC CC CC AC AC CC
69395168
7627 rs10216189 7 AA AA AA AA GG
AG AG AG AG AG AA AG AA
AA AA AA AG AG GG GG AG
GG AG GG AG AG AA AA AA
AA GG AA AG AG AA AG
5524805
7628 rs10216225 7 AG AG AA AA GG
AG AG GG AG GG AA AA AA
AG AG AG AG AG AA GG AA
GG AG GG AG GG GG GG GG
AG AG AG AG GG AG GG

28941454
7629 rs10216298 7 AA AA AA AA AA
AA AA AG AA AG AA AA AA
AA AA AA AA AG AA AA AA
AA AA AA AA AA AA AA AA
AA AA AA AA AA AA AG
48665024
7630 rs10216324 7 AA AC AA AC AC
AC AC AC AC CC AC AC AA
AA AA AA AA AC AC AC AC
AA AA AA AA AA AA AA AA
AA AC AA AA AC AC AA
15369119
7631 rs10216388 8 AC AC AC CC CC
AC AC AC AC AC AC CC AC
AC AC AA AC AC AA AA AC
AC AA AC AA AC AA AC AA
AA AC CC AC AC AC AA
18844382
7632 rs10216403 8 GG AG GG GG GG
GG GG GG GG GG AG GG GG
GG AG AG GG AG GG GG GG
AG AG AG AG GG GG GG GG
GG AG GG AG GG AG GG
27607934
7633 rs10216426 8 AG GG AG GG GG
AG GG GG GG GG GG AG GG
GG GG GG GG GG GG GG GG
AG GG AG GG GG GG GG GG
GG GG GG GG GG GG GG
14875723
7634 rs10216529 8 AG AG AA AG AG
GG AA GG GG GG AG AG AA
AA AG GG AG GG AA AA AA
AG AA AG AA AG AA AA AG
AG GG AG GG AG AA GG
133733145
7635 rs10216585 8 AG AG AG AA AA
AA AA AG AA AA AA AA AA
AG GG AA AA AA AA AA AG
AG AA AA AG AA AG AA AA
GG AG AG GG AA AG AA
49071490
7636 rs10216603 8 GG AG AA AG AG
AG AG AG AG AA AA AA AA
AA AG AG AG AA AA AG AA
AA AA AA AA AA AA AA AA
AA AA AG AG AA AA AA
30636140
7637 rs10216611 8 CC CC CC AC CC

	--	CC	CC	CC	CC	CC	CC	CC
	CC	CC	CC	CC	CC	CC	CC	CC
	CC	CC	CC	CC	CC	CC	CC	CC
	CC	CC	CC	CC	CC	CC	CC	
113281792								
7638	rs1021662		3	AG	AG	AA	AG	AA
	AG	AG	GG	AG	AA	AG	AG	AG
	GG	GG	AA	AG	AA	AG	AG	AG
	AA	AA	AG	AG	AA	AA	AA	AA
	AG	AG	AG	AG	GG	AG	GG	
192727562								
7639	rs10216636		8	AG	AA	AG	AA	AA
	AA	AG	AA	AA	AA	AA	AA	AA
	AA	AA	AA	AA	AA	AA	AA	AA
	AA	AA	AA	AA	AA	AA	AA	AA
	AA	AA	AG	AA	AA	AA	AG	
2973634								
7640	rs10216665		8	GG	GG	GG	GG	GG
	GG	GG	GG	GG	GG	GG	GG	GG
	GG	GG	GG	GG	GG	GG	GG	GG
	GG	AG	GG	AG	AG	GG	AG	AG
	GG	GG	GG	GG	GG	GG	GG	
3695148								
7641	rs10216697		8	AG	AA	AG	AA	AA
	AA	AA	AA	AG	GG	AA	AG	AG
	AG	AG	AA	AA	AG	AA	AG	AG
	AG	AG	GG	GG	GG	AG	AG	AG
	GG	AG	AG	GG	GG	AG	GG	
74299041								
7642	rs10216757		8	GG	GG	GG	GG	GG
	GG	GG	GG	GG	GG	GG	GG	GG
	GG	GG	GG	AG	GG	GG	GG	GG
	GG	GG	GG	GG	GG	GG	GG	GG
	GG	GG	GG	GG	GG	GG	GG	
127836529								
7643	rs10216796		8	AA	AA	AA	AA	AA
	AA	AA	AA	AA	AA	AA	AA	AA
	AA	AA	AA	AA	AA	AA	AA	AA
	AA	AA	AA	AA	AA	AA	AA	AA
	AA	AA	AA	AA	AA	AA	AA	
95478445								
7644	rs10216814		8	AA	AA	AG	AG	AA
	AG	AA	AA	AA	AA	AA	AA	AA
	AA	AA	AA	AA	AA	AA	AA	AA
	AA	AA	AA	AA	AG	AA	AG	AA
	AA	AG	AA	AA	AA	AA	AA	
123172675								
7645	rs10216841		8	AG	AG	AG	AG	GG
	AG	GG	AG	AA	AG	AG	GG	AG
	AA	AA	GG	AG	AG	AG	AG	AG

GG AG AG AA GG AG AG AG
GG GG AG GG AA AG AA
141142549
7646 rs10216846 8 AG GG AG AG AG
GG AA AA AG AG AG AG AG
AG AA AA AG AG GG GG AG
AA AA AG AA AG AG AG AA
AG AA GG AG AA AA AG
22661286
7647 rs10216876 8 GG GG AG AG AG
GG AG GG GG GG GG GG AG
AG AG GG GG GG GG GG GG
GG GG GG GG AG GG GG GG
AG AG GG GG AG AA GG
140274384
7648 rs10216900 8 AC CC AC CC CC
AC CC AA AC AC AA AC AC
AC AC AC CC AC AC AC AC
AC CC CC CC AA CC AC AC
AC AA AA AA CC CC CC
90476925
7649 rs10216950 8 GG AG GG AG AA
AA AG AG AA AA AA AG AG
AG AG AG AG AA AG AA AA
AA AA AA AA AA AA AA AA
AA AA AA AA AA AA AA
75902603
7650 rs10216960 8 GG GG GG AG AA
AA AG AA AG AG AG AG GG
GG GG GG AG GG AG AG GG
AG AA AG AG AA AG AA AA
GG AG AG AG GG AG GG
82939088
7651 rs10216980 8 GG GG GG GG GG
GG GG GG GG AG AG GG AG
GG GG GG GG GG GG AG GG
AG GG GG AG GG GG GG GG
AG AG GG GG GG GG AG
3044961
7652 rs10216983 8 GG GG GG GG GG
GG GG GG GG GG GG GG GG
GG GG GG GG GG GG GG GG
GG GG GG GG GG GG GG GG
GG GG GG GG GG GG GG
125983513
7653 rs1021701 3 AA AA AA GG GG
GG AG GG GG AA AA AG AG
AG AG AA AG AG AA AA AA
AA AA AA AA AG AA AA AA
AG AA GG AG AA AA AA

25058757							
7654	rs10217013	8	CC	AC	CC	AC	AC
	AC	CC	AC	CC	CC	AA	AA	AA
	AA	AA	CC	AC	CC	AA	AA	AC
	CC	CC	CC	CC	AC	AC	AA	AA
	AC	CC	AC	AC	CC	CC	AC	
64878574								
7655	rs1021702	3	GG	AG	GG	AA	AA
	AA	AA	AA	AA	GG	AG	AG	AG
	AG	AG	AA	AA	AA	AG	GG	GG
	GG	AG	GG	AG	AG	AG	GG	AG
	AG	AG	AA	AG	GG	AG	GG	
25059227								
7656	rs10217030	8	AG	AG	AA	AA	AA
	AA	AA	AA	AA	AA	AA	AA	AA
	AA	AA	AA	AA	AG	AA	AA	AA
	AA	AA	AA	AA	AA	AA	AA	AA
	AA	AA	AA	AA	AG	AG	AA	
32679661								
7657	rs10217063	8	AG	AG	GG	GG	AG
	GG	AG	AG	GG	GG	GG	GG	GG
	GG	GG	GG	AG	GG	AG	AG	AG
	GG	GG	GG	GG	AG	AG	AA	AG
	AG	GG	GG	GG	AG	GG	AG	
94013592								
7658	rs1021711	5	AA	AC	AC	AC	AC
	CC	AA	AC	AC	AC	AC	CC	CC
	AA	AC	AA	AC	AC	AC	AA	AC
	AC	AC	AC	AC	AC	AC	CC	AC
	AA	CC	AC	CC	AC	CC	AA	
30984299								
7659	rs10217130	9	AA	AA	AA	AA	AA
	AA	AA	AA	AA	AA	AA	AA	AA
	AA	AA	AA	AA	AA	AA	AA	AA
	AA	AA	AA	AA	AA	AA	AA	AA
	AA	AA	AA	AA	AA	AA	AA	
33344043								
7660	rs1021714	5	AG	AG	GG	GG	GG
	GG	GG	GG	GG	GG	GG	GG	AG
	GG	AG	GG	AG	AG	GG	GG	GG
	GG	GG	GG	GG	GG	GG	GG	GG
	AG	GG	AG	AG	GG	GG	GG	
30906632								
7661	rs10217208	9	GG	GG	GG	GG	AG
	GG	AG	GG	AG	GG	AG	AG	GG
	GG	AG	GG	GG	GG	GG	GG	GG
	GG	GG	GG	GG	AG	AG	GG	AG
	GG	GG	AG	GG	GG	GG	GG	
88200133								
7662	rs1021730	3	AA	AA	AA	AA	AA

AA AA AA AA AA AA AA AA
AA AA AA AA AA AA AA AA
AA AA AA AA AA AA AA AA
AA AA AA AA AA AA AA
96869125
7663 rs10217342 9 AA AG AA AG AG
GG AA AA AA AA AG AG GG
GG AG AG AG AG AA AA AA
AG AG AG AG AG GG GG AG
AG AG AG AG AG AA AG
79561644
7664 rs10217345 9 GG GG GG GG GG
GG GG GG GG GG GG GG GG
GG GG GG GG GG GG GG GG
GG GG GG GG GG GG GG GG
GG GG GG GG GG GG GG
84077767
7665 rs10217388 9 GG GG GG GG GG
GG GG GG GG GG AG GG GG
GG GG GG GG GG GG GG GG
GG GG GG GG GG GG GG GG
GG AG GG AG GG GG GG
105797021
7666 rs10217389 9 AA AA AA AA AA
AA AA AA AA AA AA AA AA
AA AA AG AA AG AA AA AA
AA AA AA AA AA AG AA AA
AA AA AA AA AA AA AA
84528161
7667 rs10217418 9 AA AG AA AA AA
AA AA AA AA AA AA AA AA
AA AA AG AG AG AA AA AG
AA AA AA AA AA AA AA AA
AA AA AA AA AA AA AA
82335240
7668 rs10217447 9 AA AA AA AA AA
AA AA AA AA AA AA AG AA
AA AA AG AG AA AG GG AG
AG AA AG AA AA AA AA AA
AA AG AA AG AA AA AA
8554938
7669 rs10217478 9 GG GG GG AA AG
AG AG AG AG AA AG AG AG
AG AG GG GG GG AG AG AA
AG AG AG AG GG GG GG GG
AA GG AG GG GG GG AG
72512898
7670 rs1021752 3 GG AG AG GG GG
GG GG GG GG GG AG AG AA
GG AG GG GG GG AG AG GG

	GG	GG	GG	GG	GG	GG	GG	GG
	GG	GG	GG	GG	AG	GG	AG	
134996955								
7671	rs10217530		9	AA	AA	AA	CC	AC
	CC	AC	AC	AC	AC	AC	AA	AC
	AC	AA	AC	AA	AA	AA	AA	AA
	AC	AA	AC	AA	AA	AA	AA	AA
	AC	AC	AA	AA	AA	AC	AA	
80839235								
7672	rs10217550		9	AA	AA	AA	AA	AA
	AA	AA	AC	AC	AA	AA	AA	AA
	AA	AA	AC	AA	AA	AC	AC	AC
	AA	AA	AA	AA	AA	AA	AA	AA
	AC	AA	AA	AA	AC	CC	AC	
79561569								
7673	rs10217557		9	GG	GG	GG	GG	GG
	GG	GG	GG	GG	GG	GG	GG	GG
	GG	GG	GG	GG	GG	GG	GG	GG
	GG	GG	GG	GG	GG	GG	GG	GG
	GG	GG	GG	GG	GG	GG	GG	
76455915								
7674	rs10217567		9	AG	AG	AG	GG	GG
	GG	GG	GG	GG	GG	AG	AG	AA
	AA	AG	GG	GG	GG	AG	AG	AG
	GG	GG	AG	GG	AG	AG	AG	AG
	GG	AG	AG	GG	AG	AG	AG	
112751649								
7675	rs10217619		9	AA	AA	AA	AA	AA
	AA	AA	AA	AA	AA	AG	AA	AA
	AA	AA	AA	AA	AA	AA	AA	AG
	AA	AA	AA	AA	AA	AG	AG	AG
	AA	AA	AA	AA	AA	AG	AA	
27316971								
7676	rs10217639		9	AG	AA	AG	AA	AG
	AG	AG	AG	AA	GG	AG	AG	AG
	AG	AG	AG	AG	AG	AG	AG	AG
	AG	GG	AG	GG	AA	AA	AA	AA
	AG	AG	AG	GG	AA	AA	AG	
105566691								
7677	rs10217653		9	AG	AG	AG	AG	AG
	GG	AG	GG	GG	GG	GG	GG	GG
	GG	GG	GG	GG	GG	AG	AG	AG
	GG	GG	GG	GG	AG	GG	GG	GG
	AG	GG	GG	GG	AG	AG	AG	
106693503								
7678	rs10217656		9	AA	AA	AA	AA	AA
	AA	AA	AA	AA	AA	AA	AA	AA
	AA	AA	AA	AA	AA	AA	AA	AA
	AA	AA	AA	AA	AA	AA	AA	AA
	AA	AA	AA	AA	AA	AA	AA	

71796161
7679 rs10217667 9 AG AA AG AA AA
AA AG AA AA AG AA AG AG
AG AG AG AG AA AA AG AG
AA AG AA AG AA AA AA AA
AG AA AA AA AA AA AG
13902048
7680 rs10217669 9 GG GG GG GG GG
GG GG AG GG AG AG GG AG
AG GG GG GG GG AG AG AA
GG GG GG GG GG GG GG GG
AG GG GG GG GG GG GG
86042009
7681 rs10217675 9 CC CC CC CC CC
CC CC CC CC CC CC CC CC
CC CC CC CC AC CC CC CC
CC CC CC CC CC CC CC CC
AC CC CC CC CC CC CC
13615461
7682 rs1021769 5 AG AG AG GG AG
GG AG AG AG AG AG AG AG
AG AG GG AG AG AG GG AG
GG GG GG GG GG AG AG GG
GG AA GG AG AG GG AA
123192434
7683 rs1021771 5 AA AC CC AA AA
AA AA AC AA AC AA AA AA
AA AA AA AA AA AC AA AA
AA AA AA AA AC AA AA AA
AA AA AA AA AA AC AA
18795292
7684 rs10217719 9 AG AG GG AG AG
AA GG AG AA AA GG AG AG
GG AG GG GG GG GG AG AG
AA AG AA AG AG GG AG GG
GG GG GG GG AG AG GG
422157
7685 rs10217737 9 AA AA AA AA AG
AA AG AA AA AA AA AA AA
AA AA AA AA AA AA AA AA
AG AA AG AA AA AA AA AA
AA AA AA AA AA AA AA
97833775
7686 rs10217741 9 AC AA AC AC CC
AC AC AC AC AA AA AC AA
AA AA AC CC CC AA AA AC
AC AA AC AA AC AC AC CC
AC AC AA AC AC AC AA
77112159
7687 rs10217743 9 AG AG AG AA AA

	AA	AA	AA	AA	AA	AG	AG	GG
	GG	GG	AG	AA	AG	GG	GG	GG
	AG	AG	GG	AG	AG	GG	GG	GG
	AG	AG	AG	AA	AG	AG	GG	
112751482								
7688	rs10217843		X	GG	GG	GG	GG	GG
	GG	GG	GG	GG	GG	GG	GG	GG
	GG	GG	GG	GG	GG	GG	GG	GG
	GG	GG	GG	GG	GG	GG	GG	GG
	GG	GG	GG	GG	GG	GG	GG	
12604438								
7689	rs10217864		X	GG	GG	GG	GG	GG
	GG	GG	GG	GG	GG	GG	GG	GG
	GG	GG	GG	GG	GG	GG	GG	GG
	GG	GG	GG	GG	GG	GG	GG	GG
	GG	GG	GG	GG	GG	GG	GG	
136920738								
7690	rs10217875		X	AA	AA	AA	AA	AA
	AA	AA	AA	AA	AA	AA	AA	AA
	AA	AA	AA	AA	AA	AA	AA	AA
	AA	AA	AA	AA	AA	AA	AA	AA
	AA	AA	AA	AA	AA	AA	AA	
31201876								
7691	rs10217960		X	AA	AA	AA	AA	AA
	AA	AA	AA	AA	AA	AA	AA	AA
	AA	AA	AA	AA	AA	AA	AA	AA
	AA	AA	AA	AA	AA	AA	AA	AA
	AA	AA	AA	AA	AA	AA	AA	
154177977								
7692	rs10218033		X	GG	GG	GG	GG	GG
	GG	GG	GG	GG	GG	GG	GG	GG
	GG	GG	GG	GG	GG	GG	GG	GG
	GG	GG	GG	GG	GG	GG	GG	GG
	GG	GG	GG	GG	GG	GG	GG	
123602321								
7693	rs10218064		X	GG	GG	GG	GG	GG
	GG	GG	GG	GG	GG	GG	GG	GG
	GG	GG	GG	GG	GG	GG	GG	GG
	GG	GG	GG	GG	GG	GG	GG	GG
	GG	GG	GG	GG	GG	GG	GG	
154282761								
7694	rs10218078		X	GG	GG	GG	GG	GG
	GG	GG	GG	GG	GG	GG	GG	GG
	GG	GG	GG	GG	GG	GG	GG	GG
	GG	GG	GG	GG	GG	GG	GG	GG
	GG	GG	GG	GG	GG	GG	GG	
122453514								
7695	rs10218092		X	GG	GG	GG	AA	GG
	AA	AG	GG	GG	GG	GG	GG	GG
	AG	AG	AG	GG	AG	AG	GG	AA

AA GG AA AG GG AA AG GG
AG GG AA GG AG AA GG
121361941
7696 rs10218111 X GG GG GG GG GG
GG GG GG GG GG GG GG GG
GG GG GG GG GG GG GG GG
GG GG GG GG GG GG GG GG
GG GG GG GG GG GG GG
29392785
7697 rs10218132 X AA AG GG AG GG
AA GG GG GG GG GG GG GG
GG GG AG AG GG GG AG GG
GG GG GG GG GG GG GG GG
AA GG GG GG AG AG GG
99700776
7698 rs10218137 X GG GG GG GG GG
GG GG GG GG GG GG GG GG
GG GG GG GG GG GG GG GG
GG GG GG GG GG GG GG GG
GG GG GG GG GG GG GG
28118629
7699 rs10218148 X AA AA AA AA AA
AA AA AA AA AA AG AA AG
AG AG AG AG AA AG AG GG
AA AA AA AA AA AA AA AA
AA AA AA AA AA AA AA
152923490
7700 rs10218163 X GG GG GG GG GG
GG GG GG GG GG GG GG GG
GG GG GG GG GG GG GG GG
GG GG GG GG GG GG GG GG
GG GG GG GG GG GG GG
16147121
7701 rs10218175 X AA AA AA AG AA
GG AA AA AA AA AA AA AA
AA AA AA AA AA AA AA AA
AA AA AA AA AA AA AA AA
AA AA AA AA AA AA AA
127611084
7702 rs10218196 X AA AA AA AA AA
AA AA AA AA AA AA AA AA
AA AA AA AA AA AA AA AA
AA AA AA AA AA AA AA AA
AA AA AA AA AA AA AA
105197945
7703 rs10218289 X GG GG GG GG GG
GG GG GG GG GG GG GG GG
GG GG GG GG GG GG GG GG
GG GG GG GG GG GG GG GG
GG GG GG GG GG GG GG

151211854
7704 rs10218297
X -- -- -- -- -- -- -- --
-- -- -- -- -- -- -- --
-- -- -- -- -- -- -- --
-- -- -- -- -- -- -- --
-- -- -- -- 8666074
7705 rs1021830 6 AA AA AA AA AA
AA AA AA AA AA AA AA AA
AA AA AA AA AA AA AA AA
AA AA AA AA AA AG AA AA
AG AG AA AG AG AG AG
62658768
7706 rs10218300 X AA AA AA AA AA
AA AA AA AA AA AC AA AC
AC AC AA AA AA AA AA AA
AA AA AA AA AA AA AA AA
AA AA AA AA AA AA AA
99517548
7707 rs10218302 X GG GG GG GG GG
GG GG GG GG GG GG GG GG
GG GG GG GG GG GG GG GG
GG GG GG GG GG GG GG GG
GG GG GG GG GG GG GG
145836307
7708 rs1021831 12 AG AG AA AG GG
AG GG GG GG GG AG AG AG
GG AA GG AG GG GG GG GG
GG GG GG GG GG GG GG GG
GG GG GG GG GG GG GG
128141516
7709 rs10218322 X AA AA AA AA AA
AA AA AA AA AA AA AA AA
AA AA AA AA AA AA AA AA
AA AA AA AA AA AA AA AA
AA AA AA AA AA AA AA
48569693
7710 rs10218380 X AA AA AA AA AA
AA AA AA AA AA AA AA AA
AA AA AA AA AA AA AA AA
AA AA AA AA AA AA AA AA
AA AA AA AA AA AA AA
128956647
7711 rs10218384 X AA AC CC CC CC
CC CC AC CC AA AC AC AA
AA AA AC AC CC AC CC AA
CC CC CC CC AA CC AC AA
CC AC CC AA CC CC CC
125188314
7712 rs10218408 X GG GG GG GG GG

	GG	GG	GG	GG	GG	GG	GG	GG
	GG	GG	GG	GG	GG	GG	GG	GG
	GG	GG	GG	GG	GG	GG	GG	GG
	GG	GG	GG	GG	GG	GG	GG	
128848736								
7713	rs10218421		X	AA	AG	AG	AG	AA
	AA	AG	AA	AA	AA	AA	AA	AG
	AG	AA	AA	AA	AA	AA	AA	AA
	GG	GG	AA	AG	AA	AA	AA	AA
	AG	AG	AA	GG	AA	AA	AA	
4482666								
7714	rs10218498		1	GG	GG	GG	GG	GG
	GG	GG	GG	GG	GG	GG	GG	GG
	GG	GG	GG	GG	GG	GG	GG	GG
	GG	GG	GG	GG	GG	GG	GG	GG
	GG	GG	GG	GG	GG	GG	AG	
216965834								
7715	rs10218541		1	GG	GG	GG	GG	GG
	GG	GG	GG	GG	GG	GG	GG	GG
	GG	GG	GG	GG	GG	GG	GG	GG
	GG	GG	GG	GG	GG	GG	GG	GG
	GG	GG	GG	GG	GG	GG	GG	
8952029								
7716	rs10218568		1	GG	GG	GG	GG	AG
	GG	AG	GG	GG	GG	GG	AG	AG
	GG	GG	GG	GG	GG	GG	GG	GG
	GG	AG	AG	AG	AG	GG	GG	GG
	AA	GG	AG	GG	GG	GG	GG	
113925870								
7717	rs10218694		1	CC	AC	CC	AC	CC
	CC	AC	CC	AC	CC	AC	CC	CC
	CC	AC	AC	AC	AC	AC	CC	AA
	CC	AC	AC	AC	CC	AC	CC	AC
	AC	CC	CC	CC	AC	AC	CC	
216761107								
7718	rs10218698		1	AG	AA	AG	AG	AG
	AG	GG	AA	AG	AG	AG	AG	AG
	AG	AA	AG	GG	AA	AG	AG	AG
	AA	GG	AG	AG	AG	AA	AG	AA
	GG	AG	AG	AG	AG	GG	AA	
76870939								
7719	rs10218724		1	AG	AG	AG	GG	GG
	GG	GG	GG	GG	GG	GG	GG	GG
	GG	GG	GG	GG	GG	AG	AG	GG
	GG	GG	GG	GG	GG	GG	GG	GG
	GG	AG	GG	GG	GG	GG	GG	
209214739								
7720	rs10218732		1	AG	GG	AG	AG	GG
	AG	GG	GG	GG	GG	GG	GG	GG
	GG	GG	AG	AG	AG	AG	AG	GG

	GG	AA	AA	AG	GG	AG	AG	GG
	GG	GG	GG	GG	GG	GG	GG	
193493591								
7721	rs10218738		1	AA	AA	AA	AA	AA
	AA	AA	AA	AA	AA	AA	AG	AG
	AA	GG	AA	AA	AA	AA	AA	AA
	AA	AA	AA	AA	AG	AA	AA	AA
	AA	AA	AA	AA	AA	AA	AA	
221280094								
7722	rs10218752		1	AA	AA	AA	AA	AA
	AA	AA	AA	AA	AA	AA	AA	AA
	AA	AA	AA	AA	AA	AA	AG	AA
	AA	AG	AA	AG	AA	AA	AA	AA
	AA	AG	AA	AA	AA	AA	AA	
163171868								
7723	rs1021877		8	AG	AG	AG	GG	AA
	AG	AG	AG	AG	AG	GG	AG	AG
	AG	GG	AG	GG	AA	AA	AG	AA
	AG	GG	AG	GG	GG	AG	AG	AG
	GG	GG	AA	AG	AA	AA	AA	
114000137								
7724	rs1021878		8	AG	AG	AG	GG	AA
	AG	AG	AG	AG	AG	GG	AG	AG
	AG	GG	AG	GG	AA	AA	AG	AA
	AG	GG	AG	GG	GG	AG	AG	AG
	GG	GG	AA	AG	AA	AA	AA	
114085482								
7725	rs1021885		12	AG	AA	AG	AA	AA
	AA	AA	AG	AG	GG	GG	GG	AG
	AG	AG	GG	GG	GG	AG	AG	AG
	GG	AG	AG	AG	AG	AG	AG	AG
	GG	GG	AG	GG	GG	AG	GG	
60708275								
7726	rs10218913		10	GG	AG	GG	GG	AA
	AG	AG	AG	AG	AA	GG	GG	AG
	AG	GG	AA	AG	AG	GG	AG	GG
	AG	GG	AG	GG	AA	AG	AA	AA
	AA	GG	GG	GG	AG	AA	GG	
125594019								
7727	rs1021892		17	GG	GG	GG	AG	AG
	AA	GG	GG	GG	GG	AG	AG	GG
	GG	GG	GG	GG	AG	GG	GG	AG
	GG	GG	AG	GG	GG	GG	GG	GG
	AG	GG	GG	GG	GG	GG	AG	
31710015								
7728	rs10218945		10	GG	GG	GG	AA	GG
	AG	AG	AA	AG	AG	AA	AA	AA
	AA	AA	GG	GG	GG	AA	AG	AA
	AG	AG	AG	GG	AG	AG	GG	AA
	AA	AG	GG	GG	AA	AA	AA	

54864975
7729	rs10219 2	GG	GG	GG	AG	AG	AG
AG	AG	AG	AG	AA	AA	AG	AA
AA	GG	AG	AG	GG	AG	AG	AG
AG	AG	AG	AA	AA	AA	AA	AG
AA	GG	AG	AA	AA	AA	39084040
7730	rs10219008	10	GG	GG	GG	GG	GG
GG	GG	GG	GG	GG	GG	GG	GG
GG	GG	GG	GG	GG	AG	GG	GG
AG	AG	GG	AG	GG	GG	GG	GG
GG	GG	GG	GG	GG	GG	AG
36356628
7731	rs10219012	10	AG	AG	AG	AG	GG
GG	AG	GG	GG	GG	GG	GG	GG
GG	GG	GG	AG	AG	GG	GG	GG
GG	GG	GG	GG	AG	AG	AG	AG
GG	GG	GG	GG	GG	GG	GG
49871496
7732	rs10219033	10	AA	AG	AA	AA	AA
AA	AA	AA	AA	AA	AA	AA	AA
AA	AA	AG	AA	AG	AA	AA	AA
AA	AA	AA	AA	AA	AG	AG	AG
AG	AG	AA	AA	AG	AA	AG
113780938
7733	rs10219117	10	AG	GG	GG	AA	AG
AG	AG	AG	AG	AG	GG	GG	GG
GG	GG	GG	AG	AG	GG	GG	GG
AG	AG	GG	GG	GG	AG	GG	GG
GG	GG	GG	GG	GG	GG	GG
92022480
7734	rs10219141	10	GG	AG	AG	AG	AA
AA	AG	AA	AA	AA	AG	AG	AG
AG	AA	AG	AA	AG	GG	GG	GG
AG	GG	GG	GG	AA	AA	AA	AA
GG	GG	GG	GG	AG	AG	AG
125949472
7735	rs10219144	10	GG	GG	GG	GG	GG
GG	GG	GG	GG	GG	AG	AG	GG
AG	GG	GG	AG	AG	AG	AA	GG
GG	GG	GG	GG	GG	GG	GG	GG
GG	GG	AG	AG	GG	GG	AG
1715867
7736	rs10219183	11	CC	CC	CC	CC	CC
CC	CC	CC	CC	CC	CC	CC	CC
CC	CC	AC	CC	AC	CC	CC	CC
AC	CC	AC	AC	CC	AC	AC	CC
CC	CC	AC	CC	CC	CC	CC
66090032
7737	rs10219187	11	AA	AA	AA	AA	AG
AG	AA	AA	AA	AA	AA	AG	AG

	AG	AA	AA	AG	AG	AA	AA	AG
	AA	AG	AA	AA	AG	AA	AA	AG
	AA	AG	AA	AA	GG	GG	AG	
129103092								
7738	rs10219339		11	AG	AA	AA	AA	AG
	AG	AA	AA	AA	AA	AA	AA	AA
	AA	AA	AA	AA	AG	AA	AA	AA
	AA	AG	AA	AG	AA	AG	AG	AA
	AA	AA	AA	AA	AG	AA	AG	
75484850								
7739	rs10219441		12	AG	AG	AG	AA	AG
	AG	AA	AG	AG	AA	AA	AA	AA
	AA	AA	AG	AG	GG	AA	AA	AG
	AA	AA	AA	AA	AA	AA	AA	AA
	GG	GG	AG	AG	AA	AG	AA	
116192880								
7740	rs10219475		12	AG	GG	AG	GG	AG
	AG	AG	AG	GG	AG	GG	AG	AG
	AG	AG	AA	AA	AA	AG	AG	AG
	AA	AA	AA	AA	AG	AA	AA	AA
	AA	AG	AA	AG	AG	AG	GG	
100103164								
7741	rs1021948		3	AG	AA	AG	AA	AA
	AA	AA	AA	AA	AA	AG	AA	AG
	AA	AA	AA	AA	AA	AA	AA	AA
	AA	AG	AA	AG	AA	AA	AA	AA
	AA	AG	AG	AG	AG	AA	AG	
30057645								
7742	rs10219498							
12	--	--	--	--	--	--	--	--
	--	--	--	--	--	--	--	--
	--	--	--	--	--	--	--	--
	--	--	--	--	--	--	--	--
	--	--	--	--	127594114			
7743	rs10219520		12	AA	AA	AA	AA	AA
	AA	AA	AA	AA	AA	AA	AA	AA
	AA	AA	AA	AA	AA	AA	AA	AA
	AA	AA	AA	AA	AA	AA	AA	AA
	AA	AA	AA	AA	AA	AA	AA	
48541320								
7744	rs1021954							
8	--	--	--	--	--	--	--	--
	--	--	--	--	--	--	--	--
	--	--	--	--	--	--	--	--
	--	--	--	--	--	--	--	--
	--	--	--	--	128203857			
7745	rs10219580		12	GG	GG	GG	GG	GG
	GG	GG	GG	GG	GG	GG	GG	GG
	GG	GG	GG	GG	GG	GG	GG	GG
	GG	GG	GG	GG	GG	GG	GG	GG

	GG	GG	AG	GG	GG	AG	GG	
60443209								
7746	rs10219591		12	AA	AC	AA	CC	AA
	AC	AC	AC	CC	CC	AC	AC	AC
	AA	AA	AA	AA	AA	AC	AA	AC
	AC	CC	AC	CC	AC	AC	AC	AC
	AA	AA	AC	AA	CC	AC	CC	
41983883								
7747	rs10219642		12	AA	AA	AA	AA	AA
	AA	AA	AA	AA	AA	AA	AA	AA
	AA	AA	AA	AA	AA	AA	AA	AA
	AA	AA	AA	AA	AA	AA	AA	AA
	AA	AA	AA	AA	AA	AA	AA	
27075460								
7748	rs10219684		12	AG	AA	AG	GG	AG
	AG	AG	AA	AA	AA	AG	AA	AA
	AA	AA	AG	AA	AA	AA	AA	AG
	AA	AA	AA	AA	AG	AA	AG	AA
	AA	AG	AG	AG	AA	AG	AA	
10026944								
7749	rs10219701		12	GG	GG	GG	GG	AG
	GG	AG	AG	AG	GG	AA	AG	AA
	GG	GG	AG	AG	AG	GG	AG	AG
	GG	GG	AG	GG	AG	GG	AG	AG
	AG	AG	AA	AG	GG	GG	AG	
30813513								
7750	rs10219714		12	AG	AA	AG	AA	AA
	AA	AG	GG	AG	AG	AA	AG	AA
	AG	AA	AG	AG	AG	AG	AG	AG
	--	GG	AG	GG	AG	AA	AA	AG
	AG	AG	GG	AG	GG	GG	AG	
19119135								
7751	rs10219728		12	AG	GG	AG	AG	AA
	AG	AG	AG	GG	GG	AA	AG	AG
	AG	AG	GG	GG	GG	AG	GG	AA
	AG	AG	AG	AG	AG	AG	AA	AG
	AG	GG	AG	AG	AG	AG	AG	
17639821								
7752	rs10219760		12	GG	GG	GG	GG	GG
	GG	AG	AG	AG	AA	AG	AG	AG
	AG	AG	AA	AG	AG	AG	GG	AA
	AG	AG	AG	GG	AA	GG	AG	AG
	GG	GG	AG	AG	AG	GG	AG	
23636579								
7753	rs1021985		15	GG	GG	GG	AG	AA
	AG	AG	AG	AG	AG	GG	GG	AG
	AG	AG	AG	GG	GG	GG	GG	AG
	AG	AG	AG	GG	GG	AG	GG	AG
	AG	AA	AG	AA	AG	AG	AG	
98285658								

7754 rs10219892 13 AA AA AA AA AA
AA AA AA AA AA AA AA AA
AA AA AA AA AA AA AA AA
AA AA AA AA AA AG AG AA
AG AA AA AA AA AA AA
48021607
7755 rs10219897 13 AA AA AA AC AA
AC AA AA AA AA AA AA AA
AA AA AA AA AA AA AA AA
AA AA AA AA AA AA AA AA
AA AA AA AA AA AA AA
82528162
7756 rs1021996 12 GG GG GG GG GG
GG GG GG GG GG GG GG GG
GG GG GG GG GG GG GG GG
GG GG AG GG GG GG GG GG
GG AG GG GG AG GG AG
51789617
7757 rs10219971 13 AA AA AA GG AG
GG AG AA AA AA AA AA AG
AA AA AG AG AG AA AG AA
AA AG AG AA AA AG AG AA
GG AA AA AA AA AA AA
74324502
7758 rs10220127 13 CC AC CC AA AA
AA AA AC AC AC AA AC AC
AA AA AA AA AA AC AA CC
AA AA AA AA AA AA AA AA
AA AC AA AC AA AC AA
102397075
7759 rs10220129 13 GG GG GG GG GG
GG GG GG GG GG GG GG GG
GG GG GG GG GG GG GG GG
GG GG GG GG GG GG GG GG
GG GG GG GG GG GG GG
112053424
7760 rs10220207 13 AA AC AA AA AA
AA AA AA AA AA AA AA AA
AA AA AA AA AA AA AA AA
AA AA AC AA AA AA AA AA
-- -- AA AA AA AA AA
109094088
7761 rs10220253 13 AC AC AC CC CC
CC CC AC CC AC CC CC CC
AC AC CC CC CC CC CC CC
CC CC CC CC CC AC AC CC
CC CC AC CC CC CC CC
69683174
7762 rs10220265 13 GG GG GG AG GG
GG AG GG GG GG GG GG GG

GG GG GG AG AG GG GG GG
GG GG GG GG GG GG GG GG
GG GG AG AG GG AG GG
104049583
7763 rs10220273 13 GG GG GG AG GG
GG AG GG GG GG GG GG GG
GG GG GG GG GG GG GG GG
GG GG GG GG GG GG GG GG
GG GG AG AG GG AG GG
104049774
7764 rs10220279
14 -- -- -- -- -- -- -- --
-- -- -- -- -- -- -- --
-- -- -- -- -- -- -- --
-- -- -- -- -- -- -- --
-- -- -- -- 73157280
7765 rs10220374 14 GG GG AG AG GG
GG AG GG GG GG GG GG GG
AG GG AG GG GG AG AG AG
AG AA AA AG AG AG AG AG
GG GG GG GG AG AA AG
25244271
7766 rs10220383 14 AA AA AA AG AG
AG AG AG AG AG AA AA AA
AA AA AG AG AA AG AA AG
GG AG AA AA AG AA AG AG
AA AG AG AA AA AG AA
48312877
7767 rs10220418 14 GG GG GG GG GG
GG GG GG GG GG GG GG GG
GG GG GG GG GG GG GG GG
GG GG GG GG GG GG GG GG
GG GG GG GG GG GG GG
95548080
7768 rs1022044 5 AG GG AG AG AG
AG AG AA AA AA AG AG AA
AA AA AG AG AG GG AG GG
AG AG AG AG AG AG GG AG
AG GG AG GG AG GG AA
174897200
7769 rs10220481 14 AA AA AA AA AA
AA AA AA AA AA AA AA AA
AA AA AA AA AA AA AA AA
AA AA AA AA AA AA AA AA
AA AA AA AA AA AA AA
66244184
7770 rs1022049 5 AA AA AA GG GG
AG AG AG AG AG GG AA AG
AG AG GG AG AG AG GG AG
AG AG AG AG AG AG AG AG

	AG	AA	AA	AA	AG	AG	AA	
38014870								
7771	rs10220568		14	GG	AG	GG	AA	AA
	AA	AA	AG	AG	AG	AA	AA	AA
	AG	AA	AA	AG	AA	AA	GG	AA
	AG	AG	AG	AG	AA	AG	AA	AG
	GG	AG	AA	AG	AG	AA	AG	
28429366								
7772	rs10220696		14	GG	AG	AG	AG	GG
	GG	AG	GG	GG	GG	AG	GG	AG
	GG	GG	GG	GG	GG	GG	GG	GG
	AG	AG	AG	AG	GG	AA	AG	AG
	GG	AG	GG	GG	GG	AG	GG	
92560943								
7773	rs10220733		15	AG	GG	AG	GG	AG
	AG	AG	AG	GG	GG	AG	AG	AG
	AG	AG	AG	AG	AG	GG	GG	GG
	GG	GG	GG	GG	AG	GG	AG	GG
	GG	AA	AG	AA	GG	GG	GG	
85280864								
7774	rs10220773		15	AG	AG	GG	GG	AG
	GG	AG	AG	AG	AG	AG	AG	AG
	AG	AG	GG	GG	GG	AG	AG	AG
	AA	AA	AA	AA	AA	AG	AG	AA
	AG	GG	AA	AG	AA	AA	AG	
86903044								
7775	rs1022079		4	CC	CC	CC	AC	AC
	AC	CC	CC	AC	AA	AC	CC	AC
	AC	CC	CC	AC	AC	AC	AC	CC
	CC	CC	CC	CC	CC	AC	AC	CC
	CC	CC	CC	CC	AC	AA	AC	
131238094								
7776	rs10220839		15	AA	AA	AA	AA	AA
	AA	AA	AA	AA	AA	AA	AA	AA
	AA	AA	AA	AA	AA	AA	AA	AA
	AA	AA	AA	AA	AA	AA	AA	AA
	AA	AA	AA	AA	AA	AA	AA	
27072227								
7777	rs10220852		15	GG	GG	GG	GG	GG
	AG	GG	GG	GG	GG	GG	AG	AG
	AG	AG	GG	GG	GG	GG	GG	AG
	GG	AG	AG	GG	AG	GG	AG	AG
	GG	GG	GG	GG	AG	AG	AG	
53839779								
7778	rs10220974		16	GG	AG	GG	AG	AG
	AG	AG	GG	GG	GG	GG	GG	AG
	AG	AG	GG	GG	GG	AA	AG	AA
	AG	GG	GG	AG	GG	AG	AG	GG
	AG	GG	GG	GG	GG	AG	GG	
78132274								

7779 rs1022098 16 GG GG GG AG AG
AG AG GG GG GG GG GG GG
GG GG AG GG GG AA AG AG
GG AG GG GG AG GG AG AG
AG AG AG GG GG GG GG
9308914
7780 rs10220998 16 AC AC AC AC AC
CC AC AA AA AA AC AC AA
AC AC AA AA AC AC AC AA
AA AA AA AC AC AC AC AC
AC AC AC AA CC AC AC
48949909
7781 rs10221054 16 GG GG GG AG GG
AG GG GG GG GG GG GG GG
GG GG GG AG GG GG GG GG
GG GG GG GG GG GG GG GG
GG GG GG GG GG GG GG
10775647
7782 rs10221077 16 AA AA AA AA AA
AA AA AA AA AA AA AA AA
AA AA AA AA AA AA AA AA
AA AA AA AA AA AA AA AA
AA AA AA AA AA AA AA
33858001
7783 rs10221110 16 AG AG AA AG AA
AG AA AA AG AA AA AA AA
AA AA AG AG AG AA AA AA
AA AA AA AA AA AG AG AA
GG AA AA AA AA AA AA
10773407
7784 rs10221121 16 GG GG GG AG AG
GG AA GG GG GG AA AA AA
AA AG AG AA AG GG GG GG
AG AA AG GG GG GG GG GG
GG GG GG GG GG AG GG
56840328
7785 rs10221192 17 AA AA AA AA AA
AA AA AA AA AA AA AA AA
AA AA AA AA AA AG AG AG
AA AA AA AA AG AA AG AG
AG AA AG AG AA AA AG
33048655
7786 rs1022121 2 AA AA AA AA AA
AA AA AA AA AA AA AA AA
AA AA AA AA AA AA AA AA
AA AA AA AA AA AA AA AA
AA AA AA AA AA AA AA
36354424
7787 rs10221231 17 GG GG GG GG GG
GG GG GG GG GG GG GG GG

	GG	GG	GG	GG	GG	GG	GG	GG
	GG	GG	GG	GG	GG	GG	GG	GG
	GG	GG	GG	GG	GG	GG	GG	
73654642								
7788	rs10221274		17	CC	CC	CC	CC	CC
	CC	AC	AC	AA	AA	CC	CC	CC
	CC	CC	CC	AC	AC	CC	AC	CC
	AC	AA	AC	AA	AA	AA	AA	AA
	AA	AC	CC	AC	AA	AA	AC	
68093498								
7789	rs10221288		18	AA	AA	AA	AG	AG
	AA	AG	AA	AA	AA	AA	AA	AA
	AA	AA	AA	AA	AA	AA	AA	AA
	AA	AA	AA	AA	AG	AA	AA	AG
	AA	AA	AA	AA	AA	AA	AA	
18774267								
7790	rs1022129		X	AA	AA	AA	AA	GG
	AA	AG	AG	AG	AA	AG	AG	AA
	AA	AA	AG	AG	AA	AG	AA	GG
	AA	GG	GG	AA	AA	AA	AA	AA
	AG	AA	AA	AA	AA	AG	AA	
68222952								
7791	rs1022135		4	CC	CC	CC	CC	CC
	CC	CC	CC	CC	CC	AC	CC	CC
	CC	CC	CC	CC	CC	AC	AC	AC
	CC	CC	CC	CC	AC	AC	CC	AC
	CC	CC	CC	CC	AC	CC	AC	
4707192								
7792	rs10221365		18	AG	AG	GG	GG	GG
	GG	GG	AG	AG	AG	AG	AG	AG
	AA	AG	GG	AG	GG	AG	GG	AA
	AA	AA	AG	AG	GG	AG	AG	AG
	AG	AG	AG	GG	AG	GG	AG	
56896679								
7793	rs1022140		8	GG	GG	GG	AG	GG
	AG	AG	AG	GG	GG	GG	AG	AG
	AA	AG	GG	GG	GG	GG	GG	GG
	GG	GG	GG	GG	GG	GG	GG	GG
	AG	GG	GG	GG	GG	GG	GG	
49213910								
7794	rs10221410		18	GG	GG	GG	GG	GG
	AG	AG	GG	GG	GG	AG	GG	AG
	GG	AG	GG	GG	GG	GG	GG	GG
	GG	GG	GG	GG	GG	GG	GG	GG
	AG	GG	GG	GG	GG	GG	GG	
5605989								
7795	rs10221419		18	GG	GG	GG	GG	GG
	GG	GG	AG	GG	--	GG	GG	GG
	GG	GG	GG	GG	GG	AG	GG	AG
	AG	GG	GG	AG	GG	AG	AG	GG

	GG	GG	GG	GG	GG	GG	GG
7658679							
7796	rs10221434	18	AA	AA	AA	AG	AG
	AG	AA	GG	AA	GG	GG	AG	AG
	AG	AG	AA	AA	AA	AG	AG	AG
	AG	AA	AA	AG	GG	AG	AG	AG
	AA	AG	AA	AG	AG	AG	AA	
24016157								
7797	rs10221471	19	AG	AA	AA	AG	AG
	GG	AA	GG	GG	GG	AA	AG	AA
	AG	GG	GG	AG	AG	AA	AA	AA
	AG	AA	AA	AG	AA	AA	AA	AA
	GG	AA	AG	AG	AA	AA	AA	
31929293								
7798	rs10221474	0	AA	AA	AA	AA	AA
	AA	AA	AA	AA	AA	AA	AA	AA
	AA	AA	AA	AA	--	AA	AA	AA
	AA	AA	AA	AA	AA	AA	AA	AA
	AA	AA	AA	AA	AA	AA	AA	0
7799	rs10221509	19	GG	GG	GG	GG	GG
	GG	GG	GG	GG	GG	GG	GG	GG
	GG	GG	GG	GG	GG	GG	GG	GG
	GG	GG	GG	GG	GG	GG	GG	GG
	GG	GG	GG	GG	GG	GG	GG	
4049550								
7800	rs10221531	19	AA	AA	AA	AA	AA
	AA	AA	AA	AA	AA	AA	AA	AA
	AA	AA	AA	AA	AA	AA	AA	AA
	AA	AA	AA	AA	AA	AA	AA	AA
	AA	AA	AA	AA	AA	AA	AA	
12412275								
7801	rs10221578	2	AA	AG	AA	GG	GG
	GG	GG	GG	GG	GG	AA	GG	AG
	GG	AG	AG	AA	AA	GG	AG	GG
	AG	AG	AG	AG	GG	GG	GG	GG
	AA	AG	GG	AG	GG	AG	GG	
220833222								
7802	rs10221591	2	GG	GG	GG	GG	GG
	GG	GG	GG	GG	GG	GG	GG	GG
	GG	GG	GG	GG	GG	GG	GG	GG
	GG	GG	GG	GG	GG	GG	GG	GG
	GG	GG	GG	GG	GG	GG	GG	
97318034								
7803	rs10221632	2	GG	AG	AG	AG	AG
	AG	GG	GG	GG	GG	AG	GG	GG
	AG	AG	GG	GG	GG	GG	AG	GG
	GG	GG	GG	GG	GG	AA	AG	AG
	AG	AG	AG	GG	AA	AA	AG	
119547211								
7804	rs10221671	2	GG	GG	GG	AA	AG

AA AG GG AG GG AG AG AG
AG AG AG AG GG AG AG AA
AG AG AG GG AG AG AA AG
AG AG AG AG AA AA AG
15305407
7805 rs10221700 2 GG GG GG GG GG
GG GG GG GG GG GG GG GG
GG GG GG GG GG GG GG GG
GG GG GG GG GG GG GG GG
GG GG GG GG GG GG GG
111401428
7806 rs1022172 15 GG GG GG AG GG
GG AG AG AA AA AG GG GG
AG GG GG GG GG GG AG AG
GG AG AG AG AG GG AG GG
AG AG GG AG GG GG GG
77793359
7807 rs10221738 2 GG GG GG AG AG
AG GG GG GG GG AG AG GG
GG GG AG GG AG GG AG GG
AG AG AG AG AG AA AG AG
GG AG GG GG AG GG AA
236085432
7808 rs1022177 18 AA AG AG AG AA
AG AA AG AA AG AA AA AA
AA AA AG AG AG AG AG AA
AG AG AG AA AA AA AA AA
AG AG AG AG AG AG AG
1046304
7809 rs10221780 2 AA AG AA AA AA
AA AA AA AA AA AA AA AA
AG AG AA AA AA AA AA AG
AA AA AA AA AA AG AG AG
AA GG AA AG AA AA AA
146588323
7810 rs10221803 2 AA AA AA AG AA
AG AG GG GG AG AA AA AG
AA AA AA AA AA AA AA AA
AA AA AG AG GG AA AG AG
AA AG AG AG AA AA AG
110270250
7811 rs10221815 2 AA AG AA AA AA
AA AA AA AG AA AA AA AA
AA AA AA AA AA AG AG AG
AA AA AA AA AA AG AA AG
AA AG AA AA AA AG AA
88697965
7812 rs10221829 2 GG GG GG GG GG
GG GG GG GG GG GG GG GG
GG GG GG GG GG GG GG GG

GG GG GG GG GG GG GG GG
GG GG GG GG GG GG GG
113520969
7813 rs10221883 2 AC AC AC CC CC
CC AC AC AC AC AC CC AC
AA AA AC AC AC CC AC CC
AA AA AA AA AC AC CC AC
AC CC AA AC CC CC CC
140794737
7814 rs1022194 8 AA AG GG AG AA
AA AG AG AG AG AG GG AG
AG AA GG GG AG AA AA AG
AG GG GG GG AG AA AG AG
AG AA AG AA GG GG AG
40763844
7815 rs10221945 2 AA AG AA AG AG
AA GG AA AA AA AA AA AA
AA AG AA AG AG AG AG AG
AG AG GG GG AA AA AA AA
AA AA AA AA AG AG AA
200875847
7816 rs10221988 20 AA AA AA AA AA
AA AA AA AA AA AA AA AA
AA AA AA AA AA AA AA AA
AA AA AA AA AA AA AA AA
AA AA AA AA AA AA AA
51080157
7817 rs10222039 21 GG GG GG GG GG
GG GG GG GG GG GG GG GG
GG GG GG GG GG GG GG GG
GG GG GG GG GG GG GG GG
GG GG GG GG GG GG GG
38176616
7818 rs10222074 21 GG GG GG GG GG
GG GG GG GG GG GG GG GG
GG GG AG GG GG GG GG GG
GG GG GG GG GG GG GG GG
GG AG GG GG GG GG GG
28429928
7819 rs10222075 21 AG GG AG AA AG
AG AA AA AA GG AA AG AG
AG AG AG AA AA AG AA AA
AA AA AA AA AG AG GG AG
AA AG AA AG AG AG AG
28473520
7820 rs10222315 22 AA AA AA AA AA
AA AA AA AA AA AA AA AA
AA AA AA AA AA AA AA AA
AA AA AA AA AA AA AA AA
AA AA AA AA AA AA AA

18200225								
7821	rs10222352		3	AC	AC	AA	CC	CC
	CC	AC	AC	CC	AC	CC	AA	AC
	AC	AA	AC	AC	CC	CC	AC	AC
	AC	CC	AC	AC	CC	AC	CC	CC
	AC	CC	CC	CC	CC	CC	AC	
7533704								
7822	rs10222419		3	AA	AC	AC	AA	AA
	AA	AA	AA	AA	AA	AC	AA	AC
	AA	AC	AC	CC	AA	AC	AA	AC
	AA	AA	AC	AA	AC	CC	AC	CC
	AA	AC	AA	AA	AA	AC	AA	
143162091								
7823	rs1022249		6	GG	GG	GG	GG	GG
	GG	GG	GG	GG	GG	AG	AG	AG
	GG	AG	GG	AG	GG	AG	AG	AG
	GG	GG	GG	GG	GG	GG	GG	GG
	AG	GG	GG	GG	AG	GG	AA	
4655282								
7824	rs1022251		4	CC	CC	CC	CC	CC
	CC	CC	CC	CC	CC	CC	CC	CC
	CC	CC	CC	CC	CC	CC	CC	CC
	AC	AC	AC	AC	CC	CC	CC	CC
	AA	CC	AA	AC	CC	CC	CC	
122369974								
7825	rs1022258		8	AA	AA	AA	AA	AA
	AA	AA	AA	AA	AA	AA	AA	AA
	AA	AA	AG	AA	AG	AA	AA	AA
	AA	AA	AA	AA	AA	AA	AA	AA
	AA	AA	AA	AA	AA	AA	AA	
9729939								
7826	rs10222587		3	GG	GG	GG	AG	AG
	GG	AG	GG	GG	GG	GG	GG	GG
	GG	GG	GG	GG	GG	AG	AG	AG
	GG	GG	GG	GG	GG	AG	AG	AG
	GG	GG	GG	GG	GG	AG	GG	
7533618								
7827	rs10222597		3	GG	GG	GG	GG	GG
	GG	GG	GG	GG	GG	GG	GG	GG
	AG	AG	GG	GG	GG	AG	GG	GG
	AG	GG	AG	AG	GG	GG	GG	GG
	GG	GG	AG	AG	GG	GG	GG	
33264914								
7828	rs1022265		1	AG	AA	AG	AG	GG
	AG	AG	AG	AG	AA	AG	AG	AA
	AA	AA	AG	AG	GG	AA	AG	AG
	AG	AA	AG	AG	GG	AA	AG	AG
	AA	AG	AA	AG	AG	AG	AA	
61196715								
7829	rs10222715		4	AC	AA	AC	AA	AA

	AA	AA	CC	CC	CC	AA	AA	AA
	AA	AC	AA	AC	AC	AC	AC	CC
	CC	CC	CC	AC	AC	CC	CC	AC
	AA	AA	CC	AC	AC	AA	CC	
57614160								
7830	rs1022273		X	CC	AC	AC	CC	CC
	CC	CC	AC	AC	CC	AC	AC	AC
	AC	CC	AC	AC	AC	AC	AC	CC
	CC	CC	CC	CC	CC	CC	CC	CC
	CC	CC	CC	CC	CC	CC	CC	
15480006								
7831	rs10222862		4	CC	AC	AA	CC	CC
	CC	CC	AC	AC	AC	CC	AC	CC
	CC	AC	AC	AC	CC	CC	CC	AC
	AC	CC	AC	AC	AC	CC	AC	AC
	AC	CC	CC	CC	AC	AC	AC	
10950989								
7832	rs10222938		4	--	GG	--	AG	GG
	GG	AG	GG	GG	GG	GG	GG	GG
	GG	GG	AG	GG	GG	GG	GG	--
	--	GG	GG	GG	GG	GG	--	GG
	GG	GG	AG	GG	GG	GG	GG	
170598148								
7833	rs10222978		4	AG	AG	GG	AG	GG
	AG	AG	AG	AG	GG	AG	AA	AA
	AA	AA	AA	AG	AG	AG	GG	AG
	GG	AG	AG	GG	AA	AA	AA	AA
	AG	AA	AG	AG	GG	AG	GG	
117426252								
7834	rs1022304		3	CC	CC	CC	--	--
	AC	CC	CC	CC	CC	CC	CC	CC
	CC	CC	CC	--	CC	AC	AC	CC
	AC	AC	AC	AC	AC	CC	AC	AC
	CC	AC	CC	CC	AC	AA	AC	
189062649								
7835	rs10223081		5	AG	AA	AG	AA	AA
	AA	AA	AA	AA	AA	AA	AA	AA
	AA	AA	AA	AA	AA	AA	AG	AA
	AG	AG	AG	AG	AA	AA	AA	AA
	AA	AA	AA	AA	AA	AA	AA	
151540970								
7836	rs1022312		2	AA	AA	AA	AG	AA
	AG	AA	AA	AG	AA	AA	GG	AG
	AG	AG	AA	AA	AA	AA	AA	AG
	AA	AA	AA	AA	AG	AG	AG	AG
	AG	AG	AG	GG	AG	AG	AA	
10040351								
7837	rs1022314		8	GG	GG	GG	GG	GG
	GG	GG	GG	GG	GG	GG	AG	AG
	AG	GG	GG	GG	GG	GG	GG	GG

GG GG GG GG AG GG GG AG
GG GG GG GG AG AG GG
115285808
7838 rs10223190 5 AA AA AA AG AG
AG AG AA AA AA AA AA AA
AA AA AA AA AA AA AA AA
AA AG AA AA AA AG AA AA
AA AA AA AA AA AA AA
171579380
7839 rs10223225 5 GG GG GG GG GG
GG GG GG GG GG GG GG GG
GG GG GG GG GG GG GG GG
GG GG GG GG GG GG GG GG
GG GG GG GG GG GG GG
37573314
7840 rs10223236 5 GG GG GG GG GG
GG GG GG GG GG GG GG GG
GG GG GG GG GG GG GG GG
GG GG GG GG GG GG GG GG
GG GG GG GG GG GG GG
29649016
7841 rs10223241 5 AA AA AG AA AA
AG AG AG AG AG AG AG AG
AG AG AA AA AA AA AG AA
AA AA AA AA AG AG AA AG
AG AA AA AA AG AG AG
88412388
7842 rs10223384 6 AG AG AA AG AG
GG AA AG AA AG GG AG AG
AA AA AG AA AA GG AG AG
AG AG GG GG AG AA AG AG
AG GG AG AG AA AG AG
158998571
7843 rs10223507
6 -- -- -- -- -- -- -- --
-- -- -- -- -- -- -- --
-- -- -- -- -- -- -- --
-- -- -- -- -- -- -- --
-- -- -- -- 146285204
7844 rs10223516 6 AA AA AA AA AA
AA AA AA AA AA AG AG AG
AA AG AG AG AG AA AA AA
AA AA AA AA AA AA AA AA
AA AA AA AA AG AG AA
41594613
7845 rs1022357 15 AA AA AA AA AA
AA AA AG AG AG AA AA AG
AA AG GG AG AG AG AG AG
AA AA AA AG AA AG AG AA
AA AA GG AG GG AG AG

92524556
7846 rs10223615 6 GG GG GG GG GG
GG GG GG GG GG GG GG GG
GG GG GG GG GG GG GG GG
GG GG GG GG GG GG GG GG
GG GG GG GG GG GG GG
55657998
7847 rs10223664 6 GG GG GG GG GG
GG GG GG GG GG GG GG AG
GG AG GG GG GG AA AA AA
GG GG GG GG GG GG GG GG
AG GG GG GG GG GG GG
98677699
7848 rs1022367 11 AG AA AA GG GG
GG GG GG AG AG AG AG GG
AG GG AG GG AA AA AG AA
GG AG GG AG AG AA AA AA
AA AG AA AA GG AG AG
36760325
7849 rs10223811 6 AA AA AA AA AA
AA AA AA AA AA AA AA AA
AA AA AA AA AA AA AA AA
AA AA AA AA AA AA AA AA
AA AA AA AA AA AA AA
102518945
7850 rs1022387 6 AC CC AC AA AA
AA AA AC AC AC CC CC CC
AC AA CC CC CC AC AC AC
AA AA AC AA AA AC AA AA
AC CC AA AC AA AC AC
114462618
7851 rs10223916 7 AG AG AG AA AA
AA AA AA AG AA AA AA AA
AG AG AA AA AA AG AA AA
AA AA AA AA AA AA AA AA
AA AA AG AG AG AG AG
55890162
7852 rs10223928 7 GG GG GG AG AG
AG AG GG GG GG GG GG GG
AG GG GG GG GG AA AG AG
AA AA AA AA AG GG GG GG
AG GG GG GG AA AG AA
101045580
7853 rs10223962 7 CC CC CC CC CC
CC CC CC CC CC CC CC CC
CC CC CC CC CC CC CC CC
CC CC CC CC CC CC CC CC
CC CC CC CC AC CC AC
33624926
7854 rs10223990 7 AG AG AG AA AA

AA AA AG AG AG AA AA AA
AA AA AG AG AA AG AA AG
GG AG GG AG AA AA AA AA
GG GG AA AG AA AA AA
18979516
7855 rs1022400 6 AG AG AA GG AG
AG AG AG GG GG AG AG AG
AG AA GG AG GG AA AG AA
AA AA GG AG AA AG AA AA
GG AG GG AG AG AG AG
137674040
7856 rs1022404 6 AC AC AA AC AC
AC AC AA AA AA AA AA AC
AA AA AC AA AA AA AA AA
AC AC AA AA AA AA AA AA
AA AC AC AC AC AA AC
154899702
7857 rs10224066
7 -- -- -- -- -- -- -- --
-- -- -- -- -- -- -- --
-- -- -- -- -- -- -- --
-- -- -- -- -- -- -- --
-- -- -- -- 76004709
7858 rs10224075 7 AG GG AG GG GG
GG GG AG AG GG AG GG AG
AA AG GG GG GG AA AG AG
GG GG GG GG GG GG GG GG
AG AG AG AG AG GG AG
12189920
7859 rs10224134 0 AG AA AG AA AA
AA AA AA AA AA AA AA AA
AA AG AG AG AG AA AA AA
AA AA AA AA AA AA AA AA
AA GG AA AG AG AG AA 0
7860 rs1022414 19 AG AA AA AG AA
AA AG AA AA AA AG AG GG
AG AG AG AA AA AA AA AA
AA AA AA AG AG AA AG AG
AA AG AA AA AG AG AG
15742373
7861 rs10224185 7 AA AA AG AA AG
AG AA AA AG AA AA AA AA
AA AA AA AA AA AG AA AG
AG AG AG AG AA AA AA AA
AA AA AA AA AA AG AA
54208630
7862 rs10224188 7 GG GG GG GG GG
GG GG GG GG GG GG GG GG
GG GG GG GG GG GG GG GG
GG GG GG GG GG GG GG GG

GG GG GG GG GG GG GG
64040410
7863 rs10224194 7 GG GG GG GG GG
GG GG GG GG GG GG GG GG
GG GG GG GG GG GG GG GG
GG GG GG GG GG GG GG GG
GG GG GG GG GG GG GG
96439983
7864 rs10224222 7 GG GG GG GG GG
GG GG GG GG GG GG GG GG
GG GG GG GG GG GG GG GG
GG GG GG GG GG GG GG GG
GG GG GG GG GG GG GG
104424543
7865 rs10224250 7 AA AA AA AA AA
AA AA AA AA AA AA AA AA
AA AA AC AC AC AC AC AA
AA AA AA AA AA AA AA AA
CC AA AA AA AA AA AA
137618171
7866 rs10224277 7 AG AG AA AG AG
GG AA AA AG AA AA AG AG
GG GG AG AA AG AG AG AG
AG AA AG AG GG GG GG GG
AG AA AG AG GG GG GG
48281178
7867 rs1022429 8 GG AG GG GG AG
GG AG AG AG GG GG GG GG
GG AG AA AG AA AG AG AA
AG AG AG AG GG AG GG GG
AG AG GG AG GG AG GG
75211424
7868 rs1022430 8 AG AG AG AA AG
AA AG GG AG AA AA AA AA
AA AG GG AG GG AG GG GG
AG AG AG AG AA GG AG AG
GG GG AG AG AG GG AA
75211163
7869 rs10224307 7 AA AA AG AG AG
GG AA AA AG AA GG AG AG
AG AG AG AG AG AG AA GG
AA AA AA AA GG GG GG GG
AA AA AG AG AG GG AG
135313837
7870 rs10224365
7 -- -- -- -- -- -- -- --
-- -- -- -- -- -- -- --
-- -- -- -- -- -- -- --
-- -- -- -- -- -- -- --
-- -- -- -- 153920121

7871 rs10224371 7 GG GG GG GG GG
GG GG GG GG GG GG GG GG
GG GG AG GG GG GG GG GG
GG GG GG GG GG AG GG GG
GG AA GG AG AG GG AG
154516381
7872 rs10224383 7 GG GG GG GG GG
GG GG GG GG GG GG GG GG
GG GG GG GG GG GG GG GG
GG GG GG GG GG GG GG GG
GG GG GG GG GG GG GG
56375851
7873 rs10224387 7 AA AA AA AA AA
AA AA AA AA AA AG AA AG
AA AA AG AG AG AA AA AA
AA GG AA AA AA AG AG AG
AG AA AA AA AA AA AA
150313416
7874 rs10224451 7 AA AG AG GG AG
GG AG AG AG AA GG GG AG
AG AG AG GG GG GG AG AG
AA AG AG AA AG GG AG GG
GG AG GG GG AG AG AG
88604651
7875 rs10224471 7 AA AA AA AA AA
AA AA AA AA AA AA AA AG
AA AG AA AA AA AG AG AG
AA AA AA AA AG AG GG GG
AA AA AA AA AA AA AA
6104109
7876 rs10224487 7 GG GG GG AG AA
AA AG GG GG GG AG AG AA
AA AA AG AG AA AG AA GG
AG GG AG GG GG AG GG GG
AG GG AA AG AA AA AA
25454250
7877 rs10224542 7 AA AA AA AA AA
AA AA AC AA AC AC AA AC
AC AA AC AC AA AC AC AA
AA AA AA AA CC CC CC CC
AC AA CC AC AC AC AA
21212389
7878 rs10224633 7 GG GG GG GG GG
GG GG AG GG AG GG GG GG
GG GG GG GG GG GG AG GG
GG GG GG GG GG GG GG GG
AG GG GG GG GG GG GG
156809818
7879 rs10224686 7 GG GG GG GG GG
GG GG GG GG GG GG GG GG

GG GG GG GG GG GG GG GG
GG GG GG GG GG GG GG GG
GG GG GG GG GG GG GG
157128268
7880 rs10224698 7 AG AG AA AA AG
AG AA AA AA AG AG AA AG
AG AG GG AG AG AA AA AA
AG AA AA AG AG AA AA AG
AG AG AA AG AG AG AG
79048739
7881 rs1022476 22 AG GG GG GG AG
AG GG GG AG GG GG GG GG
GG GG GG GG GG GG GG GG
GG GG GG GG GG AG GG GG
GG GG AG AG AG AG AG
45821715
7882 rs10224893 7 GG GG GG GG GG
GG GG GG GG GG GG GG GG
GG GG GG GG GG GG GG GG
GG GG GG GG GG GG GG GG
GG GG GG GG GG GG GG
75913086
7883 rs1022491 6 AG AA AG AG AG
AG AA AG GG GG AA AA AA
AA AA AA AA AG AA AA AA
AA AA AG AG AG AA AG AA
AA AA AA AA AA AA AA
135259366
7884 rs10224910 7 GG AG AG GG AA
AG AG GG GG AG AA AG AG
AA AA GG AG GG GG AG AG
AA GG AG AG AG AG GG AG
AG AA AG AG AG AG GG
43125022
7885 rs10224925 7 AA AA AA AA AG
AA AG AA AA AA AA AA AA
AA AA AG AG AG AA AA AA
AA AA AA AA AG AA AG AG
AA AA AA AA AG AA AG
103647122
7886 rs10224984 7 AA AC AC AC AC
AC AC AA AA AA AA AA AA
CC AC AA AA AA CC AC AC
AC AA AA AC CC AC CC CC
AA AC AC AA AA AC AA
29833841
7887 rs10225000 7 AA AA AA AA AA
AA AA AA AA AA AA AA AA
AA AA AA AA AA AA AA AA
AA AA AA AA AA AA AA AA

	AA	AA	AA	AA	AA	AA	AA
141728209							
7888	rs10225081	7	GG	GG	GG	AG	GG
	GG	AG	GG	AG	GG	GG	GG	GG
	GG	GG	GG	GG	GG	AG	AG	GG
	GG	GG	GG	GG	GG	AG	GG	GG
	GG	GG	AG	AG	GG	GG	GG
22864724							
7889	rs10225087	7	AA	AA	AA	AA	AA
	AA	AA	AA	AA	AA	AA	AA	AA
	AA	AA	AA	AA	AA	AA	AA	AA
	AA	AG	AA	AA	AG	AA	AG	AG
	AA	AG	AA	AA	AG	AA	GG
155737659							
7890	rs10225095	7	AA	AA	AA	AA	AA
	AA	AA	AA	AA	AA	AA	AA	AA
	AA	AA	AA	AA	AA	AA	AA	AA
	AA	AA	AA	AA	AA	AA	AA	AA
	AA	AA	AA	AA	AA	AA	AA
77030333							
7891	rs10225103	7	GG	GG	GG	GG	GG
	GG	AG	GG	GG	GG	AG	AG	GG
	GG	GG	GG	GG	GG	GG	GG	GG
	AG	GG	AG	AG	GG	AA	AG	AG
	GG	GG	GG	GG	GG	GG	GG
129259743							
7892	rs10225134	7	CC	CC	CC	CC	CC
	CC	CC	CC	CC	CC	AC	AC	AA
	AA	AA	AC	CC	CC	AC	CC	AA
	CC	CC	CC	CC	CC	CC	CC	CC
	CC	CC	CC	CC	CC	CC	CC
120035706							
7893	rs1022514	6	AA	AA	AA	AA	AA
	AG	AG	AG	GG	AG	AA	AA	AG
	AA	AA	GG	GG	GG	GG	GG	GG
	AA	AG	AA	AA	AG	GG	AG	GG
	AG	GG	AA	AG	GG	AG	AG
133790391							
7894	rs10225182	7	GG	GG	GG	GG	GG
	GG	GG	GG	GG	GG	GG	GG	GG
	GG	GG	GG	GG	GG	GG	GG	GG
	GG	GG	GG	GG	GG	GG	GG	GG
	AG	AG	GG	AG	AG	GG	AA
27955785							
7895	rs10225188	7	CC	AC	AC	CC	CC
	CC	AC	CC	CC	AC	CC	CC	CC
	CC	CC	AA	AA	AA	AC	CC	AA
	AC	AC	CC	CC	CC	AC	AC	AC
	CC	AA	AC	AC	AC	AC	AC
75608674							

7896	rs10225194		7	AC	AC	AC	AA	AC
	AC	AA	AA	AA	AA	AA	AC	AA
	AA	AC	AA	AA	AA	AC	AC	AA
	CC	AA	AC	AA	AC	AA	AA	AA
	AA	AA	AC	AA	AA	AC	AC	
10278806								
7897	rs10225209		7	AG	AG	AG	AG	AA
	AG	AA	AA	AA	AG	GG	AG	AG
	AA	AA	AA	AA	AG	AA	AG	AA
	AG	AG	GG	AA	AA	AA	AA	AA
	AG	AA	AA	AA	AG	GG	AG	
40841978								
7898	rs10225212		7	CC	CC	CC	CC	CC
	CC	CC	AC	AC	AC	CC	CC	CC
	CC	CC	CC	CC	CC	CC	CC	CC
	CC	AC	CC	CC	CC	CC	CC	CC
	CC	AC	CC	AC	CC	CC	CC	
121628675								
7899	rs10225230		7	GG	GG	GG	GG	AG
	AG	GG	GG	GG	GG	GG	GG	GG
	GG	GG	GG	GG	GG	GG	GG	GG
	GG	GG	GG	GG	GG	GG	GG	GG
	GG	GG	GG	GG	GG	GG	GG	
18761408								
7900	rs10225235		7	AA	AA	AA	AA	AA
	AA	AA	AA	AA	AA	AA	AA	AA
	AA	AA	AA	AA	AA	AA	AA	AA
	AA	AA	AA	AA	AA	AA	AA	AA
	AA	AA	AA	AA	AA	AA	AA	
55039427								
7901	rs10225245		7	GG	GG	GG	AG	AG
	AA	GG	GG	GG	GG	GG	GG	GG
	GG	GG	GG	GG	GG	--	AG	AG
	AG	AG	AG	AG	AA	AG	AA	AA
	GG	GG	AG	AG	GG	GG	GG	
148100862								
7902	rs1022525		X	GG	AG	AG	AA	AA
	AA	AA	AG	AG	GG	AA	AA	AA
	AG	AG	AG	AG	AG	AA	AA	AA
	AA	GG	AA	GG	AG	GG	GG	AA
	AG	AA	GG	AA	GG	GG	GG	
29567154								
7903	rs10225252		7	AA	AA	AA	AA	AA
	AA	AA	AA	AA	AA	AA	AA	AA
	AA	AA	AA	AA	AA	AC	AA	AA
	AC	AA	AA	AC	AA	AA	AA	AA
	AA	AA	AC	AC	AC	CC	AA	
108756678								
7904	rs10225279		7	AC	AC	AC	AA	AC
	AC	AA	AC	CC	AC	CC	AC	AC

	CC	AC	CC	AC	AC	AC	AC	AA
	CC	CC	CC	CC	AA	AC	AA	AA
	AA	AC	AC	AC	CC	AC	AC	
19133072								
7905	rs10225314		7	AA	AA	AA	AA	AA
	AA	AA	AA	AA	AA	AA	AA	AA
	AA	AA	AG	AA	AA	AA	AA	AA
	AA	AA	AA	AA	AA	AA	AA	AA
	AA	AG	AA	AA	AA	AA	AG	
99203653								
7906	rs1022533		22	GG	AG	GG	AG	AA
	AA	AG	AG	GG	GG	GG	GG	GG
	GG	AG	GG	AG	AG	GG	GG	GG
	GG	GG	AG	GG	GG	GG	GG	GG
	AG	GG	AG	GG	GG	GG	GG	
40972547								
7907	rs10225353		7	AG	GG	GG	AG	GG
	AG	GG	AG	GG	AA	AG	--	AG
	GG	GG	GG	GG	GG	AG	AG	GG
	GG	GG	AG	AG	GG	--	AG	AG
	--	GG	AA	AG	AG	--	AG	
62338799								
7908	rs10225406		7	AA	AA	AA	AA	AA
	AA	AA	AA	AA	AA	AA	AA	AC
	AA	AC	AA	AA	AA	AA	AA	AA
	AA	AA	AA	AA	AA	AA	AA	AA
	AA	AA	AA	AA	AA	AA	AA	
6480123								
7909	rs10225491		7	GG	GG	GG	GG	GG
	GG	GG	GG	GG	GG	GG	GG	GG
	GG	GG	GG	GG	GG	GG	GG	GG
	GG	GG	GG	GG	GG	GG	GG	GG
	GG	GG	GG	GG	GG	GG	GG	
64695598								
7910	rs10225492		7	AG	AG	GG	AA	GG
	AG	AG	AG	AA	GG	AA	AA	AA
	GG	GG	AA	AG	AA	AG	AG	AG
	AG	AG	GG	AA	AG	AA	AG	AG
	AG	AG	AG	GG	AG	GG	AG	
40058992								
7911	rs10225587		7	CC	AC	AC	AC	AC
	AA	AC	AA	AA	AA	CC	CC	CC
	CC	AC	CC	AC	AC	CC	CC	AC
	CC	AC	CC	CC	AA	AC	AA	AA
	CC	CC	CC	CC	CC	CC	CC	
126904423								
7912	rs1022565		20	GG	GG	GG	GG	AG
	AG	GG	GG	GG	GG	GG	GG	AG
	AG	AG	AG	GG	AG	AG	AA	AG
	GG	GG	AG	GG	AG	GG	GG	GG

GG GG GG GG AG GG AG
42466539
7913 rs10225710 7 AG AA AG GG GG
GG GG GG GG GG AA AA AA
AG AG GG GG AG AG AG AA
GG GG GG GG AG AG AG AA
AG GG AG GG AA AA AA
22142742
7914 rs10225713 7 AA AA AA AG AG
AA GG AA AA AG AA AA AA
AA AA AA AA AA AA AG AA
AG AG AA GG AA AA AA AA
AA AA AG AA AG AG AG
34445471
7915 rs10225728 7 CC CC CC CC CC
CC CC CC CC AC CC CC CC
CC CC CC AC AC CC CC CC
CC CC CC CC CC CC CC CC
CC CC CC CC CC CC CC
78608574
7916 rs10225755 7 AG AG GG AA AA
AA AG AA AA AA AA AA AA
AA AA AA AA AA AA AA AA
AG AA AA AG AA AG AG AG
AG AG AA AG AA AG AA
124165999
7917 rs10225784
7 -- -- -- -- -- -- -- --
-- -- -- -- -- -- -- --
-- -- -- -- -- -- -- --
-- -- -- -- -- -- -- --
-- -- -- -- 63996773
7918 rs10225792 7 GG GG GG GG AA
AG AG AG GG AG GG GG GG
GG GG AG AA AG GG GG AG
GG GG GG GG AG AG AA AG
AG AA AG AG AG AA AG
47614032
7919 rs10225837 7 AA AA AA AA AA
AA AA AA AA AA AA AA AA
AA AA AA AA AA AA AA AA
AA AA AA AA AA AA AA AA
AA AA AA AA AA AA AA
111731478
7920 rs10225856 7 AG AA AA AG AG
AA GG AG AG AG AA AA AA
AG AG AA AA AA AA AG AG
AG AG AG AG AG AA AA AA
GG AG GG AG AG AG AG
34505794

7921 rs1022597 20 GG GG GG GG AG
GG AG GG GG GG GG GG GG
GG GG GG GG GG GG GG GG
GG GG GG GG AG GG AG GG
GG GG GG GG GG GG GG
51639389
7922 rs10225980 7 AA AA AA AA AA
AA AA AA AA AA AA AA AA
AA AA AA AA AA AA AA AA
AA AA AA AA AA AA AA AA
AA AA AA AA AA AA AA
45757847
7923 rs1022600 20 CC AC CC CC AC
CC AC CC CC CC AC AC CC
AC AC CC CC CC AC AA AC
AA CC AC AC AC AC AC AA
AA CC AA AC CC CC CC
11057427
7924 rs10226014 7 AG AG GG GG AG
GG AG AG AG AG AG AG AG
AA AG GG AG AG AG AG GG
AG AG AA AG GG AA AG AG
AG AA AA AA AG AG AG
89902533
7925 rs10226025 7 AA AA AA AA AA
AA AA AA AA AA AA AA AA
AA AA AA AA AA AA AA AA
AA AA AA AA AA AA AA AA
AA AA AA AA AA AA AA
55871429
7926 rs10226087 7 GG GG AG GG GG
GG GG GG GG GG GG GG GG
GG GG GG AG GG AA GG AG
GG GG GG GG GG GG GG GG
GG GG AG AG AG GG AA
52607698
7927 rs1022611 6 GG GG GG AG AG
GG AG AA AA AG AG AA AA
AG AA GG GG AG GG GG AG
GG GG GG GG AG AG GG AG
GG GG AG GG AA AG AA
163622658
7928 rs1022614 6 AC AC CC CC CC
CC CC CC CC CC CC CC CC
CC CC AC AC AC AC AC AC
AC AC CC CC AC CC CC AC
CC CC CC CC CC CC CC
170683419
7929 rs10226190 7 AA AG AA AA AA
AA AA AA AA AA AA AA AA

	AA	AA	AA	AA	AA	AA	AA	AA
	AA	AA	AA	AA	AA	AA	AA	AA
	AA	AA	AA	AA	AA	AA	AA	
31861834								
7930	rs10226196		7	AC	CC	AC	CC	CC
	CC	CC	AA	AA	AC	CC	CC	CC
	CC	CC	AC	CC	AA	CC	CC	AC
	CC	AC	AC	AC	CC	CC	CC	CC
	CC	CC	CC	CC	AA	AC	AC	
15883552								
7931	rs10226243		7	AC	AA	AC	CC	AC
	CC	AC	AC	CC	AC	CC	CC	CC
	CC	CC	CC	AC	CC	AC	AC	CC
	CC	CC	CC	CC	AC	AA	AC	AA
	CC	CC	CC	CC	AC	CC	AC	
18968003								
7932	rs10226258		7	AA	AA	AG	AG	AA
	AA	AG	AG	AG	AG	AA	AA	AA
	AA	AA	AG	AG	AG	AG	AA	AG
	AA	AG	AA	AG	AA	AA	AA	AA
	AA	AA	AA	AA	AA	AA	AA	
25856295								
7933	rs10226263		7	CC	CC	CC	CC	CC
	CC	CC	CC	CC	CC	CC	CC	CC
	CC	CC	CC	CC	CC	CC	CC	CC
	CC	CC	CC	CC	CC	CC	CC	CC
	CC	CC	CC	CC	CC	CC	CC	
55319979								
7934	rs10226294		7	GG	GG	AG	AG	AG
	AA	GG	AG	GG	AG	GG	AG	AG
	AG	AG	GG	GG	GG	GG	GG	GG
	AG	GG	GG	AG	GG	AG	AG	AG
	GG	GG	GG	GG	GG	GG	GG	
66058336								
7935	rs1022630		6	GG	GG	GG	AG	AG
	AA	GG	GG	GG	GG	AG	AG	GG
	GG	GG	GG	GG	AG	AG	AG	GG
	AG	AG	AA	AG	GG	AG	GG	GG
	GG	AG	GG	AG	GG	AG	GG	
107189647								
7936	rs10226317		7	CC	CC	CC	CC	CC
	CC	CC	CC	CC	CC	CC	CC	CC
	CC	CC	AC	CC	CC	CC	CC	CC
	CC	AC	AC	CC	CC	CC	CC	CC
	CC	CC	AC	CC	AC	AC	CC	
62935337								
7937	rs1022632		20	AA	AA	AA	AC	AC
	AA	CC	AA	AA	AA	AC	AC	CC
	AC	AC	AC	AC	AC	AA	AC	AA
	AA	AC	AA	AA	AC	AC	AC	CC

	AC	AA	AC	AC	AC	AC	AA
7551917							
7938	rs10226320	7	AA	AA	AA	AA	AA
	AA	AA	AA	AA	AA	AA	AA	AA
	AA	AA	AA	AG	AG	AA	AA	AA
	AA	AA	AA	AA	AA	AA	AA	AA
	AA	AA	AG	AA	AA	AA	AA	
142761542								
7939	rs1022633	14	AA	AG	AG	AG	AA
	AG	AG	AA	AG	AA	AG	AA	AG
	AG	GG	AG	AA	AA	AG	AA	AA
	AA	AG	AG	AG	AG	AG	AG	AG
	AG	AG	GG	AG	AG	AG	AA	
99172380								
7940	rs10226429	7	AA	AA	AA	AA	AA
	AA	AA	AA	AA	AA	AA	AA	AA
	AA	AA	AA	AA	AA	AA	AA	AA
	AA	AA	AA	AA	AA	AA	AA	AA
	AA	AA	AA	AA	AA	AA	AA	
4983253								
7941	rs10226436	7	AA	AA	AG	AG	AG
	GG	AA	GG	GG	GG	AG	AG	AA
	AA	AA	GG	GG	AG	GG	AG	GG
	AG	GG	GG	AG	AA	AG	AA	AA
	AG	AG	AA	AA	AG	AA	GG	
26633863								
7942	rs10226440	7	AA	AA	AA	AC	AC
	CC	AA	CC	AC	CC	AA	AA	AA
	AA	AA	AA	AA	AA	AA	AA	AA
	AA	AA	AA	AA	AC	AA	AC	AC
	AC	AC	AC	AA	AC	AC	AA	
141888080								
7943	rs10226451	7	GG	GG	GG	GG	GG
	GG	GG	GG	GG	GG	GG	GG	GG
	GG	GG	GG	GG	GG	GG	GG	GG
	GG	GG	GG	GG	GG	GG	GG	GG
	GG	GG	GG	GG	GG	GG	GG	
54954317								
7944	rs1022646	6	GG	GG	GG	GG	GG
	GG	GG	AA	AA	AA	AG	AG	GG
	GG	GG	GG	GG	AG	GG	GG	GG
	GG	GG	GG	GG	GG	AG	GG	GG
	AG	GG	GG	GG	GG	GG	GG	
112029331								
7945	rs10226466	7	AC	AA	AC	AA	AA
	AA	AA	AA	AA	AA	AA	AA	AA
	AA	AA	AA	AA	AA	AA	AA	AC
	AA	AA	AA	AA	AA	AA	AA	AA
	AA	AA	AA	--	AA	AA	AC	
96357756								

7946 rs10226473 7 AG GG AG AA AA
AA AA AA AA AA AG AA AG
AG AA AA AA AA AA AG AA
AA AG AG AA AA AG AG AG
AG AG AA AA AA AG AA
102394076
7947 rs10226521 7 GG GG GG GG GG
GG GG GG GG GG AG AG AG
GG GG AG GG GG GG GG GG
AG AG AG AG GG GG GG GG
GG GG GG GG GG GG GG
1489229
7948 rs10226576 7 AA AA AA AA AA
AA AA AA AA AA AA AA AA
AA AA AA AA AA AA AA AA
AA AA AA AA AA AA AA AA
AA AA AA AA AA AA AA
55320838
7949 rs10226583 7 AA AA AA AA AC
AC AC AC AA AC CC AC AC
AC AA AA AA AA AC AC AA
AC AC AC AC AC AA AA AC
AC AA AA AA CC CC AC
68358970
7950 rs10226644 7 AA AA AA AA AA
AA AA AA AA AA AA AA AA
AA AA AA AA AA AA AA AA
AA AA AA AA AA AA AA AA
AA AA AA AA AA AA AA
5334782
7951 rs10226747 7 GG GG AG AG GG
AG GG GG GG GG GG GG GG
GG GG GG GG GG AG AG GG
GG GG GG GG GG GG GG GG
GG AG GG AG GG GG GG
41851037
7952 rs10226775 7 GG GG GG GG GG
GG GG GG GG GG GG GG GG
GG GG GG GG GG GG GG GG
GG GG GG GG GG GG GG GG
GG AG GG AG GG GG AG
148620408
7953 rs10226794 7 AA AA AA AA AA
AA AA AA AA AA AA AA AA
AA AA AA AA AA AA AA AA
AA AA AA AA AA AA AA AA
AA AA AA AA AA AA AA
18985390
7954 rs10226819 7 GG AG GG GG GG
GG GG AG AG GG GG GG GG

AG AA AG AA AG GG GG GG
GG GG GG GG AG GG AG GG
GG AA GG AG AG AG GG
15758899
7955 rs10227001 7 CC CC CC CC AC
AC CC CC CC CC CC CC CC
CC CC CC CC CC CC AC CC
AC AA AC CC CC CC CC CC
CC CC CC CC CC AC CC
63852916
7956 rs10227063 7 AA AA AA AA AG
AG AA AG AG AG AG GG AG
AG GG AA AG AG AG GG AA
AG GG AG AG AA AA AA AA
AA GG AG AG AA AA AA
121275477
7957 rs10227093 7 AA AA AA AA AA
AA AA AA AA AA AA AA AA
AA AA AA AA AA AA AA AA
AA AA AA AA AA AA AA AA
AA AA AA AA AA AA AA
37771035
7958 rs1022713 14 GG GG AG AG AA
AG AA GG AA GG AG AG GG
GG AG AA AG AA AG AA AG
AA AA AA AA AG AA AG AA
AA AA AA AA AA AA AG
39426108
7959 rs10227136 7 AC AA AC AA AA
AA AA AC CC CC AA AC AC
AC AC AC AA AA AC AC CC
AA CC AA AA CC CC CC CC
AA AC AA AA AC AC CC
151125123
7960 rs10227141 7 GG GG GG GG GG
GG GG GG GG GG GG GG GG
GG GG GG GG GG GG GG GG
GG GG GG GG GG GG GG GG
GG GG GG GG GG GG GG
76019590
7961 rs10227167 7 AA AA AA AA AA
AA AA AA AA AA AA AA AA
AA AA AA AA AA AA AA AA
AA AG AA AA AA AA AA AA
AA AA AG AA AG AG AA
157631868
7962 rs10227226 7 GG GG AG AA AA
AA AA GG GG GG GG AG AG
AA AA AA AA AG GG AG GG
GG GG GG GG AG AA AG AA

GG AG AG AA AG AG AG
54678150
7963 rs1022723 14 GG GG AG AG AA
AG AG AA AG AA AG AG AG
AG GG AG AA AA AG AG GG
GG AA AG AG AA AA AA AA
AG AA GG AG GG GG GG
60357110
7964 rs10227239 7 AA AA AA AA AA
AA AA AA AA AA AA AA AA
AA AA AA AA AA AA AA AA
AA AA AA AA AA AA AA AA
AA AA AA AA AA AA AA
93090586
7965 rs10227240 7 AA AA AA AA AA
AA AA AA AA AA AA AA AA
AA AA AA AA AA AA AA AA
AA AA AA AA AA AA AA AA
AA AA AA AA AA AA AA
19406054
7966 rs10227293 7 CC CC CC CC AC
AC CC CC CC CC CC CC CC
CC AC CC CC CC CC AC AC
CC CC CC CC CC AC AC AC
CC CC CC CC CC CC AC
97431234
7967 rs10227301 7 AA AA AA AA AA
AA AA AA AG AA AA AA AA
AA AA AA AA AA AA AG AA
AA AA AA AA AA AG AG AG
AA AA AA AA AA AA AG
19439393
7968 rs1022732 20 AG GG AG AA AG
AG AA AA AA AA AA AA AA
AA AA AA AG AA AG GG AA
AG AG AA AG AG GG GG GG
GG AA AG AA AG AA GG
31152094
7969 rs10227322 7 AA AA AA AA AA
AA AA AA AA AA AA AA AA
AA AA AA AA AA AA AA AA
AA AA AA AA AA AA AA AA
AA AA AA AA AA AA AA
77201995
7970 rs10227349 7 AA AA AA AA AA
AA AA AA AA AA AA AA AA
AA AA AA AA AA AA AA AA
AA AA AA AA AA AA AA AA
AA AA AA AA AA AA AA
64555184

7971 rs10227381 7 AG GG GG AG AG
AA GG AA AA AG AA AG AG
GG GG GG AG GG GG AA GG
GG GG AG AG GG AA AG AG
AG AG AG GG AG AA GG
49041510
7972 rs1022749 9 AG AG AG AA AG
AG AA AG AG AA AA AA AA
AA AA AA AG AA AG AG AA
AA AA AA AA AG AA AG AG
AA AA AA AA GG AG AG
104595776
7973 rs10227532 7 AG GG GG AG AG
AA GG AA AA AG AA AG AG
GG GG GG AG GG GG AA GG
GG GG AG AG GG AA AG AG
AG AG AG GG AG AA GG
49048208
7974 rs10227709 7 CC CC CC AA AC
AC AA AA AC AA AA AC AA
AC AC AA AA AC AC AC AA
AA AA AA AA AC AC AC AC
AA AC AC AA AA AA AA
1180515
7975 rs10227851 7 AA AA AA AA AA
AA AA AG AG AG AA AG AG
AG AA AG AG AG AA AG AA
GG GG AG AG AA AA AA AA
AA AA AA AA AA AG AA
79973826
7976 rs1022787 6 AG AG AA GG AG
GG AG GG GG GG AG AG AG
AG AG AA AG AA AG AG GG
AA AA AA AA AG AA AG AG
GG AG GG GG AA AA AG
95202197
7977 rs10227895 7 GG GG GG GG GG
GG GG GG GG GG GG GG GG
GG GG GG GG GG AG GG GG
GG GG GG GG GG GG GG GG
GG AG GG GG GG GG AG
62534685
7978 rs1022790 20 AG AA AA AG AA
AA AG GG AG AG AA AA AA
AA AG AA AG AA GG AG GG
AA AA AA AA AG AA AG AA
AG AG AA AG AG AA GG
10730759
7979 rs10227915 7 AA AA AG AA AG
AA AG AA AA AA GG AG AG

AG AG AA AA AA AA AA AA
AA AA AA AA AG AA AG AG
AA AA AA AA AA AG AG
39431588
7980 rs10227958 7 AA AA AA AA AA
AA AA AA AA AA AA AA AA
AA AA AA AA AA AG AA AG
AA AA AA AA AA AA AA AA
AA AA AA AA AG AA AG
17008701
7981 rs10227970 7 AG AG GG AG AG
AG AG AG AG AG AA AA AG
AG AG AG GG AG GG AG AG
AG GG AG GG AG GG GG GG
AA AA AG AA AG AG GG
34478120
7982 rs10227993 7 CC CC CC CC CC
CC CC CC CC CC CC CC CC
CC CC CC CC CC CC CC CC
CC CC CC CC CC CC CC CC
CC CC CC CC CC CC CC
17164027
7983 rs10228007 7 AA AA AA AA AA
AA AA AA AG AA AA AA AA
AA AA AA AA AA AG AG AA
AA AA AA AA AA AA AA AA
AA AA AA AA AA AA AA
148958052
7984 rs10228010 7 GG GG GG GG GG
GG GG GG GG GG GG GG GG
GG GG GG GG GG GG GG GG
GG GG GG GG GG GG GG GG
GG GG GG GG GG GG GG
158697604
7985 rs10228025 7 AG AG AG GG AA
AG AG AG AA AG AA AG AG
GG GG AG GG GG GG AA AG
AA AG AA AG AG AA AA AA
GG AG GG GG AG AA GG
40789894
7986 rs10228030 7 CC CC CC CC CC
CC CC CC CC CC CC AC AC
AA AC CC CC CC CC CC CC
AC CC AC AC CC CC CC CC
CC CC CC CC CC CC CC
157111112
7987 rs1022804 14 GG AG AG GG GG
GG GG AG AG AG AG GG AG
AG AG AG AA AG AG GG AG
GG GG GG GG GG GG GG GG

AG GG GG GG GG AG GG
38180813
7988 rs10228050 7 AA AA AA AA AA
AA AA AA AA AA AA AA AA
AA AA AA AA AA AA AA AA
AA AA AA AA AA AA AA AA
AA AA AA AA AA AA AA
40081265
7989 rs10228095 7 AA AA AA AA AA
AA AA AA AA AA AA AA AA
AA AA AA AA AA AA AA AA
AA AA AA AA AA AA AA AA
AA AA AA AA AA AA AA
154058755
7990 rs10228122 7 AA AA AA AG AA
AG AG AG AG AG AG AG GG
GG AG AA AA AA AG GG AG
AG AA AG AG GG AA AG AG
AG AG AA AG GG AG AG
121300485
7991 rs10228200 7 AA AA AA AA AA
AA AA AA AG AG AA AA AA
AA AA AA AA AA AG AA AA
AA AA AA AA AA AA AA AA
AA AA AA AA AA AA AG
153368459
7992 rs10228214 7 CC CC AC CC CC
CC CC AC AC AC CC CC CC
AC AC AC AC AC AA CC AC
AC AC AC AC AC CC AC CC
CC CC CC CC CC AC CC
54134202
7993 rs10228266 7 AG AA AG AG GG
AG AG GG GG GG GG GG GG
GG GG GG GG GG AG AG GG
AG AG AG AG AG AG GG GG
GG AG AA AA GG GG GG
113347199
7994 rs10228308 7 GG AG AG AA AA
AA AA AA AA AG AA AA AA
AA AA AA AA AA AG AG AG
AA AA AG AG AA AG AA AA
AG AA AA AA AA AG AA
66515614
7995 rs10228327 7 AA AG AG AG GG
AG AG AG AG AG AG AA AG
AG AA AG AA AA AG AG AG
AA AG AG AA AG AA AA AA
AA AG GG AG AA AG AA
114104389

7996 rs10228341 7 AA AA AA AA AA
AA AA AA AA AA AA AA AA
AA AA AA AA AA AA AA AA
AA AA AA AA AA AA AA AA
AA AA AA AA AA AA AA
18920499
7997 rs10228360 7 GG AG AG GG AG
GG AG GG GG AG GG GG GG
GG GG GG GG GG AG AG AG
GG GG AG GG GG GG GG GG
AG AG GG GG GG GG GG
97000087
7998 rs10228393 7 GG GG GG GG GG
GG GG AG AG GG AG AG GG
GG AG GG GG GG AG AG GG
GG GG AG GG AG AA AG AG
AG AG AG AA AG GG AA
95088489
7999 rs10228406 7 GG GG GG AG GG
GG AG GG GG GG GG GG GG
GG GG GG AG GG AG AG AG
GG GG GG GG AG AG AA AG
AG AG GG AG GG GG GG
19133464
8000 rs10228407 7 AA AC AA AC CC
AC CC CC CC CC AC AA AC
AA AA AC AC AC CC AC AC
AA AA AA AA AA AC AA AA
AA AA AA AA AA AC AC
143127771
8001 rs10228472 7 AA AA AA AA GG
AG AG AG AG AG AA AA AA
AA AA AG GG GG GG AA AG
AG GG AG AG GG AG GG GG
AA AA AG AA AA AG AA
95711278
8002 rs1022849 14 GG GG GG GG GG
GG GG GG GG GG GG AG GG
GG GG GG GG GG GG GG AG
GG GG GG GG GG AG GG AG
GG GG GG GG AG GG AG
86267543
8003 rs10228495 7 AA AA AA AG AG
AA GG AG AG AG AA AG AG
AG AG AA AG AG GG AA AG
AG AA AG AG AG AG AA AA
AA AA AA AA AG AA AG
102440184
8004 rs10228514 7 AC AC AC AA AA
AA AA AA AA AA AA AA AA

AA AA AA AA AA AA AA AC
AA AA AA AA AC AA AC AC
AA AA AA AA AA AA AC
35463795
8005 rs10228515 7 AA AC AA AA AA
AA AC CC AC AC AC AC AA
AA AA CC CC CC AC AA AC
AA AC AA AC AA AA AA AA
AA AA AC AA AC AC AC
12013256
8006 rs10228546
7 -- -- -- -- -- -- -- --
-- -- -- -- -- -- -- --
-- -- -- -- -- -- -- --
-- -- -- -- -- -- -- --
-- -- -- -- 153451431
8007 rs10228558 7 GG AG GG AA AG
AA AG AA AA AA AA AA AA
AG AG AA AA AA AA AG AG
AA AA AA AA AA AA AA AA
AG AG AG AA AA AA AA
19911915
8008 rs10228575 7 GG GG GG GG GG
GG GG AG GG AA AG GG AG
AG AG GG GG GG GG AG GG
AG GG AG AG GG GG GG GG
AG AG GG GG GG GG GG
156810665
8009 rs10228625 7 AA AA AA AA AA
AA AA AA AA AA AA AA AA
AA AA AA AA AA AA AA AA
AA AA AA AA AG AA AG AG
AA AA AA AA AA AA AA
103728400
8010 rs10228665 7 GG GG GG GG GG
GG GG AG GG AA AG AG GG
GG GG GG GG GG GG GG GG
GG GG GG GG GG GG GG GG
GG GG GG GG GG GG GG
62560780
8011 rs10228666 7 GG GG GG GG AG
AG GG GG GG GG GG GG GG
GG GG GG GG GG GG AG GG
GG AG AG GG AG GG AG GG
GG GG GG GG GG GG GG
64076602
8012 rs1022874 13 AG AG AA AG GG
AG AG AG AG AG AG AA AG
AA AA AA AA AA AA AG AG
AG AG AA AG GG AG AG AG

GG AG AG AA AG AG AG
107933487
8013 rs10228755 7 GG AG AG GG AG
AG GG GG GG GG AG AG AG
AG GG GG GG GG GG GG GG
GG AG AG GG GG GG GG GG
GG AG GG GG AG AG GG
93890183
8014 rs10228761 7 GG GG GG GG GG
GG GG GG GG GG GG GG GG
GG GG GG GG GG GG GG GG
GG GG GG GG GG GG GG GG
GG GG GG GG GG GG GG
73826693
8015 rs10228782 7 GG GG GG GG GG
GG GG GG GG GG GG GG GG
GG GG GG GG GG GG GG GG
GG GG GG GG GG GG GG GG
GG GG GG GG GG GG GG
960412
8016 rs10228817 7 AA AA AA AA AA
AA AA AA AA AA AA AA AA
AA AA AA AA AA AA AA AA
AA AA AA AA AA AA AA AA
AA AA AA AA AA AA AA
2350750
8017 rs10228823 7 AA AA AA AA AC
AA AC AA AA AA AA AA AA
AA AA AC AC AC AC AA AA
AA AA AA AA AA AA AA AA
AA AC AA AC AC AA AC
44082242
8018 rs10228842 7 GG GG GG GG GG
GG GG GG GG GG GG GG GG
GG GG GG GG GG AG AG AG
GG GG GG GG AG GG GG AG
GG GG GG GG GG GG AG
11965352
8019 rs10228847 7 AG AG AA AG GG
AG AG AA AA AA AA AG AG
AG AG AA AA AA AA AA AA
AA AA AA AA AA AA AA AA
AG AA AA AA AA AA AG
108328266
8020 rs10228870 7 GG GG GG GG GG
GG GG GG GG GG GG GG GG
GG GG GG GG GG GG GG GG
GG GG GG GG GG GG GG GG
GG GG GG GG GG GG GG
92959070

8021	rs10229026		7	AG	AG	AG	GG	GG
	GG	GG	GG	GG	GG	AG	AG	GG
	AG	AA	GG	AG	AG	AG	AG	AG
	GG	GG	GG	GG	AG	AG	AG	AG
	AG	GG	GG	GG	GG	GG	GG	
157400389								
8022	rs10229063		7	GG	GG	GG	GG	GG
	GG	GG	GG	GG	GG	GG	GG	GG
	GG	GG	GG	GG	GG	GG	GG	GG
	GG	GG	GG	GG	GG	GG	GG	GG
	GG	GG	GG	GG	GG	GG	GG	
107099568								
8023	rs1022907		10	AG	GG	GG	GG	GG
	GG	GG	AG	AG	GG	AG	AG	AA
	AG	GG	AG	AA	GG	AG	AG	AG
	GG	GG	GG	GG	GG	AA	AG	AG
	GG	GG	GG	GG	AG	GG	AA	
114394794								
8024	rs1022909		13	AG	GG	AG	AG	AG
	AG	GG	GG	GG	GG	GG	GG	GG
	GG	GG	AG	GG	AG	GG	GG	GG
	GG	GG	GG	GG	GG	AG	GG	GG
	GG	GG	AG	GG	GG	GG	GG	
108670773								
8025	rs10229091		7	AA	AA	AA	AG	AG
	GG	AG	AG	AA	AG	AG	AA	AG
	AG	AG	AG	GG	GG	GG	GG	GG
	GG	AG	GG	GG	AG	GG	GG	GG
	AG	GG	GG	GG	GG	GG	GG	
156584904								
8026	rs10229206		7	GG	AG	AG	GG	GG
	GG	GG	AG	AG	GG	GG	GG	GG
	GG	AA	GG	AG	AG	AG	AG	AG
	GG	GG	GG	GG	AG	AG	AG	GG
	AG	AG	AG	AA	GG	GG	GG	
79585451								
8027	rs10229228		7	CC	AC	AC	AC	AC
	AC	AC	CC	CC	AC	CC	AC	AC
	AC	AA	AC	CC	CC	AC	AA	AC
	AC	AC	CC	CC	CC	AC	AC	AC
	CC	AC	CC	AC	AC	AC	AC	
80998755								
8028	rs10229230		7	AG	AA	AG	AA	AA
	AA	AA	AA	AA	AA	AG	GG	AG
	AA	AA	AG	GG	AG	AG	AG	AG
	GG	AG	GG	AG	GG	AA	AG	AG
	AG	AA	AG	AG	AA	AA	AG	
14279074								
8029	rs10229310		7	AG	GG	GG	AG	AA
	AG	AA	AG	GG	AG	GG	GG	GG

	GG	GG	AG	AG	AG	GG	AG	AG
	GG	GG	GG	GG	AA	AG	AA	AA
	AG	AG	GG	GG	AG	GG	AA	
134417742								
8030	rs10229388		7	AA	AG	AA	AA	AA
	AA	AA	AA	AG	AG	AG	AG	GG
	GG	GG	AA	AA	AA	AA	AG	AA
	AG	AA	AG	AG	AG	GG	AG	AG
	AG	AA	AG	AG	AA	AG	AA	
151705546								
8031	rs10229392		7	AG	AA	AG	AA	AG
	AG	AA	AG	AG	GG	AG	AG	AA
	AA	AG	AA	AA	AA	AG	AG	AG
	AA	AA	AA	AA	AA	AA	AA	AA
	AA	AA	AG	AG	AA	AA	AA	
82328200								
8032	rs10229478		7	GG	GG	GG	GG	GG
	GG	GG	GG	GG	GG	GG	GG	GG
	GG	GG	GG	GG	GG	GG	GG	GG
	GG	GG	GG	GG	GG	GG	GG	GG
	GG	GG	GG	GG	GG	GG	GG	
51214940								
8033	rs1022949		9	AA	AA	AA	AG	AG
	AA	AG	AA	AA	AG	AA	AA	AA
	AA	AA	AA	AA	AG	AA	AA	AA
	AA	AA	AA	AA	AA	AA	AA	AA
	AA	AG	AA	AA	AA	AA	AA	
116545758								
8034	rs10229500		7	GG	GG	GG	GG	AG
	AG	GG	AG	GG	AG	AA	AA	AA
	GG	AG	AG	GG	GG	GG	GG	GG
	AG	AA	AG	AA	GG	GG	GG	GG
	AG	GG	GG	GG	GG	GG	GG	
28389465								
8035	rs10229502		7	AA	AA	AA	AA	AA
	AA	AA	AA	AA	AA	AA	AA	AA
	AA	AA	AA	AA	AA	AA	AA	AA
	AA	AA	AA	AA	AA	AA	AA	AA
	AA	AA	AA	AA	AA	AA	AA	
74488253								
8036	rs10229537		7	GG	GG	AG	GG	GG
	GG	GG	GG	GG	AG	GG	GG	GG
	GG	GG	GG	AG	AG	GG	GG	GG
	GG	GG	GG	GG	GG	AG	GG	GG
	GG	GG	GG	GG	GG	GG	AG	
71797644								
8037	rs10229551		7	GG	GG	GG	GG	GG
	GG	GG	GG	GG	GG	GG	GG	GG
	AG	AG	GG	GG	GG	GG	AG	GG
	GG	GG	GG	GG	GG	GG	GG	GG

	GG	AG	GG	AG	GG	GG	GG
42559775							
8038	rs1022958		13	AC	AC	AA	AC	CC
	CC	AC	CC	CC	CC	AC	CC	CC
	CC	CC	CC	CC	CC	CC	CC	CC
	CC	CC	CC	CC	CC	CC	CC	CC
	CC	CC	CC	CC	CC	CC	CC	
72661436								
8039	rs10229581		7	GG	GG	GG	AA	AG
	AG	AA	GG	GG	GG	GG	GG	GG
	AG	AG	AG	AA	AG	AG	GG	GG
	GG	GG	GG	GG	AG	GG	AG	GG
	AG	GG	GG	GG	AG	GG	AA	
46802560								
8040	rs10229585		7	AA	AA	AA	AA	AA
	AA	AA	AA	AA	AA	AA	AA	AA
	AA	AA	AA	AG	AA	AA	AA	AA
	AA	AA	AA	AA	AA	AA	AA	AA
	AG	AA	AG	AA	AA	AA	AA	
11597387								
8041	rs10229649		7	GG	GG	GG	GG	GG
	GG	GG	GG	GG	GG	GG	GG	GG
	GG	GG	GG	GG	GG	GG	GG	GG
	GG	GG	GG	GG	GG	GG	GG	GG
	GG	GG	GG	GG	GG	GG	GG	
53103257								
8042	rs10229660		7	AC	AA	AC	CC	AC
	AC	CC	AA	AA	AA	AA	AC	AC
	AA	AA	CC	AC	CC	AA	AA	AA
	CC	AC	AC	CC	AC	AC	AC	CC
	AC	AC	AA	AC	AC	AC	AC	
42304214								
8043	rs10229699		7	AA	AA	AA	AA	AA
	AA	AA	AA	AA	AA	AA	AA	AA
	AA	AA	AA	AA	AA	AA	AA	AA
	AA	AA	AA	AA	AA	AA	AA	AA
	AA	AA	AA	AA	AA	AA	AA	
100688994								
8044	rs10229707		7	GG	GG	AG	GG	GG
	GG	GG	GG	GG	GG	GG	GG	GG
	GG	GG	AG	GG	GG	GG	GG	GG
	GG	GG	GG	GG	GG	AG	GG	GG
	GG	GG	GG	GG	GG	GG	GG	
71940548								
8045	rs10229725		7	AA	AA	AA	AA	AA
	AA	AA	AA	AA	AA	AA	AA	AA
	AA	AA	AA	AA	AA	AA	AA	AA
	AA	AA	AA	AA	AA	AA	AA	AA
	AA	AA	AA	AA	AA	AA	AA	
112579920								

8046 rs10229727 7 GG GG GG GG GG
GG GG GG GG GG GG GG GG
GG GG GG GG GG GG GG GG
GG GG GG GG GG GG GG GG
GG GG GG GG GG GG GG
75914683
8047 rs10229737 7 AA AG AG AA AA
AA AA AG AG AA AA AA AA
AA GG AA AG AG AG AG AG
AA AA AA AA AG AG AG AA
AG AG AG GG AA AA AA
79594834
8048 rs1022974 1 AG AG AG AA AG
AA AG AA AA AA AA AA AA
AG AA AA AA AG AA AA AA
AA AG AA AG AA AG AG AG
AA AA AG AA AG AG AG
82469609
8049 rs10229831 7 GG GG GG GG GG
GG GG GG GG GG GG GG GG
GG GG GG GG GG GG GG GG
GG GG GG GG GG GG GG GG
GG GG GG GG GG GG GG
49232572
8050 rs1022984 6 AG GG AG -- --
-- GG AG GG AG GG GG GG
-- -- AG AG AG GG GG --
-- -- AA AG GG -- -- --
GG AG AG AG AG AG AA
156921064
8051 rs1022989 13 CC AC CC AA AA
AA AC CC CC CC CC CC AC
CC CC CC CC CC CC CC CC
CC CC CC CC AA CC AC AC
CC CC CC CC CC AC CC
40869048
8052 rs10229918 7 GG GG AG AG AG
AG AG AG AA AA AA AA AG
AA AA AG AA AA AG AA AA
AA AG AA AG AA AG AA AA
AA AG AA AG AA AA AA
31573901
8053 rs1022994 9 AA AA AA AC AC
AC AC AC CC CC AC AC AC
AC CC CC CC CC AA AC AA
AA AC AC AC AC AA AC AA
AC AC AC AA CC CC AC
89537928
8054 rs1023000 9 AA AG AG AG AG
GG AA GG AG AG AG AG AA

AA AA AG AG AG AA AG AA
GG GG GG GG AG AG AG GG
AA AG GG AG GG GG GG
132662786
8055 rs10230001 7 GG GG GG GG GG
GG GG GG GG GG GG GG GG
GG GG GG GG GG GG GG GG
GG GG GG GG GG GG GG GG
GG GG GG GG GG GG GG
121619616
8056 rs10230004 7 CC AC AC CC CC
CC CC AC CC AC CC CC CC
CC CC CC CC CC CC CC CC
CC CC CC CC CC CC CC CC
CC CC CC CC CC CC CC
135573783
8057 rs10230036 7 AG AA AG AA AG
AG AA AA AA AA AG AG AA
AA AG AG AG AG AA AA AA
AA AA AA AA AA AA AA AA
AA GG AA AG AG AG AA
63442188
8058 rs10230064 7 AG AG AA AG AG
AG AG GG GG GG AA AG AG
GG GG AG GG GG AG GG AG
GG GG GG GG AG GG AG AG
AA GG GG GG GG GG GG
38483081
8059 rs10230144 7 GG GG GG GG GG
GG GG GG GG GG GG GG GG
GG GG GG GG GG GG GG GG
GG GG GG GG AG GG GG GG
GG GG GG GG GG GG GG
39113120
8060 rs10230205 7 AG AA AG AG AG
GG AA AG AG AG GG GG GG
GG GG AA AA AA GG GG GG
AG AG AA GG AG GG GG AG
AG AG AG AG GG GG GG
20726832
8061 rs10230224 7 GG GG GG GG GG
GG GG GG GG GG GG GG GG
GG AG GG GG GG GG GG GG
GG GG GG GG GG GG GG GG
GG GG AG AG GG GG GG
98311136
8062 rs10230246 7 AA AA AA AA AG
AG AA AG AG AG AA AA AA
AG AG AA AA AA AG AA AG
AG AA AG AA AG AA AA AG

AA AG AG AG AA AA AG
21836072
8063 rs10230326 7 GG GG GG GG GG
GG GG GG GG GG GG GG GG
GG GG GG GG GG GG GG GG
GG GG GG GG GG GG GG GG
GG GG GG GG GG GG GG
146461144
8064 rs10230368 7 AG AA AG AA AG
AG AG AA AA AG AA AG AG
AG AG AA AA AA AA AG AG
AA AG GG AG GG AA AG AG
AA GG AA AG AG AG AA
79809984
8065 rs10230371 7 AA AG AA AG AG
GG AA GG AG GG AG AG GG
GG GG AA AA AG GG AG GG
AA GG AG AG AG GG GG AG
AA AG AA AG AG AG AA
18894897
8066 rs10230385 7 AG AG AG AG AA
AA AG AG AA AG AA AG AG
AA AA AA AA AA AA AA AA
AG AG AG AG AG AA AA AG
AG AA AA AA AG AA AG
50452552
8067 rs10230426 7 AC AC AC CC AC
AC CC CC CC CC CC CC CC
CC CC CC CC CC AC AC CC
CC CC CC CC CC CC CC CC
AC CC CC CC CC AC CC
18733158
8068 rs1023045 10 GG GG GG AG GG
AG GG GG GG AG GG GG AG
AG GG AG GG GG AG GG GG
GG GG GG GG GG GG GG GG
AG GG AG AG AG AA AG
125093310
8069 rs10230479 7 AA AC AA AA AA
AA AA AA AA AA AC AA AC
AC AC AA AA AA AC AC AC
AA AA AA AA CC AA AC AC
AC AA AA AA AA AA AA
146575682
8070 rs10230566 7 GG GG GG GG GG
GG GG GG GG GG GG GG GG
GG GG GG GG GG GG GG GG
GG GG GG GG AG GG AG GG
GG GG GG GG AG GG AG
64844720

8071 rs10230651 7 GG GG GG AG GG
AG GG AG GG GG GG GG GG
AA AG GG GG AG AG AG GG
AA AG AA AG GG AG AG AG
AG GG AG AG GG GG AG
7688023
8072 rs10230686 7 AA AA AA AA AA
AA AA AA AA AA AG AA AG
AG AA AA AG AA AA AA AA
AA AA AA AA AG AA AA AG
AG AA AA AA AA AA AA
13641674
8073 rs10230692 7 AG AA AG AA AA
AA AA AA AA AA AA AA AA
AA AA AG GG GG AA AA AG
AA AA AA AA AA AA AA AA
AA AA AG AG AA AA AA
139421180
8074 rs10230714 7 AA AA AA AA AA
AA AA AA AA AA AA AA AA
AA AA AA AA AA AA AA AA
AA AA AA AA AA AA AA AA
AA AA AA AA AG AG AA
94540416
8075 rs10230764 7 GG GG GG GG GG
GG GG GG GG GG GG GG GG
GG GG GG GG GG GG GG GG
GG GG GG GG GG GG GG GG
GG GG GG GG GG GG GG
2980708
8076 rs10230845 7 AG AG AG GG GG
GG GG GG AG GG GG GG GG
AG AG GG GG GG AG GG GG
GG GG GG GG GG GG GG GG
GG GG AG AG AG AG AG
55905896
8077 rs10230847 7 GG GG GG GG GG
GG GG GG GG GG GG GG GG
GG GG GG GG GG GG GG GG
GG GG GG GG GG GG GG GG
GG GG GG GG GG GG GG
1894435
8078 rs1023095 8 AG AG AG AG AA
AG AG GG GG GG AA AA AG
AG GG GG GG AG GG GG AG
AG GG AG AG AG AG AA AG
GG AG GG GG AG AG AA
132022957
8079 rs10230969 7 AG GG GG GG GG
AG GG GG AG AG AG GG AG

	GG	GG	GG	GG	GG	GG	GG	GG
	AG	AG	AG	AG	GG	GG	GG	GG
	GG	AG	AG	GG	AA	AG	AG	
153976598								
8080	rs10231055		7	AC	CC	CC	CC	AC
	AC	CC	CC	AC	CC	CC	AC	AC
	CC	CC	CC	AC	CC	CC	CC	CC
	CC	CC	AC	AC	CC	CC	CC	CC
	CC	CC	AC	AC	AC	CC	AC	
53553734								
8081	rs1023106		6	AA	AA	AA	AA	AG
	AG	AA	AA	AG	AA	AA	AA	AG
	AA	AA	AG	GG	AA	AG	AG	GG
	AG	AG	AG	AG	GG	AG	AG	AG
	AA	AA	AA	AA	AG	AG	AA	
120250121								
8082	rs1023107		6	AC	AC	CC	CC	AC
	AC	CC	AC	AA	AC	AA	CC	AC
	AC	AC	AC	CC	CC	AC	AC	AC
	CC	CC	CC	CC	AA	CC	AC	AC
	AA	CC	AC	AC	CC	CC	AC	
14030470								
8083	rs10231084		7	GG	GG	GG	GG	GG
	GG	GG	GG	GG	GG	GG	GG	GG
	GG	GG	GG	GG	GG	GG	GG	GG
	GG	GG	GG	GG	GG	GG	GG	GG
	GG	GG	GG	GG	GG	GG	GG	
92799552								
8084	rs10231133		7	AA	AA	AG	AA	AA
	AG	AA	AA	AG	AG	GG	AG	AG
	AA	AG	AG	AG	AG	AA	AG	AG
	AA	GG	AA	AA	AA	AG	AG	AG
	AG	GG	AG	AG	AG	AA	AG	
150223136								
8085	rs1023120		14	CC	CC	CC	CC	CC
	CC	CC	CC	CC	CC	CC	CC	CC
	CC	CC	CC	CC	CC	CC	CC	CC
	CC	AC	AC	AC	CC	AC	AC	CC
	CC	CC	CC	CC	CC	CC	CC	
56777393								
8086	rs10231201		7	GG	GG	GG	GG	GG
	GG	GG	GG	GG	GG	GG	GG	GG
	GG	GG	GG	GG	GG	GG	GG	GG
	GG	GG	GG	GG	GG	GG	GG	GG
	GG	GG	GG	GG	GG	GG	GG	
72906228								
8087	rs10231253		7	AC	AA	AC	AC	AC
	AA	CC	CC	CC	CC	AC	AC	CC
	CC	AC	CC	CC	CC	AA	CC	AC
	CC	AC	CC	CC	CC	CC	CC	CC

	CC	CC	CC	AA	AC	AC	
157983353							
8088	rs1023128	21	AG	GG	AG	AG	GG
	GG	AG	AA	AG	AG	AA	AA	AA
	AA	AA	AA	AA	AA	AG	AA	AA
	AG	AG	AG	AG	AA	GG	AG	AG
	AA	AA	GG	AG	GG	AG	GG	
20848066							
8089	rs10231308	7	GG	AG	AA	AA	AA
	AA	AA	AG	AG	AA	AA	AA	AA
	AG	GG	AA	AA	AA	AG	GG	AA
	AA	AA	AG	AG	AG	GG	AG	GG
	AG	AA	AA	AA	AA	AA	AA	
76005696							
8090	rs10231310	7	AC	CC	AC	AC	AC
	AC	AC	AA	AC	AA	CC	CC	CC
	AC	AC	CC	AC	AC	AC	AC	CC
	AA	AC	AA	AC	AA	CC	AC	AC
	AC	CC	AC	CC	AC	AC	CC	
24499951							
8091	rs10231383	7	GG	AG	AG	GG	GG
	GG	AG	GG	AG	GG	GG	GG	GG
	GG	GG	GG	GG	GG	GG	GG	GG
	GG	GG	GG	GG	AG	GG	GG	GG
	GG	GG	AG	AG	GG	GG	GG	
131385220							
8092	rs10231433	7	AG	AG	AA	AG	AA
	AA	AG	AG	AG	AG	AG	AG	AA
	AA	AA	AG	AG	AG	AA	AG	AA
	AA	AA	AG	AG	AA	AG	AA	AA
	AA	AA	AA	AA	AG	AG	AA	
82222805							
8093	rs1023148	21	AG	AG	AG	AG	AG
	AG	AG	AA	AA	AA	GG	AG	AG
	AG	AA	AG	AG	AG	AG	AG	AG
	AG	AA	AA	AA	AA	AG	AG	AA
	GG	AA	GG	AG	AG	AA	AG	
37002204							
8094	rs10231529	7	AA	AA	AA	AG	AG
	AG	AG	AA	AA	AA	AA	AA	AA
	AG	AA	AG	AA	AA	AG	AG	AA
	AA	AA	AA	AA	AA	AA	AA	AA
	AG	AG	AA	AG	AA	AA	AA	
100721337							
8095	rs10231578	7	AG	GG	GG	AG	AG
	GG	AA	AA	AG	AA	AG	AG	AA
	AA	AA	AA	AG	AG	AA	AA	AA
	AG	AA	AA	AG	AG	AA	AG	AA
	AA	AA	AG	AG	GG	GG	AG	
27929030							

8096 rs10231613 7 GG GG GG AG GG
GG AG GG GG GG GG GG GG
GG GG GG GG GG GG GG AG
GG GG GG GG GG GG GG GG
GG GG GG GG GG GG GG
56207415
8097 rs10231616 7 GG GG GG GG GG
GG GG GG GG GG GG GG GG
GG GG GG GG GG GG GG GG
GG GG GG GG GG GG GG GG
GG GG GG GG GG GG GG
151934558
8098 rs1023166 13 AG AA AG AG AG
AA AG GG GG AG AA AA AA
AA AA AG AA AG GG GG AG
AA AA AA AA AG AG AG AG
AA AG AA AA GG AG GG
29845810
8099 rs10231668 7 GG GG GG GG GG
GG GG GG GG GG GG GG GG
GG GG GG GG GG GG GG GG
GG GG GG GG GG GG GG GG
GG GG GG GG GG GG GG
52301000
8100 rs10231676 7 AA AA AA AA AA
AA AA AC CC AC AA AA AA
AA AA AA AC AC AA AA AC
CC AA CC CC CC AA AC AC
AC AA AC AA AC AC AA
132056155
8101 rs10231685 7 AA AA AA GG AA
AG AG AA AA AA AG AA AG
GG AA AA AG AG AA AA AA
AA AA AA AA AG AG GG GG
AG AA AA AA AG AG AA
104176289
8102 rs10231717 7 AA AC AC AA AA
AA AA AC AC AC CC CC CC
CC AC AC CC CC CC CC CC
CC CC CC CC AA CC AC AC
AA AC AC AC CC CC CC
123218522
8103 rs10231785 7 GG GG GG GG GG
GG GG GG GG GG GG GG GG
GG GG GG GG GG GG GG AG
GG GG GG GG GG GG GG GG
GG GG GG GG GG GG GG
25898226
8104 rs10231874 7 AA AA AA AG AG
AA GG AA AA AG AG AA AG

GG GG AA AA AG AA AG AA
AG AA AA AA AA AG AA AA
AG AA AA AA AA AA AA
14349699
8105 rs10231884 7 GG GG GG AG AG
AA AG AA AA AA GG AG AG
AG AA AG GG GG GG GG AG
AA AG AG AA AG GG GG AG
AG AG AG AG AG AA AG
79084800
8106 rs10231956 7 GG GG GG AG GG
GG AG AG AG GG GG GG GG
GG GG AG AA AA GG GG AG
AG AG AG AG AA GG AG AG
AG AA AG AA AG AG AA
64762394
8107 rs10231967 7 AA AA AA AG AG
AA GG AG AG GG AG AG AG
AA AA GG AG AG AA AA AG
AG GG AG GG AA AA AA AA
AA AA AA AA AA AA AA
1458931
8108 rs10232013 7 AG GG AG AG AG
AA GG GG GG GG GG GG GG
AG AG AG GG GG AA GG AG
GG AG GG GG GG GG GG GG
GG AG AG AG GG AG GG
156737354
8109 rs10232026 7 AA AA AA AC AA
AC AA AC AC AA AA AA AA
AA AA AC AA AA CC AC AC
AA AA AC AC AA AA AA AA
AA AA AA AA AC AA CC
67236758
8110 rs10232153 7 GG AG AG GG GG
GG GG GG GG GG GG GG GG
GG GG GG GG GG GG GG GG
AG GG GG AG GG AG AG AG
AG AG AG AG GG GG GG
97764162
8111 rs10232172 7 CC CC CC CC CC
CC CC CC CC CC CC CC CC
CC CC CC AC CC CC CC CC
CC CC CC CC CC CC CC CC
CC CC CC CC CC CC CC
54182781
8112 rs10232236 7 AA AA AA CC CC
CC CC CC CC CC CC CC CC
CC AC CC AC CC CC CC CC
CC CC CC CC AC AC CC CC

CC CC AC CC CC CC AC
11529374
8113 rs10232259 7 GG GG GG GG GG
GG GG GG GG GG GG GG GG
GG GG GG GG GG GG GG GG
GG GG GG GG GG GG GG GG
GG GG GG GG GG GG GG
73160543
8114 rs1023229 13 AG GG AG AA AA
AA AA AG AG AA AA AA AA
AA AA AG AG AA AG GG AA
AG AA AA AA AA AA AA AA
AA AG AA AA AA AA AA
20270925
8115 rs10232337 7 AC AC CC CC AC
AC CC CC AC AC AC CC CC
CC CC CC CC CC AC AC AC
CC AC CC AC CC CC CC CC
AC AA CC AC CC AC AC
4584559
8116 rs10232355 7 AA AG AG AG AG
AG AG AA AA AG AG AG GG
AG AG AG AA AA AA AA AG
AG AG AG AG AG AG AG AG
AG AA AG AA AG AA AG
2357284
8117 rs10232413 7 AA AG AG GG GG
GG GG GG GG GG GG GG AG
GG AG GG GG GG AG AG GG
GG GG GG GG GG GG GG GG
AG GG GG GG GG GG GG
2286347
8118 rs10232432 7 AA AA AA AG AA
AG AA AA AA AA AA AA AA
AG AG AA AA AA AA AA AA
AG AA AG AA AA AA AA AA
AG AA AA AA AA AA AA
11258798
8119 rs10232435
7 -- -- -- -- -- -- -- --
-- -- -- -- -- -- -- --
-- -- -- -- -- -- -- --
-- -- -- -- -- -- -- --
-- -- -- -- 75986166
8120 rs10232489 7 AA AA AG AG AA
AA AG AA AG AG GG AG AG
AA AA AG AA AG AG AG AA
AA AA AA AA AG AA AA AG
AA AG AA AG AA AA AA
55347512

8121 rs1023249 9 AG AG AG AG AG
AG AG GG AG AG GG AG AG
AG AG GG GG GG AA AA AA
GG AG GG AG GG GG GG GG
GG AG GG GG GG AG GG
101752484
8122 rs10232566 7 CC CC CC CC CC
CC CC CC CC CC CC CC CC
CC CC CC CC CC CC CC CC
CC CC CC CC CC CC CC CC
CC CC CC CC CC CC CC
114179466
8123 rs10232581 7 GG GG AG GG GG
GG GG AG GG AG AG GG AG
GG GG GG GG GG GG GG GG
AG GG GG AG GG GG GG GG
GG GG GG GG GG GG GG
56317685
8124 rs10232703 7 GG AG AG GG GG
GG GG GG AG AG GG GG GG
GG GG GG GG GG GG GG GG
GG GG GG GG GG AG GG GG
AG GG AG GG GG GG AG
4336477
8125 rs10232758 7 AA AC AC AA AC
AC AA CC CC CC AC AA AC
CC AA AC CC CC CC AC CC
AC AC CC AC AC AC AC AC
CC AA CC AC AA AA AC
98411980
8126 rs1023276 6 GG GG GG GG GG
GG GG AA AA AA GG GG AG
AG AG AA AG AA AG GG GG
GG GG GG GG GG AG GG GG
GG GG GG GG GG GG AG
10245760
8127 rs10232819 7 GG GG AG AG AA
AA AG GG AG GG GG GG GG
AA AG AG GG AG AG AA GG
AA AG AA AG AG AA AG AA
AG AG AA AA AA AA AG
25876612
8128 rs10232859 7 GG GG AG AG GG
AG GG GG GG AG GG GG GG
GG GG AG AG AG GG GG AG
GG GG GG GG GG GG GG GG
AG GG GG GG GG GG GG
39605204
8129 rs10232915 0 GG GG GG GG GG
GG GG GG GG GG GG GG GG

GG GG GG GG GG GG GG GG
GG GG GG GG GG GG GG GG
GG GG GG GG GG GG GG 0
8130 rs10232926 7 AA AA AA AA AA
AA AA AA AA AA AA AA AA
AA AA AA AA AA AA AA AA
AA AA AA AA AA AA AA AA
AA AA AA AA AA AA AA
62254482
8131 rs10233020 7 AA AA AA AG AG
AA GG AG AA AG AA AA AA
AA AG AA AG AG AA AA AA
AG AA AG AG AA AG AG AG
AA AG AA AG GG AG GG
132624847
8132 rs10233032 7 CC CC CC CC CC
CC CC CC CC CC CC CC CC
CC CC CC CC CC CC CC CC
CC CC CC CC CC CC CC CC
CC CC CC CC CC CC CC
51882617
8133 rs10233143 7 GG GG GG GG GG
GG GG GG GG GG GG GG GG
GG GG GG GG GG GG GG GG
GG GG GG GG GG GG GG GG
GG GG GG GG GG GG GG
73938629
8134 rs10233228 7 AG GG AG AA AA
AA AA AA AA AA AA AA AA
AA AA AG AA AA AA AG AG
AA AG GG AG AA AA AA AA
AG AA AA AA AG AG AA
54746604
8135 rs1023325 1 AA AA AA AG AG
GG AA GG GG GG AG AG AA
AA AA AG AG AG AG AA AG
AA AA AA AA AA AG AA AA
GG AG AA AG AG AA AG
34682856
8136 rs10233387 7 AA AA AA GG GG
GG GG AG AG AG AG AG GG
AA AG GG GG GG AG GG AA
AG AG AG AG AG AG AG GG
AA AA AG AG GG AG AG
27243106
8137 rs10233402 7 AG AG GG AG AG
AG AG AG AG AG AG GG AG
AA GG AG AG AG AG AG AG
AG AG AA AG GG AG AG AG
AA GG GG GG AG AA GG

101659801
8138 rs10233439 7 GG GG GG GG GG
GG GG GG GG GG GG GG GG
GG GG GG GG GG GG GG GG
GG GG GG GG GG GG GG GG
GG GG GG GG GG GG GG
53907084
8139 rs10233448 7 AG AG GG AG GG
AG GG GG GG GG GG GG GG
GG GG AG AG AG GG GG GG
GG GG GG GG GG AG AG AG
GG GG GG GG GG GG GG
132411901
8140 rs10233491 7 AG GG GG GG GG
GG GG GG AG GG GG GG GG
GG GG AG GG GG AG GG AG
GG AG AG GG GG AG AG AG
GG GG GG GG GG GG GG
25466521
8141 rs10233510 7 AG AG GG GG AA
AG AG AA AA AG GG GG AG
AG AG AG AG AG AG GG AA
AA AA AA AA AG AG AG AA
AG AG AA AA AG AG GG
88770627
8142 rs1023355 21 CC AC CC CC CC
CC CC CC CC CC AC CC AC
CC CC CC CC CC AA AC AC
CC AC CC CC CC CC CC CC
CC AC CC AC AC CC AC
47892806
8143 rs10233600 7 AG GG AG AG GG
GG AG AG AG AG AG AG AA
AG AG GG GG GG AA AG AG
GG AG AG GG GG GG GG GG
AG GG GG GG AG GG AG
23938828
8144 rs1023361 21 GG GG GG GG GG
GG GG GG GG GG GG GG GG
GG GG AG GG GG GG GG GG
GG GG GG GG GG GG GG GG
AG GG GG GG AG AG GG
23344282
8145 rs10233618 7 AG AG AG AG AG
AG AG GG GG GG GG AG AG
AG GG GG GG GG GG GG GG
GG AA GG GG AG AG GG GG
AG GG AG AG AG GG AG
145069168
8146 rs10233641 7 AA AA AA AG AA

	--	AA	AA	AA	AA	AA	AA	AA
	AA	AA	AA	AA	AA	AA	AA	AA
	AA	AA	AA	AA	AA	AA	AA	AA
	AA	AA	AA	AA	AA	AA	AG	
26243108								
8147	rs10233701							
7	--	--	--	--	--	--	--	--
	--	--	--	--	--	--	--	--
	--	--	--	--	--	--	--	--
	--	--	--	--	--	--	--	--
	--	--	--	--	101058438			
8148	rs10233716		7	AA	AA	AA	AA	AA
	AA	AA	AA	AA	AA	AA	AA	AA
	AA	AA	AA	AA	AA	AG	AA	AG
	AA	AG	AA	AG	AA	AA	AA	AA
	AA	AA	AA	AA	AA	AA	AA	
13485785								
8149	rs10233720		7	AA	AA	AA	AA	AA
	AA	AA	AA	AA	AA	AA	AA	AA
	AA	AA	AA	AA	AA	AA	AA	AA
	AA	AA	AA	AA	AA	AA	AA	AA
	AA	AA	AA	AA	AA	AA	AA	
64775777								
8150	rs10233726		7	--	CC	--	--	CC
	--	CC	CC	CC	CC	CC	CC	--
	--	--	CC	CC	CC	--	--	AC
	--	CC	CC	CC	CC	AC	--	--
	CC	--	AC	CC	AC	CC	AA	
26488008								
8151	rs10233736		7	AG	AG	AA	AA	AG
	AG	AG	AA	AG	AA	AG	GG	AG
	AG	AA	GG	AG	AG	AA	AG	AA
	AG	AG	AG	AG	GG	AA	AG	AG
	AA	AG	AG	AG	AA	AG	AA	
143066020								
8152	rs10233740		7	AA	AA	AG	AA	AA
	AG	AA	GG	AA	AG	AA	AA	AA
	AA	AA	AG	AG	AG	AA	AA	AA
	AG	AG	AG	AG	AA	AA	AA	AA
	AG	AA	AA	AA	AG	GG	AG	
148567263								
8153	rs102338	19	AA	AA	AA	AA	AG	AG
	AA	AG	AG	AG	AG	AG	GG	AG
	AA	AG	AG	GG	AG	AA	AG	AA
	AA	AA	AA	AA	AA	AA	AA	AA
	AA	AG	AG	AA	AA	AA	31401756	
8154	rs10233804		7	AC	CC	AC	AA	AC
	AA	AC	AC	AC	CC	CC	CC	AA
	AA	CC	AC	CC	CC	AA	CC	AC
	CC	CC	AC	CC	AC	AC	AC	AA

AC AC CC CC AC AC AC
92995752
8155 rs10233878 7 AG AA AG GG AA
AG AG AG AG GG AA AG AG
AG GG AA AG AG AG AA AG
AA AG AA AG AA AG AA AA
AA AG GG GG GG GG AG
17144007
8156 rs10233882 7 AG AA AG AA AG
AG AA AA AA AA AG AG AG
AA AA AG AG AA AA AA AA
AA AA AA AA AG AA AG AG
AA AG AA AA AA AA AA
4439722
8157 rs10233902 7 CC CC AC AA AC
AC AC AC AC AC AC CC AC
AC AC AC AC AC AC CC AC
AA AC AA AA CC CC CC CC
AC CC AC CC CC AC CC
129881673
8158 rs10233905 7 GG GG GG GG GG
GG GG GG GG GG GG GG GG
GG GG GG GG GG GG GG GG
GG GG GG GG GG GG GG GG
GG GG GG GG GG GG GG
100734113
8159 rs10234024 7 AG AG AG AG GG
GG AG AG AG GG AA AG AG
AA AA AG GG AG AG AG AG
AA GG AG AG AG AG AG AA
AG GG GG GG AG GG AA
41983613
8160 rs10234091 7 GG GG GG AG GG
AG AG AG AG AG AA AG AA
AG AG AG GG AG AA AA AG
AG AG AG AG AA AG AA AA
GG AG AG AA AG AG AG
11605462
8161 rs10234185 7 AA AA AA AC AA
AC AA AC AC CC AC AA AA
AA AA AA AA AA CC AC AC
CC AC AC AC AA AC AC AC
AA AA AC AA AC CC AA
12298311
8162 rs10234187 7 AG GG AG GG AG
AG GG GG GG GG AG GG AG
AG AG GG GG GG AG GG AG
GG GG GG GG AG AG GG GG
AG GG GG GG AG AG AG
11216782

8163 rs10234191 7 GG GG GG GG GG
GG GG GG GG GG GG GG GG
GG GG AG AG AG GG GG GG
GG GG GG GG AG GG AG AG
GG GG GG GG GG GG GG
103666588
8164 rs10234196 7 GG GG GG GG GG
GG GG GG GG GG GG GG GG
GG GG GG GG GG GG GG GG
GG GG GG GG GG GG GG GG
GG GG GG GG GG GG GG
102999943
8165 rs1023421 1 AG AA AG GG AA
AG AG AG AG AG AA AA AA
AA AG AA AA AG AG GG GG
AG AG AA AG AA AG AA AA
AA AG AA AG AG AG AG
160094644
8166 rs10234240 7 AG GG AG AG AG
AG AG AA AA AA AA AA AA
AA AA AG AA AA AA AG AG
AG AG AA AG AG AA AG AG
AA AG AG GG AG AA AG
97107457
8167 rs10234275 7 GG GG GG GG GG
GG GG GG GG GG GG GG GG
GG GG GG GG GG GG GG GG
GG GG GG GG GG GG GG GG
GG GG GG GG GG GG GG
55616264
8168 rs10234294 7 AA AA AA AA AA
AA AA AA AA AA AA AA AA
AA AA AA AA AA AG AG AA
AA AA AA AA AA AA AA AA
AA AA AA AA AA AA AA
4704532
8169 rs1023435 X GG AG AG GG GG
GG GG AG AG GG AA AA AA
AA AA AG AG AA GG AG GG
AA AA AA AA AA AA AA AA
GG AA AA AA AA AA AA
152207464
8170 rs10234360 7 GG GG GG GG GG
GG AG GG GG GG GG GG GG
GG GG GG GG GG GG GG GG
GG GG GG GG GG GG GG GG
GG GG GG GG GG GG AG
75261476
8171 rs10234418 7 CC CC CC CC AA
AC AC AC AC AC AC AC AC

CC AC CC CC CC AC AC CC
CC CC CC CC AC AC AC AC
AC CC AC CC CC CC CC
95931247
8172 rs1023442 16 GG AG GG GG AG
GG AG AA AA AA AG AG AG
AA AG AG AG AA GG GG GG
AG AG AG AG AA AG AG AA
GG AA AG AG GG GG AG
19331565
8173 rs10234423 7 AA AA AA AA AC
AC AA AA AA AA AA AA AA
AA AA AA AA AA AA AA AC
AA AC AC AA AA AA AA AA
AA AA AC AA AA AA AA
46442370
8174 rs10234472 7 AG AA AA AA AA
AA AA AG AA AG AG GG AG
AA AG AA AA AA AA AG AA
AA AA AA AA AG AA AA AG
AA AG AA AA AA AA AA
64118662
8175 rs10234489 7 GG GG GG AG GG
GG AG AG AG AA GG GG GG
GG GG GG GG GG AG GG AG
AG AG AG AG GG GG GG GG
GG GG GG GG GG AG GG
39165537
8176 rs10234525 7 AG AA AG AA AA
AA AA AG AG AG AA AA AA
AA AA AG AA AA AA AA AA
AA AA AA AA AA AA AA AA
AA AA AA AA AA AA AA
156477064
8177 rs10234533 7 GG AG GG GG AA
AG AG AG AG AG AG AG AA
AG AG GG GG AG AA AA AA
AG AA AG AA AG AG AA AG
AG AG AA AG AA AG AG
22101207
8178 rs10234562 7 GG GG GG GG GG
GG GG GG GG GG GG GG GG
GG GG GG GG GG GG GG GG
GG GG GG GG GG GG GG GG
GG GG GG GG GG GG GG
65536107
8179 rs10234640 7 AA AA AC AC AC
AC AA AA AA AA AA AC AC
AC AC AA AA AA AA AA AA
AC AA AA AC AA AC AC AC

AA AC AA AC AA AA AA
66346373
8180 rs10234642 7 GG GG GG GG GG
GG GG AG AG AG GG GG GG
GG GG GG GG GG GG GG GG
GG GG GG GG AG GG AG GG
GG GG GG GG GG GG GG
22528662
8181 rs10234652 7 GG AG GG AA AA
AA AA AA AA AA AG AG AA
AA AA AA AG AA AA AA AA
AA AA AA AA AA AA AA AA
AA AA AA AA AA AA AG
13188639
8182 rs10234656 7 GG GG GG GG GG
GG GG AG AG GG GG GG GG
GG GG GG GG GG GG GG GG
GG GG GG GG GG GG GG GG
GG GG GG GG GG GG GG
40962660
8183 rs10234665 7 GG GG GG GG GG
GG GG GG GG GG GG GG GG
GG GG GG GG GG GG GG GG
GG GG GG GG GG GG GG GG
GG GG GG GG GG GG GG
75564873
8184 rs10234666 7 AA AA AA AA AA
AA AA AA AA AA AA AA AA
AA AA AA AA AA AA AA AA
AA AA AA AA AA AA AA AA
AA AA AA AA AA AA AA
5295617
8185 rs10234672 7 AA AA AA GG GG
GG GG AG AG AG AA AA AA
AA AA AG GG GG AG GG AG
AG AA AG AG AA AG AG AG
AA AG AG AG AA AA AG
137118980
8186 rs1023470 22 AA AA AA AA AA
AA AA AG AG GG AG AG AA
AA AG AA AA AA AA AA AA
AA AG AA AA AA AA AA AA
AA AA AG AG AA AA AA
46235677
8187 rs10234711 7 AG GG AG AG GG
AG GG AG AG AG AA AG AG
AG GG AG AA AA AG AA GG
GG GG GG GG GG GG GG GG
AG AA GG AG AG AG GG
121229994

8188 rs10234747 7 AA AA AG AG AG
GG AA AA AA AA AA AG AG
AG AG AA AA AA AA AA AA
AG AA AA AG AA AG AG AG
AA AG AA AG AA AA AG
66251369
8189 rs10234749 7 CC AC CC AC AC
AA AC CC CC CC AA AC AC
CC CC CC CC CC CC CC CC
CC CC CC CC AC CC AC AC
CC CC CC CC AC CC AC
152387869
8190 rs10234768 7 AA AG AG GG GG
GG GG AG AA GG GG GG GG
GG GG AG AG AA GG GG GG
GG GG GG GG GG AG AG AG
AG GG GG GG GG GG AG
50182079
8191 rs1023479 1 AG AG GG AA AA
AA AA AG AA AG AG AG AA
AA AG AA AA AA AA AA AA
AG AA AA AA AG GG AG GG
AA AA AA AA AA AA AA
172355132
8192 rs10234801 7 GG GG GG GG GG
GG GG GG GG GG GG GG GG
GG GG GG GG GG GG GG GG
GG GG GG GG GG GG GG GG
GG GG GG GG GG GG GG
56110814
8193 rs10234834 7 GG GG GG AG GG
AG GG GG AG GG GG GG GG
GG GG AA AA AG GG GG GG
AG AG AG AG AA GG AG AG
GG AG GG GG AG GG AG
29745196
8194 rs10234844 7 CC AC CC CC AC
AC CC AA AC AA AC AA AC
AC AA CC CC CC CC AC CC
AC AA AA AC AC CC AC CC
CC AC CC CC AC AC CC
69811212
8195 rs10234926 7 AA AA AA AG AA
AG AA AA AA AA AA AA AA
AA AA AA AG AG AG AG AA
AA AA AA AA AA AA AA AA
AA AA AG AG AA AA AG
82984827
8196 rs1023511 X GG GG GG GG GG
GG GG GG GG GG GG GG GG

	GG	GG	GG	GG	GG	GG	GG	GG
	GG	GG	GG	GG	GG	GG	GG	GG
	GG	GG	GG	GG	GG	GG	GG	
14025443								
8197	rs10235116		7	AC	AC	AA	AC	AC
	CC	AA	AC	AA	AC	AA	AA	AA
	AC	AC	AC	AA	AC	AC	CC	AC
	CC	CC	AC	AC	AC	AC	AC	CC
	CC	AA	AC	AC	AC	AC	AC	
53688656								
8198	rs10235144		7	AG	AA	AA	AG	AG
	GG	AG	AG	GG	AG	AG	AG	AA
	AG	GG	AA	AG	AG	AG	GG	AG
	AA	GG	AA	AA	GG	AA	AG	AG
	GG	AA	AG	AG	AG	AA	GG	
140108096								
8199	rs10235198		7	GG	GG	GG	GG	GG
	GG	GG	GG	GG	GG	AG	AG	AG
	AG	GG	GG	GG	GG	GG	GG	GG
	GG	GG	GG	GG	GG	AG	GG	GG
	GG	AG	GG	AG	GG	GG	GG	
88311477								
8200	rs10235210		7	AA	AA	AA	AG	AG
	AA	AG	AA	AA	AA	AG	GG	AG
	AG	AG	AG	AG	AG	AG	AG	AG
	AA	AA	AA	AA	AG	GG	AG	AG
	AG	AA	AA	AA	GG	AG	GG	
116003445								
8201	rs1023522		7	AA	AG	AA	GG	AA
	AG	AG	AG	GG	AG	AG	AA	AG
	AG	AA	AG	AG	GG	AG	GG	AA
	AG	AA	AG	AA	AG	AG	AG	AA
	AG	AA	AG	AG	GG	GG	AG	
20413590								
8202	rs10235235		7	AA	AA	AA	AA	AG
	AG	AA	AA	AA	AA	GG	AG	AG
	GG	AA	AA	AA	AA	AA	AA	AA
	AA	AA	AA	AA	AA	AA	AA	AA
	AA	AA	AG	AG	AA	AA	AG	
99075831								
8203	rs10235241		7	GG	GG	GG	AA	GG
	AG	AG	AA	AA	AG	GG	GG	AG
	AG	GG	GG	AG	AG	GG	GG	GG
	AA	AG	GG	AG	GG	AG	AG	AG
	AG	GG	GG	GG	AG	AG	GG	
86019034								
8204	rs10235248		7	AA	AA	AA	AA	AA
	AA	AA	AA	AA	AA	AA	AA	AA
	AA	AA	AA	AA	AA	AA	AA	AA
	AA	AA	AA	AA	AA	AG	AA	AA

AA AA AG AA AG AA AG
26504359
8205 rs10235270 7 AG GG AG AG AG
GG AA AG AA AG GG GG AG
AG AG AA AA AA AA AG AA
AG GG GG AG GG AA AG AG
AG AA AG AG AA AA AG
30894229
8206 rs10235329 7 AC AC AA AC AC
AA CC CC CC AC AA AA AA
AC AC AC CC AC CC AC CC
CC CC AC AC AA CC AC AC
AA CC AA AC CC AC CC
55743106
8207 rs10235430 7 GG GG GG AG AG
AG AG GG AG AG AA AG AG
GG GG GG GG GG GG GG AG
GG AG AA AG GG AG GG GG
AG AA AA AA AG AG AG
53652231
8208 rs10235436 7 GG GG GG GG GG
GG GG GG GG GG GG GG GG
GG GG GG GG GG GG GG GG
GG GG GG GG GG GG GG GG
GG GG GG GG GG GG GG
155353750
8209 rs10235546 7 CC CC CC CC CC
CC AC AC AC AC CC CC CC
AC AC CC CC CC CC CC CC
CC CC CC CC CC CC CC CC
CC AC CC CC CC CC CC
150078038
8210 rs10235566 7 GG GG GG GG GG
GG GG GG GG GG GG GG GG
GG GG GG GG GG GG GG GG
GG GG GG GG GG GG GG GG
GG GG GG GG GG GG GG
102575562
8211 rs10235606 7 AG AA AG AG AG
GG AA AG AG AA AA AA AA
AG AA AA AA AA AG AG AA
AA AG AG AA AA AG AG AG
AG AG GG GG AA AG AG
92848066
8212 rs10235648
7 -- -- -- -- -- -- -- --
-- -- -- -- -- -- -- --
-- -- -- -- -- -- -- --
-- -- -- -- -- -- -- --
-- -- -- -- 34899235

8213 rs10235674 7 AG AA AA AG AA
AG AA GG GG AG AG AA AG
GG GG AG -- GG GG AG AG
AA AA AA AA GG AA AG AG
AG AG AA AG AG AA AG
75816671
8214 rs1023570 17 GG GG GG AG AG
AG GG GG GG GG GG GG GG
GG GG GG GG GG GG GG AG
GG GG GG GG GG GG GG GG
GG GG GG GG GG GG GG
70595268
8215 rs1023571 5 AG AG AG -- GG
GG -- AG AG AG GG GG --
AG -- AG AG AA AG GG AA
AG AG AG AG AG AG AG AG
-- AG -- GG AG AG --
57070495
8216 rs10235715 7 AC AC AA AA AA
AA AA AA AA AA AA AA AA
AA AA AA AA AA AA AA AA
AA AA AA AA AA AA AA AA
AA AC AA AC AA AA AA
136350740
8217 rs10235763 7 AG AA AA AA AA
AA AA AA AA AA AA AA AA
AA AA AA AA AA AA AA AA
AG AA AA AG AA AG AG AG
AA AA AA AA AA AA AG
56693762
8218 rs10235860 7 AA AG AA AG AG
GG AA GG AG GG AG GG AG
AG GG AA AA AA AA AG AA
AG GG GG AG AG AA AG AA
AA AG AA AA AG AG AA
69570759
8219 rs10235866 7 AG GG AG AA AG
AG AA GG GG AG GG GG GG
GG GG GG GG GG GG GG GG
AG AG GG AG AG GG AG AG
GG AG GG GG GG GG GG
98269772
8220 rs10235893 7 AG GG GG GG GG
GG GG AA AG AG GG GG GG
GG GG AG AA AA AA AG AG
GG AG GG GG GG AA AG AG
GG AG AG GG AA AG AA
152178955
8221 rs10235911 7 AA AG AA AG AG
GG AG AA AA AG GG AG AG

AA AG AA AA AA AG AA AG
AA GG AA AA AG AA AG AG
AG GG AA AG AG AG AA
158038989
8222 rs10235951 7 GG GG GG AA AG
AA AG AA AA AG AA AA AG
AG AA AG AA AA GG GG GG
GG AG AG GG AG AA AG AA
AA GG AA AG AG AG AA
85608916
8223 rs10235979 7 AA AA AA AA AA
AA AA AA AA AA AA AG AG
AG AA AG AG AG AA GG AG
AA AA AA AA AA AA AA AA
AA AA AA AA AG AG AA
156173632
8224 rs1023598 2 AA AA AA AG AA
AA AG AA AA AA AA AA AA
AG AG AA AA AA AA AA AA
AA AA AA AA AA AG AA AG
AA AA AA AA AA AA AA
57051996
8225 rs1023599 3 AG AG AG GG AG
GG AG GG AA AG AA AA AG
GG AG GG AG AG AA AA AA
AG AG AA AG AA AG AA AG
AA GG AG GG AA AA AA
170255506
8226 rs10236091 7 AG AG GG AG AA
AG AA GG GG AG AA AA AA
AA AA AA AA AA AG AG AA
AA AG AG AA AG AA AG AA
AG AA GG AG AG AA AG
51542925
8227 rs10236093 7 AA AA AA AA AA
AA AA GG AG GG AA AA AA
AA AA AA AG AA AG AG AA
AA AA AA AA AA AA AA AA
AA AA AA AA AA AA AA
20185196
8228 rs10236105 7 AG AG AA AA GG
AG AG AG AG GG AA AG AG
AA AA AA AG AG AG AG AG
GG AG AA GG AG GG AG GG
AG GG AG GG AA AA AA
44643802
8229 rs1023613 5 AA AA AA AA AA
AA AA AA AA AA AA AA AA
AG AG AA AG AG AA AA AA
AA AA AA AA AA AA AA AA

AA AA AA AA AG AA AG
125712172
8230 rs10236169 7 AG AG AG AA AG
AG AA AA AG AG AA AA AA
AA AA AA AA AA AG AG AA
AG AA AG AA AG GG AG AG
AA AG AA AG AA AG AA
40408314
8231 rs1023617 5 GG AG AG GG GG
GG GG GG GG GG GG GG GG
GG GG GG AG AG AA AG AA
GG GG GG GG AG GG AG GG
AG AG AG AG AG AG GG
172740329
8232 rs10236183 7 CC CC CC AC AC
CC AC AC AC AA CC AC AC
AC CC AA AC AC CC AC CC
AC CC CC AC AC CC CC AC
AC CC AC AC AC CC AA
79750012
8233 rs10236225 7 GG AG GG AA AG
AG AA AG AG AG AA AG AA
AA AG AA AA AG AG GG AA
GG AG AG AG AG AG AA AA
AA AA AG AA AA AG AG
10226430
8234 rs10236323 7 AA AA AA AA AA
AA AA AA AA AA AA AA AA
AA AA AA AA AA AA AA AA
AA AA AA AA AA AA AA AA
AA AA AA AA AA AA AA
98141680
8235 rs10236334 7 AA AG AG AG GG
AG AG AG AG AG AA AA AA
AA AA AA AA AA AG AG AG
AA AA AA AA AA AA AA AA
AA AA AG AG AA AA AA
111329336
8236 rs10236343 7 GG GG GG GG GG
GG GG GG GG GG GG GG GG
GG GG GG GG GG GG GG GG
GG GG GG GG GG GG GG GG
GG GG GG GG GG GG GG
106481440
8237 rs10236360 7 GG GG GG AG GG
AG GG GG GG GG GG GG GG
GG GG GG AG AG AG AG GG
GG GG GG GG AG GG GG AG
GG GG AA AG GG GG AG
82982193

8238 rs10236483 7 CC CC CC AC AC
CC AA AC AC AA AC AC AA
AC AC AC AC AC CC AC CC
AC AC AC AC CC AA AC AC
AC CC AC CC AC AA AC
91797296
8239 rs10236516 7 GG GG GG GG AG
AG GG GG AG GG AG AA AG
GG AG GG AG AG GG GG GG
GG GG GG GG GG AG AG AG
AA GG GG GG AG AA GG
73703236
8240 rs10236619 7 GG GG GG GG GG
GG GG GG GG GG GG GG GG
GG GG GG GG GG GG GG GG
GG GG GG GG GG GG GG GG
GG GG GG GG GG GG GG
73673172
8241 rs10236699 7 GG GG GG GG GG
GG GG GG GG GG GG GG GG
GG GG GG GG GG GG GG GG
GG GG GG GG GG GG GG GG
GG GG GG GG GG GG GG
64162098
8242 rs10236729 7 GG GG AG GG AG
AG GG AG GG AG GG GG GG
GG GG AG GG GG GG AG GG
GG GG GG GG GG GG GG GG
GG GG GG GG GG GG GG
64402765
8243 rs10236747 7 GG GG GG AG AA
AG AG AG AG AG GG AG AG
GG GG GG GG GG AG GG AG
GG GG GG GG GG AG AG AG
AG GG GG GG GG GG AG
12870484
8244 rs10236791 7 AA AA AA AA AA
AA AA AA AA AA AA AA AA
AA AA AA AA AA AA AA AA
AA AA AA AA AA AA AA AA
AA AA AA AA AA AA AA
53448760
8245 rs10236808 7 GG GG GG AG AG
AG AG GG AG GG AG AA AG
AG GG GG GG GG AG AG AG
GG GG GG GG GG GG GG GG
GG AG AG AA GG GG AG
148698474
8246 rs10236813 7 AA AA AG AA AA
AA AA AA AA AA AA AA AA

	AA	AA	AA	AA	AG	AA	AA	AA
	AA	AA	AA	AA	AA	AA	AA	AA
	GG	AG	AA	AG	AG	AA	AG	
49710785								
8247	rs1023682		17	AA	AA	AA	AA	AA
	AA	AA	AG	AA	AA	AA	AA	AA
	AA	AA	AA	AA	AA	AA	AA	AA
	AA	AA	AG	AG	AA	AG	AA	AA
	AA	AA	AA	AA	AA	AA	AA	
27114600								
8248	rs10236829		7	AA	AA	AA	AA	AA
	AA	AA	AA	AA	AA	AA	AA	AA
	AA	AA	AA	AA	AA	AA	AA	AA
	AA	AA	AA	AA	AA	AA	AA	AA
	AA	AA	AA	AA	AA	AA	AA	
17991604								
8249	rs10236906		7	AG	AG	AG	GG	AG
	AG	GG	GG	GG	GG	GG	GG	GG
	GG	GG	GG	GG	GG	AG	AG	GG
	GG	GG	GG	GG	GG	GG	GG	GG
	AG	GG	GG	GG	GG	AG	GG	
18739670								
8250	rs10236912		7	AA	AA	AA	AA	AA
	AA	AA	AA	AA	AA	AA	AA	AA
	AA	AA	AA	AA	AA	AA	AA	AA
	AA	AA	AA	AA	AA	AA	AA	AA
	AA	AA	AA	AA	AA	AA	AA	
75160045								
8251	rs1023692		2	GG	GG	AG	GG	GG
	GG	GG	GG	GG	GG	AG	GG	AG
	GG	GG	AG	GG	AG	AG	AG	AG
	AG	AG	AG	AG	AG	AG	AG	GG
	AG	AG	GG	AG	AG	GG	AG	
67868756								
8252	rs10236925		7	AG	AG	AA	AG	AA
	AA	AG	AG	AA	AG	AA	AA	AA
	AA	AA	AG	AG	GG	AG	AA	AG
	AA	AG	AG	AA	GG	AA	AG	AG
	AG	AA	AA	AA	AG	AG	AA	
54455062								
8253	rs10236956		7	AC	AC	AC	CC	AC
	AC	CC	AC	AC	AC	AC	AC	AA
	AA	AC	AC	AC	AC	AC	AC	CC
	CC	AC	AC	CC	AA	AC	AC	AC
	AA	AC	AC	AA	CC	AC	AC	
67649847								
8254	rs10236961		7	AC	AC	AC	CC	CC
	CC	CC	CC	AC	CC	CC	CC	CC
	CC	CC	CC	CC	AC	AC	CC	AC
	AC	CC	CC	AC	CC	AC	CC	CC

CC AA CC AC CC AC CC
31074214
8255 rs10237001 7 AA AA AA AG AG
GG AA AA AG AA AG AA AG
AG AG AA AA AA AA AA AA
AA AA AA AA AA AA AA AA
AA AG AA AA AG AA GG
131939267
8256 rs10237039 7 GG GG GG GG GG
GG GG GG GG GG GG GG GG
GG GG GG GG GG GG GG GG
GG GG GG GG GG GG GG GG
GG GG GG GG GG GG GG
4350065
8257 rs1023711 7 AA AA AA AA AA
AA AA AA AA AA AA AA AA
AA AA AA AA AA AA AA AA
AA AA AA AA AA AA AA AA
AA AA AA AA AA AA AG
26803063
8258 rs1023719 19 GG GG GG GG GG
GG GG GG GG GG GG GG GG
GG GG AG GG AG GG GG GG
GG GG GG GG GG GG GG GG
GG GG GG GG GG GG GG
24092968
8259 rs10237193 7 AC AC AC AC AC
CC AA CC CC AC CC CC CC
AA AA AC CC AC AA CC AC
AC AC CC CC CC AC CC CC
CC AA CC AC AA AA AA
49836460
8260 rs10237207 7 CC CC CC CC CC
CC CC CC CC CC CC CC CC
CC CC CC CC CC CC CC CC
CC CC CC CC CC CC CC CC
CC CC CC CC CC CC CC
3831096
8261 rs10237272 7 AA AG AG GG AA
AG AG AA AA AG AA AA AA
AG AG AG GG GG GG AG GG
AG AA AG AG AG AG AA AG
AG GG AG AG GG AG GG
93201902
8262 rs1023742
10 -- -- -- -- -- -- -- --
-- -- -- -- -- -- -- --
-- -- -- -- -- -- -- --
-- -- -- -- -- -- -- --
-- -- -- -- 98423739

8263	rs1023744	18	AA	AG	AG	AG	AG
	AG	AA	AA	AA	AA	AA	AG	AA
	AA	AA	AA	AA	AA	AG	AG	GG
	GG	GG	AG	GG	AG	AA	AA	AA
	AA	AA	AA	AA	AA	AA	AA	
67484495								
8264	rs10237489	7	AA	AA	AA	AA	AA
	AA	AA	AA	AA	AA	AA	AA	AA
	AA	AA	AA	AA	AA	AA	AA	AA
	AA	AA	AA	AA	AA	AA	AA	AA
	AA	AA	AA	AA	AA	AA	AA	
3814566								
8265	rs10237528	7	GG	GG	GG	GG	GG
	GG	GG	GG	GG	GG	GG	GG	GG
	GG	GG	GG	GG	GG	GG	GG	GG
	GG	GG	GG	GG	GG	GG	GG	GG
	GG	GG	GG	GG	GG	GG	GG	
47506868								
8266	rs10237588	7	GG	AG	GG	GG	GG
	GG	AG	AG	AG	GG	AG	AG	GG
	GG	GG	AG	AA	AG	AG	GG	AG
	GG	AG	GG	AG	GG	GG	GG	GG
	GG	GG	AG	GG	AG	AG	GG	
12032707								
8267	rs10237610	7	--	AG	AG	--	GG
	GG	--	--	GG	GG	GG	GG	--
	GG	GG	AG	GG	GG	AG	AG	GG
	GG	AG	AG	--	AG	GG	AG	AG
	GG	GG	--	GG	GG	GG	GG	
101020021								
8268	rs1023763	1	AG	AA	AG	AA	AA
	AA	AA	AA	AA	AA	AA	AA	AA
	AA	AA	AA	AA	AA	AG	AG	AG
	AA	AA	AA	AA	AA	AA	AA	AA
	AA	AA	AA	AA	AA	AA	AA	
214276204								
8269	rs10237771	7	GG	GG	GG	GG	GG
	GG	GG	GG	GG	GG	AG	AG	GG
	GG	GG	GG	GG	GG	GG	GG	GG
	GG	AG	AG	GG	GG	GG	GG	GG
	GG	AG	GG	GG	GG	GG	GG	
93797426								
8270	rs10237781	7	GG	GG	AG	GG	AG
	GG	AG	GG	GG	GG	GG	AG	AG
	AG	AG	AG	AG	GG	GG	AG	GG
	GG	GG	GG	GG	AG	AG	AG	AG
	AG	GG	GG	GG	AA	AG	AG	
39205006								
8271	rs10237799	7	GG	GG	AG	GG	GG
	GG	GG	GG	GG	GG	GG	GG	GG

GG	GG	GG	GG	GG	AG	GG	AG
GG	GG	GG	GG	GG	GG	GG	GG
GG	AG	GG	AG	AG	GG	AG	

37534044

8272	rs10237831	7	GG	GG	GG	GG	GG

GG	GG	GG	GG	GG	GG	GG	GG
GG	GG	GG	GG	GG	GG	GG	GG
GG	GG	GG	GG	GG	GG	GG	GG
AG	GG	GG	GG	GG	GG	GG	

75179843

8273	rs10237868	7	AA	AA	AA	AA	AA

AA	AA	AA	AA	AA	AA	AA	AA
AA	AA	AA	AA	AA	AA	AA	AA
AA	AA	AA	AA	AA	AA	AA	AA
AA	AA	AA	AA	AA	AA	AA	

53831125

8274	rs1023792	4	AG	AA	AG	GG	AG

AG	GG	AG	AG	AG	AG	GG	GG
AG	AA	AA	AA	AA	AG	GG	AG
AA	GG	GG	AG	GG	AG	GG	AG
AG	AG	AG	GG	AA	AA	AA	

107537390

8275	rs10237942	7	CC	AC	AA	AA	AA

AA	AA	AA	AA	AA	AA	AA	AA
AA	AA	AC	AC	AC	AA	AC	AA
AC	AA	AA	AC	AC	AA	AC	AA
AA	AA	AA	AA	AA	AA	AA	

77239456

8276	rs10237947	7	AC	AC	AC	AA	AA

AC	AA	CC	AC	AC	CC	AC	AC
AC	CC	AC	CC	CC	AC	AC	AA
AA	AC	AA	AA	AC	AA	AA	AA
CC	AC	AA	AC	CC	CC	AC	

157258109

8277	rs1023798	5	CC	CC	CC	CC	CC

CC	CC	CC	CC	CC	CC	CC	CC
CC	CC	CC	CC	CC	CC	CC	CC
CC	CC	CC	CC	CC	CC	CC	CC
CC	CC	CC	CC	CC	CC	CC	

124650768

8278	rs10237984	7	AC	CC	CC	CC	CC

CC	CC	CC	CC	CC	AC	CC	AC
AC	CC	CC	CC	CC	AC	CC	AC
CC	CC	CC	CC	CC	CC	CC	CC
AC	CC	CC	CC	CC	CC	AC	

69298131

8279	rs10238006	7	CC	AC	CC	AC	CC

CC	AC	AC	AC	CC	AA	AA	AC
CC	CC	AC	AC	CC	AC	CC	CC
CC	CC	CC	CC	CC	CC	CC	CC

AA CC AA AC CC CC AC
32376953
8280 rs10238015 7 GG GG GG AG GG
GG AG AA AG AA AA AA AG
AA AG AG GG AG AG AG GG
AG AA AA AG AG AG GG AG
GG GG AA AG AG AA AG
24395900
8281 rs10238021 7 AC AA AC AC AA
AC AA AA AA AA CC AC AC
AC AA AA AA AA AA AA AC
AA AA AA AA AA AA AA AA
AA AA AC AC AA AA AA
107525846
8282 rs10238042 7 AA AA AA AA AA
AG AA AG AG GG AA AA AA
AA AA AA AG AG AA AA AA
AA AG AA AA AG AG GG GG
AA AA AG AG AA AG AA
150124569
8283 rs10238077 7 AG AA AG AG AG
AG AG AG AA AA AA AG AA
AG AA GG AG AG AA AA AA
AA AA AA AA AA AA AA AA
AA AA AG AA AA AA AA
4598404
8284 rs10238099 7 GG GG GG GG GG
GG GG GG GG GG GG GG GG
GG GG GG GG GG GG GG GG
GG GG GG GG GG GG GG GG
GG GG GG GG GG GG GG
63529880
8285 rs10238114 7 AA AA AA AA AA
AA AA AA AA AA AA AA AA
AA AA AG AA AA AA AA AA
GG AA GG GG AA AA AA AA
AA AA AG AA AA AA AG
144865200
8286 rs1023825
2 -- -- -- -- -- -- -- --
-- -- -- -- -- -- -- --
-- -- -- -- -- -- -- --
-- -- -- -- -- -- -- --
-- -- -- -- 79246108
8287 rs10238254 7 AG AA AG AA AA
AA AA AA AA AA AG AG AA
AA AA AA AG AA AG AG AG
AA AG AG AG AA AG AA AA
GG AA AA AA AG AA AG
16719073

8288 rs10238278 7 AA AA AA GG AG
AG GG AA AA AA AA AA AA
AA AA AA AA AA AA AA AA
AA AA AA AA AG AA AG AG
AA AA AA AA AG AG AA
98312921
8289 rs10238349 7 AC AC AC CC CC
CC CC CC CC CC CC CC CC
CC CC CC AC AC CC AC CC
CC CC CC CC CC CC CC CC
AC AC CC AC AC AC CC
54751771
8290 rs10238377 7 AG AA AG AA AG
AA AG AA AA AA GG GG AG
AG AA AA AG AG AG AA AG
AA AA AA AA GG AA AG AG
AA AG AG GG AA AA AA
88102745
8291 rs10238396 7 GG AG AG AA AA
AA AG AG AG AG AG AG GG
GG GG AA AG AG GG GG AG
AG AG AG AG AA GG AG AG
AG AA AA AA AG AA GG
122186718
8292 rs10238421
7 -- -- -- -- -- -- -- --
-- -- -- -- -- -- -- --
-- -- -- -- -- -- -- --
-- -- -- -- -- -- -- --
-- -- -- -- 102044690
8293 rs10238440 7 AA AG AA AA AA
AA AG AG AG AG AA AA AA
AA AA AG AA AA AG GG AA
AA AG AA AG AA GG AG AG
GG AG AA AG AA AA AA
14092993
8294 rs10238442 7 GG GG GG AG AA
AG AA AG AG GG GG GG GG
GG AG GG AG GG GG GG GG
GG GG GG GG GG GG GG GG
AG AG AG GG AG AA GG
16486969
8295 rs10238561 7 AA AG AG AG AG
GG AA AG AG AA AG AG AG
AG AG AG GG GG AG GG AG
AA AG GG AA AA AG AG AG
AA AA AA AA GG AG AG
95241819
8296 rs10238572 7 AA AA AG AG AG
AA AG AG AG AA AA AA AA

AA AG AA AG AG AG GG AG
AA AA AA AA AG AA AG AG
AA AA AA AA AG AA AG
147607585
8297 rs10238581 7 CC CC CC CC CC
CC CC AA AC AA CC CC CC
CC CC CC AC AC CC CC CC
CC CC CC CC CC CC CC CC
AC AC CC CC AC AC AC
67587520
8298 rs10238642 7 CC CC CC CC CC
CC CC CC CC CC CC CC CC
CC CC CC CC CC CC CC CC
CC CC CC CC CC CC CC CC
CC CC CC CC CC CC CC
75967656
8299 rs10238661 7 AA AA AA AA AA
AA AA AA AA AA AA AA AA
AA AA AA AA AA AA AA AA
AA AA AA AA AA AA AA AA
AA AA AA AA AA AA AA
156778724
8300 rs10238664 7 GG GG GG AG AG
GG AG AG AG AG AG AG GG
GG AG AG AG AG GG AG GG
GG GG GG GG GG GG GG GG
AG AG GG AG AG GG AA
111473040
8301 rs10238732 7 GG GG GG GG GG
GG GG GG GG GG GG GG GG
GG GG GG GG GG GG GG GG
GG GG GG GG GG GG GG GG
GG GG GG GG GG GG GG
65429527
8302 rs10238783 7 AA AG AG AG AG
AA GG AA AG AG GG GG AG
AG AA AG GG AG AG AG GG
AG GG GG GG AG GG AG AG
GG GG AA AG GG GG GG
3577665
8303 rs10238785 7 GG GG GG GG GG
GG GG GG GG GG GG GG GG
GG GG GG GG GG GG GG GG
GG GG GG GG GG GG GG GG
GG GG GG GG GG GG GG
6668579
8304 rs10238880 7 AA AA AA AA AA
AA AA AA AA AA AA AA AA
AA AA AA AA AA AG AG AG
AA AA AA AA AA AG AA AA

AA AA AA AA AG AA GG
26474874
8305 rs10238918 7 AC AC AC AC AC
AC AC AC AA AA CC CC CC
AC AC CC AC AC AA AA AA
AC CC CC AC AA AA AA AA
CC AA AA AA AC AC AC
31377369
8306 rs10238921 7 AG GG GG AG AA
AG AA AA AA AA AA AA AA
AA AG AG AG AG AG AA AG
AA AA AA AA AA AG AG AG
AG AA AG AG AG GG AA
135715070
8307 rs10238949 7 AG AA AG GG AA
AG AG AG AG AA GG GG GG
GG GG AG AG AG AA GG AA
AG AG AG AG AG AG AG GG
AA GG AG AG AG AA AG
52245441
8308 rs10238965 7 GG GG GG GG AG
AG GG GG GG GG AA AG AG
AA GG AG GG GG GG GG GG
GG GG GG GG GG GG GG GG
GG AG AG AG GG GG AG
99145845
8309 rs10238969 7 GG AG GG AG AG
GG AA AG AG AG GG GG AG
AG AG GG AG AG AG AA GG
AG AG GG AA AG AA AA AA
GG AG AA AG GG AG GG
35609340
8310 rs10239000 7 GG GG GG AG GG
AG GG AG GG AG GG GG GG
AG AG AG AG AG AA AG AG
GG AG AG GG GG GG GG GG
GG AG GG GG GG GG GG
50332196
8311 rs10239079 7 AA AA AA AA AA
AA AA AA AA AA AA AA AA
AA AA AA AA AA AA AA AA
AA AA AA AA AA AA AA AA
AA AG AA AG AA AA AA
14503078
8312 rs10239100 7 AA AA AA AA AA
AA AA AA AA AC AA AA AA
AA AA AA AC AC AC AA AC
AC AC AC AC AA AA AA AA
AA AA AA AA AC AC AC
87564277

8313	rs10239102	7	AA	AA	AA	AA	AA
AA	AA	AC	AA	AA	AA	AA	AC
AA	AA	AA	AA	AC	AA	AA	AA
AA	AC	AC	AA	AA	AA	AA	AA
AA	AA	AA	AA	AA	AA	AA
33684991
8314	rs1023916	5	AG	AG	GG	AG	AG
GG	AG	GG	GG	GG	GG	GG	AG
AG	AG	GG	GG	GG	GG	GG	GG
GG	GG	GG	GG	GG	GG	GG	GG
GG	AG	GG	AG	GG	GG	GG
178083799
8315	rs10239188	7	GG	GG	GG	GG	GG
GG	GG	GG	GG	GG	AG	AG	AG
GG	AG	GG	GG	GG	GG	AG	GG
GG	GG	GG	GG	AG	GG	GG	AG
AG	AG	AG	AA	GG	GG	AG
94299429
8316	rs10239194
0	--	--	--	--	--	--	--	--
--	--	--	--	--	--	--	--
--	--	--	--	--	--	--	--
--	--	--	--	--	--	--	--
--	--	--	--	0
8317	rs1023921
11	--	--	--	--	--	--	--	--
--	--	--	--	--	--	--	--
--	--	--	--	--	--	--	--
--	--	--	--	--	--	--	--
--	--	--	--	55494172
8318	rs1023924	15	AA	AA	AA	AA	AG
AG	AG	AG	AG	AG	AA	GG	AG
AA	AG	AG	AA	AA	AG	AG	AG
AG	AG	AG	AG	AG	AG	AA	GG
AA	GG	AG	AG	AG	AA	AG
73438622
8319	rs10239242	7	AA	AA	AA	AA	AA
AA	AA	AA	AA	AA	AA	AA	AA
AA	AA	AA	AG	AA	AA	AA	AA
AG	AG	AA	GG	AA	AA	AA	AA
AA	AA	AA	AA	AG	AG	AA
21090300
8320	rs10239261	7	GG	GG	GG	GG	GG
GG	GG	GG	GG	GG	AA	AG	AG
GG	GG	GG	GG	GG	GG	AG	GG
GG	GG	GG	GG	GG	GG	GG	GG
GG	GG	GG	GG	GG	AG	GG
42090075
8321	rs1023933	4	AC	CC	AC	CC	CC
CC	CC	AC	AC	AC	AC	AC	AC

	AC	AC	AC	AC	CC	CC	CC	CC
	CC	AC	AC	CC	CC	CC	CC	CC
	CC	AC	AC	CC	AC	AC	AC	
35149660								
8322	rs10239337		7	AG	AG	AG	GG	GG
	GG	GG	GG	AG	AG	GG	AG	AG
	AG	GG	AG	AG	AG	GG	GG	GG
	GG	GG	GG	GG	GG	GG	GG	GG
	GG	AG	GG	GG	GG	GG	GG	
149507369								
8323	rs10239387		7	AG	AA	AA	AA	AA
	AA	AA	AA	AG	AG	AA	AG	AG
	AA	AA	AG	AG	AG	AA	AA	AA
	AA	AG	AA	AG	AA	AA	AA	AA
	AA	AA	AG	AA	AA	AA	AA	
42604257								
8324	rs10239394		7	AA	AA	AA	AA	AA
	AA	AA	AA	AA	AA	AA	AA	AA
	AA	AA	AA	AA	AA	AA	AA	AA
	AA	AA	AA	AA	AA	AA	AA	AA
	AA	AA	AA	AA	AA	AA	AA	
52174862								
8325	rs10239402		7	AG	AA	AG	GG	AG
	AG	GG	AG	AA	GG	AA	AA	AA
	AG	AG	AG	AG	GG	GG	AG	AG
	GG	AG	AG	GG	AA	AA	AA	AA
	GG	AG	AG	AG	AG	AA	AG	
36788357								
8326	rs1023943		18	AG	AA	AA	GG	GG
	GG	GG	AG	GG	GG	AG	AA	AG
	AG	GG	AG	GG	AG	GG	AG	AG
	GG	GG	GG	GG	AG	GG	AG	AG
	GG	AG	GG	AG	GG	GG	AG	
46170879								
8327	rs10239445		7	GG	AG	GG	AG	AG
	GG	AA	AA	AA	AA	AA	AG	AG
	AA	AA	AA	AG	AA	GG	AG	GG
	AG	AA	AA	AA	AA	AG	AA	AA
	GG	AA	AG	AG	GG	GG	AG	
13674145								
8328	rs10239446		7	AC	AC	CC	AA	AC
	AC	AA	CC	CC	CC	AC	AC	CC
	CC	CC	CC	CC	CC	CC	CC	CC
	CC	CC	CC	CC	CC	CC	CC	CC
	CC	CC	AC	CC	AA	AA	AC	
82960004								
8329	rs1023945		1	AG	AG	AA	AA	AG
	AA	AG	GG	GG	GG	GG	GG	GG
	GG	AG	AG	AA	AG	AG	AG	AA
	AA	AG	AA	AG	GG	AG	AG	AG

AG GG AG AG AG GG AA
145703115
8330 rs10239501 7 AG AG AG AA AG
AG AA AG AA AG GG GG GG
AA AA AG GG AG AA AG AG
AG AG GG GG GG AG GG GG
AG AA GG AG AA AA AA
49852730
8331 rs10239513 7 AA AG AA AG AG
GG AA AA AG AA AA AA AA
AG AA AA AG AA AG GG AA
AA AA AA AA AG AA AA AG
AA AG AA AA AG AG GG
25358941
8332 rs10239517 7 AA AA AA AA AA
AA AA AA AA AA AA AA AA
AA AA AA AA AA AA AA AA
AA AA AA AA AA AA AA AA
AA AA AA AA AA AA AA
73922355
8333 rs10239546 7 AA AA AA AA AA
AA AA AA AA AA AA AA AA
AA AA AG AA AA GG AA AG
AA AA AA AA AA AG AG AG
AG AA AG AG AG GG AA
52992974
8334 rs10239591 7 AA AA AA AG AG
AG AG AG AG AG AG AG GG
AG AG AG AG AA AG GG AG
AA AG AA AG AG AG AG AA
GG AA AA AA AA AA AG
19757731
8335 rs10239645 7 GG GG GG GG GG
GG GG GG GG GG GG GG GG
GG GG GG GG GG GG GG GG
GG GG GG GG GG GG GG GG
GG GG GG GG GG GG GG
149100349
8336 rs10239703 7 AG GG AG GG GG
AG GG AG GG AG GG AG AG
AG GG AG AG AG AG AG GG
GG GG GG GG GG AG GG GG
GG GG AG AG GG AG GG
146486101
8337 rs10239759 7 GG GG GG GG GG
GG GG AG GG AG GG GG GG
GG GG GG GG GG GG AG GG
GG GG GG GG GG AG AG AG
GG AG GG AG GG GG GG
149542524

8338 rs10239799 7 GG GG GG GG GG
GG GG GG GG GG AG GG AG
AG GG GG GG GG GG AG GG
GG GG GG GG GG GG GG GG
GG GG GG GG GG GG GG
120405335
8339 rs10239810 7 AA AA AA AA AG
AA AG GG AG GG AG AG GG
AG GG AG AG AG AA AA AG
GG GG GG GG AA AG AA AA
GG AG AA AG AA AA AA
28416417
8340 rs1023993 4 CC AC AC AA AC
AC AC AA AA AC AC AC AA
AC CC AA AC AC AC AC AC
CC CC CC CC AC AC AC AA
AC AC CC AC CC AC CC
42336312
8341 rs10239961 7 GG GG GG GG AG
AG GG GG GG GG GG GG GG
AG AG GG GG GG GG GG GG
AG GG AG GG GG GG GG GG
GG GG AG GG AG GG AG
24966522
8342 rs10239988 7 AG AG GG GG GG
GG GG AG AG AG GG GG GG
GG GG GG GG GG GG GG GG
AG GG AG AG AG GG GG GG
GG GG GG GG AG AG GG
125623199
8343 rs10240034 7 AG AG AG AG GG
AG GG AG GG AA GG GG GG
GG GG AG AG AG AG AG GG
AG AG GG GG AG GG GG AG
AG AG GG GG AG AA GG
51954436
8344 rs10240138 7 AA AA AA AG AA
AA AG AG AG AA AG AG AA
AA AG AA AA AA GG AG AG
AA AA AG AA AG GG AG AG
AG AG AG GG AG AA GG
95081804
8345 rs10240188 7 GG GG GG GG GG
GG GG AG GG AG GG GG GG
GG GG GG GG GG GG AG GG
GG GG GG GG GG GG GG GG
AG GG GG GG GG GG GG
156806463
8346 rs10240222 7 AG AG AA AG AG
AA GG AG AG AA GG GG GG

	GG	GG	AG	AA	AA	AG	AG	AA
	AG	AA	AA	AG	AA	GG	AG	AG
	AG	AG	AG	AA	AG	AG	AA	
39962864								
8347	rs10240229		7	GG	GG	GG	GG	GG
	GG	GG	AG	GG	AG	GG	GG	GG
	GG	GG	GG	GG	GG	GG	GG	GG
	GG	GG	GG	GG	AG	AG	AG	GG
	AG	GG	GG	GG	GG	GG	AG	
19767162								
8348	rs10240250		7	GG	GG	GG	GG	GG
	GG	GG	GG	GG	GG	GG	GG	GG
	GG	GG	GG	GG	GG	AG	GG	AG
	GG	GG	GG	GG	GG	GG	GG	GG
	AG	GG	GG	GG	AG	GG	AG	
42456634								
8349	rs1024027		2	AA	AA	AA	AG	GG
	GG	AG	AG	AA	AG	AA	AA	AA
	AA	AA	AA	AA	AG	AG	AA	AA
	AA	AA	AA	AA	AA	AA	AA	AA
	AG	AG	AG	AA	GG	GG	AG	
4136145								
8350	rs10240278		7	AC	AC	AC	--	AA
	AC	AC	AC	AC	AC	AC	AA	AC
	AC	AC	AC	CC	AC	AA	AC	AA
	AC	AC	CC	AC	CC	AC	AC	AC
	AC	AC	AA	AA	CC	CC	AC	
12846898								
8351	rs1024029		5	AG	AG	GG	AG	AG
	AG	AG	AG	AA	AG	AG	AA	AG
	AA	AA	AA	AA	AA	GG	AA	AG
	AA	AA	GG	AG	GG	AG	GG	AG
	AA	GG	AA	AG	AG	AG	GG	
2869505								
8352	rs10240295		7	AA	AA	AG	AA	AG
	AG	AA	AG	AA	AG	AG	AG	AA
	AA	AG	AG	AG	AG	AA	AA	AA
	AA	AA	AA	AA	AA	AA	AA	AA
	AA	AA	AA	AA	AA	AA	AA	
63591551								
8353	rs10240320		7	AG	AG	AA	AG	AG
	AG	AG	AG	AG	AG	GG	AG	AG
	AG	AA	GG	GG	GG	GG	AG	GG
	GG	GG	GG	GG	AG	GG	GG	GG
	GG	GG	AA	AG	GG	GG	GG	
106107315								
8354	rs10240330		7	GG	GG	GG	GG	GG
	GG	GG	AG	AG	AG	GG	GG	GG
	GG	GG	GG	GG	GG	GG	GG	GG
	AG	GG	GG	AG	GG	GG	GG	GG

GG AG GG GG GG GG GG
113436542
8355 rs10240361 7 GG GG GG GG GG
GG GG GG GG GG AG AG GG
GG GG GG GG GG GG GG GG
GG AG AG GG GG GG GG GG
GG AG GG GG GG GG GG
93898399
8356 rs10240398 7 AA AA AA AG AA
AA AG GG GG AG AG AG AA
AA AG AG AA AA GG AG GG
AG AG AG AG GG GG GG GG
GG GG GG GG AG AA GG
95082010
8357 rs10240407 7 AA AA AA AA AA
AA AA AA AA AA AG AG AA
AA AA AA AA AA AA AA AA
AA AG AG AA AA AA AA AA
AA AG AA AA AA AA AA
93864890
8358 rs10240422 7 AG AG AA AG AG
AA GG AA AA AA AA AG AA
AA AA AA AA AG GG GG GG
AA AA AA AA AG AA AA AA
GG AG AA AA AA AA AA
4906887
8359 rs1024048 5 AG AG GG AA AA
AA AG AA AA AA AA AG AG
AA AA AG AG GG AG AA AG
AG AG AA AG AA AA AA AA
AG AG AA AG AG AA AG
2783561
8360 rs10240486 7 AA AA AA AA AA
AA AA AA AA AA AA AA AA
AA AA AA AA AA AG AA AA
AA AA AG AG AA AA AA AA
AA AA AA AA AA AA AG
62296695
8361 rs10240503 7 AA AA AA AA AA
AA AA AG AA AA AA AA AA
AA AA AG AA AA AA AA AA
AA AA AA AA AA AA AA AA
AA AA AA AA AA AA AA
147674978
8362 rs10240510 7 AA AA AA AA AA
AA AA AA AA AA AA AA AG
AA AG AA AA AA AA AA AG
AA AA AA AA AA AG AG AG
AA AA AA AA AA AA AA
6324215

8363 rs10240623 7 AA AA AA AA AA
AA AA AA AA AA AA AA AA
AA AA AA AA AA AA AA AA
AA AA AA AA AA AA AA AA
AA AA AA AA AA AA AA
57311000
8364 rs10240771 7 GG GG GG GG GG
GG GG GG GG GG GG GG GG
GG GG GG GG GG GG GG GG
GG GG GG GG GG GG GG GG
GG GG GG GG GG GG GG
151836228
8365 rs10240790 7 GG GG GG GG GG
GG GG AG AG AG GG GG GG
AG GG GG AG AG AG GG GG
GG AG AG GG GG AG GG GG
AG GG AA AG AG AG AG
89880949
8366 rs1024080 3 AA AG AG AG AG
AG GG GG AG AG AG AG AG
AG AG GG AG AG AG AG AA
AG AG AG AG AG AA AG AA
GG AG AG AA AA AA AG
145709954
8367 rs10240943 7 AA AA AA GG AA
AG AG AG AG AG AG AG GG
AG AG AA AA AA AA AA AA
AA AA AA AA AG AG AG AA
AA GG AA AG AA AA AA
23010545
8368 rs1024098 5 AA AA AG AA AA
AA AA AA AA AA AA AA AA
AA AA AG AG AG AA AA AA
AA AA AA AA AA AA AA AA
AA AA AG AG AA AG AA
40559387
8369 rs10240990 7 AA AG AG AA AG
AA AG AA AA AA AA AG AG
AG AG GG GG GG AA AG AA
AA AG AG AA AG AG AG AG
AG AA AG AG GG AG AG
39267066
8370 rs10241023 7 AA AG AG AA AA
AA AA AA AA AA AG GG AG
AA AA AA AG AA AA AA AA
AA AG AA AG AA AA AA AA
AA GG AA AG AA AG AA
3320807
8371 rs10241053 7 GG GG GG AG AG
AG AG GG GG GG GG GG GG

	GG	GG	GG	GG	GG	GG	GG	GG
	GG	AA	AG	AG	GG	GG	GG	GG
	AG	AG	GG	GG	AA	AA	AA	
24062080								
8372	rs10241074		7	AG	AA	AG	AA	AG
	AG	AA	AA	AA	AA	AA	AA	AA
	AA	AA	AA	AA	AA	AA	AA	AA
	AG	AA	AA	AG	AA	AA	AA	AA
	AA	AA	AA	AA	AA	AA	AA	
107464932								
8373	rs10241138		7	AA	AA	AA	AA	AG
	AG	AA	AG	AG	AG	AA	AA	AA
	AG	AG	AA	AA	AG	AA	AA	AA
	AA	AG	AG	AA	AG	AA	AA	AA
	AG	AG	AA	AA	AG	AA	AG	
31096363								
8374	rs1024114		2	AG	GG	--	AG	AG
	AG	AG	AG	AG	AA	AG	--	GG
	AG	AG	AG	GG	AG	AG	GG	AG
	--	GG	GG	GG	GG	AG	AG	GG
	--	AA	AA	AA	AG	AG	AA	
115817986								
8375	rs10241170		7	CC	CC	CC	CC	CC
	CC	CC	CC	CC	CC	CC	CC	CC
	CC	CC	CC	CC	CC	CC	AC	CC
	CC	CC	CC	CC	AC	CC	AC	CC
	AC	AC	CC	AC	CC	CC	CC	
14876917								
8376	rs10241172		7	AA	AC	AC	AA	AA
	AA	AA	AA	AA	AA	AA	AA	AA
	AA	AA	AA	AA	AA	AA	AA	AA
	AC	AA	AA	AC	AA	AC	AC	AC
	AC	AC	AC	AC	AA	AA	AA	
97782116								
8377	rs10241187		7	GG	GG	GG	GG	GG
	GG	GG	GG	GG	GG	GG	GG	GG
	GG	GG	GG	GG	GG	GG	GG	GG
	GG	GG	GG	GG	GG	GG	GG	GG
	GG	GG	GG	GG	GG	GG	GG	
993373								
8378	rs1024119		4	AA	AA	AG	AA	AA
	AG	AG	AG	AG	AG	GG	AA	AG
	AG	AA	GG	AG	AG	AG	AG	AG
	AG	AA	AA	AG	AG	AG	GG	AG
	GG	AA	AG	AA	AA	AG	AA	
127964490								
8379	rs10241207		7	GG	GG	GG	AG	AG
	AG	AG	AG	GG	GG	AA	AA	AG
	GG	GG	GG	GG	GG	GG	GG	GG
	AG	AG	AG	AG	GG	GG	GG	GG

AA GG AG GG GG GG GG
31346073
8380 rs10241209 7 CC CC CC CC AC
AC CC CC CC CC AA AC AC
AC AC AC CC CC CC CC AC
CC AA AC AC CC CC CC CC
CC CC AC CC CC CC CC
40939081
8381 rs10241241 7 AG AA AG AA AA
AA AA AA AA AA AA AA AA
AG AG AA AA AA AA AA AA
AA AA AA AA AA AA AA AA
AA AG AA AG AA AG AG
98531883
8382 rs10241243 7 AA AA AA AA AA
AA AA AA AA AA AA AA AA
AA AA AA AA AA AA AA AG
AA AA AA AA AA AA AA AA
AA AA AA AA AA AA AA
76048676
8383 rs10241355 7 GG GG GG AG AG
AA GG AG AG AA AG AG GG
AG GG GG AG AG AG GG AG
AA AG -- AA AG GG GG GG
GG AG AG GG GG GG GG
96431346
8384 rs1024139 1 AA AA AA AG AA
AA AG AG AA AA AG AG AG
AG AA AG AG AG AG GG AA
AA AA AA AG AG AA AG AA
AA AG AG AG AA AG AA
4640902
8385 rs10241406 7 GG GG GG GG GG
GG GG GG GG GG GG GG GG
GG GG AG AG AG AG AG GG
GG GG GG GG AG GG GG GG
GG GG GG GG AG AG GG
107938376
8386 rs10241412 7 AC CC AC AC CC
CC AC AC AC AC CC CC CC
CC CC CC AC CC CC CC CC
CC AC CC AC CC AC AC AC
AC AC AC AC AC AC AC
7873385
8387 rs10241441 7 CC CC CC CC CC
CC CC CC CC CC CC AC AC
CC CC CC CC CC CC CC CC
CC CC CC CC AC CC AC CC
AC CC CC CC CC CC CC
78027834

8388 rs10241449 7 AG AG AG GG GG
GG GG GG GG GG AG AA AG
AG GG AG AG AG AG AG GG
AG GG AG AG GG AG AG AG
GG GG AG GG AA AA AA
138449419
8389 rs1024148 4 AC AC AC AC AA
AC AC CC AC CC AC AC AC
AC AA AC AA AA CC CC AC
AC CC AC AC AC AA AA AA
AC CC CC CC AA AC AC
153960368
8390 rs10241520 7 AA AA AA AA AA
AA AA AA AA AA AG AG GG
GG GG AG AA AA AG AG GG
AA AA AA AA AA AG AG AG
AG AA AA AA AA AA AA
119856856
8391 rs1024154 21 GG GG GG AG AG
AG AG AA AA GG GG GG GG
GG GG AA AG AG AG AG AG
GG GG GG GG GG GG GG GG
GG GG GG GG GG GG GG
36640545
8392 rs10241552 7 AA AA AA AA AA
AA AA AA AA AA AA AA AA
AA AA AA AA AA AA AA AA
AA AA AA AA AA AA AA AA
AA AG AG AA AA AA AA
106954721
8393 rs10241576 7 GG AG GG AG AG
AG AG AG AA GG GG GG GG
GG GG GG GG GG GG GG GG
AG AG AA GG GG GG GG GG
AA GG GG GG AG AA GG
37859423
8394 rs10241609
7 -- -- -- -- -- -- -- --
-- -- -- -- -- -- -- --
-- -- -- -- -- -- -- --
-- -- -- -- -- -- -- --
-- -- -- -- 24312294
8395 rs10241710 7 AG AG AA GG AG
AG GG AG AG AG AA AA AA
AG AG AG AG AG AA AG AG
AG AG AG AG AG AA AA AA
GG GG GG GG GG AG GG
34485137
8396 rs10241753 7 GG GG GG AG AG
AA GG GG GG GG AG AG GG

AG AG AG GG AA GG GG AG
GG AG AG GG GG AG AG AG
AG GG AG GG AG GG AA
19042101
8397 rs10241764 7 AA AA AA AA AA
AA AA AA AA AA AA AA AA
AA AA AA AA AA AA AA AA
AA AA AA AA AA AA AA AA
AA AA AA AA AA AA AA
65372882
8398 rs10241858 7 AA AA AA AA AA
AA AA AA AA AA AA AA AA
AA AA AA AA AA AA AA AA
AA AA AA AA AA AA AA AA
AA AA AA AA AA AA AA
64712619
8399 rs1024187 6 GG AG AG GG AG
AG GG GG GG GG GG GG GG
GG GG GG GG GG GG GG GG
AG AG AG AG AG AG AG AG
AG AG GG GG GG GG GG
62415600
8400 rs10241873 7 CC AC CC AA AA
AA AC AC AC AC AC AC AA
AC AC CC CC CC AA AA AC
AC CC AC AC CC AA AC AC
CC CC CC CC AA AC AC
148940873
8401 rs10241890 7 AG GG AG AG AG
AA GG AA AA AA AA AG AG
AG AG AG AG AG AA AA AG
GG AG GG AG AA AG AG AG
AA GG AA AG AG GG AA
3220259
8402 rs1024196 6 AA AA AA AA AA
AA AA AA AA AA AA AA AA
AA AA AA AA AA AA AA AA
AA AA AA AA AA AA AA AA
AA AA AA AA AA AA AA
56507489
8403 rs10241971 7 AA AA AA AA AA
AA AA AA GG GG AG AG AA
AA AA AG AA AA AA AG AA
AG AA AG AG AA AA AA AA
AG AA AA AA AG AG AA
152604776
8404 rs10241998 7 AA AG GG AG AG
GG AA AG AG AG AA AG AG
AG AA AA AA AA AG AG AG
AA AG AA AA AG AG AA AA

	AA	GG	AA	AG	AA	AG	AG	
134262441								
8405	rs1024206		20	CC	CC	CC	CC	CC
	CC	CC	CC	CC	CC	CC	CC	CC
	CC	CC	CC	CC	CC	CC	CC	CC
	CC	CC	CC	CC	CC	CC	CC	CC
	CC	CC	CC	CC	CC	CC	CC	
41704829								
8406	rs10242122		7	GG	GG	GG	GG	GG
	GG	GG	AG	GG	GG	AG	GG	AG
	GG	AG	AG	AG	GG	GG	GG	GG
	AG	GG	GG	GG	GG	AG	AG	AG
	AG	GG	GG	GG	GG	GG	GG	
4125585								
8407	rs10242146		7	CC	CC	CC	CC	CC
	CC	CC	CC	CC	CC	CC	CC	CC
	CC	CC	CC	CC	CC	CC	CC	CC
	CC	CC	CC	CC	CC	CC	CC	CC
	CC	CC	CC	CC	CC	CC	CC	
155322045								
8408	rs1024217		3	GG	GG	GG	AG	AG
	AG	GG	GG	GG	GG	AG	AG	AG
	AA	AA	GG	GG	GG	GG	GG	GG
	GG	AG	GG	AG	GG	GG	GG	GG
	GG	GG	AG	GG	GG	GG	GG	
197102197								
8409	rs10242171		7	AG	AG	AA	AG	GG
	AG	GG	GG	AG	GG	GG	GG	GG
	AG	GG	AG	AG	AG	GG	GG	GG
	GG	GG	GG	GG	GG	GG	GG	GG
	GG	AG	GG	GG	GG	GG	GG	
28944145								
8410	rs10242174		7	AG	GG	GG	AG	GG
	AG	GG	AG	GG	AG	GG	GG	GG
	GG	GG	AG	AG	AG	GG	GG	GG
	AA	GG	AG	AG	GG	AA	AG	AG
	AG	GG	AG	GG	AG	AA	GG	
42160959								
8411	rs10242204		7	AG	AG	AG	GG	GG
	GG	GG	GG	GG	GG	AG	AA	AG
	GG	GG	GG	GG	GG	GG	AG	GG
	GG	GG	GG	GG	GG	GG	GG	GG
	GG	AG	GG	GG	GG	AG	GG	
11821125								
8412	rs10242247		7	AA	AG	AA	AA	AA
	AA	AA	AA	AA	AA	AG	AG	AG
	GG	AG	AA	AA	AA	AA	AG	AA
	AG	AG	AG	AG	AA	AA	AA	AA
	AA	AA	AA	AA	AA	AA	AG	
22831954								

8413 rs10242264 7 AG AA AA AA AG
AA AG AA AA AA AA AA AA
AA AA AG AG AG AA AG AA
AA AG AA AG AA AA AA AA
AA AG AA AG AA AG AA
26626743
8414 rs1024229 1 AC AC AC AA AA
AA AA AA AC CC AA AA AA
AA AC CC AC AC AA AA AA
AA AC AA AC AA AC AC AC
AC AC CC CC AA AA AA
67512903
8415 rs10242296
7 -- -- -- -- -- -- -- --
-- -- -- -- -- -- -- --
-- -- -- -- -- -- -- --
-- -- -- -- -- -- -- --
-- -- -- -- 151912679
8416 rs10242397 7 GG AG GG GG GG
GG GG AG AA AA GG GG GG
GG GG GG GG AG GG GG GG
AG AG GG AG GG GG GG GG
GG AG AG AA AG GG AG
8398372
8417 rs10242581 7 GG GG AG GG AA
AG AG AG GG AA AG AG GG
AG AA AA AG AG AG AG AG
AA AA AG AG GG AA AG AG
AG GG AA AG AG AG GG
63801896
8418 rs10242595 7 AA AA AA AA GG
AG AG AA AG AA AA AA AA
AA AA AG AG AA AG GG AA
AG AG AG AG AG AG AG GG
AA GG AG GG AA AG AA
22774231
8419 rs1024265 10 AA AA AA AC AA
AC AA AA AA AA AA AA AA
AA AA AA AA AA AA AA AA
AA AC CC CC AA AA AA AA
AA AC AA AC AA AA AA
119487704
8420 rs10242670 7 AA AG AG AA AG
AA AG GG GG GG AA AA AA
AA AA GG GG GG AG GG AG
AG GG GG AG AG AA AG AA
AG AG AG GG AA AG AG
48711664
8421 rs1024268 1 AA AA AA AG GG
GG AG AA AG AG GG GG GG

	GG	GG	AA	AG	AA	AG	AG	AG
	AG	AG	AG	GG	GG	AG	AG	AG
	AG	AG	AA	AA	GG	AG	GG	
70117404								
8422	rs10242681		7	AG	AG	AG	GG	AA
	AG	AG	AA	AG	AG	AA	AG	AG
	AA	AA	AA	AA	AA	AG	AG	AA
	AA	AA	AA	AA	AG	AG	AA	AG
	AG	GG	AG	GG	AA	AA	AG	
41286287								
8423	rs10242687		7	GG	GG	GG	GG	GG
	GG	GG	GG	GG	GG	AG	AG	AA
	GG	AG	GG	GG	GG	GG	GG	AG
	GG	GG	GG	GG	GG	GG	GG	GG
	GG	GG	AG	GG	GG	AG	AG	
26690417								
8424	rs1024276		11	AG	GG	AG	AG	AG
	GG	AA	AA	AA	AA	AA	AG	AG
	AG	AG	AG	AA	GG	AG	AG	GG
	AG	GG	AG	GG	AA	AG	AA	AG
	AG	AA	AG	AA	AG	AG	AG	
34621004								
8425	rs10242784							
7	--	--	--	--	--	--	--	--
	--	--	--	--	--	--	--	--
	--	--	--	--	--	--	--	--
	--	--	--	--	--	--	--	--
	--	--	--	--	140831341			
8426	rs10242803		7	GG	GG	GG	GG	GG
	GG	GG	GG	GG	GG	GG	AG	AG
	AA	AA	AG	AG	AA	AA	GG	AA
	AG	GG	GG	AG	GG	AG	AG	AG
	AG	GG	GG	GG	GG	GG	GG	
49408840								
8427	rs10242831		7	GG	GG	GG	GG	AG
	AG	GG	AA	AG	AG	GG	GG	GG
	AG	AG	GG	AG	GG	AG	GG	GG
	GG	GG	GG	GG	AG	AA	AA	AG
	GG	GG	GG	GG	GG	GG	GG	
49281219								
8428	rs10242846		7	AA	AA	AA	AG	AG
	GG	AA	AG	AG	AA	GG	GG	GG
	AG	AG	AA	AA	AA	AG	AG	AA
	GG	AA	AG	AA	GG	AG	AG	AG
	GG	GG	GG	GG	AA	AA	AA	
17588072								
8429	rs10242865		7	AC	AC	CC	AA	AA
	AA	AA	AC	AC	AC	AC	CC	AC
	AC	AC	CC	CC	CC	CC	CC	AC
	AA	AA	AC	AA	AC	AC	CC	CC

CC AC AA AA CC CC AC
101230768
8430 rs10242889 7 GG GG GG GG GG
GG GG GG GG GG GG GG GG
GG GG AG GG GG AG GG GG
GG GG GG GG GG AG GG GG
GG GG GG GG GG GG GG
46745813
8431 rs10242903 7 GG GG GG AG GG
AG GG GG GG GG AG GG AG
AA GG AG GG GG GG GG AG
AG AA AG AG AG GG GG GG
GG GG GG GG AG GG AA
97741795
8432 rs10242920 7 AC CC CC CC AC
AC CC CC CC CC CC AC AC
CC CC AC AC AC AC CC AC
AC CC AC CC AC CC CC CC
CC AC CC AC AC AC AC
37369557
8433 rs10242940 7 GG GG GG GG GG
GG GG GG GG GG GG GG GG
GG GG GG GG AG GG GG GG
GG GG GG GG AG GG GG AG
GG GG AG GG GG GG GG
42906180
8434 rs10242944 7 AA AG AG AA AA
AA AG AA AA AA AA AA AA
AA AA AA AA AA AA AA AA
AA AA AA AA AA AA AA AA
AA AA AG AA AA AA AA
126648289
8435 rs10242959 7 AG GG AG GG GG
GG GG GG GG GG AG GG AG
AG GG AG GG GG GG GG AG
AG AG GG AG AG GG GG GG
AG AG AG AG GG AG GG
115900262
8436 rs10243038 7 AA AA AA AA AA
AA AA AA AA AA AA AA AA
AA AA AG AG AG AA AA AA
AA AA AA AA AG AA AG AA
AA AG AA AA AA AA AA
93455006
8437 rs1024307 14 AG GG GG GG GG
GG GG GG GG GG GG GG GG
GG GG AG AG GG GG GG GG
GG GG GG GG GG AG AG GG
GG GG GG GG GG GG GG
83308389

8438	rs10243089	7	AA	AA	AA	AA	AG
	AA	AG	AA	AA	AA	AA	AA	AG
	AA	AG	AA	AA	AA	AG	AG	GG
	AA	AA	AA	AA	AA	AG	AG	AG
	AA	AA	AA	AA	AA	AA	AA	
6329903								
8439	rs10243091	7	AA	AA	AA	AA	AA
	AA	AA	AA	AA	AA	AA	AA	AA
	AA	AA	AA	AA	AA	AA	AA	AA
	AA	AA	AA	AA	AA	AA	AA	AA
	AA	AA	AA	AA	AA	AA	AA	
15753487								
8440	rs1024311	1	CC	CC	CC	CC	CC
	CC	CC	CC	CC	CC	CC	CC	CC
	CC	CC	CC	CC	CC	CC	CC	CC
	AC	CC	AC	AC	AC	CC	CC	AC
	CC	CC	CC	CC	CC	CC	CC	
52457159								
8441	rs1024314	1	GG	GG	GG	GG	GG
	GG	GG	GG	AG	GG	GG	GG	GG
	GG	GG	GG	GG	GG	GG	GG	GG
	GG	GG	GG	GG	GG	AG	AG	AG
	AG	GG	GG	GG	GG	GG	GG	
88212437								
8442	rs1024318	21	AC	CC	AC	CC	AC
	CC	AC	AA	AA	AA	AC	AA	AC
	AC	AC	AC	AC	AC	CC	CC	AC
	AC	AA	AC	AC	AA	AC	AA	AA
	CC	AC	AA	AC	AC	CC	AA	
25553212								
8443	rs10243213	7	GG	GG	GG	GG	GG
	GG	GG	GG	GG	GG	GG	GG	GG
	GG	GG	GG	GG	GG	GG	GG	GG
	GG	GG	GG	GG	GG	GG	GG	GG
	--	GG	GG	GG	GG	GG	GG	
98060601								
8444	rs1024323	4	AA	AG	GG	GG	GG
	AG	GG	GG	GG	GG	AG	AG	AG
	AA	AA	GG	AG	AG	AG	GG	AA
	AG	AG	AG	AG	AG	GG	AG	AG
	GG	GG	GG	GG	AG	AG	GG	
3006043								
8445	rs1024326	X	GG	AG	AG	AA	GG
	AA	AG	GG	GG	GG	AG	AG	GG
	AG	AG	GG	GG	GG	GG	GG	GG
	GG	GG	GG	GG	GG	AA	AG	GG
	AG	GG	GG	GG	AA	AG	AA	
103287989								
8446	rs10243282	7	GG	AG	AG	AA	GG
	AG	AG	AA	AA	AG	AG	AG	AG

AG GG GG AG AG GG GG AG
AG AG GG GG AA AA AA AA
AG AG GG AG AG AA AG
85711326
8447 rs10243348 7 AG AG GG AG AG
AG AG AA AA AG AA AA AG
AG GG AA AG AG AA AA AA
AG GG GG AG AA AG AA AA
AG AA AG AG AG AG AA
82508211
8448 rs10243418 7 AG GG GG AG AG
AG GG GG GG AG GG AG AG
AG GG GG GG GG GG GG GG
GG GG GG GG GG GG GG GG
AG GG GG GG GG GG AG
155989669
8449 rs10243420 7 AG AA AG GG AG
GG AG AA AA AG AA AA AG
GG AG GG GG GG GG AG AG
AG GG GG AG GG AA AG AG
GG AG AG AA AG GG AA
93066173
8450 rs1024344 11 AA AA AG GG AG
AG GG AG AA AA AG AG GG
AG GG AG AG AG AG AG GG
AG GG AG AG AA GG AG AG
AG AG GG GG AG GG AG
114958994
8451 rs10243460 7 GG GG GG GG GG
GG GG AG GG GG GG GG GG
GG GG GG GG GG GG GG GG
GG GG GG GG GG GG GG GG
GG GG GG GG GG GG GG
153194173
8452 rs10243471 7 AA AA AA AA AA
AA AA AA AA AA AA AG AG
AA AA AA AA AA AA AA AA
AG AA AA AG AA AA AA AA
AA AA AA AA AA AA AA
49335962
8453 rs10243532 7 GG AG GG GG GG
AG AG AG AA AG AG AG AA
AG GG AG AG AG AA AA AA
AG AG AG AG AG AA AA AA
AA AG AA AA AA AA AA
156438462
8454 rs1024355 8 GG GG GG GG GG
GG AG GG GG GG GG GG GG
GG GG GG GG GG AG GG GG
GG GG GG GG GG GG GG GG

GG GG GG GG GG GG GG
17218629
8455 rs10243554 7 AA AA AA GG AA
AG AG AG GG GG GG GG GG
AG AG AG AG GG AG GG AA
GG GG GG GG AG GG GG GG
GG GG GG GG AG AG AG
33888495
8456 rs10243593 7 AG AG AA AG AA
AA AG AG AG AA GG GG GG
AA AA GG AG AG AG AG AA
AG AA AA AG AG GG AG AG
AA AG AG AA AA AA AA
39972125
8457 rs10243603 7 GG GG AG GG GG
GG GG GG AA AG GG GG GG
AG AG AG AG AA AG AG AA
GG AG AG GG AG GG AG AG
AG GG AA AG AA AA AG
31587618
8458 rs10243634 7 GG GG AG GG GG
GG GG GG GG AG GG GG AG
AG AG AA AG AA AG GG GG
GG AG GG AG GG AG AG AG
AA GG AA AG AG GG AG
34792221
8459 rs10243659 7 AC CC CC AA AC
AC AA CC AC CC AC AC CC
AC AC AC CC CC CC CC AC
CC CC CC CC CC CC CC CC
AC CC CC CC CC CC CC
28389760
8460 rs10243669 7 AG AG AG AA AG
AA AG AA AA AA AA AA AA
AA AA AA AA AA AA AA AA
AG AA AA AG AA AA AA AA
AA AA AA AA AA AA AA
45915907
8461 rs10243674 7 AA AA AA AA AG
AG AA AA AA AA AA AA AA
AG AG AA AA AA AG GG AA
AA AA AA AA AG AG AG GG
AA AG AA AG AG AG AA
17112981
8462 rs10243696 7 GG GG GG GG GG
GG GG GG GG GG GG GG GG
GG GG GG GG GG GG GG GG
GG GG GG GG GG GG GG GG
GG GG GG GG GG GG GG
56775205

8463 rs1024371 17 GG GG GG AG GG
AG GG GG GG GG GG GG GG
GG GG GG GG GG GG GG AG
GG GG AA AA GG AG AG GG
AG AA GG AG GG GG GG
10054325
8464 rs10243723 7 GG GG GG GG GG
GG GG GG GG GG GG GG GG
GG GG GG GG GG GG GG GG
GG GG GG GG GG GG GG GG
GG GG GG GG AG GG AA
46532584
8465 rs10243744 7 GG GG GG AA AA
AA AG GG GG GG GG GG GG
GG AG AG AA AA GG GG GG
AA AG AA AA GG GG GG GG
AG AA AA AA GG GG GG
148636289
8466 rs10243783 7 GG GG GG GG GG
GG GG GG GG GG GG GG GG
GG GG GG GG GG GG GG GG
GG GG GG GG GG GG GG GG
GG GG GG GG GG GG GG
150110040
8467 rs10243801 7 GG AG GG GG AG
AG GG AG GG AG AA AG AG
GG GG AG AG AG AG GG AA
GG GG GG GG AG AG AG AA
GG AG AG AG AG AG GG
43228198
8468 rs10243825 7 AA AA AA AA AA
AA AA AA AA AA AA AA AA
AA AA AA AA AA AG AG AA
AA AA AA AA AA AA AA AA
AA AG AA AG AA AA AA
88607314
8469 rs10243842 7 GG GG GG GG AG
AG AG AA AA AG AG GG AG
AA AA GG GG GG AG AG AG
AG AG AG AG AG AG AG GG
AG AG AA AG GG GG GG
13249235
8470 rs10243846 7 AA AA AA GG AG
AG GG AG AA AA AG AG AA
AG AG AG AG AG GG GG GG
GG AG GG AG GG AG GG GG
AA GG AG GG AG AG AG
8062590
8471 rs10243870 7 AA AA AA AA AA
AA AA AA AA AA AA AA AA

	AA	AA	AA	AC	AA	AC	CC	AC
	AC	AA	AA	AA	AA	AA	AA	AA
	AA	AA	AA	AA	AA	AA	AA	
15215277								
8472	rs10243871		7	GG	GG	GG	AG	AA
	AA	AG	AG	AG	GG	GG	AG	AG
	AA	AA	AG	AA	AA	AG	AA	AA
	AA	AA	AA	AA	AG	AG	AA	AG
	AA	AG	AA	AG	GG	GG	AG	
55423050								
8473	rs10244045		7	GG	GG	GG	AG	AG
	AG	AG	GG	GG	GG	GG	GG	GG
	GG	GG	GG	GG	GG	GG	GG	AG
	GG	GG	GG	GG	GG	GG	GG	GG
	GG	GG	GG	GG	GG	GG	GG	
20777911								
8474	rs10244128		7	GG	GG	GG	GG	AG
	AG	GG	GG	GG	GG	GG	GG	GG
	GG	GG	GG	GG	GG	GG	GG	GG
	GG	GG	GG	GG	GG	GG	GG	GG
	GG	GG	GG	GG	AG	AG	GG	
69169544								
8475	rs10244143		7	GG	GG	GG	AG	AG
	AA	GG	AA	AA	AG	GG	GG	GG
	GG	AG	GG	AG	GG	GG	GG	AG
	AA	AG	AA	AG	AA	AG	AG	AG
	AG	AG	AG	AG	AA	AG	AG	
15042959								
8476	rs10244145		7	GG	GG	GG	GG	GG
	GG	GG	AG	AG	AG	GG	GG	GG
	GG	GG	AG	GG	GG	GG	GG	GG
	GG	GG	GG	GG	GG	GG	GG	GG
	GG	GG	GG	GG	GG	GG	GG	
156601463								
8477	rs10244230		7	GG	GG	GG	GG	GG
	GG	GG	GG	GG	GG	GG	GG	GG
	GG	GG	GG	GG	GG	GG	GG	GG
	GG	GG	GG	GG	GG	GG	GG	GG
	GG	GG	GG	GG	GG	GG	GG	
94571629								
8478	rs10244247		7	GG	GG	GG	GG	AG
	AG	GG	GG	GG	GG	GG	GG	GG
	GG	GG	GG	GG	GG	GG	GG	GG
	GG	GG	GG	GG	GG	GG	GG	GG
	GG	GG	GG	GG	AG	AG	GG	
69193819								
8479	rs10244268		7	AA	AG	AG	AA	AA
	AA	AA	AA	AA	AA	AA	AA	AA
	AA	AG	AA	AA	AA	AA	AG	AG
	GG	GG	GG	GG	GG	AA	AG	AG

AA AA AA AA AA AA AA
81466303
8480 rs10244276 7 GG GG GG GG GG
GG GG GG GG GG GG GG GG
GG GG GG GG GG GG GG GG
GG GG GG GG GG GG GG GG
GG GG GG GG GG GG GG
94766767
8481 rs1024429 X GG AG AG AA GG
AA AG GG GG GG GG GG GG
AG AG AA AG AG AA AG AA
AA AA AA AA AG GG GG AA
AG AG GG GG AG AA GG
109206199
8482 rs10244429 7 AA AG AG AG GG
GG AG AA AA AG AG AA AG
AA AA AG AG AA GG AG AG
AA AA AA AA AG GG GG AG
GG GG AG GG AG AG AG
57246707
8483 rs10244430 7 AA AA AA AA AA
AA AA AA AA AA AA AA AA
AA AA AA AA AA AA AA AA
AA AA AA AA AA AA AA AA
AA AA AA AA AA AA AA
73522031
8484 rs10244456 7 GG GG GG GG GG
GG GG GG GG GG GG GG GG
GG GG GG GG GG GG GG GG
GG GG GG GG GG GG GG GG
GG GG GG GG GG GG GG
65360715
8485 rs1024447 19 GG GG GG GG GG
GG GG GG GG GG GG GG GG
GG GG GG GG GG GG GG GG
GG GG GG GG GG GG GG GG
GG GG GG GG GG GG GG
18959552
8486 rs10244562 7 GG GG GG AG AG
GG AG GG GG GG GG GG GG
AG AG GG GG GG GG GG GG
GG GG GG GG GG AG GG GG
GG GG GG GG AG GG AG
156353250
8487 rs10244616 7 AG AG AG AA AA
AA AA AG AG AA GG AG AG
AA AA AA AA AA AA AA AA
AA AA AA AA AA AG AG AG
AA AA AA AA AA AA AA
54365034

8488	rs10244620		7	AG	AG	GG	AG	AA
	AG	AA	AG	AG	AG	AA	AG	AG
	AG	AG	AG	AG	AG	GG	GG	GG
	AG	AA	AA	AG	AG	AG	AG	AG
	AA	AG	AG	AG	AA	AA	AG	
113315926								
8489	rs10244685		7	GG	GG	GG	GG	GG
	GG	GG	GG	GG	GG	GG	GG	GG
	GG	GG	GG	GG	GG	GG	GG	GG
	GG	GG	GG	GG	GG	GG	GG	GG
	GG	GG	GG	GG	GG	GG	GG	
76962178								
8490	rs10244707		7	GG	AG	GG	GG	GG
	GG	GG	GG	AG	GG	GG	GG	GG
	GG	GG	GG	GG	GG	AG	GG	AA
	GG	GG	GG	GG	GG	GG	GG	GG
	GG	GG	AG	GG	GG	GG	GG	
45170510								
8491	rs10244748		7	AA	AA	AA	AG	AG
	AG	AA	AA	AG	AA	AA	AA	AA
	AA	AA	AA	AA	AA	AA	AA	AG
	AA	AA	AA	AA	AG	AA	AA	AA
	AA	AA	AA	AA	AA	AA	AA	
150041610								
8492	rs10244774		7	GG	GG	GG	GG	AG
	AG	GG	GG	GG	GG	AA	AG	AG
	GG	GG	GG	GG	GG	GG	GG	GG
	AG	GG	GG	AG	GG	GG	GG	GG
	GG	GG	GG	GG	GG	GG	GG	
41401679								
8493	rs10244840		7	AG	AA	AA	AA	AA
	AA	AA	AA	AG	AG	AA	AG	AG
	AA	AA	AG	AG	AG	AA	AA	AA
	AA	AG	AA	AG	AG	AA	AG	AA
	AA	AG	AG	AG	AA	AA	AA	
42505123								
8494	rs1024489		5	GG	GG	GG	AG	AG
	AA	AG	GG	AA	GG	AA	AA	AG
	AG	AG	AG	GG	AG	AG	AG	GG
	AG	AG	GG	AG	GG	GG	GG	GG
	AG	GG	GG	GG	AG	GG	AA	
9356174								
8495	rs10244948		7	AG	AG	AG	AG	AA
	AG	AA	AG	GG	AG	AA	AA	AA
	AA	AA	AA	AG	AG	AA	AG	AA
	GG	AA	AG	AG	AA	AA	AA	AA
	AA	AA	AG	AG	AG	AG	AA	
27988862								
8496	rs10244971		7	AA	AA	AA	AA	AA
	AA	AA	AA	AA	AA	AA	AA	AA

AA AA AA AA AA AA AA AA
AA AA AA AA AA AA AA AA
AA AA AA AA AA AA AA
49446606
8497 rs10244984 7 AA AA AA AA AG
AG AA AA AA AA AA AA AG
AA AG AA AA AG AA AA AG
AA AA AA AA AA AA AA AA
AA AA AA AA AA AG AA
7150584
8498 rs10245006 7 AG GG GG GG GG
GG GG GG GG AG GG GG GG
GG GG AA AA AA AG AG AG
GG GG -- GG GG GG GG GG
AG GG AG AG AG AG GG
49617533
8499 rs10245008 7 AG AG GG AG AG
AG AG AA AA AA GG AG AG
AG AG AG AG AG AA AG AA
AA AA AG AA AG AA AG AG
AG AG AG AG AG AG AG
101513404
8500 rs10245045 7 AG GG AG GG AG
AG GG GG AG AG AG AG AG
AG AG GG GG GG AG GG AA
AG GG AG GG AG AG GG GG
AG GG GG GG GG GG AG
11203816
8501 rs10245106 7 GG GG GG AG GG
AG GG AG AG AG AG AG AA
AG AG AG AG AG GG AG GG
GG AA AG AG AG GG AG GG
AA GG GG GG GG GG AG
19650053
8502 rs10245185 7 GG AG GG AG AG
AG AG AG GG AG AA AG AG
GG AG AG AG AG AG GG GG
AG AG AG AG AG GG AG AG
AA GG AA AG AA AA AG
147900046
8503 rs10245186 7 AA AA AA AA AG
AG AA AA AA AA AA AA AG
AA AG AA AA AA AG AG AG
AG AA AG AA AG AG AG AG
AA AA AA AA AA AA AA
6304923
8504 rs10245203 7 AA AG AG AA GG
AG AG AA AA AG AG AA AG
AA AA AG AG AA GG AG AG
AA AA AA AA AG GG GG AG

GG GG AG GG AG AG AG
57217249
8505 rs10245239 7 AG AA AG AA AA
AA AA AA AA AA AA AA AA
AA AA AG AG AG AA AA AG
AG AA AG AG AA AA AA AA
GG AG AA AG AA AA AG
137743236
8506 rs10245387 7 CC CC CC CC CC
CC CC CC CC CC CC CC CC
CC CC CC CC CC CC CC CC
CC CC CC CC CC CC CC CC
CC CC CC CC CC CC CC
100915162
8507 rs10245396 7 AA AA AA AA AA
AA AA AC AC AA AA AA AA
AC AC AC AC AC AA CC AA
AA AA AA AA AA AA AA AA
AC AC AA AC AC AA AC
55705566
8508 rs10245403 7 GG AG AG AA AA
AA AG GG GG GG AG GG AG
AG AG AA AG AG GG GG GG
GG AG GG GG GG AA AG AG
AG AG AG AA AG AG AA
125604657
8509 rs10245441 7 AA AA AA AA AA
AA AA AA AA -- AA AA AA
AG AG AA AA AA AA AA AA
AA AA AA AA AA AA AA AA
AA AA AA AA AA AA AA
43010631
8510 rs10245472 7 GG GG GG GG GG
GG GG GG GG GG GG GG GG
GG GG GG GG GG AG GG GG
GG GG GG GG GG AG AG AG
GG GG GG GG GG AG GG
55147478
8511 rs1024551 7 GG GG GG GG GG
GG GG GG GG GG GG GG GG
GG GG GG GG GG GG GG GG
GG GG GG GG GG GG GG GG
GG AG GG GG GG GG GG
28918565
8512 rs10245560 7 GG GG GG AA AG
AG AG GG GG GG GG GG GG
GG GG AG AA AA AG GG AG
GG AA GG GG AA GG AG AG
GG GG AG AG GG AG AG
129199328

8513 rs1024557 7 AG AG AA AA AA
AA AA AA AA AA AG AG AA
AA AG AG GG GG AG AA AA
AG GG GG AG GG AG AG AG
AG AG AG AG AG AG GG
89680075
8514 rs10245592 7 AG AA AG AG AG
AA AG AG AG AG AA AA AA
AA AA AA AA AA AA AA AG
AG AG AA AG AA AA AA AA
AG AA AA AA AG AA AG
115527064
8515 rs10245608 7 AA AA AA AA GG
AG AG GG AG AG GG AG AG
GG GG GG GG GG AG AG GG
GG GG GG GG AG AG GG AG
AA AA AG AA AG GG AA
41241212
8516 rs10245618 7 GG GG GG GG GG
GG GG GG GG GG GG GG GG
GG GG GG GG GG GG GG GG
GG GG GG GG GG GG GG GG
GG GG GG GG GG GG GG
66379783
8517 rs10245620 7 GG GG GG GG GG
GG GG GG GG GG GG GG GG
GG GG GG GG GG GG GG GG
GG GG GG GG GG GG GG GG
GG GG GG GG GG GG GG
98922374
8518 rs10245696 7 CC CC CC AA AC
AA AC AA AA AA AA AA AC
AC AC AC AC AC AC AC AA
AC AC CC AC AC AC AC AA
CC CC AC CC AC AA AC
90449362
8519 rs10245710 7 GG GG GG GG GG
GG GG GG GG GG GG GG GG
GG GG GG GG GG GG GG GG
GG GG GG GG GG GG GG GG
GG GG GG GG GG GG GG
73665016
8520 rs10245730 7 GG GG GG GG GG
GG GG AG AG AG GG AG AG
GG AG AG GG GG GG GG GG
GG GG GG GG AG GG GG GG
AG AG AG AG AG GG AG
103609900
8521 rs10245760
7 -- -- -- -- -- -- -- --

	--	--	--	--	--	--	--	--
	--	--	--	--	--	--	--	--
	--	--	--	--	--	--	--	--
	--	--	--	--	93746499			
8522	rs1024577		7	GG	AG	GG	AA	AA
	AA	AA	AA	GG	AG	AG	GG	AG
	GG	AG	AA	AA	AA	AA	AA	AG
	GG	AA	GG	GG	AG	AA	AG	AG
	AA	AA	AA	AA	GG	GG	AG	
148321180								
8523	rs10245818		7	GG	GG	AG	AA	AA
	AA	AA	AA	AA	AA	AA	AA	AA
	AA	AA	AA	AA	AA	AA	AA	AG
	AG	AA	AG	AG	AA	AA	AA	AA
	AA	AA	AA	AA	AG	AA	AG	
129257508								
8524	rs1024582		12	GG	AG	AG	GG	GG
	GG	AG	AA	GG	GG	GG	AA	AG
	AG	AG	AG	AG	AG	GG	GG	GG
	GG	GG	GG	GG	AG	AG	AG	AA
	GG	GG	GG	GG	GG	GG	GG	
2402246								
8525	rs10245886		7	AA	AA	AC	AC	AA
	AA	AC	AA	AA	AA	AA	AA	AA
	AA	AA	AC	AC	AC	AC	AA	AC
	AC	AA	AA	AC	AA	AC	AC	AC
	AA	AA	AA	AA	AA	AA	AC	
47580445								
8526	rs10245919		7	AA	AA	AA	AG	AA
	AG	AA	AG	AG	AG	AG	AA	AG
	AG	AA	AA	AG	AA	AG	GG	AA
	AA	GG	AG	AG	AG	AA	AG	AA
	GG	AA	AA	AA	AA	AA	AA	
19549650								
8527	rs10245938		7	AA	AG	AA	AG	AA
	AA	AG	GG	GG	GG	AA	GG	AG
	AA	AG	AG	AA	AA	AG	AA	GG
	AG	GG	AG	GG	GG	AG	GG	GG
	GG	AG	AG	AG	AA	AG	AG	
9569435								
8528	rs10245966		7	GG	GG	GG	GG	GG
	GG	GG	GG	GG	GG	AG	GG	AG
	AG	GG	GG	AG	AG	GG	GG	GG
	GG	AG	AG	GG	GG	GG	GG	GG
	GG	GG	GG	GG	GG	GG	GG	
104240125								
8529	rs1024599		4	GG	GG	GG	GG	GG
	GG	GG	GG	GG	GG	GG	GG	GG
	GG	GG	GG	GG	GG	GG	GG	GG
	GG	GG	GG	GG	GG	GG	GG	GG

GG GG GG GG GG GG GG
110875113
8530 rs10246006 7 AG GG AG AG AG
GG AG GG GG GG AA AA AA
AA AG AA AG AG GG GG AG
AA AA AA AA AA AG AG AG
AG AG AA AA AA AA AA
115757442
8531 rs1024603 14 AG GG GG GG AG
AG GG GG GG GG AG AG GG
GG GG GG GG AG GG GG GG
AG GG AG GG AA GG AG AG
AG GG GG GG GG GG GG
74295380
8532 rs10246082 7 AA AA AA AA AA
AA AA AC AC AC AA AC AC
AC AA AA AA AA AA AA AA
CC CC AC AC AA AA AA AA
AA AA AA AA AA AA AC
80048871
8533 rs10246111 7 GG GG GG GG GG
GG GG GG GG AG GG GG GG
GG AG GG GG GG GG AG GG
GG GG GG GG GG AG AG AG
AG GG AG GG GG GG GG
77747902
8534 rs10246118 7 AA AA AA AA AA
AA AA AC AC AA AA AA AA
AA AA AA AA AA AA AA AA
AA AA AC AA AA AA AA AA
AC AA AA AA AA AA AC
97298855
8535 rs10246131 7 GG GG GG AG GG
AG GG AG GG AG GG GG GG
GG GG AG AG AG GG GG GG
GG GG GG GG GG AG GG GG
AA GG AG AG GG GG AG
137972605
8536 rs10246132 7 AA AC AC AA AA
AA AA AA AA AC CC CC CC
AA AA AA AA AA AC AC AA
AA AC AA AC AA AA AA AA
AA AA AA AA AA AA AA
36148652
8537 rs10246139 7 AG AG AG AG AA
AA AG AG AG GG AA AG AG
AG AG GG GG GG AG AG AA
GG AA AG AG AA AG AG AG
GG AG AG GG AA AG AA
43541932

8538 rs10246149 7 GG GG GG GG GG
GG GG GG GG GG GG GG GG
GG GG GG GG GG GG GG GG
GG GG GG GG GG GG GG GG
GG GG GG GG GG GG GG
149524665
8539 rs10246169 7 AG AG AA AG GG
AG GG GG AG AG GG GG GG
GG GG GG AG GG GG AG GG
AG GG AG GG GG GG GG GG
GG AG AG AA GG AG GG
40910751
8540 rs1024618 5 AG AG AA AG AG
AA AG GG AA GG GG GG AG
AG AG GG GG GG AG GG GG
GG GG AG GG AG AG AG GG
AG AG GG GG AG AA GG
11054511
8541 rs10246200 7 AG AG GG AG GG
AG GG AA AA AG AG AA AG
GG GG AA AA AG AG AG GG
AA AA AA AA AG AG AG AG
AA AG AG AG AG AA GG
869846
8542 rs1024625 19 AA AA AA AA AA
AA AA AA AA AA AA AA AA
AA AA AG GG AG AA AG AA
AG AA AG AG AG AG AA AG
AG AA AA AA AG GG AG
1707526
8543 rs10246267 7 AA AA AA AA AA
AA AA AA AA AA AA AA AA
AA AA AA AG AG AG AA AA
AA AA AA AA GG AA AG AG
AA AA AA AA AA AA AA
103601185
8544 rs10246310 7 AC CC AC AC CC
AC CC CC CC CC CC CC CC
CC CC CC CC CC AC AC CC
AC AC CC AC CC AC AC AC
AC CC CC CC AC AA CC
101693308
8545 rs1024639 17 GG GG GG AG AG
AA GG GG GG GG GG GG GG
AA AG GG AG GG AG AG AG
AG AA AG AG GG AG AG GG
GG AG GG GG AG GG AG
15323365
8546 rs10246420 7 CC CC CC CC CC
CC CC CC CC CC CC CC CC

CC CC CC CC CC CC CC CC
CC CC CC CC CC CC CC CC
CC CC CC CC CC CC CC
55107369
8547 rs1024647 16 GG GG GG GG GG
GG GG GG GG GG GG AG AG
AG GG GG GG GG GG GG GG
AG GG GG AG GG GG GG GG
GG GG GG GG GG GG GG
7792248
8548 rs10246481 7 GG GG GG AG AG
AG AG AG GG AA AG AG AA
AA AG AA AA AA AA AA AA
AA AG AA AA GG AG AG AG
GG AG AA AG AA AG AG
156184748
8549 rs1024651 16 AC AC AC AA AC
AA AC AC CC AC AA AA AA
AA AC AA AA AA AC AC AC
AA CC CC AA AC AA AC AC
AC AC AA AA AA AA AA
7706864
8550 rs10246565 7 CC CC CC CC CC
CC CC CC CC CC CC CC CC
CC CC CC CC CC CC CC CC
CC CC CC CC CC CC CC CC
CC CC CC CC CC CC CC
41235998
8551 rs10246586 7 AA AG AA AA AA
AA AA AA AA AA GG AG AG
AG AG AA AA AA AA AA AA
AA AA AA AA AA AG AG AG
AA AA AA AA AA AA AA
36715569
8552 rs10246618 7 AA AA AA GG AG
GG AG AG AG GG GG AG AG
AG AG AG AA AA AG GG AG
AG GG AG AA AG GG AG GG
GG AG GG GG AG AG GG
53283134
8553 rs10246620 7 GG GG GG GG GG
GG GG GG GG GG GG GG GG
GG GG AG AG AG GG GG AG
AG GG AG GG GG AG GG GG
GG GG AG AG AG GG AA
43424112
8554 rs10246631 7 AA AG AA AA AG
AG AA GG AG GG AG GG AG
AG GG AA AA AA AA AG AA
AG GG GG AG AG AA AG AA

	AA	AG	AA	AA	AG	AG	AA	
69804574								
8555	rs10246654		7	GG	AG	GG	GG	GG
	GG	GG	GG	GG	GG	GG	GG	GG
	GG	GG	AG	AG	AG	AG	AG	GG
	GG	AG	GG	AG	GG	GG	GG	GG
	GG	AG	GG	GG	GG	GG	GG	
6505901								
8556	rs10246702		7	AA	AA	AA	AA	AA
	AA	AA	AA	AA	AA	AA	AA	AA
	AA	AA	AA	AA	AA	AA	AA	AA
	AA	AA	AA	AA	AA	AG	AA	AA
	AG	AA	AG	AG	AG	AA	AG	
154966534								
8557	rs10246767		7	AG	GG	AG	GG	AG
	AG	GG	GG	AG	GG	GG	GG	GG
	AG	AG	AG	AG	AG	AG	AG	GG
	GG	AG	AG	GG	AG	AG	GG	AG
	AA	GG	AG	GG	GG	AG	GG	
21267438								
8558	rs10246773		7	AA	AA	AA	AA	AA
	AA	AA	AA	AA	AA	AA	AA	AA
	AA	AA	AA	AA	AA	AG	AA	AA
	AA	AA	AA	AA	AA	AA	AA	AA
	AA	AG	AA	AG	AA	AA	AA	
148460536								
8559	rs10246779		7	AA	AA	AA	AA	AA
	AA	AA	AG	AA	AA	AA	AA	AA
	AA	AA	AA	AA	AA	AG	AA	AA
	AA	AA	AA	AA	AA	AA	AA	AA
	AA	AA	AA	AA	AA	AA	AA	
153624869								
8560	rs10246872		7	AA	AA	AA	AA	AA
	AA	AA	AG	AA	AA	AA	AA	AG
	AA	AA	AA	AA	AG	AA	AA	AA
	AA	AA	AA	AA	AA	AA	AA	AA
	AA	AA	AA	AA	AA	AA	AA	
33701425								
8561	rs10246939		7	AG	AG	AG	GG	AG
	AG	GG	AG	GG	AG	GG	AG	AG
	AA	AG	AA	AA	AA	AA	AG	AG
	GG	GG	GG	GG	AG	AA	AG	AG
	AG	AG	AG	GG	AA	AA	AA	
141672604								
8562	rs10246957		7	GG	AG	AG	AG	AG
	AA	AG	AG	AA	AG	AA	AA	AA
	AG	AG	AG	AG	AG	AG	AA	AG
	AG	AA	AG	AG	AA	AA	AA	AA
	AA	GG	GG	GG	AA	AA	AA	
155450991								

8563 rs1024696 16 GG GG GG GG GG
GG GG GG GG GG GG GG GG
GG AG GG AG GG GG AG GG
GG GG GG GG GG GG GG GG
GG GG AA AG GG GG AG
7587513
8564 rs10247014 7 AA AC CC AC AA
AC AC AC CC AC CC CC CC
CC CC CC AC AC AC CC AC
AC AC AC AC CC AA AC AC
CC CC AC CC AC AC AC
133431038
8565 rs10247026 7 AG AG AG AG AG
AG GG GG AG AG GG AG AG
AG GG AG AA AA GG AG GG
AA AG AA AA GG AG AG AG
AG GG AG AG GG GG GG
158230595
8566 rs10247053 7 AG GG GG GG AG
GG AG GG GG GG GG GG GG
AG AG GG GG GG GG AG GG
GG GG GG GG AA GG AG AG
GG AG AG AA AG AA GG
26637362
8567 rs10247107 7 AG GG GG GG GG
GG AG GG AG GG GG GG GG
AG AA AA AG AG GG GG GG
AG AG AG AG GG AG GG GG
GG GG AA AG AG AG GG
150683083
8568 rs10247159 7 AA AA AA AA AA
AA AA AA AA AA AA AA AA
AA AA AA AA AA AA AA AA
AA AA AA AA AA AA AA AA
AA AA AA AA AA AA AA
155510917
8569 rs1024716 7 AG GG AG GG GG
GG GG GG AG GG GG GG GG
GG GG GG GG GG AG GG AA
AG AG AG AG AG GG AG AG
GG GG GG GG AG AG GG
157213644
8570 rs1024723 7 GG GG GG GG GG
GG AG AA AA AG AG AG GG
AG AG AG GG GG AG GG AA
GG AG AA AG AG AG GG AG
GG AG AG AG GG AG GG
77236148
8571 rs10247269
7 -- -- -- -- -- -- -- --

	--	--	--	--	--	--	--	--
	--	--	--	--	--	--	--	--
	--	--	--	--	--	--	--	--
	--	--	--	--	76288272			
8572	rs10247300		7	AC	AC	CC	AA	CC
	AC	AC	AC	AC	CC	CC	CC	CC
	CC	CC	AC	AC	AA	CC	AC	CC
	CC	CC	CC	CC	CC	CC	CC	CC
	CC	AC	AC	CC	AC	CC	AA	
40344782								
8573	rs10247302		7	AG	GG	GG	GG	GG
	GG	GG	AG	GG	AG	GG	GG	GG
	GG	GG	GG	AG	AG	GG	GG	GG
	AG	AG	AG	AG	AA	GG	AG	AG
	AA	GG	GG	GG	GG	GG	AG	
155259640								
8574	rs1024732		22	AA	AA	AA	GG	GG
	GG	GG	AG	AG	GG	AG	GG	AG
	GG	AG	AG	AG	AG	AG	AG	AG
	AA	AA	AA	AG	GG	AA	AG	AG
	GG	GG	AG	GG	AG	GG	AG	
17443381								
8575	rs10247327		7	AA	AA	AA	AG	AG
	AA	AG	AA	AA	AA	AA	AA	AA
	AA	AA	AA	AA	AA	AG	GG	AA
	AA	AA	AA	AA	AA	AA	AA	AA
	AA	AA	AA	AA	AA	AG	AA	
76911470								
8576	rs10247424		7	CC	CC	CC	CC	CC
	CC	CC	CC	CC	CC	CC	CC	CC
	CC	CC	CC	CC	CC	CC	CC	CC
	CC	CC	CC	CC	CC	CC	CC	CC
	CC	CC	CC	CC	CC	CC	CC	
6357407								
8577	rs10247482		7	GG	GG	GG	GG	GG
	GG	GG	GG	GG	GG	GG	GG	GG
	AG	AG	GG	GG	GG	GG	AG	GG
	GG	GG	GG	GG	GG	GG	GG	GG
	GG	GG	AG	GG	AG	AG	GG	
16826463								
8578	rs10247487		7	GG	AG	AG	AG	GG
	GG	AG	AG	AG	AG	AG	GG	AG
	AG	GG	AG	AA	AA	AG	AG	AG
	AG	AG	AG	AG	AG	AG	AA	AA
	AG	AG	AG	AA	AG	AA	AG	
107420354								
8579	rs10247526		7	AC	CC	CC	CC	CC
	CC	CC	CC	CC	CC	AC	CC	AC
	AC	AC	CC	CC	CC	CC	CC	CC
	CC	CC	CC	CC	CC	CC	CC	CC

CC AC CC CC AC CC AC
65780696
8580 rs10247570 7 AA AC AC CC CC
CC CC CC CC AC AA AC AC
CC CC AC AC CC CC CC AC
CC AC AC CC AC CC AC CC
AC AC AC AC CC AC CC
49650978
8581 rs10247576 7 CC CC CC CC CC
CC CC CC CC CC CC CC CC
CC CC CC CC CC CC CC CC
CC CC CC CC CC CC CC CC
CC CC CC CC CC CC CC
114289257
8582 rs10247578 7 GG AG GG AA GG
AG AG GG GG GG AG GG GG
AG AG GG AG GG GG GG GG
GG GG GG GG AA GG AG AG
GG AG GG AG AG AG GG
8959373
8583 rs10247580 7 GG GG GG GG GG
GG GG GG GG GG GG GG GG
GG GG GG GG GG GG GG GG
GG GG GG GG GG GG GG GG
GG GG GG GG GG GG AG
99258524
8584 rs1024761 7 GG GG GG AG AG
AG AG AG AG AG GG GG GG
GG GG AA AA AA GG GG AG
AA AG AG AA AG GG AG AG
AG AG GG GG AA AA AA
106088278
8585 rs10247618 7 GG GG AG GG GG
GG GG GG GG GG GG GG GG
AG AG AA AG AA GG AG GG
GG AG AG GG GG AA AG AG
GG AG AG AA GG AG AG
64109625
8586 rs10247670 7 AC CC AC AC AC
CC AA AC AC AC CC CC CC
AC AC CC AC AC AC AC AC
AA AC AC AA CC CC CC CC
AC AA CC AC AC CC AA
21228873
8587 rs1024774 14 AG GG GG GG AG
AG GG AG AG AG GG GG GG
GG GG GG GG GG GG GG AG
GG GG GG GG AG GG GG GG
GG GG GG GG AG AA AG
75998984

8588 rs10247745 7 AC AC AA AA AA
AA AA AA AA AA AC AA AC
AC AC AA AA AA AA AC AA
AA AC AA AA AA AA AA AA
AA AC AA AC AC AA AC
136312729
8589 rs10247772
7 -- -- -- -- -- -- -- --
-- -- -- -- -- -- -- --
-- -- -- -- -- -- -- --
-- -- -- -- -- -- -- --
-- -- -- -- 16633321
8590 rs1024782 19 GG GG GG AG AG
AA GG AG GG GG AG AG GG
GG GG GG AG GG AG GG AG
GG AA AG AG AA GG AG AG
GG AA GG AG GG GG GG
46887567
8591 rs10247832 7 GG GG GG GG GG
GG GG GG GG GG GG GG GG
GG GG GG GG GG GG GG GG
GG GG GG GG GG GG GG GG
GG GG GG GG GG GG GG
64086228
8592 rs10247918 7 AG AG GG AG GG
GG AG AG AG AG AA AA AG
AA AA GG GG GG GG GG AG
AA AA AA AA GG AG GG GG
GG AA GG AG GG GG GG
31854395
8593 rs10247946 7 AA AA AA AA AA
AA AA AA AA AA AA AA AA
AA AA AA AA AA AA AA AA
AA AA AA AA AA AA AA AA
AA AA AA AA AA AA AA
98935333
8594 rs10247961 7 GG GG GG GG GG
GG GG GG GG GG GG GG GG
GG GG GG GG GG GG GG AG
GG AG AG GG AG GG AG GG
GG GG AG AG AG GG AG
75309746
8595 rs10247980 7 AG AA AG AA GG
AG AG AA AA AA AG AG AA
AG AA GG AG AG GG AA AG
AG AA AA AG GG AA AG AG
GG AG AG AG AG AG AG
100366284
8596 rs10248011 7 AG AA AG GG GG
GG GG GG GG GG AA AA AA

	AA	AG	AG	AA	AA	AG	GG	AG
	AA	GG	AG	AA	GG	GG	GG	GG
	AG	AA	AG	AA	AG	AA	AG	
120778814								
8597	rs10248034		7	AG	AG	AG	AA	AA
	AA	AA	AA	AA	AG	AA	AA	AA
	AA	AA	AA	AA	AG	AG	GG	AA
	AG	AG	AG	AG	AA	AA	AA	AA
	AA	GG	AG	GG	AA	AG	AA	
12053230								
8598	rs10248040		7	AG	AG	GG	AG	AG
	AG	AG	AA	AG	AG	GG	AG	AG
	AA	AA	AG	AG	AG	AG	AG	AG
	AG	AG	AG	AG	AA	AA	AA	AA
	AG	AG	AG	AG	AA	AG	AA	
48354820								
8599	rs10248048		7	AA	AC	AA	CC	CC
	CC	CC	CC	CC	CC	AA	AA	AA
	CC	CC	AC	AC	CC	AA	CC	AA
	AA	AA	AA	AA	CC	AC	AC	AC
	AA	CC	AA	AC	AC	AA	AC	
53103267								
8600	rs10248055		7	AA	AA	AA	AA	AA
	AA	AA	AA	AA	AA	AA	AA	AA
	AA	AA	AA	AA	AA	AA	AA	AG
	AA	AA	AA	AA	AA	AA	AA	AA
	AG	AA	AA	AA	AA	AA	AA	
92201473								
8601	rs10248075		7	AA	AA	AA	AA	AA
	AA	AA	AC	AC	AC	AA	AA	AA
	AA	AA	AC	AC	AC	AC	AC	AA
	AA	AA	AA	AA	AA	AA	AA	AA
	AA	AA	AC	AC	AA	AC	AA	
85165289								
8602	rs1024809		2	AG	AA	AG	AA	AA
	AA	AA	GG	AA	AA	AG	AG	AG
	AG	AG	AG	AG	AG	AA	AG	AG
	AA	AA	AA	AG	AG	AA	AG	AG
	AA	AA	AG	AA	AG	AG	AG	
207105300								
8603	rs10248136		7	AA	AA	AG	GG	AA
	AG	AG	AG	AA	AG	AA	AG	AG
	AG	AG	AG	AA	AA	AG	AG	GG
	GG	GG	GG	GG	AA	AG	AA	AA
	AG	AG	AA	AA	GG	AG	AG	
39077397								
8604	rs10248172		7	GG	GG	GG	GG	GG
	GG	GG	GG	GG	GG	GG	GG	GG
	GG	GG	GG	GG	GG	GG	GG	GG
	GG	GG	GG	GG	GG	GG	GG	GG

GG GG GG GG GG GG GG
41672931
8605 rs10248196 7 GG GG AG GG AG
AG GG GG GG GG AG AG GG
GG AG AG AG AG GG GG GG
GG GG GG GG GG GG GG GG
GG GG GG GG GG GG GG
63426198
8606 rs10248211 7 GG GG GG AG AA
AA AG AA AG AA AG GG AG
AG GG AG AG AG AG AA AG
AG AA AG AA GG GG -- GG
AG GG AG GG AG AG AG
20961711
8607 rs10248220 7 AG AG AG AA AA
AA AA AA AA AA AA AA AA
AA AA AA AG AG AA AA AA
AA AG AA AA AA AA AA AA
AA AA AG AA GG AG GG
146985808
8608 rs10248254 7 GG GG GG GG GG
GG GG AG AG GG GG GG AG
AG AG GG GG GG GG GG GG
GG GG GG GG GG GG GG GG
GG AG GG AG GG GG AG
88550492
8609 rs1024826 17 AA AA AA AA AA
AA AA AA AA AA AA AA AA
AA AA AA AA AA AA AA AA
AA AA AA AA AA AA AA AA
AA AA AG AA AA AA AA
70147581
8610 rs10248323 7 AG AA AG AA AG
AG AA AG GG GG AA AA AA
AG AA AA AA AG AA AA AG
AA AA AA AA AG AG AA AA
AA AA AA AA AG AA AG
5198328
8611 rs10248341 7 CC CC CC CC CC
CC CC CC CC CC CC CC CC
CC CC CC CC CC AC CC AC
CC CC CC CC CC AC AC AC
CC CC CC CC CC AC CC
52995247
8612 rs10248355 7 AG AG GG AA AA
AA AA AG AA AG GG GG GG
AG AG AG GG GG GG AA AG
GG AG AG GG AG AG GG AG
AG AG AG GG GG GG GG
52575811

8613 rs10248368 7 AA AA AA AA AA
AA AA AA AA AA AG AG AA
AG AG AA AG AG AA AA AA
AA AA AA AA AA GG AG AG
AA AA AA AA AA AA AA
141819278
8614 rs1024839 12 AA AG AG AG AG
AA AG AA AG AG AG AG AG
AG AG GG AG AG AA AA AG
AG AG AG AG AA AA AA AA
AG AA AA AA GG GG GG
16519385
8615 rs10248396 7 AG GG AG AA AA
AA AA AA AA AA AA AA AA
AG GG AG AA AA AA AA AA
AA AG AG AG AG AA AA AA
AA AG AG AA AA AA AA
8797698
8616 rs10248417
7 -- -- -- -- -- -- -- --
-- -- -- -- -- -- -- --
-- -- -- -- -- -- -- --
-- -- -- -- -- -- -- --
-- -- -- -- 15270751
8617 rs10248441 7 AA AA AA AG AA
AA AG AA AA AA AG AG AG
AG AA AA AA AA AA AA AA
AA AG AG AA AA AG AA AA
AG AG AA AG AG AA AG
88416536
8618 rs1024852 3 AA AA AA AA AA
AA AA AG AA AA AG AG GG
AG AA AA AA AA AA AA AA
AA AA AA AA AA AA AA AA
AA AG AG AG AG AG AA
179482482
8619 rs10248553 7 GG GG GG GG GG
GG GG GG GG GG AG GG AG
GG GG AG AG AG GG GG GG
GG AA GG GG GG AG AG AG
AG GG GG GG GG GG GG
150317472
8620 rs10248576 7 AG AG AG AA AG
AA AG GG AG AG AA AG AG
AA AA AA AA AA AG AA AG
AA AG AG AA AA AG AG AG
AA AG AG AA GG AG AG
52965371
8621 rs10248609 7 GG GG GG AG GG
GG AG GG AG GG GG GG GG

GG GG GG GG GG AG GG AG
GG GG GG GG AG GG AG GG
GG GG AG AG GG GG GG
56262054
8622 rs10248618
7 -- -- -- -- -- -- -- --
-- -- -- -- -- -- -- --
-- -- -- -- -- -- -- --
-- -- -- -- -- -- -- --
-- -- -- -- 21828080
8623 rs10248665 7 AG AG AA AA AA
AA AG AA AA AA AG GG AG
AG AA GG AG AG AG AA GG
AG AA AG AG AA AA AA AA
GG AG AG GG AG AG AG
126932944
8624 rs10248857 7 AA AA AA AA AA
AA AA AA AA AA AA AA AA
AA AA AG AG AG AA AA AA
AA AG AA AA AA AA AA AA
AA AA AA AA AG AG AA
152016244
8625 rs10248875 7 AG AG GG AG AG
GG AG GG AG GG AG AA AG
AG AG GG GG GG AG GG AG
AG AG AG AG GG AG GG GG
AA AG AA AA AG AA GG
152220077
8626 rs10248887 7 GG AG GG AA AG
AA AG AG AG AG GG GG GG
GG GG AA AA AA GG GG AG
GG GG GG GG GG GG GG GG
GG AA AA AA GG GG GG
23711767
8627 rs10248899 7 CC CC CC CC CC
CC AC CC CC CC CC AC AC
AC CC CC AC AC CC AC CC
CC CC CC CC CC CC CC CC
CC CC CC CC CC CC CC
146039066
8628 rs10248914 7 GG AG AG AA AA
AA AA AA AA AA AA AA AA
AA AA AG AA AA AA AA AA
AG AA AA AG AA AA AA AA
AA AA AA AA AG AG AA
43935301
8629 rs10248924 7 AG AG GG AG AG
GG AG GG GG GG GG AG AG
AG GG GG GG GG GG GG GG
AA GG AA AA AG GG AG AG

GG GG AG AG GG GG GG
138831707
8630 rs10248934 7 AA AA AA AA AA
AA AA AA AA AA AG AG AA
AA AA AA AA AA AA AA AA
AA AG AG AA AA AA AA AA
AA AG AA AA AA AA AA
93826432
8631 rs10248945 7 GG GG GG GG GG
GG GG GG GG GG GG GG GG
GG GG GG GG GG GG GG GG
GG GG GG GG GG GG GG GG
GG GG GG GG GG GG GG
97076007
8632 rs10248996 7 GG GG GG GG GG
GG GG GG GG GG GG GG GG
GG GG GG GG GG GG GG GG
GG GG GG GG GG GG GG GG
GG GG GG GG GG GG GG
75941940
8633 rs10249014 7 AG AG AG AA AG
AA AG AG AG AG AA AA AA
AA AA AA AG AG AA AG AA
AG AA AG AA AG GG GG AG
AG AA AA AA GG GG AG
21556942
8634 rs10249071 7 GG GG GG GG GG
GG GG GG GG AG GG GG GG
GG GG AG GG GG GG AA GG
GG GG GG GG AG GG AG GG
GG GG AG AG GG GG GG
91257948
8635 rs1024908 18 -- AG GG GG GG
GG GG AA AA AG AG GG GG
AG AA AG AG AG AG AG GG
GG GG AG GG GG GG GG GG
AG GG GG GG AG AA AG
71907776
8636 rs1024909 1 AA AA AA AA AG
AA AG AA AA AA AA AA AA
AA AA AA AG AG AG AG AG
AG AA GG AG AG AA AA AG
AG AG AA AA AG AA AG
42571439
8637 rs1024913 2 AC AC AC AA AA
AA AA AC CC AC AC CC CC
AC AC AA AC AC AA AA AA
AA AA AC AA CC AC AC AC
CC AA CC AC AC AC AA
16960303

8638 rs10249138 7 GG GG GG GG GG
GG GG GG GG AG GG GG GG
GG GG AG GG GG GG GG GG
GG GG GG GG AG GG GG AG
GG GG GG GG GG GG AG
51730792
8639 rs10249139 7 AA AA AA AA AA
AA AA AA AA AA AA AA AA
AA AA AA AA AA AA AA AA
AA AA AA AA AA AA AA AA
AA AA AA AA AA AA AA
1271819
8640 rs10249143 7 AA AA AA AG AA
AG AA AA AA AA AG AG AA
AA AA AA AA AA AG AA GG
AG AA AG AG AG AA AA AA
GG AG AG AA AG AG AG
122691242
8641 rs10249179 7 AA AA AA AA AA
AA AA AA AA AA AA AA AA
AA AA AA AA AA AA AA AA
AA AA AA AA AA AA AA AA
AA AA AA AA AA AA AA
148922228
8642 rs10249282 7 AA AA AA AA AA
AA AG AA AA AA AG AG AA
AA AA AA AA AA AG AG AA
AG AA AA AG AA AA AA AA
AA AA AA AA AA AA AG
117411796
8643 rs10249283 7 AA AA AA AA AA
AA AG AA AA AG AA AG AG
AG AA AG GG GG AA AA AA
AA AA AA AA AG AA AA AA
AA AA AA AA AA AA AA
155648007
8644 rs10249292 7 AA AA AA AA AA
AA AA AA AA AA AA AA AA
AA AA AA AA AA AA AA AA
AA AA AA AA AA AA AA AA
AA AA AA AA AA AA AA
155073487
8645 rs10249318 7 AA AG AA AG AG
AG GG AG AG AG AG GG AG
AG AG AG AA AA AA AG AA
AA AG AA AA AG AG AA AA
AA AA AG AA AA AA AA
159084383
8646 rs10249436 7 CC CC CC CC CC
CC CC CC AC CC CC CC CC

AC AC CC AC AC AC AC AC
CC CC CC CC CC CC CC CC
CC CC CC CC CC CC CC
146266525
8647 rs10249437 7 GG GG GG GG GG
GG GG GG GG GG GG GG GG
GG GG GG GG GG GG GG GG
GG GG GG GG GG GG GG GG
GG GG GG GG GG GG GG
154116796
8648 rs10249489 7 AC CC CC CC CC
CC CC AC AC CC AA AA AA
AC AC CC AC AC AC CC AA
AC AA AA AC AC AA AA AC
AC AC AC CC AC AA CC
52722258
8649 rs10249528 7 AA AA AC CC AA
AC AC CC AC AC AC AA AC
AA AA AA AA AC AA AA AC
AA AA AC AC AC AC AA AC
AA AA AA AA AC AA AC
56572536
8650 rs10249530 7 AG GG AG AG AG
GG AA AA AA AA AG AG AG
AA AG GG GG GG AG AA AG
AA AA AA AA AG AA AA AG
AA AG AA AG AG AG AG
90992321
8651 rs10249537 7 AA AA AA AA AA
AA AG GG AG GG AA AA AA
AA AA AG AA AA AA AG AA
AA AG AA AA AA AG AA AA
AA AG AA AG GG AG AG
157792493
8652 rs10249588 7 GG GG GG GG GG
GG GG GG GG GG GG GG GG
GG GG GG GG GG GG GG GG
GG GG GG GG GG GG GG GG
GG GG GG GG GG GG GG
15775095
8653 rs10249596 7 GG GG GG AG AG
GG AG GG GG GG GG GG GG
AG AG GG GG GG AG GG AG
GG GG GG GG AG GG GG AG
GG AG AG AG AA AG AA
66962579
8654 rs10249757 7 GG GG GG GG GG
GG GG GG GG GG AG AG GG
GG GG GG GG GG AG AG GG
AG GG GG AG GG AG GG GG

GG GG GG GG GG GG GG
27482830
8655 rs10249759 7 AC AC AC CC AC
AC CC AA AC AC AC AC CC
AC CC AA AA AC AA AA AC
AC AC AC AC CC AC CC CC
CC AA CC AC CC CC CC
6065746
8656 rs10249802 7 CC CC CC CC CC
CC CC CC CC CC CC CC CC
CC CC CC CC CC CC CC CC
CC CC CC CC CC CC CC CC
CC CC AC CC CC CC CC
50926949
8657 rs10249826 7 GG GG GG AG AG
GG AA GG AG GG GG GG GG
GG GG GG GG AG AG AG GG
GG GG GG GG GG AG GG GG
GG GG GG GG GG GG GG
20240680
8658 rs1024985 5 GG GG GG GG GG
GG GG GG GG AG AG GG AG
AG AG GG GG GG AG GG GG
AG AG AG AG AG GG GG GG
AG AG AA AG AA AG AA
76943223
8659 rs10249854 7 AG AG GG AA AG
AA AG AG AG GG AG AG AA
AG AG AG AG AG AG AG AG
AA AG GG AA GG AG AG AG
AG AG AG AA GG GG AG
97035760
8660 rs10249855 7 AG AA AA AG AG
GG AG AG AA AG AA AA AA
AA AA AG AA AA AG AA AG
GG GG GG GG AG GG AG AG
GG AG AA AA AG AG AG
147698173
8661 rs10249869 7 AG AG GG GG GG
GG GG AA AA AA GG AG AG
GG AA AG AA AA AA GG AG
AG AG AG AG GG GG GG GG
GG GG GG GG GG GG GG
104335994
8662 rs10249881 7 AG AA AA AG AG
AA AG GG GG GG AA AG AG
AG AA AA AA AA AG GG GG
AG AG AG AG AA AG AA AA
AG AG GG AG GG AG AG
143045402

8663 rs10249912 7 GG GG GG GG GG
GG AG AG GG AG GG GG GG
GG GG GG AG AG AA AG AG
AA AG AA AA AG GG AG AG
AG GG GG GG GG AG GG
133738113
8664 rs10249920 7 GG GG GG GG AG
AG GG AA AA AA AA AA AA
AA AA AA AA AA AG AG AA
AG AA -- AG AG AG GG GG
AA AG AG AG AG GG AA
114778640
8665 rs10249951 7 -- GG GG AA AG
AG AA GG GG GG GG GG GG
GG -- AG GG -- GG GG AG
-- AA AG AA GG GG -- GG
AG AA GG AG GG GG --
5598053
8666 rs10250025 7 AA AA AA AA AA
AA AA AA AA AA AA AA AA
AG AG AA AA AA AA AA AA
AA AA AA AA AA AA AA AA
AA AA AG AA AA AA AA
157611949
8667 rs10250062 7 AG GG GG GG GG
GG AG AG AG AG GG GG GG
GG GG GG GG GG AG GG AG
GG GG AG AG GG GG GG GG
AA GG AA AG GG GG AG
68042545
8668 rs10250225 7 GG GG GG GG GG
GG GG GG GG GG GG GG GG
GG GG GG GG GG GG GG GG
GG GG GG GG GG GG GG GG
GG GG GG GG GG GG GG
55266227
8669 rs10250274 7 AA AA AA AA AA
AA AA AA AA AA AA AA AA
AA AA AA AA AA AA AA AA
AA AA AA AA AA AA AA AA
AA AA AA AA AA AA AA
96278518
8670 rs10250332 7 AG GG AG AG AA
AA AG AA AA AA AG AA AG
AG AA AA AA AA AA AA AG
AA AA AA AA AG AA AG AG
AG AG AA AG AA AA AA
8992884
8671 rs10250333 7 AA AA AA AA AG
AG AA AA AA AA AG AG AA

	AA	AA	AA	AA	AA	AG	AA	GG
	AA	AG	AG	AA	AG	AG	GG	AG
	AG	AA	AA	AA	AA	AG	AA	
27762622								
8672	rs10250368		7	AA	AA	AA	AG	AG
	AG	AG	AA	AA	AA	AA	AA	AG
	AA	AA	AA	AA	AA	AA	AG	AA
	AG	AA	AA	AG	AA	GG	AG	AG
	AG	AG	AG	AG	AA	AA	AA	
30842782								
8673	rs10250376		7	AA	AA	AA	AA	AA
	AA	AA	AA	AA	AA	AA	AA	AA
	AC	AC	AA	AA	AA	AA	AA	AA
	AA	AA	AC	AC	AA	AA	AA	AA
	AA	AA	AA	AA	AC	CC	AA	
49422750								
8674	rs10250461		7	GG	GG	GG	AG	GG
	AG	GG	GG	GG	GG	AA	AG	AG
	AG	GG	GG	GG	GG	GG	AG	GG
	GG	AG	GG	AG	GG	GG	GG	GG
	AG	GG	GG	GG	GG	GG	GG	
22289364								
8675	rs10250463		7	AG	GG	AG	AG	GG
	AG	GG	GG	GG	GG	GG	AG	AG
	AG	GG	GG	GG	GG	GG	GG	GG
	GG	GG	GG	GG	AG	GG	GG	GG
	GG	GG	GG	GG	GG	GG	GG	
127693537								
8676	rs10250522		7	AC	AC	CC	CC	CC
	CC	CC	CC	CC	CC	CC	CC	CC
	CC	CC	CC	CC	CC	CC	CC	CC
	AA	AC	CC	AC	CC	CC	CC	CC
	CC	CC	CC	CC	CC	AC	CC	
81723042								
8677	rs1025053		2	AG	AG	AG	AA	AA
	AA	AG	AG	AG	AG	GG	AG	GG
	GG	GG	AG	AG	GG	GG	AG	AG
	AG	AA	AG	AG	AG	GG	GG	GG
	AA	AG	AG	GG	GG	AG	GG	
8216404								
8678	rs10250544							
7	--	--	--	--	--	--	--	--
	--	--	--	--	--	--	--	--
	--	--	--	--	--	--	--	--
	--	--	--	--	--	--	--	--
	--	--	--	--	65766561			
8679	rs10250570		7	AG	GG	AG	GG	GG
	AG	AG	AG	AG	AG	AG	GG	AG
	AG	AG	GG	GG	GG	AA	AA	AG
	AG	AG	AG	AG	AG	AG	GG	GG

AG AA AG AA GG GG GG
147073363
8680 rs10250599 7 AG AG AG GG GG
GG AG AG AA AG AG AG AA
AG AG AA AA AA AG AG AA
AA AA AA AA AA AA AA AA
GG AA AA AA AA AA AA
150564361
8681 rs10250659 7 CC CC CC AC CC
AC CC AC AC AC AA AC AC
AC CC CC CC CC AC AC CC
AC CC CC CC CC AC AC AC
AC CC AC AC CC CC AC
3952043
8682 rs10250685 7 AA AA AA AA AG
AG AA AA AA AA AA AA AA
AA AA AG AG AG AA AA AA
AG AA AA AG AA AG AA AA
AG AG AA AG AA AA AA
100873835
8683 rs10250694 7 GG GG GG GG AG
AG GG GG GG GG GG GG GG
GG GG GG GG GG GG GG GG
GG GG GG GG GG GG GG GG
GG GG GG GG GG GG GG
57227638
8684 rs10250712 7 AA AA AA AA AG
AG AA AA AA AA AA AA AA
AA AA AA AA AA AA AA AA
AA AA AA AA AA AA AA AA
AA AA AA AA AA AA AA
51953172
8685 rs10250721 7 AA AA AA AG AA
AG AA AA AA AA AA AA AA
AA AA AA AA AA AA AA AA
AA AG AA AA AA AA AA AA
AA AA AA AA AA AA AA
130887794
8686 rs10250756 7 AA AA AA AA AA
AA AA AA AA AA AA AG AA
AG AA AA AG AA GG GG AG
AG AA AG AA AA AA AA AA
AA AA AG AA AA AG AA
7528385
8687 rs10250763 7 CC CC AC CC CC
CC CC CC CC CC CC CC CC
CC CC CC AC CC AA CC AC
CC CC CC CC CC CC CC CC
CC CC CC CC AC CC AA
52622294

8688 rs1025077 2 AG AG AG AG AG
AA AG AG AA AA AG AG AG
AG AA GG GG GG GG GG GG
AG AG AA AG AG AA AG AG
GG AG GG AG GG GG GG
207373828
8689 rs1025082 2 AA AA AA GG AG
GG AG AA AA AA GG AA AG
AG AG AG AG AG AA AA AA
AG AG AG AG AG GG GG GG
AG GG AA AG AG GG AA
222882027
8690 rs10250825 7 GG GG GG GG GG
GG GG GG GG GG GG GG GG
GG GG GG GG GG GG GG GG
GG GG GG GG GG GG GG GG
GG GG GG GG GG GG AG
102943847
8691 rs10250876 7 AG GG GG AG AG
GG AA AG AG AG GG GG GG
AG AG GG GG GG GG GG GG
GG AG GG AG AG GG GG GG
AA AG AA AG GG GG AG
34497585
8692 rs10250905 7 AG AG AA GG GG
GG GG GG GG AG GG AG AG
GG GG AG AG GG AG AG GG
GG AG GG AG AG AA AA AA
GG GG GG GG GG GG GG
37907304
8693 rs10250993 7 AA AG AG AA AA
AA AA GG GG GG AA AA AA
AA AG GG AG AG AA AA AA
AA AA AA AA AG AG GG GG
AA AA AG AG AG AA AG
127077326
8694 rs10251001
7 -- -- -- -- -- -- -- --
-- -- -- -- -- -- -- --
-- -- -- -- -- -- -- --
-- -- -- -- -- -- -- --
-- -- -- -- 4416399
8695 rs10251097 7 AG AG AA AG GG
AG GG AG AG AG AG GG AG
AG GG AA AG AG AA AA AA
AG AA AA AG AA GG AG AG
GG AG AG AA AG AG AG
103965228
8696 rs10251159 7 AC AC AC AA AC
AC AA CC CC AC AA AA AA

AC AC AA AA AA AC AC AC
AA AC CC AC AC AC CC AC
AA AA CC AC CC CC CC
49313567
8697 rs10251185 7 AA AG AA GG AG
AG GG GG AG AG AG AG AG
AG AG GG GG GG AG GG AA
AG GG AG GG AG AG GG GG
AA GG AG GG AG AG AG
11155829
8698 rs10251209 7 AA AA AA AA AA
AA AA AA AA AA AA AG AG
AA AA AA AA AA AG AA AA
AG AA AA AG AA AA AA AA
AA AG GG AG AA AA AA
49614427
8699 rs10251294 7 AA AA AA AA AA
AA AA AA AA AA AA AA AA
AA AA AA AA AA AA AA AA
AC AA AA AA AA AA AA AA
AA AA AA AA AA AA AA
10130672
8700 rs1025131 2 GG GG GG GG GG
GG GG AG AG AG GG GG GG
AG AG GG GG GG GG GG GG
AA AG AA AG GG GG GG GG
GG AG GG AG GG GG AG
63361516
8701 rs10251365 7 GG GG GG AA AG
AA AG GG GG GG AG AG AA
AG AG AG GG GG AG GG AA
AA AG AG AA GG GG GG GG
AA GG AA AG AG GG AG
103420619
8702 rs10251367 7 GG GG GG GG GG
GG GG GG GG GG GG GG GG
GG GG GG GG GG GG GG GG
GG GG GG GG GG GG GG GG
GG GG GG GG GG GG GG
103119961
8703 rs10251417 7 GG GG GG AG GG
AG GG AG AG GG GG GG GG
AG GG AG GG GG AA GG AG
GG GG AG GG GG AG AG AG
AG GG GG GG GG GG GG
93009888
8704 rs10251492 7 AG AA AG AG AA
AA AG AG AA AA AA AG AA
AA AA AG AA AG GG GG GG
GG AA AG AA AG AG AG AG

	GG	AA	AG	AA	GG	GG	GG
7284546							
8705	rs10251705		7	AG	GG	AG	GG	GG
	GG	GG	GG	GG	GG	GG	GG	GG
	GG	GG	GG	GG	GG	GG	GG	GG
	GG	GG	GG	GG	GG	GG	GG	GG
	GG	GG	GG	GG	GG	GG	GG	
155866470								
8706	rs10251762		7	CC	CC	CC	CC	CC
	CC	CC	CC	CC	CC	CC	CC	CC
	CC	CC	CC	AC	AC	CC	CC	AC
	CC	AC	AC	CC	CC	CC	CC	CC
	CC	CC	AC	CC	CC	CC	AC	
68335032								
8707	rs10251790		7	AA	AA	AA	AA	AA
	AA	AA	AA	AA	AA	AA	AG	AG
	AA	AA	AG	AA	AA	AA	AA	AA
	AA	AA	AA	AA	AA	AA	AA	AA
	AA	AA	GG	AG	AA	AA	AA	
12097701								
8708	rs10251825		7	GG	AG	AG	AA	AA
	AA	AA	AG	AG	AA	AA	AA	AA
	AA	AA	AG	AG	AG	AA	AA	AA
	AA	AA	AA	AA	AG	AG	AA	AG
	AA	AA	AA	AA	GG	GG	AG	
55603590								
8709	rs10251853		7	GG	GG	GG	GG	GG
	GG	GG	GG	GG	GG	GG	GG	GG
	GG	GG	GG	GG	GG	GG	GG	GG
	GG	GG	GG	GG	GG	GG	GG	GG
	GG	GG	GG	GG	GG	GG	GG	
41857321								
8710	rs10251858		7	GG	GG	GG	GG	GG
	GG	GG	GG	GG	GG	GG	GG	GG
	GG	GG	GG	GG	GG	GG	GG	GG
	GG	GG	GG	GG	GG	GG	GG	GG
	GG	GG	GG	GG	GG	GG	GG	
5882048								
8711	rs10251943		7	CC	CC	AC	CC	CC
	AC	AC	CC	AC	AC	AC	AC	CC
	CC	CC	CC	CC	CC	CC	CC	CC
	AC	CC	AC	AC	AC	CC	CC	CC
	CC	AC	CC	CC	CC	CC	CC	
153886615								
8712	rs10251988		7	CC	CC	CC	CC	CC
	CC	CC	CC	CC	CC	CC	CC	CC
	CC	CC	CC	CC	CC	CC	CC	CC
	CC	CC	CC	CC	CC	CC	CC	CC
	CC	CC	CC	CC	CC	CC	CC	
152990490								

8713 rs10252 1 AG GG AG GG GG GG
GG GG AG AG GG GG GG GG
GG GG AG AG AA AG AG GG
GG GG GG AA AG AA AA AG
AG GG AG AG AA GG 46830430
8714 rs1025206 18 AA AG AG AA AA
AG AA AA AA AA GG AA AG
AG AG AG AG GG AA AA AA
AA AA AG AG AG GG AG AG
AA AG AG GG AA AA AA
36125658
8715 rs10252087 7 AA AA AA AA AA
AA AA AA AA AA AA AA AA
AA AA AA AA AA AA AA AA
AA AA AA AA AA AA AA AA
AA AA AA AA AA AA AA
73925395
8716 rs1025212 2 GG GG GG GG GG
GG GG GG AG AG GG GG GG
GG GG GG GG GG AG AG GG
AG AA AG AA AG GG AG AG
GG GG AA AG AG AG AA
64523758
8717 rs10252126 7 AG AG AA AG AG
GG AG GG GG GG AA AG AG
AG AG AG AA AA AA AA AG
AG AG AG AG AG AA AA AG
AG AG AG AG AG GG AA
79097483
8718 rs1025217 3 AA AA AA AG GG
AG AG AG AA AA AA AA AG
AG AG AG AA AG GG GG GG
AA AG AA AG AA AG AG AA
GG AG AA AG GG AG AG
168212344
8719 rs10252209 7 GG GG GG GG GG
GG GG GG GG GG GG GG GG
GG GG GG GG GG GG GG GG
GG GG GG GG GG GG GG GG
GG GG GG GG GG GG GG
11180740
8720 rs1025229 8 AC AC AC AA AA
AC AA AC AA AA AC AA AC
AC AC AC AC AC AC CC AC
AC AC AA CC AC CC AC CC
AC AC AA AC AA AA AC
18812020
8721 rs10252297 7 CC AC CC AA AA
AA AC AA AC AC AC CC AC
AC AC CC AC AC AC AC CC

CC AC CC CC AC CC CC CC
AC AC AC AA AC CC AC
157212045
8722 rs10252308 7 GG AG GG AG AG
GG AA GG AG AG GG AG AG
AA AA AG AG AG AG AA AA
GG GG GG GG AG AA AA AA
GG GG AG AG AG GG AG
148501703
8723 rs10252313 7 GG GG GG GG GG
GG GG GG GG GG GG GG GG
GG GG GG GG GG GG GG GG
GG GG GG GG GG GG GG GG
GG GG GG GG GG GG GG
147568974
8724 rs10252324 7 GG GG GG GG GG
GG GG GG GG GG GG GG GG
GG GG GG GG GG GG GG AG
GG GG GG GG GG GG GG GG
AG GG GG GG GG GG GG
92144751
8725 rs10252363 7 GG GG GG GG GG
GG GG AG AG AG GG GG GG
GG GG GG GG GG GG GG GG
GG GG GG GG GG GG GG GG
AG GG AG GG GG AG GG
16417575
8726 rs10252405 7 AA AA AA AA AA
AA AA AA AA AA AA AA AA
AA AA AA AA AA AA AA AA
AA AA AA AA AA AA AA AA
AA AA AA AA AA AA AA
150008190
8727 rs10252428 7 AC AC AC CC CC
CC CC CC AC CC CC CC CC
AC AC CC CC CC CC CC CC
CC AC AC CC CC CC CC CC
CC CC CC CC CC CC CC
38268811
8728 rs1025248 2 AA AA AG AA AA
AA AA AG AA AA AA AA AA
AA AA AA AA AA AA AA AA
AA AA AA AA AA AA AA AA
AA AG AA AA AA AA AA
49882063
8729 rs10252508 7 AC CC AC AC AC
AA AC AC CC AC AC AC AA
AA AC AC AA AA AA AA AA
AA AC AA AA AC AA AA AA
AC AA AA AA AA AA AA

156616855
8730 rs10252540 7 AC AC AA AA AC
AA AC AA AA AA AA AA AA
AC CC CC AC AC AA AA AC
AC CC AC AA AA AA AA AA
CC AC AC AA AA AA AA
66864120
8731 rs10252544 7 AG AG AG GG AG
AG GG AG AG AG GG GG GG
GG GG AG AG GG AG GG AA
AG GG GG GG AG AA AA AG
AA AG AG GG GG GG AG
17605599
8732 rs10252546 7 AA AA AA AG AA
AA AG AA AG AA GG GG AG
AG AG AA AG AA AG AG AG
AA AA AA AA AA AA AA AA
AA AA AA AA AA AA AA
31934481
8733 rs10252578 7 AG AG AG AA AA
AA AA AG AG AG AA AA AA
AG AG AG AG AG AG AA GG
GG AA AG AG AA AA AA AA
AA AG AA AG AA AA AA
25365550
8734 rs10252635 7 GG GG GG GG GG
GG GG AG GG AA GG GG GG
GG GG AG AG AG AG GG AG
GG GG GG GG GG AG AG AG
GG AA GG AG GG GG GG
47029506
8735 rs10252817 7 GG GG GG AG AA
AA AG AG AG AA AG AG AA
AA AA GG AG AG AG GG AG
AG AG AG AG AG GG AG GG
AA AG AG AG AA AG AA
90904938
8736 rs10252824 7 AA AG AA AG GG
AG AG AG AG AG GG GG GG
GG AG GG AG GG GG GG GG
GG GG GG GG AA GG AG AG
GG GG GG GG AG GG AG
12937656
8737 rs10252831 7 AG AG AG GG GG
GG GG GG GG GG GG GG GG
GG GG GG GG GG GG GG GG
GG GG GG GG AG GG AG GG
GG AG GG AG GG GG GG
68180684
8738 rs1025286 4 CC CC CC CC CC

CC CC CC CC CC CC CC CC
CC CC CC CC CC CC CC CC
AC CC CC AC CC CC CC CC
CC CC CC CC CC CC CC
124081430
8739 rs10252923 7 AA AA AA AC AA
AA AC AC AC AA AA AA AA
AA AA CC AC AC AA CC AC
AC AA AA AC AC CC AC CC
AA AC AC AC AA AC AA
90905041
8740 rs1025294 5 AG AG AA AA AG
AG AA AA AG AA AG AG AA
AA AA AG AA AG AA AA AG
AG AG AA AG AA AA AA AA
AG AA AA AA AA AA AG
11714657
8741 rs10252968 7 AA AG AA AA AA
AA AA AA AA AG AA AA AA
AA AA AG AG AG AA AA AG
AA AA AA AA AG AA AG AG
AA AA AA AA AA AA AA
156135838
8742 rs1025304 5 GG GG GG AG AG
GG AG GG GG GG GG GG GG
GG GG GG GG GG GG GG AG
GG GG GG GG GG GG GG GG
AG GG GG GG GG GG AG
26537760
8743 rs10253050 7 CC CC CC CC CC
CC CC CC CC CC CC CC CC
CC CC CC CC CC CC CC CC
CC CC CC CC CC CC CC CC
CC CC CC CC CC CC CC
73912918
8744 rs10253095 7 GG AG AG GG AG
GG AG GG GG GG GG AG AG
AG AG AA AA AA GG GG GG
GG AG AG GG AG AG AG AG
AG GG AG AG AA AG AG
39254659
8745 rs10253104 7 AA AA AA AA AA
AA AA AG AG AG AA AG AG
AG AA AA AG AG AG AG AG
AG AA AG AG AA AA AA AA
AG AG AG AG AA AA AA
131708955
8746 rs1025315 16 GG GG GG GG GG
GG GG GG GG GG GG GG GG
GG GG GG GG GG GG GG GG

GG GG GG GG GG GG GG GG
GG GG GG GG GG GG AG
77335929
8747 rs1025318 16 GG GG GG GG GG
GG GG GG GG GG AA AA AG
AG AG GG GG GG AA AG AG
AG GG AG AG GG GG GG GG
GG AG AG AG GG AG GG
77268935
8748 rs10253216 7 GG GG GG AG GG
AG GG GG GG GG AG GG AG
AG GG GG GG GG GG AG AG
AG AG AA AG GG GG GG GG
GG AG AA AG AG AG AG
16861849
8749 rs1025322 16 AG AG AG AG AA
AA AG GG AG GG AG AG AA
AA AA -- AG AG AA AA AG
AG AG AA AG AA AG AG AA
AG AA AA AA AG AG AG
77420288
8750 rs10253237 7 AA AA AA AA AA
AA AA AA AA AA AA AA AA
AA AA AA AA AA AA AA AA
AA AA AA AA AA AA AA AA
AA AA AA AA AA AA AA
97154365
8751 rs1025324 5 AG AG AG AG AG
AG AG GG GG GG AA AA AG
AG AG AG GG AG AA AA AG
AG AG AG AG AG AG GG AA
GG GG GG GG GG GG GG
35345497
8752 rs10253271 7 AA AA AA AA AA
AA AA AA AG AG AA AG AG
AG AG AG AA AA AG GG AG
AA AA AA AA AA AA AA AA
AG AA AG AG AG AG AA
148273685
8753 rs10253325 7 AA AA AA AA AA
AA AA AG AG AA AA AG AG
AA AA AA AA AA AA AA AA
AA AA AA AA AG AA AA AG
AA AA AA AA GG AG AG
14486957
8754 rs10253342 7 GG GG GG GG AG
GG AG AG GG GG GG GG GG
GG GG GG GG AG AG GG GG
GG GG GG GG GG GG GG GG
GG GG GG GG GG GG GG

33346932							
8755	rs10253361	7	GG	GG	GG	AG	AA
	AG	AA	AA	AA	AA	AG	AA	AG
	AG	AA	AG	AG	AG	AA	AA	AG
	AG	AA	AG	AG	GG	GG	GG	GG
	AG	AA	AG	AG	AA	AG	AG	
121275229								
8756	rs10253384	7	AA	AA	AA	AA	AA	
	AA	AA	AA	AA	AA	AA	AG	AG
	GG	GG	AA	AA	AG	AA	AA	AG
	AG	AG	AG	AG	AG	GG	AG	GG
	AA	AA	AA	AA	AA	AA	AA	
49359804								
8757	rs1025341	3	AC	AC	AC	CC	CC	
	CC	CC	CC	CC	CC	CC	CC	CC
	CC	CC	CC	CC	CC	CC	CC	CC
	CC	CC	CC	CC	CC	CC	CC	CC
	CC	CC	CC	CC	CC	CC	CC	
155548882								
8758	rs10253501	7	CC	CC	CC	CC	CC	
	CC	CC	CC	CC	CC	CC	CC	AC
	CC	AC	CC	CC	CC	CC	CC	AC
	CC	CC	CC	CC	CC	AC	AC	AC
	CC	CC	CC	CC	CC	CC	CC	
6258898								
8759	rs10253511	7	AA	AA	AA	AA	AA	
	AA	AA	AG	AG	AG	AA	AA	AA
	AA	AA	AA	AG	AG	AA	AA	AA
	AA	AA	AA	AA	AA	AA	AA	AA
	AA	AA	AA	AA	AA	AA	AA	
130796652								
8760	rs10253514	7	AA	AA	AA	AA	AA	
	AA	AA	AA	AA	AA	AA	AA	AA
	AA	AA	AA	AA	AA	AA	AA	AA
	AA	AA	AA	AA	AA	AA	AA	AA
	AA	AA	AA	AA	AA	AA	AA	
102621945								
8761	rs10253536	7	AA	AA	AA	AA	AG	
	AG	AA	AA	AA	AA	AA	AA	AA
	AA	AA	AG	AA	AA	AA	AA	AA
	AA	AA	AA	AA	AG	AA	AA	AG
	AG	AA	AA	AA	AG	AG	AA	
78894075								
8762	rs10253572	7	AG	AA	AG	AA	AG	
	AA	AG	AA	AG	AG	AG	AA	AA
	AA	AA	AA	AG	AA	AA	AA	AA
	AA	AA	AA	AA	AA	AA	AA	AA
	AA	AG	AA	AA	AA	AA	AA	
4655524								
8763	rs1025366	18	AG	AG	AG	GG	GG	

GG GG AG AG AG GG GG GG
GG GG AG AG GG AG AG GG
AG AA AG GG GG GG GG GG
GG GG GG GG GG GG GG
24414687
8764 rs10253670 7 CC CC AC CC CC
CC CC AC AC CC AA AC AC
CC CC CC CC AC CC CC CC
AC CC AC CC CC CC CC CC
AC CC CC CC CC CC CC
41478897
8765 rs10253714 7 AC AC AC CC CC
CC CC AC AC AC CC CC CC
AC AC CC AC CC CC CC CC
CC CC CC CC CC CC CC CC
CC AC AC AC CC CC CC
54394095
8766 rs10253778 7 AA AA AA AA AA
AA AA AA AA AA AA AA AA
AA AA AA AA AA AG AA AA
AA AA AA AA AA AA AA AA
AA AA AG AG AA AA AA
52643487
8767 rs1025378 2 AA AA AA AA AG
AG AG AA AA AA AG AA AA
AA AA AG AA AG AA AA AG
AA AG AA AA AG AG GG AA
GG AA AA AA AG AG AA
109931911
8768 rs10253804 7 AG AG AG AG AA
AG AA AG AA AG GG GG GG
AG AG GG GG AG AA AA AG
AG AG GG GG AG GG AG GG
GG GG GG GG GG GG AG
54807822
8769 rs10253855 7 AG AA AG AG AG
AG AG AG GG GG AG AA AG
GG GG AG AA AA AG GG AG
AA AG AG AG AG AG AG AG
AA AA AA AA AA AA AG
9341294
8770 rs10253857 7 GG GG GG AA AG
AG AA AG AG AG GG AG AG
GG AG GG GG GG GG AG GG
AG AG AG AG GG GG GG GG
GG GG GG GG AG AG AG
17713450
8771 rs10253872 7 AG AA AG AA AG
AA AG AG AG AG GG AA AG
AG AG AG AA AA AG GG AA

	AA	AA	AA	AA	AA	AA	AA	AA
	AA	GG	AA	AG	AA	AA	AA	
11321893								
8772	rs1025397		4	AG	AG	GG	AG	AG
	GG	AA	AG	AG	GG	AG	GG	GG
	GG	GG	AA	AA	AG	GG	GG	GG
	AG	AG	AG	AG	GG	GG	GG	GG
	GG	GG	GG	GG	AG	AG	GG	
116848573								
8773	rs10253972		7	GG	GG	GG	GG	GG
	GG	GG	AG	AG	AG	GG	GG	GG
	GG	GG	GG	GG	GG	GG	GG	AG
	AG	GG	GG	GG	GG	GG	GG	GG
	GG	GG	GG	GG	GG	GG	GG	
18076575								
8774	rs1025400		3	CC	CC	CC	AC	AC
	AA	CC	CC	CC	CC	CC	CC	CC
	CC	CC	CC	CC	CC	CC	CC	CC
	AC	AA	AA	AC	CC	AC	AC	AC
	AA	AC	AC	AC	CC	AC	CC	
113585154								
8775	rs10254047		7	AA	AA	AA	GG	AA
	AG	AG	AA	AG	AA	AA	AA	AA
	AA	AA	AG	AG	AG	AA	AA	AG
	AA	AA	AA	AA	AA	AG	AG	AG
	AA	AA	AA	AA	AG	AA	AG	
27336004								
8776	rs10254084		7	AG	GG	AG	GG	GG
	GG	GG	GG	GG	GG	AG	GG	GG
	AG	AG	AG	AG	AA	AG	AG	AG
	AG	AG	AG	AG	GG	GG	GG	GG
	GG	GG	AG	GG	AG	AG	GG	
9909504								
8777	rs10254096		7	GG	AG	GG	AG	AG
	AG	AG	GG	AG	AG	GG	AG	GG
	AA	AG	GG	GG	AG	AA	AA	AA
	AA	AG	AA	AG	AG	AA	AA	AA
	GG	AG	AA	AG	AG	AA	AG	
7668666								
8778	rs10254114		7	AA	AA	AA	AA	AC
	AC	AA	AA	AA	AA	CC	AC	AC
	AC	AA	AA	AC	AC	AA	AA	AC
	AA	AA	AA	AA	AC	AA	AA	AC
	AC	AA	AC	AC	AA	AA	AC	
79136577								
8779	rs10254116		7	AA	AA	AA	AG	AA
	AG	AA	AA	AG	AA	AA	AA	AG
	AG	AG	AG	GG	AG	AG	AA	GG
	AA	AA	AA	AA	AA	AG	AA	AA
	AA	AA	AG	AA	AG	AG	AG	

33237489
8780 rs1025412 11 AA AG AG AA AG
AG AG AA AG AG AG AG AG
GG GG GG GG GG AG AG GG
AG AG GG GG AG AG GG AA
AA AG AA AA GG AG GG
14246296
8781 rs10254181 7 AG AG AA AA AG
AG AA AA AA AA AA AA AA
AA AG AA AG AG AG GG GG
AA AG GG AA AA AG AG AG
AG AA AG AG AG AA AG
97388270
8782 rs10254192 7 CC CC CC CC CC
CC CC AC AC AC CC CC CC
CC CC CC CC CC AC CC AC
CC CC CC CC CC CC CC CC
CC AC CC AC CC CC CC
21912204
8783 rs10254214 7 GG GG GG GG GG
GG AG GG GG GG GG GG GG
GG GG GG AG AG AG AG AG
GG GG GG GG AG GG AG AG
GG AG AG AG GG GG GG
141017055
8784 rs10254229 7 GG AG GG GG GG
GG AG AG AG AG AG AA AG
AG AG AA AG AG GG AG AG
AG GG AG AG AG AA AA AA
GG GG AG AG GG GG AG
153957101
8785 rs1025426 12 AA AG AA AA AG
AG AA AG AA AG AA AA AA
AA AA AA AA AG AA AA AA
AG AG AA AG AA AA AA AA
AA AA AA AA AG AG GG
128291707
8786 rs10254291 7 GG GG GG GG GG
GG GG GG GG GG GG GG GG
GG GG GG GG GG GG GG GG
GG GG GG GG GG GG GG GG
GG GG GG GG GG GG GG
49752764
8787 rs10254364 7 AG GG AG AA AG
AG AA AA AA AA AA AG AG
AG AG AA AA AA AG AA AG
AA AA AA AA AG AA AA AA
AG AG AG AG AG AG AA
103646910
8788 rs10254402 7 AA AA AA AA AA

	AA	AA	AA	AA	AA	AA	AA	AA
	AA	AA	AA	AA	AA	AA	AA	AA
	AA	AG	AG	AA	AA	AA	AA	AA
	AA	AA	AA	AA	AA	AA	AA	
104192398								
8789	rs10254431		7	AA	AA	AA	AA	AA
	AA	AA	AA	AA	AA	AA	AA	AA
	AA	AA	AA	AA	AA	AA	AA	AA
	AA	AA	AA	AA	AA	AA	AA	AA
	AA	AA	AA	AA	AA	AA	AA	
92861426								
8790	rs10254562		7	AC	AC	AC	AC	CC
	CC	AC	AC	AC	CC	CC	AC	AC
	AC	AC	AA	AC	AC	AC	CC	AC
	AA	AC	AA	AC	CC	AA	AC	AC
	CC	CC	CC	CC	AC	AA	AC	
39661524								
8791	rs10254574		7	AG	AG	GG	AA	GG
	AG	AG	AG	AA	GG	AA	AA	AA
	GG	GG	AA	AG	AA	AG	AG	AG
	AG	AG	GG	AA	AG	AA	AG	AG
	AG	AG	AG	GG	AG	GG	AG	
40011801								
8792	rs10254635		7	AA	AA	AA	AA	AA
	AA	AA	AA	AA	AA	AA	AA	AA
	AA	AA	AA	AA	AA	AA	AA	AA
	AA	AA	AA	AA	AA	AA	AA	AA
	AA	AA	AA	AA	AA	AA	AA	
764910								
8793	rs1025466		5	AG	AA	AG	AG	AA
	AG	AG	AG	AG	AG	AA	AA	AA
	AA	AA	AG	AG	AA	AG	AG	AG
	AG	AG	AG	AG	AA	GG	AG	AG
	AG	GG	AG	GG	GG	AG	GG	
159333536								
8794	rs10254679		7	AA	AA	AA	AA	AA
	AA	AA	AA	AA	AA	AA	AA	AA
	AA	AA	AA	AA	AA	AA	AA	AA
	AA	AA	AA	AA	AA	AA	AA	AA
	AA	AA	AA	AA	AA	AA	AA	
155423197								
8795	rs10254690		7	AG	AG	AA	AG	AG
	AG	AG	AG	AG	AG	GG	AG	GG
	AG	GG	AG	GG	AG	AG	AG	AA
	AG	AG	AG	AG	AA	AG	AG	AG
	AG	AG	GG	AG	AA	AA	AA	
2673288								
8796	rs10254725		7	AA	AA	AA	AA	AA
	AA	AA	AA	AA	AA	AA	AA	AA
	AA	AA	AA	AA	AA	AA	AA	AA

AA AA AA AA AA AA AA AA
AA AA AA AA AA AA AA
63369823
8797 rs1025476 5 GG GG GG AG AA
AA AG AG AG AG AA AA AG
AG AG AG AA AG AA AA AG
GG GG GG GG AG AG GG AG
GG GG AG GG GG AG AG
148389763
8798 rs10254777 7 AG AG GG AG AG
GG AA AG AA AG AG GG AG
AG AA AA AA AA AA AA AA
AA AG AA AA AG GG GG GG
AA AG AA AG AG AG AA
140233585
8799 rs10254800 7 AA AA AG GG GG
GG GG AG AG AG GG AG AG
AG AG AG AA AA AG AG AG
AA AA AA AA GG AG GG GG
AG AG AG AG GG AG AG
48093679
8800 rs10254840 7 AA AA AA AA AA
AA AA AA AA AA AA AA AA
AG AG AA AA AA AG AA AG
AA AA AA AA AA AA AA AA
AG AA AA AA AA AA AA
92333408
8801 rs1025489 5 GG GG GG AG AG
GG AA AG AG AG GG GG AG
AA AA AG GG AG AG AG GG
GG GG AG AG GG GG GG GG
AG AG GG GG GG GG GG
147188475
8802 rs10254965 7 GG AG GG GG GG
GG AG AG GG AG GG GG GG
AG AG AG GG GG GG AG GG
GG GG GG GG GG GG GG GG
AG AG GG AG AG GG AG
157848770
8803 rs10254967 7 AA AA AA AA AA
AA AA AA AA AA AA AA AA
AG AG AA AA AA AA AA AA
AA AA AA AA AA AA AA AA
AA AA AA AA AA AA AA
158635072
8804 rs10255004 7 AG GG AG AG AG
AA GG AG GG AA AA AG AG
AA AG AA AG AG GG GG GG
AG AA AG AA GG AG GG GG
GG AG AA AA AG AA GG

1701592
8805 rs10255045 7 AA AA AA AA AA
AA AA AA AA AA AA AA AA
AA AA AA AA AA AA AA AA
AA AA AA AA AA AA AA AA
AA AA AA AA AA AA AA
150866464
8806 rs1025512 10 AG AG AG GG GG
GG GG GG GG GG AG AG GG
GG GG GG GG GG GG AG GG
GG GG GG GG AG GG GG GG
AG GG GG GG GG GG GG
127237449
8807 rs10255125 7 AA AA AA AA AA
AA AC AC AC AA AC CC AC
AA AC AA AA AA AC AC AC
AA AA AC AC CC AC AC AC
CC AC CC CC AC AA AC
78346008
8808 rs10255142 7 AG AG AG GG AG
GG AG GG GG AG AA AG AG
GG GG GG GG GG GG AG GG
AG AG AG AG AG GG GG AG
GG AG AG AG AG GG AG
47160224
8809 rs10255174 7 GG GG AG AG AG
GG AA AG AG GG GG AG AG
AA AA GG GG GG GG AG AG
AG AG AA AA AG AA AA AG
AG AG AG AG AG GG AG
51978391
8810 rs10255200 7 AG AG GG AG AG
AA AG AA AA AA AG AA AG
AG GG AG AA AA AA AG AG
AG AG AG AG GG AG GG GG
AA GG AG AG AG AA AG
123679743
8811 rs10255304 7 GG GG GG AA GG
AG AG GG GG GG AG GG AG
AA GG GG AG AG GG GG GG
GG GG GG GG AG AG AA AA
AG GG GG GG AG AG GG
104172232
8812 rs10255332 7 AA AA AA AA AA
AA AA AA AA AA AA AA AA
AA AA AA AA AA AA AA AA
AA AA AA AA AA AA AA AA
AA AA AA AA AA AA AA
3984462
8813 rs1025542 2 AG GG AG AG GG

	GG	AG	GG	GG	GG	AG	GG	GG
	AG	GG	GG	GG	GG	AA	AA	AG
	AG	AG	GG	AG	GG	GG	GG	GG
	GG	GG	GG	GG	GG	AG	AG	
26385849								
8814	rs1025547		9	AC	AC	AA	AC	AA
	AA	AC	AA	AA	AC	CC	AC	CC
	AC	AC	CC	CC	CC	AC	AC	CC
	CC	CC	CC	CC	AC	AC	AC	AC
	CC	CC	AC	AC	AA	AC	AA	
8664838								
8815	rs10255498		7	GG	GG	GG	GG	GG
	GG	GG	GG	GG	GG	GG	GG	GG
	GG	GG	GG	GG	GG	GG	GG	GG
	GG	GG	GG	GG	GG	GG	GG	GG
	GG	GG	GG	GG	GG	GG	GG	
66336305								
8816	rs1025558		10	AC	AA	AC	CC	AC
	CC	AC	AA	AA	AC	AC	AC	CC
	CC	CC	CC	AC	AC	CC	CC	CC
	CC	CC	AA	AA	CC	CC	CC	CC
	AC	AC	CC	CC	CC	CC	CC	
80386576								
8817	rs10255595		7	CC	CC	CC	CC	CC
	CC	CC	CC	CC	CC	CC	CC	CC
	CC	CC	CC	CC	CC	CC	CC	CC
	CC	CC	CC	CC	CC	CC	CC	CC
	CC	CC	CC	CC	CC	CC	CC	
49382062								
8818	rs10255640		7	GG	GG	GG	GG	GG
	GG	GG	GG	GG	GG	GG	GG	GG
	GG	GG	GG	GG	GG	GG	GG	GG
	GG	GG	GG	GG	GG	GG	GG	GG
	GG	GG	GG	GG	GG	GG	GG	
148502169								
8819	rs10255662		7	AA	AA	AA	AA	AG
	AG	AA	AG	AG	AG	AG	AA	AG
	AA	AG	AG	AA	AA	GG	GG	GG
	AG	AG	AG	AG	AG	AG	AG	AG
	AA	AG	AA	AA	AA	AG	AA	
6291937								
8820	rs1025572		9	AA	AA	AA	AA	AA
	AA	AA	AA	AA	AA	AA	AA	AA
	AA	AA	AA	AA	AA	AA	AA	AA
	AA	AA	AC	AA	AC	AA	AC	AC
	AA	AA	AA	AA	AA	AA	AA	
110887234								
8821	rs10255750		7	GG	AG	AG	AA	GG
	AG	AG	AA	AG	AG	AA	AA	AG
	AG	GG	AA	AG	AA	AG	AG	AA

	AA	AG	AG	AG	AG	AA	AA	AA
	GG	AA	AA	AA	AG	AG	AA	
4279500								
8822	rs10255772		7	CC	CC	CC	CC	CC
	CC	CC	CC	CC	CC	CC	CC	CC
	CC	CC	CC	CC	CC	CC	CC	CC
	CC	CC	CC	CC	CC	CC	CC	CC
	CC	CC	CC	CC	CC	CC	CC	
121223								
8823	rs10255814		7	GG	GG	GG	GG	GG
	GG	GG	GG	GG	GG	GG	GG	GG
	GG	GG	GG	GG	--	GG	GG	GG
	GG	GG	GG	GG	GG	GG	GG	GG
	GG	GG	GG	GG	GG	GG	GG	
55390684								
8824	rs10255837		7	AA	AA	AA	AG	AA
	AG	AA	AA	AA	AA	AA	AG	AG
	AG	AA	AA	AA	AA	AA	AA	AA
	AA	AG	AA	AA	AA	AG	AG	AG
	AA	AA	AG	AG	AA	AG	AG	
135055741								
8825	rs10255854		7	AG	GG	GG	AG	GG
	GG	AG	AG	GG	AG	GG	GG	GG
	GG	GG	GG	GG	GG	GG	GG	GG
	GG	AA	AG	AG	GG	AG	GG	GG
	GG	GG	GG	GG	AG	GG	AG	
38959842								
8826	rs1025595		11	AA	AG	GG	AG	GG
	AG	GG	GG	GG	GG	GG	GG	AG
	GG	AG	GG	GG	GG	AG	AG	AA
	AA	AG	AA	GG	AG	AG	AG	AG
	AG	GG	AA	AG	AG	AG	AG	
78690093								
8827	rs10255956		7	AG	GG	GG	GG	GG
	AG	GG	GG	AG	AG	GG	GG	GG
	GG	GG	AG	AG	AG	GG	GG	GG
	AG	GG	AG	AG	GG	AG	GG	GG
	GG	GG	AG	GG	AG	GG	AG	
147870469								
8828	rs10255959		7	AA	AA	AA	AA	AA
	AA	AA	AA	AA	AA	AA	AA	AA
	AA	AA	AA	AA	AA	AA	AA	AA
	AA	AA	AA	AA	AA	AA	AA	AA
	AA	AA	AA	AA	AA	AA	AA	
157378725								
8829	rs1025601		18	AA	AA	AG	AA	AG
	AG	AG	GG	GG	GG	AG	AG	GG
	AG	AA	GG	AG	AG	AG	GG	AG
	AG	GG	AG	GG	AG	AG	AG	GG
	AA	AA	AG	AA	GG	GG	GG	

73056278
8830 rs10256019 7 GG GG GG AG AA
AG AG AG AG AG GG AG AG
GG GG GG GG GG GG GG GG
GG GG GG GG GG AG AG AG
GG AG GG AG GG GG AG
12934776
8831 rs10256029 7 AA AA AA AA AA
AA AA AA AA AG AA AA AA
AA AG AA AA AA AG AA GG
AG AG AA AA AG AG AG GG
AA AA AG AG AA AA AA
71168010
8832 rs10256062 7 AA AG AA AA AG
AG AA GG AG GG AG GG AG
AG GG AA AA AA AA AG AA
AG GG GG AG AG AA AG AA
AA AG AA AA AG AG AA
69787054
8833 rs10256080 7 AG AA AG AG AG
AA GG GG GG GG AG AG GG
GG AG GG GG GG AG GG AG
GG AG GG GG AG GG GG GG
GG GG GG GG AA AG AG
158019437
8834 rs10256093 7 AA AA AA AA AG
AG AA AA AA AG AG AG AA
AA AG AA AA AA AA AA AA
AG AG AA AA AA AA AA AA
AA AA AA AA AG AG AA
69682576
8835 rs10256154 7 AA AA AA AA AA
AA AA AA AA AA AA AA AA
AA AA AA AA AA AA AA AA
AA AA AA AA AA AA AA AA
AA AA AA AA AA AA AA
2091571
8836 rs10256180 7 AG AG AG GG AG
GG AG GG GG GG GG GG GG
GG AG GG GG GG GG AG GG
GG GG GG GG GG GG GG GG
GG GG AG GG GG GG GG
8961636
8837 rs10256183 7 AG GG GG AA GG
AG AG AG AG GG AA AA AA
AA AA AG AG GG GG AG GG
AG AG AG AG AG GG GG AG
GG AG AG AG GG GG GG
52222322
8838 rs10256186 7 AA AG AA AA AG

AG AA GG AG GG AG GG AG
AG GG AA AA AA AA AG AA
AG GG GG AG AG AA AG AA
AA AG AA AA AG AG AA
69641784
8839 rs10256188 7 AG AG GG AG GG
AG GG GG GG GG AA AA AA
AA AA AA AA AA AA AA AA
AG GG AG AG AG AA AA AA
AG GG GG GG AG AG AG
132317536
8840 rs10256282 7 AA AA AG AA AA
AA AA GG AG GG AG AG GG
GG AG AG AA AA AA AA AG
AG AG AG AG AA AA AA AA
AG GG AA AG AA AG AA
139528444
8841 rs10256308 7 AA AA AA AG AG
GG AA AA AA AA AA AA AA
AA AA AA AA AA AA AG AG
AA AA AA AA AA GG AG AG
AG AG AA AG AA AA AA
104460747
8842 rs10256342 7 CC CC CC AC AC
AA CC AC CC CC CC CC CC
CC CC AC AC AC CC CC CC
CC AC CC CC CC CC CC CC
AC CC CC CC AC CC AC
152595807
8843 rs10256347 7 GG GG GG GG GG
GG GG GG GG GG GG GG GG
GG GG GG GG GG GG GG GG
GG GG GG GG GG GG GG GG
GG GG GG GG GG GG GG
115883585
8844 rs1025637 10 GG GG GG AG AG
GG AG GG GG GG GG GG GG
GG GG AG GG AG AG AA AG
AA AA AA AG GG AG GG AG
GG GG GG GG GG GG GG
34803585
8845 rs10256379 7 GG GG GG GG AG
AG GG GG GG GG GG AG AG
AG GG AG AA AA AG AG AG
AA AA AA AA AG AA AG AG
AG AA AA AA AG AG AG
140955139
8846 rs1025640 1 AA AA AA AA AA
AA AA AA AA AA AA AA AA
AA AA AA AA AA AA AA AA

AA AA AA AA AA AA AA AA
AA AA AA AA AA AA AA
34450727
8847 rs10256416 7 CC CC CC CC CC
CC AC CC CC CC AC AC CC
CC CC CC CC CC AA AC AC
CC CC CC CC CC CC CC CC
CC CC CC CC CC CC CC
77871607
8848 rs1025644 3 AA AA AA AA AA
AA AA AG AG AA AA AA AA
AG AG AA AA AA AA AA AA
AA AA AA AA AA AA AA AA
AA AA AA AA AA AA AA
29331859
8849 rs10256461 7 AG AA AG AA AA
AA AA AG AG GG AA AA AA
AG AA AG AG AG AG AA AA
AA AG AG AA AG AG AA AA
AA AA AA AA AG AG AA
97160590
8850 rs10256544 7 GG AG AG AA AG
AA AG AA AA AG AG AA AG
AG AG AG AG AG GG AG AG
AA AA AA AA AA AG AA AA
AG GG AG AG GG AG GG
65675128
8851 rs10256700 7 GG GG GG GG GG
GG GG GG GG GG GG GG GG
GG GG GG GG GG GG GG GG
GG GG GG GG GG AG AG AG
GG GG GG GG GG GG GG
147812591
8852 rs10256711 7 AA AA AA AA AA
AA AA AA AA AA AA AA AA
AA AA AA AA AA AA AA AA
AA AA AA AA AA AA AA AA
AA AA AA -- AA AA AA
41645139
8853 rs10256772 7 AG AG AG GG GG
GG AG GG AG GG GG GG GG
GG GG AG GG GG GG AG GG
AG GG AG AG AG GG GG GG
AG AG AG AA AA AG AG
157514189
8854 rs10256791 7 AG AG AG AA AG
AA AG GG AG AG GG GG GG
GG GG GG GG AG AG AA GG
AG GG AG GG AG AA AG AG
GG AG GG AG GG AG GG

40364726								
8855	rs10256854		7	AA	AA	AA	AA	AA
	AA	AA	AC	CC	AC	AA	AA	AA
	AA	AA	AC	AA	AA	AC	AA	AC
	AA	AA	AA	AA	AC	AA	AC	AC
	AA	AA	AA	AA	AA	AA	AA	
136612314								
8856	rs10256873		7	AA	AG	AG	GG	GG
	GG	GG	GG	GG	GG	AG	AG	GG
	GG	GG	GG	AG	AG	AA	AG	AA
	AG	AG	GG	AG	GG	AG	GG	GG
	GG	GG	AG	GG	AG	GG	AA	
126368292								
8857	rs10256885		7	GG	GG	GG	GG	GG
	GG	GG	GG	GG	GG	GG	GG	GG
	GG	GG	GG	GG	GG	GG	GG	GG
	GG	GG	GG	GG	GG	GG	GG	GG
	GG	GG	GG	GG	GG	GG	GG	
94233290								
8858	rs10256906		7	AG	AG	AG	AA	AA
	AG	AA	AA	AG	AA	AA	AA	AA
	AA	AA	AA	AA	AA	AG	AA	AA
	AA	AA	AA	AA	AA	AG	AA	AA
	AA	AA	AA	AA	AA	AA	AA	
145770180								
8859	rs10256915		7	AG	AA	AG	AA	AA
	AA	AA	AG	GG	AG	AG	GG	AG
	AG	AA	AA	AA	AA	AG	AG	AG
	AG	AA	AG	AG	AA	AG	AA	AA
	AG	AG	AG	AA	AG	AG	AA	
136913223								
8860	rs10256959		7	AA	AA	AA	AA	AA
	AA	AA	AA	AA	AA	AA	AA	AA
	AA	AA	AA	AA	AA	AA	AA	AA
	AA	AA	AA	AA	AA	AA	AA	AA
	AA	AA	AA	AA	AA	AA	AA	
41395538								
8861	rs10257046		7	AG	AG	AG	GG	AG
	AG	GG	GG	GG	GG	GG	GG	GG
	GG	GG	GG	GG	GG	AG	AG	GG
	GG	GG	GG	GG	GG	GG	GG	GG
	AG	GG	GG	GG	GG	AG	GG	
18720135								
8862	rs10257047		7	GG	GG	GG	GG	GG
	GG	GG	GG	GG	GG	GG	GG	GG
	GG	GG	GG	GG	GG	GG	GG	GG
	GG	GG	GG	GG	GG	GG	GG	GG
	GG	GG	GG	GG	GG	GG	GG	
38169198								
8863	rs10257090		7	GG	GG	GG	GG	GG

GG GG GG GG GG GG GG GG
GG GG GG GG GG GG GG GG
GG GG GG GG GG GG GG GG
GG GG GG GG GG GG GG
98924645
8864 rs10257155 7 CC CC CC CC CC
CC CC CC CC CC CC CC CC
CC CC CC CC CC CC CC CC
CC CC CC CC CC CC CC CC
CC CC CC CC CC CC CC
35425269
8865 rs10257158 7 GG GG GG GG GG
GG GG GG GG GG GG GG GG
GG GG GG GG GG GG GG GG
GG GG GG GG AG GG GG AG
GG GG GG GG GG GG GG
14591967
8866 rs10257161 7 AA AA AA AA AA
AA AA AA AA AA AA AA AA
AA AA AA AA AA AA AA AA
AA AA AA AA AA AA AA AA
AA AA AA AA AA AA AA
18273033
8867 rs10257247 7 AA AA AA AA AA
AA AA AA AG AG AA AA AA
AA AA AA AA AA AA AA AA
AG AA AG AA AA -- AA AA
AA AA AA AA AG AA AG
6594288
8868 rs10257272 7 GG AG GG AG GG
AG GG GG GG GG GG GG GG
GG GG GG GG GG GG GG GG
GG GG GG GG AG AG AG AA
GG GG GG GG GG GG GG
29936886
8869 rs10257276 7 GG GG GG GG GG
GG GG AG GG AG AA AG AG
AG AA AG GG GG AG AG AA
GG GG GG GG GG AA AG AG
GG GG GG GG AG GG AG
136370812
8870 rs10257285 7 AA AA AA AG AG
AA GG AG AA AA AG AG AA
AG AG AG AG AG AA AA AG
GG AA AG AA GG AG GG GG
AA GG AG GG AA AG AA
8123427
8871 rs10257413 7 AA AA AA AA AA
AA AA AA AA AA AA AA AA
AA AA AA AA AA AA AA AA

	AA	AA	AA	AA	AA	AA	AA	AA
	AA	AA	AA	AA	AA	AA	AA	
95863039								
8872	rs10257427		7	AA	AG	AG	GG	AG
	GG	AG	GG	GG	AG	AG	GG	AG
	AG	AG	AG	AG	AG	AA	AG	AG
	GG	GG	AG	AG	GG	AG	GG	GG
	AG	AA	AG	AG	AA	AG	AA	
65743208								
8873	rs10257454		7	AA	AA	AA	AA	AA
	AA	AA	AA	AA	AA	AA	AA	AA
	AA	AA	AA	AA	AA	AA	AA	AA
	AA	AA	AA	AA	AG	AA	AG	AA
	AA	AA	AG	AA	AA	AA	AA	
77093557								
8874	rs10257462		7	CC	AC	AC	AC	AA
	AC	AA	AC	AC	AA	AA	AA	AA
	CC	CC	AA	AC	AC	AC	AC	CC
	CC	AC	AC	CC	AC	AC	AA	AC
	AC	AA	AA	AA	AC	AA	AC	
57520410								
8875	rs1025750		2	AA	AA	AA	AA	AA
	AA	AA	AA	AA	AA	AA	AA	AA
	AA	AA	AA	AC	AA	AC	CC	AA
	AC	AA	AA	AC	AA	AA	AA	AA
	AC	AA	AA	AA	AA	AA	AA	
165246738								
8876	rs1025752		2	AA	AA	AA	AG	AG
	GG	AA	AG	AG	AG	GG	AA	AG
	AA	AG	AG	AA	AA	AA	AG	AG
	AG	AG	AG	AA	AG	AG	AG	AA
	AG	GG	AG	AG	AA	AG	AG	
212723459								
8877	rs10257539		7	GG	GG	AG	GG	AG
	AG	GG	AG	AG	AG	AG	AG	GG
	GG	GG	AG	AG	AG	GG	GG	GG
	GG	AG	GG	GG	AG	AA	AA	AA
	AG	GG	GG	GG	AG	GG	AG	
135439915								
8878	rs1025758		4	CC	CC	CC	AC	AC
	AC	AC	CC	CC	CC	CC	CC	CC
	CC	CC	CC	CC	CC	CC	CC	CC
	AC	AC	AC	CC	AC	CC	AC	AC
	CC	CC	CC	CC	AC	AC	CC	
182480385								
8879	rs10257625		7	CC	CC	CC	CC	CC
	CC	CC	CC	CC	CC	CC	CC	CC
	CC	CC	CC	CC	CC	AC	AC	CC
	CC	CC	CC	CC	AC	AC	AC	AA
	CC	CC	CC	CC	CC	CC	CC	

17099149
8880 rs10257673 7 CC CC CC CC CC
CC CC CC CC CC CC CC CC
CC CC CC CC CC CC CC CC
CC CC CC CC CC CC CC CC
CC CC CC CC CC CC CC
92881494
8881 rs1025768 15 AA AA AA AA AA
AA AA AA AA AA AA AA AA
AA AA AA AA AA AA AA AA
AA AA AA AA AA AG AG AA
AA AA AA AA AG AA AG
93517214
8882 rs10257690 7 AG AG AA AG AG
AA GG AA AG AA GG AG AG
AG GG AG AG AG AG AG GG
GG AG GG GG GG GG GG GG
GG AA AG AG GG GG AG
143728163
8883 rs10257697 7 AG AA AG AG AG
AA GG GG GG GG AA AG AG
AG GG AG GG AA AG AG GG
AG AA AA AA AA AG AA AA
AG AG AA AG AG AA GG
18090280
8884 rs10257747 7 AA AG AA AA AG
AG AA AG AA AA GG AG AG
GG AG AA AA AG AG AA AG
GG AG GG AG AG AA AG AA
AG AA AG AA AG AA AG
11742205
8885 rs10257756 7 AA AG AG AA AA
AG AA AG AG AA GG GG GG
AG AG AA AA AA AG AA AA
GG AA GG GG AG AG AA AA
AG AG AG AG AA AG AG
148385725
8886 rs10257936 7 AA AA AA AA AA
AA AA AA AA AA AA AA AA
AA AA AA AA AA AA AA AA
AA AA AA AA AA AA AA AA
AA AA AA AA AA AA AA
77455786
8887 rs10257969 7 GG GG GG GG GG
GG GG GG GG GG GG GG GG
GG GG GG GG GG GG GG GG
GG GG GG GG GG GG GG GG
GG GG GG GG GG GG GG
93771885
8888 rs1025797 8 CC CC CC AC AA

	AC	AC	AC	AC	AC	CC	CC	CC
	CC	CC	CC	CC	CC	AC	CC	AC
	CC	CC	CC	CC	CC	AC	CC	AC
	CC	AC	CC	AC	AC	CC	AC	
2037281								
8889	rs10257971		7	AG	AA	AG	AA	AA
	AA	AA	AA	AA	AA	AA	AA	AG
	AA	AG	AA	AA	AA	AA	AA	AG
	AA	AA	AA	AA	AG	AG	GG	GG
	AA	AA	AA	AA	AA	AA	AA	
6162839								
8890	rs10257984		7	GG	AG	GG	AG	AG
	AG	AG	AG	AA	GG	GG	GG	GG
	GG	GG	GG	GG	GG	GG	GG	GG
	AG	AG	AA	GG	GG	GG	GG	GG
	AA	GG	GG	GG	AG	AA	GG	
37833185								
8891	rs10258053		7	GG	AG	AG	AG	AG
	AA	GG	AG	AG	AG	AG	AG	AG
	AG	AG	AG	AA	AA	AG	AA	AG
	AG	AG	AG	AG	GG	AG	AG	AG
	GG	GG	GG	GG	GG	AG	GG	
95271501								
8892	rs1025806		1	AG	AA	AG	AG	AG
	AG	GG	GG	GG	GG	GG	GG	GG
	GG	GG	AG	AG	AG	AG	AG	GG
	GG	AG	GG	GG	AG	GG	AG	GG
	--	GG	GG	GG	GG	GG	GG	
47139103								
8893	rs10258097		7	AG	GG	AG	GG	AG
	GG	AG	GG	AG	GG	GG	GG	GG
	AG	AG	AA	AA	AA	AA	GG	AA
	GG	GG	GG	GG	GG	GG	GG	GG
	GG	AG	GG	GG	AG	AG	AG	
30876432								
8894	rs10258251		7	AC	AA	AC	AA	AA
	AA	AA	AA	AA	AA	AA	AA	AA
	AA	AA	AA	AA	AA	AA	AA	AA
	AA	AA	AA	AA	AA	AA	AA	AA
	AA	AC	AA	AC	AA	AA	AA	
13021705								
8895	rs10258322		7	AG	AG	AG	AG	AG
	AA	GG	--	GG	AG	GG	GG	GG
	AG	AG	AG	AG	AG	GG	AG	GG
	AA	AG	AG	AA	GG	AG	AG	AG
	GG	GG	AG	GG	AG	AG	GG	
46261337								
8896	rs1025833		15	GG	GG	GG	GG	GG
	GG	GG	GG	GG	GG	AG	AA	AG
	AG	AG	GG	GG	GG	GG	GG	GG

GG GG GG GG GG AG AG GG
GG AG GG AG GG GG GG
98224547
8897 rs10258425 7 AA AA AA AA AA
AA AA AA AA AA AA AA AA
AA AG AG AA AA AA AG AG
AA AA AA AA AA AG AG AG
AG AA AA AA AA AA AA
119772461
8898 rs10258484 7 AA AA AA AA AA
AA AA AG AG AG AA AA AA
AA AA AA AG AA AA AG AA
AA AG AG AA AG AG AG AA
AA AG AG AG AA AA AG
22452083
8899 rs10258578 7 AG AG AG AG GG
AG GG GG GG GG AG AG AA
AG AA GG AG GG GG GG GG
AG AG GG AA AG AG AG AA
GG AG GG GG -- GG GG
29509169
8900 rs10258594 7 AA AG AG AG GG
GG AG AG AG GG GG GG GG
GG AG AG AG AA GG AG AG
AG AG GG GG AG GG GG AG
AG AA AG AA GG AG AG
64320829
8901 rs1025864 3 GG GG GG AG GG
GG AG GG GG GG GG GG GG
GG GG GG GG AG AG AA AG
GG AG GG AG AG GG AG GG
GG GG GG GG GG AG GG
179452888
8902 rs10258646 7 AA AA AA AA AC
AC AA AA AA AA AC AC AA
AA AA AC AC AC AA AA AA
AA AA AA AA AC AA AA AA
AC AA AC AA AC AC AA
104084837
8903 rs10258649 7 AA AA AG AG AG
AA AG AG AG AA AG AA AG
AG AA AA AA AA AA AG AA
AA AA AA AA AA AA AA AA
AA AA AA AA AA AG AA
68751088
8904 rs10258680 7 AA AA AA AG AA
AA AG AA AA AA AA AA AA
AA AA AA AA AA AA AA AA
AA AA AA AA AA AA AA AA
AA AA AG AA AA AA AA

50954338
8905 rs1025869 2 CC CC CC CC CC
CC CC CC CC CC AC CC AC
CC CC CC CC CC CC AC CC
CC CC CC CC CC CC CC CC
CC CC CC CC CC CC AC
106781670
8906 rs10258696 7 AC AC AA CC AC
AC CC AC AA AC CC CC CC
AC AC CC CC AC AC AC AC
AC CC CC AC AC CC AC CC
CC AC AC AC CC CC AC
54478502
8907 rs10258707 7 AG AG AG AA AG
AG AA AA AA AG AA AA AA
AA AA AG AG AA GG AG AG
GG AG AG AG AG AA AA AG
AG AG AG AG AA AA AA
15889830
8908 rs1025876 2 AC AC CC AA CC
AC AC AC AC AC CC CC CC
AC AC AC CC AC AA AA AA
AC AC AC AC CC CC CC CC
AC AC AA AC CC CC CC
60012090
8909 rs10258763 7 AG GG GG -- --
-- GG AA AG AA GG -- GG
GG -- AG GG -- GG AG --
-- AG -- -- AG AG -- GG
-- GG -- -- -- -- AA
144679309
8910 rs1025877 4 AG AA AA GG GG
AG AG AA AA AA AG AG AG
AG AA AG GG AG AA AA AA
AG AG AG AA AG AG AG GG
AA GG AG AG AG AA AG
12074137
8911 rs10258805 7 GG GG GG GG GG
GG GG GG GG GG GG GG GG
GG GG GG GG GG GG GG GG
GG GG GG GG GG GG GG GG
GG GG GG GG GG GG GG
26308744
8912 rs10258822 7 AG AA AG AA AG
AG AA AA AG AA AA AA AA
AG AG AG AG AG AG AG AA
AA AG AG AA AG AG AA AG
GG AA AG AA AA AG AA
21266973
8913 rs10258862 7 GG GG AG AG GG

GG AG AG AG AA GG AG AG
AG AG AA AA AA AG AG AG
AG AA AA AG AG GG GG GG
GG GG AG AG AA AA AG
39116798
8914 rs10258887 7 AA AA AA AA AA
AA AA AA AA AA AA AA AA
AA AA AA AA AA AA AA AA
AA AA AA AA AA AA AA AA
AG AA AA AA AG AA GG
27940578
8915 rs10258890 7 AA AA AA AG AG
AG AG GG GG GG AG AG AA
AA AG GG GG GG AG AG AG
AG GG GG AG GG GG GG GG
GG GG GG GG AG AA AG
96243946
8916 rs10258925 7 AG GG GG AG AG
AA GG GG GG GG AA AG AG
GG GG GG AG GG GG AG GG
GG GG GG GG GG AA AG AG
AG AG AG GG AG AA GG
49060792
8917 rs1025896 18 GG GG GG AG AG
GG AG GG GG GG GG GG GG
GG GG GG GG GG GG GG GG
GG GG GG GG AG GG GG AG
GG GG GG GG GG GG GG
18942583
8918 rs10258969 7 AA AA AA AG AG
GG AG GG GG AG AG AG AA
AG GG AA AA AA AG AA AA
AG GG AG AG AA GG AG AG
GG AG GG GG AG AG GG
156157753
8919 rs10259001 7 GG GG GG GG GG
GG AG GG GG GG GG GG GG
GG GG GG GG GG GG GG GG
GG GG GG GG GG GG GG GG
GG GG GG GG GG GG AG
75265792
8920 rs10259011 7 GG GG GG GG GG
GG GG GG GG GG GG GG GG
GG GG GG GG GG GG GG GG
GG GG GG GG GG GG GG GG
GG GG GG GG GG GG GG
93714157
8921 rs10259019 7 AA AA AA AA AA
AA AA AA AA AA AA AA AA
AA AA AA AA AA AA AA AA

	AA	AA	AA	AA	AA	AA	AA
	AA	AA	AA	AA	AA	AA	AG
67831297							
8922	rs10259038	7	GG	GG	GG	GG	GG
	GG	GG	GG	GG	GG	AA	AA
	AG						
	GG	GG	GG	GG	GG	GG	GG
	GG						
	GG	GG	GG	GG	AG	GG	AG
	AG						
	AG	AG	GG	AG	GG	GG	GG
32081165							
8923	rs1025905	17	AA	AG	AG	GG	AG
	AG	AG	AG	GG	GG	AA	AA
	AA						
	AA	AA	AA	AA	AA	AG	AG
	GG						
	AG	AG	AG	AG	AG	AA	AG
	AG						
	AG	AA	AA	AA	AA	AG	AA
52704312							
8924	rs10259063	7	AA	AA	AA	AA	AA
	AA	AA	AA	AA	AA	AA	AA
	AA						
	AA	AA	AG	AG	AA	AA	AA
	AA						
	AA	AA	AA	AA	AG	AA	AG
	AA						
	AA	AA	AA	AA	AA	AA	AA
20142634							
8925	rs10259067	7	AG	GG	AG	GG	GG
	GG	GG	GG	GG	GG	GG	GG
	GG						
	GG	GG	AG	GG	GG	GG	GG
	GG						
	GG	GG	GG	GG	GG	GG	GG
	GG						
	GG	GG	GG	GG	GG	GG	GG
95231456							
8926	rs10259085	7	AG	AA	AG	AG	AA
	AA	AG	AG	AA	AA	AG	AG
	AA						
	AA	AA	AG	AG	AG	GG	GG
	GG						
	GG	AG	AG	AG	GG	AG	GG
	GG						
	GG	AA	AG	AA	GG	GG	GG
7268431							
8927	rs10259095	7	AG	GG	AG	AA	AA
	AA	AA	AA	AA	AA	AG	AA
	AG						
	AG	AG	AG	AG	AG	AG	AG
	AA						
	AA	AA	AA	AA	AG	AA	AG
	AG						
	AA	AG	AA	AA	AA	AA	AG
132476411							
8928	rs10259109	7	AC	CC	AC	CC	CC
	CC	CC	CC	CC	AC	CC	CC
	CC						
	CC	CC	AC	CC	AC	AC	CC
	AC						
	AA	CC	AC	CC	AA	AC	AC
	AC						
	AC	AC	CC	AC	AC	CC	AA
17540774							
8929	rs10259132	7	AA	AA	AA	AA	AA
	AA	AA	AA	AA	AA	AA	AA
	AA						
	AA	AA	AA	AA	AA	AA	AA
	AA						
	AA	AA	AA	AA	AA	AA	AA
	AA						
	AA	AA	AA	AA	AA	AA	AA

50853634
8930 rs10259163 7 GG AG AG GG AG
AG AG AA AA AA GG AG AG
AG AG GG GG GG GG AG GG
GG AG AG GG GG AA AG AG
GG AG AG AG AA AA AG
109885059
8931 rs10259185 7 AA AA AA AA AA
AA AA AA AA AA AA AA AA
AA AA AA AA AA AA AA AA
AA AA AA AA AA AA AA AA
AA AA AA AA AA AA AA
98428722
8932 rs1025921 10 GG AG GG AG AG
AG GG AG GG AG GG GG GG
GG GG GG AG GG AG GG GG
GG GG GG GG GG GG GG GG
AG GG GG GG GG GG GG
4713705
8933 rs10259230 7 AG AA AG AG AA
AA AG AA AA AA AG AG AG
GG AA AG GG GG AG AG AA
GG AG AG GG AG AA AA AG
AA AA AA AA AA AA AA
89635741
8934 rs10259251 7 GG GG GG GG GG
GG GG GG GG GG AG GG GG
GG GG GG GG GG GG GG GG
GG GG GG GG GG GG GG GG
GG AG GG AG GG GG GG
9062830
8935 rs1025926 8 GG AG GG AG AG
GG AA GG GG GG AG AG GG
GG GG GG GG GG GG AG GG
AG GG GG GG GG GG GG GG
AG GG AA AG GG GG AG
72953158
8936 rs1025928 8 AG GG AG AG AG
AA GG AG AG AA AG GG AG
AG GG GG GG GG AA AG AA
GG GG GG GG AG AA AG AG
AG GG -- GG AG GG AG
72963258
8937 rs10259281 7 AA AA AA AA AG
AG AA AG AA AG GG AG AG
AG GG AG AG AG AG AG GG
AA AG AA AA AA GG AG AG
AG AA AA AA AG AG AG
136389040
8938 rs10259320 7 AA AA AC AC AA

	AA	AC	AC	AC	CC	CC	AC	AC
	AA	AA	AC	AA	AC	AC	AC	AA
	AA	AA	AA	AA	AC	AA	AA	AC
	AA	AC	AA	AC	AA	AA	AA	
55334727								
8939	rs10259329		7	GG	GG	GG	AG	AG
	GG	AG	GG	GG	GG	GG	GG	GG
	GG	GG	GG	GG	GG	AG	GG	AG
	GG	AG	GG	GG	AG	AG	GG	GG
	AG	AG	GG	GG	GG	GG	GG	
131126531								
8940	rs1025934		17	AC	AC	AC	CC	AC
	AC	AC	AC	AC	AA	AC	AC	CC
	AC	AC	AC	AC	CC	AC	AA	AC
	AA	AA	AC	AC	AC	AA	AA	AC
	AA	AC	CC	AC	AA	AA	AC	
74905595								
8941	rs1025935		12	GG	GG	GG	GG	GG
	GG	GG	GG	AG	GG	AG	AG	AG
	AG	AG	GG	AG	AG	GG	GG	GG
	GG	GG	GG	GG	AG	GG	GG	GG
	GG	GG	AG	GG	AG	GG	AA	
62520165								
8942	rs10259381		7	AA	AA	AA	AG	AA
	AA	AG	AA	AA	AA	AG	AG	AA
	AA	AA	AA	AA	AA	AA	AA	AA
	AA	AG	AG	AA	AA	AG	AA	AA
	AG	AA	AA	AA	AG	AA	AG	
88410695								
8943	rs10259435		7	GG	GG	GG	GG	GG
	GG	GG	GG	GG	GG	GG	GG	GG
	AG	AG	GG	GG	GG	GG	GG	GG
	GG	GG	GG	GG	GG	GG	GG	GG
	GG	GG	GG	GG	GG	GG	GG	
4749527								
8944	rs10259462		7	AA	AA	AA	AA	AA
	AA	AA	AG	AG	AG	AA	AA	AA
	AA	AA	AA	AA	AA	AA	AA	AA
	AA	AA	AA	AA	AA	AA	AA	AA
	AG	AA	AA	AA	AA	AA	AA	
130706995								
8945	rs10259485		7	GG	GG	GG	GG	GG
	GG	GG	GG	GG	GG	GG	GG	GG
	GG	GG	GG	GG	GG	GG	GG	GG
	GG	GG	GG	GG	GG	GG	GG	GG
	GG	GG	GG	GG	GG	GG	GG	
151110581								
8946	rs10259568		7	GG	GG	GG	GG	GG
	GG	GG	GG	GG	GG	GG	GG	GG
	GG	GG	GG	GG	GG	GG	GG	GG

GG GG GG GG GG GG GG GG
GG GG GG GG GG GG GG
154836444
8947 rs10259603 7 AG AG AA AG AA
AG AA AG AG AG AG AA AG
GG AA AG AG AG AG AG AG
AG AG AG AG AG AG AG AG
GG AG AA AG AG GG AA
101653744
8948 rs10259665 7 AG GG AG AG GG
AG AG AA AA AG AG AG AA
AA AG GG GG GG GG GG AG
AG AG AG AG GG GG GG GG
GG AG AG AG GG AG GG
79061837
8949 rs10259679 7 AA AA AA AA AA
AA AA AA AA AA AA AA AC
AA AA AA AA AA AA AA AC
AA AA AA AA CC AC CC CC
AA AC AA AC AA AA AA
33774440
8950 rs10259709 7 GG GG GG GG GG
GG GG GG GG GG GG GG GG
GG GG GG GG GG GG GG GG
GG GG GG GG GG GG GG GG
GG GG GG GG GG GG GG
65705424
8951 rs10259734 7 GG GG GG AG AG
GG AA GG AG GG GG GG GG
GG GG GG AG GG AG GG AG
GG GG GG GG GG GG GG GG
GG GG GG GG AG AG AG
55718401
8952 rs10259755 7 AC CC AC AA AA
AA AA AA AA AA AA AA AA
AC AC CC CC AC AC AA CC
AC AA AC AA AA AA AA AA
AC AC CC CC CC AC CC
38897012
8953 rs10259757 7 CC CC CC AC AA
AC AA AA AA AC AC AC AA
AA AA AA AA AA AC AA AC
AC AC AA AC AC AA AA AC
CC AA AA AA AA AA AA
90854065
8954 rs10259772 7 AA AA AA AA AA
AA AA AA AA AA AA AA AA
AA AA AA AA AA AA AA AA
AA AA AA AA AA AA AA AA
AA AA AA AA AA AA AA

65337133
8955 rs10259781 7 AG GG GG GG GG
GG GG GG GG GG GG GG GG
GG GG AG GG GG GG GG GG
GG GG GG GG GG GG GG GG
GG GG GG GG GG GG GG
152867025
8956 rs10259786 7 AG AA AG AA AG
AA AG AA AA AA GG AG AG
AG AG AG AG AG AG AA AA
AA AG AG AA GG AA AG AG
AA AA AG AG AA AG AG
39444551
8957 rs10259797 7 CC CC CC CC AC
CC AC CC AC AC AC CC CC
CC CC CC AC CC CC CC CC
AC CC AC CC CC CC CC CC
AC AC CC CC CC CC AC
4667201
8958 rs10259821 7 GG GG AG AA AA
AG AG AG GG GG AG AG AA
AG GG AG AA AA AG AA AG
AA AG AA AA AG GG AG AG
AG GG GG GG AG GG AG
151645934
8959 rs10259858 7 GG AG GG AG AG
AG AG GG AG AG AG AA AG
AG AG GG AG GG AA GG AG
AG AG AA AA AG AA AA AG
AG GG AG GG AA AG AG
64360891
8960 rs10259929 7 AA AA AA AA AA
AA AA AA AG AG AA AA AA
AA AA AA AA AA AG AG AG
AA AA AA AA AA AG AA AA
AG AA AA AA AA AG AA
4321571
8961 rs10259939 7 AA AA AA AA AA
AA AA AA AA AA AA AA AA
AA AA AA AA AA AA AA AA
AG AG AG AG AA AA AA AA
AA AA AA AA AA AA AA
52774983
8962 rs10260014 7 GG GG GG GG GG
GG GG GG GG GG GG GG GG
GG GG GG GG GG GG GG GG
GG GG GG GG GG GG GG GG
GG GG GG GG GG GG GG
114262059
8963 rs10260028 7 AA AA AA AA AA

AA AA AA AA AA AA AA AA
AA AA AA AA AA AA AA AA
AA AA AA AA AA AA AA AA
AA AA AA AA AA AA AA
69547244
8964 rs10260037 7 GG GG GG AG AG
GG AG GG GG GG GG GG GG
GG GG GG AG AG GG GG GG
GG AG GG GG GG AG AG AG
AG GG GG GG GG GG GG
127507991
8965 rs10260052 7 AA AA AA AA AA
AA AA AA AA AA AA AA AA
AG AA AA AA AA AA AA AA
AA AA AA AA AA AA AA AA
AG AA AA AA AA AA AA
7254279
8966 rs1026006 5 GG AG GG GG GG
GG GG AG AA AG GG GG AG
GG GG AG AA AG GG AG GG
GG GG GG GG AG GG AG AG
AG GG AG GG AG AA AG
114423353
8967 rs10260062 7 CC CC CC CC CC
CC CC CC CC CC CC CC CC
CC CC CC CC CC CC CC CC
CC CC CC CC CC CC CC CC
CC CC CC CC CC CC CC
7081064
8968 rs10260065 7 AG AG AG AA AA
AA AA AA AA AA GG AG AG
AA AA AG AA AA AA AA AA
AA AA AA AA AA AA AA AA
AA AG AA AA AA AA AG
49859810
8969 rs1026009 5 AG AG AG AG AG
AG AA AG AG AG AG AG GG
AG AG GG AG AG AG AG AG
AA AA AA AA AA AG AA AG
AG AG AA AG AG GG AG
124257374
8970 rs10260111 7 GG AG GG GG AG
GG AG GG GG GG AG AG AG
AG GG GG GG GG GG AG AG
AG AG AG AG GG GG GG GG
GG GG AG GG AG GG AG
22816495
8971 rs10260159 7 AA AA AA AA AA
AA AA AG AG AG AA AA AA
AA AA AG GG GG AA AA AA

AA AA AA AA AA AA AA AA
AA AA AG AG AG AG AA
123441559
8972 rs10260162 7 AC AC AC CC CC
CC CC AC AC AC AA CC AC
AC CC AC AC AC CC CC CC
CC CC CC CC CC AC CC CC
CC CC CC CC AC CC AA
12793361
8973 rs10260200 7 CC AC CC AC AC
AA CC AA AA AC CC AC AC
CC CC AA AA AA CC CC CC
AC AC CC CC AC AA AA AC
AC AA AC AC CC CC CC
70525507
8974 rs1026021 11 GG AG AA AA AA
AA AG AA AA AA AA AG AG
AG AG AG AG GG AA AA AA
AA AA AA AA AG AA AG AA
AG GG AA AG AA AA AA
8564778
8975 rs10260278 7 AA AG AA AG AG
AG AG AG AG AG AG AA AG
GG AG AG AA AA GG AG GG
GG AG AG GG AG AG AG GG
GG AG AG AA AG AA AG
21690187
8976 rs1026029 2 AG AA AG AA AA
AA AA AA AA AA AG AG AA
AA AA AA AA AA AG AA AG
AA AA AA AA AA AA AA AA
AA AA AA AA AA AA AA
177065248
8977 rs1026031 2 AA AA AA AG GG
AG GG AA AA AA AA AA AA
AA AA AA AA AA AG AA AA
AA AA AA AA AG AA AA AA
AA AA AG AA AG AA AG
177065030
8978 rs10260350 7 AG GG AG AA AG
AG AG AA AA AA GG GG GG
GG GG AA AA AA AA AG AG
AG AG AA AG AG GG GG AG
AG AG AG AA AG AG GG
13701869
8979 rs10260381 7 GG GG GG GG GG
GG GG GG GG GG AG AG AG
AG GG GG GG GG GG GG GG
GG GG GG GG GG GG GG GG
GG GG GG GG GG GG GG

24381949
8980 rs10260426 7 CC AC AC AA AC
AA AC AA AA AC AC AA AC
AC AC AC AC AC CC AC AC
AA AA AA AA AA AC AA AA
AC CC AC AC CC AC AC
65736042
8981 rs10260430 7 AA AA AA AA AA
AA AA AA AA AA AA AA AA
AA AA AA AA AA AA AA AA
AA AA AA AA AA AA AA AA
AA AA AA AA AA AA AA
93637358
8982 rs10260443 7 AG AG AA AG AG
GG AA GG GG GG GG GG GG
GG AG GG AG AG AG AG GG
AA AG AG AA AG GG GG GG
AG AA GG AG AG GG AG
126639525
8983 rs10260451 7 GG GG GG GG GG
GG GG GG GG GG GG GG GG
AG AG GG GG GG GG AG GG
GG GG GG GG GG GG GG GG
GG GG GG GG GG GG GG
143208225
8984 rs10260526 7 GG GG GG AG GG
GG AG GG GG GG GG GG GG
GG GG GG GG GG GG GG GG
GG GG GG GG GG AG GG GG
GG AG GG GG GG GG AG
41249901
8985 rs10260565 7 AG GG AG AA AG
AG AA AA AG AA AG AG AA
AG GG AA AG AG AG AA AG
AG AG AG AG GG AG AG AG
AG AG AA AG AG AG AG
142194668
8986 rs10260614 7 AG AA AG AA AA
AA AA AA AA AA AA AA AA
AA AA AA AA AA AA AA AA
AA AA AA AA AA AA AA AA
AG AG AA AA AG AG AA
108821664
8987 rs10260637 7 AG GG GG GG GG
GG AG GG GG GG GG GG GG
GG GG GG GG GG GG GG GG
GG GG GG GG GG GG GG GG
GG GG GG GG GG GG GG
137516329
8988 rs10260650 7 GG GG GG GG GG

	GG	GG	GG	GG	GG	GG	GG	GG
	GG	GG	GG	GG	GG	GG	GG	GG
	GG	GG	GG	GG	GG	GG	GG	GG
	GG	GG	GG	GG	GG	GG	GG	
99767269								
8989	rs1026085		12	AG	AG	GG	AG	AG
	AG	AA	AG	AG	AG	AA	AA	AA
	AG	AA	AG	AG	AG	AA	AA	AG
	GG	AG	AG	AG	AG	AG	AA	AA
	AG	AA	AA	AA	AA	AA	AG	
86355735								
8990	rs10260896		7	GG	GG	GG	GG	GG
	GG	GG	GG	GG	GG	GG	GG	GG
	GG	GG	GG	GG	GG	GG	GG	GG
	GG	GG	GG	GG	GG	GG	GG	GG
	GG	GG	GG	GG	GG	GG	GG	
99137274								
8991	rs10260900		7	AG	AG	GG	GG	AG
	AG	GG	AG	AG	AG	GG	AG	GG
	AG	GG	AG	AG	AA	AG	AG	AG
	GG	AG	AG	AG	GG	AG	GG	GG
	AG	AG	AG	AG	AG	AG	AG	
4251135								
8992	rs10260920		7	AA	AG	GG	GG	AG
	AG	AG	GG	GG	GG	GG	GG	GG
	GG	GG	AG	AG	AG	AG	GG	AA
	AG	AG	AG	AG	GG	AG	AG	AG
	AG	AG	AA	AG	GG	AG	GG	
130065429								
8993	rs10260974		7	AG	AA	AG	AG	AG
	AG	AG	AG	AG	AG	AG	GG	AG
	AG	GG	GG	AG	AG	AA	GG	AG
	AA	AA	AG	AA	AG	GG	AG	AG
	AG	GG	GG	GG	AG	GG	AA	
105228825								
8994	rs10260986		7	GG	AG	GG	GG	GG
	AG	GG	GG	GG	GG	GG	GG	GG
	GG	GG	GG	AG	AG	GG	GG	GG
	GG	AG	GG	GG	GG	GG	GG	GG
	GG	AG	AG	AG	AG	AA	GG	
156524010								
8995	rs10261051		7	AG	AG	AA	GG	GG
	GG	GG	AG	AG	AG	AA	AA	AA
	AA	AA	AG	AG	AG	AG	AG	AA
	GG	AG	AG	GG	AG	GG	AG	AG
	AA	AG	GG	GG	AA	AA	AG	
112633232								
8996	rs10261109		7	AA	AA	AA	AA	AA
	AA	AA	AA	AA	AA	AA	AA	AA
	AA	AA	AA	AA	AA	AA	AA	AA

AA AA AA AA AA AA AA AA
AA AA AC AC AA AA AA
75317046
8997 rs10261122 7 GG AG GG GG GG
GG GG GG GG GG GG GG GG
GG GG GG GG GG GG GG GG
GG GG GG GG GG GG GG GG
GG GG GG GG GG GG GG
153913520
8998 rs10261178 7 AG AG AA AG GG
GG AG AG AA AG AA AA AA
AG AG AG AA AG AG GG AG
GG AG AA AG AG AG AG GG
GG AA AG AG GG AG GG
53715983
8999 rs10261193 7 GG GG GG AG AG
GG AG GG GG GG GG GG GG
GG GG GG GG GG GG GG GG
GG GG GG GG GG GG GG GG
GG GG GG GG GG GG GG
121246641
9000 rs10261257 7 AG AA AA AA AA
AA AA AA AA AA AA AA AA
AA AA AA AG AG AG AG AG
AG AA AG AG AG AA AG AG
AG AG AG AG AG AA AG
141091477
9001 rs10261309 7 AG AG GG AA AA
AA AA AG AG AA AA AG AG
AA AA AA AA AA AA AA AG
AG AA AA AG AA AA AA AA
AA AA AG AG AA AA AA
51718636
9002 rs1026134 11 AA AA AA AG AA
AG AA AG AG AG AA AA AA
AA AA AA AA AA AA AA AA
AA AA AA AA AA AA AA AA
AA AA AG AG AA AA AG
132058818
9003 rs10261373 7 AA AA AA AA AA
AA AA AA AA AA AA AA AA
AA AA AA AA AA AA AA AA
AA AA AA AA AA AA AA AA
AA AA AA AA AA AA AA
151369864
9004 rs10261379 7 AA AA AG AG AG
AA GG AA AG AA AA AG AG
AA AA AA AA AA AG GG AA
AA AG AG AA AA AG AG AG
AA AA AA AA AA AG AA

70935693
9005 rs10261398 7 AA AG AG GG AG
GG AG GG GG AG AG GG AG
AG AG AG AG AG AA AG AG
GG GG GG GG GG AG GG GG
AG AA AG AG AA AG AA
65750164
9006 rs10261430 7 GG GG GG GG GG
GG GG GG GG GG GG GG GG
GG GG GG GG GG GG GG GG
GG GG GG GG GG GG GG GG
GG GG GG GG GG GG GG
6604052
9007 rs10261471 7 GG GG GG GG GG
GG GG GG GG GG GG GG GG
GG GG GG GG GG GG GG GG
GG GG GG GG GG GG GG GG
GG GG GG GG GG GG GG
64832701
9008 rs10261478 7 AG AG AG GG AG
AG GG GG AG AG GG GG GG
GG GG GG GG GG AG AG GG
AG GG AG GG AG AA AG AG
GG AG GG AG GG AG GG
40388695
9009 rs10261505 7 AC AC AA CC CC
CC CC AA AC AC AC AC AA
AC CC AC AC AC AC AC AC
CC AC CC CC AA AA AA AA
AC CC AA AC AA AA AC
154498135
9010 rs10261522 7 AG AA AG AA AA
AA AA AA AA AA AG AG AA
AA GG AA AG AG AG AG AG
AG GG GG AG AA AG AA AA
AG AG AG AG AG AG AG
85335684
9011 rs10261558 7 AG AG AA AG AG
AG AG AA AA AA AA AA AA
AG AA AA AA AA AG AA AA
AA AA AA AA AA AA AA AA
AA AA AA AA AA AG AA
96284589
9012 rs1026158 13 AG AA AA AA AA
AG AA AG AG AG GG GG GG
AG AA AG AG AA GG AG GG
AA AG AG AA GG GG GG GG
AG AA AG AG AG AG AG
103400083
9013 rs10261581 7 AA AA AA AG AG

AA GG AA AA AA AG AG AG
AG AG AG AG AA AG AG AG
AA AG AG AG GG AA AG AG
AA AG AA AA AA AA AA
4457707
9014 rs10261781 7 GG GG GG GG GG
GG GG GG GG GG GG GG GG
GG GG GG GG GG GG GG GG
GG GG GG GG GG GG GG GG
GG GG GG GG GG GG GG
98718386
9015 rs10261824
7 -- -- -- -- -- -- -- --
-- -- -- -- -- -- -- --
-- -- -- -- -- -- -- --
-- -- -- -- -- -- -- --
-- -- -- -- 7053611
9016 rs10261869 7 GG GG GG AG GG
GG AG GG GG GG GG GG GG
GG GG GG GG GG GG GG GG
GG GG GG GG GG AG GG GG
GG GG GG GG GG GG AG
41214917
9017 rs1026194 7 AA AG AG AG AG
GG AG GG GG GG GG GG GG
GG AG AA AG AG AG AA GG
GG AG GG GG GG AA AG AG
AG AA AA AA GG AG GG
131913645
9018 rs10261983 7 AA AG AG GG GG
GG GG GG GG AG GG AG AG
GG GG AG AG AG AA AG AG
GG AG GG AG AG AA AA AA
GG AG AG GG AG GG AA
37885121
9019 rs10262028 7 CC CC CC AA CC
AC AC AA AA AC AC AC CC
CC CC CC AC AC CC CC CC
CC CC CC CC AC AC AC AA
AC CC CC CC CC AC AC
85867258
9020 rs1026208 2 GG GG GG GG GG
GG GG GG GG GG GG GG GG
GG GG GG GG GG GG GG GG
GG GG AG GG GG AG AG GG
GG GG AG AG GG GG GG
2723155
9021 rs10262175 7 CC CC CC CC CC
CC CC CC CC CC AC CC AC
AC CC CC CC CC CC CC CC

CC CC CC CC CC CC CC CC
AC AC CC AC CC CC AC
76958980
9022 rs10262180 7 GG GG GG GG GG
GG GG GG GG GG GG GG GG
GG AG GG GG GG GG GG AG
GG GG GG GG GG GG GG GG
GG AG AA AG GG GG GG
78182068
9023 rs10262191 7 CC CC CC CC CC
CC CC AC CC AA CC CC CC
CC CC CC CC CC CC AC CC
CC CC CC CC CC CC CC CC
AC CC CC CC CC CC CC
156804510
9024 rs10262192 7 GG AG AG AG AA
AG AG AG AG AG AG GG AG
AG GG AG GG GG AG AG AG
GG AG AG GG AG GG GG GG
GG AG AA AG GG AG GG
114091753
9025 rs10262194 7 AA AA AA AA AG
AG AA AA AA AA AG AG GG
AA AG AA AA AA AG AG AG
AG AA AG AA AG AG AG AG
AG AA AA AA AA AA AA
6274585
9026 rs1026220 2 AG AA AA AG AA
AA AG AG AG AG GG AG AG
AG AG GG GG GG GG GG GG
AG AA GG GG AG GG AG GG
AA GG AG AG GG GG AG
105504322
9027 rs1026228 8 AA AA AA AG GG
GG AG GG GG GG GG AG AG
GG GG GG AG GG AA AG AG
AG AG AG AG AG AA AG AA
AG AG GG GG AA AA AG
102007701
9028 rs10262302 7 AA AA AA AG AA
AA AG AA AA AA AA AA AA
AA AA AA AA AG AA AA AA
AA AA AA AA AA AA AA AA
AA AA AA AA AA AA AA
52110570
9029 rs10262333 7 AA AA AA AA AA
AA AA AA AA AA AA AA AA
AA AA AA AA AA AA AA AA
AA AA AA AA AA AA AA AA
AA AA AA AA AA AA AA

5047611
9030 rs10262356 7 AG AG AG AA AA
AA AA AA AA AA AG AG AG
AA AA AA AA AA AA AA AA
AA AG AA AG AA AA AA AA
AA GG AG GG AA AA AA
12581042
9031 rs10262667 7 AG AG AG GG GG
GG GG GG GG GG AG AA AA
AG GG AG GG GG GG GG GG
GG AG AG AG GG GG GG GG
GG GG AG AG GG GG GG
12385391
9032 rs10262677 7 GG GG GG GG GG
GG GG GG GG GG GG GG GG
GG GG GG GG GG GG GG GG
GG GG GG GG GG GG GG GG
GG GG GG GG GG GG GG
3082192
9033 rs10262736 7 GG GG GG GG GG
GG GG GG GG GG GG GG GG
GG GG GG GG GG GG GG GG
GG GG GG GG GG GG GG GG
GG GG GG GG GG GG GG
152637384
9034 rs10262791 7 AA AA AA AG AG
AG AG GG AG GG AA AG AG
AA AA AG AA AA AA AG AG
AG AA AA AG AA AA AA AA
AG AA AG AG AA AA AA
36335759
9035 rs10262803 7 GG GG GG AG GG
AG GG GG GG GG GG AG AG
GG AG AG GG GG GG GG GG
AG GG AG AG GG GG GG GG
GG GG GG GG GG GG GG
103489028
9036 rs10262813 7 AA AA AA AA AA
AA AA AA AA AA AA AG AG
AA AA AG AA AA AG AG AA
AG AA AG AA AA AA AA AA
AA AG AG GG AA AA AG
15114140
9037 rs1026290 2 AG AG GG AA AG
AG AG AA AA AA GG GG AG
AG AG AG AG GG GG AG GG
AG AG AG AG AG AG AG AG
AG AA AG AG GG GG GG
173841435
9038 rs10262915 7 AA AA AA AA AA

AA AA AG AG AA AA AA AA
AA AA AG AA AA AA AA AA
AA AA AG AA AA AA AA AA
AA AG AA AA AG AG AA
94000473
9039 rs10262917 7 GG GG GG GG GG
GG GG GG GG GG GG GG GG
GG GG GG GG GG GG GG GG
GG GG GG GG GG GG GG GG
GG GG GG GG GG GG GG
149518272
9040 rs10262929 7 GG GG GG GG GG
GG GG GG GG GG GG GG GG
GG GG GG GG GG GG GG GG
GG GG GG GG GG GG GG GG
GG GG GG GG GG GG GG
16225133
9041 rs10262962 7 GG GG GG GG GG
GG GG GG GG GG GG GG GG
GG GG GG GG GG GG GG GG
GG GG GG GG GG GG GG GG
GG GG GG GG GG GG AG
13441694
9042 rs10262992 7 GG GG GG GG GG
GG GG GG GG GG GG GG GG
GG GG GG GG GG GG GG GG
GG GG GG GG GG GG GG GG
GG GG GG GG GG GG GG
15978602
9043 rs10262995 7 AG GG GG GG GG
GG GG AG GG AG GG GG GG
GG GG GG GG GG GG GG GG
AG GG GG AG GG GG GG GG
GG GG GG GG GG GG GG
33550041
9044 rs10263007 7 CC CC CC CC CC
CC CC AC AC AC CC CC CC
CC CC CC CC CC CC CC CC
CC CC CC CC CC CC CC CC
CC CC CC CC AC CC AA
101035356
9045 rs1026301 2 AC CC AC AC AC
AC AC CC CC AC AC AC AC
AC AC AC AA AC AC AC CC
AA AA AA AA AC AC AC AC
CC AC AC AA AC AA CC
35210500
9046 rs10263021 7 GG GG GG GG GG
GG AG GG AG AG AG AG GG
GG GG GG GG GG AG GG GG

AG AG AG AG GG AG AG AG
GG GG GG GG AG AA GG
147925492
9047 rs10263050 7 GG GG GG AG GG
AG GG GG GG GG AG AG GG
GG GG GG AG AG GG AG GG
GG GG GG GG GG GG GG GG
GG AG GG AG GG GG GG
106593608
9048 rs10263063 7 AG AG AG GG GG
GG AG AG AA AA GG GG GG
AG AA AG AG AG AA AA AA
AG AA AG AG AG AA AG AG
AG AA AA AA GG AG AG
154186526
9049 rs10263072 7 AG GG GG GG GG
GG GG GG GG AG GG AG AG
AG AG GG GG GG AG AA GG
GG GG GG GG AG GG GG GG
GG GG GG GG GG GG GG
37144095
9050 rs10263077 7 AG AA AA GG AG
AG GG GG AG GG GG AG AG
GG AG GG GG GG GG AG GG
AG GG GG AG GG AG GG GG
AA AG AG AG GG AG GG
70295629
9051 rs10263175 7 GG AG GG GG GG
GG GG GG GG GG AG AG GG
GG GG GG GG GG GG GG GG
GG GG GG GG GG AG GG GG
GG AG GG AG GG GG GG
16043897
9052 rs10263178 7 GG GG GG GG GG
GG GG GG GG GG GG GG GG
GG GG GG GG GG GG GG GG
GG GG GG GG GG GG GG GG
GG GG GG GG GG GG GG
56278472
9053 rs1026318 11 GG GG GG GG GG
GG GG GG GG GG GG GG GG
GG GG GG GG GG GG GG GG
GG GG GG GG GG GG GG GG
GG GG GG GG GG GG GG
59204920
9054 rs10263196 7 GG GG AG GG GG
GG AG GG GG GG AG AA AG
AG AG AG AG AG AG AG GG
AG AG AG AG GG GG GG GG
AG AG GG GG AG AA AG

132956182
9055 rs1026327 X AA AA AA AA AA
AA AA AA AA AA AA AA AA
AA AA AA AA AA AA AA AA
AA AA AA AG AA AA AA AA
AA AG AA GG AA AA AA
5117394
9056 rs10263303 7 AA AA AA AG AG
AA AG GG GG GG AG AG GG
GG AG AG GG GG AG AA AA
AA AA AA AA AA AG AA AA
GG AG AA AA AG AG GG
157314762
9057 rs10263338 7 AG AG GG AG GG
AG GG AG AG AG GG GG GG
AG AG GG GG AG AG AG GG
AA AG AA AG AG GG AG AG
AG GG AA AG GG GG GG
3676463
9058 rs10263341 7 AG AG GG AG AG
GG AA GG GG GG GG AG AG
AG AG AA AA AA AG AA GG
AG GG GG AG GG AA AG AG
AG AG GG AG GG GG AG
107545799
9059 rs10263424 7 AA AA AA AA AA
AA AA AA AA AA AA AA AA
AA AA AA AA AA AA AA AA
AA AA AA AA AG AA AG AA
AG AG AA AG AG AA AG
17562902
9060 rs10263442 7 AA AA AA AA AA
AA AA AA AA AA AA AA AA
AA AA AA AA AA AA AA AA
AA AA AA AA AG AG AG AG
AG AA AA AA AA AA AA
9791575
9061 rs10263444 7 GG GG GG GG GG
GG GG AG AG AG GG GG GG
GG GG GG GG GG GG AG GG
GG GG GG GG GG GG GG GG
GG GG GG GG GG GG GG
111323799
9062 rs10263466 7 AG AA AG AG GG
AG AG GG GG GG AA AA AA
AA AA AA AA AA AA AA AA
AG AG AA AG AA AA AA AA
AA AA AA AA AA AA AA
122595412
9063 rs10263482 7 CC CC CC CC CC

CC CC CC CC CC CC CC CC
CC CC CC CC CC CC CC CC
CC CC CC CC CC CC CC CC
CC CC CC CC CC CC CC
156799578
9064 rs10263532 7 GG GG GG GG GG
GG GG GG GG GG GG GG GG
GG GG GG GG GG GG GG GG
GG GG GG GG GG GG GG GG
GG GG GG GG GG GG GG
2324344
9065 rs10263533 7 GG GG GG GG GG
GG GG GG GG GG GG GG GG
GG GG GG GG GG GG GG GG
GG GG GG GG GG GG GG GG
GG GG GG GG GG GG GG
63742126
9066 rs1026358 15 AG AG AA AA AA
AA AA AA AA AA AA AA AA
AA AA AG AG AA AG AG AG
AG AG AG AG AA AA AA AA
AA AA AG AG AA AA AA
27490200
9067 rs10263615 7 GG GG GG GG GG
GG GG GG GG GG GG GG GG
GG GG GG GG GG GG GG GG
GG GG GG GG GG GG GG GG
GG GG GG GG GG GG GG
155204766
9068 rs10263656 7 AG AA AG AA AG
AG AG AG AA GG AA AA AA
AG AG AG GG GG AA AA AA
AG AA AA AG GG AG GG GG
AG AG AA AG AG AG AA
67227790
9069 rs10263657 7 GG GG AG AA AA
AA AA AA AA AA AG AG AA
AG GG AA AA AA AA AA AG
AA AA AA AA AA AG AG AG
AA AA AA AA AA AA AA
155366240
9070 rs1026371 5 GG GG GG AG AG
AG GG GG GG GG GG GG GG
GG GG GG GG GG GG GG GG
GG GG GG GG AG GG GG AG
GG GG AG AG AG GG AG
57226653
9071 rs10263716 7 GG GG GG AG AG
AA GG GG GG GG GG AG AG
AG GG GG GG GG AG AG AG

GG AG GG GG GG AG GG GG
GG AG GG GG GG AG AG
134301397
9072 rs1026378 8 AG GG AG GG GG
GG GG GG GG GG GG GG GG
GG GG GG GG GG GG GG GG
GG GG GG GG GG GG GG GG
GG GG GG GG GG AG GG
2662135
9073 rs10263803 7 AC CC AC CC AC
AC CC AC CC AC AC AA AC
CC AC AC AC CC AC CC AA
CC AC AC CC CC AC AC AC
AC AA CC AC CC CC CC
20437823
9074 rs10263828 7 AA AA AA AA AG
AA AG AA AA AA AA AA AA
AA AA AA AA AA AA AA AA
AA AA AA AA AA AA AA AA
AA AA AA AA AA AA AA
103830377
9075 rs10263829 7 CC CC CC CC CC
CC CC CC CC CC CC CC CC
CC CC CC CC CC CC CC CC
CC CC CC CC CC CC CC CC
CC CC CC CC CC CC CC
63398863
9076 rs10263831 7 CC CC CC CC CC
CC CC CC CC CC CC CC CC
CC CC CC CC CC CC CC CC
CC CC CC CC CC CC CC CC
CC CC CC CC CC CC CC
15713304
9077 rs10263846 7 AG AA AG AG AG
AA GG GG GG GG AG AG GG
GG AG GG GG GG AG GG AG
GG AG GG GG GG GG GG GG
GG GG GG GG AA AG AG
157990941
9078 rs10263926 7 GG GG GG AG GG
AG GG GG GG GG AG GG AG
AG AG GG AG GG AA AG AG
AG GG GG GG GG AG GG GG
AG GG AA AG GG GG --
10679384
9079 rs1026394 4 AG AG AG AA AA
AA AA AA AA AA AG GG GG
AG AA AA AA AA AG GG AA
AA AA AA AA AG AG AG AG
AG AA AA AA AG AG AA

80108181							
9080	rs1026395	12	AG	AG	AG	GG	GG
	GG	GG	GG	GG	GG	GG	GG	GG
	GG	GG	GG	GG	GG	GG	GG	GG
	GG	GG	GG	GG	GG	GG	GG	GG
	GG	GG	GG	GG	GG	GG	GG	
123377821							
9081	rs10263964	7	AG	AA	AA	AG	AG
	GG	AG	AG	AG	AG	AG	AG	GG
	GG	AG	AG	AA	AA	GG	AG	GG
	GG	GG	GG	GG	AG	GG	AG	AG
	GG	GG	AA	AG	AG	GG	AG	
147679621							
9082	rs10263979	7	CC	CC	CC	CC	AC
	AC	CC	CC	CC	AC	CC	CC	CC
	CC	AC	AC	CC	CC	CC	AA	AC
	CC	CC	CC	CC	AC	AC	AA	AA
	AC	AA	CC	AC	AC	AA	AC	
97496179							
9083	rs10264029	7	GG	GG	GG	GG	GG
	GG	GG	GG	GG	GG	GG	GG	GG
	GG	GG	GG	GG	GG	GG	GG	GG
	GG	GG	GG	GG	GG	GG	GG	GG
	GG	GG	GG	GG	GG	GG	GG	
73018173							
9084	rs10264031	7	AG	AG	AG	AA	AA
	AA	AA	AG	AG	AG	AG	AG	AA
	AA	AA	AG	AA	GG	GG	AG	AG
	AA	AG	AG	AA	AG	AG	GG	AG
	AA	GG	AA	AG	AA	AA	AA	
19254640							
9085	rs10264045	7	AA	AA	AA	AA	AA
	AA	AA	AA	AA	AA	AA	AA	AA
	AA	AA	AA	AA	AA	AA	AA	AA
	AA	AA	AA	AA	AA	AA	AA	AA
	AA	AC	AA	AC	AA	AA	AA	
15760654							
9086	rs10264143						
7	--	--	--	--	--	--	--	--
	--	--	--	--	--	--	--	--
	--	--	--	--	--	--	--	--
	--	--	--	--	--	--	--	--
	--	--	--	--	151907250			
9087	rs10264162	7	CC	CC	CC	AC	CC
	CC	AC	CC	CC	CC	CC	AC	AC
	AC	AC	CC	CC	CC	AC	CC	AC
	AC	CC	CC	AC	AC	AC	AC	AA
	AC	CC	AC	CC	CC	CC	CC	
49620107							
9088	rs10264222	7	AA	AA	AA	AC	AC

	AC	AA	AA	AA	AA	AA	AA	AA
	AA	AA	AA	AA	AA	AA	AA	AA
	CC	AA	CC	CC	AC	AC	AC	AC
	AA	AA	AC	AA	CC	AC	AC	
155212392								
9089	rs10264247		7	AA	AA	AA	AA	AA
	AA	AA	AA	AA	AA	AA	AA	AA
	AA	AA	AA	AA	AA	AA	AA	AA
	AA	AA	AA	AA	AA	AA	AA	AA
	AA	AA	AA	AA	AA	AA	AA	
64228122								
9090	rs10264281		7	AA	AA	AA	AA	AG
	AA	AG	GG	GG	AG	GG	GG	AG
	AG	AG	AG	GG	AG	AG	AG	AG
	AA	AG	AA	AG	AG	AG	AG	AG
	AA	GG	AG	GG	AG	GG	AA	
2370412								
9091	rs1026442		12	AG	AG	AA	GG	AG
	AG	AG	AA	AA	AA	AA	AG	AA
	AG	AA	AG	AG	AG	AA	AA	AG
	AA	AA	AA	AA	AG	AA	AG	AA
	AA	GG	AG	AG	AA	AA	AG	
10765031								
9092	rs10264462		7	GG	GG	GG	GG	GG
	GG	GG	GG	GG	GG	GG	GG	GG
	GG	GG	GG	GG	GG	GG	GG	GG
	GG	GG	GG	GG	GG	GG	GG	GG
	GG	GG	GG	GG	GG	GG	GG	
5370606								
9093	rs10264499		7	AC	AC	CC	AC	AC
	AC	AC	AC	AC	CC	AA	AA	AA
	AA	AA	AC	CC	CC	AC	AA	AC
	AA	AC	AA	AA	AA	AC	AC	AC
	AC	AC	AC	AC	AC	AA	AC	
155355522								
9094	rs10264545							
7	--	--	--	--	--	--	--	--
	--	--	--	--	--	--	--	--
	--	--	--	--	--	--	--	--
	--	--	--	--	--	--	--	--
	--	--	--	--	115566788			
9095	rs10264568		7	AA	AA	AA	AA	AA
	AA	AA	AA	AA	AA	AA	AA	AA
	AA	AA	AG	GG	GG	AG	AA	AA
	AG	AG	AG	AG	AG	AA	AA	AG
	AA	AA	AA	AA	AA	AA	AA	
89682186								
9096	rs10264662		7	AA	AA	AA	AG	AG
	AA	AG	AA	AA	AA	AA	AA	AA
	AG	AG	AA	AA	AA	AG	AA	AA

AG AA AG AG AA AA AA AA
AG AG AA AA AA AA AA
147855874
9097 rs1026469 1 AG GG AG GG AG
AG AG AG AA AA GG GG GG
AG GG GG AG GG AG AG GG
AA GG AG AG AG AG AG AA
AG AG GG AG AG GG AG
85568972
9098 rs10264696 7 AG AG AG AG AG
AA AG AA AA AA AA AG AG
AG AA AA AA AA AA AA AA
AA GG AA AA AA AA AA AA
AG AA AA AA AA AA AA
145048455
9099 rs10264737 7 -- AG AG -- GG
AG AG AG AG AA AA -- --
AA -- AA -- -- AG -- --
-- -- AG AG -- AG -- --
AG AG -- AG -- GG AA
50933370
9100 rs10264760 7 AG AA AA AG AA
AG AA GG GG AG AG AA AG
GG GG AA AA AA AG AG AA
AA AA AA AA GG AA AG AG
AG AA AA AA AA AA AA
75749694
9101 rs10264843 7 AA AA AA AG AG
GG AA AG AA AA AG AA AA
AG AG AG AG AG GG GG AG
AG AG AG AG AG AA AG AG
AA AA AG AG AA AG AA
2724098
9102 rs10264866 7 GG GG GG GG AG
AG GG GG GG GG AG AG GG
GG GG AG GG GG GG GG GG
GG GG GG GG AG GG GG GG
GG AG AG AA AG AG AG
101537526
9103 rs10264872 7 AA AA AA AA AA
AA AA AG AG GG AA AA AA
AA AA AA AA AA AA AA AG
AA AA AA AA AA AA AA AA
AA AA AA AA AA AA AA
15394591
9104 rs10264873 7 AA AA AG AG AG
GG AA AA AA AA AA AG AG
AG AG AA AA AA AA AA AA
AG AA AA AG AA AG AG AG
AA AG AA AG AA AA AG

66404770
9105 rs10264890 7 CC AC CC CC CC
CC CC CC CC CC AC AA AC
CC CC AC AC AC AC AC AC
CC AC CC AC AC CC AC AC
CC AC AC AC AA AA AC
7719987
9106 rs10264996 7 GG GG GG GG GG
GG GG GG GG GG GG GG GG
GG GG GG AG AG GG GG GG
GG GG GG GG AG GG AG AG
GG GG GG GG GG GG GG
141084969
9107 rs10265027
7 -- -- -- -- -- -- -- --
-- -- -- -- -- -- -- --
-- -- -- -- -- -- -- --
-- -- -- -- -- -- -- --
-- -- -- -- 3135770
9108 rs1026504 14 AA AA AA AC AA
AC AA AC AC AC AC CC AC
CC CC AC AA AA AA AA AA
AC CC AC AC AA AC AC AA
AC AA CC AC AC CC AA
98830005
9109 rs10265057 7 AA AA AA AA AA
AA AA AA AA AA AA AA AA
AA AA AA AA AA AA AA AA
AA AA AA AA AA AA AA AA
AA AA AA AA AA AA AA
47275737
9110 rs10265064 7 AC AA AC AC AC
AA CC AC AC AC CC AC AC
AC AC AA AA AA AC AC AA
AC AC AC AC AA AA AA AA
CC AC AC CC AA AA AC
137753520
9111 rs10265101 7 AA AA AA AG GG
GG AG AA AA AA AG GG AG
AG AG AA AA AA AA AA AG
AA AG AG AA AG AG AG AG
AA GG AA AG GG GG AG
103353742
9112 rs10265123 7 AG GG GG GG GG
GG GG GG GG GG GG GG GG
GG GG GG GG GG GG GG GG
GG -- GG GG GG GG GG GG
GG GG GG GG GG GG GG
152414618
9113 rs10265128 7 AA AA AA AA AA

	AA	AG	AG	AA	AG	AA	AA	AA
	AA	AA	AA	AA	AA	AG	AG	AA
	AG	AG	AG	AG	AA	AG	AG	AG
	AA	AA	AA	AA	AA	AA	AA	
135211446								
9114	rs10265132		7	AG	AG	AG	AG	GG
	GG	AG	GG	GG	GG	GG	GG	AG
	AG	AG	GG	AG	GG	GG	GG	AG
	GG	AG	AG	AG	AG	GG	AG	AG
	GG	GG	AG	AG	AA	AG	AG	
8637693								
9115	rs10265155		7	AA	AA	AA	AA	AA
	AA	AA	AG	AG	AA	AG	AA	AG
	AG	AG	AA	AA	AA	AA	AA	AA
	AG	AA	AA	AG	AA	AA	AA	AA
	AA	AG	AG	AG	AA	AA	AG	
79078161								
9116	rs10265196		7	GG	GG	GG	AG	GG
	AG	GG	AA	AG	AG	AG	GG	GG
	GG	GG	GG	GG	AG	AG	AG	AG
	GG	AG	GG	AG	AG	AG	AG	AG
	GG	AA	AG	AA	AG	AG	AG	
4282613								
9117	rs10265256		7	AA	AG	GG	AG	AA
	AG	AG	GG	GG	GG	AG	AG	AA
	AA	AG	AG	AA	AA	AG	GG	AG
	GG	AG	GG	GG	AG	AA	AA	AA
	AA	AG	AG	AG	AG	AG	AG	
134729244								
9118	rs1026526		6	AG	AA	AG	AG	AA
	AA	AG	AA	AA	AA	AA	AA	AA
	AA	AA	AG	AA	AG	AA	AA	AA
	AA	AA	AA	AA	AA	AG	AA	AA
	AA	AA	AA	AA	GG	AG	AG	
39587757								
9119	rs10265380		7	AA	AA	AA	AA	GG
	AG	AG	AG	AA	AA	AG	AA	AA
	AA	AA	AG	AA	AA	AA	AA	AA
	GG	AA	AG	AA	AA	AA	AA	AA
	AA	AG	AA	AA	AA	AA	AA	
4553521								
9120	rs10265425		7	AG	AA	AG	AG	GG
	AG	GG	GG	GG	AG	AA	AA	AA
	AG	AA	AA	AA	AA	AA	AA	AA
	AA	AA	AA	AA	AA	AA	AA	AA
	AA	AG	AA	AG	GG	AG	AG	
95421030								
9121	rs10265439		7	AA	AA	AA	AA	AA
	AA	AA	AA	AA	AA	AA	AA	AA
	AA	AA	AA	AA	AA	AA	AA	AA

AA AA AA AA AA AA AA AA
AA AA AA AA AA AA AG
96250511
9122 rs10265554 7 AA AA AA AA AA
AA AA AA AA AG AG AA AG
GG AG AA AA AA AA AA AA
AA AA AA AA AG AA AG AA
AA AA AA AA AA AG AA
68051712
9123 rs10265626 7 CC CC CC AC AC
CC AC CC CC CC CC AC AC
AC CC AC AA AA CC CC AC
AC AC AC AC AC AC AA AA
AC AC CC CC CC CC AC
147175188
9124 rs10265651 7 AG AA AG AA AA
AA AA AA AA AA AG AA AG
AA AA AA AG AG AA AG AA
AA AA AA AA AA AA AA AA
AA AA AA AA AA AA AA
157692304
9125 rs10265670 7 AA AA AA GG GG
GG AG AG AG AG GG AG AG
AG AG AA AA AA AG AA AA
GG AA GG GG GG GG GG GG
AA AA AG AG AA AG AA
150462394
9126 rs10265677 7 AC AC CC AC AC
AA AC AA AA AA AA AA AA
AA AC AC AA AA AC AA AC
AA AA AC AC AA AA AA AA
AC CC AC CC AA AA AA
68286270
9127 rs10265687 7 GG GG GG AG GG
GG AG GG GG GG GG AG AG
AG AG GG GG GG AG GG AG
AG GG GG AG AG AG AG AA
AG AG AA AG GG GG GG
49615332
9128 rs10265714 7 AA AA AA AA AA
AA AA AA AA AA AA AA AA
AA AA AA AA AA AA AA AA
AA AA AA AA AA AA AA AA
AA AA AA AA AA AA AA
40236067
9129 rs10265730 7 AG AG AA AG AG
AA GG GG GG AG AA AG AG
AG AG AG AA AA GG GG AG
GG GG AG AG AA AA AA AA
AA AG AA AG AG AG GG

81723112
9130 rs10265774 7 AG AA AG AA AA
AA AA AG AG AG AA AG AG
AG AA AG AA AA AA AA AA
AA AA AA AA AA AA AA AA
AA AA AA AA AA AA AA
156525513
9131 rs1026580 10 AG AA AA AA AG
AA AG AG AG AA GG AG AG
AG AG AG AG GG AA AA AA
AA AA AG GG AA AG AA AA
AA AA AG AG AG AA AG
13981162
9132 rs10265885 7 AA AA AA AA AA
AA AA AA AA AA AA AA AA
AA AA AG AG AG AA AA AA
AA AA AA AA AA AA AA AA
AA AG AA AG AA AA AA
89341519
9133 rs10265891 7 AG GG GG GG GG
GG GG GG GG GG AA AA AA
AG AG GG AG GG GG GG GG
GG AA AG AG GG AG GG GG
AG GG GG GG GG GG AG
38678273
9134 rs10265924 7 GG GG GG GG GG
GG GG GG GG AG GG AG AG
AG GG AG GG GG GG GG GG
GG GG GG GG GG GG GG GG
GG GG GG GG GG GG GG
156521894
9135 rs10265943 7 AA AA AA AG AA
AA AG AA AA AA AA AA AA
AA AA GG GG GG AA AG AA
AA AG GG AG AA AG AA AA
AA AA AA AA AG GG AA
82187231
9136 rs10265967 7 AA AA AA AG AA
AG AA AA AA AA AA AA AA
AA AA AG AG GG AA GG AG
AG GG AG GG AA AG AG AG
AG AA AG AA AG AA AG
33821645
9137 rs10266018 7 GG AG AG GG GG
GG AG AG AA AG GG GG GG
GG AG GG GG GG AG AG AG
AG AG AG AG AG GG GG GG
GG AG AG AG AG AG AG
134884526
9138 rs10266069 7 AA AG AG AA AA

AA AA AA AA AA AG AA AG
AG GG AG AG AG AA AG AG
AG GG AG AG AA AG AG AG
AG AG AG AA AG AA AG
150349937
9139 rs10266114 7 AA AA AA AA AA
AA AA AG AA AG AG AG AA
AA AG AA AA AA AG AA AA
AA AG AA AA AA AA AA AA
AA AG AG AA AG AG AA
139297145
9140 rs10266126 7 CC AC CC AC AC
AA CC AA AC AA AC AC CC
AC AA CC CC CC CC CC CC
AC AA AC CC CC CC CC CC
CC CC CC CC AC AC CC
69375390
9141 rs10266129 7 AG AG AG AA AA
AG AA AG AA AG AA AA AA
AA AA AA AG AG AA AG AA
AA AA AA AA AA AA AA AA
AA AG AA AA AG AA AG
157032387
9142 rs10266145 7 GG GG GG GG GG
GG GG GG GG GG GG GG GG
GG GG GG GG GG GG GG GG
GG GG GG GG GG GG GG GG
GG GG GG GG GG GG GG
69844679
9143 rs10266166 7 GG GG GG GG GG
GG GG GG GG GG GG GG GG
GG GG GG GG GG GG GG GG
GG GG GG GG GG GG GG GG
GG GG GG GG GG GG GG
66196435
9144 rs10266190 7 AG AG GG GG AG
AG GG GG AG AG AG GG GG
GG GG GG GG GG AG AG AG
GG AG GG AG GG GG GG GG
AG AA GG AG GG AG AG
4580498
9145 rs10266233 7 AA AA AA AA AA
AA AA AA AA AA AA AA AA
AG AG AA AA AA AA AG AA
AA AA AA AA AG AA AA AA
AA GG AA AG AA AA AA
157481050
9146 rs1026628 2 AG AG AG AG AA
AG AA AG GG GG AG AG AG
AG AA GG AG AG AA AG AA

AG AG AA AA AG AA AG AG
AG AA AG AA AG AG GG
196622252
9147 rs10266293 7 AA AA AA AA AA
AA AA AA AA AA AG AG AA
AA AA AA AA AA AA AA AA
AA AA AA AA AA AA AA AA
AA AA AA AA AA AA AA
94733635
9148 rs10266297 7 GG AG AG AG AA
AG AA AG AG AG AG AG AA
AA AG AA AA AA AG AG AG
GG AG AG GG AG GG GG GG
AA AG AA AG AG AA AG
114143407
9149 rs10266303 7 AA AA AA AA AA
AA AA AG AG AG AG AG AA
AA AA AG AA AA AA AA AA
AA AA AA AA AG AA AA AA
AA AA AA AA AA AA AA
122219899
9150 rs10266306
7 -- -- -- -- -- -- -- --
-- -- -- -- -- -- -- --
-- -- -- -- -- -- -- --
-- -- -- -- -- -- -- --
-- -- -- -- 5115684
9151 rs10266317 7 AG GG AG GG GG
GG GG GG GG GG GG GG GG
GG GG AA AG AG GG GG GG
GG GG GG GG GG GG GG GG
GG AA AG AA AG AG AG
98382569
9152 rs10266423 7 AA AA AA AA AA
AA AA AA AA AA AA AA AA
AA AA AA AA AA AA AA AA
AA AA AA AA AA AA AA AA
AA AA AA AA AA AA AA
152729857
9153 rs10266424 7 -- -- -- AG AG
-- AA AG AG AA AG AG AA
AG AG AG AG AG -- AG --
AG AG AG AG GG AA AG AG
AG -- AG -- AA AA AA
91503228
9154 rs1026648 16 AG AG AG AA AA
AA AA GG AG GG AA AA AA
AA AA AA AA AA AA AA AA
AA AA AA AA AA AA AA AA
AA AA AA AA AA AA AA

77511121
9155 rs10266480 7 GG GG GG AA AG
AA AG AA AA AA AG AG AA
AA AA GG GG GG AG AG AA
AG AA AG AG AG GG GG GG
AA GG AA AG AG AG AG
103575689
9156 rs1026652
18 -- -- -- -- -- -- -- --
-- -- -- -- -- -- -- --
-- -- -- -- -- -- -- --
-- -- -- -- -- -- -- --
-- -- -- -- 4332068
9157 rs10266554 7 AC CC AC AA AA
AA AA -- AC CC -- AC AC
AC AC AC AA AA AA AA AA
-- AC AA AC AC AA AC AA
AC AC AA AC AC AC AA
52179114
9158 rs10266558 7 GG GG GG GG GG
GG GG GG GG GG GG GG GG
GG GG GG GG GG GG GG GG
GG GG GG GG GG GG GG GG
GG GG GG GG GG GG GG
1773262
9159 rs10266580 7 AA AA AA AA AA
AA AA AA AA AA AA AA AA
AA AA AA AA AA AA AA AA
AA AA AA AA AA AA AA AA
AA AA AA AA AA AA AA
102049901
9160 rs10266589 7 AA AA AA AA AA
AA AG AG AG AA AA AA AA
AA AA AA AA AA AA AA AA
AA AA AA AA AA AA AA AA
AA AA AA AA AG AG AA
150182338
9161 rs10266594 7 AA AA AA AA AA
AA AA AA AA AA AA AA AA
AA AA AA AG AG AA AA AA
AA AA AA AA AG AA AG AG
AA AA AA AA AG AA AG
141093612
9162 rs1026663 4 GG AG AG GG AG
AG AG AG AG AA AG GG AG
AG AG AG AG AA AA AA AA
AA AG AG AG AA AG AA AG
AG AG GG GG GG AG AG
88789140
9163 rs10266642 7 AA AA AA AG AG

AA GG AG AG AG AG AG AG
AA AA GG AG AG AA AA AG
AG GG AG GG AA AA AA AA
AG AA AA AA AA AA AA
1477113
9164 rs10266663 7 AA AA AA AA AA
AA AG AA AA AA AA AG AG
AG AA AA AA AA AA AA AA
AA AA AA AA AA AA AA AA
AG AA AA AA AA AA AA
158115424
9165 rs10266672 7 -- -- AA AA AA
-- -- AA AA AA -- AA --
-- -- -- -- -- -- -- AA
AA -- -- AA AA AA -- AA
AA -- -- AA -- -- --
70073376
9166 rs1026673 15 GG GG GG AG GG
GG AG GG GG GG AG GG AG
AG AG AG AG AG AG GG AG
AG AG AG AG GG GG GG GG
GG AA AG AG GG GG AG
66493310
9167 rs10266796 7 AA AA AA AA AA
AA AA AA AA AA AA AA AA
AA AA AA AA AA AA AA AA
AA AA AA AA AA AA AA AA
AA AA AA AA AA AA AA
158678485
9168 rs10266821 7 GG GG GG GG GG
GG GG GG GG GG GG GG GG
GG GG GG GG GG GG GG GG
GG GG GG GG GG GG GG GG
GG GG GG GG GG GG GG
100270410
9169 rs10266891 7 AG GG AG AA AA
AA AA AA AA AA AA AA AA
AG AG GG GG AG AG AA GG
AG AA AG AA AA AA AA AA
AG AG GG GG GG AG GG
38914805
9170 rs1026690 3 AC AC CC AC AC
AC AC CC CC CC AA CC AC
AC AC AA AA AA AC AC AC
AC AA AC AC AC AC AA AC
AC CC AC CC AC AC CC
21506347
9171 rs10266905 7 AA AA AA AA AA
AA AA AA AA AA AA AA AA
AA AA AA AA AA AA AA AA

	AA	AA	AA	AA	AA	AA	AA	AA
	AA	AA	AA	AA	AA	AA	AA	
56062562								
9172	rs10266948		7	AA	AA	AA	AA	AG
	AA	AG	AA	AA	AA	AA	AA	AA
	AA	AA	AA	AA	AA	AG	AA	AA
	AA	AG	AG	AA	AA	AA	AA	AA
	AG	AA	AA	AA	AA	AA	AA	
53412123								
9173	rs10267051		7	AG	GG	AG	GG	GG
	GG	GG	AG	AG	AG	GG	GG	GG
	AG	AG	GG	AG	GG	GG	GG	GG
	GG	AG	AG	AG	AG	GG	GG	AG
	GG	AG	AA	AA	AG	AG	GG	
11819562								
9174	rs10267117		7	GG	GG	GG	GG	GG
	GG	GG	GG	GG	GG	GG	GG	GG
	GG	GG	GG	GG	GG	GG	GG	GG
	GG	GG	GG	GG	GG	GG	GG	GG
	AG	GG	AG	GG	GG	GG	GG	
135831413								
9175	rs10267134		7	AG	AA	AA	AG	AA
	AA	AG	AG	AG	AG	AA	AA	AA
	AA	AA	AA	AA	AA	AG	GG	AG
	AA	AG	AG	AA	AG	AA	AG	AG
	AA	AA	AA	AA	AG	AG	AA	
34803103								
9176	rs10267156		7	GG	GG	GG	GG	GG
	GG	GG	GG	GG	GG	GG	GG	GG
	GG	GG	GG	GG	GG	GG	GG	GG
	GG	GG	GG	GG	GG	GG	GG	GG
	GG	GG	GG	GG	GG	GG	GG	
52437934								
9177	rs1026716		3	AC	AA	AC	CC	AC
	AC	AC	AA	AA	AC	AA	AA	AA
	AC	AA	AC	CC	AA	AA	AC	AA
	AC	AC	AC	AC	AC	AC	AA	AA
	AC	AC	AC	AC	CC	CC	CC	
140983891								
9178	rs10267175		7	AA	AA	AA	AA	AA
	AA	AA	AA	AA	AA	AA	AA	AA
	AA	AA	AA	AA	AA	AA	AA	AA
	AA	AA	AA	AA	AA	AA	AA	AA
	AA	AA	AA	AA	AA	AA	AA	
104165607								
9179	rs10267195		7	AG	AG	AG	AA	AA
	AA	AA	AA	AA	AA	AA	AG	AG
	AA	AA	AA	AA	AA	AA	AA	AG
	AG	AA	AA	AG	AA	AA	AA	AA
	AA	AA	AG	AG	AA	AA	AA	

51730710
9180 rs10267275 7 AA AA AA AA AA
AA AA AG AG AG AA AA AA
AG AA AA AA AA AG AA AA
GG AG AA GG AA AA AA AA
AG AA AA AA AA AA AA
102499076
9181 rs10267294 7 GG GG GG AG AG
GG AA GG GG GG GG GG GG
AG AG AG GG GG AA AG AG
GG GG GG GG GG AG AG AG
AG AG GG GG AG GG AA
152279204
9182 rs10267310 7 GG GG GG GG GG
GG GG GG GG GG GG GG GG
GG GG GG GG GG GG GG GG
GG GG GG GG GG GG GG GG
GG GG GG GG GG GG GG
100230028
9183 rs10267331 7 CC CC CC AC CC
CC AC CC CC CC AC AC AA
AC CC AC CC AC CC CC CC
CC AC AC CC AC AA AC AA
CC CC AA AC CC CC AC
63673852
9184 rs10267374
7 -- -- -- -- -- -- -- --
-- -- -- -- -- -- -- --
-- -- -- -- -- -- -- --
-- -- -- -- -- -- -- --
-- -- -- -- 88560427
9185 rs1026741 4 GG GG AG GG GG
GG GG GG GG GG GG GG GG
GG GG GG AG AG GG GG GG
GG GG GG GG GG GG GG GG
AG GG GG GG AG AG GG
763407
9186 rs10267413 7 AA AA AA AA AG
AG AA AA AA AA AA AA AA
AA AG AA AA AA AA AA AA
AA AA AA AA AA AA AA AA
AA AG AG AG AA AA AA
115327840
9187 rs10267426 7 GG GG GG GG GG
GG GG GG GG GG GG GG GG
GG GG GG GG GG GG GG GG
GG GG GG GG GG GG GG GG
GG GG GG GG GG GG GG
99994300
9188 rs10267446 7 AG GG AG GG GG

	GG	GG	GG	GG	GG	GG	GG	GG
	GG	GG	GG	GG	GG	GG	GG	GG
	GG	GG	GG	GG	GG	GG	GG	GG
	GG	GG	GG	GG	GG	GG	GG	
17001266								
9189	rs10267468		7	AA	AA	AA	AG	AG
	AA	AG	AA	AA	AA	AG	GG	AG
	AG	AG	AA	AA	AA	AA	AA	AA
	AA	AA	AA	AA	AG	AA	AA	AA
	AG	AA	AG	AG	AA	AA	AA	
125104792								
9190	rs10267474		7	AA	AA	AA	AG	AG
	AG	AG	GG	GG	AG	GG	GG	GG
	AG	AG	GG	GG	GG	AG	AG	AG
	AG	AG	AG	AG	GG	AG	AG	AG
	GG	AG	GG	GG	AG	AG	GG	
94152988								
9191	rs10267521		7	GG	GG	AG	GG	GG
	GG	GG	AG	GG	AG	AG	GG	AG
	GG	GG	GG	GG	GG	GG	GG	GG
	GG	GG	GG	GG	GG	GG	GG	GG
	GG	GG	GG	GG	GG	GG	GG	
56580547								
9192	rs10267525		7	AG	AA	AA	AG	AA
	AG	AA	AA	AG	AG	AG	AG	AA
	AA	AA	AG	AG	AG	GG	GG	AG
	AG	AA	AA	AG	AA	AG	AA	AA
	AA	AG	AG	AG	AA	AA	AA	
75298032								
9193	rs1026756		X	GG	GG	AG	AA	GG
	AA	AG	AA	AA	AA	AA	AA	AA
	AA	AA	AA	AG	AG	AG	AG	GG
	AA	AA	AA	AG	GG	GG	GG	GG
	AG	AG	AA	AA	AA	AA	AA	
26936592								
9194	rs10267583							
7	--	--	--	--	--	--	--	--
	--	--	--	--	--	--	--	--
	--	--	--	--	--	--	--	--
	--	--	--	--	--	--	--	--
	--	--	--	--	24476930			
9195	rs10267610		7	AC	AA	AC	AA	AA
	AA	AA	AA	AA	AA	AA	AA	AA
	AA	AC	AC	AC	AC	AA	AA	AA
	AA	AA	AA	AA	AA	AA	AA	AA
	AA	CC	AA	AC	AC	AC	AA	
63397111								
9196	rs10267619		7	AG	AG	AG	GG	GG
	GG	GG	AG	AG	GG	GG	AG	AG
	GG	GG	AA	AG	AG	AG	AG	AG

GG AG AG AG AG GG AG GG
GG GG AA AG GG GG AG
14369294
9197 rs10267633 7 CC CC CC CC CC
CC CC CC CC CC CC CC CC
CC CC CC CC CC CC CC CC
CC AC AC CC CC AC AC AC
CC CC AC CC CC CC CC
105036690
9198 rs10267636 7 AC AA AC CC CC
CC CC AA AA AC CC CC AC
AA AC AC AA AA AC CC AC
AA AA AC AA AC AA AA AA
AA AC AC AA AC CC AC
96526030
9199 rs10267676 7 GG GG GG GG AG
GG AG AG AG AG AG GG AG
AA AG GG GG GG AG GG GG
GG GG GG GG GG GG GG GG
GG AG AG AG GG GG GG
97256347
9200 rs10267684 7 AG GG GG AG GG
AG GG GG GG GG GG GG GG
AG AG GG GG GG AG GG AG
GG AG AG GG AG GG GG AG
GG AA AA AA GG AG GG
71626255
9201 rs1026779 3 AG AG AA GG GG
GG GG AA AA AG AA AA AA
AG AG AA AA AA AG AG AG
AG AG AA AG AG GG AG AG
AA AA AG AA GG GG GG
140088658
9202 rs10267816 7 AA AA AA AA AA
AA AA AA AA AA AA AG AG
AA AG AA AA AA AA AA AA
AA AG AA AG AA AA AA AA
AA AA AA AA AG AG AA
22129599
9203 rs10267936 7 AA AA AA AA AC
AC AA AA AA AA AC AA AC
AC AA AA AA AA AA AA AA
AA AA AA AA AA AA AA AA
AC AC AA AC AA AA AC
77027076
9204 rs1026804 8 GG AG GG AG AG
AG AA GG GG AG AA AA AA
AA AG AA AA AG AG AG AA
AA AA AA AA AG AA AG AG
AA AG AA AA AA AA AA

81518320								
9205	rs10268122		7	AG	GG	AG	GG	GG
	GG	GG	AG	AG	AG	GG	GG	GG
	GG	GG	GG	GG	GG	AG	AG	AG
	GG	GG	GG	GG	GG	GG	GG	GG
	GG	GG	AG	GG	GG	GG	AG	
5100411								
9206	rs10268180		7	AG	AG	AG	AA	AA
	AA	AA	AG	AG	AG	AA	AA	AA
	AA	AA	AG	AG	AA	AG	AA	AG
	GG	AG	AG	GG	AA	AG	AA	AA
	GG	GG	AG	GG	AA	AA	AA	
19019671								
9207	rs10268224		7	GG	GG	GG	GG	GG
	GG	GG	GG	GG	GG	GG	GG	GG
	GG	GG	GG	GG	GG	GG	GG	GG
	GG	GG	GG	GG	GG	GG	GG	GG
	GG	GG	GG	GG	GG	GG	GG	
21892435								
9208	rs10268225		7	AA	AA	AA	GG	GG
	AG	GG	AA	AA	AG	AA	AG	AG
	AA	AG	AA	AA	AA	GG	GG	AG
	GG	GG	GG	GG	AA	AG	AA	AA
	AA	AA	AG	AG	GG	GG	AG	
162145								
9209	rs1026824		4	AA	AA	AA	AA	AA
	AA	AA	AG	AG	AA	AA	AG	AA
	AA	AA	AA	AA	AA	AA	AA	AA
	AA	AG	AG	AA	AA	AG	AG	AA
	AA	AA	AA	AA	AA	AA	AA	
128072800								
9210	rs10268257		7	AA	AA	AA	AA	AA
	AA	AA	AA	AA	AA	AA	AA	AA
	AA	AA	AA	AA	AA	AA	AA	AA
	AA	AA	AA	AA	AG	AA	AG	AA
	AA	AA	AA	AA	AA	AA	AA	
70003835								
9211	rs10268269		7	GG	GG	GG	GG	GG
	GG	GG	GG	GG	GG	GG	GG	GG
	GG	GG	GG	GG	GG	GG	GG	GG
	GG	GG	GG	GG	GG	GG	GG	GG
	GG	GG	GG	GG	GG	GG	GG	
148392459								
9212	rs1026830		8	GG	AG	GG	AA	AG
	AA	AG	AG	AG	AG	AG	AA	AG
	AG	AA	AG	AA	GG	AA	AA	AA
	AG	AG	AA	AA	AA	AG	AA	AA
	AA	AA	AA	AA	AG	AG	AA	
81958305								
9213	rs10268319		7	AG	AG	GG	GG	AG

GG AG GG GG AG GG AG AG
AA AA AG AG AG AG GG GG
AA AA AA AA AG GG AG AG
GG AG AG GG AG GG AA
36970835
9214 rs1026832 8 GG AG GG GG GG
GG GG GG AG AG GG GG GG
GG AG GG GG GG GG AG AG
GG AG AG AG GG GG GG GG
AG GG AG AG AG AG AG
70254430
9215 rs1026833 11 GG GG GG AG AA
AG AA AG GG GG AG AA AG
AG AG GG GG GG AG GG AG
AG AG AG AG AG GG AG AG
AG AG GG AG GG AG AG
38566424
9216 rs1026834 11 AA AA AA AG AG
AA GG AA AA AA AG AG AA
GG AA AG AA AG AG AA AG
GG AA AG AG AG AG GG AG
AG AG AG GG AG AG GG
38685169
9217 rs10268365 7 AA AA AA AA AA
AA AA AG AG AG AA AA AA
AA AA AA AA AA AA AA AA
AA AA AA AA AA AA AA AA
AA AA AA AA AG AA GG
101024309
9218 rs10268409 7 AA AA AA AG AA
AG AA AA AA AA AA AA AA
AA AA AA AA AA AA AA AA
AA AA AA AA AA AG AG AG
GG AA AA AA AA AA AA
130998106
9219 rs10268421 7 AG AA AG AG AG
AG AG AA AA AA AA AA AA
AA AA GG GG AG AG AG AA
AA AA AA AA AA AA AA AA
AA AG AG AG AG AA AG
9650438
9220 rs10268441 7 AA AA AA AG AG
AA AG AA AA AA AG AA AG
AG AG AA AA AA AA AA AA
AG AA AA AG GG AA AG AG
AG AA GG AG AA AA AA
115317894
9221 rs10268447 7 GG GG AG AG AG
AG AG GG AG GG GG GG GG
GG GG GG GG AG GG GG GG

AG GG AG GG GG AG AG AG
GG AG GG AG GG AG GG
30187932
9222 rs10268448 7 AG AG AG AG AG
GG AA AG AG AG AA AG AG
AA AG AG AG AG AG AA GG
GG AG AG GG AG AA AG AA
AG AG AG AG AG AA AG
20071322
9223 rs10268458 7 GG AG AG AG AG
GG AA GG GG AG GG GG AG
AG AG AA AG AG GG GG GG
GG AG AA GG GG AG GG GG
AG AG AA AA AG GG AG
88751897
9224 rs10268479 7 AG AA AG AG AG
AA AG AG AG AG GG GG GG
GG GG AG GG GG AA AA AG
AA AG AG AA AG AG GG AG
GG AG GG AG GG GG AG
78122616
9225 rs1026849 8 CC CC CC AC AC
AA CC CC CC CC CC CC CC
AC AC AC AC CC CC CC AC
AA AC AA AC CC AC CC AC
CC CC CC CC AC AC CC
3589776
9226 rs10268528 7 GG GG GG GG GG
GG GG GG GG GG GG GG GG
GG GG GG GG GG GG GG GG
GG GG GG GG GG GG GG GG
GG GG GG GG GG GG GG
75867646
9227 rs10268548 7 AG AA AA AA AA
AA AG AG AG AA AA AA AA
AG GG AA AA AA AG GG AA
AA AA AG AG AA AA AA AA
AG AG AG AG AA AA AA
76307066
9228 rs10268574 7 CC AC AC CC CC
CC CC CC CC CC CC CC CC
CC CC CC CC CC AC CC AC
CC CC AC CC CC AC CC CC
CC AC CC AC AC AC AC
87768174
9229 rs10268584 7 GG AG GG GG AG
AG GG GG GG AG AG AG GG
AG AG AG AA AA AG GG AA
AA AG AG AG GG AG GG GG
AG AG GG AG AG GG AA

13938185
9230 rs10268585 7 CC AC AC CC AC
AC CC AC AC AC AA AC AC
AC CC CC CC AC AC AC AC
AA CC AC CC AA CC AC AC
AC AA AA AA AC AC AA
3760988
9231 rs10268597 7 AA AC AA CC CC
CC AC AC CC AC AC CC AC
CC AC CC AC AC AA AC AC
AC AC AC AC CC CC CC CC
AC CC CC CC AA AC AA
146975480
9232 rs102686 5 GG AG AG AG AG AA
AG GG GG GG AG AG AG AG
GG AG GG AG GG AG GG AG
AA AG AG AG GG AG GG AG
GG AG GG GG GG AG 170135225
9233 rs10268609 7 AA AA AA AC AC
AC AC AC AA AA AC AC AC
AC AC CC CC AC AA AA AA
CC CC CC CC AA AC AC AC
AA AC AC AC AA AA AC
1962163
9234 rs10268610 7 AA AA AA AG AA
AG AA AG AG AG AA AA AG
AA AG GG GG GG AA AA AA
GG AA AG AG AA AG AA AA
AA AG AG AA GG GG GG
23274764
9235 rs10268695 7 CC AC AC CC CC
CC CC CC AC AC CC AC AC
CC CC AC AA AA AC AA AC
AC AA AC AC CC CC CC CC
CC AC AC AA AC CC AA
47929189
9236 rs10268699 7 CC CC CC CC CC
CC CC CC CC CC CC CC CC
CC CC CC CC CC CC CC CC
CC CC CC CC CC CC CC CC
CC CC CC CC CC CC CC
19478176
9237 rs10268780 7 AA AA AA AA AG
AG AA AG GG AG AA AA AA
AA AA AA AA AA AA AG AA
AG AA AA AG AA AA AA AA
AG AA GG AG AA AA AA
25132183
9238 rs10268819 7 GG GG GG GG GG
GG GG GG GG GG GG GG GG

GG GG GG GG GG AG GG GG
GG GG GG GG GG GG GG GG
GG AG GG AG GG AG GG
50610071
9239 rs1026882 2 AG GG AG AG AG
AA GG AG AA AG AA AG AG
AG AG AG AG AG AA AG AA
AG AG AG GG AG AA AG AG
AG AG AG AG AA AA AG
213387389
9240 rs10268833 7 CC CC CC CC CC
CC CC AC AC AC CC CC AC
AC CC CC CC CC AC CC AC
AC CC -- AC CC CC CC CC
CC CC CC CC CC AC CC
94181480
9241 rs1026885 14 AG AG AG AG GG
GG AG GG GG GG GG GG GG
GG GG GG AG GG GG GG GG
GG GG GG GG GG GG GG GG
AG GG GG GG GG GG GG
34428218
9242 rs1026889 2 AA AG AA AG AG
GG AG AG AG GG AG AG GG
AG AG AA AA AA AA AA AA
AA AA AA AA AA AG AG AG
AA AG AG AG AG GG AG
149983918
9243 rs10268902 7 GG AG GG AG AG
AG AG AA AA AA AA AA AA
AG AA AG AG GG AA AA AA
AA AG AA AG AA AA AA AA
AG AG AA AG AG AA AG
27438124
9244 rs10268943 7 AA AA AA AG AG
AA GG AA AA AG AA AA AA
AA AA GG AG AG AA AA AA
AA AA AA AA GG AG AG AG
GG AA AA AA AG AG GG
90068856
9245 rs10268957 7 AA AA AA AA AA
AA AA AA AA AA AA AA AA
AA AA AA AA AA AA AA AA
AA AA AA AA AA AA AA AA
AA AA AA AA AA AA AA
16444499
9246 rs10268984 7 GG GG GG GG AG
AG GG GG GG GG AG GG AG
AA GG GG GG GG GG GG GG
GG GG GG GG GG GG GG GG

	GG	GG	AG	AG	GG	GG	AG
98963340							
9247	rs1026907		X	AA	AA	AG	AA	AA
	AA	AA	AG	AG	GG	AG	AG	AG
	AG	AA	AA	AA	AA	AA	AA	AA
	AA	AA	AA	AG	AG	GG	GG	GG
	AA	AA	AA	AA	AA	AA	AA	
5521547								
9248	rs10269101		7	AA	AA	AC	CC	AA
	AC	AC	CC	AC	AC	AC	AA	AC
	AA	AA	AA	AA	AC	AA	AA	AC
	AA	AA	AC	AC	AC	AC	AA	AC
	AA	AA	AA	AA	AC	AA	AC	
56617474								
9249	rs10269151		7	GG	GG	GG	GG	GG
	GG	GG	GG	GG	GG	GG	GG	GG
	GG	GG	GG	GG	GG	GG	GG	GG
	GG	GG	GG	GG	GG	GG	GG	GG
	GG	GG	GG	GG	GG	GG	GG	
1126876								
9250	rs10269153		7	AA	AA	AG	AA	AG
	AG	AA	AA	AA	AG	AA	AG	AG
	AA	AA	AG	AA	AA	AA	AG	AA
	AG	AG	GG	GG	AG	AG	AG	AA
	AA	AA	GG	AG	AA	AA	AA	
67909312								
9251	rs10269209		7	AA	AG	AG	GG	AG
	GG	AG	GG	GG	GG	GG	GG	GG
	AG	GG	GG	GG	GG	GG	GG	GG
	AG	GG	GG	GG	GG	AA	AG	AG
	AG	AG	AG	AG	GG	GG	AG	
5324257								
9252	rs10269341		7	AC	AC	AC	AA	AA
	AA	AA	AA	AA	AA	CC	AC	AC
	AA	AA	AA	AA	AA	AA	AC	AA
	AA	AC	AA	AC	AA	AA	AA	AA
	AC	AA	AA	AA	AC	AA	AC	
38534816								
9253	rs10269349		7	AG	AG	GG	AA	GG
	AG	AG	AG	AG	GG	GG	AG	AG
	GG	GG	AG	AG	AA	GG	AG	GG
	AG	GG	GG	AG	AG	AA	AG	AG
	GG	AG	AG	GG	AG	GG	AG	
40242537								
9254	rs10269406		7	AG	AA	AG	GG	GG
	GG	GG	AA	AA	AA	GG	AG	AG
	GG	AG	AA	AG	AG	GG	GG	AG
	GG	AG	AG	AG	AA	GG	AG	AG
	GG	AG	AG	GG	GG	GG	GG	
10904755								

9255 rs10269471 7 AA AA AA AA AG
AG AA AA AA AA AA AA AA
AA AG AA AA AA AA AG AG
AA AA AA AA AA AG AG AG
AA AA AA AA AA AA AA
97464809
9256 rs10269496 7 GG GG GG GG GG
GG GG GG GG GG GG GG GG
GG GG GG GG GG GG GG GG
GG GG GG GG GG GG GG GG
GG GG GG GG GG GG GG
41407678
9257 rs1026952 3 AG AG AG GG AG
AG AG AG AA AG GG AG AG
AG AG AG GG GG GG AG GG
AG AG AG AG AG AG GG AA
AG AG AG AG AG AA AG
58645595
9258 rs10269566 7 AA AA AA AA AG
AA AG AA AA AA AA AA AA
AA AG AA AA AA AA AG AA
AG AG AA AG AG AG GG GG
AG AG AG AA AA AA AA
96744453
9259 rs10269654 7 GG GG GG GG GG
GG GG GG GG GG GG GG GG
GG GG GG GG GG GG GG GG
GG GG GG GG GG GG GG GG
GG GG GG GG GG GG GG
155341982
9260 rs10269693 7 AA AA AA AG AA
AG AA AA AA AA AA AA AA
AG AG AG AA AA GG AG AG
AA AA AG AG AA AA AA AA
AA AA AA AA GG AG GG
67177415
9261 rs10269717 7 AG GG AG GG GG
GG GG GG GG GG GG GG GG
GG GG GG GG GG GG GG AG
AG AG GG AG GG GG GG GG
AG AG GG AG GG GG GG
115911312
9262 rs10269830 7 AG GG GG AG AG
AA GG GG GG GG GG GG GG
GG GG GG GG GG AG GG AG
GG GG GG GG GG AG AG AG
AG GG AG GG GG AG AG
70109813
9263 rs1026985 5 AA AA AA AG AG
AG AG AA AG AA AA AA AA

AG AG AA AG AG AG AG AG
AA AA AA AA GG AA AG AG
AG AA AG AA AA AG AA
178805612
9264 rs10269850 7 AG AA AG GG AG
GG AG AG AG GG AG GG AG
AA AG AG AG AG AA AA AA
AG AG GG AG AA AG AG AG
AG AG AA AG AA AA AA
15421880
9265 rs1026989 3 AA AG AA AA AA
AA AA AA AA AA AG GG AG
AA AG AA AA AA AA AA AA
AG AA AG AA AA AA AA AA
AA AG AA AA AA AA AG
59892473
9266 rs10269921 7 AG AG AG GG GG
GG GG GG GG GG AA AA AA
AG AG AG AG AG GG GG GG
GG AA AG AA AG AG AA AG
AG AG AG AG AG AG AG
17997940
9267 rs10269969 7 GG GG GG GG GG
GG GG GG GG GG GG GG GG
GG GG GG GG GG GG GG GG
GG GG GG GG GG GG GG GG
GG GG GG GG GG GG GG
4682180
9268 rs1026997 1 AG AG AG AG AG
GG AA AG AA AA AG AG AG
AG AG AA AG AA AG AG AG
AG AG AG AG AA AG AA AA
AG AG AG AG GG GG AG
72516287
9269 rs10269975 7 GG GG GG GG AG
AG GG AG AG AG GG GG GG
GG GG GG GG GG AA AA AA
AG AG AG AG AG AA AA AA
AG GG AG GG AG GG AA
112321309
9270 rs10270 12 AG -- AG -- -- --
-- AA AA AA -- -- -- AG
AG -- -- -- -- -- AG --
-- -- -- -- AG -- -- AG
-- -- -- -- AG -- 122756342
9271 rs10270000 7 AG GG AG GG AG
AG GG AG AG AG GG GG GG
GG GG GG GG GG AA AA AA
AG AG AG AG AG AA AA AA
AG GG AG GG AG GG AA

112321378							
9272	rs10270008	7	GG	GG	GG	GG	GG
	GG	GG	GG	GG	GG	GG	GG	GG
	GG	GG	GG	GG	GG	GG	GG	GG
	GG	GG	GG	GG	GG	GG	GG	GG
	GG	GG	GG	GG	GG	GG	GG	
35593568							
9273	rs1027002	2	AA	AA	AA	AA	AA
	AA	AA	AG	AG	AA	AA	AA	AG
	AA	AA	AA	AA	AA	AA	AG	AA
	AG	GG	AG	AG	AG	AA	AA	AG
	AG	AA	GG	AG	AA	AG	AG	
108724001							
9274	rs10270076	7	AG	AG	AG	AG	GG
	GG	AG	AG	AG	AG	GG	GG	GG
	AG	AG	AA	AG	AA	AG	GG	AA
	AA	AG	AA	AG	AG	AG	AA	AG
	GG	AG	AA	AA	GG	GG	GG	
25363037							
9275	rs10270078	7	GG	GG	GG	GG	GG
	GG	GG	GG	GG	GG	GG	GG	GG
	GG	GG	GG	GG	GG	GG	GG	GG
	GG	GG	GG	GG	GG	GG	GG	GG
	GG	GG	GG	GG	GG	GG	GG	
5429685							
9276	rs10270083	7	GG	GG	GG	AA	GG
	AG	AG	GG	GG	GG	GG	GG	GG
	GG	GG	GG	GG	GG	GG	GG	GG
	AG	AG	GG	AG	GG	GG	GG	GG
	GG	GG	AG	GG	GG	GG	GG	
101526248							
9277	rs10270099	7	AA	AG	AA	AG	GG
	AG	GG	AG	AG	AG	AA	AA	AA
	AA	AA	AG	AA	AA	AG	GG	AG
	AA	AG	AG	AA	AG	AG	GG	AG
	GG	AA	GG	AG	GG	AG	GG	
26377088							
9278	rs10270146	7	CC	CC	CC	CC	CC
	CC	CC	CC	CC	CC	CC	CC	CC
	CC	CC	CC	CC	CC	CC	CC	CC
	CC	CC	CC	CC	CC	CC	CC	CC
	CC	CC	CC	CC	CC	CC	CC	
99374510							
9279	rs10270171	7	AC	AA	AC	AC	AA
	AA	AC	AA	AC	AA	AA	AA	AA
	AA	AA	AA	AA	AA	AC	AA	AC
	AC	AA	AA	AC	AA	AC	AA	AA
	AA	AA	AC	AC	AA	AA	AC	
22813879							
9280	rs10270197	7	AC	AA	AC	AA	AA

	AA	AA	AA	AA	AA	AA	AA	AA
	AA	AA	AA	AA	AA	AA	AA	AA
	AA	AA	AA	AA	AA	AA	AA	AA
	AC	AC	AA	AA	AC	AC	AA	
108862337								
9281	rs10270230		7	AC	AC	CC	AA	AA
	AA	AA	AA	AA	AC	AA	AC	AC
	AA	AA	AC	AC	AA	CC	AC	AC
	AA	AC	AA	AC	AC	CC	CC	CC
	AA	AA	AC	AA	AC	AA	CC	
37896146								
9282	rs10270332		7	GG	GG	GG	GG	GG
	GG	GG	GG	GG	GG	GG	GG	GG
	GG	GG	GG	GG	GG	GG	GG	GG
	GG	GG	GG	GG	GG	GG	GG	GG
	GG	GG	GG	GG	GG	GG	GG	
65182085								
9283	rs10270339		7	AA	AA	AA	AA	AA
	AA	AA	AA	AA	AA	AA	AA	AA
	AA	AA	AG	AA	AA	AA	AA	AA
	AA	AG	AA	AG	AA	AA	AA	AA
	AA	AA	AA	AA	AG	AA	AG	
3232907								
9284	rs10270358		7	GG	AG	AG	AG	AA
	AG	AG	AG	AG	AG	GG	AG	AG
	AG	AA	AG	GG	GG	GG	GG	AG
	AG	AA	AG	AG	AA	GG	AG	AG
	GG	AG	AG	AA	AG	GG	AA	
114948351								
9285	rs10270385		7	AG	AG	AA	AG	AG
	GG	AA	AG	AA	AG	AG	AA	AG
	AG	GG	AA	AG	AG	GG	AG	AG
	AG	AG	AG	AG	AA	GG	AG	AG
	GG	AG	GG	AG	AG	AG	AA	
149560348								
9286	rs10270413		7	GG	GG	GG	AA	GG
	AG	AG	GG	GG	GG	AG	AA	AG
	AG	GG	AA	AG	AG	GG	GG	AG
	AA	GG	AG	GG	GG	AG	AG	AG
	GG	GG	AG	GG	GG	GG	AG	
4304727								
9287	rs10270424		7	CC	CC	CC	CC	CC
	CC	CC	CC	CC	CC	CC	CC	CC
	CC	CC	CC	CC	CC	CC	CC	CC
	CC	CC	CC	CC	CC	CC	CC	CC
	CC	CC	CC	CC	CC	CC	CC	
21018240								
9288	rs10270441		7	AC	AA	AC	AA	AA
	AA	AC	AC	AC	AA	AC	AC	CC
	AC	AA	CC	AC	AC	AC	AC	AC

AA AA AA AA AC AC CC AC
AC AC AA AA AA AA AA
14558320
9289 rs1027045 4 GG AG AG GG GG
GG GG GG GG AG GG GG GG
GG GG GG GG GG GG GG AG
GG GG GG GG GG GG GG GG
GG GG GG GG GG GG GG
111319763
9290 rs10270482 7 GG AG AG AG GG
AG AG AA AA AA AA AA AA
AA AA AG GG GG AG GG AG
GG AG AA AG AG AA AG AA
AG AG AG AA AA AA AG
77222886
9291 rs10270530 7 AA AG AA AG AG
AG AG AA AA AA AA AA AA
AG AA AA AG AA GG GG GG
GG AG GG AG AA AA AA AA
AG AG AG AG AG GG AG
8488691
9292 rs1027061 8 GG AG GG AA AG
AG AG AA AA AA AG AA AG
AG AG AA AG AA AA AA AG
AA AA AA AA AG AA AA AG
AG AG AG AG AA AA AA
89928732
9293 rs10270627
7 -- -- -- -- -- -- -- --
-- -- -- -- -- -- -- --
-- -- -- -- -- -- -- --
-- -- -- -- -- -- -- --
-- -- -- -- 70201815
9294 rs10270681 7 AA AC AC AC AC
AA AC AC AC AC AA AC AC
AC AA AA AA AA AA AA AA
AA AA AA AA AC AC AC AA
AA AA AC AC AC AA AC
79316164
9295 rs10270726 7 AA AA AA AA AA
AA AA AA AA AG AG AG AG
AG AG AA AA AG AA AA AA
AA AA AA AA AA AA AA AA
AA AG AA AG AG AG AA
1714703
9296 rs10270793 7 GG GG GG GG GG
GG GG AG AG AG AG GG AG
AG AG AG AA AA GG AG GG
AG AG AG AG GG AG GG GG
GG GG AG AG AG AG GG

123336804							
9297	rs10270844	7	AA	AG	AG	GG	GG
	GG	GG	AA	AA	AG	AG	AG	AG
	AG	GG	AA	AG	AG	AG	AG	AA
	GG	AG	AG	GG	AG	AG	GG	AG
	AA	AG	AG	AG	GG	AG	GG	
83109037							
9298	rs10270931	7	AG	AG	AG	GG	AG
	AG	AG	AG	AG	GG	AG	AA	AA
	AA	AG	AG	GG	AG	GG	AG	GG
	GG	AA	AG	AA	GG	GG	GG	GG
	GG	AG	AG	AA	GG	GG	GG	
12418964							
9299	rs10270979	7	AA	AA	AA	AG	AG
	AG	AA	AA	AG	AG	AA	AA	AA
	AA	AA	AG	AG	AG	AA	AA	AA
	AG	AA	AG	AG	AA	AG	AA	AA
	AA	AA	AA	AA	AG	AG	AA	
154578547							
9300	rs10271156	7	CC	CC	CC	AA	AC
	AA	AC	AC	CC	AC	AC	AA	AC
	CC	AC	AC	AC	AC	CC	CC	CC
	AC	CC	CC	AC	AC	CC	AC	CC
	AC	AC	CC	AC	CC	CC	CC	
22512880							
9301	rs10271159	7	AA	AA	AA	AA	AA
	AA	AA	AG	AA	AA	AA	AA	AA
	AA	AA	AA	AA	AA	AA	AA	AA
	AA	AA	AA	AA	AG	AA	AA	AA
	AA	AA	AA	AA	AA	AA	AA	
31804088							
9302	rs10271240	7	GG	GG	AG	GG	GG
	GG	GG	GG	GG	GG	GG	GG	GG
	GG	GG	AG	AG	AG	GG	GG	GG
	GG	GG	GG	GG	GG	GG	GG	GG
	GG	GG	GG	GG	GG	GG	GG	
62284518							
9303	rs10271255	7	AA	AA	AA	AA	AG
	AG	AA	AA	AA	AG	AA	AA	AA
	AG	AG	AA	AA	AA	AG	AG	AG
	AA	AG	AG	AA	AA	AA	AA	AA
	AG	AA	AA	AA	AG	GG	AG	
63910447							
9304	rs10271311	7	AA	AA	AA	AC	AC
	AC	AC	AA	AA	AA	CC	AC	AC
	AC	AC	AA	AA	AA	AA	AA	AA
	AA	AA	AA	AA	AA	AA	AA	AA
	AA	AC	AA	AC	AA	AA	AA	
45069584							
9305	rs10271343	7	AG	AG	GG	GG	GG

GG GG GG GG GG AG GG AG
AG AG GG GG GG GG AG GG
GG AG GG GG AG GG GG GG
GG AG AA AA AG GG AG
136272383
9306 rs10271349 7 GG GG GG AG AG
AA GG AG AA AG AA AA AA
AA AG GG AG AG AG GG AA
AA AG AA AA AA GG AG AG
AG AG GG GG AA AG AA
131942569
9307 rs10271370 7 CC CC CC CC CC
CC AC AC AC CC CC CC CC
CC CC AC CC CC AC AC CC
CC CC CC CC AC AC CC AC
CC CC CC CC CC CC AC
78158609
9308 rs10271403 7 AC AC AC AA AA
AA AC AA AA AA AA AA AA
AA AA AA AA AA AA AC AA
AA AA AA AA AA AA AA AA
AA AA AC AA AA AA AA
155240753
9309 rs10271414 7 AG AA AA AG GG
AG GG AA AA AG AA AA AA
AG AG AA AA AA AA AA AA
AA AA AA AA AA AA AA AA
AG AG AA AA AA AA AA
70641761
9310 rs10271444 7 GG GG GG GG GG
GG GG GG GG GG GG GG GG
GG GG GG GG GG AG AG GG
GG GG GG GG GG GG GG GG
GG GG GG GG GG GG GG
137689901
9311 rs10271463 7 AA AG AA AA GG
AG AG AG GG GG AG AG AG
AG GG GG AG GG AG AG GG
AA GG AG GG AG AG AA AA
GG GG AG AG AA AG AA
8225890
9312 rs10271617 7 AG AA AG AA AA
AA AA AA AA AA AA AA AA
AA AA AA AA AA GG AG AG
AG AG AA AG AG AA AG AG
AG AA AG AA AA AA AA
126045700
9313 rs10271715 7 AA AA AA AA AA
AA AA AA AA AA AA AA AA
AA AA AA AA AA AA AA AA

AA AA AA AA AA AA AA AA
AA AA AA AA AA AA AA
52818156
9314 rs10271744 7 AG GG GG GG GG
GG GG AG GG GG GG GG GG
GG GG AG AG AG AG GG GG
GG AG GG GG AG GG GG GG
GG GG GG GG GG AG GG
153088772
9315 rs10271765 7 GG GG GG GG GG
GG GG GG GG GG GG GG GG
GG GG GG GG GG GG GG GG
GG GG GG GG GG GG GG GG
GG GG GG GG GG GG GG
157888398
9316 rs10271783 7 GG GG GG GG GG
GG GG GG GG GG GG GG GG
GG GG GG GG GG GG GG GG
GG GG GG GG GG GG GG GG
GG GG GG GG GG GG GG
75755152
9317 rs10271818 7 AG AG AG AG GG
AG GG AG AG AG GG AG AG
GG GG GG GG AG GG AG GG
GG GG GG GG GG GG GG GG
GG GG GG GG GG GG GG
10137638
9318 rs10271820 7 GG AG AG GG GG
GG GG AG AG AG AA AG AG
AG AG AG AG AG AA AA AG
GG AG GG GG AA AG AG AG
GG AA AG AG AG AG AG
118777401
9319 rs1027183 3 GG AG GG AG AG
AG GG AA AG AA GG GG GG
AG AG GG AG GG AG AG AA
GG GG AG GG AG AA AA AG
AA AA GG AG AA AA AA
176372607
9320 rs10271895 7 GG GG GG GG GG
GG GG GG GG GG GG GG GG
GG GG GG GG GG GG GG GG
GG GG GG GG GG GG GG GG
GG GG GG GG GG GG GG
100871669
9321 rs10271991 7 AA AG GG AG GG
AG GG AA AA AA GG GG GG
AG AA AG AG AG AG GG AA
AG AG AA AA AA GG AG AG
GG AG AA AG AA AG AG

76956774
9322 rs10271999 7 -- AG -- -- --
-- -- -- AG -- AA AA --
-- AG AG AG AG AG AG --
-- AG AA AG -- -- -- --
-- AG -- -- AG AG AG
27509208
9323 rs1027201 4 AA AA AG AG AG
AA GG AG AG GG GG AG GG
GG AG AG AG GG AG AG GG
AA AG AG AG GG AG AG AG
GG AG GG AG GG GG GG
44167271
9324 rs10272095 7 AA AA AA AA AA
AA AA AA AA AA AA AA AA
AA AA AA AA AA AA AA AA
AA AA AA AA AA AA AA AA
AA AA AA AA AA AA AA
76006522
9325 rs10272099 7 AG GG AG AA AG
AG AA AA AA AA AG AG AA
AG AG AG AG GG AG AG AA
AA AG AA AG AG AA AG AG
AA AA AA AA AG AG AA
1016948
9326 rs10272110
7 -- -- -- -- -- -- -- --
-- -- -- -- -- -- -- --
-- -- -- -- -- -- -- --
-- -- -- -- -- -- -- --
-- -- -- -- 75449338
9327 rs10272173 7 AA AG AA AG GG
GG AG AG GG GG AA AA AG
AG GG AA AA AA AA AA AA
AA AG AG AG AA AG AA AA
AA AG AA AA AA AA AA
7263117
9328 rs10272211 7 GG GG GG GG GG
GG GG GG GG GG GG GG GG
GG GG GG GG GG GG GG GG
GG GG GG GG GG GG GG GG
GG GG GG GG GG GG GG
54461764
9329 rs10272217 7 AG GG GG AA AA
AG AA AA AA AA GG GG GG
GG AG AG AG AG AG GG AG
AG GG AG AG AG AA AG AG
AG AA AG AG AA AG AG
145721952
9330 rs10272302 7 AG GG AG AG GG

AG GG AG AG AG AA AG AG
AG GG GG AG AG GG GG GG
GG AG AG GG GG GG GG GG
AG AA AA AA AG AA GG
20527578
9331 rs10272326 7 AA AA AA AA AA
AA AA AA AA AA AG AG AA
AA AA AA AA AA AA AA AA
AA AA AA AA AA AA AA AA
AA AA AA AA AA AA AA
154435853
9332 rs10272334 7 AG AG AG GG GG
GG GG GG GG GG AG GG GG
GG GG AG AA AG GG GG GG
GG GG GG GG GG GG GG GG
GG GG GG GG GG GG GG
9350385
9333 rs10272338
7 -- -- -- -- -- -- -- --
-- -- -- -- -- -- -- --
-- -- -- -- -- -- -- --
-- -- -- -- -- -- -- --
-- -- -- -- 156882526
9334 rs10272379 7 AG AG AG AG GG
GG AG GG AG GG GG GG GG
GG GG GG GG AG GG AG GG
GG GG AG GG AG GG GG AG
AG AG GG GG AG AG GG
45277431
9335 rs1027238 3 AA AG AG AA AG
AG AA AA AA AA AA AA AA
AA AA AG AA AA AG GG AA
AA AG AG AA AA AA AA AA
AA AG AA AA AG AG AG
68330052
9336 rs10272408 7 GG GG GG GG GG
GG GG GG GG GG GG GG GG
GG GG GG GG GG GG GG GG
GG GG GG GG GG GG GG GG
GG GG GG GG GG GG GG
65801614
9337 rs1027242 3 AG AG AG GG GG
AG GG AA GG AG AG AG AG
AG AG GG GG AG AG AG GG
AA AA AG AA GG AA AG AG
AG GG GG GG AG AG AG
167578227
9338 rs10272439 7 GG GG AG GG AG
AG AG GG GG GG AG AG AA
AG GG GG AG AG AG AG AG

```
        GG      GG      GG      GG      GG      GG      GG      GG
        GG      AA      AG      AG      GG      AG      GG
67373364
9339    rs1027253       4       AG      GG      GG      AG      GG
        AG      GG      GG      GG      AG      GG      GG      GG
        GG      GG      GG      GG      AG      GG      GG      GG
        GG      AG      AG      GG      AG      GG      GG      AG
        GG      AG      AA      AG      GG      GG      GG
108117467
9340    rs10272533      7       AA      AA      AA      AA      AA
        AA      AA      AA      AA      AA      AA      AA      AA
        AA      AA      AA      AA      AA      AA      AA      AA
        AA      AA      AA      AA      AA      AA      AA      AA
        AA      AA      AA      AA      AG      AA      AG
92527551
9341    rs10272549      7       GG      GG      GG      AG      AG
        AA      GG      GG      GG      GG      GG      GG      GG
        AG      GG      AA      AG      AA      GG      GG      AG
        AG      GG      AG      GG      GG      GG      GG      GG
        AA      AG      AG      AG      GG      GG      AG
26116505
9342    rs10272564      7       GG      GG      GG      AG      AG
        AG      AG      GG      AG      GG      AG      AA      AG
        AG      GG      GG      GG      GG      AG      AG      AG
        GG      GG      GG      GG      GG      GG      GG      GG
        GG      AG      AG      AA      GG      GG      AG
148705490
9343    rs10272597      7       AG      AA      AG      AG      GG
        AG      GG      GG      GG      GG      AG      AG      GG
        GG      AG      AG      AG      AG      AG      GG      AG
        GG      AG      AG      GG      GG      --      AG      AG
        GG      AG      GG      AG      GG      GG      GG
121109271
9344    rs10272622      7       AG      AA      AG      AG      AA
        AA      AG      AG      AG      AG      AG      AG      GG
        AG      AG      AA      AA      AA      GG      AG      GG
        AG      AA      AA      AG      AG      AA      AA      AG
        AA      AG      AA      AG      AA      AA      AA
24076656
9345    rs10272625      7       GG      GG      GG      GG      GG
        GG      GG      GG      GG      GG      GG      GG      GG
        GG      GG      GG      GG      GG      GG      GG      GG
        GG      GG      GG      GG      GG      GG      GG      GG
        GG      GG      GG      GG      GG      GG      GG
38418891
9346    rs1027263       9       AG      GG      AG      GG      GG
        GG      GG      GG      GG      GG      AG      GG      AG
        AG      GG      AG      AG      AG      GG      GG      GG
        AG      AG      AG      AG      AG      AG      AG      AA
        AG      GG      AG      GG      GG      GG      GG
```

81776086
9347 rs10272635 7 AA AA AG AG AA
AG AA AA AG AG AA AA AA
AA AA AG GG AG AG AG GG
AA AG AA AG AA AA AA AA
AG AA AA AA GG GG AG
39874986
9348 rs1027266 8 AG AA AG AA AA
AA AA AG AG AG AG AG GG
GG GG AG AA GG AA AA AG
GG AG AG AG AG AG AA AG
AG AA AG AA AG AG AG
96425734
9349 rs1027269 9 AG AG AG AA GG
AG AG AG GG AG AG AG AG
AG AA AA AG AG AG AA AG
AG GG AG GG AG AA AA AA
AG AG AG AG AG AG GG
94368561
9350 rs10272701 7 AA AA AG GG GG
GG GG AG GG AG AG AG GG
GG GG AG AA AG AG AG GG
GG GG GG GG AG GG AG GG
AG GG AG AG GG GG GG
25911033
9351 rs10272732 7 GG GG GG GG GG
GG GG GG GG GG GG GG GG
GG GG GG GG GG GG GG GG
GG GG GG GG GG GG GG GG
GG GG GG GG GG GG GG
95104293
9352 rs10272763 7 GG GG GG GG GG
GG GG GG GG GG GG GG GG
GG GG GG GG GG GG GG GG
GG GG GG GG GG GG GG GG
GG GG GG GG GG GG GG
154097392
9353 rs1027277 16 CC AC CC CC CC
CC CC AC CC CC CC CC CC
CC CC CC AC CC AA AC AC
CC AC AC CC AA AC AA AC
AA AC AA AA AA AC AC
62684996
9354 rs10272782 7 AA AA AG AG AA
AG AA AA AA AG GG AG AG
AA AA AG AG AG AA AA AG
AA AG AG AA AA AA AA AA
AG AG AA AG AG AG AA
39623495
9355 rs10272816 7 AG GG GG AG AG

	AA	GG	AG	GG	AA	AG	AA	AG
	GG	GG	GG	GG	GG	AG	AG	GG
	GG	GG	AG	AG	GG	AA	AG	AG
	AG	GG	AA	AG	AG	AA	AG	
62360809								
9356	rs10272873		7	AA	AG	AG	AG	AG
	AG	AG	GG	GG	GG	GG	GG	GG
	AG	GG	GG	GG	GG	AG	GG	AG
	GG	GG	GG	GG	GG	GG	GG	GG
	GG	GG	GG	GG	GG	AG	AG	
96301696								
9357	rs10272888		7	GG	GG	GG	GG	GG
	GG	GG	GG	GG	GG	GG	GG	GG
	GG	GG	GG	GG	GG	GG	GG	GG
	GG	GG	GG	GG	GG	GG	GG	GG
	GG	GG	GG	GG	GG	GG	GG	
75570664								
9358	rs1027307		1	AG	GG	AG	AG	GG
	GG	AG	AG	AG	AG	AG	AG	AG
	GG	AG	AG	AG	AG	GG	GG	GG
	GG	AG	GG	AG	AA	AA	AA	AA
	GG	AA	GG	AG	AG	AG	GG	
95173343								
9359	rs1027313		12	CC	CC	CC	CC	CC
	AC	CC	CC	CC	CC	CC	CC	CC
	AC	AC	AC	AC	AC	AC	AA	AA
	AA	AC	AC	AC	AC	AA	AA	AA
	CC	AA	AC	AA	CC	CC	CC	
92593517								
9360	rs10273260		7	AG	AA	AG	AG	AG
	AG	AG	AA	AA	AA	GG	AG	AG
	AG	AG	AA	AG	AA	AA	GG	AG
	GG	AA	AG	AG	AA	AG	AA	AA
	AG	AA	AG	AG	AA	AA	AA	
43140213								
9361	rs10273271		7	AG	AG	GG	AG	GG
	GG	AG	GG	GG	GG	AG	AG	AA
	AG	AG	AA	AG	AG	GG	GG	GG
	AG	AG	GG	AG	GG	GG	GG	GG
	AG	GG	AG	AG	AG	GG	AA	
96847312								
9362	rs10273348		7	AA	AA	AA	AA	AA
	AA	AA	AA	AA	AA	AA	AA	AA
	AA	AA	AA	AA	AA	AA	AA	AA
	AA	AA	AA	AA	AA	AA	AA	AA
	AA	AA	AA	AA	AA	AA	AA	
96468641								
9363	rs10273411		7	AA	AC	AC	AC	CC
	AC	CC	AA	AA	AA	AC	AC	CC
	AC	AA	AC	AC	AC	AC	CC	AA

AC AC AA AA AA CC AC AC
AC AA AA AA AA AC AA
76928011
9364 rs10273435 7 AG AG AA AG AG
AG AG AG AG AG AG AA AG
AG AA AA AA AA AG AA AG
AG AA AA AG AA AG AA AA
AA AA AA AA AA AG AA
104067111
9365 rs10273453 7 GG GG GG AG GG
AG GG AA AA AA AG GG AG
AG GG AA AG AG GG AA GG
GG GG GG GG AG AG GG GG
AG AG GG GG GG AG AG
111732480
9366 rs1027348 5 AG GG GG AG GG
AG AG GG GG GG GG GG GG
GG GG GG GG GG GG GG GG
GG GG GG GG GG GG GG GG
AG GG GG GG AG GG AG
41786266
9367 rs10273526 7 AA AA AA AA AA
AA AA AG AG AG AA AA AA
AA AA AA AA AA AA AA AA
AA AA AA AA AA AA AA AA
AA AA AA AA AA AA AA
101491772
9368 rs10273608 7 GG GG GG GG GG
GG GG GG GG GG AG AG GG
GG GG GG GG GG GG GG GG
GG AG AG GG GG GG GG GG
GG AG GG GG GG GG GG
93758613
9369 rs10273644 7 AA AA AA AA AA
AA AA AA AA AA AA AA AA
AA AA AA AA AA AA AA AA
AA AA AA AA AA AA AA AA
AA AA AA AA AA AA AA
1940906
9370 rs10273658 7 AG GG AG AG AA
AA AG AG GG AG AG AG AA
AA AA GG GG GG -- GG GG
AG GG GG AG GG AG AG AG
GG AA GG AG AG GG AG
66452054
9371 rs10273681 7 AA AC AC AA AA
AA AA AA AA AA AA AA AA
AA AA AC AC AC AA AA AA
AA AA AA AA AA AA AA AA
AA AA AA AA AA AA AA

71211570
9372 rs10273694 7 AA AA AG AG AG
AA GG AG AG GG AA AG AG
AA AA GG GG GG AG GG AG
AG GG GG AG AG GG AG AG
GG AG AG GG GG GG AG
39194416
9373 rs1027384 9 AG AA AG AG AA
AA AG AA AA AA GG AG GG
GG GG AG AA AA AG AG AG
AG AA AG AA AG AA AG AA
AG AG AA AA AG AG AG
78932693
9374 rs10273853 7 AA AA AA AA AA
AA AA AA AA AA AG AG AG
AG AA AA AA AA AA AA AA
AA AA AA AA AA AA AA AA
AA AA AG AG AA AA AA
86863060
9375 rs10273864 7 AC AC CC CC CC
CC AC AA AA AC AC AC CC
AC AC AA AC AC AC AC AA
CC AC AA AC AA AC AC AC
CC AC AC AC CC AC CC
77259283
9376 rs1027388 11 AC AA AC AC AC
AA AC CC AC AC CC AC AC
CC AC CC CC CC CC CC CC
CC AA AC AC AC CC AC AC
AC AA AC AC AA AC AC
40619828
9377 rs10273902 7 GG GG GG GG GG
GG GG GG GG GG GG GG GG
GG GG GG GG GG GG GG GG
GG GG GG GG GG GG GG GG
GG GG GG GG GG GG GG
36562244
9378 rs10273912 7 AG GG GG AG AG
AG GG AG AG AG AA AG AG
GG AG GG AG AG AA AA AG
AG GG AG AG AG AG AG AG
AG AG AA AG AG AA AG
148022695
9379 rs10273927 7 AA AA AA AA AA
AA AA AA AA AA AA AA AA
AA AA AA AA AA AA AA AA
AA AA AA AA AA AA AA AA
AA AA AA AA AA AA AA
101462011
9380 rs10274003 7 AA AA AA AA AA

AA AA AA AA AA AA AA AA
AA AA AA AA AA AA AA AA
AA AA AA AA AA AA AA AA
AA AA AC AA AA AA AA
50933678
9381 rs10274012 7 AG AG GG AA AG
AG AA AA AA AA -- GG GG
AA AG AG AG AA AA AA AA
AA AG AA AG AA AA AA AA
AG GG AA AG AG GG AA
3540241
9382 rs10274025 7 GG AG GG GG GG
GG GG GG GG GG AG AG GG
GG GG GG GG GG GG GG GG
GG AG GG GG AG GG GG GG
GG GG GG GG GG GG GG
153967280
9383 rs10274031 7 AG GG AG GG AG
GG AG GG GG GG GG AG AG
GG AG GG AG GG GG GG GG
GG AG AG GG AA AG AG AG
GG AG GG AG GG GG GG
21680751
9384 rs10274082 7 AA AA AA AA AG
AG AA AG AA AG AA AA AA
AA AA AA AA AA AA AA AA
AG AA AA AG AG AA AA AG
AG AA AG -- AG AG AA
42575827
9385 rs10274186 7 GG GG GG GG GG
GG GG GG GG GG GG GG GG
GG GG GG GG GG GG GG GG
GG GG GG GG GG GG GG GG
GG GG GG GG GG GG GG
75221658
9386 rs10274211 7 AA AA AA AG GG
AG GG GG AG AG GG GG GG
GG GG GG GG GG AG AG GG
GG GG GG GG AG GG GG AG
AG AG AG AG AG GG AG
41179594
9387 rs10274222 7 AG GG GG AG GG
AG GG GG GG GG GG GG GG
GG GG GG GG GG GG GG AG
GG GG GG GG GG GG GG GG
GG AG GG GG AA AG AG
76958730
9388 rs10274279 7 AA AA AA AA AA
AA AA AA AA AA AA AA AA
AG AG AA AA AA AG AG AG

AA AA AA AA GG AG GG GG

AG AA AA AA AA AA AA

157387441

9389 rs10274328 7 GG AG AG GG GG

GG GG GG GG GG GG GG GG

GG GG GG GG GG GG GG AG

GG GG GG GG AG GG AG AG

GG AG AG AA GG GG GG

133409403

9390 rs10274363 7 AA AA AA AA AA

AA AA AA AA AA AA AA AA

AA AA AA AA AA AA AA AA

AA AA AA AA AA AA AA AA

AA AA AA AA AA AA AA

64029281

9391 rs1027438 7 AG AA AG GG GG

GG GG AG GG AG GG AG AG

AG AG AG AG AG AG AG GG

AG GG GG AG AA GG AG AG

AG GG GG GG GG GG AG

45909998

9392 rs10274412 7 AG GG AG GG AG

GG AG GG GG GG GG AG AG

GG AG GG AG GG GG GG GG

GG AG AG GG AG AG AG GG

GG AG GG AG GG GG GG

21680935

9393 rs10274448 7 CC CC CC CC CC

CC CC CC CC CC CC CC CC

CC CC CC CC CC CC CC CC

CC CC CC CC CC CC CC CC

CC CC CC CC CC CC CC

19522934

9394 rs10274467 7 GG GG GG GG GG

GG GG AG AG AG GG GG GG

GG GG GG GG AG GG GG GG

GG GG GG GG GG GG GG GG

GG GG GG GG GG GG GG

5448407

9395 rs10274504 7 AA AA AA AG AG

AG AG AA AA AA AA AA AA

AA AG AG AG AG AA AA AA

AA AA AA AA AA AG AG AG

AA AA AA AA AA AA AA

82665830

9396 rs1027454 11 AC AC CC CC CC

CC CC AA CC CC AC AC CC

AC CC AC AC AC AC AC AA

AC AC AC AC CC CC CC CC

AC AC AC CC AC AA AC

91834246
9397 rs10274562 7 AG AA AG AA AG
AG AA AA AA AA AG AG AG
AG AG AA AA AA AG AA AG
AA AA AA AA AG AG AA AA
AG AA AA AA AG AG AG
11222521
9398 rs1027457 16 CC CC CC CC CC
CC CC CC CC CC AA AA AA
AC AC CC AC AC CC CC CC
AC CC AC AC AC CC CC AC
CC CC CC CC CC CC AC
20781194
9399 rs10274639 7 GG GG GG AG AG
AG GG GG GG GG AG GG AG
AG AG AG GG GG AA GG AG
GG AG GG GG GG GG GG GG
GG AG AG AG GG AG GG
156757453
9400 rs10274707 7 AG AA AG AG AG
GG AA GG GG AG GG AG AG
AG AG GG GG GG GG GG AG
AG AG AG AG AA AA AA AA
GG AG GG GG AG AG GG
79780292
9401 rs1027472 4 AA AA AA GG GG
AG AG GG GG AG GG GG AG
AG AG AA AA AA GG GG GG
AG AG AG AG AG AG GG AG
AG AG GG GG AA AG AG
140552399
9402 rs10274751 7 AA AA AG AA AA
AA AA AA AA AG AA AA AA
AA AA AA AG AG AA AA AA
AA AA AA AA AA AG AA AA
AA AA AA AA AA AA AG
71808745
9403 rs10274796 7 AA AA AG AG AG
AA GG AA AG AA GG GG GG
AA AA AG AG AA AA AG AG
GG AG AG GG AA AG AA AA
AG AA AA AA AA AA AA
43070454
9404 rs10274825 7 AA AA AA AC AC
CC AA AA AA AA AA AA AA
AC AC AA AA AA AA AA AA
AA AA AA AA AA AA AA AA
AA AA AA AA AA AA AA
31631594
9405 rs1027486 15 GG AG AG AA AA

AA AG GG AG AG AG AA AA
AG AA AG AA GG GG AA AG
AG AG AG AG GG AA AG AG
AA GG AG AG AA AG AG
36059349
9406 rs10274861 7 GG GG AG GG AG
AG GG AG GG AG GG GG GG
GG GG AG GG GG GG AG GG
GG GG GG GG GG GG GG GG
GG GG GG GG GG GG GG
64339349
9407 rs10274869
7 -- -- -- -- -- -- -- --
-- -- -- -- -- -- -- --
-- -- -- -- -- -- -- --
-- -- -- -- -- -- -- --
-- -- -- -- 4427374
9408 rs10274872 7 GG GG GG GG GG
GG GG GG GG GG GG GG GG
GG GG GG GG GG GG GG GG
GG GG GG GG GG GG GG GG
GG GG GG GG GG GG GG
1285662
9409 rs10274882 7 AA AA AA AA AG
AG AA AA AA AA AA AA AA
AA AA AA AA AA AG AA AG
AA AA AA AA AA AA AA AA
AG AA AA AA AG AG AG
42466264
9410 rs10274990 7 GG GG GG GG GG
GG GG GG GG GG AG AG GG
GG GG GG GG GG AA GG AG
AG AA AA AG AG GG AG AG
GG GG GG GG AG GG AA
109409473
9411 rs10275022
7 -- -- -- -- -- -- -- --
-- -- -- -- -- -- -- --
-- -- -- -- -- -- -- --
-- -- -- -- -- -- -- --
-- -- -- -- 54322252
9412 rs10275054 7 AA AA AA AG AA
AA AG AA AA AA AA AG AG
AG AG AA AA AA AG AA AG
AG AA AA AG AG AG AG GG
AG AA AG AA AA AA AA
49625207
9413 rs10275082 7 GG GG GG GG AG
AG GG AG AG GG GG GG GG
GG GG GG GG GG AA AG AG

GG AG AG GG AG GG AG GG
AG GG GG GG GG GG GG
42311183
9414 rs10275088 7 AG AA AG AA AA
AG AA AG AG GG AG GG AG
AG AG AG GG GG AA AA AG
AA AA AA AA GG AG GG GG
AG AG AG GG AG AG AA
155860445
9415 rs10275110 7 AG GG GG GG GG
GG GG GG GG GG GG GG GG
GG GG GG GG GG GG AG GG
GG GG GG GG GG AG GG GG
GG GG GG GG GG GG GG
71165833
9416 rs10275126 7 GG GG GG GG GG
GG GG GG GG GG GG GG GG
GG GG GG GG GG GG GG GG
GG GG GG GG GG GG GG GG
GG GG GG GG GG GG GG
54605422
9417 rs10275178 7 AA AA AA AA AA
AA AA AA AA AA AA AA AA
AA AA AA AA AA AA AA AA
AA AA AA AA AA AA AA AA
AA AA AA AA AA AA AA
51258968
9418 rs10275267 7 AA AA AA AG AA
AG AA AA AA AA AG AA AG
AG AA AA AA AA AA AA AG
AA AA AA AA AA AA AA AA
AA AA AA AA AA AA AA
97774300
9419 rs10275279 7 AA AA AA AA AG
AG AA AG GG AG GG GG AG
AA AA AA AA AG AA AA AA
AG AA AG AA AA AA AA AA
AA AG AG AG AG AG AA
30141435
9420 rs10275282 7 GG AG AG AG GG
GG AG AG GG AA AA AG AG
GG GG AG GG GG AG GG GG
GG GG GG GG AG AG GG AG
GG AG GG AG AG GG AG
55382925
9421 rs10275303 7 AA AG AG AG AA
AG AA AA AA AA AG AG AA
AA AA AA AG AG AG AG AA
AA AG AA AA AA AA AA AA
AA AA AA AA AG GG AA

134397507
9422 rs10275341 7 AA AA AA AG AG
AG AA AA AA AA AA AA AA
AA AA AA AA AA AA AA AA
AA AG AA AA AA AA AA AA
AA AA AA AA AA AA AA
158710642
9423 rs10275366 7 AA AA AA AA AA
AA AA AA AA AA AA AA AA
AA AA AA AA AA AA AA AA
AA AA AA AA AA AA AA AA
AA AA AA AA AA AA AA
54346673
9424 rs10275374 7 GG GG GG GG GG
GG GG GG GG GG GG GG GG
AG AG GG GG GG GG GG GG
GG GG GG GG GG GG GG GG
GG GG AG GG GG GG GG
158147706
9425 rs10275409 7 AA AA AA AA AA
AA AA AA AA AA AA AA AA
AA AA AA AA AA AA AA AA
AA AA AA AA AA AA AA AA
AA AA AA AA AA AA AA
93282831
9426 rs1027544 5 CC CC CC CC CC
CC AC CC CC CC CC AC CC
CC CC CC CC CC AC AC AC
CC CC CC CC CC CC CC CC
CC CC CC CC CC CC CC
21047987
9427 rs10275462 7 AG AG AA AA AG
AG AA AG GG AA GG GG GG
GG GG AA AG AA AA AG AA
AG AG AG AG GG GG GG GG
AG AG AG AG AG AG AA
36751049
9428 rs10275469 7 AA AA AA CC CC
AC CC AA AA AC AA AC AA
AA AA AA AA AA AC AC AC
AC AC AC AC AA AA AA AA
AA AA AC AC CC AC AC
139113
9429 rs10275488 7 GG GG GG GG GG
GG GG GG GG GG GG GG GG
GG GG GG AG GG GG GG AG
AG GG GG GG GG GG GG GG
GG GG GG GG GG GG GG
17303778
9430 rs10275546 7 AA AA AA AA AA

AA AA AA AA AA AA AA AA
AA AA AA AA AA AA AA AA
AA AA AA AA AA AA AA AA
AA AA AA AA AA AA AA
54465770
9431 rs10275570 7 AA AA AA AA AA
AA AA AA AA AA AA AA AA
AA AA AA AA AA AA AA AA
AA AA AA AA AA AA AA AA
AA AA AA AA AA AA AA
19484937
9432 rs10275628 7 GG GG GG GG GG
GG GG AG AG AG GG GG GG
GG GG GG AG GG AG AG GG
GG GG GG GG AG GG AG GG
GG AG GG GG GG GG GG
22604319
9433 rs10275635 7 AG GG AG AG GG
GG AG GG GG GG GG GG GG
GG GG GG GG GG GG GG GG
GG GG GG GG GG GG GG GG
GG GG GG GG GG GG GG
95265377
9434 rs10275686 7 AA AA AA AA AA
AA AA AA AA AA AA AA AA
AA AA AA AA AA AA AA AA
AA AA AA AA AA AA AA AA
AA AA AG AA AA AA AA
9085905
9435 rs10275721 7 AA AA AA AA AC
AC AA AA AA AA AA AA AA
AA AA AA AA AA AA AA AA
AA AA AA AA AA AA AA AA
AA AA AA AA AC AC AA
69290396
9436 rs1027587 19 GG GG GG GG GG
GG GG GG GG GG GG GG GG
GG GG AG AG GG GG GG GG
GG GG GG GG GG GG GG GG
GG GG GG GG GG GG GG
21658045
9437 rs10275897 7 AA AG AA AA AA
AA AA AA AA AA AA AA AG
AA AA AA AG AA AA AA AG
AA AG AG AA AG AG AG AG
GG AA AG AA AA AA AA
31812263
9438 rs10275929 7 GG GG GG GG GG
GG GG AG AG AG GG GG GG
GG GG GG AG GG GG AG GG

	GG	AG	AG	GG	AG	AA	AA	AG
	GG	AG	AA	AA	GG	GG	AG	
22473556								
9439	rs10275948		7	GG	GG	GG	GG	GG
	GG	GG	GG	GG	GG	GG	GG	GG
	GG	GG	GG	GG	GG	GG	GG	GG
	GG	GG	GG	GG	GG	GG	GG	GG
	GG	GG	GG	GG	GG	GG	GG	
99210835								
9440	rs10275972		7	AG	AG	AA	AG	AG
	AA	AG	AA	AA	AA	AA	AA	AA
	AA	AA	AG	AA	AA	AA	AA	AG
	AA	AA	AA	AA	AA	AA	AA	AA
	GG	AA	AA	AA	AA	AG	AA	
118769366								
9441	rs10276006		7	AC	CC	AC	CC	CC
	CC	CC	CC	CC	CC	CC	CC	CC
	CC	CC	CC	CC	CC	CC	CC	CC
	CC	CC	CC	CC	CC	CC	CC	CC
	CC	CC	CC	CC	CC	CC	CC	
16973077								
9442	rs10276022		7	AA	AG	AG	GG	AA
	AG	AG	AG	AG	AG	AA	AA	AG
	GG	GG	AA	AG	AG	GG	GG	AG
	AA	GG	AG	GG	GG	GG	GG	GG
	AG	AA	AG	AG	GG	AG	GG	
4560681								
9443	rs10276031		7	AA	AA	AA	AA	AA
	AA	AA	AA	AA	AA	AA	AA	AA
	AG	AG	AA	AA	AA	AA	AA	AA
	AA	AA	AA	AA	AA	AA	AA	AA
	AA	AA	AG	AA	AA	AA	AA	
157618692								
9444	rs1027604		10	GG	GG	GG	AG	AA
	AA	AG	AG	AG	AG	AG	AG	AG
	AA	AG	AG	AG	AA	AG	AG	AG
	AA	AA	AG	AA	AG	GG	GG	GG
	GG	AG	AA	AG	AA	AA	AG	
1335849								
9445	rs1027606		15	AG	AG	GG	GG	GG
	AG	GG	AG	AA	AG	GG	GG	GG
	AA	GG	AG	GG	AG	AG	GG	AA
	AG	AG	AG	AG	GG	AG	GG	GG
	AG	AG	GG	AG	GG	AG	AG	
53715879								
9446	rs1027617		11	AG	AG	AA	AA	AA
	AA	AA	AG	AA	AA	AG	AG	AG
	GG	GG	AA	AA	AA	GG	GG	AG
	AG	AG	AG	AG	AG	AG	AG	AG
	GG	GG	AG	AG	AA	AG	AG	

16842787
9447 rs10276222 7 AA AA AA AA AA
AA AA AA AA AA AG AG AA
AA AA AA AA AA AA AA AA
AA AA AA AA AA AA AA AA
AA AA AA AA AG AG AA
94577529
9448 rs10276241 7 GG GG GG AG GG
GG AG GG GG GG GG GG GG
GG AG GG GG GG GG GG GG
GG GG GG GG GG GG GG GG
GG GG GG GG AG GG AG
81273423
9449 rs10276265 7 AG AA AG AA AA
AA AA AA AA AA AA AA AG
AA AG AA AA AA AA AA AA
AA AA AA AA AG AG GG GG
AA AA AA AA AA AA AA
4920499
9450 rs10276300 7 CC CC CC AC AC
CC AC CC CC CC AC CC AC
CC CC CC CC CC AC AC AC
AC AC AC AC CC CC CC CC
AC CC CC CC CC CC CC
154233833
9451 rs1027635 5 AG AA AG AA AA
AG AA AA AA AA AA AA AG
AA AG AA AA AA AA AA AA
AA AA AA AA AA AA AA AA
AA AA GG AG AG AG AA
166443856
9452 rs10276351 7 AA AA AG AG GG
AG GG GG GG GG AA AG AG
AG AG GG GG AG AG AG AG
GG AG AG GG GG GG GG GG
GG AG AA AA GG AG GG
47784994
9453 rs10276369 7 GG GG GG GG GG
GG GG GG GG GG GG GG GG
GG GG GG AG AG AG GG AG
GG AG AG GG GG GG GG GG
GG GG GG GG GG GG GG
111976546
9454 rs10276390 7 AA AA AA AA AA
AA AA AA AA AA AG AG GG
GG GG AG AA AA AG AA GG
AA AA AA AA AA AA AA AA
AA AA AA AA AA AA AA
120109606
9455 rs10276393 7 AA AA AA AA AA

AA AA AA AA AA AA AA AA
AA AA AA AA AA AA AA AA
AA AA AA AA AA AA AA AA
AA AA AA AA AA AA AA
64200641
9456 rs10276414 7 AA AA AA AA AA
AA AA AA AA AA AA AA AA
AA AA AA AA AA AA AA AA
AA AA AA AA AA AA AA AA
AA AA AA AA AA AA AA
40935574
9457 rs1027643 5 GG GG GG GG GG
GG GG AG AA AA GG GG GG
GG GG GG GG GG GG GG GG
GG GG GG GG GG GG GG GG
GG GG GG GG GG GG GG
91893792
9458 rs10276432 7 AC AC AC AA AC
AA AC AA AC AC CC CC AC
AC AC AA AC AA AA AC AC
AA CC AC AC AA CC AC AC
AA AA AA AA AA AA AC
34017577
9459 rs10276437 7 AA AA AG AA AA
AA AA AA AG AA AA AA AA
AA AA GG AG AG AA AA AA
AA AA AG AA AG AA AG AA
AA AA AA AA AA AA AG
47806077
9460 rs10276445 7 AA AA AA AG AG
GG AG AG GG GG AG AG AA
AA AA AA AA AA AA AA AA
AG AA AG AG AA AA AA AA
AA AG AG AG AA AA AG
152540119
9461 rs10276467 7 AG GG AG GG GG
GG GG GG GG GG GG GG GG
GG GG GG GG AG GG GG GG
GG GG GG GG GG GG GG GG
GG AG GG AG GG GG GG
2734713
9462 rs10276544 7 AA AC AC CC CC
CC CC AC AA AC AA AC AC
AC AC CC CC CC CC CC CC
CC AC AC CC CC CC CC CC
AC AC AC AC CC AC CC
38550840
9463 rs10276579 7 GG GG GG GG GG
GG GG GG GG GG GG GG GG
GG GG GG GG GG GG GG AG

GG GG GG GG GG GG GG GG
GG GG GG GG GG GG AG
101109073
9464 rs10276619 7 AA AA AA AG AA
AG AA AG AA AG GG AG AG
GG GG AG AG AG GG AG AG
AA AG AG AA AA AA AA AA
AA AG AA AA AA AA AG
50313352
9465 rs10276643 7 GG GG GG GG GG
GG GG GG GG GG GG GG GG
GG GG GG GG GG GG GG GG
GG GG GG GG GG GG GG GG
GG GG GG GG GG GG GG
1495208
9466 rs10276674 7 AG AA AG AG AG
AA AG AA AA AA AA AA AA
AA AA AA AA AG AA AA AG
AA AG AA AG AG AA AA AG
AA GG AG GG AG AA AG
14922007
9467 rs10276899 7 CC CC CC CC CC
CC CC CC CC CC CC CC CC
CC CC CC CC CC CC CC CC
CC CC CC CC CC CC CC CC
CC CC CC CC CC CC CC
11061524
9468 rs1027695 3 AA AG AA AA AA
AA AA AG AG AG GG GG AG
AA AG AA AA AA AA AA AG
AA AA GG AA AG AG AA GG
AG AA AA AA AG AA AG
147215564
9469 rs10276962 7 AG AA AG AG GG
AG GG AA AG AA AA AA AA
GG AG GG GG AG AG AG GG
GG AG GG AG AG GG AG GG
GG GG AA AG GG GG GG
25076112
9470 rs10277 5 AG GG AG AG AG AG
AG AG GG AG GG GG AG AA
AA AG AG GG AA AA AG GG
GG GG GG GG AG AG AG AA
GG AA AG GG AG GG 179264731
9471 rs10277002 7 AA AC AA AC AC
CC AA AA AA AA AA AA AA
AA AA AA AA AA AA AA AA
AA AC AC AA CC AA AC AC
AC AA AA AA AA AA AC
30906193

9472 rs10277008 7 GG GG GG GG GG
GG GG GG GG GG GG GG GG
GG GG GG GG GG GG GG GG
GG GG GG GG GG GG GG GG
GG GG GG GG GG GG GG
101620678
9473 rs10277012 7 GG GG GG GG AG
AG GG GG GG GG GG GG GG
GG GG GG AG GG AA AG AG
GG GG GG GG GG GG GG GG
GG AG AG AG GG GG AG
21298242
9474 rs1027702 1 AA AA AA GG AA
AG AG AA AG AA AG AG AG
AA AA AG GG GG AG AG AA
AA GG AG GG GG GG GG GG
AG AG AG GG AG AA AG
161712857
9475 rs10277069 7 GG GG GG GG GG
GG GG GG GG GG GG GG GG
GG GG AG AG AG GG GG GG
GG AG GG GG GG GG GG GG
GG GG GG GG AG AG GG
151755007
9476 rs10277108 7 GG GG GG GG GG
GG GG GG GG GG GG GG GG
GG GG GG GG GG GG GG GG
GG GG GG GG GG GG GG GG
GG GG GG GG GG GG GG
99688144
9477 rs10277163 7 AG GG AG AA AG
AA AG AG AA AG AA AA AA
AA AA AA AG AG AG AG AG
AG AA AG AA AA AA AA AA
AA AA AA AA AG GG AA
21569089
9478 rs1027719 17 GG GG GG AG AG
AG GG AG AG AG AA AA AG
AG AG AG GG AG AG GG AA
GG GG AG AG AG GG GG AG
GG GG AG AG GG GG GG
72597739
9479 rs10277203 7 AA AA AA AA AA
AA AA AA AA AA AA AA AA
AA AA AA AA AA AA AA AA
AA AA AA AA AA AA AA AA
AA AA AA AA AA AA AA
8506059
9480 rs10277243 7 AG GG GG AG AG
AG AG AG AG AG GG GG GG

GG GG GG GG GG AG GG GG
GG GG GG GG AA AG AG AG
GG GG GG GG GG GG GG
145768088
9481 rs10277378 7 GG GG GG GG GG
GG GG GG GG GG GG GG GG
GG GG GG GG GG GG GG GG
GG GG GG GG GG GG GG GG
GG GG GG GG GG GG GG
100037209
9482 rs10277410 7 CC CC CC CC CC
CC CC CC CC CC CC CC CC
CC CC CC CC CC CC CC CC
CC CC CC CC CC CC CC CC
AC AC AC CC CC CC CC
122447312
9483 rs10277422 7 AG AG AG AA AG
AG AA AG AA GG AA AA AA
AA AA AA AA AA AG AG AG
AA AG AG AA AG AA AG AG
AA AG AG GG AA AG AA
40117098
9484 rs10277430 7 AG GG GG GG GG
GG GG GG GG GG GG GG GG
GG GG GG GG GG AG AG AG
AG GG AG AG GG GG GG GG
AG AG AG AG GG GG GG
141087803
9485 rs1027745 5 GG AG AG GG GG
GG GG GG GG GG GG GG GG
GG GG GG GG GG GG GG GG
AG AG AG AG GG GG GG GG
GG GG GG GG GG GG AG
172106310
9486 rs10277496 7 GG GG GG GG GG
GG GG GG GG GG GG GG GG
GG GG GG GG GG GG GG GG
GG GG GG GG GG GG GG GG
GG GG GG GG GG GG GG
64204328
9487 rs10277506 7 AA AA AG AA AA
AA AA AA AA AA AA AA AA
AA AA AG AG AG AA AA AA
AA AA AA AA AA AA AA AA
AG AA AA AA AA AA AA
36253012
9488 rs10277544 7 GG AG AG AG AG
AG AG AG AA AG AA AG AG
GG GG AA AA AA AA AA AA
AG AA AG AG AA AG AA AA

AA AG AA AA AG GG AA
151540633
9489 rs10277562 7 AA AA AA AA AA
AA AA AA AA AA AA AA AA
AA AA AA AA AA AA AA AA
AA AA AA AA AA AA AA AA
AA AA AA AA AA AA AA
69798648
9490 rs10277718 7 AA AA AA AA AA
AA AA AA AA AA AA AA AA
AA AA AA AA AA AA AA AA
AA AA AA AA AA AA AA AA
AA AA AA AA AA AA AA
151776615
9491 rs1027772 5 AG AA AA GG GG
AG GG AG AG AG AG AA AG
AG AG GG GG GG AG AG AG
AG AG AG AG AA AA AA AA
AG GG AG GG AA AA AA
39619119
9492 rs10277771 7 AA AA AA AA AA
AA AA AG AG AG GG AG AG
AG AG AA AG AA AG AG AA
GG AA AG AA AA AG AG AG
AG AA AG AG AA AA AA
4004884
9493 rs10277774 7 AA AA AA AA AA
AA AA AG AG AA AA AG AG
AG AA AG AG AG AG AG GG
AG AA AG AG GG AG GG GG
AA AG AG GG AA AA AG
150588800
9494 rs10277837 7 AA AA AA AA AA
AA AA AA AA AA AA AG AG
AG AG AA AA AA AA AA AG
AG AG AG AG AG AG AG GG
AA AA AA AA AA AA AA
49365151
9495 rs1027784 4 AG GG GG AG GG
AG AG AA AG AG AG AG AG
AG AA AG AA AG AG AG AG
AG GG AG AA AG GG AG AG
AG AG AG AG GG GG GG
16666332
9496 rs10277859 7 AA AA AA AA AA
AA AG AA AG AA AG AA AG
AA AA AA AA AA GG GG AG
AG AA AG AG AG AG GG GG
AA AG AA AA AA AA AA
151387197

9497 rs10277895 7 AA AA AA AA AA
AA AA AA AA AA AA AA AA
AA AA AA AA AA AA AA AA
AA AA AA AA AA AA AA AA
AA AA AA AA AA AA AA
90870373
9498 rs10277927 7 AA AA AA AA AG
AA AG AA AA AG AA AA AA
AA AA AG AG AG AA AG AG
AG AA AA AG AA AA AA AA
AA GG AG GG AA AA AA
52516374
9499 rs1027797 11 AA AA AA AG AA
AG AA AA AA AA AA AA AA
AA AA AA AA AA AA AA AA
AA AA AA AA AA AG AG AA
AA GG AA AG AA AA AA
132757774
9500 rs1027802 8 GG GG GG GG GG
GG AG GG GG GG AG AG AG
AG AG GG GG GG GG AG GG
GG GG GG GG GG GG GG GG
GG AG GG AG GG GG GG
18975171
9501 rs1027804 8 AG AA AG AG AG
AG GG AG GG GG GG GG AG
AG GG GG GG GG GG GG GG
AG AG AA GG AG GG AG GG
GG GG AA AG GG GG GG
18919857
9502 rs10278046 7 AA AA AC AA AA
AA AA AA AA AA AA AA AA
AA AA AA AA AA AA AA AA
AA AA AA AA AA AA AA AA
AA AA AA AA AA AA AA
155901297
9503 rs10278047 7 GG GG GG GG GG
GG GG GG AG GG GG GG GG
GG GG GG GG GG AG GG AG
GG GG AG AG GG AA AG AG
GG GG AG AG GG AG AG
52104548
9504 rs10278073 7 GG GG GG AA AA
AG AA AG AG AG GG GG GG
AG AG GG GG GG GG AG GG
GG GG GG GG GG AG AG AG
GG AA AG AA GG GG GG
151662184
9505 rs10278107 7 AA AA AA AA AA
AA AG AA AA AA AA AA AA

AA AG AA AA AA AA AG AA
AA AG AG AA AA AG AA AA
AG AA AA AA AG GG AA
125278195
9506 rs10278126 7 GG GG GG GG GG
GG GG GG GG GG GG GG AG
GG GG GG GG GG GG GG AG
GG GG GG GG GG GG GG GG
GG GG GG GG GG GG GG
33772241
9507 rs10278139 7 GG GG AG AG AG
AG AG AG GG AG AA AA AA
AG AG AA AG AA GG GG GG
GG AG AG GG AG AA AG AA
GG GG AA AG GG GG AG
63681328
9508 rs10278176 7 AA AA AA AG AG
AG AG AG AG AA AG AG AA
AA AA GG GG GG AA AA AA
AA AG AG AA AG AG AG AA
AG AA AG AA AG AA AG
94104205
9509 rs10278248 7 AA AC AC AA AA
AA AA AC AC AC AA AC AC
AC AC AA AA AA AC AA CC
AC AA AA AC CC AA AC AC
AC AA AA AA AA AA AA
112802475
9510 rs10278268 7 GG AG GG GG AG
GG AG AG AG AG AG AG GG
GG GG GG AG AG GG GG GG
GG GG GG GG GG GG GG GG
GG AG AG AG AG AG GG
23802003
9511 rs1027832 3 AG GG AG AA GG
AG AG GG GG AG AG AG AA
AG AG GG GG GG AG AG GG
AG GG GG AG AG AG AG GG
AG AG GG AG GG AG GG
78663956
9512 rs10278362 7 AA AA AA AG AG
AG AG GG GG AG AA AA AA
AA GG AG AG AG AG AA GG
AG GG GG AG AA GG AG AG
AG AA AG AG AG AA AG
81029279
9513 rs1027837 11 AG GG AG AG AG
AG GG AG AG AG GG GG GG
AG AG GG AG -- AA AA AG
GG GG AG AG AA GG AG AG

AG AG AG AA GG AG AG
13946865
9514 rs10278373 7 GG GG GG GG GG
GG GG GG GG GG GG GG GG
GG GG GG GG GG GG GG GG
GG GG GG GG GG GG GG GG
GG GG GG GG GG GG GG
46280490
9515 rs1027840 3 GG AG GG GG GG
GG GG GG GG GG GG AG AG
AG AA GG GG GG GG AG GG
GG GG GG GG GG GG GG GG
GG AG GG GG GG GG AG
36859083
9516 rs10278457 7 AG AA AA AA AA
AA AG AA AA AA AA AA AA
AA AA AG AA AA AA AA AA
AA AA AA AA AA AA AA AA
AA AA AA AA AA AA AA
152859520
9517 rs10278498 7 AA AA AA AA AA
AA AA AA AA AA AA AA AA
AA AA AA AA AA AA AA AA
AA AA AA AA AA AA AA AA
AA AA AA AA AA AA AA
53750447
9518 rs10278501 7 CC AC CC AA AC
AC AA AC AC CC AC AC AA
AA AA AC AC AC AC AA AC
AC AA AA AA AC AC AC AA
AC CC AC AC AA AC AC
15389221
9519 rs10278502 7 AG GG GG AA AA
AG AA GG AG GG AG AG GG
GG GG GG GG GG GG GG AG
GG AG GG GG GG GG GG GG
GG GG GG GG GG GG GG
148044472
9520 rs10278521 7 AG AA AG AA AA
AA AA AA AA AA AA AA AA
AA AA AA AA AA AA AA AG
AA AA AA AA AA AA AA AA
AA AA AA AA AA AA AG
96320830
9521 rs10278546 7 AA AA AA AA AC
AA AC AA AA AA AA AA AA
AA AA AA AA AA AA AC AC
AC AA AA AC AA AC AA AA
AA AC AC AC AC CC AA
100516003

9522 rs10278557 7 GG AG GG AG GG
GG AG GG GG GG GG GG GG
AG AA AG AG GG AG AG AG
GG AG AG AG GG GG GG GG
AG AA GG AG AG AG GG
15699036
9523 rs10278615 7 AG AG GG AG AA
AG AA AG GG AG AG AG AA
AA AA AA AG AG AA AA AA
GG AA AG AG AA AA AA AA
AA AA AG AG AG AG AA
27950576
9524 rs10278661 7 AG AG AG GG AG
AG GG AG AA AA GG GG GG
AG AG GG AG AG AA AA AA
AG GG GG AG AA AA AA AA
GG AA AG AA AG AG AG
31361612
9525 rs10278673 7 GG AG AG AG AG
AA GG AG GG AG AG AG GG
AG AG GG GG GG AG AG GG
GG GG GG GG GG GG GG GG
AA GG AG GG GG GG GG
150518393
9526 rs10278728 7 GG GG GG AG AG
AG AG GG GG GG GG AG AG
AG AG AG GG GG GG AA GG
AG AG AG AG GG AG GG GG
AG AG AG AG GG GG GG
54274831
9527 rs10278745 7 AG AG AA AA AG
AA AG AG AA AG AA AA AA
AA AA AG AG AG AG AA AA
AG AG AG AG AG AG AA AG
AA AG AA AA AA AG AG
48439043
9528 rs10278782 7 AA AA AA AG AA
AG AA AA AA AA AA AA AA
AA AA AG AG AG AG AG AG
AG AA AA AG AA AG AA AA
AA AA AA AA GG AG AG
116147401
9529 rs10278878 7 AA AA AA AA AA
AA AA AA AA AA AA AA AA
AA AA AA AA AA AA AA AA
AA AA AA AA AA AA AA AA
AA AA AA AA AA AA AA
54305125
9530 rs10278888 7 AG AG AA AA GG
AG AG AG AG GG GG GG AG

AA AG AA AG AG AG AG AA
AG GG AG AG AG AG AG AG
AG AA AG AA AA AA AA
95755635
9531 rs10278891 7 CC AC CC AC AC
CC AA AA AA AC CC AC AC
AC AC AC AC AC AA AA AC
AA AC AC AA AA AA AA AA
CC CC CC CC AC AC AC
49697140
9532 rs1027894 17 GG GG GG GG GG
GG GG GG AG AG AG GG AG
GG GG GG GG GG GG GG GG
GG AG AG GG GG GG GG GG
GG GG GG GG GG GG GG
46527718
9533 rs10278980 7 CC CC CC CC CC
CC CC CC AC CC CC CC CC
CC CC CC CC CC AC CC AC
CC CC AC AC CC AA AC AC
CC CC AC AC CC AC AC
52099373
9534 rs10278993 7 AA AA AA AA AA
AA AA AA AG AA AA AG AG
AA AA AG AG AG AA AA AA
AG AG AA GG AG AA AG AA
AA AA AA AA AA AA AA
42497358
9535 rs10279013 7 GG GG GG GG GG
GG GG GG GG GG GG GG GG
GG GG GG GG GG GG GG GG
GG GG GG GG GG GG GG GG
GG GG GG GG GG GG GG
73151889
9536 rs10279016 7 AG AG GG AA GG
AG AG AG AA GG AA AA AA
GG GG AA AG AA AG AG AG
AG AG GG AA AG AA AG AG
AG AG AG GG AG GG AG
40049437
9537 rs10279017
7 -- -- -- -- -- -- -- --
-- -- -- -- -- -- -- --
-- -- -- -- -- -- -- --
-- -- -- -- -- -- -- --
-- -- -- -- 66924597
9538 rs10279064 7 GG GG GG GG AG
GG AG GG AG GG GG GG GG
GG GG GG GG GG AG GG AG
GG GG GG GG GG AG AG AG

GG GG GG GG GG GG AG
19468258
9539 rs10279112 7 AA AG AA AG AG
AG AG AG AG AA AG AA AG
AG AA AA AA AA AA AG AA
AG AG AG AG AA AG AG AG
AA GG AA AG AG AA AG
17280135
9540 rs1027912 16 AG AG GG GG AG
GG AG AG GG AA AG AG AA
GG GG GG GG AG AA AG AG
AG AG GG GG GG AG GG AG
AG AG AG GG AG GG AG
86308375
9541 rs10279137 7 AC CC AC CC CC
CC CC CC CC CC CC CC AC
CC AC CC CC CC CC CC AC
CC CC CC CC AC AC AA AA
CC CC CC CC CC CC CC
6170312
9542 rs10279197 7 AA AA AA AA AA
AA AA AA AA AA AA AA AA
AA AA AA AA AA AA AA AA
AA AA AA AA AA AA AA AA
AA AA AA AA AA AA AA
54611800
9543 rs10279206 7 AG AG AG AG AG
AG GG GG GG GG AG GG AG
GG GG GG GG GG GG GG GG
AA GG AA AA AG AG AG AG
GG AG AG AG AA AA AG
155213800
9544 rs10279234 7 AA AA AA AA AG
AG AA AA AA AA AA AA AA
AA AA AA AA AA AA AG AA
AA AG AG AA AG AA AG AA
AA AA AA AA AA AA AA
64054804
9545 rs1027928 2 GG GG GG GG GG
GG GG AA AG GG GG AG GG
GG GG GG GG GG GG GG GG
AG AG AG AG GG GG GG GG
AG GG AG GG AG GG AG
239524082
9546 rs10279346 7 GG GG GG GG AA
AG AG AA AG AG AA AA AA
AA AA AA AG AA AG AG AA
AA AA AA AA AG AG AA AG
AG AG AG AG AG AA GG
41154382

9547 rs10279350 7 GG GG GG GG GG
GG GG AG AG AG GG GG GG
GG GG GG AG AG AG AG GG
AG AG GG AG GG GG GG GG
GG GG GG GG AG GG AA
126405246
9548 rs10279375 7 AG AG AA AG AG
AA GG GG GG GG AA AG AG
AA GG AG GG GG GG AA AG
AG AG AA AG AG AG AA AA
AA AA AG AG AG AG AG
104319876
9549 rs10279396 7 AA AA AA AA AA
AA AA AA AA AA AA AA AA
AA AA AA AA AA AA AA AA
AA AA AA AA AA AA AA AA
AA AA AA AA AA AA AA
97807351
9550 rs10279415 7 GG GG GG GG GG
GG GG GG GG GG GG GG GG
GG GG GG GG GG GG GG GG
GG GG GG GG GG GG GG GG
GG GG GG GG GG GG GG
4777419
9551 rs10279430 7 GG GG GG GG GG
GG GG GG AG AG GG GG GG
GG GG GG GG GG GG GG AG
AG GG AG GG GG GG GG GG
GG GG GG GG GG GG GG
11198193
9552 rs1027945 X GG GG GG GG GG
GG GG GG GG GG GG GG GG
GG GG GG GG GG GG GG GG
GG GG GG GG GG GG GG GG
GG AG GG AA GG GG GG
134756293
9553 rs1027949 12 AG AG AG AA AG
AA AG AG GG GG AA AA AA
AA AA AA AA AA GG GG GG
AG AG AA AG GG AA AG AG
AG AA AA AA GG AG AG
110430968
9554 rs10279520 7 AA AA AA AA AA
AA AA AA AA AA AA AA AA
AA AA AA AA AA AA AA AA
AA AA AA AA AA AA AA AA
AA AA AA AA AA AA AA
48028771
9555 rs10279551 7 AG AG AA AG AG
GG AA AA AA AA AA AA AA

AG GG GG AG AG AA AA AG
AG AA AA AG AA AA AA AA
AA AG AA AA AA AA AA
70972962
9556 rs10279558 7 AA AA AA AA AA
AA AA AA AA AA GG GG GG
GG GG AG AA AA AG AA AG
AA AG AA AA AA AA AA AA
AA AA AA AA AA AA AG
114862948
9557 rs10279635 7 GG GG GG GG AG
AG GG GG GG GG GG GG GG
GG GG GG GG GG GG AG GG
GG GG GG GG GG GG GG GG
GG GG GG GG GG GG GG
52135471
9558 rs10279638 7 GG GG GG GG GG
GG GG GG GG GG GG GG GG
GG GG GG GG GG GG GG GG
GG GG GG GG GG GG GG GG
GG GG AG GG GG GG GG
155024830
9559 rs10279754 7 CC CC CC CC CC
CC CC CC CC CC CC CC CC
CC CC CC CC CC CC CC CC
CC CC CC CC CC CC CC CC
CC CC CC CC CC CC CC
1874927
9560 rs10279772 7 AG AA AA GG AG
GG AG AG AG GG GG GG GG
AG AG AG AA AA GG GG GG
AG GG AG GG GG GG GG GG
AG AG AG GG AG AG GG
38706384
9561 rs10279792 7 AC CC AC CC CC
CC CC CC CC CC AC AC CC
CC CC CC AC CC CC CC CC
CC CC CC CC CC CC CC CC
CC CC CC CC CC CC AC
13198073
9562 rs10279898 7 AG AG AG AG GG
GG AG GG GG GG GG AG AG
AG AG AG GG GG AG GG AG
AG AG AA AG AA AG AG AG
AG AG AG AG GG AG AG
15363293
9563 rs10279900 7 GG GG GG GG GG
GG GG GG GG GG GG GG GG
GG GG AA AG AG GG AG GG
GG GG AG GG GG AG GG GG

GG GG GG GG GG GG GG
103338055
9564 rs1027992 18 GG GG GG AA AA
AA AG GG GG GG AG GG GG
GG GG GG GG GG AG GG AA
AG AG GG GG GG GG GG GG
GG AG AG GG AG GG AG
36002191
9565 rs10279927 7 AG AA AG AA AG
AG AG GG GG GG GG AG AG
GG AG GG GG GG AA AG AA
AG AA AA AG AG AA AA AG
AA AG AA AG AG GG AA
67261526
9566 rs10279978 7 GG GG GG AG AG
AG AG AA AG AG AG AA AG
GG GG AG AA AG GG GG AG
AG AG AA AG GG GG GG GG
AG AG AG AG AG AA GG
5319387
9567 rs1027998 4 GG GG GG GG AG
GG AG GG GG GG GG AG GG
GG AG GG GG GG GG GG AG
AA AG AG AG GG AA AG AG
AG GG GG GG AG GG AG
26017406
9568 rs10279985 7 GG GG GG GG GG
GG GG AG AG GG GG GG GG
GG GG GG GG GG AG AG AG
GG AG AG GG AG GG AG GG
GG GG GG GG GG GG GG
42271960
9569 rs1028000 4 AG GG AG AA AA
AA AG AA AA AA AG AA AA
AG AG AA AA AA AA AA AG
AG AA AA AA AA GG AG AG
AG GG AA AG AA AG AA
54460531
9570 rs10280013 7 AA AA AA CC AA
AC AC AC AC AA AC AC AA
AC AC CC CC CC AA AC AA
AA AC AC AA AC AC AA AA
AA AC AA AC AC AA AC
94481267
9571 rs10280038 7 GG GG GG GG GG
GG GG GG GG GG GG GG GG
GG GG GG GG GG GG GG GG
GG GG GG GG GG GG GG GG
GG GG GG GG GG GG GG
148117547

9572 rs10280108 7 AA AA AA AA AA
AA AA AA AA AA AA AA AA
AA AA AA AA AA AA AA AA
AA AA AA AA AA AA AA AA
AA AA AA AA AA AA AA
152340220
9573 rs1028011 3 GG GG GG GG GG
GG GG GG GG GG GG GG GG
AG AG GG GG GG GG GG GG
AA GG AG AG AG GG GG GG
GG GG AG GG AG AG GG
89498619
9574 rs10280113
7 -- -- -- -- -- -- -- --
-- -- -- -- -- -- -- --
-- -- -- -- -- -- -- --
-- -- -- -- -- -- -- --
-- -- -- -- 63043186
9575 rs10280152 7 AA AA AA AC AA
AC AA AA AC AA AA AA AA
AA AA AA AA AA AA AC AA
AA AA AA AA AA AA AA AA
AC AA AA AA AA AA AA
49826804
9576 rs10280228 7 GG GG AG GG GG
GG GG GG AG AG GG GG GG
AA AA AA AA AG AG AG AA
AG AG AA AG AA GG AG AG
AG GG GG GG AG GG AG
47886707
9577 rs10280239 7 AA AA AG GG AG
AG AG GG AG GG AG AG AA
AA AA GG GG GG AG GG AG
AG AG AG AG AG AG GG GG
GG GG AG AG AA AG AA
138715731
9578 rs10280300 7 AC CC CC CC CC
CC CC CC CC CC CC CC CC
AC AA AC AA AA AC AC AC
CC CC CC CC CC AA AC AC
CC AC CC CC CC CC CC
145209049
9579 rs10280334 7 AA AA AA AA AA
AA AA AA AA AA AA AA AA
AA AA AA AA AA AA AA AA
AA AA AA AA AA AA AA AA
AA AA AA AA AA AA AA
151822990
9580 rs10280353 7 AA AA AA AG AA
AA AG AG AA AA AA AA AA

AA AA AA AG AG AA AA AA
AA AA AA AA AG AA AG AG
AA AA AA AA AG AA AG
3070171
9581 rs10280384 7 GG GG GG GG GG
GG GG AG AG AG GG GG GG
GG GG GG GG GG GG GG GG
GG GG GG GG GG GG GG GG
GG GG GG GG GG GG GG
97324281
9582 rs10280425 7 GG GG AG AG AG
GG AG GG GG GG AG GG AG
AG AG GG GG GG -- GG AG
GG GG GG GG GG AG GG GG
AG GG GG GG GG AG GG
148245802
9583 rs10280428 7 AA AA AA AA AA
AA AA AC AC AC AA AA AA
AA AA AA AA AA AC AA AC
AC AC CC CC AC AA AC AA
AA AA AA AA AA AA AA
81915186
9584 rs10280445 7 AG GG GG GG AG
GG AG AG AG GG AG GG AG
AG AG AG AG AG GG AG GG
AA AG GG AG GG GG GG GG
GG AG GG AG GG GG GG
55798357
9585 rs10280461 7 CC CC CC AC CC
CC AC CC CC CC AA AC AC
AC AC AC AC AC CC AA CC
AC AC AA CC CC AC AC AC
CC AC AC AA AA AA AC
38149932
9586 rs10280469 7 AG AG AG AG AA
AG AA AG AG AA AA AA AA
AA AA AA AA AA AA AA AA
AG AA AG AA AA AA AA AA
AA AG AA AG AG AA AG
41412994
9587 rs10280475 7 GG GG AG AG AG
AA GG AG AA AG GG GG GG
AG GG AA AG AA AG AG AA
AA AG AA AG AG AA AG AA
AA AG AG GG GG AG AG
25963672
9588 rs10280568 7 CC CC CC AC AC
CC AC CC CC CC CC CC CC
CC CC CC CC CC CC CC CC
CC CC CC CC AC CC CC AC

CC CC CC CC CC CC CC
71489191
9589 rs10280571 7 AA AG AG AA AG
AA AG AA AA AG AA AA AA
AA AA AA AA AA AG AG AG
AA AA AG AA AA AA AA AA
AG AG AA AA AA AA AA
96986462
9590 rs1028060 17 AG AG AG AG AG
AA GG AA AA AA AA AA AA
AA AA AG AG AG GG GG GG
AA AA AA AA AG AG GG AG
GG AG AG GG AG GG AA
77436724
9591 rs1028067 11 GG GG AG AG AG
GG AG AG GG GG GG GG GG
GG GG AG GG GG AG GG GG
AG AG AA AG AG GG GG GG
AG GG AG AG GG GG GG
22519082
9592 rs10280681 7 AA AA AA AA AA
AA AA AA AA AA AA AA AA
AA AA AA AA AA AA AA AA
AA AA AA AA AA AA AA AA
AA AA AA AA AA AA AA
33018462
9593 rs1028071 13 AG GG GG AG AA
AA AG AG AG AG AG GG AG
AG AG AG AG AG AA AA AG
AG AG AA AG AG GG AG AG
AA GG AG AG AG GG AG
61323885
9594 rs10280713 7 AG AG AG GG AG
GG AG AG GG AG GG GG GG
GG GG GG GG AG GG GG GG
AG GG GG AG GG GG GG GG
GG AA AG AG GG AG GG
40699189
9595 rs10280736 7 AA AA AA AA AA
AA AA AA AA AA AA AA AA
AA AA AA AA AA AA AA AA
AA AA AA AA AA AA AA AA
AC AA AA AA AA AA AA
11124069
9596 rs10280773 7 AG AG AG GG GG
GG GG GG GG GG GG GG GG
GG GG AG AG AG GG GG GG
GG AG GG AG GG GG GG GG
GG GG AG AG GG GG GG
7304142

9597	rs10280808		7	AA	AA	AA	AA	AA
	AA	AA	AA	AA	AA	AA	AA	AA
	AA	AA	AA	AG	AA	AA	AA	AA
	AA	AA	AA	AA	AA	AA	AA	AA
	AG	AA	AG	AA	AA	AA	AA	
11595832								
9598	rs10280848		7	AA	AG	AG	AG	AG
	GG	AA	AG	AA	GG	GG	GG	GG
	GG	GG	AG	AA	AA	GG	AG	GG
	AG	AG	AG	AG	GG	AG	AG	AG
	AA	AG	AA	AA	GG	GG	AG	
68104188								
9599	rs10280909		7	AA	AA	AA	AA	AA
	AA	AA	AA	AA	AA	AA	AA	AA
	AG	AG	AA	AG	AA	AA	AA	AG
	AA	AA	AA	AA	AG	AA	AA	AA
	AG	AA	AA	AA	AG	AA	AG	
34407843								
9600	rs10280942		7	GG	GG	GG	AG	GG
	AG	GG	AA	AG	AG	GG	GG	GG
	GG	GG	GG	AG	GG	AG	AG	AG
	AG	AG	AG	AG	AG	AG	GG	AG
	AG	AG	GG	GG	GG	GG	GG	
62573214								
9601	rs10280960		7	GG	GG	GG	AG	AG
	GG	AA	AA	AG	AA	GG	GG	GG
	GG	GG	AG	AG	GG	AG	AG	AA
	AA	AG	AA	AG	GG	AG	AG	AG
	AA	AG	AG	AA	AG	AG	AA	
1103697								
9602	rs10280998		7	AC	AC	CC	CC	CC
	CC	CC	CC	CC	CC	CC	AC	AC
	AC	AC	CC	CC	CC	CC	CC	CC
	CC	AC	CC	CC	CC	CC	CC	CC
	CC	CC	CC	CC	CC	CC	CC	
116290966								
9603	rs10281056		7	AA	AG	AG	AG	GG
	AG	AG	GG	GG	GG	AA	AA	AA
	AA	AA	GG	GG	GG	AA	AG	AG
	AA	GG	AG	AA	AG	AG	GG	GG
	GG	AG	AG	AG	GG	AG	AG	
127204844								
9604	rs10281065		7	CC	CC	CC	AA	CC
	AC	AC	CC	CC	AC	CC	CC	CC
	CC	CC	CC	AC	CC	AC	CC	AC
	CC	CC	AC	AC	CC	CC	CC	CC
	AC	CC	CC	CC	CC	CC	CC	
50829973								
9605	rs10281073		7	GG	GG	GG	AA	GG
	AG	AG	AG	AG	GG	AG	AG	GG

GG AG AA AA AA GG AG GG
GG AG AG GG GG AG GG GG
GG GG GG GG AG GG AG
94420238
9606 rs10281099 7 AA AA AA AA AA
AA AA AA AA AA AA AA AA
AA AA AA AA AA AA AA AA
AA AA AA AA AA AA AA AA
AA AA AA AA AA AA AA
7045290
9607 rs10281107
7 -- -- -- -- -- -- -- --
-- -- -- -- -- -- -- --
-- -- -- -- -- -- -- --
-- -- -- -- -- -- -- --
-- -- -- -- 4261767
9608 rs10281202 7 AG AG AA AG AA
AG AA AG AG AG AG AG AA
AA AG GG AG AG AA AA AA
AG AG AG AG AG AG GG GG
AG AA AG AA AG AA AG
134923406
9609 rs10281213 7 GG GG GG GG GG
GG GG GG GG GG GG GG GG
GG GG GG GG GG GG GG GG
GG GG GG GG GG GG GG GG
GG GG GG GG GG GG GG
119605536
9610 rs1028122 6 AA AA AA AA AA
AA AA AA AG AA AA AA AA
AA AG AA AA AA AA AA AA
AG AG AA AG AA AG AA AA
AA AA AA AA AA AA AA
112342709
9611 rs10281230 7 GG GG GG GG GG
GG GG GG GG GG GG GG GG
GG GG GG GG GG GG GG GG
GG GG GG GG GG GG GG GG
GG GG GG GG GG GG GG
156578126
9612 rs10281294 7 GG GG GG AG AA
AG AA AA AA AA AG AG AA
AG AG AG AA AA AA AA AG
AA AG GG AA AA AA AA AA
AG AA AA AA AA AA AG
93341409
9613 rs1028133 16 AA AA AA AA AA
AA AA AG AG AG AA AA AA
AA AA AA AA AA AA AA AA
AG AA AG GG AA AA AA AA

AG AA AA AA AA AA AA
27138785
9614 rs10281361 7 AA AC AA AC CC
CC AC AC AA AC CC AC AC
AC AC AC CC AC CC AA CC
AC AC AC AC AC CC CC AC
CC AC AC AC CC CC --
43433926
9615 rs10281369 7 AA AA AA GG AG
AG GG AG AG AG AG AG GG
GG GG AG AG AG AG AG AG
AA AG AG AA AA AG AG AG
AA AA AG AG GG GG AG
19333027
9616 rs1028144 2 AG AG AG AG AA
AG AG AG AG AG GG AG GG
GG GG AG AG GG AG AG AA
AG AA AG AG AG AG AA GG
AA AG AG GG GG AG GG
99279354
9617 rs10281442 7 AA AA AA AA AA
AA AA AA AA AA AA AA AA
AA AA AA AA AA AA AA AA
AA AA AA AA AA AA AA AA
AA AA AA AA AA AA AA
65899855
9618 rs10281519 7 AA AA AA AG AA
AG AA AA AA AA AA AA AA
AA AA AA AA AA AG AA AG
AG AG AA AG AA AA AA AA
AG AA AA AA AA AA AA
90910024
9619 rs10281563 7 AA AA AG AG AG
AA GG AA AA AA AA AG AG
AG AG AA AA AG GG AG AG
AG AG AG AG AG AG GG AG
GG GG AA AG GG AG GG
49733053
9620 rs1028157 3 AA AA AA AA AA
AA AA AA AA AA AC AC AC
AC AC AA AA AA AA AA AC
AA AA AA AA AC AA AC AC
AA AA AA AA AA AA AA
13617049
9621 rs10281637 7 AA AA AA AG AA
AG AA AA AA AA AA AA AA
AA AA AG AG AG AG AG AG
AG AA AA AG AA AG AA AA
AA AA AA AA GG AG AG
116151338

9622 rs10281675 7 GG GG GG GG GG
GG GG GG GG GG GG AG AG
AG AG GG AG AG GG AG GG
GG GG GG GG GG GG GG GG
AG AA GG AG AG AG AG
150485401
9623 rs10281731 7 GG GG GG AA AA
AG AG AG AG GG GG GG GG
GG GG GG GG GG GG GG GG
GG GG GG GG GG GG GG GG
GG GG GG GG AG AG GG
155661335
9624 rs10281760 7 AA AA AA AA AC
AA AC AA AA AA AA AA AA
AA AA AA AA AA AA AA AA
AC AA AA AC AA AA AA AA
AA AA AA AA AA AA AA
63806918
9625 rs10281877 7 AG AA AG GG AG
AG GG GG GG GG AA GG AG
AG GG AG AA AG AG AG GG
GG AG GG AG GG AG GG GG
AA GG GG GG AG AG AA
12819414
9626 rs10281887 7 GG GG GG GG GG
GG GG GG GG GG GG GG GG
GG GG GG GG GG GG GG GG
GG GG GG GG GG GG GG GG
GG GG GG GG GG GG GG
6572094
9627 rs1028189 2 AA AA AA AA AA
AA AA AA AA AA AA AA AA
AA AA AA AA AA AG AG AG
AA AA AA AA AA AA AA AA
AA AA AA AA AA AA AA
57673796
9628 rs10281898 7 GG GG GG AG GG
GG AG GG AG AG GG GG GG
GG GG GG AG GG GG GG GG
AG AG AG AG AG GG AG AG
GG GG AG AG AA AG AG
6735409
9629 rs10281917 7 CC AC AC AA AC
AC AA AA AA AA AA AA AA
AA AA AC AA AA AC AA AA
AC AC AC AC AA AC AC AC
AA AA AC AC AC AC AA
43700346
9630 rs10281924 7 GG GG GG GG GG
GG GG GG GG GG GG GG GG

GG GG GG GG GG GG GG GG
GG GG GG GG GG GG GG GG
GG GG GG GG GG GG GG
69620465
9631 rs10281994 7 AA AA AA AA AA
AA AA AG AG AG AA AA AA
AA AA AA AA AA AA AA AA
AA AG AG AA AA AA AA AA
AA AA AG AA AA AA AA
125484796
9632 rs10282048 7 AA AA AG AG AG
AA GG AA AG AA AG AG AA
AA AA AA AA AA AA AA AA
AA AA AA AA AA AA AA AA
AA AA AA AA AA AA AA
68025407
9633 rs10282109 7 CC AC CC AA AC
AC AA AC AC CC AA AA AA
AA AA AA AC AC AA AC AA
AA AC AA AC AA CC AC AC
AC AA AC AC AA AA AC
15699816
9634 rs10282116 7 AG GG AG AA GG
AG AG AG AG GG AG AG AG
GG AA AA AG AG GG AA GG
AA AG GG AA AG AG AG AG
AG AG AA AA GG AG AG
94566679
9635 rs10282136 7 AG AG AG GG GG
GG GG AG AG AG GG GG GG
GG GG AG AG GG AG GG AG
AA AA AA AA GG AG GG GG
AA AA GG AG GG GG GG
18991904
9636 rs10282159 7 AA AA AA AA AA
AA AA AA AA AA AA AA AA
AA AA AA AA AA AA AA AA
AA AA AA AA AA AA AA AA
AA AA AA AA AA AA AA
69854259
9637 rs10282181 7 AG AG GG AG GG
AG GG AG AG GG AG AA AA
GG AG AG GG GG AG AG GG
AG AG AG AG AG GG AG AG
GG GG GG GG AA AA AA
1551716
9638 rs10282189 7 AA AA AA AA AA
AA AA AA AA AA AA AA AA
AA AA AA AA AA AA AA AA
AA AA AA AA AA AA AA AA

AA AA AA AA AA AA AA
18660425
9639 rs10282190 7 AA AA AA GG GG
GG AG GG AG GG GG AG AG
AG GG GG GG GG AG GG AG
AG AA AG AG AG AG AG AG
GG GG AG AG AG GG AG
131383556
9640 rs10282214 7 AA AG AG AA AA
AA AA AA AA AA AA AA AA
AA AA AA AG AG AA AA AA
AA AA AA AA AG AA AG AG
AA AA AG AG AA AA AA
103681973
9641 rs10282220 7 AA AG AA AG AG
AA GG GG AG AG GG AG GG
AG AG AG AG AG AA AA AA
AA AA AA AA AG AA AG AG
AG AG AA AG AG AG AA
6650687
9642 rs10282225 7 AA AA AA AA AA
AA AA AA AC AA AA AA AA
AC AC AC CC CC AC CC AA
AA AC CC AC AC AA AA AC
AC AA AA AA CC AC AC
56755583
9643 rs10282270 7 AA AA AA AA AA
AA AC AC AA AC AC AA AC
AC AC CC AC AC AC AA AC
AA AC AA AA AA AA AA AA
AC AA AC AC AA AA AA
158285315
9644 rs10282321 7 GG GG GG GG GG
GG GG AG GG GG GG GG GG
GG GG GG GG GG GG GG GG
GG GG GG GG GG GG GG GG
GG GG GG GG GG GG GG
153254763
9645 rs10282353 7 AC CC CC AA AC
AC AA AA AC AA CC AC AC
AA AA AC AC AA AC AA AC
AC AC AC AC CC AA AC AC
AA AC AA AC AC AA AC
42112533
9646 rs10282356 7 AA AA AA AA AA
AA AA AA AA AA AA AA AA
AG AG AA AG AA AA AA AA
AA AA AA AA AA AA AA AA
GG AA AA AA AA AA AA
34336057

9647 rs10282370 7 GG AG GG AA AA
AA AA AA AA AA AA AA AA
AA AG AA AA AA AA AG AA
AG AA AG AG AA AG AA AA
AA AA AA AA AA AA AA
152668222
9648 rs10282396 7 GG GG GG GG GG
GG GG GG GG GG GG GG GG
GG GG GG GG GG GG GG GG
GG GG GG GG GG GG GG GG
GG GG GG GG GG GG GG
55008502
9649 rs10282404 7 AG AG AA AA AA
AA AA AA AG AA AA AA AA
AA AA AA AA AA AG AA AG
AG AA AG AG AA AG AA AA
AA AA AG AG AA AA AA
134441703
9650 rs10282436 7 AA AA AC CC CC
CC CC AC CC AC AA AA AA
CC AC CC AC CC CC CC CC
CC CC CC CC AC CC AC CC
AC AC CC CC CC CC AC
25873110
9651 rs1028245 2 AC AC AC AA AA
AA AC AA AA AA AA AA AC
AA AA AA AA AA AC AC AA
AA AA AC AC CC AC AC CC
AC AA AC AA AC AA AC
114601335
9652 rs10282512 7 AG AG GG AG GG
AG GG GG GG AG GG GG GG
AG AG GG GG GG GG GG GG
GG GG GG GG GG AG AG AG
GG AG AA AA AG GG AA
70192653
9653 rs10282520 7 GG GG GG GG GG
GG GG GG GG GG GG GG GG
GG GG GG GG GG GG GG GG
GG GG GG GG GG GG GG GG
GG GG GG GG GG GG GG
41844895
9654 rs10282532 7 AC AC AC CC CC
CC CC CC CC CC AA AA AA
AC AC AA AA AA CC AA CC
AC CC CC AC AC AA AC AA
AC AC AA AA AA AA AC
54773399
9655 rs1028254 8 AA AA AA AA AA
AA AA AA AA AA AA AA AA

AA AA AA AA AA AA AA AA
AA AA AA AA AA AA AA AA
AA AA AA AA AA AA AA
91485194
9656 rs10282605 7 AA AA AA AA AG
AA AG AG AG AG AA AA AA
AG AA AG AA AA AG AA AA
AA AA AG AA AG AG AA AA
AG AA AA AA AG AG AA
103162370
9657 rs10282671 7 GG AG AG AG AA
AG AA AG AG AG AA AA AA
AA AA AA AA AA AG AG AG
AA AG AG AA AG AG GG AG
AG AA AG AG AG AG AA
90456475
9658 rs10282696 7 GG GG GG GG GG
GG GG GG GG GG GG AG AG
GG AG GG GG GG AG GG AG
GG GG GG GG GG GG GG GG
AG GG GG GG AG AG AG
17241565
9659 rs10282700 7 GG AG GG AG GG
AG GG GG GG AG AA AG AG
GG GG GG GG GG AG GG GG
GG GG GG GG AG AG GG AG
GG AG GG AG AG GG AG
55442803
9660 rs10282706 7 GG GG GG GG GG
GG GG GG GG GG GG GG GG
GG GG AG GG GG GG GG GG
GG GG GG GG GG GG GG GG
GG AG GG GG GG GG AG
99217424
9661 rs10282707 7 GG GG GG AG AG
AG AG AA AA AG GG GG GG
AG AG AA AG AG AG AG AA
AA GG AG GG AG GG GG AG
AG GG AG GG GG GG GG
17911038
9662 rs10282743 7 AG GG AG AG AG
AG AG AG AA AG AG AA AG
AG AA GG AG -- AG AG AG
AA GG AG AG GG GG GG GG
GG AG AG AA AG AG AG
19682148
9663 rs10282751 8 GG GG GG AA AG
AA AG GG GG AG AG AG AA
AG AG AA AG AG AG AA AA
AA AA AA AA AG AG GG AG

GG AA AG AG AA AA AA
124977830
9664 rs10282752 8 AG AG GG GG GG
GG GG GG GG GG AA GG AG
AG AG GG GG GG AG AG AA
GG AA AA AG GG AA AG AG
AA GG GG GG GG GG GG
48049106
9665 rs10282797 8 GG GG GG AG AG
GG AA GG GG GG GG AG GG
AG AG GG GG GG GG GG GG
GG AG AG GG GG AG AG GG
GG GG AG AG AG GG AG
2481966
9666 rs10282829 8 GG GG GG AG AG
AG GG GG GG AG AG GG AG
AG GG GG GG GG GG AG GG
AG GG GG AG AG AG AG AG
GG GG GG GG GG AG GG
26280723
9667 rs10282854 8 GG GG GG GG GG
GG GG GG GG GG GG GG GG
GG GG GG GG GG GG GG GG
GG GG GG GG GG GG GG GG
GG GG GG GG GG GG GG
97119916
9668 rs10282859 8 GG AG AA AG AG
GG AA GG GG GG AA AA AA
AA AG AG AG AA AG AG AG
GG GG GG GG AG AA AA AG
AA AG AG AG AG AG AA
112997877
9669 rs10282908 8 GG GG GG GG GG
GG GG GG GG GG GG GG GG
GG GG GG GG GG GG GG GG
GG AG GG AG AG GG AG AG
GG GG GG GG GG GG GG
138153441
9670 rs10282916 8 GG GG GG GG GG
GG GG GG GG GG GG GG GG
GG GG GG GG GG AG AG AG
GG GG GG GG AG GG AG AG
GG GG GG GG GG GG GG
38558577
9671 rs10282929 8 CC AC AC CC AC
AC CC AC AC AA AA AC AC
CC CC AC AC AC AC AC AC
AC AC AC AC AC AA AA AA
AA AA AC AC AC CC AC
144681777

9672	rs10282940	8	GG	GG	GG	GG	AG
	GG	AG	AA	AA	AA	GG	GG	GG
	GG	GG	AG	GG	AG	GG	GG	AG
	GG	GG	GG	GG	AA	AG	AG	AG
	AG	GG	GG	GG	GG	GG	GG	
118187826								
9673	rs10282994	8	AA	AA	AA	AA	AA
	AA	AA	AG	GG	GG	AA	AA	AA
	AA	AA	AA	AA	AA	AG	AA	AA
	AG	AA	AG	AA	AG	AA	AA	AG
	AA	AA	AA	AA	AA	AA	AA	
15290119								
9674	rs10283028	8	GG	GG	GG	GG	GG
	GG	AG	GG	GG	AG	GG	GG	GG
	AG	AG	AG	AG	GG	GG	GG	AG
	GG	GG	GG	GG	AG	GG	AG	AG
	AG	GG	GG	GG	GG	GG	AG	
1243574								
9675	rs10283122	8	CC	AC	AA	AA	AC
	AC	AA	AC	AC	AC	CC	CC	CC
	AC	AC	CC	AC	AC	AC	AA	CC
	AC	AA	AC	AA	AA	AA	AA	AA
	AA	AC	CC	AC	CC	AC	CC	
117778570								
9676	rs10283134	8	GG	GG	GG	AG	GG
	AG	GG	GG	GG	GG	GG	GG	GG
	GG	GG	AA	AG	AG	GG	AG	AG
	AG	AG	AG	AG	AG	AG	AG	AA
	AG	GG	GG	GG	GG	GG	GG	
126272646								
9677	rs10283235	8	AA	AA	AA	AA	AA
	AA	AA	AA	AA	AA	AA	AA	AA
	AA	AA	AA	AA	AA	AA	AA	AA
	AA	AA	AA	AA	AA	AA	AA	AA
	AA	AA	AA	AA	AA	AA	AA	
93289262								
9678	rs10283307	8	AG	AG	GG	AA	AA
	AA	AA	AA	AA	AA	AG	AG	AG
	AG	AA	AA	AA	AA	AG	GG	AG
	AA	AA	AA	AG	AA	AG	AA	AA
	AG	AA	AA	AA	AA	AG	AA	
133737802								
9679	rs10283372	8	AG	AG	AG	AA	AA
	AA	AA	AA	AG	GG	AA	AG	AG
	AG	AG	AA	AA	AG	AG	GG	AG
	AA	GG	AG	AG	GG	AG	AG	AG
	AG	GG	AG	GG	GG	AG	GG	
74335491								
9680	rs1028343	22	AA	AA	AA	AA	AA
	AA	AA	GG	AG	GG	AA	AA	AA

AA AA AG AG AG AG AG AG
AA AA AG AG AG AA AG AG
AA AG AG AA AA AG AA
35202152
9681 rs10283463 9 GG AG GG AG AG
GG AG GG GG GG AG GG GG
GG AG AA AG AG GG GG AG
AG AG AG AG GG GG GG GG
GG AG GG GG AA AG AA
77789607
9682 rs10283502
0 -- -- -- -- -- -- -- --
-- -- -- -- -- -- -- --
-- -- -- -- -- -- -- --
-- -- -- -- -- -- -- --
-- -- -- -- 0
9683 rs10283595 9 AA AA AA AC AC
CC AA CC AC AA AC CC AC
AA AC AA AA AA AC AC AA
AA AC AA CC AA CC AC AC
AA AC AC CC AC AA AC
5407628
9684 rs10283760 9 AA AA AA AA AA
AA AA AA AA AA AA AA AA
AA AA AA AA AA AA AA AA
AA AA AA AA AA AA AA AA
AA AA AA AA AA AA AA
140712387
9685 rs10283767 9 AA AA AA AC AA
AC AA AA AA AA AA AA AA
AA AA AA AA AA AA AA AA
AA AA AA AA AA AA AA AA
AA AA AA AA AA AA AA
101182698
9686 rs10283769 9 AA AA AA AA AA
AA AA AA AA AA AA AA AA
AA AA AA AA AA AA AA AA
AA AA AA AA AA AA AA AA
AA AA AA AA AA AA AA
1830840
9687 rs10283808 9 AG AA AG GG GG
AG GG GG GG AG GG GG AG
AG AG AG GG GG AG AG AG
GG GG GG GG AG AG GG AG
GG GG GG GG GG GG GG
34942073
9688 rs10283912 9 AG AG AA AG AG
AA GG GG GG AG AA AG AA
AG AG AA AA AA AG GG AG
AG GG AG AG GG AG GG AG

AA AG GG GG AG AG AG
12285170
9689 rs1028394 20 GG AG GG GG GG
GG GG GG AG GG GG GG GG
GG AG AG GG GG GG GG AG
GG AA AG GG AA AG AA AA
AG AG AG AG AA AG AA
6860895
9690 rs1028395 20 AG AA AA AA AG
AA AG AG AA AA AA AA AA
AA AA AA AG AG AG AA AG
AA AA AA AA AA AG AA AA
AA AA AA AA AA AA AA
6860859
9691 rs1028400 1 CC AC CC AC AC
AC AC CC CC CC CC AC AC
AC CC CC AC AC CC CC CC
AC AC AC AC AC AC AC AC
CC CC AC CC CC CC CC
208113731
9692 rs1028404 X AA AA AA AA AA
AA AA GG AG AA GG AG AG
GG GG AG AG AA AG AG GG
GG GG GG AG AG AA AG AA
AA AG GG GG AA AG AA
107870501
9693 rs10284068
X -- -- -- -- -- -- -- --
-- -- -- -- -- -- -- --
-- -- -- -- -- -- -- --
-- -- -- -- -- -- -- --
-- -- -- -- 143495603
9694 rs10284071 X AA AA AA AA AA
AA AA AA AA AA AA AA AA
AA AA AA AG AG GG AG GG
AA AA GG AG AA AA AA AA
AG AA AA AA AA AG AA
100409142
9695 rs10284076 X GG GG GG GG GG
GG GG GG GG GG GG GG GG
GG GG GG GG GG GG GG GG
GG GG GG GG GG GG GG GG
GG GG GG GG GG GG GG
143882379
9696 rs1028408 20 AG GG GG AA GG
AG AG AG GG GG AG GG AG
AG AG GG AG AG AA AG AG
AG AA AG GG GG AG GG GG
GG AG GG AG AG AG GG
2721474

9697	rs10284107	X	AA	AA	AA	AG	AA
	AA	AG	AG	AG	AA	AA	AA	AA
	AG	AG	AA	AA	AA	AA	AG	AA
	GG	GG	AA	AG	AA	AA	AA	AA
	AA	AA	AA	AA	AA	AA	AA	
142290645								
9698	rs10284108	X	AA	AA	AA	AA	AA
	AA	AA	AA	AA	AA	AA	AA	AA
	AA	AA	AA	AA	AA	AA	AA	AA
	AA	AA	AA	AA	AA	AA	AA	AA
	AA	AA	AA	AA	AA	AA	AA	
123151923								
9699	rs10284188	X	AA	AA	AA	AA	AA
	AA	AA	AA	AA	AA	AA	AA	AA
	AA	AA	AA	AA	AA	AA	AA	AA
	AA	AA	AA	AA	AA	AA	AA	AA
	AA	AA	AA	AA	AA	AA	AA	
129593426								
9700	rs10284192	XY	GG	GG	GG	GG	GG
	GG	GG	GG	GG	GG	GG	GG	GG
	GG	GG	GG	GG	GG	GG	GG	GG
	GG	GG	GG	GG	GG	GG	GG	GG
	GG	GG	GG	GG	GG	GG	GG	
2249822								
9701	rs10284195	X	--	AA	AA	AA	--
	--	AA	AA	AA	AA	AA	AA	AA
	AA	AA	AA	AA	AA	AA	AA	--
	--	AA	--	AA	AA	AA	AA	--
	AA	AA	AA	AA	AA	AA	AA	
114511905								
9702	rs10284211	X	AA	AA	AA	AA	AA
	AA	AA	AG	AG	AA	AG	AG	AG
	AG	AG	AA	AA	AA	AG	AA	GG
	GG	AA	GG	AA	AA	AA	AA	AA
	AA	AA	AA	AA	AA	AA	AA	
125723304								
9703	rs10284228	Y	GG	--	--	--	GG
	GG	--	--	--	GG	--	--	--
	--	--	--	--	--	--	--	GG
	GG	GG	GG	--	--	GG	--	GG
	--	--	GG	GG	--	--	GG	
21939210								
9704	rs1028441	20	AG	AA	AA	AG	AA
	AA	AG	AG	AA	AA	AA	AG	AA
	AA	AA	AG	AA	AA	AG	AA	GG
	AA	AA	AG	AG	GG	GG	GG	GG
	AA	AG	AA	AG	AA	AG	AA	
2600186								
9705	rs1028471	6	GG	GG	GG	AG	GG
	AG	GG	AG	GG	AG	GG	GG	GG

GG GG AG AG GG GG GG GG
GG GG GG GG GG GG GG GG
GG GG AG AG AG AG GG
140263141
9706 rs1028479 20 AG AG AG AG GG
AG GG GG GG GG AG AG AA
AG AG GG AG AG AG AG AG
AA AG AA AG AG AA AA AG
AG AG AG AG AG GG AA
11144338
9707 rs1028492 22 GG GG GG GG GG
GG GG GG GG GG GG GG GG
GG AG GG GG GG GG GG GG
GG GG GG GG AG GG AG AG
GG AG GG AG GG GG GG
45392369
9708 rs1028503 22 GG GG AG AG AG
AA GG GG AG AG GG GG GG
GG AG AG AA AA AA AG AG
AA AG AA AG AA AG AG AG
AA AA AG AA AA AG AG
49068920
9709 rs1028530 14 CC AC CC AC CC
AC CC AC CC AC CC AC CC
CC CC CC CC CC CC CC CC
AC AC AC AC CC CC CC CC
CC AC AC AC CC CC AC
27441159
9710 rs1028539 20 GG GG GG GG AG
AG GG GG GG GG GG GG GG
GG GG GG GG AG GG AG GG
AG AA AA AG AG AG AG AG
AG GG AG GG GG AG GG
16445463
9711 rs1028546 20 AG AG AG AA AA
AA AA AA AA AA GG GG GG
AG AG AG GG AG AA AA AA
AG AG AG AA AA AG AA AA
AA AA AA AA AA AA AA
53738000
9712 rs1028549 20 AG AG AG GG GG
GG GG GG AG AG GG GG GG
AG GG GG AG AG GG AG AG
GG GG GG GG AG AG AG GG
AG AG GG AG AG AA GG
5198287
9713 rs1028565 6 GG AG AG AG AG
GG AA AG GG AG GG GG GG
GG AG AA AA AG AA AG AA
GG GG AG AG AA AG AG AG

AG AG AG AG AA AG AG
137041365
9714 rs1028598 2 AC CC CC CC CC
CC CC CC CC CC CC CC CC
AC AC AC AC CC CC AC CC
AC AC AA AC CC AC CC AC
AC CC CC CC AC AC CC
33260019
9715 rs1028644 20 GG GG AG AG AG
AG AG GG GG AG GG GG GG
AG GG GG AG GG AG GG AG
AG AG AG AG AG GG GG GG
GG GG AG AG GG GG GG
55809072
9716 rs1028651 6 AA AG AG AG AG
GG AA GG GG GG GG AG GG
GG GG GG AG AG GG AG GG
GG GG AA AG AA AG AG AA
AG AG GG AG GG GG AG
152937865
9717 rs1028653 9 AG AA AG GG GG
GG GG GG GG GG GG GG GG
GG GG GG GG AG GG GG GG
AG AG AG AG GG GG GG GG
GG GG GG GG GG AG GG
101651948
9718 rs1028655 9 GG GG GG AA GG
AG AG AG AA GG AG AA AA
AA GG AG AA AG AA AA AA
AG AG AG AG AA AG AA AA
AA GG AG AG AG GG AG
101652106
9719 rs1028695 13 AG GG AG GG AG
GG AG GG GG GG AG GG AG
AG AG GG GG GG GG GG GG
GG GG GG GG GG GG GG GG
GG GG GG GG GG GG AG
24752767
9720 rs1028697 13 GG GG GG AA AG
AG AG AA AA AA AG AG GG
AG GG AG AG AA AA AG AG
GG AA AG AA AG GG AG GG
AG GG AA AG GG GG GG
24752983
9721 rs1028704 9 AA AA AA AA AA
AA AA AA AA AA CC CC CC
AC AC AC AC AC AC AC AC
AA AA AA AA CC AA AC AC
CC CC AA AC AA AC AC
14426289

9722 rs1028717 14 AG AG AG AA AG
AG AA GG GG GG AA AA AA
AG AG AA AA AA AA AA AG
AA GG AA AG AA AA AA AA
AA AA AG AA AG AA AG
86322284
9723 rs1028723 14 AA AG AG AG AG
AG AG AG AA AG AG AG AG
AA AA AG GG GG AA AG AA
AA AG AA AG AA AG AG AA
AA AG AA AG AA AG AG
90208263
9724 rs1028724 13 AG AG AA AG AA
AG AG AG AA AA AG GG GG
GG AG AA AG AG GG GG GG
AG AG AA AA AG AA AA AG
AG AG AG GG AG AA GG
109832182
9725 rs1028733 13 AA AA AA AA AA
AA AA AA AA AA AA AA AA
AA AA AA AA AA AA AA AA
AA AA AA AA AA AA AA AA
AA AA AA AA AA AA AA
43637807
9726 rs1028753 13 AG AA AG GG AG
AG AG GG GG AA GG AG AG
GG AG AG AG AA AG AG AA
GG AG AG AG AG GG AG GG
AA AG GG AG AG AG AG
26655826
9727 rs1028774 13 AA AA AG AG AA
AG AA AG AG AG AA AG AA
AA AA GG AG AG AG AG AG
AG AA AG AG AA AA AA AA
AA AG AA AG AA AA AA
55602303
9728 rs1028806 13 AA AA AA AG AG
AG AA AA AA AA AG AG AG
AG AG AA AA AA AG AA GG
AA AA AA AA AA AA AA AA
AG AG AA AA AA AG AA
95119427
9729 rs1028818 6 AG AG GG AA AA
AA AA AA AA AA AA AA AA
AA AA AA AA AA AA AA AA
AA AA AA AA AA AA AA AA
AA AA AA AA AG AA AG
41586580
9730 rs1028819 1 AA AG AA AA GG
AG AG AA AA AA AA AA AA

AA AA AA AA AA AG AG AA
AG AG AG AG AA AA AA AA
AA GG AA AG AG AG AA
101222807
9731 rs1028850 13 AG AA AA AA AA
AG AA AA AG AG GG GG AG
AG AG AA AA AA AG AG AA
AG AG GG GG AA AA AA AA
AA AG AA AG AG GG AG
40941480
9732 rs1028879 9 AA AA AA AA AA
AA AG AG AG AA AG AG AG
AG AA AG AG AG AA AG AA
AG GG AG GG AA AA AA AA
AA AG GG GG AA AA AA
73406551
9733 rs1028880 9 AG GG AG GG GG
GG GG AG GG AG AG GG AG
AG GG AG AG GG GG AG GG
GG AG AG AG AA AA AA AA
AG AG AG AG GG GG GG
89444725
9734 rs1028905 20 GG AG AG GG GG
GG GG GG GG GG GG AG AG
AG AG AG AA AA GG GG AG
GG GG GG GG AA AG AG AG
AG AG AG AA GG AG GG
60037066
9735 rs1028915 10 AA AG GG AG AG
AG GG GG GG GG GG GG GG
GG AG AG GG AG GG GG GG
GG GG GG GG GG GG GG GG
GG AG AG AG GG GG GG
106265362
9736 rs1028935 10 CC AC AC CC CC
CC CC CC CC AC AC AC CC
CC CC CC CC CC CC CC CC
CC CC CC CC AC CC AC CC
CC CC AC AC CC CC CC
93349092
9737 rs1028945 8 AG AG AG AG AA
AG AG GG GG GG AA AA AG
AG GG GG GG AG GG GG AG
AG GG AG AG AG AG AA AG
GG AG GG GG AG AG AA
132022101
9738 rs1028961 6 AG AA AG AG AA
AA AG AG AA AG AG AG AG
AG AA AA AA AA AA AA AA
AA AA AA AA AA AG AG AG

AA AA AA AA AA AA AA
120229469
9739 rs1028985 21 AA AA AA AA AA
AA AA AA AA AA AA AA AA
AA AA AA AA AA AA AA AA
AA AA AA AA AA AA AA AA
AA AA AA AA AA AA AA
22711531
9740 rs1028990 21 AA AA AA AG AG
AA GG GG GG AG AG AG AA
AA AA -- AG AG AG AG AA
GG AG AA AA AG GG AG --
AA AG GG AG AG GG AG
29962377
9741 rs1029001 21 AG AG AG GG GG
GG AG GG GG GG GG GG GG
GG GG AG AG AG GG AA GG
GG GG GG GG AG AG AG AG
GG GG AA AG AG AG AA
39368145
9742 rs1029011 21 AG GG AG GG GG
GG AG GG GG AG GG GG GG
AG GG GG GG GG AG GG GG
AA AA AA AA AG GG AG GG
GG AG GG AG AG AA AG
42474200
9743 rs1029035 9 AC CC AC AA AA
AA AA AC AC AC AA AA AA
AA AA AC AC AC AA AA AA
AA AC AA AC AA AA AA AA
AA AA AA AA AC AA CC
78706951
9744 rs1029059 9 AA AA AA AA AA
AA AA AA AA AA AA AA AA
AA AA AA AA AA AA AA AA
AA AA AA AA AA AA AA AA
AA AA AA AA AA AA AA
83784070
9745 rs1029074 10 AG AA AA AG GG
AG AG AA AA AG AG AA AG
AG AG AA AA AA GG GG AG
AA AA AG AA AA AG AG AG
AG AG AA AA AA AA AA
90999036
9746 rs1029124 6 GG GG GG GG GG
GG GG GG GG GG GG GG GG
GG GG AG AG GG GG GG GG
GG GG GG GG GG GG GG GG
GG GG GG GG GG GG GG
10179366

9747	rs1029133		10	AA	AA	AG	GG	AG
	AG	AG	AG	AA	GG	AA	AG	AG
	AG	AA	AG	AG	GG	AG	AG	AG
	AG	AG	AG	AG	GG	AA	AG	AG
	AA	AG	AA	AA	AG	GG	AG	
85423893								
9748	rs1029134		10	CC	CC	AC	AC	CC
	CC	AC	AC	CC	AA	CC	AC	AC
	AC	CC	CC	AC	AC	AC	AC	AC
	AC	AC	AC	AC	AC	CC	AC	CC
	CC	CC	CC	CC	AC	AA	AC	
85423938								
9749	rs1029143		13	AC	AC	AC	CC	CC
	CC	CC	CC	CC	CC	AC	AC	AC
	AC	AC	CC	AC	AC	CC	CC	CC
	CC	CC	CC	CC	CC	CC	CC	CC
	CC	CC	CC	CC	CC	CC	CC	
86665005								
9750	rs1029158		6	AA	AA	AA	AG	AA
	AG	AA	AA	AA	AA	AA	AA	AA
	AA	AA	AA	AA	AA	AG	AG	AG
	AG	AG	AG	AG	AG	AA	AG	AG
	AA	AA	AA	AA	AA	AA	AA	
63250796								
9751	rs1029182		10	AG	AA	AG	AA	AA
	AA	AA	AG	AG	AA	AG	AG	GG
	GG	GG	AG	AG	GG	AG	AA	AG
	AA	AA	AA	AA	AA	AA	AA	AA
	AA	AA	AG	AA	AA	AA	AA	
1965937								
9752	rs1029282		3	AA	AG	AA	AA	AA
	AA	AA	AA	AA	AG	AA	AA	AA
	AA	AA	AA	AA	AA	AG	AG	AA
	AA	AA	AA	AA	GG	AG	AG	AG
	AG	AA	AA	AA	AA	AA	AA	
96738205								
9753	rs1029289		16	CC	AC	AC	CC	CC
	CC	CC	CC	CC	CC	AA	AC	AC
	CC	AC	CC	CC	CC	AC	CC	CC
	CC	CC	CC	CC	CC	AC	AC	CC
	CC	CC	CC	CC	CC	CC	CC	
13875238								
9754	rs1029290		16	AA	AG	AG	AA	AG
	AA	AG	AA	AA	AA	AA	AA	AA
	AA	AA	AG	AG	AA	AA	AA	AG
	AA	AG	AA	AA	AG	AA	AA	AG
	AG	AG	AG	GG	AA	AA	AA	
13941352								
9755	rs1029297		22	GG	GG	GG	GG	AG
	AG	AG	GG	GG	GG	AG	AG	GG

GG GG GG GG GG GG GG GG
GG GG GG GG GG AG GG AG
GG AG GG GG AG AG GG
34015991
9756 rs1029305 X GG AG AG AG GG
GG AG GG AG AA GG GG GG
GG GG AG GG AG GG GG GG
GG GG GG GG AG AA AG AA
AA GG AA GG AA AG AA
102339606
9757 rs1029321 6 AA AA AA AA AC
AA AC AA AA AA AC AC AA
AC AA AA AA AA CC CC AC
AA AA AA AA AC AA AA AA
CC AA AA AA AC AC AC
106140281
9758 rs1029322 1 GG GG GG AG GG
AG GG GG GG GG GG GG AG
AG AG AG AA AA GG GG GG
GG GG GG GG AA GG AG AG
GG GG GG GG GG AG GG
7396032
9759 rs1029396 7 AA AA AA AA AA
AA AA AA AA AA AA AA AA
AA AA AA AA AA AA AA AA
AA -- AA AA AA AA AA AA
AC AA AA AA AA AA AA
117024820
9760 rs1029413 17 AG AG AG AG AG
AG AA AG AA AA GG GG GG
AG AG GG AG AG AG AA GG
AA AG AA AG AG GG GG AG
GG GG AA AG AA AA AG
67829458
9761 rs1029466 7 AG AG AG AA AA
AA AA AA AA AA AA AA AA
AA AG AA AA AA AA AA AA
AA AG AG AG AG AG AA AA
AA AA GG AG AA AA AG
11512299
9762 rs1029496 X AA AA AA AA AA
AA AA AA AA AA AA AA AA
AA AA AA AA AA AG AG GG
AA AA AA AA AA AA AA AA
AA AA AA AA AA AA AA
24398091
9763 rs1029541 7 GG AG AG AG GG
GG AG GG GG GG GG GG AG
AG GG AG AG AG GG AG GG
AG AA AA AG GG GG GG GG

AG GG AG AG AG AG GG
84139004
9764 rs1029544 7 AC CC AC AC AC
AA CC AC AC AC AA AA AA
AA AA AC AA AA AC CC AA
AC AC CC AC AA AC AC AC
CC AC AA AC AC AC AC
14124065
9765 rs1029559 7 CC CC AC AC CC
CC AC AC AC AC AC AC AA
AA AA AC AC AA AC CC AA
AC AC AC AC AC AC AC AA
AC CC AC CC AC AC AC
26588410
9766 rs1029563 7 AA AA AA AC AA
AA AC AC AC AA AA AA AA
AA AA AA AA AA AA AA AC
AA AA AA AA AA AC AA AA
AC AA AC AC AC AA CC
84738495
9767 rs1029568 7 AG AG AA AG AG
AG AG GG GG GG AG AA AG
AG AA AG AA AA AA AA AA
AG AA AA AG GG AA AG AG
AA AG AA AA AA AA AA
81309505
9768 rs1029607 17 AC AA AC CC AC
CC AC AC AC AC CC CC AC
AC AC CC CC CC CC AC CC
AA AC AC CC CC CC CC CC
AC CC CC CC CC AC CC
69866254
9769 rs1029618 7 GG GG GG GG GG
GG GG GG GG GG GG GG GG
GG GG GG GG GG GG GG GG
GG GG GG GG GG GG GG GG
GG GG GG GG AG GG AG
42390706
9770 rs1029643 17 GG GG GG GG AG
GG AG AG AG AG AA AA AG
AG AG GG GG GG AA AG AA
AG GG AA AG AG GG GG AG
AG AG AG AA GG GG GG
70296032
9771 rs1029645 17 AA AA AA AA AG
AA AG AG AG AG GG GG GG
GG GG AG AA AA GG AG GG
AG AA GG AG GG AA AG AG
GG AG GG GG AG AA AG
70295760

9772	rs1029648	17	AG	AA	AG	AG	AG
	AG	GG	AG	AG	AG	GG	GG	GG
	GG	GG	GG	GG	GG	GG	GG	GG
	AG	AA	GG	AG	GG	AG	GG	GG
	GG	GG	GG	GG	GG	GG	GG	
70258662								
9773	rs1029652	19	AG	AG	GG	GG	GG
	GG	GG	AG	AG	AG	GG	AA	AG
	AG	AG	GG	GG	GG	GG	AG	GG
	GG	GG	GG	GG	GG	AG	GG	GG
	AG	GG	GG	GG	AG	AA	GG	
14978284								
9774	rs1029661	19	GG	GG	AG	AG	AG
	GG	AG	GG	AG	AG	AG	AG	GG
	AG	AG	GG	GG	GG	GG	GG	GG
	GG	AG	AG	GG	AG	GG	AG	GG
	GG	GG	GG	GG	AG	AG	GG	
28897301								
9775	rs1029663	19	--	AA	AC	CC	CC
	CC	CC	--	AC	--	CC	AC	AC
	CC	CC	AA	AA	AA	CC	CC	AC
	CC	--	--	CC	AC	CC	CC	--
	CC	AC	CC	AC	CC	CC	CC	
28898965								
9776	rs1029682	17	GG	GG	AG	GG	GG
	GG	GG	AG	AG	AG	GG	GG	GG
	GG	GG	AG	GG	AG	AG	AG	AG
	AG	AG	AG	AG	GG	AG	GG	AG
	GG	GG	AG	GG	GG	GG	AG	
13404176								
9777	rs1029709	19	AG	AG	AG	GG	GG
	GG	GG	AG	GG	GG	GG	GG	GG
	AG	AA	AG	GG	GG	GG	GG	GG
	GG	GG	GG	GG	GG	GG	GG	GG
	GG	GG	GG	GG	GG	GG	GG	
57046007								
9778	rs1029719	17	GG	AG	AG	GG	AG
	AG	GG	AA	AG	AG	AG	GG	AG
	GG	GG	GG	GG	AG	AG	GG	GG
	AG	AG	AG	AG	AG	GG	GG	GG
	GG	AG	AG	AG	AG	GG	AA	
32550864								
9779	rs1029723	17	AG	AG	AG	AA	AG
	AG	AA	AA	AG	AA	AA	AA	AA
	AA	AA	AG	AA	AA	AG	AG	AA
	AG	AG	AG	AG	AA	AG	AA	AA
	AG	AG	AG	GG	AG	GG	AA	
54767548								
9780	rs1029756	17	AG	AG	AA	AA	AA
	AA	AA	AA	AA	AA	AA	AA	AA

AG AG AG AG AG AA AA AA
AA AA AA AA AA AA AA AA
AA AA AA AA AA AA AA
68725240
9781 rs1029757 17 GG GG GG AG GG
AG GG AG AG GG AG AG GG
GG GG GG GG GG GG GG GG
GG AG AG AG GG GG GG GG
GG GG AG AG GG GG AG
14929127
9782 rs1029809 17 AG AG AA AG AA
AG AA AA AA AG AG GG AG
AG AG AG GG AG GG AG GG
AG AG AG AG AG AG AG GG
AG AG AG AG AG AA AG
16156235
9783 rs1029859 X AA AA AA AA AA
AA AA AA AA AA AA AA AA
AA AA AA AA AA AA AA AA
AA AA AA AA AA AA AA AA
AA AA AA AA AA AA AA
27449542
9784 rs1029864 2 GG AG AG GG AG
GG AG GG GG GG AG AG AA
AG AG AA AG AG AA AG AA
AG GG AA AA AG GG GG AG
AA AA GG AG GG GG GG
107784393
9785 rs1029867 4 GG GG GG GG GG
AG GG GG AG GG GG GG GG
GG AG GG GG GG AG AG AG
AA AG AA AG GG AG GG AG
AG GG AA AG AG AG GG
11311393
9786 rs1029888 7 AA AA AG AA AA
AG AA GG AA AG AA AA AA
AA AA AG AG AG AA AA AA
AG AG AG AG AA AA AA AA
AG AA AA AA AA AG AG
148469716
9787 rs1029905 X GG GG GG GG GG
GG GG GG GG GG GG GG GG
GG GG AG AG GG GG GG GG
GG GG GG GG GG GG GG GG
GG AG GG GG AG GG AA
116672213
9788 rs1029931 12 AG AG AA AA AA
AA AA AA AG AG AA AA AA
AA AA AA AA AA AG AG AA
AG AG AA AG AG AA AG AG

AG AG AA AA AA AG AA
21629313
9789 rs1029938 15 GG GG GG AA AA
AA AA AA AA AA AG AG AG
AA AG AG GG AG GG GG AG
GG GG GG GG AG AG AG AG
AA AG AA AA AG AA AG
27372071
9790 rs1029956 7 AG AG AG GG GG
AG AG AG AA AA GG GG GG
AG AA GG AG AG GG GG GG
AG GG AG AG GG AG AG AG
AG AG GG AG AG GG AA
153534165
9791 rs1029965 7 GG AG GG GG AG
GG AG GG GG GG GG GG GG
GG GG GG GG GG GG GG GG
GG GG GG GG GG GG GG GG
AG GG GG GG AG GG AG
30418224
9792 rs1029973 4 GG GG GG GG GG
AG GG AG AG AG GG GG GG
GG GG AG GG AG GG GG AG
GG GG GG GG GG GG GG GG
GG GG AG GG AG AG AG
15438851
9793 rs1029990 12 AA AA AA AA AA
AA AG AA AA AA AA AA AG
AG GG AG AA AA GG GG AG
GG GG AG GG GG AG GG GG
AA AG AG AG AG AG AG
9926397
9794 rs1029999 7 AA AG AG AA AG
AG AA AG AG AA AG AG AG
AG AG AA AA AA AA AG AA
AA AG GG AA AG AG AA AG
AA AG AA AA AG GG AA
93904262
9795 rs1030008 7 AA AG AA AG AA
AG AA AG GG GG AA AG AA
AA AA AG AA AA GG GG AG
AG GG AG GG AA AA AA AA
GG AA AG AG AG AG AA
9072385
9796 rs1030009 19 AA AA AG AA AG
AG AA GG AG AG AG AG AG
AG AG GG AG AG AG AG AG
AG AG GG AA AG AA AG AA
AA AG AA AA AA AA AA
46788020

9797 rs1030015 7 AC AA AC AC AC
AA AC CC CC AC CC CC CC
AC AC AC CC CC AA AA AC
AA AC AC AA AC AC CC AC
AC AC AC AC CC CC AC
78139581
9798 rs1030016 7 AC AA AC CC CC
CC CC CC CC AC AA AC AC
CC CC CC CC AC CC CC CC
CC CC CC CC CC AC CC CC
AC CC CC CC CC CC CC
38439572
9799 rs1030078 14 AG AA AA AA AA
AA AA AA AG AA AA AA AA
AA AA AA AA AA AA AG AA
AG AA AG AA AA AA AA AA
AA AA AA AA AA AA AG
87456796
9800 rs1030080 8 AG GG AG AA AA
AA AG AG AG AG AG AG AG
GG GG AG GG AG AG AG AA
AA AA AA AA AG AG GG AG
AG AG AA AA AG AG GG
5194048
9801 rs1030092 2 AG AG AA AA AG
AA AG AA AG AG GG GG GG
AA AA AA AA AA AG AG AG
AA AG AA AG AG AG AA AG
AA GG GG GG AA AA AA
56617866
9802 rs1030105 8 AG AG AG AA AA
AA AA AG AG AA AG AG AA
AA AG AG GG AG AG GG AG
AA AG AA AG AA AA AA AA
AA AA AG AA AA AA AA
129127793
9803 rs1030110 18 AG AG AG AG AG
AG GG GG GG GG AG AG AG
AG AG GG AG AG GG GG GG
AG GG GG AG AG GG AG GG
AA AA AG AG GG GG GG
21937021
9804 rs1030121 1 GG GG GG GG AG
GG AG GG GG GG GG GG GG
GG GG GG AG AG AG AG GG
AG GG AA AG AG GG GG AG
GG GG GG GG GG GG GG
42522715
9805 rs1030127 14 AG AA AA AA AA
AA AA AG AA AG AG AA AA

AA AA AG AA AG AG AG AA
AA AA AA AA AA AG AG AG
AA AA AG AA AA AA AA
80161440
9806 rs1030154 5 GG GG GG GG GG
GG GG AG AG AG AG AG AG
AG GG GG GG GG GG GG GG
GG GG GG GG AG GG AG AG
GG GG GG GG AG GG AG
165051330
9807 rs1030198 18 AA AG AA GG GG
AG AG GG AG GG AA AA AA
AA AA AG AG AA AG AA AG
AA AG AG AA AG AG AG GG
AA AA AG AA GG AG AG
24479960
9808 rs1030219 16 AA AA AA AG AG
AG AG AA AA AA AG AG AG
AG AA AA AA AA AA AA AA
AG AG GG AG AA AA AA AA
AA GG AG GG AG AA AG
26541993
9809 rs1030227 5 GG GG GG GG GG
GG GG AG AG AG GG GG GG
GG GG GG GG GG GG GG GG
GG GG GG GG GG GG GG GG
GG GG GG GG GG GG GG
52786688
9810 rs1030228 5 AA AG AG AG AG
AA AG GG GG GG AG GG AG
GG AA GG GG GG AG GG AG
AG AG AG AG AG GG GG AG
GG AG GG AG GG GG GG
52842569
9811 rs1030278 2 AG GG AG AG GG
AG GG AA AA AG AG AG AG
AG AG AG AG GG AA AA AG
GG GG GG GG AA AA AA AA
AG GG AA AG AG AG AA
38435241
9812 rs1030304 9 AA AA AA AA AA
AA AA AA AA AA AA AA AA
AA AA AA AA AA AA AA AA
AA AA AA AA AA AA AA AA
AA AA AA AA AA AA AA
88165749
9813 rs1030321 2 AG AG GG AA GG
AG AG AA AA AA AG GG GG
AG AG AG GG AG GG GG GG
AG GG AG GG GG AA AG AG

	GG	AA	AG	AG	AG	AG	AG	
60135937								
9814	rs1030328		2	AG	AG	GG	AA	AG
	AG	AA	GG	AG	AG	AG	AG	AG
	GG	GG	AG	AA	AA	AG	AA	AG
	AA	GG	AG	AG	AG	AG	AG	AA
	AG	AG	AG	GG	AA	AG	AG	
160386260								
9815	rs1030356		4	GG	AG	GG	GG	GG
	GG	GG	GG	GG	GG	GG	AG	GG
	GG	AG	AA	AG	AG	GG	GG	GG
	GG	GG	GG	GG	GG	GG	GG	GG
	GG	AG	GG	AG	AA	AG	AA	
141826780								
9816	rs1030370		8	GG	GG	GG	GG	GG
	AG	GG	GG	GG	GG	AG	AA	AG
	AA	AG	AG	AG	AG	GG	GG	AG
	AG	GG	GG	GG	GG	AG	AG	AG
	GG	GG	AG	GG	AA	AA	AG	
41310010								
9817	rs1030392		11	GG	GG	GG	AG	AG
	AG	AG	GG	GG	AG	GG	AG	GG
	AG	AG	GG	AG	AG	AG	GG	GG
	AG	AA	AG	AG	AG	AG	GG	AA
	GG	AG	AG	GG	GG	GG	AG	
4443472								
9818	rs1030406		2	AA	AA	AA	AA	AA
	AA	AA	AA	AC	AC	AA	AA	AA
	AC	AC	AA	AA	AA	AC	AC	AA
	AA	AC	AA	AC	AC	AA	AC	AC
	AC	AC	CC	CC	CC	CC	CC	
64517459								
9819	rs1030439		3	GG	GG	GG	GG	GG
	GG	GG	GG	GG	GG	GG	GG	GG
	GG	GG	AA	AG	AG	GG	GG	GG
	GG	GG	GG	GG	GG	GG	GG	GG
	GG	GG	GG	GG	AG	GG	AG	
69758910								
9820	rs1030467		11	AA	AA	AA	AA	AA
	AA	AA	AA	AA	AA	AG	AA	AG
	AA	AG	AG	AA	AG	AG	AA	AG
	AA	AA	AA	AA	AA	AG	AG	AA
	AA	AA	AG	AG	AA	AA	AA	
129744252								
9821	rs1030565		18	AC	CC	CC	CC	AC
	CC	AC	CC	CC	CC	AC	AC	AC
	AC	CC	AC	CC	AC	CC	CC	AC
	CC	AC	AC	AC	CC	AC	CC	AC
	AC	CC	CC	CC	CC	CC	CC	
73763670								

9822 rs1030567 18 AG AG GG AG AG
AG GG GG GG GG GG GG GG
GG AG AG GG AG AG AG AG
GG GG AG GG AG AA AA AA
GG GG AG AG GG AG GG
73851672
9823 rs1030569 18 GG GG GG GG GG
AG GG AA AA AG AG AA AG
AG AG AG AG GG AG AG AG
AG GG GG GG AG GG GG GG
AG GG GG GG GG GG AG
73851795
9824 rs1030593 18 AG AG AG AA AA
AG AA AG AG GG AA AA AA
AA AA AA AG AG GG AG AG
AG AG AG AG AA AA AA AA
AA AG AA AA AA AG AG
50374155
9825 rs1030599 2 GG GG GG AG AG
GG AA AA AA AA AA AA AG
AG AG AA AG AA AA AA AG
AG AA AG AA GG AA AG AG
AA AA AG AA AG AG AA
157141280
9826 rs10306163 9 AA AG AA AA AA
AA AG AA AG AA AA AA AA
AA AA AA AA AG AA AA AA
AA AG AA AG AG AA AG AG
AG AG AG AG AG AA AG
125149970
9827 rs1030635 5 AA AA AA AA AA
AA AA AA AA AA AA AA AA
AA AA AA AA AA AA AA AA
AA AA AA AA AA AA AA AA
AA AA AA AA AA AA AA
30585860
9828 rs1030655 3 GG AG AG GG GG
AG AG AG AG AG GG GG AG
AG AA GG AG AG AG AG AG
AA AA AG AA AG GG AG AG
GG AA AA AA AG GG AA
127773676
9829 rs1030668 5 AG GG AG GG GG
GG GG GG GG GG AG AG AG
AG AG GG GG GG GG GG GG
GG GG GG GG GG GG GG GG
GG GG GG GG GG GG GG
166876427
9830 rs1030669 5 AG AG AG AA AG
AA AG GG GG GG GG GG GG

AG GG AA AG AG AA AA AG
AG AA AG AG AG AG AG AG
AA AG AG AA AA AG AG
166881177
9831 rs103067 13 AG AG AA GG GG GG
GG GG GG GG GG GG GG GG
GG GG GG GG AG AG GG GG
GG GG GG GG GG GG GG GG
GG GG GG GG GG GG 112713156
9832 rs1030674 5 AA AC AA AC CC
CC AC AA AA AA AC AC AA
AA AA AA AA AA AC AA AA
AC AC AC AC AA AC AA AA
AC AA AC AA AA AA AC
163753090
9833 rs1030700 4 AG AG GG AG AG
AA GG AA AA AG GG AG GG
GG GG GG GG GG GG GG AG
GG GG GG AG GG GG GG GG
GG AG GG AG GG AG GG
35912892
9834 rs1030726 11 AC AC CC AC AC
AC AC CC AC AA CC AC CC
CC CC AA AA AA AC AC CC
AC AC AC AC CC CC CC CC
AC CC AC CC AC CC AA
4790268
9835 rs1030734 2 AA AA AA AA AA
AA AA AG AA AG AA AA AA
AA AA AA AG AG AA AA AA
AG AG AG AG AA AG AA AG
AA AA AG AG AA AA AA
80171888
9836 rs1030738 7 AC CC AC AC AC
AA CC AC CC AA AA AC AC
AC CC AA AC AC CC CC CC
AA AA AA AA CC AC CC CC
CC AC CC AC AA AA AC
1636539
9837 rs1030790 4 GG GG GG AG AG
GG AA GG GG GG GG GG AG
GG AG GG GG GG AG AG AG
GG GG GG GG GG AG AG GG
AG AG GG GG AG AG AG
182318445
9838 rs1030810 12 AC CC AC AA AC
AA AC AC CC AC AC CC AC
CC CC AC AC AC AC AC AC
CC CC CC CC AC CC AC AC
CC CC CC CC AA AC AC

92969355
9839 rs1030845 1 AA AA AA AA AG
AG AA AG AA AG AA AA AA
AA AA AA AA AA AA AA AG
AA AA AA AA AG AA AA AA
AG AG AA AA AG AA GG
217116732
9840 rs1030891 2 AA AA AG AG AG
AG AA AA AA AA AG AG GG
GG GG AA AA AA AA AA AA
AA AA AA AA AA AA AA AA
AA GG AA AG AG AG AA
240539765
9841 rs1030901 2 AG AA AA AG AG
AG GG GG GG GG GG AG AG
AG AG GG AG AG AA AA AA
AG GG AG AG GG AA AG AG
AG AA AG AA AG AA AG
101007314
9842 rs1030956 2 AA AA AG AA AA
AA AA AA AA AA AA AA AA
AA AA GG AG AG AA AA AA
AG AG AG AG AA AG AG AA
GG AA AG -- AG AG AA
77411737
9843 rs1030976 14 CC AC AC CC CC
CC CC AC AC AC AC AA AC
AC AC AC AC AC CC CC AC
CC CC AC AC AC AC AC AC
AC CC AC AC CC AC CC
33925740
9844 rs1030980 8 AG AG AA AG AG
AG AG GG AG AG AA AG AG
GG GG AA AG AA AG AG AA
AA AA AA AA AA AA AA AA
AA AA AA AA AG AA GG
122815018
9845 rs1030982 18 AG GG AG AG GG
GG AG AG AA AA AG GG GG
AG AA AG AA GG AG AG AA
GG GG AG GG AG AG AA AG
AG AG AG AA AA AA AA
68052952
9846 rs1031062 18 AA AA AA AC AC
AC AA AA AA AA AA AA AA
AA AA AA AA AA AA AA AA
AA AA AA AA AA AC AC AC
CC AC AC AA AA AA AA
50038122
9847 rs1031092 2 CC CC CC CC AC

AC CC AC AA AC CC CC CC
CC CC AC CC AC CC CC AC
CC CC CC CC AC AC AC AC
CC CC CC CC AC AC AC
24680249
9848 rs1031107 17 AA AA AA AG AG
AA AG AA AG AG AA AG AG
AA AG GG GG GG AG AG GG
AG AG AG GG AG AA AG AG
AG AA AA AA AG GG AA
33766947
9849 rs1031116 18 GG GG GG GG GG
GG GG GG GG GG GG GG GG
GG GG GG GG GG GG GG GG
GG GG GG GG GG GG GG GG
GG AG GG AG AG AG GG
7861456
9850 rs1031144 8 GG AG AG AG GG
GG AG AG AG AG GG AG GG
AG AA AG AG GG GG GG AG
AG GG GG AG AG GG GG GG
AA AG AA AA AA AA AA
53969642
9851 rs1031176 11 AA AA AG AA AA
AA AA AA AA AA AA AA AA
AG AG AA AA AA AA AA AA
AA AA AA AA AA AA AA AA
AA AA AG AA AA AA AA
27075551
9852 rs1031178 8 GG AG GG GG GG
GG GG GG GG GG GG GG GG
GG GG AG AG GG GG GG GG
GG GG GG GG GG AG AG AG
AG AG AG AG GG GG GG
72224557
9853 rs1031210 Y GG -- -- -- GG
GG -- -- -- GG -- -- --
-- -- -- -- -- -- -- GG
AA AA AA -- -- GG -- GG
-- -- GG GG -- -- GG
18957208
9854 rs1031232 11 AA AA AA AG AG
GG AA AG AG AG AA AA AA
AG AA AG AG AA AA AA AA
AG AG AG AG AG GG AG GG
AA AA AA AA AA AG AG
57872281
9855 rs1031238 8 AA AA AA AG AG
AG AG AA AG AG AG AA AG
AG AA AG AG AA AA AA AG

AA AA AA AA AA AA AA AA
AA AG AA AG GG AG AG
94290812
9856 rs1031246 3 AA AA AA AA AA
AA AA AG AG AA AA AA AA
AA AA AA AA AA AG AA AG
AA AA AA AA AA AA AA AA
AG AG AA AA AA AA AA
63028072
9857 rs1031262 8 CC AC CC AC CC
AC CC AC AC AA AC AA AC
AC AC AC AC AC AC AC AC
AC AC CC CC AC AC AC AC
AC CC CC CC AC AC CC
141985905
9858 rs1031267 2 GG GG GG GG GG
GG GG GG GG GG GG GG GG
GG GG GG GG GG GG GG GG
AG GG AG AG AG GG GG GG
GG GG GG GG AG GG AG
6428941
9859 rs1031274 7 GG GG GG AG GG
AG GG GG GG GG GG GG GG
GG GG GG GG GG GG GG GG
GG GG GG GG GG GG GG GG
GG GG GG GG GG GG GG
17566650
9860 rs1031318 4 AG AG AG AG AG
GG AA AG AG AG AG AG AG
AG AA AA AG AG GG AG GG
AG GG GG GG GG AA AG AG
AG GG AG GG AG AG AG
163315293
9861 rs1031333 16 GG GG GG GG GG
GG GG GG GG GG GG GG GG
GG GG GG GG GG GG GG GG
GG GG GG GG GG GG GG GG
GG GG GG GG GG GG GG
56053163
9862 rs1031334 16 AG AG AG AG AG
GG AA AA AA AA GG GG GG
GG AG AG AG GG AG AG AG
AG AA AG GG AG AG GG AG
AG GG GG GG GG AG AG
56108703
9863 rs1031352 2 GG GG GG GG GG
GG GG GG AG GG AA AA AG
AG AG AG AG AG AG AG GG
GG AG AG GG GG AG GG AG
GG AG GG GG GG GG GG

2619629								
9864	rs1031360		11	AG	GG	AG	AG	AG
	AA	GG	AG	AA	AA	AA	AG	AG
	AA	AG	AG	AA	AG	GG	GG	AG
	AA	AA	AA	AA	AG	AA	AG	AA
	AG	AG	AA	AG	AA	AA	AA	
57871866								
9865	rs1031399		8	AC	CC	AC	AA	AA
	AA	AA	AC	AC	AA	AA	AA	AA
	AA	AC	AA	AA	AA	AC	AC	AA
	AC	AC	AC	AA	AA	AC	AA	AA
	AA	AA	AA	AA	AC	AC	AA	
63461952								
9866	rs1031420		4	AC	AC	AC	AA	AC
	AC	AA	AC	AC	AC	CC	AC	AC
	AC	AC	AA	AA	AA	AC	CC	AC
	AA	AC	AA	AC	AA	AA	AA	AA
	AA	AA	AC	AC	AA	AC	AA	
72147944								
9867	rs1031434		2	AG	AA	AG	AG	AG
	GG	AA	AA	AG	AG	AA	AG	AA
	AA	AA	AA	AA	AA	AA	AG	AA
	GG	GG	AG	AA	AA	GG	AG	AG
	GG	AA	AG	AA	AA	AA	AA	
207750902								
9868	rs1031436							
Y	--	--	--	--	--	--	--	--
	--	--	--	--	--	--	--	--
	--	--	--	--	--	--	--	--
	--	--	--	--	--	--	--	--
	--	--	--	--	3480985			
9869	rs1031477		12	AG	AG	GG	AG	GG
	GG	AG	GG	AG	GG	AA	AA	AA
	AA	AA	AA	AG	AG	AG	GG	GG
	GG	GG	GG	GG	AA	AA	AA	AA
	AA	AG	GG	AG	AG	AA	GG	
50218644								
9870	rs1031490		7	AA	AA	AG	AA	AG
	AG	AA	AA	AA	AG	AA	AA	AA
	AG	AG	AA	AA	AA	AA	AA	AA
	AA	AA	AA	AA	AA	AA	AA	AA
	AA	AA	AA	AA	AA	AA	AA	
54651526								
9871	rs1031500		4	AG	GG	GG	GG	GG
	GG	GG	GG	GG	GG	GG	GG	GG
	GG	GG	GG	GG	GG	AG	AG	AA
	GG	GG	GG	GG	GG	AG	AG	GG
	AG	GG	GG	GG	GG	AG	GG	
182187373								
9872	rs1031509		2	CC	CC	CC	AC	AC

CC AC AA CC CC AC AC AC
AC CC CC CC -- CC AC CC
CC CC CC CC CC AC AC CC
CC CC AC CC AC AA AC
192010189
9873 rs1031567 1 GG GG GG AG AA
AG AG AA AA AA AA AA AA
AA AA GG GG GG GG GG GG
AG AG GG AG AG AG AG AG
AG AG GG AG GG AG GG
18377790
9874 rs1031588 8 CC CC CC CC CC
CC CC AC AC AC CC CC CC
CC CC CC AC CC CC CC AC
CC CC CC CC AC CC CC AC
CC CC CC CC CC CC CC
128093277
9875 rs1031602 9 AG AA AG AG AG
AA AG AG AG AG GG GG AG
AG AA AG AG AG AA AA AG
AA AG AA GG GG AG AG AG
AG AG GG AG GG AG GG
20272411
9876 rs1031609 19 GG GG GG AA AG
AG AA GG GG GG GG AG GG
AG AG GG GG GG AA AA AA
AG GG GG AG AG AA AG AA
AG GG AG AG AG AG GG
28511434
9877 rs1031644 15 AA AA AA AC CC
AC CC AC AC AC AA AA AA
AA AC AC CC CC AA AA AC
AC CC CC AC AC AC CC AA
AA CC AA AC AC CC AA
95509944
9878 rs1031675 1 GG GG GG GG AG
AG GG AG AG GG AG GG GG
GG GG GG GG GG GG GG GG
GG GG GG GG GG AG AG GG
GG AG AG GG AG AG AG
221605304
9879 rs1031735 2 GG GG GG GG GG
GG AG GG GG GG AG GG GG
GG GG GG GG GG AA AG AA
AG GG GG AG GG GG GG GG
GG GG AG AG GG GG GG
143397450
9880 rs1031773 2 GG GG GG GG GG
GG GG GG GG GG GG GG AG
AG AG AG AG GG AA GG AA

GG GG GG GG GG AG AG GG
GG AG AA AG AG AA GG
170771022
9881 rs1031783 14 GG GG GG GG GG
GG GG GG GG GG GG GG GG
GG GG GG GG GG GG GG GG
GG GG GG GG GG GG GG GG
GG GG GG GG GG GG GG
98320023
9882 rs1031794 4 AA AA AA AG GG
AG AG AA AA AG AG AA AG
GG GG GG AG AG AA AA AA
AG AG AG AG GG GG GG GG
GG AG GG AG AG AG AG
88451182
9883 rs1031796 4 CC CC CC AC AA
AC AC CC CC AC AC CC AC
AA AA AA AC AC CC CC CC
AC AC AC AC AA AA AA AA
AA AC AA AC AC AC AC
88451579
9884 rs10318 15 GG GG GG GG GG GG
GG GG GG AG AG GG GG GG
GG AG GG AG GG GG GG GG
GG GG GG GG GG GG GG AA
AG GG AG GG GG AG 33025979
9885 rs1031808 2 GG GG GG AG AG
GG AA GG GG GG AG GG GG
GG GG GG GG GG GG AA GG
GG AG GG AG GG AA AG AG
GG GG GG GG GG GG GG
157591413
9886 rs1031837 3 AG AG AG AA AG
AG AG AG AA AA AG AG AA
AA AA AG GG AA GG GG AG
AG AG AG AG AG AA AG AA
AA AG AG AG AG AA AG
162237495
9887 rs1031857 13 AG AG AA AG AA
AA AG GG GG GG AG AG GG
AG AG AG AG AA AG AA AG
AG AG GG GG AG AA AA AA
AG AG AG GG AA AG AG
81775869
9888 rs1031862 1 AA AG AG GG AG
AG AG AG GG AG GG AG AG
GG GG AG AG AG AG AG AG
GG GG GG GG AG GG AG AG
GG GG GG GG AG GG AA
237727970

9889 rs1031874 8 AC AC AC CC AC
AC AC AA AA AA AA AA AA
AA AA AA AA AA AA AC AA
AC AA AA AA AA AA AA AA
AA AA AA AA AA AA AA
83273087
9890 rs1031895 12 AC CC AC AC AC
AA CC AA AA AA AC AA AA
AA AA CC AC AC AA AA AA
AC AC AC CC AA CC AC AC
AA AC CC AC AC AA AC
23732165
9891 rs1031910 7 AG GG GG AA GG
AG AG GG AG GG GG GG GG
AG AG GG GG GG AG GG AG
AG AG AG AG GG GG GG GG
AG AG AG AA AG AG AA
52024776
9892 rs1031914 X GG AG AA AA GG
AA AG GG GG GG AG AG AG
AG GG GG AG AG AG AG GG
AA AA AA AG GG AA AG GG
AG AG GG GG AG AG AA
15172189
9893 rs1031915 5 AA AA AA GG GG
GG GG GG GG GG GG GG GG
GG GG GG GG GG GG AG GG
GG GG AG AG GG GG GG GG
AG AG GG AG GG GG GG
146429843
9894 rs103194 6 GG GG GG GG GG GG
GG AG AA AG GG GG AG GG
AG GG GG AG GG GG GG AG
AG AG AG GG AG AG AG GG
GG GG GG GG GG GG 80569885
9895 rs1031996 12 AG AG AG AA AG
AA AG AG GG GG GG GG AG
GG GG AG AG AG GG AG GG
AG GG AG GG GG AG GG GG
AG AA AG AG AG AA GG
40527395
9896 rs1032015 14 AG AG AA AG AG
GG AA AA GG AA AG AA AG
AG AG GG AG AG AA AA AA
AG AG AA AA AG AA AA AG
AG AA GG AG AG AG AG
34401104
9897 rs1032036 X GG GG GG GG AA
GG AG GG GG GG GG GG GG
GG GG GG GG GG GG GG GG

	GG	GG	AA	GG	GG	GG	GG	GG
	GG	AG	GG	GG	GG	GG	GG	
5620255								
9898	rs1032044		3	AA	AA	AA	AA	AC
	AA	AC	AC	AC	AC	AA	AA	AC
	AC	AC	AA	AA	AC	AC	AC	AC
	AA	AA	AA	AA	AA	CC	AC	AC
	AC	AC	AC	AA	AA	AA	AC	
156707330								
9899	rs1032051		4	AA	AA	AA	AG	AG
	AA	GG	GG	GG	AG	AG	AG	AG
	AG	AA	AG	AA	AA	AG	GG	AG
	AA	AA	AA	AA	AG	GG	AG	AG
	AA	GG	AA	AG	AA	AG	AG	
156499985								
9900	rs1032070		17	AG	AG	AG	GG	GG
	GG	GG	AA	AA	AA	GG	GG	GG
	GG	AG	AG	AG	AG	AG	AG	AG
	GG	AA	AA	AG	GG	GG	GG	GG
	GG	GG	AG	AG	GG	GG	GG	
40618251								
9901	rs1032077		3	AC	AC	AC	CC	CC
	CC	CC	AC	CC	AC	AC	AC	AC
	CC	AC	AC	AC	AC	CC	CC	AC
	CC	AC	CC	AC	CC	CC	CC	CC
	CC	CC	AA	AC	AC	AC	CC	
176025504								
9902	rs1032080		12	AG	AA	AG	AA	AA
	AA	AA	AA	AA	AA	AA	AA	AA
	AA	AA	AA	AA	AA	AA	AA	AA
	AG	AG	AA	AG	AA	AA	AA	AA
	AA	AA	AA	AA	AA	AA	AA	
31895011								
9903	rs1032119		18	GG	GG	GG	GG	GG
	AG	GG	GG	GG	GG	GG	GG	GG
	GG	GG	GG	GG	GG	AG	GG	GG
	GG	GG	GG	GG	AG	GG	AG	AG
	GG	AG	GG	GG	GG	GG	GG	
43329719								
9904	rs1032233		11	AG	AA	AG	AG	GG
	AG	GG	GG	GG	GG	AG	GG	AG
	GG	AG	AG	AA	GG	GG	GG	GG
	GG	AG	GG	AG	GG	GG	GG	GG
	GG	AG	GG	AG	GG	AG	GG	
103838023								
9905	rs1032290		8	AG	AA	AA	AG	GG
	AG	GG	AA	AA	AG	GG	GG	GG
	GG	AG	AG	AG	AA	AG	AA	GG
	AG	GG	AG	GG	AG	AG	AG	GG
	GG	AG	AG	AG	AA	AG	AG	

127731869
9906 rs1032296 4 GG AG GG AG AG
AG AG AG AG AG GG GG GG
GG GG GG AG AG GG GG AG
AG AG AG AG AG AA AA AA
GG GG AG AG AG AA AG
145434688
9907 rs1032297 4 AG AA AG AG AG
AG AG AG AG AG AG AG AA
AA AG GG AG AG AG AG AG
AG AA AG AG AG AA AA AA
GG GG AG AG AG AA AG
145434744
9908 rs1032366 9 GG GG GG AA AG
AG AG GG GG GG AG AG GG
GG GG GG GG GG AG AG GG
GG GG GG GG GG GG GG GG
GG GG GG GG GG GG GG
76670504
9909 rs1032380 3 AA AA AA AA AA
AA AA AA AA AA AA AA AA
AA AA AA AA AA AA AA AA
AA AA AA AA AA AA AA AA
AA AA AA AA AA AA AA
94199360
9910 rs1032412 2 AA AA AA AG AG
AA GG AG AG AG AG AG GG
GG GG AA AA AA AG AG AG
AA AA AA AA AA AG AA AA
AA AG AG AG AA AG AA
163639107
9911 rs1032429 1 AG AG AG AA AA
AA AA AA AA AA AA AA AA
AA AG AA AA AA AA AA AA
AA AA AA AA AA AA AA AA
AA AA AA AA AA AA AA
111151567
9912 rs1032438 4 AG AG AG AG AG
AA GG AG AA AA AA AA AG
AG AA AG AG AG AA AA AA
AA AA AA AA AG GG GG GG
AA AG AA AG AA AA AA
134463193
9913 rs1032442 11 AA AA AA AA AA
AA AA AA AG AG AG AG GG
AG AG AA AG AG GG AG GG
AA GG AA AG AG AG AG AG
AA GG AG GG AG GG AA
129324220
9914 rs1032466 14 AC AC AC AC CC

CC AC AC AA AC AA AA AA
AA AC AC AA AC AA AA AA
AC AA AC AC AC CC AC AC
AA CC CC CC AC AC AA
51074461
9915 rs1032491 5 AG GG GG GG GG
GG GG GG GG GG GG GG GG
AG AG GG GG GG GG GG GG
GG GG GG GG GG GG GG GG
GG AG GG GG GG GG GG
116671392
9916 rs1032502 4 AA AG GG GG GG
GG GG GG GG GG AG GG AG
AG AG AG GG AG AG AG AG
GG GG GG GG AG GG AG AG
GG GG GG GG GG AG GG
11547106
9917 rs1032506 3 AA AA AA AA AA
AA AA AA AA AA AA AA AG
AA AG AG AA AA AA AA AA
AG AA AA AG AG AA AA AA
AA AA AA AA AA AA AA
84911212
9918 rs1032601
1 -- -- -- -- -- -- -- --
-- -- -- -- -- -- -- --
-- -- -- -- -- -- -- --
-- -- -- -- -- -- -- --
-- -- -- -- 217507115
9919 rs1032608 1 GG GG GG AG GG
AG GG GG GG GG GG GG GG
-- GG AG GG GG GG GG GG
GG GG GG GG GG AG GG GG
AG GG AG GG GG GG GG
167131321
9920 rs1032622 1 AA AA AA AA AA
AC AA AA AA AC AA AA AA
AA AA AA AC AC AA AA AC
AA AA AA AA AA AA AA AA
AA AA AA AA AA AA AA
61447172
9921 rs1032669 3 AG GG AG GG GG
AG AG GG AG GG AG AG GG
GG AG AG AA AG AG AG AG
AG AG AG AG AG AG AG AG
GG AG AG AG GG GG AG
1183679
9922 rs1032673 4 AC AC AC AA AA
AA AC AC CC AC AA AA AA
AA AA AA AA AA AC AC AC

AA AA AA AA AA AC AA AC
AC AA AA AA AC AC AC
129465489
9923 rs1032748 5 AG AA AA AA AG
AG AG AG GG AG GG GG AG
GG GG AG AG AA AG GG AA
AG GG AG GG AG AG AG GG
AG AG AG AA AA AG AA
5903890
9924 rs1032763 5 GG GG GG GG AG
AG GG AG GG AG AG AG GG
AG AG AG AG GG AG AG AA
AG AG GG AG AG AG AG GG
AG AA AG AA GG GG GG
17118930
9925 rs1032778 11 AC AA AA AC AC
AC AC AA AC AC AA AA AA
AA AA AC CC AC AA AA AA
AC AA AC AA AC AA AA AA
AA AC AC CC AA AA AA
100512650
9926 rs1032779 11 CC CC CC CC CC
CC CC CC CC CC AC CC AC
CC AC AC CC CC CC CC AC
AC AC AC AC AC AC AC AA
AA CC CC CC CC AC AC
100513913
9927 rs1032807 2 AA AA AA AG GG
GG AG AA AA AA AG AA AA
AG AG AG GG AA AG AG AG
AG AA AG AA AA AG AG AA
AA AG AG AG AA AA AA
68166080
9928 rs1032843 2 AG AG AA GG GG
GG GG GG GG GG GG GG AG
AA AA AA AG AG AA AG AG
GG GG GG GG GG GG GG GG
AG AG AG GG GG GG GG
164184329
9929 rs1032844 3 AA AG AA AG AA
AA AG AA AA AA AA AA AA
AG AG AA AA AA AA AA AA
AG AG AA AG AG AA AG AA
AA AA AA AA AG AA AG
153453684
9930 rs1032886 12 AA AC AA AC AC
AC AC AA AA AC AA AA AA
AA AA AA AA AA AA AA AA
AA AA AA AA AA AA AA AA
AC AA AA AA AA AA AA

44529060
9931 rs1032915 18 AG AG AG AA AA
AA AA AA AA AA AA AA AA
AA AA AG AG AA AA AG AA
AA AA AG AA GG AG AG GG
AG AA AA AA AA AA AA
75479829
9932 rs1032928 8 GG GG GG GG GG
GG GG GG GG GG GG GG GG
GG GG AA AG AG AG AG AG
AG AA GG AG GG GG GG GG
GG GG AG AG AG AG AG
140029711
9933 rs1032939 11 AC AC CC AA AA
AA AC AC CC CC AC AC CC
CC CC AA AC AA AC AC AC
CC AC CC AA AA AA AA AA
AA AA AA AA AC CC AA
60531346
9934 rs103294 19 GG GG GG GG GG GG
GG GG GG GG GG GG GG GG
GG GG GG GG GG AG GG AG
AG AG AG GG GG GG GG GG
GG GG GG AA AG AG 54797848
9935 rs1032955 15 AG AA AG AG GG
AG AG AG AG AG AG GG GG
GG GG GG AG AG AA AG AG
AA AA AA AG AG AG AG AG
AA GG AG GG AG GG AG
98231297
9936 rs1032980 1 AC AA AC AA AC
AC AA AC AA AC AA AA AA
AA AA AC AA AA AA AA AC
AC AC AC AC AA CC AC AC
AA CC AA AC AC AC AC
207640345
9937 rs1032984 4 AG GG GG AG GG
AG AG AA AA AA AA AA AG
AG AA AG GG AG AG AG AG
AG GG GG AG AG AG GG AG
AG AA AG AA AG AG AG
40982744
9938 rs1033027 15 AG GG AG GG GG
AG GG GG GG GG GG GG GG
AG GG AG AG AG GG AG GG
GG AG AG GG AA GG AG AG
AG GG GG GG GG GG GG
60616715
9939 rs1033046 16 AA AA AA AG AG
AG AG AG AG AA GG GG GG

GG AA AA AA AA AG AG AG
AG AG AG AA AG AA AG AG
AG AA AA AA AG AG AG
54217720
9940 rs1033054 X GG AG AA AG AA
GG AA AA AA AA AG AG AG
AG AG AG AA AA AA AA AA
GG GG AA AG AG AA AG AA
AA AG AA AA AG AA GG
23629569
9941 rs1033064 7 AG AG AA AG AG
AA GG AG AG AG AA AG AG
AG AG AG AG AG AA AA AA
AG AG GG AG AA AA AA AA
AG AA AA AA AA AA AA
118341243
9942 rs1033070 2 GG GG GG AG AA
AG AG AG AA AA GG AG AG
AA AG GG GG GG AG GG AG
AA AA AA AG AA AG AA AG
GG AG AG AG GG GG GG
214880748
9943 rs1033075 8 GG GG GG AA AG
AG AA GG GG GG AA AG AG
GG GG AG AG AA GG AG GG
AG GG AG GG AG AA AA AG
AG GG AG AG GG GG AG
112795983
9944 rs1033106 1 AA AA AA AA AA
AA AA AA AA AA AA AA AA
AA AA AA AA AA AA AA AG
AA AG AA AA AA AG AG AG
AA AA AA AA AG AG AA
68235077
9945 rs1033113
8 -- -- -- -- -- -- -- --
-- -- -- -- -- -- -- --
-- -- -- -- -- -- -- --
-- -- -- -- -- -- -- --
-- -- -- -- 138891978
9946 rs1033148 5 AG AA AA AA AG
AG AA GG AG GG AG AG AA
AG AG AG AG AA AA AA AA
AG AG AG AG AG AG AG AA
AA AG AG AG AA AA AG
17956340
9947 rs1033173 1 CC CC CC CC CC
AC CC AC AC AC CC CC CC
CC AA CC CC CC AA AC AC
AC AA AC AC CC AA AC AC

AA AC AA AC CC CC CC
61208397
9948 rs1033190 1 AC CC AC CC CC
CC CC AC CC AC AC AC AC
AC AC CC CC CC CC AC CC
AC CC AC CC CC AC AC AC
AC AC CC AC CC CC CC
18528848
9949 rs1033195
3 -- -- -- -- -- -- -- --
-- -- -- -- -- -- -- --
-- -- -- -- -- -- -- --
-- -- -- -- -- -- -- --
-- -- -- -- 19616738
9950 rs1033216 9 CC AC AC CC CC
AC CC AA AA AC CC CC AC
AC AA AC CC CC CC CC AC
AA AC AA AC AA AC AC AC
AC AC CC AC AA AA AC
30647718
9951 rs1033217 2 AG AG GG GG GG
GG AG GG AG AG GG GG GG
AG AG GG AG GG AG GG AG
AG AG AG GG GG AG GG AG
GG GG GG GG AG AG GG
172510158
9952 rs1033221 3 AA AA AA AA AA
AA AA AA AA AA AG AG AA
AA AA AA AA AA AA AA AA
AA AA AA AA AA AA AA AA
AA AA AA AA AA AA AA
130841101
9953 rs103323 3 AA AA AA AA AG AG
AA AG AA AA AA AA AA AA
AA AG AG AG AG AA AG AA
AA AA AA AA AA AA AA AG
AG GG GG AA AG AA 64174577
9954 rs1033242 7 GG GG GG GG GG
GG GG GG GG GG AG AG GG
GG GG GG GG GG AA GG AG
AG AA AA AG AG GG AG AG
GG AG GG GG AG GG AA
109349375
9955 rs1033251 3 GG GG GG AG GG
AG AG AG AG AG AA AA AG
AG AG AG GG AG AG AA AG
AA AA AA AA GG AG GG GG
AG AG AG GG GG AG AG
6081827
9956 rs1033254 2 AG AG GG AA AA

AA AG AG AG AG AA AA AA
AA AA AG AG GG AG AG AA
AG AG AA GG AA AG AA AA
AG GG AG GG AG GG AG
156521982
9957 rs1033272 1 GG AG GG GG AG
GG AG AA AA AA AG AG AA
AA AG GG AG AG AG AG AG
GG AG AG AG GG GG GG GG
AG AG GG GG AG GG AG
207030985
9958 rs1033287 2 AA AA AA AA AA
AA AA AG AA AG AA AG AA
AA AA AA AA AA AA AA AA
AA AG AA AA AG AG AA GG
AA AG AG AG AG GG AA
86649747
9959 rs1033294 2 GG GG GG AG AG
AG AG AG AG AG AG GG GG
AG AG GG GG GG GG GG GG
AG GG AA AA AG AG GG AA
GG GG GG GG GG GG GG
123967985
9960 rs1033330 19 AG AG AG AG AG
AA AG AG AG AG GG AG GG
GG AG AG GG AG AG AG AG
AG AG AG AG AA AG AG AG
AA GG AG GG AG GG AA
36069632
9961 rs1033349 14 AG AG GG GG AG
GG AG AG GG AG GG GG GG
GG GG AG GG AG GG GG AG
AG AG AG AG AG AG AA GG
GG AG GG GG AA AG AG
78727497
9962 rs1033391 6 AG GG AG GG AG
GG AG AA AG AA GG GG GG
AG AG AG AG GG AG AG GG
AA AA AA AA AG AG AG AG
AG AA AG AG GG AG AG
113760063
9963 rs1033392 6 AG AG AA AG AG
GG AA AA AG AA AG AG AG
AG AG AG AA AG GG GG GG
GG GG AG GG AG AG AG AG
AG AA AG AA AG AG AA
114714143
9964 rs1033394 6 AG AG AA AG AA
AG AA AA AA AA AG AG AG
AG AG AG AA AG AG AG GG

	AG	AG	AG	AG	AG	AG	AG	AG
	AG	AA	AA	AA	AA	AA	AA	
114713323								
9965	rs1033398		20	AA	AA	AA	AA	AG
	AG	AA	AG	AG	AA	AA	AA	AA
	AG	AG	AA	AG	AG	GG	AG	GG
	AA	AA	AA	AA	GG	AG	AG	AG
	AG	GG	AA	AG	GG	GG	AG	
8138483								
9966	rs1033411		X	AA	AA	AA	AG	GG
	AA	AG	AA	AA	AA	AA	AA	AA
	AA	AA	AA	AG	AG	AG	AG	AA
	AA	AA	AA	AA	AA	AA	AA	AA
	AG	AA	AA	AA	AA	AG	AA	
43029509								
9967	rs1033420		X	AA	AC	CC	AA	AA
	AA	AA	AC	AC	CC	AC	AC	AC
	AC	AC	AC	AA	AC	AA	AA	AA
	AA	CC	AA	AC	CC	CC	CC	CC
	AA	AC	AA	AA	AC	AC	AA	
136554799								
9968	rs1033433		6	AG	AA	AG	AG	AG
	AA	GG	AG	AG	AG	AA	AA	AG
	AG	GG	AG	AG	AG	AG	GG	AA
	AG	AG	GG	AG	AG	AG	GG	GG
	GG	GG	AG	GG	AG	AG	AG	
137745237								
9969	rs1033437		X	CC	AC	AA	CC	CC
	CC	CC	CC	CC	CC	AA	AC	AC
	AC	AC	CC	CC	CC	AC	AC	CC
	CC	CC	AA	AC	AC	AA	AC	AA
	AC	CC	CC	CC	AA	AC	AA	
112238384								
9970	rs1033464		X	CC	CC	AC	AC	CC
	AA	CC	AC	AC	AA	AC	AC	AA
	AC	AC	AC	AA	AC	AC	AC	AA
	AA	AA	AA	AC	AA	CC	AC	AA
	AC	AC	AA	AA	CC	AC	CC	
45816411								
9971	rs1033470		20	AG	GG	AG	GG	AA
	AG	AG	AA	AG	AA	AG	AG	GG
	AG	AG	AG	AG	AG	GG	GG	AG
	AG	AA	AA	AG	AG	AA	AA	AG
	AA	AA	AA	AA	AA	AA	AA	
9725697								
9972	rs1033480		6	GG	AG	GG	AG	AA
	AA	AG	GG	GG	AG	GG	GG	GG
	GG	GG	AG	AG	GG	GG	GG	GG
	AG	AG	AG	GG	GG	AG	AG	AG
	AG	AA	AG	AA	GG	GG	GG	

38320943								
9973	rs1033485		6	CC	CC	CC	CC	CC
	CC	CC	CC	CC	CC	CC	CC	CC
	CC	CC	CC	AC	AC	AC	AC	AC
	CC	AC	CC	CC	AC	CC	AC	CC
	CC	CC	CC	CC	CC	CC	CC	
1035010								
9974	rs1033520		6	GG	GG	GG	GG	GG
	GG	GG	GG	GG	GG	AG	GG	AG
	GG	AG	GG	GG	GG	GG	GG	GG
	GG	GG	GG	GG	GG	AG	AG	AG
	GG	GG	GG	GG	GG	GG	GG	
69384471								
9975	rs1033521		6	AA	AG	AG	AG	AG
	GG	AG	AA	AA	AA	AA	AA	AA
	AG	AG	AA	AA	AA	AA	AA	AA
	AG	AG	AA	AG	AA	AA	AA	AA
	AA	AG	AA	AA	AG	AA	GG	
69504861								
9976	rs1033530		6	GG	GG	GG	GG	GG
	GG	GG	GG	GG	GG	GG	GG	GG
	GG	GG	GG	GG	GG	GG	GG	GG
	GG	GG	GG	GG	GG	GG	GG	GG
	GG	AG	GG	GG	GG	GG	GG	
46518407								
9977	rs1033541		6	GG	GG	GG	GG	GG
	GG	GG	GG	GG	GG	AG	AG	GG
	GG	GG	GG	GG	GG	GG	GG	GG
	AG	AG	AG	AG	GG	GG	GG	GG
	GG	GG	GG	GG	GG	GG	GG	
52383067								
9978	rs1033567		20	CC	CC	CC	AC	CC
	CC	AC	AA	AC	AA	AC	CC	CC
	CC	CC	CC	AC	AC	CC	AC	CC
	CC	CC	CC	CC	AC	AC	AC	AC
	AC	CC	CC	CC	CC	CC	CC	
59458079								
9979	rs1033581		6	AA	AG	AG	AA	AA
	AA	AA	AA	AA	AA	AA	AA	AG
	AA	AG	AA	AA	AG	AA	AA	AG
	AA	AA	AA	AA	AA	AA	AA	AA
	AA	AA	AG	AA	AA	AA	AA	
95112651								
9980	rs1033592		14	AA	AC	AA	AA	AA
	AA	AA	CC	AC	CC	AA	AA	AA
	AA	AA	AA	AA	AC	AC	AA	AA
	AA	AA	AC	AC	AA	CC	AC	AC
	CC	AC	AA	AC	AC	AC	AC	
37211415								
9981	rs1033634		1	AC	CC	AC	AC	AA

AA AC CC CC CC AC AC AC
AA CC AC AC AC CC CC CC
AC AC AA CC AA AA AA AA
AC CC CC CC AC AC AC
101768868
9982 rs1033651 X CC AC AC AC CC
AA AC AA AA AA AA AA AA
AA AA AA AC AC CC AC CC
CC CC CC AC AA AA AA AA
AA AA AA AA AC AC AA
145650554
9983 rs1033662 20 AA AA AA AA AG
AA AG GG AG AG AA AA AG
AG AA AA AA AG GG GG AG
AG AG AG AG GG GG GG GG
AG AG AG AG AG GG AA
51025381
9984 rs1033684 20 AG AA AA GG AG
GG AG AG AA AA AG AA AG
AG AA AA AG AG AG GG GG
AG AA AA AG AA AG AG AG
AG AG AA AA GG AG GG
8269583
9985 rs1033702 14 AC CC CC AC CC
CC AC CC CC CC CC AC CC
CC AC CC CC CC CC AC CC
CC CC CC CC AC CC AC CC
AC CC AC AC CC CC AC
94647299
9986 rs1033706 14 GG GG AG GG AA
AG AG AA AA AA AA AG AA
AA AA AA AG AG AG AA AG
AG AG AG AG GG AA AG AG
GG AG AG GG AA AG AA
30649776
9987 rs1033729 1 AG GG AG GG GG
GG GG AG AG AG AA AA AG
AG AG AA AA AA AA AG AG
AG AA GG AG GG AA AG AG
AG AG GG GG AG AA GG
57316268
9988 rs1033731 14 AA AA AA AA AA
AA AA AA AA AA AA AA AA
AA AA AA AA AA AA AA AA
AA AA AA AA AA AA AA AA
AA AA AA AA AA AA AA
60829493
9989 rs1033739 14 CC AC AC AC AA
AC AA CC AC CC AA CC AC
AC AA AA AA AA AC CC CC

AC AC AC AC AA AA AA AA
AA AA AC AC CC AC AC
99324205
9990 rs1033762 6 AC AC AA AA AA
AA AC AA AA AA AA AC AA
AA AA AA AA AC AA AA AA
AA AA AA AA AA AC AA AA
AA AA AA AA AA AA AA
16948558
9991 rs1033767 6 GG AG AG GG AG
AG GG GG GG GG GG GG GG
GG GG GG GG GG GG GG GG
AG AG AG AG AG AG AG AG
AG AG GG GG GG GG GG
62491817
9992 rs1033773 10 AA AA AA AA AA
AA AA AC AC AC AA AA AA
AA AC AA AA AA AA AA AC
AA AA AA AA AA AA AA AA
AC AC AA AA AA AA AA
115248259
9993 rs1033777 9 GG GG GG GG GG
GG GG GG GG AG GG GG GG
GG GG GG GG GG GG GG GG
GG GG GG GG GG GG GG GG
GG GG GG GG GG GG GG
77722267
9994 rs1033780 10 AA AA AA AA AA
AA AA AA AA AA AA AA AA
AA AA AA AA AA AA AA AA
AA AA AA AA AA AA AA AA
AG AA AG AA AA AA AA
4300041
9995 rs1033797 20 AA AA AG AA AA
AA AA AA AA AA AA AA AA
AA AA AA AA AA AG AG AG
AG AG AA AG AG AA AA AA
AA AA AG AG AG AG AA
33719687
9996 rs10338 17 AA AA AA AG AA AG
AG AA AA AG AA AA AG AA
AA AG AG AA GG AG GG AA
AA GG AG AG AA AG AA AG
AA AG AA AG GG AG 73773106
9997 rs1033804 6 GG AG AG AG AG
AG AG GG GG GG GG GG AG
GG AG GG GG GG AG AG AG
GG GG GG GG AA AG AG AG
GG GG GG GG GG GG GG
114214825

9998 rs1033806 1 GG GG GG GG GG
GG GG GG AG GG GG GG GG
GG GG GG GG GG AA AG AG
AG GG AG GG GG AG GG GG
AG GG AG AG GG GG GG
168952479
9999 rs1033837 9 GG GG GG GG GG
GG GG GG GG GG GG GG GG
GG GG AG AG AG GG GG GG
GG GG GG GG GG GG GG GG
GG GG GG GG GG GG GG
14274950
10000 rs1033844 14 GG GG GG AG AG
AA GG AA AA AA AA AA AA
AG AA AA AA AG AA AG AA
AG AA AA GG AG AA AG AA
AG AA AG AG GG AG GG
24352231
10001 rs1033845 14 AG AA AG AG AG
GG AA GG GG GG GG GG GG
AG GG GG GG AG GG AG GG
AG GG GG AG GG GG GG GG
AG GG AG AG AA AG AA
24352312
10002 rs1033848 13 AC AC AC AC AC
AC CC AA AA AA AC AA AC
CC AC AC AA AC AC CC AC
AC CC AC CC AC CC CC AC
AC AA CC AC AA AC AA
24714679
10003 rs1033884 6 CC CC CC AC CC
AC CC CC CC CC AC AC AC
AC CC CC CC CC CC CC CC
AC AC AC AC CC CC CC CC
AC CC CC CC CC CC CC
114811379
10004 rs1033908 14 AA AA AA AG AG
AA AG AA AA AA AA AA AA
AA AA AA AG AA AA AG AG
AA AG AG AG AA AG AG AA
GG AG AG GG AA AG AA
61816227
10005 rs1033952 6 GG AG AG AA AG
AG AG AG AA AG GG GG GG
AG AG GG GG AG AA AA AA
GG GG GG GG GG AG GG GG
AG GG GG GG GG AG AG
67257014
10006 rs1033962 10 AA AG AG AG GG
AG GG AA AG GG GG GG GG

GG GG GG AG AG GG GG AG
GG GG GG GG GG GG GG GG
GG GG AG AG AG GG AG
25216806
10007 rs1034044 9 AA AA AA AA AA
AA AA AA AA AA AA AA AA
AA AA AA AA AA AA AA AA
AA AA AA AA AA AA AA AA
AA AA AA AA AA AA AA
87838678
10008 rs1034052 13 GG AG GG AA GG
AG AG AG AG AG GG AG AG
AG AG GG GG GG AA AG AA
AA AA AG AG GG GG GG GG
AG GG GG GG GG AG GG
86448054
10009 rs1034091 1 AG AA AG AA AA
AA AA AA AA AA AA AA AA
AA AA AA AA AA AA AA AA
AG AG AA AG AG AA AA AG
AA AA AA AA AA AA AA
38768561
10010 rs1034099 9 AA AA AA AA AA
AA AA AA AA AA AA AA AA
AA AA AA AA AA AA AA AA
AA AA AA AA AA AA AA AA
AA AA AA AA AA AA AA
100365473
10011 rs1034116 14 AG AG GG GG AG
GG AG GG GG GG GG GG GG
GG GG GG GG GG GG GG GG
AG GG AG GG GG GG GG GG
GG AG AG AG GG GG GG
89955176
10012 rs1034117 21 AA AG AG GG AG
AG GG AA AA AA AA AA AG
AG AG GG AG AG AG AA AG
AG AG AA AG AA AA AA AA
AA AA GG AG AG AG AA
17431897
10013 rs1034137 6 GG GG GG GG AG
AG GG GG GG GG GG AG AG
GG GG GG GG GG AG AG GG
AG AG AG AG GG AG GG GG
AG GG GG GG AG GG AG
66237309
10014 rs1034237 6 GG GG GG GG GG
GG GG GG GG GG GG GG GG
GG GG GG GG GG GG GG GG
AG AG AG AG GG GG GG GG

GG AG GG GG GG AG GG
12395762
10015 rs1034240 6 AA AG AG AA AG
AA AG AG AA AG AA AA AG
AG AG AA AA AA AA AA AA
AA AA AA AA AG AA AA AA
AA AA AA AA AA AA AA
97093037
10016 rs1034269 1 GG GG GG AG AG
AA AG AG AG AG AG AG GG
GG AG AG AA AA GG GG AG
GG AG GG AG AG AA AG AA
AG AG AG GG AA AG AG
179304892
10017 rs1034314 21 AG GG AG GG GG
AG AG AG AG AG AG AG GG
GG GG AA AA AA AG AG AG
GG GG GG GG AA AG AA AA
AA AG GG GG AG AG AA
32670434
10018 rs1034323 6 GG AG AG GG AG
GG AG AA AG AA AG AG AG
AA AG AG AG AG AG AG AG
AA AA AA AG AA GG AG AG
AA AG GG GG AG AG GG
30368635
10019 rs1034353 21 GG AG AG GG GG
GG GG AG AG AG GG GG GG
GG GG AG AA AA AG GG GG
AG AG AG AG AG GG AG GG
GG AG AG GG AG AG AG
27115785
10020 rs1034355 21 AA AA AA GG GG
GG GG GG GG AG AG AA AG
AG AG AA AG AG AA AA AG
AG AG GG GG AG AG AG GG
AA AG AA AA AG AA AG
28649659
10021 rs1034375 11 AA AA AA AA AA
AA AA AA AA AA AA AA AA
AC AA AA AA AA AA AA AA
AA AA AA AA AA AA AA AA
AA AA AC AC AA AA AA
102695472
10022 rs1034394 22 AA AA AA AG AG
AA GG AG AG AG AG AG AG
GG AG AG AA AA AA AA AA
AG AG AG AA AG AA AG AG
GG GG AA AG AG AA AG
34823828

10023	rs1034411		X	GG	GG	GG	GG	GG
	GG	GG	GG	GG	GG	GG	GG	GG
	GG	GG	GG	GG	GG	GG	GG	GG
	GG	GG	GG	GG	GG	GG	GG	GG
	GG	GG	GG	GG	GG	GG	GG	
101326094								
10024	rs1034434		X	GG	GG	GG	GG	GG
	GG	GG	AG	AG	GG	GG	GG	GG
	GG	GG	AG	GG	AG	GG	GG	GG
	GG	GG	GG	GG	GG	GG	GG	GG
	GG	GG	GG	GG	GG	GG	GG	
124504968								
10025	rs1034443		6	AG	AG	AA	AA	AG
	AG	AA	AA	AA	AA	GG	GG	AG
	GG	AG	AA	AA	AA	AA	AA	AA
	AA	AA	AA	AA	AG	AA	AG	AG
	AA	AA	AA	AA	AG	AA	GG	
122203693								
10026	rs1034444		X	GG	GG	GG	GG	GG
	GG	GG	GG	GG	GG	GG	GG	GG
	GG	GG	GG	GG	GG	GG	GG	GG
	GG	GG	AA	AG	GG	GG	GG	GG
	GG	GG	GG	GG	GG	GG	GG	
102164984								
10027	rs1034470		22	AC	AC	CC	AC	AA
	AC	AA	CC	CC	AC	CC	AC	AC
	AA	AC	CC	AC	AC	AC	AC	AC
	AC	AC	AC	AA	AA	CC	AC	AC
	AC	AC	CC	CC	CC	AC	CC	
18093102								
10028	rs1034473		5	CC	CC	CC	CC	CC
	CC	CC	CC	CC	CC	CC	CC	CC
	CC	CC	AC	AC	CC	CC	CC	CC
	AC	AC	CC	AC	CC	CC	CC	CC
	CC	CC	AC	CC	CC	CC	CC	
28725225								
10029	rs1034511		8	AG	AG	AG	AA	GG
	AG	AG	AG	AG	AG	AA	AG	AG
	AG	AA	AG	AA	GG	GG	AG	AG
	AG	AA	AG	AA	AA	AG	AG	AG
	AA	AA	GG	AG	GG	GG	GG	
114073148								
10030	rs1034538		9	AG	AA	AG	AG	AG
	AA	AG	AA	AA	AA	AG	AG	AA
	AA	AA	AA	AA	AA	AA	AA	AA
	AA	AA	AA	AA	AA	AG	AA	AA
	AG	AA	AA	AA	AA	AG	AG	
73442724								
10031	rs1034565		22	AG	AG	AG	GG	GG
	GG	GG	GG	GG	AG	GG	AG	AG

	AG	AG	GG	GG	GG	AG	AG	AG
	GG	GG	GG	GG	AA	GG	AG	AG
	GG	AG	AG	AG	GG	GG	GG	
19984211								
10032	rs1034575		7	GG	GG	GG	GG	GG
	GG	GG	GG	GG	GG	GG	GG	GG
	GG	GG	AG	AG	AG	AA	GG	AA
	GG	GG	GG	GG	GG	GG	GG	GG
	GG	GG	AG	AG	GG	GG	GG	
91861656								
10033	rs1034593		17	AG	GG	AG	AG	AG
	GG	AA	AA	GG	GG	AG	AG	GG
	AG	GG	AA	AG	AA	GG	GG	AG
	GG	GG	GG	GG	AA	AG	AA	AA
	AG	AG	AG	AG	AA	AA	AA	
30861865								
10034	rs1034603		12	GG	GG	AG	AA	AG
	AG	AA	AA	AA	AG	AG	GG	AG
	AG	AA	AA	AA	AA	AG	AA	AA
	AG	AG	GG	AG	AG	AG	AG	GG
	AG	AG	AA	AA	AA	AA	AG	
111649344								
10035	rs1034620		7	AA	AA	AA	AG	AG
	AG	AG	AG	AG	AA	AG	AG	AA
	AA	AA	GG	GG	GG	AA	AA	AA
	AA	AG	AG	AA	AG	AG	AG	AA
	AG	AA	AG	AA	AG	AA	AG	
94063415								
10036	rs1034687		12	AG	AA	AG	GG	GG
	GG	GG	GG	GG	GG	AG	AG	AA
	AA	AG	GG	GG	GG	GG	GG	AG
	AG	AG	GG	AG	GG	AG	AG	GG
	GG	GG	GG	GG	AG	GG	AG	
113170167								
10037	rs1034691		7	AA	AA	AA	AG	AG
	AG	AG	GG	GG	GG	AG	AG	AA
	AG	AG	AG	GG	GG	AG	GG	AG
	AG	AA	AA	AG	AA	AG	AG	AG
	AG	AG	GG	AG	AG	AG	AG	
25202369								
10038	rs1034718		7	AC	AC	AC	CC	CC
	CC	CC	CC	CC	CC	CC	CC	CC
	CC	CC	CC	CC	CC	CC	CC	CC
	CC	CC	CC	CC	CC	CC	CC	CC
	CC	CC	CC	CC	CC	CC	CC	
29436971								
10039	rs1034727		22	AG	AG	GG	GG	GG
	GG	GG	AG	AG	AG	AG	GG	AG
	GG	AG	GG	GG	GG	GG	GG	AG
	GG	GG	GG	GG	GG	AG	AG	GG

AG GG AG GG AA AG AG
19097700
10040 rs1034740 7 AG AG AG AA AG
AG AA AA AG AG AA AA AA
AG AG AA AA AA AA AA AG
AA AA AA AA AA AG AA AA
AG AA AA AA AG AG AG
11600616
10041 rs1034763 17 AG AG GG AG GG
AG GG GG AG AG GG AG AG
AA AG AA AG AG GG GG AG
AA GG AG AG AG GG GG AG
AG AA AG AA GG AG AG
63726734
10042 rs1034826 7 GG AG AG AA AG
AG AA AA AA AA AG GG AG
AG AG AG AA AA AA AA AA
AG AA AA AG AG AG GG GG
AA AA AG AG AA AG AG
108083604
10043 rs1034851 17 AA AA AA AA AA
AA AA AG AA AA AA AA AA
AA AA AA AA AA AG AG AA
AG AG AA AA AG AG AG GG
AA AG AA AG AA AA AA
69463993
10044 rs1034859 22 AC AA AC AC AA
AC AC AA AC AA AA AA AA
AA AA AC -- -- AC AC AA
AA AA AA AA AA AA AA AA
AA AC AA AA AA AA AA
17630486
10045 rs1034865 7 GG GG GG GG GG
GG AG AG AG AG AA AG AG
GG GG GG GG GG GG GG AG
GG AG GG GG GG AG GG GG
GG GG GG GG GG GG GG
148100402
10046 rs1034876 7 AG AA AA AA AA
AA AA AA AA AA AA AA AA
AA AA AA AA AA AA AA AA
AA AA AA AA AA AA AA AA
AA AA AA AA AG AA AG
34607981
10047 rs1034899 17 GG AG AA AA AA
AA AG AG AG AG AG AG AA
GG GG AG AA AA AG AA GG
AG AG AA AA AG AG AA AG
AA AG AG AG AA AG AG
11306660

10048 rs1034937 12 GG GG GG GG GG
GG GG GG GG GG GG GG GG
GG GG AG AG AG AG GG AG
GG GG GG GG GG GG GG GG
GG AG GG AG GG AG GG
2687764
10049 rs1034948 X AA AG GG GG GG
GG AG GG GG GG GG GG GG
AG AG AG GG GG AG AG AA
GG GG GG GG GG GG GG GG
AG GG GG GG GG AG GG
30331349
10050 rs1035009 18 AA AA AA AA AA
AA AA AG AA AG AA AA AA
AA AA AA AA AA AG AA AA
AA AA AG AG AA AA AA AA
AG AG AG GG AA AA AA
53760629
10051 rs1035061 17 AA AA AA AA AA
AA AA AA AG AG AG GG AG
AG AG AG AA AA AA AA AA
AA AA AA AA AA AA AA AA
AG AA AA AA AA AG AA
65647063
10052 rs1035064 19 GG GG GG GG GG
GG GG GG GG GG GG GG GG
GG GG GG GG GG GG GG GG
GG GG GG GG GG GG GG GG
GG GG GG GG GG GG GG
20108539
10053 rs1035066 12 GG AG AG AG GG
AG AG AA AG AG AG AG GG
AG GG AA AG AG AG AG AA
AG AG AG AG GG AG GG GG
AG AG AA AA AA AG AG
5756592
10054 rs1035087 7 GG GG GG GG GG
GG GG GG GG GG GG GG GG
GG GG GG GG GG GG GG GG
GG GG GG GG GG GG GG GG
GG GG GG GG GG GG GG
20083533
10055 rs1035103 2 AA AA AA AA AA
AA AA AA AA AA AA AA AA
AA AA AA AA AA AA AA AA
AA AA AA AA AA AA AA AA
AA AA AA AA AA AA AA
59726568
10056 rs1035142 2 CC CC CC AA AC
AC AA AC CC CC AC AC AC

AC AC CC CC CC AC AA CC
AC AC CC CC AA AC AC AA
AC AC AC CC AC AC CC
202153078
10057 rs1035147 7 AA AC AA AC AA
AC AC AC AC AA AC AA AC
CC AC AA AA AA AA AA AA
AA AA AA AA AA AA AA AA
CC AC AA AC AC AA AC
12094966
10058 rs1035165 2 GG GG GG GG AG
AG GG GG GG GG GG GG GG
AG AG AG AG AG AG AG GG
AG GG AG GG GG AG AG GG
AG GG AG GG GG GG GG
65159596
10059 rs1035169 7 AG AG GG AG AG
AG AG AG AG AG GG AG AG
AG AG GG GG GG AA GG AG
GG AG AG GG GG AG GG GG
AG GG AG GG AA AA AG
103843369
10060 rs1035209 10 GG GG GG GG GG
GG GG GG GG AG GG GG GG
GG GG GG GG GG GG GG GG
GG GG GG GG GG AG GG GG
AG GG GG GG GG GG GG
101345366
10061 rs1035221 7 AA AG AA AG AA
AA AG AG AG AG AA AA AA
AA AA AA AA AG AA AA AA
AA AA AA AA AA AG AA AA
AA AA AA AA AA AA AG
35536505
10062 rs1035236 12 GG AG GG GG GG
GG GG GG GG GG AG AG AG
AG AG AG GG AG GG GG GG
GG AG AG GG AG GG AG AG
GG GG AG AG AG AG AG
107310797
10063 rs1035316 2 AA AG AA AG AG
GG AA AA AG AG AA AG AG
AG AG AA AG AA AA AG AG
AA AA AA AA AA AG AG AA
AG GG AA AG AG AG AA
225140153
10064 rs1035339 11 GG GG GG GG GG
GG GG GG AG AG GG GG GG
GG GG GG GG AG AG AG AG
GG AG GG AG AG GG AG AG

	AG	GG	AG	GG	AG	AA	AG	
99866660								
10065	rs1035363		19	AG	AG	GG	AG	GG
	GG	AG	AG	AA	AA	GG	AA	AG
	AA	AA	GG	GG	GG	AG	AG	AG
	AG	GG	--	GG	AG	AG	AG	GG
	GG	AG	AG	GG	AG	GG	AG	
18371776								
10066	rs1035364		19	AG	AA	AG	GG	GG
	GG	GG	AG	AG	AG	GG	GG	GG
	AG	AG	AG	AG	GG	GG	AG	GG
	GG	GG	GG	GG	AG	AG	AG	AA
	GG	GG	AG	GG	GG	GG	GG	
18428880								
10067	rs1035373		5	GG	GG	GG	GG	GG
	GG	GG	GG	GG	GG	GG	GG	GG
	GG	GG	GG	GG	GG	GG	GG	GG
	GG	GG	GG	GG	AG	GG	AG	AG
	AG	GG	AG	AG	GG	GG	GG	
167296428								
10068	rs1035379		8	CC	AC	AC	AC	CC
	AC	AC	AA	AC	AC	CC	CC	CC
	AC	AC	AC	CC	CC	AC	AC	AC
	AC	AA	AC	AA	AA	AA	AA	AA
	CC	CC	CC	CC	AA	AA	AA	
140619534								
10069	rs1035395		19	AG	GG	AG	GG	GG
	GG	GG	GG	GG	GG	GG	AG	AG
	GG	GG	GG	AG	AG	GG	AG	AG
	GG	AG	AG	AG	AG	GG	AG	AG
	GG	AG	GG	AG	GG	GG	GG	
14553995								
10070	rs1035411		5	AG	AG	AA	GG	GG
	AG	GG	AG	AG	AG	AA	AA	AG
	GG	GG	AG	GG	AA	AG	AG	AG
	AA	AA	AG	AG	AG	AA	AA	AG
	AG	AA	GG	AG	GG	GG	AG	
179729519								
10071	rs1035421		5	AA	AG	AA	AA	AG
	AA	AG	AG	AG	AG	AG	AG	AG
	AA	AA	AA	AG	AG	AA	AA	AA
	AA	AA	AA	AA	AG	AG	GG	GG
	AA	AA	AG	AA	AG	AA	AG	
167768114								
10072	rs1035424		5	AA	AA	AA	AA	AA
	AA	AA	AA	AA	AA	AG	AG	AG
	AG	AA	AA	AA	AA	AA	AA	AA
	AG	AA	AG	AG	AA	AA	AA	AA
	AA	AG	AA	AA	AG	AA	AG	
169583173								

10073 rs1035428 5 AG AG GG GG GG
GG GG GG GG GG GG GG GG
GG GG GG GG GG GG GG GG
GG GG GG GG GG GG GG GG
GG GG GG GG GG GG GG
169600855
10074 rs1035441 19 AG GG GG AG GG
AG GG AG GG GG GG AA AG
AA AA GG AG AG GG AA GG
AG GG GG AG AG AG AG AA
GG GG GG GG AG AG GG
37353791
10075 rs1035450 19 GG GG GG GG GG
GG AG GG GG GG GG GG GG
GG GG AG GG GG GG GG GG
AA AG AG AA AG GG AG GG
AG AG GG AG AG GG AA
54962374
10076 rs1035458 19 AA AA AG GG AA
AG AG AG AG AG AG AG GG
AG AG GG GG GG AG AG GG
AA AA AG GG AG AG AG AA
AG AG AA AA AA AA AG
12516150
10077 rs1035465 19 GG GG GG GG GG
GG GG GG GG GG GG GG GG
GG GG GG GG GG AG GG GG
AG AG GG GG GG GG GG GG
GG AG GG GG AG GG AG
7691465
10078 rs1035496 5 AG AG AG AG AA
AA AG AA AA AA AA AA AA
AA AA AG AG AG AA AA AG
AG AA AA AG AG AA AG AA
AA AA GG AG AA AA AA
41138249
10079 rs1035500 5 GG AG GG AG AG
AG AA AG GG AG GG AG GG
GG AG GG GG -- GG GG GG
AG AA AA AG GG AG GG AG
AG AG GG GG GG GG GG
37918856
10080 rs1035516 5 AA AA AA AG AA
AA AG GG AG GG AG AA AA
AA AA AA AA AA GG AG AG
AG GG GG AG AA AA AA AA
AA AG AA AG AG AG AA
42823799
10081 rs1035550 16 GG GG GG GG GG
GG GG GG GG GG GG GG GG

	GG	GG	GG	GG	GG	GG	GG	GG
	GG	GG	GG	GG	AG	GG	GG	AG
	AG	AG	GG	AG	AG	AG	GG	
86602707								
10082	rs1035564		16	AA	AG	AA	AG	AG
	AA	GG	AG	AG	AG	AA	AG	AG
	AA	AA	AA	AA	AG	GG	AG	AG
	GG	AA	AA	GG	AA	AA	AA	AA
	AG	AG	AA	AG	AA	AG	AG	
6110547								
10083	rs1035569		16	AA	AA	AA	AA	AG
	AA	AG	AG	AG	AA	AA	AA	AA
	AG	AA	AG	AA	AA	AA	AA	AA
	AA	AA	AA	AA	AG	GG	AG	GG
	AA	AG	AA	AA	AA	AA	AG	
83348824								
10084	rs1035595		18	AG	AA	AA	AG	AG
	AG	AG	AG	AG	AG	AG	AG	AA
	AA	AG	AA	AG	AG	AA	AG	AG
	AA	AA	AG	AA	GG	AG	GG	GG
	AA	AA	AG	AA	AG	GG	AA	
53695861								
10085	rs1035666		8	AC	AC	AC	CC	AC
	AC	AC	AC	AC	AC	AC	AA	AC
	AC	AA	AA	AA	AA	AC	AC	AC
	AA	AC	AC	AC	AA	AA	AA	AA
	AA	AA	AC	AA	CC	AC	AC	
72517046								
10086	rs1035671		2	GG	GG	GG	GG	GG
	GG	GG	AG	GG	GG	GG	AG	GG
	GG	GG	GG	GG	--	GG	GG	GG
	AG	AG	AG	AG	GG	GG	GG	GG
	GG	GG	GG	GG	GG	GG	GG	
218674484								
10087	rs1035703		15	AG	AA	AG	AG	AG
	AG	AA	AG	AG	AG	AA	AG	AA
	AA	AA	AG	AG	AA	GG	AA	AG
	AG	AG	GG	AG	AG	AA	AG	AG
	AG	AA	AA	AA	AG	AG	AA	
47828485								
10088	rs1035744		15	AG	GG	AG	GG	GG
	AG	GG	AA	AG	AG	AG	AA	AG
	AG	AG	AG	AG	AG	AA	AG	AG
	AG	AG	AG	AA	AG	AA	AA	AG
	AG	AA	AA	AA	AA	AG	AA	
72566615								
10089	rs1035796		10	GG	AG	AA	AA	AG
	AG	AG	AG	AG	AA	AG	AA	AG
	AG	AA	GG	GG	GG	AG	AG	AG
	GG	GG	AA	AG	AG	GG	GG	AG

	AG	AG	GG	AG	AG	AG	GG
90051697							
10090	rs1035799		18	AA	AA	AA	AA	AA
	AA	AA	AA	AA	AA	AA	AA	AA
	AA	AA	AA	AA	AA	AA	AA	AA
	AA	AA	AA	AA	AA	AA	AA	AA
	AA	AA	AA	AA	AA	AA	AA	
30047388								
10091	rs1035838		2	AA	AA	AA	AA	AA
	AA	AA	AA	AA	AA	AA	AA	AA
	AA	AA	AA	AA	AA	AA	AA	AA
	AA	AA	AA	AA	AA	AA	AA	AA
	AA	AA	AA	AA	AA	AA	AA	
145423804								
10092	rs1035839		2	AA	AA	AA	AA	AA
	AA	AA	AA	AA	AA	AA	AA	AA
	AA	AA	AA	AA	AA	AA	AG	AA
	AA	AA	AA	AA	AA	AA	AA	AA
	AA	AA	AG	AG	AA	AA	AA	
171809485								
10093	rs1035872		19	AA	AG	AG	AG	GG
	GG	AG	AG	GG	GG	GG	AG	AG
	GG	GG	AG	AA	AG	AG	AA	AG
	AA	AA	AA	AA	AG	AA	AG	AA
	AG	AA	AG	AG	AA	AA	AA	
17546855								
10094	rs1035924		5	AG	GG	GG	GG	GG
	GG	GG	GG	GG	GG	AA	AG	AG
	AG	AG	GG	GG	GG	AG	AA	AG
	AG	AG	AG	AG	GG	GG	GG	GG
	GG	GG	GG	GG	GG	GG	AG	
21735023								
10095	rs1035936		19	AA	AG	AA	AA	AA
	AA	AG	GG	GG	GG	AA	AG	AG
	AA	AA	AA	AA	AA	AG	AG	GG
	AA	AG	AA	AA	AA	AA	AA	AA
	GG	AA	AA	AA	AA	AA	AA	
30115289								
10096	rs1035941		19	GG	AG	AG	AG	GG
	GG	AG	AG	AG	AG	AG	AG	AG
	GG	GG	AG	GG	GG	GG	GG	AG
	GG	GG	GG	GG	GG	GG	GG	GG
	AG	AA	AG	AA	AG	AA	GG	
7199939								
10097	rs1035946		16	AA	AA	AA	AA	AA
	AA	AA	AA	AA	AA	AG	AG	AG
	AG	AA	AA	AA	AA	AG	AG	AG
	AG	AG	GG	AG	AA	AA	AA	AA
	AA	AA	AA	AA	AG	AA	AG	
24974426								

10098	rs1035979		2	AA	AA	AA	AA	AG
	AG	AA	AG	AA	AG	AA	AG	AA
	AA	AA	AG	AG	AA	AA	AA	AA
	AA	AA	AA	AA	AA	AA	AA	AA
	AG	AG	AG	AA	AG	GG	AA	
83614035								
10099	rs1035999		5	AG	AG	AA	GG	AG
	GG	AG	GG	GG	GG	AG	AA	AG
	AA	AA	AG	GG	GG	AG	AA	AA
	GG	GG	GG	GG	AG	AG	AA	GG
	AA	AG	AG	AG	AG	AA	AG	
117895058								
10100	rs1036067		1	AA	AA	AA	AA	AA
	AA	AA	AC	AC	AC	AA	AA	AA
	AA	AA	AA	AA	AA	AA	AA	AA
	AA	AA	AA	AA	AC	AA	AA	AC
	AA	AC	AC	AC	AA	AA	AA	
68660092								
10101	rs1036150		2	AA	AA	AG	AA	AG
	AG	AA	AG	AG	AA	AA	AA	AA
	AA	AA	AA	AA	AG	AA	AA	AA
	AA	AA	AA	AA	AA	AG	AA	AG
	GG	AA	AA	AA	GG	AG	AG	
43948153								
10102	rs1036165		2	GG	AG	AA	GG	AG
	AG	GG	GG	GG	GG	GG	GG	GG
	AG	AG	GG	GG	AG	GG	GG	AG
	AG	AG	AG	AG	AG	AG	GG	AG
	AG	AG	GG	AG	AG	AG	AG	
56341068								
10103	rs1036196		5	GG	GG	GG	GG	AG
	GG	AG	GG	GG	GG	GG	GG	GG
	GG	GG	GG	GG	GG	GG	GG	GG
	GG	GG	GG	GG	GG	GG	GG	GG
	AG	GG	GG	GG	GG	GG	GG	
151012257								
10104	rs1036231		19	GG	AG	AG	AA	AA
	AA	AA	AG	AG	AA	AA	AA	AA
	AA	AA	AA	AG	AA	AG	AA	AA
	AA	AA	AA	AA	AA	AA	AA	AA
	AG	AA	AA	AA	AA	AA	AA	
55497843								
10105	rs1036235		19	AG	AG	GG	GG	GG
	GG	AG	AG	GG	GG	AA	GG	AG
	GG	GG	AG	GG	AG	GG	GG	GG
	GG	GG	GG	GG	GG	AG	AG	AG
	AA	GG	GG	GG	GG	GG	AG	
22279541								
10106	rs1036242		19	GG	GG	AG	GG	GG
	GG	GG	GG	AG	AG	GG	GG	GG

	GG	AG	GG	GG	GG	GG	GG	AG
	GG	GG	GG	GG	AG	GG	AG	GG
	GG	AG	AG	GG	GG	AG	AG	
56750675								
10107	rs1036265		8	AA	AA	AA	AG	AG
	AG	AG	AA	AG	AG	AG	AA	AG
	AA	AA	AA	AG	AA	AG	AG	AG
	GG	GG	GG	GG	AA	AG	AA	AA
	AG	AA	AG	AG	GG	GG	GG	
122149252								
10108	rs1036304		15	AG	AA	AG	AG	AG
	AA	AG	AG	AA	AA	AA	AA	AG
	AG	AA	AG	AG	AG	AA	AA	AA
	AG	AA	AA	AA	AA	AA	AA	AA
	AA	AA	AG	AA	AG	GG	AA	
94908380								
10109	rs1036308		3	AA	AA	AA	AG	AG
	AA	AG	AA	AA	AA	AA	AA	AA
	GG	AG	AA	AA	AA	AA	AA	AA
	AA	AG	AA	AA	AA	AA	AA	AA
	AA	AA	AA	AA	AG	AA	GG	
30235221								
10110	rs1036342		4	AA	AG	AG	AA	AA
	AG	AA	AG	AG	AG	AG	GG	GG
	GG	AG	AA	AA	AA	AA	AG	AG
	AA	AA	AA	AA	AA	AG	AG	AA
	AA	AG	AA	AG	AG	AA	AG	
158492974								
10111	rs1036429		12	GG	GG	GG	GG	GG
	AG	GG	AG	AG	AG	GG	GG	GG
	GG	GG	GG	GG	GG	GG	GG	AG
	GG	GG	GG	GG	AG	GG	GG	GG
	AA	AG	GG	AG	GG	AG	GG	
96271428								
10112	rs1036460		15	AA	AA	AA	AA	AA
	AG	AG	AA	AA	AA	AG	AG	GG
	AG	GG	AA	AA	AA	AA	AA	AG
	AA	AA	AA	AA	AA	AA	AA	AA
	AA	AG	AA	AG	AA	AA	AG	
35003398								
10113	rs1036477		15	AG	AA	AG	AA	AA
	AA	AA	AA	AA	AA	AA	AA	AA
	AA	AA	AG	AA	AG	AA	AG	AA
	AA	AA	AA	AA	AG	AG	AG	AG
	AA	AA	AA	AA	AG	AA	AG	
48914926								
10114	rs1036486		16	GG	GG	GG	GG	GG
	GG	GG	GG	GG	GG	GG	GG	GG
	GG	GG	GG	GG	GG	GG	GG	GG
	GG	GG	GG	GG	GG	GG	GG	GG

	AG	GG	GG	GG	GG	GG	GG	
64386493								
10115	rs1036495		17	AA	AA	AC	AC	AA
	AC	AC	AC	AC	AC	AC	CC	CC
	AC	CC	AA	AA	AC	AA	AC	AC
	AA	AC	CC	AC	AA	AC	AC	AC
	CC	AA	AC	AC	AA	AA	AA	
32803877								
10116	rs1036504		7	AA	AA	AA	AG	AG
	AG	AG	AG	AA	AA	GG	AG	AG
	GG	AG	AG	AG	AG	GG	GG	AG
	AG	AA	AG	AA	AG	AA	AA	AA
	AA	AG	GG	GG	AG	AG	AG	
2609765								
10117	rs1036515		12	AA	AA	AG	AA	AA
	AA	AA	GG	GG	GG	AA	AG	AG
	AG	AA	AA	AG	AG	GG	AA	AG
	GG	AG	AG	GG	AA	AA	AA	AA
	GG	AA	AA	AA	AA	AA	AA	
109745620								
10118	rs1036517		15	GG	AG	AG	AA	AA
	AA	AG	AG	AA	AA	AG	AA	AG
	AG	AG	AG	AG	AG	AA	AA	AG
	AA	AA	AA	AA	GG	AA	AG	AG
	AG	AA	AA	AA	GG	GG	GG	
96646200								
10119	rs1036519		15	AA	AG	AG	AG	GG
	AG	AG	AA	AG	AG	AG	GG	AG
	AG	AG	AA	AA	AA	AG	AA	AG
	GG	AG	AG	AG	AA	AG	AG	AA
	AA	AG	AA	AG	AA	AA	AA	
96645410								
10120	rs1036556		8	AA	AG	AA	AA	AG
	AG	AA	AG	AG	AG	GG	GG	GG
	GG	GG	AG	AG	GG	AG	AG	AA
	AG	AG	AG	AG	AG	AA	AA	AA
	AG	AA	AG	AG	GG	GG	GG	
4217304								
10121	rs1036598		9	AG	AA	AG	GG	AG
	GG	AG	GG	GG	GG	AG	GG	AG
	AG	GG	GG	GG	GG	GG	GG	GG
	GG	AG	GG	AG	GG	GG	GG	GG
	GG	GG	GG	GG	GG	GG	GG	
81628944								
10122	rs1036668		2	AA	AA	AA	AA	AA
	AA	AA	AA	AA	AA	AA	AA	AA
	AA	AA	AA	AA	AA	AA	AC	AA
	AA	AA	AA	AA	CC	CC	CC	CC
	AA	AC	AA	AA	AA	AA	AA	
12132638								

10123 rs1036673 15 AA AA AA AA AA
AG AA AA AG AG AG AG AG
AA AG GG AG AG AA AA AA
AA AA AA AA AA AG AA AG
AG AA AG AG GG GG AG
74338462
10124 rs1036689 8 AA AG AA AG AG
AA GG GG GG AG AG AG AG
AG AG GG AG AG AA AA AA
GG GG GG GG AG AG GG AG
AA AG AG AG GG GG AG
2112130
10125 rs1036699 9 AG AG AG AG AG
GG AA AA AG AG AG AG AA
AA AG GG GG GG GG GG GG
AG AG GG AG GG GG GG GG
AG GG GG GG GG GG GG
92765694
10126 rs1036731 10 AA AA AA AA AC
AC AC AC AA AA AA AA AA
AA AA AA AA AA AA AA AA
AA AA AC AA AC AA AC AA
AC AA AC AC AA AC AA
34467741
10127 rs1036741 6 AA AA AA AA AG
AG AA AA AA AA GG AA AG
AA AA AG AA AA AG AA AG
AA AA AG AA AG AA AG AG
AG AG AG AG AA AA AA
159016076
10128 rs1036745 13 AA AA AA AA AA
AA AA AA AA AA AA AA AA
AA AA AA AA AA AA AA AA
AA AA AA AA AA AA AA AA
AA AA AA AA AA AA AA
79601701
10129 rs1036747 10 AA AA AG AA AA
AA AA AA AG AA AA AA AG
AA AG AA AA AA AA AA AA
AA AA AG AA AG AA AG AG
AA AA AA AA AA AA AA
3506070
10130 rs1036767 4 GG GG GG GG GG
GG GG GG GG GG GG GG GG
GG GG GG GG GG AG AG GG
AG GG GG GG AG AG AG AA
AG GG AA AG GG GG GG
180068417
10131 rs1036796 3 AA AG AG GG GG
GG AG AG AA AA AA AA AA

	GG	AG	GG	AG	AG	AA	AG	AG
	GG	AG	GG	AG	AG	GG	AG	GG
	AG	AA	AA	AA	AG	AA	AG	
64886335								
10132	rs1036797		3	GG	AG	AG	AG	AG
	AA	GG	AG	GG	GG	GG	GG	GG
	AA	AG	AG	GG	GG	GG	AG	AG
	AA	AG	AA	AG	AG	GG	GG	AG
	GG	GG	GG	GG	GG	GG	GG	
64886110								
10133	rs1036806		15	AA	AA	AA	AA	AA
	AA	AA	AA	AA	AA	AA	AA	AA
	AG	AA	AG	AA	AA	AA	AA	AA
	AA	AA	AA	AA	AG	AA	AG	AA
	AA	AG	AA	AG	AG	AG	AG	
88844814								
10134	rs1036811		15	AG	AG	AA	AA	AA
	AA	AA	AA	AA	AA	AG	AA	AA
	AA	AA	AA	AA	AA	AA	AA	AA
	AA	AG	AG	AA	AA	AA	AA	AA
	AA	AG	AA	AG	AA	AA	AA	
87987940								
10135	rs1036915		9	AA	AG	AA	AG	AG
	AA	GG	GG	AA	GG	AA	AG	AA
	AA	AA	AG	AG	GG	GG	GG	GG
	AG	AA	AG	AA	GG	AA	AG	AG
	AA	AA	AA	AA	GG	GG	GG	
87437849								
10136	rs1036920		3	AG	GG	AG	GG	AG
	AG	GG	AA	AG	GG	GG	GG	GG
	GG	GG	GG	GG	GG	GG	GG	GG
	GG	GG	GG	GG	AG	AG	AG	AG
	GG	GG	GG	GG	GG	GG	GG	
64543563								
10137	rs1036955		9	AG	AA	AG	AG	GG
	AG	GG	AG	GG	AG	AG	GG	AG
	AG	GG	AG	GG	GG	AG	AG	GG
	AA	AA	AA	AA	AG	GG	AG	GG
	AG	AA	AA	AA	GG	GG	AG	
80057559								
10138	rs1036959		1	AG	AG	AG	GG	AG
	AG	GG	GG	GG	GG	GG	GG	GG
	AG	AG	GG	GG	GG	GG	GG	GG
	GG	AG	AG	AG	GG	GG	GG	GG
	GG	AG	GG	AG	GG	GG	AG	
112451134								
10139	rs1037010		9	CC	CC	CC	CC	CC
	CC	CC	CC	CC	CC	AC	AC	AA
	AA	AC	AC	AC	CC	AC	AA	AC
	AC	AA	AA	AA	CC	CC	CC	CC

	CC	CC	CC	CC	CC	AC	AC
89696756							
10140	rs1037072	13	AA	AA	AA	AG	AG
	AG	AA	AA	AA	AA	AA	AG	AG
	AG	AG	AA	AA	AA	AA	AA	AA
	AA	AA	AA	AA	AA	AA	AA	AA
	AA	AG	AG	AG	AG	AG	AA	
94240082								
10141	rs1037079	4	GG	AG	AG	GG	GG
	GG	GG	GG	GG	GG	GG	GG	GG
	GG	GG	GG	GG	GG	GG	GG	GG
	GG	GG	GG	GG	GG	GG	GG	GG
	GG	GG	GG	GG	GG	GG	GG	
32590048								
10142	rs1037089	11	GG	GG	GG	GG	GG
	GG	GG	GG	GG	GG	GG	GG	GG
	GG	GG	GG	GG	GG	GG	GG	GG
	GG	GG	GG	GG	GG	GG	GG	GG
	GG	GG	GG	GG	GG	GG	GG	
105550003								
10143	rs1037094	2	AC	AC	CC	CC	CC
	CC	CC	CC	CC	CC	AC	AC	CC
	CC	CC	AC	CC	CC	CC	CC	CC
	CC	CC	CC	CC	CC	CC	CC	CC
	CC	CC	AC	CC	AC	CC	AC	
155656548								
10144	rs1037100	5	AG	GG	AG	AG	AG
	GG	AA	GG	AG	GG	GG	GG	AG
	AG	AG	AG	GG	AG	AA	AG	AA
	AG	AG	AA	AA	GG	GG	GG	GG
	GG	GG	AG	GG	AG	GG	AG	
142938663								
10145	rs1037124	15	GG	GG	GG	GG	GG
	GG	GG	GG	AG	AG	AG	AA	AA
	GG	AA	GG	AG	AG	GG	GG	GG
	GG	AG	AG	AG	GG	GG	GG	GG
	GG	AG	GG	AG	GG	GG	GG	
80731815								
10146	rs1037154	12	GG	GG	GG	GG	GG
	GG	GG	AG	GG	AG	GG	AG	AG
	AG	GG	GG	AG	GG	AG	AA	GG
	AG	GG	AG	AG	GG	GG	GG	GG
	AG	AG	AG	GG	GG	AG	AG	
127688001								
10147	rs1037170	17	AA	AA	AA	AA	AG
	AA	AG	AA	AA	AA	AG	AG	AG
	AG	AG	AA	AA	AA	AA	AA	AA
	AA	AA	AA	AA	AA	AG	AA	AA
	AA	AA	AA	AA	AA	AA	AG	
72702914								

10148 rs1037175 2 AA AA AA AA AA
AA AA AA AA AA AA AG AA
GG GG AA AA AA AA AG AA
GG AG AG AG AG AA AG AA
AA AG AG AG AG AA AG
133629564
10149 rs1037193 X GG AG AA AG GG
GG AG AG AG AA AA AA AG
AA AA AG AA AA AA AA AA
GG AA GG AA AG AA AG AA
GG AG GG AA GG AG GG
27132374
10150 rs1037257 17 AG AG AG AG GG
GG AG GG GG GG GG GG AG
GG GG AG GG AG AA AG AG
AG AG GG GG GG AG AG AG
AG GG AG AG AG AG AG
71151353
10151 rs1037260 17 AA AA AA AA AA
AA AA AA AA AA AA AA AA
AA AA AA AA AA AC AA AC
AA AA AA AA AA AA AA AA
AA AA AA AA AA AA AA
71157265
10152 rs1037266 2 AG AG AG AG AG
AG AG GG AG AG GG GG GG
AG AG AG AA AA AA GG AG
AG AG AG AG GG GG GG GG
AG AG GG AG AG AG AG
142784277
10153 rs1037272 13 GG AG GG AG AG
AG GG GG GG GG GG GG GG
GG GG GG GG GG AG AG AG
GG GG GG GG GG AG AG GG
GG GG AG AG GG GG GG
42790458
10154 rs1037303 X AA AG GG GG GG
GG GG GG GG GG AG AG AG
AG AG AG GG AG AG AG AA
GG GG GG GG GG GG GG GG
GG GG AA GG AG GG AA
6715164
10155 rs1037306 8 AA AA AG AA AA
AA AA AA AA AA AA AA AA
AA AA AA AA AG AA AA AA
AG AG AG AG AG AA AA AG
AA AG AA AG AG AG AA
106561277
10156 rs1037349 18 AG GG GG AG AG
GG AA AG AG AG AG AG AA

	AA	GG	GG	AG	AG	GG	AG	AG
	GG	GG	AG	AG	AA	AA	AA	AA
	GG	GG	GG	GG	GG	AG	GG	
22452090								
10157	rs1037351		7	AG	AA	AA	AA	AA
	AA	AA	AG	AG	AA	AA	AA	AA
	AG	AG	AA	AG	AG	AG	AG	AA
	AA	AA	AA	AA	AA	AA	AA	AA
	AA	GG	AA	AG	AG	AG	AG	
50565404								
10158	rs1037363		11	AA	AA	AA	AA	AA
	AA	AA	AA	AA	AA	AA	AG	AG
	AG	AG	AG	AG	AA	AA	AA	AA
	AA	AA	AA	AA	AA	AA	AA	AA
	AA	AA	AG	AA	AA	AA	AA	
45429381								
10159	rs1037381		2	GG	GG	GG	AA	AA
	AA	AA	GG	GG	GG	AG	AG	AG
	AG	AG	AG	AA	AG	AG	AA	AA
	AA	AA	AG	AG	GG	AG	AG	GG
	AA	GG	AG	AG	AG	AG	AA	
106303243								
10160	rs1037386		3	AG	AG	AG	AG	AG
	GG	AG	AA	AG	AA	AA	AG	AA
	AA	AA	AG	AA	AG	AA	AA	AA
	AG	AG	AG	AG	AG	GG	GG	AG
	AA	AG	AG	GG	AG	AG	AG	
1478453								
10161	rs1037390		1	AA	AC	AA	CC	CC
	CC	CC	AC	AC	AC	AA	AA	AC
	CC	AA	CC	AC	AC	CC	CC	CC
	AC	AC	AC	CC	AC	AA	AC	AA
	CC	AC	CC	AC	AC	CC	AA	
196010327								
10162	rs1037392		18	AA	AA	AA	AA	AC
	AA	AC	AC	AA	AA	AC	AA	AA
	AA	AA	AA	AA	AA	AC	AA	CC
	AC	AC	AA	AA	AA	AA	AA	AA
	AC	AC	AA	AC	AA	AA	AA	
52338064								
10163	rs1037399		11	GG	AG	AA	AG	GG
	GG	AG	AA	AA	AA	AA	AA	AG
	AA	AG	AG	GG	AG	AA	AA	AG
	AA	AA	AA	AA	AG	GG	AG	AG
	GG	AG	AG	GG	GG	AG	GG	
80147284								
10164	rs1037401		12	GG	GG	GG	GG	GG
	GG	GG	GG	GG	GG	GG	AG	GG
	AG	AG	GG	GG	GG	AG	GG	AG
	GG	GG	GG	GG	GG	AG	GG	GG

```
        GG      GG      GG      GG      GG      GG      GG
42866709
10165   rs1037405       4       AG      AA      AG      AG      GG
        AG      GG      GG      GG      GG      AG      GG      GG
        GG      GG      AA      AG      AG      GG      GG      GG
        GG      GG      GG      GG      GG      GG      GG      GG
        AG      GG      AG      GG      GG      GG      GG
82347502
10166   rs1037416       2       AA      AA      AA      AA      AG
        AG      AA      GG      AG      GG      GG      AG      GG
        GG      GG      AA      AA      AA      AG      AA      AA
        GG      GG      GG      GG      AG      GG      GG      GG
        AG      AA      AA      AA      AA      AA      AG
208890373
10167   rs1037435       12      AA      AG      AA      AG      GG
        AG      GG      AA      AA      AG      AA      AG      AA
        AG      AG      GG      GG      GG      AG      AG      AG
        AG      AG      AA      AG      AA      AA      AA      AA
        AG      AG      GG      GG      AA      AG      AG
44941956
10168   rs1037439       15      AA      AG      AG      AG      AG
        AG      GG      AA      AA      AA      GG      AA      AG
        AA      AG      AG      AA      AG      AA      AG      AA
        AA      AG      AG      AG      AA      AG      AG      AG
        AA      AG      GG      AG      AG      AG      AA
37026565
10169   rs1037476       4       AA      AA      AA      AG      AA
        AG      AG      AG      AG      AA      GG      GG      AG
        AA      AA      AG      AA      AG      AG      AG      AA
        AG      AG      AG      AG      AA      AG      AG      AA
        AG      AA      AA      AA      AA      AA      AA
99952607
10170   rs1037519       1       GG      AG      GG      AA      AA
        AA      AA      AA      AA      AA      AG      AG      AG
        AA      GG      GG      AG      AG      AG      AG      AG
        AG      AG      GG      AA      GG      AA      AG      AG
        AA      AG      AG      GG      AG      GG      AA
74267230
10171   rs1037524       X       AA      AG      AG      AG      AA
        GG      AA      AA      AA      AA      AG      AG      AG
        AG      AA      AG      AA      AG      AA      AA      AA
        AA      AA      AA      AA      AA      GG      AG      AA
        AA      GG      GG      GG      AA      AG      AA
8034993
10172   rs1037569       5       GG      GG      GG      AG      AG
        AG      GG      GG      GG      GG      GG      GG      GG
        GG      GG      AG      AA      AA      AG      GG      GG
        AG      AG      AG      AG      GG      GG      GG      GG
        AG      GG      AG      GG      GG      GG      AG
119926305
```

10173 rs1037644 4 GG GG GG AA AA
AA AA AG GG GG GG GG GG
AG GG GG GG GG AG AG AG
AG AG GG GG AG AA AA AA
GG AG GG AG AG AG AG
190290460
10174 rs1037661 9 GG GG GG GG GG
GG GG AG AG GG GG GG GG
GG GG GG GG GG AG AG GG
GG AG GG AG GG GG GG GG
GG GG GG GG GG GG GG
106041092
10175 rs1037666 1 AG AG AG AG AA
AG AG AA AA AA AG AG AG
AA AA AG AA AG AA AG AG
AG AA AA AA AG AG GG AG
AA AG GG AG AG AG AG
240358485
10176 rs1037676 7 AA AA AA AA AC
AA AC AA AC AA CC AC AC
AA AA AA AA AA AA AA AA
AC AC AA AA AA AC AA AA
AA AA AA AA AA AA AA
50243488
10177 rs1037680 15 AG GG AG GG GG
AG GG AA AA AA AG AA AG
AG AG AA AA AA AA AG AG
AA AA AA AA AG AA AA AG
AG AA AA AA AA AA AA
72514319
10178 rs1037693 8 AA AA AA AA AA
AA AA AA AA AA AG AG AA
AA AA AA AA AG AG AG AA
AA AA AA AA AA AA AA AA
AA AA AA AA AA AA AG
119028915
10179 rs1037698 8 AA AA AA AA AA
AA AA AA AA AA AA AA AA
AA AA AA AA AA AA AA AA
AA AA AA AA AA AA AA AA
AA AA AA AA AA AA AA
103292724
10180 rs1037719 5 CC CC CC CC CC
CC CC CC CC CC CC CC CC
CC CC CC CC CC CC AC CC
AC CC CC AC AC CC CC CC
CC CC AC CC CC CC CC
113623738
10181 rs1037738 11 AA AA AA AA AA
AA AA AA AA AA AA AA AA

AG AA AG AG AG AA AA AA
AA AA AA AA AA AA AA AA
AA AA AG AG AG AG AA
44439267
10182 rs1037754 15 GG GG GG AG AG
GG AA AG AG AA AG GG GG
GG GG GG GG GG GG GG GG
GG AG AG AG AG GG GG --
GG GG GG GG GG GG GG
35299625
10183 rs1037791 7 AA AA AA AA AA
AA AA AA AA AG AA AA AA
AG AG AA AA AA AA AG AA
AA AA AA AA AA AA AA AA
AA AA AG AA GG AG AG
16824662
10184 rs1037831 8 CC CC CC AC CC
AC AC AC AC AC AA AA AA
AA AC AA AA AC CC AC AC
AC AA AC AC AA AC AA AA
AA AA CC AC CC CC CC
76323329
10185 rs1037846 15 AG GG AG AG AG
AG AA AG AA AG AG AG AG
AA AG GG AG AG AG GG AG
AA AA AA AG AA AA AA AA
AA AG AA AG AG AA AG
64204870
10186 rs1037855 4 GG AG AA GG GG
GG AG AG AG AA AA AG AG
AA AG AA AG AG AG AG GG
GG AG AG GG GG AA AG AG
AA AG AA AA AG AG AA
43927834
10187 rs1037859 2 GG GG GG GG GG
GG GG GG GG GG GG GG GG
GG GG GG GG GG GG GG GG
GG GG GG GG GG GG GG GG
GG GG GG GG GG GG GG
84721892
10188 rs1037863 2 AA AA AA AA AA
AA AA AA AA AA AA AA AA
AA AA AA AA AA AG AG AG
AA AA AA AA AA AA AA AA
AA AA AG AG AA AA AA
84771480
10189 rs1037868 18 AC AA AA AC AA
AA AC AC AA AA AA AA AA
AA AA AC AA AC AA AA AC
AA AA AA AA AC AA AA AA

AC AA AC AC AA AA AC
65495994
10190 rs1037882 6 CC AC AC AC AC
CC AC CC AC CC CC CC CC
AC AC CC CC CC CC CC CC
AC AC AC AC AC CC AC AC
CC CC AC AC AC AC CC
66672165
10191 rs1037883 6 GG GG GG GG GG
GG AG GG GG GG GG GG GG
GG GG GG GG GG AG AG AG
GG GG GG GG GG GG GG GG
GG GG AG GG GG GG AG
66672155
10192 rs1037921 12 AA AA AA AA AA
AA AA AA AA AA AA AA AA
AA AA AA AA AA AA AA AA
AA AA AA AG AA AA AA AA
AA AA AA AA AA AA AA
27553566
10193 rs1037924 12 -- -- -- AA AA
AA AG AG AA AA AG AG AG
GG -- AA AG AG AA AA AG
AG AG AG AA AG AG AG AG
AA AG AG -- -- AG AA
27498107
10194 rs1037939 18 AG AA AG AG AG
AG GG AA GG AG GG GG GG
GG AG AG AA AG AA AA AA
AG AA AA AG AG AA AA AA
AG AG GG AG AG AA AG
42296342
10195 rs1037972 16 AA AA AA AG GG
AG AG AG AA GG GG GG AG
AA AG AG AG GG AA AA AA
AG AA AG AG AA GG AG AG
AA AA AG AA AG AA AG
81400127
10196 rs1037976 1 GG AG AG AG AG
AG GG AG AG AG GG GG GG
GG GG GG GG GG AA AG AA
GG AG GG AG AG AG AG GG
GG GG GG GG GG GG GG
38501187
10197 rs1038008 1 AG AG AG AG GG
AG GG GG AG AG AG AG AG
AA AG GG GG GG AG GG AG
AG GG AG GG AA GG AG AG
GG AA AG AG AA AG AA
8664922

10198 rs1038024 17 GG GG GG GG GG
GG GG GG GG GG GG GG GG
GG GG GG GG GG GG GG GG
GG GG GG GG GG GG GG GG
GG AG AG AA GG GG GG
25725605
10199 rs1038050 9 GG GG GG AG AA
AA AG AG AG AG AG AG AA
AA AA GG AG AG AG GG AG
GG GG GG GG GG AG GG GG
AG AG AG AA GG AG GG
1714233
10200 rs1038057 11 AA AA AA AA AA
AA AA AA AG AG AA GG AG
AG AG AA AG AA AG AA AA
AA AA AA AA AA AA AA AA
AG AA AA AA AA AG AA
109917159
10201 rs1038061 8 AA AA AA AG GG
AG GG AG AG AG AG AA AG
AA AA AG AA AG AG GG GG
AG GG GG AG GG AG AG GG
AG GG GG GG GG AG GG
4502531
10202 rs1038074 5 AG AG AG AA AA
AA AA AG AG AG AG AG AG
AG AA AA AA AA AA AA AA
AA AA AA AA AG AA AG AA
GG AA AA AA AG AG AA
150358957
10203 rs1038098 11 AG AG AG GG GG
GG GG AG GG GG GG GG GG
GG GG GG GG GG GG GG GG
GG GG GG GG GG GG GG GG
GG GG GG GG GG GG GG
127184036
10204 rs1038106 16 AG AG AG AA AA
AA AA AA AA AA GG AG AG
AA AG AA AA AA AA AA AA
AA AA AA AA AA AA AA AA
AA AA AA AA AA AA AA
7165350
10205 rs1038115 4 GG AG AG AG GG
AG GG AA AA AG GG GG GG
GG GG AG GG AG GG GG GG
GG AG AG AA AG GG AG GG
AG GG GG GG AG GG AA
57108271
10206 rs1038189 16 GG GG GG GG GG
GG AG GG GG AG AG AG AG

	AG	GG	AA	AG	AG	AA	AA	AG
	GG	GG	GG	GG	GG	GG	GG	GG
	GG	GG	GG	GG	GG	GG	GG	
27780378								
10207	rs1038273		3	AG	GG	AG	AG	GG
	AG	GG	AG	AG	GG	GG	GG	GG
	GG	GG	GG	GG	GG	AA	AA	AG
	AG	AG	GG	AG	GG	AG	AG	AG
	AG	AA	AG	AG	AA	AA	AA	
98786601								
10208	rs1038299		3	GG	GG	GG	GG	GG
	GG	GG	AG	AG	AG	AA	AG	AG
	AG	AG	AA	AA	AA	GG	AA	AG
	GG	GG	GG	GG	GG	AG	GG	GG
	AG	GG	AG	AG	AG	AG	GG	
14306278								
10209	rs1038317		6	GG	AG	AG	GG	GG
	GG	AG	AG	AA	GG	AG	AG	AA
	AG	AG	AG	AG	AG	GG	GG	GG
	AG	AG	AG	AG	AG	GG	AG	AG
	GG	AG	AG	GG	AG	AG	AG	
64712730								
10210	rs1038371		2	GG	GG	GG	AG	AA
	AG	AG	AA	AA	AG	GG	AG	AG
	GG	GG	AG	AG	GG	GG	AG	GG
	AA	AA	AA	AA	AG	GG	AG	GG
	AG	GG	AG	GG	AG	AG	GG	
111775206								
10211	rs1038378		5	AA	AA	AA	AA	AG
	AA	AG	AA	AA	AA	AA	AA	AA
	AA	AA	GG	AG	AG	AA	AA	AA
	AA	AA	AA	AA	AA	AA	AA	AA
	AA	AA	AA	AA	AA	AA	AG	
125885214								
10212	rs1038390		6	AA	AA	AA	AA	AG
	AG	AA	AA	AG	AG	AA	AA	AA
	AA	AA	AA	AA	AA	AA	AA	AA
	AG	AG	AA	AG	AA	AA	AA	AA
	AA	AA	AG	AA	AA	AG	AG	
143827102								
10213	rs1038419		16	AC	AC	CC	CC	AC
	CC	AC	CC	AC	CC	AC	AC	CC
	CC	CC	AC	AC	AC	AC	CC	AC
	CC	CC	AC	AC	CC	AA	AC	AC
	AA	CC	CC	CC	CC	AC	CC	
85433445								
10214	rs1038553		15	AA	AA	AA	AG	AG
	AA	AG	AA	AA	AA	AA	AA	AA
	AA	AA	AA	AA	AA	AA	AG	AA
	AA	AG	AG	AG	AA	AA	AA	AA

AA AA GG AG AA AA AA
53679121
10215 rs1038555 6 AG GG AG AG GG
AG GG GG AG GG GG GG GG
AG AG AG AG AG AG AG AG
AG AG GG AG AG AA AG AG
AG GG AG GG GG AG GG
153671375
10216 rs1038565 2 AA AC AC AC AA
AC AA AC CC CC AC AC CC
AC AC CC AC CC CC CC AC
CC AC CC CC AA CC AC AC
CC CC CC CC AC AC CC
173060526
10217 rs1038570 2 AA AA AC AA CC
AC AC AA AA AA AC AA AA
AC AC AA AC AA AC AC AC
CC AC CC AC AC AA AC AC
AA AA AA AA AC AC AC
62890171
10218 rs1038587 2 GG GG GG GG GG
GG GG AG GG AG GG AG AG
AG AA GG GG GG GG GG GG
GG GG AG AG GG GG GG GG
GG AG GG AG AG GG AG
186905790
10219 rs1038591 5 GG AG AA GG GG
GG GG GG AG GG GG GG GG
AG GG AG AA AG GG GG GG
AG AG AG AG AA GG AG AG
AG GG GG GG AG GG AG
22670144
10220 rs1038598 4 CC CC CC CC CC
CC CC CC CC CC CC CC CC
CC CC CC CC CC CC CC CC
AC AC CC CC CC CC CC CC
CC CC CC CC CC AC CC
188007418
10221 rs1038599 4 CC CC CC CC CC
CC CC CC CC CC AC AC CC
CC CC CC CC CC CC CC CC
AC AC AC CC AC CC AC AC
AC CC AC CC CC CC CC
188110164
10222 rs1038638 7 AA AG GG GG GG
GG GG GG GG GG GG GG GG
GG GG GG GG GG AG GG AG
AA AG AA AA GG GG GG GG
GG AA AG AG AG AG AG
130361092

10223	rs1038644		3	AA	AA	AA	AA	AA
	AA	AA	AG	AA	AA	AA	AA	AA
	AA	AA	AA	AA	AG	AA	AA	AA
	AA	AA	AA	AA	AA	AA	AA	AA
	AG	AA	AA	AA	AA	AG	AA	
173468188								
10224	rs1038670		18	GG	GG	GG	GG	GG
	GG	GG	GG	GG	GG	GG	AG	GG
	GG	GG	GG	AG	AG	GG	GG	AG
	AG	AG	AA	AG	GG	GG	GG	GG
	GG	GG	GG	GG	GG	GG	GG	
45898195								
10225	rs1038699		2	AA	AA	AA	AG	AG
	AA	GG	AG	AA	AG	AA	AG	AG
	AA	AG	GG	AG	AG	AG	AG	GG
	AG	AG	GG	AG	GG	AG	--	AG
	GG	GG	AG	GG	AG	AA	GG	
18528678								
10226	rs1038723		11	GG	AG	AA	AA	AA
	AA	AG	GG	GG	AG	GG	AG	AG
	GG	AG	GG	GG	GG	GG	GG	GG
	GG	AG	GG	AA	AG	GG	AG	AG
	AG	GG	AG	GG	AA	AA	AA	
1562713								
10227	rs1038727		11	GG	GG	GG	GG	GG
	AG	GG	GG	GG	GG	GG	GG	GG
	GG	AG	GG	GG	GG	AG	GG	GG
	GG	GG	GG	GG	GG	GG	GG	GG
	GG	GG	GG	GG	GG	GG	AG	
1681001								
10228	rs1038854		4	AA	AG	AG	AG	AA
	AG	AG	GG	GG	AG	GG	AA	AG
	AG	AG	AG	AG	AG	AG	AG	AG
	AA	AA	AA	AA	AG	AA	AA	AG
	AA	AA	AG	AA	AG	AG	AG	
108189246								
10229	rs1038864		16	AG	AA	AG	AA	AA
	AA	AA	AA	AA	AA	AA	AA	AA
	AG	AG	GG	GG	AG	AG	AA	AG
	AA	AA	AA	AA	AA	AA	AA	AA
	AA	AA	AA	AA	AG	AG	AA	
73762557								
10230	rs1038890		6	AG	GG	AG	AG	AA
	AA	AG	GG	AG	GG	AA	AA	AA
	AA	AA	AA	AA	AA	AG	AG	AG
	AA	AA	AA	AA	AG	AG	AG	AG
	AG	AA	GG	AG	GG	AG	GG	
42369308								
10231	rs1038920		5	GG	GG	AG	GG	GG
	GG	GG	AG	AG	GG	GG	AG	AG

GG GG AG AG GG AG GG AA
GG GG GG GG AG AG GG AG
GG AG GG AG AA AG AG
75957624
10232 rs1038962 11 GG AG AA AA GG
AG AG AG AA AA AA AA AA
AA AA AG GG AG AA AA AA
AA AA AA AA AA AA AA AA
AA AA AG AA AG AG GG
97045544
10233 rs1038983 1 AG GG AG AA AA
AA AA AA AA AA GG GG GG
AG GG AG GG AA AG AG AG
AA GG AG AG AG GG GG AG
AG AA AA AA GG GG GG
78104852
10234 rs1038993 5 AC AA AC AA AA
AA AA AA AA AA AC AC AC
CC AC CC AC AC AC CC CC
AA AA AA AA CC AC CC CC
CC AA AA AA AC AA CC
157713315
10235 rs1039001 6 GG GG GG AG AG
GG AG GG GG GG GG AG AG
AG AG AG AA AG GG GG GG
GG GG GG GG GG AA AG AG
GG GG AG AG GG GG AG
166146354
10236 rs1039002 6 GG GG GG GG GG
GG GG GG GG GG GG GG GG
GG GG GG GG GG GG GG GG
GG GG GG GG GG GG GG GG
GG GG GG GG GG GG GG
166155457
10237 rs1039032 6 AG AA AG AG AG
AA GG AG AG AG AG AG AA
AG AA AA AG AA AA AA AG
AA AA AG AA AG AA AA AA
AA AG AG AA AA AA AG
101161931
10238 rs1039038 11 CC AC AC CC AC
AC CC AA AC AC AC CC AC
AA AC AA AA AA CC AC AC
AC AC AA CC CC AC CC AC
AC AC AC CC AA AC AC
40511611
10239 rs1039047 8 GG GG GG GG GG
GG GG GG GG GG GG AG AG
AG AG GG GG GG AG AG AG
GG AG GG GG GG AG AG AG

AA GG GG GG GG GG GG
80547209
10240 rs1039053 4 CC AC AC AC AA
AC AA AC AA AA CC CC AC
AA AC CC AC AC CC CC AC
CC AA AA AC AC AC AC AC
AC AC CC AC AC AC CC
81402774
10241 rs1039084 6 AG AG AA AG AG
GG AA AA AA AA AG AG GG
GG GG GG AG AG GG GG GG
AA AA AA AA GG GG GG GG
AG AG AG AG AG AG GG
147635413
10242 rs1039089 5 GG GG GG GG GG
GG AG AG AG AG GG GG AG
AG AG AG GG AG GG GG GG
GG GG GG GG GG GG GG GG
GG GG GG GG GG GG GG
174892501
10243 rs1039122 8 AG AG AG GG GG
GG GG GG AG AG GG AG GG
GG AG GG GG GG AG GG AG
GG GG GG GG AG GG GG AG
GG GG GG GG GG GG GG
19972368
10244 rs1039131 1 GG GG GG GG GG
GG GG GG GG GG GG GG GG
GG GG GG GG GG GG GG GG
GG GG GG GG GG GG GG GG
GG GG GG GG GG GG GG
48515250
10245 rs1039137 8 CC CC AC AC AC
AA CC AC AC AC CC CC CC
AC AA CC CC CC AC CC AC
AC AC AC AC CC AC CC CC
AA AA CC AC CC CC CC
115301032
10246 rs1039141 8 AA AA AC AC AC
CC AA AC AC AC AA AA AA
AC CC AA AA AA AC AA AC
AC AC AC AC AC AC AA AC
CC CC AA AC AC AC AA
115327416
10247 rs1039183 1 AA AA AA -- AC
CC AA AA AA AA AA AA AA
AA AA AA AA AA AC AA AA
AC AA AC AA AA AC AA AA
AC CC AA AC AA AA AA
31058785

10248 rs1039197 10 AA AC AA CC CC
CC CC CC CC CC CC CC CC
CC CC AA AC AC AC AA AC
CC CC CC AC CC CC CC CC
AC CC CC CC AC AC AC
4659197
10249 rs1039229 12 AG AA AG AG AG
GG AG AA AA AG GG AG GG
AA AA AG AA AG AA AA AG
AG AG AA GG AA AA AA AA
GG AA AG AA AA AA AG
24185520
10250 rs1039282
17 -- -- -- -- -- -- -- --
-- -- -- -- -- -- -- --
-- -- -- -- -- -- -- --
-- -- -- -- -- -- -- --
-- -- -- -- 18554514
10251 rs1039298 12 AA AA AA AG AG
GG AA GG AG GG GG GG GG
AG GG AA AA AA GG AG AG
AA AA AA AA AA AG AA AG
AA AA AA AA AA AA AG
115839791
10252 rs1039302 12 AG AG AG GG GG
GG GG GG GG GG GG GG GG
GG GG AG AG GG AG AG GG
GG GG GG GG AG AG GG AA
GG AG GG AG GG GG GG
121236258
10253 rs1039321 5 AA AG AG AA AA
AA AA AA AA AA AA AA AA
AA AA AA AA AG AA AA AA
AA AG AA AA AG AG AG AG
GG AA AG AG AA AA AA
73937506
10254 rs1039324 5 AA AA AA GG GG
AG AG AA AA AA AA AG AA
AA AA AA AA AA AA GG AG
AA AA AA AA AA AG AA AG
AA AA AG AA AA AG AG
85102498
10255 rs1039343 16 AC AA AC AC AC
AA AC AC AC AC CC CC AC
AC AC AC AC AC AC AC CC
AA AA AA AA AA AC AA AC
CC AC AC AC CC AC CC
48577507
10256 rs1039441 5 AG GG GG GG GG
GG AG GG GG GG GG GG GG

GG GG AG GG GG GG GG AG
GG GG GG GG AG AG AG AG
GG GG AG GG GG GG GG
50560963
10257 rs1039443 5 AA AA AA AA AA
AA AG AA AA AA AG AA AA
AA AA AA AA AA GG GG AG
AA AA AG AA AG AA AG AG
AG AA AA AA AG GG AA
16038102
10258 rs1039457 15 AA AA AA GG GG
GG AG GG AG GG GG GG GG
GG GG GG AG AG GG AA GG
AG AG AG AG GG GG GG GG
GG AA AG AG GG GG GG
38373830
10259 rs1039458 15 GG GG GG GG GG
GG GG GG GG GG GG GG GG
GG GG GG GG GG GG GG GG
GG GG GG GG GG GG GG GG
GG GG GG GG GG GG GG
38367621
10260 rs1039460 5 CC AC CC CC CC
CC CC CC CC CC CC CC CC
AC AC CC AC AC AC AC AC
AA AC CC AC AC CC CC CC
CC CC CC CC CC CC CC
3703076
10261 rs1039462 5 AG AG AA GG AG
AG AG AA AG AA AA AA AA
AG AG AG AA AG AG AG AG
GG GG AG GG AA AA AA AA
AA AG AA AG AA AG AG
3771121
10262 rs1039481 11 AG AA AA AA AA
AA AA AA AA AA AG AA AG
GG AG AG AA GG AG AG AA
AA AA AA AA AA AA AA AA
AA AG AG AA AG AG AA
48182237
10263 rs1039524 3 AG GG AG AG AG
GG AA AG AG AA AG GG AG
AA AA AG AA AG AG AG AG
GG GG GG GG AG AG AA AA
GG AG AG AG AG AG GG
114012274
10264 rs1039528 1 GG AG GG AG GG
AG GG AG AG GG AA GG AG
AG AG GG GG GG AG AG AG
GG GG GG GG GG AG AG GG

AG AG AA AA GG GG GG
242710738
10265 rs1039599 5 AA AA AA AA AA
AA AA AA AA AA AA AA AA
AA AA AA AA AA AA AA AA
AA AA AA AA AA AA AA AA
AA AA AA AA AA AA AA
168300723
10266 rs1039609 18 AG AG AA AG GG
AG AG AA AA AG AA AA AA
AG AG AA AA AA AA AA AG
AG AG AA AG AG AG GG AG
AG AA AA AA AG AG AG
75949051
10267 rs1039611 18 AC AA AA AA AC
AA AC AA AA AA AA AA AA
AC AC AC AC AA AC AC AA
AA AA AA AA AC AC AA AC
AC AC AA AA AA AA AA
75967361
10268 rs1039630 1 AG AA AG AA AA
AG AA AG AG AG AG AG AA
AA AA AG AA AA AA AA AA
AA AA AG AA AG AG AG AG
AA AG AA AG GG GG GG
31881472
10269 rs1039664 7 CC AC AC AA AA
AA AA AA AA AA AC AA AC
AC AC AA AA AA AA AA AA
AA AA AA AA AA AA AA AA
AA AC AA AA AC AA AC
65449716
10270 rs1039669 12 CC CC CC CC CC
CC CC CC CC CC CC CC CC
CC CC AC AC AC AC AC CC
CC CC CC CC CC CC CC CC
CC CC CC CC CC AC AC
45313082
10271 rs1039682 4 GG GG AG GG GG
AG GG GG AG GG AG GG AG
AG GG GG GG GG GG GG AG
GG GG GG AG AG AG GG AG
GG AG GG GG GG GG GG
11050420
10272 rs1039797 5 AA AA AG GG GG
GG GG AA AA AA GG GG GG
AG GG AA AG AG GG AG GG
AA AG AG AA AG GG GG AG
AA AA AG AA AG AG AA
49651872

10273 rs10398 11 AA AA AA AG AG AA
GG AA AA AA AA AA AA AG
AG AG AA AA AA AA AA AG
AA AG AA AA AA AA AA AA
AA AA AA AA AA AA 308180
10274 rs1039832 11 AG AA AG AA AG
AA AG AG AA AA AA AG AA
AA AA AA AG AA AA AG AA
AA AA AA AA GG AA AG AG
AA AA AG AG AA AA AA
113478201
10275 rs1039857 8 AA AA AA AA AA
AA AA AA AA AA AA AG AG
AG GG GG AG GG AA AA AG
AA AG AG AG AG AG AG AG
AA AA AG AA AA AG AA
78551702
10276 rs1039878 3 AG AG GG AG AG
GG AG AA AA AA AG AA AG
AG AG AA AA AA AA AA AA
AA AG AG AA AG AG AG GG
AA AA AA AA GG GG AG
68475128
10277 rs1039887 2 AG AG AG AA AA
AA AA AA AA AA AA AA AA
AA AA AA AA AA AG AA AG
AA AA AA AA AA AA AA AA
AA AA AA AA AA AA AA
239434892
10278 rs1039888 2 AG GG GG GG GG
AG GG AA AG GG GG GG GG
GG GG AG AG AG GG GG GG
GG GG GG GG GG GG GG GG
AG AG AG GG GG GG GG
239533669
10279 rs1039916 8 GG GG GG GG GG
GG AG GG AG AG AA AG AA
AG AG AG AA AG AG AA AG
AG AG AA GG AG AA AG AG
AA AG AG GG AG AG AG
8685854
10280 rs1039938 4 AG AA AA AG AG
AA AG AG AG GG AG AA AA
AG AG AA AG AG AG AG GG
AA AG AG AG AA GG AG AG
AA AG AG AG AG AG GG
93918343
10281 rs1039944 3 AA AA AA AA AA
AA AA AG AG AG AA AA AA
AA AA AG AA AG AA AA AA

	AA	AA	AA	AA	AA	AA	AA	AA
	AG	AA	AA	AA	AA	AA	AA	
138707522								
10282	rs1039950		11	AA	AA	AA	AA	AA
	AA	AA	AA	AA	AA	AA	AA	AA
	AA	AA	AA	AA	AA	AA	AA	AA
	AA	AA	AA	AA	AA	AA	AA	AA
	AA	AA	AA	AA	AA	AA	AA	
97624190								
10283	rs10399658		1	AC	AC	AA	AA	AC
	AA	AC	CC	CC	CC	CC	CC	CC
	CC	AC	AC	AA	AC	AC	AC	AA
	AA	AC	AA	AC	CC	AC	AC	AC
	AC	CC	AC	AC	AC	CC	AA	
145686437								
10284	rs10399665		1	AG	GG	AG	AG	GG
	GG	AG	AG	GG	GG	AG	AG	GG
	GG	GG	AA	AA	AA	AG	AA	GG
	GG	AG	AG	AA	GG	GG	GG	GG
	GG	GG	GG	GG	GG	AG	GG	
8407287								
10285	rs10399738		1	GG	GG	GG	GG	GG
	GG	GG	GG	GG	GG	GG	GG	GG
	GG	GG	GG	GG	GG	GG	GG	GG
	GG	GG	GG	GG	GG	GG	GG	GG
	GG	GG	GG	GG	GG	GG	GG	
66252128								
10286	rs1039987		4	GG	GG	AG	AG	AG
	GG	AA	GG	GG	AG	GG	GG	GG
	GG	AG	GG	GG	GG	AG	AG	GG
	AA	AG	AA	AA	GG	GG	GG	GG
	GG	GG	AG	AG	GG	AG	GG	
141262661								
10287	rs1039990		3	GG	GG	GG	GG	GG
	GG	GG	GG	GG	AG	AA	AG	AG
	GG	GG	AG	GG	GG	GG	GG	GG
	GG	GG	GG	GG	GG	AG	GG	GG
	GG	GG	GG	GG	GG	GG	AG	
89558086								
10288	rs10399934		1	AA	AA	AA	GG	AA
	AG	AG	AG	AG	AG	AA	AA	AA
	AG	AG	AA	AA	AA	AA	AA	AA
	AA	AA	AA	AA	AG	AA	AG	AG
	AA	AA	AA	AA	AG	GG	AG	
117996342								
10289	rs10399991		10	AG	GG	GG	GG	GG
	GG	GG	GG	GG	GG	GG	GG	GG
	GG	GG	GG	GG	GG	GG	GG	AG
	GG	GG	GG	GG	AG	GG	GG	AG
	GG	GG	GG	GG	GG	GG	GG	

64637188								
10290	rs1040002		12	GG	GG	GG	GG	AG
	GG	AG	GG	GG	AG	GG	GG	GG
	GG	GG	AG	AG	AG	AG	AG	AA
	GG	GG	GG	GG	AG	AG	AG	AG
	GG	AG	AG	AG	GG	GG	AG	
76948360								
10291	rs10400070		10	AG	AG	AA	GG	GG
	AG	GG	AG	AG	AG	AG	GG	AG
	AG	AG	AG	AG	GG	GG	GG	GG
	GG	AG	GG	AG	GG	GG	GG	GG
	GG	AG	GG	GG	GG	GG	GG	
113691771								
10292	rs10400240		11	AC	AC	AA	AC	AC
	AA	AC	CC	AC	AC	AA	AC	AC
	AA	AC	AC	AA	AC	AA	AC	AA
	CC	AA	AC	AC	AC	CC	AC	AC
	AA	AC	AC	AA	AC	AA	CC	
43559991								
10293	rs10400291		11	CC	CC	CC	CC	AC
	CC	AC	AC	AC	AC	CC	CC	CC
	CC	CC	CC	CC	CC	AC	AC	CC
	CC	CC	CC	CC	CC	CC	CC	CC
	CC	AC	CC	CC	AC	AC	CC	
86304054								
10294	rs10400305		11	AA	AA	AA	AA	AA
	AA	AA	AA	AA	AA	AA	AA	AA
	AA	AA	AA	AA	AA	AA	AA	AA
	AA	AA	AA	AA	AA	AA	AA	AA
	AA	AA	AG	AG	GG	AG	GG	
57148731								
10295	rs10400327		11	AA	AA	AA	AA	AA
	AA	AA	AA	AA	AA	AA	AA	AA
	AA	AA	AA	AA	AA	AA	AA	AA
	AA	AA	AA	AA	AA	AA	AA	AA
	AA	AA	AA	AA	AA	AA	AA	
71778075								
10296	rs1040033		3	AC	AC	AC	AC	AC
	CC	AA	AC	AC	AA	AC	AC	AC
	AA	AA	AC	AC	AC	AC	AC	AA
	AA	AA	AA	AA	AC	AA	AA	AA
	AA	AC	AA	AC	AA	AA	AA	
188485240								
10297	rs10400334		11	AG	AG	AG	AA	AG
	AA	AG	AA	AA	AA	AG	AG	AG
	AA	AG	AA	AA	AA	AG	AA	AG
	GG	AA	GG	AG	AG	AA	AA	AG
	AA	AA	GG	AG	AG	AA	GG	
128521468								
10298	rs10400381		11	AA	AA	AA	AA	AA

AA AA AA AA AA AA AA AA
AA AA AA AA AA AA AA AA
AA AG AA AG AA AA AA AA
AA AA AA AA AA AA AG
125143775
10299 rs10400410 12 GG GG GG AG GG
GG AG GG AG AA GG GG GG
GG GG GG GG GG AG GG GG
AG AG GG AG AA GG AG AG
AG GG GG GG GG GG GG
111193499
10300 rs10400432 12 GG GG GG GG GG
GG GG GG GG GG GG GG GG
GG GG GG GG GG GG GG GG
GG GG GG GG GG GG GG GG
GG GG GG GG GG GG GG
87383479
10301 rs1040046 6 CC CC CC CC CC
CC CC AC AC AC CC CC CC
AC AC AC AC AC CC CC CC
CC CC CC CC AC AC CC CC
CC CC AC AC AC AC CC
83473573
10302 rs10400464 12 CC CC CC CC CC
CC CC CC CC CC CC CC CC
CC CC CC CC CC CC CC CC
CC CC CC CC CC CC CC CC
CC CC CC CC CC CC CC
133170442
10303 rs10400465 12 AA AA AA AC AC
AC AA AA AA AA AA AA AA
AA AA AA AA AA AA AA AA
AA AA AA AA AA AC AA AA
AC AA AA AA AC AA AC
75469690
10304 rs1040049 2 AA AG AA AG AG
AG GG AA AA AA AA AG AG
AG AG AG AA AG AA AG AG
AA AA AA AA AA AG AA AG
AA GG AG GG AG AA AG
124488139
10305 rs10400510 12 AA AA AA AA AA
AA AA AA AA AA AA AA AA
AA AA AA AA AA AA AA AA
AA AA AA AA AA AA AA AA
AA AA AA AA AA AA AA
42799013
10306 rs10400545 12 AA AA AG AG AA
AG AA AA AA AA AA AG AA
AA AA AA AA AA AA AG AA

	AA	AA	AA	AA	AA	AG	AG	AA
	AA	AA	AA	AA	AA	AA	AA	
121779733								
10307	rs10400629		13	GG	AG	GG	AA	AA
	AA	AG	GG	AG	AG	AG	AG	GG
	AG	AG	AG	AA	AA	AA	AA	AA
	AA	GG	AA	AA	GG	GG	GG	GG
	GG	AG	AA	AA	AG	AG	GG	
44852020								
10308	rs10400673		13	AG	--	AG	--	--
	--	--	--	--	--	AG	AG	--
	--	--	AG	AG	AG	--	--	--
	AG	--	--	--	AG	--	AG	--
	--	--	--	--	--	--	--	
21253566								
10309	rs10400697		14	AA	AA	AA	AA	AA
	AA	AA	AA	AA	AA	AA	AA	AA
	AA	AA	AA	AA	AA	AA	AA	AA
	AA	AA	AA	AA	AA	AA	AA	AA
	AA	AA	AA	AA	AA	AA	AA	
60770367								
10310	rs10400789		14	AG	AG	GG	AG	AG
	AA	GG	GG	GG	GG	GG	AG	AG
	AG	AG	GG	GG	AG	GG	GG	GG
	GG	GG	AG	AG	GG	GG	GG	GG
	AG	GG	AG	GG	GG	GG	GG	
51967896								
10311	rs10400812		15	AA	AA	AA	GG	AG
	GG	AG	AG	AA	AA	GG	AG	AG
	GG	AG	AG	AG	AG	AA	AA	AG
	GG	GG	GG	GG	AG	GG	GG	AG
	GG	AA	GG	AG	AA	AA	AG	
83689688								
10312	rs10400854		15	AG	AG	AA	AG	AA
	AG	AA	AA	AA	AA	AA	AG	AA
	AG	AG	AA	AA	AA	AA	AA	AA
	AA	AA	AA	AA	AA	AA	AA	AA
	GG	AA	AA	AA	AA	AA	AA	
100660150								
10313	rs10400880		15	AA	AA	AA	AA	AA
	AA	AA	GG	AG	AG	AA	AG	AG
	AG	AG	AG	AA	AA	AG	AG	AA
	GG	AG	AG	AA	AA	AG	AA	AG
	AG	AG	AG	AA	AA	AG	AG	
69927554								
10314	rs10400943		16	CC	CC	CC	CC	CC
	CC	CC	CC	CC	CC	CC	CC	CC
	CC	CC	CC	CC	CC	CC	CC	CC
	CC	CC	CC	CC	CC	CC	CC	CC
	CC	CC	CC	CC	CC	CC	CC	

81198306								
10315	rs10400951		16	GG	GG	GG	GG	GG
	GG	GG	GG	GG	GG	AA	AA	AA
	AA	AG	AG	GG	GG	AA	AA	AG
	GG	GG	GG	GG	GG	GG	GG	GG
	GG	GG	AG	GG	GG	GG	GG	
7536036								
10316	rs10400954		16	GG	GG	GG	AG	AA
	AG	AA	AG	GG	AG	GG	AG	AG
	AG	GG	AG	AG	AG	AG	AA	AG
	AG	AA	AA	AG	GG	AG	GG	AG
	--	GG	GG	GG	GG	GG	AG	
8694634								
10317	rs10400958		16	AG	GG	AG	GG	AG
	GG	AG	GG	GG	GG	GG	GG	GG
	GG	GG	AG	GG	GG	AG	GG	GG
	AA	AA	AG	GG	GG	AG	AG	AG
	GG	GG	GG	GG	GG	GG	GG	
89297952								
10318	rs10400986		16	GG	GG	GG	GG	GG
	GG	GG	GG	GG	GG	GG	GG	GG
	GG	GG	AG	AG	AG	GG	GG	GG
	GG	GG	GG	GG	GG	AG	AG	GG
	AG	GG	AG	AG	GG	GG	AG	
81200465								
10319	rs10401006		17	AA	AA	AA	AA	AA
	AA	AA	AA	AA	AG	AA	AA	AA
	AA	AA	AA	AA	AA	GG	AG	AG
	AG	AA	AA	AA	AA	AA	AA	AA
	AA	AA	AG	AA	AA	AA	AA	
11820872								
10320	rs10401041		17	AA	AA	AA	AA	AA
	AA	AA	AA	AA	AA	AA	AA	AA
	AA	AA	AA	AA	AA	AA	AA	AA
	AA	AA	AA	AA	AA	AA	AA	AA
	AA	AA	AA	AA	AA	AA	AA	
21532851								
10321	rs10401042		17	AG	AA	AA	AA	AA
	AA	AA	AA	AA	AA	AG	AA	AA
	AA	AA	AA	AA	AA	AG	AG	AA
	AG	AG	AA	AA	AA	AA	AA	AA
	AA	AG	AA	AA	AA	AG	AA	
13840831								
10322	rs10401044		17	GG	GG	GG	GG	GG
	GG	GG	GG	GG	GG	GG	GG	GG
	GG	GG	GG	GG	GG	GG	GG	GG
	GG	GG	GG	GG	GG	GG	GG	GG
	GG	GG	GG	GG	GG	GG	GG	
80283968								
10323	rs10401045		17	GG	GG	GG	GG	GG

GG GG GG GG GG GG GG GG
GG GG GG GG GG GG GG GG
GG GG GG GG GG GG GG GG
GG GG GG GG GG GG GG
78705820
10324 rs10401065 18 AA AC AC CC CC
AC AC AC AA AC AA AA AA
AA AA AA AA AA AC AA CC
AC AA AA AC AA AA AA AA
AA AA AA AA CC CC CC
38652375
10325 rs10401070 18 AG GG GG GG GG
GG GG AG AG AA GG GG GG
GG GG AG GG AG GG GG GG
AG AG GG AG AG GG AG AG
GG GG GG GG AG GG AG
45877135
10326 rs10401133 19 AA AA AA AG AA
AA AG AA AA AA AG AA AG
AA AA AA AA AA AA AA AA
AA AA AA AA AA AA AA AA
AA AA AA AA AA AA AA
16574508
10327 rs10401166 19 GG GG GG AG GG
AG GG GG GG GG GG GG GG
GG GG AG GG AG GG AG GG
AG GG GG AG GG GG GG GG
AG GG GG GG GG GG GG
24203419
10328 rs10401177 19 GG AG AA GG GG
GG AG AA GG GG GG GG GG
AG AG GG AG AG AG AG GG
AG GG AA AA GG AA AG AG
GG GG GG GG AA AA AA
3874604
10329 rs10401209 19 GG GG GG GG GG
GG GG GG GG GG GG GG GG
GG GG GG GG GG GG GG GG
GG GG GG GG GG GG GG GG
GG GG GG GG GG GG GG
24252858
10330 rs1040125 2 AA AA AA AA AA
AA AA AA AA AA AA AA AA
AA AA AA AA AA AA AA AA
AA AA AA AA AA AA AA AA
AA AA AA AA AA AA AA
158161523
10331 rs10401250 19 AA AA AA AA AA
AA AA AA AA AA AA AA AA
AA AA AA AA AA AA AA AA

AA AA AA AA AA AA AA AA
AA AA AA AA AA AA AA
23031075
10332 rs10401267 19 GG GG GG GG GG
GG GG GG GG GG GG GG GG
GG GG GG GG GG GG GG GG
GG GG GG GG GG GG GG GG
GG GG GG GG GG GG GG
8606775
10333 rs10401300 19 AG AG AG AA AG
AA AG AA AA AA AA AA AA
AA AA AA AA AA AA AA AA
AA AA AA AA GG AA AG AG
AA AG AA AA AA AA AA
8673881
10334 rs10401357 19 GG GG GG GG GG
AG GG AG AG AG AG AG AG
AG GG GG GG GG AG AG GG
GG GG GG GG GG AG GG GG
AA AG AG AG AA AA AA
54139159
10335 rs10401460 19 AA AA AA AA AA
AA AA AA AA AA AA AA AA
AA AA AA AA AA AA AA AA
AA AA AA AA AA AA AA AA
AA AA AA AA AA AA AA
32069257
10336 rs10401492 19 AG AG AG AG GG
AG GG GG GG GG GG AG AG
GG GG AA AG AG GG GG AG
GG AG AG GG AG GG GG AG
AG AA AG AA GG GG GG
28816938
10337 rs10401509 19 AG GG GG GG GG
GG GG GG GG GG GG GG GG
GG GG GG GG GG GG GG AG
GG GG GG GG GG GG GG GG
GG GG GG GG GG GG AG
51369975
10338 rs10401545 19 GG GG GG GG GG
GG GG AG AA AA GG GG GG
AG AG GG GG GG AG AG AG
AG AG GG AG AG AG AG AA
AG GG AG GG GG GG GG
29437307
10339 rs1040155 6 AG AG AA AA GG
AG AG AA AA AA AG AG AG
AG AG AG AA AA AA AA AA
AG AG AG AG AA AA AA AA
AA AA AA AA AG AG AA

93561150								
10340	rs10401693		19	AA	AG	AA	GG	AG
	AG	GG	AA	AG	AG	AG	AA	AA
	AA	AA	GG	GG	GG	AA	AG	AA
	GG	GG	GG	GG	AG	AA	AG	AA
	AG	AA	GG	AG	AG	GG	AA	
24070297								
10341	rs10401699		19	AA	AA	AA	AA	AA
	AA	AA	AA	AA	AA	AA	AA	AA
	AA	AA	AA	AA	AA	AA	AA	AA
	AA	AA	AA	AA	AA	AA	AA	AA
	AA	AA	AA	AA	AA	AA	AA	
54192352								
10342	rs10401716		19	GG	GG	GG	GG	GG
	GG	GG	GG	GG	GG	GG	GG	GG
	GG	GG	GG	GG	GG	GG	GG	GG
	GG	GG	GG	GG	GG	GG	GG	GG
	GG	GG	GG	GG	GG	GG	GG	
17654420								
10343	rs1040173		2	AG	GG	AG	GG	GG
	AG	AG	AG	AA	AA	AA	AG	AA
	AG	AG	AG	AG	AA	AG	AA	AG
	GG	AG	AG	GG	AG	GG	GG	AG
	AG	AG	GG	GG	GG	GG	GG	
146120511								
10344	rs10401797		19	CC	CC	CC	CC	CC
	CC	CC	CC	CC	CC	CC	CC	CC
	CC	CC	CC	CC	CC	CC	CC	CC
	CC	CC	CC	CC	CC	CC	CC	CC
	CC	CC	CC	CC	CC	CC	CC	
19071422								
10345	rs10401848							
19	--	--	--	--	--	--	--	--
	--	--	--	--	--	--	--	--
	--	--	--	--	--	--	--	--
	--	--	--	--	--	--	--	--
	--	--	--	--	24340861			
10346	rs10401899		19	AA	AA	AA	AG	AG
	AA	AG	AA	AA	AA	AA	AA	AA
	AA	AA	AA	AA	AA	AA	AA	AA
	AA	AA	AA	AA	AA	AA	AA	AA
	AA	AA	AA	AA	AA	AA	AA	
5650351								
10347	rs10401901		19	AC	AC	AC	AC	AC
	AC	AA	AC	AA	AA	CC	AC	AC
	CC	CC	AC	CC	AC	CC	CC	CC
	AA	AC	AC	AA	AA	CC	AC	AC
	AC	AA	AC	AC	AA	AC	AA	
54956254								
10348	rs10401910		19	AA	AA	AA	AA	AA

	AA	AA	AA	AA	AA	AA	AA	AA
	AA	AA	AA	AA	AA	AA	AA	AA
	AA	AA	AA	AA	AA	AA	AA	AA
	AA	AA	AA	AA	AA	AA	AA	
22805656								
10349	rs10401970		19	AA	AA	AA	AA	AG
	AG	AA	AA	AA	AA	AA	AA	AA
	AA	AA	AA	AA	AA	AA	AA	AA
	AA	AA	AA	AA	AA	AA	AA	AA
	AG	AA	AA	AA	AG	AG	AA	
23641583								
10350	rs10401987		19	AA	AA	AA	AA	AA
	AA	AA	AA	AA	AA	AA	AA	AA
	AA	AA	AA	AA	AA	AA	AA	AA
	AA	AA	AA	AA	AA	AA	AA	AA
	AA	AA	AA	AA	AA	AA	AA	
18208448								
10351	rs10402072		19	AA	AA	AA	AG	AA
	AA	AG	AA	AA	AA	AA	AG	AG
	AA	AA	AA	AA	AA	AA	AA	AA
	AA	AA	AA	AA	AA	AA	AA	AA
	AA	AA	AG	AG	AG	AG	AA	
15785642								
10352	rs1040209		4	AA	AA	AG	AG	GG
	AG	AG	AG	AA	AG	AA	AG	AA
	AA	AG	AA	AA	AA	AA	AA	AA
	AG	AG	AG	AA	GG	AG	AG	GG
	AG	AA	AG	AG	AG	AG	AG	
16072487								
10353	rs10402091		19	GG	GG	GG	GG	GG
	AG	GG	AG	GG	GG	AG	GG	AG
	GG	GG	AA	AG	AG	AG	GG	AG
	GG	GG	GG	GG	GG	GG	GG	GG
	AG	AG	GG	AG	AG	AG	AG	
58397475								
10354	rs1040219		15	AG	AG	GG	GG	GG
	GG	GG	AG	GG	AA	AA	AA	AA
	AG	AA	AG	GG	AG	AG	GG	AA
	GG	GG	GG	GG	AA	GG	AG	AG
	GG	AG	GG	GG	AG	GG	AA	
36487472								
10355	rs10402201		19	AA	AA	AA	AA	AA
	AA	AA	AA	AA	AA	AA	AA	AA
	AA	AA	AA	AA	AA	AA	AA	AA
	AA	AA	AA	AA	AA	AA	AA	AA
	AA	AA	AA	AA	AA	AA	AA	
56810118								
10356	rs10402217		19	AC	AC	AC	AC	AA
	AC	AA	AA	AA	AA	AA	AC	AC
	AA	AA	AC	AA	AC	AA	AA	AC

AA AC AC AA AA AA AA AA
AA CC AC CC AA AA AA
28786657
10357 rs10402222 19 GG GG GG GG GG
GG GG GG GG GG GG GG GG
GG GG GG GG GG GG GG GG
GG GG GG GG GG GG GG GG
GG GG GG GG GG GG GG
55727511
10358 rs10402271 19 AC AA AA AC AA
AC AA AA AA AA AA AC AA
AC CC AA AC AC AA AC AA
AC AA AA AC AA AC AA AA
AC AC AC AA AA AA AC
45329214
10359 rs10402308 19 GG GG GG GG GG
GG GG AG GG GG GG GG GG
GG GG GG GG GG AG GG GG
AG GG GG AG GG AG GG GG
GG GG AG GG AG AG GG
19657500
10360 rs10402336 19 GG GG GG AG AG
GG AG GG GG GG GG AG GG
GG GG GG GG GG AG AG GG
GG GG GG GG GG GG GG GG
GG GG GG GG GG AG GG
3637380
10361 rs10402371 19 CC CC CC CC CC
CC CC CC CC CC CC CC CC
CC CC CC CC CC CC CC CC
CC CC CC CC CC CC CC CC
CC CC CC CC CC CC CC
5010047
10362 rs10402422 19 AA AG AA AG AA
AA AG AG AA AA AG AG AG
AG AG AA AA AA AA AA AG
AA AA AA AA AA AA AA AA
GG AA AA AA AA AA AG
16475388
10363 rs10402423 19 AG AG GG GG GG
GG GG AA AG AA GG GG GG
GG GG GG GG GG GG GG GG
GG GG GG GG GG GG GG GG
GG AG GG GG GG GG GG
1546180
10364 rs1040247 8 AG GG AG GG AG
AG GG GG GG GG AG AG AG
AG GG GG GG GG GG GG GG
GG GG GG GG GG AG GG GG
GG GG GG GG GG GG GG

139533367
10365 rs10402501 19 AA AA AA AA AA
AA AA AA AA AA AA AA AA
AA AA AA AA AA AA AA AA
AA AA AA AA AA AA AA AA
AA AA AA AA AA AA AA
57920318
10366 rs10402525 19 AA AA AA AA AA
AA AA AA AA AA AA AA AA
AA AA AA AA AA AA AA AA
AA AA AA AA AA AA AA AA
AA AA AA AA AA AA AA
11514566
10367 rs1040258 6 AA AG AG AA AA
AA AA AG AA AG AG AG AA
AA AA AA AA AA AA AA AA
AA AA AG AA AA AA AA AA
AA AA GG AG AG AA AG
114572279
10368 rs10402645 19 GG GG GG GG GG
GG GG GG GG GG GG GG GG
GG GG GG GG GG GG GG GG
GG GG GG GG AG GG AG GG
AG GG GG GG GG GG GG
13197752
10369 rs10402677 19 GG GG GG GG GG
GG GG GG GG GG GG AA AG
AG AG GG GG GG AG GG GG
GG AG GG AG GG GG GG GG
GG AG GG GG GG GG GG
43025170
10370 rs10402680 19 AG AG AG AG AA
AG AA AA AA AA AA AG AG
AA AA GG AG AG AA AA AG
AA AG AG AA AG AA AA AG
AG GG AG GG AA AA AA
28817141
10371 rs1040278 4 GG AG GG AA AA
AA AA GG GG AG GG AA AG
AG AG AG AG AG AA AA AA
AG AA AA AG AG AA AG AA
AG AG AG GG AA AA AG
113079391
10372 rs10402841 19 GG GG GG GG GG
GG GG GG GG GG GG GG GG
GG GG GG GG GG GG GG GG
GG GG GG GG GG GG GG GG
GG GG GG GG GG GG GG
6047963
10373 rs1040285 6 GG GG GG AG GG

	AG	GG	GG	AG	GG	AG	AG	AG
	AG	GG	GG	GG	GG	GG	GG	GG
	AG	AG	AG	AG	GG	GG	GG	GG
	GG	GG	AG	GG	AG	GG	AA	
109583186								
10374	rs10402888		19	AA	AC	AA	AC	AA
	AC	AA	AA	AC	AC	AA	AA	AA
	AA	AA	AA	AC	AC	AA	AC	AA
	AC	AC	AC	AC	AC	AA	AC	AA
	AC	AA	AC	AA	AC	AC	AA	
24152735								
10375	rs10402912		19	AA	AG	AA	AA	AA
	AA	AA	AA	AA	AA	AA	AG	AG
	AA	AA	AA	AG	AG	AA	AA	AA
	AA	AA	AA	AA	AA	AG	AG	AG
	AG	AA	AA	AA	AA	AA	AA	
14837091								
10376	rs1040297		14	AC	AC	CC	AA	AC
	AA	AC	CC	CC	CC	CC	AC	CC
	AC	CC	AA	AA	AC	CC	CC	AC
	CC	CC	AC	AC	AC	CC	AC	CC
	CC	AC	AA	AA	CC	AC	CC	
22428984								
10377	rs10402974		19	AA	AA	AA	AA	AA
	AA	AA	AA	AA	AA	AA	AA	AA
	AA	AA	AA	AA	AA	AA	AA	AA
	AA	AA	AA	AA	AA	AA	AA	AA
	AA	AA	AA	AA	AA	AA	AA	
7730128								
10378	rs1040302		14	CC	CC	CC	AC	AC
	AC	CC	CC	CC	CC	CC	CC	CC
	AC	CC	CC	CC	CC	CC	CC	CC
	CC	CC	CC	CC	CC	CC	CC	CC
	CC	CC	CC	CC	CC	CC	CC	
22580623								
10379	rs10403047		19	AA	AA	AA	AA	AA
	AA	AA	AA	AA	AA	AA	AA	AA
	AA	AA	AA	AA	AA	AA	AA	AA
	AA	AA	AA	AA	AA	AA	AA	AA
	AA	AA	AA	AA	AA	AA	AA	
56065127								
10380	rs10403072		19	AG	AG	AG	AA	AA
	AA	AA	GG	GG	GG	GG	GG	GG
	GG	GG	AG	AG	GG	GG	GG	GG
	AG	GG	GG	AG	GG	AG	GG	GG
	GG	GG	AG	AG	GG	AG	GG	
31762453								
10381	rs10403164		19	AG	AA	AG	GG	GG
	AG	AG	AG	AG	GG	AG	AG	AG
	AA	AA	AG	AA	AG	AA	AA	AA

AG AA AA AG GG AG AG AG
GG AG GG GG GG AG GG
55773590
10382 rs1040319 3 CC CC CC CC CC
CC CC CC CC CC CC CC CC
CC CC CC CC CC CC CC CC
CC CC CC CC CC CC CC CC
CC AC CC AC CC AC CC
88098062
10383 rs10403191 19 AG AG AG AG AA
AG AA AA AG AG AG AG AG
AG AG AA AG AG AA AG AA
AA AA AA AA AA AG AA AA
AA AA AG AA GG AG GG
13389377
10384 rs10403210 19 AG AG AG GG GG
GG GG AA AA AA AG AA AA
GG GG AG AA AG AG AA AA
AG AG AA AG AG AG GG AG
GG AG AG AG AG AA GG
13103638
10385 rs10403272 19 GG GG GG AG GG
AG GG GG AG AG GG GG GG
GG GG AG GG AG GG GG GG
GG GG GG GG GG GG GG GG
GG GG GG GG GG AG GG
19189316
10386 rs1040328 21 AG GG AG AG AG
GG AG AA AA AG GG GG GG
GG GG AG AG AG AG GG AG
AG AA AA AA AG AG AG AG
GG AA GG AG GG GG AG
36910664
10387 rs10403316 19 GG GG GG GG AG
AG AG GG GG GG AG AA AG
AG AG GG GG GG AA AG AA
AG AG AG AG GG AA AG AG
AG AG GG GG AA AG AA
28654617
10388 rs10403331 19 GG GG GG AG AG
GG AA AG AG AG AG GG AG
GG GG GG AG GG AG AA AG
AA AA AG GG GG AG GG GG
GG GG AG AG AA AG AG
11757963
10389 rs10403340 19 AG GG GG GG AG
AG AG AA AG AG AG GG AG
AG AG AG AA AG AA AA AG
AA GG GG AA AA AG AA AA
AA GG AG GG AG AG AA

28773902							
10390	rs1040336	3	AG	AG	AG	AA	AG
	AG	AA	AA	AA	AA	AA	AA
	AA	AA	AA	AA	AA	AA	AA
	AA	AA	AA	AA	AA	AA	AA
	AA	AA	AG	AG	AG	AA	AG
60075192							
10391	rs10403369	19	AA	AA	AA	AA	AA
	AA	AA	AA	AA	AA	AA	AA
	AA	AA	AA	AA	AA	AA	AA
	AA	AA	AA	AA	AA	AA	AA
	AA	AA	AA	AA	AA	AA	AA
11781532							
10392	rs10403390	19	AA	AA	AA	AG	AA
	AG	AA	AA	AA	AA	AA	AA
	AA	AA	AG	AA	AG	AA	AG
	AG	AA	AA	AG	AA	AA	AA
	AG	AA	AA	AA	AA	AA	AA
24328633							
10393	rs10403430	19	GG	GG	AG	GG	GG
	GG	GG	GG	GG	GG	GG	GG
	GG	GG	AG	AG	GG	GG	GG
	GG	GG	GG	GG	AG	AG	AA
	--	GG	AG	AG	AG	GG	AA
21986292							
10394	rs10403495	19	GG	GG	GG	GG	GG
	GG	AG	GG	GG	GG	GG	GG
	GG	GG	AG	GG	AG	GG	GG
	GG	GG	GG	GG	GG	GG	GG
	GG	GG	GG	GG	GG	GG	GG
55065706							
10395	rs10403531	19	AA	AA	AA	AA	AA
	AA	AA	AA	AA	AA	AA	AA
	AA	AA	AA	AA	AA	AA	AA
	AA	AA	AA	AA	AA	AA	AA
	AA	AA	AA	AA	AA	AA	AA
23680857							
10396	rs10403542	19	AA	AA	AA	AG	AA
	AA	AG	GG	GG	GG	AG	AA
	AA	AA	AA	AG	AG	AG	AA
	AG	AG	AG	AA	AG	AA	AG
	AA	AA	AG	AG	AA	AA	AA
13697037							
10397	rs1040362						
21	--	--	--	--	--	--	--
	--	--	--	--	--	--	--
	--	--	--	--	--	--	--
	--	--	--	--	--	--	--
	--	--	--	--	36929531		
10398	rs10403667	19	AA	AA	AA	AA	AA

AA AA AA AA AA AA AA AA
AA AA AA AA AA AA AA AA
AA AA AA AA AA AA AA AA
AA AA AA AA AA AA AA
57727728
10399 rs10403668 19 AG AG GG GG GG
GG GG GG GG GG AG GG GG
GG GG GG GG GG AG AG AG
AG AG GG GG GG GG GG GG
GG GG GG GG GG GG GG
10686123
10400 rs10403676 19 CC CC CC CC AA
AC AC CC AC AC AC AC AA
CC CC AC AC AC AA AA AC
CC CC CC CC AA AC AC AC
CC CC AC AC AC AA CC
16419365
10401 rs10403777 19 GG GG GG GG GG
GG GG GG GG GG GG GG GG
GG GG GG GG GG GG GG GG
GG GG GG GG GG GG GG GG
GG GG GG GG GG GG GG
23724762
10402 rs10403787 19 AA AG AG AG AA
AA AG GG AG AG GG GG GG
GG GG GG GG GG AG AG AG
AG AG GG GG AG GG GG AG
GG GG AA AG GG GG GG
10166375
10403 rs10403842 19 AG AA AA AG AG
AG AG AA AA AA AA AA AA
AG AG AA AA AA AA AG AA
AG GG GG AG GG AA AG AG
AA AG AG AA AG AG GG
14857891
10404 rs10403872 19 AA AC AC AC AC
AA AC CC CC CC AC CC CC
CC CC AC AA AC AC AC CC
AA AC AC AA AC AC AA AC
CC CC CC CC CC CC AC
28621251
10405 rs10403907 19 GG GG GG AG AG
AG AG AA AG AG AG AG AA
GG GG GG GG AG GG AG GG
AA AG AG AG AG AG AG GG
AG AA GG AG GG AG GG
8989095
10406 rs10403930 19 CC CC CC CC CC
CC CC CC CC CC CC CC CC
CC CC CC CC CC CC CC CC

CC CC CC CC CC CC CC CC
CC CC CC CC CC CC CC
13193003
10407 rs1040397 1 AG AG AG GG AG
AG GG GG GG GG AG AG GG
-- GG GG -- -- GG AG GG
GG GG GG GG GG GG GG GG
GG AG GG AG GG GG GG
7952427
10408 rs10404057 19 AA AA AA AA AA
AA AA AA AA AA AA AA AA
AA AA AA AA AA AA AA AA
AA AA AA AA AA AA AA AA
AA AA AA AA AA AA AA
23095056
10409 rs1040410 6 GG GG GG GG GG
GG GG GG GG GG GG GG GG
GG GG GG GG GG AG AG AG
GG GG GG GG GG AA AG AG
GG GG GG GG AG GG AG
15547476
10410 rs10404123 19 GG GG GG GG GG
GG GG GG GG GG GG GG GG
GG GG GG GG GG GG GG GG
GG GG GG GG GG GG GG GG
GG GG GG GG GG GG GG
57051565
10411 rs1040415 X AA AA AG AA AA
AA AA AG AG AA AA AA AG
AG AG GG GG GG AG AG AA
AA GG GG AA GG AA AG GG
AA AG AA AA AG AG AA
44339785
10412 rs10404197 19 AG AG AA AG AG
GG AG GG AG AG AA AG AG
AG AG AG AA AG AG AG AA
GG AG AG GG AA AA AA AA
AG AG GG AG AG AG AG
28418794
10413 rs10404243 19 AG AG AG AA AG
AG AA AG AG AG AA AG AG
AG AG GG AG AG AA AA AA
AG AG AG AG AA AA AA AA
AG GG AG AG AA AG AG
28528961
10414 rs10404250 19 AG AG GG AA AA
AA AA AA AA AA AG AG AA
AG AG AA AA AA AA AA AA
AA AG AG AA AA AA AA AA
AG AG AA AG AG AG AA

29046301								
10415	rs10404348		19	AG	GG	GG	AG	AG
	GG	AA	GG	GG	GG	AG	AG	GG
	AA	AA	AG	AA	AA	GG	AG	GG
	AA	AA	AG	AG	AG	AG	AA	AG
	GG	AG	AG	AA	AA	AG	AA	
10840201								
10416	rs10404355		19	AA	AA	AA	AA	AA
	AA	AA	AA	AA	AA	AA	AA	AA
	AA	AA	AA	AA	AA	AA	AA	AA
	AA	AA	AA	AA	AA	AA	AA	AA
	AA	AA	AA	AA	AA	AA	AA	
12280336								
10417	rs1040436		6	GG	GG	GG	AG	GG
	AG	GG	GG	GG	GG	GG	AG	GG
	GG	GG	GG	AG	GG	GG	GG	AG
	AA	AA	AA	AG	GG	AG	AG	AG
	AG	GG	AG	AG	GG	AG	GG	
35367909								
10418	rs10404371		19	GG	GG	AG	AG	AG
	GG	AG	GG	GG	GG	GG	GG	GG
	GG	GG	AG	AG	AA	GG	GG	AG
	AG	AG	AG	AG	GG	GG	GG	GG
	AG	AG	AG	AA	GG	AG	AG	
33294082								
10419	rs10404426		19	AG	GG	GG	GG	AG
	AG	GG	GG	GG	GG	GG	AG	AG
	GG	GG	GG	GG	GG	GG	GG	GG
	AG	GG	GG	AG	GG	GG	GG	GG
	GG	GG	GG	GG	GG	GG	AG	
28472343								
10420	rs10404532		19	AA	AA	AA	AG	AG
	AG	AG	AA	AG	AG	GG	GG	GG
	GG	AG	AA	AA	AG	AG	GG	AG
	AG	GG	GG	AG	AA	AG	AG	AG
	AG	AA	AG	AG	AA	AG	AA	
57162288								
10421	rs10404538		19	GG	GG	GG	GG	GG
	GG	GG	GG	GG	GG	GG	GG	GG
	GG	GG	GG	GG	GG	GG	GG	GG
	GG	GG	GG	GG	GG	GG	GG	GG
	GG	GG	GG	GG	GG	GG	GG	
20439342								
10422	rs10404613		19	GG	GG	GG	GG	GG
	GG	GG	GG	GG	GG	GG	GG	GG
	GG	GG	GG	GG	GG	GG	GG	GG
	GG	GG	GG	GG	GG	GG	GG	GG
	GG	GG	GG	GG	GG	GG	GG	
6394910								
10423	rs10404622		19	GG	GG	AG	GG	GG

GG GG GG GG GG GG GG GG
GG GG AG AG GG GG GG GG
GG GG GG GG AG AG AA AG
GG GG AG AG AA AG AA
21934891
10424 rs10404638 19 GG GG GG GG GG
GG GG GG GG GG GG GG GG
GG GG GG GG GG GG GG GG
GG GG GG GG GG GG GG GG
GG GG GG GG GG GG GG
10533019
10425 rs10404693 19 GG GG GG GG GG
GG GG GG GG GG GG GG GG
GG GG GG GG GG GG GG GG
GG GG GG GG GG GG GG GG
GG GG GG GG GG GG GG
11576797
10426 rs10404712 19 AA AG AA AG AA
AG AA AG AG AG AG AA AG
AA AA AG AG -- AA AA AG
AG AG AA AG -- -- -- --
AA AA -- AG AA AA AG
29224672
10427 rs10404713 19 AG AG GG AG AG
AA AG AG AG AG AG AG AG
AG AG AA AA AA AG AG GG
AA AA AA AA AA AG AG AG
AA AG AA AG AA AG AA
23748279
10428 rs10404737 19 GG AG GG GG GG
GG GG GG GG GG GG AG GG
GG GG GG GG GG AG AG AG
GG GG GG GG GG GG GG GG
GG GG GG GG AG GG AG
58494665
10429 rs10404739 19 AA AA AA AA AA
AA AA AA AA AA AA AA AA
AA AA AA AA AA AA AA AA
AA AA AA AA AA AA AA AA
AA AA AA AA AA AA AA
29455816
10430 rs1040474 X AA AG AG AA AA
AA AA AA AA AA AG AG AG
AG AG AG AA AG AA AA AA
AA AA GG AG AA AA AA AA
AA AA GG AA AA AG AA
141616460
10431 rs10404783 19 GG GG GG AG AG
GG AG GG GG GG GG GG GG
GG GG GG GG GG GG GG GG

GG GG GG GG GG GG GG GG
GG GG GG GG GG GG GG
4537013
10432 rs1040481 20 AG AG AG AA AA
AA AA AG AG GG AA AA AA
AA AA GG GG AG AG AA AA
AA AA AA AA GG GG GG GG
AA AA AG AG AG AG AA
41337310
10433 rs10404810 19 AC AC AC CC CC
CC CC CC CC CC CC AC AC
CC CC CC AC AC CC CC AC
CC CC CC CC CC CC CC CC
CC AC CC AC CC CC CC
28909429
10434 rs10404839 19 GG AG GG GG GG
GG GG GG GG GG GG GG GG
GG GG GG GG GG GG GG GG
GG GG GG GG GG GG GG GG
GG GG AG AG GG GG GG
29268056
10435 rs10404882 19 GG GG GG GG GG
GG GG GG GG GG GG GG GG
GG GG GG GG GG GG GG GG
GG GG GG GG GG GG GG GG
AG GG GG GG GG GG GG
12122834
10436 rs10404947 19 AG GG GG AG AG
GG AG GG GG GG GG GG GG
GG GG AG AG GG GG GG GG
GG GG GG GG GG GG GG GG
GG GG AG GG GG GG AG
1081617
10437 rs10404954 19 AA AG AA AG AA
AG AA AA AG AG AA AA AA
AA AA AA AG AG AA AG AA
AG AG AG AG AG AA AG AA
AG AA AG AA AG AG AA
24170483
10438 rs10404975 19 GG GG GG GG GG
GG GG GG GG GG GG GG GG
GG GG GG GG GG GG GG GG
GG GG GG GG GG GG GG GG
GG GG GG GG GG GG GG
18642306
10439 rs1040499 16 CC CC CC AC AA
AA AC CC AC CC AA AA AA
CC AC AA AC AA CC AC CC
AC AC AC AC AC CC CC CC
AC AC CC CC AC CC AA

1538363								
10440	rs10405036		19	CC	CC	CC	AC	AC
	AC	CC	CC	CC	CC	AC	AC	AC
	AA	AA	AC	CC	CC	CC	AC	AC
	AC	CC	CC	AC	AC	AC	AC	AA
	CC	AC	CC	AC	CC	CC	CC	
58093044								
10441	rs1040508		1	GG	AG	AG	GG	AA
	AG	AG	AG	AG	AG	AG	AG	AG
	AG	AG	GG	AG	AG	AG	GG	GG
	AA	AA	AA	AA	GG	AA	AG	AG
	GG	AG	GG	AG	AG	AA	AG	
168500750								
10442	rs10405094		19	GG	GG	GG	GG	GG
	GG	GG	GG	GG	GG	GG	GG	GG
	GG	GG	GG	GG	GG	GG	GG	GG
	GG	GG	GG	GG	GG	GG	GG	GG
	GG	GG	GG	GG	GG	GG	GG	
31076083								
10443	rs10405199		19	GG	GG	GG	GG	GG
	GG	GG	GG	GG	GG	GG	GG	GG
	GG	GG	GG	GG	GG	GG	GG	GG
	GG	GG	GG	GG	GG	GG	GG	GG
	GG	GG	GG	GG	GG	GG	GG	
12636822								
10444	rs1040521		6	GG	AG	GG	AA	AA
	AA	AG	AA	AA	AA	AG	AG	GG
	AG	GG	AA	AG	AG	AG	AG	AG
	AG	AG	AA	AG	AA	AG	AA	AA
	GG	AA	AG	AG	GG	GG	GG	
3039609								
10445	rs10405210							
19	--	--	--	--	--	--	--	--
	--	--	--	--	--	--	--	--
	--	--	--	--	--	--	--	--
	--	--	--	--	--	--	--	--
	--	--	--	--	14994345			
10446	rs10405242		19	AA	AG	AA	GG	AG
	AG	GG	AA	AG	AG	AG	AA	AA
	AG	AG	GG	GG	GG	AA	AG	AA
	GG	AG	AG	GG	AG	AA	AG	AA
	AG	AG	GG	AG	AG	GG	AA	
24374419								
10447	rs10405246		19	AA	AG	AG	AG	AG
	AA	AG	AG	AG	AG	AA	AG	AG
	AG	AA	AG	AG	AA	AA	AA	AA
	AA	AG	AG	AA	GG	AA	AG	AG
	AA	AG	AA	AA	AG	AG	AG	
35768949								
10448	rs10405317		19	AA	AA	AA	AA	AA

AA AC AC CC AC AC AC AA
AA AA AA AA AC CC AA AC
AA AC AA AA AC AC AC CC
AC AC AC AC AA AA AC
1691725
10449 rs10405345 19 CC CC CC CC CC
CC CC CC CC CC CC CC CC
CC CC CC CC CC CC CC CC
CC CC CC CC CC CC CC CC
CC CC CC CC CC CC CC
7605271
10450 rs1040535 6 GG AG AG GG GG
GG GG AG AG GG GG GG GG
GG GG GG GG AG GG GG GG
AA AA AA AA AG GG AG AG
GG GG GG GG GG GG AG
22647683
10451 rs10405364 19 AA AA AA AA AA
AA AA AA AA AA AA AA AA
AA AA AG AA AG AA AA AA
AA AA AA AA AA AA AA AA
AA AA AA AA AA AG AA
18972008
10452 rs10405376
19 -- -- -- -- -- -- -- --
-- -- -- -- -- -- -- --
-- -- -- -- -- -- -- --
-- -- -- -- -- -- -- --
-- -- -- -- 9276166
10453 rs10405393 19 AG AA AA AA AA
AA AG AA AA AA GG GG GG
AG AA AG AG AA AG AA AA
AG AA AG AA AG AG AG GG
AA AG GG AG AG GG AG
48471422
10454 rs10405402 19 AA AA AA AA AA
AA AA AA AA AA AA AA AA
AA AA AA AA AA AA AA AA
AA AA AA AA AA AA AA AA
AA AA AA AA AA AA AA
12647642
10455 rs1040546 20 AA AA AA AA AA
AA AA AA AA AA AA AA AA
AA AA AG AA AA AA AA AG
AG AG AA AG AA AA AA AA
AG GG AA AG AA AA AA
42668388
10456 rs10405476 19 CC CC CC CC CC
CC CC CC CC CC CC CC CC
CC CC CC CC CC CC CC CC

CC CC CC CC CC CC CC CC
CC CC CC CC CC CC CC
53658883
10457 rs10405539 19 AC CC CC CC CC
CC CC AC CC CC CC CC CC
CC CC CC AC AC CC CC AC
CC CC CC CC AC AC CC AC
CC AC CC AC CC AC CC
7063372
10458 rs1040559 20 GG AG AG AA GG
AG AG AA AG AG GG GG AG
AG AG AA AA AG AG GG AA
AG GG AG GG AA AG AA AA
AG AA GG AG AG GG AG
47314445
10459 rs10405607 19 AA AA AA AA AA
AA AA AG AA AA AA AA AA
AA AA AG AA AA AA AA AA
AA AA AA AA AA AA AA AA
AA AA AG AA AG AA AG
56684824
10460 rs10405655 19 AA AA AA AA AA
AA AA AG AA AA AA AA AA
AA AA AA AA AA AA AA AA
AA AA AA AA AA GG AG AG
AA AA AA AA AA AA AA
48682779
10461 rs1040566 20 AA AA AA AA AA
AA AA AA AA AA AA AA AA
AA AA AA AA AA AA AA AA
AA AA AA AA AA AA AA AA
AA AA AA AA AA AA AC
56137687
10462 rs10405674 19 CC CC AC AC AC
CC AC AC AA AA AC AC CC
AC AC AC AC CC CC CC CC
CC AC AC CC AA CC AC AC
CC CC CC CC AC AC CC
28884610
10463 rs10405694 19 GG GG GG GG GG
GG GG GG GG GG GG AG GG
GG GG GG AG GG GG AG GG
GG AG GG GG GG GG GG GG
GG AG GG AG GG GG GG
3342306
10464 rs10405717 19 AG AG AG GG GG
AG GG GG GG GG GG AG GG
GG GG GG AG GG GG GG GG
AG GG GG AG GG AG AG AG
AG GG AA AG AG GG AG

48751808
10465 rs10405725 19 AG GG GG AG AG
AA AG AG GG GG AG GG AG
AG AG GG GG GG AA AA AG
AA GG GG AA GG AA AG AG
AG AG AA AG AG AG AA
28833272
10466 rs1040577 20 AA AC AC AC AC
CC AC AC AC AC CC CC CC
CC AC AA AA AC AA AC AA
AC AC AC AC CC AA AC AC
AA AC AA AA AA AA AA
19907294
10467 rs10405772 19 AA AA AA AA AA
AA AA AA AA AA AA AA AA
AA AA AA AA AA AA AA AA
AA AA AA AA AA AA AA AA
AA AA AA AA AA AA AA
10887626
10468 rs10405787 19 AA AA AA AA AA
AA AA AA AA AA AA AA AA
AA AA AA AA AA AA AA AA
AA AA AA AA AA AA AA AA
AA AA AA AA AA AA AA
5364606
10469 rs10405819 19 GG AG GG AG AG
GG AG GG AG AG GG GG GG
AG AG GG AG AG GG GG GG
GG GG GG GG AG GG AG GG
GG AG AG GG GG GG GG
28310061
10470 rs10405845 19 AG GG GG GG GG
GG GG AA AG AG GG GG GG
GG GG GG GG GG GG GG GG
GG GG GG GG GG GG GG GG
GG GG GG GG GG GG GG
9765366
10471 rs10405853 19 AA AA AA AA AA
AA AA AA AA AA AA AA AA
AA AA AA AA AA AA AA AA
AA AA AA AA AA AA AA AA
AA AA AA AA AA AA AA
53651893
10472 rs10405913 19 AA AA AA AA AA
AA AA AA AA AA AA AA AA
AA AA AA AA AA AA AA AA
AA AA AA AA AA AA AA AA
AA AA AG AA AA AA AA
20190141
10473 rs10405944 19 AG AG AA AG AG

	GG	AA	AA	AG	AG	AG	AG	AA
	AA	AG	AA	AG	AG	AG	AA	AG
	GG	GG	GG	GG	AG	AG	AG	AA
	AA	AA	AA	AA	AA	AG	AA	
33728001								
10474	rs10405948		19	GG	GG	GG	GG	GG
	GG	GG	GG	GG	GG	GG	GG	GG
	GG	GG	GG	GG	GG	GG	GG	GG
	GG	GG	GG	GG	GG	GG	GG	GG
	GG	GG	GG	GG	GG	GG	GG	
14074959								
10475	rs10405957		19	AG	GG	GG	AA	AA
	AA	AG	AA	AA	AA	AG	AG	AA
	AG	AG	AG	AA	AG	AG	AG	GG
	AG	AA	AA	AG	AA	AG	AG	AG
	AA	AG	GG	GG	AA	AA	AA	
23957937								
10476	rs10405958		19	GG	GG	GG	GG	GG
	GG	GG	GG	GG	GG	GG	GG	GG
	GG	GG	GG	GG	GG	GG	GG	GG
	GG	GG	GG	GG	GG	GG	GG	GG
	GG	GG	GG	GG	GG	GG	GG	
30870695								
10477	rs10405985		19	AA	AA	AA	AA	AA
	AA	AA	AA	AA	AA	AA	AA	AA
	AA	AA	AA	AA	AA	AA	AA	AA
	AA	AA	AA	AA	AA	AA	AA	AA
	AA	AA	AA	AA	AA	AA	AA	
14501626								
10478	rs10405994		19	AG	GG	GG	AG	AG
	GG	AG	AG	GG	GG	AG	GG	GG
	GG	GG	GG	GG	GG	AG	GG	AA
	GG	GG	GG	GG	GG	GG	GG	GG
	GG	GG	GG	GG	AG	GG	AG	
6097151								
10479	rs10406037		19	GG	GG	GG	GG	GG
	GG	GG	GG	GG	GG	GG	GG	GG
	GG	GG	GG	GG	GG	GG	GG	GG
	GG	GG	GG	GG	GG	--	GG	GG
	GG	GG	GG	GG	GG	GG	GG	
24353780								
10480	rs1040608		14	CC	CC	CC	AA	AC
	AC	AC	AC	AA	AC	CC	CC	CC
	CC	CC	AC	AC	AC	AC	CC	AC
	CC	AC	AC	AC	AC	CC	CC	AC
	CC	CC	CC	CC	AC	AC	AC	
97548129								
10481	rs10406103		19	GG	GG	GG	GG	GG
	AG	GG	GG	GG	GG	AG	AA	AG
	AG	GG	AG	AG	AA	AG	AA	AG

GG AG AG GG GG GG GG GG
GG GG GG GG GG GG GG
57139555
10482 rs10406118 19 AA AA AA AA AA
AA AA AA AA AA AA AA AA
AA AA AA AA AA AA AA AA
AA AA AA AA AA AA AA AA
AA AA AA AA AA AA AA
23560051
10483 rs10406121 19 GG GG GG GG GG
GG GG GG GG GG GG GG GG
GG GG GG GG GG GG GG GG
GG GG GG GG GG GG GG GG
GG GG GG GG GG GG GG
20834979
10484 rs10406142 19 GG GG GG GG GG
GG GG GG GG GG GG GG GG
GG GG GG GG GG GG GG GG
GG GG GG GG GG GG GG GG
GG GG GG GG GG GG GG
58451969
10485 rs10406172 19 AA AA AG AA AA
AA AA AA AA AA AA AA AA
AA AA AG GG AG AA AA AA
AA AA AA AA AG AG GG AG
AA AA AG AG AG AA GG
21957865
10486 rs10406190 19 GG GG GG GG GG
GG GG GG GG GG GG GG GG
GG GG GG GG GG GG GG GG
GG GG GG GG GG GG GG GG
GG GG GG GG GG GG GG
58227184
10487 rs10406213 19 AG AG GG GG AG
AG GG GG GG GG GG GG GG
GG GG GG GG GG AG GG AG
GG GG GG GG AG GG GG AG
AG AG GG GG GG GG GG
34550916
10488 rs10406292 19 AG AG AG AA AA
AA AA AA AA AA AA AG AG
AA AA AA AG AG AA AA AG
AA AA AA AA AA AA AA AA
AA AG AA AG AA AA AA
28878703
10489 rs10406324 19 AA AA AA AA AA
AG AA AA AA AA AA AA AA
AA AA AA AA AA AA AA AA
AA AA AA AA AG AA AA AG
AA AG AA AG AA AA AA

2249112
10490 rs10406326 19 AA AA AA AA AA
AA AA AA AA AA AA AA AA
AA AA AA AA AA AA AA AA
AA AA AA AA AA AA AA AA
AA AA AA AA AA AA AA
23654444
10491 rs10406347 19 GG GG GG GG GG
GG GG GG GG GG GG GG GG
GG GG GG GG GG GG GG GG
GG GG GG GG GG GG GG GG
GG GG GG GG GG GG GG
19339285
10492 rs10406382 19 AA AA AA AA AC
AA AC AA AA AA AA AA AA
AA AA AA AA AA AA AA AA
AA AA AA AA AA AA AA AA
AA AA AA AA AA AA AA
12450725
10493 rs10406393 19 AA AA AA AA AA
AA AA AA AA AA AA AA AA
AA AA AA AA AA AA AA AA
AA AA AA AA AA AA AA AA
AA AA AA AA AA AA AA
28690559
10494 rs1040644 6 CC AC AC AC AA
AC AC AC AA CC AC AC CC
CC CC AA AA AC AA AC AC
AA AA AA AA CC AC AC CC
AC AC CC AC CC CC CC
164347626
10495 rs10406453 19 GG GG GG GG GG
GG AG GG GG GG GG GG GG
GG GG AG GG GG GG GG GG
AA AG AG AA AG GG AG GG
AG AG GG AG AG GG AA
54974318
10496 rs10406458 19 CC AC AC AC AC
AC AC CC CC CC AC AC AC
CC CC AC AC AA AA AC AA
AC AC AC AC AA AA AA AA
AA AA AC AA AC AC AA
53117809
10497 rs10406462 19 CC CC CC CC CC
CC CC CC CC CC CC CC CC
CC CC CC CC CC CC CC CC
CC CC CC CC CC CC CC CC
CC CC CC CC CC CC CC
24338282
10498 rs1040649 X GG GG GG GG GG

	GG	GG	GG	AG	GG	GG	GG	AG
	GG	GG	AG	AG	AA	AG	AA	GG
	AA	AA	AA	AG	AG	AA	AG	AA
	AA	AG	AA	GG	GG	AG	GG	
135365362								
10499	rs10406494		19	AA	AA	AA	AA	AA
	AA	AA	AA	AA	AA	AA	AA	AA
	AA	AA	AA	AA	AA	AA	AA	AA
	AA	AA	AA	AA	AA	AA	AA	AA
	AA	AA	AA	AA	AA	AA	AA	
22083222								
10500	rs10406539		19	AG	AG	AG	GG	GG
	GG	GG	AG	AG	AG	GG	GG	GG
	GG	GG	GG	GG	GG	AG	AG	GG
	GG	AG	AG	GG	GG	GG	GG	GG
	GG	GG	GG	GG	GG	GG	GG	
35836770								
10501	rs10406550		19	AA	AA	AA	AA	AA
	AA	AA	AA	AA	AA	AA	AA	AA
	AA	AA	AA	AA	AA	AA	AA	AA
	AA	AA	AA	AA	AA	AA	AA	AA
	AA	AA	AA	AA	AA	AA	AA	
14280632								
10502	rs10406553		19	AC	AC	AA	CC	CC
	CC	CC	CC	AC	AC	CC	AC	CC
	AC	AC	CC	CC	CC	CC	AC	AC
	AA	CC	CC	AA	CC	CC	CC	CC
	CC	CC	CC	CC	AC	AA	CC	
22237801								
10503	rs10406557		19	AG	GG	AG	AG	GG
	GG	AG	GG	GG	GG	GG	GG	GG
	GG	GG	GG	AG	AG	GG	GG	GG
	AG	AG	AG	AG	GG	GG	GG	GG
	GG	GG	GG	GG	GG	AG	GG	
16250914								
10504	rs10406589		19	AA	AG	AG	AA	AA
	AA	AA	AA	AA	AA	AG	AG	AG
	AG	AA	AA	AA	AA	AG	AG	GG
	AA	AG	AA	AG	GG	AA	AG	AG
	AA	AG	AA	AG	GG	AG	GG	
31922887								
10505	rs10406595		19	AA	AA	AG	AG	AG
	AA	GG	AG	AG	AG	GG	AG	AG
	AG	AG	AG	GG	GG	GG	AG	AG
	AG	AG	AG	GG	AA	AA	AA	AA
	AA	AA	AG	AG	AG	AG	AA	
14319558								
10506	rs10406613		19	AG	AA	AG	AG	AG
	GG	AG	AG	AG	AG	GG	AG	--
	--	--	--	--	--	--	--	--

-- -- -- GG AG AG AA AG
GG GG AG GG AG AG --
30276911
10507 rs10406628 19 AA AA AA AG AG
AA GG AA AA AA AA AG AG
AA AA AA AA AA AA AA AA
AA AA AA AA AA AA AA AA
AA AA AA AA AA AA AA
4530261
10508 rs10406636 19 AG AG AG GG GG
AG GG AG AG AG GG AG AG
GG GG AA AA AG GG GG GG
GG GG AG GG AA GG AG AG
AG AG GG GG GG AG AG
53794858
10509 rs10406651 19 GG AG GG AG GG
AG GG AG AG AG GG GG GG
GG GG AG GG GG AG AG GG
AG AG AA GG GG GG GG GG
AG GG GG GG GG AG GG
46361607
10510 rs10406794 19 AA AG AG GG GG
GG AG GG AG AG AG AA AG
AG AG GG GG GG AA AG AG
GG GG GG GG GG GG GG GG
GG AA GG AG AA AA AG
29228056
10511 rs10406797 19 GG GG GG GG GG
GG AG GG GG GG AG GG AG
AG AG AG GG GG GG GG GG
AA AG AG AG AG AG AG GG
GG GG GG GG GG AG GG
4500205
10512 rs10406863 19 AA AA AA AA AA
AA AA AA AA AA AA AA AA
AA AA AA AA AA AA AA AA
AA AA AA AA AA AA AA AA
AA AA AA AA AA AA AA
28216031
10513 rs10406875 19 CC CC CC CC CC
CC CC AC CC CC CC CC CC
CC AC AC AC AC AC CC CC
CC AC AC CC AC CC CC AC
CC AC CC CC CC CC CC
57926486
10514 rs10406988 19 AA AA AG AG AG
AA AG AG AA AA AA AA AA
AG AG AG AG AA AA AG AA
AG AG AG AG AA AG AA AA
AA AA AG AA AG AA GG

42022444
10515 rs10407038 19 GG GG GG GG GG
GG GG GG GG GG GG GG GG
GG GG GG GG GG GG GG GG
GG GG GG GG GG GG GG GG
GG GG GG GG GG GG GG
17690582
10516 rs10407058 19 GG GG GG GG GG
GG GG GG GG GG GG GG GG
GG GG GG GG GG GG GG GG
GG GG GG GG GG GG GG GG
GG GG GG GG GG GG GG
11633206
10517 rs10407076 19 CC AC CC AA CC
AC AC AC CC CC AA AA AA
AA AA CC AC AC CC AC AC
CC AC AC CC AC AC CC AC
AA CC AC CC CC AC AC
17920758
10518 rs10407092 19 GG GG GG GG GG
GG GG GG GG GG GG GG GG
GG GG AA AG AG GG GG GG
GG GG GG GG GG GG GG GG
GG GG GG GG GG GG GG
24141092
10519 rs10407135 19 AA AA AA AA AA
AA AA AA AA AA AA AA AA
AA AA AA AA AA AA AA AA
AA AA AA AA AA AA AA AA
AA AA AA AA AA AA AA
624196
10520 rs10407185 19 AA AA AA AC AC
AA AC AC AA AA AC AA AA
AA AA AA AA AA AC AA CC
AA AA AA AA AA AA AA AA
AA AA AA AA AC AA AC
6110864
10521 rs10407220 19 AA AA AA AA AA
AA AA AA AA AA AA AA AA
AA AA AA AA AA AA AA AA
AA AA AA AA AA AA AA AA
AA AA AA AA AA AA AA
23546135
10522 rs10407229 19 AA AA AA AA AA
AA AA AA AA AA AA AA AA
AA AA AA AA AA AA AA AA
AA AA AA AA AA AA AA AA
AA AA AA AA AA AA AA
57479482
10523 rs1040731 14 AA -- AA AG AG

AA GG AG AA AG AA AA AA
AG AG GG AG AG AG AA AA
AG AG AG AG AA AA AA AA
AG AG AG GG AG AG AA
63406938
10524 rs10407327 19 AA AG AG AG GG
AG AG AG AG AG GG GG GG
GG GG AA AA AA AG AG GG
GG AA AG AG AG AG GG AG
AA AA AG AG AA AA AA
38935948
10525 rs10407348 19 AA AA AA AG AG
AA AG AG AA AA AG AA AA
AA AA AG AG AA AA AG AA
AA GG AG AG AA AG AG AG
AA AA AG AG AG AA AG
41953430
10526 rs10407397 19 GG GG AG GG GG
GG GG AG AG AG GG GG GG
GG GG GG GG GG GG AG GG
GG GG GG GG GG AG AG AG
GG AG GG AG GG GG GG
20970367
10527 rs10407424 19 AG AG GG AG AG
AG GG GG GG GG AG AG AG
AG AG AG AG GG AG GG AG
AG AG GG AG AG AG GG AG
AG GG GG GG AG AA GG
53780966
10528 rs10407469 19 GG GG GG AG GG
AG GG GG GG GG GG GG GG
GG GG GG GG GG GG GG GG
GG GG GG GG GG GG GG GG
GG GG GG GG GG GG GG
22505636
10529 rs10407513 19 AG AG AG AA AA
AA AA AA AA AA AA AA AA
AA AA AA AA AA AG AA AG
AA AA AA AA AA AA AA AA
AA AA AA AA AA AA AA
44445816
10530 rs10407538 19 AG AG AG AG GG
AG GG GG GG GG GG AG AG
GG GG GG GG GG GG GG AG
GG GG GG GG GG GG GG GG
GG AA AG AA GG GG GG
28818003
10531 rs10407667 19 GG AG AG AG AG
AG AG GG AA AA GG GG GG
GG GG GG AG AG AG AG AA

	AG	AA	GG	GG	GG	AG	AG	AG
	GG	AG	AG	AG	GG	AG	GG	
2731527								
10532	rs10407682		19	GG	GG	AG	GG	GG
	GG	GG	GG	GG	GG	AG	GG	GG
	GG	GG	AG	GG	GG	GG	GG	GG
	AG	AG	AG	AG	GG	GG	GG	GG
	AG	GG	GG	GG	GG	GG	GG	
14975040								
10533	rs10407708		19	AG	AG	AG	GG	GG
	GG	GG	GG	GG	GG	--	AG	--
	--	--	--	AG	AG	GG	--	GG
	GG	GG	GG	GG	AG	GG	GG	AG
	AG	GG	GG	GG	GG	GG	GG	
58079659								
10534	rs1040771		20	AG	AG	GG	GG	AG
	GG	AG	AA	AA	AG	GG	GG	GG
	GG	AG	AG	AG	GG	AG	AG	AG
	AG	GG	AG	GG	GG	GG	GG	GG
	AA	AG	AG	AG	GG	GG	AG	
49610797								
10535	rs10407723		19	AG	AA	AA	AA	AA
	AG	AG	AA	AA	AA	AA	AA	AA
	AA	AA	AA	AG	AA	AG	AA	AA
	AG	AG	AG	AG	AA	AA	AA	AA
	AG	AG	AA	AG	AA	AA	AA	
482984								
10536	rs10407762		19	GG	GG	GG	GG	GG
	GG	GG	GG	GG	GG	GG	GG	GG
	AG	AG	GG	GG	GG	GG	GG	GG
	AG	AG	GG	AA	GG	GG	GG	GG
	AG	AA	GG	AG	GG	GG	GG	
53288101								
10537	rs10407789		19	AA	AA	AA	AA	AA
	AA	AG	AA	AA	AA	AA	AA	AA
	AA	AA	AG	AG	AG	AG	AA	AG
	AG	AG	AA	AA	AA	AA	AA	AA
	AA	AA	AA	AA	AA	AA	AA	
1972214								
10538	rs1040782		20	AA	AA	AA	AA	AA
	AA	AA	AA	AA	AA	AA	AA	AA
	AA	AA	AA	AA	AA	AA	AA	AA
	AA	AA	AA	AA	AA	AA	AA	AA
	AA	AA	AA	AA	AA	AA	AA	
59730574								
10539	rs10407836		19	AG	AG	AG	GG	GG
	GG	GG	AG	GG	GG	AG	GG	GG
	GG	GG	GG	AG	AG	GG	GG	AG
	GG	GG	GG	GG	GG	AG	GG	GG
	GG	GG	GG	GG	AG	GG	AG	

15917010
10540 rs10407901 19 GG GG GG GG GG
GG GG GG GG GG GG GG GG
GG GG GG GG GG GG GG GG
GG GG GG GG GG GG GG GG
GG GG GG GG GG GG GG
8039726
10541 rs10407916 19 AG AG GG AA AA
AA AG AG AG AG AA AA AA
AA AA AG AG GG AA AA AA
GG AA AG GG AG AG GG AG
AG AG AG AG GG GG GG
30725134
10542 rs10407999 19 CC CC CC CC CC
CC CC CC CC CC CC AC CC
AC AA CC CC CC CC CC AC
AC AC CC AA CC AC CC CC
CC CC AC CC CC AC CC
42192835
10543 rs10408006 19 GG GG GG GG GG
GG GG GG GG GG GG GG GG
GG GG GG GG GG GG GG GG
GG GG GG GG GG GG GG GG
GG GG GG GG GG GG GG
9564982
10544 rs1040806 6 AC AA AC AA AA
AA AA CC AC CC AA AA AA
AA AA AA AA AA AA AA AA
AA AA AA AA AA AA AA AA
AA AA AA AA AA AC AA
135176176
10545 rs10408153 19 AA AA AA AG GG
AA -- AG GG AA AA GG AA
AA AA AA AA AA AG AG AG
AA AA AA AA AA AA AA AA
AA AA AA AA AA AA AA
11858484
10546 rs10408164 19 AG GG GG GG GG
GG GG GG GG GG GG GG GG
GG GG AG AG AA GG GG AG
AA AG AG AG GG GG GG GG
GG GG GG GG GG AG AG
419407
10547 rs10408179 19 GG AG AG GG AG
AG AG AG GG GG AG AG AA
AA AG AG AG AG GG AA GG
AG AG AA GG AG AA AA AG
AG AG AA AG AG AG AG
46157004
10548 rs10408191 19 CC CC CC AC AC

CC AC CC CC CC CC CC CC
CC CC CC CC CC CC CC CC
CC CC CC CC CC CC CC CC
CC CC CC CC CC CC CC
2358775
10549 rs10408195 19 GG AG GG AA AG
AG AA GG AG AG AG GG GG
GG GG GG AG AG GG AG GG
AA AA AA AA AG GG AG GG
AG GG AA AG AG AA GG
24165702
10550 rs1040822 6 AC AC CC AA AC
AC AA AA AC AA AC CC CC
AC AC AC AC AC AA AA AC
AA AA AC AC AA AC AC AA
AC AC AA AC AA AA AA
154518638
10551 rs10408252 19 AG GG GG GG GG
GG GG GG GG GG AG AA AG
AA AA AG AG GG GG AG AG
AA AG AG AA AG AA AG AA
AG AG AG GG AG AA GG
35002775
10552 rs10408272 19 GG GG GG GG GG
GG GG GG GG GG GG GG GG
GG GG GG GG GG GG GG GG
GG GG GG GG GG GG GG GG
GG GG GG GG GG GG GG
56962005
10553 rs10408278 19 GG GG GG GG GG
GG GG GG GG GG GG GG GG
GG GG GG GG GG GG GG GG
GG GG GG GG GG GG GG GG
GG GG GG GG GG GG GG
30887910
10554 rs10408331 19 AG GG GG AG AG
AA AG AG GG GG GG AG AG
GG GG AG GG AG GG AG GG
AA AG AG AA GG GG GG GG
AA GG AG AG GG AG AG
28221474
10555 rs10408355 19 AC CC CC AC AC
CC AC AC CC CC CC CC CC
CC CC AC CC AC CC CC CC
CC AA AC AC AC CC CC AC
AA CC CC CC CC AC CC
33005903
10556 rs10408377 19 GG GG GG GG GG
GG GG GG GG GG GG GG GG
GG GG GG GG GG GG GG GG

GG GG GG GG GG GG GG GG
GG GG GG GG GG GG GG
638399
10557 rs10408380 19 AG AG AG AG AG
GG AG GG GG GG GG GG GG
GG GG GG GG GG AG AG GG
AG AG AA GG GG GG GG GG
AG GG AG GG GG GG GG
46414628
10558 rs10408395 19 AA AA AA AA AA
AA AA AA AA AA AA AA AA
AA AA AA AA AA AC AC AA
AC AA AA AA AA AA AA AA
AA AA AA AA AA AA AA
19299535
10559 rs10408621 19 AG AG AG GG GG
GG GG GG GG GG GG GG GG
AG AG GG GG GG GG AA GG
GG AG AG GG AG GG GG AG
GG GG AA AG AG AG AG
40061350
10560 rs10408643 19 AA AA AA AA AA
AA AA AA AA AA AA AA AA
AA AA AA AA AA AA AA AA
AA AA AA AA AA AA AA AA
AA AA AA AA AA AA AA
23533528
10561 rs10408649 19 GG GG AG AG AG
GG AG GG AG AG AG AG GG
AG AG GG GG GG GG GG GG
GG AG AG GG AG GG AG GG
GG GG GG GG AG AG GG
28896872
10562 rs10408676 19 GG GG GG AG GG
GG AG GG GG GG GG GG GG
GG GG GG GG GG GG GG GG
GG GG GG GG GG GG GG GG
GG GG GG GG GG GG GG
15290007
10563 rs10408765 19 GG GG GG GG GG
GG GG GG GG GG GG GG GG
GG GG GG GG GG GG GG GG
GG GG GG GG GG GG GG GG
GG GG GG GG GG GG GG
28659624
10564 rs10408771 19 CC CC CC CC CC
CC CC CC CC CC CC CC CC
CC CC CC CC CC CC CC CC
CC CC CC CC CC CC CC CC
CC CC CC CC CC CC CC

12184785							
10565	rs10408793	19	AG	AG	AG	AA	AG
	AG	AA	AA	AA	AA	AA	AA	AA
	AA	AA	AG	AG	AA	AA	AA	AA
	AG	AA	AA	AG	AA	AA	AA	AA
	AG	AA	AG	AA	AG	AG	AG	
23079898							
10566	rs10408846	19	AG	AA	AG	AG	AG
	AA	GG	GG	GG	GG	AA	AG	AG
	AA	AA	AG	AG	GG	AG	AG	AG
	AA	AA	AA	AA	AA	AG	AG	AG
	GG	AG	AA	AG	AG	GG	AG	
30817913							
10567	rs10408852	19	GG	GG	GG	GG	GG
	GG	GG	GG	GG	GG	GG	GG	GG
	GG	GG	GG	GG	GG	GG	GG	GG
	GG	GG	GG	GG	GG	GG	GG	GG
	GG	GG	GG	GG	GG	GG	GG	
23769373							
10568	rs10408984	19	GG	AG	GG	AA	AG
	AA	AG	GG	GG	GG	GG	AG	AG
	GG	GG	GG	GG	GG	AG	GG	AG
	AG	AG	AG	GG	GG	GG	GG	GG
	GG	AG	AG	GG	GG	GG	AG	
15298270							
10569	rs10409034	19	AA	AA	AA	AA	AA
	AA	AA	AA	AA	AA	AA	AA	AA
	AA	AA	AA	AA	AA	AA	AA	AA
	AA	AA	AA	AA	AA	AA	AA	AA
	AA	AA	AA	AA	AA	AA	AA	
58389803							
10570	rs10409036	19	GG	GG	GG	GG	GG
	GG	GG	GG	GG	GG	GG	GG	GG
	GG	GG	GG	GG	GG	GG	GG	GG
	GG	GG	GG	GG	GG	GG	GG	GG
	GG	GG	GG	GG	GG	GG	GG	
7097489							
10571	rs10409042	19	AG	GG	GG	GG	GG
	GG	GG	GG	GG	GG	GG	GG	GG
	GG	GG	GG	GG	GG	GG	GG	GG
	GG	AG	GG	GG	GG	GG	GG	GG
	GG	AG	GG	GG	GG	GG	GG	
3361995							
10572	rs10409055	19	GG	GG	GG	GG	GG
	GG	GG	GG	GG	GG	GG	GG	GG
	GG	GG	GG	GG	GG	GG	GG	GG
	GG	GG	GG	GG	GG	GG	GG	GG
	GG	GG	GG	GG	GG	GG	GG	
8220233							
10573	rs1040910	9	GG	GG	GG	GG	AG

```
        AG      GG      AG      AG      GG      GG      AA      AG
        AG      AG      GG      GG      GG      AG      AA      GG
        GG      GG      GG      AG      GG      AG      GG      AG
        GG      AG      AG      GG      GG      GG      GG
10637778
10574   rs10409100      19      AA      AA      AA      AA      AA
        AA      AA      AA      AA      AA      AA      AA      AA
        AA      AA      AA      AA      AA      AA      AA      AA
        AA      AA      AA      AA      AA      AA      AA      AA
        AA      AA      AA      AA      AA      AA      AA
11050590
10575   rs10409120      19      AA      AA      AG      AG      AG
        AA      AG      AG      AG      AG      AA      AA      AA
        AA      AA      AA      AA      AG      AA      AA      AA
        AA      AA      AA      AG      AG      AA      AA      AG
        GG      AG      AA      AG      AA      AA      AA
53114267
10576   rs10409145      19      AA      AA      AA      AA      AA
        AG      AA      AA      AG      AG      AG      AG      AG
        AA      AG      AA      AG      AA      AG      AG      GG
        AG      AG      AG      AG      GG      AA      AG      AG
        GG      AA      AA      AA      GG      AG      AG
57671661
10577   rs10409179      19      GG      GG      GG      AG      AG
        GG      AG      AG      AG      AG      AG      AA      AG
        AG      AG      AG      GG      AG      AG      AG      GG
        GG      GG      GG      GG      AA      AG      AA      AA
        GG      GG      GG      GG      AG      AG      GG
32608345
10578   rs10409187      19      AG      AA      AG      AG      AG
        GG      AA      AG      AA      AA      GG      GG      GG
        GG      GG      AG      AG      AG      GG      GG      GG
        AG      AG      AG      AG      GG      AA      AG      AG
        AG      GG      AA      AG      GG      AG      GG
54075317
10579   rs10409209      19      AA      AA      AC      AA      AA
        AA      AC      AA      AC      AC      CC      AC      AC
        AC      AC      AA      AA      AA      AC      AC      AC
        CC      AC      AC      AC      AA      CC      AC      AC
        AA      AC      AA      AA      CC      CC      AC
6145564
10580   rs10409224      19      AG      AG      AA      AA      AG
        AG      AA      AG      AG      AG      AA      AA      AA
        AG      AG      AA      AA      AA      GG      AG      GG
        AA      AA      AA      AA      AG      AG      AA      AG
        AG      GG      AG      GG      AG      AG      GG
34535507
10581   rs10409248
19      --      --      --      --      --      --      --      --
        --      --      --      --      --      --      --      --
```

-- -- -- -- -- -- -- --
-- -- -- -- -- -- -- --
-- -- -- -- 28531174
10582 rs10409280 19 AG AG AG AG AA
AG AG GG AG AG AG GG AG
GG GG AG AA AG AA GG AA
AG GG GG AG GG AG AG AG
GG AA AA AA AG AG AG
39546960
10583 rs10409289 19 GG AG GG GG AG
AG GG GG AG AG AG AG AG
GG GG AG AG GG GG GG GG
AG AG AG AG GG GG GG GG
GG AG GG AG AG AG GG
20806824
10584 rs1040930 6 CC CC CC CC CC
CC CC CC CC CC CC CC CC
CC CC CC CC CC CC CC CC
CC CC CC CC CC CC CC CC
CC CC CC CC CC AC AC
124435941
10585 rs10409301 19 GG GG GG GG GG
GG GG GG GG GG GG GG GG
GG GG GG GG GG GG GG GG
GG GG GG GG GG GG GG GG
AA GG GG GG GG GG GG
4056346
10586 rs10409339 19 GG GG GG AG GG
AG GG GG GG GG AG GG GG
GG GG GG GG GG GG GG GG
GG GG GG GG AG GG GG GG
GG GG GG GG GG GG GG
16655808
10587 rs1040936 10 AA AG GG AA AA
AA AA AA AA AA AA AA AA
AA AA AA AA AA AA AA AA
AA AA AA AA AA AA AA AA
AA AG AG AG AA AA AA
78084407
10588 rs10409375 19 AG AG AG AG GG
GG AG AG GG GG AG GG GG
AG AG AG AG AG AA AA AG
GG AG GG GG AG AA AG AA
AG AA GG AG GG AG GG
9208186
10589 rs1040939 6 AG GG AG GG GG
GG GG GG GG GG GG GG GG
GG AG GG GG GG GG GG GG
GG GG GG GG GG GG GG GG
GG GG GG GG GG GG GG

44513341
10590 rs10409398 19 GG GG GG GG GG
GG GG GG GG GG GG GG GG
GG GG GG GG GG GG GG GG
GG GG GG GG GG GG GG GG
GG GG GG GG GG GG GG
20949627
10591 rs10409409 19 AA AA AA AA AA
AA AA AA AA AA AA AA AA
AA AA GG AG AG AG AA AA
AA AA AA AA AG AG AA AG
AA AA GG AG AG AA AG
58215467
10592 rs10409452 19 AA AG GG AA AA
AA AA AG AG AG GG AG AG
GG GG GG GG AG AG AA AG
AG GG AA AA AA AA AA AA
AA GG AA AG AA AA AA
288246
10593 rs10409463 19 AG GG AG AA AA
AA AA AG AG AG AA AG AA
AG AG AG GG GG AG AG AG
GG GG AG AA AG AA AG AA
AG GG AG GG AG AA AG
13632383
10594 rs10409487 19 AA AA AA AG AG
AA AG AA AA AA AA AA AA
AA AA AA AA AA AA AA AA
AG AA AG AA AA AA AA AA
AA AA AG AA AG AA AG
38285111
10595 rs10409516 19 AC AC CC CC AC
CC AC CC CC CC CC AC AC
CC CC CC CC CC CC AC CC
CC CC CC CC CC CC CC CC
CC CC AC AC CC CC AC
7281212
10596 rs10409518 19 GG AG GG GG GG
GG GG GG GG GG GG GG GG
GG GG GG GG GG GG GG AG
GG GG GG GG GG GG GG GG
GG GG GG GG GG GG AG
16606070
10597 rs10409520 19 GG GG AG AA AA
AA AG AG AA AA GG AA AG
AA AA AG AA AG AA AA AA
AA AA AA AA AA AG AG AG
AG AG AG GG AA AA AG
22512477
10598 rs10409531 19 AG AG AG AG AG

	GG	AG	GG	GG	GG	AG	GG	AG
	GG	AG	GG	AG	GG	AA	AG	AA
	GG	AG	AG	GG	AA	GG	AG	AG
	AG	AA	GG	AG	AA	AA	AA	
57066013								
10599	rs10409535		19	AC	CC	CC	AC	AC
	CC	AC	AC	CC	CC	AC	CC	CC
	CC	CC	CC	CC	CC	AC	CC	AA
	CC	CC	CC	CC	CC	CC	CC	CC
	CC	CC	CC	CC	AC	CC	AC	
6106463								
10600	rs1040954		6	AG	GG	AG	GG	AA
	AG	AG	GG	AG	GG	AA	AA	AG
	AA	AG	AG	AG	AG	AA	AA	AA
	AG	AG	AG	AG	AG	AA	AA	AA
	GG	GG	AA	AG	AG	AG	AA	
120413143								
10601	rs10409568		19	AA	AA	AA	AA	AA
	AA	AA	AA	AA	AA	AA	AA	AA
	AA	AA	AA	AA	AA	AA	AA	AA
	AA	AA	AA	AA	AA	AA	AA	AA
	AA	AA	AA	AA	AA	AA	AA	
19911014								
10602	rs10409575		19	CC	CC	CC	CC	CC
	CC	CC	CC	CC	CC	CC	CC	CC
	CC	CC	CC	CC	CC	CC	CC	CC
	CC	CC	CC	CC	CC	CC	CC	CC
	CC	CC	CC	CC	CC	CC	CC	
24167504								
10603	rs10409627		19	GG	GG	GG	GG	AG
	GG	AG	GG	GG	GG	GG	GG	GG
	GG	GG	GG	GG	GG	GG	GG	AG
	GG	GG	GG	GG	AG	GG	AG	GG
	GG	GG	GG	GG	GG	GG	GG	
8373778								
10604	rs10409717		19	AG	GG	GG	GG	AG
	AG	AG	AA	AG	AG	AA	AG	AG
	AA	AA	AG	AA	AG	AA	AA	AG
	AA	GG	GG	AA	AA	AA	AA	AA
	AA	GG	AG	GG	AA	AA	AA	
28787800								
10605	rs1040974		20	AG	AA	AG	AA	AG
	AG	AA	AA	AA	AA	AA	AG	AA
	AA	AA	AA	AA	AA	AG	AG	GG
	AA	AA	AG	AG	AG	AG	AG	GG
	AG	AA	AA	AA	AA	AA	AA	
1268250								
10606	rs10409769		19	GG	GG	GG	GG	GG
	GG	GG	GG	GG	GG	GG	AG	GG
	GG	AG	AG	AG	AG	GG	GG	GG

GG GG GG GG GG AG AG AG
AG GG GG GG GG GG GG
45022672
10607 rs10409794 19 AA AA AA AA AA
AA AA AA AA AA AA AA AA
AA AA AA AA AA AA AA AA
AA AA AA AA AA AA AA AA
AA AA AA AA AA AA AA
31539263
10608 rs10409801
19 -- -- -- -- -- -- -- --
-- -- -- -- -- -- -- --
-- -- -- -- -- -- -- --
-- -- -- -- -- -- -- --
-- -- -- -- 17407415
10609 rs10409851 19 AA AA AA AG AG
AG AG AA GG GG AA AA AA
AA AA AA AG AG AG AG GG
AA AG AA AA AA AG AG AG
AA AG AG AG AA AG AA
2726199
10610 rs10409854 19 GG GG GG GG GG
GG GG GG GG GG GG GG GG
GG GG GG GG GG GG GG GG
GG GG GG GG GG GG GG GG
GG GG GG GG GG GG GG
1756939
10611 rs10409865 19 AA AA AA AA AA
AA AA AA AA AA AA AA AA
AA AA AA AA AA AA AA AA
AA AA AA AA AA AA AA AA
AA AA AA AA AA AA AA
21271647
10612 rs1040992 6 CC AC AC CC CC
CC CC CC CC CC CC CC CC
CC CC CC CC CC CC CC CC
CC AC CC AC AC CC AC CC
AC CC CC CC CC AC CC
12196910
10613 rs10409978 19 GG GG GG AG AG
AG GG GG GG GG AA AG AA
AG GG GG GG GG AG GG GG
GG GG GG GG GG GG GG GG
AG AG GG AG GG GG GG
57796678
10614 rs10410018 19 AG AG AG AA AA
AA AA GG GG GG GG GG GG
GG GG AG AG GG GG GG GG
AG GG GG AG GG AG GG GG
GG GG AG AG GG AG GG

31754719								
10615	rs1041004		14	AA	AA	AG	AG	AG
	GG	AA	GG	AG	GG	GG	AG	GG
	GG	GG	AA	AG	AG	AG	AG	AG
	AG	GG	AG	AG	AG	AG	GG	AA
	AA	AA	AA	AA	AA	AA	AG	
86216993								
10616	rs10410062							
19	--	--	--	--	--	--	--	--
	--	--	--	--	--	--	--	--
	--	--	--	--	--	--	--	--
	--	--	--	--	--	--	--	--
	--	--	--	--	43646784			
10617	rs10410070		19	AG	GG	GG	GG	GG
	GG	GG	AG	AG	AG	GG	AG	GG
	AG	AA	AA	AA	AA	GG	GG	GG
	AG	GG	GG	AG	GG	GG	GG	GG
	AA	GG	GG	GG	GG	GG	GG	
31830613								
10618	rs10410090		19	AC	AC	AC	CC	CC
	CC	CC	CC	CC	CC	CC	AC	AC
	CC	CC	CC	CC	CC	CC	CC	CC
	AC	CC	CC	AC	CC	CC	CC	CC
	CC	CC	CC	CC	CC	AC	CC	
23389542								
10619	rs10410124		19	AG	GG	GG	GG	GG
	GG	GG	AA	AG	AG	GG	GG	GG
	GG	GG	GG	GG	GG	GG	GG	GG
	GG	GG	GG	GG	GG	GG	GG	GG
	GG	GG	GG	GG	GG	GG	GG	
9841875								
10620	rs10410156		19	GG	AG	GG	GG	GG
	GG	GG	GG	GG	GG	AG	AG	AG
	AG	GG	GG	GG	AG	GG	GG	GG
	AG	AG	AG	AG	GG	GG	GG	GG
	AG	GG	GG	GG	GG	GG	GG	
53005007								
10621	rs10410179		19	GG	GG	GG	GG	GG
	GG	GG	GG	GG	GG	GG	GG	GG
	GG	GG	GG	GG	GG	GG	GG	GG
	GG	GG	GG	GG	GG	GG	GG	GG
	GG	GG	GG	GG	GG	GG	GG	
5914537								
10622	rs10410235		19	GG	GG	GG	GG	GG
	GG	GG	GG	GG	GG	GG	GG	GG
	GG	GG	GG	GG	GG	GG	GG	GG
	GG	GG	GG	GG	GG	GG	GG	GG
	GG	GG	GG	GG	GG	GG	GG	
28797575								
10623	rs10410286		19	AA	AG	AG	AG	AG

AA AG GG GG GG AG GG GG
GG GG AG AA AG AG AG GG
AA AG AG AA AG AG AA AG
GG GG GG GG GG GG AG
28597615
10624 rs10410306 19 AG AG AG GG GG
GG GG GG GG GG GG GG GG
GG GG AG AA AA GG AG GG
GG GG GG GG GG GG GG GG
AG GG AG AG GG AG GG
44601537
10625 rs1041039 9 AG AG AG GG AG
GG AG GG GG GG GG AG AG
AG AG GG GG GG AA AA AG
GG GG GG GG GG AG GG GG
AG GG AG AG GG AG AG
83462500
10626 rs10410459 19 GG GG GG GG GG
GG GG GG GG GG GG GG GG
GG GG GG GG GG GG GG GG
GG GG GG GG GG GG GG GG
GG GG GG GG GG GG GG
7928462
10627 rs10410479 19 GG GG GG GG GG
GG GG GG GG GG AG GG AG
AG AG GG GG GG AG GG AG
AG AG AG AG GG GG GG GG
GG GG AG GG GG AG GG
1917925
10628 rs10410487 19 GG GG AG AG AG
AG AG GG AG AG GG AA AG
AG AG GG AG AG AG AG GG
AG GG GG AG GG AA AG AG
GG AA AG AG AA AG AA
17829608
10629 rs10410493 19 AA AA AA AA AA
AA AA AA AA AA AA AA AA
AA AA AA AA AA AA AA AA
AA AA AA AA AA AA AA AA
AA AA AA AA AA AA AA
28466721
10630 rs10410495 19 GG GG GG GG GG
GG GG GG GG GG GG GG GG
GG GG GG GG GG GG GG GG
GG GG GG GG GG GG GG GG
GG GG GG GG GG GG GG
28245112
10631 rs10410515 19 AG AG AA AA GG
AG AG AG GG GG AA AA AA
AG AG AG GG GG AG AA AA

AA AG AG GG AG GG GG GG
AG AG AG AG GG GG AG
15953243
10632 rs10410525 19 AA AA AA AA AA
AA AA AA AA AA AC AA AC
AA AA AA AA AA AA AA AA
AA AA AA AA AA AA AA AA
AA AC AA AA AA AA AA
30042913
10633 rs1041053 13 AC AA AC CC CC
CC CC AC CC CC AC AC AC
AA AA AC AA AA AC AC AA
AA CC AA AA CC AC AC CC
AC CC AC AC CC CC AC
43920860
10634 rs10410539 19 GG GG GG AA AA
AA AG GG GG GG GG GG GG
GG GG GG GG GG GG GG GG
GG GG GG GG AA GG AG AG
GG AG GG AG GG GG AG
2917612
10635 rs10410545 19 AG GG GG AG AG
AG GG AG AG AG AA AA AA
AG GG GG AG GG AA AG AG
AG GG GG AG AG GG AG GG
AG AG AG GG GG GG GG
57790971
10636 rs10410546 19 GG GG GG GG AG
AG AG AA AA AA AG AG AG
GG GG AG AA AG AA AG AA
AG AA AA AG AA AA AA AA
AG AG AG AA AG AA AG
20482817
10637 rs10410581 19 CC CC CC CC CC
AC CC CC CC CC CC CC CC
CC CC CC CC CC CC CC AC
AC CC CC AC AC AC AA AC
CC AC CC AC CC CC CC
54297234
10638 rs10410611 19 GG GG GG GG GG
GG GG GG GG GG GG GG GG
GG GG GG GG GG GG GG GG
GG GG GG GG GG GG GG GG
GG GG GG GG GG GG GG
23962168
10639 rs1041063 1 AG AG AG AG GG
AG GG AG AG AG GG GG GG
AG AG GG GG GG GG GG AG
GG AG AG AG GG GG GG GG
AG GG GG GG GG GG AG

191023397
10640 rs1041070 13 AG AG AA AG GG
AG GG AG AG AG GG GG GG
AG AG AG AG AG AG AG AA
GG GG GG GG AA AG AA AG
AG AG GG AG AG GG AA
48505493
10641 rs10410702 19 AG AG AG AG AA
AG AA AG AG AG AA AA AA
AA AA AA AA AA AG AA AG
AG GG AA AA AA AA AA AA
AA AG AA AG AG AA GG
4622380
10642 rs10410806 19 GG GG GG GG GG
GG GG GG GG GG GG GG GG
GG GG GG GG GG GG GG GG
GG GG GG GG GG GG GG GG
GG GG GG GG GG GG GG
53887192
10643 rs1041082 13 GG AG GG GG GG
GG GG AG GG AG GG AG GG
GG GG GG GG GG GG GG GG
AG AG AG AG GG AG GG AG
AG AA GG AG GG AG GG
71568719
10644 rs10410885
19 -- -- -- -- -- -- -- --
-- -- -- -- -- -- -- --
-- -- -- -- -- -- -- --
-- -- -- -- -- -- -- --
-- -- -- -- 10143082
10645 rs10410886 19 GG GG GG GG GG
GG GG GG GG GG GG GG GG
GG GG AG AG AG GG GG GG
GG GG GG GG GG GG GG GG
GG GG GG GG GG GG GG
56209018
10646 rs10410943 19 AG AG AG AG AG
AG AG GG GG GG AG GG GG
GG GG AG GG GG AG AG AG
GG GG GG GG AG AA AG AA
AA GG AA AG AG AG AG
8808900
10647 rs10410996 19 AA AA AA AG AG
AA AG AA AA AA AA AA AA
AA AA AA AA AA AA AA AA
AA AA AA AA AA AA AA AA
AA AA AA AA AA AA AA
5358628
10648 rs10411009 19 GG AG AG GG AG

AG GG AG GG GG AA GG AG
AG AG AA AA AA GG AG AG
AG AA AA AA AA AG AA AA
AG AG AA AA AG AG AA
18606266
10649 rs10411019 19 AG AG AG AG AG
AA AG AG GG GG AA AG AA
AG GG AG AG AG AG GG AG
GG GG GG GG AG GG GG AG
AG AG GG AG AG AG AG
58516230
10650 rs1041102 9 AG AG AA AA AA
AA AA AG AG AG AA AG AG
AG AG AA AA AA AG AA AA
AG AA AG AA AG AG GG AA
AA AA AG AG AG AA GG
12513587
10651 rs1041111 1 AG AG AG AG GG
AG GG AA AG AA AA AG AG
AA AA AG AA AG AA AA AG
AG AA AA AA AG AG AG GG
GG AG AA AG AA AA AG
236154012
10652 rs10411141 19 AA AA AA AG AG
AA GG GG AG AG AA AG AG
AG AG GG AG GG AA AG AG
AG AA AG AG AG AG GG AG
AG AA AA AA AG AG AA
9536203
10653 rs10411195 19 AA AA AA AA AA
AA AA AA AA AA AA AA AA
AA AA AA AA AA AA AA AA
AA AA AA AA AA AA AA AA
AA AA AA AA AG AG AA
20036176
10654 rs10411220 19 GG GG GG GG GG
GG AG AG GG GG GG AG AG
AA AG GG AG AG GG GG AG
GG AG AG GG GG AG GG GG
GG GG AG GG AG AA GG
32675407
10655 rs10411311 19 AG GG AG GG GG
GG GG GG GG GG AG AG AA
AG AG AA AG AA GG GG GG
AG GG AG AG AG GG GG AG
GG AA AA AA GG AG AG
4735663
10656 rs10411325 19 GG GG GG GG GG
GG GG GG GG GG GG GG GG
GG GG GG GG GG GG GG GG

GG GG GG GG GG GG GG GG
GG GG GG GG GG GG GG
28316179
10657 rs10411344 19 AA AA AA AC AA
AA AC AA AA AA AC AA AC
AA AA AA AA AA AA AA AA
AA AA AA AA AA AA AA AA
AC AA AA AA AA AA AA
16520403
10658 rs10411374 19 AA AA AA AC AC
AC AC -- AC AC AC AC --
AA AA AA AA AC AA AC AA
-- AC AC AC AC AC AC AA
AC -- AA AC AA AC AA
8985957
10659 rs10411394 19 GG GG AG GG GG
GG GG AG GG GG AA GG AG
AG AG GG AG GG AG AA AG
GG AG GG GG GG AA AG AG
AG AA AG AA AG AG AG
4325327
10660 rs10411412 19 GG GG GG GG GG
GG GG GG GG GG GG GG GG
GG GG GG GG GG GG GG GG
GG GG GG GG GG GG GG GG
GG GG GG GG GG GG GG
23405796
10661 rs10411423 19 GG AG AG AG AG
AA GG AG AG AG AG GG AG
GG GG AA AG AG AG AG GG
GG GG GG AG AG AG GG AG
AA AG AG GG AG AG GG
13603320
10662 rs10411434 19 GG GG GG GG GG
GG GG GG GG GG GG GG GG
AG AG GG GG GG GG GG GG
GG GG GG GG GG GG GG GG
GG GG GG GG AG GG AG
48310542
10663 rs1041148 6 CC AC AC AA AA
AA AC AC AA AC AA AC AC
CC AA AC AA AA AA AA AC
AA AA AA AA AC AA AA AC
AA AA AA AA AA AA AA
53853589
10664 rs10411526 19 -- -- -- -- AG
AA AG AG AG AG GG AA AG
AG AG -- AG AG AG -- AG
-- -- AG AG AG -- AG --
AG AG -- GG -- -- AG

8172681
10665 rs10411539 19 AA AA AA AA AA
AA AC AA AA AA CC AA AC
AA AA AC AC AA AA AA AA
AA AC AC AA AC AA AC AA
AA AC AA AC AA AA AA
29972230
10666 rs10411547 19 AC AC AA AC AC
AA AC AA AA AA AA AA AA
AA AA AA AA AA AA AA AA
AC CC AA AA AA AA AA AA
AA AA AC AA AC AC AA
2617422
10667 rs10411606 19 AA AA AA AA AA
AA AA AG AA AA AA AA AA
AA AA AA AA AA AA AA AA
AA AA AA AA AA AA AA AA
AA AA AG AG AA AA AA
11173129
10668 rs10411617 19 GG GG GG GG GG
GG GG GG GG GG GG GG GG
GG GG GG GG GG GG GG GG
GG GG GG GG GG GG GG GG
GG GG GG GG GG GG GG
13084626
10669 rs10411640 19 AA AA AA AA AA
AG AA AA AA AA AA AA AA
AA AA AA AG AA AA AA AA
AA AA AA AA AA AA AA AA
AA AG AA AA AA AA AA
56105161
10670 rs10411642
19 -- -- -- -- -- -- -- --
-- -- -- -- -- -- -- --
-- -- -- -- -- -- -- --
-- -- -- -- -- -- -- --
-- -- -- -- 53917508
10671 rs10411676 19 AA AA AA AG AA
AG AA AA AG AG AG AG AA
AA AA GG AG GG AA AA AA
AG AA AG AG AA AA AA AA
GG AG AA AG AA AG AA
7273991
10672 rs1041174 9 AC AA AC AA AA
AA AA AA AA AA AA AA AA
AA AA AA AA AA AC AA AA
AC AA AC AC AA AA AA AA
AA AA AA AA AC AA CC
12763001
10673 rs10411749 19 CC CC AC AA AC

	AC	AA	AC	AC	AC	CC	CC	CC
	CC	CC	AC	AC	AA	AC	AA	AA
	AC	CC	CC	AC	AA	AC	AC	AC
	AA	AC	AC	AC	AA	AC	AC	
30105969								
10674	rs1041177		9	AA	AA	AA	AA	AA
	AA	AA	AG	AG	AA	AA	AG	AG
	GG	GG	AA	AA	AA	AA	AA	AA
	AG	AA	AG	AA	AG	AA	AA	AG
	AG	AA	AG	AA	AG	AA	AG	
12795698								
10675	rs10411772		19	AC	AC	AC	AA	AA
	AA	AA	AA	AA	AA	AA	AA	AA
	AA	AA	AA	AA	AA	AC	AC	AA
	AA	AA	AA	AA	AA	AA	AA	AA
	AA	AA	AA	AA	AA	AA	AA	
54048951								
10676	rs10411776		19	AA	AG	AA	AG	AA
	AG	AA	AA	AG	AG	AA	AA	AA
	AA	AA	AA	AG	AG	AA	AG	AA
	AG	AG	AG	AG	AG	AA	AG	AA
	AG	AA	AG	AA	AG	AG	AA	
24176399								
10677	rs10411861		19	AA	AA	AA	AA	AA
	AA	AA	AA	AA	AA	AA	AA	AA
	AA	AA	AA	AA	AA	AA	AA	AA
	AA	AA	AA	AA	AA	AA	AA	AA
	AA	AA	AA	AA	AA	AA	AA	
24177255								
10678	rs10411875		19	AC	AC	CC	CC	AC
	CC	AC	AA	AC	AC	AC	AC	AC
	AC	AC	AC	CC	CC	CC	AC	AC
	AC	AC	AC	CC	CC	AC	CC	CC
	AC	AC	AC	AC	AC	CC	AC	
14367138								
10679	rs10411891		19	GG	GG	GG	AG	GG
	AG	GG	GG	GG	GG	GG	GG	GG
	GG	GG	--	AG	AG	GG	AG	GG
	AG	GG	GG	AG	GG	GG	GG	GG
	AG	GG	GG	GG	GG	GG	GG	
24368534								
10680	rs10411931		19	AA	AA	AA	AG	AA
	AG	AA	AA	AA	AA	AG	AA	AG
	AA	AA	AA	AA	AA	AA	AA	AA
	GG	AA	AG	AG	AA	AA	AA	AA
	AA	AA	AG	AA	AG	AG	AA	
37442926								
10681	rs10411961		19	GG	AG	GG	AG	AG
	AG	AG	AA	AA	AA	AA	AA	AA
	AG	GG	AG	AG	AG	GG	AA	AG

	AA	AA	AA	AA	AA	AG	AA	AA
	AA	AA	AA	AA	AG	AG	AG	
57193344								
10682	rs10411991		19	AC	AC	CC	CC	CC
	AC	CC	AC	AC	AC	CC	CC	CC
	CC	CC	CC	CC	CC	CC	CC	AC
	CC	CC	CC	CC	CC	CC	CC	CC
	CC	CC	CC	CC	AC	CC	AA	
670828								
10683	rs10411996							
19	--	--	--	--	--	--	--	--
	--	--	--	--	--	--	--	--
	--	--	--	--	--	--	--	--
	--	--	--	--	--	--	--	--
	--	--	--	--	28248413			
10684	rs1041200		20	AA	AA	AG	AA	AA
	AA	AA	AA	AA	AA	AG	AA	AG
	AG	AA	AA	AG	AA	AG	AA	AA
	AG	AG	AG	AG	AA	AG	AG	AG
	AA	AA	AA	AA	AA	AA	AG	
10979521								
10685	rs10412028		19	GG	AG	GG	AA	AG
	AG	AG	GG	AG	AG	GG	GG	GG
	AG	AG	AA	AA	AA	GG	AG	GG
	AG	GG	GG	AG	AG	GG	AG	GG
	AG	AG	AG	GG	GG	GG	GG	
24398519								
10686	rs10412052		19	AG	AG	AG	GG	GG
	GG	GG	GG	GG	GG	GG	GG	GG
	GG	GG	AG	GG	GG	GG	GG	GG
	GG	GG	GG	GG	AG	GG	AG	AG
	GG	GG	GG	GG	GG	GG	GG	
16097075								
10687	rs10412087		19	--	--	AG	--	--
	--	--	AG	AG	AG	--	AG	AG
	--	--	--	--	--	AG	AG	--
	--	--	--	--	--	--	--	--
	--	AG	AG	--	--	--	--	
2853566								
10688	rs10412098		19	AA	AA	AA	AA	AG
	AG	AA	AA	AA	AA	AA	AA	AA
	AA	AA	AG	AA	AG	AG	AA	AA
	AA	AA	AA	AA	AA	AG	AG	AG
	AG	AA	AA	AA	AA	AA	AA	
38905683								
10689	rs1041210							
13	--	--	--	--	--	--	--	--
	--	--	--	--	--	--	--	--
	--	--	--	--	--	--	--	--
	--	--	--	--	--	--	--	--

	--	--	--	--	40940787			
10690	rs10412129		19	AG	AA	AA	AA	AA
	AA	AA	GG	AG	AG	AA	AA	AA
	AA	AA	AA	AA	AA	AA	AA	AA
	AA	AA	AA	AA	AA	AA	AA	AA
	AA	AA	AA	AA	AA	AA	AA	
9800690								
10691	rs10412132		19	AA	AG	AG	AG	AG
	GG	AG	GG	GG	GG	AA	AG	AG
	AA	AA	AG	AA	AG	AG	AG	AG
	AA	AA	AA	AA	GG	AG	GG	GG
	AG	GG	GG	GG	AG	AG	AG	
30914411								
10692	rs10412141		19	AG	GG	GG	AG	GG
	AG	AG	AA	AG	AG	AA	AG	AA
	AA	AG	AA	AA	AA	AG	AG	AA
	AA	AG	AG	AA	AA	AA	AA	AA
	AA	AG	AA	AG	AA	AA	AA	
33034189								
10693	rs10412149		19	GG	GG	GG	GG	GG
	GG	GG	GG	GG	GG	GG	GG	GG
	GG	GG	GG	GG	GG	GG	GG	GG
	GG	GG	GG	GG	GG	GG	GG	GG
	GG	GG	GG	GG	GG	GG	GG	
23970806								
10694	rs10412159		19	GG	GG	GG	GG	GG
	GG	GG	GG	GG	GG	GG	GG	GG
	GG	GG	GG	GG	GG	GG	GG	GG
	GG	GG	GG	GG	GG	GG	GG	GG
	GG	GG	GG	GG	GG	GG	GG	
1225642								
10695	rs10412189		19	AA	AA	AA	AC	AA
	AC	AA	AA	AC	AC	AA	AA	AA
	AA	AA	AA	AA	AA	AA	AC	AA
	AC	AC	AC	AC	AA	AA	AA	AA
	AC	AA	AA	AA	AC	AC	AA	
24080338								
10696	rs10412221		19	GG	GG	GG	GG	GG
	GG	GG	GG	GG	GG	GG	AG	GG
	GG	AG	GG	GG	GG	AG	GG	GG
	GG	GG	GG	GG	GG	GG	GG	GG
	GG	GG	GG	GG	GG	GG	GG	
43202770								
10697	rs10412222		19	AA	AG	AG	AA	AA
	AA	AA	AA	AA	AA	AA	AA	AA
	AA	AA	AA	AA	AA	AA	AG	AA
	AA	AA	AA	AA	AA	AA	AA	AA
	AA	AA	AA	AA	AA	AA	AA	
32286166								
10698	rs10412238		19	AA	AA	AA	AA	AA

AA AA AA AG AG AG AA AG
AA AA AA AA AA AG AG AA
AA AA AA AA AA AA AA AA
AA AA AA AA AA AA AA
19318158
10699 rs10412262 19 AA AA AG GG GG
GG GG AG AA AA AA GG AG
AG AG AG AG AG GG AG AG
AG AG AG AG AG AG AA AG
AG GG AA AG AG AG AA
5975180
10700 rs10412317 19 AG GG AG AG AG
GG AG AG AG AG GG AG GG
AG AA AG AG AG GG AG GG
AG AG AA GG GG GG GG GG
AG AG AA AG AA AG AG
48507720
10701 rs10412431 19 GG GG GG GG GG
GG GG GG GG GG GG GG GG
GG GG GG GG GG GG GG GG
GG GG GG GG GG GG GG GG
GG GG GG GG GG GG GG
24315797
10702 rs10412464 19 AA AA AG AA AG
AA AG AA AA AA AA AA AA
AA AA AA AA AA AA AA AA
AA AA AA AA AA AA AA AA
AA AA AA AA AA AA AA
7432176
10703 rs10412473 19 GG GG GG GG GG
GG GG GG GG GG GG GG GG
GG GG AG AG AG GG GG GG
GG AG AG GG GG GG GG GG
GG GG GG GG GG GG GG
54945174
10704 rs10412497 19 GG GG GG GG GG
GG GG GG GG GG GG GG GG
GG GG GG GG GG GG GG GG
GG GG GG GG GG GG GG GG
GG GG GG GG GG GG GG
16206017
10705 rs10412509 19 AG AG AG GG GG
GG AG AG GG GG GG GG GG
GG GG GG GG GG AG GG GG
GG AG AG GG GG GG GG GG
GG -- GG AG GG GG GG
21284501
10706 rs1041251 9 AG AG GG AG AG
AA AG AG AG GG AG AG GG
GG AG GG AG AG AG AG GG

GG GG GG GG GG AG GG GG
AG AA AA AA AG AA GG
34187354
10707 rs10412546 19 GG GG GG GG GG
GG GG GG GG GG GG GG GG
GG GG GG GG GG GG GG GG
GG GG GG GG GG GG GG GG
GG GG GG GG GG GG GG
28680488
10708 rs10412550 19 GG GG GG GG AG
AG GG GG GG GG GG AG GG
AG AA GG GG GG GG GG GG
GG GG GG GG AG AA AG AA
AG GG GG GG GG GG GG
34071781
10709 rs10412559 19 AA AA AG AA AA
AA AA AA AA AA AA AA AA
AA AA AA AG AA AG AA GG
AG AG AG AG AA AG AG AG
AG GG AA AG AA AA AA
2699585
10710 rs10412560 19 GG GG GG AG AG
GG AA GG GG GG AG AG AG
AA AG GG AG GG AG GG AG
AG AG AG AG AG AA AG AA
AG AA AA AA AG GG AG
57258083
10711 rs10412585 19 AA AA AA AA AA
AA AA AA AA AA AA AA AA
AA AA AA AA AA AA AA AA
AA AA AA AA AA AA AA AA
AA AA AA AA AA AA AA
58878833
10712 rs10412628 19 GG GG GG GG GG
GG GG GG GG GG GG GG GG
GG GG GG GG GG GG GG GG
GG GG GG GG GG GG GG GG
GG GG GG GG GG GG GG
28643186
10713 rs10412635 19 GG GG GG GG GG
GG GG GG GG GG GG GG GG
GG GG GG GG GG GG GG GG
GG GG GG GG GG GG GG GG
GG GG GG GG GG GG GG
53556045
10714 rs10412670 19 GG GG GG GG GG
GG GG GG GG GG GG GG GG
GG GG GG GG GG GG GG GG
GG GG GG GG GG GG GG GG
GG GG GG GG GG GG GG

24315937
10715 rs1041272 20 AG AG AG AG GG
AG GG GG GG GG GG GG GG
GG GG AG AG AG GG GG AG
AG GG AG GG GG GG GG GG
GG GG GG GG GG GG GG
57987864
10716 rs10412776 19 GG GG GG GG GG
GG GG GG GG GG AG GG AG
AG AG GG GG GG GG AG GG
GG AA AG GG AG AG AG AA
AG GG GG GG AG GG AA
29924013
10717 rs10412787 19 GG GG GG GG GG
GG GG GG GG GG GG GG GG
GG GG GG GG GG GG GG GG
GG GG GG GG GG GG GG GG
GG GG GG GG GG GG GG
19596117
10718 rs10412913 19 GG GG GG GG GG
GG GG GG GG GG GG GG GG
GG GG GG GG GG GG GG GG
GG GG GG GG GG GG GG GG
GG GG GG GG GG GG GG
9433682
10719 rs10412982 19 AG AG GG AA AA
AG AG GG AG AG AG AG GG
GG GG GG AG GG AG GG AG
GG GG -- GG AG AG GG AG
GG AG AG AG GG AG AG
3274604
10720 rs10413039 19 GG GG GG GG AG
AG GG AG GG GG GG GG GG
GG GG GG GG GG GG AG GG
GG GG GG GG GG GG GG GG
AG GG GG GG AG AG GG
23655760
10721 rs10413055 19 CC CC CC CC CC
CC CC CC CC CC CC CC CC
CC CC CC CC CC CC CC CC
CC CC CC CC CC CC CC CC
CC CC CC CC CC CC CC
28418407
10722 rs10413089 19 AA AA AA AG GG
AG AG GG AG AG AA AA AA
AA AA AA AG AG AA AG AG
AA AG AA AG AA AG AG AG
AG AA AA AA AG AA AG
45455588
10723 rs10413093 19 GG GG GG GG GG

	GG	GG	GG	GG	GG	GG	GG	GG
	GG	GG	GG	GG	GG	GG	GG	GG
	GG	GG	GG	GG	GG	GG	GG	GG
	GG	GG	GG	GG	GG	GG	GG	
7987997								
10724	rs10413136		19	AA	AA	AA	AA	AA
	AA	AA	GG	GG	GG	AG	GG	GG
	AG	AG	AA	AG	AA	AG	AA	AG
	AA	AA	AG	AG	GG	AA	AG	AG
	AG	AG	AA	AG	AA	AG	AG	
8452879								
10725	rs10413189		19	GG	GG	GG	AG	AG
	GG	AG	GG	GG	GG	GG	GG	GG
	GG	GG	GG	GG	GG	GG	GG	GG
	GG	GG	GG	GG	GG	GG	GG	GG
	GG	GG	GG	GG	GG	GG	GG	
2334538								
10726	rs10413199		19	GG	AG	AG	GG	GG
	AG	AG	GG	GG	GG	GG	AG	AG
	GG	GG	AG	AG	GG	GG	GG	GG
	GG	GG	GG	GG	GG	AG	AG	AG
	GG	AA	AG	AG	GG	GG	GG	
431187								
10727	rs10413216		19	AA	AA	AA	AA	AA
	AA	AA	AA	AA	AA	AA	AA	AA
	AA	AA	AA	AA	AA	AA	AA	AA
	AA	AA	AA	AA	AA	AA	AA	AA
	AA	AA	AA	AA	AA	AA	AA	
19185425								
10728	rs10413288		19	AG	AA	AG	GG	GG
	GG	AG	GG	AG	AG	AG	AG	AG
	AG	AG	GG	GG	AG	AG	AG	AA
	GG	AG	AG	GG	AA	AA	AA	AA
	GG	GG	AG	AG	AG	AA	AG	
54197633								
10729	rs10413358		19	GG	GG	GG	GG	GG
	GG	GG	GG	GG	GG	GG	GG	GG
	GG	GG	GG	GG	GG	GG	GG	GG
	GG	GG	GG	GG	GG	GG	GG	GG
	GG	GG	GG	GG	GG	GG	GG	
6246163								
10730	rs10413359		19	AG	AG	AG	AA	AG
	AG	AG	GG	GG	GG	AA	GG	AG
	AG	AG	AA	AG	AG	GG	GG	AG
	AG	GG	GG	AG	GG	AG	AG	AG
	AG	GG	GG	GG	GG	GG	AG	
42271006								
10731	rs10413398		19	AG	AA	AG	AG	AG
	AA	AG	AG	AA	AA	AG	AA	AG
	AG	AG	AA	AG	AA	GG	AA	AG

	AA	AG	AG	AG	AA	GG	AG	AG
	AA	AA	AA	AA	AG	GG	AA	
2106696								
10732	rs10413439		19	CC	CC	CC	CC	CC
	CC	CC	CC	CC	CC	CC	CC	CC
	CC	CC	CC	CC	CC	CC	CC	CC
	CC	CC	CC	CC	CC	CC	CC	CC
	CC	CC	CC	CC	CC	CC	CC	
54240981								
10733	rs1041345		12	AA	AA	AA	AG	AA
	AA	AG	AG	GG	AG	AG	AG	AG
	AG	AG	AG	AA	AA	AG	AG	AG
	AG	GG	AA	GG	AG	AG	AA	AA
	AA	AG	AA	AG	AA	AA	AA	
41197785								
10734	rs1041359		13	GG	GG	GG	GG	GG
	GG	GG	GG	GG	GG	GG	GG	GG
	GG	GG	GG	GG	GG	GG	GG	GG
	GG	GG	GG	GG	GG	GG	GG	GG
	GG	GG	GG	GG	AG	AG	GG	
73793864								
10735	rs10413613		19	GG	GG	GG	AG	AG
	GG	AG	GG	GG	GG	GG	GG	GG
	GG	GG	GG	GG	GG	GG	GG	GG
	GG	GG	GG	GG	GG	GG	GG	GG
	GG	GG	GG	GG	GG	GG	GG	
20456739								
10736	rs10413625		19	AG	AG	AG	GG	GG
	GG	AG	AG	GG	GG	AG	GG	AG
	GG	GG	AG	AG	GG	AG	AG	AA
	GG	AG	AG	GG	AA	AA	AA	AA
	GG	GG	AG	GG	AA	AG	AG	
20731545								
10737	rs10413667		19	GG	GG	GG	GG	GG
	GG	GG	GG	GG	GG	GG	GG	GG
	GG	GG	GG	GG	GG	GG	GG	GG
	GG	GG	GG	GG	GG	GG	GG	GG
	GG	GG	GG	GG	GG	GG	GG	
9573480								
10738	rs10413734		19	AC	AA	AA	AC	AC
	AC	AC	AC	CC	CC	CC	CC	CC
	AA	AA	AA	AA	AC	AA	AA	AA
	AA	AA	AC	AC	AC	AA	AC	AA
	AA	AC	AA	AC	AC	AC	AC	
7227871								
10739	rs10413767		19	AA	AA	AA	AA	AG
	AG	AA	AG	AA	AA	AA	AA	AA
	AA	AA	AA	AA	AA	AA	AG	AA
	AA	AA	AA	AA	AA	AA	AA	AA
	AG	AA	AA	AA	AG	AG	AA	

23657403
10740 rs10413821 19 GG AG AG AA AA
AA AA AA AG AG AA AG AG
AA AA GG AG AG AA AG AA
AA AG AG AA GG AG GG GG
AG AG GG GG AG GG AA
17741047
10741 rs10413841 19 AG AG AG AA AA
AA AG AA AA AA AA AA AA
AA AA AA AA AA AA AA AA
AA AG AG AA AA AA AA AA
AA AA AA AA AA AA AA
21180787
10742 rs1041385 13 AA AG AA GG AG
AG AG AA AA AA AG AG AG
AG AA AG AG AA AA AG AA
AA AA AG AG AG AA AA AG
AA AG AG GG AG AG AG
113306882
10743 rs10413858 19 CC CC CC CC CC
CC CC CC CC CC CC CC CC
CC CC CC CC CC CC CC CC
CC CC CC CC CC CC CC CC
CC CC CC CC CC CC CC
12946079
10744 rs1041389 10 GG AG AA AG AG
AG GG AG AG AG GG GG GG
GG AG AG GG AG GG GG GG
GG GG GG GG AG GG GG AG
GG GG AG AG AG AA GG
90991179
10745 rs10413924 19 AA AA AA AA AA
AA AG AA AA AA AG AG AG
AG AG AG AA AA AA AA AG
AG AG AG AG AG AG AA AG
AA AG AG AG AA AG AG
53528414
10746 rs10413944 19 CC CC CC CC CC
CC CC CC CC CC CC CC CC
CC CC CC CC CC CC CC CC
CC CC CC CC CC CC CC CC
CC CC CC CC CC CC CC
4566429
10747 rs10413967 19 AA AA AG AG AA
AG AA AG AG AG GG AG AG
AG AG AG GG AG AA AA AG
AA GG GG AA GG AG GG GG
AA AG AG AA AG GG AA
22678681
10748 rs10413998 19 GG GG GG GG GG

	GG	GG	GG	GG	GG	GG	GG	GG
	GG	GG	GG	GG	GG	GG	GG	GG
	GG	GG	GG	GG	GG	GG	GG	GG
	GG	GG	GG	GG	GG	GG	GG	
20486867								
10749	rs10414009		19	GG	GG	GG	AG	GG
	AG	GG	GG	GG	GG	GG	GG	GG
	GG	GG	GG	GG	GG	GG	GG	GG
	GG	AG	AG	GG	AG	AG	GG	AG
	GG	GG	GG	GG	AG	AG	GG	
29526586								
10750	rs10414040		19	AA	AA	AA	AC	AA
	AC	AA	AA	AA	AA	AA	AA	AA
	AA	AA	AA	AC	AC	AA	AA	AA
	AA	AA	AA	AA	AA	AA	AA	AA
	AA	AA	AA	AA	AA	AA	AA	
35042631								
10751	rs10414075		19	AA	AG	AA	AG	AG
	GG	AA	AA	AG	AG	AA	AA	AA
	AA	AA	GG	AG	AG	AA	AG	AA
	AG	AG	AG	AG	AA	AA	AA	AA
	GG	AA	AG	AA	AG	GG	AA	
24238451								
10752	rs10414102		19	GG	GG	GG	GG	GG
	GG	GG	GG	GG	GG	GG	GG	GG
	GG	GG	GG	GG	GG	GG	GG	GG
	GG	GG	GG	GG	GG	GG	GG	GG
	GG	GG	GG	GG	GG	GG	GG	
8654354								
10753	rs10414149		19	AA	AG	AA	AA	AA
	AA	AA	AA	AA	AA	AA	AA	AA
	AA	AA	AG	AG	AG	AA	AA	AA
	AA	AG	AA	AG	AA	AA	AA	AA
	AA	AA	AA	AA	AA	AG	AA	
52127744								
10754	rs1041416		21	GG	AG	AG	AA	GG
	AG	AG	AG	AA	AA	AG	AA	AG
	AG	AG	GG	AG	AG	AG	GG	GG
	GG	GG	GG	GG	AG	GG	GG	AG
	AG	GG	AG	AG	GG	GG	AG	
23542443								
10755	rs10414166		19	AG	AG	AG	GG	GG
	GG	GG	AG	AG	AG	AG	GG	AG
	GG	GG	AA	AG	AG	GG	AG	AG
	GG	AG	GG	AG	GG	AG	GG	GG
	AG	GG	GG	GG	GG	GG	GG	
43519442								
10756	rs10414170		19	AC	CC	AC	CC	CC
	CC	CC	CC	AC	AC	AC	AC	AC
	CC	AC	CC	CC	CC	AC	CC	AC

	CC	CC	CC	CC	AC	AA	AA	AC
	AC	AC	CC	AC	CC	AC	CC	
57246309								
10757	rs10414199		19	AG	AA	AG	AA	AA
	AA	AG	AA	AA	AA	AG	AG	AG
	AA	AG	AG	GG	AG	AG	AG	AG
	AA	AG	AA	AG	GG	AG	AG	AG
	AG	AA	GG	AG	GG	GG	GG	
35867279								
10758	rs1041420		21	AG	GG	AG	GG	GG
	GG	GG	GG	GG	GG	GG	GG	GG
	GG	GG	AG	AG	AG	GG	GG	GG
	GG	GG	GG	GG	GG	AG	AG	AG
	AG	AG	GG	AG	GG	GG	GG	
27443650								
10759	rs10414225		19	CC	CC	CC	AC	AC
	CC	AA	AA	AC	AC	CC	CC	CC
	AC	AC	AC	AA	AA	AA	AC	AA
	AA	AA	AA	AA	AC	AC	AC	AC
	AC	AC	AC	CC	AC	AC	AC	
15062223								
10760	rs10414271		19	CC	AC	AC	CC	CC
	CC	CC	CC	CC	CC	AC	AC	AC
	AC	CC	CC	CC	CC	AC	CC	AA
	CC	AC	CC	AC	AC	CC	CC	AC
	CC	CC	CC	CC	AA	AC	AA	
31958905								
10761	rs10414297		19	AA	AA	AA	AA	AA
	AA	AA	AA	AA	AA	AA	AA	AA
	AA	AA	AA	AA	AA	AA	AA	AA
	AA	AA	AA	AA	AA	AA	AA	AA
	AA	AA	AA	AA	AA	AA	AA	
22736573								
10762	rs10414304		19	CC	CC	CC	CC	CC
	CC	CC	CC	CC	CC	CC	CC	CC
	CC	CC	CC	CC	CC	CC	CC	CC
	CC	CC	CC	CC	CC	CC	CC	CC
	CC	CC	CC	CC	CC	CC	CC	
58404160								
10763	rs1041434		21	AA	AA	AA	AG	AG
	GG	AG	AA	AA	AG	AA	AA	AA
	AA	AA	AA	AA	AA	AG	AA	AA
	AA	AA	AA	AA	AG	AA	AG	AA
	AA	AA	AG	AA	GG	AG	AG	
37639689								
10764	rs10414354		19	GG	GG	GG	GG	GG
	GG	GG	AG	AA	AG	GG	GG	GG
	GG	GG	GG	GG	GG	GG	GG	GG
	GG	GG	GG	GG	GG	GG	GG	GG
	GG	GG	GG	GG	GG	AG	GG	

1313763							
10765	rs10414401	19	AG	AG	GG	AG	AG
	AA	AG	AG	AG	AG	GG	AG	AG
	AG	AG	AG	AG	AA	AG	AG	GG
	AA	AG	AG	AA	AG	GG	AG	GG
	AG	AG	AA	AG	AG	AA	AG	
28411971							
10766	rs10414407	19	AA	AA	AA	AA	AA
	AA	AA	AA	AA	AA	AA	AA	AA
	AA	AA	AA	AA	AA	AA	AA	AA
	AA	AA	AA	AA	AA	AA	AA	AA
	AA	AA	AA	AA	AA	AA	AA	
23129421							
10767	rs10414433	19	AC	AC	AC	CC	CC
	CC	CC	CC	AC	AC	CC	CC	CC
	CC	CC	CC	CC	CC	AC	CC	AA
	CC	AC	CC	AC	CC	CC	CC	CC
	CC	CC	AC	CC	AC	CC	AC	
38544631							
10768	rs10414451	19	AA	AA	AA	AA	AA
	AA	AG	AA	AA	AA	AG	GG	AG
	AG	AA	AA	AA	AA	AA	AA	AA
	AA	AA	AA	AA	AA	AA	AA	AA
	AA	AA	AG	AG	AA	AA	AA	
58131576							
10769	rs10414457	19	GG	GG	GG	GG	GG
	GG	GG	GG	GG	GG	GG	GG	GG
	GG	GG	GG	GG	GG	--	GG	GG
	GG	GG	GG	GG	GG	GG	GG	GG
	GG	GG	GG	GG	GG	GG	GG	
13665981							
10770	rs10414458	19	AG	AG	GG	AG	AG
	AA	GG	AG	AG	AG	AA	AG	AA
	AG	AG	AA	AA	AA	AA	AG	AA
	AG	GG	AG	AG	AG	AG	AG	AA
	AA	AG	AG	GG	AG	AA	AG	
31304355							
10771	rs10414485	19	AG	GG	GG	AG	AG
	GG	AA	AA	AG	AG	GG	GG	GG
	GG	GG	AG	GG	GG	GG	GG	GG
	GG	GG	AG	AG	GG	AG	GG	GG
	GG	AG	GG	GG	GG	AG	GG	
9868404							
10772	rs10414512	19	GG	GG	GG	GG	GG
	GG	GG	GG	GG	GG	GG	GG	GG
	GG	GG	GG	GG	GG	GG	GG	GG
	GG	GG	GG	GG	GG	GG	GG	GG
	GG	GG	GG	GG	GG	GG	GG	
14822948							
10773	rs10414528	19	AA	AG	AG	AG	AA

AA AG GG AG AG GG GG GG
GG GG GG GG GG AG AG AG
AG AG GG GG AG GG GG AG
GG GG AA AG GG GG GG
10148364
10774 rs1041454 21 AA AA AA AA AA
AA AA AA AA AA AA AA AA
AA AA AA AA AA AA AA AA
AA AA AA AA AA AA AA AA
AA AA AA AA AA AA AA
45112660
10775 rs10414579 19 CC CC CC CC CC
CC CC AC AC AC CC CC CC
CC CC AC CC AC CC CC CC
CC CC CC CC CC CC CC CC
CC CC CC CC CC CC CC
28654541
10776 rs10414615 19 AA AA AA AA AA
AA AA AA AA AA AA AA AA
AA AA AA AA AA AA AA AA
AA AA AA AA AA AA AA AA
AA AA AA AA AA AA AA
4752762
10777 rs10414625 19 AA AG AA AG AA
AG AA AA AG AG AA AA AA
AA AA AA AG AG AA AG AA
AG AG AG AG AG AA AG AA
AG AA AG AA AG AG AA
24141606
10778 rs10414676 19 GG GG GG AG AA
AA AG AG AA AA AG GG AG
AG AG AG GG AG AA AG AG
AG AG AG AG AA AG AA AA
AG AG AA AG AG AG AG
19243843
10779 rs1041469 1 GG GG GG GG AG
AG GG AG GG AG AG GG AG
AG AG AG AG AA GG GG GG
GG GG GG GG AG GG GG GG
GG GG GG GG GG AG AG
232284603
10780 rs10414691 19 AG AG AG AA AA
AG AA AA AA AA AG AG AG
GG AG AA AA AG AA AA AG
AG AA AG AA AG AA AA AG
AA AG AA AG AA AA AG
51209949
10781 rs10414696 19 AA AA AA AA AA
AA AA AA AA AA AA AA AA
AA AA AA AA AA AA AA AA

AA AA AA AA AA AA AA AA
AA AA AA AA AA AA AA
55665270
10782 rs10414716 19 AA AA AA AA AA
AA AA AA AA AA AA AA AA
AA AA AA AA AA AA AG AA
AA AA AA AA AA AA AA AA
AA AA AA AA AA AA AA
32179999
10783 rs10414740 19 CC CC CC CC CC
CC CC CC CC CC CC CC CC
CC CC CC CC CC CC CC CC
CC CC CC CC CC CC CC CC
CC CC CC CC CC CC CC
16561596
10784 rs10414776 19 AA AA AA AA AA
AA AA AA AA AA AA AA AA
AA AA AA AG AA AA AA AG
AA AA AA AA AG AA AA AG
AA AA AG AG AA AA AA
47201818
10785 rs1041483 9 -- AA AA AA AA
AA AA AA AA AA AA AA AA
AA AA AA AA AA AA AA AA
AA AA AA AA AA AA AA AA
AA AA AA AA AA AA AA
78671654
10786 rs10414862 19 AG AA AG AA AA
AA AA AG AG AG AG AG GG
GG GG AG GG GG AG GG AG
AG AG AA AA AG GG GG AG
GG AG AG AG AG AA AG
2534533
10787 rs10414884 0 AA AA AA AA AA
AA AA AA AA AA AA AA AA
AA AA AA AA AA AA AA AA
AA AA AA AA AA AA AA AA
AA AA AA AA AA AA AA 0
10788 rs10414890 19 AG AG AG GG AG
AG AG GG GG GG AG AA AG
AA AA GG GG GG AG AA AA
AG AA AA AG AG AG AG AA
GG GG AA AG AG AG AG
40106388
10789 rs10414941 19 AG AG AG GG GG
GG GG GG AG AG AG GG GG
GG AG AA AA AG AG GG AA
GG GG GG GG AG GG GG AG
GG GG GG GG GG AG GG
51988280

10790	rs10415034		19	AA	AA	AG	GG	AG
	AG	GG	AG	GG	GG	AA	AG	AA
	GG	GG	AG	AG	GG	AG	AG	AA
	AG	AA	AA	AG	AG	AA	AA	AA
	AG	AG	GG	AG	AA	AG	AA	
16945205								
10791	rs10415055		19	GG	GG	GG	GG	GG
	GG	GG	GG	GG	GG	GG	GG	GG
	GG	GG	GG	GG	GG	GG	GG	GG
	GG	GG	GG	GG	GG	GG	GG	GG
	GG	GG	GG	GG	GG	GG	GG	
14688725								
10792	rs1041506		9	AG	AG	GG	GG	GG
	GG	GG	AG	AG	AG	GG	GG	GG
	AG	AG	GG	GG	AG	GG	AG	GG
	GG	GG	GG	GG	GG	AG	GG	AG
	GG	GG	GG	GG	AG	AG	AG	
9022772								
10793	rs10415081		19	AG	AG	GG	AG	AG
	AA	AG	AG	AG	AG	AG	AG	AG
	AG	AG	AA	AA	AA	AG	AG	GG
	AA	AA	AA	AA	AA	AG	AG	AG
	AA	AG	AA	AG	AA	AG	AA	
23761513								
10794	rs10415132		19	AG	GG	GG	AG	AG
	GG	AA	AA	AG	AG	GG	GG	GG
	GG	GG	AG	GG	GG	GG	GG	GG
	GG	GG	AG	AG	GG	AG	GG	GG
	GG	AG	GG	GG	GG	AG	GG	
9863483								
10795	rs10415141		19	AC	AC	AA	CC	CC
	CC	CC	AC	AC	AC	AA	AC	AA
	AC	CC	AC	AC	AA	CC	AA	CC
	CC	AC	AC	CC	CC	AC	AC	AC
	AC	CC	CC	CC	AC	AC	AC	
57237574								
10796	rs10415143		19	CC	AC	CC	AA	AC
	AC	AA	CC	AC	AC	AC	CC	CC
	CC	CC	CC	AC	AC	CC	AC	CC
	AA	AA	AA	AA	AC	CC	AC	CC
	AC	CC	AA	AC	AC	AA	CC	
24180916								
10797	rs1041522		6	GG	GG	GG	AG	AG
	GG	AA	AG	GG	AG	AA	AA	AA
	AG	AG	AG	AG	AG	AG	AG	AG
	AA	AA	AA	AA	AG	AG	GG	GG
	AG	AG	AG	GG	AA	AA	AA	
65829390								
10798	rs10415225		19	AA	AA	AA	AA	AG
	AA	AG	AA	AA	AA	AA	AA	AA

AA AA AA AA AA AA AA AA
AA AA AA AA AA AA AA AA
AA AA AA AA AA AA AA
10154673
10799 rs1041524 6 AA AA AA AA AG
AG AA AG AG AA AA AG AG
GG AG AG AG AG AA AA AA
AG AG AG AG AA AA AA AA
AA AG AG AG AG AG AA
44156470
10800 rs10415281 19 AG GG GG AG GG
AG GG AA AG AG GG AG AG
AG AG AA AG AG GG AG AG
AG AG AG AG AA AG AA AA
GG GG GG GG AG AG GG
9599034
10801 rs10415386 19 GG GG GG GG GG
GG GG GG GG GG GG GG GG
GG GG AG AG AG AG AG AG
GG GG GG GG GG GG GG GG
GG AG GG AG AG AG GG
14630476
10802 rs10415451
19 -- -- -- -- -- -- -- --
-- -- -- -- -- -- -- --
-- -- -- -- -- -- -- --
-- -- -- -- -- -- -- --
-- -- -- -- 20203499
10803 rs10415481 19 GG GG GG GG GG
GG GG GG GG GG GG GG GG
GG GG GG GG GG GG GG GG
GG GG GG GG GG GG GG GG
GG GG GG GG GG GG GG
5371399
10804 rs1041551 10 GG GG GG GG AG
AG GG AA AA AG GG GG GG
GG GG AG GG GG GG GG GG
GG GG GG GG AG GG GG GG
GG GG AG GG AA AG AG
20588380
10805 rs10415602 19 AA AG AA AA AG
AA AG AA AA AA AA AA AA
AG AG AG AG AG AA AA AG
AA AA AA AA AG AA AG AG
AA AA AA AA AA AA AA
14005618
10806 rs10415607 19 AA AA AA AA AA
AA AA AA AA AA AA AA AA
AA AA AA AA AA AA AA AA
AA AA AA AA AA AA AA AA

AA AA AA AA AA AA AA
24472968
10807 rs10415665 19 AA AA AA AA AA
AA AA AA AA AA AA AA AA
AA AA AA AA AA AA AA AA
AA AA AA AA AA AA AA AA
AA AA AA AA AA AA AA
13123902
10808 rs10415672 19 AA AG AA GG AG
AG GG AA AG AG AG AA AA
AA AA AA AG AG AA AG AA
GG GG GG GG AG AA AG AA
AG AA GG AG AG GG AA
24175453
10809 rs10415693 19 GG GG GG GG GG
GG GG GG GG GG GG GG GG
GG GG GG GG GG GG GG GG
GG GG GG GG GG GG GG GG
GG GG GG GG GG GG GG
24216218
10810 rs10415721 19 GG GG GG GG GG
GG GG GG GG GG GG GG GG
GG GG GG GG GG GG GG GG
GG GG GG GG GG GG GG GG
GG GG GG GG GG GG GG
24016956
10811 rs10415741 19 AA AA AA AA AA
AA AA AA AA AA AA AA AA
AA AA AA AA AA AA AA AA
AA AA AA AA AA AA AA AA
AA AA AA AA AA AA AA
23844456
10812 rs10415742 19 AA AC AA AA AC
AA AC AC CC CC AA AA AA
AA AA AC AC AC AA AA AA
AA AA AA AA AA AA AA AA
AA AA AA AA AA AA AA
13433292
10813 rs10415787 19 GG GG GG GG GG
GG GG GG GG GG GG GG GG
GG GG GG GG GG GG GG GG
GG GG GG GG GG GG GG GG
GG GG GG GG GG GG GG
28810255
10814 rs10415801 19 AG AA AG GG GG
GG GG GG GG GG AG AG AG
AG AG AG AG AG GG GG AG
AA AA AG AG AA AG AA AA
GG AG GG GG GG AG GG
53164172

10815 rs10415849 19 GG GG GG GG GG
GG GG GG GG GG GG GG GG
GG GG GG GG GG AG GG GG
GG GG GG GG GG AG GG GG
GG GG AG GG GG GG GG
19505087
10816 rs10415893 19 AG AG AG GG GG
GG GG GG GG GG GG GG GG
GG GG GG GG GG GG GG AG
GG GG GG GG AG GG AG GG
AG GG AG AG GG GG GG
17512412
10817 rs10415916 19 AA AA AA AA AA
AA AA AA AA AA AA AA AA
AA AA AA AA AA AA AA AA
AA AA AA AA AA AA AA AA
AA AA AA AA AA AA AA
5233172
10818 rs10415938 19 GG AG GG GG GG
GG GG GG GG GG GG GG GG
GG GG AG GG GG GG GG GG
GG GG GG GG GG GG GG GG
AG GG GG GG GG GG GG
14984836
10819 rs10415976 19 AA AA AA AA AA
AA AA AA AA AA AA AG AG
AA AA AA AA AA AA AG AA
AA AG AA AA AA AG AA AA
AA AA AA AA AA AG AA
941603
10820 rs10416093 19 GG GG GG GG GG
GG GG GG GG GG GG GG GG
GG GG GG GG GG GG GG GG
GG GG GG GG GG GG GG GG
GG GG GG GG GG GG GG
10847881
10821 rs10416139 19 GG GG GG GG AG
GG AG GG GG GG GG GG GG
GG GG AG GG GG GG GG GG
GG GG GG GG GG GG GG GG
GG AG AG GG GG AG GG
10627322
10822 rs10416146 19 AA AA AA AG AA
AG AA AA AA AA AA AA AA
AA AA AA AG AG AA AA AA
AA AA AA AA AA AA AA AA
AA AA AA AA AA AA AA
35026796
10823 rs10416209 19 GG GG GG GG GG
GG GG GG GG GG GG GG GG

GG GG GG GG GG GG GG GG
GG GG GG GG GG GG GG GG
GG GG GG GG GG GG GG
9738753
10824 rs10416239 19 GG AG AG GG GG
GG GG GG GG GG AG AG GG
GG GG AG GG GG GG GG GG
GG GG GG GG GG AG AG AG
AA GG GG GG GG GG GG
2058331
10825 rs10416242 19 AA AG AA AG AG
GG AA GG AG AG GG AG GG
AG AG AA AA AG AG AG AA
GG GG AG AA AA GG AG AG
AA GG GG GG AA AA AG
13750618
10826 rs10416286 19 AA AA AA AA AA
AA AA AA AA AA AA AG AA
AA AA AA AA AA AA AA AA
AA AA AA AA AG AA AG AA
AA AA AA AA AA AA AA
6002288
10827 rs1041631 9 GG GG GG GG AG
AG GG GG AG AG GG AG AG
AG AG GG GG GG AA AA AA
GG GG GG GG GG GG GG GG
GG AG GG GG GG GG GG
101781858
10828 rs10416325 19 AG GG GG GG GG
GG GG GG GG GG AG GG GG
GG GG GG GG GG AG GG GG
GG AG AG AG AG GG AG GG
AG GG AG AG GG GG GG
17046191
10829 rs10416372 19 AA AA AA AA AA
AA AA AA AA AA AA AA AA
AA AA AA AA AA AA AA AA
AA AA AA AA AA AA AA AA
AA AA AA AA AA AA AA
5249037
10830 rs10416425 19 AG AG AG AG AG
AG AG GG GG GG AG AA AG
AG GG AG AG AG GG AG GG
AG AG AG AG GG GG GG GG
GG AG AA AA GG AG AG
57665345
10831 rs10416428 19 AC CC CC AC CC
AC CC CC CC CC CC AA AC
AC AC CC AC AC CC AA CC
AA CC AC AC CC AC AC AC

CC CC CC CC AC AC CC
37617609
10832 rs10416524 19 AG AA AA AA AA
AA AA AA GG GG AA AA AA
AA AA AG AA AA AG AG AA
AA AA AG AG AA AA AA AA
AG AA AA AA AG AG AA
2505737
10833 rs1041654 6 AG AA AG GG AG
AG GG GG AG GG GG GG GG
GG GG AG GG AG GG AG AG
AG AG AG AG AG AA AG AG
AG AG AG AG GG AG GG
143289361
10834 rs10416569 19 GG GG GG GG GG
GG AG AG GG GG GG AG AG
GG GG GG GG GG GG GG AG
GG GG GG GG AA GG AG AG
GG GG GG GG GG AG GG
2572666
10835 rs10416610 19 AA AA AA AA AA
AA AA AA AA AA AA AA AA
AA AA AA AA AA AA AA AA
AA AA AA AA AA AA AA AA
AA AA AA AA AA AA AA
57033629
10836 rs10416634 19 AA AA AA AA AA
AA AA AA AA AA AA AA AA
AA AA AA AA AA AA AA AA
AA AA AA AA AA AA AA AA
AA AA AA AA AA AA AA
8096158
10837 rs10416645 19 AA AA AA GG AA
AG AG AG AA AA GG AA AG
AA AA AA AA AA AG AA AG
AA AA AA AA AG AA AA AA
AG AA AA AA AA AA AG
16637009
10838 rs10416692 19 GG GG GG GG GG
GG GG GG GG GG GG AG AG
GG GG GG GG GG GG GG GG
GG GG GG GG GG GG GG GG
GG GG GG GG GG GG GG
15206661
10839 rs10416699 19 GG GG GG GG GG
GG GG GG GG GG GG GG GG
GG GG GG GG GG GG GG GG
GG GG GG GG GG GG GG GG
GG GG GG GG GG GG GG
28743206

10840 rs1041670 10 GG AG AG GG GG
AG GG AA AA AA AG AG AA
AG GG AG GG AG AG AG GG
AG AG GG GG AG GG GG AG
GG AG AG AG AA AA AA
110139563
10841 rs10416725 19 AA AA AA AC AA
AC AC AA AA AA AC AC AA
AC AC AC AC AA AA AA AC
AA AC AC AA AC AA AC AA
AC AA AC AA AC AC AA
29831649
10842 rs10416742 19 AG AA AA -- GG
-- -- AA AG AG GG GG GG
AG AG AG GG -- AG AG GG
GG -- -- -- AG GG GG --
AA GG GG GG GG GG GG
36722832
10843 rs1041676 1 AG AG AG AG AA
AG AG GG AG AG AG AG AG
AG AG GG GG AG GG GG AG
AG AG AG AG AA AG AA AA
AG GG GG GG AG AG GG
72104447
10844 rs1041677 10 AA AA AA AA AA
AA AA AA AA AA AA AA AA
AA AA AA AA AA AA AA AG
AA AA AG AG AA AA AA AA
AA AA AA AA AG AA AG
30669430
10845 rs10416878 19 GG GG GG GG GG
GG GG GG GG GG GG GG GG
GG GG GG GG GG GG GG GG
GG GG GG GG GG GG GG GG
GG GG GG GG GG GG GG
32229218
10846 rs10416919 19 AA AA AA AA AA
AA AA AA AA AA AA AA AA
AA AA AA AA AA AA AA AA
AA AA AA AA AA AA AA AA
AA AA AA AA AA AA AA
46372867
10847 rs10416935 19 AA AC AA CC AC
AC AC AA AA AA AC AA AC
AA AA AA AA AA AA AA AA
AA AA AA AA AC AA AA AC
AC CC AC CC AC CC AA
30050566
10848 rs10416950 19 GG AG AA GG AG
AG AG AA AG AG AG AA AA

AA AA AG GG GG AA AA AA
AG GG AA AA AA AA AA AA
AA AG AG AG AG AG GG
6629209
10849 rs10416969 19 AA AA AA AA AA
AA AA AA AA AA AA AA AA
AA AA AA AA AA AA AA AA
AA AA AA AA AA AA AA AA
AA AA AA AA AA AA AA
29138923
10850 rs10417008 19 AA AA AA AG AG
AA AG AA AA AA AG AG AG
AA AA AA AA AA AA AA AA
AA AA AA AA AG AA AA AG
AA AA AA AA AG AA AG
50203553
10851 rs10417017 19 AC AC CC AC CC
AC AC CC CC CC CC AC CC
CC CC AC AC AC CC CC AC
AC AC CC CC AA AC AA AA
CC AC AC AA AC AC CC
4212345
10852 rs10417021 19 GG GG GG GG GG
GG GG AG GG GG GG GG GG
GG AG AG AG AG AG GG GG
GG AG AG GG AG AG AG AA
GG AG GG GG AG GG AG
57950377
10853 rs10417057 19 AA AA AA AA AA
AG AA AG AA AA AG AA AG
AA AA GG AG AG AG AG AG
AA AA AA AA AA AG AA AA
AG AG AA AG AG AA GG
58177308
10854 rs10417082 19 AG AA AA AA AG
AG AA AA AA AA AA AG AG
AA AA AG AG AA AA AA AA
AG AA AA AG AA AA AA AA
AA AA AA AA AA AA AG
28505900
10855 rs10417084 19 AA AA AG AG GG
AG GG GG GG GG AG GG AG
-- GG GG GG GG GG GG --
AG -- GG AG AG -- AG GG
AG GG GG GG AA AA AG
20365515
10856 rs1041709 13 AA AA AA AA AA
AA AA AA AA AA AA AA AA
AA AA AA AA AA AA AA AA
AA AA AA AA AA AA AA AA

AA AA AA AA AA AA AA
110675198
10857 rs10417094 19 CC AC AC AA AA
AA AA AA AC AC AA AC AA
AA AA AA AA AA AA AA AC
AA AA AC AC AC AC AC CC
AA AC AC AC AA AA AC
11775815
10858 rs1041711 13 AG GG GG GG GG
GG GG GG GG GG GG GG GG
GG GG GG GG GG GG GG GG
GG GG GG GG GG GG GG GG
GG GG GG GG GG GG GG
45106018
10859 rs10417118 19 AA AA AA AA AA
AA AA AA AA AA AA AA AA
AA AA AA AA AA AA AA AA
AA AA AA AA AA AA AA AA
AA AA AA AA AA AA AA
17967456
10860 rs10417119 19 AA AA AA AA AA
AA AA AA AA AA AA AA AA
AA AA AA AA AA AA AA AA
AA AA AA AA AA AA AA AA
AA AA AA AA AA AA AA
32165582
10861 rs1041714 6 GG GG GG GG GG
GG GG AG AG AG GG GG GG
GG GG GG GG GG AA AG AA
AG GG AG AG AG GG GG AG
AG GG GG GG GG GG AG
134141309
10862 rs1041718 6 AA AA AA AA AA
AA AA AA AA AA AA AA AA
AA AA AA AA AA AA AA AA
AA AA AA AA AA AA AA AA
AA AA AA AA AA AA AA
62257582
10863 rs1041719 17 AC AC CC CC AC
AC CC AC CC CC AC AC AC
CC CC AC AC AA AA AC AC
AC AC AC AC CC AC CC CC
AC CC AC CC CC CC AC
32200518
10864 rs1041731 21 AG GG AG AA AA
AA AA AA AA AG GG GG GG
GG GG AG AG AG AG AA AG
AG AA AA AA AA AG AG AG
AG AG AG GG GG GG AG
27225043

10865 rs10417350 19 CC -- -- -- CC
CC -- CC CC CC CC CC --
-- -- -- -- CC -- -- --
-- -- -- CC -- -- -- --
-- -- -- -- -- -- CC
6313415
10866 rs1041738 21 AA AA AA AA AA
AA AA AA AA AA AA AA AA
AA AA AA AA AA AA AA AA
AA AA AA AA AA AA AA AA
AA AA AA AA AA AA AA
31011878
10867 rs10417381 19 AA AA AA AG AG
AA GG AG AG AG AA AA AA
AA AA AA AA AA AG AA AA
AA AA AA AA AA AG AG AG
AG AA AA AA AA AA AA
3036350
10868 rs10417383 19 AA AA AA AA AA
AA AA AA AA AA AA AA AA
AA AA AA AA AA AA AA AA
AA AA AA AA AA AA AA AA
AA AA AA AA AA AA AA
23963115
10869 rs10417391 19 AA AA AA AG AG
AG AG AA AA AA AG AA AA
AA AA AA AA AA AA AA AA
AA AG AG AG AA AA AA AA
AA AG AA AG AA AG AA
17989420
10870 rs10417397 19 GG GG GG GG GG
GG GG GG GG GG GG GG GG
GG GG GG GG GG GG GG GG
GG GG GG GG GG GG GG GG
GG GG GG GG GG GG GG
9872907
10871 rs10417403 19 AA AA AA AG AG
AA GG AA AA AA GG AG GG
AA AA AA AA AA AG AA AA
AA AA AG AG AA AA AA AA
AA AA AA AA AA AA AA
10172516
10872 rs10417407 19 AA AA AA AA AA
AA AA AA AA AA AA AA AA
AA AA AA AA AA AA AA AA
AA AA AA AA AA AA AA AA
AA AA AA AA AA AA AA
24431261
10873 rs10417436 19 AG AG AG AG GG
AG GG AA AG AG AG AA AG

	AG	AG	AG	GG	AG	GG	AA	GG
	AG	AA	AA	AG	AA	GG	AG	AG
	AA	GG	GG	GG	AG	AG	AG	
39528932								
10874	rs10417437		19	AA	AA	AA	AA	AA
	AA	AA	AA	AA	AA	AA	AA	AA
	AA	AA	AA	AA	AA	AA	AA	AA
	AA	AA	AA	AA	AA	AA	AA	AA
	AA	AA	AA	AA	AA	AA	AA	
4019399								
10875	rs10417454		19	AG	AG	AA	GG	GG
	GG	GG	GG	GG	GG	AG	AG	GG
	AG	AG	GG	GG	GG	GG	GG	GG
	GG	AG	AG	GG	GG	GG	GG	GG
	AG	AG	GG	AG	AG	AG	GG	
29052303								
10876	rs10417520		19	AG	GG	GG	AA	AG
	AA	AG	GG	GG	GG	AA	AG	AA
	AA	AA	GG	AG	AG	GG	AG	GG
	AA	AG	AG	AG	AG	AG	AG	GG
	AG	AG	GG	GG	AG	AG	AG	
9102315								
10877	rs10417533		19	GG	GG	GG	GG	GG
	GG	GG	AG	GG	GG	GG	GG	GG
	GG	AG	AG	AG	AG	AG	GG	GG
	GG	AG	AG	GG	AG	GG	GG	AG
	GG	AG	GG	GG	GG	GG	GG	
57931553								
10878	rs1041755		21	AA	AC	AA	CC	CC
	AC	CC	AC	AC	AC	AC	AA	AC
	AC	AC	AC	AA	AC	CC	CC	AC
	AC	CC	AC	AC	AC	AC	AA	CC
	AC	AA	AC	AC	AC	AC	AC	
33238952								
10879	rs10417567		19	GG	AG	GG	AG	AG
	AA	GG	GG	GG	GG	GG	AG	AG
	GG	GG	GG	GG	GG	AG	GG	GG
	AG	AG	AG	GG	GG	GG	GG	GG
	GG	AG	AG	GG	GG	GG	AG	
15308068								
10880	rs10417573		19	AA	AA	AA	AA	AA
	AA	AA	AA	AA	AA	AA	AA	AA
	AA	AA	AA	AA	AA	AA	AA	AA
	AA	AA	AA	AA	AA	AA	AA	AA
	AA	AA	AA	AA	AA	AA	AA	
23963173								
10881	rs10417587		19	AA	AA	AA	AA	AA
	AA	AA	AA	AA	AA	AA	AA	AA
	AA	AA	AA	AA	AA	AA	AA	AA
	AA	AA	AA	AA	AA	AA	AA	AA

AA AA AA AA AA AA AA
12890148
10882 rs10417597 19 AG GG GG GG GG
GG GG GG GG GG AG GG AG
GG GG AG AG AA AG AG GG
AG AG AA AG GG GG GG GG
GG AA AG AG GG GG GG
30190658
10883 rs10417715 19 GG GG GG GG GG
GG GG GG GG GG GG GG GG
GG GG GG GG GG GG GG GG
GG GG GG GG GG GG GG GG
GG GG GG GG GG GG GG
19427576
10884 rs10417780 19 GG AG GG GG AG
GG AG AG AG AG GG GG GG
AG AG AG AA AA AA AG AA
AA AA AA AA AG GG AG AG
AG GG AG GG AG AG AG
15094134
10885 rs10417825 19 GG GG GG GG GG
GG GG GG GG GG GG GG GG
GG GG GG GG GG GG GG GG
GG GG GG GG GG GG GG GG
GG GG GG GG GG GG GG
29113231
10886 rs10417909 19 GG GG GG GG GG
GG GG GG GG GG AG AG AG
AA AG GG GG GG GG GG GG
GG GG GG GG AG GG GG AG
AG GG GG GG GG GG GG
55921579
10887 rs10417932 19 AA AA AG AG GG
AG GG GG GG GG AG GG AG
GG GG GG GG GG GG GG GG
AG GG GG AG AG GG AG GG
AG GG GG GG AA AA AG
20358832
10888 rs10417945 19 GG GG GG GG GG
GG GG GG GG GG GG GG GG
GG GG AG GG -- GG GG GG
GG GG GG GG GG GG GG GG
GG GG GG GG GG GG GG
24177406
10889 rs1041795 21 AA AA AA AA AA
AA AA AA AA AC AA AA AA
AA AA AA AA AA AA AA AA
AA AA AA AA AA AA AA AA
AC AA AA AA AA AA AA
39852396

10890 rs10417957 19 AA AA AA AG AA
AG AA AG AG AG GG AA AG
AG AG AA AA AA AG AA AG
AA AG AA AA AG AA AG AA
AA AG AA AG AA AA AA
4647231
10891 rs1041805 21 GG GG GG GG AA
AG AG AG AG AG AA AG AG
AG AG GG AG AG AG AG GG
AG AG GG GG AG AG GG AG
AA AG AG AA AA AA AA
19700510
10892 rs10418104 19 GG GG GG AG AG
GG AA AA AG AG GG AG AG
AG AG AG AG AA GG AG AG
AG GG AG AG AG AG AA AG
AG GG GG GG AG AG GG
9455390
10893 rs10418111 19 AG GG GG GG GG
GG GG GG GG GG GG AG GG
GG GG AG GG GG GG GG GG
GG GG GG GG AG AG AA AG
GG AG AG GG GG AG GG
10719270
10894 rs10418141
19 -- -- -- -- -- -- -- --
-- -- -- -- -- -- -- --
-- -- -- -- -- -- -- --
-- -- -- -- -- -- -- --
-- -- -- -- 50360046
10895 rs10418203 19 AA AA AG AG GG
AG AG AG GG GG AA GG AG
GG GG AG GG AG GG GG GG
GG GG GG GG GG GG GG GG
AG AG AG AA GG GG AG
22567144
10896 rs10418205 19 AG AA AA AG AG
AA GG AG AG AG GG AG AG
AA AA AA AA AG GG AG GG
GG AG AG AG AG AG GG AG
AA AA AA AA AG AG AG
6100433
10897 rs10418264 19 GG GG GG AG AG
AG AA AA AG AG AG AG AG
AA AG AG AG AG GG AG AG
AG AG AG AG AG AA AA AG
AG AA AG AA AA AA AA
54298576
10898 rs10418296 19 AG AG AG GG GG
GG GG GG GG GG GG GG GG

GG GG GG GG GG GG GG GG
GG GG GG AG AG AG AA AG
GG AA AG AA GG GG GG
53902613
10899 rs1041832 21 GG AG GG AG AG
AG AG AA AA AA GG GG GG
AG GG AG AA AA AA AA AG
AG AG AG AG GG AA AG AG
AA AG AG AG AG AG AG
38183129
10900 rs10418352 19 AA AA AA AG GG
GG AG AA AG AG AA AG AG
AA AA AA AA AA AA AA AG
AA AA AA AA AA GG AG AG
AA AA AA AA AA AA AA
18054851
10901 rs10418368 19 AA AA AA AA AA
AA AA AA AA AA AA AA AA
AA AA AA AA AA AA AA AA
AA AA AA AA AA AA AA AA
AA AA AA AA AA AA AA
19095191
10902 rs10418417 19 AA AA AA AG AA
AG AA AA AG AG AA AA AA
AA AA AA AA AA AA AG AA
AG AG AG AG AA AA AA AA
AG AA AA AA AG AG AA
24080807
10903 rs10418462 19 GG GG GG GG GG
GG GG GG GG GG GG GG GG
GG GG GG GG GG GG GG GG
GG GG GG GG GG GG GG GG
GG GG GG GG GG GG GG
7255881
10904 rs10418480 19 AG AG GG GG GG
AG GG GG AG AG AG AG AG
AG AG AG AA AA AA AG AA
AG AG AG AG GG GG GG GG
AA GG AG GG AA AG AG
57755055
10905 rs10418502 19 AA AA AA AA AA
AA AA AA AA AA AA AA AA
AA AA AA AA AA AA AA AA
AA AA AA AA AA AA AA AA
AA AA AA AA AA AA AA
16387700
10906 rs10418530 19 GG GG GG GG GG
GG GG GG GG GG GG GG GG
GG GG GG GG GG GG GG GG
GG GG GG GG GG GG GG GG

	GG	GG	GG	GG	GG	GG	GG	
28665204								
10907	rs10418535		19	GG	GG	AG	AG	AA
	AA	AG	GG	AG	AG	GG	AA	AG
	AA	AA	AA	AA	AA	AA	AA	AA
	AG	AG	AG	AG	AG	AA	AA	AG
	AA	AA	AA	AA	AA	AA	AA	
17850189								
10908	rs1041856		14	AA	AA	AG	AG	AG
	GG	AA	AG	AG	AA	GG	AG	GG
	AG	GG	GG	AG	AG	GG	AG	AG
	AG	GG	GG	AG	GG	AA	AG	AG
	GG	AG	AG	AG	AA	AG	AA	
22010960								
10909	rs10418574		19	GG	GG	AG	GG	AG
	AG	GG	GG	GG	GG	AG	GG	GG
	GG	GG	GG	GG	GG	GG	GG	GG
	AA	AG	AA	AA	GG	AG	GG	GG
	GG	GG	AA	AG	GG	GG	GG	
6366481								
10910	rs10418577		19	AA	AG	AA	GG	AG
	AG	GG	AG	AA	AA	AA	GG	AG
	GG	GG	AA	AG	AG	AG	AG	AG
	AA	AG	AG	AA	GG	GG	GG	GG
	AG	AG	GG	GG	AG	AG	AG	
29525811								
10911	rs10418662		19	CC	AC	AC	AA	AA
	AA	AA	CC	AC	AC	AC	AC	CC
	AC	AA	AA	AA	AC	AC	AC	AC
	AA	AC	AC	AA	AA	AC	AC	AC
	CC	AC	CC	CC	AA	AA	AA	
46930025								
10912	rs10418706		19	GG	GG	GG	GG	GG
	GG	GG	AG	GG	GG	GG	GG	GG
	GG	GG	GG	GG	GG	AG	GG	GG
	GG	GG	GG	GG	GG	GG	GG	GG
	GG	GG	GG	GG	GG	GG	GG	
21178242								
10913	rs10418710		19	AG	GG	GG	GG	GG
	GG	AG	GG	GG	GG	GG	GG	GG
	GG	GG	GG	GG	GG	AG	AG	GG
	GG	GG	GG	GG	GG	GG	GG	GG
	AG	GG	GG	GG	GG	GG	GG	
31147868								
10914	rs10418716		19	AA	AA	AA	AA	AA
	AA	AA	AC	AA	AA	AA	AA	AA
	AA	AA	AA	AA	AA	AA	AC	AA
	AA	AA	AA	AA	AC	AA	AC	AA
	AC	AA	AA	AA	AA	AA	AA	
13155405								

10915 rs10418759 19 GG AG AG AA AG
AA AG AA AA AA AA AA AA
AA AA AG AA AA AA AG AA
AA AA AA AA AA AG AA AA
AA AA AG AA AA AG AA
11340242
10916 rs10418797 19 AG GG GG AG AG
AA AG AG GG GG AG GG AG
AG AG GG GG GG AA AA AG
AA GG GG AA GG AA AG AG
AG AG AA AG AG AG AA
28856154
10917 rs10418830 19 AA AG AG GG AG
AG GG AG AA AA GG GG GG
GG GG AG AG AA AG GG GG
AG AG AG AG AG GG GG AG
AG GG AG GG AA AG AG
29724513
10918 rs10418839 19 GG GG GG AA GG
AG AG AG AG AG GG GG GG
AG AG GG GG GG GG GG GG
GG GG GG GG AG AG AA --
GG AG AG AG GG GG GG
7855781
10919 rs10418864 19 AA AA AA AA AG
AA AG AG AG AG GG AA AG
GG GG GG AG GG AG AG AG
AG GG AA AA GG AG AG AG
AG AA AG AG AA AG AA
9104971
10920 rs1041887 22 AG AA AA AG AG
GG AA AG AA AA AA AA AA
AA AG AA AA AA AA -- AA
AG AG AA AG AA AA AA AA
AG AG AG AG AG AG AG
44771763
10921 rs10418906 19 GG GG GG GG GG
GG GG GG GG GG GG GG GG
GG GG GG GG GG GG GG GG
GG GG GG GG GG GG GG GG
GG GG GG GG GG GG GG
22610670
10922 rs10418968 19 GG AG GG GG GG
GG GG GG GG GG GG GG GG
GG GG AG GG GG GG GG GG
AG AG AG AG GG AG GG GG
GG GG AG AG GG AG AG
9395584
10923 rs10418984 19 GG GG GG GG AG
AG GG AG AG AG AG GG AG

GG GG GG GG GG GG GG GG
AG GG AG GG GG GG GG GG
GG GG AA AG GG GG GG
31838378
10924 rs10419049 19 GG GG AG GG AG
AG AG AG AG AG AA AG AG
AA AA AA AA AA AA AA AA
AA AA AA AA AA AA AA AA
AG AA AG AA AG AG AA
29956608
10925 rs10419074 19 GG GG GG GG GG
GG GG GG GG GG GG GG GG
GG GG GG GG GG GG GG GG
GG GG GG GG GG GG GG GG
GG GG GG GG GG GG GG
56879136
10926 rs10419078 19 GG GG AG AG AG
AG GG AG AG AG AA AG AG
GG GG AG AG AG AA AG AA
GG GG AG GG AG AA AA AG
GG GG GG GG AG AG GG
51073532
10927 rs10419095 19 AA AA AA AA AA
AA AA AA AA AA AA AA AA
AA AA AA AA AA AA AA AA
AA AA AA AA AA AA AA AA
AA AA AA AA AA AA AA
43465171
10928 rs10419113 19 AA AA AA AA AA
AG AG AG AA AA AA AG AA
AA AA GG GG AG GG AG GG
AA AG AG AA AG AG AA AG
AA AA GG AG AG AA GG
58211530
10929 rs10419143 19 AA AA AA AA AA
AA AA AA AA AA AA AA AA
AA AA AA AA AA AA AA AA
AA AA AA AA AA AA AA AA
AA AA AA AA AA AA AA
28017085
10930 rs1041915 6 AA AA AA AA AA
AA AA AA AA AA AA AA AA
AA AA AA AA AA AA AA AA
AA AA AA AA AG AG AG AA
AA AG AA AG AA AA AA
130014253
10931 rs10419172 19 AA AA AA AA AA
AA AA AA AA AA AA AA AA
AA AA AA AA AA AA AA AA
AA AA AA AA AA AA AA AA

AA AA AA AA AA AA AA
9405975
10932 rs10419191 19 AG AG AA AA AA
AA AA AA AA AA AG AG AG
AG AG AA AA AA AA AA AA
AA AA AA AA AA AA AA AA
AA AA AA AA AA AA AA
55159164
10933 rs10419205 19 GG GG GG GG GG
GG GG GG GG GG GG GG GG
GG GG GG GG GG GG GG GG
GG GG GG GG GG GG GG GG
GG GG GG GG AG AG GG
19987491
10934 rs10419232 19 GG GG GG GG GG
GG GG GG GG GG GG GG GG
GG GG GG GG GG GG GG GG
GG AG AG AG GG GG GG GG
GG GG GG GG GG GG GG
18404148
10935 rs10419308 19 AG AG AG AA AA
AG AA GG GG AG GG GG GG
AG AG AG AG GG AG GG AG
GG GG GG GG AG GG GG AG
AG AG GG GG GG GG GG
55739813
10936 rs10419315 19 GG GG GG GG GG
GG GG GG GG GG GG GG GG
GG GG GG GG GG GG GG GG
GG GG GG GG GG GG GG GG
GG GG GG GG GG GG GG
11872407
10937 rs10419361 19 AG AG AG GG GG
GG GG GG GG GG GG AG AG
GG GG GG AG AG GG GG AG
GG GG GG GG GG GG GG GG
GG AG GG AG GG GG GG
28911340
10938 rs10419363 19 AG AG GG AA AG
AG AG GG GG GG GG AG GG
GG GG AG AA AG GG GG AG
AA AG AG AG GG GG GG GG
GG AG AG AA AA AA AG
4280186
10939 rs10419472 19 GG GG GG GG GG
GG GG GG GG GG GG GG GG
GG GG GG GG GG GG GG GG
GG GG GG GG GG GG GG GG
GG GG GG GG GG GG GG
13544806

10940 rs10419474 19 GG GG GG GG GG
GG GG GG GG GG GG GG GG
GG GG GG GG GG GG GG GG
GG GG GG GG GG GG GG GG
GG GG GG GG GG GG GG
13982006
10941 rs10419482 19 AA AA AA AA AA
AC AA AA AA AA AA AA AA
AA AA AA AA AA CC AC AC
CC AA AC AC AA AA AA AA
AA AA AA AA AA AA AA
49089564
10942 rs10419486 19 CC CC CC CC CC
CC CC CC CC CC CC CC CC
CC CC CC CC CC CC CC CC
CC CC CC CC CC CC CC CC
CC CC CC CC CC CC CC
23024635
10943 rs10419533 19 AA AA AA AA AA
AA AA AA AA AA AA AA AA
AA AA AA AA AA AA AA AA
AA AA AA AA AA AA AA AA
AA AA AA AA AA AA AA
14032762
10944 rs10419562 19 GG AG GG AG AA
AA AG AG AA AA AA AG AG
AA AA AA AG AG AG AG AG
GG GG GG GG AG AA AA AA
AA AA GG AG AA AG AG
16223287
10945 rs10419638 19 CC CC CC CC CC
CC CC CC CC CC CC CC CC
CC CC CC CC CC CC CC CC
CC CC CC CC CC CC CC CC
CC CC CC CC CC CC CC
57511021
10946 rs10419660 19 GG GG GG AG GG
AG GG GG GG GG GG GG GG
GG GG AA AG AG GG AG GG
AG GG GG AG GG GG GG GG
AG GG GG GG GG GG GG
24339653
10947 rs10419663 19 AA AA AA AG AA
AG AA AA AA AA AA AA AA
AA AA AA AA AA AA AG AA
AG AA AA AG AG AA AA AG
GG AA GG AG AA AA AG
35666248
10948 rs1041967 17 AA AA AA AA AA
AA AA AA AA AA AA AA AA

AA AA AA AA AA AA AA AA
AA AA AA AA AA AA AA AA
AA AA AA AA AA AA AA
1340365
10949 rs10419684 19 GG GG GG GG GG
GG GG GG GG GG GG GG GG
GG GG GG GG GG GG GG GG
GG GG GG GG GG GG GG GG
GG GG GG GG GG GG GG
19304555
10950 rs10419687 19 CC AC AC AA AC
AC AA AC CC CC AC AC AC
AC AC CC CC CC AC CC CC
AC AC AC AC AA CC AC AC
CC AA AC AA AC AC CC
17789409
10951 rs10419707 19 AG AA AA AG AG
AA AG AG AG AG AG AA AG
AG AG AG AA AA AG AA AG
AA AA AA AA AG AG AG GG
AA AA AG AA AA AA AG
1038445
10952 rs10419869 19 GG GG GG GG GG
GG GG GG GG GG GG GG GG
GG GG GG GG GG GG GG GG
GG GG GG GG GG GG GG GG
GG GG GG GG GG GG GG
15200156
10953 rs10419939 19 AG AG AG AA AA
AA AG AG AG AG AA AG AA
AG GG AG AA AG AG AG GG
GG AG AG GG AG AG AA AG
AG AG AG AG AG AA AG
57027606
10954 rs10419948
19 -- -- -- -- -- -- -- --
-- -- -- -- -- -- -- --
-- -- -- -- -- -- -- --
-- -- -- -- -- -- -- --
-- -- -- -- 696234
10955 rs10419990 19 AG AG AG AG AA
AG AG AA AA AA AG AG GG
AG AG GG GG GG AG AG AG
AG AA GG GG AG AG AA AG
AG GG GG GG AG AG AA
5037083
10956 rs10420007 19 AA AA AA AG AG
AA AG AG AA AA GG AG GG
AG AG AG AA AG AG AA AG
AG AA AG AG AA AA AA AA

AA AA AG AG AA AA AA
4724069
10957 rs10420029 19 GG GG GG GG GG
GG GG GG GG GG GG GG GG
GG GG GG GG GG GG GG GG
GG GG GG GG GG GG GG GG
GG GG GG GG GG GG GG
13784215
10958 rs10420138 19 AG AG AG GG GG
AG GG AG AA AA AA AG AG
AG AG AG AG AG AG AG AG
AG AA AG AA AG AG GG AG
AG AG AG AG GG GG GG
52704961
10959 rs10420211 19 AA AA AA AA AA
AA AA AA AA AA AA AA AA
AA AA AA AA AA AA AA AA
AA AA AA AA AA AA AA AA
AA AA AA AA AA AA AA
23488306
10960 rs10420262 19 GG GG GG GG GG
GG GG GG GG GG GG GG GG
GG GG GG GG GG GG GG GG
GG GG GG GG GG GG GG GG
GG GG GG GG GG GG GG
23728949
10961 rs10420291 19 AA AA AA AA AA
AA AA AA AA AA AA AA AA
AA AA AA AA AA AA AA AA
AA AA AA AA AA AA AA AA
AA AA AA AA AA AA AA
18908381
10962 rs10420331 19 GG GG AG AA AA
AG AG AG AA AA AG AA AA
AA AA AA AA AA GG AG GG
AG GG GG AG GG GG GG GG
AG AG AA AA GG GG AG
54469068
10963 rs10420371 19 AG GG GG GG GG
GG GG AA AG AG GG GG GG
GG GG GG GG GG GG GG GG
GG GG GG GG GG GG GG GG
GG GG GG GG GG GG GG
9728350
10964 rs10420392 19 AA AG AG AG AG
AA AG GG GG GG AG GG GG
GG GG AG AA AG AG AG GG
AA AG AG AA AG AG AA AG
GG GG GG GG GG GG AG
28586861

10965 rs10420405 19 AA AA AA AA AA
AA AA AA AA AA AA AA AA
AA AA AA AA AA AA AA AA
AA AA AA AA AA AA AA AA
AA AA AA AA AA AA AA
56613447
10966 rs10420421 19 GG GG GG GG GG
GG GG GG GG GG GG GG GG
GG GG GG GG GG GG GG GG
GG GG GG GG GG GG GG GG
GG GG GG GG GG GG GG
1560832
10967 rs10420500 19 CC CC CC CC CC
CC CC CC CC CC CC CC CC
CC CC CC CC CC CC CC CC
CC CC CC CC CC CC CC CC
CC CC CC CC CC CC CC
54462073
10968 rs10420546 19 GG GG GG GG GG
GG GG GG GG GG GG GG GG
GG GG GG GG GG GG GG GG
GG GG GG GG GG GG GG GG
GG GG GG GG GG GG GG
18032236
10969 rs10420567 19 AA AA AA AA AA
AA AA AA AA AA AA AA AA
AA AA AA AA AA AA AA AA
AA AA AA AA AA AA AA AA
AA AA AA AA AA AA AA
29768465
10970 rs10420573 19 CC CC CC CC CC
CC CC CC CC CC CC CC CC
CC CC CC CC CC CC CC CC
CC CC CC CC CC CC CC CC
CC CC CC CC CC CC CC
19179948
10971 rs10420623 19 GG GG GG GG GG
GG GG GG GG GG GG GG GG
GG GG GG GG GG GG GG GG
GG GG GG GG GG GG GG GG
GG GG GG GG GG GG GG
20225267
10972 rs10420661 19 GG GG GG GG GG
GG GG GG GG GG GG GG GG
GG GG GG GG GG GG GG GG
GG GG GG GG GG GG GG GG
GG GG GG GG GG GG GG
28816178
10973 rs10420686 19 AA AA AA AA AA
AA AA AA AA AA AA AA AA

	AA	AA	AA	AA	AA	AA	AA	AA
	AA	AA	AA	AA	AA	AA	AA	AA
	AA	AA	AA	AA	AA	AA	AA	
17565170								
10974	rs10420727		19	AA	AG	AG	AG	AG
	GG	AG	GG	AG	AG	AG	GG	AG
	AG	AA	AG	AG	AG	GG	AG	GG
	AG	AG	AG	AG	GG	AG	GG	GG
	GG	GG	AG	AG	AG	AG	AG	
57181967								
10975	rs10420736		19	GG	GG	GG	GG	GG
	GG	GG	GG	GG	GG	GG	GG	GG
	GG	GG	GG	GG	GG	GG	GG	GG
	GG	GG	GG	GG	GG	GG	GG	GG
	GG	GG	GG	GG	GG	GG	GG	
11623152								
10976	rs10420780		19	GG	AG	GG	AG	GG
	GG	AG	AG	AG	AG	AG	AG	AG
	AG	AG	AG	AG	AG	AG	AG	AG
	AA	AA	AA	AG	AA	GG	AG	AG
	AG	AG	AG	GG	AG	AG	AA	
14731686								
10977	rs10420802		19	AA	AA	AA	AA	AA
	AG	AG	AA	AA	AA	AA	AG	AA
	AG	AG	AA	AG	AA	AA	AA	AA
	AA	AA	AA	AA	AA	AA	AA	AA
	AA	AA	AA	AA	AG	AA	AG	
57202924								
10978	rs10420813		19	GG	AG	AA	AG	AG
	GG	AA	AG	AG	AG	GG	AG	GG
	AG	AG	GG	GG	GG	GG	AG	GG
	AG	GG	GG	AG	AG	AA	AA	AG
	AG	GG	GG	GG	AG	GG	AG	
31354223								
10979	rs10420817		19	AG	AG	AA	AA	AA
	AA	AA	AA	AA	AA	AA	AG	AG
	AA	AA	AA	AA	AA	AA	AA	AA
	AG	AA	AG	AG	AG	AA	AA	AG
	AG	GG	AA	AG	AA	AA	AA	
4605239								
10980	rs10420834		19	CC	CC	CC	CC	CC
	CC	CC	AC	AC	AC	CC	CC	CC
	CC	CC	CC	CC	CC	CC	CC	CC
	CC	CC	CC	CC	CC	CC	CC	CC
	CC	CC	AC	AC	CC	CC	CC	
46347542								
10981	rs10420877		19	GG	GG	GG	GG	GG
	GG	GG	GG	GG	GG	GG	GG	GG
	GG	GG	GG	GG	GG	GG	GG	GG
	GG	GG	GG	GG	GG	GG	GG	GG

GG GG GG GG GG GG GG
28816240
10982 rs10420886 19 GG GG AG AA AA
AA AG AA AG AG AA GG AG
AG AG GG AG AG AG GG AG
GG AA AA GG AA AG AG AG
AA AG AA AA AG AG AG
23436759
10983 rs10420981 19 GG AG GG AG GG
AG GG GG AG AG GG GG GG
GG GG GG AG AG GG AG GG
AG AG AG AG AG GG AG GG
AG GG AG GG AG AG GG
24112777
10984 rs10421009 19 AA AA AA AA AA
AA AA AA AA AA AA AA AA
AA AA AA AA AA AA AA AA
AA AA AA AA AA AA AA AA
AA AA AA AA AA AA AA
32361209
10985 rs10421011 19 AG AG AA GG GG
GG AG AG GG GG AA AG AA
AA AA AG AG AG AG AA GG
GG AG AG GG AG GG GG AG
AA GG AG GG AA AG AG
57977730
10986 rs10421012 19 AA AG AA AA AG
AA AG AG AA AA AA AA AA
AG AG AA AA AA AG AA AA
AA AA AA AA AG AA AA AG
AG AG AA AG AA AA AA
12291610
10987 rs10421078 19 GG GG GG AG AG
GG AG GG GG GG GG GG GG
GG GG GG GG GG GG GG GG
GG GG GG GG GG GG GG GG
GG GG GG GG GG GG GG
6340463
10988 rs10421113 19 AG AG AG AG AA
AG AG AA AG AG AG GG AG
AG AG GG GG GG GG GG AG
GG GG GG GG GG GG GG GG
GG AG GG AG GG GG GG
21929885
10989 rs10421126 19 AA AA AG AG AG
AA GG AA AA AA AA AA AA
AA AA AG AA AA AA AA AA
GG AG AG AG AG AG AG AA
AA AA AA AA AA AG AA
4471797

10990 rs1042122 19 AA AA AG AG AG
AG AG AA AA AA AA AG AG
GG GG AG AG AA AA AG AA
AA AG AA AG AG AA AA AG
AG AA AG AG AG AG AA
49989424
10991 rs10421273 19 GG GG GG AG AA
AG AA AG GG GG GG GG GG
AG AG AG AG GG AA AA AG
AG AG AG AG GG GG GG GG
AG GG GG GG GG GG GG
29048625
10992 rs10421280 19 AA AA AG AG AG
AG AG AA AA AA AG AA AG
AA AA AA AA AA AG AA AA
AA AA AA AA AA GG AG AG
AA AA AG AA AG GG AA
19973452
10993 rs10421281 19 AA AA AA AA AA
AA AA AA AA AA AA AA AA
AA AA AA AA AA AA AA AA
AA AA AA AA AA AA AA AA
AA AA AA AA AA AA AA
55412835
10994 rs10421336 19 AA AA AA AG AG
AA GG AA AA AA AA AA AA
AA AA AG AA AA AA AA AA
AA AA AG AG AA AG AA AA
AA AG AA AA AA AG AA
9939831
10995 rs10421355 19 AC AC AC CC CC
CC CC AA AC AC CC CC CC
AC AC CC CC CC AC CC CC
CC CC CC CC AA AC -- --
CC AC CC AC AC AC CC
16148728
10996 rs10421359 19 AC AC AA AC AC
AC AA AC AC AC AC AA AC
[truncated: 397,666 more chars]
